# Supplementary material for: Pangenome dynamics and population structure of the zoonotic pathogen Salmonella enterica serotype Hadar
Source: Nat Commun. 2026 Jan 24;17:1270. doi: 10.1038/s41467-025-68026-3 (PMC12868874; doi:10.1038/s41467-025-68026-3)
Supplement: Supplementary file 1 — Supplementary Information [file 41467_2025_68026_MOESM1_ESM.pdf]

Pangenome dynamics and population structure of the zoonotic pathogen *Salmonella enterica* serotype Hadar

SUPPLEMENTARY INFORMATION

Supplementary Table 1. Cramer's *V* associations between pairs of categorical variables of interest.

categorical variable	all JI-groups	JI-A	JI-B	JI-C	JI-D	JI-E	JI-F	JI-G	JI-H
YearOfIsolation	0.286	0.572	0.593	0.423	0.516	0.320	0.066	0.137	0.081
StateOfIsolation	0.129	0.191	0.280	0.160	0.240	0.138	0.087	0.119	0.212
human clinical-backyard flock contact	0.320	0.020	0.214	0.173	0.116	0.098	0.040	0.032	0.029
human clinical-turkey consumption	0.017	0.052	0.032	0.000	0.021	0.000	0.000	0.000	0.000
human clinical-unknown exposure	0.240	0.087	0.185	0.000	0.000	0.017	0.074	0.000	0.000
backyard flock sampling	0.000	0.000	0.012	0.027	0.000	0.000	0.000	0.000	0.000
cattle	0.000	0.000	0.000	0.000	0.000	0.000	0.000	0.000	0.000
chicken	0.245	0.050	0.038	0.049	0.031	0.000	0.000	0.000	0.223
swine	0.367	0.010	0.000	0.038	0.015	0.000	0.000	0.000	0.000
turkey	0.518	0.165	0.480	0.140	0.121	0.049	0.038	0.070	0.027
other	0.000	0.003	0.000	0.000	0.055	0.000	0.000	0.000	0.000
NOAARegion	0.121	0.135	0.210	0.113	0.170	0.093	0.076	0.060	0.131
HHSRegion	0.097	0.088	0.159	0.104	0.118	0.088	0.094	0.054	0.064
Age	0.108	0.150	0.422	0.220	0.180	0.090	0.169	0.000	0.000
Sex	0.285	0.146	0.437	0.170	0.121	0.061	0.037	0.057	0.056
Hospitalized	0.286	0.083	0.285	0.238	0.086	0.078	0.065	0.037	0.042
REPcode	0.840	0.571	0.546	0.281	0.337	0.148	0.120	0.101	0.096
AlleleCodeCondensed	0.605	0.638	0.869	0.205	0.871	0.386	0.843	0.163	0.000
NCBISNPCluster	0.703	0.396	0.187	0.164	0.888	0.396	0.830	0.000	0.319
ResistanceDeterminants	0.450	0.410	0.300	0.339	0.736	0.655	0.038	0.394	0.000
PlasmidReplicons	0.631	0.819	0.789	0.916	0.953	0.947	0.439	0.565	0.350
PlasmidTaxonomicUnit	0.915	0.604	0.214	0.975	0.999	0.999	0.023	0.285	0.000
Prophage1	0.898	0.628	0.598	0.262	0.368	0.162	0.131	0.111	0.087

Supplementary Table 2. Odds Ratio calculations for select JI-groups.

JI-group	source: backyard poultry related	source: commercial turkey	source: other	total	odds ratio	95% lower confidence interval	95% upper confidence interval
JI-A	431		1407	1838	1.1	0.9	1.3
JI-A		219	1619	1838	0.4	0.3	0.5
JI-B		285	142	427	17.5	13.7	22.3
JI-C	184		265	449	2.8	2.3	3.5
JI-C		16	433	449	0.1	0.1	0.2
JI-D	74		104	178	2.6	1.9	3.6
JI-E	24		16	40	5.2	2.7	10.6
JI-G		10	8	18	5.9	2.1	17.1

Supplementary Table 3. US and non-US JI-groups.

JI-groups	number of US genomes	% US	number of non-US genomes	% non-US genomes
A + K	1911	56.42	208	18.17
B	489	14.44	36	3.14
C	453	13.37	2	0.17
D	191	5.64	1	0.09
E	40	1.18	1	0.09
F	29	0.86	4	0.35
G	20	0.59	0	0
H	20	0.59	0	0
I	17	0.5	70	6.11
J	13	0.38	0	0
L	9	0.27	3	0.26
M	7	0.21	0	0
N	6	0.18	0	0
O	6	0.18	0	0
P	5	0.15	28	2.45
Q	5	0.15	26	2.27
R	5	0.15	5	0.44
S	0	0	90	7.86
T	0	0	46	4.02
U	0	0	27	2.36
V	0	0	22	1.92
W	0	0	19	1.66
X	0	0	17	1.48
Y	0	0	10	0.87
Z	0	0	8	0.7
AA	0	0	8	0.7
AB	0	0	8	0.7
AC	0	0	7	0.61
AD	0	0	7	0.61
AE	0	0	7	0.61
AF	0	0	6	0.52
AG	0	0	5	0.44
AH	6	0.18	96	8.38
AI	4	0.12	81	7.07
AJ	1	0.03	36	3.14
AK	2	0.06	16	1.4
AL	1	0.03	8	0.7
AM	2	0.06	9	0.79
AN	1	0.03	8	0.7
singletons	141	4.16	220	19.21

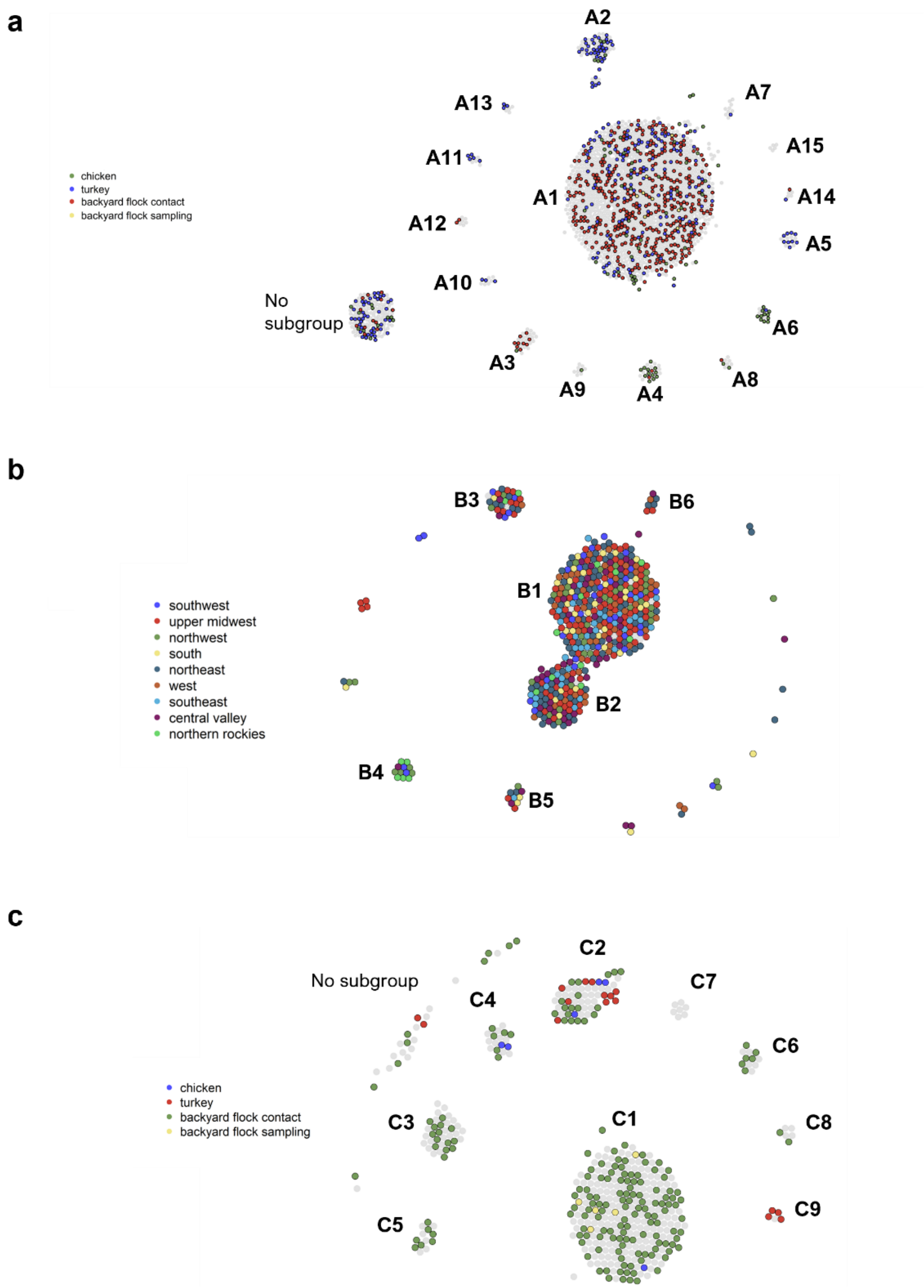

Supplementary Figure 1: Networks of JI-subgroups.

a) JI-A subgroup network colored by source, defined at JI=0.995. Grey nodes indicate genomes from sources other than those colored. b) JI-B subgroup network colored by National Oceanic and Atmospheric Administration (NOAA) region, defined at JI=0.992. Grey nodes indicate genomes from unknown NOAA region. c) JI-C subgroup network colored by source, defined at JI=0.992. Grey nodes indicate genomes from human clinical sources with no exposure information reported, or no contact with backyard poultry.

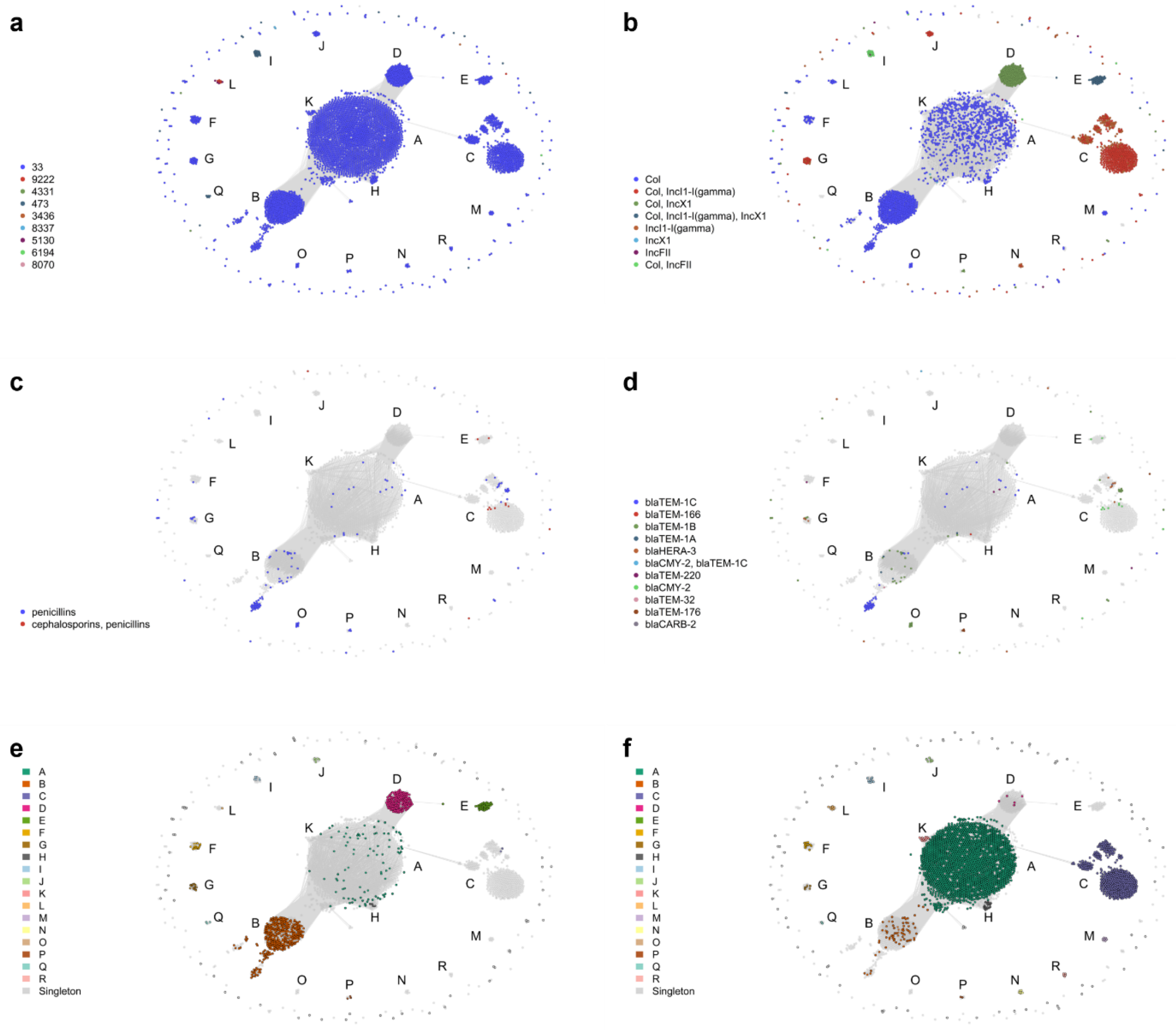

Supplementary Figure 2: Distribution of Hadar genomes by variables of interest.

a) Full network colored by Sequence Type (ST). b) Full network colored by four most common plasmid replicons across JI-groups. c) Full network colored by predicted resistance to cephalosporin and penicillin. d) Full network colored by cephalosporin and penicillin antibiotic resistance genes. e) Full network colored by JI-groups present between years 2016 and 2019. f) Full network colored by JI-groups present between years 2020 and 2023.

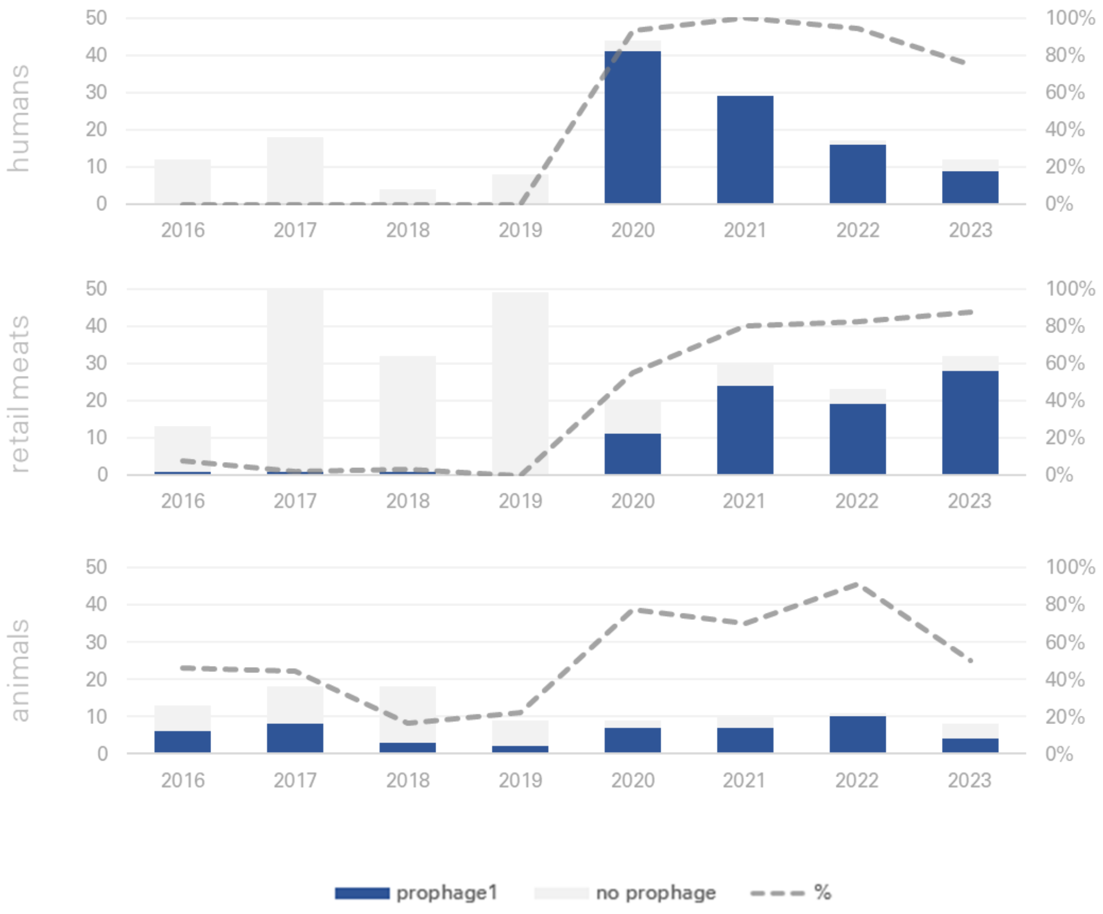

Supplementary Figure 3: Occurrence of prophage in NARMS surveillance sequencing over time. NARMS data from CDC (humans), FDA (retail meats) and FSIS (animals) from 2016-2023 are included. Year is displayed on the x-axis and counts of isolates with and without prophage 1 are displayed on the y-axis. Dotted line represents % of isolates containing prophage 1.

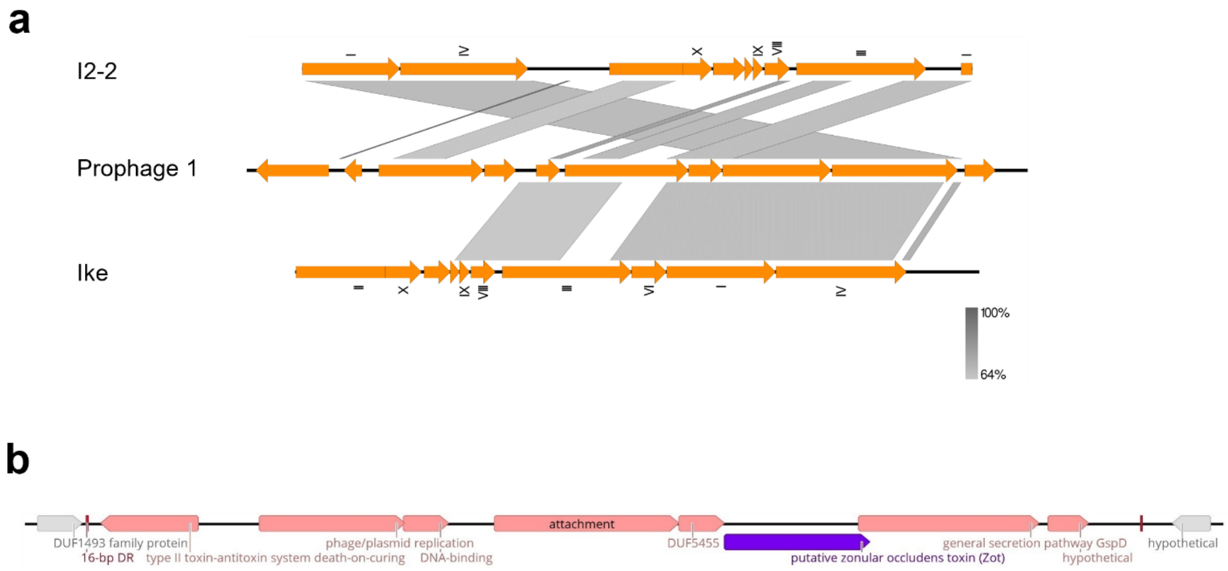

Supplementary Figure 4: Analysis of prophage 1.

a) Genomic alignment of prophages I2-2 (NCBI Reference Sequence: NC_001332.1), Prophage 1, and Ike (NC_002014.1), visualized using Easyfig. Orange arrows represent annotated genes, with arrowheads indicating the direction of transcription. Shaded gray regions between the prophages denote areas of sequence homology, with shading intensity reflecting the percentage of sequence identity (64%–100%, as indicated by the scale bar on the right). Conserved regions highlight shared sequences, while gaps or unshaded areas represent regions lacking significant similarity. Gene annotations for I2-2 and Ike correspond to those provided in their respective GenBank entries. b) Prophage 1 annotated in Geneious Prime. Chromosomal coding sequences colored in gray, prophage 1 coding sequences colored in pink, putative zonular occludens toxin coding sequence colored in purple (annotations were obtained from NCBI).

a

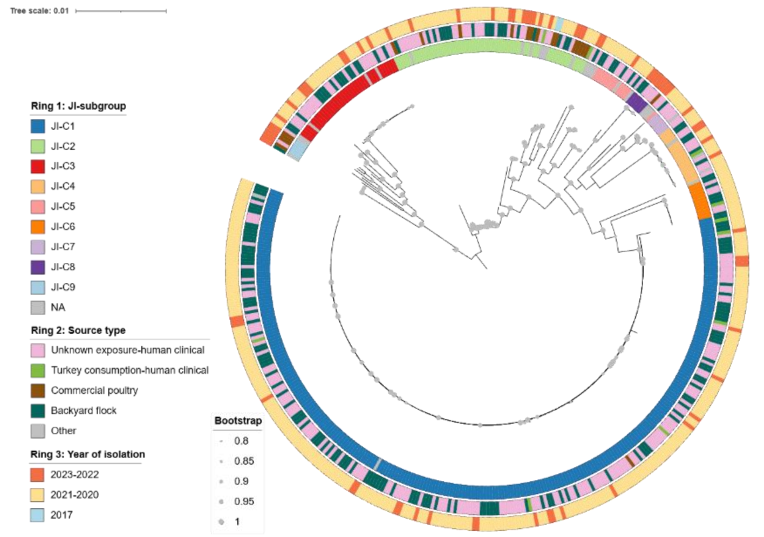

b

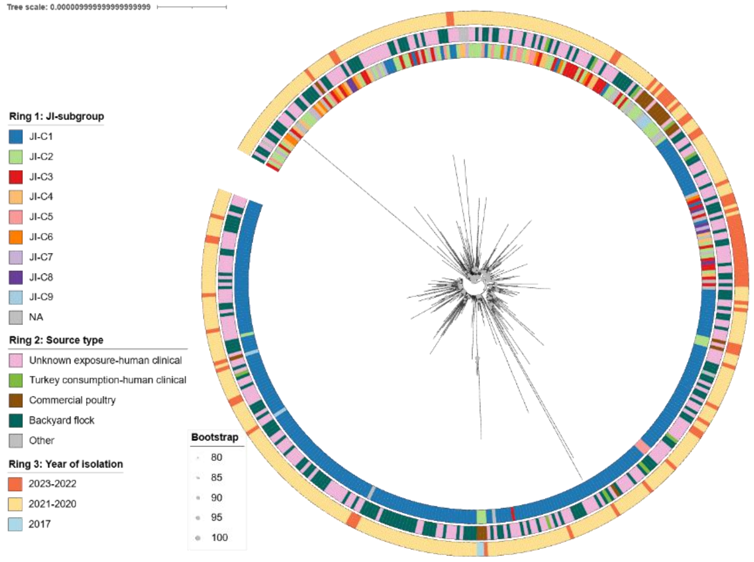

Supplementary Figure 5: Phylogenetic analysis of JI-C isolates.

Maximum Likelihood trees were generated from the core genome-SNP alignment of either a) 453 PTU-I1 plasmids from JI-C or b) JI-C chromosomes. Substitution models TVM+F+I+R4 (panel a) or TIMe+ASC (panel b) were used. UFBootstrap values > 80% are indicated by circles on the corresponding nodes. Rings, colored according to the legend, are ordered from the inner to the outer (Ring 1 is the innermost) and display JI-subgroup, source type, and time range, respectively.

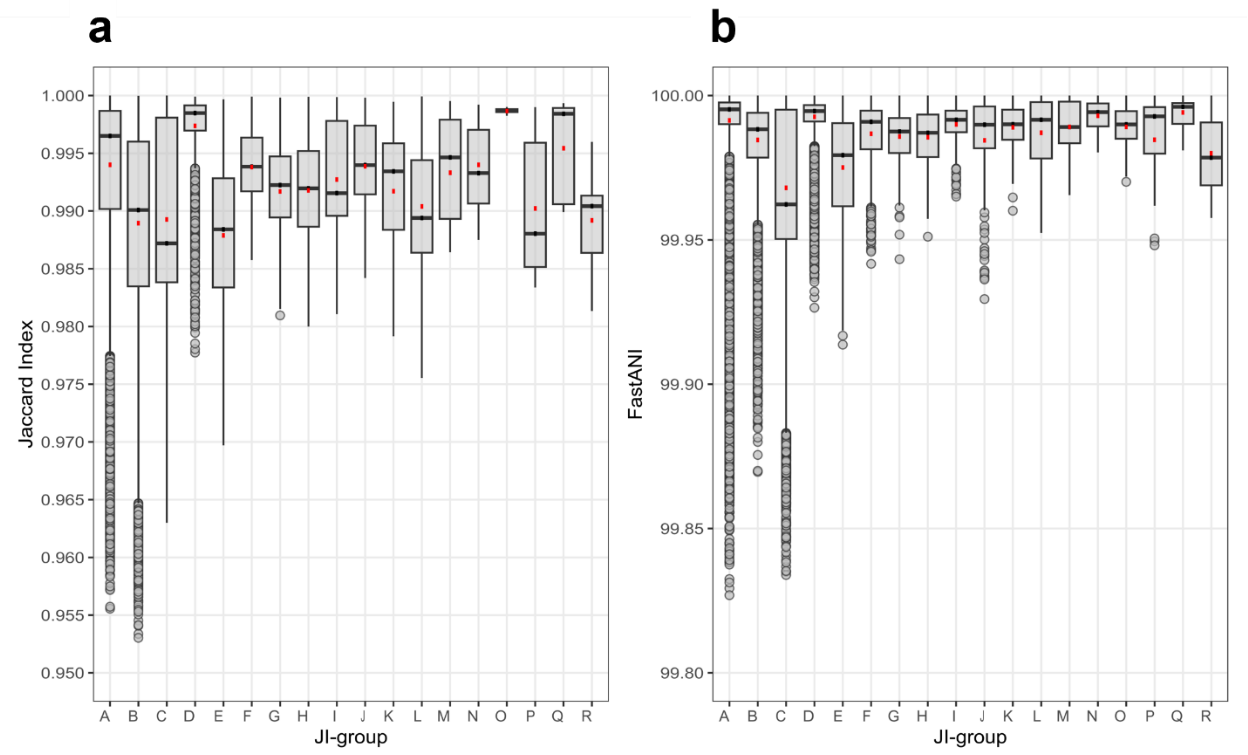

Supplementary Figure 6: Relatedness of genomes within each JI-group.

Boxplot illustrating the distribution of a) Jaccard Index (JI) and b) FastANI values across different JI-groups. The boxplot displays the interquartile range (IQR) of JI values within each JI-group, with the lower and upper edges of the box indicating the first quartile (Q1) and third quartile (Q3), respectively. Within each boxplot, horizontal lines represent the median (black) and the average (red) values. The 'whiskers' extend to the most extreme values within 1.5 times the IQR from the edges of the box, while outliers are depicted as individual points beyond the whiskers.

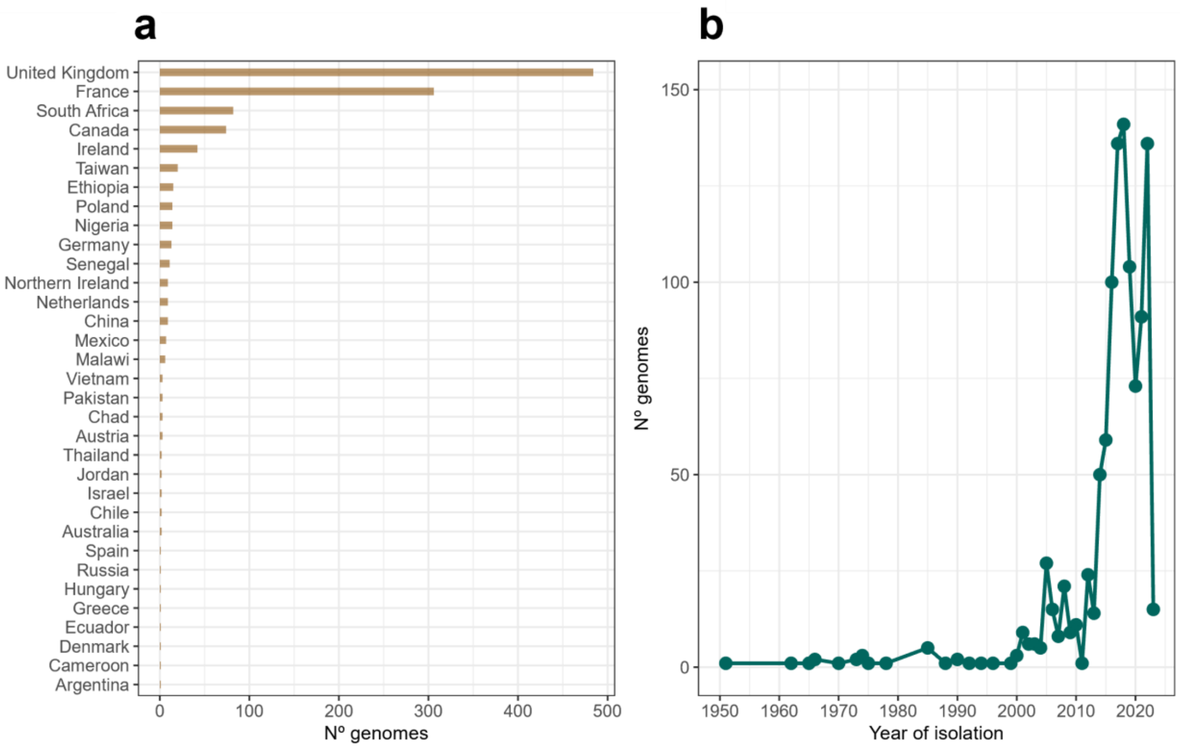

Supplementary Figure 7: Characteristics of the non-U.S. *Salmonella* Hadar dataset (n=1145).

a) Number of genomes per country. b) Number of genomes per year of isolation.

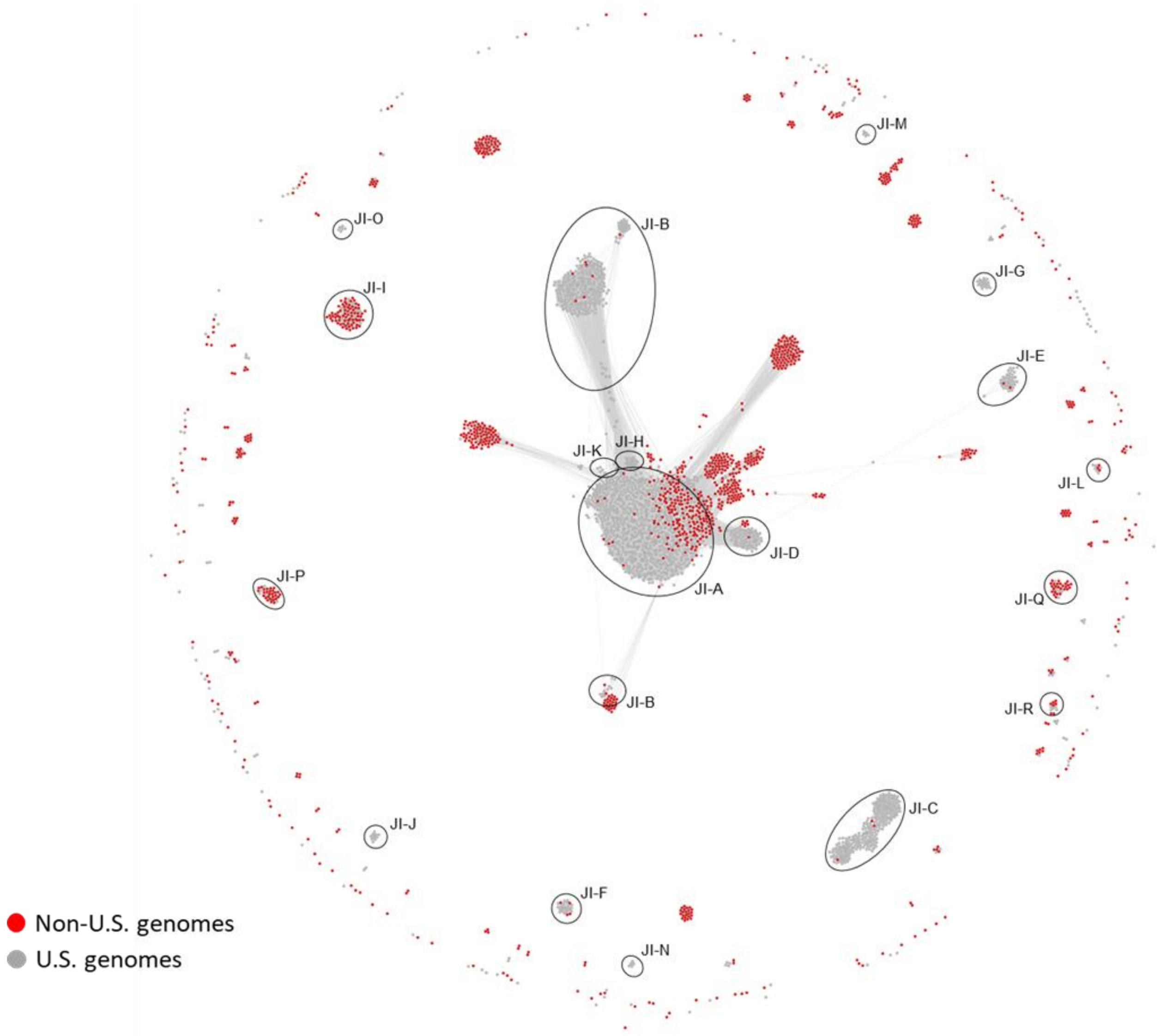

Supplementary Figure 8: Distribution of U.S. and non-U.S. *Salmonella* Hadar genomes by JI-group. The JI network contains 1145 non-U.S. Hadar genomes from EnteroBase (Supplementary Table S2) and a reduced U.S. dataset (n=1516), using JI ≥ 0.988 as a threshold. U.S. genomes are represented by grey nodes and circled when belonging to a JI-group (Supplementary Table S1), while non-U.S. genomes are represented by red nodes. To select the U.S. genomes, the complete U.S. Hadar dataset was first clustered at JI ≥ 0.99916, a threshold in which only practically identical genomes were connected. A greedy set algorithm was implemented to reduce the dataset. The connectivity degree (number of connections) of each node was calculated to select the most connected genome as a representative. All nodes connected to the representative were removed. This process was repeated until the network exclusively contains unconnected representative nodes.

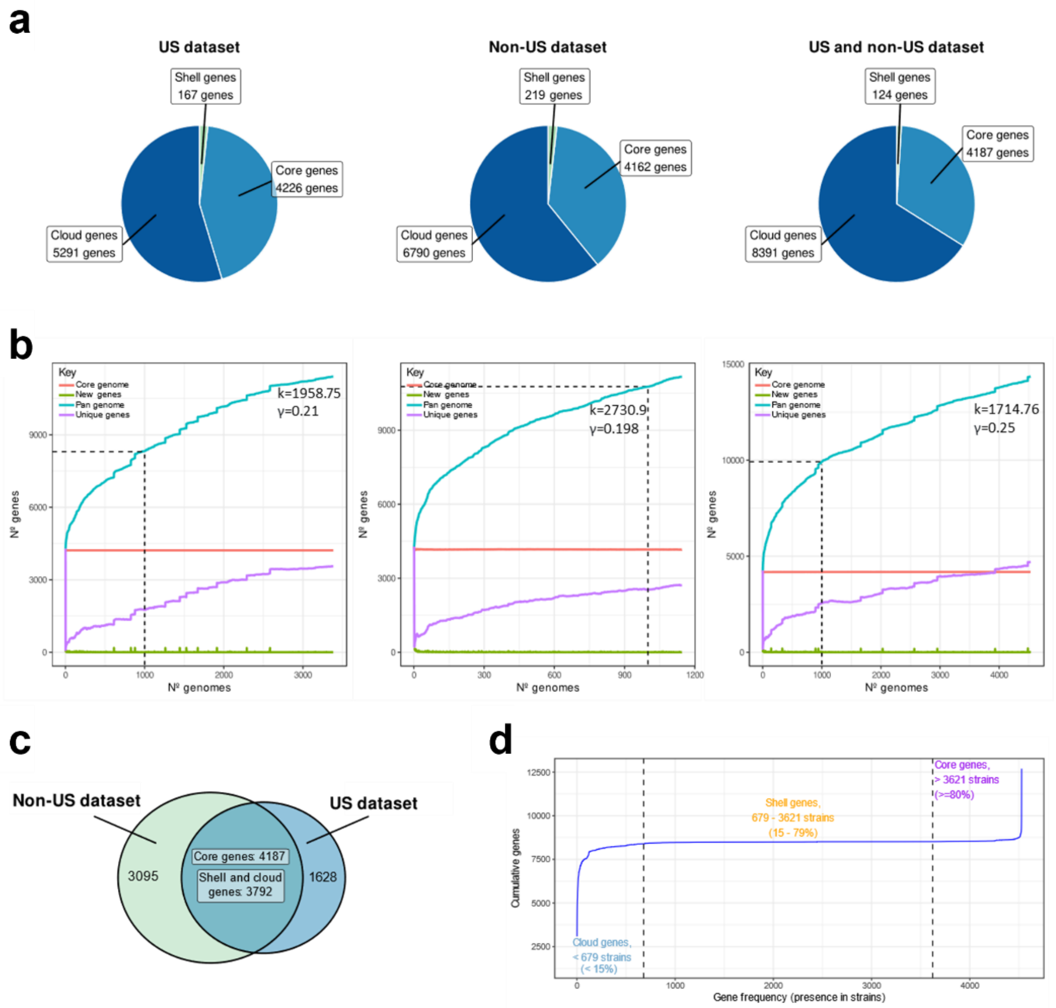

Supplementary Figure 9: Comparative analysis of pangenome distribution across U.S. and non-U.S. datasets using Roary.

a) Pie charts showing number of core, shell, and cloud genes within each dataset. Core genes (dark blue) are present in > 80%, shell genes (medium blue) are present in 15-79%, and cloud genes (light blue) are found in ≤ 15% of genomes. b) Accumulation curves for core, new (previously unseen), pan, and unique (observed only once) genes across the increasing number of genomes analyzed. The x-axis represents the number of genomes. The y-axis represents the cumulative number of genes in each category. Parameters k and γ of the Heaps' law are shown for each curve. c) Venn diagram comparing the U.S. and non-U.S. datasets. d) Cumulative gene frequency distribution function. The curve represents the accumulated abundance of genomes in which a gene is present (gene frequency). Discontinuous lines divide the cumulative gene frequency curve into the three gene categories: cloud, shell, and core.

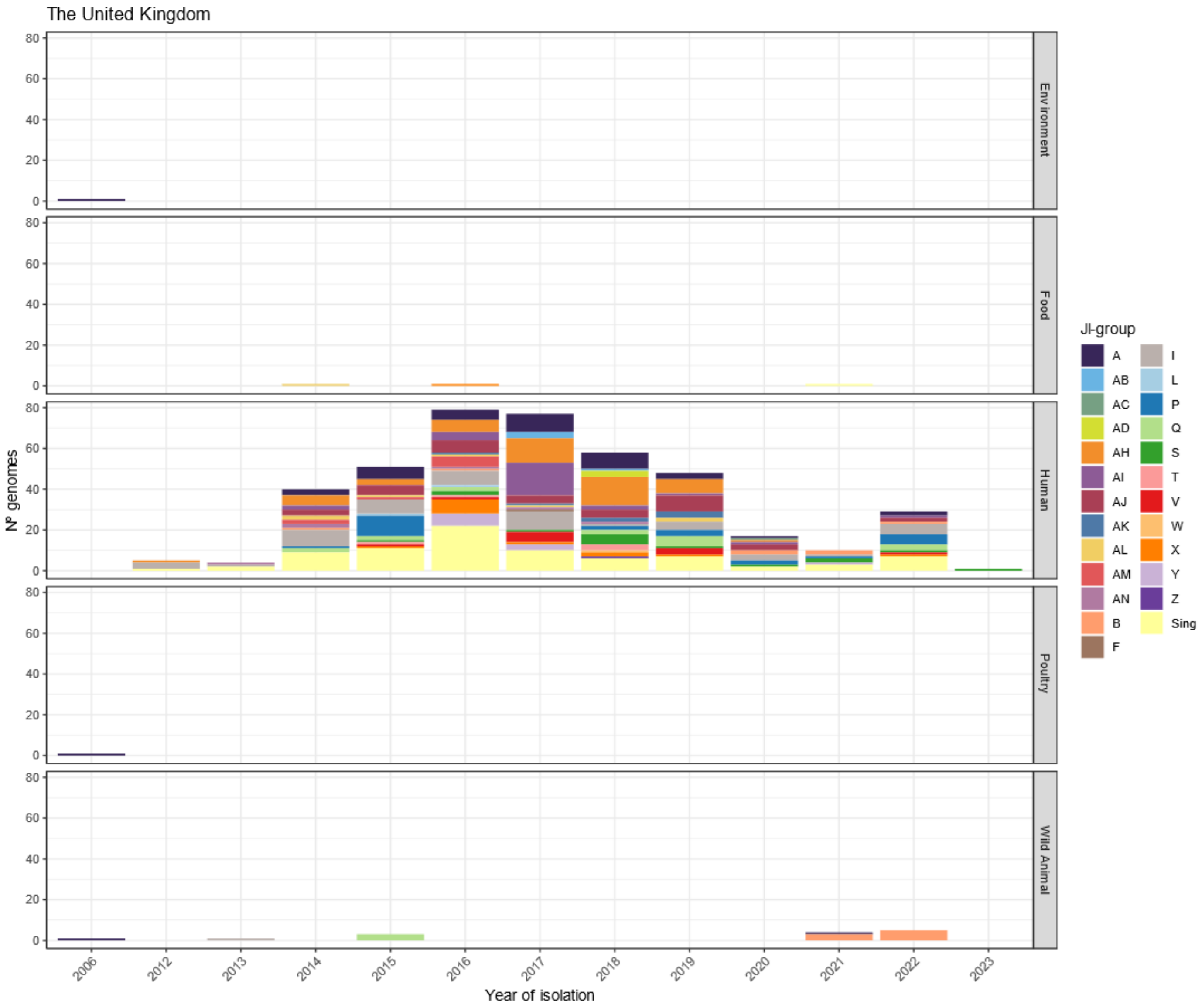

Supplementary Figure 10: Distribution of U.K. Hadar dataset by source and year of isolation. The bar plot shows the number of genomes (y-axis) isolated each year (x-axis) from different sources: environment, food, human, poultry, and wild animal. Each bar is color-coded according to the JI-group classification, as indicated in the legend on the right.

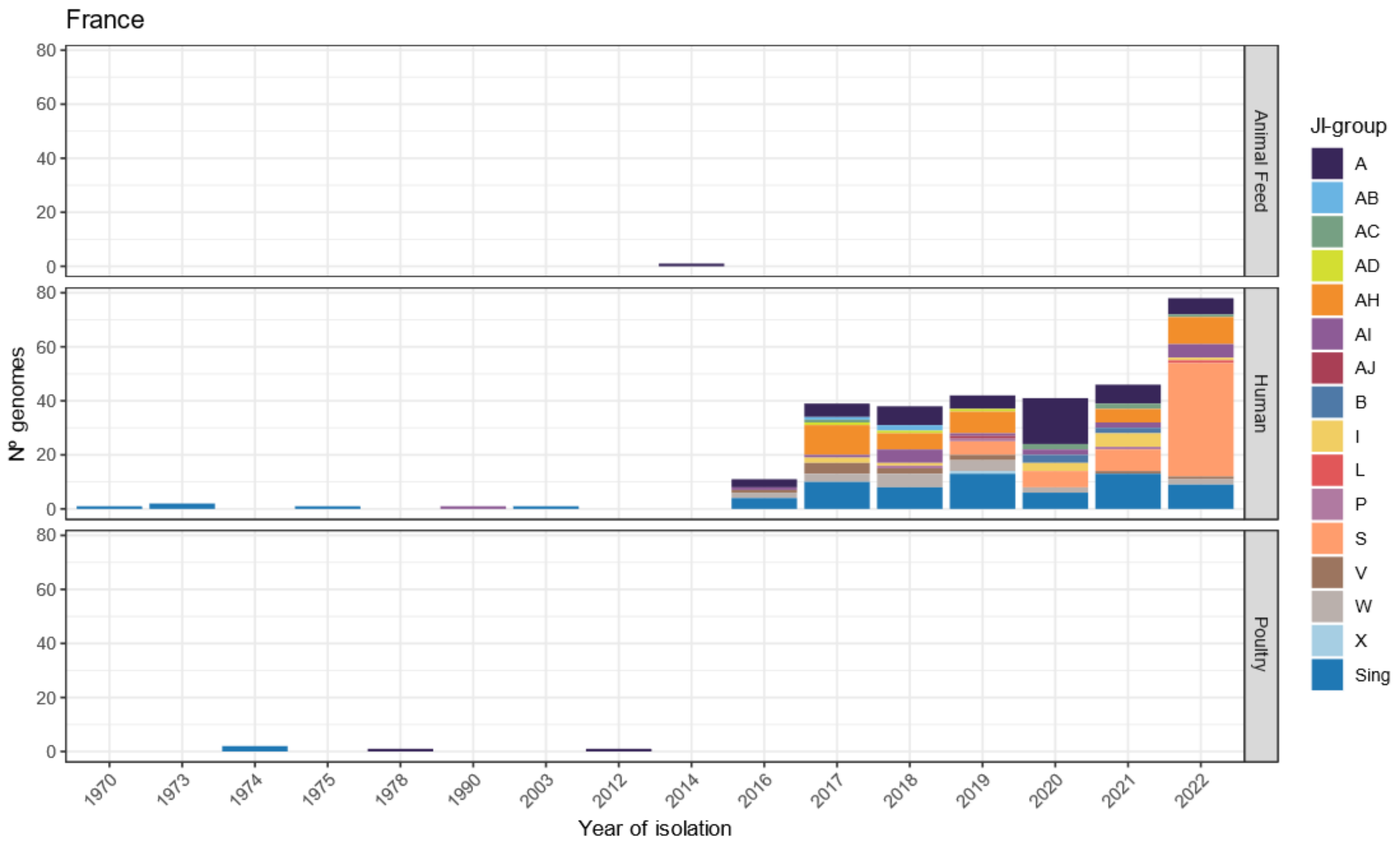

Supplementary Figure 11: Distribution of France Hadar dataset by source and year of isolation. The bar plot shows the number of genomes (y-axis) isolated each year (x-axis) from different sources: environment, food, human, poultry, and wild animal. Each bar is color-coded according to the JI-group classification, as indicated in the legend on the right.
