# Supplementary material for: Pangenome dynamics and population structure of the zoonotic pathogen Salmonella enterica serotype Hadar
Source: Nat Commun. 2026 Jan 24;17:1270. doi: 10.1038/s41467-025-68026-3 (PMC12868874; doi:10.1038/s41467-025-68026-3)
Supplement: Supplementary file 3 — Description of Additional Supplementary Files [file 41467_2025_68026_MOESM3_ESM.pdf]

Description of Additional Supplementary Files

Supplementary Data 1: List of U.S. genomes, along with available genomic and epidemiological metadata.

Supplementary Data 2: List of non-U.S. genomes, along with available genomic and epidemiological metadata.

Supplementary Data 3: List of long-read sequenced genomes, including sequencing information.

Supplementary Data 4: Pancontigs differentially present in JI-groups.

Supplementary Data 5: List of additional PTI-I1 plasmids from Enterobacterales hosts, accessed from RefSeq200.
