# Supplementary material for: Pangenome dynamics and population structure of the zoonotic pathogen Salmonella enterica serotype Hadar
Source: Nat Commun. 2026 Jan 24;17:1270. doi: 10.1038/s41467-025-68026-3 (PMC12868874; doi:10.1038/s41467-025-68026-3)
Supplement: Supplementary file 7 — Supplementary Data 4 [file 41467_2025_68026_MOESM7_ESM.pdf]

Supplementary Data 4: Pancontigs differentially present in JI-groups

Abbreviations: AR (antimicrobial resistance), CRISPR (clustered regularly interspaced short palindromic repeats), ICE (integrative conjugative element), MOB (relaxase family), MPF (mating pair formation), PTU (plasmid taxonomic unit), Tn (transposon)

AR-encoding Tn 1.1

>BDIOMP_08195 DUF4158 domain-containing protein
ATGCCGCGTCGCGTCACTCTAACCGATCGGCAGAAAGACGCGCTGTTGCGCTTGCCGACTTCACAGACGGATTTGCTCAAGCACTATACGCTGAGTGATGAAGACTTTGGGCATATCAGGC
TGCGTCGGCGCGCTCACAACAGGTTCGGCTTCGCCCTGCAATTGTGTGTCCTGCGCTATCCCGGCCGGGTGCTGGCTCCAGGCGAACTGATCCCTGCAGAGGTCATCGAATTTATCGGAGC
GCAGCTTGGCCTGGGTGCCGACGATCTCGTAGACTATGCTGCCCGCGAGGAAACACGGCACGAGCATCTTGCCGAGTTACGGGGGCTCTACGGCTTCCGCACCTTCTCCGGACGTGGTGC
GAGCGAGCTGAAGGAATGGTTGTTCCGAGAAGCCGAGATGGCGGTGTCGAACGAGGATATCGCCCGTCGCTTCGTAGCCGAGTGCCGACGCACCCGCACTGTCCTTCCCGCGACATCCAC
GATCGAGCGGCTTTGTGCCGCGGCTCTCGTCGATGCCGAGCGACGCATCGAGACGAGGATCGCCAGTCGGCTGCCTATGTCGATCCGAGAACAGTTGCTGGCATTGCTCGAGGAGACGGC
TGATGATCGGGTGACCCGTTTTGTGTGGCTGCGCCAGTTCGAGCCTGGCTCGAACTCTTCGTCGGCCAACCGGCTGCTCGACCGGCTCGAATATCTGCAACGCGTCGATCTCCCCGAGGATC
TGCTTGCCGGCGTTCCTGCCCATCGGGTGACTCGTCTGCGCAGGCAGGGTGAACGGTATTATGCCGACGGCATGCGCGATCTCCCGGAGGACAGGCGGCTTGCGATCTTGGCTGTTTGCG
TCTCGGAATGGCAGGCGATGTTGGCCGACGCAGTGGTCGAAACCCACGACCGGATCGTCGGCCGTCTCTACCGTGCTTCGGAGCGTATTTGCCATGCAAAGGTCGCAGACGAAGCGGGG
GTGGTGCGTGACACCCTGAAATCCTTCGCCGAGATCGGGGGCGCCCTGGTCGATGCACAGGATGATGGCCAGCCGCTGGGCGATGTCATCGCGAGGGCACTGTTGCAAAGTTAG
>BDIOMP_08200 IS6 family IS15DII transposase
ATGAACCCATTCAAAGGCCGGCATTTTCAGCGTGACATCATTCTGTGGGCCGTACGCTGGTACTGCAAATACGGCATCAGTTACCGTGAGCTGCAGGAGATGCTGGCTGAACGCGGAGTGA
ATGTCGATCACTCCACGATTTACCGCTGGGTTCAGCGTTATGCGCCTGAAATGGAAAAACGGCTGCGCTGGTACTGGCGTAACCCTTCCGATCTTTGCCCGTGGCACATGGATGAAACCTAC
GTGAAGGTCAATGGCCGCTGGGCGTATCTGTACCGGGCCGTCGACAGCCGGGGCCGCACTGTCGATTTTTATCTCTCCTCCCGTCGTAACAGCAAAGCTGCATACCGGTTTCTGGGTAAAAT
CCTCAACAACGTGAAGAAGTGGCAGATCCCGCGATTCATCAACACGGATAAAGCGCCCGCCTATGGTCGCGCGCTTGCTCTGCTCAAACGCGAAGGCCGGTGCCCGTCTGACGTTGAACA
CCGACAGATTAAGTACCGGAACAACGTGATTGAATGCGATCATGGCAAACTGAAACGGATAATCGGCGCCACGCTGGGATTTAAATCCATGAAGACGGCTTACGCCACCATCAAAGGTATT
GAGGTGATGCGTGCACTACGCAAAGGCCAGGCCTCAGCATTTTATTATGGTGATCCCCTGGGCGAAATGCGCCTGGTAAGCAGAGTTTTTGAAACGTAA
>BDIOMP_08205 aminoglycoside O-phosphotransferase APH(3'')-Ib
TTGAATCGAACTAATATTTTTTTTGGTGAATCGCATTCTGACTGGTTGCCTGTCAGAGGCGGAGAATCTGGTGATTTTGTTTTTCGACGTGGTGACGGGCATGCCTTCGCGAAAATCGCACCT
GCTTCCCGCCGCGGTGAGCTCGCTGGAGAGCGTGACCGCCTCATTTGGCTCAAAGGTCGAGGTGTGGCTTGCCCCGAGGTGATCAACTGGCAGGAGGAACAGGAGGGTGCATGCTTGGT
GATAACGGCAATTCCGGGAGTACCGGCGGCTGATCTGTCTGGAGCGGATTTGCTCAAAGCGTGGCCGTCAATGGGGCAGCAACTTGGCGCTGTTCACAGCCTATCGGTTGATCAATGTCCG
TTTGAGCGCAGGCTGTCGCGAATGTTCGGACGCGCCGTTGATGTGGTGTCCCGCAATGCCGTCAATCCCGACTTCTTACCGGACGAGGACAAGAGTACGCCGCAGCTCGATCTTTTGGCTC
GTGTCGAACGAGAGCTACCGGTGCGGCTCGACCAAGAGCGCACCGATATGGTTGTTTGCCATGGTGATCCCTGCATGCCGAACTTCATGGTGGACCCTAAAACTCTTCAATGCACGGGTCT
GATCGACCTTGGGCGGCTCGGAACAGCAGATCGCTATGCCGATTTGGCACTCATGATTGCTAACGCCGAAGAGAACTGGGCAGCGCCAGATGAAGCAGAGCGCGCCTTCGCTGTCCTATTC
AATGTATTGGGGATCGAAGCCCCCGACCGCGAACGCCTTGCCTTCTATCTGCGATTGGACCCTCTGACTTGGGGTTGA
>BDIOMP_08210 aminoglycoside O-phosphotransferase APH(6)-Id
ATGTTCATGCCGCCTGTTTTTCCTGCTCATTGGCACGTTTCGCAACCTGTTCTCATTGCGGACACCTTTTCCAGCCTCGTTTGGAAAGTTTCATTGCCAGACGGGACTCCTGCAATCGTCAAG
GGATTGAAACCTATAGAAGACATTGCTGATGAACTGCGCGGGGCCGACTATCTGGTATGGCGCAATGGGAGGGGAGCAGTCCGGTTGCTCGGTCGTGAGAACAATCTGATGTTGCTCGAA
TATGCCGGGGAGCGAATGCTCTCTCACATCGTTGCCGAGCACGGCGACTACCAGGCGACCGAAATTGCAGCGGAACTAATGGCGAAGCTGTATGCCGCATCTGAGGAACCCCTGCCTTCTG
CCCTTCTCCCGATCCGGGATCGCTTTGCAGCTTTGTTTCAGCGGGCGCGCGATGATCAAAACGCAGGTTGTCAAACTGACTACGTCCACGCGGCGATTATAGCCGATCAAATGATGAGCAAT
GCCTCGGAACTGCGTGGGCTACATGGCGATCTGCATCATGAAAACATCATGTTCTCCAGTCGCGGCTGGCTGGTGATAGATCCCGTCGGTCTGGTCGGTGAAGTGGGCTTTGGCGCCGCCA
ATATGTTCTACGATCCGGCTGACAGAGACGACCTTTGTCTCGATCCTAGACGCATTGCACAGATGGCGGACGCATTCTCTCGTGCGCTGGACGTCGATCCGCGTCGCCTGCTCGACCAGGCG
TACGCTTATGGGTGCCTTTCCGCAGCTTGGAACGCGGATGGAGAAGAGGAGCAACGCGATCTAGCTATCGCGGCCGCGATCAAGCAGGTGCGACAGACGTCATACTAG
>BDIOMP_08215 Micrococcal nuclease-like protein
ATGAAAATATTCACCTTATTAACATTCACAATCGCCATATCGTCGCCCTCTTTACTGATTGCAGCGGAATTAAGCGGCAAAGTAACACGCGTTCTCGACGGCGACACCATCGAGGTACTACAGG
ATAAAACGCCTGTTAGAGTTCGCCTTGCTAACATCGATGCGCCGGAAAAGAAACAGCCCTTTGGGAGCTGGTCCAGTAAACAGCTTAAAAGTCTGATAGCAGGGCAGCCTGTTACCGTTAC
ATATATGCAAAAAGACCGCTACGGACGTGTTATCGGGCGCGTCTTCACGGCGAACGGTACGGAATCAAACCGCTTTATGGTGCAATCCGGCGCTGCATGGGTATATGACCACTTCAACACAG
ATACTTCCTTGCCTGGTCTGCAACGGGATGCTCAAGAGCAAAAGCGTGGTCTATGGGCTGACAGTCAACCAGCGCCGCCGTGGGAATGGCGTCACAATCATTAG
>BDIOMP_08220 hypothetical protein
ATGAATAATTCTGAGAAAGAGCGTTTTAACAATCGGGTTTGTGTGGGGCAGGTACGTATCAGCGCAGACGTTTATATAAACTCCGGTATGTCTGAAAGCGCGGCAGAAGTGATGCATTCAAA
AATGAATTTAATAAGTATGAAGTTCTAA
>BDIOMP_08225 Tetratricopeptide repeat protein
ATGAGAAATGGTTTTGTCAGCGATGAACAATATCAGCACTCATTGGCCGTTTTTAAAGAGCTTCTTGATGCCGATGTTTATGAAAGCGGTAATAAAAAAATTATCGAAAAGTTCGAATCGGCT
TTAGATATTTGTTTGGCTTATGAACGTCAATGCTCTAGTCAGGATTTACATAAGTCTCTCATAAGTTCTTATTCAGTCTTGCAGCCCAAGCGGGCTGCTTATCCGATTATATCCATATATTGA
>BDIOMP_08230 Omega-protein
ATGAGACTCTTTATTGCTGAAAAACCATCTTTAGCTAAAGCCATTTTCGAAGGATTAGGTGGCAACCCTGCTACTGAAAAGAAAAATGGTTGTTATGAACATGGAACTGATGTTGTCACGTGG
TGTTTTGGTCATATGCTTGAGCTTTATGATCCTCAAGACTATGACGTGAAATACGCCGCCTGGCGTTTTGATGACCTGCCGATCAAAACGCCCTGGCCGCCCAAGTACAAAATCAGAGCCGAT
GCACAGCAGCAAACTAACATCATCTTTTCTCTCATCGAGAAAGCGACCAGTATCGTTCATGCCGGAGACCCGGACGATGAAGGCTGCCTGTTGGTTGATGAAATTTTAGATTACGCAAAAAA
TACAAAACCCGTTCAGCGCCTTCTCGTTGCAGATCTCAACCTCGCTCCCGTTCAGAAGGCTCTCGCCAATATGCAGCCTAACGAGAAATTCAGGGGCATGACAAATAGCGCTCTGGCACGCT
CTCTATGCGATCAGGGATTCGGCTACAACCTTACGCGCGGCTGTACCCTTAAAGGGCAGGAGAAAGGCTTTCATGGCGTCCTGAATGTTGGTCGTGTGCAAAGCGCTGTTCTTGGGCTGGT
TAACCAGAGAACTCTGGCTAACCAGAACCACACAGAAAGCTTTTATTATGATGTTCAAGCGGCATTGTCGATGAACGGGCATCTCTTAAAGGCCAAATATCAAGTCGCTGAAGGTGATGAAA
AGGATGAAAAAAATCGTCTGATCTCTGAGGCTCAGGCGAAGGCGGTTGTTGAGCATGTCACGGGTAAAAAAGCTGTTATTTCTGAAACGGCCACCAAACCCGAACACACTAAACCGCCTAT
GCCGCTGAACCTTTCGACACTGCAACAAATCTGCGCAAGACGTTTCGGTTATAAGGCAAAAGAAACGCTCGATATCATGCAAGGTCTTTACGAAACTCATAAGCTTTTGACCTATCCGCGAA
CAGATAACCGCTATCTGTCTGACGAGCATTTCACTCAGGCTGGCGATATTGCCGACGCTATCGGCGCGACGTTACCAGAGCTGGCGACCGCGACCGCTGGCATGGATAAAACGCAAAAACA
TAAAGCTTTTAACGCAAGCAAAATTGAGGCTCACCACGCTATTATCCCGACCACTAAAAGCGGGAAAGGCGTTCAGCTTAATGAGGGGAGCCCGCAGAATTCGGAAAAAATCGTACGCTAA
>BDIOMP_08235 DDE-Tnp-Tn3 domain-containing protein
ATGTTCATACTCTTCGCCGAGCGAAAAGTTGGGGAACAGCACGGGCCGGCCGCGCAAGGCGTGCTGGCAGCCGTGCAGACCCTGCGTGAGATGAACGCCGACAACCTGCGCAAGGTGC
CGGCCGATGCACCCACGGCCTTCATCAAGCCGCGCTGGAAGCCGCTGGTGATCACCCCGGAAGGCCTCGACCGGAAATTCTACGAAATCTGCGCCCTGTCCGAGCTGAAGAACGCCCTGC

GCTCCGGCGACATCTGGGTCAAGGGCTCGCGGCAGTTCCGCGACTTCGACGACTACCTGCTGCCGGCCGAGAAGTTCGCCGCACTCAAGCGCGAGCAGGCCCTGCCCCTGGCGATCAAC
CCGAACAGCGACCAGTACCTGGAAGAGCGTTTGCAGCTGCTGGACGAGCAGTTGGCCACCGTCACCCGCCTGGCCAAGGACAACGAGCTGCCCGATGCCATCCTCACCGAGTCAGGGCT
GAAAATCACCCCGCTGGATGCGGCGGTGCCGGATCGGGCGCAGGCGCTGATCGACCAAACCAGCCAGTTACTGCCGCGCATCAAGATCACCGAACTGCTGATGGACGTGGACGACTGGA
CGGGCTTCAGCCGCCACTTCACCCACTTGAAGGACGGGGCCGAGGCCAAAGACAGGACGTTGCTGCTGTCCGCAATCCTCGGTGATGCGATCAACCTCGGGCTGACCAAGATGGCCGAG
TCGAGCCCCGGCCTGACCTACGCCAAGCTGTCCTGGCTGCAAGCCTGGCACATCCGCGACGAAACCTATTCGGCGGCCTTGGCCGAGCTGGTCAACCACCAGTATCGCCACGCCTTTGCCG
CCCACTGGGGCGACGGCACGACCTCATCCTCCGATGGCCAGCGCTTCCGCGCGGGTGGCCGGGGCGAGAGCACCGGGCACGTCAACCCGAAGTACGGTAGCGAGCCGGGACGGCTGTT
CTATACCCATATCTCCGACCAGTACGCGCCGTTCAGCACCCGCGTGGTGAATGTCGGCGTCCGCGATTCCACCTATGTGCTCGACGGCCTGCTGTACCACGAGTCCGACCTGCGGATCGAGG
AGCACTACACCGACACGGCCGGCTTCACCGATCACGTCTTTGCCCTGATGCACCTGCTAGGCTTCCGCTTCGCGCCGCGCATCCGCGACCTCGGCGAAACCAAGCTGTACGTGCCGCAGGG
CGTGCAAGCCTACCCGACGTTGCGCCCGCTGATCGGCGGCACCCTGAACATCAAGCACGTGCGTGCCCACTGGGACGACATCCTGCGCCTGGCCAGCTCGATCAAGCAGGGCACCGTCAC
CGCCTCGCTGATGCTGCGCAAGCTCGGCAGCTACCCGCGCCAGAACGGACTGGCCGTGGCCCTGCGCGAGCTGGGCCGGATCGAGCGCACGCTGTTCATCCTGGACTGGCTGCAAAGTG
TTGAACTGCGCCGCCGCGTGCATGCCGGCCTGAACAAAGGTGAGGCGCGCAACTCGCTGGCCAGGGCGGTGTTCTTCAACCGCCTTGGGGAAATCAGGGATCGGAGCTTCGAGCAGCA
GCGCTACCGGGCCAGCGGCCTCAACCTGGTGACGGCGGCTATCGTGCTGTGGAACACGGTGTACCTGGAACGCGCCACCCAGGGGTTGGTCGAGGCCGGCAAGCCGGTGGACGGCGAG
CTGCTGCAATTCCTGTCGCCGCTGGGCTGGGAGCACATCAACCTAACCGGCGATTACGTCTGGCGGCAGAGCCGCAGACTGGAAGACGGGAAGTTTCGGCCCTTACGGATGCCCGGAAA
ACCTTAG
>BDIOMP_08240 EamA family transporter
ATGTCATTACGCACGCCCGATTTATTGTTCACAGCGATAGCACCTGCCATTTGGGGCAGCACCTACATTGTCACCACCCAATACCTGCCGAACTTCTCACCGATGACGGTCGCGATGCTGCGG
GCGTTGCCGGCGGGTTTATTGCTCGTGATGATCGTCCGACAGATTCCAACGGGAATCTGGTGGATGCGCATCTTCATCCTCGGCGCACTTAATATTTCGCTATTCTGGAGCTTGTTGTTTATTT
CGGTCTACCGCCTGCCGGGCGGGGTCGCGGCGACGGTAGGCGCTGTGCAGCCGCTGATGGTCGTGTTCATCTCTGCCGCTCTGCTAGGTAGCCCGATACGATTGATGGCGGTCCTGGGGG
CTATTTGCGGAACTGCGGGCGTGGCGCTGTTGGTGTTGACACCAAACGCAGCGCTAGATCCTGTCGGCGTCGCAGCGGGCCTGGCGGGGGCGGTTTCCATGGCGTTCGGAACCGTGCTG
ACCCGCAAGTGGCAACCTCCCGTGCCTCTGCTCACCTTTACCGCCTGGCAACTGGCGGCCGGAGGACTTCTGCTCGTTCCAGTAGCTTTAGTGTTTGATCCGCCAATCCCGATGCCTACAGG
AACCAATGTTCTCGGCCTGGCGTGGCTCGGCCTGATCGGAGCGGGTTTAACCTACTTCCTTTGGTTCCGGGGGATCTCGCGACTCGAACCTACAGTTGTTTCCTTACTGGGCTTTCTCAGCC
CGGGGACCGCCGTGTTGCTAGGATGGTTGTTCTTGGATCAGACGCTGAGTGCGCTTCAAATCATCGGCGTCCTGCTCGTGATCGGGAGTATCTGGCTGGGCCAACGTTCCAACCGCACTCC
TAGGGCGCGTATAGCTTGCCGGAAGTCGCCTTGA
>BDIOMP_08245 tetracycline efflux MFS transporter Tet(A)
GTGAAACCCAACAGACCCCTGATCGTAATTCTGAGCACTGTCGCGCTCGACGCTGTCGGCATCGGCCTGATTATGCCGGTGCTGCCGGGCCTCCTGCGCGATCTGGTTCACTCGAACGACGT
CACCGCCCACTATGGCATTCTGCTGGCGCTGTATGCGTTGATGCAATTTGCCTGCGCACCTGTGCTGGGCGCGCTGTCGGATCGTTTCGGGCGGCGGCCGGTCTTGCTCGTCTCGCTGGCCG
GCGCTGCTGTCGACTACGCCATCATGGCGACGGCGCCTTTCCTTTGGGTTCTCTATATCGGGCGGATCGTGGCCGGCATCACCGGGGCGACTGGGGCGGTAGCCGGCGCTTATATTGCCGA
TATCACTGATGGCGATGAGCGCGCGCGGCACTTCGGCTTCATGAGCGCCTGTTTCGGGTTCGGGATGGTCGCGGGACCTGTGCTCGGTGGGCTGATGGGCGGTTTCTCCCCCCACGCTCC
GTTCTTCGCCGCGGCAGCCTTGAACGGCCTCAATTTCCTGACGGGCTGTTTCCTTTTGCCGGAGTCGCACAAAGGCGAACGCCGGCCGTTACGCCGGGAGGCTCTCAACCCGCTCGCTTC
GTTCCGGTGGGCCCGGGGCATGACCGTCGTCGCCGCCCTGATGGCGGTCTTCTTCATCATGCAACTTGTCGGACAGGTGCCGGCCGCGCTTTGGGTCATTTTCGGCGAGGATCGCTTTCAC
TGGGACGCGACCACGATCGGCATTTCGCTTGCCGCATTTGGCATTCTGCATTCACTCGCCCAGGCAATGATCACCGGCCCTGTAGCCGCCCGGCTCGGCGAAAGGCGGGCACTCATGCTCG
GAATGATTGCCGACGGCACAGGCTACATCCTGCTTGCCTTCGCGACACGGGGATGGATGGCGTTCCCGATCATGGTCCTGCTTGCTTCGGGTGGCATCGGAATGCCGGCGCTGCAAGCAAT
GTTGTCCAGGCAGGTGGATGAGGAACGTCAGGGGCAGCTGCAAGGCTCACTGGCGGCGCTCACCAGCCTGACCTCGATCGTCGGACCCCTCCTCTTCACGGCGATCTATGCGGCTTCTATA
ACAACGTGGAACGGGTGGGCATGGATTGCAGGCGCTGCCCTCTACTTGCTCTGCCTGCCGGCGCTGCGTCGCGGGCTTTGGAGCGGCGCAGGGCAACGAGCCGATCGCTGA
>BDIOMP_08250 tetracycline resistance transcriptional repressor TetR(A)
ATGTTTATCAGTGATAAAGTGTCAAGCATGACAAAGTTGCAGCCGAATACAGTGATCCGTGCCGCCCTGGACCTGTTGAACGAGGTCGGCGTAGACGGTCTGACGACACGCAAACTGGCG
GAACGGTTGGGGGTTCAGCAGCCGGCGCTTTACTGGCACTTCAGGAACAAGCGGGCGCTGCTCGACGCACTGGCCGAAGCCATGCTGGCGGAGAATCATACGCATTCGGTGCCGAGAGC
CGACGACGACTGGCGCTCATTTCTGATCGGGAATGCCCGCAGCTTCAGGCAGGCGCTGCTCGCCTACCGCGATGGCGCGCGCATCCATGCCGGCACGCGACCGGGCGCACCGCAGATGG
AAACGGCCGACGCGCAGCTTCGCTTCCTCTGCGAGGCGGGTTTTTCGGCCGGGGACGCCGTCAATGCGCTGATGACAATCAGCTACTTCACTGTTGGGGCCGTGCTTGAGGAGCAGGCC
GGCGACAGCGATGCCGGCGAGCGCGGCGGCACCGTTGAACAGGCTCCGCTCTCGCCGCTGTTGCGGGCCGCGATAGACGCCTTCGACGAAGCCGGTCCGGACGCAGCGTTCGAGCAGG
GACTCGCGGTGATTGTCGATGGATTGGCGAAAAGGAGGCTCGTTGTCAGGAACGTTGAAGGACCGAGAAAGGGTGACGATTGA
>BDIOMP_08255 Relaxase
GTGAAGACGGCCGCCCTGGACCTCGCCCGCGAGCGCCAGGCGCACGAGGCCGGCGCGCGGACCCGCGCCACGGCCCACGAGCGGACGCCGCAGCAGGAGCGCCAGAAGGCCGCCAG
AGAGGCCGAGCGCGGCCGTGAGGCTTGGACGCTAGGGCAGGGCATGAAAAAGCCCGTAGCGGGCTGCTACGGGCGTCTGACGCGGTGGAAAGGGGGAGGGGATGTTGTCTACATGGC
TCTGCTGTAG
>BDIOMP_08260 Transposase for transposon Tn21
ATGCCACGTCGTTCCATCCTGTCCGCCGCCGAGCGGGAAAGCCTGCTGGCGTTGCCGGACTCCAAGGACGACCTGATCCGACATTACACATTCAACGATACCGACCTCTCGATCATCCGACA
GCGGCGCGGGCCAGCCAATCGGCTGGGCTTCGCGGTGCAGCTCTGTTACCTGCGCTTTCCCGGCGTCATCCTGGGCGTCGATGAACTACCGTTCCCGCCCTTGTTGAAGCTGGTCGCCGAC
CAGCTCAAGGTCGGCGTCGAAAGCTGGAACGAGTACGGCCAGCGGGAGCAGACCCGGCGCGAGCACCTGAGCGAGCTGCAAACCGTGTTCGGTTTCCGGCCCTTCACCATGAGCCATTA
CCGGCAGGCCGTCCAGATGCTGACCGAGCTGGCGATGCAAACCGACAAAGGCATCGTGCTGGCCAGCGCCTTGATCGGGCACCTGCGGCGGCAGTCGGTCATTCTGCCCGCCCTCAACG
CCGTCGAGCGGGCGAGTGCCGAGGCGATCACCCGTGCTAACCGGCGCATCTACGACGCCTTGGCCGAACCACTGGCGGACGCGCATCGCCGCCGCCTCGACGATCTGCTCAAGCGCCGG
GACAACGGCAAGACGACCTGGTTGGCTTGGTTGCGCCAGTCTCCGGCCAAGCCAAATTCGCGGCATATGCTGGAACACATCGAACGCCTCAAGGCATGGCAGGCACTCGATCTGCCTACC
GGCATCGAGCGGCTGGTTCACCAGAACCGCCTGCTCAAGATTGCCCGCGAGGGCGGCCAGATGACACCCGCCGACCTGGCCAAATTCGAGCCGCAACGGCGCTACGCCACTCTCGTGGC
GCTGGCCACCGAGGGCATGGCCACCGTCACCGACGAAATCATCGACCTGCACGACCGCATCCTGGGTAAGCTGTTTAACGCTGCCAAGAATAAGCATCAGCAGCAGTTCCAGGCGTCAGG
CAAGGCCATCAACGCCAAGGTACGTCTGTACGGGCGCATCGGTCAGGCGCTGATCGACGCCAAGCAATCAGGCCGCGATGCGTTTGCCGCCATCGAGGCCGTCATGTCCTGGGATTCCTTT
GCCGAGAGCGTCACCGAGGCGCAGAAGCTCGCGCAACCCGATGACTTCGATTTCCTGCATCGCATCGGCGAGAGCTACGCCACCCTGCGCCGCTATGCACCGGAATTCCTTGCCGTGCTCA
AGCTGCGGGCCGCGCCCGCCGCCAAAAACGTGCTTGATGCCATTGAGGTGCTGCGCGGCATGAACACCGACAACGCCCGCAAGCTGCCAGCCGATGCACCGACCGGCTTCATCAAGCCG
CGCTGGCAGAAACTGGTGATGACCGACGCCGGCATCGACCGGCGCTACTACGAACTGTGCGCGCTGTCCGAGTTGAAGAACTCCCTGCGCTCGGGCGACATCTGGGTGCAGGGTTCACG
CCAGTTCAAGGACTTCGAGGACTACCTGGTACCGCCCGAGAAGTTCACCAGCCTCAAGCAGTCCAGCGAATTGCCGCTGGCCGTGGCCACCGACTGCGAACAATATCTGCATGAGCGGCT
GACGCTGCTGGAAGCACAACTTGCCACCGTCAACCGCATGGCGGCAGCCAACGACCTGCCGGATGCCATCATCACCGAGTCGGGCTTGAAGATCACGCCGCTGGATGCGGCGGTGCCCG
ACACCGCGCAGGCGCTGATAGACCAGACAGCCATGGTCCTGCCGCACGTCAAGATCACCGAACTGCTGCTCGAAGTCGATGAGTGGACGGGCTTCACCCGGCACTTCACGCACTTGAAAT
CGGGCGATCTGGCCAAGGACAAGAACCTGTTGTTGACCACGATCCTGGCCGACGCGATCAACCTGGGCCTGACCAAGATGGCCGAGTCCTGCCCCGGCACGACCTACGCGAAGCTCGCTT
GGCTGCAAGCCTGGCATACCCGCGACGAAACGTACTCGACAGCGTTGGCTGAACTGGTCAACGCTCAGTTTCGGCATCCCTTTGCCGGGCACTGGGGCGATGGCACCACATCATCATCGG
ACGGACAGAATTTCCGAACCGCTAGCAAGGCAAAGAGCACGGGGCACATCAACCCAAAATATGGCAGCAGCCCAGGACGGACTTTCTACACCCACATCTCCGACCAATACGCGCCATTCCA
CACCAAGGTGGTCAATGTCGGCCTGCGCGACTCAACCTACGTGCTCGACGGCCTGCTGTACCACGAGTCCGACCTGCGGATCGAGGAGCACTACACCGACACGGCCGGCTTCACCGATCA
CGTCTTTGCCCTGATGCACCTGCTAGGCTTCCGCTTCGCGCCGCGCATCCGCGACCTCGGCGAAACCAAGCTGTACGTGCCGCAGGGCGTGCAAGCCTACCCGACGTTGCGCCCGCTGATC
GGCGGCACCCTGAACATCAAGCACGTGCGTGCCCACTGGGACGACATCCTGCGCCTGGCCAGCTCGATCAAGCAGGGCACCGTCACCGCCTCGCTGATGCTGCGCAAGCTCGGCAGCTAC
CCGCGCCAGAACGGACTGGCCGTGGCCCTGCGCGAGCTGGGCCGGATCGAGCGCACGCTGTTCATCCTGGACTGGCTGCAAAGTGTTGAACTGCGCCGCCGCGTGCATGCCGGCCTGAA
CAAAGGTGAGGCGCGCAACTCGCTGGCCAGGGCGGTGTTCTTCAACCGCCTTGGGGAAATCAGGGATCGGAGCTTCGAGCAGCAGCGCTACCGGGCCAGCGGCCTCAACCTGGTGACG

GCGGCTATCGTGCTGTGGAACACGGTGTACCTGGAACGCGCCACCCAGGGGTTGGTCGAGGCCGGCAAGCCGGTGGACGGCGAGCTGCTGCAATTCCTGTCGCCGCTGGGCTGGGAGC
ACATCAACCTAACCGGCGATTACGTCTGGCGGCAGAGCCGCAGACTGGAAGACGGGAAGTTTCGGCCCTTACGGATGCCCGGAAAACCTTAG

AR-encoding Tn 1.2

>BDIOMP_08195 DUF4158 domain-containing protein
ATGCCGCGTCGCGTCACTCTAACCGATCGGCAGAAAGACGCGCTGTTGCGCTTGCCGACTTCACAGACGGATTTGCTCAAGCACTATACGCTGAGTGATGAAGACTTTGGGCATATCAGGC
TGCGTCGGCGCGCTCACAACAGGTTCGGCTTCGCCCTGCAATTGTGTGTCCTGCGCTATCCCGGCCGGGTGCTGGCTCCAGGCGAACTGATCCCTGCAGAGGTCATCGAATTTATCGGAGC
GCAGCTTGGCCTGGGTGCCGACGATCTCGTAGACTATGCTGCCCGCGAGGAAACACGGCACGAGCATCTTGCCGAGTTACGGGGGCTCTACGGCTTCCGCACCTTCTCCGGACGTGGTGC
GAGCGAGCTGAAGGAATGGTTGTTCCGAGAAGCCGAGATGGCGGTGTCGAACGAGGATATCGCCCGTCGCTTCGTAGCCGAGTGCCGACGCACCCGCACTGTCCTTCCCGCGACATCCAC
GATCGAGCGGCTTTGTGCCGCGGCTCTCGTCGATGCCGAGCGACGCATCGAGACGAGGATCGCCAGTCGGCTGCCTATGTCGATCCGAGAACAGTTGCTGGCATTGCTCGAGGAGACGGC
TGATGATCGGGTGACCCGTTTTGTGTGGCTGCGCCAGTTCGAGCCTGGCTCGAACTCTTCGTCGGCCAACCGGCTGCTCGACCGGCTCGAATATCTGCAACGCGTCGATCTCCCCGAGGATC
TGCTTGCCGGCGTTCCTGCCCATCGGGTGACTCGTCTGCGCAGGCAGGGTGAACGGTATTATGCCGACGGCATGCGCGATCTCCCGGAGGACAGGCGGCTTGCGATCTTGGCTGTTTGCG
TCTCGGAATGGCAGGCGATGTTGGCCGACGCAGTGGTCGAAACCCACGACCGGATCGTCGGCCGTCTCTACCGTGCTTCGGAGCGTATTTGCCATGCAAAGGTCGCAGACGAAGCGGGG
GTGGTGCGTGACACCCTGAAATCCTTCGCCGAGATCGGGGGCGCCCTGGTCGATGCACAGGATGATGGCCAGCCGCTGGGCGATGTCATCGCGAGGGCACTGTTGCAAAGTTAG
>BDIOMP_08200 IS6 family IS15DII transposase
ATGAACCCATTCAAAGGCCGGCATTTTCAGCGTGACATCATTCTGTGGGCCGTACGCTGGTACTGCAAATACGGCATCAGTTACCGTGAGCTGCAGGAGATGCTGGCTGAACGCGGAGTGA
ATGTCGATCACTCCACGATTTACCGCTGGGTTCAGCGTTATGCGCCTGAAATGGAAAAACGGCTGCGCTGGTACTGGCGTAACCCTTCCGATCTTTGCCCGTGGCACATGGATGAAACCTAC
GTGAAGGTCAATGGCCGCTGGGCGTATCTGTACCGGGCCGTCGACAGCCGGGGCCGCACTGTCGATTTTTATCTCTCCTCCCGTCGTAACAGCAAAGCTGCATACCGGTTTCTGGGTAAAAT
CCTCAACAACGTGAAGAAGTGGCAGATCCCGCGATTCATCAACACGGATAAAGCGCCCGCCTATGGTCGCGCGCTTGCTCTGCTCAAACGCGAAGGCCGGTGCCCGTCTGACGTTGAACA
CCGACAGATTAAGTACCGGAACAACGTGATTGAATGCGATCATGGCAAACTGAAACGGATAATCGGCGCCACGCTGGGATTTAAATCCATGAAGACGGCTTACGCCACCATCAAAGGTATT
GAGGTGATGCGTGCACTACGCAAAGGCCAGGCCTCAGCATTTTATTATGGTGATCCCCTGGGCGAAATGCGCCTGGTAAGCAGAGTTTTTGAAACGTAA
>BDIOMP_08205 aminoglycoside O-phosphotransferase APH(3'')-Ib
TTGAATCGAACTAATATTTTTTTTGGTGAATCGCATTCTGACTGGTTGCCTGTCAGAGGCGGAGAATCTGGTGATTTTGTTTTTCGACGTGGTGACGGGCATGCCTTCGCGAAAATCGCACCT
GCTTCCCGCCGCGGTGAGCTCGCTGGAGAGCGTGACCGCCTCATTTGGCTCAAAGGTCGAGGTGTGGCTTGCCCCGAGGTGATCAACTGGCAGGAGGAACAGGAGGGTGCATGCTTGGT
GATAACGGCAATTCCGGGAGTACCGGCGGCTGATCTGTCTGGAGCGGATTTGCTCAAAGCGTGGCCGTCAATGGGGCAGCAACTTGGCGCTGTTCACAGCCTATCGGTTGATCAATGTCCG
TTTGAGCGCAGGCTGTCGCGAATGTTCGGACGCGCCGTTGATGTGGTGTCCCGCAATGCCGTCAATCCCGACTTCTTACCGGACGAGGACAAGAGTACGCCGCAGCTCGATCTTTTGGCTC
GTGTCGAACGAGAGCTACCGGTGCGGCTCGACCAAGAGCGCACCGATATGGTTGTTTGCCATGGTGATCCCTGCATGCCGAACTTCATGGTGGACCCTAAAACTCTTCAATGCACGGGTCT
GATCGACCTTGGGCGGCTCGGAACAGCAGATCGCTATGCCGATTTGGCACTCATGATTGCTAACGCCGAAGAGAACTGGGCAGCGCCAGATGAAGCAGAGCGCGCCTTCGCTGTCCTATTC
AATGTATTGGGGATCGAAGCCCCCGACCGCGAACGCCTTGCCTTCTATCTGCGATTGGACCCTCTGACTTGGGGTTGA
>BDIOMP_08210 aminoglycoside O-phosphotransferase APH(6)-Id
ATGTTCATGCCGCCTGTTTTTCCTGCTCATTGGCACGTTTCGCAACCTGTTCTCATTGCGGACACCTTTTCCAGCCTCGTTTGGAAAGTTTCATTGCCAGACGGGACTCCTGCAATCGTCAAG
GGATTGAAACCTATAGAAGACATTGCTGATGAACTGCGCGGGGCCGACTATCTGGTATGGCGCAATGGGAGGGGAGCAGTCCGGTTGCTCGGTCGTGAGAACAATCTGATGTTGCTCGAA
TATGCCGGGGAGCGAATGCTCTCTCACATCGTTGCCGAGCACGGCGACTACCAGGCGACCGAAATTGCAGCGGAACTAATGGCGAAGCTGTATGCCGCATCTGAGGAACCCCTGCCTTCTG
CCCTTCTCCCGATCCGGGATCGCTTTGCAGCTTTGTTTCAGCGGGCGCGCGATGATCAAAACGCAGGTTGTCAAACTGACTACGTCCACGCGGCGATTATAGCCGATCAAATGATGAGCAAT
GCCTCGGAACTGCGTGGGCTACATGGCGATCTGCATCATGAAAACATCATGTTCTCCAGTCGCGGCTGGCTGGTGATAGATCCCGTCGGTCTGGTCGGTGAAGTGGGCTTTGGCGCCGCCA
ATATGTTCTACGATCCGGCTGACAGAGACGACCTTTGTCTCGATCCTAGACGCATTGCACAGATGGCGGACGCATTCTCTCGTGCGCTGGACGTCGATCCGCGTCGCCTGCTCGACCAGGCG
TACGCTTATGGGTGCCTTTCCGCAGCTTGGAACGCGGATGGAGAAGAGGAGCAACGCGATCTAGCTATCGCGGCCGCGATCAAGCAGGTGCGACAGACGTCATACTAG
>BDIOMP_08215 Micrococcal nuclease-like protein
ATGAAAATATTCACCTTATTAACATTCACAATCGCCATATCGTCGCCCTCTTTACTGATTGCAGCGGAATTAAGCGGCAAAGTAACACGCGTTCTCGACGGCGACACCATCGAGGTACTACAGG
ATAAAACGCCTGTTAGAGTTCGCCTTGCTAACATCGATGCGCCGGAAAAGAAACAGCCCTTTGGGAGCTGGTCCAGTAAACAGCTTAAAAGTCTGATAGCAGGGCAGCCTGTTACCGTTAC
ATATATGCAAAAAGACCGCTACGGACGTGTTATCGGGCGCGTCTTCACGGCGAACGGTACGGAATCAAACCGCTTTATGGTGCAATCCGGCGCTGCATGGGTATATGACCACTTCAACACAG
ATACTTCCTTGCCTGGTCTGCAACGGGATGCTCAAGAGCAAAAGCGTGGTCTATGGGCTGACAGTCAACCAGCGCCGCCGTGGGAATGGCGTCACAATCATTAG
>BDIOMP_08220 hypothetical protein
ATGAATAATTCTGAGAAAGAGCGTTTTAACAATCGGGTTTGTGTGGGGCAGGTACGTATCAGCGCAGACGTTTATATAAACTCCGGTATGTCTGAAAGCGCGGCAGAAGTGATGCATTCAAA
AATGAATTTAATAAGTATGAAGTTCTAA
>BDIOMP_08225 Tetratricopeptide repeat protein
ATGAGAAATGGTTTTGTCAGCGATGAACAATATCAGCACTCATTGGCCGTTTTTAAAGAGCTTCTTGATGCCGATGTTTATGAAAGCGGTAATAAAAAAATTATCGAAAAGTTCGAATCGGCT
TTAGATATTTGTTTGGCTTATGAACGTCAATGCTCTAGTCAGGATTTACATAAGTCTCTCATAAGTTCTTATTCAGTCTTGCAGCCCAAGCGGGCTGCTTATCCGATTATATCCATATATTGA

CRISPR_system

>BDIOMP_04635 Lactamase-B domain-containing protein
ATGGCGTTAAGGATCAGGGTATTGCTCGAAAATCATAAGGGAGCTGGAGCGGATAAATCGTTGAAGGCCCGGCCAGGATTAAGCCTGTTGGTCGAGGATGAGTCTACGTCGATCTTGTTCG
ATACCGGCCCTGATGGCAGTTTTATGCAAAACGCGTTGGCGATGGGGATCGACCTGTCCGATGTGTCTGCTGTGGTGCTTTCGCATGGTCATTACGATCATTGCGGCGGCGTGCCGTGGCTT
CCTGATAACAGTCGAATCATCTGCCATCCCGATATTGCGCGTGAACGTTATGCGGCAATGACTTTTCTCGGTATTACCCGGAAAATAAAAAAATTGTCGCGTGAGGTGGACTATTCACGCTACC
GAATGATGTACACGCGTGACCCCCTGCCGATTGGCGAAAACTTTATCTGGTCAGGGGAAATACCAGTGGTCGCGCCTGAAGCCTACGGCATTTTTGGCGGCCACGATGCGGAGCCGGACTC
TATTTTGGATGAGGGTGTACTGATTTACCAATCGGCAAAAGGATTAGTCATCATTACGGGATGCGGGCATCGGGGGATCGCAAATATTGTCCGGCACTGTCAGAATATTACCGGTATAAAGCG
GATTTACGCGCTTGTCGGCGGATTTCATCTCCGCTGCGCATCACCGTTCACGCTGTGGCGAGTCAGGCGCTTTCTGCAAGAACAAAAACCTGAAAAACTATGCGGTTGTCACTGTACAGGG
GCCTGGGGGCGGTTGTGGCTACCGGAAATAACCGCACCTGCAACCGGTGATGTGCTGCGCTTTTAA
>BDIOMP_04640 6-carboxy-5,6,7,8-tetrahydropterin synthase
ATGTCCACCACGTTGTATAAAGATTTCACCTTTGAAGCCGCTCACCGCCTGCCTCACGTGCCTGAAGGGCATAAGTGCGGCCGCCTGCACGGACACTCGTTTATGGTGCGTCTCGAAATTACC
GGTGAAGTCGATCCGCATACCGGTTGGATTATGGATTTCGCGGATCTCAAAGCGGCGTTTAAACCGACTTACGATCGTCTTGATCACTACTATCTGAATGATATTCCAGGGCTGTCCAATCCCA
CCAGCGAAGTGCTGGCGAAATGGATTTGGGATCAGGTTAAGCCCGTCGTGCCGCTACTGAGCGCCGTGATGGTGAAAGAAACCTGTACAGCCGGCTGCGTCTATCGCGGCGAATAA
>BDIOMP_04645 Sulfite reductase [NADPH] flavoprotein alpha-component

ATGACGACACCGGCTCCACTGACTGGGTTGCTTCCGCTTAATCCGGAGCAACTGGCGCGCCTTCAGGCCGCGACAACCGACCTTACTCCCGAACAGCTTGCCTGGGTCTCCGGCTATTTTT
GGGGCGTACTGAACCCGCGCTCAGGCGCTGTTGCCGTCACGCCTGCTCCTGAGGGGAAGATGCCGGGAGTTACGCTTATCTCCGCTTCGCAAACCGGCAATGCGCGTCGTGTGGCGGAA
GCGCTGCGTGATGATCTGCTGGCGGCCAACCTTAATGTGACACTGGTGAACGCCGGCGACTACAAATTCAAACAGATTGCCAGTGAAAAACTGTTGGTGATAGTGACGTCCACCCAGGGA
GAAGGCGAGCCGCCGGAAGAGGCCGTCGCGCTGCATAAATTCCTGTTCTCCAAAAAAGCGCCGAAACTGGAAAACACCGCTTTTGCCGTGTTTAGCCTCGGCGACACCTCTTATGAATTTT
TCTGCCAGTCCGGAAAAGATTTCGACAGCAAACTGGCCGAACTGGGCGGTGAACGTCTGCTCGACCGTGTCGATGCTGACGTTGAATATCAGGCAGCTGCCAGCGAATGGCGCGCCCGCG
TGGTTGATGTTCTGAAGTCCCGCTCACCTGTCGCGGCACCTTCGCAATCCGTCGCTACTGGCTCGGTAAACGACATCCACACCAGCCCCTATACCAAAGACGCGCCGCTGGCCGCGCCCCTC
TCCGTGAATCAGAAAATCACCGGTCGCAACTCGGAAAAAGATGTGCGCCATATTGAGATTGATTTAGGCGACTCCGGTCTGCGTTACCAGCCGGGCGATGCGCTGGGCGTCTGGTATCAGA
ACGATCCGGCTCTGGTGAAGGAGCTGGTTGAGTTGCTGTGGCTGAAGGGCGATGAGCCGGTGACGGTTGACGGTAAAACCCTGCCGCTCGCCGAAGCGCTGGAGTGGCACTTCGAGCT
GACGGTGAATACCGCCAACATTGTTGAGAACTACGCGACCCTGACGCGCAGTGAGTCGCTGTTGCCATTAGTGGGCGACAAAGCGCAGCTCCAGCATTACGCCGCGACCACGCCCATTGTT
GATATGGTGCGTTTCTCTCCGGCACAGCTGGATGCTCAGGCGCTAATCGACCTGCTGCGTCCGCTAACGCCGCGTCTGTACTCCATCGCCTCCGCACAGGCTGAAGTGGAGAGCGAAGTGC
ATATCACCGTTGGCGTGGTTCGTTATGACATTGAAGGCCGCGCCCGCGCCGGTGGCGCTTCCAGCTTCCTCGCCGACCGCGTGGAAGAAGAGGGCGAAGTTCGCGTCTTTATTGAGCATAA
CGATAACTTCCGCCTGCCAGCTAACCCGGAAACGCCGGTGATCATGATTGGCCCCGGTACCGGCATCGCGCCGTTCCGCTCCTTTATGCAACAGCGCGCGGCGGAAGGGGTGGAAGGTAA
GAACTGGTTGTTCTTCGGCAACCCGCACTTCACCGAAGACTTCCTTTATCAGGTTGAATGGCAGCGCTACGTGAAAGAGGGGGTGTTAAGCCGTATCGATCTCGCCTGGTCACGCGATCAA
AAAGAAAAAATTTACGTACAAGACAAGCTGCGTGAACAGGGCGCCGAGCTGTGGCGCTGGATCAATGATGGCGCGCATATTTATGTCTGCGGCGACGCCAGGCGTATGGCCGCAGATGTC
GAAAAAGCATTGCTGGAAGTGATCGCTGAATTCGGCGGTATGGACCTCGAATCGGCAGATGAATATTTAAGTGAGCTGCGCGTAGAGCGCCGTTATCAGCGAGATGTCTACTAA
>BDIOMP_04650 Putative sulfite reductase [NADPH] hemoprotein beta-component
ATGAGCGAAAAACATCCAGGCCCACTGGTGGTCGAAGGTAAACTGTCCGATGCCGAGCGCATGAAGCTGGAAAGCAACTACCTGCGCGGTACCATTGCGGAAGATCTGAACGACGGTCTC
ACCGGCGGTTTTAAAGGCGACAACTTCCTGCTAATACGCTTTCACGGCATGTATCAGCAGGATGACCGCGATATTCGCGCCGAGCGTGCGGCGCAGAAGCTGGAGCCGCGCCACGCCATG
CTGCTGCGCTGCCGCCTGCCGGGCGGGGTGATTACTACGACGCAATGGCAGGCGATCGATAAATTCGCGGCGGATAACACGATTTACGGCAGTATCCGTCTGACCAACCGCCAGACCTTCC
AGTTCCACGGCATTCTGAAGAAGAACGTTAAGCCGGTGCACCAGATGCTGCACTCGGTGGGGCTGGATGCGCTGGCAACCGCCAACGACATGAACCGTAACGTGCTGTGTACCTCTAACC
CGTACGAGTCTCAGCTGCATGCTGAAGCCTATGAATGGGCGAAGAAGATCTCCGAACATCTGCTGCCGCGCACCCGTGCGTATGCGGAGATCTGGCTCGATCAGGAGAAGGTCGCGACCA
CTGACGAAGAACCGATCCTCGGCGCAACCTATCTGCCGCGTAAATTTAAAACCACGGTGGTGATCCCGCCGCAAAATGATATCGATCTGCACGCCAACGACATGAACTTTGTGGCGATTGCC
GAGAACGGCAAGCTGGTCGGCTTTAACCTGCTGGTGGGCGGCGGTCTGTCTATTGAGCATGGTAATAAGAAAACCTACGCTCGTACGGCCAGCGAGTTTGGCTATCTGCCGCTGGAGCAC
ACCCTGGCGGTGGCCGAAGCGGTAGTGACGACGCAGCGCGACTGGGGTAACCGTACCGACAGGAAAAATGCCAAAACCAAATACACCCTTGAGCGCGTGGGACTGGAGACATTTAAAG
CGGAAGTCGAGCGTCGCGCGGGGATCAAATTTGAGCCGATCCGCCCGTATGAGTTTACCGGGCGTGGCGACAGGATTGGCTGGGTGAAAGGCATTGATGATAAATGGCACCTGACGCTGT
TTATTGAGAACGGCCGTATCCTGGATTATCCGGGGCGTCCGCTGAAAACCGGCCTGCTGGAGATCGCGAAGATTCATCAGGGCGAGTTCCGTATCACCGCTAACCAGAACCTGATTATTGCC
AGCGTGCCGGAAAGCCAGAAAGCGAAGATCGAGACGCTGGCGCGCGACCACGGGTTGATGAATGCGGTGAAACCGCAGCGTGAAAACTCAATGGCCTGCGTGTCGTTCCCAACCTGCCC
GCTGGCAATGGCGGAAGCCGAGCGTTTCCTGCCGTCGTTCACGGACAAAGTGGAAGCGATTCTGGAAAAACACGGCATTCCTGATGAGCATATCGTGATGCGCGTGACCGGCTGCCCGAA
CGGCTGTGGCCGCGCGATGCTGGCGGAAATTGGCTTAGTAGGGAAAGCGCCTGGTCGTTATAACCTGCATCTTGGTGGCAACCGCATCGGGAGTCGTATCCCACGGATGTATAAAGAAAAT
ATCGCCGAGCCGGATATTCTGGCCTCGCTGGATGAACTGATTGGGCGCTGGGCGAAAGAGCGTGAAGCGGGTGAAGGCTTCGGCGACTTTACGGTGCGTGCGGGCATCATTCGCCCGGT
GCTCGATCCCGCGCGTGATTTCTGGGAATAA
>BDIOMP_04655 Phosphoadenosine 5'-phosphosulfate reductase
ATGTCCCAACTCGATCTCAACGCATTGAATGAATTGCCAAAAGTCGATCGCGTAATGGCTCTGGCGGAAACCAACGCCCAACTGGAAAAACTGAGCGCCGAAGAACGCGTGGCCTGGGCG
CTGGAAAATCTGCCCGGCGAATACGTGCTCTCCTCAAGCTTTGGCATCCAGGCGGCGGTGAGCCTGCACCTGGTGAACCAGATTCGCCCGGATATCCCGGTGATCCTCACCGATACCGGCTA
TTTGTTCCCGGAAACCTACCAGTTTATTGATGAGCTAACGGACAAACTCAAACTCAACCTGAAAGTTTACCGCGCGGGCGAAAGTCCGGCCTGGCAGGAAGCGCGCTACGGCAAGCTGTG
GGAGCAGGGCGTTGAGGGCATTGAGAAATACAATGAGATCAACAAAGTCGAGCCGATGAACCGGGCGCTAAAAGAGCTGAAAGCGCAGACGTGGTTTGCGGGTCTGCGTCGCGAGCAG
TCCGGCAGCCGCGCGCATTTACCCGTTCTCGCTATCCAGCGCGGCGTGTTTAAAGTGCTACCGATTATCGACTGGGATAATCGCACCGTTTACCAGTACCTGCAAAAGCATGGCCTGAAATAC
CATCCGCTGTGGGATCAGGGCTATCTGTCGGTGGGCGATACGCACACCACCCGCAAATGGGAGCCGGGTATGGCGGAAGAAGAGACACGCTTCTTCGGGCTGAAGCGCGAGTGCGGGCT
GCATGAAGGGTAA
>BDIOMP_04660 SPI-1 type III secretion system effector SopD
ATGCCAGTCACTTTAAGCTTCGGTAATCATCAAAATTATACGCTTAATGAAAGTCGGCTTGCTCATCTGTTAAGCGCAGATAAAGAAAAAGCAATCCATATGGGGGGTTGGGATAAAGTCCAG
GATCATTTTAGAGCGGAAAAAAAGGACCATGCGCTGGAAGTGTTACACTCCATCATTCACGGGCAGGGCCGTGGTGAACCTGGAGAGATGGAAGTTAACGTAGAGGATATAAATAAAATTT
ACGCCTTTAAACGGCTACAACATCTGGCCTGCCCGGCTCATCAAGATCTGTTTACTATCAAGATGGACGCTTCTCAGACACAATTTTTACTTATGGTCGGCGACACGGTGATCAGCCAAAGCA
ATATTAAGGACATCCTTAATATATCAGATGATGCAGTGATAGAATCTATGAGTAGAGAGGAGAGACAATTATTTTTACAAATATGTGAGGTGATTGGTTCGAAGATGACCTGGCACCCGGAATT
ACTTCAGGAGTCGATTTCAACTCTACGAAAAGAAGTGACGGGAAATGCACAAATAAAAACGGCGGTTTATGAGATGATGCGTCCCGCAGAGGCTCCAGACCACCCGCTTGTCGAATGGCA
GGACTCACTTACTGCAGATGAAAAATCAATGCTGGCCTGTATTAATGCCGGTAACTTTGAGCCTACGACTCAGTTTTGCAAAATAGGTTATCAGGAGGTACAAGGTGAAGTCGCTTTTAGCAT
GATGCATCCGTGCATCTCCTATTTACTACATAGCTATTCACCGTTTTCAGAGTTTAAACCAACAAATTCTGGTTTTTTAAAAAAACTTAATCAGGATTATAACGACTATCATGCAAAAAAAATGTT
TATTGATGTAATTCTTGAAAAACTATATCTAACCCATGAACGTTCTTTGCACATTGGTAAAGATGGGTGCAGTCGTAATATATTACTGACATAA
>BDIOMP_04665 CRISPR-associated endonuclease/helicase Cas3
ATGACACAGATAGAATATCTGGACTACCTCTGCTATTGGGGGAAAACTGATAAATCAACAAAAAACATAATCTGTAATTATCATTTACTGGCATACCATTGCCTGGACGTTGCTGCATGCGGGA
AATACATAATAAAAAACAATATATTCAATTCATGTAATATATTAAAGGAATGTAATATCTCAGGTACAAATGCAGAAAACTGGATAATCTGGTTTCTTGCCAGCCACGATATCGGAAAATTCGCCA
GAGGATTTCAAAAATACGCCGAATTCCCAAACTCTCCCTTAGTCTCTCCCGTTAGCGGTGTTTCCGCGTTTGAGAGACATGATTCCTTAGGCTTCTATTTATGGGGTAAATTATTTGAAGCGTG
GAGTTCAGGCAGTAATGAAATTATCGCAGGTATTGAACCAGAAAATCGCGCCAGGTTTGAAAGCGCTTTAAATAGCTGGATGCTTATTAGCACCGGGCACCATGGCATCCCGCCGGACACC
ATGAAAAACAGAAGCTCGCTGGCATTCACCGATGAAGACATTGTCGCAGCAACGCATTATCTGGAAGCATTAAGTGAATTATTTCCTTTCACTCTACCTCAGGAGTGGAAAACCAAAGCAGG
GAGAAAATGTTTAAAACAGCACAGTTGGTTTTTCGCTGGGCTTGTCACCCTTGCCGACTGGATGGGCTCGGATGAAAGCCAGTTTCCACTGCTCAGCAGCGCCATGCCATTGAAAGATTAT
TGGCCTCTCGCTTGTGAAAAAGCCCAACAGGCCATCCTCCGAATGCCACCGTTATCTCAGCACAGCCATTATCAAGATCACCGGGCATTATTTCCGTTCATTCAGACACTCACCCCTCTTCAAC
AACGAGCAAGCGAACTGGATATTTCAGCACCCGGTGCACAACTGGTTGTCCTGGAAGACGTTACCGGAGCAGGCAAAACAGAGGCCGCGCTGATTCTGACTCACCGCTTAATGTCAGCCA
ATAAAGGCCACGGTCTCTACGTTGGGTTGCCGACAATGGCCACCGCAAACGCCATGTATCAGCGCCTGGCATCGGCTTATCATGCGCTATTTACCGATGAGTCCAGGCCATCGCTCATCCTGG
CACACGGTGGGCGAGAAATGTCGGACTCTTTCCGACAATCCATCTGGCAGCCAACAGAAAATATAGCTGAAGATTATGCCCGTGATGATGGCAACGCGACTACTGAGTGCCATACCTGGTTT
GCAGATTCACGCAAAAAAGCGCTGCTGGCGGAAGTCGGCGTAGGTACTCTGGATCAACTCTTAATGGCGGTGATGCCTTTTCGCCATCAATCACTCCGATTGCTAGGGATGCGCGACAAAA
TCCTGCTGCTGGATGAAGTGCACGCCTATGATGGTTATATGGTTAAATTGCTGGAAGGTTTATTGCGCTTCCATGCCGCCCAGGGCGGTAGCGCTATTATTCTGAGCGCCACTCTTCCCGCCG
CTTTACGGGAAAAATTACTTAATGCGTTTAGCGATGGCGCGGGATTTATGAGCGCTGGTGGTAGCGATAACGCGGGTTATCCATGGTTAAGCCACCTCACTTCCTCCGGTCTGTTAGAGCAA
CCACTGGCCACACGTCCCGAAGTCCAAAGGACTGTAGCCGTTAATTGGATACAGCAGCGCCAGGAAGCACTGGACATTATTTATCGCGTTGTGGCAACCGGACAGTGCATTTGTTGGATCC
GCAACACGGTTGATGATGCGCTGGATACTTACCAGCAGTTGCTACACGAGGGAATCGTTCCACAGCAGGATCTTTTGCTTTTCCACAGCCGTTTTGCTTTTATCGATCGTATTGCTATTGAAA
ATAAAACGTTAAACTGGTTTGGTAACAATGCCCCCGTTTCTGAACGACGTGGTAAGGTATTAATCGCCACACAAGTCGTTGAACAAAGTCTCGATCTGGACTTTGACTGGATGATCACCGATC
TGGCGCCGATTGATTTATTGATCCAGCGCGCCGGTCGCCTGCAACGCCATATCCGCGATGCCCATGGGCAACGAAAATCTACGCTTCCGGATGAACGCCAGCCACCAATATTGCATATTCTGG
CCCCTCACTGGCAGGAGCAGGCAGAAGAGAGCTGGTTGGGGCAAGAGCTAAAAGGTACCGGCTTTGTTTACACCGATCATGCCTGCCTGTGGCGTACACAGGCGCTGCTGCGTCAACATG
GTGAGATCCGTATGCCGGATAATGCCAGAGCACTCGTTGACGGCGTATACGAGCAGAAAATTGCTGCGCCAGCAGGCTTGCAGACGATCTCTGACGTTGCCTTTGGCAAAGTCCTTAGCCA
ACGTTCAGTGGCTGCGCAAAATTTATTACGTTACGATCTCGGTTACGATCGGGAAGCCAGCGACTTTTTATGGGATAAAGATAGAGAATTTTCTACCCGTCTGGGTGAAGAGAGTGTTGATG
TTTATCTCGCCAGAAAAGACATTGATGGACAGCTACGCCCTTTAGTTGATGAAATAGATTTCTGCTGGGAAAAAAGTCGCTTATCCGTCCGAAAATCATGGTGGCAAAAAAAATTCTGGTACAT

TCCAATGTCCAGATGAGGAAACATTAGCCTGTTTCAGAAAACGCCACCATCGCCCTTCCGGACAGATTGTGCTGGTATCTGATGCCGGAGAAGCTAGCTATTACAGTAAACGTTTTGGATTG
GTTGGATAA
>BDIOMP_04670 Type I-E CRISPR-associated protein Cse1/CasA
ATGGATTTAACCAAAGAGAAATGGCTTCCCGTCATATTCTCAAACGGCGATAAAAAGAAAATATCATTACGCGATCTTCTGGATAACCGCATTCAGGATCTTGCCTATCCTCGGGCTGACTTTC
AGGGGGCGGCATGGCAAATGCTGATTGGTATTTTACAATGTACCGTCGCGCCGGAAGATAAAGAAGAATGGGCAGATATCTGGCATGAAAGTATCGAATTCGAACAATGGGAGAAGGCGT
TAAATACGATTTCTCTGGCTCTACAGTTCGGCGAGCAAAAACCTTCCTTCCTGCAAAGTTTTGATCCTCTCGATAGTGAATATGGTTCTATTGCCGGGCTGCTGGTGGATGCGCCGGGCGGGA
ATGCGCTCAAGCTCAATAAAGATCATTTTGTAAAACGTGGCAACGTAGAACAAATATGTCCTCACTGCGCGGCGATAGCGCTATTTGCGATTCAAACCAATTCACCTGCCGGCGGGGCGGGT
TACCGGGTAGGGATGCGCGGCGGTGGTCCGCTGACTACGCTGGTGGTACCGCAGGAAGAAGATAAATATCCACTATGGAAAAAACTTTGGCTTAACGTTTTGCCGCAGGAAGAGCCGCCG
AATGTTACACAGCATCCACTCATTTTTCCCTGGCTTGCGCCGACGAAAACCAGCGAAAAAGCGGGGAATGTGGTCACACCGGATAATGCGCACCTCTTGCAAGCCTACTGGGGGATGCCGC
GGCGCATAGAACTGGATTTCACCCACACTGTGGCAGGTATCTGCGATTTGTGCGGGGAGCATCACGAATCACTGCTACTGCAAATGCGTAGTAAAAATTATGGCGTTCAGTACGACAGTTGG
CTACATCCCTTCTCCCCATATCGGCAGGCATTGAAAGATCCATCCGCACCCTGGCTGGCGTTTAAAGGGCAGCCGGGCGGGTTAAGTTATAAAGACTGGTTGGGGCTGATGCTCAATCGTGA
GGATAAGTTCAACAAAATGCAGCCTGCAAAGGTCGTTCGTGCCGCTGGTCAGCGGAACAAAATGAGCCTGTGGTGCTTTGCCTGGGATATGGATAAGGCCAAGGTCCGCTGCTGGTATCA
GCACCGAATTCCGCTCATTAGCGTTTCGCACGAAGAGCAATTTCTCGCTGCGCTTAACATTGTGCTGGTGCTGGCTAGTGAGTCGCTGTCGCTGTTACGGAACGCGTTAAAGAGCGCCAAAT
TCGATTGTCCGAAAGAAGCCAAAATGGATTTTAGTATGGTGGATATCGCCTTCTGGCAGGAAACCGAACCCGCTTTTCGGACGTTGCAAGAGGCGCTGGCTGTCGATCCGCTTCGGCAGGA
TACACAGACTCGTCACGCAGTAAGTCAGTGGGAGGCGGAATTAGCACACTATCTATTTCACGTTTTTGACCGTGATGCCCTGACCAACCCCGACTGCCCGGACGATATCCTGCAGCGCCAGC
TGACGGCCCGACAGGAGTTAGCCAGCAGCTATCGTAAACATAAAGCGCGCAAGGATGTGTTGGCGCTGGTCGAATAA
>BDIOMP_04675 Type I-E CRISPR-associated protein Cse2/CasB
ATGCTGAATAGCAATACAGCAGTGTTATGCAGAATTCTCCACCCCGATGCGCAGAAGGCGCTACTTGACTGGTTTGCTACGCTGTCTGAGCGTTATGAGCGCAAGGATGGTAAACGAGTCAA
CGGTCGCGCCTGGCGGGCTGAGTTGAAAAGAATGGCGCCGCCATATGGCGTAATGATATGTGAAGGGCATGACGCTCTCCGCCAGGCTCTGCTTAAACATATGCGACTGCAACCTCTTGAT
GAGATGGCTCTGGCTCTGTTTGTCAGCGTCGCTGTGCATATTAAGAGTCATAAGGCGAATATTAGTTTCGCTGCGCAGTTAGGGGAAAAGCTTAAAGGTTCCACGTCGTGCGTTTCTGGTCT
GCGTTTTGAACGTTTGCAAAAAGCGTCCGACCCGGAAACATTTTGCCAGTTGTTGATTCAGGCAGTGAAGATTCGCGGTACGGAGGGGGTGAATGTTCTTTCACTTGCCGATGGCATTTTC
CTGTGGATGGAAGAGTGGCAACGACGCGAAAACCATCAGCCGGAATTCCGTAACCCCTTTGAACGAAATCGCATTCGTTGGGCGAACGAATATCTTTCCACGTCGCGTGGCAAATAA
>BDIOMP_04680 Type I-E CRISPR-associated protein Cas7/Cse4/CasC
ATGACAACTTTTATTCAACTGCATTTATTGACTGCTTATGCGCCCGCTAACCTGAACCGTGATGAGTCTGGCAGACCGAAGACCGCGTTTATGGGAGGAGTTGAGCGTTTACGCGTATCGTCG
CAAAGTCTGAAGCGTGCCTGGCGGGTGTCGGAAACTTTTGAAGCGGCAATGGATGGCTTTATGGGTAAGCGTACTCGTCGAATTGGCGTGGATTATGTTTATCGTCCGATGAAAGATGCCG
GAATTGAAGAAAAAATTGCTAAGAGTTCGTCTGAGTTAATCGCTAAGCAATTTGGCAAGCTTAAAAGTGATAAAGATGCGAAACCGGAAAAAAATCTCGAAATTGAGCAGATCGTTCACGT
CAGTAACCATGAAATTAGTTTGATTAAGCAACTGGTTGATACGCTAATTGCCGACAAACGCGAACCTAACGATGAAGAAGTGGAGTTGTTGCGCAAAAAGCAGCGTAGCGTAGATATGGCG
CTGTTTGGCCGTATGCTGGCGTCATCGCCTGAGTTCAACGTCGAGGCTGCTTGTCAGGTATCCCATGCGTTGGGCGTAAGTGCCGTGACCGTTGAGTCTGATTTTTTCACCGCCGTTGACGA
TCTGAATAATAAAGAGGAAGACGCAGGTTCCGGGCATATGGGCGAGCAGGGTTTTGCCTCGGCGCTTTTCTATACCTATGTCTGTATCAGCCGCGATCTGCTGGTGGAAAACCTGGGTGGTA
ACGAAGAACTGGCGAAGCGTACTATCGCGGCACTCACTGAAACAGCGTTAACCGTCTCGCCAACCGGTAAACAAAACAGCTTCGCTTCTCGTGCCTATGCCACCTATGCGTTGGCCGAAGT
CGGGCAAAAACAACCGCGTTCTCTGGCGGCGGCATTTTTCCAGCCGGTACGCGATACCGATCAAATTCCTGCGGCAATAACCCGTTTAAAACAGCAGCGCGCCAGCTTCGATAGCGTATAC
GGAAACTGTGCAGACGATTATCGCGAGTTGAACGTTCAGGAAGGTACGGGATCTCTGGCGGAATTACTGGCCTTTGTTAGCCAGTAA
>BDIOMP_04685 CRISPR system Cascade subunit CasD
ATGAAGGAATATCTGGTTTTTCAGCTCTATGCGCCGCTGGCGTCGTGGGGGGAAGAAGCGTCTGGCGAGATTCGCCACTCAGCTACGGTTCCCACGCGCTCTGCGCTATTGGGTCTGCTTG
CCGCAGCGTTAGGAATTCGTCGCGACGAAGAAGCGCGGCTGAATAACTTTAACCAGCACTATCATCTGGCGGTGCACGCGTTGGCTTCACAAGATCGCTGGTTGCGAGATTATCACACCGT
TGGCGCGCCGCGAGAAAACAAAAAGTATCGTTATTACACGCGCCGGGATGAACTGACTCTTGCGCCGGATGAAGTGGGGACGCTTATCTCGCAGCGAGAATATCGCTGCGACGGCTACTG
GCATGTGGCAATAAGCGCCACGCCTGATGCACCACATTCGCTTAGCGAATTGCGAGAGGCGCTGCTGACACCGCATTTTCCGCTCTATCTGGGGAGAAAATCCTGCCCGCTGGCGTTACCGT
TAGCGGCGCGTTTAATGACCGGCACCTTAAAAGAGGTATTCACCCATGCTGTGGAGGAGATTTCCGCAGCGGAATTAAGTGGATTTACCCTGCGTGAGGGCATTTGTTATTGGGATGATCCT
GATGAGGAGAGTCTGGTCTGGCAACAAAAGCAGCATAGCAATAACCAGCCAGTTAGCCGCCAACGCTGGCAGTTTGGCGGCTACACCCGCTTCAATGGGCCGTTGCAGGAGAGAACATA
A
>BDIOMP_04690 CRISPR system Cascade subunit CasE
ATGTATCTTTCTCGTATTCAACTCCGGTTTAATAATTTGCGACCGGAGATGTTGGCAAAGTGGAATTCAGCCAGGCCTTATGCCAGCCACCAGTGGTTATGGCAGCTCTTTCCGGAACAGGAA
TTGCGTCAGTTTCTGTTTCGCGAAGAGGCGCATGGCGGCTTTTTTATGCTCTCGGCAATACCGCCGTTATTGCAGCACTCCCTGTTTCTTATTGAAACCAAACCATTTAATCCGCAACTGACAA
ATGGTCTTGAGCTTGATTTTCAACTACGAGCTAACCCGGTGATAACCCGTAACGGTAAGCGCAGTGACGTGATGATGAACGCGAAACATCAGGCGAAGGCGAATGGTGTGGAGAAAGAGC
GGTGGTGGGAGCTTCAACAGCAGGCCGCGCAGGCGTGGCTGGAACAACAAGGGCAGCAGCATGGTTTCCGGTTGATTGCACCGGAACCTGATGATTTCGCTATGTGGGCTGGTGACGAG
TACAGTGAATTGCAGGCGCATTGTGGATGCGTGCAAGCGTATCAGCAGCATCGTTTTGTCCGTAAAGACCAGCAAAAACCGATCACTTTCAGTAGCGTTGATTTTAACGGTGCGCTCTGTAT
CACCGATGCAGCACTGTTTAAACAGGCTTTATTTTCCGGATTAGGTAAAAGTAAAGCGCTGGGCTGCGGAATGCTGATGGTAAAAAGGAAACGCTGA
>BDIOMP_04695 CRISPR-associated endonuclease Cas1
ATGAGCTGGCTGCCGCTGAATCCCATCCCGTTGAAAGACCGTGTTTCGATGATATTTCTCCAGTACGGACAAATAGATGTGATCGACGGTGCGTTTGTGCTTATCGATAAAACGGGTGTACGT
ACCCATATTCCTGTTGGATCGGTGGCCTGCATCATGCTGGAACCGGGGACGCGGGTTTCCCATGCCGCCGTGCGACTTGCGGCAACGGTGGGTACGTTACTGGTGTGGGTGGGGGAAGCG
GGCGTACGCGTTTACGCTTCGGGGCAGCCTGGTGGTGCCCGTTCCGACAAATTGCTTTATCAGGCGAAACTTGCACTGGATGAAGATTTGCGGCTGAAGGTCGTGCGTAAAATGTTTGAAT
TACGTTTTGGCGAACCCGCGCCGAGCCGTCGTTCTGTAGATCAATTGCGTGGTATTGAGGGGAGCCGTGTGCGGGCAACCTATGCACTACTTGCTAAGCAGTATGGCGTGAAATGGCAGGG
ACGTCGCTACGATCCGAAAGACTGGGAGAAAGGCGATGTCATTAATCAGTGTATCAGCTCGGCAACCTCCTGCCTCTATGGCGTAACGGAGGCGGCAATACTGGCTGCCGGATATGCGCCC
GCGATTGGATTTGTGCACACCGGCAAGCCGCTTTCTTTTGTCTATGATATTGCCGATATCATTAAATTTGAGACCGTTGTACCGAAAGCATTTGAAATTGCGCGACGTAATCCTGCCGAGCCTG
ATCGTGATGTCCGTATTGCCTGCCGGGATATCTTCCGCAGTGGAAAAACATTGGCGAAATTGATTCCTCTTATTGAAGATGTTCTCGCGGCAGGGGAAATTCAACCGCCGTTACCTCCTGAAG
ATTCACAACCCATAGCGATCCCTCTTCCTGTTGCGTTGGGAGATTCCGGTCATCGGAGTACCTAA
>BDIOMP_04700 CRISPR-associated endoribonuclease Cas2
ATGAGTATGCTGGTTGTCGTTACCGAAAATGTTCCTCCTCGTCTGCGGGGGAGGCTGGCCGTCTGGTTACTGGAAATTCGAGCTGGTGTGTATGTTGGTGATGTTTCCACAAAGATCCGCGA
GATGATATGGCAACAGGTTTCCGTTCTGGCAGATGAGGGAAATGTTGTTATGGCGTGGGCGACAAATACAGAATCAAGTTTTGAGTTTCAGACTTTTGGTGTAAATCGACGTATTCCGGTAG
ATCTTGATGGACTGCGATTGGTGTCGTTTCTACCTGTTGAAAATCAATAA

ICE 1 (MOBH, MPF (type G, type I))

>DLDGEG_03780 Tyrosine-protein kinase CpsD
ATGCAAACTGTATCTCGCGTTAAGCGTTCCCCCCAGGTTTCAACCGGGGAGTCTGTAGAAAGAAAACCAGATGACAGCATTCATTTGGCACAGCAGGTTTCACCGCAATCGTTGTTACTCCC
TGATGAGCAACAATTCCCGGTAGTATTGCCAGTAGTATCCACTAAGGGTGGTGAAGGGAAGTCAACCAAAGCAGGCAATATTGCGGGTTACACCGCCGATGCTGGTCTCAAAACGCTGCTA
ATCGATGGTGATTATAATCAGCCAACAGCCAGCAGTATTTTTAAACTCCACTATGAAGCACCCTGCGGACTGTATGAATTACTCATGCAGACTGCTGACCTTAACAACCCTGACAGCATCATTT

CCCGCACGGTTATTCCCAATCTTGATGTCATCATTTCCAACGACCCCGACGATCGTCTTTCCAATGATATGCTGCATGCAGCTGATGGCAGAATGCGTCTGCGTAATGTCCTGCAGCATTCTCTT
TTCAGACAATATGACGTCATAATCGTCGATTCCAAAGGCGCTGGCGGGGTGATGGTGGAGCTCGTGGTGCTCGCTGCGACTCAAAGCGTCATGGGTGTTATCAAACCGATTTTACCCGATGT
ACGTGAGTTCCTGCGCGGCACTGTTCGTCTTTTATCCAAACTTCTGGTTCTGGAACCCTACGGTATCCATATTCCCGATATTCGAATTCTCGCCAACTGTGTTGAACCCACTGTACTGGATCGA
AACACCCTCAACGAACTCAAGGCAATCGTGGATAAAGGTCAGTACCCCCAGTCAGACCGTATTGCCATATCAATGCTGAATACCGAAATAGAGCAACTGGAAGTCTACAAACGTGGGCATGC
GTGCGGGCAGCCAGTTCATCGTCTCGAATATAAAACTGACCGGGTAAGCCTGCCGGCAGCGGAGTCCATGCACCATCTGGTCTGTGAGTTATTTCCTCAATGGAAAGACAAGTTTGATGCG
GTTCTGGTTAACCGGCCTCAGCCCGGATTTGGGCAGGGGGCGGATGTATGA
>DLDGEG_03785 HrcA family transcriptional regulator
ATGAATCACAACTTACTCTGGCGGGCTGTGGCATTTACGGTAAATCACGAAGAAATACATGTCTTCGTTGAAGCTCCGGACAGTCTTACTGCACAAAAACGCTGTTATGAACGCCTTGCTGA
AGAATGGAATGTGGCGAGGGGATTCATCGATATCTACAACCTCTGGAACGAAACCGAACTGCGGGACATGGCTAACGGTTCTCAGGAACCAGCAGGGTTGCCCTTACTGGAATCAGGCGG
AGGAAATGGTGAGGTTTTTTATGACCAAAATCCACTGATTCTTGTTGCATCACCACGCTTACGTGAAGTCCTAGAGAACGCTCTGCAGGAGGTGAAATCATGA
>DLDGEG_03790 Cytoplasmic protein
ATGAACACCAATGAAGATTGGCGGGATGAACACGAGCGTAAATATCAACAGTGGGAGTCCGACAAAGCACTTATCAGTGACAAAAGCCATAAGTTTTACGCTCTTGTCGCGGAAAAATACC
ACGGGGTATATCCCGGCCCCGTTCTTGCACAACAATATTTCCGTATGCTCTGGCTGGGAGAATACCTTCGTCAGAAATACAACTGGCATCACCAGTTTCACGAAATCAGTCCGCAAATGGCGC
TGAGATACGCGCTGATTAAACAGTATGGGGAAAAAATCACAGACATTGATGCGCTTACACAGGAAGAAATGTCTCTGGTCCTGACTGATTACTGGAGCGAATTTATGGCAGATAAGACATGG
AAATCGAAGCGTTATGCTATCGAGAAAGCACTGGACTCTCTCGATTTCTGGACGCCCGGATTTAGCTCCGCAGCCTGA
>DLDGEG_03795 Replicative DNA helicase
ATGAGCCATAAATCTGTTTCTGCAGAATCCATGCTGTTTAATGTCGGGGCTGAAACCGGTGTTCTGGGCGGACTGATGCTCGATAACGATCGCTGGGATGAAATTGCGCCGCTTCTGAATTCC
GGAGATTTTTATTATGGCCAGCATCAGACCATTTTTCGGGAGATAGAACGGTTGGTCAGTGCCGGCCTGCCCATTGACCTGATAACCCTGTCCGAATCCATGGAGCGAAAAGATCTGCTGGA
GCGGTGCGGGGGTTTCGCTTATCTGGCTGAACTGTCAAAAAACACGCCCAGCGCCGCCAATATTGTTGCTTATGCCGAAATTGTGCGCGAGTACAGTCGTAAGCGACGGCTGGTTAAGCTG
GGGCATGAGCTACATCAACTGGCCTCAGTCGGCGATGCGGATATATCCGGTCTGATTGAAAAGGCCGAAAAGCAGTTATTTAGTCTGGCTCAGCAGGCCTGTGACCCCGACATTTGTCTGAG
CGTAACGACTCAGGTCGCTGACACGGTCAGCTGGCTGGAATCGGTCAGTGGGGGAAGCGGTGTGACCGGTACAGCGACAGGTTTTGCTGAACTGGATGAGAAAACCTGTGGCTGGCAG
GACGGTGACCTGATTTTACTAGGTGCCCGGCCATCAATGGGAAAAACGGCAGAAGCGCTGAAACATGCCGTTGCTGCGCTGGAAGGCAGTCCCGGCAAGACCGTTCAGTTCTACAGCATA
GAGATGCCAACCCAGCAGCTGATAATGCGTCTGCTTTCAATGCTGGCCAGAGTGCCCTTCGATAACCTGCGCAAAGGTCTGCTGAGCGAAGGGCAGTGGATTTTACTTGCGGATGCCGTAG
CAAAACTGACCTCCTGGGAAGGACGTTTGCTGATAGACGATACCAGTTATCAGACGCCTTCCTCGTTACGTATCAGCGCGCGGCGTAATGTGCGTAAATACGGTCAGCCCTCACTTATTCTTG
TCGATTATCTACAGTTGATGAGTTGTCCTGGACGCGAGAACCGCACTCAGGAAATTCAGGAGATTTCCCGCTCGCTCAAGTCACTGGCCAAAGAGATAAGGTGCCCTGTGGCAGCTTTATCC
CAGCTCAATCGCAGCGTTGAACAGCGTCAGGATAAACGTCCGATGGTCAGTGACCTTCGGGATTCCGGCTCACTGGAGCAGGATGCGGATGTCATCATGTTCCTGTACCGCGATGAAGTTT
ATAACGAGCACTCCCCGGATAGAGGCATAGCTGAAATCATTCTGGGCAAGCAACGTCAGGGGCCGCTGGGCACTGTCAAAGTTCGCTTTGAGGGGGAGTATATGCGCTTCTCGGAGTATCA
CCTTGGTTATGGGGAGGTGAGCTATGGCTAA
>DLDGEG_03800 Chromosome partitioning protein ParB
ATGGCTAAAGCACCACTGTTAAATCTTAATAATGCGCTGCTACAGGGGGCGAAGGAATCATCTGCAGCTACAACCGCTGCTGTTGCGATTATGCCGACCAGTGAAATGCCGATGGTGCTGAC
GCTCGATGAGGTAGCTCCTAATCCGGACAATCCTCGCACAACACGCAATCCTAAGTACGATGAAATCAAGGAATCCATCAGAGCCCGTGGTCTGGATACGGTCCCGAAAGTGACGAAAAAC
CCGGATATCCCCGGCTCTCCTTATATTTTCAGCGATGGTGGTAACACCCGCTACGCCATTCTGCGAGAGCTGTTTGCTGAAACACAGGATGAGCGCTTCTATCGTTTACCCGTACTTTTCAAAC
CCTGGCCTGGCAGGCTGAGATGTTTGCTGGGACATCTGGCCGAGAACGATGTTCGTGGGGATCTGACGTTTATTGATAGGGCATTAGGAGTTCGTAAAGCCAGATGCATTCATGAGGAAAT
ACAGGGACGCTCGGTAACCCTGAGGGAACTCTCTGAATTGTTAAAGCTGGAGGGGTATCCAATTCACAATTCAATGATTAGCCGTATGGAGCATGCTCTTGAGTTTCTTTTTCCCTATATGCC
TGAATTACTGACATCTGGATTAGGAAAACATCAGATTGAAACCTTATTAAATTTGAGAGCTGACGCTTTAAAAATTTGGCAGCAATATTCGGTGATGACTGAGACTGATAGTGATTTTAATGA
GATTTTCGGTCGTGTCTGTACTCAGTTTGACGATCCGGAGGTGTATTCCTTCGAAATGTTCAGAGATGAGTTTATCGGTATGTTGGTGAATGTTTTACCACACCCTTCTCTTAACTATGATCGGT
GGCTACTTGAGCTGGATCCTAAGGCACGTAACCAGAGAAAGTTATTTGGTGAGCCAGAACCTGTGGCATCTCATCTGGTGGATGCAGATCGCCAGACATGGCAGAGCACTGCATCATTGCC
GGGTACTGCAAACGATGAGAATCAACAAGGCGATTCCTCTTTAAAACCGAAGCTGAATAACGCCCCTGTCTTACCTAAACAACCAGATCCAGACAATGACAATGGGGATAACGATGATGTGA
CCGGCGAATGGTTCGGTTCTTGTGGATCACCTCGTTTACCTAAAGCTGAAGTCCAGAACGATTTCCTCGGGGGGCCCTCAGTTTTAACCGGTGATGTTATTCCTGATGAGTACCACTTTATTC
CCGACGCCGGAAATATCGCTGTTAGTGCATCAGCTTTCGCTGGCGCTCAGGTGGGATTGACTGCGGATACTCCTTCTGACGCCGTTAGTATTGCCAGCGAGATGCCGGGTCTCTCACTTTTAC
CCCCAGAACCAGCACTTTCCTCAGTTGAGTTTGCCAACGTAGGGCTCGAGCCTGTTACTGATATCTGGGCCATTCCGGCTCTGCAGGATGATATCGAACATCTGCAGGACATGGCTTACCGG
CTTGCTTATGAACTTGCAGAGGCGATGGGCTGTGAGGTTCATGTCAGTGAAGATAAAAATGCCAGTGCGGCCGGGTTCTCTGTTTCAGAACACGGCGGAGAATTTGCTCTTTTTCTGGCAG
GGCTTTCCGGTCACGTGCCTAATAAACAATTCAACATGTTTATGTTCTGTCTCAACTTCTTTGGCTCCCAGTCAGCGGGAGATACGCCTGTTTTTGACGATGTGCATGTAGTGAAGAGTCTGC
GCCTTATTCGTGTTATTCGCCGTCTGCGCGAGCTGCAGAGAAATATGGCGGCTGAACAAAATGACGGGAGGAGGCATGAGCATTGA
>DLDGEG_03805 DksA C4-type domain-containing protein
ATGAGCATTGATGTTGATGATGCGATGGCTCAGAACGAATCGCAGGTAAACATTGACAGCAGGTTTGCCGATATCCGGGCTGCTCTTCGGATTCAGGGACATGTATCACGACGACATTGTCG
GGACTGTGGTGATGAAATCCCTGACTCACGCCGTGAGTCCATCCCCGGGGTAAGCCTGTGCATGCACTGTCAGGAATGGCGAGAATCAGGAGACAAAAAACAATGA
>DLDGEG_03810 Transposase
ATGAAGAGTCTGAAAAAGCCCCGCTCGCATTACCAGTGGGTCGGTGCCACGGTTGTTACCACACAGGAGTTGAGCAGTGGACTGGCGGTTATTCCTGTCGGAAGTCGAGGCGTTGTGAAT
GCAGCAAAGCGCGGGCTGTCGGTAATATTTGATGCCTGCCCGTGTTGCGGTGTTCAGCTAAGGCTGACCCGTATTCGTCCTGAGATGCTTGATATTGTGGCATATCCGGAAATTGAGGAGGT
AACTCGTGTCGGCGAATGA
>DLDGEG_03815 DUF2786 domain-containing protein
GTGTCGGCGAATGAAAAAATTCTTTCACGTATCAAGAAGCTAATGGTACTGGCCGAAAAAACAGGTAATCCCTATGAGGCATCAGTGGCTTTACGGCAGGCCCAGGCATTGATGATGAAATA
CGATGTAACCCCGGCGAGCGTGGTACCGGGAGAAATAACAAGTGAGACCTGCAGGAATATCCCCAGTAATGCTCTTTCGGTTCCGGGTTGGCTGAATGCTCTGGTTACAGTCGTTTGCATG
ACCGCGGGCTGTCGCAGCTATTACGGCTGGTATCAGAACAGTGATCATAAGAAGCGTCGCAGCGTAACATTTTACGGGCTTGGTGAACGTCCGGCGGTGGCGGGTTATCTGTTTGGCGTTG
TCTCCCGTCAGTTGAAGCGTGATGCCGAACATTATCTCCATACCGCGTGCGCACATCCGTTACTGAAACTTTCCACTATCCGTCGCCGTATGGATGAATACCGTTTGTACTGGGTGGCCGGAGT
ATGGACGGTTCTGGAGTCATTTGAACCTGAAACATCAGAAAAAGTTCTACTCGACCGCTGGTTAACACAGCAGCAGCGCAATCTGACCCCTGCGAAAGTACGGCAGCCGGAAGGGTGTCG
TAACGCCAAAAAAGTGAGGCAGGCTGCATGGACAGCTGGTAGGCAGGCTGAGATTTATCCGGCTATGGACTATCGCAGTGAGGAGCATTCGCTTCAGGAGGTATCCAATGGTTAA
>DLDGEG_03820 hypothetical protein
ATGGTTAACGTTCTGGCGTTTATTCTCGCTCCAGGGGCCTGTAATCAATTAATTGTCTTGGATAATGAAAGTGTTCACGTTCAGGAGGCGATATGA
>DLDGEG_03825 DUF2857 domain-containing protein
ATGACTTTTTCACTTGCACAGGGTGCCAACAGTCTTTTGTTTAATCTGGTTATGGAACTGAGAGGTGGAAACATTCGGCGATGTGAGGCGCTGGGGTTGAAACCTGAAGAAATGCGTCTCC
TCAGAAGTCTGACGATGGAAGAACTGCACTATCTGTCAGGCAGCCCTGTTTCTGTTTTAAATGTAGCAATACATCATGAGAATCTAAAACGAATGCTGGACCAGGCCAATCGTGAGCAAAAG
CGTAGCGAACGAATTGATCGTGCTATTGCTCTTGGTGCATCAATAGAGATGATGGGGATCTTCTTTGGTCTGAATGCTTCAGATGTCAGTTCTAAACGGCGGCTAGAGGGGATCCAGACACG
TCAGGGACGTTGTCAGTCACCTGATGAGGAGTGTGAAGCGAAGATATGGCGTCTCTGGCATGAGGCCAATATCAGCGATATAGAAAGTCTTAACTCTCTTGAAACTATGATGCTTATTGCGG
AAGAGGCCGATGTCAGTCTCACTGTGATCTGGGGATTAATCAAAAACTGGTGTACGGGGGAGGTAGCCTGA
>DLDGEG_03830 TonB-dependent receptor
ATGGATTCACTGGCACCCTCCTTTCAGTTTTACCGTACTGACCCGAGAAATGTGAGTAAAGCTGTCACGTCAAAGGCGCGTACACTGCTTTCCCTGTACCAGCAGGGAAAGCGAGTCTACAG
CCAGATAGGACAGACAGGATATCTCAAGATTGATCTGGGATTACGCTGGCGTCTGCTGAGTAAGGATGCAGGTAAAAGCTGGTTAATTATGTCTCACCAGACCTATAACCGGGAACTGAAAC
GATGA

>DLDGEG_03835 Helix-turn-helix domain-containing protein
ATGAGAGACGCATCGATAACAACAGAAAGAACGATGCAAACACCGGCTCAGGTGATGGAAGAACCCGGTACATCCGTTCACCGTTTGCCTGCAGATGATATCATTGACTACACCTTGTCGA
AGATGCAGGCACGGCTTGACGGTACGCCGGGTTCGTCGGGGCAGGAGCGTAACGGACTGCTGTTTACAGGTAATATTCACGACGCCTACCCGCGGGCGCTAATCCTGGATATGCGCCTTTC
TCCTCTGGATAAAATGTGCTGGATAATGATACGGCAGTATGCCCTGCAGAACGACGGTGCCATCTTCCCCTCTTATGATGAACTTCAGAAACTGCTTTCTTCCCCCGGTTCCGGACAGGCGTC
ACGCGATACGGTGAGCCGTGCGCTGACCATGCTTCGACTAACGGGCTGGTTGAGTTTGTGCAAGCGGGTGCGTGATAACGGGGGGCGGGTACGGGGAAATATTTACGCCCAGCATGATGA
GCCAGTCAGTGCGAAGGATGCAGAGATGTTTGACCCGGGATGGCTGGATATGGTGGGTAAAGCCTGCCAGCACAAATACAGAGAGGTCAGCGACACGGCCTTTCATGTACTGAACGGGAT
ACTTGATGATCCGATGATGCGTCACAGGCATTCGCGGATGACGGAAATTGCGGAACGTCTGACCCGCCCGTCAAAGGTGGGTGATGTTGCCCGCACTCATCAAAATCCGGGTACCGGACTC
AGTCTGAATAGCATAAAAAATGAGCATAAATCACTTAGTCCGGTAAACAGACCCAGTAGTGAGAGGGGGGAGAAATCACCGAGTCCGGATAGCAGACTCAGCCTGAAATCAGACAGTTAC
GACGGAGTCCGGCAACCGGACTGCAATAACGTACGTTCTTTTACACAAAGTGTGATTAAAAAAACGTACGTATCTCCCCAGAGCGGCAAAAATGCGGTTGCCGGAATACCCGAGGGGTTCG
TGTGGCCTGATGGTCTGCAGTCCATCCTGCCGGAAAGTGAACAACCCATGCTGGCTCAACAGTTGAATCAGCTTGCACAGAAATCACCAGTTCAGGCTGAACAGATCGCCCTCAGCGTGGT
GAATGGCTGGCACCAGAAACGTATCAGCAATCCGGTGGGCTACTTGCTGACTACACTCAGGCAAGCGAGGGCAGGACTGTATCGTCTGGAACCTGCTGTCACTCAGCCTGTTAAGAGCAG
ATCTGTCGTGCCAGCTGAGCCATCCGCTGCCGGGAAAATCCCGACATGCTCAGCTGAAGCTGATGTCCCTGCAGGTCAGGAAGTCGTGAAGGCGATGGTGGAGCAAATTCGTCAGAGAAT
GAATTCCTCTCAATGA
>DLDGEG_03840 hypothetical protein
ATGAGTGAGAACGAACAGGTATCAGTGTCCAGAAAAGCGGGTGCTTTACGTTCGGCGCTGAACATAGCGTTGAATACTGATTATGCAATAAATTTGTGGCAGGGGCGTCCTCCGGAAGAAG
AGGGGGAAAAGGAGGGAAAGAAAAAAGAGCGTCGTCAGAGTCTTATTTTCAGTATGCCAGCCTTCATTCAAAAAGCAGGTCGTATTAATGCCGATTCATTTAATGACAATCCGTATGCTGAT
CAGAAGATGCTTGAGCTTGAGACACTTTTGCAGTCCGCTTCGGTCAAGATGAATGAAGAGCTTGTTGCACTCAAGAAGTCAATGTCAATGCTACCCCCTCAGGCGACTATTTCTGAAGTCAA
CTGTGCTTCTCCTTTAAATATTGGAGTTTTCAGTCGTACACCTCTGGGTTATCGATGTGTATGGCTTCTGGTTGGCTTTGACCAACTGGCAATGCAGGCTTTTCAGGCTGCGCATTATGGTTTT
ATCTCCCGGCAAGAGCTACACCGTAGCCTTCGACGGGGAGGGCATCTAATCCGTCAGATCTATGGTGCCGCTCAGAAATATCGATTTTTTCAGGTGAACCGCCGCGATTTTGCATTACAGAAT
GCTCAGTACCATGAGGCAATCAGGAACGCTGGCGAAATTGACGAAGCTATTTTGTTGGGGCAAAAACGTTCCTCTTTTTCACCCCCAGTCAGTAAGGAGAGTATTGAACTTCTACTGGCGG
CAAATGCAAAAGCAGACAAAGCAGATATTGTGCAATTACTGTAA
>DLDGEG_03845 DNA topoisomerase
ATGCGTCTGGTGTTGTGTGAAAAACTTTCTCAGGGCAGAGATATTGCGAAGTTTCTTGGCGCCACTCAAAGAGGCGAAGGATTCCTGAGTGGTCCGGGAGTTACAGTCACCTGGGCTCGC
GGCCATCTGCTGGAGACAGCTGAACCGGAAGTATATGGCGAACAGTATGGTAAGCCATGGCGCACCGATGTGCTTCCTTTATTACCACAGCAGTGGAAACTGGTAGTCAAAGCGGATGCAA
AAGCCCAATTCGCGGTAATTAACCGTCTGCTTAAACAGGTAGATGAGGTTGTAATTGCCACGGATGCTGACCGGGAAGGTGAAGTAATCGCCCGTGAGTTGCTGGATTACTGCAAGTTTCA
GGGGCGTGTATTCCGGTTGTGGATGTCAGCTCTGGATGATGCCAGTATCCGGTCAGCGTTAGCTGATTTGTGGCCGTCTTCCCGAACTGAATCGCTTTATTATGCTGGCGTGGGCAGGAGCA
GGGCTGACTGGCTGATCGGTATGAATCTGACTCGCCTGTTTACACTTCTGGGGCGTGAGGCGGGTATCGGTAACGTTCTTTCAGTTGGCCGGATACAAACGCCAACGCTGGCGATCGTGGT
AAATCGTGATCGTGAAATTGATAACTTCGTGTCGAAACCGTACTTCGAAGTTATTGCCATGCTGGCCGTGAACGGCGTGTTGTTTCCATCCAAATGGGTACCGGCTGCGCAGTATTGCGATGA
AGAGAAGCGTTGTATCCAGTCGGGTGTAGCGGCACAGGTGGTTCAGTTGTGTCGGCAAACGCCAACGGCTGCGATAATCAATTGCCAGACAGAGAGGAAAAAACAATCAGCGCCGCTGG
CATTCAGCCTGAGCACACTACAACAGGCCTGCTCCCGACACTGGGGGATGCCTGCCCAGACGGTACTTGATATCGCCCAGAAGTTATATGAAACCCACAAACTGACGACCTATCCCAGAACT
GACTGTGGTTATCTGCCGTTATCGATGCGAGAAGAAATCCCACAGGTACTGTCTGCTGTGGTCGGAACTGATCCGGCGCTACAGCCGGTGGTCGCGAGTCTGGACACTCAGTTTGTGTCAC
GTATCTGGAATGATAAAAAAATTACCGCTCATCACGGTATCATTCCGACAAAACATACTGGTGATCTGAGCAAACTCAGTGATGCTGAGCGCAATGTCTATCAGCTGGTCAGGTTGCACTACCT
GGCCCAGTTCCTGCCCCAGATGGAAATTGACGCGACTGAGGCCACATTTAATATTGGCGGTCAGCTTTTCCGTACCCGTGGTAACGTTTTGGTGAAACCCGGCTGGAAATCTCTGTTTGGCA
AAATGTCCGAAGATGATGAGGACAATACTGGTGAAACCACACTGCCCGCCATGCAGGCGGGCCAGGTCTGCCAGGTGCAGGGCGCTGAGCAGAAGGTACTGCAGACAAAACCGCCTGCT
CCGTACAACGATGGAACTCTTATTGCCGCGATGACCAATGCGGCGGCGTTCGTTACTGACGCCGCGCTGAAAAAGGTGCTCAAAGAGAATGCGGGGATAGGAACTGAAGCAACACGGGC
CGGGATCATCGACACGCTGTTGAAGCGTGGGTTTCTGGTGCGTGAGAAAAAGACGCTTAAGTCAACACCTCTTGGACGTAACCTGGTGGATGTGTTGCCAGGAACGTTAACGAATCCCGG
ACTTACGGCACTGTGGGAACAGATGCTGGATGAGGTAGCTGCAGGCAGGGTGAGCCTGGATGACTTTATGGCAAAGCAAAGTCAGTGGGTCAGTCAGCTGGTCGCTCAGGGGAGATCAC
AGCCATTGACTATGCAGGCGCCACCTTCACCCCCCTGTCCTGTATGTGGCGGCAAGACGTTGCAGCGTAATGGAAAGAGTGGCTTGTTCTGGGGTTGCCTGAAATATCCGGAGTGCAAAGG
TATCGTGAACAATGGTAACAAGGGCCCCAGAGGACGCAGACGAAAACCTTCGCCAGCCTAG
>DLDGEG_03850 hypothetical protein
ATGGCTGTGGAACGGTTCGAAAGCTTTGCATGCCTTTCGCAACTCCAGTCAGGAGAACGATCTTTACTCGAAGGATCCGTCCTCAAAGCGCAATCACTCCGTGAAAACCGCGCTGAGCCAT
TGACCGGCTTCGGAGATTTGATTTTACCTCATGTTCTGGTGCAACAGTTTGGTTGA
>DLDGEG_03855 STAXI RNA
TGTCGCTTAGCGACCACCAATGTTCCAGTGATCTGAGAGGAGCGCCTTCGGGTGATCACAACACCAATGTTCCGGCAATCTGAGAGGGACGCCTTCGGGTGATTGCACACCAATGTCACCG
TAGCCTGAGAGGTGACGCCTTCGGGTGGTACAGC
>DLDGEG_03860 DUF3577 domain-containing protein
ATGTCTAAAGAAACTAACTACTTTAATCTGAATATCAACGGTCTGGGCTATATCACGAATGTACGTCAGGTTGTCAACGGTAACAGTAAGTTTACCTGTTGCACGCTGAATGCCCTTAGTGGTC
CGACAGATAATGCAGACTACACCCGCTTTGATGTCACAGTCGCAGGCAAAGACGCAACGAGTTTAATTAACCGCTGCCAGAAGTCCTGTGATGAGGACAAAAAAGTAATGATCGGTTTTGT
TCTCAGTGGGATCAAATCCGATATTTTTACCCTTGCAAAAGGTGATCATGCCGGTGAAAACCGTGTCAGCCTGAAGACTCGCCTCATCCGGGTTGACTGGATAAAAATTGACGGTGCAATTG
CCTATAAGGCAGAAAAACCGGATTCCACTCCTCCGGCCCAGAGCCAGCCCGCGCAGAAGCAGTACGCGGAAGATTCGTTCTGA
>DLDGEG_03865 Transketolase
ATGTTAACTTCACCATCATCTTCTACTGGTTTACTGGCAGCAAAATTTGCCGGTTTATCCTTACCTTTACAGGTCCTTCGCAGTGGTGCCGGATATTACATCGGCACGCAAAATGAAGAAGGGC
CGGTATCCCGTGAATCTGTTGAGTACTTTTCCACTCAGTCTCAGGCAGAACATGCGCTCAAACAGGGCACATGGTCACAGCGTGAACAGGCTTAA
>DLDGEG_03870 Single-stranded DNA-binding protein
ATGGCTTCTAAAGGTGTAAACAAAGTTATTCTGGTGGGAAATCTGGGACAGGATCCTGAGGTGCGTTATATGCCAAATGGCGGTGCCGTGGTAAACCTCAGTCTGGCAACCTCAGATACCTG
GACAGATAAACAGACCGGTGACAAAAAAGAGCGCACTGAATGGCACAGGGTGGTTCTTTACGGCAAGCTGGCCGAAATTGCCAGTGAGTATCTGCGTAAGGGCTCCCAGGTATACATCGA
AGGTGCATTACGCACCCGCAAATGGACGGATCAGTCTGGCGTTGAAAAGTACACCACAGAAGTGGTCGTCAGCCAGTCAGGTACGATGCATATGTTGGGCGGACGAAATAGTGCGGGAAG
TGGGCAGCAACAGGGCGGCTGGGGTCAGCCTCAGCAACCAGCAGCGCCTTCTCACAGCGGGACGCCACCTCAACAGCATCCTGCAAATGAGCCGCCAATGGACTTCGACGATGATATCCC
GTTCGCGCCGTTCGGGCACAGTGTGGCCAGACATGCGCTTTATGTGCTGTCCTGA
>DLDGEG_03875 Rpn family recombination-promoting nuclease/putative transposase
ATGGATAACGAAAAGGGCCATAACCGGCCCGGGCACGATGGCCTGTTTAAGCTTTTTCTGCGTGAGCCGGACACTGCGAGGGATTTCCTGGCAGTTCATCTTCCCGCAGATATCCGCGCGC
AGGTTCGTCTCGATACGCTGAAGCTTGAACCGGGTAGTTTTGTGGATCAGAAGCTTCGCGAACTGCATTCGGATGTACTGTATTCGGTGGAAACCGCAGAAGGTCACGCCGGGTACATATAC
TGTCTTGTGGAACACCAGTCGACCGCAGACCGGATGATGGCCTGGCGGATGATGCGTTACTCGATGGCAGTGATGGATGCCCACCTGAAAAAGGGTAACGGGACACTGCCGGTGGTGGTG
CCGTTGCTGTTTTATCAGGGAACGGTGCGGCCTTACCCATACAGTACCGACTGGATGGACTGTTTCGATGTACCGGCGCTGGCGCGGGAGGTTTACTCCCGACCGTGGCCCCTGGTGGATG
TCAGCGTCATGGAGGACTGCGACCTGCAGTCTCACCGTCGCATGGCATTACTGGAGCTGGTTCAGCGGGATATCTGGCATCGCGACGCTGCATCGCTGCTGCGTGATGTTGTACAGCTGATT
CGACTAGCCGGAAATACCCGGGAGCAGGTGGAAGCCGTCCTGTGTTATATTATCTACAATGGCATGACAAGCGAGAGTATCACGCCGTTTTTATACGAACTGGCCGGTGAGATCCCGGAGTA
TAAGGAGTTGATCATGGGCACAATTGCACAGCAACTGAAAGAAGAAGGTATCCAGCAGGGTATTCAGCAGGAGCGTCAGGCCAGCCTTGAGCGTGAGCAGAAGACGTTACTGGAAACTG
CGTATGCCTTGCTTGACAATGGTGTCAGCCTGGAAGTGGTGATTAAATCCACAGGTCTGAGCCGCGAGACGCTGGAACAACCGCGTCATTAA
>DLDGEG_03880 TcpQ domain-containing protein

ATGAACACACAAGCGCTTTTCTGCCTGAGCCTGCCGCTGGTGATTGCCGGGTGTGCACAGCAGACCTCCACCCAAACCGCTCGCGACAGCGCATTTGCACAGAGTCAGCAGCCTTCCGTCT
TCTCTGATATTTACCAGCGATCACCGGAGGTGACTCGCAGCGGGCGTTACACGCTGGTCAGTATTAAAAGTGAGGATGCGCAGCGGGAACCGCTCAACCAGCTCATCGATATTACTATGCCA
GGGCAACTGGTTAACTCAGTCGGTGACGGATTTCGCTACCTGCTGTTTCAGTCTGGTTATTCCCTGTGCGGACATTATGGCGCTGACTTTTCTGAGCTGCTGAACCGGCCGTTGCCGGCCGT
TCAGCGCAAAATTGGGCCGATGCGACTCAGTGAGGCACTGCAGGTGGTTGCCGGTCCTGCCTGGCGAATGTCCGTGGATGAAGTCAACCGGGAAGTCTGTTTCGTATTGCGTGATGCATAT
CGGGTACAGGCTAAAACTAAAGCGCTGACATCTGTCACACCTGCCGTGGCCGGCGCCGCAAGAACCTCCTACCCGGAAACCACCCGCAATCCTTATACCGGAGTTCTGCCGGGGAATAAAG
CTGAACCCGTGATCGCTGGCCAGCATCTGGCGGCGAAGAAAGGTACTGAACTGAAACCGGTCAGTCAGTCACTTCCTGTCCCTGCCCTGAGATCTGGTGGGACACCGGTAACGTCAGGAA
TGTCCTCTGTCCCCCCCGTGAAACCAGTCTCTTCTCAGGCTCCGCGCAATCCGTTCACAGGAAAAAACCTGACCGGGACCACCCTGGCGGCGTCTCATTCACCGGCGCCTGTGGGAGAGAA
GCCTCAGGTCAAAACACCGTCTCTTTCAACCGTTAAGGCTGTAAATCCGGCGACGGCTACGGTACTGGCCACGGCAACAAAACCGTTAACCGGCACGCCTGTAGCTGCAGTGGCCAGTGG
CCCTGAGTGGAAGGCGGTTGTCGGCAGCACGCTCAAAGAGTCCCTGACTGACTGGGCCGGAAGAGCTGACTGTCCGGGGGGAGGACACTGGGTGGTTATCTGGCAGACCCCTACGGAT
TACCGCATTGATGCACCGCTGGTATTTAAAGGCAACTTTGAGACGGCGCTGGTTCAGGTGTTTGACCTTTATAAGAAAGCTGACAAGCCCTTATTTGCTGAAGCTTCCCGCCTGCAGTGCCT
GGTTTCAGTTGCGGATAAACCGGCTGACCGGTCGTAG
>DLDGEG_03885 Pilus assembly protein PilP
ATGGGCTGGTTCATTGTCGCTCTGGGCACAGTATTTCTGCTTGTCGTCGGACATGTCCAGACCACTCAGCGAAGCGAGGTTGTCAAAGCGCAACAGTCGGGGAGTTCGTATCTGTTGGCCC
GGCAGATGTTATTGCTGGCCGCGGGCATTAATGACTGGCGCTACCGACATACCCTGACGGACGGAACGGTCGCCTTGTCAGAACTGGCCCTGCCGGTGGCTCCTGACAGCCGTATCCGACA
TGTCATTGTGGCTAACCGGCTGTGGGTCTGGATGCCTGAAGTCCCGGGTCTGGTGGATGCGCTGCGTGAACAATCAGGTGGTTCGGCATTAACTGGCACGGTGACGCAGGGGCAACTGGT
CTGGTTATCCGGTGTGGCTTCGGGGTTGCCTTTACCAGCAGGTATTCAGAACGGGGATGTGGTCTACCTCAATTAG
>DLDGEG_03890 Secretin-N-2 domain-containing protein
ATGATAAGAAAATATTACAAAGTCAGGCTCCGTGTTGCAGCTACAGCCATCGCCGCCGCACTTTCGCTGTCGGCCTGTACTTTTCAGGAAATGGACAGGATGTCGCGCAGTGCGCAAGCCA
GCGGTGATATTGCCCGCGGTCATGTCAGTGAGCAGATGCGATCCTCACAGGGAGCGCTGGTCTGGACGGATAAACCCTGGGTCAATCTCAAACCCGTCCCGACAGTGTCACGGGGTATCCC
TCAGGAAAACTTCCCTGACTGTCAGATAACGCTGGCACGGGACGGACTGACTCTGCCGGAAGTTGGAGAGCGAATCACTGCGCTTTGCGGCGTCAGGGTAATTTTTTCACCCGATGCACT
GGCAGCAGGTCAGTCGTCCGGTACCACGCGGGCACTGAATGGTCCATTACCGGTACCGGATAACAATGGAAGAATTCCGTTGGATCAACTGGGCGGCGCTGTTGACTCGGCACGCGTGAC
CGCCACACCAGTGGTACTGAGTAGCCTGCGCTGGCAGGGCAAACTGTCCGGTCTGCTGGACATGATTTCGGCTAGGACAGGCCTCACCCCGCGTATGGATAACGGGGCAATTCTGTTTTCA
CTCCTGGAGACGCGTACTTTCCAGTTCACATTTCTGAATACCAATATTACCAGTAATGCCAGTGTTACCTCGGGTTCAACAAGCAGTATGGGAACCAGTGGTGGCAGTACAAACAGTTCGGT
GTCAGGGGATTCAAGCTCTTCGCAGCAGACGACGGTCGACCAGAGCCGTAATGTCTATGACGATATGAAAAAGACGCTGGAAACCATGGTGACTCCGCAAAAGGGACGTTTCTGGCTGTC
GGCCAGTACCGGCACTCTGACGGTAACGGATACCCCTTCTGTACTCAACAGTATCGCGGCGTATGTTGAGCAGCAGAACCGTCAGATGAACCGCCAGGTCAGGCTCAACGTTCAGGTACTG
AGTGTCAGTACTTCACGTAAGGAGCAACTGGGACTGGACTGGAATATCGTTTACAAGTCCCTGCGCTCAGCCGGTGCTTCACTCAATAATGTAAGCGGTGATATCACCAACGCCACGTCGGC
GTCGGTATCTATTCTGGATTCAGCAACCGGAAGTGCGGCTAAATTTGCCGGCTCCAGTCTGCTGATTAAAGCTCTGTCAGAGCAGGGCGATGTCAGTGTGGTCACCTCGCAGGGTAGCGTG
ACCACGAACCTTACACCTGTACCTATTCAGATGGCCGACCAGACGGTCTACGTGGCACAGAGTTCAACTACTACCACAACCGATGTGGGTGCAACCACATCCCTGACGCCGGGTATGATGAC
TACCGGCTTCAACATGACGTTACTGCCACTGATTCAGGATGGTGGTGACGTGCAACTGCAGGTGGCATTCAACCTGTCTGATCCACCGACTATTCGTAACTTTACGTCCAAAGACGGCAACTC
GTACATCGAGATGCCGTACACGAAACTCCGCTCGCTCAGCCAGAAAACTAACCTTCGCGCAGGGCAGGCACTGGTGCTGACAGGTTTTGATCAGAGTAATACCCGGATGACCAAAAACGG
CACCTTTACCCCGGGTAATTTCCTGCTGGGTGGAGGTCGGGACGGCGATGATTCCCGCAGTACGCTGGTCATTATTATCACCCCGGAGCTGATGGGGAACGGTGGCTGA
>DLDGEG_03895 Pilus assembly protein
ATGGCAGATATTCCGTTATCCGGCAAACCCCGTACGCAGGTTTTGCGCTGGCAGCGCCAGAACTGGGTAGTGGGGCTTCGCTGGGAAGAAAAGCAAAAGTCGGTACGTCGAGCCCCTGTA
CAACGTCGGGGTGTTCTGAACGTTGTGTTGACGGTGAAGCAGGGCATGAGCCGGTTGACCGCGTTGGGGCAAATTGAGGCCGGCTGGCGTCACCAGAAGCTGTATTCCCTGGCGGCGCT
GTTCAGCCAGCAGTGTACGCCCAATGGCTATGGTGTGTACCAGCTTGATGATGGCAGGCAGGTCTTTCTGGCCACGGTGAAGGGGATGCCATCTCCGATGGCAGATGTGATCGGAACGCCA
GATCAAATTATCCGGGCGAGAGAGCTCTTTCTGTCCTTCAACCCTGCTCCTGAGGACGGATGGACGATACCTTCATCACCGGAATTCCCCCAGTCCTGGGACACTCTGGTCGGTCAGGTATCT
TCGGCACAGCTCCGGTTTGCCAGAGTGGCGGGCGTGTCTAATCCTTTGCGGGCATACGGTGCGGGAGTGCTGTCAGTGACTGCCCTGCTTGGTGGTGCAGTCTGGTGGAGCAGGTTACCC
GAACCTCCGGTCGTGCCTGTCGTGCAGGAGTTACCGCCACCGGTGCAGGATGTGTTTAAAAAAGCACCTGAACCTGTTTACCTTCCACATCCCTGGGCAGATATGCCTGTGGCCAGACACTT
TTTGAGCCGCTGTCAGCGCTGGCGTGCCGCAGTTCCTGTGAGTCTTGACCGTTGGCGTCTCGAACGGATTCGTTGCAGTGCTGAAGGGCTGGAGACGGTGTATCAGCGGCAACCCGGTGG
GACGGCGGCACGCTTTGCTGAGCGGGTGAAGCAGGTCTTTAAGCGTACACCAGTATTCAACCTTGTTTCAGGTGGCAACGAAGGTGTGGTGTTTATTCCATGGGCGAAATTCACATTACAG
GATGAGACTGCACCGGATGCGGGGACCCAGTTGATGCAGGCGGTGTCGTGGTTTCAGTCACGCCAGGTCAGTTTTTCTCTGTCCGAAGTGAAAACTCCTCCCGCGATGCCCGGTGATGGC
ATCGGCAACGATGCGCCCCAGCCTATACAGGACTGGCATGAATACACGTTCAGTATCACTGATAAACACATGCCTGAATGGATTTTACAGGGACTCGATATGCAGGGGGTACGGCTGAGTTC
TGTGGGGTACACCCTGAGTCCTCAGGGACAGTTTACTTATCAGATTGAGGGGCATCTTTATGCTTATGCCAAAAAATAG
>DLDGEG_03900 Type IV pilus biogenesis protein PilP
ATGCTTATGCCAAAAAATAGTGTTTTCATCAGTGGTGTTCTCCTGATGGCGTTCAGCGCAGGGGCATTTTCGCAGCCCACACCCGCAGTTCCTCCTTCCGCAGAGGTGATTTCTGCAATGTCT
GCCAGTGAACCCGCTACGGAAGCAGAGGCATCGATGTCCTCTGCAGACTTATCAGAGACAGTCCCCATTTTCCCCGGTAACAGCGTGACGGCCGGACAGCTGGAGGCGCTGCAGGGGAA
AAACCTGCTCCTGGAGGCAAAGGTTCAGGCAGCCCGGCTGCTAAAGGAGCTGACTTCGGCACAAACTCCGGGGGATGTTGGCAATGTGCCCTCGACCAGTCCGTTCGTTATGGGAATGCC
GGCAGACATGAATGCGGCTCCTGTTCACACCCCCCCTGCAACTGGCCGAATTACGGTTCTGGAAGTGAGTGGTCGTGGTAACGCCCTGCAGGCCACGCTGTCATTTCCGGATGGCCGACAA
AGTCAGGTACACACAGGCAGCCTGGTGCCAGGAACATCGCTGAAGGTTAAGGCAATAACACTGTCATCCGTCACGCTCAGCGACGGTCAGCAACTGACGTTTTAG
>DLDGEG_03905 ATP-binding protein
ATGTCTGAACACAACGATGCGGTTCTGCTTTACCACAACCCGGACACCACGATCCTCGAGCTGCTGGTTGACAGCCAGCGGCGTACCGACGTGGCGGTGCAGCAGAAGGTGATGTCTCTG
ATGGAGGAAAACCCTGGCGTCACAATGAGGCTGGTAACCCGTTCAGAGTTGCTCAGGTACAGTGAAATGGCTCGCCAGCAGTCAGAGAAGGGAGAAAAAGGACTGCGCCTTGAGGACA
TCAAAAAAGAGGTCAGCAGCCGTCAGGGCGATGTTCTCAACTATTTTATGGTCGCGAAAGATATCGGCGCGTCTGATATTCACGGTACCACCCGGCCAGGACTGACGCGCATCGAGATGCG
TGTGCACGGTGAACTGGAGACCGTTTCGGAACTGGCTGAAGAAGACGGAAAAGCACAGGCGGCCACCATCATCCTTTCCATGTGTGATGTGACCGAACCCCAGTTCTACGCAGGCCGAAA
ACAGGATGGTCGTGTGGCTGCAAAGTTTCTGCGAAAGGTGGGGTTATTTGGCGCCCGTTACAGCCACACCCCGACAGCAGATGGCCTTCACTTTGTGATGCGCGTGATCCCGGATGATGGT
GATGTGGTTCCCACGCTCGAAAAACTCGGTTTTCTGCCGGAACAAATCCGCCTGATAAAGCGCACTTTGCGCCTGCCGGAAGGGATGATTATTTTGTCCGGCCCGACCGGAGCCGGGAAAT
CCACCACCCTGCGTGCCTGCAGCGACCTCTATCTGAAGCGTACCCGGTATAAAAAGCGCCTGCTGACGGTTGAAGATCCGCCGGAGGGACGCATCGTTGGGGCAATACAGACTCCTATTATC
TGTGACAAAGCCGATGAGGCGGAAGTCAGGCTGGCCTGGCAGCGGTCGCTGACCTCAGCGTTACGTCTCGATCCGGATGCCATTTTACCTGGTGAACTTCGTGACCTTATCTCCATTCTGGC
CGGGATTTTTGCCGCCCAGACCGGCCATCTGGTGCTGAGTACCCTGCATTCAAACTCGGCCTTTTCCATCCCTGAGAGAATGATAACGATGGGTGCAAATGCCGGACTGGTGGCAGATGCC
CAGTTGTTGGTCGGGCTTATCAGCCAGCGTCTGGTTCAGGTGCTGTGTCCCGAATGCCGTATTCCCTGGTCGGTTAAAAAAGAACATCTCACCACCGAAGAGCGTGAATATCTGGAAAAGTA
CTGTTCAGTGGACGGGGTATGCCAGCCCGAAAATCTTCACTTCAGAAATGAGGAGGGGTGCCCGACCTGCCGGAAGGCGGTCATTATCGGTGGCAAACAGGTGGCAACAGTCGGTAAGG
GTGTTGCCGGACGCAGTGTGGTGGCTGAGGTGGTCCGCACGGATTCCACGCTTTTCAGGCTGTTATTACAGCAGGGTAAGGAAGCTGCAAAAGCTTACTGGATCCGTGAAATGGGGGGC
ATTACCCGGCGCATTCATCTGCTTCACAAACTGAACGAGGGGATGGTTGACCCACTGGATGCCGATTTGATTATCCCTCTTGATGAGGATGAGGTGATGCAGGTGGTAAGTGATCAGGCTGC
ATCGTCGACTGACATTGATACGTCGCTGATTGAACGCCTTGCCATGGTAATTGCAGCCAGCGTCAGAGAGGGGCTGAAGCAGGGCGTTGAGGAAGCTATTAAAGAAGGCGTTGCTGCCGG
GATGGCTGCGCTGAAGGAGGTTGCATGA
>DLDGEG_03910 Pilus assembly protein PilR
ATGAGTCCTTTATACCGTCTTCGCCGGGCGCTGGCGCGACGTTTTTTTACGTCCGGATACCGTCTGCGTTTTTACCGCATGCTGTCGTTGCAGATGAGAAATGGCGTGAAGTTGTTAGAGGC
GCTGGAGCAAATCAGCAATATGTATACCGATTTTGGGCAACGGACCCATGGATTCGGTCTTCTTGTAGACGACTGCCGGGCGGCACTCACTGACAACAGTGGAGATAACTCCCTTGAGCATG
CCCTGGCGAACTGGGTTCCGGCGGAGGAGGCGGCGCTCATCAGCGCGGGAATGTTCAGTGGCCGCTTACCAGAGGCGCTTGCTGAGGCGGAAATCCTGATTGACTGCCGGCGGCGTATC
CGACAGGCGGTCCTGAGGATGGCTATTTATCCCCTGGGGTGCATTGCGATGCTTGGTGGGACACTAGGTGTTATTCAGATGGAGCTTATTCCAACGCTGGCCGGGATGTCAGATCCGCAGA

CATGGACCGGAGTGCTGGGAAGCCTTCATGGCCTGCTGATTTTTTTTGCTGAGCATGGCGTGGTGATGGGGGCCGGGCTGGCACTGTTGGTCGCATGGACGAAGTGGTCCCTGCCGAACT
GGATACGTCCGGACCGTCTGCGGCGTCTTGCGGACAGACATGTACCCTGGTCGGTTTACAGTGATATTCAGGGCGCGATTTTTCTCATCAATATGGGGGCTCTGCTGTGCGCCGGGGTCAGA
ACCCTGACTGCGCTGCAGGTTATCCACCGCTTTGCTTCTCCCTGGCTCGCTGTCCGTCTGAACGCGGTGATGGAGGAAGTGGAGGAGGGGGCCTCTTTTGGTATGGCGCTGCGTGAATGTG
GTTATGCCTTCCCCTCAAAAGAAGCCGTGAACTATCTCTCTATGGTCAGTGGGGACGGGGCCGCTCAGATGATTGCGCGCTTTGGGCAGGAATGGCTCGAGGAAACTGTGAAACGAGTTA
ACCGACGGGGAATAGCCGTGATGCTGTTCTCAATGGTGATGTTATTTGCGCTTATTGGCGTTGTGGTCGTGGCGGTGATTGAGGTGACTTCGATGACCGAGAGTATGGGGCAGTACTGA
>DLDGEG_03915 Prepilin
ATGAATGAGGTATCAACATTAAATCCATGCAACAGGCCTGACCGGGGAATGTCCGCAGATGCCGGTGCCACTGCACTGTTTATTCTGGTGATCATTGGGGTGATAGCCGCGGCGGTCTGGTC
GATGTGGGGTAAAAAGGATGCGGGTACGGAGCTGACAAACTATCAGACCCTGGCCACGAACACCATTGGCATGATGAAAGGCGTTGACGGTTATGCTTTTACCAGTGGCGCTAAAATGAC
CGGCACGTTAATTCAGGCCGGGGCGGCAAAAGGGATGACGGTCAGTGGAGACCCTGCTTCCGGTTCCGCGACACTGTGGAACTCATGGGGGGGCCAGATTGTGGTGGCGCCGGATACA
GCGGGGGGAACCGGGTTTAATAATGGGTTCACCATTACCACGAATAAAGTTCCGCAGTCGGCCTGCGTATCCATTTCAACAGGTATGAGCCGTTCAGGCGGCACGTCCGGTATTAAAATCAA
CGGCAATAACCACACCGATGCGAAAGTAACGGCAGAAATCGCGAGCAGTGAATGCACTGCAGATAATGGCCGGACCGGTACCAATACGCTGGTTTTTACCTACAACGGCTGA
>DLDGEG_03920 PilT lytic transglycosylase_SLT domain protein
ATGTGTGGATATTTTTCCCGACTGGCTTTATTCATGGCGCTGTGCAGTGTGCCGCTGTGGGTACAGGCATTCTGCTTTGATGCCGCTGCGGCGAAATACCATGTCTCCCCGCTGCTGATTAAGT
CCATGGCCATTGGTGAAAGTAATCTTGATCCGCATGCAACAAATGATAACCGGGACAAAAAAACGGGGAAAATCAAAAGCACGGATTATGGACTTATGATGGTGAATTCGACGCATATTCCC
CGTCTTGTCTCGATGGGGGTCATCAGGGACAAAAATGATTTGCTGAACAAACCCTGCCTGAATGTGCAGATTGGAACGTGGATCCTTGCGAAACATTTTCAGGTCTGTGGGGTGAGCTGGA
ACTGCCTGGGCTCTTATAACGCAGGTTTCCGGCCTGACCGGCATGAAACACGCGAGCGTTATGCAAACCGGATCTGGAAAATTTATCAGCGGCAAACGGGGGCTCAATGA
>DLDGEG_03925 Type-IV secretion leader peptidase/N-methyltransferase
ATGACAGTCTCTCTTATCGGTACCATTTTTCCTCCTGTGTATGCCATACCTGCTTTTATCGGATGCGCCATTTTCGGGGCGGTACTGTTAAGGCCGGTACGTCTTTATCTGGCCGCAGCGGAGG
TGGATATCTCACGCCATTGTGCCGTCGTGGTGGGTATCTGGCTGTTTGCGGTAACGGGTTTGCTGATACTTATGGCACCTCAGGTCTGGATGACACGTATCGGAATGCTGTTTATGTGCAGTTT
TTTGCTGCAACTGGGAGTGATGGATGCGACCAGCGGTTGGTTGCCCCGACCGTTTACCGCAGCCTGTTTATGTAGCGGATTATTGTTCTGTCTCGCTTTTCATCGGGAGCCGGAACTTCGCT
TTATGGAAACTGCGGCGATGGCGGTGGTGATGGGGTCTATTTGTCACGGTGTTAACCGACGGCGACCTCAGCTTGGCGTGGGAGATGTCTGGCTGGTTTGTGCCTTGGTAGGCTGGATGG
GGGTAACGGATGCACTGCAGGCGGCGTTTTTTGGGCTGAGTGGTTTTATGTTGTGGCAGTGGATAGTCCATCAGGACTTTCTGCGTTGTGGGGTTCTGGGCCCCTGGCTGTGTGCAGGGT
GCATACCAGTCATTGTGGATAGGTTGTACCAGCCTGAGTGGATACTGAGATGA
>DLDGEG_03930 Shufflon-N domain-containing protein
ATGAAAAAACAAAAACATGATGGCGGCTTTGTGGCGATGAGCGTGGGAGCCGGGTTACTCATTGTTCTGGTGATGGCCTCGTTGGCTGCCCGATATATGGGAGATTATCTGAAATCCCGTGA
GTGGCAGGTTGTGGCCATGCAAACCAATCGTTTCACTCAGGCAGTCTCATCCTATGTTGGAAAGTTTTATCCGACGGTGCTGGCATCAGCGACCATAACTACACCTGCAGTGGTAACAACTC
AAATGCTGAAGAATACAGGTTTGCTGCCGGCGAGCTTTAGTGAGACCAACAGTTATGGCCAGCAGTATCAGGCGATGATAGTCCGGAATCAGCAAAATCAGGAGTTGCTTCAGGGCATGGT
GGTCAGCCGCGGTGGTCATGCTATGCCTTTTACAGCGCTAAGCCAGATCTCGAAAGACATTACCGCCGGCTTCGGAGGATACGTTGAAGACGGGCAAACGGCGGTGGGAGCGATGCGCA
GCTGGCGAATTCCGCTGTCCTCCTACGGGACCAGCACCGGAAGAGGTCATCTGGCCGTCATGTTGAGCACGGATGACCTTTTAGGTGCGCGTGAAGACGGGGACAGGCTGTATCGTTTTC
AGGTCAATGGTCGTCCGGATTTGAACAAGATGCACACAGCTATTGATATGGGAGGAAATAACCTGAATAGCGTCGGCACTGTTACGGCCAGTAATGTGGCTGGCCAGAATGGGAATTTTGG
CGTAAGTCTGGTCAGTAATGGTCCGATCACTGCGGGGGGAGATATTCGAAGCACAGGGGGATGGATTGTAACCCGCAGTGGTAAAGGTTGGATGGATGAGACCCATGGTGGTGGTTTGTA
TATGTCAGATAACGACTGGTTACGGATCCTGAATAACAAGGGGTTTTATACCGGTGGTGAAATTCGCGGTGGGAAGATTCGCTCGGATGGGGACGTATCGGCAGGCGGTATTTTAACGCTG
GATAAGATTAATGTTGCCGGCACTGCTTGTCCTGTAAATGGTGCAATAAGTCGTGATACAGCAGGTGCCACACTTTCGTGCCAATCCGGTATCTGGGTGTCGGGTCAAAACAGATTCACTACA
CAGCTCTACGACATAGGGCAGAATGTGAATGCGCAGTACATAGGCATTCATGCTTATTGCAGTTGGACACATCTATTTTCTGCTCCTCTAGGGGGGCGCCAGAGGGTCTACAATGTAGGGAA
CGCTTGGTATGTCACGAATACCCCTTACGGAGGTTTTCAAACAGGATCGACCGTTTCCGTAACATGCCTGAATCTGCCGGGTGCAGGGCTTTATTCATAA
>DLDGEG_03935 Transposase
ATGTTTAACACCAGTCAGAATAATAAGATGGTTGAAGTGTTATCAGGACCTGAGCGGCGTCGCCGTCGTACTCCGCAGGAAAAAATTGCCATTATTCAGCAGACTATGGAGCCCGGCATGAC
CGTGTCTCATGTCGCTCGTCTGCATGGCATCAATGCTAACCAGATCTTCAAATGGCGCAGGCAGTATGAAGACGGCTCTCTGACCGCCCTGGCATCAGGTGAAGAGGTCGTTCCCGCCTCTG
AACTTGCTGCTGCCAATAAACAAATCCGCGAGCTTCAGCGCCTTCTGGGCAAAAAGTCGATGGAAGCTGAAATACTTAAAGAAGCTGTGGAGTTCGGTCGGGCAAAAAAATGGATTACGC
ATGCGCCCTTGTTGCCCGGGGACGACGAATAA
>DLDGEG_03940 Integrase catalytic domain-containing protein
GTGTCGCGTGCGCAACTGAGCATCAGGGTTAACCGACCATCTGACTGGCAGGATCGCAGACGACAATCCCATTTTGACGACAGTGAGGTGTTGTCCCGGATAAACACAGCGGTCGCTGAC
CTGCCGACGTATGGCTATCGCCGTGTGTGGGCAGTGCTGCGACGGGAGTCAGAGCGGGATGGTCTGCCAGTGGTTAATGCGAAGCGGGTGTATCGTATTATGAGAGTCCACCATCTGCTTC
TTGAGCGTAAACCTGCTGCTCCCTGCCGGAAACGAGCCCATAAGGGTCGAGTGGCGGTAGGTGAAAGTAACCGGCGCTGGTGCTCAGATGGCTTTGAGTTCCGCTGTGACAACGGTGAA
AAGCTGCGGGTAACCTTCGCGCAGGACTGTTGCGACAGGGAGATCATAGACTGGGCGGCGGGCACAGGTGGCTATGACAAAGAGACGGTGCAGGATGTCATGCTGGGCGCAGTAGAAA
AACGCTTCGGGAGGCAACAGCCAACGGAGCCGCTGGAATGGCTGACGGATAACGGCTCAGCCTACAGAGCACATGAAACAAGGGCGTTCGCCCGGATGCTGGGGCTTGAGCCATGCAC
GACAGCGGTGAGGAGCCCGGAAAGTAACGGCATAGCAGAAAGCTTCGTGAAGACGATAAAGCGGGATTACATTAGCATCATGCCGAAACCGGACAGCCGTACGGCGGTGATGAATCTGG
CGGTGGCGTTCAGTCATTACAATGAACATCATCCACACAGTGCGCTGGGATATCGTTCGCCACGGGAATTTATACGCAGGAAGTTATCGCAACCGTAA
>DLDGEG_03945 Shufflon-specific DNA recombinase
ATGTCTCGCCCACAACGCATAAAAAAAATGTCTCTGTCGAAAGCCCTTGATAAATACTACGCCACTGTGTCTGTGCATAAACGGGGGCATCAGCAGGAATTCTATCGTGTTAGGGTCATTCAG
CGACATCCTTTGGCAGAGAAGATGATGGATGAAATCACAACAGTGGATATTGCGTCTTACCGCGATGATCGGTTGTCTCAGGTGAATACGCGTACCGGCCGGTGTATTTCAGGAAACACAGT
CCGGCTCGAACTCGCTTTACTGTCATCACTGTATAATCTGGCCAGCGTAGAGTGGGGAACATGCAGAACTAACCCCGTTGAAATGGTTCGTAAGCCAAAAATATCAGGAGGACGAGATCGAC
GTTTAACATCACAAGAGGAACGGCGCTTATCCCGCTACTTCCAGGAACAGAATCCAGCGCTTCACGCCATATTTCACCTCGCCATTGAAACAGCGATGAGACAAGGAGAGATCTTGTCACTT
CGATGGGAACATATCGACCTGCAGCATGGCGTTGCCCATTTGCCAATGACAAAAAACGGCAGTTCGCGAGATGTTCCCTTATCCCGCAAAGCTCGTCATTTGCTACAGGGAATGACGGTCGC
ACTTAGCGGTAATGTTTTTCACTATTCATCATCTGGTTTTAAAAGCGCCTGGAGGGTGGCTCTGCAGCGCCTGAATATCGTCGATCTCCATTTTCACGATCTGCGTCATGAAGCGATTAGCCGA
CTGTTTGAACTGGGAACACTCAACATTATGGAAGTGGCGGCTATTTCCGGCCACCGTTCCCTGAATATGCTCAAACGTTATACCCATCTCAGAGCATACCAGCTTGTCAGTAAACTTGACGCT
CGCCGAAGACAAACACAAAAGATTGCTCCCTATTTTGTCCCTTATCCTGCCTGTATTGAATCCGTTAATGAAGGATCAGATGGCTGTTGTGGTTTTCGTGTTCACCTACCCGATTTTGACAATC
TTTCAGTGTCAGCGGCATCGAGGGAGAGTGCGCTGGAGGCTGCAGGTGTTCTGTTGCTACGAACGCTGGCTAAAGCGGCTCAGCGGGGAGAGCGTGTGCCTCGCCCCGGGGATTTGCCG
GAAGGCAAGCATGAGCGGGTAATGATCCATCCTCTTTTGAGTGCTGCCTGA
>DLDGEG_03950 Exported protein
GTGGAGTACGCTTTGTTTGTGATGTTCTGTCTGGCGCTGTATTGCCCGTGTTTTACGTTAATTGCGCTGTTCACGCCCTGGAAAGGGTTTGCTGTATTCAGTGTAACGCATCGTCGCCGTCAAT
ACTGGGCTGTACATCTTGCCTTGCTTGATCGGGTGGGGGCGATGAACCGCCGCCAGGCCCGACGCTATCACCAGGCATTTGTTCAGGCGCTAGAGACAGCCCTCGTGGCGCGGCCGGTGC
GTCCCGTTTTTTTCCGTTCACATCTTATGCGGCCAGCTCAGGTAGCGCTTACCTGTCAGGTTCTTTCACACAGGACGGGATATCGCTGTCGGATCGCTCCCATTACGCTTCCGCGCTGGGAAC
GTGTTGCTATTTTTGCGCAAATGCTGCTACAGGAGTGGCGGCTTATTCAATTGCCGTCTGAGGAGGGGATAATGGTAGTCATTCATCGGTTGGCGGAATAA
>DLDGEG_03955 IncI1 plasmid conjugative transfer protein TraE
ATGAATGCAACTTATAACGCGCTAATTGCATTAATGAGATCTGGTGAAATACCACTTGAATCGGGGGCATCAGTACCTGCATCCAGTGCACAGTTTTCAGTAAGAATACGGCCGGAAACTCGG
GTATTTCTGGACAGGTGTGCAGAGCATCTGGGTATATCCCGGGCCGCTATGTTTGGTATGTGCATTGATGGCATTGTAGCAGAGGCGAGAGAGAGTATAGCGGACAGAACGTCCACGCTTTA
TGAGCGATTCTGTCTGCTGTTGGATGCTCATGGATTAAATGTTATCGAACAGGCACAGCTACTGGCTTCCTGGGGAATAAGGGCCAGTGTATTAGCGAGTCAGGATAGGACGCTTGATCTCC
TGAACAAACCTTTGCTGCAGCAATTGTCGACATGGTTTGATGTAGATGTTAACTGGCTGCTGGGAAACTCTTCCTATCCGGTAGATATGGCTGACGGGCAGCGCGACTGGGCTGTGCAGACT
CCCTCTCTGTTATGCCGTCTGCAGTCAATTCAGTCAGTGGATCCGGTTGAGTTAATTTTCTTCTGGCAGCAGGGGCGTAAACCTCAAAGTGTGGGGTTGTGTCTGCGCTATCGCCCCCTTATT

TCAGGTGAGCCGGTCGGGATCCTCAGGGTATATCAGGCACTAGACTGGCAGGATGGGCAGGTTCAGCAGGCATATAATCAATTGCGGCGTATAACCAGTATCACTCCCGGCGGGTATACAA
AAGATAAAGTAGAAAAATATCCTCCTATCCGCATGCGTTATTTCACTTTCTCAGCGCGACAAATGCAGGCATTGGATAATGGAGCGGTTATTCCTGCCATGATATTCGATAAACCTAAGGGTGA
ATATCCAGGTATTCCCTGA
>DLDGEG_03960 Conjugal transfer protein TraF
ATGAAATTTAAATCTCGTCAGTATTCTCTGGTGTATTTCTCTGTTCTTATCGCTTTATCAGGAACTGCGAATGCGGCAACAACTTATATGGATGCCCGCAATGACGCAATGGGCGGAACCGGCG
TGGCCTCATCTCATTACGGAACAGCGGCATTCGCAAACCCTGCTTTGCTTACGCATTTTGGGGCTTCTGAGGATTTCAGTCTTATTCTGCCGTCTGTCGGTGCGCAGGTTTCAGATCCGGATC
ATCTTCAGAAGGGTGTGGATGATGTAAAAGATGCCTGGGATCGCTATGACAGCGAATTGTCACCGGGTTCAGCAGCTGATTTGAAGCAGCAGCTTTTGAAATTCAAAAACACGCATGCCAG
TGCGCAGGCCAGTGTGGGGATGGTGGCAACCGTACCGAATCATGTCGCGCCTTTTGCACTGATGGTGAAATCGTGGGGAACGGCAACGGTGGACGGCAAAGTCAGCGACTATGACCTGC
AGTATCTTGATGATGTGGCCAACGGCAAAATTATCCCGACTGACGCTGACCGTGATTCGCTGACATCCCGTGCCTATGGCCGCGCCGCGGTAGTTAGTGATGTAGGTATTTCGATAGCCCGGG
AGTTTAATACTGGCGGGCTTAAATACTCAGTGGGTGTGACCCCAAAATACCAGCGTGTTGACCTGTTTAATTACAATGTCACCATTCAGAACTATGACAGTCATGACTTCCGCAGCAGTGATTA
CCGCAACACTTCCACTGGATTTAACGCCGACATTGGCTTTGCCACTGACCTGAACGCTAACTGGACACTGGGCCTGGTTGCTCAGAATATTGTTCCGCGCAGTATCGATACAAAAGAAGTTA
ACGGGCTGAAAGAAACGTTCAAAATTCGCCCTCAGGCGACGGCTGGCGCCTCATGGCACAACACGCTGGTGACAACGGCGCTCGATGTTGACCTGACCCCGGCTAGTGGCTTCACATCGG
ACAAAAAGCGCCAGTTTGCCTCCCTGGGTGCTGAATTCAATGCGTGGAAATGGGCGCAGCTGCGCGCGGGTTATCGCCAGAATATGGCTTCTTCTGAAGGTAGTGCCTTCACCGCCGGTAT
TGGTATTTCCCCGTTCGATGTGATGCATCTTGACCTTACCGGCCTGGTGGGGACGGATCGCACATACGGTGCGGTGGCACAGCTGAGAGTAACGTTCTGA
>DLDGEG_03965 MobD
ATGACACAACCTGCCAGGAAAAAAGAGGCCGCCACGCACCTGGAGCTGCTTGAAGCTGAACTGACTGCCGCCCGTAAGGTGACGGCCAGATACAGGACCGCGATGGAGAAAGCAGAAA
AACGGCTGGATGCAGCTGAAGACTCTCAGGCTGACGTGCAATACCGGTATGACTGTGCCCTTGTGGCCTCATGGGGCGACACGCCTGACTGGCTGACACTGCTTGACGGCGATGAAAGCC
GGAGTTCAGTGATGTATGAGCTGGCGCGCGATGGTCTGGAACGTCTGGGACTCGGGACATCTATGATTAACATGGAAACCGGCCAGCGGGTGGTCTGGCTGGGGTTCAGTACGGACAGTG
AAGCCGAACTGCAGCATAAGCTTCACGGTGTGCAGTTTATCCTGCCGTTCTTGAAAGCCGGGAGCCAGGGCCAGCGGGAAATCAGTATCTGCCAGCCGCAGCGCGATAAATTTGCGCTGTC
CCTGATGGTGGATGCACGGACTCAGGCGGTCAGCGTGATGAAGCGGGTGTATGGCAGGGAAAAAGAGCGTACCGGTTTTTCGGGGCTGGAGGCGGCGCTTCGCTATATTCGCAGTATCCA
TTTCGATACCAGTATTGAGGCCGGCGGCATGGCTACATGA
>DLDGEG_03970 Inner membrane protein
ATGGTAAATTTAATACCTGATTTAGCAGGCATAAGTGCTTTGTTAAACGGAGATCTTGGAGGTGTCGTACTCGTCATTTTGTTTTTAGGTACACTGTTATTTGTGGGAAATCTTATGCTTATAGT
TTTGGCTTCAGTTAGATTCTTTTTATTTTCTTTTTCTTTGTTGAAAAGTCCTCCTCAGATATAG
>DLDGEG_03975 DUF4231 domain-containing protein
ATGAAAAACACTCATGATAATTTAAGCGCCGCCCTGGAGTCTTTCGGTAGAAAACATGGCAGGGTAAAAATACTATCGGAATCCAAAGAGTTATTGGATTACTTTCATGCTAACACAAGAAGT
CGCCCAATATTCTTTTGGTTATTTATTTTAAGTTTAGCCGAATTCTTTATTATGACAGCTCTATTTAATATCATGAAAAACAATAGAGCAGCAGGAATTTATGATGTCGAACTTTTCATGCAATTCG
CAGTTATGTCTGTTATAATTTTAATGATGCCAGCCATTTATCTTCTGGTGAAGCAAAAAAGTCTAAGCAGAATGGATGACATAATCATAGAACAATGTATTAAAAAAAACGACTGGTCTGTTCCA
GCATTAAAAGCATCATCAAAAATAATATCATCTATTCAGGGTTCTCTTGAAAAAAGGCTCAACACCTTCAAAATCATCTGCGGAGGATTTGCCTTTATCATTGTTCTTTTCACTAATAAGATAAC
AACTGCTATACCTTATGACATAATCAACCAGATTTTATCTGATACCTACCTCACTGGTACATCTTTCCTTATCATCTGCATCACAACAGCAATTATCATTTATACATATATAGAATTTTGCTATTTAGT
TTTTTACATCGGACACTGTTCAAAAATGAAAGCTTCAATTGATTTTCTTATTTCTATATCTGAGGAGGACTTTTCAACAAAGAAAAAGAAAATAAAAAGAATCTAA
>DLDGEG_03980 Plasmid replication protein
ATGGCGACAACGACGGCATCTTCTGTCGATAATCCGTCAGATAATGATTTACCTTTATCCGCTCACCTCACTGAACCTGCCGGACCGGCTATTGATACGTCGGAAAGCCCTGTTACACCAGACA
CTTCAGCACAGTCAGTTGCACACCGACTGGGGGGAAAGGGGAGTCGTCTGTGGCATCCTTCTTCAGACCGCCGTTCCCTCATATTTAAAGTGTCATCCGTTCTGGTGTACTGCGTATTACTG
GGCGCTGTCTTATTCCTCTGGCTGACAGTACGTCAGCAGCAGATACAGCTTACCACCCTTGATGCGGCGTTCCGCAGCGGACAGCTGCAGACACTTCCCGATCGTATGCAGGCCATAGAAAA
CAGGCAGCAACAATTCGTTCCGAAACTGCAGGGAGAAAAATGGCAGCTCAGGCTGAGTGAGCAGGAGCAGCGACTCACCGCGTTTGAGCAGCAATTTTCTTCGCTGGTTACAGAGGTTA
AAGAGACTCAGGCGGCGACTGCCAGGGCTGTTGCCCGACAGGATGAAACGGGCATGAAGCTCGACGCTGTTGATAAAACGCTGACTGAGCAGACTTCGCGTATAGATACCCTCGCTTCCT
GGAAAGACACGTGGAGTAAACGCACTGTGGCGACTGCCACGAAGGCGACCCCGTCACCCGGAGGTGGACAGTCTGCCGCAAAAAAGTCGTCAAAACCACGGGTCATTAAGCCGCCTTTC
ACATTGGTGAGTATTGAACGCAGGGGAGGGCAGGCTTACGCCGTTATTCTGCCGGCGGCGAACAGTGGCTGGACACAGCTGCGCATGGTAAGCCCGGGAGAGAGTCTGTCAGGCTGGG
TGCTGGTGAACATCAGCGGTAACCAGGCTGAGTTTCAGGTTAACGGGCAGGTACAACGTTTAACGCTCTGA
>DLDGEG_03985 TIGR03759 family integrating conjugative element protein
ATGAAACCGATGAAATCAACGTTAAATTGCCCCCGGCAGTGGACTGCGTCAGTCATGCTGGCTGTTATCGTAAGTCTGCCTGCTCTTGCATCAGTGAGTACGGGGCGCAGTGTAAGCGCACC
CCAGCAGCAAAATACGACTACAGAAGGAAGGACGCAAACGTCAGGAACGGCATGGGGACTCAGCGAAAAAGAATGGCAGCAGTATCAACAGGTACTTAAAGGTCCCAGAGCATTTCAGT
CACCTGGAATTGACCCGTTGATGGCACTGGGGCTGGAGGCCGGTTCGGCAAGCGAACGCCGGCGTTTTGCTGAAATGTGGGTGAAGGAAGAGTATGCCCGGGTTGAAAAGGAGCTGGC
CTTTCAGCGGGAAGTAGATGCTGCCTGGAAACGCCTTTATCCCAATACTCTCTCCGTAAACATGGGCAATGCCAGCGGTATTGCCCATGATACACAGGGAAGACTGGCGTTATTTGTGAAGC
GCAACTGTGTACCGTGCGATGCCAGAGTAAGTGCGATTCTGGCAGACAACCGTCCGGTTGATATTTACCTGGTCGACAGTGGCGGCAGTGATGACACCATTCGCCAGTGGGCGCTTGCACA
TCATATTCCGGTCGATAAAGTTCGTAGCCGGCAGATAACCCTCAACCACGATAACGGCAACTGGCTGAAGTATGGTCAGGGATATATGCCGGTTATGCTTCAGCAGGGAGTATCCGGATGGC
AAATCGCAGCATTCTGA
>DLDGEG_03990 SLT domain-containing protein
ATGGCAAATCGCAGCATTCTGACAGCAGTCAGATACAGTGTCCCTGTTCTGATCCTTCTTATCGGGGGTGTTGGTAGTGCAGCTGCGGCAGAAAAGGGACGCGGTCTGCAAACAATTCCCC
CGGGTTACCACCTTGTGGCAACTGAAGTCGGCGTACCCGCTGAAGCCCTGTATTCAGTTTCTCTTGCAGAAACATCCCGAAAACTACCACATGGAGAGCGACCGTGGCCGTGGACACTCAA
TGTTGCCGGGAAAGGGTATCGGTATAACACCCGCCAGGAAGCGTATGGCGCAATGTTGTCGTTCATGCGTCGCTATCCGTTAAAGCGAATTGATGTTGGGATCGCGCAGGTTAACCTGGGCT
GGAATGGAACTTATTTCAACAGTTACTACGAAGCGCTGGATCCATATACCAACCTTCGGGTTGCCGCCCGTATTCTTAAATCCTGTTATGACAACAATCCGGGAAGCTGGATACAGGCCGCGG
GGTGTTATCACCATCCTGCAGGTGGAAAACCAGCACGAATCTATAAGTCCATCGTGAAACGCAAGCTGGCAACACTGGATTTAAACGTGCCAGTAACGTACTCCGAACTTCAGATCGCACAG
ATATCGCCATCACAGCGCCATTTCTCACATTACAACCGTGAACTGCAATGGGTTGAACCCAGGGAGTCCCGCCAGTGA
>DLDGEG_03995 Integrating conjugative element protein
ATGGTCAGTATTTTGCTGACAACATCATCGATGGCAGCACTCAATGTGATTGCTGATCTTGGCGGAGAACCAACAGCGCCGCTTTTTGATGCCATCAATAATAAGAACAACGAGTTCACTCCG
CCGAGGTCACTGAACGCCACGGGCGCGTCTGTACCCGCAGCCCCTGCGGATGTAAGTGATATGCTGCCCGTTACCACACCGGAGATGCGTGCAGGGAAAGTGGAAGCCCGTGAGTTAAAT
CTGAACGGCATGACACCGGTCTTTCTTGTTGGAGATGATTCGCTTTCACGCGAGTGGTTGACTATGCGGCGTGATGAGCTTAAGCGACTTCATGCTACAGGTCTGGTGGTGAATGTCAGCGA
TAAGGACGCATTGTCGGAACTTCAGCAAATCATACCTGATGTCATTCTGCTACCGGCATCGGCAAGCGAAATAGCGCGTCGTTTGCATCTCAACCACTACCCGGTGTTGATTACCGCCACCGG
TCTGGCCCAGTAG
>DLDGEG_04000 Mrr-cat domain-containing protein
ATGATCACAGCCATTCTTCTTTTACTTTTCTGTCTTCTTATTCCTGCCATATGGCGTAAGAAAATGCCGTCCGCACGCGACCGCCGGCACCGGCGCTACCGACAGAACGCGGAGCGTGTGCTG
TGCCGTCTGACAGCGCTTGATTCAGACGGGGCCCGTCTAAAGTATCTTCGAAAAATTAACCCGTATGTGTTTGAAGAGCTGCTGTTGTTGGCGTTTGAGAGGCAGGGACTCCTGGTTGTGA
GAAACCAGACCTACAGTAGCGATGGCGGACTTGACGGGCAGGTTTTTATTGACGGAAAGCGCTGGTTAATTCAGGCAAAACGTTACAGACGGACTATTTCTCCGCAGCATATACGTGAGTT
CGGAGAACTGATTACCCGGGAGCAATGCTGTGGATTTTTTATTCATACAGGGCGGACGGGCAGAAAGAGCGTTAATTACCTGCAGTGCTGGCGAACAATACAACTGATCAGCGGGCAGCG
ACTACTTGATTTGCTGGCGGGGCGCCAGTGCTGGTACCACCGACCAGTAACTAAGTCAGTTGCCGGGCAGCCGGTTATTACATAA
>DLDGEG_04005 TraG-D-C domain-containing protein
ATGAGTAACCGTTATGTCATGGAGGCGTTATTGAGGCCAGCAGTAGAACTCTATTCAGCTGCGGCTGGCGGGGCTGCCACTTTTGTTCTGGTTGTCGCACCATGGTCGATGGCGTTAGCACC
ACAGGTCAGTTATGTGACTGCAGCCGGTTTTGCCATTTTCACAACAATCAGAACTCGTCAGGGTTTGCGCATCTTGCGCTATCGACGCAATATCCGTCGGTTACCCCGGTATCAAATGTCCAG

TGAATCCATACCGGTAAGCCGACATTTTTTGTTTCTGGGGAAAGGCTTTATGTGGGAGCAAAAGCATACCCAACGTTTGCTTGAGACGAAACGGCCGGAAGTTGCTCCCTTTCTGAAGCCG
TCAGTTTTTTACTCCCTGGCTCGTCAGTTTGAACGACGGTATGAGTATACATTCCCTGGGTTATGTAGCCTGACAAGACGAGATTCATTTTTTAATCCGGTACGCCCATTACCCCCTGTTGGTG
GTAATTCGGCGATACATGGTGTCGAGATGGAGGAAGATAATGTTTTGATGAGTCTGACCGAGCGCGTTGGACATCTTCTTGTATTAGGAACGACACGAGTTGGTAAGACCCGTCTGGCAGA
ATTGTTAATTACGCAGGATATTCGCAGAGGAAATGTCACTATCGTTTTTGATCCCAAAGGTGATGCTGATCTGTTGAAACGAATGTGGGCTGAGACGAAACGAGCTGGGCGTAGCGACGAG
TTTTATATCTTTCATCTTGGCTGGCCAGATATCAGTGCCCGTTATAATGCAGTCGGCCGTTTTGGACGTGTATCTGAAGTGGCCTCACGTGTTGCCGGGCAGCTTGGGGGAGAAGGAAATAAC
GCTGCGTTTAAAGAGTTTGCATGGCGCTTTGTCAATATCATCGCTCGTGCTCTTGTCGTTCTTGGTTTGCGACCTGATTATTCACTTATCCTGCGGTACGTAACAAATATTGCAGAGCTTTATGA
GACCTATGCCGAGAAGTTACTGACTCAACAGGCTCCGGAGTTGTGGCTGATGGTTCAAAACCAGATGGGGATCCTGACTGAGGATGATTTACCACGTACGATGCAGGGACAGGTTAATGCT
ATTCGGGTTATGGCCATTGAAGTGGCATTATCCAGTGAGCGGGGCAAAAAAATATACGATCCCGTACTGGATGGGTTGCGTAGCGCTGTACGTTATGATCGAACATATTTCGATAAAATAGTT
GCTTCATTGTTACCTCTTCTGGAAAAACTGACCACAGGCAAAACTGCGGCACTATTATCTCCTGACTACAGTGATATGAATGATCCGCGCCCATTAATAGACTGGCAGGAAATTATACGGAAA
GGCGGTGTTGTTTACGTCGGTCTTGATGCTCTTTCAGATTCTGAAGTTGCTTCTGCGGTTGGTAACAGTATGTTTGCCGATCTGGTTTCAACAGCTGGATATATTTATAAGTTTGGACTGTCTG
ATGATATGCCCGAAGGGAGTAGCAAGCGGTTACCCATCAGTCTTCACTGTGATGAATTCAATGAGTTAATGGGAGAAGAATTCATTCCGATGATAAATAAAGGCGGTGGGGCAGGTATTGAA
GTTACAGCCTATACCCAGACCTGGTCAGATATTGAGGCCAAAATTGGTAATGCAGCTAAAGCTGCCCAGGTCACAGGCAATTTCAATAATATGATTATGCTTCGTGTGAGAGAGAAAACGAC
AGCTGAGTTGCTGACAACGCAATTACCTGAAGTTGAAATTTATACAAAAACACTGGTTTCCGGTGTGACAGATATATCCAATCCGGATCAGGGGAGTGATTTCACATCCAACGTTCAGGACA
GAGTAAGTAGTACACGTGTTCCTCTTTTATCTCCTGCAGAAGTAATGAATTTACCCAAAGGGCAGGCTTTTGCACTGCTGGAAGGAGGGCGATTATGGAAGATTCGTATGCCGCTGCCGGCA
TCCGTTGATGATGAAATGATGCCGGTAAACCTGCAGAGTATTGTCACTACAATGCGTAAACACTACCGAACGGGAGACACCTGGTGGTCAGGGAGTGTGCTCCCCGGAGATAATCTGGTGG
ATGCGCTATGA
>DLDGEG_04010 TIGR03747 family integrating conjugative element membrane protein
ATGAGTACAGAAAAAAACAGCTCTCAAAGTTCTCCACCACCGCGCAAGCCACCAGGTCTGCTGACCCTTGTTTTGTGGATCTGGCCAGTCAGGCTTTTGGCTTTTCTGCTGGTATCCTGGAT
GGCTGGTGTTTTTATTGAATGGGCAGGAATGTTTTTTTTCTGGTCAGCTCAGGGAGCTTTGCATTCCCAGTCCGTTATGAATAAAGAACTTGGCTATTTATCAGCAGATTTTACTCAAAGCCTT
ATTTTTTCTTCACCATCAGTAACTACGATGGGCTGGATTTCGTCAGCTTACCAGTGGGCATTTGTGGACAGTGGATTACTCAACTGGATTCAGAAAGAACAAAGGGAGACATTAAGCAGTTC
TGACTCTGTTGTTTTTTTTCTGGGACAAGTGCAGGCCTGGTTATTATCAGCGTTAAGCGATTATCTGCTTGCGCTGGTTTATGTCACCGTCGTTTTTGCCGTCAGGGTATTAATTCTTGTTTTAT
CCATCCCGTTATTTGTTCTGGTTATCATCGTCGCTGTTATAGACGGGTTGTGTCGTCGTGACCTGAGACGTTACGGGGCGGGTTATGAGTCGAGTTTTCTGTATCACCATGCCAAACGGTTTGT
TAAACCGGCTGTTTATCTCCCTTGCTTACTTTACTTGTCATGGCCTGCGTCGATTTATCCAAATCTGTTACTTCTGCCCGGGGCATTGTTACTGGGACTGACGGTTACTGTAGTGACATCTACATT
CAAAAAATATCTGTGA
>DLDGEG_04015 Conjugal transfer protein
ATGCAAAGATGTCAATTATCTTCCAGACCTTTAAGGGTTTTACTCGTCCTCTCTCTGCCAGTGATGGCCAGTGCAGCTGAAAAGGATGAACTGGCCCTCGCTCTCAGGCAGCTGGACCAGGT
CCAGTCTGCTCTTGAACGCGCGAAAATTGTAGCGGTACAGGACAAGTCTGACGGGCGTTTCTTCTTCGACTATGAGCGGGCAACACGTGATTTGAAAACCATGAAGCAGGGGATTGAAAC
TTATCTGGAGCCTTCCCGGGCACAGCCAAGAGACAAAGGTTCTCTTGTCGGACAATACAGGAAGGAGCAGCCCTGA
>DLDGEG_04020 Integrating conjugative element protein, PFL_4701 family
ATGGCTATGAATTCTGAACAGGCAAACGCTTTTAAAGCAGGGTCAGGTATCGACCCTACGGTACTTAATAAGTTTGTTCTGGGTTTTGTTCTGAGCGTTCTTTTTTTGTGGTTTGCCTGGTCG
GTATTAGTTGTGTTTCGTGGATGGCGGGCAGGAAAAGTCACTGAGCAAAATGCTCTCCATTTTTTTATCAGTACGGCCATTTTGGTTGTGTTTTCAGTATGGATGTTCGCCAGCTGA
>DLDGEG_04025 TIGR03745 family integrating conjugative element membrane protein
ATGCTTTCAAAGAGTAAACATGGGTTAAGAGTACCACTTCCCTGTCTTTTGATTCTGCTGCTACTGGTGGCGAGCTTCTCCTCTCATGCAGGATTACCTACGGTCAATATTCCCGGTGGGGGC
GGTGGGGGTGACATGATGCAAACGATCAAGCTTCTGTTTTCTGGGTTTCTCGTCCTGATGGGGCTAATCATTTGTGCTGTCGGATTTTATATGGTTTCCGGCGCTATTCTGGCGACATTCTCAG
AAATACGCTCCGGTAAAGCAGAATGGGGTAAATTTGTTGCGATTGTCGTTGTCGGTATAGCCATTCTCGTTGTCATTGTCTGGTTGTGTACTGAAGCCGCGAAGATTTTGAATTGA
>DLDGEG_04030 TIGR03750 family conjugal transfer protein
ATGAAAACAATCGAGTTTTTACCGGACAGGCTGAATGCCGAAACGGTAGTTTTCAGAGGGTTTACAACACCAGAACTGGGCTATGCAGCACTTATTGGTGCCGTGTCAGGCTTAATTTTGTC
TCTGATAATGATGCTTATAACGGGGATCGGCTGGCCAATTATTCCAACCTGTACTCTTATCACTCCACTGATATTTATTTTTATCGGTGGACGCTTTCTGGTTCGTTTTAAACGTAATAAACCCAA
TTTTTACCTCTACCTCCGGATTCAAAAAATACTGAGCCATATTGGTATCGGGAGCAAAGTTCTGGTCCGTCATGATCGACGCTGGTCATTACGCAGGAGTAAGCCTGTACGCAAGGGGGGAA
TATGA
>DLDGEG_04035 TIGR03746 family integrating conjugative element protein
ATGAGCCGATTTCGCCATGCTCTGAAGGATCGCGATCAGCATATTTTGACCCTGAGATTAGCGTGTGGAGTTCTGCTTCTCTTATTGATTCTGGTTATTACCGGATGGATGCGGGCGCCCAGT
GACCTGACAATACATAACCCGCCAGATTTACGCAGCGGGAGTACGCGTAAATGGTGGGAGATCCCTCCGAGTACGGTCTACAGCTTTGCCTTTTATATTTTTCAGCAAATTAATGCCTGGCCG
AAGGACGGCGAAAAAGACTATCCGATGAAAATTGCTCAGTTGAGCCCTTATCTTACGCCATCATGCCAGGATTTTTTGAATAAGGATGCTGAGCTACGGAGTCAAAAAGGTGAGTTGCTTG
ATCGCGTTCGCGTCGTTTATGAGATCCCCAAACGTGGTTACAAACCTGAAAGTGTCATTATTGAGAGCGATGACAGTTGGGAGGTCAGTCTGGATTTGGTAGTCGATGAATACTATCACACA
GAACCTGTAAAACGGGCACTGGCCAGATACCCCCTGCATATTGTCCGCTGGGAAGGTGATCCGGAGCGCAATGCTTTTGGTCTGGCGCTTGATTGCTATAAAGGCGTCCCACAACGACTTG
AAGCTGCAGTGGTCCCTGAGCCAGAGAAAAAGGGGATGTTTTAA
>DLDGEG_04040 TIGR03749 family integrating conjugative element protein
ATGGCGATGTTTAAAGTCCTTAATTTTAAGTGTAAATGGTGGCCGATAATTCTCCTTTTATGGTTTTCATCAGCACAGGCTGTGGAGTTGATGAAATGGGAACGGATCCCTCTGCTTATTCCAC
TTTCTGTTAACCATGAGCGAATTGTCTTTGTCGACCGCAACGTTCGTGTAGGTTTTCCGGCAATGCTCAAAGGTAAGCTGAGGGTTCAAAGCAGCGGTGGTACGGTTTATTTACAGGCCAGC
GAACCTTTTGAACAGACCCGACTTCAACTGCAGGGTGTTGAATCGGGCGAGATTATATTACTGGATATTGCTGCCAAAAATGACAATAAGGCGCTTGAGCCTGTTCGCCTGGTTTATTCGGG
AGAAGTGTTATCGGTGAGCGAAAAAGGAATGGAAACGGGTTCTTCGTTGCCAGTCGCCTCTTTGGATACAGAAGATAAAAATCAGACTGTCAGAAAAACGCCGGTGTACAGTGCTCCATT
GCCAGTGGTGTTGACTCGTTATGCTGCACAAAGTCTGTATGCCCCTCTACGCACTGTTGAAGCTGTACCCGGAATTCATCCGGTAAATTTACGCCTTCCCTCGAGACTCACAACTTTATATCCG
TCTGAGAATGTAGTTGTTTCGCCAATAGGGGCATGGGGTATTCAGGAACTGAATGTTGTTGCACTGAAAATCCGGAATCGTTCTTCCTCCAAAGTGATACTTGATGCCCGGACTTTATCAGGA
TCGTTTGTCAGTGCGACTTTCCAGCATCGTTGGCTTGGGGCGGCTGGTACGCCTGAAGATACAACAACTCTTTATCTTGTGATCAGAGGGCGCCCTGAGAATGCCTTTATTGCTGAACCTGC
AGTATTGCCGGCAGTAAAAAAAGGGGAACAGCATGCAAATTAA
>DLDGEG_04045 TIGR03752 family integrating conjugative element protein
ATGCAAATTAAAGCCAATGGCCTGATGAAGGTTTTACTTCCAGTATTGATCGTGGTGGTTTGTTTTATTGGCGTTAAAGCCTGTGGTTTAAAAGATACTACTGAACCTGAACCCGCTAAAACC
ACTAATGCATTGACTGAGTTGAGCCAGGATGATTTGAAAGCACTGGGTATTGAGGGGGATACGTCAGCAGACACGCTACGTACACTTATTGGAGCGTTGCGTGATGTCCGGGCCCGCCAGG
CTGTGCTGGATGAGCAAAATAAGGCACTCATTTCTGAAAATAATAAGCTCAAGGGTGAGAATTCATCGGTAGGACTCCAGATATCAAATGCTGTAAATGCCGCAAAACAGGAAACGAACGA
GGCTCTTGAAAAACAGAAAACGTCCCTACTGGAGAAGCTCAATGACCTGACCGGAACACTGAAATCCCAGGGACAAAACACTGACATGGTGTCGACAGGTTCGTCTTCAGGAACCGATTT
CCCGGTTGGCCTTGGACTGGATCCAGAGAATGCATCCTCTGCAACTCAGGGGGCGGTGATCTGGATATCTCCTCAGGATGCTAAAAAGACAGAGGGTCAAAGCCAGAGTGATAATGATAAA
TTCACCTTTCCCACCTCTTTTCTGGATGATAACCCCGTCAGTCGTCAGCGAGCTGAGTACGATCGCGTTGTTAAAAACAAGCAGGAACTGGACGGAAACGGTGCAGAGTCCGATACACGGC
CTGTTTATACGTTGCCTGAGAACTCAACCCTGGTAGGCAGTAAGGCGATGACGGCATTAATTGGTCGTATACCAATTAATGACAAAGTCACCGATCCTTATCCATTTAAAGTCCTGATCGGTAA
GGATAACCTAACTGCAAACGGTATCGAATTGCCCGATGTTGAGGGGGCTATTGTTTCCGGGACAGCTACAGGGGATATGGTGCTTTCGTGTGTCAGAGGAACGATACATTCGATCACGTTTG
TTTTTTCCGATGGACGTATAAGAACACTTCCCAGACCCGTAGCAAACACTCAGGGCAACAATAATGACAACGCCCGAGACAGGGGCAGCATTGGCTGGATATCAGATAACAACGGAATTCC
CTGTATCAGTGGTGAGCGCAAATCTAATGCTTCAACTTATCTGCCAACGGTTGCGTTACTGGGGATGGGAAGCTCCGCAGGTGATGCGTTAACACAAAATCAATATACCACTCAGAACAATGT
GAATGGTGGGGTGACATCAGCCTTAACCGGCAATGTGGGGCAGGCGGTCCTGGGGCAGACGCTGGGAGGGGGATTTAAAGAAGTTACCGACTGGGTTAAACAACGTTACGGACAGACG
TTTGATGCGGTGTATGTCCCACCTGGTGCAAATGTCGCTATTCATATTACTCGTCAACTGGCCGTTGATTATGAAGACCATGGTCGTAAGGTTCGATATGACTTTAGCTTGCCCGGAACAGGAG
GCCATTATGGTCTTGACTGA

>DLDGEG_04050 TIGR03751 family conjugal transfer lipoprotein
ATGGTCTTGACTGATAATCAGAAAATCACTTCACGTACAGTGAAAATAACTCCCGTTTTATTTTTTATAGCGGGCATGTTCAGTGTCGCGCTTGGCGGATGCTCATCCAGCAAAGATGAATTAT
TGCCTCCGGGAGACAGTTCAATGATGGAGCTCTGGCAGGGGAATGCTCCGACATCTCACGCCGTTGTCAAAGGGCGGGAGGCTATCAGGCGGAGTCTTTCGTCTCAGGAAACACTGGCTT
CAGGGCGTATTGATGAAAGCTACAGCCGCACTCAGGAGAATGAGCTGAGCCAGATTTTCCCCCGGTTACCTAATCCGGATATGGTGATGTACATATTTCCGCACCTGGCTAATGGAAATACTC
CTGTTCCTGGCTACAGCACTGTCTTTCCTTTTTATGCTCAAACCCAGTATGCGCTTCCTGGTGAACGGACGGAGGCACTGTGA
>DLDGEG_04055 Conjugal transfer protein
ATGGTCAGTTTTTCTTTGTTTAACAAAAACAGGCTGAAGTCATCTTCTCCCCGGGAGCCTGACGAGTTACCGGAAAGTGACTTTGCTGTTCATGGCCGTGACCCACTGAAACGCCCGGGCA
TGATGAGTCACGCTGATGAATCCCGTTTATACGAAACCCGTCCGTCCATTATTGATTTTTTGCCCTGGGCCGAGTACCTGAGTCGCGAACAATGTCTGCTGCTTGACGATGGCCAGTCTGTCG
GTGCGGTATACAGTATATCGCCAGTGGCATCAGAGGGGCGCACAGCTGCCCGTCTGGAAGAAATAAGGGATTCGGTTGAAGATGCACTTCAGGACAGCCTTGAAGAGTTTGATTCTAACCC
ATGGGTAGTACAGTTTTTTTGTCAGGATGAAACTGACTTCAGTGCCTATATGAATCGGTTAGCCAGTTACATCAAACCACATGCTCAGGGGACGGCATTTACGAAGGCCTGGCTGGCTGAAA
CAGAACAGCATCTGCGAAATATCACCCGGCCGGAAGGTTTGTTCACTGATTCACTTGTCACCGGGGTTCCTTGGCGGGGACAAAATCGTTCCGTCAGGATGGTGGTATATCGATACCTGCCA
GCGAATTACAAAGATGATTCGCCGGTTGCCGCTCTCAACCAGGTTTGTGAGCGCCTGACTTCAGCCTTATCTGCAGCCGGCGTTCTGAGTGTCCGACAGAATGGCGAGCAGATACATCGAT
GGTTGTTACGTCACTTCAATCCTGAGCCCGACTGGATAGACAAAGATACGCTTTACCGGGAGGCTCGTTATTATGATGATGCACCTGATTCATTGCCTCTTGGCAATGATTTCACTGAGACGTT
GTGGTTTAACCCTCCACGTTCTGATACCCGAAATGGTGTGTGGTGGTTTGATAATGTGGCGCATCGTGCCGTTCCTGTTGAAACATTACGTCGCCCTCCGGGACCCGGTCATCTGACGGGGG
AGGTTAAGCGTGGCGACAATATTAATACTCTGATGGATTTATTGCCTGAGGGCACAATGGTATCGCTCACTATTGTGGCGCAGGCTCAGGACACGCTGGAAGCCGGGTTTGACACGCTGGG
TAAAAATGCAATAGGGCAGAACACGGAATCCAGGCGGACCCGTAGTGATGTGGAGCAGGTGAAGAGTTTGCTGGGCATGCGTCACAAACTTTATCGTGCATCGATAACTTTTTTGATGCGG
GCACCTGATGTCAGTGAGCTGAATAAAAAAATACAGCAACTTACCGCTACATTGCTGACTGCGGGGCTCCAGCCCACGAAACCTGAATACGAAGTGGCGCCCCTGAATGACTATTTGCGTGC
GCTTCCTATGTGTTTTAACCCGCAACTGGATAAAAAAAACTGGTATACCCGCCTGACCTGGGTTCAGCATCTGGCTGGATTGCTACCTGTGACCGGTCGCAGCCAGGGAACCGGACACCCCG
GGTTTAGTTTTTTTAACCGTGGCGGAGAACTGTTGTCTTTTGATCCCTTAAATAGTCTGGACAGAACGCAGAATGCTCACCTTTTGCTTTTTGGTCCGACCGGGGCGGGTAAATCAGCCACC
TTATGTAGTGCGTTATCACAGTTAATGGCCGTTCATCGTCCACGCCTCTTTATTGCCGAAGCCGGCAACAGTTTTGGTTTGCTGGCGGAGTATTTCCGGGGGCTGGGGCTGAGCGTAAATGT
ACTGAGTATCAAACCAGGCTGCGGTATTTCTTTACCGATGTTTGCTGATGCTCATTCCTTAATGCGAGTGGATCCAAAGAAAAACGTGGTTTCTGAGGAAGCTCTACCTGAGCTGGAGCCAG
CCAATACCGACAGCGCAGAAGATGAAAGTGAAGAGCGTGACATTCTTGGCGAAATGGAAATTGCAGCCCGGATGATGATCACCGGTGGCGAGAAGCGGGAGGAGGAACGTCTTACCCG
GGCTGACCGCGGTCTTATTCGTGAAGCTATCATGATGGCGGCGGAGAAAACATATCAGGACGGACATCAGATGCTGGCTGAGGATTTACAGGCTGCCCTGTGGAATATCTCACGTGATAATT
CCATTGGTGCAAATGGCGAGCCTATGCGGAATCAGTCCCGTCGCGATCGTGCTGCTGAGATGGCAGAAGCCATGGCCATGTTCACTCAGGGTTTTGAGGGAGAGTTATTCAACCGTCCGGG
AACACCATGGCCAGAGGCTGATGTCACACTGATCGATTTAGGTCATCTGGCACGTGAAGGCTATGGTGCACAAATGGCGCTGGCGATGGTCTCTTTAATCAACACGATTAACAATATCGCCG
AACGTGACCAGGGAACAGCCCGGGATATTGTGGCCGTATTTGATGAAGCCCATATTTCCACGACGAATCCTTTGTTGTCGCCCTATATGACCAAGGTTGTGAAAATGTGGCGAAAACTGGGG
GCATGGCTGTGGCTGGCGACACAGAACCTTGAGGATTTTCCTGATATTGCGCAGAAAATGCTGAACATGGCTGAATGGTGGTTGTGCCTGGTTATGCCTCCCGATGAGGTGGAGCAGATTG
CCCGTTTCAAAACCCTCAGCGAGGAGCAAAAAGTTATTTTGCGATCAGCCAGTAAGCTCAAAAACTGTTATACCGAGGGCGTCGTTTTATCAAAGCAGGTTGAGGCACTGTTCCGGGTTGT
ACCTCCCAGTCTGTATCTCGCCCTGGGGATGACTGAAAAAGAGGAAAAAGCGGAACGTCAGCGGCTGATGCGAGAGTATCAGTGCAGTGAGCTTGAGGCAGCGATCCGGGTGGCCCGCA
GGCTGGATGAGGCACGGGGTTTTTGTCCACAAAAAGCGGCCTGA
>DLDGEG_04060 Transposase
ATGAGCCTGTTAAAGCATGAGCAATGGTTAATTCTCGGTGAAGATGAATCGCATCGTATTCCGCCTGATGCGCTGCTGGGTTTGCCTCCGCAGGGTAAAGCGTGGATCCGCAAGGAACATGA
TGAGGATATGAGTCGTCTGACTTCTCATATTCAGCGAGCTCTTGGCAATACTTATCGTGGGGTTTGTCATCCACAGCGTATCGGGCATCGTGCCTGCCTGGCAATCCATCTTGAGAATTTACGC
GGAAAAAAGCTGGATATATTAATTACGGTAAGCGGAAAGACAGCATTACCTGCGGAGGAAAGTTACGTTAATCCCCGATGGTACATCGATGTTGCCGATGCCGCAGATGCCCTTTATCTGGC
GCTGTGGCTTATCAGGTAG
>DLDGEG_04065 TIGR03757 family integrating conjugative element protein
ATGAAACGAATCCTCTCTTCGCTGATATTAATCAGCTCCTTTTCTGCAAGTGCGCAAATCGTTGTTTACACCGACCAGGGGCATCCTCCGGTCGGTGCAACACCGGCTATCCGGGTTGTTTATC
TCGATGCCAGCGAACGCTGGCTGCAGCAACAATTTGGAGAGCTTTCTTCTGATCCTAAACAGGCTGTCAGACAGGCTCATTTTCTGCTGAATTCTCCTGACTGGAAGAGACGGCAAAAAGC
GCTTATCGAACATTACAGGGGAATGATTGAAGCATGGCAGCTTGGGTTACTGAAATATCCGGCGGTGGTTTTTGACAACCGTGATGTGGTTTACGGGACTGCTGATATATCCCGGGCAGTGG
CGCTGCGCAGGGGAGAGAATCAATGA
>DLDGEG_04070 TIGR03756 family integrating conjugative element protein
ATGAAATCTCTTCGTCTGAAGCGAGCGTTACTGTCAGTCATGCTTATCACCGGCGGCATGGATGTTCAGGCTGCGTTGAACACTGCCTCGATTGTGGCCAGCAGTATTTCTCCATCGTGTATCA
GCTGGAGAATCAGCGGTATCTGCTACTGGCTGATGTGTACCCCCTTCGGTTGTACAGTAAAAACCTCGATAAAAGTGCATCACTTTATTCCGGAAGCTGTTGTTTCGACTTATCAGGCGTCTG
GTGGAAATCCGTGGACCGAAATGTCATTAGTGAGTCAGACGGCCGGAGGGGTAGAGAATGCTGTAACGGGGACGTTATCCGGGCTTGCTGCCGGTGGCGGAAACCAGGAACAGAAATTC
CCGGGTACCCGTAAGAGTAATGTGCGTTTTAAATATGCAGATGCCATTGGCCATCCTTCAACATCGATTATTGGCGGCCAAATCCCCGGTTACTCATGCAACAGCGCCGCCACACCACTGGTA
CCCTATTTTCTTAGCACGCTTGATGCGCTTGTCTGGCGTACTGGTGTTCCTGAATCGGTTTATCCTGAGGCACTCATTCCTGGCAGACGCGAAATCGGCTCCACATCGGCACAAAATATGTGG
GGAAATCTCTATCCCCGAAGCGGCTTTGTTACTCAGCAGGATGACTACAAATCCGGGGCAATCGTTGCGCAGCGTGTAGCTGACATTATCACCCGAACTAACCAGATCCACGTTTATAAGCCC
CTCACCGGTAACCCGTCTGCCGGTTACTGGCCTCCCGAACCTGTTAAAGAGAATACCGGCACGCAAAATCATAAATGGCAGCAGTTATCACCAAAGCTCTCTATGTCATGCAGCGTATTCCCG
GATACAGGAAAAATTGCGGAAAACGGTAATTACGCCTGGACACTCTGGCAGCCATATAGCTGCTGTAAACGCCGTGGGCAAACGTTTCTCTATAGCACCGACATAAATTAA
>DLDGEG_04075 Integrating conjugative element protein
ATGAGAGCTCAATCCATTTTATCCCTCTGTCTCATGCTGGGTATCTTCAGTTCGGGACAGTTAAGTGCTGCATTACTGGATGCCCCCACGTTGAGTGGCGATGGATATGGAAAGTCAGTAAAT
GGCGCGGTTAGCGACAAGCTGTTTTACAGCATTGGTGGTGGTTCGGTGATTTCCACTCCCGGGACGATGTACGGAATGAAACGTATCGGGCTGGATGCCGGACTGAGCAGTGATTTGATGT
GTGGCAATTTTGATATAAAGACTACTGTCGGAAACCAGCTTAATGGCGTCACGAATGGTTTCAAAGATTTGATGGGGGATGTGATCACTGGTGCGACCGGGGCAGTCTCAAGTATGCCGGC
GATGATCCTTCAGCGAGCAAATCCGGGTTTGTATGAAATGATCACCAACGGTGTACTGCAGGCAGGTCTGGCTTTTGACAAATCGAAACTGAATTGTGAAAGCATGGCCAACAAGCTGGCT
GATTACACTTTGGGTGACAAGAGCAGCTGGAGAGAATCAGCTGTGAGGGAAGAGTACAAGGAGGCGGTCAGTGCCAGTGGCGGGGATGCGCTCCGGGCAGAAGAGGCCAGAACCAAA
GCTTCAGGTAAGGAGGGCGCTACCTGGGTAGGCGGGAAGAAAGCCGGAGGGAAAGGCCAGCCTGCCATTCAGCCAACGCGTGATCTGGCTAAAGCAGGATACAACATGATGAATAACCT
GAGCGTTACCAGTAACAGCACGGTATCCGCCGGTAGTTGCAAGGGGTCAGCATGCCAGAAATTTAAAAACTCTGAAGAAGCTGCAGCAGCGGTCGTAAAAGTTCTGGGCGATCGGAGTAT
TCGCACCTGTAAAGATGCCACAGAATGCGGAAGCGGCGGGATTGATAATGCCCAGGGCACGACAATTGCCGGTACCGGTTTTGCTCCCATGCTTGAGGATACAACCAAAGAGAATCTGGAT
CAGCTGGTAAAGCTTGTAAATGGTACTGAGGCTCCGACTCAGGCCAATCTGGATAAAATTAAAAGCGGCAGCCTTAAGGTAACGCGTGGTGTCATCGATGCCCTGAAACGCGATCCTGATG
CCGGTGCGTTGGTTCAGAGACTGGCTGGCGAACTGGCCATGTCCGACACGATTGAAACTGGTCTGGCGATGCGTCGCATGTTAACGACTGGACAGTCTGAACCCAATGCCGCTGATCAGCC
TCAGGCAATGGCTGAAGCCGATCGCCGTATTGAAGCTCTCGATCGCGAGCTTAACTCTCTTAAGCTGGAGATGGAACTGCGTCGGGAAGTGGCAAAAAATACCGCGTTGGTTGTGGTTGCG
CGCGAAGAAAATCGTTCGGTCACCAATCCTCAAAAAGAATCGCAGACAAATGCTGATACCCGGCTGAGTGAGCTTGAAACACGTAAACCAGGGGACAGCCAATGA
>DLDGEG_04080 Exported protein
ATGAAACTGAGGATCTTAATCAAAACATCTTCATTTCTGAGATTTATTCTGACGCTTGTGTTCATGCTGGTTATGTTTACGTGCCTGTCCCTTGCACTGGCTGATTTAGGGTTTCGTTATCAGTCT
GAAATAGCCCAGTTCCGGATCTGGATGAAGGAAACCTGGTTGTTGTGGCTTCTGTGGCGGCTGATGCTTTACAGCGTGACTGGTTGGGGGATATGGCGTTGTTATCACTCCCCTGGCTCTTC
TCAGGCTCTGCGTCAGAGTCTTCTGCGGATTGCTGTCGTTTCTGTTGGTTTTGTCGCGATTTGCGAATATTCAGTGTTGAGGTAG
>DLDGEG_04085 TraG-N domain-containing protein
ATGACGACATCGAGCTATCTTGAGTACATCCTGACGATTCTGGGCTGGGTTGTTAATAACGGTTTGTGGGATGTTCTGACAGGCACAGGACTTTTTGCATTACCTCTGGGATTTAAAATCATT
GGTATCTGGATCAAAACACGGGAAGAAGGGGATGATGAGGGTAATAAAGGCATCCAGTCGGCCCGACGGATGGAGCACGCTATTTACGGGGCATTTATCGTTATTATCGCCTGCTGTGTCC
CCACTCAGACAGTGTCACTGACAACGCTGACGTTTGATACAACCCGTGCGAAACAGTGTGGTTTCTGGACACCGGTCAAGCCCGGGGATTCAGGCTACGGTAGTGTGGTTTCAACAATGA

GTGGTATGACTGCATCGGCGCCGGTGTGGTGGGCATTTATGCATATGATTTTCAAAGGCGTCACTCAGGCGGCTGTGGCAAGTTTACCCTGCCGGCCAGACCTGCGGCAGGTGCGTTTTGA
CGTTCAGCATACGCAGATAACCAATCCTGCTCTGGCGCAGGAGTTGCAGGATTTTACCAGTGACTGTTATGCACAGGCTTTTGCGTTATGGAAACGGCAGGATGCGGGGAGAACAACGGAT
ATTGATGTACTCAGGGATATTGAATGGCTGGGGTCGAAAATATTTTTGAAGGATTTTTACCCGCAGCTGCATTCGAAGTTGCCCAGGTCCGCGTTCCCCTGGTCGCAAAGCCGCGATGACGG
GTACGCAAATACCGGGCAGGGGGGGTATCCGACGTGTAGCGAGTGGTGGTCTCAGAACAAGAGCGGCCTGAAATCCCGTGTGCTGGATACGGTAAATACCACCACGATGACACGCATGGC
AGCAGCATTTAAGGGAATGGTCAGCAAAGAGGAATACACGGAAGCGCTAATTCGACGTCTGGTCAGTCCGACAAGTCTCTCTGTGTCACAGAATGGACGTACTTATGCTGGTTATGGCGGT
AATGCCGATTTTACGTTAGATAATGCTGTAAATCGCATGGCTTCGGTGATGGGGACCAGTGTGGGTGCCCTTGGAGCATTCCCCGCATTTGATGCCATGCGCCAGGCGTTACCGATGGTTCA
GGCGCTCTTATTGATGGCCATGTACGTACTAATCCCCCTGGTGCTGGTATTTGGGACGTATGAATATAAGACAGTTGTCACTCTCACATTTGTGACGTTTGGGATGCATTTTCTTACGTTCTGGT
GGGAGCTGGCGCGCTGGCTGGATAGCTGGCTTATGGAGATCATGTATGGTTCCGACAGCCATAGCCGATGGAATGTGGCCGGTTTTCAGAATAGCGCAGATGACCTGATCATGAACTTTGTT
ATGGGGACAATGTTCGTGGTGTTGCCGGCAGTATGGATGGGGGCGCTTTCCTGGGCTGGGGTCAGGATTGGTGGAATTGTTGACACGAACTTAAATAAGGGAGTACAGCCAGCTGGGCA
GCATGGTGGCAAAATTGGTGATAAAGTTGGTACTGGAATAGTTAAATAA
>DLDGEG_04090 DUF3742 domain-containing protein
ATGAACTTGATGTTTTCTTCGCCAGTTGAGATGCTAGGGCTATGGAAATACCTACATTCAGGAGCACAGCAAATGAACGCACAAGCACGCGGTGCCCGCGCCGCAAGGCTTTATAAAAGAG
CTAAGTCAAAGCTGATTGCACTCGACCAGTTTTGTGTTCAAAAAGCCAGAAAACATAGGCTGCCTGCATGGACTGGTCATCTCCCGGTAACTACTCTAACAGGGATTCTTATTGCTGGAGTG
ATTTTTGGCTCACTGTTATTTGTGAGTTTAGGCTTATTTATATTTGCACTTATTCTGGTTACATCGTTGCCTGGAAAAAGCACAAACTCAGCGGACTCGTGGAGTGATAACTCTTGGGGAAGCT
CAATGAGAGATGGAAGTGATGGATTTGGTCTATACACTGGCCCAGAAAATAGCCATATTCCAGCAAGTAGGATAGATAATGAGGACGACCTATAA
>DLDGEG_04095 putative exported protein
ATGTGTAGAAGTATTTACAGATTTAGGGTAATTTCAGCTTTAATATTTCCTCTTGTGGCTTTCAATGTCTCAGCCTCTCCTTGTTTCAGTGATAATTCTGTGACAAAAGGCACACAAATGAGTTC
CAAGTTATCCCGCAACAAGGATTGTAATGATTCAGATTCAAGTTCACGTAAATCAGTAAGTGAAAAAATCATAGAATCCATCGCCGAGAAACAGAAGATAAACCAAGAAATTATGAAAATAG
ATCTTGAAATAGGGTTGTTACGAACGAAAAAAGGGAACATGACCGCACAGGATTATGATAGTAAAATGCTTGAGTTGACAAATAAACGTTCGGCTTTAAAAAGAAAAATTTAA
>DLDGEG_04100 Phosphoadenosine phosphosulfate reductase
ATGTCAGATAACGTCATTATGCAACGTAATCAAATACAGAAGATCAGAACCGGGGAGGATGTGCTGTCTGCGGTTACAGGCCGTGTGGAGTGGATATTTGATACCTTTTCTCAGGTTTGTCTG
TCTTTTTCAGGTGGAAAAGATTCCACCGTTTTGTTTCATATCGCCGCCACCATAGCCCGCAGGAAACACCGGCGTTTCTCTGTCCTCTTCATTGACTGGGAAGCCCAGTATCAGTCCACGATT
GAGCATGTTCAGAAAATGAAGGATCTCTACAGTGATGTGACGGAGACATTTTTCTGGGTGGCTCTTCCGCTGACGACGGTAAATGGTGTTTCACAGTTTCAGCCGGAATGGGTCTGCTGGG
AGCCCGATATGGAGTGGGTCCGTCAGCCACCGGCTGATGCCATTACTGATATGGCGTATTTTCCATTTTACCGTTACGCCATGACGTTTGAAGAGTTTGTCCCGGCATTTTCGACATGGTTCAC
GGGCAATCAGTGTGGAGTGGCCATACTAACCGGCGTTCGTGCTGATGAATCCCTGAACCGGTTTATAGGCCTTACCAGTCAGCGAAAGCTCCGTTATGCCGATGACAAACCCTGGACCACC
GCTTCTCCCGAGGGGTTCTATTACACGATGTGCCCACTGTATGACTGGAAAGCCCGGGACATCTGGATTTACCACGCCCGCACGGGAGCCATTTACAACCCGTTGTATGACCTGATGTACAG
AGCCGGCGTCCCCCTGAGGAATATGCGGGTCTGTGAACCTTTTGGCCCCGAACAGCGCCGTGGTCTGTGGCTTTATCATGTACTGGAGCCTGAGACCTGGGCGCGAATGTGTGCCCGCGTC
TCCGGCGCGGCCAGCGGGGCCATTTATGCTAATGAAAGTGGCGCTTATTTTGCCCTGCGCAAACGCATCTCCAGACCAGCACATCATACCTGGCGCAGCTATGCCATGTTTCTTCTCGATGTC
ATGCCACAAAAAACAGCCGAGCATTACCGGAACAAGATAGCGGTATACCTGCGCTGGTATCAGACGCACGGTTATCCGGAAGATATTCCGGATGAACAGGAGAATGACCTGGGCAGCAGG
GATATTCCCTCCTGGCGGAGAATATGCAAAACACTGATTAAAAATGACTTCTGGTGCCGGACCTTATCATTCAGTCCGAATAAGCCCCGGCATTACGAACGTTACCAGGAGCGTATGAAACAA
AGGAGGAAGGAATGGGGGATATTGTAA
>DLDGEG_04105 ParB domain-containing protein
ATGGGGGATATTGTAACTAGGGAACAGGTGGTCAGTCTCATTACCCGGTATTTCAGTCAGCAGATTTCAGATGACGAAAAAGTGGCGGCCCTTAATCATTTTCGCCAGACACTTCATGAACT
CAGTCCCTTTTCCCGGGAGCCTGTGGACCTGGTGTTGTGGGTGAAAGCCGGCGAAGTGGTGGCCAACGATTACAACCCCAATGTTATGGCTGCCGGAGAGAAAAAATTGCTGCAGCACTC
ACTGCAGGAGGACGGTTTTACCCAGCCCGTCGTGGTATCTGAAGAGAAAGGGCAGTACCTGGTCGTGGATGGTTTTCACCGGCAGTTGCTGGGCAGACGCGAGTCAGGAGCAGGAAAA
CGCCTGAAAGGCTGGCTGCCCGTCACCTGTATCAATCCCGAACGAAAGGGGCAGGCGGAACGAATTGCCTCCACGATCAGGCATAACCGCGCCCGGGGGAAGCATCAGATAGCGTCGATG
TCAGATATCGTCAGAGACCTTGCCCGTCTCGGATGGGCAGATGAACGTATTGGCAAGGAGCTGGGAATGGACAGGGATGAAGTTCTTCGTCTGAAACAGATAAGTGGACTGACTGAATTAT
TTGAAGAAGAAGATTTCAGCCCGGCCTGGACAGTTCGCTGA
>DLDGEG_04110 hypothetical protein
ATGTATAAAATCAGTACATTAACCGCAATCTTGTTGATTTCAGTTTCCTCTGGCGTAGTTCAGGCTAAAAGTAACTTTACGCTCGGGGCGGGGGTTGGTGTGGTTGAACACCCTTACAAAGAT
TACGATACCGATATTTATCCTGTTCCGGTTATCGACTATGAGAGTGAACATTTCTGGTTCAGCGGATTAGAGGGGGGTTATTACCTGTGGAACGATGAAACGGATAAACTGTCGATCATGGCG
TACTGGTCTCCGGTTTATTTTAAGCCTGGGGACAGTAATGATCGCCAGCTGCGCCGTCTCGACAAGCGAAGCAGTACCATGATGGCCGGGATGACTTACGTTCATAATACCCAGTCAGGTTTT
CTGCGTACTACCCTGGCGGGAGATACTCTTGATAAAAGTAATGGTATCGTCTTGGATCTTGCGTGGCTTTATAACTACTCCACAGGCAGACTGACTTTAACTCCGGGAATTGGGTTTGAGTGG
AACAGCAAGAATCAGAACAAATATTATTATGGTATTTCCCGTAAGGAATCCACTCGTAGCGGGCTACGTAATTACAACCCTAATGACAGCTGGAGCCCATAG
>DLDGEG_04115 hypothetical protein
ATGAGTGCAAACTATAACTTTCTGGGAAACTGGAATGTATATGGTTTGGCACGTTATACACGTCTGTCTGACGAAATTACCGACAGCCCGATGGTCAATAAATCCTGGAGTGGCCTGATTTCTA
CCGGTATAACCTATGAGTTTTGA
>DLDGEG_04120 DUF1062 domain-containing protein
ATGAAAGTAACATGGACTGTCACTCCTGTGGGGTATCAGCACATCGCAAAACGCTGTCCGTCATGCAATGTAAAACGAGACTTCACGCCTTCAGGTGCATTCAGGGTTAATTCACAAAAGAA
AATGCTGGACGTATGGAGTATCTATAAATGTACTCACTGCGATTACACCTGGAATATAGCGCTGTATTCCCGCCTTCCTGTAAGCAAAATAAATCAGGAACTTCATCACCGGCTGATGACAAAC
GATGAATCCGCAATCAGGCATTTTGCCTATGACACTGCTGTTCTGAAGCAAAACAATGCAGAACTATCACGTCAACCTGATTTCCGTATTCAGGAGCGCTGTCAGACCAGTATAACGAGGCA
ATATCAGATATGTGTCCGAATCAGGTTAACCCACTCTTTCCAAGTAAGCCTGCTGTCCATACTGAAAAAACAACTTATGCTGAGTTCGGCAGATATCAGGAGGCGAATAGACTCCAGGCAGAT
AACTGGCGTTACGGCAAAAATGCTTAAATCGAGAAAGTTGAGGCAGACTGAATATTGTCTTGAGATAACGGAGGAAACATTCTATGCCAGACGCAGAATTAAGTGGATGCAGGCTTAA
>DLDGEG_04125 STAXI RNA
TGGTGCATTAGCTGCACCCACCAATGCCCCGGTGATCTGAGAGGGGCGCCTTCGGGTGATCACAACACCAATGTTCCGGCAATCTGAGAGGGACGCCTTCGGGTGATTGCTTACCAATGCC
ACCGTAGCCTGAGAGGTGGCGCCTTCGGGTGGTACAGC
>DLDGEG_04130 EFASI RNA
TCGTCATGATATTGAGGCGTCAGTAATTACTGACGGGGAGTTAAATTCGCCGTACTGCGTACTGGTTGAACTCCTGCAAAAACTCAACATAGCGCTACCCTGCTACACAATATCAGACG
>DLDGEG_04135 DNA-binding protein
ATGGCAGGGAAAACAGCTCGTTCAAAAGTGGAGAAATTCCGTCGCGATTTTGTGACCTTAGCCCGGGATGCCGGCAGGGCGTACGCGACGGCTGCAGACAGCATGCGTATCGCGAAATAC
TTTCTGAACTACCTTCGTGATAACGGTATTAAGCTACGTCATACCGACAGTATTAAAACACGTCATGTCACCGGATATCTTCAGTTCAGAAAATCACAGGGGATTTCCGTGCGGACAATTCAGA
ATGAGCGGGCTGCTATTCGTGGCGTTTTGAATAAGGCGGGACGTTATAAGCTGGCAGACCCGAATAATCCGCTCCTGAGTAATAAGGCTCTTGGATTAGAAAAAAGTAGCAGGTCGGGAAC
AAAATTACCGTTAAGCCCGGAAGAATTTCAAAAGGCATTTAAGGAGGTTGAGAAAAGCAACCGGGGGGTTGCAGCAACAATGTTACTTTCTTATACGCTGGGTTTAAGAACTAAAGAGGC
AGTGGAGTCCTGTAAATCCGTGATGACATGGAAACGTGCTCTTGAGTCAGGGGAACATTCAGTTCGTGTAATTTTTGGTACGAAAGGAGGAAGAGGACGAAATACGCTGATTGTTGATAAG
AACGCTTTAAGGCAGGCAATAAATTATGCTGAAAAAGTAATGAAGGAAAATAACGGCAAATTGATTGATCGTCCTGATATTCGTAAGGCGATTGATACCTACCGTTATCATGTCAGACGAGCG
GGTCTGACAGGTGAAAAAGCACCTCACAGTATGCGTTATCATTTTTCACAGGAAGCTCGGGAATATTATGAGAAGAATGGTTTTTCTGGTAAGGAAATATATGCTCAGGTGTCAATGGATTTA
GGTCATGGAGACGGAAGAGGGAGATATGTAAAACAGGTTTATTTCAGAGATGGTACTGAAGATGAGTAA
>DLDGEG_04140 STAXI RNA
CGTTGCCTTAGGCAACCACCAATGTTCCAGTGATCTGAGAGGAACGCCTTCGGGTGATTGCACACCAATGTCACCGTAGCCTGAGAGGTGACGCCTTCGGGTGGTACAGCCCTTTAATCTTT
GGAAATGGCTGTCGCAACTTTTGCGATGAAGATATCC

>DLDGEG_04145 DNA-binding protein
ATGAACATCTTTCTGACTTCTCTGGTCAGTATTCTGCGTAAGGCACTGCCACGTAAACGTCACGGCAAAAGTGAATGGATCGCCAATCACACCGGTTATCTGCGTTTTCAGGCAGAGGTCTG
GCTGGATGATAACGACCATTTTCACGCAGTCGTCAACAAACGTTCCGGCTGGATGAATCCACGCTATGAGCAGGTGGTGGATTGTGGCGAGTTTGATTCATTTCACTGTGCCATGAATACTG
CGTACTCCCAGGCACTTGAACTTGCACATCTTCGCTATGCCTGGGAACTGACCGATTAG
>DLDGEG_04150 hypothetical protein
ATGAACGTTTATCAACTGAAAAACCGCATTGACGTTTTGATCTGGCTTTCGCTGATTGAGGGCGATTTGCTGAGTATCCTGGCTTCTCTGAACGCCGGTTTATATCCGCTTCATGATGACAGGC
AGGAGGAACCGGAGTTTGAATGTGCGGTATTTAACTGTGGTATGGCTTTCGGTGAATTTATGGAAAGGCTTGAATCAGAAGATATTGACGTTCTGACAACAGCCGGACATGAACTGACTGG
CTCGATTGAACATATGGGGAGAATGTTGTGTGAACCCGTTTGGACACAGGCTGTACTTCTGGGGCGCAATGAAGCCCGGGCAGACCGGGCTATATGCGCTGCAGAAGCTGATGGCTGGCT
GTATGCTCCTGCTTAG
>DLDGEG_04155 Phage protein
ATGTCTCACATTCACCTTCCGTCGGTTTGTGAAAAAACCGGCAACACACTGACTCCGGAGCAGGAAAACCGCGTGCGCCATCTGGTGCATGAGGAGTGTTATTCCCGCGACCGCTCAGCAC
TGATAAAGCTTATTGGCCTGATGCTCCTGCTGAAGCTGGCGGGAACCCTCATGCTGGCAATGTTGTTACTGGTGCTGCGTCTGTTCTGA
>DLDGEG_04160 Acetyltransferase
ATGATGTTAACCACCCGCTTGTTTGTATTTTTGTTGCACCATCTCTGGCGCGTCATGCGCTGGACCGGGCTGACGGTGTATCACGGTATTTCGGTGTTATATCGTCGAAGGCAGGCCAGACTG
CAGGCCTCCGACTGGCGCCCCCGAACGGTGTATACCGGTCGCTGGCAGCTGGCCGATGATGGCCAGTTCTGCGGTCGGGCCGTTGTTCTCCGGCTCAGGGATGCGCTGAAACCCGGCGTG
GAGCTGCTTTACTTCGGTGAAGAGGCTGCGGAGCCGTATTACAGCGAGCGACAATTTGTAGACGACAGGGAATCCGTGATGGTGGCTGAGCAGATGTTGCTGCGGTTTCTTGGCAGCCGG
CACCGGCGGCAGAACCGGCGAAATGCTCCAGTGTCAGTGCTGGTTACTGACGTAGTGACAACAGCCCCGGTTAATGATGAGGCCGCATGA
>DLDGEG_04165 DUF4864 domain-containing protein
ATGGATATGATGGCGCTGCTGAGCAGCACGCCGCCGGCCGTCGCTATCGAGGAGCCGGTCTGGGAACAACTGGTAAAATACCCCCAGGCTCCGGACTGTGAAAACGAACGGCTGCAGCG
GTTGATATACCACGGTTTTCAGGCACTCAGTCAGGCGCCTTCGGGGCAGGGAATAGTGGAGTTTGGCTATTTCTGTCTCCCGCCTGATGGTGACCTCCATGCCCCACTGTGGCAGAACGTAT
GTATTAAACGTGAATATTCAGACGGTCAGGTGACACTCACTTTCGATCGTTAA
>DLDGEG_04170 Transmembrane protein
ATGAGCGTAATGACCACTAACGAAACCCCTGCATCCACAGTAGCAGAACCGGAAGTTTTTCGAAGAACGCGTAACCGTTTCAACCAGTGGTTACAGGCGGAATTTGACCGACATTATCACA
CGATGAGGGATGGCGGTTATCGCAGCTTTCTGAAGAAGAACCATCCGACGGAGCTGCTGCGCTATGACGAGGCATGCGAGGCCTTACGGCGGGAAGAATTTGCCCGCTTTGCCGAGCTGC
AGACGCTGGGGCTGTACCTGCATCTGCAGGTACAGCAAAAAGAGGCGCAGTTCCGGCGGCGGCGTAACCGCCTGCTGTTGGGGATGACCTTAACGGGCGTGGTGACATCAGTGTTGTTAT
ACGGTCACTTTCATCCTGAGCAGTTTTTGCTGATCGGACAGGAGCTGGCGACGTTGCCTGGCCGTATCCTCGGATTTGTTAGCTGGCTGGTACCGTAA
>DLDGEG_04175 Type I restriction-modification system methyltransferase subunit
ATGAACACACCGTTACATCCGGATGATATCAGCCGGTTTATCTCCGGCCGACTTATCAGTAGCCTGGCTGCAGGCCAGGTACCGTGGCGGGGCACAATTCCCGGGTTACCGGAACACGCGCT
TACGGGCGTGCCGTTTACCGGTATTAACGTACTGTTATTGTGGCAGGCCATGCAGCAGCGTTCGCTTCGTTCAGGAAGGTGGCTGACCGGAGATGACCTCCGCCAACTGGGCGGTCAGGTC
AGATCCGGTGAAAAGCCAGTCACACTGGTCCGCTACCGGCCTTCGTTATCGCTTTTCAAGGTAATTAACCCTGAACAGTGTGATGGTTTGCCGGATACGCTGCAACCGGGGTGGCCACTGC
CTCCACGACCTCAGCCGTCACTGAATGTGATCCGCGACCTGCTTCAGAACAGTGGGGTTCCCGTGATCCACCGGGACAACGTTTTGCCGGTATACCGGGCATTGCATGACCGGATTGAGCT
TCCACCCGTGGCATCGTATGTCGGAGAGGAGACATACTGGCAGGACATACTGAATCTGCTGGTTCAGGCTACCGGACATCCGCAGCGCTTACATCGCTTCGGATTAACAGTGGATACACATA
CCGATGAGGTTCATGAGGCCCTGGTTGCAGAACTGGGCGCCGCATTTCTCTCGGCTGCTCTGGGATTACCGGGAGCGATGCTATCCAGGCTGGATGTGGCGCCCTGGGTGACATATTTACA
GGGGGACCCGTGGCGTCTCTTTCGGGCTGCGGAGGCAGCGCGAAAGGCGATGATGTGGCTGAAAGAGCGAAGACCCTCGATGACGACAGTGGAGATGTGGCAGAAGATGGCCTCATTG
ATTCTTGAAACGCATTACGGTTTTTCTCTCGATGACACCACGCTGGGATGTTGCAGTGTGGTTGAGCGGCATCTTGAATGTGGGATCACTCCATTGATGGCGATTAATGCACTGGCCCGCATT
TACCAGTGGGAACGCTACGACCAGCCTCAGCGGTCGCTGTTTATCAACGAAGCAGGACCAGACAGTGAAATACTGACTTTGTCTGAAATTCGTCCCGAACTGCTGACCTGTTACCGCGTGC
CTGCGCCATCCGGTATACCGGACAGGAAAGCGGAAGCGGTGCAGGTCGAAAGTTTACCTTTGCTCATGGCACCGGCGGTGTCGGAAGGTAAGAGTGCGGCGAATGATGACGGGCCTGAT
GACCCGGATGGTAATGACAATGTTGTGGCGCTGCCCTGGGCTGCCCGCAGGGGAAAGGAGAACCCGCATATACATCGTTTTGTCAGTATCTTTAACGGTATTGCACCTCATGAAAGCCGCTG
GCAGGTATTCAGTGATTTCGTCCATATGGCGGCCTGTTCACTGTACAACGCTGTACATCGGGATCCTGATTTTGAAGCGGACTACATGAGGCGGGTATCCCACTATTCAGCTGAAGATGCAAA
CAACATGGCCCGTTTACTGTCAGAAGTTGTTATGGGGCTGGAATTCAGTCCAACAGATTTCCTTGGGCGAATTTATATGATATCCGGACTGGGAAATTTTCATAACGCACAGTATTTCACGCCT
TACAGCGTTTCGTATGCGATGGCGCGAATGACACTCAGTGACCGTATACCTGAACTTTCCAGCGGGGAACGAGACTTTATTACTGTCAGCGATCCTGCCAGTGGTGCCGGAAGTATGATCGT
TGCGCTGGCAGAAGCCATGCTGGAGGCGGGGTTTAATCCGCAGAAACAGATGGTAGCGTACTGTGTCGATATTGACCCGGTGGCCGCGATGATGTGTTACATCCAGCTATCCCTGATGGGT
ATTCCGGCCATTGTGGCCACCGGTAACAGCCTGACCGTTGAGATTAAACGGGAGATGGCAACACCAATGTTTGTACTGGGTCGTTGGCATCACCGGTGGCAGGTAGAACGGACGCGTAAA
GCGGCCTAG
>DLDGEG_04180 Adenylate cyclase
ATGCTGGCCAGTTTCTATATTCAACGGCAGCTCAGTAAAGCACTGGACCTCAGTGTGAACGCTGAGGAGGTTTTTTACCAGGTGGACGATCGGGAGTCGGATTACGTCAATACCGATATGGT
ACTGAACCGTGACCGGTTATTGTACGTGATGCAGTTTATGCTGGATGACGTGGCGCTTAATCCGGAGCTCCGGGAAAGATGCCGTCAGGCAGAGCGTATTCTGACGCTATGGATTCGTGGTC
TCGACGCGCTGGCAACAGTGTCTGAGGATATGTCGATACTGCCCCGGACGATTCCGGAGTGCAGTGGACGAGTTGATCGTCTGCTGCCGGGGGATCCACAGGCACTGCTGGCGCTTCCGG
ATGATGCATTCTTACGCCTGACTGCCCAGTGTCATCTGATGTCCGGAGAGCAGTTCCCCCGGGAACAGCTTGCAGCGACGATGCCTTACTGGACGCGTTTTATGGCGTGGATAGCCCGTGAA
CTGTATCAGGTGGAGGATCGTTGTCTGGTGCAACTCGGGCGTCTTTATCGCCGGCTGCACGTGGAGCCCCGGAAAATACGGTGCTTTAACCTGGTTTTCGGACGTATTGAGCTGCATATGTC
CGGACGGGATATTGATGAATGTCAGTATCTCTACGCGTATGACGACGCATCGCTGGAAGATTATCTGGAGGAAATTATGGCCGGTAATCTGACGCCGGTTCGCTTTGAAGTGCGGGTAATATA
CCGCAACGATTCGGAGCTGAACGTCTTCAGGCGAGACGCCGATGTGATTGACGTGGAGCATCCCCATGTCAGCGACTGGCAGAATGTGGTCAGCGAGGCCCTGGACTGGATACGCCAGG
AACGGACGTCGCTGGTTGAGATACCTTTAACCCGGCCGGCACTGAAACTGGCTGCCTGA
>DLDGEG_04185 TIR domain-containing protein
GTGAGCATTATTGACGAATATATCAGCCAGCATTTCTCAGAGCGGCTGTGTCTGGACGTAACCGAAGAGGATATTACCTGGCAGCTTCGCGGCAGCCGTTCCGACTACGTGAATACCCGGAT
ACAGTTTGACCGGGAAAAACTGATGGCGGTGATGAATGTCATGCTGTCCGGTCTGGACAGTGATGAAGCCACGCTGGCGCGCTGCCGGCAGGTACTGACGCTGTGGATAGCCGGACTGG
ATATGCTGTCGAAGGAGGCAGAACAACCTGACTGGCTTCCCCGGGTTCATCCTCACAGCAGTGGTCAGTGCGACCTGCTGCTGAAGGGGAATCCTGCGGCGCTGACGGAGGCGGACGAA
GAAACGTACCTGCGGGTGACCGGCCAGCAGGATTTACCGGCACACCGGCGTATTCCACAGGTGACATTCAGCAAAACGGTACGATACTGGCACCGTTTTGAGAGCTGGCTGGCGCAGCAG
TTGCAGGACATTACTCAGCATTGCTATCAGAAACTGAAATGCTTTGTGGCGAACTGCACGACAGAGCCCAGACAGCTGCGCGAATTCCGCGGGGAGTACGGTTCACTGCGTTTATTTGTGG
GGCCGCAGGATATCGATGAGATAGACATTCTGGAGTTCAATCCGGAATACATCGTGTCCTGGGTCGACAAAGTCGCGGACGGGCTTTTTACTCCGGTCTGCTTTGTGGTGAATGTTTACTATA
AAAACGGCATTTTGCTGGAGTCGTTCACCTGGGACAGTGAGGTGGATAACATCAACAGGATGACAAGCAGCGATTATGGTGAGGCGATGAGCCAGGCCATCAGCTGGGTACGTGAGCAG
TTTGAGCAGCCGGTGATTGATCAACTGGTACCACAGCAGCCACGACTGGCTGCATAA
>DLDGEG_04190 DUF3085 domain-containing protein
ATGCTGAAATTCAGTGCAAAAGACTTAAAACCGGTACTGCTTGAGGCACGTAAAAACCACTGTGGCGTGGTACTGGTCAAGGATCATGGCGTTTATATTATGTCTGAAACCGGTGAGCTCAC
TCCGCGTGGCCGAAAAGTGGCTTATGCGAAGAGATGCCATCCGGAAAAGGATGAGGCCTGGTGGGATACCGCCCGGGCAGAAGTAGGGGGTGATGATTTTGGTGAGACGATAAATCTGA
CGAAAAGCATGATTAACCGCATTCTGAACGAGAGGAGAATGTTGTCTATCACGGTCAGCGACGAGGCATGTAAGAGCGAGTGTTAA
>DLDGEG_04195 Integrase
ATGCGTAAATATATTTCATATGAAGAATGGAAGCAGTTTTTTAACGCAATAAAAGGAGGTCCTAATGAACTTAGGGATAAAGCTATTTTTAATATGATATATATACATGGTCTCAGGGTCAGTGA
ACTGACAGGTGTCAGGATAACCGACCTGGATTTGACGAATAAAACAATCTATATAAGACGGCTGAAAAATGGTTTTTCAACCACTCATCCTCTGCAGAAAGAGTCTTACCAACTTATAAGAAA
ATGGCTTAAGGTAAGGCCATTTTACATTAAAGTCGAAGATAATGACTGGTTGTTTCTTACATCACGTGGTGAAAGTATCTCACGTCAGTGGATATATAAGCTGACCCGAAAATATGGTATAAAA

GCTGGGTTGCCACTTAAAATTCACCCACATATGCTACGTCATTCCTGTGGCTATGCATTGGCAAATCAGGGCCTTGATACTCGCTTAATACAAGACTATCTGGGACACATAAATATCAACCACAC
GGTACATTATACGGCAAGTAATTCTGCAAGGTTTATGCGTGCATGGCAACACTGCGCGGATTAA
>DLDGEG_04200 Helix-turn-helix domain-containing protein
TTGACAGTCTGGCAACTTGTTGAATGGATTGAGCATCATCTCCATCAGGAGATTACACGAACTGTTCTAGAAAAAAAGGCCGGTTACAGTTCCCGGCATCTTTACAAATTATTTATAGCAGAT
ATTGGGATCAGCGTAACTGCCTATATCCGTAAACGACGGTTAACGCTGGCATCTGTCATGCTGCGGGAAACCAATAGAAGTGTAACTGAAATAGCGCTGATGTATCAGTTTAACTACCTGCAG
ACATTCTCAAGAGCATTCAGAAAACAATTCAGATTATCACCGCTACAATATCGTCATGCCAGATATTGGGATATGAAAGATTATTACCCTTCTGCAATAGTAAAAAAGTTTAGCTGTAAAACACA
TGTAGCCTGCGTTAACAAAGATGCGTTTATCTCACCTAAAAACAAATTTTCTTACAAAATTAACTTTGGATACACTTTCATTATCAACATAAAAGATAAAAAAATTAACTCTTACCCTCAAATTTA
TCAAGACTGCATTAATTTCATATTCCAGAAAAAGACCAATCAGCCTTTAGTTGTTTTTGGAGAACTAAAACCAGGTATAAAATCCGATACTGTAATCGATATTTTCTCCGGATACTTCACCACTC
AAAAAAGGCCAGGTGTCATTAATATTCCTGATGGCTATTATTTCTGTTTCACTTCAATAGGCACGCCTTTAGAATTAATGAAATTTATCAACTGGGCTAAAGGTCATGGTATGCACTGTTTTGGA
CAGGTACTAAAAAAAGGTCCAACCTTTACAGTTTTTACCGAAACCAACACCAAGGATGTCTATAAGGCAGAACACTATATGCCCTGTATTAATCCGCGCAGTGTTGCCATGCACGCATAA
>DLDGEG_04205 Tyr recombinase domain-containing protein
ATGTCCAAACGTAAGTTCCTTACTCGTAATGAAGTTTTTTCCATGCTGAAGGCTTCCCTTGACAGTTCGCATCCTGAGCGTAATCAATGCCTGATCCTGATGGCCTTTATCCATGGCTTTCGTGC
CAGTGAACTCCTTTCCCTGCGTCTGTCAGATTTTGATTTACGCAGTCGGATTATCCTGGTGACTCGACTAAAGAATGGTTTTTCCACTGTACATCCCCTGTCCCCCCGGGAACTTAGAGTGCTG
CAGGACTGGTTGCGGATACGCCAGCAGATAATTTCGCAGGGCCGGTATGAAGATGAGGGGTGGCTATTTATTTCGGATAGGGCTCCCAGCCTGTCACGCCAACAGTTTTACAATATTATCAG
TCACACCGGGCGGGCTGCCGGGCTGACTGTAGGGGCACATCCCCATATGCTCAGACATGCATGTGGTTATGCTTTGGCAGATAACGGGGTGGATACACGGTTGATACAAGATTATTTAGGAC
ACCGAAACATCCGACATACGGTCAGATACACGGCCTCCAGTGCTGCTCGATTTGAAGGAATATGGCAACCTGCAAAACGCAAAAAGGGGCAACATTCTGACCCAAAATGTAAACCTACTTTT
TACCTGTGA
>DLDGEG_04210 Type 1 fimbrial protein
ATGATGACTAAATCTCTCGTTGCAGGTGCGCTGACCGTTCTGCTGGCTTCTTTTGGTGCTAACGCAGCCAATCAGGGACAGGGCGTTGTGAACTTCAAAGGTACCGTTATTGATGCACCTTG
TAGTATCGCTCCGGAATCTGCAGACCAGACCATCGACTTTGGTCAGATCTCCAAAGCGCAGTTAGCCAATGGTGGTACTTCCATTCAGAAGAATGTAGACATCAAACTGACTCAGTGTGACC
CGGCTGCTCTGACCAAAGGTGTGGCAGTGACGTTCACCGGTAACACTGCGACCGGTCAGGCATCAGAGTTGGCGACAGCGGGTGCCACCAACACGGCTATCGTCCTGAATGGTTACGGCA
GCGATATCACTTTTGGTACGGCGTCTGACCATGTGAAACTGGGTGCAGGAGATAACACTCTGCGCTTCACCTCCTGGGTCAAGCAGGCTACAGGCAAAACTGTCGCAGAGGGTGACTTCAC
GGCGATTGCGAACTTTAACCTGTCTTACGAGTAA
>DLDGEG_04215 PAP fimbrial minor pilin protein
ATGAGCTGGCGAGCTGTGATGCTGGTGGTGATGGTCGTGGGGGGGGCTCGGGCTTTTGCCTCCGGCGTATTTCCTCCGCCTGGTATGTCGCTGCCGGATTACTGGGGAGAAGATAATGTCT
GGTGGGAAGGGCGGACTTCATTCCACGGACAGGTCATTGTACCGGCCTGTGCGCTGGCCATGGAGGATGCCTGGCAGGTGGTGGATATGGGAGCAACACCAGTCAGAGATTTGCAGAAT
GTTTACGCCGGACCGGAAAAACGCTTTCAGCTACGTCTGCGTGATTGTGAACTGGCCGGTACAGGGAAAAAGGTGTTTACCGGTAGCCGGATACGGGTCACGTTTGATGGCGTGGAGGG
AGAGACACCCGACAAGTTTTCCACTGTGGGCCAGGCCCGGGGGGTGAATCTGCAGATAATGGATGGTCAGGGGTATACCGCGCGCGCGGGTAAAGTGATGCCGGCACAGTTGCTGGAAG
GTAATGAACAGGGGCTGGATTACACCCTGCGGGTGGTACGCAACGGTGAGCCATTGAAGGCCGGGGATTATTCTGCCTCCCTGCGTTTTAAAGTCGATTATGAATAA
>DLDGEG_04220 Outer membrane usher protein PapC
ATGATAATGAATAATAAAAATACCCCCTTTAAAATAAATCGGCTGGCGCTTGCGGTACTGATGATTATGTTCTGTGATCAGGCACAGGCTGTGGATTTTAATACTGACGTGCTGGATGCTGCGG
ACAGGAAGAATATTGATATTGGTCGTTTTTCGCAGGCCGGATATATCATGCCGGGCCAGTATCAGATGCAGGTTATGGTCAATGATCAGAGTATTTCACCTTCTGCGTTTACGGTTTCGGTGGA
GGAGCCAGCGGTAAATAGTGCCGCATCCGATAAAAAAGCCCTGCCTCAGGCCTGTCTGACGACAGAAATGGTGGACAAAATGGGTCTAACGACGGCTTCCCGGGAGAAGGTGACTTTCCG
TGATAATGGTCGGTGTGCCGACTTCAGTGCCCTGCCTGGCGTGGAAATACGGACTAATCCGGCTGAGGGAATATTATATATTAATATGCCGCAGGCATGGCTGGAGTATTCCGATGCTTCTTG
GCTCCCTCCTTCCCGTTGGGACAATGGTATTCCTGGACTTCTTTTTGACTACAACATCAATGGCAGTGTGAACAAGCCTCATCAGGGCAAAGAATCTCAGTATGTCAGTTATAACGGGACTAC
TGGGGCGAACCTGGGGGCCTGGCGTTTCCGCGCTGATTACCAGGGATATCTTTCACATACCACCGGGCAGAGATCGGGAACGGATAACCAGTTTGACTGGAGTCGTTTTTACCTGTACAGG
GCGCTCCCGCGCTGGCAGTCAAAATTGACGCTGGGTGAGAACTATACCAGCTCAGAAATCTTCAGTACCTGGCGCTATACCGGTGCCAGTCTGGAAAGTGATGATCGCATGTTGCCGCCGA
AACTACGTGGTTATGCACCGCAGATATCGGGGATTGCGGACACCAACGCGCGCGTGGTGGTTTCGCAGCAGGGACGTATTCTTTACGATTCCACCGTGCCGGCAGGGCCGTTCACCATTCA
GGACCTGGACAGCTCCATTCGCGGACGTCTGGATGTGGAGGTCATTGAACAGAACGGGCAGAAGAAAAACTTTCAGGTGGATACCGCGTATGTCCCGTATCTGACCCGACCAGGGCAGAT
TCGCTACAAACTGGTCAGCGGGCGTTCACGGACTTGGGAGCATTCGACGGAAGGACCGGTTTTTGTCGGCGGCGAAATGTCCTGGGGGATCAGCAACCGCTGGTCGCTGTACGGTGGCG
GTATTATTGCCGGGGACTACAATGCGCTTGCACTAGGTATCGGACGGGATCTGCTGCAGTTCGGGACACTGTCAGCTGATGTCACACAATCGGTAGCCCGGTTTAAAGACGAGGGTGACCG
GCAGGGTAAATCTTGGCGTCTGAGTTATTCCAAACGTTTTGATGAAGCTGACGCCGATATCACCTTCGCGGGATACCGTTTCTCTGAACGCAACTACATGACGATGCAGCAGTATCTGGACGC
ACGCTACCGGAATGACTTTACCGGACGTGAGAAGGAACTGTACACCGTTTCGCTGAACAAGCATTTCGAAGACTGGCAGACCTCAGTGGGGTTGCAATACCAGCATCAGACTTACTGGGA
CCGTGGAGAGTCGGATTATTACACCCTGTCCATTAACCGCTATTTCGATGCCTTTGGTCTGCACAATATTTCTGCCGGTTTGAGTGCATCCCGTTCCAAATACCTTGACCGGGATAATGATTCTG
TTTTTCTGCGCCTGTCAGTCCCGCTGGGAACCGGGACTGTCAGCTACAGCGGCAGTATGAGTAATGACCGGTACACTCAGATGGTCGGGTACAGCGACACGCTTAATAACGGGCTGGACAG
CTACAGCGTGAATGCGGGTCTGAATCATGGTGGCAGTGAGCGCAGTCAGGGGCAGATGAATGCGTATTACAGTCACAGCAGCCCTGTGGCAAACCTGTCAGCGAATTTTGCATCGGTGCA
GAATGGTTACACCTCCTATGGCATCAGCGCTTCGGGCGGGGCAACGCTGACTACAAAAGGGGCAGCACTGCATGCGGGAGGGATGAATGGTGGAACCCGGCTGCTGGTTGATACCGATG
GTGTCAGTGGCGTACCTGTCGACGGTGGCCGGGTTTACACCAACCGCTGGGGGCTGGGGGTGGTGACGGACGTCAGCAGTTACTACCGCAACACCACCACGGTTGACCTGAATAACTTAC
CTGATGATATGGAAGCGACCCGTTCGGTGGTGGAATCCGTCCTGACCGAAGGGGCTATCGGTTACCGGGAGTTTGAGGTTCTGAAAGGAGCCCGGTTGTTCGCGGTTCTGCGTCTGTCGG
ATGGCAGTTATCCGCCGTTTGGTGCCAGTGTGACGAACCCGAAAGGTCGCGAGCTCGGGATGGTGGGAGACAGCGGACTGGCCTGGCTCAGTGGTGTCAGTGAAGGGGAAATCCTGAAT
GTTAACTGGGATGGCAGGGCGCAGTGTGCCGTGGACATTCCGAAGAAGCTGGATCCGGCTCAGCAGCTGTTGCTGCCCTGTCGTCAGGCGACGCGTTAA
>DLDGEG_04225 Chaperone protein PapD
ATGGTGATACTGAACCCGGTGATGAGAACAACACTGATGGCGGTAGCGTGCCAGGCCGTGGTCCCGGCTGTGCAGGCAGCAATATCGCTTGACCGGACACGGGCCATTTTTAACGGGGCT
GACAAGTCCATGACGCTGAATATTGCCAACGATAACAAACAGCTGCCTTATCTTGCACAGGCGTGGCTGGAAAACGCAACCCAGGAAAAAATTACGGCGGGTCCTCTGGTCGTCACGCCGC
CCGTACAGCGTCTGGAGCCGGGGGCGAAAAGTATGGTGCGTCTGGCCTCCACACCGGATATCAGAAAACTGCCACAGGACCGCGAGTCACTGTTTTACTTCAACCTGCGGGAAATTCCGC
CGAAGAGTGAAAAAGCCAACGTGCTGCAGATTGCCCTGCAGACCAAAATTAAGCTGTTCTATCGTCCGGCCGCCATTCAGGCAAAACCCAATGCGGTATGGCAGGACCAGCTGGTGCTGA
ACAAAACGCCAGGCGGATACCGGATTGAAAACCCGACACCGTATTACGTGACCGTGATAGGCCTGGGCGGGACGGAGAAAGAGGCAAAGGAAGGCAAATTTGATGCCGTGATGCTGGC
GCCAAAATCAGAGCAGACGGTGACGGCCGGCAGTTACGGGACGCCCTGGCTGACGTATATCAATGATTACGGCGGGCGTCCGGCCCTGCAGTTTCAGTGTGCGGGCAGTCGCTGTACGGT
GGTGAAAAAATAA
>DLDGEG_04230 PixJ protein
ATGAAAAAAATGACCATAACGCCAGGTCTGACGGCCCTGATACTCAGTACAGCCATGAACAGCGTACTTCATGCCGGAGAATTACTGACCCGGGATGATTTTTATCTGGCGGATGAATCCCG
GCACGTCATAAGTAATGAGCATCATGGCCGGATGGGGGAGCTGGAGGTACGTGGTGCCCTGCTGTCATCACCCTGTACGCTGATAACGAATGAACTGGAGCTGCCTCTGGCACGAGAACCG
GGGGGGGGAGTGAGCCGTTATCCCCTGCAGCTGGCGCTGACGGGGTGTGGTGACGGGGATGATGTAACTTCAGCCTCGACGCCGGCCGGACGTTCTTCGGTGATGGTAGTTTACAGCGC
ACTGCTGAGCGGTACTGAAGGGGGCGTACTCCACCCCGCTCAGAGGATGGTGGGGGCAGGCAGGGCCATACTGCGGGGAGGGGCGTCGCAGATGACCTGGTACCTGACAGAGGCTCAG
CAACTTGCGCTGCAAAAGTCAGTCAGGGGACTGGACAGAGCGCCTTTTAACAGCCCGGTATCCGGCTCGCTGTTGCGACTGCGGATGGATTATGAATAA
>DLDGEG_04235 Type 1 fimbrial protein
ATGAAGCACACGGTACTGAAGAGCTGGTTCCGCGCGGGATTGTTGCCCGCCATAGTATCAATGGCCCTGTCCGGCCAGGCACAGGCGGTGACGGTAAATTTCGACGGCGAACTGCTTGAC
CGTCCCTGTCAGGTGGCACCGGAGAGCCTGCAGCAGGACGTCATTTTTCTGGAGCGACCCGCGCGGGATTTCTGGATGGCACCCGCCCGTAGCCCGGTAGAGAAATTCACTATCCGGCTG
ATGGAGTGTGACACCACATCCATCTGGAAAGTGGTCAAACTGAAATTCAGCGGCACGCCTGAAAACAACATGACGGGCCAGGCCGATTATTTTCTGAAAGTGACAGGCGATAACGCGGGA

AAACTCGCGGTGGGTCTGCTTGATACCGACGGAAAAACACCTCTGAAACTGGGCGAGTTTCAGAATAACGGTCAGGGAACGCAGATAGATGGCCGGGATGTGTTGCTGAAGTTTGGGGC
TTTTGTTCAGGCGACGCCGGATGCGGTCACACAGAAAAGTGTGCGGCCTGGTCAGTATACCTCTACTGCTAACTTTGAGTTGATTTATGAATAG
>DLDGEG_04240 peptidylprolyl isomerase
ATGAATAGGCCGGAACGCTTTTTTTTAAGGATGCTGCAGTTTTTCTCCCTGCTGCTTATTCTGGCGGTTGCGCTGGCTGGTGGGATGTTAATCAGCTGTCAGTCAGCACAGGCAGCTGAAAA
TGGTTCCCGGATACCAGGCGTACTGGAGTATGCCCGTAAATACGGTGAAGAAAAGCAGAAAGCAGCGGCGACGCAGAGTGGGGGTAAAAAAGCAGAGCAACGCCCTCAGGCGGTGAGA
AAGGAAAGCGCGAATAAAGTCCCGTCGGAAACCAGACTGAGGCAACGTCTCCGCCAGCAGGAGGTGATGCTGGAGCAGGTCAGGCGGGAAAACCAGCAGTTGCGTCAACAGTGGGCA
AAACAGGCCGGGGCGGCCTCCGGTGAAAATGGGATGTTACAGAAACAGGTAGTAACACTCAGTGCTGAGCTGAAGCAACTGCAGGTGGTGCAGAAATCTGCACAGGAAGAGCAGGCCC
GTCTGAAATCGCTGCTGGCTGAAATGCCGATAGCGACGGCGGATGAACTGAAGAAGCCGGTAGCCCGGCAGGATTATGCTGCAGGAGTGATGACTGGTCGCGACATGCTGGTGATGCAG
GAAGGGCAAGGACTACTGGGGTTGAAGACGGACAACCGTCTTCTGCTGGCTGGTCTACGGGATGCTCTGAATCAGCAGGTGAAGTTGAATTCAACTGCCCTGCAGGAGGCTTTGTCGGC
TGCAGAAGTCCGGACCCGTCAGGCGCGGGAACGGATAGTTATCGCTCAGAAGGCTGTCGGGGAGAAATATCTTGCAGCGTTCAGGAAAGGAAAAGGTACGGAAAAGGATGACAGCGGG
TTCTGGTACCGACTGGAGTATGCCGGAGACGGTGAGCCTATCCGTGGGGAGGATACCCGGGTGGAAGTGGTGGTGAACGAAAAACTAACGGACGGCAGTGTGGTGGAGGATATGGATGC
GTCGGGACGCAGTCTGGTGATGAAGCTGGGGGATTACCCTCCGCTGTTCCGCCGGGCACTTGAACGGATGAAAAACCACGCCACAATGACACTGGTTGTGCCGCCTGAGCTGGCCTACGG
GGATGAAGGGTATCCGCCGAAAGTTCCCCCTGGAGCGACCATGGTTTATACCCTGCGGGTGGAGAGCGTGACGGCAGCAGAAAAAGGTGTCCCCGGGAAAGACGGGGAAAACAGTGCT
GCAGGTGAGAAAAATGAGAGTCGTAAACCGAAATTATAA
>DLDGEG_04245 Fimbrial protein
ATGAACATTCTTTTAAAAGTAATATGTCCGGTGGTGGTTGGTGGGGTGATGCTTAATATCAACAGCGCCCAAGCGATAGATGTAAATGTCAGGATCACGGGGGAGATATTAATCCCGCCCTGC
AAAATAAATAATGATGCCGTTGTTAACGTTACATTTGGCAAGATCGCTTTGCAGAAAGTAGATGGCGTACAGTCAGCTGTAAGTAAAACACTGGATGTGAACTGTGATTACTATCAGGGCACA
CCGTACATAGTGGTGGCAGGCAATGCAATGACAGGAGTGGTGGATAAGAATGTACTGGAAACAGCGGGTGATAATGCGGGACGATTGGGGATTGCATTGTATCAGGGGAATGGAGTGAAC
ACCTCTGCTCCGATGAAAATTGGGACAGGAGGGGATAAAGGTGCTTACGGTTATCCAGTAATCCGGGGGCTGTCAGGCAGTGGTGCAAGCGGGCAGTTTACATTCACTGCTGTGCCGTGG
AAAAAAAGTACCGCGCAGCTGCAGGCCGGACAGTTCAGCGCTACTGCAACGATGAGCATCAGTTATCTTTGA
>DLDGEG_04250 Lipoprotein
ATGAGTGACGGGAACAGAATAATGAAGAAACATATTGTATTGCTGGGAGCTATGGTCGCATTGGCTATGTCAGGGTGCTCGATGAGGGATAAAAAAATACCATTAGTCTCCCAGAATAAAAG
TCCGTCAGTGCAAACGGTGGGGGGGGCTGACTCTCTGGATGATGCGGTCCGGATGGAGCGGTGTCGGCGTGAACTGGATGCGCTGAAGAAAATAAACGGAGCAGTCTATGCAAAACGTA
AACAAGAGTTTGATACGCTGATGTCCGGTGCAGAAATTTATACAGGTGTAAGGGAAGATGTTTCTACGTATACACAACAGGCGGTTGATGCATATTATCGCTACCATGCTGATAAACTCTGTGC
CGATATCGCAATAGATGTGCTAAGTAATTTGGCAAAGAAGAAATAA
>DLDGEG_04255 hypothetical protein
ATGAATAAATTATTTTTCTCGATGCTAATTGTAATACTTCCTATGGATGCAATTGGTGCTGTTTATATGCAGTCAAACGGTAGTGGACGTGTTAATGCGACTATTTCGAATGTGGAAAATGGTGT
GGGACGTACCATATTAAGTTTTGTAAGCCCCGCAGGTGGGCGCGGGTATGGTATGTATTGTAGTCAATCCGGCATGCAAAGATATAATTATGTTCGTGCTTTTTCAATATTAGAGAAAACGGTG
ACTGTCAAAAATAGTTTGACAGGTGTGTCATATAATGTTCCTGTAACCTGTCCCTCTTGCTCATATTCAGGTGAAAATGGCTTTCTAAATGTTAGTGGTCCACAATCTTACCTTCAAGATCATGTA
TGCTATAGTGGTTTAACACCAGATCCATATGGCGCTACGACTTCGCTTCAGACAACAGTATATATTTCATTGCAAGACTCTCGTATACCAGCTGGTCGTTATGAAGGAACTATAGTGGGGAGCG
TTGGCGAAATATCCGAATATTATGCGGGAGAGTATGTTAATTATAGTAATGAAATGATAAATAAAGGAATGCAAACAGGAACAGCAGGTGTGGTTGCAATGAAATTCGATGTAATTGTGCCAG
AGTATACTGTATGTACATCTGCAAGTCCTATATTAATAGACCACGGCTCGCTCACGCCTGAACAAGTGCGCGGGAATGCAAGGAGTCAACAATTCTCTATTTCATGTAGTGGTCCGGCGAATAT
AAATGTATCTTTGAGTGATTTAATGCCAAAGATTGGTGATGGTATTTATAGTAAATTGAGTCTTTCTGCTGACAATGTGACTTGGAAAGATAAGGTAGATACCATTTTAAATGATAGGGGAACA
CAGATTATATATATATCCTCGACTCTAGAGGCAAGTGGTGATATTTATGCTCAGTCTTTAAGTGGGTATTCAGTAATAACAATAAACTATCATTAA
>DLDGEG_04260 Helix-turn-helix transcriptional regulator
ATGCCCAATAATATATCTGTCATTGTACTGACACCGTGTCATTTTTTATTTTCGGGTCTGAAAAATTTGCTATGTGATACCCAACTACAACTGCAGTTTCTGAATGCTAGTACTGTAGATGAAGTT
CTGAAGTTACAGACGATTCCCGGAGTATCGATGATAATGGTGGCCAGCGAATCCTTTACCCCAGTGGAGAGATCTCGAGCACTGGTGCTGGTTCGGCATCTGGATTGGCTGATGTTAAGCGG
AGCAATGCCACGGATTCCCTGTCTATTGTTGATGAGTGATATGAGTATCAGTGTATCCGGGAAAATCTTTTGTCTGACACGTGGGCAGGCGGGATATGATCTTGAGGTACTATTGGGTAGCAT
ACTAGCGCATATGGATCTATATCAGGATATTTCTGCCTGGAGTCCGTTGAGTGAACAGCAGAAAATAATCCTGAAGGGCACGTTATCCGGACTGAAAGTGGAAGAGTTGGCTGAACAGATGA
ATATCCTTCCCCGCACTGTATTTGTTCATCGTGATGTTCTTATAAAAAAACTGGGGCTGCGTAATCGTCTTGAGTTGATGTGTCTTAATGTCAATAATTTTTCTGATATATACGACGAAAGGCAAT
ATATCACCACAATTGCTAATATATGA
>DLDGEG_04265 SH3 domain-containing protein
GTGCCATTAAGGTTTACAGCCACAGATTCGCATATCAGCGAATATCCTGTTCCTATTACTTTCACCCGTGGTACCAGCCTGATAATTCACGAAAAGTATGAGGGAGAGGAAGGCTGGGATAAC
TGGTTCTACTGCATAATTCCCGGTCATACTTGCGGCTGGGTGCCCGGACAGATCATCGAGAGATGTGAGGGGACGTATCGGGGCATTGCAAGGGAGGATTACAACGCCCGGGAACTGGAG
GTGAAGAGAGGGGATGAGGTTTACGGACTAAAATCACTCAATGGCTGGATGTGGCGTGAGCTTACTTCTGACTGGCAGACTGGATGGGTTCCTCTGTCGCTGCTGCGTAAGCATGGGAAG
AAAATCTGA
>DLDGEG_04270 VOC domain-containing protein
ATGTTTTCTTATATCATGCTTGGGATCAATGACTTGCCAGGCGCCATCAGATTCTACGACCCATTGATGGAATTACTGGGTTATCCAAAGGCAGGCCGTAACGAAGAAGGTGCTTCCTGGGGG
ACGTTTTGTGATAATTACACAACCGGACTGTGCGTCGGAAGGCCGTTTAATCAGTTGCCGGCGAGCGTGGGTAATGGAACGATGGTGGCTTTGAATGCCCGCTCGTTTGAACATATCCAGC
AATTGTATGCACTTGCGATCAGCCTTGGAGCCAGAGATGAAGGTTCTCCGGGTCACAGGCCGCAATATGGTCAAGGATTTTATAGCGCATATGTACGCGATCCGGATGGAAACAAGTTAGCG
TTTGTCTATTACGATAATGCTGGCTGA
>DLDGEG_04275 UPF0386 protein
ATGAATTTATCCCGTCAGGAACAGCGTACCTTACATGTGCTCGCAAAAGGTGGACGTATTGTGCACGTCCGCGATGCATCTGGCCGTATTACCTCTGTTGAATGCTATAGCCGCGAAGGGCTG
TTGCTTGCCGATTGTACGCTCGCCGTCTTCAAAAAAACTCAAAACCAAAAAACTGATCAAGTCCGTCAACGGTCAGCCTTACCGCATTAATACCACCGGGCTTAACAACGTACGTTCACAGCCC
GATAACCGTTAA
>DLDGEG_04280 3-demethylubiquinone-9 3-methyltransferase
ATGGATCTCAGCACGTTAATTTCTGTTACCGGCCGACTGCAGCTCTCTGCTGAAGAAAGCAAAATACCCTGGGACGAACCGGCGTTCAGCCAGCGCATGCTGGAAAATCATTTATCGCAGG
AGCATGACTGGGCCAGTCGCAGACAGACAGTGATTGAACAACAGGTAACATGGATAGCGAATCAGCTACCCGCGGGCGCACGCGTTCTGGATCTCGGCTGTGGCCCCGGCTTTTATACCCG
CCGGCTGGCAGAGCGTGGATTCAGGTGCACAGGTGTGGATTTTTCACCGGCCTCCATAGAATGGGCTCGACAGCAGGCGCAGGCCGCTGAACTGAACATTGATTATATCCACCAGGATATT
CGTACTTACTGGCCGGAAGCACCGTTCGATTTCATCATGATGACGTTCGGAGAGCTGAATGTCTTTAGCGTTGCTGATGCACGTGCGCTCATTACTCGTTGTGAGAAGTGGATGAAGCCGCA
CGGCAGGTTGCTCGCAGAAGTGCATACCTTCGATGAAGTTAAACGTCAGGGCATAGCACCGGCGAGCTGGCAGCGTTGTCCGCAGGGGTTGTTCCTTGCGGTACCTCATCTGTTGTTGACG
GAGAACGCCTGGGATGAAGAAACCCAAATCAGTTCGACATTGTTCTGGGCGATAGAAGAAAACGGTCGCGTTACCCGGTTCGGCAGCCAGATTACAGCCTGGCGTGATGAGGAGTATTTC
CGTCTGCTCGGTGATGCCGGATTCACTGTGGTACAAGGCCCGGACAGTCACTCATGGCCGGTAAGTGAGACATTTGAGGGCAGGTTGTTTACTTTGCTGGCTGAAAAATCAACAAATCAGA
TTTCCCCGGACATACAACCGAATGCAGGAGTTAAGTGA
>DLDGEG_04285 Uncharacterized protein YjhP
ATGGGTATTCCACGTATTTTTACCATCAGCGAAAGCGAACACAGGATTCATAATCCGTTTACCCCGGAAAAGTATGCCATGCTGGGACGTGTTTTACGCATGAAGCCTGGTACGAGTATTCTC
GATCTTGGCAGCTGCTCGGGAGAGATGCTTTGCACGTGGGCTCGTGATTACGGTATTACCGGTACGGGCATCGATATGAGTCCGCTCTTCACATCACAGGCAAAGATGCGCGCACAGGAGC
TGGGCGTCAGCGACCACGTGTATTTTATCCATAACGACGCAGCGGGTTACGTCGCAGACGAAAAATGCGATGTGGCGGCGTGTGTCGGCGCGACATGGATTGCAGGTGGCGTTGCCGGGA
CGCTCAGGTTGCTGGCGAAAAGCCTTAAGCCCGGTGGTGTGATGCTCATCGGCGAACCTTACTGGCGTCTGGTTCCGGCAACGGAAGAGATTGCACAGGCCTGTGGTGTTACTTCCGTTAC
AAATTTCCTTACCCTGCCAGCTCTGATTGCGTCATTTGGTGAAAAGGGATATGACGTGGTGGAAATGGTACTGGCAGACCAGGAAGGCTGGGACAGATATGAAGCCGCGAAATGGCTGAC

GATGCGCCGTTGGCTGGAAGAAAACCCTGACGATGACTTTGTACAGGAAATTCGTACGGAACTGAGTATTGCGCCCAAACGCCATGTCACATATACACGTGAATACTTAGGGTGGGGTGTCT
TTGCGTTAATGGCACGCTAG
>DLDGEG_04290 hypothetical protein
ATGCTGAAATTAATAAAAAATAATGAAAATGCTGTCGTGGTTTTACATGAAATATATGGAATTAATGAGCACATAAAAGGCGTATGTGCAGAATACCATGATAGAGGGTTTGATGTTTATTGCCC
CCCTCTGTTTGAATATGGGACCCCATTTAAGTATGAGCAACAGGATCAAGCTTACAAAAACTTTGTTAATACATGTGGTTTTGATGCCACAAAAATTAATTTGTTACTTGGTGAAATCAGGGG
GAGGTACAAAAAAATAATAATGATAGGATTTAGCGTGGGTGCCACTATTGCCTGGCTTTCAGCAGAAAGTAACCTGTGA
>DLDGEG_04295 hypothetical protein
GTGCCACTATTGCCTGGCTTTCAGCAGAAAGTAACCTGTGATGGAATTGTATGTTACTACGGCTCACGTATAAGAGATTACACTGATATTATGCCGCAGTGCCCTGTACTGGTTATTATGGCTCA
ATTCGAAAAGTCATTTGATCCTTCTCTTCTGCAAAAAAAACTGGCTGAAAGGACAACTGTTACGAGTCATGTACTCAATAGTCATCATGGTTTTAGTGACCCCTATAGTGAAACTTATAGCTGC
AGAAATACAGAGATTGCCAGAGACTTGGTTTATGATTTTATTAGCGCAATAGTTGATAAAACCTCTGAAATTTAG
>DLDGEG_04300 MFS domain-containing protein
ATGAATAAATGGATTAAGCTTTGCTTCCTGATACTGGGTGGAGGCACTATCTTTAAACTGTCCAGTATGAAGGACGTATTTTACGTCCCGATGCAGGCCGACTGGGGGCTGACCAATACTCAA
ATTGGGTTTGGTTTTACCGTTTACGCAATTATCCAGACGATTGGATTATTCAGTTCCTTATATATTGCCGACCGTTATTCAAAGAAAATACTCCTCCCTGTAGGTCTGATTGGCGTGGGGTTATG
CGGTGCTTACCTGACTACGCTTCCCCCCTTCACTGGCTATCTGGTTGCATTTGGGGCCATGGCTTTTTTTGGCGAAGTGGTTTACTGGCCAGTTCTGCTTAAAGCTGTACGTCTGCTTGGTAC
CAGAGAAGAACAAGGGCGCATGTTTGGATTTCTGGAGGCCGGGAGAGGTGTCGTGGATGTTGTAATAGCCTCCGGTGCGCTTTTTGTTTTCGTTCAGTTTGGTGAGGGAAAAGCGGGTAT
GCAGGCAGGACTTATCTATTACACTCTGGTGACTATTCTTGTGGGCATCATAACCTACTTTATTGTGGATGCCGATGACAAAGTCAGTTCAGACAATGCTGCCGATGCTAATAAACAGGTATTC
ATGGGAATTAAGAAAGTCATTAAATCACCTAATTTATGGCTGGCTTCATTTTGTATTTTCTTCGTTTACTCAGCATATTGCGGGCTCACCTACTTCATTCCATTCCTGAAAGATATCTATGCTTTGC
CTGTCGCTCTGGTTGGGGCGTATGGAATTATCAATCAGTACGGATTGAAAATGGTAGGCGGTCCGGTAGGCGGGTTCCTTGCTGATAAGATAGCCCATTCACCCGTTGTATACCTGAAATGGA
CATTTCTGATCTCAGCGATTGCAATGGTGCTTTTCATCCAGTTGCCTCATAACTCCATGAACGTCTATCTTGGTATGGCGGCAACACTGGGCTTTGGTGCAATTATCTTCTCACAACGTGCTATA
TTCTTTGCCCCTATGGATGAGATTGGAACACCAAGGGAGTTTGCAGGATCAGCGATGGCTTTCGGTTGTATTATTGGATATATGCCGTCTATGTTTGCCTATACACTTTATGGTTCACTACTGGA
TAATTTTGCTGGCATAAGGGGATATAACTACGTTTTCTCTGTAATGGTTGCCTTCAGTCTCCTTGGTTTTGTTTGTGCGACACTATTGACAAAAAGAATGAGAAGTAAAATTTAA
>DLDGEG_04305 YjgF-family lipoprotein
ATGATTATCTCCACACAGCATGCCCCTGCGGCTATCGGCCCTTATTCCCAGGCAATTAATACCGGAGGCCTTTTGTTTGTATCCGGCTGTTGCCCATTCAAACCTACAGATGGTTCTGTTAATGG
AATTAATATTGAAGAACAAACAAAACAGGCCATGGACAATCTCAAATCTATTATCGAGTCAGCAGGATTAGATATGGATTGTGTCGTCAAGACAACCTGTTTCATTAGTGATTTAAATGATTTT
CAGTTATTTAATTCTGTATATAGCAGTTATTTCTCAACAGGGAAATTCCCTGCTCGCTCATGTGTAGAAGTTGCAAGATTACCCAAAGATGTGCTGATAGAAGTTGAAGCCATTGCTGTTGTCA
AACAATAG
>DLDGEG_04310 AP-endonuc-2 domain-containing protein
ATGAAACTTGGCCTTGAAACTGAGTCGTATCACCTTTTCTTTCAACAGGGAGCTATGGATATCTTTGGCTTCATTGCTAAAACAAGCGAGCTGGGGTTGGACGGCGTCGAAATCAATATCATC
CCTGATGAAGGGTTACATCCTGAATTCGGTGTGCTGAACAGCGATGATCCTGCCTATCTGGCAAAAGTCAGACGCGCTATAGAAGAACGCGGGCTTTACTGTGAAATTGATACACGCTTCAC
AACACCTGACGCGATAGAAAAAGCAGTACGTATAGCATCTGCATTGAATGCAGATGTCATCAGAACCTATATGTTCCGCAGAGGGGAATATGACCCGGAGAAGTATCCTCAAATTATTGAACA
ACTCAGGTCACTCATACCATTGCTTAAAAAACATCGCATCCGGCTGGCCATTGAAAACCATGAAGACGAAACAGCAGATGAAATCATTGATGTTATCAGTGCCGTGGACTCCGTATGGATAGG
AGCTCATTGTGATATTGGCAATGGCATGATGGCATGGGAAGAGCCAGTACATACCGTAAAAAAACTGGCACCTTACGCGTACAGCACCCACTTCAAGGATCACATTGTAACCATGAGCGGGG
ATGAGTTTGTGGTTTGTGGCGTTCCTGTAGGAGAAGGCAGCATTGACATAGATACCTGCTTCAAAACATTAGTGGATGAATCCAGTGTAACCCACATTAATATTGAAACTTGCTTTCCTTATGC
CTCCCGCTTTGCCAGGCCAAAAGGAACGGGAGGAGTAAGAGATCTCAAAGACACTTTTTCTGTTAAGCCTGCGCCCTTTGATGACACAAAAATAAAACCTCTTGAGTATTACTACCCGGCA
AAGGTTTCAGAAGAACAGCTTCATGAGCTAATGGAAGCTCAGGAAAGATGCGTTCAGATTTCTGTTCAGACTCTTAAAAATCTTCGAAAAAAATATTGCTAA
>DLDGEG_04315 Hypothetical DNA-binding protein
ATGGAATCCATTATTAATCATAAGTTTACTGATGATGACCGGAACTTGTTGGCTACGTTTGTTCCCTTCATTAACTGCCTTGGCGCGTTTTTGGGTGAGTACTGTGAGATTGTTTTGCACAGCTT
CGAGGATTTACACCATTCAGTCATCCACATTGTGAATGGTCATGTGACGGGCAGAGAGCCGGGAGCCCCGGTAACAAATGTGGCACTGGAAAAACTTGCGGAGTTTAACTCAACCGATGAT
ATGTGGGATGTTTACTTCAGCAATAAGGGTATCCGGCCCTTTAAATCTTCCTCCACACTCATTGTGAACAAGAATAAATCTCCTGTTGGCATGATATGTATGAACTTTGCGCTTGATATGCCGCT
GAATGCTTTTGTTGATAATTTTGCTAAATCAAATAATTGTAAAAACGAAAGCTTTACTCAGGATATTAACAATCTGGTTCAGAGCCATCTTGAACCGGTTAAAAATATGATTTATGCCAATAAGA
GTATCCCAAGTAAGAGTAAAAATTTTGAAATTATCAAGGAGTTGAACTCTATAGGGTTGTTTGATTTTCCGGTGACTAATAAAATTGTATCATCTAGCCTGGGAATCAGCCCAAATACCGTTTAT
AAACACTTGCGGGCATTGAATGGTAAAATTTAA
>DLDGEG_04320 hypothetical protein
ATGCTTTTATCCGGAGCCCGACTATGTCCCACACTGATACTCCTGAACAGGCTGCTGTCATCGCATGGAAGGGCGAACGTCTGGTTGTCTGTGCTTTTGCCGGGAGTGGCAAAACCACCACA
CTTCGTCGTTATGCTGAGGAAAATCCAACGGAACGAATGCTCTATATTGCTTATAACCGTGCCATCAGAAGATGAGGCGGAGCAGAAATTCCCCTTCAACGTTACCTGCAAAACATCGCATCA
ACTGGCCTGGCCGACGGTCGGGAAACATTACAGTCATCGTCTGATTAATTCACTACGACTGACCGATGTCGCCCGGGCGCTAAACTCCCGCAACTGGTTGCTGGCCAGACTGACGCTTAATG
TTCTGAATCGCTTTATGTACAGCGCGGCCACCGATATTACGCCTGAACACATGCCTGATCCTGACGACTGTAAGGGGCTACAACCAGGTCAAATCCTGCTTTCAGCAAAGAAAATCTGGGCA
ATAATGTCGGCCCGTCAGGGGGATTTTCCGGTCACCCATGATACTTATCTAAAGCTTTACCAGCTGAGCAAACCGAATCTTTCTTCCCGCTATACCACGTTATTGTTTGATGAAGCACAGGATG
CCAATCCTGTCATCAGCGCCATTGTGCTCAACCAGACCTGCCGGGTAGTTCTGGTTGGAGATACTCATCAGCAAATCTACCGCTTTCGTGGGGCGGATAATGCCATGCAGGCTCCGCAACTG
GAGAACTCAGACCGTCTTTGGCTAACCCACAGCTTCCGCTTCGGTCCGGAGGTAGCCGGGGTGGCCAACAGGCTGCTGGCCCTGAAGGGCGAAACGCATCAGATAACTGGAAAGGGGG
GCGTAGACCGTGTTCTGCTCACAATGCCGCGGAATTTTGGCCATCATGCTGTTCTTCACCGATCTGTCTGTGGTGTCATCCGGACGGCACTGCACTGGTCGCTGGCTGGCAGGAAAGTATTC
TGGGTTGGTGGTATGGAAAGCTACAGAATTGAGGATTTACTGGATTTGTACTGGTTCTCAATTGATATGTCGGAGCGTATGCGGCACGACAGGCTGACCCGTGAATACCGGGACTATGATGA
ATATCTGCAGATAGCCGAAGATACCGGTGATGTTGAGATGAAGCAGGCGATATTTATTCTTGAACAGTTTTTTCCGTTACCGGACAGACTGAATACGTTACGTGAGCAACGCGTCATCAGTGA
ATCTGAAGCTGATGTGACGGTATGTACAGCACACAGGAGTAAGGGGCTCGAATGGGACAGGGTGAAGTTACATGATGACTTTCCCGATATTCTGGATCCTGACATGCCTGCAAGTCAGCGG
CAGGATGAAATTAACCTCATGTATGTGGCAGCCACACGGGCGCGTAAAGTGCTTATAACCGATCCGATATTGCACGAGCTTCTGATCCAGCCCCGGGCTGAAGATATAGCCGGAACTTCTGC
GGATAAATCGGGTGAGTCAGAAGTGAGTACAGAAAACGATAAAAAAGGGACTGAATATGTTCCGGAAAGTTAA
>DLDGEG_04325 Relaxase
ATGTTCCGGAAAGTTAAGTCATGGATGGAGAAGAAAAACCTCAGACAGATAGAAGAAAATAAATCGGGAAATGCGAAGAAGAGACTTATTCCCTGTACGGATGGATATTTTAACCCTGCGG
CCATTTCAGATTTGTTACAGCATGAACGACGGCAGCAATGGTTAAGGATTTTGTGGGAGAACAGCGCGCTACCTAAAGAGCGTTTTGAACGTTATTTTTACTCCCTTTACACGGCGTTGTG
GGTTTATGTCAGAGACTCCCGGCTTCGGCTCAGGGAAAATTTGCCTATACAGACGGCATGGTGGATTACGTCATACAGACGACGGTCTTTGCCGTACGTTTATCAAAAGGATATATGCTGCCC
CGAGGCGCATCTGCAGAGGAACAATCCGCGCAGTCAGTCAGCTGGAGTGCTGTCGTTTATTACGCTGCATTATTCCACTCGTTGGGGCGCTTATGGAATATTGAAGGCGAGCTCTGTAGTGG
TGCTGTATGGCGCCCGGGATTAAGTGTTCCGGAAGAACCTTATCGTTTTCGTTTTAAAGCAGAACCCGATATAGCAGGTGCTCAGGTTTATGGTGAATTGATCGCTCTCAGATTTTTGCCAGA
ACCGGTTCTTCAGTGGCTGGGTAAAAACCCTGATATTCTGAGAACATTGCTGGCTTTCATAAGCGGACGTTACAGTGATGCAATGGATATTACGGATATCGTTAATGAGGCCATTGTGCATGC
CGGAGGGATACCTCTTGGTTTTCCCATTGCACAGGAAGAGCAGACTTCTGAATTAGCAGAGATACTTAACCCTGCCGCGACAGTAGCTCCCGTGTTGAGGAAAGGTTCTTCTACAACTCCTC
CTGATGTCCCTGTCATGGTTGATGGAGGGCAGGAGACTACTGTCCTTTTATCTTCCCTTGATTCTGATGCTAAGCCTGAACGTGGGGAAGGGCAGCCTACTGCTGAAACAGATCCAGATGTT
TTTCAGGTCATGTCGATTCTGGGAGGGCTGACGAACACTGACACACAGTCGCCTGAAAAGGAAGGCGTGAACACAGAGACCGATATGGTGGCAGGAGAATCGTTTCCGGAAGTCGCGGT
TGCACTTGAGACTTCAGCAGCAGAAGAGGTTATCAATGACACCGTTACCACTGAATGCACTGGATCATCCTCGGGTGGCGTATCATCGCTTTCCTCAACGGAGCTGGGAGATTTGTTCTGGT
CCTGGCTAAGGGATGGGCTTCGTGAGGGGGATATACCGGTAAATACTGCTGATGCCTGTGTTCATCTGACTTGTGGCTTCATATTTATCAGCGTCCCGGGTGTTTTTTTTCTGTTTTTGAAATC
ACATTTGCGTTCCTGTTCTTCTGGTCTGAAAGAAAGCGGGAGGAAGGAGCAGGTGCAGGCGGCATTTGAGAAGATGCGAAAACATCGGGTCAGTGATTCCCGGCGATTCTGGCAGTGCT

GTCTCTATGAAGAGCCAGGGGGCAGGGGGAGATACAAAAAGTTAACAGGGTATCTGATAAAAATGTCAGAGATTTATGCTAATGGTAACTTTCCCGACGACAGTTTATTTCTAAAGGTCATC
AATTGA
>DLDGEG_04330 Integrase
ATGAAATTACGGGATTTTAAACCCACACAAGTTGTGTGTATTAATAATAAAAGCTTAATAGCGGTGATAAAAATGGAAACAAATATTACCTGGCAACAATTGATAGATGAATACTTCTTCGCAA
AACCTCTGCGCTCAGCATCTGAATGGAGTTACACCAAAGTCTTCAAATCATTTGTACATTATATGGGGCCGTTAAGCTGCCCTAATGATGTGACATATCACAAAGTGCTTGCCTGGCGCCGTTT
TCTTTTAAAAGAGAAAAAACTGTCCGGACGTACCTGGAATAACAAGGTGGCGCATATGCGGGCCATCTTTAACTACGGTATACAGCGTGGGTTACTGCACTATGACGAAAATCCGTTTAACA
ATTCGGTAGTTAAACCGGATAAAAAGAGAAAGAAAACGCTCACTCAGGCACAGATTGAATATGCCTATCAGATCATGGAGCAGTATGAAAATCAGGAGAATACAGGGCTGGGACTGAAATA
TTCCCGCTGCGCCTTATTTCCTGCCTGGTTCTGGCTCACTGTCCTGGATACGCTCTATTACACAGGGATACGTCAGAACCAGTTATTACATATTCGGCTGAATGATGTTGATTTGAGAGAGGGG
CAGATTCGGCTGATTACTGAGGGGTGTAAAAATCACAAAGAACACTATGTGCCGGTGATCAGTTTTCTGCGTCCCCGGCTGACCTGTTTAATGGAGAAAGCGCAGAGCGAAGGATTGAAA
GGCAATGACCGCCTGTTCAATATTGCACTTTTTACCGGCAAAGATCCCGCCATTGGCGATGACATGGATTCTCCTCAGGTAAGAGCATTCTTCCGTCGTCTGTCCAAGGAGTGTCAGTTTGCG
ATCAGTCCTCATCGTTTCAGACACACGCTGGCAACGGAGATGATGAAAATGCCGGAACAGAATCTGCATATGGCGCAAAGTGTGCTGGGTCATTCAAACATGAAATCCACGCTGGAGTATGT
GGAGAATGATATTGCAGTGATGGGGAGGGCTCTGGAAGCACAGTTTATGCAGATTAAGGCAGCACATGCCCGAAGCATTTACAGTGGGTTGACAAAGAATAGATAA

ICE 2 (MOBH, MPF (type G, type I, type T))

>KGDGIN_03765 tRNA-Ser(cga)
GCCCGTAACTATCTGAATTTTATGACATGGGGATTGAAAATCCCCGTGTCCTTGGTTCGATTCCGAGTCCGGGCACCA
>KGDGIN_03770 Tyrosine-protein kinase CpsD
ATGAAAATACTTCCCGTTGTCTCAACAAAAGGTGGCGAAGGTAAATCAACACAATCCGCTAACCTGGCTGGTTTTCTCGCCGATGCAGGTATCCGTACCCTGCTGATTGACGGAGATCATGCT
CAGCCCACGGCAAGCAACATCTTTCCGCTGACATATGAAGCGTCAGCCGGTCTTTTTGAGTTGCTGATGCGCACGGCTGACCTCAGTTATCCCGATAACATCATTTCCCGCACAGCTATCGAC
GGACTCGATCTGATAGTCTCCAATGACCCTCATGAGCAGTTAAAAACTGCGATGCTTCACGCCCCGGATGGTCGTCTGCGCCTCCGGAATGTCCTGCAGCATCCGCTTTTTCTGAACAACTAC
GATGTCATTATAGTTGATTCTCAGGGTGCGCGTTCTGTCATGCTCGAAATGATTGTGCTGTCGGCCACTGATTCGGTTGTCGGGATGGTCAATCCGGTACTCCCTGATGTGCGCGAGTTTATTC
GCGGCACTGTCAACGTTATGGAGAACCTGCTGCCTTACCGGGAACTGGGGATACCCCTGCCGAAAGTACGCACGCTGGTTAACTGCATGGATTATACGGCACTGGCCAGGAAGACGCTGG
ATGAGCTGGCCGGAATTATTCAGACCGGTCGTTACAGCAGGCAGTTACCTGAAGGTGCGGTTTCCCTTCTTTCCACACAGATTTACGACCTGAATATTTACAAGCAGGGGCATGCGGCTGGT
CAACCCGTACATCGTCTCGAAAAAGAATCCACACGTCGCAGTGATTCCGCGCTTGTCACGATGCACAGCCTGGCCTGTGAACTATTTCCTGAGTGGAAACAGGCGTTTGACGGGCTACTGG
CTAACGGGGGAATACGTCAGTGA
>KGDGIN_03775 Mediator of RNA polymerase II transcription subunit 28
ATGAATAACAGCAGCAATACCACAACCCGTAATACGCTTTCACCCGTTGAGTCAGCCTGTCATGCCTTACTGGATTTTATTGCCCGGGAAGTTTTTGAGAATAAAGCACCGGAAGAGGACGT
CAGGGAACGTTGCAAAGTATTTACAGCGCTGCTTAACGAAGGATTTAAAGCCCTGCTTGATGAGGCAGGGCTGAATCTGTCAAACCTTCGTAAACAGTGTGAGGAACTGGAATCTCAGATA
ACCAGACTTGATTCTCAGTCCGATGCTATTTGTCGCAGAGGTGCTCAAATTGAACGCTTCATTGGTCACCATTTACCTTTTTTCGATTTGAACAAAGTTTTTGATACTGCCAGTCAGTCATGTG
AACCAGCGCCTTCTCCGGCTCAGTCGGCTGTGTCCAGTAGTCAGCCCAGTAATCTGTCAGAGCAAGGGACATCTCAGCGGGTGTCATCTGTTCAACCGTTGCAGGGAGCCAGTGATGTTGA
TGAATAA
>KGDGIN_03780 DUF1799 domain-containing protein
ATGAACACAAATCATCAGCAACTGCCGTCCGGCATTGATAACAACCATGATGAGGATGGCAACATGGCAAAATATTATGAAAACTGGAAAGTCGGAGGTATGAGCACCGACGATGTGGAAC
GGTGCAAAACCTTCTTTGACCAGGGAACAGAAATCTTTCCCGGAGCTGACCGGAAGCTCGCTTACCGACTTTGCTGGCTCGGACACTGGCTGATTAACAGCCGCCGCTGGTTTTTTCTGGA
CGATAACAGTATCAGCGTTCAACAAGCCGTTAAGTTTGCTCTTATTCATCAACATCACTGGCTCCCTGCAACGGTTGAACAGATGACACCCGCTGAGATGTCCCTTGCTCTGACAGATTACTG
GGCTGACTACTGGACACAGCCGACTGAGCCGGAGAAGGCGCTGGTTCACATGACTGACTGGCAGTATCAAAAACTTTGTTCAAATCGAAAAAAGGTAAATGGTGACCAATGA
>KGDGIN_03785 Replicative DNA helicase
ATGTCTGAACGCTATATTCCACGCTCCGTTGATGCAGAGCAGGCCGTGCTGGGCGGCCTGATGCTCGATAATGAACGCTGGGATGAGGTGGTACTGATGCTCCGGGGGGAGGATTTTTCTC
TGGCCGCACACCGGGTGATATTCCGCACCATGTCAGAGCTGGCAACCAGCGGTCAGCCGTTCGACCTCATCACGCTGAGTGAGCATATTGAGAAAACAAACAATATTGAGGCACTGGGTGG
TTTCGCTTATCTGGCTGAACTCAGCAAAAATACGCCTTCTGCAGCCAATATTATCGCTTATGCCCGTATTGTTGCTGAAAAGAGCATGCTGCGTCAGTTGCAGGAGACGGGGAATGCGCTGAT
TACGGATGTGGCCGCACCGGATGCGACACCACGCACTGTTCTTGAATCCGCTGAACGTCGGCTCTTTTCACTTTCACAGACTGGCATGTTGCATGAGCGATGTGAAGTCAGTCTCACCTCGG
CTATGGAGCTGGCGCTGCGTCAACTGGAAGAGGGACTGAACGCCGGCGGGATGACGGGCACGCCCACGGGATTTGATGAACTGGATGAGATGACCTGTGGTCTGCAGCCCGGCGACCTC
ATTCTTCTGGCTGCACGCCCGTCTATGGGGAAAACAGCGCTGGGCCTTACCTTCTGTCTTAATGCCCTGTGTAACCGGACCGATGAAACGGTATTTCTTTTCAGTATCGAAATGTCCCGGGAG
CAGCTGATTCTGCGTGTCCTGTCAATGCTCTCCAGGGTGGAGCTGACACGTCTTCGCAGCAGCATGATGGATGATGAAGAGTGGGCGAGAGTCACGGCAACCATGGGACGCTTCATGAAT
GAAGCGTCATTCATCGGAGATGGAAACCGCCTGATAATTGATGACACCTCCTTCCAGACGCCGTCATCGCTGCGTGCCAGCGCCCGTCGTTATGTCCGTCTGTATGGCAGACCTTCCCTGATA
ATGGTTGATTACCTCCAGCTGATTCGTTCTCCGGACCAGGAGAACCGAACGCAGGAAATTGCGGAAATATCCCGCGCGCTTAAGGCGTTAGGTAAAGAGCTGGGATGCCCGGTACTGGCAC
TGTCTCAGCTTAACCGTCAGGTGGAGCAGCGGGCGGATAAACGCCCCAATAACGGTGACCTGCGTGATTCCGGGGCGCTGGAGCAGGATGCTGACCTCATTATGTTTATTTACCGTGACGA
GGTTTATAACTCCGGCACGCTGGATAAGGGCGTTGCTGAAATCATTGTGAGCAAACAGCGGCAGGGACCAACCGGAACGATACGGGTGAACTTCGACGGCCGCTACACCCTGTTTTCACC
GTTTGAACGTGGATGGAATTTTGACCGTGCCGGAGGAAATGCGTATGTCAATTAA
>KGDGIN_03790 Chromosome partitioning protein ParB
ATGTCAATTAAACGAAATCATTCCGTTGATCTGGGGGCTGCCATCATGCAACCGGGCCGTCAGGCCAGCACGGCAGGTAACGTGGTGGCGCTGGGGGAAACACCGATGATTCTGACGCTG
GATCAACTGCGCCCCAACCCGGATAACCCGAGGACCACGCGTAATCCACGGTACGACGATATTAAAAGTTCAATTTTCTCCCGTGGACTGGATACGGTGCCCAAAGTCACCCGGATACCTGA
GAGTGAGCCGGATATCTATATTTTCAGCGATGGCGGTAACACGCGATATCAGATCCTGAAAGAGCTGTGGGAAGAGACCGGTGATCAGCGTTTCTACCGTATTCATGTGCTTTTCAAGCCATG
GCCGGGACGGCTTCAGTGCGTCATTGGTCACCTGGCGGAGAATGAAGTCAGGGGCGAACTGACGTTTATTGAAAAGGCCCGGGGTATCCATAAAGCACGGATGATTTATGAAGAGCAGTT
AAGCAAAAGTGTGTCCATGCGAGAGTTGTCATCCCTTTTGACTGAGCAGGGATTACCGGTGGATGCATCCAGTATCAGCCGTATGGAGAACACCCTGAAATACCTGTATCCGTGGATCCCCG
AACTTCTGGAATCAGGAATGGGGAGTCCTCAGGTCAGGGCTTTACTGTCTCTCCGGCAGGATGCGGAACGTATCTGGGGACAGTTTGCGGTGGCAACAGAGAACGGGGGGGATTTTGAC
CGCGTGTTTGGTGAATGCTGCAGCAAATTCAATTCACCGGAGCTCTGGTCACTGGAGATGTTCCGGGATGAGCTGATTGGTGATCTGCTGAAAGCGTTACCACATCCGGTGCTGGATTATGA
CCGCTGGCTGCTTGAACTGGATCCGCGGGAACGTAACCGACGTCATCATTTTGGTGAACCTGAACCGGTGCTCATTCCCGCAGACAGTGATGCGATGCCAGAGCCTGTGACTCACGGTACG
TCGGCAGTGACGGTGGAGAAGGACAGCGGAAGGGAGCAGTCTGTGATTTCCGCACCGGCTGACCATGACACAGTCATGAATACGGAGCAATCCCCTGTTGGAAAGGCGGAGAGTTCAG
TTACTGAACAACGCGAAGAACGCCGCAGAAAGGAAGTCCAGCCGGATATGTATGGTGGTGAAGAAACATACAGCGGCGATTACGAAGAGGATGGCGATACCCGTGATGCTGCGGATCTGC
CGGTAACGTCACCGGAAGAGGGTGCTCAGCAGAGCAGCGGTGAAATTTTCAGTGAGCCGGAATTCAGTGTTGGTGAACTGGATGATATCGAATATCTGCAGAATGAGGCATTTCACCTGG
CATGGGAGCTGGCGTCACACACAGGCTGTGTCGATGAACTGGCTATGGACAGGGAAAGCGAACTGTCTGCGGGATTCGGCGCGGCAGAGGCGAATTGTTCTCCGGTCATGGCATTTCTTG
TCAGGCTGACCGGTGAGGAGGTTCTGCGGGTTAATCCGGTTTCTCTTGATGCACTGATGATTGGTGGCAGTACTGAAGAAGAGCCACCACTGCTGGATGATGAGCATGCCGGGAAGTTACT
CCGTCTGCTTGGGGTGTTGCGTCGTCTGCGGGGGCTGCAGCGCCAGGTGGAAGAAGAGAACGTGGAGGGGCGTGATGAATAA
>KGDGIN_03795 DUF2786 domain-containing protein
ATGAATAATGATGCAGCACGTCAGGAGCAACTGATTAAACGGATAAGAAAACTGCTGGCGCTGAGCCGGAATAACAGTAATCCCCATGAGGCAGGTCAGGCTCTTTCCCGCGCACAGGCG
CTGATGCGGAAGCATGGTATCACCGAACTGGATGCTGATATTTCGGTGATAAAAACCTCATCATCAAAGGGAGCACCGTCAGAAGCGGAAAAAGTTCCGGTATGGATGGGGCGTCTTATGA

ATGTTGTTGCAGGGGCATTCGGATGCCATACATACCTGGATTGGCGTATCACAACCGGAGGATACTGGCGGCGGATTGTCACATTTTATGGTTTTGGGGAGCGTCCGGTTGTGGCTGCGTAT
GCCTTTGATGTTCTCTGCCGGCAGATGCAAAAGGCGACAAAAGAGTATCTCTCAACCCAGAGCCGGAAATTAAAACACTCAACCCGTCGTGCAAGAGCTGCGATGTTTCGTGACGGCTGG
GTTTATGGGGTGCGTGATGTGGTGGATGCATTCCGGCTCACGGAAGATGAGACGCGTGTAATGAATACGTGGCTGCAGAACCAGCAATTTGAGGGAATGGTCGTCTTCCGGGAGGTAAAG
GACTGCCATGGCGGTGACCATGCACGCTGGCAGGGATACAACGAGGGGAAAAAGGCAACGCTGAATCACGGTGTTGGTTCCCGGGATCTGAGACCGGAAAGCCTGATATCAGGCGGAG
GTGAGGTATGA
>KGDGIN_03800 DUF2857 domain-containing protein
ATGAGTAATAACTTGTCACAGGCCACCAACGGACTGTTGATGCAGCTGGTGATGGATCTCAAAAGTGGCTATCTGCGCCGCTGTGAAGCGCTGGGGCTTTCCCGGGAAGAAATGCAGATGC
TGCAGGGGCTGAGTATTGAGGAAATTCACTATCTGTCAAATTCAGAGGTGTCAGTGCTGCGGCTGGATATTAACCACAATAACCTTGTACGCATGCTGCAGCAGGCCAGGACTGAACAGAA
ACGCCTGCAGCGAATCGATCGCGCTCTTGCACTGGGGGGATCGATTGAACTCATGGCGTTTTATTTTGGCCTTTCCAGTGTGGACGTTGCAGCACGGCGCCGTATTTCAGGAATTGATGTGC
GCCCGGGGCGGGGTATCACACTCAGCGATGATGAAAACTCTGAACTCTGGAGACTGTGGCAGAAAGCAGGGATTAACGATGTGGAGAGCGCTGACGGGCTGGACGTCATGATGCTCTGT
GCAGAGCAGATGAATATCCCGCTCACGGCCGTCTGGCATGCTGTCAGGGGGTGGCATACAGCCGGAAGCCCTGAACAGGTAAGGAATGCGTCATGA
>KGDGIN_03805 Transposase
ATGAAGATGAAAGAGCTGGTTCCCGGTCTGCTGAGCTGTCCCGGGGTACCGCCAGCCTGCCAGCGGAAGGCGGTGGACCTCGTTAACCGGTTTCGTCGCGGGGAGAGGAATTACCATGA
ACTGAAGGAAAAGGGATTCGGGTATATCAAGATTAATGTTGGTCCGGACTGGCGGTTACTGAGCAGGAACCGGGGACAAACCTGGGCGCTGTTGAGTCACGAGCGATACAACAATATGAG
ACTGAGGTAG
>KGDGIN_03810 hypothetical protein
ATGACTGATTTTGGTTCAACTAAATATAACGCCAGTTTTGAAGAATGGCATGAAGTGTTAATGGACTATGCCGGGTTACGTGGTGGAAGTGCCGCCGATGCCGAAGCATGGCGCGATGACTA
TGAAGCTGGAAAAACGCCTGTCGAAGCATATTGCGATGAGTGGGGTGATGAATGA
>KGDGIN_03815 hypothetical protein
ATGAGCAAAATTGACTATCAGGAGCTGCGTGAGGCGGCAGAAAAGGCAATGCATGACGACTGGGGATTTGACACGGACCTTTTCTATGAGCTGGTAGAACCATCGATTGTGCTGGCTCTGC
TGGATGAATGCGAGACGCTGGCAACTGAGAATGCGAAGCTGAAGACAGCACACCCTCAACTGTTCGGGAAAGCTGAAGGAGCGACAAAATGA
>KGDGIN_03820 hypothetical protein
ATGAGCAAGCCAACCGACGAAGAAATAGTTCGGGTGTTGGAAGAACACGGGCGCTGTATGACTTACGTCGTGACCAACTGGCTTAGGGATAAATATCGCACACTCAAAACGGCATACGTTT
TGCGTCGTCTAAAGAAACTGGAGTTCGACGGGAAAGTAAAGCGCGTTAACAGTTCTTATATAAGACAAATTTGCTGGGAGGCAACCAGTGAGCGAGATTGA
>KGDGIN_03825 Ead/Ea22-like family protein
GTGAGCGAGATTGATTATCAGGCGCTACGGGAACGTTATTCACCTAAGCCAGTACCTGAATGCTCTGTTTGTGGCGAGGAAATGTCAATACAGCGCATATCTGGAGCACATGTTGTTTATGCC
TGTTCCGGTGAGGGTGATGACGGATATTTTAAGATAGGGCGTACTTTTGCAGACGAACATTATGAAGAATCACGCGTAACAGTAGTTGATGATAGTGATCCCGATGTGATTGCACTACTGGAC
GAACTGGAGGCAGCAAAATCAAAACTCAACGAGCAGCGTGAATATTACGAGGGAGTAATCGCGGATGGGAGTAAGCGTATTGCAGAACTGGAAGCACGGGAAATAAAACCAGCCAAAG
GCGAAGTTCTTGTCGTTGTATCTGGTTTTACTGGTTGCGGAAAAAGCGCCATTGCCGGGGAAATAGAAATCGCGATGAAGGCTATTGGTGTGCCGGTTCTATGGACTAATGGCGATGCGGA
AAAGCGCATGACAGGAGCTGACTGGCTGACAGCGATTGAGATGTACAAACCAAATGTGCGCATCGTGGAAGTTAATGTGCCACGCGTCGCTGGCATTCGCACCAAAGGAGAGTGA
>KGDGIN_03830 MmcB family DNA repair protein
ATGTGCCACGCGTCGCTGGCATTCGCACCAAAGGAGAGTGATATGGCGTTAACACACCGCGAACTCTGTCAGATTGCGTACAAGTTCCTTAAGCGCAACGGGTTCAAGGTTTGTTTTCATGA
CCGCTTTATAGCTGTAACCAGTACCGGAGAACAGCCAGATGCTATGGGATTCAGAAATTCAGCATCATGCCTGATAGAGGCGAAATGTTCTCGTGCTGACTTGTTGGCAGATAGAAAAAAGC
GTTTTCGTAAAAATCCATCTCTTGGAATGGGCGACTGGCGATTCTTTATTAGTGAGCCGGGAATTATTTCAATTGAGGATTTACCACCTGGCTGGGGATTACTTCACGTTGTTAACGGAAGAG
TACGGAAAGTACATGGGTGGCCCAGGGGTAATTGCTGTTGGGGTAATCCTGACGATAAGCCATTTACTGGAAATAAGCAGGTTGAATGCGATTACATGTTATCTGCATTAAGGCGCATGGAG
TTGAGAGGGCACCTTAATGAAATATATGACGGTGTAATTGTTAATAAGAAAGAAGGAAACGCAGCATGA
>KGDGIN_03835 DUF551 domain-containing protein
ATGATCACTATTACCAAAGAGCGACTGCTGACAATCCAGCAGTGGCGCGAAACATACGGACCTGGTAGCAACGTTGTATTGCCAGCAGAAGAAGCGGAAGAACTGGCACGAATTGCACTG
GCATTGCTGGAAGCAGAACCGCTGGTGCCCGTCATGTATAAAGGAATGAAACTGTTAACAAAAGAGGGGCTTGAGCTTATTCGTGACGGTATTGCTGAGGCTACAGGACTGGAAGCAATG
TGTATGGCTGAGGCTTTGCTGTCTGGGGCGTCAGTGCCGTCAGTGCCAGAAGAAATGTATTGGCAGGATGCGCCTGTTGGAGGCAGTAGCAAAGCAGCTGCATACGCGACAGGCTGGAAT
GCCTGTCGCGCCGCAATGCTTCAGGGAAAAGGAAAGTGA
>KGDGIN_03840 hypothetical protein
ATGGCTAACTCGTTACTTGAAACCTGCAACAACTGGCAGATTCAGAGGGCGGAGATTTTATCTCGCAATCCAGATATGGCAATGACAATTGATAATCTGGACACGCTAATTGAACGGACCGT
GCGTTCTGCAATTGATATAGCACATCGAGTGGATTGGGATTTCAGAGAAGCGGAGCGGCTTGCTAAAGAGCAGGAGAAAGCAGCGGGTAAAGGAGACTGA
>KGDGIN_03845 hypothetical protein
ATGGATAAAAACACCACTGCTTACTGGAATCTGTCACTTGATACCGAATGCCCAAAATGCGGTCACAATTTCGATCTGCTTTGTGATCCAGATTTCTGGGAATTTTCTGGAGCAAAACAGGCA
TGTGAAGAAATAAAAGGTTACGAAACATGCTGTCCAGAATGTAACCATGAATTTAAAACAGATTTTGTGTATTGA
>KGDGIN_03850 DUF551 domain-containing protein
ATGACCACTATAACCAAAGAGCGACTGCTGACAATCAAGCAGTGGCGCGAAACATACGGACCGGGTAGCAACGTTGTACTGCCAGCAGAAGAAGCGGAAGAACTGGCACGGATTGTACT
GGCATCGCTGGAAGCGGAACCGGTGGCGTGGATGTGTGATGACGAAGATGGTCGTGAATATAACGGTCATAACGAGTTTTCTTATGGAAGACGCGGGATTCCCCTTTACGCCGTCCCGCCA
GCGCCGAATGGATTGGTTAAAGCCGTAGCTTTCTATGAGCAGGTTAAACGTGAAAATCCGCCAGTTGAAACGGGAGCATGGAAAGACGCCGTTGACTGGGTGCTTAAACAAGCTTGTCAG
GCTGTAAACACTGGCATCAATGAAAGAAGAACAATTGAAGAAAATGATGATTGA
>KGDGIN_03855 Prophage protein
ATGAGAAAAATCAGTGGAATGTTGAACCTGGAGAAGGCCAGGATGCAGTTGCGTGTTTTGTTATCAGGACCGGTTCCTGCGATCACTCCGATGTCGCATGCCGTGGCAATTAAAGTTGTGC
CTGATGCTCCGGATGCGAGTGAGCGGAGGGTGGCTGAACTGGAACAGGCCTGCGCTGCCGCGGGCATTCGTATCAAAGGGGAACAAGTGAAATGA
>KGDGIN_03860 hypothetical protein
ATGAAACTGAAGATGCAGGCAATGGACGGCCCGGTGATTATAGAGAGTAGTGATGTGACACAGTTTTACCCTGACCATGAAAGTGGCGGGGAACTCACCGCTGTTGAGTACCTTGCTGAC
GGGGGAAGAATCACCGCGAGGGTGAGGCACTCTTTTTATCAGGTTGCTGCGGCACTTGCCGGGGCATGGCGTGCGGATGATGCATCAAAAAGCGGTGGGTGA
>KGDGIN_03865 Helix-turn-helix domain-containing protein
ATGGCGCTACCTGCAGAAAGCCTTATTGCGCATACGCTCAGAAAAATGAAGGCCAGCATGGCAGAGAAAGCCAGTCGTGAGGACGGGAGGATACGCAGTGGGCTGTTGTTTACCGGCAA
TGTTCACGATTCGTATCCCCGCAGGCTGTTGCTGGATACGCGCCTTTCTCCCCTTGATAAAACTGCGTGGATGATGATTCGTCTGCATGCCCTGAATAATGAGGGCGCAGTATTTCCGACGTAC
GATGAACTGCAGTTACAACTGGCATCTCCCGGGTGCGGGAAGGCGTCCCGTGAAACAGTGAGCCGGGTGCTGACAATGCTGCGGCTTACGGGCTGGCTGAGTCTGTGTAAGCGGGTCAG
GGATGAAAAAGGGCGGGTCCGGGGGAATATTTACGCCCTGCATGATGAGCCACTGACCTGCTGTGATGCAGAAAAGTTTGACCCACGATACCTGGATATGGTTCTGGATACTGTGAGCAGG
AGCCGGAACAAGACAATCCGGCACACAGCCAGTGAAGTGCTCAGTGATATCAAAAATGAGCCCGGTATGCGGCATTACCACAGCCATATCACTCTGTTTGAATCGCGTATGGGGGCAGCAC
AGACACCTGGTCAGCTGGCGGCGCGTCAGCGGGTGAACCCGATGGTGAATGAAAACACAGGCAGAGAGAGTGGTTCGGATTCCGTACATACCCCTGCACCTGTCGTGCAGGTAACCGAA
CTCAGTGCTCCGTTAACCGAACTCAGTGAAAAACAGGAAATGAGCCCACTCAGTTCGGATTCCGTACCGCCTGAAAAATCAGTAACTTATCACTCGGTTCGGAAACCGAACAATTACGTACG
TAATATCACACACAGTGTGATTAAAAATACGTACGTATTGCCTGAGTCGGTGAAATCGCAAATCAGCACAGAAGACACGGACATGCTGACAAAACAACTGCAGGCGTTGCCGGAAGAACTG
GCAGGTAGTGTGCTGAGCAGCCTGTCACAGATGCTCAGCAAAAACACGCTCCAGAATCCGGTTGGCTGGATGCTGGCTGTGATGAAACGTGCGCGTGAGGGAAGTTTTAATACGCCGGAT
ATGTCCGTGCATGAATCCCGGTCGCCAGCCCGTGCACCATATCAGCCTGTGTACAACCGTGATGAACGTGAACGTCGTCGCAGGAAAGTGGCGTCACCTGAAACGGTGAGCAACATTGTCG
CAGAATTCCGTGCCAGCCTGAAACGTGCAAATGCTGCCCGTCTGGCGGCGCGTGTTTGA

>KGDGIN_03870 hypothetical protein
TTGCGTCATGTTGGCAGTGCCAACTCAGTACAATTTTCAAACACTGTTGCCACTGGCAACGCCGGACACTTTTTAACCACTGTTGCCACTGGCAACACCGTACATTTTTTAATCGCTGTTGCT
ACTGCCAACGCCGTACACTTTTTAACCGCTGTTGGCACTGCCAACACCGTACATTTTTTAATCACTGTTGGCACTGCCAACACCGCACAATTTTTAACCACTGTTGGCACTGCCAACACCGCG
CAATTTTTAACCACTGTTGGCACTGCCAACACCGTACATTTTTTAATCACCGTTGGCACTGCCAACACCGTACATTTTTTAATCACTGTTGGCACTGCCAACACCGTACATTTTTCAATCACTGT
TGGCACTGCCAACGCCGTACACTTTTTAACCGCTGTTGGCACTGCCAACACCGCGCAATTTTTAACCACTGTTGGCACTGCCAACACCGTACGATTTTTAACCATCAAACACGCGCCGCCAG
ACGGGCAGCATTTGCACGTTTCAGGCTGGCACGGAATTCTGCGACAATGTTGCTCACCGTTTCAGGTGACGCCACTTTCCTGCGACGACGTTCACGTTCATCACGGTTGTACACAGGCTGAT
ATGGTGCACGGGCTGGCGACCGGGATTCATGCACGGACATATCCGGCGTATTAA
>KGDGIN_03875 TIGR03761 family integrating conjugative element protein
ATGTCTGAAGAACAAATGAAGGAACCCGCATCCCGTCGTGTAGGTGCGCTTACTTCACCAGTGAATATCGAGTTGCACTCCCGTTATGCAATCATGCTGTGGGAAGGGAGACGAGGCACGA
AAAGTAAAAGTGCTGAAGATCGCAGGGCAGCGAAAATAATGAGTATGCCGGAAGCTATTAAGATGACTGGCGGCCTCTACAGAGATTCTCTTGCTGATAATCCCTATGCGGATGAAACGATG
TGGAAAGTTGAAAATGCGATTGTTGAGGCAACCGATAAAATTAATGCTGAAGTTCGTGTACTTGAAGGGATCCTCAGAAATATTCCGTCAGTGATCACTGTCCGGGATGCTCTTTCTCGTTCT
CCTGTCGATATTGGGGTTTTCAGCCGCTCTCCTCTGGGCTACCGCTGTGTATGGCTTCTTGTCGGGTATGATCAGCTTGCATTAAAAACGTTTCAGGCAGCACATCTGGGGCTTATTTCACGA
AACAGGCGTGATGAGTTGTTGTCTTCTGGTGCGCATCTTATCCGCAAAATTTACGGTATTCTGCAGGGGTATCGCACTATGGCGGTAACTCGGGATGATATTGAGAATAAAACAGAAAAGGC
AGAGAAAGCTATTGCCCGTTATGGTATGCCTCATGATGATATTCTATCCGGTAAGCTCAGATCTTCATTTTCTCCGCCCCTGAACCGTAAGTCCCGTGATGCATCCCGGCAATGA
>KGDGIN_03880 DNA topoisomerase
ATGCGTCTCTTTTTGTGTGAGAAACCTTCACAGGCCGGTGATATAGCCAGAGTACTTGGGGCTGCCAGACGTGTTGATGGTGCGATTGAAGGTAAGGACTTTGTTGTGACCTGGGCCTTTG
GTCACTTGCTGGAGCAGGCGCCACCGGAGGCGTATGGTGAGCAGTTTGGCAGTCCCTGGAAAATTGACGTTTTGCCGGTCTTTCCTGAACAGTGGCAGATGGTGGTGAAGGATAATGTCG
GGAAGCAGTTTAATGTGATCAGCCGCCTTCTCCGTAAGGCTGATGAAGTGGTGATTGCCACTGACGCCGATCGTGAAGGCGAAATTATCGCAAGGGAACTGCTGGATTATTGTGGTTATACC
GGCGTTGTGCGCCGTCTCTGGCTGTCCGCACTGGATGAGAGCAGTATCAGGGAGGCGCTGGGCAGGATACTGCCCGGAGAGGAGACCGAGAAGTTATATGAAGCCGGTAAGGGACGCA
GCCGGGCTGACTGGCTGACGGGGATCAACCTGACGCGTCTGTATACCCTGAAAGCCAGGGAGTGTGGTGTGTCCGGCGTCCTCTCTGTTGGCAGGGTGCAGACGCCCACGCTTGCGATG
GTGGTCAGACGGGATGCAGAAATCAAAAATTTTATTCCGGTACCTTACTGGCAACTGTATCTCTGGCTGGAGAAGGACAATGTGCGTTTTCGTACCTCCTGGATTGCGGCAGCGCAGTACTG
CGATACTGAAAAACGGTGTGTGAATGTGCAGGCGGCAACAGCCGTCCTGTCACAGTGTCGTCAGGCCGGTGTTGCTGTTGTCAGGGATGTCAGCAGCAGACGGGAGAAAAAGCAGCCTC
CGCTGTGTTTTTCGCTCGGAACGCTGCAGGAGGTCTGTTCGCGCAAATTTGGCCTCGGCGCGCAGGCCGTTCTTGATATAGCCCAGTCGCTGTACGAAAAGCATAAGGCGACAACATATCC
GCGTACAGATTGCGGTTATCTGCCTGTGTCGATGTACAAAGAAGTGCCTGACGTACTGGCAGCGCTGGAAAAAAACCGATCCGGATATTGCCCCGGAGCTGGCAAAACTCAGTCCTGATATTG
TGTCGGGGATCTGGAACGACAGGAAGATCACCGCGCATCATGCGATAATCCCCACCCGTCAGCCGTTTAATATTGGGGCGCTGAGTGATAATGAGCTTAAGGTGTACCGGTTAATTCGTCAG
CATTATCTGGCTCAGTTTTTACCCGTTCAGGAGCTGGATGTCACAGAGGCCACTTTTCTTGTTGCGGGGCAGTTGTTTGGAACGCGGGGGCGAGTGGAGGTTGTCAGGGGATGGCGTGCG
CTTTTTGTGTCAGATTCGGCGGGAGAGGAAGAGGATGTTGCAGACGATGAGCAGGCGATGCCCCTTCCCGTACTGGCAAAAGGCGATGAATGTCGTCCTGCTGAAGCTGAACTCAAAGA
GATGCAGACCACGCCACCGCGTCATTATACGGAAGGCACGCTGATTGCGGCGATGAAAAACGCTGCGGGGATGGTGAGTGATCCGGCACTGAAAAAAGTGTTACGCGAAAATGCGGGGC
TGGGCACTGAAGCGACCCGCGCAGAAATACTGGAAACGTTGTTCACCCGCAAATATATCGAGAAGCAGGGTAAGCATATCCGTGCCACGCAACTGGGGGCGGAGCTGATAGCGGGATTGC
CGGAGGCGCTGACCAGTCCGGGGACAACGGCGCTGTGGGAACAGGCTCTGGATGATATTGCACAGGGTAAACTGTCACTGTCGTCATTTATGGAAAAGCAGAAACAGTGGCTTAACCACC
TGGTGGCCGGTGCAAGGCAGCAGCAACTGCAGATCACGGCACCGGTTACGCCGGCATGCCCTCTTTGTGGGAGCAGAATGCGGAAAGTGAAGATGAAAAGCGGGAAAGGGGAATTCTG
GAGCTGCCAGCGTTATCCGGAGTGTAAGGGAGTGATTTCAGAGCAGAAACCGACATCCGGGAAACGGGGCGGCAGAAAAATTAAAAAGCATGGATGA
>KGDGIN_03885 STAXI RNA
TGTCATCTTCGGATGACCAGCCAATGTTCCAGCAGTCTGAGAGGAGCGCCTTCGGGTGACTGCCAGCCAATGTTTCAGCGATCTGAGAGAAACGCCTTCGGGTGATTGCAATATCCTCGTAG
CCTGAAAGGGGATGCCTTTATGGCGGTACATC
>KGDGIN_03890 DUF3577 domain-containing protein
ATGTCTGCAACTAACTCTACTTCTGCAAAAACTGAATACTTCAACCTGACCATGAAAGGTCTTGGGTATCTCAGCAATGTACGTCAGGTTAACCATCAGAACGGTTCGTTCCTCAGCTGCGTG
ATTAACGCACTGAGTGGACCAACGGACAATCCGTCCTATACGCGTTTTGATGTCACGGTTGCCGGTAAAGAGGCGACCACCCTGATCTCCCGCTGCCAGAAAGCAGTGGATGAGGACCGCA
AGGTACTGCTTGGTTTTACGTTAAGCAACCTTGCCACCGACATTTTCACCCTGAACAAGGGGGAGCATGCCGGTGAGCAACGCGTCAGCCTGAAAGCCCGCCTGATCAAGGTGGACTGGA
TAAAAATAGGCCAGGAGCAGGTCTATAAGGCTGAAAAAGCGGAGTCCCAGCCACCGAGCCATGGCACCACAAAACGTGAATACGCAGAAGACTCCTTCTGA
>KGDGIN_03895 GAF domain-containing protein
ATGACTTCTACAAACGGTATTCTCGCAGCTGCGGCAGGTTATACATTACCTCTGGAAATACTCAAAAGTGGGCGCGGATATTATATTGGCACTCAGTGTTCTGAAGGACCGGTATCCCGTGAA
TCAGAAGAGTATTTCAAAAAATATGAGCAGGCTGAGCGGGCGCTGAAAAATGGCACCTGGACACAACGTCATGCCTGGTAA
>KGDGIN_03900 Single-stranded DNA-binding protein
ATGGCTTCACGCGGCGTAAACAAAGTTATCCTGATAGGACATCTGGGGCAGGATCCGGATATCCGGTATTTCCCCAATGGCGGTGGCGCTGTGGCGAATCTGTCACTGGCGACCAGCGACA
CCTGGCGGGATAAACAGAGCGGTGAAATGAAGGAAGTTACTGAATGGCACCGCGTTGTTCTCTATGGAAAACTGGCTGAAGTTGCCGGCGAGTACCTGCATAAGGGGTCGCAGGTGTATA
TAGAAGGCCAGCTTAAAACCCGTAAATGGTTTGACCAGCAGAATGGTGTGGACCGTTATTCAACTGAGGTTGTTGTGGGCGTGAATGGTGTCATGCAGATGCTGGGTTCATCGCGTCAGCA
GGGCGCTTCCGGTAATGGTGCTTCAGCACTGGCACAAGGGGGCCAGGGACATCCGCCGAAGACGACCGGACAGACTGGTCGTGCCACCCGGACGACGTCAACGGGTAATGGTGCTCCA
CCAATGGACTTTGACGATGATATCCCCTTTATGGGGCTCGGGTATGGCATGGAACGACGAATTATTCATGCCATGTAA
>KGDGIN_03905 Transposase-31 domain-containing protein
GTGAGTGAAAAAGTTAACAAAATCACTATGATTCATGACGGGGCGTTCAAGAAGACGCTTGAAGATCCCGCCGCAGTAAGGGACTTTCTTGAACAGGTCTTGACACACGATCAGATATCCC
GCTGTAATCTTGATACAATAGAAGTTGAAAAGAATTCGTTCGTGACGGAAAGTTTGCGTCAGCATGCCTGTGATGTTTTGATCAGCATGAAGACAAACGACGGCAGGGACGGATACATTTAT
ACCTTAATCGAGCACCAGAGTTCGCCGGATAAATTCATCCCGCTGCGTATGATGCGATATATCCTGGCTGTAATGGAGCAACATGTTGAAAAGCATAAGTGTGCACCTGTGGTGATCCCCGTG
TTGTTTTATCATGGTGCAATGCGGCCATATCCGTATCCGCTGAACTGGGTGGATTGTCTGGATGACCCGGAGTACGGACGGGAGATTTATGGCGAGTCAAAGTCATTCAGTCTGGTGGATGT
CAGTACGCTTGATGATGATGAAATTGAGCAGTATCGCCGGATGGCGGCGCTGATGTACACCATGAAGTCCGGAACTCAAGGTGATTTGATTGAGCTGATTGGTAAATCGATCACACTGACGG
ATAAGTATGGCAGTAGCATTCACCTGAATACGGTGCTTACTTATCTGATGGAACTTTACCAGATGGATGTTGCTGAACTTTTTGAAGCAATATCCACACACTATCCCAGCCATAAGGGGGTGAT
TATGACGATAGCGGAACAACTGGAAGAGAAAGGTCGGGTTGAAGGTCTGGAGAAAGGCCTTGAGAAAGGCCGTGCTGAAGGCCGTGCTGAAGAGCGTCAAAAAGCGCTTGCAGAAAC
TTATGCATCTGTCCGTCGCATGAGCGATATGGGGATGTCAACGGAAGTTATTAAGCAGGCACTTCATCTTTCTGATGAACAGATTCAGGAAGCGCTTAATAATTAA
>KGDGIN_03910 Transposase
ATGGATTTTGCTGAACTTTCTGAAGCAATATTCACACACTATCCCAGCCACAAGGGGGTGATTATGACGATAGCGGAACAACTGGAAGAGAAAGGCCTTGAGAAAGGCCGTGCAGAAGAG
CGTCAAAAAGCGCTTGCAGAAACCTATGCATCTGTCCGTCGCATGAGTGATATGGGGATGTCAACAGAAGTTATTAAGCAAGCTCTTCAGCTTTCTGATGAACAGATTCAGGAAGCGCTTAA
TAATTAA
>KGDGIN_03915 hypothetical protein
ATGAAAATATCCCTTCTTTTTTCCGCGCTGTCTGCCTGTGTGTTGCTGGCGGGGTGCCAGAGTCCGCAAAAACTGCAGCAGAGGGCGGCTGCCGTCCCGGTACCGTCGGTGACAGTCAGCA
ACAATGTCCGTCCGGCAAATCCGGATGTGTACGCCGGTGCGGCGACACCGGAGGTGGTGCGTTATGACCGCTATCTGCTGGTTAACACGGCACCGGATACGGTACAGCGTGACCCGCTTTC
CCAGGTGATTGACATCCGCATTCCGGCATCACTGAAGCCGACGGTGGCGGATGCGATGCGTTATGCCCTGAAACAGTCCGGCTACACACTCTGCGCCACGGGGCCTGCCAACGGTGTGCTC
TACCGTCAGCCGCTACCGGCCGTGCAGTACCAGACCGGGCCGGTCAGACTGCGGAGCGCGCTGCAGATGATGGCGGGGCCGGCATGGCAGCTTGAGGTGGATGATGTGCAGCGCGTGG
TCTGTCACAGTCTGCGTGAGGGTTATCAGTTACCGGCACGCCAGCTGGCGCCGGTACCGGCGGTGGTGAAGGTATCCGGTGCGGAGCAGAAAAAATGA
>KGDGIN_03920 Membrane-bound metallopeptidase

ATGAGTGAAAAGCAACAGGAGATATCAGCCGCTGCAGGTGGTTCAGGTGGCACCACACCGTCAGGAAGGGTGAAACGGCGTCCCTCTCCCGGTCTGTTCGCAGGGCTGGTGACGGTGGT
GATGATGGCGGTCGCGGTTGCCGGTCTGGCTCGTTATACGGGAGAGCAGGACAGGCGAATGACTGCGCTGGCGGTGCAGATGAAGGCGTATTCTGATGCGCTCGCCTTGTCCGGAACGG
AACTGGTACCACTGAGGGAGAGGCTGGCTGAGACCGAAAAACAGCTGAAGAGTGTGGCGGAACGTATTTCTCAGCTGGAAAAGCGTCCTGCACTGACGGAGGAAGAGCATGCGCGTAC
GGGAGAGATGGCCGCCAGCCTGGCATCACTGCAGACCTCACTGGCGCAACAGGGAGAAACGCTTCGCACGTATGAGCAGCGACTGGAGAAGGTGGAGAAGACTTCAGTCACCCCGGTA
TCACCGTCATCTGCGAAAGGCGCGGGCGGGACGATGAAGGGCACAGCGGCGGTTGCGCCACAGAAGAAACGAATGTCTGCCGGCGTGCCGGTGGTCAGTCGTACCGCGCCGTTTGTTCT
GACCGGGACAGAGCAGCGGGGAGGCCGTACGTATGCGGCAGTGGCCCCCCGTGGATTCAGCAGTCTTGCTGATGTGACGTTACTGGGTGAAGGGGAGAGCACGACAGGCTGGGTGCTT
GAGCGTGCAGGACGTGGCGAGGCCACGTTCCGGGTGAATGGTCGTCAGGTCCGGGTCATGGTGGAGTGA
>KGDGIN_03925 hypothetical protein
ATGAAGCTGAAAAGTCTGTCACTGGCGCTCATGCTGCTGTCCCTGCAGGCGATGGCCGCCGTACAGGAGAGTACGCAGCAGGGAACGCGCACCTCCTCATCCTCTCAGGTCAGTACCACCA
CACAGGAACTGCAGCAGCAGGCCGGGCAGTGGGGACTCAGTGAGGAAGATTACCGGCGTTACCGTACCCTGATGGAAGGTCCCCGCGGTATTCAGTCTCCGGGGCTGGACCCGCTGACG
GTACTGGGGATTGAAGCGCGGACGGATGCAGAGCGCAGGGTGCTGGCCGAAAAATGGGTTAAAGCTGAGTATGAACGCACTGAGAAGGAACTGGCTTTCCAGCGTGAAGTGAATGCCG
CCTGGAAACGGTTGTATCCGGGGGTGATGCCGGTCAGCCTGGGGAATTCTGCCGGCATCGCCCGTGACACCGGGGGACGTCTTGCCCTGTTTGTCAGGTCGAAAGACTGCAGTACCTGTG
ATGCGCGGCTGGCCGCCGTGCTTTCATCCGGTCGTCAGGTGGATATTTACCTGGTCGACAGTCAGGGAAATGATGAAAAGCTGAGGCAGTGGGCCCGGGAGCACAGTATCCCGGCAGACC
GTGTGCGCAGTCGCCACATCACGCTGAACCATGACGGCGGTCGCTGGCTGCGGTTTGGGGAGGGGAGAATGCCGGTGGTACTTCAGCAGGGGGCTGACGGATGGCGCGTGGCAGCCTT
CTGA
>KGDGIN_03930 Putative Lytic transglycosylase, catalytic
ATGGCGCGTGGCAGCCTTCTGATGTCGCTGCTGCTGATGGCAGTCAGCATTCCGGCGGGGGCGGCGCTGCATGATATCCCGGAGGGATACGTGCGTGTGGCGGAACAGCATGGTGTTCCG
GCAGAAGTACTCTATGCCGTCTCGCTGACCGAGACGGCCATGGTCCCCTCCGCCATTGGTGCTGTAATGAAAAAACGGAATGAGGATTACAGGGTACCAGCAGTGGCACGTCCCTGGCCCT
GGACCATCAATGTGGCGGGCCGGGGATACCGCTATGCCAGTCGACTGGAGGCCTGGCAGGCCCTCCAGGTTTTTCTGAAACGATATCCGGCCCGACGGATTGATGTGGGGATAGCCCAGG
TCAATCTGGGCTGGAACGGGCACCGTTTTGCGTCCACATGGGCGGCATTTGACCCGTACACCAGTCTGAACACGGCGGCGGTCATCCTGAGAGAATGTCGCCGTCGCCATCCTGACAGCTG
GCTGCAGGCTGCCGGCTGTTATCACCATCCGGCAGGCGGCGCACCCGCAGTACGCTACATCGCGCTGGTGAGGCGGCATATTGACCGCCTCCACCGTGATGCGGTCAGTACGTCAGGTGTG
ACTGCCATGAATCCCCGTTATATCCGGACAGAACCCCGGGAGTTAACCCCATGA
>KGDGIN_03935 Integrating conjugative element protein
ATGAAACGATTTAAACGATATGTCATGACGACCGCAACGGGTCTGTTGCTGTGCGCGGCTCAGGCCGGCGCGGAGCTCAGGGTGATTGCTGACCTTGGTGGCAGTGATGCCGGCAGGTTT
TATGAAGCAATAAACCGCCAGGCGGACAGCGGAACGTCGTCGCCTGTCACGGCGCTGCTGCCGCAGGGGGAGAGTGCGATGCTGCCTGTTTCCACGCCGGAAATGTCTCCCGGAGAGGT
GACGCCGAGGCCACTGCAGTTACCGGGGCTGGGGGCACTGTTCCTCGTGGGGGATGATGCACTCTCCCGTCAGTGGCTGCAGGCGAATGCGGCCGCGCTGCTTGAAAGGAAGGCTGCC
GGTATGGTGGTGAGCGTGACGAATGAGAGCGGTCTGCAGGCGCTGCGGGCGCTGGCTCCCGGTGTGATGATGGTACCGGCTTCCGGTTCAGCACTGGCCCGCCGTCTGCAGCTTGACCA
CTGGCCGGTGCTGATAACGGACAGTGGTATCAGCAGTGTGGTGATGCCGTGA
>KGDGIN_03940 Mrr-cat domain-containing protein
ATGCAGAGACAACTGACGGACCTGTTACAGGAAGGCGCGTGGCACGGCATTCTGCTGTGCGTTCTGGTCGTGCTGTTTCTGTTTTTTCCGCGCCTGTGGGGGCGATGCAGGCATCGTGAA
CGCGCCAGTGAGCGTCGTCACCGCCGTTACCGCGCTGCGGCTGAACGTGTGCTGACAAAGCTGCCGGAAGGTGGGGCGGGTGTGAAATACCTGCGAAAAATTAACCCGTATGTGTTTGAA
GAACTGCTGCTTCTGGCGTTTGAACGCAGGGGGATGGCGGTCATCCGTAACACACGTTACAGCGGAGATGGTGGCCTGGATGGTCAGGTGCTGATTGACGGCGAGCGGTGGCTGATTCA
GGCCAAACGTTACGGACGGACCATCCGTCCGTCACATATTGATGATTTTGGTGACCTGCTGCGCCGTGAAGGCTGCCGTGGATTTTTTATTCACACCGGTCGTACGGGACCGGCCAGCCGG
GCACGGCTGGCGCTGTGGCCGGATATTGAGCTGGTCAGCGGGCAGCGTCTGCTGAAACTGCTGAACGGGGCATTGCTGCCGGATGAACTTACCAGAGGAGCGCGGAGATGA
>KGDGIN_03945 Lipoprotein
ATGATGACGGTAAAACGGATGTTTATGTTTTTATTCAGAATAACGATTGTCTGGCCATCCGTGCTGACAGGATGCGTGATGATGCTGTATTTCGCGAAGGTCGCCGGCGGTGGTGTGGCGGG
ACTGGAGCAGCGTTACGGGGTGGAATCCGCCCGCTGGCGTGATGCGCCGGAAGGATGGTGATGACAGTGCAGTGCCCGTCGCGTCCCTTTATGGCGTCTGTGAGCACCGGGGAGTGGC
GTGCACTGGCTGCATCTGATGATGAATGCAGCCAGGTTTCGCGCTCTTTCAGAGAAGCTGCAGAGGGCGATGCGCAGTCGCTGCGCAGAGTGGCAGTGACACTGCTGCTGGCATCCGTGG
TTCTGGAAGCGTTTTCAGCCTGCATTCTGCGCCGGGTAACGGCATCCGTCCGGGTATCACCACACTGTGGATATGGCTGCGGACGTGGTGCGGTGGCGCACTGGCCAGAGAGCAGCGTTC
ACACTGCACCGGAGAAAGGAGAGCGGGTGTGA
>KGDGIN_03950 Carbazole 1,9a-dioxygenase, terminal oxygenase component CarAa
GTGAACGTTTCATTTACTGAACAGGTGCAGAGGGATATGCCGCAATGGGCGAATGACCCGTTACTGGCATGGGATGCCACCGCGTTTGCGGCAATGTCGGATTTTATTACAGAGCACTACTG
GACGGGACAGGGCAGCATCAATGTGTTCCGCATTGTGGGAACTGACCATCCACAGTATGCCGGAATGACCTGGCTGGAGCTGCTGGAGCGCGGTAACCGTATGGACATCAATATCCCGTTA
CTGGAAAAAAATCCGGGATACTACACGCAGGCAGAGCAACAGCATGCCGGGATGAGTTTTGTCAGCACGGATGGCGTTGACTGGTATGTCAGTGCAGACGGCAATCACCGCAGTTGCCTG
GCACGCTTTCTGTTTCACCTGCAGGGGGAGGAGCGCACACAGCTTCACAACGTGGCACAGAGTATTTATCACACAGACCGGGCGTTCTGGTCTGCCTGCCGGGAAATCCACAACCTGACT
GACGCGCTGTCCCGGCATGGTGTGTATATCCGTCTGCAGACCCGACGGCAGTGTGTTGCCCGCGAAGACCTGGCCTGCTGGAAAGTGGACCGTTTCAGCACAGAGGCGCAGCTGACAGT
GGATGACGTGCGTGCCGGCGGACATGATATGCCTGCGGTGTATAAGACGTTGCTGCTGAATGCAGCAGACGCATGGCGTGAGGTGATGGTATTACAGCGCAGACTGGAGGCGCTGTCTGC
ATCACCGGGGAACGATTTGCCACGCACATGGTGGCGACGTCTTTTACGGAAGGGGGACATGTAA
>KGDGIN_03955 TraG-D-C domain-containing protein
ATGAGCAACCGCTATGTCATTGAGGCATTGCTGAGACCGGCTGTGGAGTTCAATACGGCAGTGGTGGCAGCAACAGCAGCATGTATCTGTGTGACAGCCCCCTGGGCGGTGGCGCTGGCA
CCCACCGTCAGTTACGCCACTGCCGGTGGATTTGCCCTGCTGGCCGTGGTGCGTTTCCGGCAGGGGATGAACATCATCCGGTATCGCCGTAATCTGCGCCGGCTGCCCCGCTATGTGATGAG
TACACGGCAGATCCCGCTCAGCCGTCAGCGGCTGTTTCTGGGGCGGGGATTTCGCTGGACCCAGAAGCATACCCAGCGTCTCCAGGATACGCTGCGACCGGAGGTGGCACACTATCTGCA
GCCGGGCGGACTTTACCGGCTGGCGCGCTGGCTGGAAATGAAAACGGAACACCGTCTGCCCGCGCTGGGGAAACTGCTCCGGCATGAGTCGTCGCTGAATCCTGTGCGTCCTCTGCCGCC
GGTGGGGGGAAATCCCGCCCTGCATGGTATTGAGCCGGATGAACAGGATGTGACGCTGGCTCTGGGGGAGCGTGTGGGGCATACCATTGTCTACGGCACCACCCGTGTCGGTAAAACTCG
CCTGGCAGAACTGCTGGTCACTCAGGATATCCGCAGGGGAGAGGTGACCATTGTGTTTGATCCCAAAGGTGATGCTGATCTGCTGCTGCGTGTCTGGGCGGAAGCACACCGTGCCGGGCG
GGGTGATGAACTGTATATTTTCCATCTGGGATGGCCGGAAATTTCGGCCCGATATAATGCGGTGGGACGCTTCGGGCGTGTTTCGGAAGTGGCTTCCCGTGTGGCCGGGCAGTTGTCCGGT
GAAGGGAACTCGGCGGCTTTCCGCGAGTTCGCCTGGCGGTTCGTCAACATCATTTCCCGCGCCCTGGTGGCACTCGGTGAGCGCCCGGACTACCTGCTGATTGTGCGGTATGTGAACAAC
ATAGCCGATCTCTATATCCGCTATGCCGGAAAGGTGATCCGCGAGCGCATGCCGGAACTGGAGCAGGCGATTGCGAATAACCAGAATGTGATCAGCGAGGAGGATGTTCCCCGCACCATGC
AGAACCAGCCGGATGCGGTACGTATCTGGTCCATCGAGATGGCCCTGAGCAGTGAGGAGGGTAAAAAAATTTACGATCCGATCCTGGACGGGCTTCGCAGTGCGGTCCGCTATGACCGTAC
CTATTTTGACAAAATCGTGGCGTCCCTGCTGCCGCTGCTGGAAAAACTCACCACGGGCAGGATCGCAGAGCTGCTGGCACCGGACTATCTGGACATGAACGATGCGCGACCGATTTTTGAC
TGGGAACAGGTGATCCGTAAAAAGGCGGTGGTCTATATCGGCCTGGATGCACTCAGCGACAGTGTGGTAGCCAGTGCTGTGGGGAATTCGATGTTTGCGGACCTGGTGAGCGTGGCGGG
GCATATCTACAAGCACGGCATGCATGCCGGTCTGCCCGGTAAGGGGGAAGGCAAAAATGTGGTGAACCTGCACTGCGATGAGTTTAACGAACTGATGGGGGATGAGTTTATTCCGCTCATT
AACAAGGGGGGCGGTGCCGGTATGCAGGTCACCGCGTACACCCAGACATCGTCAGATATTGAAGCGCGTATCGGTAATGCAGCCAAAACGGCGCAGGTTCAGGGAAACTTCAACAACATG
ATCATGCTGCGTGTCAGGGAGAACAGGACGGCAGAGCTGCTGACCACACAACTGCCTCAGGTGGAGATCTACACCAAGACGCTGGTCTCCGGTCACCAGGATACGGCGGACGTGTCTGCC
GGTCAGGATTTCACGTCCAGCACCCAGGATCGTGTCGGCACGGTGAAGGTACCTCTGCTGGAGCCGTCAGATATTGTCACCCTGCCCAAGGGACAGGCGTTTGCCCTGCTGGAAGGGGG
GCAGCTGTGGAAAATCCGGATGCCGCTGCCGGCAGGGGATACGGATGACGTGCTGATGCCGGAGAACATTGCCCGGGTGGCAGAGGCGATGAGACAGAACTATCACACCGGTGAAGGA
TGGTGGAATGAGCCGCCGTCAGCGCAGGCCGCCGGAACAGATGAAGGAGGCGGATATGGCGGAAGCGCGTCGTGA
>KGDGIN_03960 Integrating conjugative element membrane protein, PFL_4697 family
ATGGCGGAAGCGCGTCGTGATGCGGAACGCAGCCCGCAGTCCCGGGCGCCCCGTCAGCATGGTCTGCTGTACAATCTGGTGTGGGGCTGGCCGTGGGCACTGGTGGGGCTGATTTTATCC
TCCCTGCTGCTGAGTCTGTTGATTGAGTATGCCGGTATGACGTTTTTCTGGAGTGAGGAGGGGGCGGCGCACAGCCGCGCTGTCATGAACACGGAGCTGGGCTGGCTGTCAACGGAGTTC

ACCCGCAGTCTGCTGCTTTCCGCGCCGGCAGAAACGGTGCCGGCGTGGATTTCCGCCGCGTATCAGTGGCTGTTTGTGGACAGTGGCATGCAGACATGGCTGCATGCACAGCAGGCGGG
GACGGCGGCAGGCAGAAGCGTGTCTGAATTCAGCAGTGAGCTGAGTCAGTGGCTGGCTTATCATCTTCATGACTACCTGCTGGCGACGGTGTATGTGACGGTGGTGACGATGGTCAGGGG
GGTTATTCTCGTCCTGTCCGTTCCGTTGTTTGTGATGGTGGTTCTGGTGGCCGTTGTGGAGGGGCTGGGGCGCCGTGATTTGCGGCGTTACGGCGCGGCTTATGAGTCCAGCTTTGTCTATC
ATCATGCAAAAAAATCAGTCAGACCGGCGATTTATGTCCCCTGCATTATTTACCTTTCCTGGCCCGGCGCGGTATATCCGAACCTGCTGATATGGCCGGCGGCCATGCTGCTGGGACTGGCGG
TTACCGTGACTGTGGCATCGTTCAAAAAATATCTCTGA
>KGDGIN_03965 hypothetical protein
ATGGAATTTCTCCTCACCGCCCTTCCGTACCGGGCCCTGTTCAGTCTTACCTGGTTTTTTGTTGTGTTATATATCGTCAGGGAAGAGCCACAGTGCCCTGTCTGGTTATATGACGTCATCAGGG
GCATCAACCTGCTCATCATCGTCATCACCATTGTTGTCATTCCCTTGCTGCCGGTGCTCTTACGCTGA
>KGDGIN_03970 Integrative conjugative element protein, RAQPRD family
ATGTTTTCTCTTCCCGCATTTCCCCGGCGTGCCGCTGCCCTGGCAGGTACTACCGGGATGATGCTTCTTGCCATTGTGGCTGTGCCGGTTCAGGCCGCCGAAAAGGATGAACTGGCAACGG
CCATGCGCCAGCTTGACCAGGTACGGGCGGCGCTTGACCGTGCCAGAACGGCATCCCTTCAGGCCACGCCTCATGACGGGCGTTATTTCTTCGACTACCCGCGCGCACAGGCTGACATTCA
GACCATGCAGGCGGGTATTGACCGTTATCTCACGCCGTCCCGTGCCCAGCCCCGAAACGGCAGTGCCGTGACCGGTGAATACCGGCGGGAGCGTCCCTGA
>KGDGIN_03975 Putative membrane protein
ATGGCGCTGACGTCGGCACAGATCACGGCCTTTAAGGTGGCATCCGGTGATGTGGATCTGAATGTGGTGCATCTGACATCGGTGGGAATGCTGGTTGCCGTTCTCTTTCTCTGGGCCGCCTG
GGGACTGTCGGATGCATGGTCCGGGTGGCGTAACGGTGACGTCAGGGAATCTGCGTTTATCTGGTTTACCGTCCGTACCGCACTGCTGCTTGTCAGCAGTATCTGGATGTTTGCGGGCTGA
>KGDGIN_03980 TIGR03745 family integrating conjugative element membrane protein
ATGTTTAAAAACGCTTTTACTTTCCGTCACCTGCGTCTGGCGGGAATGGGGCTGATTGCTTTTCTGCTGGGCAGTCTGACCCCGGCACTGGCCGATTTGCCCAAAATTGAGCCGCCATCATC
CGGTGGGGGTGGTGGTTTTTTTAATCAACTCAAAGGCTATATCCAGGATGGCATTGTGCTGTTTGGTCTGGTAGTTGCAGCGGCGGCCTTTGTGAAGGTGGCGGAGGCTGCCGTTACCACT
TTTTCTGAGGTGCGTGACGGCAAGACGGGCTGGGGACAGTTCGGGGCCATTATCGTGGTCGGGGTGGTGCTGCTGGTTGCGGTTATCTGGCTGCTCGGCAAATCCGCGAACATCATCCTG
TAA
>KGDGIN_03985 TIGR03750 family conjugal transfer protein
ATGGAAACCATTCGTTTTCTGCCCGATCGTCTGAATGCCGAACCGACGGTGTTCCGGGGATTCACCACGCCAGAGCTGGGTCTGGCCGCACTGAGCGGTGTCTTTCTCGGTCTTCTCTGGA
CACTGCCACTGATACCGCTGTTCGGCTGGGTGATTATTCCCACCGGTGCCCTGCTCACTCCACTGCTTCTGGTCTGGACCGGCGGCGTGTGGATCACCCGGCTGAAACGGGGCAAACCGGA
TAACTGGCTCTGGCAGCGTATTGAAGATAAAAAAGCGGCGCTGGGTGTGGGGCGCCCTGCATTTATCAGGGAAGCACAGGGCTGGTCCCTGCGTCGTTCCCGCGTCAGGGGAGGAAAAG
GATGA
>KGDGIN_03990 TIGR03746 family integrating conjugative element protein
ATGAGTCGTTTTCGTCATGCCCTGCAGGATCGTGACCAGCATATTCTGACGCTTCGTGTGTTCAGTGGTGTGCTGGCGGGGCTGCTTCTGATGGTCACCCTCGGCTGGATGTCTGCACCTGA
CCGGCTGACCATCCATAATCCACCTGATTTACGTTCCGGCAGTACCCGTCCGTGGTGGGAGGTTCCGCCTTCCACTGTCTATTCCTTTGCGTATTACATTTTCCAGCAACTGAATGCCTGGCCG
AAAAACGGTGAGGTGGATTATCCGGCAAAAATTCATGCCCTGTCGGCCTATCTGACACCGTCGTGCCGGGAAATCCTTGAAACGGATGCACGACGCCGTAAGGACGCGGGGGAACTGCTG
GATCGTGTTCGTGTGGTGTATGAGATCCCTGAGCACGGTTACGGCGGACGTTACAACCGTAATCCGGTCACGGTGGAAGGCCGGGACAACTGGCAGGTTCGTCTCGACTTAGTGGTGGAT
GAGTATTTTCATGCTGAGCCGGTCAAACGGGCGCTGGTCCGCTATCCGATGAAGGTGGTGCGTATGGAGGGCGATCCGGAACTTAATCCGTTTGCTCTGGCGCTGGACTGTTACAGCTCCA
CGCCGCAGCGTCTGGAAGCCGTGAAGGGAGGTGGTGAATGA
>KGDGIN_03995 TIGR03749 family integrating conjugative element protein
ATGAGAACCTGTGTACTGCGCAACCTGCTGCGTCCTGCCGCTGTGATGTTGTCGCTGTCAGTGATGTCCCTGACGGCGCAGGCAGATGAACTGATGAAGTGGGAGCGTATTCCGCTGGATG
TCCCGCTGATGGTGGGGCAGGAGCGGGTCATTTTTGCCGATAAAAACGTGAGGGTGGGCTTTCCGCCTGCACTGAACGGCAGGCTGCGGGTGCAGAGCACCGGCGGTGCCGTCTACCTG
AAAGCGGACAGCGCCTTTCCGAAAACGCGGGTTCAGCTGCAGGATGTGGAAAGCGGGGAGATGATCCTGCTGGATGTGACGGCCGCAGAAAAGGGGCCCACTGAGCCTGTCCGGATTG
TGTATGACGGGGAGGTGAACACGGTGAGCCGTGCCGGCGGCAGCGCTCCGGAGAGCGCGAAGGCGGGCAGCAGTGACGGACGTCCCGGGAAAGTCGGTGCGGATGACGGCACGCAG
GCCCGCCGGACCGCCCCCCGTTATGAGGAGCCCCTGCCGGTTCTGCTGACCCGGTATGCGGCACAGTCCCTGTATGCTCCGCTGCGCACCCTTGAACCGGTTGCGGGGATCAGACCGGTG
GTGCCACCTCTTCCCCGGCAAATCACCACGCTTTACCCGTCTGAACCCCTGACGGTATCCCCGGTGGCGGCATGGTCCGCCGGCGGACGAACCGTGGTGGCCCTGAAACTCACCAGTACGG
TGAGCCGGCGTATTGTGCTTGATCCGCGAGCCCTGCAGGGCCGTTTTGTGGCGGCCACCTTCCAGCACCGCTGGCTGGGTGCTGCCGGGACACCGGAAGACACCACAATGCTGTATGTGG
TGACGGCAGGCCGCCCGGAGAAGGCCTTTGTGGCAGAGCCTGTGGCACCGAGCAGGACGGGCGGGGAGGTGAAACATGCAGATTAA
>KGDGIN_04000 Integrating conjugative element protein, PFL_4705 family
ATGCAGATTAAGTCCAATACCCTGGTGAAATTTGTGGTACCTGCTGTGGTACTGGCGGGGCTGGTGATCACGATGAAAAGCTGCAGCACACCGGAGAGCGGGAGTGAGAGTACAGCACAG
AGCGGTGCGCTGGCCGGATTATCGTCAGAAGAGCTGCGTGCGCTGGGCGTGGAAGGGGACACGCCGGAGGATACCCTGCGGACCCTGGTTGCCCGCCTGAACCAGGTCAGGGATAACC
AGAAAACGCTGGAGAGCCAGAATGCGCAGTTGCTGAAGGACAACGAACAGCTGCGTCGCCGGGGGACGGATGTGTCCGGCCAGGTCAGTGAAGCCGTGGCGGGAATGCAGAAAACCT
ACGATCAGAAGCAGCGTGAACTGCAGGAGCAGCAGCGCTCCCTGATGTCGCAGATTGATGTGCTGCGGGGGCAACTGAGTGACCAGCTGACGGGAAGTCACACGAAGCTCAGCGATATT
CCTGACGGGCTGGGACTGGATTCAGGCAGTGGTGCCGGGTCGTTGATCTCGCAGGGGGCGGACGGGCTGATGTGGGTGAGTCCGGCCGATCAGTCACAGGATGATCCGCTGAGCAGCG
GCGGAAAGAGCGGTCCGCGTTTTCCGACCTCCTTCCTTGAAGATAACCCCCTGACCCGGCAGAAAGCAGCCTATGAGCAGCAGGTGAAGGGGTACAGCAATGAGCAGCAGGCGGCCACC
ACGCAGGCCGTGTACACCCTGCCGGAGAACTCAACCCTGACGGGCAGCCGTGCCATGACGGCCCTGCTGGGCAGGGTGCCGGTAAACGGCACGGTGACCGATCCGTATCCGTTCAAGATC
CTCGTGGGGCGCGATAACCTCACGGCAAACGGCATTGAACTGCCGGATGTGGAGGGGGCTGTGGTGTCAGGGACGGCCAGCGGTGACTGGACGCTTTCCTGTGTGCGCGGACAGGTGA
ACAGCATCACCTTTGTGTTTGCTGACGGCACGGTACGCACGCTGCCACAGGCGGATGTGAACGGTACCACCAACAGCAATCAGCAGGCGAAAAACGGGAATAACGCAGGCGGGATTGGC
TGGATTTCGGATGAGGATGGGATCCCGTGTATCAGCGGCACCCGTAAATCCAATGCGTCAACCTACCTGCCGACGATATTCGGGCTGGCCGCAGCCGGCGCGGCCGGGGATGCCCTGAGTG
AGGGGCAGTACACCTCGCAGAACAATGTCAACGGTATCACCTCGACGCTGACCGGCAGTGCCGGTGAAGCGGCGCTGGGTAAGGCCCTTTCCGGCGGGATGAATGAGACCGCGGACTGG
GTAAGACAGCGTTACGGGCAGACCTTTGATGCCATTTATGTGCCGCCGGGAAAAACGCTGGCCGTTCACATTACCCGTCAGCTGGCGATTGATTATGAAGAGCGTGGACGTCGTGTGCGTT
ATGACTTCACCCTGCCGGGAGAAGCGGGCGACCGGGGCGGCCTTGACTGA
>KGDGIN_04005 TIGR03751 family conjugal transfer lipoprotein
GTGAAACGATTACTGTTCGTGATGAGTGTGGCACTGGTGCTGTCGGGCTGTGCCACCTCTAAAGAAGAGCTGCTGCCTGCCGGAGAGCAGACGATGCTGGAGCTCTGGCAGGGTAAAACC
GCAGGTGGCGGTCAGCGGCATGCGCTGGCCGCCCGGGAAACGTTGCGCCGTCCCCTTACGGTAACGGAGCAGCAGGGGATGAACGCGGAGGCGTACAGCTACAGCCGGACGCAGGAA
AGCGAAATCAGCCAGCAGTTTCCGCGTCTGCCGAATCCCGACATGGTGATGTATGTGTTTCCGCATCTGGCCGGCGGTACGTCGCCGGTTCCGGGCTACAGCACGGTATTTCCTTTTTACAG
CAGGACGCAGTATGCGCTGCCAGGTGAGCGTACGGAGGCTCTGTAA
>KGDGIN_04010 Conjugative transfer ATPase
ATGACATTCTCGCTTTTCAGACGCCGTGAGGAAAAAGGTACAGCAGAGACCAGGGAACGACCGGAAGCAGAAGCGGGGGCATCTTCATCGGCCTTTGTTCCGCCCTGTCCTCCGGGGGC
ACAGCCCCTGCGACGTCCGGGACAGATGACCCGTGATGCTGAGGCGGCGGTTTATCATGCCGGTCCGTCGGTTATTGATTTTCTGCCATGGGTGGAATATCTGGATGAGGAGCAGTGTCTG
CTGCTTGACGACGGGGTGTCGGTGGGAGCGGTCTATTCGGTGACGCCGGCCGCAACGGAAGGGCGTACGGAAGAGCGCCTGGCGCAAATCAGGGACATGGTGGAGGATGCCCTGCAG
GACAGTTTTGATGAGTACGACACGCATCCCTGGGTGGTGCAGTTCTACTGTCAGGATGAAAATGATGTGGATGCGTACCTGGACTATCTGAAGGGGTATGTGAAACCCCACGCGCAGGGAA
CGGCATTCACGGAGGCCTGGCTGGGTGAAATGGCCCGCCATCTCAGGGGGATTGCCCGTCCTGAGGGACTGTTTACGGACACCCTGGTCACCGGTCAGCCCTGGCGGGGACAGCAGCGA
CGAACCCGGATGGTGGTTTACCGTCGTATCGGGAAGAACAATAACGATCCGATGTCGCCGGTGGCGATGCTGAACCAGGTCTGCGACCGCGTGGTGTCCGCACTGGGTGGAGCCGGTATC
CGCTGTACCCGGATGAACGGTCAGCAGGTGCACAGCTGGCTGCTGCGGTTATTTAATCCGGCACCGGACTGGATTGCCCGTGAAGAGCTGTACCGGATGGCCTCACGGGTTGATCCGCGT
GAGCGTGGTGATGATGTGATGCCGGTGATGACGGATTTTGCCGAGAGCCTGTGGTTTAACCCGCCGTATTCCGATCCGGAGAACGGGGTGTGGTGGCTGGACGCCATGCCGCACTGTGCC
GTGGTGGTGGAAAAGCTGCGTACGCCGCCGGAGCCGGGGACCATCACCGGAGAGATGCCCCGTGGGGAAAAAACGGTGAATGCGCTGATGGACACCTTTCCGGAGGGAACGGTGATGT

GCATGACCATCGTGGTGCAGCCCCAGGACACCCTGGAGGACCGTTTCACCCGGCTGTCGAAAAATGCGGTGGGTGAGAACACGGAATCCATCCGGGCGCGTCAGGACGTGAAGGTGGTG
AAGGAGCTGCTGGGGAACCGGCACAAGCTGTACCGGGCAGGGCTCACCTTTCTTCTGCGGGCAGAGGATATGGACAGCCTGAAACGCCGGCGTGTGGAGCTGACCACGGTGCTTCTGG
GGGCTGGTCTGCAGCCGGTGCGGCCTGAGTTTGACGTGGGGCCGCTGAACAGCTGGCTGCGGGCGCTGCCGATGTGTTTTGATCCGGATACGGACAAAAAGCAGTGGTACACGCGTCTG
ATGTGGGTACAGCACATGGCAGGATTGTTGCCGGTGACCGGCCGGGATACGGGGACCGGACATCCGGGGTTCAGTTTTTTTAACCGGGGTGGTGATGTGCTGACTTTCGATCCGCTGAAC
AAGCTGGACCGTACCCAGAACGCGCATCTGCTGCTGTTCGGACCAACGGGGGCCGGTAAATCGGCCACGCTGTGTGCTTCACTGTCGCAGGTGATGGCAGTACACCGTCCCCGCCTGTTCA
TTGCCGAAGCGGGGAACTCGTTTGGTCTGCTGGCCGATTATTTTGAGAGTCTCGGGCTGTCGGTGAACAAAATCAGCGTCAGACCGGGAACCGGTGTGACCCTGCCGCCGTTTGCAGATG
CGCATAAACTGGTTGAGGAAGGGCAGGATCTGCAGTGCGTGGATGAGCACTCACTGCCTGATATTGAGGATGAAGCCGGGGATGAAGGGGAGGATAAACGAGACATTCTGGGCGAGATG
GAAATCTCAGCCAGGATGATGATCACCGGGGGGGACCCGAAAGAAGAGGCCGCGCTGAAGCGTGCGGACAGGGCGATGATCCGCGAGGCGCTGCTGATGGCCACACACCGGACATACC
GTGAGGGGCGGCAGATGCTGCCGGAGGATTTGCAGTCCGCCCTGTGGGAGATCTCCCGTGACAGCAGCCGTAACGATGTGCGCAATGCAAAGGTGGCGGAGATGGCGGAGTCTCTGGG
GATGTTCACCCAGAAGGGGAGTTTTGAGGCGGAGTTGTTCAACCGTGAGGGCGAACTGTGGCCGGAAGCGGATGTGACGCTGATTGACCTGGGGCATCTGGCCCGTGAGGGGTATGAG
GCACAGATGGCGCTGACCATGGTGTCGATGACCAACATGATCAATAACATTGCCGAACGTGATCAGTATCTGGGGCGTGACATCGTGTTTGCGGTGGATGAGGCGCATATCGTGACGGTCAA
TCCGCTGCTTTCGCCGTACATGACGAAGGTTGTGAAGATGTGGCGTAAGCTGGGAGCGTGGCTGTGGCTGGCGACACAGAACCTGAAGGATTATCCGGATGTGTCGGAGAAGATGCTGAA
TATGGCGGAATGGTGGGTGTGTCTGACGATGCCACCGGAGGAAGTGAACAACATTGCACGCTTTAAATCACTGACGGAAGAGCAGAAAGCGGTGCTGATGTCGGCGAGTAAGCTGTCCG
GCTGTTATACCGAAGGCGTGGTGCTGGCGAAACAGATTGAGGCGCTGTTTCGTGCGGTGCCGCCAAGTCTCTATCTGGCGCTGGGCATGACGGAGAAAGAAGAGAAGGCTGAGCGGCGC
ATGCTGATGAAGGAACATAACTGCAGCGAACTGGAGGCGGCGTTCCATGTGGCGCGTAAGCTGGACAGGCTGCGTGGTCTGTCGGCCATGGAGGCGGATGTATGA
>KGDGIN_04015 hypothetical protein
ATGATGTGTCTGAAATACCCGAAACCGGAGGTGATGACAGAGGTCATGCCCGGGGGCAGCGTGTTTTTTCTGCCGCCGCAGGGAAAACCGGGTGTTGCGGATCTGGCGCAACCGCATCTG
CAGCGCCTGCGGTCACAGCTTGAACGCAGGCTGGGTACGCTGCATCGTGTGGTGTGTCAGCCCCAGCGGGTGGGGCAGAGCAGCAGTGTGGCCGTGACAGCAGAAGGGGCGTGTGGC
GAGGTGCATCTTCTGCTGACAGTAGGGGGGCATGAGAGCTGGCCCTCTGAAGAGGAATACCGGCATCCGCGCTGGTATATCCAGGTGGTGGATGCGGCCGATCTGTTCTACCTGCTGCTCT
GGCTGTGTGATGGCGGGCAGGAAGCGTGA
>KGDGIN_04020 bifunctional siderophore receptor/adhesin Iha
ATGCGAATAACCACTCTGGCTTCAGTAGCCATCCCTTGTCTCGGATTTTCAGTCAGCAGCATGGCTGCGGAAGATGTGATGATAGTCACCGCATCCGGCTATGAAAAAAAATTAACCAGTGCA
GCTGCGAGTGTTTCGGTGATCAGTCAGGAGGACTTGCGATCCAGCCAGTATCATGATCTTGGGGAGGCACTGAGATCCGTAGAGGGGGTGGATGTTGAAAGCGGTACGGGTAAAACCGG
TGGACTGGAAATCAGCATCCGTGGAATGCCTGCCAGTTATACGCTGATACTGATTGATGGTGTCCGTCAGGGCGGGAGCAGAGACGTAACCCCGAACGGTTTTTCTGCCATGAACACGGGG
TTTATGCCACCTCTGGCCGCCATCGAACGTATTGAGATTATCAGGGGCCCCATGTCCACTCTGTACGGCTCTGATGCCATGGGGGGCGTGGTGAATATCATCACCAAAAAGAATGCAGACAA
ATGGCATTCTTCAGTTAATGCCGGGCTGAACCTGCAGGAAAGTAACAAATGGGGGAACAGCAGCCAGTTTAATTTCTGGAGCAGCGGCCCCCTGGTGGATGATTCTCTCAGCCTGCAGGTA
CGTGGCAGCACGCAGCAGCGACAGGGCTCATCGGTCACATCACTGAGCGGCACCTCAGCCACGCGCATCCCTTATCCAACAGAGTCACAGAATTATAATGCAGGTGCTCGTCTTGACTGGA
AAGCAACGGACAAGGATATGTTCTGGCTTGATATGGATACTGCACGACAGCGTTATGATAACCGGGACGGCCAGCTGGGCAGCCTTACTGGCGGTTATGATCGTACCCTGCGCTATGAACGT
AATAAAATTTCGGCAGGTTATGATCATGCATTCACCTTCGGAACCTGGAAGTCATCCCTGAACTGGAATGAGACGGAAAATAAAGGCCGTCAGCTTGTACGCAGCGTACTGAAGCGTGATAA
ATGGGGACTTGCCGGCCAGCCGAGGGAGCTTAAGGAGTCGAATTTTATCCTGAATTCCTTACTGCTTGCACCTGTGGGTGAATCTCACCTGGTTACTGTCGGTGGTGAGTACCAGAACTCAT
CCATGAAAGACGGGGTTGTTCTCACCAGTACAGGCGAAACCTTCCGGCAGAAAAGCTGGTCGGCATTTGTTGAGGATGAGTGGCATATCACGGATGCGGTTGCACTGACTGTGGGCAGTC
GTTATGAACATCATGAGGTGTTCGGAGGACACCTCAGTCCGCGGGCATATATGGTCTGGGATGTGGCTGATGAGTGGACGCTGAAAGGTGGGGTGACCACGGGATATAAGGCACCCGGCA
TGGGGCAGTTACATAAGGGGATCAGCGGTGTGTCCGGTCAGGGGCGTACGAATCTTCTCGGTAACCCGGACCTGAAGCCGGAGGAGAGTGTCAGCTATGAAGCGGGCGTGTATTACGATA
ATCATGCCGGTCTGAACGCCAATATCACGGGCTTTATGACAGAGTTCTCCAACAAGATCGTATCTTATTCTATTGATGATAATACCCGCAGCTATACAAACAGCGGAAAGGCCAGGACTCAGG
GTGTGGAGTTTGCCGGCACACTGCCTCTGTGGTCAGAGGATGTGACGCTGTCTCTGAATTATACCTGGACGCAGAGTGAACAACGGGATGGGGATAATAAAGGTGCACCACTGAGTTACAC
CCCTGAGCATATGGTGAATGCGAAACTGAACTGGCAGATTACTGAAGATGTGTCATCATGGCTGGGTGCCCGTTACCGCGGGAAAACGCCACGTTTCACCCAGAATTATTCGTCACTCTCAG
CTGTACAGAAGAAAGTGTATGATGAGAAAGGTGAATATCTGAAAGCCTGGACGGTACTGGATGCGGGCATGTCGTGGAAAGTGACGGATGCACTGACGCTGAATGCAGCGGTGAATAATG
TGCTTAACAAGGATTACAGTGACGTAAGTCTGTATCGTGCCGGCAGTGGTACGCTTTATGCGGGTGATTACTTCCAGACGGGGGCATCGACAACAGGTTATGTGATACCGGAGAGAAATTAC
TGGATGTCGCTGAGCTATCAGTTCTGA
>KGDGIN_04025 TIGR03757 family integrating conjugative element protein
ATGTACCGGATTTTCTGTTCCCTGTTTTTCCTGCCGCTGGCAGCGTCTGCCGGCACAACAATTTATACCGATTCTCAGCACCTCCCCGTTAATCCTCCCGATGGTGTCAGGGTGGTTCTGCTGG
ATGCACCGGAGCAGCTTCAGACCCGCTTTTTTGGCACGTTGCCTGCTGATGCGGATAAGGCTGAAGCGGTGGTCAGGGCACGTATGCAGTCTCCCGAATGGCAGATGATGCAGGCGGAAC
TGGCTGCTCATTACCGCGATGTGGCACATGCCTGGTCGCTGGGCGTGAAGAAATATCCTGCCGTGGTGTTTGATGACAGTGAGGTGGTTTACGGCATCACGGATGTGGTGGTGGCCGGGA
AACTCCGTACTGAGGGAGGAGTTCAGCCATGA
>KGDGIN_04030 TIGR03756 family integrating conjugative element protein
ATGACCCTGAAAAGTCGTCTGCGTGCCTGCGTACTGTTACTGAGTGTGGCATCACTGCCTCTGGCGTCTGCGTCACTGAATACTGCCAGCATTATCGCCAGTGCGGCGGCACCGGACTGTAT
CAGCTGGCGTGTCAGCGGGATTTGTTACTGGCTTTACTGCAGCGCGTCTGGCTGTACGGTCAGAACTTCCGTGAAGGTGACACATTTCATCCCGGAAGTGGTTATTTCCACCTATACGGCAC
CGGGTGGTAATCCTTGGAAAGAGATGTCGCTGGTCAGCCGGACCGCAGGTGGCCCGGAGAATGCCATCACTGACGGCCTGTCCGGGCTGAGTGCCGGCGGGGGAAATCCGGCGGATATG
AAAATACAGGGGAAGCGCCGTTCAGCCATTCGTTTCAAATACGCAGATGCGCTGGGTCATCCGGCCACACAGCTTATCGGCGGGATGGTACCCGGATTCAGCTGTGATTCACCGGTGACAC
CGGCCATGCCCTATTTCCTCAGTACCCTGGACAGCATCGTCTGGCGAACGGGATTACCTGAATCCCTGTATCCAGAGGCACAGGTTCCCGGCAAACGCGAGATTGGCACCCAGGCCGATAA
AAACATGTGGGGGAGCGTTTATCCCCGGAGTGGTTTTGTGATGCAGACCGATGATGACCGGGCCGCGGCGGTGGTGGCCCAGCGGGTGGCCGACATTATCACGCGGACGGGGGAGCCG
CATGTCTACAGGGAGGTTAAGGGCGTGAAACGTGACGGCTACTGGCCCCCGGAGGCGGTGGAGGAAAACACCGGCACCCGAAATCACAAATGGCAGCGACTGACGCCGTCAGTGTCGC
GTTCCTGTGCCGTTTTCCCGGATGGTGAACATCAGTCAGCGGAGAACGGTAATGCCGCCTTTTCACTGTGGCAGCCCTATAGCTGCTGTAAAAAGCGCGGGCAGCGATTTCTTGGCAGTAC
GAATTTTTAA
>KGDGIN_04035 Integrating conjugative element protein
ATGATGAAGATGAAAACAGGTGTGGTGGCACTGAGTGTGATGCTGTGCTGCAGTACTCCGGTGATGGCTGATCTTCTTTCTCTGCCGAAACTGAATGACAGCACGTTCGGTTACGGGAAAT
CCGTTGATGGCGCCGTATCCGACAAACTGTTCTATACGCTGGGTGGCGGCTCGGTGATCTCACAGCCGGCCACGCGCAGCAACATGCAGAAGCTGGGTATGGACCTGGGCTGGAGCAGCG
ATCTGATGTGCGGCAACTTTGACCTGAAGACCACGGTGGGTAATCAGCTTAACGGCGTGACTGAAGGCTTTAAAAATCTGATGGGGGATGTGATTCAGGGGGCGACCGGCGCCGTTGCCA
GTCTGCCGGCGATGGTGATACAGCGTGCCAATCCAGGCCTGTACGACATGATCACCAACGGTGTGCTGCAGGCGAATGTGGCTTTCGACAAGGCGCAACTGAACTGCCAGAACATGGCAA
AACGGATGATGGACTTTTCTGATCGCAGCAAATGGACGCAGGCTGCGATGCTGGAAGAGTACAAGAAGGTGGTGAACAGCGGTGACGGTGATGCGGTGAAAGGGGATGAGGCCGGGC
GTCAGGCCACCGGGAAGGAGGGTAAGAAATGGATTGGCGGACAGCAGCGCGGCGGGGCAGGTCAGCCCGCCATCCGGCCGGTGCATGATCTGACAGCGGCCGGGTTTAACATGATGAA
CAGTCTGCCCGTGACCAGTAGGGCCAGCGTCAGTGCCGGTAACTGTAACGGCAGCGCCTGTGAGATGTTCCGGAATGCAGAAGAGGCCGCTAAGGCGGTGGTAAAGGTGCTGGGTGATC
GTACCATGCGCACCTGTACGGATGCGAAGGCCTGTACCAGCGGGGATAATGAACAGCAACAGGGAACCGTTGCAGGAACGGGGTTTGCGCCGGTGCTGGAAGAGGCGACTAGGGTGAA
CACGGAACAATTGGTCCGTCTGGTGAATGGTCAGGAAAAACCCACTGCAGAGAATCTGGCAAAACTGAAAACCGGCAGTCTGGCTGTATCTGCAGGGGTGATCCATGCCCTGCGTCGTGA
CCCGGACAATATGTCCCTGACGTCACGGCTGGCGGGAGAACTGGCGATGGCGCATACCGTGGAAACGGCTCTGGTGATGCGCAGAATGCTGCTCACCGGGATGTCTGAGCCGTACGCGGC
CGCAGAAGATGCCGCGATTCAGGAAGGCGACCGGCGTATCGCTGCACTGGACAGGGAAATCATGGCGCTGAAGAACGAGATGGAGCTGAAACGCGATCTGGCACGTAACAGCGTGCTG
ACCATCATTGAACGCGACAATGAGCGCGTGGGCAGCAACCCGATGATCCAGCCTGCAGACAATACTGACAGTCGTGTCCGGGCACTGGACATTCCGGACGGGGAGTAA
>KGDGIN_04040 Exported protein

ATGGCGATGACTCAGGGGACAAAAGCGGGGCTGGTTCTGAAGGGCGTTAAGGTGGCCGGCATGTGGCTGTCGGGCCTTATTGCCATGATGATTCTGGCCCTCCTGGTGGCTGATACGGGC
ATGCGGCATACGCAGGAAGTGGAAGCATTACGGCTCTGGCTCCACGAGACACGTTTCGGATGGCTGGGGTGGCGGGTGTGCCTCTACGGTGCGCTGGCATGGGGATGCCGGAAAACCTG
GCGTGCGCCGGGGTTCAGACCGGAATACCGCCGTCCCTTTGCGCGGATTTGTCTGGTCAGTGCCCTGTTTGCGTTACTTTGCGAGTACAGCATTTTTTTCGGACCGGGGGTTGAACTGTGA
>KGDGIN_04045 TraG-N domain-containing protein
GTGACCACGAATAACTATCTGGAACACTTTCTGACCCTGCTGGGCTGGCTGGTGAATAATGGCCTGTGGGATCTGCTGACGGGGACGGGGCTGTTTGCCCTGCCACTGGCCTTCAGGGTTA
TCGGGATCTGGCTGAAAGTGCGGGAAGAGGGGGCGGATGAGGGAAATAAAGGGATGCTCTCACTGCCCCGCATTGAGCATGCCCTGTATGGTGCATTTTTTGTGATGGTGGCCTGTTGTG
TGCCGCTGGTACAGGTGACGCTGGACACGCTGAAATTTGATCGCAGCCGTGCCCAGACCTGCGGTGTGTGGACACCGAAAGCACCCGGTGATACCGGGTACAGCGGGGTGATCAGCAGC
CTGGGTGACAGGACAGCGGCCGCCCCGGTGTGGTGGGTGGTGGTGCATAAACTCTCCAAAGGTGTGACGCAGGCTGCTGTGGCTTCCATTCCCTGCCGGCCGGATATACGGCAACTGCGT
TTTGAGGTGCAGCGGACATTCATTGCGAACCGGGCGCTGGCCGACGAGCTGCAGGATTTCACCAACGACTGTTACTCGCTGGCGCTGTATCAGTGGAAGCAGCGTGACCAGGGGATGAC
GAAGGACAGGAAGCTGCTTTCGGATATCAGCTGGATTGGCAGCAGTACGTTCATGCAGGGTGACTACGGAACACTGCAGTCGAAAATGCCACGTGCGGCATTCCCGTGGAATAGCGCCCG
TGACAGCGGCCGTCCGGATACGGGACGGGGTGGTTATCCCACATGCCGTGAATGGTGGAGTGACAGTAGTGTCGGGCTGAAGAAGCGGGTGACAGAGGAAGTCGATGAAGGACTGTGG
TTGCGTATTTCGGCGGCAATGAAAATGCTGGGGAACGCGCAGGCGAACTACCAGGAGGAAATCATCAAACGTCTGGTGAGTCCGGCGAACCTGACCCTGTCACAGGGTGGTGAGGTGTA
CCGCGGCTATGGCGGTAATGCAGATATGACGGCGGCGAATGCGGTCACGCGTGCAGCAGGCACGGCGGGGTCTGCCGTTGCGAGTCTGGCAGCGTTTCCGGCGTTTGATGCGATGCGGC
AGGCGCTGCCGATGGTGCAGAGTATTCTGCTGATGGCGGTGTATATCATGCTTCCGTTGATTCTGGCGTTTGCGGCGTATGAATTCAGCACGCTGATCACACTGACGTTTGTGGTGTTTGCGC
TGAACTTCCTGACGTTCTGGTGGGAGCTGGCGCGGTGGCTTGACAGCTGGATGCTGACGGCCTTGTACAGTTCGGACACGCATTCCCGAGTGAACATGATGGGGCTGCAGAACACGTCGG
ATGATATCATCATGAATTTTGTGATGGGCACGATGTTTATTATATTGCCAGCGGTGTGGCTTGGGGCTTTATCCTGGGCGGGTATTAGCGCAGGTGTTGCTATTGCTAATGCAGCCAGAGATGG
AACAAGTGCAGCACATCAAGCAGCTTCAAAGGCAGGAGATCTTGTTGGGAAAGTAGGAAATTCTTTAGGTAAAAATCAATAA
>KGDGIN_04050 hypothetical protein
ATGAACAATGAAACAGAAAAAGCAATGTGGAGTTGCAAAACATCTGATACCCCATGGACTTTCAGCTCTCTGCTGAAAAAACTTTTGGCTGTTGAGTTGTCAAATTCCATAAAATTGACAAA
TTCACCAGAAAAAAATGATGAATTACCTGCCTTTTTAGACTCTCGCCCCTATTGGGAATATCAACAATGGGAAGAACAAAAATAA
>KGDGIN_04055 Lipoprotein
ATGAAAATATCAAAACTGGTTGTTATGGCTACAGTATGTTCCACGTTGAGTGCATGCTCTGTGGAAGAGATGGCTGCTGTGAACAAGAAAATCAGTGACGGTGCATTTGCTGTGACGAAAGC
GCTCAACGGGGGAACGGGCTACGCGGATAATGGTATGCCGATCCTTAACAAGCAATTGCCGCCGGAGCAGGATAAACGCGAAAATATTATTGATGTCCCTGTTGATGTGGATACCGCAGCA
GGCCGACTGAAACGCTATTACAAGTTTTACTCAAATGATGAGGTGAATGCGATACGCAATAACGGCACAACTGACGGAAAATGGCAGGCAAGTGCGATTATTGACAATGGGCATATCTGGG
ATGCGATGCCGGGCAGTTACTACAAAATGGGAGGAAACTGGCGTAAAGGCAGCTTTGATGATCACCTGACGATTGAGGTGGAAAAGAACGGTAAGGGAAGCCGGGTCTATATCACCTATG
CTTCAGCTTCTCAGGCGCATTTGCAGGCAAGTTCTCTTGAGCCTCTGTTTAAGCGAGTGAAGGATGTGGCTGAAGGAAAGGTTCGTTGA
>KGDGIN_04060 hypothetical protein
ATGAAACCGAATATCCATCCTGAATACCGTACTGTGGTGTTCCACGATACCAGTATCGATGAGTACTATAAAATTGGTTCGACCATCAAGACTGAACGTGTGATTGAGCTTGATGGTGTCACCT
ATCCGTACGTAACAATTGATGTTTCCTCTAAGTCGCATCCTTTCTACACTGGCAAACTGAGAACTGTTGCTTCAGAAGGTAACGTGGCGCGCTTTAACCAGCGTTTCGGTCGTTTTGTTGACA
CCAAAAAAGGCGCATGA
>KGDGIN_04065 Transposase
ATGAACAGTACCCGGAAAGAGAAATACAGTGCTTTCTGTCGTTTACTCAACGAAACACTTAAATATGAATTACGTCTTATACTCCCTCCGGGACATCAGGCAGTAATTCCTCTGCTTGAATCTC
CACGAGGAGAAATAAGCCGGGATACCATTGAGAAAATGCGCGACATACTCACACCGGATGTGACACACCGAATAAAAGAATCCATCAATGCCTGGACAGAAGATGAGTTAAGTTATCTGGA
TTGTACTGTTGATGTTGAGTATGTGAAGGAACAGAAACGAAAATTATTTGCAATGCTGGACTGCGAACAATAA
>KGDGIN_04070 Transmembrane protein
ATGGATTTTGCTGTTATATTTTTCTGGAGCTTTGCATGTGTTGTTATATTTCTGTGTCTGAAATCATCAATAAACAACCAGGAAAAAATGCAGTCATTATTATTTATATTTTTGTTGCTGACAGGTG
GGTATCTTTCATCCCATATCTTTAATACAGGTTCAGGTAAATGGTTATTTATAACAATTGCCATCACATTTCTGTTAAACACTGCGCTTATTTTTCTTTTTATATTTACAAAAGCATATTTCTTTTCGC
AACACGTAAATAAGATGCGAGAAAAAGCAAAACAAACAAATTCCTTAGACTTTATAAATTGCCTGATAAAACTTCATAAAAAAATATCCGGTATACGTATTATACGCACCTTCAGAAAACACAGT
GGAAATCTGTTACAATATTTTCAATGTAAATCCTGTTATTGGAAAGAAACTTTATTTAAAAACACTGAGTAATCGTCATATCCGGTTCACAGTAAAAAACATAATCCTGCTACCTGCATTAAATGA
TGATTTTATATGCACACTTGAATCTTTTTATAATAATAGTGATGAAACAAAAGATATTATTGATAACTACATCCGAAAAATACAGGGTAATCAACAATTGCCGTGGCTAATAAACAATGCAGTACC
CACTGACACAAAAGAAAAGTGA
>KGDGIN_04075 STAXI RNA
TGTCATCTCAGGATGACCAGCCAATGTTCCGGCAGTCTGAGAGGAGCGCCTTCGGGTGACTGCAATATCCCATGAGCCTGGAAGAGGATGCCTATATGGCGGCAACATCCGTTTAGCCTCTG
GAAACGGATGTCACCAGTAGCTGGTGACTGAGGTACGT
>KGDGIN_04080 EFASI RNA
TCGTTCTGATGTCGGAAGGCGTCATCGTAAGATGACGGGGAGTAAAAAATCGCCCTGGTGCACAGGTAGCGAACTCCTGTAAAAACTCAACATGTGCAAGCTCTGCTACATCGACATCAAA
CG
>KGDGIN_04085 DNA-binding protein
ATGTCCCGTAAATCGTCACGTTCCAAAGTTGAAGTCTTTCGCAAGTCATTCATCTCACTTGCCCGTGATTGTGGTGGCAGCTACGCAACAACCGCTGACAGAATGCGAATTGCCAGATACTTT
TTGAATTATTTGCAAGTAAACGGTATCAAACTTCGTCAGACGGGCAGTATTAAGACGAGGCATATTTCTGGTTATCTTCTCAGCAGGAAAGAGCAGGGGATCTCTCACCGCACAATTCAGAA
TGAACGGGCAGTTATGCGCGCAATTCTGGAGAAAGATGGTCGCCTTAAAATAGCGGATCCGTCCAATCCTTTATTGAGCAATGAAGCTCTTGGACTCAGGGATACATGCCGTGATGGTAAAA
AGATACCACTCTCACAGGATGAGTTTAATAAAGCCTTCAGTAAGGTTGAAAAAAATAATCCCGGTGTTGCGGCAGTAATGCAACTTTCTTATGTTCTTGGACTGAGGACAAAAGAAGCTGTT
CAGTCTTGTAAATCAGTTAACTCGTGGCTACGTGAACTTGATTCAGGGCATGATTCTCTTCTCGTTGTTTTTGGGACGAAAGGTGGAAGGCCCAGAGATACAACGATCATTAATCGTGATGCA
GTGAAACATGCGCTCATTTATGCAAAAAACATTATGGATAAACAGAATGGTAAACTTATCGATCGCCCCAATATTAAACAGGCTATAAACGTATATCGTTACCATGTAAGAAAGGCTGGTTTATC
TGGAGTGAAGGCACCACATAGTATGCGTTATCATTTTTCGCAGGAAGCAAGGCAGTTTTATCGAAAAAGTGGATATGGAGATAAAGAAATTTACGCAAGGGTTTCCATGGACCTTGGTCATG
GTGACGGAAGAGGGCGTTATGTAAAACAGGTCTACTTTAAAGGCGCTGATGAGAGCTGA
>KGDGIN_04090 Phage-int-SAM-2 domain-containing protein
ATGAAATCGAAATACAAAAAATTAAAAGATGAACTAATCAAAATAGCTAAAGCATGCGCCCCGACACCAGAAGACATACTGGTATATATGGGGAGAGCCAGAAGATTTGCTTCATTTCTTAAA
GAATCCAATATACAGATAAAATCAATAAATAGTATAAAACTACGTCATATTGAGCTTTACTTTCAACAGCGTTATCGAACTGGCGTCCGTTCAAAAATACTCCGAGAAGAATTAGACACAATAAA
GCATATTCTCACTGACTGTGGAAAACGAAACATGATGAAAAATGAACGTCTGACTTATGCAGCACTGAATATTGCAGATGTTCGTCCAATTGTAATCTGCACTTACTGTGGTAATAAGGCACAA
CTCAGAAAAGGTGCACTTATGCCATTTTCATCGACACCCACTACAGAAAACAAATATTACTGGATTTGCAGCCCATGCAATGCATGGGTTGGATGTCATAAAAATTCAGGGAGACCATTAGGT
ACTCCAGCTAAAGAAAATTTGAGAATACTCAGAGCACAAGTGCGTAAACTTTTTGACAGCTATCAGCAAAAAACAAATATATCCCGTAACGAGGCAAACAGATGGCTATCCAGAAAACTAAA
TTGCCGCATCCATGAATGCCATATAGGATACTTTAATGAGAGCATGTGTAATCGTGCATCAGAAATTCTTATTACAGAAATTAATAAGTTCGCGAAAAACACATATCCTCCGGATTCATTTTAA
>KGDGIN_04095 STAXI RNA
TGTCATCTTCGGATGACCAGCCAATGTTCCAGCAGTCTGAGAGGAGCGCCTTCGGGTGACTGCCAGCCAATGTTTCAGCGATCTGAGAGAAACGCCTTCGGGTGATTGCAATATCCTCGTAG
CCTGAAAGGGGATGCCTTTATGGCGGTACATC
>KGDGIN_04100 Transposase
ATGGCGGTACATCCGTTTAGCCTCTGGAAACGGATATCACCACTGGTGATTGAGGTACGTTACTACCAATACCGGATTTATCGGTTACATTTCTTAATTAACTCTGCTGGCTGTATTGTCACCAG
TCAGCATTACCTGACGGATTTTCCAGTCCGTCAGGGCGGAACGTCCGTCTTTTATCCGGAGGTTCCGATGTTTATTCCACTTCTGTTTCGCGCAGAGATGCGCCTGACACCTTTCTTTCAGCG
ACTCAGACAACGTCTGTGTTATCCGGTGAGTCGTGTTCATACACAACATCCCTGGTCTTCTCCACAGTTGCGGCACCTTTACTGCACGCCGTGGTTTGTTAACGCCACCGGCAATCTGGCAGT

CCGTGCCTGCATTGTTTCCATTGACGGTTCTTTGCGACACTGCTTTGAGTTCCGTGAAGGCTCTCACAGCCGTGAATACCGTTTCTGGTTTGAGGCGCTTCCTTTGTCGGTACTGCGTTTTAA
CGCTTTTACCGGTCGAATATCCGATCGCCGTACACTCTCTGCTGCCTCCCGTATGGCCGCGCAGCTTACTTCCTTTCGCTATCGCTTCGGTTTGCGCTGA
>KGDGIN_04105 hypothetical protein
ATGGGACTCGATATTTTTTTCCTTGGGCGTGATCGTGTTTGTGTTACCGACGCAGTGGTATCGGTACCGGAAACGCGGGAAGTGGGGTACTTTCGCAAGGTTAACCCACTGATTGCCTGGTT
TGAGAAGCATTGTGGACCGATTGAAAATGCCGTGGAGCGTCCGGTTTCCAGAACGGAACTGGAGGCACTACTGAGCGATCTTGAATGCCTTACGCCGGAGAACTGCCGGGAATTCTTCCC
CACAACAGAAGGATTCTTTTTTGGCTCTCAGGAGTATGACCAGTATTACTGGAAGGATGTTGAGGACGTGAAGTCGTGGGTCAGACGCACGCTTGATTCGTTTGATTTTGAACGAAAAATA
CTGTTGCTCTGGGCATGGTGGTAA
>KGDGIN_04110 hypothetical protein
ATGCAAATAAGTGCAGAAACTCGCGCTGTTTTACGTCACTACAGACAGATCATTAACGCCAGACGGCGTGAAGATGGCATGAAAGCTCTCAGCACGCCGCAAATCGTTGATGAGATCTGTG
AGTTTATGCTCAGCCAGCCAGCAGTGTATCTGGGGGGGCAGTTCATCATGCAGAAGGCCGGAACAAGATAG
>KGDGIN_04115 Antirestriction protein
ATGAAAACGAAAACTTCTGCACCTGCTGTATATGTTGGTACCTGGGCTAAATACAACAATGGCAGCATAGAAGGATGCTGGTTTAATGATTTAACCGTTTTTGAAACTGAAAGCGATTTTTTA
GCTGCGGCCCGTAAGTTTCATAAGGATGAAAAAGATCCTGAATTTATGTACCAGGATTATGAAAACTTTCCTTCGGGAATGGCTTCAGAATATGGTTTCAACTGGGCTTATGTTGAAGGTTAT
CGTCTTGCCCGTGAAGAAGGCTGTGAAGATGCATGGTATGCATGGGTTTCTAATACCGGTGATACCTGTTTCGAACAGTTCCGGGATGCGTGGCTTGGTGGTCCTTATGACAGTGAACTCGA
GTTCGCCGCGCAGTACGTGGATGAATGCGGTTTTCTGTCAGAGGTACCTGATACTGTTGCACGCTATTTTGATTATGAGGCTTTTGCGCGCGATTTGTTCATGGAACTGGATTTTGTCGACGG
ACACGTTTTCCATCGGTAA
>KGDGIN_04120 hypothetical protein
ATGTATTCGCGTTTTATTACTGGTTACTGGCATCTTCAGACGCAGTCCGCTTTGCTGGCACATCTCTGTCAGCTTGAAGGTGAACTGACGATACTTCGCGCCTGGCAACGTCTCGGAATTACCC
CGGTGCCGGAGGATGAAGATGAGCCTTCACCACTTTACTCAATTTTGTGGGATGTTGGTGAGGCTCTGGGCTGGTTGCTGTATGACCTGGAGATGGAAAATGTTCCTGCACCGGGAACGAT
TTTCCACGAGGTATTCGACAGGGTTATATCTCTGGGGTATGAGACTGAGCCCGGTATCTGGGCTGAAAGTATCAGTACGGGAAAGCGCTACGCTGCAATGCCTGACGAATTTTAA
>KGDGIN_04125 CdiI-2 domain-containing protein
ATGAGACCCGATTTGAATACTTTACCCGGAGATTCCGGCTGCAGCGTCTGGTTCTATGACGGAATGTCTCAACCGCGTCTGCTGGCGGGCAGTATTGCCGGGTTACTGACGGATGTTACGAT
CACATCGAATTACCGTGGTGACGTCACATCTGAAATTCATGACGTGGTGCAGGAGTGGCTTGCGACCGGCAGGGGGAACCTGGCGGATTTAAAGGAAGAACTCTGGTACTACAACCTCTAC
ATAAATCCGTCGGCAGACGAACTTATGAATGCCAACAGGCGGTATGGCCTCGGACATACCACCTGGCTGAAAGGCTTTATCAATAACGCGGCGTGA
>KGDGIN_04130 LPD29 domain-containing protein
ATGAACGTTAAGTCTGTGACTGAGGTTGACGACGCTGTGGTTGCCCGTGTCAGTGAAGTTCTGGAATTCGCATTTCCGGGGCAGAAATTTGAAGTGCTGAAGGTATGCGATTCCGGCGTTT
ACAACATGATTAATGTGTACTGGCTGGACGGCCCGACAGAGGCTGAAGTGCGGTTTATCACCCGTGCGTTTGAAGGCAAAAACGGGCTGAGGTTCGTGCACGAATCCCGCGAATTCAGTA
ATGAATTTGTGCAGGAGTGCATTGACCGGCTTCGCCAGAAGTACGGGCAGAGCAACGTTCCGCCAGATGTGACCGTGGAGCGTTACTGGAAAAACGATCTGTGGAAGATTAAAACAGATC
GTTTCCCGGGCAATATTGAGGTTGCCATCAACGAAATGGGGATGGAAACCAGTAAATACCGGAAGGTTGTATAA
>KGDGIN_04135 DUF1281 domain-containing protein
ATGGCTGAATGGTGCAGAAATCGTTTTGAAATCACCGGTAAGTCAGTCTGTCTTGACGTACTGATTCAGTGGATTGAAGGGAATGATGCCCCCCGGTACCGTCATGCGATCCAGCAGAGCAT
ACTGCTGTTTCTTGCAGGCTGTGCGGGGATACTGAAACCGGTAAGAACGGTGACATATCCGCCTTATCCCGGGCTGGTCAGTCACGGTGCCGGCTCAACAACTGTAGCCAGCCAGGCGTTT
GAACAATGGCTGGGATTACTGACCAGGGATGCCTTCCTTGATCACGAGACGGTGAAGACAATAGATCGTCTTTATCATCAGACAGGGACAGGCGCGATCCGGTGGAGTAATCTGCCTGATA
CGGCGAAGGAGATAATCGGTGGTGTGCTTGCACAACAATATGCTGACTGGTTTGGCGTGGCGGATGTGTATGAACGCCCTGATAACGCCTTTCTCTGGGAGTCGTTGTTAACATTTGCGGA
GTATTCGCAACCCTGCGATATGCTGCAGGTGATGCCAACGCGGCTGGCCACGGAGCTGAACGGAAGTGGTGGTCTGCTGAAAGGCACCGGAACGACCCGCAGTTTTTACTGCCGCCAGTT
TGGTGTGGAGTACCCACTGGGGCAGAACGTCCGGTGGAAACGAACCGGGCTGAGTACGCTGATACTGGAGTTTGATACCATCTGGTGTCCGCCTTCAGGTGTACTGGTGGGTGAGATTTC
ATCGGTGTTTGACTGCGAGATACGCCACTGGTACAGCGAACCAAAGAACGGGATCAATGGTTATGACTGCTATGATGGTGGCGAACATATGGACAGTAAAATGGAAGCTGAATGGCCGGAG
TCTGACCACGCAGAGGAGAACGAACAGACTCATCTGTATCTGGTCAGTGATTCGCAGCCAGGTGAACGTGCCACGGCATCAGCTGAGTCCTCAGGGACGACAGCAAAAATAGTGTAA
>KGDGIN_04140 Phage-CRI domain-containing protein
GTGCTAACAAAAAATAAATTGGGGTTTATTTCGATGAAGGTTGTGTTTAATGTAATGGATGGTTTTGATAAGATTTTTTTACCCTTGGAGTTTTCTGGTTTTCATGGCCGGAATGGGTACTGTT
ATTTAAGGGTTCAGATTAAACATGGCTTTATTGTTTTCTCATGCGCTCAATTATTGAACTATTATCGTACGTCAGTAACAAATGCAATTGAGCAGGTCAGAGAGGCTGCGGTTAATGCTCTCTTC
AGGGAGGGGGTATTGTCATACACGCAGCAAAAAGAATTTCTGGATATATTAAAAACATCACAACGAGTAGATAAAGAAATTGATTCGCAATTATGGGATTACATAAATGCTAATTCAATTTGG
TTTGAATACTATAACCATAATGAATCATTGTTTCTGAATGATCGCTTTCATATCGCCTCTTTTGAGGGAAACAGGAATCCAGTGTGGAAGAAAACTTCACTGGAGGATTTAGAGAGAGCATATC
CCGAGTTTGACTTTGTTATTCATAAACATCATCTTGAAAAATGGATTAATGGAGGACTTAGTCCTGAAAATGTAAAAAAGACAATAAAAGAAAAAGGCTGGAGCAACAAGATGTTGGCTGCG
CGCTGGGGGTGCACAGAGGTATGGGTGAGTAAAATCATTAACGATGAAAATAGAAAGGTTCAGTGGAATGATGCTATTAATGGGCTTCCTGTGATAAGTGATAATATGATTTGA
>KGDGIN_04145 hypothetical protein
ATGGAAAAAGTATCAATAAGAAAAATCAACAGGACAGAGCATTTTCCTTCGTTAAATTTTTCTTCTGATACATCCTGGGTTGTTTGGTGGTATGGTTTTTTAAAAAAGAATAAAAGACGAGGT
GAACAACCTAAAGTAGCAATTACCTTTCGGGAAGTTCAGGCCGATGGAACTCTTTGTGATAGATATTATGAGCAAGAATGTCTGTTGACTGATTTGTCTTTTTTTAAAATAGGCAGTGTCTGG
ACAAGAGGAAGGTGTGTTGCTGAAATTCTGATGGAGGAAAAAATATTTAATGTGGATTTCTCTTATGCAAATGGAGGGTGGAAGATTAATTCTCTTTATCAGGCATATAAAGAAGGTGTGTC
ATTACCTTATGATTTTTCAGATTATCAACTGAAATCTCCATATGATAAAAATTGGCTCCTTGAGTTTTTAACAGATGATGCGGATAAGGTGATCATCCATCCGCTTGAGTTTTTTTATCGTTGCTAT
GGACATTCCGATGAGTTGAAGAGGGTTATTCTTACTTATTCAGGAAATGAACTGCGTAACAGGATTTTTTCTGAGGCATACGTGGAGGGAGACAAAATCTTTATATAA
>KGDGIN_04150 hypothetical protein
ATGAATAAATATGATGCTCCTTTTGCTTCTTTCTTTGCCCTTGATGGGTTTACCCGGAAAGTGATTGACAGAATACAACGTCAATTAGATGTATGTGAAGATCGTAAAATATACCTCAAAGTGGC
TCCCTGGTTTAAAGGAAAGGCCAGAATAAAAGTTAAAGGATTTTGGTTGGAAAATAATGTTACTTTCCTTGGGCTTTCTGTTACAGGTGTTTCTTTGCCTGCAGGATATACTATAAATTATGAC
AGAGATAACAGTAACCTGGTTGATGAAAAAGCTGAACAAGGGAGCAGGCAAGCCTGTAGTAATGTTCCAGTTCGGCATATAAAAAATCCGGATGAACCTTTCAGTGTTGATAACGCTTTTTC
TCCTGATTCTGGAGCATCGACTCTTGAACTTGAAGCGTCAGTTTTTGAATTTCTTGACGATGGTTGTGAATTGAGTCCTTTAAGGAGAAAAAAAGCAAAATATATATCCGTTACAAAAAATGA
AAGTGATTTACACTCATTATATTCGGTAAATGAAGTTGTTGATGAACAAAATAGCATAAGCACAGGATATATGTCATTAGCTGATAAAAATGAACCTGAAAGATTGTATATGGAGTCAAAAGGG
ATTGTTCGTGACATGTGGAATGCTATGGTTTATTTGAGGGAGGCTTATTCTGAAAGAATTAAGTCTCTGGACTGGTATACCCCAGAAAGCTCATTCTTAAATACGCAAGAACCTGAACTTGTT
GCAATCCCGGAGTTCAATGAAAGCGAAGTGGAAAATGATAAAATTTCCAAGTCGATTCTTAACTGGCCTTACCTTGATGTTGTGAATAAGAAAGATATAAGAGGTTTTCTGATATCAAGGATG
TTGATTGATGATAATGTGATTTATTTTATGGAAATACAGAAGGAGAGTGTGCAATGA
>KGDGIN_04155 Conserved domain protein
ATGAAAACTAATAAGAAAGACACAAAATGGTACATTTTTTACCGTGAAAATTCAGGCGAGGAAATTTTGCTTGAAATGTCATCATTTAAGGAATGTCTTTCGGCATCCAAAGAATTAATGACC
CCATCAAATTACATGATATGCATAGAAAGAAATGGCGAAAGAATAAAACGTTGGGACAGGGAAATCATAGCAGTCTCTAAAAAATGGATTAACTGCCCACCTGATAACTTCGAAATATTAGGT
GAGCTAATAACCATTAATAGAATTATCAAAAAGTAA
>KGDGIN_04160 hypothetical protein
ATGATTCAACGCATTATCGGCTATATTGCCCGTCTTATTCAGTGCCAGATTTGTCTTGAAATGATGGTGCTTACCCTCCTGTATTGTGAAAGTTGCTGCGCGGCTTCATTAGTAAAATCTCTCCCC
CGGTCATTACCCACGCGAAGACGTCCGCCTTCAATATCACGCATTCTGGCAAGCATAATGGTTTTTGATAACGGTGAAAAACCCAGCTGCAATCCTGCCGGTGTGCTCTTCCTGTTTCTCATCA
GTCATTGTCCCTGTTTCAGTGTAGTTGCCAGCATGCCGCTGACGGTATGCGGCATGCTGTCTGGGTGTTCAGAATGTGACGCTCAGTTGTGCCACTGCCCCGTATGTATTGTCCGTACCGGCC
AGACCGGCAATATCCAGGTGCACCACATCGAACGGCGAGAGACCAATACCGGCAGTGAATGCACTGCCGTTGTTGCTGGCCAGATTCTGGCGGTAACCGGTACGTAA
>KGDGIN_04165 Conjugal transfer protein TraF

ATGATGATGAAAAAGCATATTACCCGTACTCTCATCGCGTCTGCCGTTTTATTTTCCTTTAATTCGACTGCAGCAACGTCATATTTTGAAGCCCGCAATGATGCGATGGGAGGAACGGGGGTT
GCCTCCTCTCACTATGGTGTGGCCCCACTGGCAAACCCGGCACTGCTGACAAAATCCGGTGCAAAAGATGACTTCAGTCTTCTGCTTCCGTCCGTGGGTGCGCAGCTGTCCGATCCGGGTAA
TATCGCCGACAACGCCGACAAAATCAGCGATGACTGGAAAGCCTTCGACCGTGCCGTCGACAGCCACTCCGGGGTACCGCAGACAGCGGCCCGCCTGAAAGAGAGACTGCAGGACTTCC
GGAACACCCATGCCAGCGCACAGGCCGGGGGATCTGCTGTGGCGGCCCTTCCCGGTGATACCCTCGCTGCTGCACTGATGCTGAAAACGTTCGGGACTGTCAGCGTCGACGGTAAAGTCA
GCGATGCCGACCTGAACTATCTGGAAAGCATTGCAGACAGTGGCAGCCAGGATGTGGATAAAAACAGACTGACCTCACAGGCTTTTGCCCGTGCCGCACTGATCACCGATGTGGGCGTTG
CGCTGGCCACTGAACTGGAAACGGCCGGACAAAAATGGTCTCTGGGTTTCACACCAAAATTCCAGCGTGTTGATCTGTTCAACTACAACGTGCTGGTCAAAAATTATGACAGCAGCGCATT
CAGGGGGGACCGCTACCACAACACGAAAAATGGCATCAACGCCGACATCGGTGCCAGCATGGACCTGGATGACAACTGGACGCTGGGTCTGGTCGCCCAGAACCTGATCCCACGCAGCAT
TGAGACCAAAGAAGTAAATGGTGTCACTGAAACCTTCAGGATCCGTCCGCAGGTAACGACCGGCGTATCATGGCACAACGACATGTTCACCACGGCACTGGATGTGGATCTGACCCCGGCC
AGTAGCTTCACATCAGACAGCAAACGTCAGTTTGCCGCCGTGGGTGCTGAGTTTAATGTCTGGAAATGGGCACAGTTACGTACCGGTTACCGCCAGAATCTGGCCAGCAACAACGGCAGTG
CATTCACTGCCGGTATTGGTCTCTCGCCGTTCGATGTGGTGCACCTGGATATTGCCGGTCTGGCCGGTACGGACAATACATACGGGGCAGTGGCACAACTGAGCGTCACATTCTGA
>KGDGIN_04170 Conjugal transfer protein TraE
ATGAAAACTGACAGCACAGGTATTGCCGCCCGTATGATGCTCTCCCTGGACAGGGAGCGAATTTGCGAATGCCTGCTCAGTCATCGCCAGCTGCAGTCCACACCCCTGCAGGTTCGTTATCC
GCAGGGAGTCAGGGATGCCCTTGGTATCATGAGCGAACAGCTTTCTCTTTCCGTCTCTGACCTGACGCGTATCCTGGTCGAAGACGCGTTAAGTGAAATGTTTCTTCCTGCAGATAACATCG
TCCGCCGTCTTCTCAGCAGGATGGAGCACATCATGCAGGCACATGATATCAGCGCAACAACGATGGCTGCATTACTGGCCCCCTGGAATATCCGCCCTGCCGTCTTCCGGGAGCCGGACAGA
CTCACTGACTATCTGACCGGGGAGATTCTTGCAGCACTGGCTGACTGGTTTTATCTCAGTCCGGAATGGCTGAACGGCAGGGTGCATTATCCACTGTATCGTCCGGGAGACTGGCCAGCCAC
ACAGGAGATATTCTGCAGGATTATTTCAGCCCGCGAGAATATGGACATTATTCTCTGGCATGGTTTTCCGTTTGCCGGAACGCATTCCGGAGAATATTGCGGCGTTCTGCTCAGGCAGAAAA
AAGAAATTAATAACACCATTATTTATCCGGTACTTTCGTTATATCCGGTCAGAATGGATATAGAAAAAGAAGGCTGGTTTCAGATGGCCCTAAAAATATCTCCGGATATCCCTGTTCGCGCCGTA
ACACTCACTCCGGCACAGGCAGAATATCTGATAACAGGAAAAATATTACCCACTGCGTTATTCAGGGTTCCGTTATCCCCCTGGTAA
>KGDGIN_04175 Shufflon-specific DNA recombinase
ATGTCCACATCCACTCGTTCCGTAAACAGTGAAATGTTCCTTTTCTTTGTTACCCTTCCACGAACCGGATTGGCACAGAGACATTGCATTTCTGATTCAGTATTCCTACCTTCCTCGGATTTATC
GTGGAAGGCAGATATGTTTCGAAAAATAAAAATTCGCAAAATGACTCTCAACCGGGCGCTGGACAAATACCTGAAGACAGTATCCATCCACAAAAAAGGTCATCTGCAGGAGTTTTATCGC
GTCAATGTGATTAAACGTCACCCTATGGCCGAGCGCTATATGGATGAAATCACCACCGTTGATATCGCCACTTACCGGGATCAGCGTTTGGCTCAAATTAACCCACGAACAGGGCGTCAAATC
ACCGGAAACACCGTCCGTCTTGAACTTGCTCTTTTATCATCATTATTCAATATCGCCAGTGTGGAATGGGGGACATGCCGTATGAATCCTGTTGAACTGGTCCGAAAACCCAAAATCAGTAGT
GGACGCGATCGTCGCCTGACTTCTGGCGAAGAACGCCGACTCTCCCGCTATTTCAGAGATAAAAATCAGCAGCTCTACGTTATCTTCCATCTGGCCCTGGAAACAGCAATGCGACAGGGGG
AAATCCTCACATTACGCTGGGAACACCTCGATTTGCAGCACGGAGTGGCGCATCTTCCTGAAACGAAAAATGGTTTACCACGCGATGTCCCACTCTCCCGGAAAGCTCGCAACTACCTGCAA
ATTCTGCCACAACAGATAAACGGCAATGTGTTCAGTTACACCTCATCCGGTTTCAAAAGCGCCTGGCGTACCGCACTTCTGGACCTGAAAATAGAAAATCTGCATTTTCACGATCTCCGTCAT
GAAGCCATCAGCCGATTTTTCGAACTTGGTACATTGAATGTCATTGAAGTTGCCGCTATTTCAGGCCACCGCTCCCTGAACATGTTAAAACGGTACACGCATCTCCGGGCATATCAGCTGGTC
AGCAAACTCGATGCCAGGCGTAAACAGACCAGCAAAATTTCCCCTTATTTTGTTCCCTACCCTGCAACTGTCAGGTGCAGAAACGGGCTGTTTGTCGTGACACTGCACGATTTCGACCTTGA
AACCCGTGCAGAAACCCGGGAACTGGCAATATCTCATGCCAGCGTATTGTTGCTGCGCACACTGGCACAGGCGGCTCAGCGGGGTGAACGCGTTCCCACGCCAGGCGAGCTACCCGCAAA
CATTGATGAACGGGTCATGATTTGTCCGTTAACAAACTGA
>KGDGIN_04180 hypothetical protein
GTGAAAAAAATTCAGACACGATGACGATAAAGGTTTTATTCTGCTTGAAATTCTGGCAGGCCTGATAGTAATTGGTATCGCCACGCCAATGATTTATTCAGAAATTGAAGACTGGCTGAATGAA
CAACTTTATCAAAGTGCGGCTTTACATGCTGATGAATATAATAATGCAATAAAGAATTATGTGGCTGATAAAACCAGCTCACTTTCCAGTAAAACCATCACGGTAAATGAATTAATTCAGCAGG
GATATCTGAACAACGGTTTTTCCCGCTCCCCCTTTGGACACAGCTATATTACCGGTATCAGAAAAAATAATTTATCCGGTCGTCTGGAAGCACTTACCTGCACAACCGGAGGACAGGATATTAA
AGAAGATGGGTTACGTCGCATAGCGGGCCAGATTAATGGACTCGGTGGTTTTATGCTCCAGAATAATTCCGTTACAGGTGCCTTTGGCGGCTGGACCGATCTGGGAAGTAATTATCAGATTA
CCTGCAACAAGGGGCATATCGCCATGCGAATGGCTGGAAAAGATCTGGAGGAAAGCGATCGGCTTTACCGCTACAGCGTTCAGGGAAGACCCGATTTAAACAGAATGCATACTGGCATTG
ATATGAATAATAACAGCATCACCAATATCAATGAAGCCAGTGGTAAAAACGCCAGATTTTCAGGCGATGTCACATCAAATCGTTGGTTACATCCAAATGGTGGCGGCTTCACCATGACGGACA
GCCAATGGATACGTGCTGTTAACAACAAAGGGATCACAACCGAAGGAGAGCTCAAAGGAGGAAAAGTCAGTGGTGGAACTGTCCGGTCTGAGGGGCGTCTTTCCACGGGTGAGTATTTA
CAGCTGGATAAAACAGCCACCGCAGGTACTTCCTGCTCACCGGACGGACTTGTAGGCCGCGACAGTACAGGGGCGATACTTTCGTGCCAATCCGGTATCTGGACAACGACAAAAATGAACT
TCACTACCAGTACATACAATATCGGGAAAAATACTCGTAATCTGAGCATTGGCGTACATGCGTACTGCTCGTGGACATACCTTAATGGCTCGCCTTTTGGTGGTTTTCAGCAGATATACGCCGA
CCAAAACAAGGTTTGGTATGTGAATAATTATGCGTGGGGAAATTATGAGTCCGGTGGCACAATTACAGTGACATGCCTCAACCTTCCTGGTGCCGGAATCTAA
>KGDGIN_04185 Prepilin peptidase
GTGTCTGAATTACTCCCGATATGCATTATCCTGATTGTCCTTGCGGCAATTCTGTATCTTTCATACAGCGCCGCCACAGTATATTTATCAGAAGTACACAATACCCGCCCACACAACGCTGCGGT
GTGCATCTGTTGTCTTCTGGCTGTCCTGAGCATTTGCCTCAACGGTGCATATGGTGTTGACGCGACAGGACTGTTATTCCTTTCCATTCTGACGGGATTATTAACTGTAATGACGCTGACGGAT
ATTGCTGTCTGTCGGCTTCCCCGGATATTCACGCTTTCTCTTATTATTCTGGGCGCAGCGTTCAGATACAGCCAGGACTCACTGGCTCATGCCCTGCTCAACGCGTCACTGTGGTTCGGCATGA
TATACCTGTTACGACGCATTTTTCTGGCAGCGAAAGGGACGGAAGCGCTTGGCCTTGGTGATGTGTTCCTGATTGCGGGGATCGCAATGTGGACGCAACCGCAACACACTCCACTCATAATC
ACCGCCGCAGCCTCCGGAGCATTTCTGTTTATCCTCATTTTCTGCCGTCGCAGACGCCAGCTGGCGCTGCCGTTTGCCCCGTTTCTCTGCGCGTCCCTGTATGTGCTGACCCTCTTCCCTGATT
CCGTTATCCACACATCGGAGATATTCACGTGA
>KGDGIN_04190 hypothetical protein
ATGAAATGGTTATATCTCTCTGTCCTGCTTTTATTCAGTCACACATCAATCGCATCTGTGATGAATAATAATCATCTCGATTTCTGTTTTATTCAGGCCGGAAAACGTTACCTGATTGCCCCTGAG
CTTCTGAAAACCATTGCACAGCAGGAAAGCGGCCTGGTCGCCACTGCGATAAATCACAATAAAAACAAATCCGGAAAAATCATCAGTACGGATTACGGAATAATGCAGATTAATTCAGTGCA
TATTCCTGAACTAAAAAAACTGGGCGTCATTAAAAACAAGGATTAA
>KGDGIN_04195 hypothetical protein
ATGAACTTCAGATCATGTCTTTATTCTTATTTTGCTTTAAAAAAAGAGAAAGAGGAGGATAAAGGTTTAACCTTACTGGAAGTTGCCGGTGCACTGATTATCATCGGTATTGTGGTATCTCTCG
CAATAAGTCAGATGTCCAGCACAATGTCATCCAGTGATAACCAGCGTGAACTGAAAAACCTGCAGACTATTGCAACAAAAATGAAGCAGTACAAGTTCCAGGGGCAATATACCAGCAGTGAT
TATATCAAAACGCTGACGCAGAGTGGTTCCCTGCCGGCCGATATGATCGCCGGTGGTAACAAGGCCAAAAATGCCTGGGGCGGTGATGTCACCATTAAGGCAACAGCAGATAAGTATGGTT
ATACGATTACCAGCAATAATGTACCGAAAGAAAATTGCGTGGAACTCATTAACAGCCTGCGCAGTTCATCCATGTTTACCAAAATCATGAATACCGCCCCGGCTACTGTGGATCCCGTAACAGT
ATGTAGTAACGACAAAAATAACATCACCCTTGAAACAAACAGCTAA
>KGDGIN_04200 Conjugative transfer inner membrane protein PilR
ATGAAATTTCTGGAAAAACTCAACAGCACCGACATTGAGCTGACAGCGCTGACCATCTGGATGGCCAGAAAAACCTTTGCCCTGAACCGGGTGGAGTTCTACGAAGCCCTGGGCGGCATG
ATACGTGACGGAACGCCGTCCCTGCGTGCACTCGAATTTCTCTGCGAGATCGAAACCGATTTTGGTGAAAAGAAAGGCACATCCGGCATGTATTTTCTGGCAAAAGAATGCATCGATTCCAT
CAAAGCCAGCGGCATGTTATCCGGTGCCCTGTTAACCTGGGTGCCACCGGAGGAAGCCACGTTAATCAGAAACGGGGAAGAGCGCGGAAATATTGCCGACGCCCTTTTCCAGGTGGCCA
GAACCGTTAAAGCCCGGCGGGAAATGACATCCAGCCTCATTGCCGTCTGCCTCTACCCCCTTCTGCTGCTTTCGCTCTGTGTGGTGAATATGTACAACGCCCATACCCGTATCATCCCCATCGT
CAGTGCGTTTGCTTCAGAAGATAAATGGTCATTCCAGATGAAATTGCTTGCCGGCATGTCGGAATTTTTCATCGGGTACGGATTCTGGCTCTGTGGGACCATTTTCGCCCTGACGCTTCTGAT
CCGTTTTTCATTCAGCCGGTATACCGGCCCCGGCCGGAGGATGCTGGACAAGGCTCCCCCCTGGTCACTGTATCAGACCTTACACGGGGTGAATTATCTGTTCAGTATTTCATCCATGCTGCA
GATTAATATTCCTCTTTCGCAGGCGCTGCTCCGAATGGAAAAAGGTGCAGAAAAGAATAAATGGTTAAAGGAGAGAATTCAGGCAATACGAAAACATGTATTATCCGGTCAGAATCTGGCCA
CAGCAATGCGAAATTCCGGTTACGATTTTCCGTCCAGAATATGCATCAATAAACTGCTCCTGAACTCCGAAAGGGAGGACAGTGTGGCAACCATGGCACTCTATGCCGATCAGTGGCTGGAG
GAAGCAAAAAAACGGGTAAAACGAATTGGATTAATTATTACCGGTATATCAGGAGGTTTTGTCTTCTTTTTTATTCTTAACATGATCTCTGCCATCTACAGTATCAGCGACATGCTGAAACAATA
A
>KGDGIN_04205 Flp pilus assembly complex ATPase component

ATGCCAGAGACTATCGAACTGCCTCCTGAAGATATTCGTGATTTCGTCGCAATTCAGCATGACGCTGACGGAAAAATCAGGGTGAAAGTCGTCGAGGCGAAGAAGCACATCACCTCTGTAC
AACGTTACCTGACGCTCTTACGCTCCCGCTATCCGGGAAAAATCAGCAAATTCGATTTTACCACTCTTGACGTTATCGCAACCCTGAACAGCAATAATACGGAACGTGCACCGGGCGTTTTAT
CCGACGGAGGGATCAACACAGTACAAAGAAAAGTTCTTGATTACATCAGAAAAGCCGCTCAACTGGATGCCTCAGATCTGCATATCACACCAGGACGCGACAACACGGACTTCACCTATGT
GGAAGCCCGGGTTCACGGCGAACTGGAAGTGCTCGATATCCTCCGGAAAGAAGAAGGTCTCGAATTACTGGGGGCCACCTATTCCGGCATGACCGACGTCATCAAGGGCACACAGTTTGA
TCCCGGGGTGCCTCAGGATGCGCGTCTTTCAGAGCAGTACCTGAAACTGGCCGGTCTGTTTGGTGCACGCTACTCCCACTATCCCTGCGTGGGCGGCCTCTATGCTGTGCTCCGTCTGATCA
AGGATGACAGTCTGCAAATCCCGACCTTCAGCATGCTGGGCTACCACCCTGAGCAGGAGCGCACCCTGCGCCGTATTCTGCAGCGCCCTGAAGGTATTATCACGCTCTCAGGACCAACAGG
TTCAGGAAAATCCACCACACTGAGAACCGCCTCTGCGGCGTACCTTGAACAGTACGGCTTCAACAATACCGGCGGTATTCTTCTGCCCCGTCGCAGGCTGTTCACCATTGAATCCCCGCCAG
AGGGCCGTATACCCGGTGCCATTCAGACTGCCGTCATGGATTCCGCACAGGGCTGGGTGGACTCCATTAAATCAGCACTGCGTCTTGACCCTGACGGGATCCTGAACGGCGAAATCCGTGA
CCACGATTCCGCCATTACGGCAATAAAAGCCGCCATGACCGGGCATCTGATGCTGACCACCATCCATGCGAACGACCCGATTAACATCCTCGAACGTCTTGAGATGGAGGGGGTACAGGCC
AGGATGATTGCCGACCCGCAACTGTTCATTGGCCTGCTGAGCCAGCGTCTGGTTCAGCTGATATGTCCGCACTGCCGTCGCCCCTGGCATGAGGTGGCTACCGAGCGCACGGATGATGAAC
GGCGCCTGGTGGAAAGCGTCTGCAACCCGGACCAGGTATATCTGCGAAATCATGAAGGCTGCCCGCACTGCTGGCGTGGCGTGACAGGACGAACAGTGATCGCAGAAGTCATCTCCCCGG
ATGCCCGTTTTTTCCAGTTGTACCGGGAAAAGGGGCGTATTGAGGCCAGAACATACTGGCACCGGGAACTGGGCGGAATGACCCGTAATCAGCATCTGCTGGGAAAAATCAACAGCGGAC
AGGTTGATCCGCTGGCGGCCCATTATATATCTCCCGTCGATGAGGACAGCTATACGCTGCTGCACTGA
>KGDGIN_04210 Pilus assembly protein PilP
ATGCACGATAAAACAAAAGCGCTGCTGATTGCAGCCCTGCTGTTACCGGCAATGGCAGTGGCCGGCAACGAAAATGACGCGTCACAGAACAAACAACTGCAGATTGAGCAGATTCAGGAA
GAAACCGCCCTGTATGAAGCGATGCTGGCAAGAGAAAAAATTCGTCATGAATTGCGGACACTGGAACAGGGTATCTCCCCGGTTTCCGGTTCTGTCACGGGACATCAGCCGGAAAATGCG
GCTGGCACCTCCTCCGGTTCACAGGCATTTTCATCCCCCCGTCTGAAAGAAATCTTTGGTATTCAGCCACAGCTTTCAGCACGCGTCATACTCCCCGGAGGAACTGAAACTGATGTCAGGGC
CGGACAGGCATTACCCGGTACACTGTTCATGGTGGAACAAATCACACCGGACAGTGTGATTCTGGAATACAACGGCAACAGGCAGACGCTACGCCCGTGA
>KGDGIN_04215 Pilus assembly protein
TTGAACGGGTCAATGCAGAAAAAAATTCGTCCGGTTTCACTGATATCGGGTAAACAGGCATTCCTTGCGGGCCTTGACTGGAAGAGCATACCTCCCGGTTACCGGAATGCCCGGCGGTTTG
CCCGGTCACAGGGCGCAGAACTGTATCTTTCCTGCCACAACCACGACGGTGAAAACGGTGATGAAATGATGATTGCCCTGCTCAGTCGTCAGGATGCAGATATCCCCCGTAACATTCGCCAG
TGCCTGTCACTGGCAATGTTAATACGCCCGCTACTCAAATCAGGTGGGTATGCCATTTTCATCATCGAGAACGACGACGCTGAACTCACCTGTGCGTTTGTATCGGTGATTAATGGCATTCTGG
TTAACGACGTTACCGGTACCCCGACTGAAATCACGGAAGCCCGCAACACGTTCCTGATGCTCAATGCGGAACCAGAAAACGGCTGGGTACGCTACGAACCTGACGGTTTTTCACCAGACAG
TCGGTGTATCCCGCTACCGCTGTCAAACCTGATCAGCACCAGAAAGCATCCGGCAGAAGCGCGTCTGCTCCCCGTGTCCAGAGGCCCACAGTTAATGACAGCCTTACTCATCATCACGCTGC
TTGGCGCATCGTGGTACGGCTGGCAACGTTATGAAATCTGGCAGGCGGAAAAAGCGGAACGGGAAGAAAGCGCCCGGAAAGCAGCTGAAGCACGTATAACGCCACCATGGACGAGGCA
GCCTGAAAGCGGAGAATTTATCCGGCAATGCAGCACAACATGGAACCAGACGCCACTGTCTGCTGCCGGATGGCGTTATACCCTGGCTGAATGTTCTACTGACGGTAACAGCGGTAATCTG
CGGTCTTCATACACCAATACAGCAGGCACGACAGTGACAGCCTTCATCCGGCGCATCACTGAACTCACAGACACCCGGCCGTTTATTGTTATGCCTGAAGGGAACTCTGGCGGCGTTGCACT
GCCGGTCACATTCCGGCTCCCGGATAATCCTGTCCCGGTTGATTCCCTTCCGGAAACCCGAGACCTGCAGGAACGACTGACCACGCTCGCACAGTCAGTTCAACTGCAGATTGACTGGCAG
GAAGTCAATAACAGCTTCACTGATGAAAACGGAAACATCATCCAGCCACCGTGGAAAGAGTATGACCTGCAGATACAGACGCTACTCCCGGCTAACCAGCTGGCAGAGCGCTTCAGCGAG
CCATCTGTCCGTTTTCTCTCTGTCACAAGACAACTGGAAAATGGTCGTTTCCGTTATCAGTTCACAGGTAAATATTATGCACGATAA
>KGDGIN_04220 Outer membrane lipoprotein BfpB
ATGAAAAAAAGACAACACATGCTCCTGCCACTGACTGCCGTCGCCCTGCTGCTGTCAGGCTGCGGTACGGCCGAACGTATCAGCAACACGGAAAAAGACGCCACCGCCACACTGGAGAAA
GCGCGCCGGCACGCGGGCGAACTCCGTAAATCCCGGCCGGTGGTCACGGAGACCACGCGACAGTGGATCAACCCCTCCCCTGTCAGCGTCAGCCCCGCACACCAGAGCCTGCCGGGCTG
CCCCATCATCATCAACACCCGCCGGGATATCAGCCTGCGGCAGATAGCCCAGCGCATCACCGGAAGCTGCGGTATCCCCGTGACCATCACCGCCGACGTCTGGCAGGCGCTGGCCACCACC
GGCCCCACACAGCAGCTGACAGGCGCAATCCCCGCACCGGACCCGAACGGCATGGTGCCGCTGGCCGCCGTCGGCAGTGGCGCACAGGTCATCCCCCCGGGCGGCGGTGCCGTCCTGCC
GGAACTCAGCGCCACCGGGCTGCGGCAGCTGCTGGATACCGTCGCCTCCCGGCTCGGTATCTCGTACCGTTACGACAGTGACCGGCGCGCCATCACCTTTTACTGGCTGGAGACACGCACC
TTCCCGGTCACCTACATGGACTCTCAGGTGACCTACAACGCCCGGGTGGTCAGCGGCACCGCCTCCTCCTCATCCGGCAGCAGTACCGGCAGTAACGCCGGCACCCTCAGCGGGGAGGCC
AGCAACACCCAGACCACCACGGTCGACATGAAGTCCACCCTCTATGAGGACCTGAAAAATGCCGTCGGTGCCATGCTGACCCCCGGGACCGGCCGGATGGTGCTTGCCACCGGCTTTCTCA
CCGTCACCGACACCCCTCGGGTCCTCGACACCGTCAGCCGTTTTGTGGAGGCACGTAACCGCGAGATGCGACGTCAGGTGGTGCTGAACGTGGAAGTCCTCAGCGTCAGAAAAACCCGCC
GTGACCAGGCCGGCATTGACTGGAATGCCGTCTTCAGCGACGGACGTCTCGGCCTCACGCTGGGAGGGGCATTCAGCAGTGCCGCCGATGACATCATCACCAGTGGCGTCTCCATTGTGG
ACGGTAAGCTCACCGGCTCGAAGGCCTTCCTGAAGGCGCTCTCCAGTCAGGGCACCGTCAGCGTGGTCACACAGAACTCCGCCGTCACCAAAAACATGACGCCGGTGCCCATGCAGGTG
GCCAGCCAGCAGGGCTATATCGAAGGTGTCACCACCGACACCACGGCCAACGTCGGGACCAGCACCTCCCTGAACGCCGCCACCATCACCACCGGCTTCAACATGACCCTGCTGCCGTTCA
TTCAGCCGGATGCGCGCAGCCTGCAGCTGCTCTTCAGCATGAACCTCTCCGACAAACCCACCTTTGAGGTGTTCGAGAGCGGCGGCAGCCGGGCACAGGTACCCACGGTGGACCTGAAA
ACCATCAACCAGACCATCGACCTGAAATCAGGTCAGACCGTCATTCTCAGCGGATTCCGGCAGTCCGGCAGCAAATCCGTGCGCCAGGGCGTCGGCAGCCCGGCATTCTTCGGGCTGGGT
GGCGGCACTGACAGCGGTGACGATGACACCATTCTGGTTGTGCTGATCACACCGCACGTAATCTGA
>KGDGIN_04225 Protein PilM
ATGGGCTGGTCGCTCTCCCTGGTCATGGCCCTGACCGTGGCGCTGACCTGGTTTCACAACAGCAGTACCCACCGGGTCACCGTGTACAGCGAGCAGGCGGAGATGCGTGAACGTGCGTGG
GAGATGGTCCGCCTCATGAACGGCATTAACGACCGGCTGTACACCCATCCGGCTGAGCGCACGGGGGGTGGCACGCTCCCCGTCACCGCCACGGGTTTCCGCCCGGTATACGGTACCCGC
CATATCATCGCCGGACAGCGGGTCTTTGTCTGGCAGGATGACCGCCCGGGGCTTGCAGGTGCGCTGGCAGACATCACCCACAAAACCGCGCTGGCAGGCCGGGTGAAGGGGCGGCGAC
TGCTGAACACCACAGGTCAGGACACCGGTATCAGCGTGCCGTCCGTCATTCCTGACAGCGCATTTGTTTATTTCAACTAG
>KGDGIN_04230 Pilus assembly protein PilL
ATGACACCACTGAAACCACTTCTTATTGTGCCGCTGCTGGCATTTTCTGTTCCACTTTTTGCTGCACCGTCACCTTCTCAGGTCAGTGCACCCTCATCGTCCACGGTCAGCAGCGCTCCGGGA
GCACTGAAATTTATTCCCCGGGACGGTAAAGGGTTATCACTGAAAAAAGCCATGCAGCAACTGATACCGCCGGGATGGACATTTGACATTGCACCGGATGTCAGGCCGCCCCGCACGGTGT
CATGGAAGGGAAATGACCAGTGGAATTACGTTACGGAACGCATGCTGAAAAAATATCAGCTCCGCGCCATCACCGACACGGAGAAGAAGCATGTACGTATTGAGCGGAACACCCTGTTCA
GTCCGTCTCAGGCCACCCCGGGCACCACCAGTCTGCAGGAAAAAACCGCGGAAAAATCAGCAAACATGCCGCAACCGGCGCATGCACGGACCACCTGGACTCTTGCCCGCGGAACCCTG
CTGCGTGCCGGTCTTGAGCAGTGGGTGGCCGCCGTTCCCTGTCAGGCCGGTGGCACCTGGCAGCTGGCCTGGCAGAACCCCGGACTCAATTACAGCATCGATGCGCCGCTGCATTTCACC
GGCGAGTTCCGCCACGCGCTCAGTGAACTCTTCCGTCTCTATCAGGACGCACAGACGCCGCTGTATGCAGCCATCAACAGCACGCAGTGTGTGGTGCACGTCAGCGACAGACCGGGAGAC
TGA
>KGDGIN_04235 DUF1187 domain-containing protein
ATGACAGCAACAACAGACACCACACCGCATAAATACCGGATTGAAGCCACCATCCTGAAGCCTGGTGGTGCACCGGTATCCTGGGTGCGTTTTCAGAACACACCGATGACTGAGCCTGAAT
GCGTCAGGCTGTTTTTAAAAGAAAAAGAGGCCGGTAAATCGTTCAGTGTGAAAGTGAACGTCACCGCATTCTCATGCAGCAGAGTTCAGCCGGAATAA
>KGDGIN_04240 DNA helicase
ATGTCCCTGAATCCCACCGATGAACAGTCAGCCATCATTCACTGGCAGGGGGAAAAACTGGTGGTGAATGCCTTTGCCGGTACAGGCAAAACCAGCACACTGGTGCAGTTCGCCGCAGCC
TGCCCTGAGAGCCGGATGCTGTATCTGGCCTATAACCGCGCGATTCGTGATGAGGCGGAGCGCCGGTTTCCCTTTAATGTGGAGTGCAAAACCTCCCATCAGCTGGCGTATTCGCGGTTCGG
AAAGCATTTCCGGCATCGTCTGGTGCCGGGGCTGTCAGTGACGGATGTAGCGAGAAAACTGAACACCCGCTACTGGACGCTTGCCCGTGTTGCCATGACCGCGCTGAATCAGTTCATCTGT
TCGGCGGACGTGGCGCCCGGAATGCAGCATATGCCGCCTCCCGACGAGATGAAGGGCATGTCGCCACAGGATGCGCTGCGTGCCGTGCAGATCCTCTGGCATGAAATGAGCAATCCGGAT
GGCAATTTTCCGGTCACTCACGACACCTATCTGAAGCTCTATCAGCTTTCCTCACCGGATCTGTCACGCAGGTGGGATACGATCCTTTTTGATGAGGCCCAGGATGCGAATCCGGTGACGTCC
GCACTGGTGCTGGGGCAGCGGTGCAGGGTGGTACTGGTTGGCGACCGTTACCAGCAGATTTATCGTTTTCGTGGTGCCAGCGATGCACTGTCGCATCCGGCACTGAACGGGGCGGACCGT
CTCTGGCTGACGCAAAGTTTTCGTTTTGGTCCTGCCGTGGCCGGGGTGGCCAGCCAGTTACTGAAGCGTGCCGGCGAGAGCCGTGAGGTGACCGGCAGGGGCGGGGATGATGAAGTGC
TGACGGCAGGTGAAATGCGTGCGCGCGTGAAGGCAGGGAAGATGACCGGTCACAGGGCGATACTGAGCCGGACGGTGGCCGGTGTGATTGGCACCGCACTGATGGCGGCAATGCGTGG

CAGGAAGGTTTACTGGGTGGGGGGCATTGAAGGCTACAGAACGGAGGCACTGGAAGATCTTTACTGGTTTCAGATGGATATGCCGGAGCGGATGCACAGTGACAGACTGCGGAAGGAC
TACCGGGGTTTTGACGAGTACCGTCATATTGCGAAGGTGACGAAGGATGTGGAAATGAATCAGTCCATTCGTCTGCTGGAGCTGTGCTTTCCGCTGCCGAAGAAACTCGAACTTCTCAGAA
AGCACACCGTCACGAATGAACAGGAGGCGGATATCACGGTCTCAACGGCACACCGCAGTAAGGGACTGGAGTGGTCACTGGTGGTACTCGATGATGATTTTCAGGATATCACCGACCCGCT
GATAAGCGAGCAGGAGCGGCGGGATGAAACCAATCTGCTCTATGTGGCGGTCACCCGGGCGCGCAGAACGCTGGTTCTCAATGAACTCATGCAGCAGCTGACGGAAGCTGCCGGCGATG
GTGGTGCACCCGGAAACGCGCCGGTGCAGGGGAAGGAAGACGATGGCGTGGAGGGAGAGGATGTTTAA
>KGDGIN_04245 Relaxase
ATGTTTAAGCGGTTCAGACGTATGCTGCCCGGCACAAAGGGCACGGAGCCTGGCGTGGTCGCGCCGGTGATACCGGCAGGGTTTTATGCGCCGCTCACGGCAGAGGCACTGTTATCACCA
GAGCACAGGGAGCTGAGGCTCAGGCAGGTCTGGGATAACAATCCGATGCCCCGTGATGTATACGACAGGATGTGCCTTGAACCACTCAGCCGGCTGCTGCTGAACACCCAGAACGTCCCG
GCAACACGGGAAGGACGGTGGTCCCGTGCCGGTGGATTTGGTGATCTGACGGTACTGTACACCACGTATGCCGTTCGTCTGGCAAGAGGGTACATGTTTCCACCGGATGCGACCCCGGAA
GACCAGGCGGCTCAGGCCGCAGTCTGGCATGCGGTGATTTTCTGGTCAGCGCTGTTTTACCACCTGCCCCTGCTGGCGCACCTTGAGGGGGAACTGCTGAGTGGGCGGGGCTGGCAGCC
AGGGATTTCTGTGCCTGACGAACCTTTCCGTTTCCGGTTCAGGAAGACGGCGCCGCAGGGTACGGAAGCGCAACAACTTGCGGCCCTGACTGCCGGCACGTTGTTACCGGACGGCGCAA
CGGCCTGGCTTGTGACGGCGCCGGGCGCACTGCAGAATCTGGCCGGGGCGCTCTGGCATCAGCATCCCGGGATGGCGCTGATTCGTGATGTGCTGCAGGAAGCGGCAAGGCAGACAGA
GAGTCCGCTGAATACCTGCGCTGTCACTGCGCCGGTTACTGCGGAGGCTTCAGCAGACATCCGTCCTGCTGACCCGGTGGTGACCGGCAGCACGGACAGTGTGGTGGCAGTCACTGAGGT
GCAGCCGGCAGAGGAGGTGAAGACTGAGAGCGTGCAGACAGAGACTGAAGGTGGTGGTGAGTCCGCCGGGGCGTCAGAGGTGCCGTCCGCACAGCAGGAACTGACGGTTCAGACAG
CGCCAGTGGTGACGGCAGATGATGAAGCGGTCAGTGCTGCTCCTGCGGATCAGGACACAGCAATGCTGCTGAGTCTTTTTTCAGCAGATGAGTCCGAAACTGCTGAACGGGATAATGGGG
ATGTGGTGATGCTGGCTGATGAGGAAGAAGCGCAGGATCTCACCGATGGACCGGATGAAGGCGATTCGTCTCCGGGCAGTATTCATGAATCGCCGTATCTGGTGGAGAGTGACGCGTCGG
TTAATCAGTCCGTATCCGGGGAATACCGCGCAACAGAAGTGAATCCGCTTACCGGAGATGATCCTGATTTAAAGGGAGAGACGTTTTTGGACTGGCTTAAAGCAGGGCTGAACAGTGGTG
AGATATCATTTAACCAGAAAGGCGACAAAATACACATTGTTGCAGGACTGGTTTTCCTGCCGGTACCAGGCATTTTCTTTGATTACCTGAAAGCAGTGCAGCTTCCCGGGGACAAACGCCCG
ATTGTTCAGTCATCATTTGAGCGGCTCTGTATCAATAAACGCCGTGATGACCAGCGTTTTTATTTTGCTCGTATTGCTGAGGAACCCGGGAACTGTGCACGTTACAAAACAGTCAAAGGTTAC
CTGGTCAAAGGACGTAGTCTGATGCATGAAAGTAAACTGAAAGACAGTCCTTATCTTTTTATTTCGTGA

Intimin-like adhesion (ila) locus 1.1

>BDIOMP_06235 intimin-like inverse autotransporter SinH
ATGGTGCGAAGGATTCGCATTCTGGTTTTGCTATTAGTGTCTGGAGTCTCAGTTGCGGCTTCCGGCGCAGATAACTCTGTATCTGCAAATGATGAAAGCCACTCCTCCTTGCCTGATTTAGCC
AGCGAATCCGCCAAAAAAGAAGAACAGGAAAATAAAGGAAAGTCCTTTAAAGAACAGGGCGCTGACTACTTTATTAATTCCGCCACCCAGGGCTTTGATAATCTTACCCCGGAAGCGCTGG
AATCCCAGGCGCGTAGCTATTTGCAAAACCAAATCACTTCCTCAGCACAGTCATATCTTGAAGGCGTAATGTCACCTTATGGCAAGATCCGTACCAGCCTCACCGTGGGCGAAGGCGGCGAT
CTGGACGGCAGTTCGCTGGATTATTTTATTCCCTGGTATGACAATCAGAGCACGCTTTTCTTTAGCCAGATTTCGGCTCAGCGTAAGGAAGACCGCACCATTGGTAACTTTGGGCTTGGCGT
GCGCCAGAACGTCGGCAACTGGCTGCTTGGCGGCAACGCATTCTATGACTACGATTTTACGCGCGGTCACCGTCGATTAGGTCTGGGAACCGAAGCCTGGACGGATTACCTGAAATTCTCC
GGCAACTATTACCATCCTCTTTCTGACTGGAAAGACTCCGAAGATTTCGACTTTTATGAAGAGCGACCCGCGCGCGGCTGGGATATCCGCATGGAAAGCTGGCTGCCGTTTTATCCGCAGCT
TGGCGCTAAATTAGTTTACGAACAGTACTACGGCGATGAAGTAGCGCTGTTTGGCACCGATAATCTGCAAAAAGATCCTCATGCCGTCACTTTGGGGCTGGAATATACGCCGGTACCGCTGG
TCACGGTTGGTACGGATTACAAAGCCGGTACTGGCGATAGTAACGATTTTAGCGTTAACGCGACCGTGAACTACCAGATAGGCACGCCGCTGGCGGCGCAACTCGATCCGGAAAACGTTAA
AATTCAGCACTCCTTAATGGGCAGCCGCACCGATTTTGTTGATCGCAATAATTTCATCATTCTCGAATATCGCGAGAAAGATCCGCTGGACGTCACGTTGTGGCTGAAGGCTGACGCCACCAA
CGAACACCCGGAATGCGTGATTGAAGATACGCCTGAGGCCGCGGTGGGGCTGGAGAAATGTAAGTGGACGGTCAACGCGCTGATTAACCACCATTACAAAATTATTTCTGCCTCCTGGCAG
GCGAAAAACAACGCCGCCCGTACGCTGGTGATGCCGGTGGTAAAAGCGAATGCGTTAACCGAAGGCAACAATAACAGCTGGAATCTGGTGCTTCCTGCCTGGGTTAATGCCGATACCGAA
GAGCAGCGCATCGCCCTGAACACCTGGAAAGTACGCATGACGCTGGAAGACGAAAAGGGCAATAAGCAGAACTCCGGCGTGGTGGAAATCACCGTCCAGCAGGATCGCAAAATTGAGCT
GATCGTCGACAACATTGCCGATACCGATCGCAGCGATCACAACCATGAAGCCAGCGCGCTGGCCGATGGCGAAGACGGCGTGGTGATGGATCTCCTGATCACCGATTCCTTTGGCGACTCC
ACGGATCGCAATGGTAACGAACTGGTGGACGATGCCATGACGCCCGTGTTGTACGACAGCAATGATAAAAAAGTCACCCTGGCCCAGACGCCCTGCACCACGGAAACCCCCTGCGTGTTTA
TTGCCAGCCGTGATAAAGAAGCGGGGACGGTGACGCTTTCTAGCACCCTGCCGGGCACTTTCCGCTGGAAGGCGAAAGCAGACGCCTATGGCGACAGTAACTATGTTGATGTCACTTTTAT
CGGCGACAACCTGAGCGCGCTGAATGCGGTGATTTATCAGGTCAAAGCCGCTAACCCTGTCAACCTTATCGGCAAAGAAGATAGGCACCCTACGGTCAATAATGCTTACCGTTTCCTGCTGT
GGCGCGACAAAAATAAAGACGGCGTATTCCAGATGTCCGAGCAGCTCACGGAAGAAGAGATGGCGCTGTACGACTACCAGTGGGAGTTTACCGGGCAGAGTACAAACGGACATACCGGC
GCGCTGGCCAACACCATGAATGAAGATCTTGTCCTGCCAGTAACCAATAAAGAGGCGGCGCAGAAGTTTGCCGCCAATGTGGAAGATGGCGTTCAGGGTTACGGCATACGCGTTACGTATA
GTCAGAAATAG
>BDIOMP_06240 SinI-like outer membrane protein
ATGCAAGCAACAGTAAAACGGCGGCTGACGAAGGTCGCTTTAGCATTAGTAGTTGCCGGGTATTGCGCCGCGCCAGCGGTGGCGGCAAACGGTAATCTGAAATCGGGGCAGTGGCAGAT
TGTCTCTGAACAGACGGGCACCATTCAGGGTACGGTGCCGTGGATTACCCGCGCGGCGGATAAAACTGCGGATACCGACAAAGATCACGTAACGGTAACTATCGATCGCGGCGATCGTACC
GTTGTGACCGAAGGCGATAAGCAGTTCCACGTTGGCGATAAGGTCACGGTGAACTGGGCGATTGGCGATACGGAAGGCGACCTGGATGCCGACAACGCCGCCACGAAACAGACGGTGC
AGTGGATGAGATACAGCGACCAGAATGGTAGCAATCCCGAAGAGATTGGCACCGAAGGTAGCGATACCTATGAAATTCAGGCGAGTGACGCAGACCATTATATCGGTATCAAAATCACGCC
AACCACCACTACCGGTGACCCTGCCGTAGCAACCGAGCTGCTGTTGAAAGACCTGTCGACCGATGCGGGTGGTGGTTCGGATGATGACGAAATTCCTGAAGGTCCGGTGGTCGATGAAAA
CGTCCATGTGGTTATTCATGAGAAAGGTTCGAATACCAACCTGTTGAAGAATACAGGGACGACACTCAAAACGAATACCACCTATCAGGTATTGCTGTGGAGCGATAAGAACGGCAACGGC
ACCTATGATGCCGGTGAAAACGTTACCGACCAGTATGACTACCGCTGGAAATTCGTCGGCACCAGTAAAATTGCAGGTACTGGTACCGGCGGTATTGTCAACGAAAGCTGGAACGATAAAG
ACCTTGTCATCCCGCTTACCAACGCCCAAGCGAAAGAGGCGTTCGAGGGTGCGGAAGGCGGCGTTACCGTGGGTAGCGACGGTGTTCAGGGCTTTGCTTTGTCCATCGACTACAAACGCA
AATAA
>BDIOMP_06245 Outer membrane protein
ATGGACGGGAATCGGCAAGTGAATAAAGTTGTACATTTCTTACTGACATTACTGATCATGTTTGCAGCGTCGATCGCGCCTGCGCAGGCGCTATTGAAGGGCGGAACCTGGCAAGAGCTGA
ACTCAGTCACCGGCGCGGTTAACGGCACCGCGCCGCTGGCCGACGGCGCGATTATTCCCCTGTATCAGGGCAGTACGCTGCTTGACCCTACCAAAACTCACGATATTGAATTCAGCGCCATG
CCGCGCGATTTTAGCGCGGATGCTACCTCAACCTCGATGCGGGCGGTCAATTCCACCGATACCGAAGGCGATCTCTTTAGCGATCCGCCCACCATCGCCTGGGAAAACCGCCAGCCGCCAG
CTATGGGGCTGGTATGGGCCGATGCCGCCACGCCCGATACGCCGCTCTCGCCGCAGCCGGTTCCGAACCTGACCTTTTGCGCCCAAAACCTCGCCGGGCGACAGCTGGTGGCCTGGGCGC
AGGTGGAAGATGAAACCAACGTTCCCGCGCTGTGGCTCTTTACGCGTACCGGGGTGCCGAATTATGCGACCATTCCGCTGTTAAGCCCGAAAGTGGCGCTGAATATCAAACCAGCGGTGAG
CGACCCTGTTTCTGTGAGCGGCGATCATGTCGATGCCAGTTTTGAAGCATCGAAAGTTAAGGTCGGAGAAAGTATTACTCTTACTGTCACCACCAAAGCGTGTGATGGCGAACCTGCGATCA
ATGCGCCGTTTGTGATCCGTCGCGAGGATGCAATAAATCGCCAGGGTGTGGTCAATAACGCTAACCCGGTGCGCGTTGGCAATACCGAGTTAACGACTGCGCAAACAGAATATCATGGTGTA
ACGGACGGGCAGGGCAACGCCACGGTGGTGGTAACGCAAGAGAACGGTCCGGGGGTAAAAACGCGACTTATCGTCTCTTCGCAAAACTATCCCACGCTGACCGACAATGTCGACGTCATT
TTCACCACCATCACCAGTCCAGACAGCGATAAGGCCAGTATGTACGGCCACATGATAGAGAGCGCTACCGCGACGCTGAACGGTATAACCTACACCTTCACCCGCCCGAAGCTGGCGGCGG
AAGCGAGCGGCGCTGACAGCCGCGTCGTCGATACCAACGAAACCTGGGCGCAGTTTACCTGGAGCGGCGCCGATAACCACTGCAACATTCTGCCGGATGCCGAACAACTGGTGGCGTTC
AGGCATGAGCACAGCACACTGGCGACATACACCGGCTGGCCGGCGACGGACGATGCCGAATACTGGTCGTCGACCAAAGATCAGATGAGCGACTACCACGCTGCCGTACATATGAACAGC
GCATCGGTCGTCAGGGCCGCCAACAGCGACACGCTGCTGGTCAGTTGTGTGGATAAAGCCCAGCCTGCGGCGCACCCGCAGATTACTCTCTCCCCACAAGGACCGTATAAAGCGCAGGTG
GGGGAGTCGATTGATCTGACTATGACCGTAGTTGACCGGGACACGCAAAAACCGCTGCCTTACCGCTACATGGAGCTGTTTATCGACCCAGCGAAAAACCGCAAAGGGGAGCACCAGGAC
GCGTGGGATAACCAGCGGGTCACCGTCGATTCCGAAGGCATGAGCGCCTCCTCGCCGGAGCACTATACCGGCGTGACTGACGTCAACGGCCAGGCGCATCTGACGTTAAAACACAACAGC

GGTATGGGGGTCGAAACGCCTATCCGCATCGTGATGCCGGACGATGAAGGCGGCAACGTCGAGCTGCCCTTTAGCGTGATTTTCACCGTGGTCACCAGCCCGGACGTGGAGGGCGCCAAC
ATGTGGGGCCATATGCGCGGCGTGGTGGATGCGGGCAACCTGTACAAACGTCCGCTGCTGGCGGTGGAGGCCAGCCATAAAGACGGCCAGTTCGACGAAAACAACGAACAGTGGGCGA
CATTTAACTCCGTGGCGTCGGCCACCGCGCAGTGCGGTACCGGCCAGGTGCCGGATCAGTCGTCGCTGGCGCATTTGTACAGCGAACATGCGGGCGGACAGATGGAAAGCGAGCACGGC
TGGCCGACCGAAGATTATTTCATCGCCGCCGACAATGACGCCTCCGGGACGGTACACGTCAACCTCGAAAACGGCGATAGCGGCAAATTTACCGATACGCCGAACTACCTGACCTGCTCGG
CCAACGAAATGGTGGCGGTGCTGGACGTCTACTTTAACGACGACCCGGTGATCAAAAACGCCGACGCCAAAGTGGGTGAACAGGTTAAGATGAACATCCATTCGCGCAACGCCCTTAACG
GCATGGCAATCGGCAATACCGACTTCACCATCACCATGGATAACGGCAGGCGGCGCGACGGGCTGACGACCGGTTTTACCGATACCAGCAACGGCGAGATGCAGTTTGACGGCGTCGGCT
ATATTGCCGGGCAGGTTTATCGGGGAGTTACCGACGCCAACGGCGATGCGACCATTATCCTCACCCAGGATAATGGCGTGGGGTTACTGACGCCGTTAAACATCACGCCCGTCGATTCATTA
ATCAGCACGCCGATTTCCCGCAGCGTGAAGTTTACCGTCGCCACCAGCCCGGATACGGCAAAAGCCAAAATGTGGGGCCATATGGCCGACACGATTACCGTGGGCGACTGGACCTTTGAG
CGGCCCAAACTGGCGGGTGAAGTGAGCAATCCGCTGCGTACCCAGGACGAGAGCAATGAGACCTGGGCGCGTGTGGCCCATAGCGACGCGGCGGGCAACCCGGAGGCGGGCGGCTGC
GCGGCGAACCGCCTGCCGCGCATCGACCAGCTTGAGGCGCTGTATAACGCCAACAGCGGCGGCAAAATTAACGGCATTCAGGGCTGGCCAGTCTATCTGAACTACTGGTCGTCCACCTTCC
AGAGCGCCAGCACGTGGAAACTGATTGCGCTGGCGAACGGCAGTGAATTTGCCAACAGCAACGTCTCGATTTACGCCAGTTGCCTGGCCAGCGACAACCCGGTACCAGCCGCCATCACCA
TTGAACCGGTCGATCCATCGCTGTGGTACGACGGCAGCGGCGTGCATGCAGTCAAAGTGAAAAAGGGCGAGACGATGCAGCTCAAGGTGACGGTGAAGGACGCCAGCGGCAACCCGAT
CCCGGAAGCGCCGTTTGTACTCACTCGCGGCGACGGCTACGACCGCCGGGGCGAGAAGTATACCGCTCAGGACGGCGACGATCTGCAAGGCATCGTGACGCCCGTGGTGATCGACGGCG
AATCGCTGGCCTGGACCACCACTAAAATGGGGTCGCTGACCGGCCCTGACGGCACCCGCATTATTAACGTTACCCGCCCGGACACCCACGGCACCCGCACCGCGATTAACGCGACGCTGTA
TGAAAATGCCGCCGTGAGCGCCAGCATCGACACCATCTTTACCGTCGTCACCAGCCCGGATGCCAGCGTCGCCCGCATGTGGGGGCATATGGCGCCGTCGCTGACGGCGGCGGATGGCGC
GGTGTATCAGCGCCCGCTGCTGTACGCTGAGCTGTCCAGTACGGACAATACTACGTCTAAGCAAGAAACCAACGAAACCTGGGCGGTTTTTCATGGGCCAGCCAGTGAAGGGGCAAATCC
GGCCCGCTGCGCGGCGGGCTATTACCCTGCCGTTGAGGCGCTGGATTCGCTCTACAGCAAATACCCGAGCCGAACGATAAATACCGCCCAGGGCTGGCCGGTCTACTATTCTTACTGGAGC
GGATCGAACTCGACGTCATTTTCCAGCGGGGCAAAAGTTGACTTCTATTACGCAGTCGATCTGGCTGATGGTTCGCGTCGGTCAGAAAACAGCTCGCCCAGCACTGCCTGGCAGTACCAAA
TTTGCGCCAGGACGCCGTTGCCGCAGGCCACGCAGATTACCCTCACCTCCCCGCAGGCGATGGATGACGCTATTCAGGCGGTGAAGGCGAAAAACAGCGAGTCGATCCCGCTGCTCATCA
CCACTACCGATGCGATGGGCAACCCGGTGCCTTATGCGACTTTTTCGCTTAAACGCGACGCCGGGACGCCGCGCAACGCCGACTATAAAAAATTTAACACCACCAACGCCACCAACATGAC
GGTAAAGCCGCTCACCGGTGCGCAGCAACAGTTTTACTATGCCACATCGGTTCTGACCGGCGCCACCGGCGCGGACGGTACGCTGACTCTGACGCTTGCCGAACCCGGCGGCATAGGGTT
GAAAAACGTGCTGACCGCGAGTCTGTACGATACGCCGACGGCGACCTCAACGCTGCCGGTGGTGTTCACCGTCCTCACCAGCCCGGACAGCGACAAGGCCAACATGTACGGCCATATGCC
GGAGACCTTCACCGCCAGCAACGGTGCGGAGTTTAAGCGCCCGCTGGTGGTAGGCGAGCCCTCATCGGAGGCGCACACGGATACCTATTTCGAAACCAATGAATACTGGGTCATGGTGAA
TAGTTTTAATACCGGTAATTACGGCGGATGTCCGATGAACCAGATGGCTGCGATGGATGATTTCACGGCGCTGTACAACGATCACCCAAGCGGCAAGGTGGCGACCGATATTGGATTGCCCG
TCGGGAAGCGGTGGTGGGCGGGCGATAGCCTGCTGAAAGGCTCAACCCTTTACTGGCAATATAAAGATCTCAAGACGGGGAAAGACTACAGCATGTCGGAAAACCCGGGTAACTACTACT
TACAACTCTGCCTGACAACATCGCGCAGCGGGCTGAATATCGCGCTCTCTTCCGACGCATGGAACGCCGATAAATCAGCGGCTGTGGCGAAAAAAGGCGAAACGATACCGATGACGGTAAC
GGTGACTAACGACGCCGGGCAGCCGCAGGCCGGGGTGGCCGTACTGTTGACGCGTGAATATGCTTATTCGCGCGGTGAGGTGGATAAGCAATACATTGAACCAGGCGTGATTGGCGAGCC
GGTTCCGTTTACAACTTCGCCGGCGAATATGATGCTGACGCCGGTTGCACCTGCGGCGAGTGCGGCAGTGGCGTTTAATAACCAGACTGGCCTCAGCACAAAATGGTCTGGCTTCACTGGC
GATGACGGTAAGTTGCGGTTTACGTTAACTCAGAATCAATCGCTGGGGTTAAAAACGTCTGTCACGGCGGCGCTTGCGAATCAATTTGATGAGGCGGCGAGCGTGGATGCGATCTTTACGG
TACAAACCAGCCCGGACACGCCGTATGCGTCATACTGGGGTCATATGCCGGATACCGTGCAGGTGAACGGCGTTACGCTGCGCCGCCCATCTCTGAAAGCCGAACTCTCTGCCATGCCCCCG
GGCTCCTGGCCGTTGAATAATGAAATCTGGGGGGCGAACTATTATTATCAGTCCCAGCACGTTGATACATCGTTGACGCATCTTTGCGGATCGCAGGAAAATATAGCCTCGCTGGACGATCTG
AAAGCGTTGCAGAGCGTTATCGGCACGTTGCAATGGCCGACGACATCATCCTGGGATTATGTGTCGCAAGATGAAGGTCAAAGCGATAAGTACTACTGTTCATTTAACGAAACGACCGGCC
AGACAACCTGTACTCGTGACAAATCGTCTACCCCGGGTTTTGGGTCGTGCCGGGTGCCGTAG
>BDIOMP_06250 Lipase
GTGAAAGCGAAAAAGTATGTCGGTTTTTTACTCTGGCTGCTGCTATGCGCCCTCAGTTTAACGCCTTTCTTCGCCCAGGCCGTGCGAAGCAGCGGCGCATGGCAGGAACTCACTGAGCAAA
CTGCGGCTATTAACGGCACTCCGCCGCAGGCCGATAGTGTGACGATCCCGGTGTATCAGGGTAGCGTTCAGTTGCGCTCTGATGCGGCGAATCCTGTAGCTCGTTAG
>BDIOMP_06255 Fibronectin-binding autotransporter adhesin ShdA
TTGAACAGGACCTACAGTATTGTCTGGAGCGCCGTGCGAAATATGTATGTTGTGGCTTCAGAACTGGCGCGAGGCCACAGCAAAGTTAAAGCGCAAGTATGCGCGAGTGAAACCCATTCCC
CAAATAAAAAGTCCGAATATGGACAAATAATAAAAGCAACGCGCAATGTGCTGGCTTGCGCCGTGGCTGCGGCGCTCGGATTTTTCAGCCCGCTGGCCATGGCCGATAATCAGGTGTCGTAT
GCTGATGCGCAGCCCCACGTTCTGGATGGGTCAACGCCGCCGATGACCTATTCAGGAGTTGAGGATGGCGCGGCACTCTATGTCTCGGGTGTTGCCACCGTCGGCTGGCAGCCCACAAAG
GTCACAGGGACGGGGCTCGTCATTGAAACAAGCGGAGGCGGAGCCGATGATCCGAATGGCGGTAAGTATGTTTCCAACGCCATTTCGCTGGATCATTATGCGATCCTTGATCTTACCGGCG
CGCAGATAACCACTACAGGCATTTATACTCAGGGTATTACCGCAGCGGATGGCAGCGAACTGACGTTAACGGACAGTAAGCTGAATATTGGCGGCAATTACGGCATAATGACGCTGTATACG
GGTTCAGCAGCGACACTGGACGGTACCATCGTTGAGGCAGCTAACAGCAGCAGCGCGCAGGTGCAACAAGGCAGTACGCTGAACGTGCTGGACGGTAGCACAATCACCCTGGCGCAAG
GCCAGATAAACGTTGTTGCCGGTACCTCTGCGACAGATGCGGGTTCCACCCTGAACCTGTCTGATAGCTCGGTCAGCTCTACAGGTACGAAGGACACCATTCAGGGCAGCAATAAGGCCGA
TCTTAATCTCACCAACGCGACCATAACGCATACCAATGCCAGCGGCGCGGCGGTGCGGGCCAATAACGCCACCACCCTGGATATTTCAGGCGGCAACATTACCTCTGCCGGTACGGGCGTGT
ACATTGTGGCGTCGGACGCCAGGATCAACGGCGCCACCATCAACGCCGACGGTGTGGCGGTGCAGGCCAATAACGCCACTACCCTGGATATTTCAGGCGGCAACATTACCTCTGCCGGTAC
GGGCGTGTATATTCTGGCGTCGGACGCTAATATCGACGGCGCCACCATCAACGCCGACGGCGACGGTATCTTTATCACCAGTAAAAAGAGAAGCACCTCGTATGAGGATCTCAACGCGCTCA
CGGTCAGCGACGCAAACGTCACCTCAAAAACTGTCGCACTGAACGTTGATGGTAGCACGACCATTAACGACCCCATTAAGCTCACCAACAGCACCTTTACGGCGCCGACAGCGATCAAATT
GGGCAGCAAAGCGGCTATTCAGGCCGAAAACATGACGCTGACCGGCAACATTGTCCAGACTGACACGTCGTCGTCATCGCTCTCTTTAAGCCAGAAGTCCACGTTAACCGGTCGTGTAGAT
GCGCTCTCCAGCACGCTGTCGCTCGACGAAACCAGCCAGTGGAACATGACCGACCCGTCAACCGTCGGCAACCTGACCAACAACGGCGGCATTACGCTGGGCAACGCGAGCGGTTCGAC
AGGTACGCTGCTGACGGTGGACAATACCCTGACTTTGCAGGACGACAGCCAGATAAACGCGACGTTGGATACCGCCAACAGCTCGCCAATTATCAAAGCCGCCAACGTCACGCTGGGCGG
GACGCTGAACCTTAGCTCAACGGCCACCTTTGTCGCGCCCGAAACCGACGAACACTTTGGATCGATCACGCTGATTGATTCACAAACTGCGATCACTACCGATTTTGACAGCGTAACGCTGG
ATGCTGACACCTCCGCTATGCCGGATTATCTGACGATCAACGCGGGCGTTGATGCGAATGATAATACCAACTACGAGTTGTCTACGGGCTTGAGTTGGTACGCGGGCGCAAATTCTGCGCGC
GCTGCGCATGGCACTTTTACCGTTGATGCAGACAGTACCTTTACCGTCACCAGCGAACTGGACGAAACCACGGCGACCTCCAACTGGAACGGCAGCAAGCTAACCAAACAAGGCGACGGC
ACGCTGATCCTCAGCAACACCGGCAACGACTACGGCGACACCGTCATTGACGGCGGTATTCTGGCCGCCAAAGACGCCGCCTCGCTCGGCACTGGCGATGTGACGATTGCAGAAAACGCA
ACGCTTGCGCTGAGCCAGGGCACGCTGGATAACAACGTGACCGGCGAGGGCCAAATTGTCAAAAGCGGTAGCGATGAGCTGATCGTGACCGGCGATAACAATTACTCCGGCGGCACCAC
CATTACCGGCGGTATTCTGGCCGCCAAAGACGCCGCCTCGCTCGGCAGCGGCGACGTGGACATTGCTGAAAACGCAAAGCTTGAGTTGAGCCAGGGCACGCTGGATAACAACGTGACCG
GCGAGGGCCAAATTGTCAAAAGCGGTAGCGATGAGCTGATCGTGACCGGCGCTAATGACTATAGCGGCGGCACCACTATTTCCGGCGGTATGCTGATTGCTGACCATGCAGATTCGCTGGG
CACTGGCGCTGTTGCTAACAGCGGCGTGCTTCAGGTCGGCGAGGGCGAGCTGGAAAATACGCTTTCCGGCACTGGTTCGCTGGTGAAAACCGGTACGGGCGAACTGACGTTAAACGGCG
ATAACGATTACTCCGGCGGCACCACCATTGACGACGGTGTGCTGATTGCTGACAATGCGGATTCACTGGGCAGCGGCGATATCGACAACAGCGGCGTGCTTCAGGTCGGCGAGGGCGAGC
TGAAAAATACGCTTTCCGGCACCGGTTCGCTGGTGAAAATCGGCACGGGCGAGCTGACGTTAAACGGCGATAACGATTACTCCGGCGGCACCACTATTTCCGACGGTACGCTGATTGCTAA
CCATGCGGATTCGCTGGGCACTGGCGCTATCGATAACAGCGGCGTGCTTCAGGTCGGCGAGGGCGAGCTGAAAAATACGCTTTCCGGCACCGGTTCGCTGGTCAAAATCGGCACGGGCG
AGCTGACGTTAAACGGCGATAACGATTACTCCGGCGGCACCACTATTTCCGACGGCACGCTGATTGCTGACCATGCGGATTCGCTGGGCACTGGCGCTATCGACAACAGCGGCGTGCTTAA
GGTCGGCGAGGGCGAGCTGAAAAATACGCTCTCCGGCAGCGGCTCGCTGGTGAAAACCGGTACCGGCGAGCTGACGTTAAGCGGGGACAACAACACTTACTTCGGCGATACCACCATCG
CCGGCGGTACGCTGATTGCCGCCAACGTCAACGCGCTGGGCAGTGGCAATATCGACAACAGCGGTACGCTGATGCTGGATGCCAACGGCGCATTCAAGCTTGCGAACATCACCACCCATAG
CGGCGCCACGACCGCGCTGGCGGCGGGCAGCACCCTTTACGCGAGCCAGCTTACTCAGGAAAACGGCAGTACGTTAAGTATTGACCTCGGCGCGGCCACCGACGACGCCATGATTACCGC
CGACAGCGTCACGCTGGGCGGCACTCTGAATGTCACCGGCATCGGCAGCGTTACAGACAGCTGGACGCCTGAGGCATACACCTACACGTTGATTGACAGCGACAGCGCCATTACGAGCGA
TTTCGACGGTCTGACCGTCGCGGGAATGAATAGAGAAGATGTTGACTTCCTGACTATCGACGGAAAAGTCGATGAAACGGACAACACCAACTATGACCTGACGGCCTCCCTCTCCTGGTAT
GCGGACAGAGACAACGCCACTACCGACGCCCACGGCACCTTTACCCTGAGCGACCCTGACGGCAGCTTTAACGTTGCGGCTACACTGACAGACGTTAATGACACGCTGGATCCGGGTAGC
AGATGGGACGGTAAATCGCTGACCAAAGAAGGCGCAGGCACGCTGATCCTCTCCGGCGACAACGACTACAGCGGCGGCACCACCATCAATGAAGGTACGCTGGTCGCCGCCAGTACGAC

GGCGCTGGGCACCGGTCTGGTAGACAATAACGCCACGCTGGTGCTGGACGCAGACGGTGAAGTCAGCGCGGTGGGCGGCATCACCACTCATAGCGGCGCGACGACCCAACTGGCGCTC
GGCACCTCGTTGGATCTCGGCGACAGCGCGTTAATTCAGCAAGATGGCAGTACGCTGAACGTTGAGCTGAATAGTGACTCAGTGCAACCGCTGATCACCGGCGGCAGCGCCACGCTTGGC
GGCGATTTGGTCGTCAGCGACGCCAGCCTGCAAGCGCGCGCCTCCGATGCGGAGTTCCAGTCCTTTAAACTGATGGATATGGACAGCGATATCAGCGGCGACTTTACCAGCCTGACGATGA
ACCTCACTGACCAGCCGGACTATCTGACCGTAACCGGGACAATCAATCCTGAAGATGCGTCAGAATATCTGCTTACCGAAGGGTTGAGCTGGAATGCGACCGCCACGTCAGCGACGCCAGC
ACACGGCACGTTTACTCTGGGCGCAGGCGACAGTTTTGAAGTCACCAGCGTACTGGGCGATAAAACCGGCAACGGCGACTGGGACGGTAAATCGCTGACCAAACTGGGCGCGGGTAAAT
TGACGCTGAGCGGCGCGAACACCTACACCGGCGATACTAACGTACAGGAAGGCACGCTGTGGCTGGCCGGAGACGGTACGATTGGCGAAGTGGGCAGCCAGCAGGCGGTGAACGTCGC
TTCCGACGCGACCTTTGGCGGCAGCAATGGTACGACCGTTAACGGTAAGGTGACCAATGAAGGGACGCTGGTCTTCGGCGACAGCGAGGAGACAGGCGCTATCTTCACCCTCAATGGCG
ATCTGATCAACATGGGGACAATGACCAGCGGTAGCAGCTCCAGCACGCCTGGCAATACGCTTTATGTTGACGGCGATTATACCGGTAACGGCGGCTCGCTTTACCTGAATACCGTCCTGGGC
GACGATGATTCGGCGACCGATAAGTTAGTCATTACCGGCGATGCCTCCGGCACCACCGATCTGTACATCAACGGCATCGGCGATGGAGCACAGACCACTAACGGTATCGAAGTGGTGGACG
TTGGCGGTGTATCGACCAGCGATGCGTTTGAGCTAAAAAATGAGGTTAACGCCGGTCTGTATACCTACCGTCTGTACTGGAATGAAAGTGACAACGACTGGTATCTGGCGTCAAAAGCACA
GTCTGATGATGACGACAGCGGTGGTGATGACAGTGATGTCACGCCTTCGGATGGCGGTGACGATGGCGGTAATGTCACGCCTCCGGACGATGGCGGCGATGTCGCACCACAGTACCGTGC
GGATATCGGCGCGTACATGGGCAACCAGTGGATGGCGCGCAACCTGCAAATGCAGACCCTCTATGACCGCGAGGGCAGCCAGTATCGTAACGCAGATGGCAGCGTATGGGCGCGCTTCAA
AGCGGGTAAAGCAGAATCAGAGGCTGTCAGCGGCAATATCGATATGGACAGCAACTACTCCCAGTTCCAGTTAGGCGGCGACATTCTGGCCTGGGGTAACGGCCAGCAGAGCGTTACCGT
TGGCGTCATGGCGAGCTACATCAACGCCGATACCGACAGCACCGGTAACCGTGGCGCAGACGGTAGCCAGTTCACCAGTAGCGGCAACGTAGACGGCTACAACCTTGGCGTCTATGCCAC
CTGGTTTGCTGATGCCCAAACGCATAGCGGCGCGTATGTCGATAGCTGGTATCAATACGGTTTCTACAACAACAGCGTAGAGAGCGGTGATGCGGGATCTGAATCTTATGATTCAACCGCTAA
CGCTGTCTCGCTGGAAACTGGTTATCGCTACGATATTGCGCTTAGCAACGGCAATACTGTCAGTCTGACGCCGCAGGCGCAGGTTGTCTGGCAGAATTACTCAGCGGATAGCGTGAAAGATA
ACTACGGCACCCGGATCGATGGTCAGGATGGCGACAGTTGGACAACGCGCCTGGGTCTGCGTGTTGACGGCAAGCTGTACAAAGGCAGCCGTACGGTTATCCAGCCGTTTGCTGAAGCTA
ACTGGCTGCACACCAGCGATGATGTGTCGGTATCGTTTGATGATGCCACGGTGAAACAGGATCTTCCGGCTAACCGTGCGGAGCTGAAAGTGGGTCTGCAGGCAGATATCGATAAGCAGTG
GAGCGTTCGCGCTCAGGTTGCCGGGCAGACTGGCAGCAATGACTTTGGCGATCTGAACGGTAGCCTGAATCTGCGCTATAACTGGTAA

Intimin-like adhesin (ila) locus 1.2

>DLDGEG_06830 intimin-like inverse autotransporter SinH
ATGGTGCGAAGGATTCGCATTCTGGTTTTGCTATTAGTGTCTGGAGTCTCAGTTGCGGCTTCTGGCGCAGATAACTCTGCATCTGCAAATGATGAAAGCCACTCCTCCCTGCCTGATTTAGCC
AGCGAATCCGCGAAAAAAGAAGAACAGGAAAATAAAGGAAAGTCCTTTAAAGAACAGGGCGCTGACTACTTTATTAATTCCGCCACCCAGGGCTTTGATAATCTCACCCCGGAAGCGCTG
GAATCCCAGGCGCGTAGCTATTTGCAAAACCAAATCACTTCCTCAGCACAGTCATATCTTGAAGGTGTAATGTCACCTTATGGCAAGATCCGTACCAGCCTGTCCGTGGGCGAAGGCGGCGA
TCTGGACGGCAGTTCGCTGGATTATTTATTCCCTGGTATGACAACCAGAGCACGCTTTTCTTTAGCCAGATTTCGGCTCAGCGTAAGGAAGACCGCACCATTGGTAACTTTGGGCTTGGCG
TTCGCCAGAACGTCGGCAACTGGCTGCTTGGCGGCAACGCATTCTATGACTACGATTTTACGCGCGGTCACCGTCGATTAGGTCTGGGAACCGAAGCCTGGACGGATTACCTGAAATTCTCC
GGCAACTATTACCATCCTCTTTCTGACTGGAAAGACTCCGAAGATTTCGACTTTTATGAAGAACGACCCGCGCGCGGCTGGGATATCCGCATGGAAAGCTGGCTGCCGTTTTATCCGCAGCT
TGGCGCTAAATTAGTTTACGAACAGTACTACGGCGATGAAGTAGCGCTGTTTGGCACCGATAATCTGCAAAAAGATCCTCATGCCGTCACTTTGGGGCTGGAATATACGCCGGTACCGCTGG
TCACGGTTGGTACGGATTACAAAGCCGGTACTGGCGATAGTAACGACTTTAGCGTTAACGCGACCGTGAACTACCAGATAGGCACGCCGCTGGCGGCGCAGCTCGATCCGGAAAACGTTA
AAATTCAGCACTCCTTAATGGGCAGCCGCACCGATTTTGTCGATCGTAACAATTTCATCATTCTCGAATATCGTGAGAAAGATCCGCTGGACGTCACGTTGTGGCTGAAGGCTGACGCCACCA
ACGAACACCCGGAATGCGTGATTGAAGATACGCCTGAGGCCGCGGTGGGGCTGGAGAAATGTAAGTGGACGGTCAACGCGCTGATTAACCACCATTACAAAATTATTTCTGCCTCCTGGCA
GGCGAAAAACAACGCCGCCCGTACGCTGGTGATGCCGGTGGTAAAAGCTAATGCGTTAACCGAAGGCAACAATAACAGCTGGAATCTGGTGCTTCCTGCCTGGGTTAATGCCGATACCGAA
GAGCAGCGCACCGCCCTGAACACCTGGAAAGTACGCATGACGCTGGAAGATGAAAAGGGCAATAAGCAGAACTCCGGCGTGGTGGAAATCACCGTCCAGCAGGATCGCAAAATTGAGCT
GATCGTCGACAACATTGCCGATACCGATCGCAGCGATCACAGCCATGAAGCCAGCGCGCTGGCCGATGGCGAAGACGGCGTGGTGATGGATCTGCTGATCACCGATTCCTTTGGCGACTCC
ACGGATCGCAATGGTAACGAACTGGTGGACGATGCCATGACGCCCGTGTTGTACGACAGCAATGATAAAAAAGTCACCCTGGCCCAGACGCCCTGCACCACGGAAACCCCCTGCGTGTTTA
TTGCCAGTCGTGACAAAGAAGCGGGGACGGTGACGCTTTCCAGCACCCTGCCGGGGACTTTCCGCTGGAAGGCGAAAGCAGACGCCTATGGCGACAGTAACTACGTTGATGTCACTTTTA
TCGGCGACAACCTGAGCGCGCTGAATGCGGTGATTTATCAGGTCAAAGCCGCCAACCCTGTCAACCTTATCGGCAAAGAAGATAAGCACCCTACGGTCAATAATGCTTACCGTTTCCTGCTG
TGGCGCGACAAAAATAAAGACGGCGTATTCCAGATGTCCGAGCAGCTCACGGAAGAAGAGATGGCGCTGTACGACTACCAGTGGGAGTTTACCGGGCAGAGTACAAACGGACATACCGG
CGCGCTGGCCAACACCATGAATGAAGATCTTGTCCTGCCAGTAACCAATAAAGAGGCGGCGCAGAAGTTTGCCGCCAATGTGGAAGATGGCGTTCAGGGTTACGGCATACGCGTTACGTAT
AGTCAGAAATAG
>DLDGEG_06835 SinI-like outer membrane protein
ATGCAAGCAACAGTAAAACGGCGGCTGACGAAGGTCGCTTTAGCATTAGTAGTTGCCGGGTATTGCGCCGCGCCAGCGGTGGCGGCAAACGGTAATCTGAAATCGGGGCAGTGGCAGAT
TGTCTCTGAACAGACGGGCACCATTCAGGGTACGGTGCCGTGGATTACCCGTGCGGCGGATAAAACTGCGGATACCGACAAAGATCACGTAACGGTAACTATCGATCGCGGCGATCGTAAA
ATTGTGACCGAAGGCGATAAGCAGTTCCACGTTGGCGATAAGGTCACGGTGAACTGGGCGATTGGCGATACGGAAGGCGACCTGGACACCGACAACACGGCAACTAAAGCCACCGTGAA
ATGGGTGAGCTTTAGCGATCAGAATGGCAGCGACCCCAAAGATCTTGGTACTGGTGACAGTTATGAAATCCAGGCCGCTGATGCGGATCGTTATATCGGTATCAAAATCACGCCAACCACTA
CCACCGGTGACCCTGCCGTGGCAACCGAACTGCTGTTGAAAGACCTGTCGACCGATGCGGGCGGTGGTTCGGATGATGACGAAATTCCGGAAGGTCCGGTGGTGGATGAAAACGTCCAT
GTGGTTATTCATGAGAAAGATTCGAATACCAACCTGTTGAAGAATTCAGGGACGACACTCAAAACGAATACTACCTATCAGGTATTGCTGTGGAGCGATAAGAACGGCAACGGCACCTATGA
TGCCGGTGAAAACGTTACCGACCAGTATGACTACCGCTGGAAATTCGTCGGCACCAGTAAAATTGCAGGTACCGGTACCGGCGGTATCGTCAACGAAAACTGGAACGATAAAGACCTTGTC
ATCCCGCTTACCAACGCCGAAGCGAAAGAGGCGTTCGAGGGTGCGGAAGGCGGCGTTACCGTGGGTAGCGACGGTGTCCAGGGCTTTGGTCTGTCCATCGACTACAAACGCAAATAA
>DLDGEG_06840 Outer membrane protein
GTGAATAAAGTTGTACATTTCTTACTGACATTACTGATCATGTTTGCAGTGTCGATCGCACCTGCGCAGGCGCTATTGAAGGGCGGAACCTGGCAAGAGCTGAACTCAGTCACCGGTGCGGT
TAACGGCACCGCGCCGCTGGCCGACGGCGCTATTATCCCCCTTTACCAGGGAAGTACGCTGCTTGACCCGAGCAAAACCCACGATATTGAATTCAGCGCCATGCCGCGCGATTTTAGCGCG
GATGCTACCTCAACCTCGATGCGGGCGGTCAATTCCACCGATACCGAAGGCGATCTCTTTAGCGATCCGCCCACCATCGCCTGGGAAAACCGCCAGCCGCCCGCCATGGGCTTGGTATGGG
CCGATGCCGCCACGCCCGACACGCCGCTCTCGCCGCAGCCGGTTCCGAACCTGACCTTTTGCGCCCAAAACCTCGCCGGGCGACAGCTGGTGGCCTGGGCGCAGGTGGAAGATGAAACC
AACGTTCCCGCGCTGTGGCTCTTTACGAATACCGGGGTGCCGAATTATGCGACCATTCCGCTGTTAAGCCCGAAAGTGGCGCTCAACATTAAACCGGCCACCGACACGCCGGTTACAGTCA
GCGGCGATCACGTTGATTCGGCCTTTGAGGCATCAAAAGTGAAGGTGGGGGAAAGCATCACCCTCACCATCGCCACCAAAGCGTGCGACGGCACGCCCGAAGGCAACGCGCCGTTTGTC
ATCCGCCGTAAAGATGCGGAAAACCGCCAGGGTGTGGTCAATAACGCCAACCCGGTTCGCGTTGGCGATACCGAGCTCACGACCACCCAAACGGAATATCGCGGCGTCACTGACGCCAAC
GGCAACGCCACGGTGGTGGTCACCCAGAAGGAAGGCCCCGGCGTCAAAACGCCGCTGGTTGTCAGCTCGGTCAACTTTCCGGCGCTTACGGCTGAAACCGCCGTCATTTTCACCACCATC
ACCAGTCCAGACAGCGATAAGGCCAGTATGTACGGCCACATGATAGAGAGCGCTACCGCGACGCTGAACGGTATAACCTACACTTTTACCCGCCCGAAGCTGGCGGCGGAAGCGAGCGGC
GCTGACAAAAGCGTCGTCGATACCAACGAAACCTGGGCGCTGTTTACCTGGAGCGGCGCGGATAACCACTGTGACATTCTGCCGGACGCCGAACAGCTGGTGCAGATGCGACATGAGCAC
AGCACGCTGGAAACCTGGACCGGCTGGCCGGTAGCGGGCGATGCTGAATACTGGTCATCGACCAAAAACCAACTGAGCGGCTACCATTCCGCGGTGCATATGAACAGCGCATCGGTCGTC
AGGGCCGCCGACAGCGACACGCTGCTGGTCAGTTGTGTGGATAAAGCCCAGCCTGCGGCGCACCCGCAGATTACTCTCTCCCCACAAGGACCGTATAAAGCGCAGGTGGGGGAGTCGATT
GATCTGACGATGACCGTAGTTGACCGGGACACGCAAAAACCGCTGCCTTACCGCTACATGGAGCTGTTTATCGACCCGGCGAAAAACCGCAAAGGGGAACATCAGGACGCATGGGATAAC
CTGCGGGTCACCGTTGATTCCGAAGGTATGAGCGCCTCGTCGCCGGAGCACTATACCGGCGTGACTGACGTCAACGGCCAGGCGCACCTGACGTTAAAACATAACAGCGGCCTGGGCGTC
GAAACGCCCATCCGCATCGTGATGCCGGACGACGAAGGCGGCAACGTCGAGCTGTCCTTTAGCGTAATTTTCACCGTGGTCACCAGCCCGGACGTGGACGGCGCCAACATGTGGGGCCAT

ATGCGCGGCGTGGTGGACGCGGGCAACCTGTACAAACGTCCGCTGCTGGCGGTGGAGGCCAGCCATAAAGACGGCCAATTCAGCGAAAATAACGAAGAGTGGGCGACGTTTAACTCCGT
GGCGTCGGCCACCGCGCAGTGCGGTGTCGGCCAGGTGCCGGATCAGTCGTCGCTGGCGCATTTGTACAGCGAACATGCGGGCGGACAGATGGAAAGCGAGCACGGCTGGCCGACTGAA
GATTATTTCATCGCCGCCGACAGCGACGCCTCCGGGACGGTACACGTCAACCTCGAAAACGGCGATAGCGGCAAATTTACCGATACGCCCAACTACCTGACCTGCTCGGCCAATGAAATGGT
GGCGGTGCTGGACGTCTACTTCAACGACGACCCGGTGATCAAAAACGCCGACGCCAAAGTGGGTGAACAGGTTAAGATGAACATCCATTCGCGCAACGCCCTTAACGGCATGGCGATCGG
CAATACCGACTTCACCATCACCATGGCGAACGGCAGGCGGCGTGACGGGCTGACGACCGGCTTTACCGATACCAGCAACGGCGAGATGCAGTTTGACGACGTGGGCTACGTTGCCGGGC
AGGTTTATCACGGGATCACCGACGCTAACGGCGATGCGACCATTATCCTCACCCAGAAAAAGGGCGTGGGGCTGCTGACGCCGTTAAATATTGCGCCCGTCGATTCATTAATCAGCACGCCG
GTTTCCCGCAGCGTGAAGTTTACCGTCGCCACCAGCCCGGATACGCCAGCGGCCAAAATGTGGGGCCATATGGCCGACACGATTACCGTGGGCGACTGGACTTTTGAGCGGCCCAAACTG
GCGGGTGAAGTGAGCAACCCGCTGCGTACCCAGGACGAGAGCAATGAGACCTGGGCGCGCGTGGCCCATAGCGACGCGGTGGGCAACCCGGATGCGGGCGGCTGCGCGGCGAACCGC
CTGCCGCGCATCGACCAGCTTGAGGCGCTGTATAACGCCAACAGCGGCGGCAAGATTCACAGCATTCAGGGCTGGCCGACCTATCTTAACTACTGGTCGTCCACCTACCAGAGCGCCACCA
CCTGGAAACTGATCGCGCTGACGAACGGCAGTGAATTTGCCAACAGCAACGTCTCGATTTACGCCAGCTGCCTGGCCAGCGATAACCCGGTGGCGGCGTCCATCACCATTGAACCGGTCAA
TCCGTCGCAGTGGTACGACGGCAGCGACGTCCACGCGGTCAAAGTGAAAAAGGGCGAGACGATGCAGCTCAAGGTGACGGTGAAGGACGCCAGCGGCAACCCGATCCCGGAAGCGCC
GTTTGTGCTCACCCGCGGCGACGGTTACGATCGCCGGGGCGAAAAGTATACCGCTCAGGACGGTGACGATCTGCAAGGCATCGTGACGCCCGTGGTGATCGACGGCGAATCGCTGGCATG
GACCACCACCAAAATGGGGTCGCAGACCGGCACTGACGGCACCCGTATCATCAGCGTCACCCGCCCGGACACCCACGGCACCCGCACCGCGATTAACGCGACGCTGTATGAAAATGCCGC
CGTGAGTGCCAGCATCGACACTATCTTTACCGTCGTCACCAGCCCGGATGTCAGCGTTGCCCGCATGTGGGGGCATATGGCGCCATCGCTGACGGCGGCGGATGGCGCGGTGTACCAGCGC
CCGCTGCTGTACGCTGAGCTGTCCAGTACGGACAATACTGCGTCTAAGCAAGAAACCAACGAAGCCTGGGCGGTTTTTCATGGGCCAGCCAGTGAAGGGGCCAATCCGGCCCGTTGCGCG
GCGGGCTATTACCCTGCCGTTGAGGCGCTGGATTCGCTCTACAGCAAATACCCGAGCCGAACGATAAATACCGCCCAGGGCTGGCCGGTCTACTATTCTTACTGGAGCGGATCGAACTCGAC
GTCATTTTCCAGCGGGGCAAAACTTGACTTCTATTACGCAGTCGATCTGGCTGATGGTTCGCGTCGGTCAGAAAACAGCGCGACCAGCACTGCCTGGCAGTATCAAATTTGCGCCACGACG
CCGCTGCCGCAGGCCACGCAGATTACCCTCACCTCCCCGCAGGCGATGGATGACGCTATTCAGGCGGTGAAGGCGAAAAACAGCGAGTCGATCCCGCTACTCATCACCACTACCGATGCGA
TGGGCAACCCGGTGCCTTATGCGACTTTTTCGCTTAAACGTGACGCCGGGAAGGCGCGCAACCCTGACTATAATAAATTTGTAGCCACTAACGGCACCAACATGACGGTGACGCCGCTCAC
CGGTGCGCAGCAACAGTTTTACTATGCCACATCGGTTCTGACCGGCGCCACCGGCGCGGATGGTACGCTGGCCCTGACGCTTGCCGAACCCGGCGGCATAGGGTTGAAAAACCAGCTGAC
CGCGAATCTGAACGATACGCCGACGGCGACCTCATCGCTGCCGGTGGTGTTCACCGTCCTCACCAGCCCGGACAGCGACAAGGCCAACATGTACGGCCATATGCCGGAGACCTTCACCGCC
AGCAACGGTGCGGAGTTTAAGCGCCCGCTGGTGGCAGGCGAGCCCTCATCGGAGGCGCACACGGATACCTATTTCGAAACCAATGAAAACTGGATCATGGTGAATAGTTTTAATACCGGCA
ATTACGGCGGGTGTCCGATGAACCAGATGGCTGCGATAGATGATTTCACTGCGCTGTACAACGATCACCCAAGCGGCAAGGTGGCGACCGATATTGGATTGCCTGTCGGGAAGCGGTGGT
GGGCGGGGGATAGCCTGCTGAAAGGCTCAACCCTTTACTGGCAGTATAAAGATCTCAAGACGGGGAAAAACTACAGCATGTCGGAAAACCCGGGTAACTACTACTTACAACTCTGCCTGAC
AACATCGCGCAGCGGGCTGAATATCGCGCTCTCTTCCGACGCATGGAACGCCGATAAATCAGCGGCCATGGCGAAAAAAGGCGAAACGATACCGATGACGGTAACGGTGACTAACGACGC
CGGGCAGCCGCAGGCCGGGGTGGCCGTACTGTTGACGCGTGAATACTCTTATTCGCGCGGTGAGGTGGATAAGCAATACATTGAACCAGGCGTGATTGGCGAGCCGGTTCCGTTTACAAC
TTCGCCTGCGAATATGATGCTGACGCCGGTTGCACCTGCGGGTACGGCAGTGGCGTTTAATAACCAGAATGGCCTCAGCACAAAATGGTCTGGCTTCACCGGCGATGACGGTAAGTTGCGG
TTTACGTTAACTCAGGATAAATCGCTGGGGTTAAAAACGTCTGTCACGGCGGCGCTTGCGAATCAATTTGATGAGGCGGCGAGCGTAGATGCAATCTTTACGGTACAAACCAGCCCGGATAC
GCCGTATGCGTCATACTGGGGTCATATGCCGGATACCGTGCAGGTGAACGGCGTTACGCTGCGCCGCCCGTACCTGAAAGCCGAGCTCTCTGCCGCGCCCCGGGATACCTGGCCGTTTAATA
ATGAATTCTGGGGGACGAACTATTATTATCAGTCCGAACACGTGGAAACGTCGTTGACGCATCTTTGCGGATCGCAGGAAAATATAGCCTCGCTGGACGATCTGAAAGCGTTGCAGAGCGTT
ATCGGCACGTTGCAATGGCCGACGACATCATCCTGGGACTATGTGTCGCAAGATGAAGGTCAAAGCAATAAGTACTACTGTTCATTTAACGAAACGACCGGCCAGACAACCTGTACTCGTGA
AAAAGCGACGACCTCGGGCTTAGGCTCGTGCCGGGTGCCGTAG
>DLDGEG_06845 hypothetical protein
GTGAAATCGAAAAAGTGTGTCGGTTTTTTACCCTGGCTGCTGCTATGCGCCCTCAGTTTAACGCCCTTCTTCGCGCAGGCCGTGCGAAGCAGCGGCGCATGGCAGGAACTCACTGAGCAAA
CTGCGGCTATTAATGGCACTCCGCCTCAGGCCGACAGTGTAACGATCCCGGTATATCAGGGCAGCGTGCAGTTGCGCTCTGATGCGGCGAATCCTGTTGACTACTCCGCGAAGCCCAGCCAG
TTCAGCACCAGTGATGCTGGCAGCGCGCTGACCTTAACCAATCCGCACGACACCGAAGGCGATATCTTCGCCGTGCCGCCGCTGGTCTGGCAGGCTGAACGCGCGCCCACCGTCACGCTG
GTGTGGGCCGACGCCGCCACGCCGGACACGCCGCTTGATCCACAACCCGTCAGCAATATAAGTTTCTGCGCGCAGAATCTGGCGGGCCGCCATCTGGTGGTCTGGTCGCAACTGGATACCT
CAACCGCCATACCGCCGCTGTGGTTGCTTACCCGCACCGGCGTGCCTTATAACACGGCGGTCGAAGTGCTTGATCAAAAGTTTGCCGTGGATATCGCGCCCGCCGTGGGCGACCCGGTAAC
GCTCGCCGCCGATCATCTGGATGAGTCGCTCAACGCCGCGAAGGTCAAAGCGGGCGAAAGCATCATGCTGACGGTAACGACCAAAGACTGCGGCGGCGAACCGGAGGGCAACGTTGATT
TCGTGATTACCCGCGGCGACGCGCAAAACCGCCAGGGCGTGGTAAACAATGCCGCGCCGGTGCGCGTCGGCAACACCGAACTGACCACCACCGCCACGGAATATCACGGTACGACCAAC
GCCGAGGGCGTGGCGACCGTGGTCGTCACCCAGGCGAACGGCCCGGGCGTTAAAACGCCGCTGATAGCGCACCCGTCGAGCGCGCCAGCGCTGAAAGCCAGCGTGGACGTGATTTTTA
CCACCCTCACCAGCCCGGACAGCAGCAGCGCCAACATGTACGGTCATATGGCAGAAGACGTCCACCGCCACGGTGGACGGCGCAAGCTACACCTTTTACCGCCCCAAACTGGCGGCGGAA
ACCGACGGTGA
>DLDGEG_06850 Intimin-like inverse autotransporter protein SinH
GTGGACGGCGCAAGCTACACCTTTTACCGCCCCAAACTGGCGGCGGAAACCGACGGTGAAGACCGCACCGCCAGCATCAACAATGAAACCTGGGCGCAGTTCACCTGGGGCCACGCCGA
CGCGCACTGCGATATTCTGCCGGATGCGCGCCAACTGGAAGGGTTAAAAATCGAGCGCGGCGATCTGGCCACCACCCTCGGCTGGCCGGTAGGGCTGACATCCGGTGATGAAGAGTACTG
GTCCTCCTCCCAGGGATCGATTGCCACTGACCATATCAGTATTGATATGCGCAGCCGCAGCCTCACGCAGATGCCGGACGCCACGCAATCGCTGGTTAGCTGCGTGGATAAAGCCCCGCCTG
CAGTCACGCCGAAATTAGTGATTAGCGCCGATAATGTTGATTCAACGGTCAACGCGGCGAAAGTGAAGGTCGGTGATGACATCAACATGAAGATCACCGTCACCGACAGCGCCACCAATAA
ACCGCTGCCTTATCGCTACTTTAATGTCTACCTGGGCGACGAGCAGAATCGTCAGAATCAAAAGAACGCCGATATTGATAGCGGGCATCAGTGGACGGATGATCCGGTAATTATCACCAATCT
GGAAGGCAGCGACGGTCACTATCACGGCGTCACCGACGCCAACGGTCAGTTCTCGCTGGTGTTGACTCAGGATAAGGGTGCGGGCGTATTAACCCCGGTGCGCGTGGTGCTGTTCGACG
GCACGGAAGCGACGCAGAACGTGATTTTTACCGTCGTCACCAGCCCGGACGTGGCGCAGGCGCGCATGTGGGGCCATATGCAGGGCGTGGTGGAAGCGGGCAATATCTACAAACGCCCG
CTGCTGGCAGAAGAGGCCGCGCAGGATACCGGTTCCGAATTTGAGAATAACGAATACTGGGCGACGTTTAACTCGGTAACGGCGGCGACGAACCAGTGCGGTACCGGCCAGGTGCCGGG
GCAACTGCTATTGGATACGCTGTATCAGGCCCATTCCGGTAACGCGATGGAAACGACCTACGGCTGGCCGACGCAGAAACACAGCTATATCGCGGCGGATACTGATGGTTCCACCACCGCGC
ACGTCAACCTGGCGACCGGCGCGGACAGCATGTTCAGCGGCGCCGAACCGAACTATCTCTCCTGCTCCGGCAATGAGTTGGTGACCAGTCTGGACGTTTACTTTAATGGCGATGAGTCGTT
ACGTAATGCGGTAGCCAAAGTCGGCGAACAAATCACGATGAACGTCCATTCCGTTAATGCGCTGAACGGGTTAAGTGTGCCCAACGCCAGCTTTACCGTCACCATGTCGCACGGCAAAAAT
CGCGACAACGCCACCACGGGCTTTACCGATCCCAGCGACGGAACGCTGGTGATGGGCGGAACGCCTTTCGGCTCTTCCCAGGCGTCGATGACGTACCAGGGCATGACCGATGCCGAGGG
CAACGCCACGCTGGTGATTGAACAGCCGCAGGGCGTGGGCTTGCTGACGCCGCTGAGCGTCCTGCCGGTCAACTCACTGATTACCACGCCCATCAACCGCAGCGTGAAATTTACCGTTCCC
ACCAGCCCCGATACGCCGGAGGCGCAGATGTGGGGCCATATGGCGGACACGCTCACCGTGGGTGGTATGGAATTCCAGCGGCCAAAGCTGGCGGCGGAAGCGACTGCCGCCACCCGTAC
CCAGGAGCAGGATAACGAAACCTGGGCGCGCGTCAGCCACGCCGACGCGCTGAATAACCCCAACGCCGGGGGCTGCGAGGCCGGCCATCTGCCGCGCACCGATCAGCTTGCCGCACTGT
ATGCCTCCAGCGACGGCAATAAAATCCACACCGTATCCGGCTGGCCGACGACGTATGACTACTGGTCTTCCACCTTCGCCAGCGCCGCTACATGGCAGGCGGTATCACTGGCGGCGGGCGG
CTATACCGCTTCCGGCGATGCGTCGGACTACGTGAGCTGTCTGGTCAGCAAAAATCCGACGGCGGCGTCTATCACCATTGAGCCGGTGGATGCGGCATTGTGGTATAACGCAAACAGCGAA
CACGCGGTGAAGGTGAAAAAGGGCGATACGCTCCAGCTTAAAGTGACGGTAAAAGACGCCAGCGGCAATCCGCTGCCGCAAGCGCCGTTCGTGTTAAGCCGCGGCGACGGCTATACCCG
CCAGGGGGAGAAACATATCGCCGGTAGCGGCGACGGCATCGTCTCGGCGGTAGTGATTGACGGCGATTCGCTCAACGACACCGCCACCAAAATCGGCGGCATGACCGGTGAAAACGGCA
GCAAAATCATCAACGTCACCCGCCCGGATGCCCACGGGACGAAAGTCGCGATTACGGCGGCGCTGTACGATAACGCCAGCGCGACGGCCAGCATCGACACTATTTTTACCGTGGTCACCAG
CCCGGACAGCGACAAAGCCAAAATGTGGGGCCATATGCCGGAAACGACGACGGCGGCCAACGGCGAGGTGTTTAAGCGCCCGCTGTTATCGGCTGAAATCGCCAGCGGCGTTACCCACG
GCGACAATACGGAAAACAACGAAGCGTGGGGAATCGTCGATTTTGAAATGGCGAACGACGCCTGCGGCGCGGGATACGTGCCGACCCTTGCCGATATGCAATCGCTGTACGACGCCAGGC
CCGGCGGCGCCATGAATACGCAGCAGGGCTGGCCGCTGGACGGTAAAAACTACCAGGACAGCACCGCCGATTTGAGCCGGAGTACGCAAAACCGCTACGTTAAGTCGATTAATCTGCGCG
ACGGCGGCGTCGGTTCGCTATTGTGGAATGAACAACTCTATTTTGTCTGCCTGCAAAACGCCCATCCGGCGGCGACGCAGATAACGCTCACTTCGCCTGTGTATAACGACAGCGACGGATTC
GCCAAAGCGAAAGTCGGCGAGACCATCCCGGTCATCATCACCACGCGGGATGCCCAGGGCAACCCGGCCGCCGACACGCCGGTTATCTTTACGCGCGGCGACAGCGTCGGGCGCGCGAA
TCAGGAGGTGAATAGCTCGGCGGCGGCGGATATACAGATAAACCACAGCGACGGGCGTAGCAGCGGCGCTAACTATTACACCGCCACGGGCGCCGACGGCACACTGACGCTGAATATCAG

CCAGGACAGCGGCGCGGGCTTTAAAACGCCGCTGACGGCGGCGATAGAACACAACGGCGTAACGAGCGCGCCGCTGCCGGTCATCTTTACCGTGGTCACCAGTCCGGACACGCCGAAG
GCCAACTACTGGGGCCATATGGCGGAAACGCTGACCGACAGCAGCGGCGTGGCGTACAGGCGTCCGCTGCTGGCCAGCGAGTTTAGCGCTACGCCCGGCAAAACGCTGACCATCGCCAA
CGGCTACTACGACAAAGGCGAAACCTGGGGCATGATAACCGTCGATAAAGCCTGGAACGGCACCGGTGGCGGCTGTGGGCGCGACACGCTACCGACCGTAGCCAACCTCCAGACGCTGT
ACGGGACATACCCGGATAACGCCATGCGCAGCCGTAACGGCTGGCCGATGACCAGTTCAGGCAACAACAACGTCAGCCGCTACTGGTGGGCGGGGGATTACGTGATATCGTCAGACGGCA
CGAAGTCGCTCTACGCGGCGGTCAACCTGTTTAACGACGGCAATGATGTCAAAACGACGACCAGCACCTCGATGTACTACATGCAGACCTGTCTTGCCTCGCCGCGCAGCGCCGCCGCCAG
CCTGACGCTGACGCTGGCAGGCCAGGATGAAACCACCGGAAGCGCAAAGGCGAAGAAGGGCGAGCAGATGGCCGCCACCATCGACGTGAAAGACGCCGCAGGTCAGCCGATGAAAAA
CGTGATGGTGAAAATCAGCCGCGGATCTTCGTACAATCGCGCCAACAGCGCCACATCGTCCTCAAGCGTCACAGACGATATTACGCTGCGTAATGTGATGCCGTCCGGCCTGGCGACATACC
TGCTGGATACGTCGGCGAAATATCTGTATGCGCAAACTGATGCCCAGGGACAGGTGACATTTACCCTTGCGCAGGACAGTACCGCCGGTTTAAAAACCACTATCAGCGCCGCCACGATGGA
CGGCAGCAACCTGACAGACAGCAAAGACGCAATCTTTACCGTAGTGACCAGCCCGGACAGCGATAAGGCCAAATACTGGGGCCATATGCCGGAAACCTTTACCAACAGTAAGGGGGTTGA
ATTTAAGCGACCGCTGCTGCGCGCTGAACTTTCTTCAACGGCGGACACGTCGGGTTATACGGAAAATAACGAGACCTGGTATACCTGGAGCCGTTATCCGAATATGTATCAGGACACCGCCA
GCCCGTGCGATCGGCTGGGGTTGCCGACGGTAAATGATTTACAGACGCTATATACCGATTATCCCAATGGCGCACTCACGACAACGCTTGGCCTACCGGTGGCATCAGGAAAATATTGGGGG
GCGGGTAACTCAGTACCGGATGCCACGCACTCAGACAGTCAGTTTCAGTATGTCAGACTCAGCGATAATAATACGCTGACAACCAAAGCCAATACCGCCACGGCGCAGCTTTGTCTGGCAA
AGCGCCGGGACTTGTCGATAGAACTCACCTCCAGCGACATGGATGCGGATAAAGGCGCTCCAGTGGCGAAGAAGGGCGAGAGCCTGCCGTTGACCGTCACTGTGAGGGATGGTTCCGGC
ACGCCGCAGCCCAATACCGCTATCCGTTTGGGACGAACGCTTTCGATAGACCGGGCGGGTGTCGTTGATGGCAGTTCCGGCGGAGGCATGGTACTCACATCGGTTGCGCCGTCTACGGGCT
CAATGACGTTTAACTGCACGGTATCTTCGTGCACAAGTTATTGGTATGGCATTACGGATGAGGATGGTAAAGCGCAACTCGAAGTGACGCAGGATGATTCCCGTGGCCTGAGAACACCGCT
GCAGGCGATGCTTGTCGACGATCCGCTGACAGTTAGCGATATGGACGTCATTTTCACCGTCATTACCAGCCCGGACAGCGATAAAGCCAAATACTGGGGACACATGCCGGAAACCGTGACC
AACAGCGCGGGCGTGAAGTTCCGGCGTCCGCTGCTGGCGGCGGAGATGACGTCGAATAGCGGTACTTACCTGGTTAACAATGAAACATGGCCCTTAGTCACCGCAGCGAACACTGAAAAA
GCGGGCGCAACCGGCTGTGATGCGGAATACCAGCCGTTGTCAGGCGATCTGCAAACGTTGTATGGCGACAATCCTAACGGCGCAATCGGTACAAACTACGGCTGGCCGGTGGCGGGGAA
CAAATCCTGGTGGGTGGCGGATCGTGCGCCGAATACCGGATATTACCAATTTATAAACCTGAATAGCGGCGGTAAGGGCACCGCCAGTTCATCGACTGCCACCGGCGCTCAGGTGTGTCTG
GTCGAGCCGCGTACTTCTACTCCTGCCAGTATTACGCTGACCTCGACGGCGATGGACAGCGCGAAAAATGCGGCGGTAGTGGCGAAGGGAAGCGCGATGCCGTTAACGGTGACGGTAAA
AGATAGCAGCGGTAACCCGGTCGCCAACGTCGGTTTTACGTTGTCACGTGGCGATTCGAAAAATCGGGCAGGTATGGTGATAACGGATGGCGACGTGGCAGCGGATGCCGGAGCAGACG
ATCTCATGCTTAAAGAGCTGACTCCCGCAAGCGCCAGCCAAAGTATGACCACCACCGGGATTGTCTTTACCGGCACTACCGGCAGCGACGGTACGGCAACGTTTACGCTTAATCAGGATAAA
AGCCTGGGGTTGAAAACGCCACTCACGGTGAAAGTGACGGACAATACGACTCTTCACGCATCGCTGGATGTGATTTTTATGGTACTTACCAGCCCGGATACGGATAAAGCGTTGTTCTGGG
GAAATATGTCGGATACGACATCCGTCAACGGAAAAACGCTGCATCGTCCGTGGCTTCAGGCTGAGATGCTGTCAGGCGTGACGCCAGTATTTACCAACGGAGTGCATGCAAATAACGAGTA
CTGGGCGATGGCGCATACGGTGGACAACACGAAATGGGATATTGCCAAACAGTGCGGTAGCCTTAGCAAAGCACCGGATAATAACGACCTGTTGACGCTGTATCATTCAATTAGCTCGTTAG
GCTGGCCGACGCAGGGCTATCCCTATTTATCTAAATCAAGCTCGAGCGGCGGCATGTATTGCGGCGTGGATGAGAATACCAAGAGTCAAAACTGCGCCATTAAGCCGGCAGGCACAGCGG
GTTATGCGACGTGCGTGGAGTAA
>DLDGEG_06855 AIDA autotransporter-like protein
TTGAACAGGACTTACAGTATTGTCTGGAGCGCCGTGCGAAATATGTATGTCGTGGCTTCAGAACTGGCGCGAGGCCACAGCAAAGTTAAAGCGCAAGTATGCGCGAGTGAAACCCATTCTC
CAAACCAAAAGTCCGAATATGGACAAATAATAAAAGCAACGCGCGCGGCGCTGGCTTGCGCCGTGGCTGCAGCGCTCGGATTTTTCAGCCCGCTGGCCATGGCCGACAATCAGGTATCATA
TGCTGATGCGCAGACCCACGTTCTGGATGCGTCAACGCCGCCGATGAGCTATTCGGGCACCGATGAAGGCTCGGCGCTATATGTCTCGGGTGTCGCCACCGTCGGCTGGCAGCCCACCACG
GTCACAGGGACTGGGCTCGTCATTGAAACAAGCGGAGGCGGGGCTGATGATCCGGATGGCGGTAAGTATGTTTCCAACGCCATTTCGCTGGATCATTACGCTATCCTTGAGCTTACCGACG
CGAAGATAACCACTACGGGTATTTATACCCAGGGTATTTCCGCAGCGGATGGCAGCAAACTGACGTTAACAGACAGTACGCTAACGATTGACGGCAATTTCGGCGTAATGACGTTGTATACG
GGTTCAGAAGCAACGCTGGACGGTACCATCGTTGAGGCAGCTAACAGCAGCAGCGCGCAGGTGCAACAAGGCAGTACGCTGAACGTGCTGGACGGTAGCACAATCACCCTGGCGCAAG
GCCAGATAAACGTTGTTGCCGGAAACACCGCGACAGACGAGGGTTCCACCCTGAACCTGTCTGATAGCTCGGTCAGCTCTGCAGGTACGATGAGCACCATCCAGGGTACCAATAAGGCCG
CTCTTAATCTCACCAACGCGACCATAACACATACCAATGCCAGCGGCGCGGCGGTACAGGCCAATAACGCCACTACCCTGGATATTACAGGCGGCAACATTACCTCTGCCGGTACGGGCGTG
TATATTCTGGCGTCGGACGCCAGGATCGACGGCGCCACCATCAACGCCGACGGCGACGGTATCTTTATAACCAGCAAAAGAAAACTCGACGGGTATGAGGATCTCAACGCGCTGACGGTCA
ACAAAGCACAGGTTAATTCAGACACGATTGCGCTGCATGTGGACACTGGCACGACGATTAACGCTCCTATCGTGCTGACCGACAGCACATTTGAAGCGCCGGAAGTGATCAAGCTTGGCAG
CAAGGTGGTAATTCAGGCCAACAATACGACATTGATCGGTGACGTTGCTCAGAGTGACATGTCATCGTCATCGCTCTCTTTAAGCCAGGGGTCCACGTTAACCGGTAGTGTAGATGCGATGT
TTACTACGCTGTCGCTCGACGACACCAGCCAGTGGAACATGACCGACCCGTCAACCGTCGGCAATCTGACCAACGACGGCGATATTACGCTGGGCAACGCGAGCGGTTCGACAGGTACGC
TGCTGACGGTGGACAATACCCTGACCTTGCAGGACGGCAGCCAGATAAACGCGACGCTGGATACCGCCAACAGCTCGCCAATTATCAAAGCCGCCAACGTCACGCTGGACGGGACGCTGA
ACCTTAGCTCAACGGCCACCTTTGTCGCGCCCGAAACCGACGAACACTTTGGATCGATCACGCTGATTGATTCGCAAACTGCGATCACTACCGATTTTGACAGCGTAACGCTGGATGCCGAC
ACCTCCGCTATGCCGGATTACCTTACGATCAACGCGGGCGTTGATGCGAATGATAATACCAACTACGAGCTGTCTACGGGCTTGAGTTGGTACGCGGGCGCAAAATCTGCGCGCGCTGCGCA
TGGCACTTTTACCGTTGATGCAGGCAGTACCTTTACCGTCACCAGCGAACTGGACGAAACCACGGCGACCTCCGACTGGAACGGCAGCAAGCTAACCAAACAAGGCGACGGCACGCTGAT
CCTCAGCAACACCGGCAACGACTACGGCGATACTGAAATTGACGGCGGCATTCTGGCCGCCAAAGACGCCGCCGCGCTCGGCAGCGGCGACGTGGCCATTGCAGAAAACGCAACGCTTG
CGCTGAGCCAGGGCACGCTGGATAACAACGTGACCGGCGAGGGCCAAATTGTCAAAAGCGGTAGCGATGAGCTGATCGTGACCGGCGCTAATGACTATAGCGGCGGCACCACTATTTCCG
GCGGTACGCTGACGGCTGACCATGCGGATTCGCTGGGTACCGGAGCTGTTGCCAACAGCGGCGTGCTTCAGGTCGGCGAGGGCGAGCTGGAAAATACGCTTTCCGGCAGCGGTTCGCTG
GTCAAAACCGGTACGGGCGAACTGACGTTAAGCGGCGATAACAGTTACTCCGGCGGCACCACCATTATCGGCGGTACGCTGACGGCTGACCATGCGGATTCGCTGGGTACCGGAGCTGTT
GCCAACAGCGGTGTGCTTCAGGTCGGCGAGGGCGAGCTGGAAAATACGCTTTCCGGCAGCGGTTCGCTGGTCAAAACCGGTACGGGCGAACTGACGTTAAGCGGCGATAACACTTACTC
CGGCGGCACCACCATTACCGGCGGTACGCTGACGGCTGACCATGCGGATTCGCTGGGCACTGGCGCTATTGCCAACAGCGGCGTGCTTCAGGTCGGCGAGGGCGAGCTGAAAAATACGC
TTTCCGGCAGCGGCTCGCTGGTGAAAACCGGTACGGGCGAACTGACGTTAAACGGCGATAACGATTACTCCGGCGGCACCACCATTGACGACGGTGTGCTGATTGCTGACAATGCGGATT
CACTGGGGACCGGAGCTGTTGCCAACAACGGCGTGCTTCAGGTCGGCGAGGGCGAGCTGAAAAATACGCTTTCCGGCACCGGTTCGCTGGTGAAAATCGGCACGGGCGAGCTGACGTT
AAACGGCGATAACGATTACTCCGGCGGCACCACTATTTCCGACGGTACGCTGATTGCTAACCATGCGGATTCGCTGGGCACTGGCGCTATCGATAACAGCGGCGTGCTTCAGGTCGGCGAA
GGCGAGCTGGAAAATACGCTTTTCGGCAGCGGCTCGCTGGTGAAAACCGGTACCGGCGAGCTGACGTTAAGCGGCGACAACAGCTATTCCGGCGCCACCACTATCACTGACGGCACGCT
GATTGCCGCCAACGTCAACGCGCTGGGCAGCGGCAATATCGACAACAGCGGTACGCTGATGCTGGACGCCAACGGCACATTTGAGCTTGCGAACATCACCACCCATACCGGCGCCACGAC
CGCGCTGGCGGCGGGCAGCACCCTTGACGCGGGCCAGCTCACCCAGGAAGACGGCAGTACGCTAAGTATTGACCTCGGCGCGGCCACCGACGACGCCGTGATTACCGCCGACAGCGTCA
CGCTGGGCGGCACTCTGAATGTTACCGGCATCGGCAGCGTTACAGACAGCTGGACGCCTGAGGCATACACCTATACGCTGATTGACAGCGACAGCGCCATTACGAGCGATTTCGACGATCT
GACCATTGCAGGGATGAACCGCGAAGATGTTGACTTCCTGACTATCGACGGAAAAGTCGATGAAGCAGACAACACCCACTATGATCTGACGGCCTCCCTCTCCTGGTATGCGGACAGAGAC
AACGCCACTACCGACGCCCACGGCACCTTTACCCTGAGCGACCCTGACGGCAGCTTTAACGTTGCGGCTACACTGACAGACGTTGACGATACGCTGGATCCGGGTAGCAGATGGGACGGT
AAATCGCTGACCAAAGAAGGCGCAGGCACGCTGATCCTCTCTGGCGACAACGACTACAGCGGCGGCACCACCATCAATGAAGGTACGCTGGTCGCCGCCAGTACGACGGCGCTGGGCAC
CGGTCTGGTAGACAATAATGCCACGCTGGTGCTGGACGCAGACGGTGAAGTCAGCGCGGTGGGCGGCATCACCACTCATAGCGGCGCGACGACCCAACTGGCGCTCGGCACCTCGTTGG
ATCTCGGCGACAGCGCGTTAATTCAGCAAGATGGCAGTACGCTGAACGTTGAGCTGAATAGTGACTCAGTGCAACCGCTGATTACCGGCAGCAGCGCCACGCTTGGCGGCGATTTGGTCG
TCAGCGACGCCAGCCTGCAAGCGCGCGCCTCCGACGCGGAGTTCCAGTCCTTTAAACTGATGGATATGGACAGCGATATCAGCGGCGACTTTACCAGCCTGACGATGAACCTCACTGACCA
GCCGGACTATCTGACCGTAACCGGGACAATCAATCCTGAAGATGCGTCAGAATATCTGCTTACCGAAGGGCTGAGCTGGAATGCGACCGCCACGTCAGCGACGCCAGCACACGGCACGTTT
ACTCTGGGCGCAGGCGACAGTTTTGAAGTCACCAGCGTACTGGGCGATAAAACCGGCAACGGCGACTGGGACGGTAAATCGCTGACCAAACTGGGCGCGGGTAAACTGACGCTGAGCG
GCGCGAATACCTATAGCGGCGATACCAACGTACAGGAAGGCACGCTGTGGCTGTCCGGGGACGGTACGATTGGCGAAATGGGCAGCCAGCAGGCGGTGAACGTCGCCTCCGGGGCGAC
CTTTGGCGGCAGCAACGGTACGACCGTTAACGGTAAGGTGACCAACGAAGGGACGCTGGTCTTCGGCGACAGCGAGGAGACAGGCGCTATCTTCACCCTCAATGGCGATCTGATCAACAT
GGGGACAATGACCAGCGGTAGCAGCTCCAGCACGCCTGGCAATACGCTTTATGTTGACGGCGATTATACCGGTAACGGCGGCTCGCTTTACCTGAATACCGTCCTGGGCGACGATGATTCG
GCGACCGATAAGTTAGTCATTACCGGCGATGCCTCCGGCACCACCGATCTGTACATCAACGGCATCGGCGATGGAGCACAGACCACTAACGGTATCGAAGTGGTGGACGTTGGCGGCGTAT
CGACCAGCGATGCGTTTGAGCTAAAAAATGAGGTTAACGCCGGTCTGTATACCTACCGTCTGTACTGGAATGAAAGTGACAACGACTGGTATCTGGCGTCAAAAGCACAGTCTGATGATGAC

GACAGCGGCGGTGATGACACGCCTTCGGATGGCGGTGACGATGGCGGTAATGTCACGCCTCCGGACGATGGCGGCGATGGCGGTAATGTCACGCCTCCGGACGACGGCGGTGATGGCGG
CGATGTCGCACCACAGTACCGTGCGGATATCGGCGCGTACATGGGCAACCAGTGGATGGCGCGCAACCTGCAAATGCAGACCCTCTATGACCGCGAGGGCAGCCAGTATCGTAATGCCGAT
GGCAGCGTATGGGCGCGCTTCAAAGCGGGTAAAGCAGAGTCAGAGGCTGTCAGCGGCAATATCGATATGGACAGCAACTACTCTCAGTTCCAGCTAGGCGGAGACATTCTGGCCTGGGGC
AACGGCCAGCAGAGCGTTACCGTTGGCGTCATGGCCAGCTACATCAACGCCGATACCGACAGCACCGGTAACCGCGGCGCGGACGGTAGCCAGTTCACCAGTAGCGGCAACGTAGACGG
CTACAACCTCGGCGTCTATGCCACCTGGTTTGCCGATGCCCAAACGCATAGCGGCGCGTATGTCGACAGCTGGTACCAATATGGTTTCTACAACAACAGCGTAGAGAGCGGTGATGCGGGTT
CTGAATCTTACGATTCAACCGCTAACGCTGTCTCGCTGGAAACCGGTTATCGATACGATATTGCGCTTAGCAACGGCAATACCGTCAGTCTGACGCCGCAGGCGCAGGTTGTCTGGCAGAAC
TACTCAGCGGATAGCGTGAAGGATAACTACGGCACCCGGATCGACGGTCAGGATGGCGACAGTTGGACAACGCGCCTGGGTCTGCGTGTTGACGGCAAGCTGTACAAAGGCAGCCGTAC
GGTTATCCAGCCGTTTGCTGAAGCTAACTGGCTGCACACCAGCGATGATGTGTCGGTATCGTTTGATGATGCCACGGTGAAACAGGATCTTCCGGCTAACCGTGCGGAGCTGAAAGTGGGT
CTGCAGGCAGATATCGATAAGCAGTGGAGCGTTCGCGCTCAGGTTGCCGGGCAGACTGGCAGCAATGACTTTGGCGATCTGAACGGTAGCCTGAATCTGCGCTATAACTGGTAA

Prophage 1

>BDIOMP_11280 hypothetical protein
ATGCAATTTTTCACATCCAGCGATACTGTAATGTTATGTGCGAAGACCTGCGATCGCTGTGGTCGCCATGCGAAGACGGTGGTAGATGACATTGAGTTCAATGAGTTTCTGTCTGTTAATCACT
TAGCTGGATACGGTTCAATTTTTGGTGACAGTAATCGCTTAAAACTGGATTTATGCCAACATTGCCTGAAAGACGTCCTGGGCCAGTGGATTACGGTCTGTGACCAATGA
>BDIOMP_11285 PIN domain-containing protein
ATGGATTCTTATGGGATTTTATCTGCTCTGGCAGAACGAACGATTACAGCGGAAGAAGCAAAAACAATGACACTTGCAGCCAGAAGTTCTTCGGCTCAGGTAAATGAAGCATCACTTGCTCT
GTTACTGATTGATAATACTCTGGATGCGATCAAAGCAGCTGCCAGTGAAGGGAAAGGATACACTTTACTTCCTTCTTATGAGATTTCTGTAAAAGCCATTGATTTAGCAGAAGAATTTTTAAG
GAAACAATGCGGCTATGTTATTGATAACCAGAATGGCGTAAGGACGGTCTATTGGTTTATATGA
>BDIOMP_11290 Virion export protein
ATGCGCTCTGTTATTGTTGCTTTTTTATTTGCCTGTTCATTCTGCGTTTCTGCTGAAACTGTTAATTTAAATAATTCATCTGTTCGCTCATTTGTTCAGTGGTATTCTTCAAAAACTGGCAAGCCTG
TTATTGTTAATCCTGATATTAAAGGAAACGTAACCGTATTTAATGCTGATGTTAATCCAACAAATATTGATGATTTCTTTAAGTCTGTTCTGAATGCTAACGGTTTTGTCATGCTTTCTGGCAATCC
TGCGGTAGTCTCTTTGCCGTCTAAATTACCTTCACAGATGGTTTCGGATTCCGATGATTCTGATAACCAGTCTTATGATACTTTTCCTTCTGAGCCATCTTACCAGCCAGTACCTGTGGCGCTTAC
GGTCAGAAATTTTAAGCTGACAAAAGTTAGATCGTCCGATGTTCAGCAACTGATTAAAATTTATCTTGATTCTAATGGTGGTGGTAATGTCGTGGATTATCCAGGCAATAACTCGCTGATTGTT
TCTGCGCCTGACGAGCTGCTGCCGGTTCTGTCCGATTTTATCAATTCTGTGGATGTTGCCCGCGATCAGGTTCTCATTCAGTCGCTGATGTTTGAAACCAGCTTGTCTGATGGCGTTGATTTAT
CGTTTGCGGCAGGTTCTGCATCCGGTCATAAGGTTGCGGGGGGCTTTAATACTTCTGCACTGGGTAGTGCTCTTTCTACGGCGGGCGGTTCTTTTGGTATTTTTGATGGTAACGTGTTGGCG
CTGTCTCTGCGTGCTGTCCAGAGTAATTCACGCTCTAAGGTGATTTCAACGCCGCGTATTCTTACTCAGTCTGGTCAGACTGGTTACATTTCCGTAGGTAAGAATGTGCCGTTTATCACCGGAA
AGGTAACGGGCGAATCTGCCGGGGTAAATAATCCGTTTCAGACTATCGAGCGTCATGATGTTGGCGTTTCTCTCAAGGTAACGCCTGTTGTCATGGGTAACGGGCAACTAGTTCTGACTATC
GACACTCGCGCTGATTCAATCAGCAATGATGATCAGGCATCCGATATTATTACTAATCAGCGCCAGATTCAGACGACAGTCCAGATTAAGGATGGTCAGACGTTGTTATTGGGGGGGCTTATC
GATTCATCGTTCAGCAATGCCGAGCGTTCAGTTCCAGTTATAAGTAAAATTCCTCTTATAGGCTGGATTTTCAGTAGCAAGGCAGACAGCAATGAACAGCGTATTATGTATATTTTGCTTACAG
CACATATCATTCGTCCACTTTGA
>BDIOMP_11295 Gene 1 protein
ATGGCGGTTTATGTAGTAACAGGCAAATTAGGCTCAGGCAAGACACTTGTTAGTGTTTCCAGAATACAGGAAAGACTTGCTAAGGGTTGTCCTGTTGCCACTAATCTTGATCTTAAATTGCAT
AATATGCCTATGGTTGGGCGTTATGCGAAAAAAACGCGCGTTATTCGCATTCCTGACAAGCCTTCATTAAATGATTTACTTGCTATTGGTATTGGGAATACATCTTACGATGAATCCCGTAATGG
CCTCCTTGTACTTGATGAATGTGGTACTTGGTTTAATTCTCGCTCATGGGGTGATAAAGACAGACAACCTGTTATTGACTGGTTTTTACATGCCAGAAAATTAGGCTGGGATATTATATTTTTGA
TTCAGGATATTTCGATAATGGATAAGCAAGCTCGTCTGGCGCTTGCTGAGCATGTTGTTTATTGTCGCCGTTCAGATAAATTAAACATTCCTTTTGTTGGCTCCATTATGAATTTGGTTTCAGGG
GCGAGATTTTCTTTACCAAAGGTACACTTTGGCATTGTCAAATATGGTGATAATGTTAATTCAATCACTGTTGATAAATGGATATATACAGGAAAATCTCTTTATTCTGCTTACAATACCAAACAA
GCGTTCACAGATAATTATCCTCATGGCGCATTTTCGCTTTTGCCACCATTTATCACGCACGGTCAATTTTCTGTTCACAGAGGATTTAGTTATTATATGCGCCTCACTAAAATTTATTTTCGCAAA
TCGAACCGTCTTATATTAATGCTTTCTTTTTTGGCGCTGGGGCTTGCGTTTGGTTTCTGGCTCCAGTCTGGAAAGAATGTTGATGAAATCTCAGCTATTAAATCTGCTTATGCTGAACAGGCGA
GGGCGGTAACGCCTGATTCGTCCAGTGACTTACCCCGACTTTCTATTAATTCTTTTTCACAACTTGGCTTTGACGTTTCCGTTACGTTTGTTGATGCAAAAGGCATGAAATATCAGTATTTTGAT
TTGATTAAAGATGGTTATTCCGTTGATATTAAAGATGCCTGTCGTGTTGTTATTAGAAAAGGCCGTTATTTACAGACTGTTACCTGTCAGGAGTAA
>BDIOMP_11300 Head virion protein G6P
ATGCCTTTATTATTAGGTATTCCTGCATTGTTGCGTTTTCTTATTGGTCTTGTTCCTTTGTTTATTGGCTATGTGGCGAGTTTTTTAGCTCGACTTGCTACCAGAACAGGATTAATCGCCTTTGCAT
TGGTCGCATTAATTACAACAACTGTTACGCTTTTAATGCAGTACCTTGCTGAGGTCATGTATAACGGTTTACCTGCTGATTTCTCCCATTTAATGGCGTCTGTATTGCCTGACCATTTTCAGGCGT
GTGTTAACGTTATTATGGTTACTCGTATCAGTGTTTTTGTTTTCGATTTAAAACAAAAATTTCTTGATTATGCAAACAGGGTGATTTAA
>BDIOMP_11305 Attachment protein G3P
ATGAAAAGGAAAATATTAATTCTTTCCGCTGTCCTCATTTCTCCTTTTTCACATGCTGAGTCATGGGAAAGCATTACTAAATCCACTTATCAAAGTTCTGCCTACGCTGAAAGTAAGCAAATAAC
CAATCAGGATGGCTCTAAGACAACGGTTTACTATATTGATGCTGCTATGCAGGCCTCCGCTTGTCAGGGTGCTAAATCCAGTGCTCAGAGTGTATTTACTCGGGTTAAACCAACTTATGAGGG
TATCTGGCCTGATTCTGAATTCCGTCTTGTTTTTACTGGTGATTGTACTTACAGCGATTCACCAGGGCAGAAGGATAAATATTGGTCTTTAACGGCTTATATTGTTGGTAATATTCAGCGTTCTGT
TCCTGATGAAAAACCTACTGACCCGACACCAGAAGAAATTTGTGAAGCGAAGCCGCCAGAAGAAGGTGTATTTAATAATGTTGATTCATATGATGGTGGTCGCTATATCTACTATAACGGCTG
TGAATATGAGGCTACTGGTGTCATTGTTTGTCAGGGTGATGGTACTGTTTGCGCTGCAACATGGAAGCCTACAGGTGCTGTAGCTGACCCCTCTGATAAACCCTCAACCCCCCAAAATGGCG
GTGGTGAGTCCGGTGGTGGTGAGTCTGGCGGTGGTGAGTCTGGCGGCGGTAGTTCTGGCGGTGGCTCTGGCGGTAGTTCTGGCGGTGGCTCTAGTGGTTCCAGTCTTTCTAAAGGTGATA
TTCAGTCTGCGATCGAAGGTGCTTCACCCAAAATAGCCAGCGATATTCATGATAAATTAACGGAGAAAGACACTTCATCAGATGATAAAAAAAATGCCGATGAACAAACCAGGAATAATATAA
ATCGTCTTGACGATTCCATTAACAATCTTACGCGGGGGGCTGGCCGTTTTGCTGACCCTTCAGGTGGTGATTCTCGTTATGGAAAGGGCGACTCTGAATTAGATGGCGCTTCTACTTTGGCTG
ATTCTGAATTGGGAATTGAAAAGGATTCTCACGGTGCTTTATGGGAAGCATTTTTAAATAAAGGTGCTATGCTGCCTAATTTACCCAATGGTAACGGCTGCTCTGATTTTATTATTTTTCCCGGA
GAGGTTTATCAGATTGATATTGGTTGCGATAAATTACTGACTATTAAAGATGTTCTTTCATGGGTTTTTTATTGCCTTACGTTCTGGTATGTATTTACTTCTTTAACTTCATTGCTTCGCAAAGGG
GGTGAGTGA
>BDIOMP_11310 Capsid protein G8P
ATGAAAATTCTGTCTACTGTTAAATATAAAGTTGCTCTGGCTTCAACTGCGCTTTTTATTTCTGCAAGTTCTTTTGCGGCTGAAGGCGCTACAGGTGGTACTGATTATGCAGGCCAGGCAATGG
ATGCTTTGTTGACTCAGGCAAATGATCTCATTGGTAAAGTATGGCCTGTTGTTGTGGCTGTGGTTGGCGCTGGGCTTGCCATTCGTATTTTCAAAAAATTCTCTTCAAAAGCGGTTTGA
>BDIOMP_11315 DNA-binding protein
ATGTCTAATTATGGTCTTTTCGTTAAGGGTAAAATGTTGGGAGCCCGCCAACGTAATAAAGTTAATGGTCAGGGCTATTATAATGAAATTGGTATTGGCCTTGAAATACCTGATGGTTTTGGTG
GTACAAAGCAGGATCAAATTATTATTCGAGTTTCTCAGGCTCTCGTTAACGCAGGTCTAATGAACCAGGCGAATGCTTTCATTGGGAAATTAGTTCAAATTCCTGTCTATGTCCGTGCGTGGTC
AATGGAGGGTAGGGAAGGTGTAACTTATAATGTTTCTTCCGATGGTGGCATCGCAGAGATCAAAGGTTAA
>BDIOMP_11320 Replication-associated protein G2P
GTGATCGATTGGCTTACCGGGATTTTCCCTTGCACACACAAACCGCTTCCGGCGGGGAGTGTTGTCAGCGTTGATGCTGACGGTGCAATTGAGTGGGAAACCGTTAAGCGACTGACTGTTC
GCGGCTCGCATGAAGCAACGATGAAAGTACGATCGATAGGATCTAATGGCGAAGGTAAAGCGACACATTTGTACATTGATGGTAATCCTTCCAAGTTTCTGCAGGGGCATTCTGTTGTTGGG

TCGGATGATATACAAGGGCTCATGTTGACGGTCTATGCCAGGATTTTATCCTTATTGAATATTCCTCATGATTTAGCGTCTTATAAAGCCGTTATGGCAGGTCAGTATAAAATTTCACGTGTTGAC
ATTAATTACATGTATTCATTATCTACGCTGGAAAATGTCAGATCATGGCTTTATGCCGCCGAATTTAAAGCTAAAACACGCCATGGTCGCGCATGTGGGAAAGGCGGTACAGTTTATTTAGGTA
AAAACTCCCGTCGATGGAGTCTGAAATTTTATTCAAAATATGATGAACACGTTTCTGGTAAAAAAGGGCACCAGATAGCCGAAGAGTTTGTACGAGCCGGGTTACTGGACTGGACGAAAGA
TAAATTACGTATTGAATTAACATTAAGAACAACTGAGCTTATTGATTTAAATTTAACGCTTGGCGCTAACTGGAATATGAAAACACCACGTCAATTATTCTCTGAATATGTAGGGAGAATAGAAA
TGAATCAGAATGCTATTTTAAGTGATGAAAAAATAACGAAACTGCCAAGAAAAAATACAGTCAACTTATTTACTTTGGAAACAAGGGGCTAACATGAAAGAAATGTTACCTCATAACACTTTTT
ATCGCCATAGAAGAGAATTGCTTTCGTTCGGTATCGATATTAATTTCTATTGTGATTCACCAGACTCTAATAACGTTGTTCCGCTAATAAGAACGCTGGAAGCCAAGCCAGCGGAAATCCCCTT
ATGGATTTATGAGAAAGGTTTTATTTTCGATTACAACCGTATTTCGCACGCCAGTAGCTGGCATTAA
>BDIOMP_11325 hypothetical protein
ATGGCAAAGCGCACGACTTATAACGTGACGGAAGAAAGAAAAATGAAACTAGAACGACTGGCTATAAATGCCAGTGTAAAACTAGGAAAAACAATTACATGGACAGAGATTTTAGGTTATC
TGATTGATAACTACTCTAAAGATGCAGCAGAAGATCTCATGTCTGCCAAACAAAAGGATTAA
>BDIOMP_11330 Fido domain-containing protein
ATGATCACTATAGAAGAAGCAACATCGGTCCATGAATATTTAACAAATTACTATCAAAACTCTGATGATCCTATCTCTCCTCCGGGAATAAAAAACATAGAGCTTTTAGAGTCTGCCATAGCAAG
ACCTTTTATGACCATGAACAGAAAGGATGTCTATCCAGATGATTTAGATAAAGCCGCAGCTCTATTTCATGGTGTCATATCCAACCACTGTTTCCATAATGGTAATAAAAGAACAGCACTTCTAC
TAACCATGTGCTTTTTAGACCGAGCTGGATATTGGCTGGATAAGTGTGATGACCTGCAACTCTTTGAATTTACAAGAGGTGTTGCGGCACATGAGATATGTGAAAATAGAATTGATGAAATAA
AAACAATCAAGGCATTTTTCCGAAGTAATTCACGGAAGAAAAAAATCACAGATGAACAATTGAGTTTTATTGCATTAAGCAGGCATCTAACCAACGCAGGTTTCAATATAGAAGACGATGGT
GACTATTACTCTATCTTTAAAAATAATAAAAGATATACAAAAATAATAAAAAAAGGCGCATCAGGACATGAAAAATACGATCCACAATACATAAAATCCCTTAGAAAAAGGTTGCTTCTTACCCC
GAAGTATGGATGGGATAGTATAAGGTTTTATCAATTATATTCATCATTGACGGAAAACATTGGTGAATTATTACGTTTACGTGGAGAGGTAATGGACTGGCTTGCAAAAATATAA

Prophage 2

>BDIOMP_13895 DinI-like family
ATGTTCGTAGAACTGGTTTATGACAAGCGTAATGTTGAAGGACTCGAAGGGGCCAGCGAGATCATTCTGGCCGAACTGACGAAGCAGGTGCACCAGATTTTCCCTGATGCCGAAGTGAGG
GTGAAGCCGATGCAGGCAAACAGCTTGAATAGTGATGCCAACAAAAGCGATCACGAAAAGTTGAACAGATGCCTGGTTTCAGATTAA
>BDIOMP_13900 hypothetical protein
GTGGTCGTTTTTTTGACTTTTACTGATTCAAAAGGAATATCGGAATATATACTTGACCCTTGGCTGGATGCGCGCATTTTCAAAAAGGAAGAGTCATATGAAATTTATAAAAACAATAGCAGTG
AATATATCAATGAAAATCATTGCTTCGAAGCATACGATAAATACACTGCTATAATGAATAGCGCAGAATATATAGACGCCATAACAAAGACAATAAATCTTTTATATAGAGTTAATCTTGATGAAAT
TCAATTAACAAATCCATTTAAGTTTATATAA
>BDIOMP_13905 Guanine nucleotide exchange factor SopE
GTGACAAAAATAACTTTATTTCCCCATAACTTTAGAATCCAAAAACAGGAAACCACACCACTAAAAGAAAAATCAACCGAGAAAAATTCTTTAGCAAAAAGTATTCTCGCAGTAAAAAATCAC
TTCATCAAATTAAATTCAAAATTATCGGAACGTTTTATTTCGCATAAGAACACTGAATCTTCTGCAACACACTTTCACCGAGGAAGCGCATCTGAGGGCCGGGCAGTGTTGACAAATAAAGTC
GTTAAAAACTTTATGCTTCAAACGCTCCATGATATAGATATTAGAGGTAGCGCGAGTAAAGACCCCGCATACGCCAGCCAGACCCGTGAAGCTATACTATCGGCAGTTTACAGCAAGTATAAA
GATCAGTATTGTAACTTGCTCATCAGCAAAGGAATCGACATAGCGCCTTTTCTTAAGGAAATTGGCGAGGCTGCGCAAAATGCAGGTCTGCCCGGAGCAACCAAGAATGACGTTTTTACGC
CAAGCGGCGCAGGAGCCAATCCTTTTATAACTCCGTTGATTACATCAGCATACAGTAAGTATCCACATATGTTTACCAGTCAACATCAGAAGGCATCCTTTAACATCTATGCGGAGAAGATCAT
TATGACAGAAGTTGTACCGCTGTTTAATGAGTGTGCTATGCCGACTCCACAGCAATTCCAACAAATACTAGAAAACATTGCTAATAAATATATCCAAAACGCTCCCTGA
>BDIOMP_13910 tail fiber assembly protein
ATGACTTTTAAAATGAGCGAACAGGCGCAGACAATTAAAATTTTTAATCTTCGTTCAGATACAAACGAATTTATTGGCGCAGGTGATGCATATATCCCGCCGCACACTGGATTACCGGCAAAC
TGTACTGATATCGCCCCTCCTGATATTCCCTCCAGTCATATTGCTGTATTTGACGCTGAAACCCAAACATGGAGTCTGCAGGAGGATCACCGCGGCGAGACGGTTTACGACACAACAACCGGC
AATCAGGTTTATATCTCCGAACTCGGCCCGTTGCCCGAAAACGTCACATCAGTTTCACCAGACGGTGAATACCAGAAATGGGATGGTAAGGCGTGGGTGAAGGATGAAGCGGCTGAAAAA
GCAGCGCAGCTTCGTCAGGCGGAAGAAACCAAAAGCAGGCTCCTGCAAATGGCATCTGAAAAAATCGCGCCGCTTCAGGATGCGGTTGATCTTGGACTCGCAACAGATGATGAGAAAGC
GCAGCTCGACGAATGGAAAAAATACAGGGTGCTGGTAAACCGGGTGGATACCTTAAATCCTGACTGGCCGGAGAAACCATCTCAGTTATAA
>BDIOMP_13915 Phage tail protein
ATGCCAGTACTTATTTCCGGCGTACTGAAAGATGCTACGGGAACGCCGGTACAGAACTGCACCATTCAGCTGAAGGCCTGCCGGACCAGTACGACGGTGGTCGTGAATACGGTGGCATCGG
AAAATCCGGATGACGCCGGGCGCTACAGCATGGATGTGGAGCAGGGGCAGTACACTGTCACGCTCCTGGTGGAAGGGTATCCCCCGTCACATGCCGGAGTTATTACGGTTTACGATGATTC
AAAGCCGGGCACCCTGAATGATTTTCTGGGGGCCATGACGGAAGACGACGTCCGCCCGGAGGCGCTGCGGCGTTTTGAGGCGATGGTGGAAGAAGTTGCCCGCCAGGCATCGGAGGCA
TCGCGGAATGCCACCGCCGCAGGGCAGGCATCTGAACAGGCGCAGACATCAGCAGGTCAGGCATCGGAAAGCGCCACGGCAGCAGTGAATGCAGCCGGAGCGGCAGAAGCATCAGCC
ACACAGGCAGCCTCATCCGCAGCATCTGCGGAGAGCAGCGCAGGTACGGCGACCACAAAAGCCGGGGAGGCATCAGCCAGCGCGGCGTCGGCTGACACAGCCAGAACGGCAGCAGCC
GCATCGGCAGCCGCAGCGAAAACATCTGAAGAGAATACAGATGCCTCCCGTACTGCCGCCGGAGATTCAGCTGCTGCCGCAGCCGCCAGCGCGACGGCGGCGCAGACATCAGCAGCGCG
CGCCGGAGCATCCGAAACCGCCGCGAAGACGTCAGAAACGCAGGCGGCTTCCAGTGCCGGTGATGCAGGTGCGTCAGCCACTGCGGCGGCAGCGTCGGAAAAGGCGGCAACCGCATC
GGCAGCCGAAGCAAAAACATCTGAGACAAACGCAGCAACGTCAGCAAGTACATCAGCGGCCAGCGCAACAGCCGCCTCGTCATCAGCATCGGAGGCATCCACTCACGCCGCCGCATCTGA
TACCAGCGCATCACTGGCGGCGCAAAGCAGTACTGCTGCCGGAGCAGCAGCCACCAGAGCAGAAGATGCCGCAAAACGGGCAGAAGATATCGCGGACGTGATTTCCCTGGAAGATGCCA
GCCTGACGAAAAAAGGTATCGTTAAGTTAAGCAGCGCCACGGACAGTGACAGCGAAGCGCTGGCAGCCACGCCAAAGGCGGTCCATGCTGTCATGGACGAGGTACAGACCAAAGCGCC
GCTGGACAGTCCGACATTCACTGGAACGCCGACCACACCGACGCCGCCAGACGATGCTAAAGGGCTTCAGACAGCAAACGCGGAGTTTGTCCGCAAACTGATTGCCGCGCTGGTTGGTTC
CGTACCGGAGTCACTGGACACCCTGCAGGAACTGGCGGACGCGCTGGGTAACGATCCGAGCTTTGCCACCACTGTACTGAATAAACTGGCGGGCAAGCAGCCGCTGGACGATACACTGAC
GGCGCTGTCAGGAAAAAGCGTTGACGGTCTTATCGAATACGTTGGTTTACGGGAAACCATAAATCACGCCGCCGATGCATTACTAAAATCACAGAACGGCGGCGATATTCCGGAAAAGCCG
CTGTTTGTACAAAATATCGGAGCGCTCCCTGCATCAGGTACGGCTGTTGCAGCGAACAGACTGGCATCACGCGGCGCGCTTCCGGCACTGACTGGTACGACAAGAGGCAGTGATAGCGGC
CTGATAATGGGCGAGGTTTACAATAACGGTTATCCAACGCAATACGGGAATATTTTGCGTCTGACCGGAACCGGTGATGGAGAGATATTAATCGGATGGAGTGGGGTTAATGGTGCTCCTGC
GCCTGCATATATTCGCAGCCATCGAGATACCGCCGACGCTGAGTGGTCAGAATGGGCGATGTTCTACACCTCACTAAATCCGCCACCGGATTCGTATCCAGTAGGTGCGGCGATAGCATGGCC
GTCTGATGTTACCCCAGCCGGTTACGCCCTGATGCAGGGGCAGTCCTTCGATAAATCTGCTTACCCGTTACTGGCTATAGCGTATCCGTCCGGCATTATCCCTGATATGCGAGGCTGGACAATC
AAAGGTAAACCCGCCAGTGGGCGAGCTGTACTTTCTCAGGAGATGGACGGCAACAAATCGCACTCGCACACCGCGCGGGCGCAGGATACTGACTTAGGGACAAAATCTACCTCATCCTTT
GATTACGGCACGAAATCGACCAATACCACGGGCAATCATACTAACCAGTTCGGCGGTTATATCAATTCATACTGGGGAGATTCCAATCACACCTCATTTCAGCCTGGAGGTGGTGCATGGACA
CAGGCCGCTGGCGACCATGCACATACAGTTTATATCGGAGGACACGAGCACACCATGTATATCGGTCCACACGGACACGTCGTTATTGTGGACGCAGACGGTAATGCGGAAACCACGGTTA
AAAATATTGCATTTAACTACATAGTGAGGCTGGCATAA
>BDIOMP_13920 isrG Hfq binding RNA
GTTTTGCGGCATCTTCTGCTCTGGTGGCTGCTGCTCCGGCAGCAGTACTGCTTTGCGCCGCCAGTGATGCGCTGGTATCAGATGCGGCGGCGTGAGTGGATGCCTCCGATGCTGATGACGA
GGCGGCTGTTGCGCTGGCCGCTGATGTACTTGCTGACGTTGCTGCGTTTGTCTCAGATGTTTTTGCTTCGGCTGCCGATGCGGTTGCCGCCTTTTCCGACGCTGCCGCCGCAGTGGCTGAC
GCACCTGCATCACCGGCACTGGAAGCCGCCTGCGTTT
>BDIOMP_13925 Secreted protein

ATGACTATGTCGCGCTTAATTTCGCTGGCGGCAGGGGTTTCCCTGTCCGTTTTATTCTCCACTGCTGCCGTTGCCGATAACGGAAGAGGAAGCGGCAACAGCAATATTGAAAACCAGACCCG
GATTTATACCGGCACCGACCGTGGGCAGAAACAGCACCGCGAGGCAAAGGGAAAAACAATCACGCGGAGCGTCCAGTGTTCGCTGCCGGCATATTTACGTGACCCGGATAATCAGTGCTG
A
>BDIOMP_13930 Gifsy-1 prophage VhsJ
ATGGGAAAGGGCGGAGGAAAAGGGCATACGCCCCGCGAGGCACCGGATAACCTTAAATCCACGCAGCTGCTGAGTGTCATCGATGCCATCAGCGAGGGACCGATAGAAGGCCCGGTGAA
CGGTCTGCAAAGTGTTCTGGTAAACCAGACGCCGGTGGTGGACCGAGACGGTAACACGAATATCCACGGCGTGAAGGTGGTATACCGCGTCGGTGAGCAGGAACAGACCCCGCTGGAGG
GATTTGAATCGTCCGGCGCCGAGACGGTGCTTGGTGTACAGGTCAAACACGACAATCCGGTGACCAGGACCATCACGGCTGCAAATATTGACCGCCTGCGTTTTACGTTCGGCGTGCAGTC
ACTGGTGGAGGCCAACAGCAAGGGCGACCGAAATCCGACATCCGTCAGGCTGCAAATCCATCTTGAGCGCTATGGTCAGTGGGTGGTGGAAAAAGAGATTACGATTACCGGGAAAACAA
CCACACAGTATCTGGCCTCGGTGATAGTGGATAATCTCCCTCCCCGGCCATTTGGTATCCGGATGGTACGTGTGACGGCAGACAGTACCACTGACCAGTTACAGAACAACACGGTCTGGTCG
TCGTATACCGAGATTATTGATGTCCGGCAGCGCTATCCCAACACCGCCGTGATTGGCCTGCAGGTGGAGTCTGAGCAGTTCGGCAGCCAGCAGGTGACGCGAAATTACCATTTTTTCGGGC
GGATTATTCAGGTGCCGTCGAATTACGATCCGGTAGCGCGAACCTACAGCGGCATCTGGGACGGCACGTTCAAGCCTGCATACAGCAATAATCCGGCATGGTGTTTATGGGATGTGCTGACA
CATCCCCGTTATGGCATGGGACAGCGAATCGGCGCGGCGGACGTGGACAGGTGGGCGCTGTATGCAATAGGCCAGTACTGCGACCAGATGGTCCCTGACGGATTCGGCGGGACAGAGCC
GCGTATGACCTTTAATGCGTATCTGGCGCAGCAGCGTAAGGCGTGGGATGTGCTGACCGACTTCTGCTCCGCGATGCGTTGTATGCCGGTGTGGAACGGGCAGAGGCTGACCTTCGTGCAG
GACAGGCCCTCGGATACAGTCTGGACCTATACCCGCAGCAATGTGGTAATGCCGGATGAGGGTACACCGTTCCGTTACAGCTTCAGTGCGCGGAAGGACCGCCATAATGCGGTAGAGGTGA
ACTGGATCGACCCTGATAATGGCTGGCAGACATCCACGGAACTGGTGGAAGACACGGTCGCCATCAGTCACTACGGACGCAATCTGGTAAAAATGGATGCGTTTGGCTGTACCAGTCGCGG
GCAGGCGCACCGCGCCGGGCTGTGGCTGATAAAAACGGAGCTGCTGGAAACCCAGACGGTAGATTTTAGTGTGGGGGCGGAGGGGTTGCGCCACGTTCCCGGTGATGTGATTGAGGTT
TGCGACGAGGATTATGCCGGCATCAGCCTGGGCGGACGGATTCTGTCCGTTGACCGCGACCGTCGCATTCTGACCCTTGACAGGGAAATTACCCTGCCGTCGTCCGGCACCACGCTGATAA
GCCTGGTGGATGGCGAAGGTTCGCCGGTCAGCGTGGACGTGCAGTCTGTTACCGACGGTGTGCAGGTTCAGGTCAGCCGGATACCGGACGGCGTGGCGGAATACAGCGTCTGGGGGCT
GAAACTGCCGTCGCTGCGCCAGCGTCTCTTCCGGTGTGTGGCTGTCCGGGAAAACGACGACGGAACGTATGCCATCACCGCCGTACAGCATGTTCCGGAAAAAGAGTCCATCGTGGACAA
CGGGGCATCGTTCGACCCGCAGCCCGGAACGATTCACGGCACTGTTCCCCCGGCGATACAGCACCTGACCACAGAAATTCTGGCGGAGGAGGGACAGTATCAGGTACTGGCGCGCTGGG
ACACACCGCGAGTCGTTAAGGGCGTCTCTTTTTCGTTGCGCCTGAACGTGGCGGCGGAAGATGGCAGTGACCGGCTGGTAAGCAGCGCAGGAACGCCGGATACGCAGTACCGGTTCCGG
GGGCTGATGCCGGGGCGCTACACCCTGTCCGTCAGGGCGGTGAACAGCCAGGGACAACAGGGAGACCCGGCCAGCACACAGTTCAGCATCTCCGCGCCGGCGGCACCATCATTTATCGA
ACTCACCCCTGGCTATTTCCAGATTACAGCCACACCGCGTCAGGCGGTATACGACCCGACGGTGCAGTATGAGTTCTGGTTTTCAGACGCGCAGATTACGGATATCCATCAGGTGGAAAACG
CCGCACGATATCTGGGAACAGCGCTGTACTGGATAGCGGCCAGCGTGAATATCAGGCCCGGCAGGGATTACTATTTTTATATCCGGGCGGTAAATCAGGTCGGTAAATCCGCATTCGTGGAG
GCGGCCGGGCAGGCCAGCAACGATGCCGCAGGCTATCTGGATTTTTTCAAAGGGCAGATAACTGAAAGTCACCTGGGTAAGGAGCTGCTGGAAAAAGTAGATCTGACGGAGGATAACGC
CAGCAAACTGCAGCAGTTTTCGAAGGAGTGGCAGGACGCTAACGATAAATGGAACGCCATGTGGGGCGTCAAAATAGAGCAGACCAAAGACGGCAAATATTATGTGGCCGGACTTGGAC
TGAGCATGGAAGACACGCCTGACGGGAAGATAAGCCAGTTCCTGGTGGCGGCGGATCGCATTGCTTATATTAACCCGGCAAACGGAAACGAGACGCCCGGATTCGTCATGCAGGGCGACC
AGATAATCATGAACGAGGCGTTCCTGAAATACCTGAGCGCGCCGACCATTACCAGTGGCGGGAATCCTCCGGCATTTTCCCTGACGCCGGATGGAAAGCTGACTGCGAAAAATGCGGATAT
CAGCGGCCATATCAACGCTGTATCTGGCTCGTTTACGGGAGAAATCAATGCCACCTCCGGTAAGTTTTCTGGCGTGATAGAAGCAAGAGAGTTTGTCGGTGATATCTGCGGCTCAAAAGTCA
TGCAGGGCGTGAGCATCAGGGCGACGAACGACGAACGCAGCACCTCAACACGGTATACCGACAGCGCCACCTATCAGATTGGGAAAACCATCACGGTGATGGCTAACTGTGAGCGTAATG
GTGGCTCCGGTGCCATCACCGTCACGATAAATATTAACGGCCAGGTGAAAACGGCGGAGGTTATGCCGTATACCGCAGGTATTCCGGCCATGTATCAGACCGTCGTCTTTTCGGTCTACACCA
CTTCACCTGTCGTGGATATCAGCGTCTCTCTGAGGGTTCGTGGGCAGTACACCACGTCTGCTTCCGTCTGGCCGTTGGTGATGGTTTCCCGGTCGGGGAACAACTTCACAAACTGA
>BDIOMP_13935 Tail assembly protein
TTGCCCGGTCTCCGGCAGAAACTGAACGACGGCTGGTATCAGGTACGCATAGCCGGAGACGATGTTACGGCTGATACCCTGACAACCAGCCTGCATGACCCGCTGCCGCCTGGCGCGGTG
ATTCATATTGTGCCGCGTCTGGCCGGGGCCAAACCTGGCGGGGTGTTTCAGGCAGTGCTTGGTGCGGCGCTGATTGCCGTTGCCTGGTGGAACCCGGCAGGCTGGCTGGGAGCGGCGGC
GGTATCCGGCATGTATATGACCGGGGCGTCGATGATTCTGGGCGGTGTGGCGCAGATGCTGGCACCCAAACCCAAAATGTCCGAAATGAGGCAGACCGATAACGGCAGGCAGAACACGTA
TTTCTCGTCGCTGGATAACATGGTTGCCAACGGTAACACGTTGCCGGTGCTGTACGGCGAGATGCAGGTGGGGTCACGCGTGATTTCCCAGGAAGTCAGTACCGCTGATGAAGGAGATGG
TGGTCAGGTTGTGGTGATTGGCCGCTGA
>BDIOMP_13940 Tail tip assembly protein K
ATGAAAGAACAGGATATTCTGGCGCACGCCCGACGGTGTGCGCCTGCGGAGTCGTGCGGCTTCGTGGTGAGAACACAGGCGGGAGAACGGTATCTCCCCTGTGTGAATATTTCTGCCGCG
CCGGAGGATTATTTCCGTATGGCGCCGGAGGACTGGCTGAGGGCTGAAACGCAGGGGGATATTGTGGCGCTGGTTCACAGCCATCCTGGCGGCCAGCCGTATCTGAGCGATGTGGACCGC
AGGCTGCAGGTTCAAAGCGACCTGCCGTGGTGGCTGGTATGCGCCGGCCAGGTACATAAATTCCGCTGTGTGCCACACCTGACCGGACGACAGTTTAAACATGGGGTTTTTGACTGTTACA
CGCTGTTCCGTGATGCCTATCATCTGGCGGGGATTGATATGCCGGATTTTCACCGGGACGACGACTGGTGGCGGCATGGTGACAATCTCTATCTGGATAATCTGGAGACGACGGGATTTTAC
CGTGTCAGCGCAGCAGTGCGCAGCCCGGCGACGTGCTGATTTGCTGCTTTGGCTCCTCCGTTCCGAACCACGCAGCGATTTACTGCGGCGACGGAGAGCTGCTGCACCATATTCCTGAAC
AACTGAGTAAACGTGAGAGGTATACCGACAAATGGCAACGACGCACGCACTCCATCTGGCGACACCGGGCATGGCGCGAATTTGCCTTTACGGGGATCTGCAACGATTTTGCCGCCGCGTC
AGCCTGCAGGTAG
>BDIOMP_13945 Phage tail component L-like protein
ATGCAGGACATTTTGCAGGATACGCTGAACGAAGCCGCTAAACTGGCGCAGTCCGCCAGGATCACTTTGTGGGAAATCGATCTGACACAGTCTGGCGGTGATCGTTATTTTTTTGTAACGA
GGCAAATGAAAAGGGGGAGGCGGTTACCTGGCAGGGACGGAAATATGACGTTTATCCTGTAGACGGTTGCGGATTTGAAATGAACGGCAAAGGCGCAGCTGCGCGCCCGTCACTGAAG
GTATCCAATCTTTACGGTATGGTGACCGGAATGGTGGAGGATTTGCATAGCCTGGTTGGGGCGACGGTCATCCGCAGGATAGTATATGCCCGGTTTCTCGATGCCGTGAATTTTCAAAGCGG
CAACCAGGAGGCCGACCCGGAGCAGGAGTCTGTAAGCCGCTGGGTGATCGAGCAGTGCAGTGATCTGACGGCGGTAAGTGCGACATTTGTCCTGGCAACACCGACTGAAACAGACGGAT
GTGTCTTCCCCGGGAGGATTATGCTGGCCAATACCTGTACATGGATATACCGCTCTGACGAATGCGGTTATACGGGACCAGCTGTCGCAGATGAATTTGATAACCCTACCGCCGATCCGGCAA
AAGATGCCTGCAGCCGCTGCGCCCGGGGATGCGCCCTGCGTAACAATACCGGAAACTTTGGCGGTTTCCTCTCCATTAATAAACTTTCACAGTAA
>BDIOMP_13950 Phage tail protein
ATGGAAACATTTAACTGGAAGATCCGCCCTGATATGACAGTGGAATCAGAACCAAAAGTCACCTCCATAAAACTGGGTGACGGGTATGAACAACGGCGTCCAGCCGGGCTGAACAACCATC
TGGCGAAGTATAACGTAACGGTCCGGATTCGTAAGGGAGAACATCAGAACCTTGAGGCATTTTTATCCCGCCACGGTGGAGTGAAATCCTTTCTCTGGACGCCGCCTTATACCTGGACACAA
ATTCGGGTGATTTGCCGCAAATGGTCGATTAGCGTTGGCTCTCTTTGGGTGACTGTGACCACGACTTTTGAACAGGTTGTTATCTGA
>BDIOMP_13955 Phage tail tape measure protein
ATGGACCAGATAGCGAACCTGGTCATTGATTTAAGTATCGACAGCGCAGAGTTCCGAAACGAAGTTCCGCGCATTAAAAAATTGCTGAACGATGCGGCTGGTGACTCAGAACGTTCAGCGG
CCCGGATGCAGCGTTTTCTGGATAAGCAGACGGAGGCGACGCGCCGGACGTCCGCCAGTCTGGAGCAAGTGACTGCCAGCAGTACCGCGTACAGTTCCGCTGTGGAGAAAAGCGCAGC
GGCCAGTACGCGTCTGGCGGCGGATGTGGATCAGACGCGACAGCGGGTGGAGGCACTGGGAAGGAAACTGCGTGAGGAACAGGCGCAGTCAGCGGCTGTGGCGGCAGCACAGGACA
GGACAAGTGCTGCTTTTTACCGCCAGATTGACAGTGTAAAACAGTTAAGCGGTGGTCTGCAGGAGCTGCAGCGTATCCAGGCGCAGGTACGACAGGCGAAAGGACGCGGAGATATCTCA
CAGGGCGATTATCTGGCGCTGGTGTCTGAAACCGCCAGGAAGACCCGTGAGCTTACCGATGCCGAAGCGCTGGCCACGCAGAAAAAAGCACAGTTTATACGCCGCCTGAAAGAGCAGAC
GACGGTACAGGGCCTCTCCCGTACCGAGCTGCTGCGGGTGAAGGCGGCTGAACTGGGTGTCAGCAGCGCCGCAGATATTTATATCCGTAAACTGGAGCGTACCGGAACTGCCACCCATAC
GCTAGGACTGAAAAGCGCTGCTGCCCGTCGTGAACTGGGCGTGCTGGCTAGTGAGCTGGCCCGTGGGAATTTCGGGGCACTGCGGGGAAGTGGTATTACGCTCGCTAACCGCGCCGGGT
GGATCGAGCAACTGATGTCTCCGAAGGGCATGATGCTCGGCGGGCTGGCTGGCGGCGTGGCTGCTGCTGTTTACGGGCTGGGCAAGGCCTACTATGAAGGCGCTAAGGAAAGCGAGACG
TTCAATAAACAGCTTATTCTGACCGGGAGTTATGCCGGAAAAACCACAGGCCAGCTTAATGCGATGGCGAAGTCGCTCGCCGGAAATGGCGTCACGCAGCACGACGCTGCAGGCGTGCTG
GCACAGGTGGTCGGTAGCGGAGCGTTTACCGGGCAGGCAGTGGCAATGGTATCCCGTACCGCGACCAGAATGCAGGAAAACGTTGGACAATCAGTGGATGAAACCATCCGCCAGTTTAA
ACGCCTGCGGGATGATCCGGTGAATGCGGCGAAAGAACTGGACAGGACACTGCATTTTCTGACCTCCACCCAGCTTGAACAAATCAGGGTACTGGGCGAGCAGGGAAGAGTGGCTGATG
CCGCGAAAATTGCCATGTCCGCGTATTCGGAAGAAATGAATAAGCGGATGGGAGACGTACACGACAATCTGGGCTGGATTGAAAGAGCATGGAATGCTGTCGGTGATGCGGCGAAGTGG

GCATGGGATCGGATGCTGGATATCGGGCGGGAAGATACGCTCGATGAAAAGATCGCGACACTGCAGGAAAAAATCGCGCGCGGCAGAAAAACGCCCTGGACGGTGTCTTCCTCCCAGAC
TGAATACGATCAGCAGCAGCTGAACGAACTTAAGGAACAGAAACGCCAGAAGGACCTGCTGGATGCGAAGGCGCAGGCAGAGCGTAATTATCAGGAAACGCAGAAACGTCGGAACGAG
CAGAATGCCGCGCTGAACCGGGATAATGAAACTGAATCCCTGCGGCACCAACGGGAGGTGGCGCGCATTACCGCCATGCAGTATGCCGATGCTGCTGTACGCAATGCCGCACTGGAGCGC
GAAAATGAACGTCATAAAAAGGCGTTGTCACAACAGGCGAAAAAGCCAAAGACTTACCACAACGACGAGGCCAGGCGACTGCTTTTGCAGTACAGCCAGCAACAGGCGCAGACTGAAG
GGCAGCTTGCCGCCGCGAAGCTTTCCACGACCGAAAAAATGACGGAAGCGCATAAGCAGCTTTTGTCATTTCAGCAGCGCATCGCTGATTTGTCCGGTAAAAAACTGACGGCGGATGAAC
AAAGCGTACTGGCACATAAGGATGAAATCGCGCTTGCGCTACAGAAGCTGGATATCTCACAACAGGATTTGCAACACCAGAATGCCCTTAATGAACTGAAGAAAAAGACGCTCACATTGAC
CAGCCAGCTCGCTGACGAAGAATCCCGCGTCAGGCAACAGCACGCAATGGCGCTGGCCACAATGGGTATGGGCGATCAGCAACGTGGCCGATACGAAGAGCGTCTGAAAATTCAGCAGC
ACTACCAGGAACAACTGGAGCAGCTTAAACGCGACAGCAAGGCAAAAGGGACATACGGTTCTGACGAATATCGTCAGGCGGAGCAGGCGCTGAAGGGCAGTCTCGATCGCCGGCTGGC
TGAGTGGGCGGATTACAATGCGAAAGTTGACGCTGCGCAGGGAGACTGGACGCTGGGGGCGTCGCGGGCGCTGGATAACTTTCTGGCGCAGGGCGGCAATGTGGCAGGCATGACGGA
GAACGTTTTCACAAACGCATTTAACGGCATGGCGGACAGTATCGCGAATTTTGCCGTGACCGGAAAGGGCAGTTTCCGGAGCCTGACGGTCTCCATCCTGGCTGACCTTGCAAAAATGGA
GGCACGTATTGCGGCTTCTAAACTGTTGGGTTCAGTGCTGGCAATGTTCGGCTTTGGCACATCGGCAGGCGGCAGTACACCATCAGGGGCATACAGTTCTGCGGCGCTGTCGGTTATTCCG
AATGCGGACGGCGGCGTGTACCGTTCGGCAGGACTCAGCCAGTACAGCGGCAGCATTGTTAATCGCCCGACATTTTTTGCTTTTGCCAAAGGTGCCGGGGTGATGGGCGAGGCAGGACC
GGAGGCAATATTACCACTTCGTCGTGGTGCTGACGGTAAGCTGGGTGTCGTGGCAGCCGGTTCAGGAGGGATGGCGATGTTTGCGCCTGAGTACAACATTGAAATCCACAACGACGCCGG
CAACGGACAGATTGGTCCGCAGGCATTACAGGCCGTATATAACATTGGAAAAAAAGCCGCCATTGATTTCTGGCAACAGCAGTCGCGTGACGGGGGTATTGCTGGAGGAGGGCGATAA
>BDIOMP_13960 Phage tail assembly protein T
ATGCGGCTCGCGCGTGAGTTCCGGCGACCAGACTGGCGGCAGATGCTGGCGGAAATGAGTGCGACAGAGCTGGGTGAGTGGGCGGAGCATTTCGGGAAGAACAGCTTCAGTGACATGT
TGCTGGATGCGGAGTTTGCAACGCTGAAATCGCTGATTTCCGGACTGGTTACAGGCACGCATCACGATGCAGAAATGTTCAGCCTGATCACTGATCCTGAGTCGTTGCACGAAAAAACGGA
TGATGAACTGATGATCCTGGGCGAAGGTATTACCGGAGGTGTCCGCTATGGACCAGATAGCGAACCTGGTCATTGA
>BDIOMP_13965 Phage minor tail protein G
ATGTTTCTCAATACAGACACCTTTAACTACGGTGGGCATTCCATCGTGCTCAGTGAGCTTTCTGCCCTGCAACGTGTGGATTATCTGAAGTTTATTCAGCAGCGGACGGCAGACTATGACGCA
CAGCCTGAAACCCTGACGGAAGCAGAGCGTCAGACAGAATTTATGCAGATGGGGGTGGATATTAATGCATGGCTGGTATCCCGCTCCCTGTGTGAAAGCAAAAAAGAGGAGGAGGCCCG
CGCCCTGTATGAGTCCGTCAGACTGGAATGGTCTTATGAGGCGCTGGGACGTGGCGCTGATATGGTTCTGTCCCTGAGTGGTATGCGTCTTCCGGCATCGCAGGAAGACGACAGCGGGAGT
GAAAAGGACACGACCACGCCGGAAAAGTCCTGA
>BDIOMP_13970 BIG2 domain-containing protein
ATGGGAACACCAAACCCACTGGTAAAAACGAAAGGCGCCGGAACCACATTCTGGCTGTATACCGGCAGCGGCGATGCGTTTAAAAATCCACTGGCTGATGATGACTGGCTGCGACTGGCA
GGTATTAAGGATCTGCAGCCCGGAGAAATGAGTGCAGATGCGGAAGACGATGACTATCTTGATGATGAAAATGCCGACTGGAAAAGCACTACGCAGGGGCAGAAAAGCGTCGGTGACAC
CACGGCCACGCTGGCCTGGAAACCCGGTGAGGCCGGACAGAAAAAACTGGTTGAGCTGTTTGACACCGGCGAAGTTCGCGCCTTCCGTATCAGGTATCCTAACGGGACGGTTGATGTGTT
CCGCGGCTGGCTGAGTTCACTGGGTAAAACCGTGACGTCCAAAGAGGTGATGACACGCAGCGTAAAAATCACCGGCGTCGGGCGTCCTTCTCTTGCGGAGGAGGATACACCTGACGTAGT
CAGAGTATCCGGCGTGACCGTTGCGCCGGCCAGTGCCACGGTGGCTGCCGGAGCCACCACCACGCTGACATTTACGGTAAAACCTGATAACGCGTCAGATAAAACGCTGCAGGTTGCGAC
CGCCGATCCGCTGATCGCCACCGTCACGCTGAAGGATAATGTGGCCACGGTTAAAGGCGTGAAGGCGGGCAGCGTGAATATTGTTGGTATCAGCAGTGACGGCAGTCTTGTCGCGGTGGC
AGCAGTGACAGTGACGGCGTCATAA
>BDIOMP_13975 Phage tail protein
ATGAGCAAACACACATTAATCCGCCGGGCCGTTCTGGAAAAGCTGGAATCCGTGACTGGCGCACCTGTCACTCTTTTTGATGGACTTCCTGCTTTCGTAGAACAGGAAGATTTACCCGCAAT
AGCTGTCTGGCTGACAGACGCACAGTATACAGGCCTTATGACCGATGAGGATGACTGGCAGGCTACTCTCCATACGGCAGTTTTTCTGAGGGCTCAGGCTCCTGATACAGAGCTTGATATCT
GGATGGAAGAAAAAATCTTTCCTGCGCTGGAAGAGGTTAGTGGTCTGGAGCGCCTTATCGATACCATGACCCCGCTGGGTTATGACTACCAGCGTGACAGCGAAATGGCAACGTGGGGGA
TGGCAGAAATTACTTACCGGATCACCTATACCAACTGA
>BDIOMP_13980 Phage tail protein
TTGAAAGGGCTTGAAAATGCGATCCGGAATCTGAACAGCCTTGACCGGCAGATGGTTCCCCGGGCCAGTATCTGGGCTGTGAATCGCGTGGCGCAGAAAGCTGTTTCAGTGGCAACCCGT
AAGGTGGCGCGGGAGACTGTCGCCGGAGATAACCAGGTAAGAGGACTTCCGCTGAAGCTGGTTCGCCAGAGGGTGAGGTTATTTAAAGCCGGTACAGACGGTAAACGCTCTGCCCGGAT
ACGGATTAACCGGGGAAACCTTCCCGCCATAAAGTTGGGCGCTGCACAGGTCAGGATGAGCAAACGGAGGGGAAAACTGCTGTATCGTGGAAGTGTGCTGAAAATCGGGCCATATCTGTT
CCGGGATGCCTTTATTCAGCAACTGGCTAACGGACGCTGGCATGTTATGCGACGCGTTAACGGGAAAAACCGTTATCCAATTGATGTAGTGAAAATTCCTCTTTCCGGACCATTGACTCAGG
CATTCGAAAGCGCCACACAAAGCTTGATTGACGAGGAAATACCGAAGCAACTGGGGTATGCCCTGAAACAACAACTGAGGCTTTATCTTTCACGATGA
>BDIOMP_13985 Phage tail protein
ATGTCGCAGTCCGAAAACCTGTTTGATACCGCGATTTCTCAGGCTGATGATGCCATCCTCCGGGTGATGGGAACGGTAGCAACAATAACCTCCGGCGTTCTGGCAGGGGCCACGCTTACGG
GCGTATTTGACGATCCTGAAAGTGTGTCGTATGCCGCCGGAGGTGTCCGGATTGAGGGGGACAAGCCTACATTTTTTGTCAAAACATCCCTGACAGTCCATCTGAAGCGCCCGGACACACT
AACCATTCTCGGTGACACCTTCTGGGTGGATCGCATCACTCCGGCTGGTGGAGACAGCAGTATTATTCTGCTGGGCAGGGGGGAGCCGCCGACGGATAACCGGCGCAGGACGGGAGGAA
TGTTTGAAAGGGCTTGA
>BDIOMP_13990 DNA packaging protein
ATGGCAACAAAAGAAGAGAATATACAGCGTCTGCGGGAGCTTGCGACGCGGCTTGGTCGTGATCCGGATGTGTCCGGGAGCGCCGCTGAACTCAGCCAGCGTGTCATGGAATGGGAAGA
GGAAGCGGAGGCGGAGGCGGAGCATTTGCCTGCTGTGGAAAATGACAGTGATGAATCCATAGTGCCGCCCGGGATCGGGCAAAGATCCGAACGGGTACTTATCAGGGCGCTTCGTACAC
TACACATCTGCGCCATCGATCCGGACAGTAACCGGGAACTGGATATGGTTATGGCGGGAAACCCGGCGCGTATTTCGCAACACGATGTGGACGAGCTGATTGCTGCAGGACTTATTATTGAA
CTGTAA
>BDIOMP_13995 P21 prophage-derived major head protein
ATGGGATTGTTTACCACCCGCCAGTTGCTGGGTTATACCGAGCAGAAAGTTAAATTTAACCCACTCTTCCTGAGCCTGTTTTTTCGCCGTACTGTGACATTTCCTACCCAGGAAGTCATGCTG
GACAAAATTACCGGAAAAACACCGATTGCCGCTTATGTATCTCCGGTGGTAGGAGGGAAGGTTCTGCGTAACCGCGGCGGGGAAACGCGCGTACTGCGTCCGGGGTATGTCAAACCGAAG
CATGAAGTTAACTATGCGCAGGTTGTTGAGCGTCTGCCGGGTGAAGACCCGGCCAGGCTTAACGATCCGGCCTACCGTCGTCTGCGCATTCTGACCGATAACCTGAAGCAGGAAGAGAATG
CCATCGTCCAGGTGGAGGAGATGCAGGCCGTCAGTGCGGTGCTGAACGGGAAATACACCATGCAGGGCGAGCAGTTTGACACCGTGGAGGTGGATTTTGGTCGCTCAGCCGGGAATAAC
ATTATTCAGGCCACAGGTAAAAAATGGTCAGAGCAGGACAGGGAAACCTTTGACCCGACTTATGATCTGGATATGTACTGCGACCAGGCATCCGGTCTGATCAATATTGCCGTGATGGACGG
GAAAGTCTGGCGTCTGCTGAACGGTTTTAAGCTGTTCCGTGAAAAACTGGATACACGCCGCGGTTCAAATTCTCAGCTGGAAACGGCGGTGAAGGACCTTGGGGCTGTGGTGTCGTTCAA
GGGGTATTACGGAGATTTGGCCATTGTGGTGGCTAAAACATCCTATGTTGCTGATAACGGGACCGAAAAGCGTTACCTGCCTGAAGGGACTCTGGTTCTGGGAAATACGGCGGCAGAAGG
TATCCGCTGCTATGGTGCCATTCAGGATTCACAGGCGCTCGCGGAGGGTATTGTTGCCGCCACCCGTTATCCCAAACACTGGCTGACGGTGGGGGACCCGGCGAATGAATATACCATGACGC
AGTCTGCGCCGCTGATGGTCCTGCCGGACCCGGATGAGTTTGTCATTGTCACTGTCGGTTAA
>BDIOMP_14000 Head decoration protein
ATGTCATTTACCACCACTATTGAGAAACGTGCGGATAACCGCATTTTCGCCGGTAACGATCCGGCACATACCGCAACGGGAGTCAGCGGTATAACGGCGGCCACACCGATGCTGACGCCCCT
GATGCTGGATGATACCACCGGGAAACTGGTGGCCTGGGACGGGCAGAAGGCCGGAACGGCAGTCGGGGTTCTGGCCCTTGAGCTGGACGGGTCGGAAAACCTGCTGACGTACTGGAAG
AGCGGCACCTTTGCCACAGAATCACTGGCATGGCCGAAGAGTGTGGATGCCATTAAGCAGGCAAATGCATTCGCCGGAAGCGCCGTCAGTCACGCCGCTTTACCGTAA
>BDIOMP_14005 Serine peptidase
GTGCAACGTAATCTCCCGCACATCATCAGCCAGGCAACCAGTGCTCCGTTGCTGCTTGAACCCGCCTATGCGCGGGTTTTCTTTTGCGCGCTGGGCAGGGAGTCAGGCATTAACAGCCTGC
ACATTCCCGGTAATAACGAAAGTCTGGATCAGTCGGATATGGCACTGGTCACAGGCGATTTTATGGCGACCGGAAAGCCGCAGGCACGTTTTTATCAGGTAGTGAACGGTATTGCGGTATTA
CCCGTGACCGGAACACTGGTTCATAAACTCGGCGGAATGCGTCCCTTTTCAGGGATGACCGGATATGACGGTGTCACTGCCCGGCTACAACAGGCGGTTTCAGATCCGGAGGTAAAAGGC

ATTCTGCTGGATATTGACAGTCCCGGCGGTCAGGCTGCCGGGGCGTTTGACTGTGCTGACATGATTTACCGGATGCGCGAACAGAAACCTGTCTGGGCACTGGCAAATGAAACAGCCTGTT
CGGCGGCCATGTTGCTGGCGGCAGCCTGTTCGCACCGCCTTGTGACCCAGACGTCCAGAATGGGATCAATTGGTGTGGTGATGGCGCATACCAGCTACGCCGAAAAACTGAAACAGGAAG
GGATCGATATCACCCTTATCTATTCTGGCGCACACAAGGCTGATCTGACGCCCAGCCAGAAATTACCGGAAAGCGTCCATGCCGACTACCAGCAGCGAATGGACGAGGCCAGAAAGATGTT
TGCAGAAAAAGTGGCCCGGTACACGGGGTTGTCTGTCGATGCGGTAATGGCGACGGAGGCGGCAGTGTATGACGGGCAGGCCATTATCACTACCGGACTGGCAGATGGAATGGTGAATG
CTGCTGACGCCATCGGCGTGATGGCAGAAGCTATCAACAGTAACAAGACAGGAGGCACTATGCCTGAATTAAGTGCAGCTGACGCTGTCACGCAGGAAAACCAGCGCGTAATGGGAATTC
TGGGTTGCCCGGAGGCCAGGGGGCATGAGGCACTGGCACAGATGCTGGCCGGGCAGCCGGGAATGAGCGTTGCTCAGGCGAAGTCTATTCTGGCTGCCGCCGCGCCGACGGACACGAC
CAGCACCGCTGACCGTATCCTTGCCCTGGAAGAAGCTGGTGGTCGGGAAACACTCGCACAGACACTGGCGGCCATGCCAGAGATGACGGTGGAACAGGCCAGAACCATTCTGGCAGCAT
CGCCGATCGCTGCGGCAACGTCACTTCATGATGCCGTGATGGCGCTTGATGAGGCGAAAGGCCGTGAAGAGTTGGCGGAAAAACTGGCTGTCATGCCTGGTATGACCACAGATCAGGCCC
GTGACCTGCTGGCTGCCGCGCCGGACAAATCCGGTAATGCGGGGCTGAGCATGAACAACGCATTTGATGCTTTCATGCAGTCTCATTCCCCGGGCCCCATATCCGGCGGCAAAGGCCACAG
TAATGATACCGAAACGACGTTGTTGATGAGTATTCCCGGTACTTCAGCCACCTGA
>BDIOMP_14010 Portal protein
GTGCAATGGTCGCCGCCGGGCACCTGTGGGGGTGAGACTGTGAAGCGCACCCCGGTTCTGGTGGATGTTCACGGCACGCCGCTGCGGGAAAGTCTGGGATACACCGGCGGGGGGATCG
GTTTCGGCGGACAGATGGCTGACTGGATGCCCCCGGCGGAAAGCGTGGATGCCGCGCTGCTGCCTTCGTTGCGCCTCGGCAATGCGCGGGCTGATGATCTGGTCCGTAATAATGGTATTGC
AGCAAATGCGGTGGCGCTGCACAAGGATCATATTGTCGGACACCTGTTTCTTATCAGCTATCGTCCAAACTGGCGCTATCTTGGTATGCGTGAGAGCGCAGCGAAGAGTTTTGTGGATGAGG
TTGAAGCTGCATGGACAGAATATTGCGATGGTATTTTTGGCGAAATGGATGCCGAGGGGAAGCGTACTTTTACAGAGTTCATCCGTGAAGGCGTGGGCGTTCACGCCTTTAATGGTGAAAT
TTTTCTCCAGCCTGTCTGGGACGCTGAAACCACGCAGGTTTTCCGTACCCGATTCAAGGCTGTCAGTCCGAAACGGGTGGACACGCCGGGGTATGCCCGCGGAAACCGCCAGCTTCGCGC
AGGAGTGGAAACGGACCGGAATGGAAAAGCCCTCGCCTATCATGTCTGTGATGATGACTGGCCGGTGGCTGGTGGGGAACGCTGGACCCGTATTCCTCGTTTTCTGCCGTCCGGACGACC
CGCGATGTTACATATTTTCGAGCCGGTTGAGGACGGACAGACGCGCGGTGCCAATCAGTTTTACAGTGTGATGGAGCGGCTGAAGATGCTTGATACCCTGCAGGCAACGCAGCTTCAGTCC
GCGATTGTCAAAGCCATGTACGCCGCCACGATCGAAAGCGAACTCGATTCCGAGAAAGCCTTTGAATACATCACGGCGGCAGATAACAAAGATACGCCCCTTGTTAACATGCTGGCAAATTA
TGCCCGCTATTACAGTACCAACAGTATCAAACTGGGCGGTGTAAAAATTCCCCACCTGTACCCGGGTGATGAGCTGAATCTGCAGACTGCGCAGGATTCCGATAATGGCTTTTCAGCGCTGG
AGCAGGCGCTGCTCCGGTATATTGCCGCCGGGCTGGGGGTCTCTTATGAGCAGCTTTCGCGTGATTATTCTCAGGTCAGCTATTCCAGCGCCCGCGCATCTGCCAATGAGTCCTGGCGCTATT
TCCTGGGGCGGCGCCGGTTCATTGCCGGACGGCTGGCGACACAAATGTTTTCCTGCTGGCTGGAGGAGGCGCTGATACGGGGAGTTATCCGGGCACCCCGGGCCAGGTTTTCCTTCTGG
GAGGCCCGATCCAGCTGGAGCCGCTCGGAGTGGATTGGTGCCGGACGTATGGCGATTGACGGACTCAAGGAGGTTCAGGAATCCGTGATGCGTATTGAGGCCGGGCTGAGTACCTATGA
AAAAGAACTCGCCATTATGGGCGAGGATTACCAGGAGATATTCCGCCAGCAGGTCAGGGAATCCGAAGAACGGCGGGCAGCCGGACTTTCGCGTCCGGTATGGATCACTGATACCTATCAA
CAACAGATCGCGGCGAGCCGTCAGACGGAGGAGGAAAAGCGTGCAACGTAA
>BDIOMP_14015 Phage tail protein
ATGGCAACAATCACTGAGCTACAGGAAGCCCGCGTCGCGCTGCATGACCTGATGACGGGAAAACGGGTGGCGACGGTTCAGAAAGACGGGCGACGGGTTGAATTTACCGCGACATCGGT
AGGGGATCTGAAAAAATATGTCGCGGAACTTGAGGCGTCACTGTGCAATGGTCGCCGCCGGGCACCTGTGGGGGTGAGACTGTGA
>BDIOMP_14020 Terminase large subunit (Gp2)
ATGATTTCCGGAGAGCGCAGGGCGAATAATGCCAACAGAGCCATAACTAACGGGCTGATAGCGCTTCATATTCCCGTACCGCTTACCACCGTGCAGTGGGCTGATAAGTATTACTATCTGCCA
AAAGAGTCCTCCTACACCCCCGGCAAATGGGAAACGCTGCCGTTTCAGGTAGCGATAATGAACGCGATGGGGTATGAACTGATCCGCGTTGTAAACCTCATTAAGTCTGCCCGCGTGGGCT
ATACCAAAATGTTGCTGGGGGTGGAAGGCTATTTCATAGAGCACAAGTCGCGCAACAGCCTGCTGTTCCAGCCGACCGACTCATCCGCTGAGGATTTTATGAAATCCCACGTGGAGCCGAC
TATCAGGGATGTTCCTGTATTGCTGGAGCTGGCCCCCTGGTTCGGGCGTAAACATCGTGATAACACGCTCACCCTGAAACGCTTTTCTTCCGGTGTCGGGTTCTGGTGCCTCGGCGGTGCAG
CAGCCAAAAACTACCGTGAAAAATCGGTGGATGTGGTCTGCTATGACGAATTGTCATCTTTTGAGCCGGATGTCGAGAAAGAAGGTTCGCCGACGCTGCTGGGGGATAAACGTATTGAAG
GTTCTGTCTGGCCTAAATCCATTCGGGGCTCCACACCAAAAGTCAAAGGGTCATGCCAGATTGAAAAGGCGGCAAATGAATCGGCGCATTTTATGCGTTTTCATGTACCGTGTCCGCACTGT
GGCGAAGAACAGTACCTTAAATTCGGTGATGGCAGTACGCCGTTCGGTCTGAAATGGGAGAAAAGCAAGCCGGAGACGGTGTATTACCTTTGTGAACATAATGGATGCGTGATCCGTCAAT
CGGAACTTGATCAGAAAGCAGGCCGCTGGATTTGCGATAACACAGGCATGTGGACACGCGATGGACTGGCTTATTTCAGCGCGTCCGGTGAGGAGGTTCCGCCGCCACGATCCATTACCTT
TCATATCTGGACGGCTTACAGTCCCTTTACCACCTGGATACAGATTATTTATGACTGGCTGGATGCGCTGAAAGATCCAAATGGTGTGAAAACCTTTATAAACACCACGTTGGGCGAGCCTTAT
GAAGAGGCGGTGGCCGAAAAACTCAGCCATGAGCTTTTGCTGGAAAAAGTGATTCATTATGCGGCGCCGGTTCCGGAGCGGGTGGTGTATCTGACCGCTGGTATCGACTCCCAGCGTAAC
CGTTATGAAATGTATGTCTGGGGCTGGGCGCCGGGCGAAGAGGCTTTCCTTATTGATAAGCAAATTATCATGGGACGGCATGATGATGAAGATACCCTGCAGCGTGTGGATGCCGTCATTAA
TAAAAAAATATCGTCATGCTGACGGGACGGATATTTCCATTTCCCGTATCTGCTGGGATATCGGCGGTATCGATGCAGAAATCGTCTATAAACGCTCAAAAAAACACGGCATTTTCCGCGTGCTG
CCTGTCAAAGGGGCCTCCGTTTACGGAAAACCCGTTATTACCATGCCTAAAAAACGCAACCAGAGCGGGGTATTCCTGTGCGAAATCGGTACTGATACTGCCAAAGAAATGCTTTACGCCAG
AATGGGGGCGGTTACTGCGCCTGCCGACGAAGCCACGCCTTATGCGATCCGCTTTCCGGATAATCCGGATGTTTTTACGGAGGTGGAAGCGAAGCAACTGGTAGCCGAAGAGCTGGTGGA
GAAACTGGTTAACGGAAAATTCCGGCTGTTATGGGATGCCAAAGGACGTCGTAACGAAGCGCTGGATTGTCTTGTCTATGCCAGTGCAGCGTTACGGGTGTCTGTGCAGCGCTGGCAACTG
GATCTGGAGGCGCTGGCGACATCAAGGAAAAGCGAAGAGCAGGATACCCCGACACTTGAACAACTGGCCGCAATGCTGGCAGGAGGAGTTAATGGCAACAATCACTGA
>BDIOMP_14025 DNA packaging protein
ATGAACGTGAACAAAAAAAAACTGGCTGAAATTTTTGGTTGTGACGTCAGAACTGTCACAGCCTGGCAAAGCCAGGGGCTGCCACTTGTTTCCGGAGGAGGAAAAGGTAACGAAGCAGT
GTTCGACACCGCGGCAGCGATTTCATGGTACGCGGAGCGTGATGCGTCTATTGAAAATGAAAAGCTGCGTAAAGAGGTTGATGATTTACGTGCCGCTGCGGAATCAGATCTTAATCCCGGC
ACCATCGACTATGAGCGCTACCGCCTGACAAAAGCCCAGGCGGATGCGCAGGAACTTAAAAATGCTGAGCGCGAAGGGCTGGTTCTTGAGACCGAACTGTTCACCTACATCCTGCAACGG
GTGGCTCAGGAAATAGCAGGGATACTGTCAAGGGTACCGCTGGTATTACAGCGCAAATATCCTGATCTGTGCCAGTCGCACATCGATGTGGTCAGAACGGAAATCGCCAGGGCGTCAGGCA
GGGCCGCCACGATAGCGGATGTGGAGAAGTGGACCGATGATTTCCGGAGAGCGCAGGGCGAATAA
>BDIOMP_14030 hypothetical protein
ATGAAATTCAAGAGTATCGCTAAAACTGTTTTTCTTTTTGCACTGCTAACCTCAGCTGGCTTTGCAACTGGTAAAAACGTGAATGTCGAATTCGATAAAGGACAAAATAGCGCCCGCTATTCC
GGCGTAATAAAGGGATACGATTACGATACATATAACTTCCAGGCCAGAAAGGGGCAGAAAGTACATGTAAGTATTTCGAATGAAGGCGCAGATACCTACCTGTTCGGGCCAGGAATTAGCG
ATTCCGTTGACCTGTCCAGATATTCATCTGAACTGGATGGCAATGGCCAGTACACGCTACCGGCGTCCGGAAAATACGAACTGAGAGTACTTCAGAGACGTAATGAAGCCCGTAAAAACAAA
GCGAAAAAAATACAGCGTCAATATTCAGATAAAATAA
>BDIOMP_14035 Lipoprotein
ATGCGCCGTATATTAGCCACCGCTGCCGCACTTTGTCTTGGCGGCTGCATTACTGTGTATGGTCCGGTTAAAACGGGCGGGCAGCAACAGCAGGACAGCCAGTCCGGGCAGCAGCCAGGG
ATGAGCGAACAGATATCAACCTCATTCATCGGTAACCGTAAACCGGATGAGTTGCTGAATGCCGTGGCGCTGTATTTCAGGGAGAAAGCCATCACAGCCAGTGTTAACGACCAGACCACAG
GAATTATCGCCGGTACCGGGGATGACCCGGAACTGAGTTCGTTGTATCTGGACTGTTCACTGTTACCGCAGACACAAAATATCCAGGAGCATTACCGTATCGTCGCGCAGGTCTGGAGTGCC
GGTGAAGGCAGTAATGTTTCGGTAATGGTGACAGGCACTGCCGGACTGGATACTGCCGACGGTAACGATAAGGTGAAGCCGGTGGAGTGTAAAAGTACCGGGATATTTGAAAAAGATTTG
CAGGAACGGCTACGTAAGTAG
>BDIOMP_14040 Spanin, inner membrane subunit
ATGATGTTCAACTGGAAAACGATGTTTGTTGGCCTGTTGCTCGTCTCGCTAATTGTTGCCGGTCGGCTGGCAAATCACTACCGCAATAACGCTATCACTTACAAGTACCAGCGTGATACTGCTA
CTCACAACCTGAAGCTGGCGAACGAGACAATTACCGACATGACGAAGCGCCAGCGTGACGTTGCCGCCCTCGATGCAAAATACACAAAGGAACTAGCTGATGCACAAAACAGGAATACTG
ATTTGCAGCGCCGCCTTGCTGCTGGTAGCCGGGTGCGCGTCGAAGGGCGATGTTCAGTGCCCACCCGGACCGAAACCGCCAGTACCCGCCGCGTGGGCAATGCTGCCACCGTCGAACTCT
CTCCAGGTGCTGGACAAAACGTTCTCAATATCCGCGCCGGAATCATCAGCGACCAGGAAAAACTGAAGTATTTGCAGGAGTACATCCGGACGCAATGTCTGAAATAA
>BDIOMP_14045 Endolysin
ATGATGAGAATCAGTGAAAAAGGCATTACCCTAATCAAAGAGTTTGAAGGTTGTAGCCTGACAGCTTATCCGGACCCGGGAACGGGGGGAGATCCCTGGACGATTGGTTATGGCTGGACC
CACTCTGTTGACGGTAAGCCAGTTAAGCCCGGAATGATGATTGACGAGGCTACTGCCGAGCGCTTGCTTAAAACTGGTTTAGTCGGTTATGAAAATGATGTGTCCAAACTGGTTAAGGTCAA

GTTGACGCAAGGCCAGTTTGATGCGCTGGTGTCGTTCGCGTACAACCTCGGCGCCCGGACATTATCCTCATCAACTCTGCTGCGTAAGCTAAACGCTGGTGATTACGCCGGCGCCGCTGATG
AGTTCCTGCGCTGGAATAAGGCTGGTGGCAAAGTACTGAACGGGCTTACCCGTCGGCGTGAGGCGGAGCGTGCTCTGTTCCTGTCATGA
>BDIOMP_14050 Holin
ATGAAAATGAATGACAAGACTCCTGAATTCTGGGCTGCGGTTTTGACCGGACTCAAAAATGCGTGGCCCCAGATACTTGGGGCGTTAATGGCCGGACTCATTGCCTACGGCCGACTGATATA
CGACGGCGCCATCCGTAAAAATAAATGGCTTGAGGGCGTCCTGTGTGGCGCTCTTTCCTTATGTGTCACCAGTGCGCTTGATGTGGTAGGCCTGCCGGTTTCCATTTCGCCTTTCGTTGGCG
GAATTATTGGCTTTGTCGGTGTGGACAAGCTGCGCGAAATCGCAATTAGCGCACTCAAAAAACGTGCAGGGGTTAATGATGAGAATCAGTGA
>BDIOMP_14055 Type III secretion system effector protease GtgA
ATGGATCCTGATATCGAAATTGATGATGATACCTATGATGAATGCCGTGAGGTGCTATCACGCATACTTGAAGATGCATACACTCAAAGCGGGACATTCCGCAGACTGATGAATTATGCCTACG
ACCAGGAATTGCATGATGTAGAACAACGCTGGTTGCTGGGAGCCGGAGAAAACTTTGGTACTACCGTAACTGATGAGGACCTGGAGAGTTCAGAAGGCAGAAAAGTGATTGCCCTCAACC
TGGATGATACAGACGATGATTCAATACCAGAGTACTATGAAAGTAATGATGGCCCACAACAATTTGATACAACACGCTCATTTATTCATGAAGTTGTACACGCGTTGACTCACCTTCAGGACAA
AGAAGACAGTAATCCAAGAGGCCCGGTAGTCGAGTATACCAATATCATTTTAAAAGAGATGGGTCACACATCACCACCAAGAATCGCCTACGAATTTAGTAATTGA
>BDIOMP_14060 Holin
ATGTCCGAATCGATTTCTGGTACTGGGTTAGCAGGTGGCATCCTGACAGGAGCCAGTGTCTATGGACTGCTGACCTGTATTAGCTCAGACCTGAACTGGTTACTGTGTTGTAGCATCGTGGG
ATTTTGCATTTTTTGA
>BDIOMP_14065 Lipoprotein
ATGAAAAACACCGCTCTGGGTAAGTTCATTTTTATCGTCGGCACCGCGTTACTGCTCGGTGGCTGTAGTGGCATGGTCATGCCTCCCTATGCCACCCACGGTACATCGGTCGGAATCATTGCG
CCGGCGGGAGGCTATAGCGAGTGGCACACGGATAGCCGCAACCACACCACAGGAGACAGTCACAGCCAGTCACAGGGAAACTGCACCCAAAGTGAAGATAGCCAGCTCAACGAAAATG
GTCTCACACGGACACACCAAAGCAACTGTAACACCCGTAGTCAAACCCACAGCAGTAGCACCAGCAAAACCCGCTCCAGCAGCGTCGGTTTCAGCGTCGGGGGGCCTGTTGGTGCTAGCA
TAGGGTTGATTAAGCAGATGGAGTCGATGAACCGTGCGCCAGCCAACGATATGAGTAGTAATGAGATGTTCAAGAATTTCGGTTTCTAG
>BDIOMP_14070 Bacteriophage protein
ATGAAAATGCACAACGATCCCCATTCAATGGACTCACAATCTATTTTTGCTGGCTCACAATTACTGCCAATGGAAAAAACTTCTCATTTGGCTCTGAGCGTCGGATTTCGCTCTCACTTAGCGT
GA
>BDIOMP_14075 isrJ Hfq binding RNA
AGTAAATCTGATATCGTCGCCATAGCTTCAATCGTCGACCAAACAAATTCAAGCCCCGCCATCGTGCGGGGCTTT
>BDIOMP_14080 Antiterminator
ATGAACACTCAATACCTCCAGTATGTACGTGAGCAGCTAATGGTAGCGACAGCCGATTTAAGCGGGGAGACTAAAGGGCAGCTTTTGGCCTGGCTGGAGAACGCGCAATTCGACACGAAA
AACTATCCCCGAAAAAAACAGCGTATATGGGACGAGGAAACAGAAAGCTGGATAACGTTAAATAACCCGCCAATCCCCGGCAAGCAGTCGCTGGCGAAAGGAAGCGCTATCCCGCTGGTG
AAGCCTGTGGAATATTCCACTGCCTCATGGCGCCGGGCGGTTCTTTCACTCGATGAACACTACAAGGCGTGGTTGTTGTGGAATTACAGTGAGAATACCTGCTGGGAACACCAGGTCGAAA
TAACACGTTGGGCGTGGTGCGAGTTCAGACAGCAGCTTGCAGGGAGGAAGATGGCTGGCAAGACAGTGGAACGGCTGAAGAAACTTATCTGGCTGGCGGCGCAGGATGTCAGAGAGG
GGCTGGCCGGACGATACGTCTACCAGCAACAGGAGCTTGCCAGCCTGTGCGGGGTTAAGCCTGACAACTGGAGCCACAACTATGCGGACTACTGGCGCGCGATGAGTAACATCTTTAAGA
GGCTTGATACCGAATCTCTGCTTTGTCTGGTGAAAACAAGATCACAACAAAAAGCGACCTTTTCGCAGCAGGGTATTGCAAAAGTCAATTAA
>BDIOMP_14085 YlcG family protein
ATGAAACCAGAACTGATCGAGATACTCCGCATGCGCTGGCAGCGCCTCCGCATTTACCGCCGTCCGGGGTCGGTGTTGGTTGACTACCGCATCCTGCGCAATTTTGTTCGTATTTATCAGTTC
ACAGGATTTACTCAATGA
>BDIOMP_14090 Protein NinG
ATGGCTAAATTACCGCGCCGTAAGTGCGCAAACAAAGAATGCCGCCAGTGGTTTCACCCGATACGCGAGGGGCAGATCGTTTGCTCGTACCAGTGTGCCAGCGCCGTCGGCAAAGAACAA
ACCAGAAAAGCTCACGAAGCCGCGCAACGTAAGGCGCAATCACTTCAGCGCGCCGCTGAGAAAAAAGAACGCGCCGCCTGGCGCCAGCGGAAAGCCGCGGTTAAGCCGCTGAAGCACT
GGATTGACTTGACGCAGCGCGCCGTAAATGACATTTGCCGCGAAACCGAACTGGCAGAAGGACTCGGTTGCATCTCCTGTGGAACGAAGACGGCGTTCGCATGGCATGCAGGCCATTACA
GGACTACGGCCGCCGCGGGGCATCTGCGCTTCACTCGCTTCAACATCCATCTTCAGTGTGATGTCTGCAACGTCTACAAATCAGGGAACATCGAAGCATATCGTACCGCGCTGGTTGAGCGT
TACGGTGAGGCGGCGGTGCTGGCACTCGAGAACAATAACACCCCGCACCGCTGGACGGTCGAGGAGCTGAAGGAAATCAGGCTCGCGGCTCTGGCGGATCTGCGTGCGCTAAAAAAGC
TGGAGGCCGCATGA
>BDIOMP_14095 Phage protein
ATGGCGATGAAATACTCCTGGTTCCATCATCACGACTGTACAACCGAGCAGGCCGACACTCTGATATCGGATTATCAGAAGCGGGGCATAAGGACAGAAAAGAGCCTGAACCCTGACTTCAT
TACCTGGACTGTCAGCGCGAAATTACCTGAATATGCACACCGGGTGCGGACGCCAAAATCCTTACGCCAAAAGGTCTGGGGGTGA
>BDIOMP_14100 DUF1367 domain-containing protein
ATGGCGCACGAATTACAACTCATCAAGCAGTCATCTGGAATTCTGATCCCCGCAACGCCGGAGACCAGTGATATTCTGCAATCAAAAATCAAACTCGGCGCCGTGCTGGTGGCTGAGTTCCG
TCAGGTGAGGAATCCTGCATTCCATCGCCGCTTTTTCGCGTTGCTTAATCTTGGGTTTGAATACTGGGAACCCACCGGCGGCGCCATTTCTGCCAATGAACGCAAACTGGTAAACGGTTATG
CAAAGTTTCTCGCTGCATATGGCGGGAATGAGGGCGCATTACTGGATGCGGCTGAACAGTATCTGGAACAGATTGCAAACCGCCGGGTAACAAACGGAATTAGCCTCTGTAAATCTTTCGAT
GCATACCGCGCATGGGTGACGGTTGAGGCTGGTCACTATGACGCCATCCAGTTACCGGACGGCACCCTTCGCAAACATCCCCGCAGCATCGCTTTTTCCAGCATGGATGAGGTCGAATTTCA
GCAGTTGTATAAATCCGCGCTTGATGTTCTCTGGCGCTGGATTTTATCACGTACATTCCGTACTCAGCGCGAGGCCGAGAACGCCGCCGCCCAGCTCATGAGCTTTGCGGGGTGA
>BDIOMP_14105 DNA damage-inducible protein I
ATGAAAGTTGAGTTAACCATTGATCGCACTAAAAAACTTCCTGACGGTGCGGAGGCGGCACTTGAGAAGGAGCTGCTTAAGCGGCTTAACAATCAGTATGAAGATTGCAGTCTGGTGATTC
GACGAGCCGGATCTGATGGACTTAACGTTTATGGCGCTGCCAAGGACGATAAAAAGCAAATTGAAATGATCCTCCTGGAGACCAGGGAGAGCGCTGACGATTGGTTTTATTAA
>BDIOMP_14110 NfeD domain-containing protein
ATGAAAAAAAGAACAGGTTTAAAGTATGCATTATATATATTGTCACTATGGCTATTGTTTGTATCACTTTTTATAATGTCATATGACAAGAATCTTTTTGTAAGTATATCAACATATCTGGACAGCA
AAGATACAGGAACATTACTTTCAAGCATCACGCCTAAAAATATAGTTTTTATTTCCAGTTTTGCAATGATAGTAGCAGGTGTTTTAATTTTTCTATATTTATTACTTTCCTTTAACTCTGGTTGGTC
GGTAGCGTGCACGGTTTCTGATGTTAGAAATGAAAGTCATGAGCATCTTGAATTTTTAACTACCTATGTTATGCCTTTGGTATTTACTGATGTGAACAGCAAGAGGACTATGCTGAATCTTTTG
ATCATGATAGTAGCTATAGGGATGATTTATGTAAAAACAAATCGTTTTTATTCTAATCCATCGTTAGCATTGCTTGGATTTAGAATTTTCAAAGCTAATATAAATGATAGAGGAACCAAAGAATTT
GTAATCATTTGCCATGGCGAAATTGATAACAACAGCAGAATAAAATATATCAAACTAGATAACAATACATGTCTTGCTAAAATCACTCAATAA
>BDIOMP_14115 DUF4868 domain-containing protein
ATGTTCACAGCAATAGATAATATATTAAATTCAACCCAACTCTCCGGTGAGGCTTATTTTGTTGCAGAACACCAAGGGCAACTAGATATTTTCAGAGTAGCTCTAGAACCAGGTGCAGAACAG
AAATTAACACAGTCTTTCAGTCGATCACTTAAGCGTGATGTTGTTGACCCAAACACTGGACAGAACACCTTGCCGTTAGTTTCTTCTCTGCTAAGTCGAGATAAGCAGGTGCATGAATATGAT
CACCAAGTAATAAACTATCTTCCACCTGCTCTAGCAAAGATGGCGGACGTGCTTAGCTTTGGAGTTAATAATACACCCACTGACTTTGACTTTGCCCAGCAAAACCTATCAACCGTGAAAGGA
ATAGTTTATTATCTTTGTGATGGTCAAGGTAACGGAGTTGTTGTTTACCAACACAAATATCCAATCGCACTACATAAAAAAACAAAATTGTCATATTTCTCTGCGAATGGTAGAACCCTTGATGA
GGTCACCCATGATAGCATTGACATAAATGGGAATGTGGATTTCTTTTACTTTGACAACAAGTATTATGCACTAAATATCAACTTACTTGAAAGAGCATATGGCCTTGAACAAGTCATAAATAATT
TAGCGGCAAATGCAACCCCTCATATAATCGCACTGAACATCTTGGACGTATCAAATCATCCGAACCCTGCAGACATTTTTAATGACATGCATCGAAATAGAAACTTCATGCGCGCAGACTTGCCAC
CACAGCAAACAGTCCACTACTACAGAATGGAACTATTAACATAGCCAACATTCAGACATTAATTCAAAATTTCCCGATTCTTGGAAGGAATATAATAATCAATCAAGCTGGATTGATAGAATTAT
CATCTAAAAAGCAAAAACTATATTTCATTCGCCTGCTAAATAATGAGGCATCTTTCACAGCATTAAACCTAGAGCCTTTTCTTGCGGTCGGAAAAGACTCAGCGGCATAA
>BDIOMP_14120 DNA helicase

ATGATGGACCAGCTTCTGCAATACGCCACAAACCGGATCATTGAACTGGAAAGCCTGCTGCTGGTGGATATTCAGGAAACCGTCTGGCCAACTGAGGTGAAAATGGTTTTTTCTCAGGTTG
ATAACGCCGGGGATCTCCCGGCTCACCACCAGCGGCGCCTACTGCATCACATTAACCGGATGTGGCTGGAGAAAATGCCGGAAAGCGACACGTTTCTTGCGGCGAGTGAACCCGGCCCGA
TTGCTTTTGCATTCAGGTAA
>BDIOMP_14125 hypothetical protein
ATGACCACTATTACCAGAGAACAGCAAAAGCAGATTTTAATTGATACGGCTAACCACGTAATCAGTCGTGATAACACGTCACCGTATAGCGAAAACCTGCGCGAACTGGCGCGTATAGCGCT
GGCCCAGCTTATCGCTGAACCTGTGGAACGTGATGGTGCAGATGCCGATCCTGTTGTGTTCACCGACGAACGAAATCTTCATCATATTGCCAGGGGTCGAGAAACCTCTTTGATTTGGGGTA
AACAAAACCAGGAGGTGGGGGATATCCCGCTCTATCGTCACGCCCAGCCCGCACCGGTAGTGCCGGAGGAAATACCAAAAGGCCTGGCGGATCAAATTGTCAGTCTGCTGGCTCATAACAT
TGGCGATAAGTTTTTGGCTCAGAAAATCTGGAACGCCTGTCGCGCTGCCATGCTTCAGGGTAATCAAGCACCCGTCAAGCAACCATCAAGCAATTCAGAAAACGTGCCGGTTATTTCGGATG
GTTGGGTGATGGTGCCGGTTGAGCTGACGGATGACATGATCGCAGCCGCTATGAACTGCGACGATGTGCTGTTCAATAGAGATGAATCATTCTGCGTACAGTTCGGGAATATCTACGCAGCC
ATGATTGCAGCAGCACCGCAGCAGGAGGCTGAATGA
>BDIOMP_14130 TfoX-N domain-containing protein
ATGAAAACTAAAAAATATGATGAGAGAAAGGACCTTGACCTTTGGTTTGGGTTGTCATATGCAGCGTTTCTCGTGATGCCACGTGTAGCAATGATGCAAATGCCGGAAGAGTGGCGGGAGA
AAATGGCCGAACTTCTCAATCAGTACGATGAAACCATTGATACCGCGGCGTTTGGTGTAAAAGGTTGTCGGGTTAATGCGCTAACTGGTGACGGCAAGTTAATGAAAATGCCGGCAGAGTT
ATTGAATTACCGCCACCCACAGCCGGAAACGGTAGAGGCGCTTTTACTGTCAAAAGGTGAGGGCTAA
>BDIOMP_14135 Ead/Ea22-like family protein
ATGAGCAACATCGACAAACGGGCGCTTCGTGAAGTGGCTGAGAGGGCTACGCCGGGGAATTGGCGCCGCACCTCATCACTGTTCAATGGCATCACGGTAACGCCATTTTCTCTTTGCGGTG
AAGAAGTGACGTTGGCCCATACTGTTGAGAAACGTGACGCGGAATTTATCGCCGCCGCTAACCCTCGCACCATGCTGGCGCTGCTGGATGAGAATATTCAACTCCAGCGGGGGAAAGACG
CAATAGAGGCCGTAGCGCTGGCGCTGCGTGATGATATGCGGGATGCGCGCGAAAAGTTGGAAGCCGCAGAACACCGCATAGCAGAACACCGCAAGGTGCTAAATAGCATTGCAGCAGTA
GCCCGTCGCTACCTGCCTGATTATGACGAACATCCTGAAATTCAGGCCGCTGACGAATTACTTGAGAGCGCTGCTGGCATCGGCGTGAAGGGGGGAGTGA
>BDIOMP_14140 Gifsy-2 prophage protein
ATGGCCCGACCTAAAACACACAGCGAACGGACGATTATTCTTGAGCGGATTATCGGTCTGGTGAAAGAGCAGGGGCGCATCACGACGAACGACGTCGTTGCGATGTTCGGCGTGCACCGA
ACCACGGCGGAGAAATATCTGCAGATCGCGCTGGAGCGAGGCGGCTTCATTCGTCATGGCCGCTGCGGCATTTTCCGAGACCAGCGTGCGGTGATTGATTATGACCTCAGGCGATACAGCA
GTAGTCAGGTAACGGGATTTTCAGCGCTGCCGGTGCTGGAGAAAAGCCCGGTAATGCAGGTTTATGGAGCATCCAAAATGAGCATCAACAAGGGGGGGAGCCCAATGA
>BDIOMP_14145 Uncharacterized protein YdaV
ATGAAAAACGTAATCGGTACTGGCAGTGCGCTTGATCGCCTGAAAAGAATTATCCCAGCCAGTGTGCAGCCGAAATTCTCGACTGCTGATGAGTGGCGGGCATGGCAGGAAGCCGAAGGG
CGTAAACGCAGTGAAGAGCTTGACAGGATGAATCAGAAATCCCGCACCGAGAAGATTTTCGGGCGATCTGGCATTCAGGATCTCCATCGTAGCTGTACGTTTGCTAACTACGAAGTAAGCG
GGGAGGGGCAGCGAAAAGCGTACACGATGGCAAAAAGTTATGCCCAGAACTTCGGTAGTGGATTTGCGAGCTTTGTGTTCAGCGGTGGTCCGGGAACCGGGAAAAACCATCTTGCGGC
GGCAATCGGAAATCATCTGCTGGCCGGCGGTCATAGCGTTCTGGTGGTAACCATTCCTGACCTGATGCTCAGGGTTCGTGAGTGCTACGACGGTGGGCAATCAGAAGCGTCCCTGCTTGAT
GACCTTTGCAAAGTTGACCTGCTGGTACTGGATGAAGTCGGTATTCAGCGCGGAAGCAGTGGTGAGAAGGTCATTCTCAATCAGGTTATCGATCGCCGTCTCTCATCGATGCGACCTGTTGG
CGTTCTGACGAATCTTAACCACGAGGGGCTGTTGGATTCACTGGGCGCGAGGGTTATCGATCGCCTCCAGATGGACGGAGGGATGTGGGTGAATTTTGACTGGGGAAGCTACCGGAAAA
ACGTTAGCCACCTCCGGATTGTGAAATAA
>BDIOMP_14150 Phage-rep-O domain-containing protein
ATGTCAAATACCGCTGAAATATACAAATTCCCTGCGCCGGTACCGACGCAACAGGAGTGCCGTATGGCTGATCTGGAAAATGGCTATTTACGTTTAGCTAATCAGATCCAGGACGCCTTGTGT
ATCGTTGAACTATCGGGGCGTGAGTTCCGTGTTTTGAATGCGATTATCCGGCTGACTTATGGCTGGTCGAAAAAATCAGATCGTATTGCCAACAGCCTCATTGCAGATAAGACAACACTGAA
GGTAAAGCACGTATCCGAAGCGGTGCTGAGTCTTGCCTATCGTAACATCATTATCCTGCGCCGTATTGGTCAAACAAGATACATAGGGATTAATACAAACCTGGATAAATGGGCTTATTCCAAG
CCACATTGCTCAAAATGTCCGGTGTCTTTTCCTGATGATGAAATTGCCACATGGATTATTTCTGTACTCGAAACCAGGGATAGTTATCCCCGAAAAGGGGGAAGGGCATCCCCGAAAACAGG
GATAGTTATCCCTGAAAACAGGGATAGCGTTTTACCCATTCAGCCATCCCTGAAAACGGGGATAGTTATCCCCGAAAAGAGGGAAGGGTATCCCCGAAAACAGGGAACACCAAAGACATT
ATTCCAAAGACAAATATAAAAGATCTAACCCCCTTTAATCCCCCTAAGGGAAAAGTGAAGTTTGATCCGTTGAGTATTCCTGTTCCCGAATGGCTGAATGCTGCGTCGTGGAACGAATGGGTC
ACCTACCGCCAGCAATCCGGAAAGCCCATAAAAAACCGAACTGACGGTAACAAAAGCTTTCAGGCTTCTGAAGGAGTGCCTGGATGAAGGCCACGATCCGGTAAACGTCATCAACACAAGC
ATTGCCAACGGCTACCAGGGACTATTCAAACCGAAGTTCGCTCTCAACGACCGAAGAGCTGGCAGAGATGTGAACCACATTTCTGCGCCAGACAAAACCATTCCTACCGGATTCAGGGGGT
AA
>BDIOMP_14155 Regulatory protein CII
ATGGAGAACGCAATTGCACGAAAGTTAGACCCACCAGAAATCAACCCGATTGAGATAGAGAGCGTCCTGCTCAACCGGCTTGCATCAGTAGGGCAGAAATCATACGCCGAGCATATGGGCA
TCAGCGAGTCGACAGTCAGCAGGCGTAAAGCTGAGGGATATTTCTGCAACATGGCGAAAGAGCTGGCTTTTCTTGGGATTCAGGCCGCGCCACCGGAGGCGGTACTGGTATCCAGAAACT
ATCTCACAGCTGTAGAGATTCTCGCTGATGCAGGGCTAAAGGCTGAACGAGCCAGGCCGGATGCGCTGGGGTGGGACTGA
>BDIOMP_14160 Helix-turn-helix domain-containing protein
ATGATGAATATGAAAACAGAACAGCATGAAAATTTTGTTCGTAGGCTCCAGCTCATTCAGGATCAAACGGGTTGGAACTTATCTGAGATTGCCAGGAGGGTTATGGTCTCTCCACAGGCGGT
TCAGCAATGGGCTAAAGGCGATACAACCCCTCGCGGCGAGAGGCTGAAAAGACTCGCAGCCGTTACAGGGAAACCTGAACATTGGTTTTTCATGCCACTTGATGCAAATGAACCGAGTAA
TTCTTTATCTGAAATTCCAACCTCAAGCAGCCGGGATATGCTGGATGACAAAGAAAAGGCTCTTTTGGCTCTTTTCAACCAGATGCCAGAAGCAGAGAAAAACCGCCTCATTGTCCATGCCA
AAGCCACTCTACAAGAGCTTGACCTTCTGAAGGATGATGTCCTCAGTATCATCAAAAATATAAGAGAATAA
>BDIOMP_14165 DNA breaking-rejoining protein
ATGGAAATCGTAAAAATCGAAATGAACCTGAAAGCAGTTAATAAGAGCATTGCTTTATTCAATTGCGAAAAGAAAGTCTCAGGCGTTATTCACTCAAATTCAACTGGCGAAACTACTGTGATT
CTCGACGGTGGATATGTACTCGGAAAGTTCGACTGTCCTCATTGTGCTGTAGAAGCCATTTCGCTGCTCACAGTCAAGGTAAGTGATGGAGAACAAGCAGGGTTTGGTAATTACCGAAGTTA
CAAGATTGATTACTCAGAAAAATTTTATCAGACCATCCATTAA

Prophage 3

>EGKIKD_01785 Integrase
ATGCCAAAACTCACAGATATGCAGATCCGCGCATGGATAAAGGCGGGAGACAGATTTGATGGCAAGGCAGACGGCAACGGCCTTTATATTTGCTACCCCAAAAACTACACAGTCCCGTTCT
GGCGATTCCGCTATAAGCTCGCAGGAAAGCAGCGGGCTATGGTTATAGGCTCTTACTCCGAACTGTCACTATCAAAAGCCAGAGAAACAGCAAAAGAGCTGTCAGCCAGAGTTGCTCTTGG
CTATGACGTAGCAGCAGAGAAACAGGAACGAAAAGCTGAAGCACTGGCAAAAATGGAGGCTGAGAAAAACGCCATGCGTGTTTCAGATCTGGCCTCCGAATACTTCGAGCGCCAGATACT
TCCGAGATGGAAACATCCCGATATCCTTCGCCGGAGAATTGATAAGGATATTAACCCCTGCATTGGGCATATGAAGGTAGAGGACGTTAAGCCACGTCACATTGACGACATGTTGAAAGGTA
TCGTTGACCGAGGAGCACCAACGATAGCAACAGATGTATTACGCTGGACACGCCGTATCTTTGACTATGGGATAAAACGCCACGCTCTGGAGATAAACCCTTGTTCAGCGTTTGAAGTATCT
GATGCTGGTGGTAAAGAGGTTTCCCGCGACCGTTGGCTAACTCGCGATGAGCTTATCAGGCTATTCGAGGCTATGCGCACCGCAAAAGGATTCAGCCGCCAGAACGAACTGACATTTAAGC
TATTGCTGGCACTATGCGTTCGTAAGATGGAGTTATGCGCCGCACGATGGGAAGAGTTTGATTTAGATGGTGCGGTATGGCATTTGCCGGAAGAACGCAGCAAAAACGGAGACCCTATTGA
TATACCTCTACCTTCCCCAGCCGTTGAATGGTTGAGAGAGCTACACACCTTTTCATGTAATAGCGCATGGGTGCTTCCAGCCAGGAAAATGCAAAACAGAATGATCCCACATATTCAGGAAAG
CACTTTACCCGTAGCACTGGCTAAGGTTCGCGCCGAAATGCCGGATGTGCCTAATTTCACGATTCACGACTTCCGACGCACCGCACGTACTCATTTAGCAGCGTTGGGTGTTGATCCTGTCGT

GGCGGAACGATGCCTCAATCATCGCATTAAGGGCGTAGAGGGGATTTATAACCGCCATCAGTATTTTGATGAGCGTAAAGCAGCACTGGCACTGTGGGCTGATCTGCTGGTGGCACTGGAA
AGCGGAAAAGACTACAACGTAACGCCTCTCAGAAGGGCGAACTAA
>EGKIKD_01790 int-alpA RNA
ATTGAGCCTAGCTCGACGGGGCGAAAAGCGGGAAGCCTTACCGCCTGGCTCAATCACAAAATAAGGC
>EGKIKD_01795 Phage protein
ATGAACGACTCTCATGAAACAACTGCGGTGCATAGAAATTTATGGCAGGAATCAGGTATCCCACCTTTGGACTACTGCACGATAGAGCGAGCATCAAAATTATTAAATTGTGAAATTGATGAT
ATATGGCATTGGAAAGATAGACTACATATAGACTTTTATGTCTATGCTGACTTTCTTGATTTCATTGAAGCTGATGCCACCATCCACTTCAAAAAAGATGCTGATTTTATAACTGATTTATTACTTA
TGTACCCAGAAGAAGTAAGGTTTACATTTCCTTTTCTTATTACCAGCGCAAGCAACGAAGTTTATATCCCAGCATCTGAAAGCGGTGCTTATGAGCTATTTTATGCACATAAACCACTGACAGT
TGAAGACAGAGACAATAAAAATGTTTTTGAACAAATTGGCAAAAACTCTTTTATTGTAAGAAAATCAATTGCATGTGGATTGTTGCGTGTACGCGGTAGTACAACACGACATTATCATTCACA
AGAATGTGGAGAGCCATATGTGCGACTTGAAGCAACATTTGATTGTGATCTATATAAATTCAAAATGCAAAGTTCCCAGATTCACCTGAGTAGCCTGTCAGATAAAAACATTTATATAACAAAG
GCTGATTTAGAATACCTTCACGACGCAAAGGTTAATGGAAGCTTTGACGCAGCTAAGAGCAAAAAACGATACGAAATGGCGGTAGAACAGGAACTAATACCTGAAATAAAAAATAGCAACC
ATAATGCCGAGAGACATGCTGAAAACAGGGAAAAACTTTTCAAATCAGCAATATATCTTTTGTCAAAGTATCCTGATGAATGCCGTGGCAAAAAGAAAGAAATAAGCCCTGAGAAGTGGCG
GGATTGCATAGTGGCTCATAAAGACGAAATTCCACCGTTAGCAATCACAAATGAAGATGTGATTTTGAGACACTTACGTGCTGCCGCCAACGGTAAGGGGTAG
>EGKIKD_01800 hypothetical protein
ATGAACACCGACACTATTCCTACAAAAGGCTATATCAGACGCTTCCGCCTAGCTGAATTGCTGGGCGTTAGTGTATCCACCATCGACCGTAAAGTAAGAAACGGTTCACTACCACGTCCGGTA
AAGCTGGGAGAAAAAATAACTGCGTTTGATGCGGTTGAGATTCACAACTGGCTGGCAGAACGCCGCGGGAAGGTGGCCTGA
>EGKIKD_01805 GIY-YIG nuclease family protein
ATGGAAAAGAAAAACCGCCCATTACAGGCGGCTAATTCAGATATTCGCGTATCTGATGTTACGCCCCTTACAAAATCCCTTCAAGCACCAAAGCGCACACCGAAAAAGCATCGTGCCAGAGT
CTATATGCTGCGTACTGGTATAGAGGGATGGACAGAAAATGACATTCTTCGCTACTGCCGTCTGTCTTCTGGTCGTAACTATGCAACAGAGTTAGAACGCCAGCTTGGAATCACTCTGGAGCG
TATCGACGAAAAGAATCCTGATGGTATCGGAACACACCTTCGCTACCGTTTCTCCTGCCGTGGTGATGTTCTGAAAGTGATCACTCATGTTAACCATCTTGCGAACATAAATGATCACAACGGA
CTTTCTCAGCAGGAAATTGCCGACATTCTGAAACTCTACCCGAACGCGTTTAACGCCGCCTAA
>EGKIKD_01810 hypothetical protein
ATGAACATCGAAAAAAGCAGATTAATTTCTGAGGCCGCCCCTCATCTGAACGCCTCTCTGGGCACAATTAACGGTAATGAATTTGCCGCAATTGTCCCGGTTATTCCTGGTCATATCGGTGGG
CGTGAAACCAATATTGTTAGCGCAAAAGCGTTACACAAAGCGTTGGGCGTGGGAAAAGACTTCTCTACATGGATCACTGATCGCATCTCTGAATATGACTTCACCATTGGGCACGATTACTCA
GTCCATAAAACAATTTCCCCAAATTTGGGGAAAAGCCCGAATGGCGCGGCTTACAGCAAGATTAAGCAGTCTGGCAGACCCGGCAAAGACTATCTGTTAAGTGTCGGAATGGCGAAAGAA
CTGGCAATGATCGAACGCAATGATCAGGGTCGCGCTATCCGTCGTTATTTCATCCAGTGCGAGGAAGAATTACAGCGTAGCGTGCCTGAAATCGCCGCCCGCTATCGTCGCCAGCTAAAAGC
CCGTATCAGTGCCGCAAACAACTTTAAGCCAATGTGCGATGCGCTGAATATGGCCCGTGCCGAGACGGGGAAAACGACGCAGCAACACCACTACACAAACGAGAGCAATATGATTTCTCGT
ATCGTTCTTGGTGGGCTAACTGCTTAA
>EGKIKD_01815 hypothetical protein
ATGAACGCAGAACAGCTTGAGCACCTCTCATATCTCGAAAGCACCAATATCACGTTAATTGATATGGGCATGGAATATGAGCAGCGCAAAGGAGAACTCACCCGCCTGTCGCAACGCTGGCT
CGCCAAGCGTCTGGAGGCGGTCCATGTTTAA
>EGKIKD_01820 DUF4222 domain-containing protein
ATGTTTAAGCCGACAGGAACACCACAACCCCAAAAACGCTACAAAGATGCCCACGGAGCACTCGTTACTGTCGAAAGCGTGTCTCACAACCGAGTGACGTTTTATCGCGACGGGTATCAAT
CGCCATGCGTACAACCGCTGGCACGTTTCATGAAGGAGTTCGCGGAGGTTAACAAATGCTAA
>EGKIKD_01825 isrK Hfq binding RNA
GCACCTCATAAAACGGGTGCCGGGATTGGCGTCCTGGTAAATCGAATGGCGACATATGACGCGCCGAGCGTCTTTTTT
>EGKIKD_01830 C4 antisense RNA
ATCAATGGTGGCTCAGGCAGGGGCTTCTTCGGAAGCGCCGGTATCCATTCGAGCCGGTTACGCCAACCCTGTCTGGGCTACCACCAGC
>EGKIKD_01835 Host cell division inhibitor Icd-like protein
ATGCTGGCTACTACCCCTACCCAAAAACCGCAATTTATCTGGATTATCGCCGCAGTTCGCCGCGATTGCCCGACAATTACCGCCAAAATTCATCATATTGCTGCCGAGTCTGAACGCGATGCTC
GCCGTTCTCTGGTGCGCGATCACGTCTGCTTTTTTGCTGGTCGTATCCGCATGGAGGTGGCACATGATTAA
>EGKIKD_01840 Prophage protein
ATGATTAAAACCTACGATGTGCATATGGATCCCCTCGAACGCACAAGCCAGATCATCACACTGACAGAAGTGATTAATGACATTCTGGTGAGCAACTCTCCATCACGAGACGAAAAACTGAA
GGCGTTACTCGCGATATTGGATCTCGCCGTTCGTGACGTTCATTTCCTGCTGGAAGGTGGACAAATGCCATGCAAAACGGGGGCAACCAATGAATAA
>EGKIKD_01845 hypothetical protein
ATGAATAACTCAATTAATACCCCTCGCCTTACGTCCGCACTTCAATTAATCGAGCAAGCAGCGGCTGTCCTGGTTGCTGTCAGTCTTTCGGCTGAAGAAATGGACGCTGCTGATGTCGTGGAT
GCGATTAAAGCGTGCTCATCTTTGGTTAACGATGCCCGTGCCGAGCTGGTAATTCTTGGGGGTGAAAAATGA
>EGKIKD_01850 hypothetical protein
ATGAATATCAACTTAATTTATCGTCATCCGTGTGAGCTGGAAATTGAATCATTGCTGGGGCGCGAAGAGCCATATCCAGACACATTCACTCCCGCAGATTGCGCGACTGAACGGCTTACCAGA
GCGCGCACAGGTCTGGTTCATGTGATGAATGAGATTATTCCCTCGGTGGGAGGGGAACAGGCGACAGTAATCAATAGCTGGCTACAAAAAGTTACCTCCCTGATAGATATCAGTTTAATCGA
TGTGGAGAGTGCGAAATGA
>EGKIKD_01855 Prophage protein
ATGACCAACATCCAGCTCATTGAAGCGCAATGTCGCATCGAACAGGTTCAGACTGTTTTAGGGTTCTGGCTTGAAGGGGCCAGCCCCAGCAACAGAGACAAGTTAATGATTGGCGCGGTTA
TGTCACTGCTCAATGGCGTACCAGAAGCTATTCAGGAAGCAGACGAATTGTTGGGCAAATATGAGTTACAGAATCATTCAGGCGAGGCGAAACATGAATAA
>EGKIKD_01860 2-polyprenylphenol hydroxylase related flavodoxin oxidoreductase
ATGAATAATTTCTTAACCTTCCATGCAGAAGCAACGCCTGACGGCGTAAACATCATGCACCGCAGCAACGATGGCATGACAGAACGCGTTGAGACCGTCTCATATATTGATGCCGTAAATCGT
CTGGATGCCGGGGATTATGACGATAAACCAGATGAAGGCATGTTTATACATCTCGCTATTGCCAGTGGCGGCAACCAGGGATATTTCGATTACACATCACAGCATCACGTGATTATGTGGCGC
TGGCTGATAGCAACAGCATTCATCAATGAAATGAGAAAGGAAAACGGCACCGTCAGCATTATTGATGACAGTGGCAATCATTCCGTGGTTTCTGTTTATTCCAATGGCATCGTCGCCATGCCG
CTGTATCCAGTAGCAGAGCGCCTCGCTATGGCAAACAACATTGAGGGCGCAATGATCGAGAAATATGGTGTTGATGTCGGAACAAAGAATGCCATCATTTTTTACAGCAACATGTTCGATGTC
GAACAGGGAACACTCACTTCGTTTGGGCGAGAAGTGCTTGCCGATCTTCACAACAGCTTTATTGCCGAACTAAACGAAAACGGCATCCCAGAAGCACCAGTGACGCACTAA
>EGKIKD_01865 Helicase
ATGCGAAACATTGATCTTATCCGTCAGGTTATCAGTGCGTCTGAAAACAACTGGCCTCATGTGCTGGGCTGCCTGAACATAAATGTCCCTGACTCTCCGCGCCGTCATGCTCCCTGCCCTGCAT
GTGGGGGCAAAGATCGATTCCGGTTCGATGACAACGGGCGCGGTAGCTTCATCTGTAATCAGTGCGGCGCTGGTGATGGGCTGGATTTAATTAAACGCGTAAATAACTGCGACACAACAGA
GGCGGCGCTTCTTGCCGCTGATGTTCTGGGTATTGATTACCGGACAACGGAAACACCAGAAGCCACCAGCCAGAAACGGGAACAACTGGAAGCCGAGCGCCAGCGACGCGAACAGGAG
CGCCTGAAAAGGGCAGAGAAGGACGAACAACAAAGACGGGATACGTTTTCCCGTCAGTTTGATGACATGCGCAGAAAGGCTGTAAACGGCAAATCTGATTATCTGGTTGCGAAAGGGGT
AGGTGATTTTACATTCCCCGTGTTGCCAGATGGATCTCTATTGCTGGCGCTGGTGGATAAATCCGGCGCAGTCACCGCAGCACAGACTATTACCTCACATGGTGAAAAAAGACTCCTGACAG
GTTCGGCAAAGCGGGGGGCATATCACGCCATAAACGCACAGAAACGACCTCACAGCATCATAATTGCTGAGGGGGTGGCTACCGCCCTGTCGTGCCATTTAATTCGCCCTGACGCAATGAC
AGTGGCAGCAATCGACGCTGGCAACCTGTTGCCAGTAGCGGAAGTCATGCGCAGAACATATCCACAGGCACAAATCATCATTGCCGCAGATAACGATCACCAGCAAGGAAACTCCGAAAGT
GGAGGGATCAACACGGGGAAAGATGCCGCAGAGAGGGCCGCTATTTCCGTAGCTGGCTGGGTGTCTCTGCCACCGACTGACTATAAAGCCGACTGGAACGACTATCACCAACAACACGG
GCTGGCGGCAGCCACAGCAGCATTTAAAGATTCGATGTACCAGCCACGGGGGAAAGGGGCGCAGGTGAAAAATCACAAACAGTCAGTCGGGGCGCTGAATGAGATCAGTTCTGGCGAG

GTGTTAAGCGATGATGAAATTGCTGTCCTCGAAGAAATCAACCGGACGTTTACGCATGTCACCATCGGCGGGAAACACAAGGTGGTGTCGCTAAAGCCTTCTCAAACCGGCGGTGTATCGC
ACGTTTTCGAGGATTTATCACAATTTCAGCATTATTTTCATCATAAACCGAGAGTCGCCAGAAAGCTGGCGGGATCGGCGTGGCTGTCATGGAGTGGGAAGAACTACAAGCCAGGAGGCGT
AGGATTTTATCCAGTACCAGACAAATGCCCTGACGATGTTTTTAATCTGTATGAGGGGCTGGCACTGGAACCAATTGAAGGAGATTGCACGGTATACCTTAATCACCTGTTGCAGGTTGTCTG
TGCCGGTAATGAGGAGGCATACCAATATCTTATCCAGTGGATGGCGCACATTATCCAAAAGCCTGATGAAAAACCGTCCGTGGCAATCGTGATGAAATCTGTCCCGGGCACAGGGAAAGGC
ACAACGGTTAAACCGCTGCTGCAAATACTGGGGCAGTACGCCGCCCACATTAACGGGGCGGGACATATTTCAGGGCGCTTCAATTCAATACTTGCTAACAAGCTACTGGTATTTGCTGACGA
AGTGACGATCCACAAGCCGTCTGAAGCTGACAGACTTAAAGCGATTATTAGCGAACCGACGTTTAACCTTGAGCGCAAGGGAATTGATGCTGAACCAATGCCGAATTTTGCCCGGTTGATAT
TTGCCAGTAACAGCACACAGGTATTACAGGCAGGGATAAGAGAGCGTCGCTATCTCGTGCTTGAGCCATCTCCTGAAAAAGCACAGAGTCGGGAGTATTTTGATCGGCTGTACAGTTGGCT
TAATGATGGCGGTGCCGCAAAGTTGCTTTGGCATCTTAAAGGGGTGGATCTCTCAGGTTTTGACCCTCAACGAGCGCCACAGACCGATGCCTTACGTGAAGAAATCTTGCTGGGGCTGTCT
GGCGTTGAATTGTTTCTTTATGGCGAGCTAATCAATGAACCTCCGTTTAATGGCGAAGTCCGTTTGTTTGCAAAGGATATGGTTAGTCGATTTGTAGCGTGGTCGCTTGAGCGTGGGGAAAA
GCTTAAAGAGCCAGCAGCCAGATCACTACTGGGTAAATCACTTGCACAAATGGGGCTGGTGAAGCATGGAAGACCAGACCGAGGGAATGGCGTGTTCTACGAACTACCAGAGGTCGGAG
TTCTACAGGCTGCGTTTGCCCGTCTGATCGGTATGGGGGGTTATGATGTTTTTTAA
>EGKIKD_01870 DUF1905 domain-containing protein
ATGATGAGCACACCATTTTACAAAGTGCGCCAGTTAGCTTCTTCCTCTGGCTGGCAGCTACGGTTTGAGGGGCGTTCAGACTGGCTACCCATTGCGGCATGGGCGAATGTTGAAATGTGTAT
TGACGGCGATACCGTTGAAATCATTATTCCCTGCGTGGCAAGTCGGGACGGCTGTATAGAGCCAACAGATATGGCAGCAGAAATCAGAGAGGTAAAACATGAACAATAA
>EGKIKD_01875 Prophage protein
ATGAACAATAACTATTGCATACTGCAGGGAATGACCAGAACGGAGCGCGAAGAATTAAAAAGTTTCGCTACACAGTGCGGGAATGCTGGCGACATCCAAAGTCTGGAACGCACTTTAATTA
TGATTGCGCACTGGATGCGCCAGGGGCAAAGAGTTTCATTTACTGAATATGCCAGCCAGTGGACAGAGGCACAGCGCGAACGGAGCGACGGTAATCACTCAACACCCGAAATGGCGAAG
CAATGGCCTTTCAGTGGTAAAAGCTGTATCAGTCCCGGTGGTTCAGATTATTACCCTGCTGGTGTGGGAGATGAGCCATGTTGTGACGAAACTGAAATCCGCCACGCAGTGACAGTAATTAC
CGCTGAATACCCACAATTTAACCTTGACGGACTGGCGCTCCACAACCGGAATGCGGACTGGGAAAACCCGCTTGATAACCCGTCATTTATCGTATCGGCGAAAAGCTGCCTGAGATGGATCA
GAGACAACGGGATGAGTAATGCCCAGATTGAGAGCTTCCCGCAGGATAACCCCACATCTGACACGTTGAAGCATGAAGTGGAGCGATATAACCAGATAAACCACCAGCACAGTGATCACCC
GCACTATATCCCCAACGGAGCATTTATTGCGGCGATGGTGGCAAGCGGCTACAAGGTTAAGCCAGCGGGAAGAATGAACGCATTTTTCAATATTTCAAAAAAGGGATTATGTGCTGCTATGG
GTAAAAATTAA
>EGKIKD_01880 hypothetical protein
TTGAACTACGAAAATTTCGTAGTGCTCACCTTCAAAAACGTCACGCCCTTGTACACTGGATTTTACAAAAGTCTCGTAAAACTCTTTGCTGTTCTGGGCTATGTGCCAAACCGCAGCGAATAC
CTCCGTCTGCCAGATGAGCAAAACATACCTTATAAAGAAAATCGCTGTTTTTGCTGTACCACCTGTACCACCATGTCACAAACCCTTGTGTGGCGCGGCCTGCAGGTGGTACAGGTGGGTAA
AAATGACCTCTCCCCACCTGTACAGACCTGTACCATTTATTTAATTTTTACCCATAGCAGCACATAA
>EGKIKD_01885 Prophage protein
ATGGCTATTTCAGTAAAGCCAGTATTGATAAGTGAGAAGCAAATGGAAGCGATAAAGAAAATTCAGGAGGAGCAGCGTAAAAAATCAGAGGTAGGAGTTGCGCCAACGATCCACGAAATT
GCTCGGGGATTAATGGATAAGGCGCTGGCTTACACTTTAACTGGACGTGGGTAA
>EGKIKD_01890 DNA transfer protein
ATGGCTACGTGGCAACAGGGTATTAATTCTGGTGGTTTTCTGGCTGGCATCGGTACGCAAAATGAGAATGCGCCAAAGGCAAGCGACATTAACGCAACGCTTGGTCTGATCCGCGAAAACA
ATGAACTGGCTCGCTCAGGTGCAAATAACGTTGGCCTGACCGCGTTACGTGGTCTGGCTGGAGTTGCTGATATTTACAATCAGGAACAGCAACAGAAAGCTATTAGTGCGTTCAATAAGGTT
CACGCTGATGCATGGGCTTCTGGTGATCCATCGGGACTATTTAAGTTTGCCCAGGAAAATCCAGCGTTTGTTGCACAGGCACAACAGGCGTTTTCCGGTCTTAATGATCAGCAACGCAACGA
TATGGGCGATTTAGCCATGAGGGCTAACGTCGCTCTTTCTCAGGGACCGGAAGCCTACAGTAAATTCATTACTGACAACAAGGACAGGTTAAATCGCGTTGGCGCTAATGCTGACTGGATGA
TTCAGACAGGTATTCAGAATCCAGAGCAGCTATCACACATGCTGACTACTATGTCTCTCGGTGCGCTTGGACCAGAAAAGGCGTTTGCTGTTCAGGATAAGATGGTTGGTCGCCAGCAGGA
GCAGCAAAGAATTAACGAAACCATTCGCAATAATGACATGACGAATGCGAGGGCTATTAGGGGGCAGGATCTTTCCTATAAGGCTCAAATGGCAAGACTGAATCACGACAAGTATGTGTTTA
AGCAGTCACAGGCGGCCCTTGAAAGAGCAGGACAACTTCAGGATATGGATGTTTTGTCTCTTAACTCACAGATAGCAGCGACGGGAATTGATCCTCTAACCGGTAAAGCTGCAACGTCAGC
CAGAATGTCTCAGGCTAAGAGATGGCTTGATGGCAACAATAATTACAACAATGCGTTGATTACTGGTGAGCGAGGGATAGAGAAAATTGATTCTTTGCTTGGTAAGAAGGAGCTTGAAGGT
ATCGGTCGCTTCGAAGGAAGAAATATAGATGGCTTCACAAGTGCTGAAGGGCTTGCAAACCGTAATGCGATAGAAGAATTAAAGTCGGGTGCGTTTGTCCAGAACGTGCAGACTATGCGA
GGTATGGGTAGCCTCTCCAATGCTGAAGGCCAAAAACTGGAAAACCTGATCGCGAAACTCGATATAACACAGCCTGAAGAGGTCGTCAGAAAACAGTTATCTGAAATCCGATCGCAATATTC
TGTATTTCAAAAGGTTGCAGCAAGGGAGGCTGAATCAATGGGATATAGTTCATCAGGTTATGACACATATGTTAGTGAGCGAAAATCAGGAAGCGACAGCAATAAGTCCGGTTTCTCGTCTT
TATGGGGTGATTAA
>EGKIKD_01895 Phage DNA transfer protein
ATGGCTAAAGCATGGAAAGATGTTATCGCCTCTCCACAGTATCAGGCGTTAGCACCAGAACAAAAAGCGCAGGCTCAGGAGCAATACTTCAATGAAGTCGTTGCCCCGAAAGCCGGAGAA
AGTGTAGAGCAGGCTAAGCAGGCTTTTTATGCTGCCTATCCACTACCATCAACGAATGAAATAGACCGATCCCAATCAGCAACTCAAAATATTCAACATACATCATCTGATAATTCTCTTGCGTC
AGGGTATGCAAAGTTAGCTACTCAGCAAAGAGAGGGGCTTGAGCGTTCAGCAGAACAGGGAGCCAGTCTTGGGGCTGCGATGCGCGATGCTATAACAGGCGAAAGCCGAATGACTCCAG
AGATGGAGAGACTGCAAAATGTCGCCTCTGCCCCAGAATTGAACTCACTAAGCATGGATGCCCTAAAGGCTGGATGGTCTCAACTTTTCGGCTCTGACGCGTCTCAGGAAAAGATTCTTCA
GGGAATGGGGGCGACATTAAGGCAGGATGAGAAGGGGAATACTATCGTTTCTCTGCCATCAGGTGATTATGCCCTGAACAAGCCGGGTTTATCACCGCAAGACCTGACCTCGTTTCTTGCT
AATGCGTTGGCGTTTACACCAGCGGGCAGGGCTGGAACGGTGCTTGGTGCCATAGGAAAATCAGCAGCTACAGATTTAGCACTACAGGGAGCCACCAGCCTTGCTGGTGGAGAAGATATT
GATCCGTTACAAACGGTAATTTCTGCTGGCATTGGTGGTATTGGTAAGGGGCTGGAAAATACAGCGAGTGCGGTTTCGAGGGCTGTTCGTGGTGATATGTCGCCTGAAGCAAAGGCTGCTG
TCGACTTTGCATCGGAAAGAAATCTGCCGTTAATGACCAGTGACATGCTGAAAGATAAAACCTTTATGCAGAGTCAGGCTCAGACATTAGGCGAAAGAGTTCCTTTTTTTGGAACCGGTAA
GAATCGGCTGAATCAACAACAAGCACGAGAAAATTTAGTCAGAACATTTAGCGATGGTCTGGGTGGCATTTCTGATAAACAGCTTTATGAATCTGCGACTAAAGGGCAACAAAAATTCATTG
AGGCAGCAGGAAAGCGATATAACCGCATAATTGACGCTATGGGGGATACCCCTATCGATCTCTCAAACACGGTAAAAGCTATCGACAATCAGATTGCCGTGTTAAGCCGCCCGGGCAAATCT
CAGGATAGAGCCGCGGTAAAAGTCTTGCAGCAATTTAAAGACGATATCACCAGCGGACCAAATGACCTGCGTCTGGCGAGGGAAAACAGAACCGATCTTCGAAAGCGATTTATGGCGTCA
TCTGACACTGTTGATAAAGATACGCTCCAGAAAGCCAGCGATATTATCTACAAGGCATATACGGCGGATATGAAAAAAGCCGTAGCCAAAAATCTTGGAGCAGACGAAGCCATTAATATGGC
AAGGGTTGATCGCTCATGGTCTAAATTCAATGACATGATGGGAAGAACGCGCGTTCAAAAGGCAATAGCCAGCGGCAAGGCCACGCCTGAGGATGTAACAAAACTCGTTTTTAGCCAAAG
CCCATCAGAACGTTCTCAGCTTTACAGGCTTCTGGATGACAATGGTAGGCAAAACGCACGAGCAGCCATAGTTCAGAATGCTGTAGATAAGGCGACTGATCCGTCTGGAAATATCAGTGTTG
AGAAGTTTATTAATGCGTTACACCGGAACAGGAAGCAATCAGCAACTTTCTTTAAAGGCGTACATGGAAAGGAACTGGACGGCGTTATTAAATACCTCAACGATACAAGACACGCGGCAAA
AGCGAACGTTCAAAACTTAAATGGTCAGCAGCTTTATGGATTGTTAGTTGGTGGTGGCATCATAAACGCAGCAGTATTAGCGGGGATGCTAAAAACGGCTGCGTTTGTTGTTCCTGCTGCTG
GTGCCGTAGGCGGAGCAGCGAAGGCATACGAAAGCCCTGTTATACGAAATGCCTTGTTACGTCTGGCAAATACGCCAAAAGGTAGCACAGCATATGACAGAGCGATCAGTACGGTCACACA
ATCGCTCACCAGAGTCGCACAGGCATCACAAAAAGAATCTCAATAA
>EGKIKD_01900 Prophage protein
ATGAAAGGCAGAACGCCACACATTGAACATCAAGCCAACCACTTCTCGGATGTTCGCAATGAGATGATTGCCATTCGCGGTGATATGCACGGCTTGGAGCGTCGAATTATTGATAAGATTGA
TGAAAATAGAGATAAGGCCAGTGAAAATCAAAAGTGGATCATCGGCCTGTTGATTTTATCAATTCTTATCCCGCTACTTATCGCATTGGTCACAAAATGA
>EGKIKD_01905 Prophage protein
ATGGTCTTTGCAATGAGCAAATCTAATCTGATCGCGTTCCGCATCCCATCTGAATTGCAGGATGAGTTTAATCGGTCTGTTCTCGCTTCTGGAGGGGGTAAAACCTCCTGGCTTGTTGATGCG
ATTCGCATGAAGCTGGGACAGCCGGAAAAGTCTATTGATTCACGAATGCTTGGGCTGGTGGAGCGCATGGAAAAAGCCGCTGCGTCGCTTATTGCCGGAAAGCCGAACATCCCACCAAAG
CCATATAACGAAACGGCAGTTATAAAGATCATTGCCGATACCATCCAACAGGGATTTGATAATGGGCGTGTGATAGCTGAACGGATCAATGAAGCAGGCTATCAAACAAAAGCTGGTAAAGC
GTGGGATAAAGATATTTACAGCGCGTGGAAGCGTCAGGGAAGCAATGCTGAAAAGTTAAAAGCGGTGATTGATTGTAAGGTCAGCGTATAA
>EGKIKD_01910 PerC family transcriptional regulator

ATGATAAGGGACAGGAAAGCTGAAGAGCTGGAGTCAAAAGGGCTATACCGGAGAGCTGCCGCACGATGGGCAGAAGTCATGATGCTGGCAGATGGCGATAAAGAACGTGAACATGCAG
CAAACAGACGCTCTGAATGTATCCGTAAGGCGGCACGCCCACCAGCAACTACAGACAGATTCGGAGATTTAAGACAGGCAGTTAAGCGGACTCATTCGGCTCTTGGTATGGATGAGGAAG
CGAGAGGGTACTTCAGGCGTTATCGCGATAAAGATTGCCAGCTACGATAA
>EGKIKD_01915 Band-7-1 domain-containing protein
ATGACTACAACGGTATATGATCGTGTTAACGCCTTAGTAGCGACCGACTCTAGGTGGTCTGTTGATCTTAGCCCACATGGCTACGATGGTCATATCCTGTATATTGACGACACCGGCTTCGGTA
AGCTAGCTCCACGAAATGATTTTGTTATGCTTTTAGCCGGTGATGGTCTTTTGATTCAGTTATGGAAGCATTGGTGGCGAGGAGACCTATCTCAACAAGAACCACCAGTTGTTTTACCTACTG
GTCAATCGGTAAATTTACATATTGTGAAAAAATCAACTAATGAAGTGATTTTTGATAAAGGTCAAAAATTAGTTGTAAAAAATAACGAAACGGAAGAACTGTTCGCTGTCTTTACAGGCTCAG
GATGCGGTGCTGCTGCGCAAAACTGGATGTATAGCCATTGCGCAAGATCAGCAATTGAAGAATCTAAAAAGCTTGACCCATACACTGGCGGCACTGTACGTTTTTTAGACTTCAGGACTAAC
GCATCATTAGTAGAAGACTCTGTTTCAACCATTAGCGAAGTAAACGAAGCTCTATTGCAGAGGGGGCTGATTATGGATACCAAAAACCCACATTCACCACACGTTTCTATCAGTGCACAAGAA
GTTGCTGAAGTTCGTCAAATGCTGGTTAGTGGTTCAATTACCCCATGTGCACCAGTAGGCCAAAGAACCCAAGATTGGGATGACAACTCCAAATTGCGTTTAGCAAATGCTATTCAGCGTATT
CGTGAAGAAGAAGCACAAATGCGATAA

Prophage 4.1

>EGKIKD_10555 Tyr recombinase domain-containing protein
ATGCACGCATGGCTGGAAAAATATGACAGCATCATATCAGTCAGAGGACTCAGGCCTAAAACACTTGCTGATTACAGAAGCAAAATAAGAGCCATAAAGGAGCGGTTTCAGGACATTCCATT
GTCAGATATAACAACCAGGGATATTGCCACCATACTCAATGATTATGTTTCAGAAGGAAAGTCGGCCACATCAAAGTTAATCAGATCGACGTTGAGTGACATTTTCAGAGAAGCTATAGCTGA
AGGCTTCATACACTCAAACCCAGTCACGGCCACAAGGGCCGCTAAATCTGAAGTTAAAAGGGTCAGATTAACCACCGATGAATTCATGAAAATATATGATGCTGCCGGGAAGCAGCCGCCAT
GGGTGAAACTGTCTATGGAGATAGCATTACTTACCGGGCAGCGTGTTAGTGATATCTGTGCAATGAAATGGGTTGACATTTCAGAAGGATTTCTACACGTACAACAACAAAAGACAGGAGTA
AAACTGGCGATACCGGTAACGATTAAACTTGATGCAGCTAACCTTTCGCTTTCAGATACGCTGAAGAGATGTAAATCACTTTCGCAAGGAGAAACAATAATTTCCTCTACACGAAGCGAAGC
GCTTTCATCAGGGACGGTATCAAGGTATTTTATGCGCGCACGCAAGGAATCTGGACTTTCTTTCAGCGGAGAACCGCCAACATTTCATGAGATACGCAGCCTGTCTGCAAGGCTCTATGAGA
AACAGTATGGCGAAAGATTTGCGCAGCACCTTCTCGGACATAAGTCTGATAGCATGGCTGCGCAATACAGGAACGATCGCGGGAGAGAGTGGGAGAGAATAGAAATCAGTTAG
>EGKIKD_10560 hypothetical protein
ATGGGAAGAAGGCGAAGCCATGAACGCCGTGATTTGCCTCCAAACTTGTATATCAGGAACAACGGTTATTATTGCTATCGAGACCCAAGGACGGGTAAGGAGTTTGGGTTAGGTCGCGACC
GGAGAATCGCAGTAACAGAAGCAGTTCAGGCGAACATTGAGTTATTTTCCAGCGCTGAGCCGAAAAAGTCTTACGTCGCGGATCAATAA
>EGKIKD_10565 hypothetical protein
ATGGAATCACACAGCCTCACACTCGATGAGGCCTGTGCATTTCTCAAGATATCCAATAACCAGCCTTGCGCTGGTTTTTTCTTGCGTGAATTTGCAGAGGTAATGCGATGTACTTAA
>EGKIKD_10570 Eac protein
ATGTCAGACCAGAGCAAGTATTACGATTACTACATGGTTGAAGGTGATGATGTTAAGGAACTTATCAGTTCATACGATACCATTAACGAACAACGTAATTCTATCCTCCCAGCCGCAGCAGAAC
AGGTTGGTGCTATAGCATGGACAACAACTCGTAATTGGGGTGGTGGAGGTGGCTTGCTGCAAAGTTTCGTTTGGGAAAAAGGATATGAATTCCCATGCCAGATAACAATCAAACGCGAGG
ATTTTTGGGACGGGAAGAGAGTTGTGATAGCGCGAGGAAAGGGAAACACAAAGGAAGGCCGCGCATACAACAAAGAGCTGGATTCAATCATGCATAACGCTAACGCCAAGCTAAAATCC
TTGCCTGAATGGAATTACTACATAACCAACCACTACGGGATTATGCGTACAGGAATTGGTGGTCAATCGGGCCGTGGACTTGGTTTCGTTATGTTATCAACGTATGGCTGTAAGCACCCGAAG
CGCAATGATTGTCTTATTTTTGCAATACCAAATAACAAAGAAGAGAGGCATGGCGAAGTTGTTATCCCTGACAGCTTCAAGAAAATAACTTACGGGAAATTCTACGACATCGCGAATGAAGTT
GAAGAAGAAGCTGTGGAGTAA
>EGKIKD_10575 ASCH domain-containing protein
GTGGCTAACCTGCAACTTGCCGTTAAAGGTGTATACTTCGATGCCATGATTCGCGGAGAGAAAACGGAAGAGTATCGCCTGTGTAATGACTACTGGAATAAGCGAATTATGTTCCGCGAGTA
TGACCGACTGATTATCACAAAGGGATATCCAAAGCGCGACGATTCCAGTCGCAGAATTGATGTTCCGTACGACGGATATGAAATCAAGACAATCACACATCCCCACTTCGGCGATAAACTGGT
GAAGGTATTCGCGATAAAGGTGAATATCGGCAATGAATAA
>EGKIKD_10580 hypothetical protein
ATGAGCACCTTCACTAAAGAGCAGTTAATAGAATACCTGAAGGAATCGACGCAGAACGCTAGTGGTATGTTTGAAATCAGCGAGGACACCATCTGCGCGTTGATGTCCATGCTCGCCTCCCC
GCCAGCGCCGGTATCTGTGCCCGCTGCGATGGAAATTGATGATGACTTTGACAGCGCGTTTGAACACGGAAAAGCTGTCGGCTGGAACGCCTATCGCGCCGCCATGCTTCAGGCCGAACCT
GTAAGTAATAGTGATGAGTTACCGCTGGACTATCTGCAAGGACACAAAGACGGCCTGGAGTGGGCTGCACAATTGGCAGAAGCCAATCATCCGCAAACAGGTGACTGGTTGTACGACGAC
CCAATCGATCTTGCCAGGGCGATTCGCAAAGGTCCGGATATGCCTACTGTTCAGGGTGGCAACTCTCCGGTAACTCCGGATGGTTGGATAAGCTGTAGTGAGCGAATGCCGGATAAGTTAAT
TCCGGTAATGGTCATGTATGAAGACGGTGAGATGTGGTCTGCAATGTGGAATGGCAATCGCTGGGATGATGGCACCGAATATCCGGATCCGCACTCAGTTACGCACTGGCGTGAAATGCCAG
CAGCACCGAAGCAGGAGGTTAACCGTGGCTAA
>EGKIKD_10585 SlyX protein
ATGAAATACGAAATCCCGGAATCAGAAGATATTGAATGGCAGCAGGCTATGCTCCGTGAAATAGACAGCGCCCTTGACGTCTTGCGTGATGAGCATGAGCACGCAGTGGTGGTAGAAGAAA
TCATCAATGATATCACCGCGAGAATAGCATCACTCCGCGCGTACTCTGGATACTGA
>EGKIKD_10590 dATP-dGTP-PPHyd domain-containing protein
GTGAGCCAGATTGATTATCAGGCACTGCGTGCTAAGGCAGAAAAAGCAACGTGTGGCGAGTGGTCGCTCGAATATGGAGAGGGCCGATTTGATGGTGATGATGCGCTAATTCATCGTGAA
GTTGCTGGATATATTCCCATTTGCAGAATTGAAGGAGCGCATCCAGAAAGCGGTTTCGATGAAGATTTCCAAATGGAACAGCAGGCCAATGCTGAATTCATCGCCGCAGCCAATCCGGCTAC
CGTACTGGCGCTGCTGGATGAGCTGGAATCCAAAGACAGGCGCAACGCTGAGCTGGAGGCGAAACTCCAAGCCACTGACAAATTGCATGATAGCGCGTTCCGTCATGGTCTCCAGCATGG
TTTTAGTTACGGTCAGACAGATGATCAAGCAGGATTTGAGAAAGCCATCCAAGCGTATGGGCAGCAGTGA
>EGKIKD_10595 WWE domain-containing protein
GTGAGTAATTCAGCACGACTACAGCTTGGTTTTTCACCGCTATCAAAAACTATCATGCTGGCAAAAATGCGCGATGTTGAAGGTGGACGTATGCGCGTTGGCAATGATCCAGGTCGTGATGT
TACCAATGAGGCTGCTCAATTGGTGTGGCGACTGGTCATGGCTGAAGGTGGTGAGATCGCGTGGGAGCTGGATGATGGTTCTCGCATGGTGTTGAAGGCAGAAAAGCAGGAGGCAACCA
GTGAGCCAGATTGA
>EGKIKD_10600 Anti-RecBCD protein 2
ATGCCAGCGCCTATGTATGGTGCGAATGACCCGCGCCGCTGTTCCGGCAATTCCGTCTCGGAGGTGCTGGATAAATTCAGAAAAAACTACGACCTGATAATGTCACTACCGCAGGAAACGAA
AGAGGAAAAGGAATTTCGCCACTGTATATGGCTTGCAGAGAAAGAAGAACGCGAGCGAATTTACCAGACATTAATCCGACCATTCCGCAAAGCCACATATACCCACTTCCCTGAATATATCG
ACCCGCGCCTGCGTAATTATCGATCACGCTATGGCGCTATCAGTAATGACTGA
>EGKIKD_10605 3'-5' exoribonuclease
ATGAATCATTTAATGGTTGACCTTGAAACAATGGGCAACGGGCCATACGCGCCCGTTATTTCGATTGGGGCAGTATTCTTTGATCTGAAAACTGGAGAAACAGGAGAAGACTTCTCGGTTAA
TATCTCGCTCGAGTCATCAATGCGATACCGGGCGCGTCCTGATGCTTCAACAATTTTATGGTGGATGGAACAGGGAGAAGATGCCAGAAAATCGCTAACCAATGACACTCAAGAGCTTTCAA
CGGCTCTTTCATGGTTATCAGACTTTATCGCAAAGCACGCCAACCCTAAATTCGTTCAGGTTTGGGGAAATGGCGCATCATTTGACTGTGTGATTCTACGAAATAGTTATGCTCTGGCCGGGC
ACCAAGCGCCCTGGCAGTGGTGGAATGACCGCGACGTCAGAACCATCGTCGAGCTTGGAAAGGCAATTGGGTTCGACCCTAAACGAGATATGCCATTCGAAGGAACTCGACACAACGCGC
TTGATGATGCCATTCACCAAGCCAAATACGTTTCAGCAATCTGGAAAAAGTTAGCTAAATAA

>EGKIKD_10610 Single-stranded DNA-binding protein
ATGGCTAGTAGAGGCGTAAATAAGGTGATCATCTTAGGCCGGGTCGGACAAGACCCGGAGGTTCGTTATTCACCATCAGGGACGGCGTTCGCTAACCTTACAGTCGCTACATCAGAGCAGT
GGCGAGATAAACAGACTGGCGAACAAAAGGAGCAGACTGAATGGCATCGTGTTGCCGTAGTCGGGAAACTTGCTGAAGTCGTGGGGCAGTATGTGAAAAAAGGTGATCAGGTTTATTTC
GAGGGAATGCTGAGAACCAGAAAGTGGCAAGACCAGACAGGGCAAGACCGCTACACCACTGAGATTAATGTTGGAATTAATGGTGTGATGCAAATGCTTGGAGGCACTGGCGACAGCAA
ACAACAAGCAGCAGACAGGCAGTCACAGATACCACAGCAGCAACCATCACCAATACAACATAACGAGCCACCGATGGATTTTGATGATGATATACCCTTTGCACCAGTAACTCTCCCCTTCCC
TCGTCACGCTATTCACGCAATTTAA
>EGKIKD_10615 Recombinase
ATGGATTTGAATAAATTCGACGACCCATTCAGTCCTGAAGATATCGAATGGCGAATACAGCAAAGCGGTAAAACACGCGATGGCAAAGTGTGGGCTATGGTGCTGGCTTATGTCACGAACA
GGGCAATCATGAAACGCCTTGACGATGTTTGCGGCAAAGCAGGATGGCGCAATGAATACCGCGATATTCCCAACAACGGCGGCGTTGAATGCGGAATATCAATCAAGATTGATTCCGAATG
GGTCACCAAATGGGATGCTGCTGAAAACACGCAGGTAGAAGCCGTCAAAGGTGGTCGTTCCGGTGCAATGAAGCGCGCTGCCGTTCAGTGGGGAATCGGTCGGTATCTGTATAACCTTGA
GGAAGGTTTCGCACAAACATCACTCGATAAAAAGCAGGGATGGCACAGGGCAAAACTGAAGGATGGAACAGGATTTTACTGGCTCCCTCCATCGCTGCCGGACTGGGCCATGCCAGCATC
AGGCAATCAACCATCACCAGAAAATACCAACCAGAAATCACCATCGTTTGACTGCGAACAAATCCTGAAAGACTTCAGAGATTATGCAGCAACAGAAACTGACAAGAAAAAGCTAATTGAG
AGATATCAGCATGACTGGCAATTATTGGCTGGTCACGATGATGCGCAGACAAAATGCGTTCAGGTAATGAACATCAGAGTTAACGAACTAAAACAGGCGGCATAA
>EGKIKD_10620 Host cell division inhibitory peptide Kil
ATGGATAAATCACTTATGGCTATTCAGTCTAAATTCGCAATTGCTGTTTATCTTGGTGACAAAATAATGTATCGCGAAGCTGTAGAAGCCTTTCGCGAATGGAGGTTGAAATGA
>EGKIKD_10625 hypothetical protein
ATGACATTTGCTATCGCGGGCGGTGCCGTCATGGGTATCGCACACCTTAATGAATCACTTTTAGAGCGTATCACCAGAAAATTACGGGCCGGATGGAAACGTCTGGTCGATATCCTGAATCAA
CCAGGAGTGCCGTGTAATGGATAA
>EGKIKD_10630 Superinfection exclusion protein
ATGAAATTACGTGTCTGGCATATCCCGCAAGTTCCTATGAAGCCATTCATTGTAGAAGTAGCAAGTGTTGAAGAGGGTGTGCGAGTGATGGATACACTGGCTGATTATGACGCATTTCAGTAT
GACAACAACATCAAACCTGATTACTGCAACGCTAACGGCCTTGAGATGTGGGATGAGAGCCTTACTGACCAGGATTTGGAAGAAATGGAGCTTACGGATCGCTGGGTGGATTGGTATAGCG
AATGCCAGTGTTACGACGACCCGCGTGAATATATCGAAAGCCTGAAAGAAGAAACAACAGCCGCCGCCTGA
>EGKIKD_10635 hypothetical protein
ATGCCCTGGCTTTTCCTTCTATTCCGAGTGATGCCGCTACGGATTGCGATCCAGACACTTCCGCTGCTGACCAGTCGCCAGTGTTGGTTGCTGCTGACCGGTTGCCAGTGTTGGTTGCTGCTG
ACCGGTTGCCAGTGTTGGTTGCTGCTGACCAGTCGCCAGTGTTGGTTGCTGCTGACCGGTTGCCAGTGTTGGTTGCTGCTGACCAGTCGCCAGTGTTGGTTGCTGCTGACCAGTCGCCAGT
GTTGGTTGCTGCTGACCAGTCGCCAGTGTTGGTTGCTGCTGACCAGTCGCCAGTGTTGGTTGCTGCTGACCGGTTGCCAGTGTTGGTTGCTGCTGACCAGTCGCCAGTGTTGGTTGCTGCT
GACCAGTCGCCAGTGTTGGTTGCTGCTGACCAGTCGCCAGTGTTGGTTGCTGCTGACCGGTTGCCAGTGTTGGTTGCTGCTGACCGGTTGCCAGTGTTGGTTGCTGCTGACCAGTCGCCA
GTGTTGGTTGCTGCTGACCAGTCGCCAGTCATGA
>EGKIKD_10640 hypothetical protein
ATGACCAAAGAAATTGTGACATTCAAGGGATTTAACAAAGACCTCACGTGCCGTGACTTTCAGTTTGCAATCGGTGAAACCTTCCATCACGATGGAAAAGTAGAGGCTTGCGGTTCTGGAT
TTCACGCCTGTGAATGTCCTTTCGATGTTTTCAGTTATTATCCGCCGGCAGAAAGCCGCTATGCGGAAACAATATCTTTTGGTGTTATAGACCGTGAAGAAGAAGGTGACACTAAAATAGCCA
GTGCCAGTATCACAATTAAGGCTGAGCCTAACGCTTCCACAGTTCATTCAGCGTGGTATTGA
>EGKIKD_10645 Antitermination protein
ATGACACGCAGAACAGTTTTCAATGGTTCAGCAGCAGGTCGTCGCCGCGAACGTCGCGCTGCGCTTCAGAATGAGACTACAGCCAGTTCAGAAGTATTACACCGCCCCACCCTTAGCCGTG
TACAGATTCAGGCCAAAGGAAAACATGAAACGCCAAAACGTATTGAAGACGCAAAATCACTTCAGTTCATGGCGAAAGATGCATTCTGGCAAGTGGAAGAGTACAGACGCCATCTGGAGC
AGGCAGCCATTGTATACGCAAATGAGTTCGGACATAAGCTACCAGAAACAGGTGTATGTTTGCCAGATGTTGCGCTTTACGCAGCAGGTCATCGTAAGTGTGGACAAGTTACCGCCAGATAA
>EGKIKD_10650 Fumarase D
ATGAAAAGCGAAGAGCTGGCGCAGTTGCGCTATCAGGAAATGTGCAGGATTGTGGGTGATGTCGTTTTTGCTATGGTTGCTGAGGGTCATGAGACCAAAAGAGTCGCTATAGCTGACGTG
ATAAGAACGGAGCTATCAAAGGGGCTGGATAAGTGGGATGTTGACCAGATTCAGGTTATGGAACTAGCGGTGAAGCTACTGGAAGAGTAG
>EGKIKD_10655 hypothetical protein
ATGAGTGGTCACAATATTGTCATACCTGTTACCATTAAAACCGCAATGCCCAAATACATATTTACCTCAAGCATTAAAGAAGGTGGATCATGGAACAGCGCATATTCATTGCTTGGAAAGCTGA
TGGATCTTTATCAGGCCAACAACGCTGTTAACAATCGATACGTAGTTATTGACAATGAATCTATCGGCTCGCAAATGCAGCAACTGATACTGCTGTTCAACGACGTAAGCCAAGTTCTTCCATT
CGAAAGCAGAAGTTTATGGCTTCCAAAATTAGCTGCATAA
>EGKIKD_10660 hypothetical protein
GTGGCAAAGAAAAACGAGAGTCCTCTGTTACAGTCGGATGTTGATCGCGTCATAAACTCCAAGAAAATAGTGGATGTTAGCAGCATAAAATGGGGTAAGAAGCCGCCACCTGGCAGGTCA
CCGATGTGGTTACAAACGGCTATCACACCTTATGAGGATGGCTCACCTTTGCCTGGCCTAAAGTTTGTTTTACAGTGGCGACCAGCTGATGAATACGGAGATTCCCCTAAAATTCAGATGGTG
GCGCTTTACTTTGGTCGAAGAATTTTTGGTGTGGACTCTTACCCAAATGATAGACATACTAACAGAGTCCGGGTATGCCATCCTGACTATGCTGAAAGCATTCTTGGCCCACATTATCACCTTT
ACTTTGAATCAGCGTTGCCGTATGAAATAGGTCTGATCATACGCGAGAAGATAGCCCCGGATGACTTGTTAGGACACTGGCGCTTTTTCTGCTATAAACTGAATGTTACATGCAAAGGAATAC
TGCCTTTGCCGACCCAAGAAGATTCGGGACAAATTCCATTGCTATGA
>EGKIKD_10665 Repressor
ATGAACTGGTATGACATAGCGAAGCAAAGGATTGATCAGCTTGGATTGAGTCAGGATAAAGTTGCTGAACACCTTGGTGTAACCAAAGGTGCTGTTAGTCATTGGCTTAACGGGAGAAGGA
ACCCATCAATACAAGAAATTGGAGCTATATTTCAATATCTTGGCGTTACAGACGCGAGGTTCAACTCTGACGGAACCTTTAGCGTTGGCGAGTCAACAGAACAAAAGCCTGTTAAACCTCAA
TTTGAATACCCATTCTTCTCTCATGTTCAGGCTGGAATGTTTACACCAGAATTTCGAACCTTCACTCAACTAGATGCTGAGGGATGGGTAAGTACAACCAAAAAGGCCAGTGAGGCAGCTTTC
TGGCTTGAAGTTGAAGGCCACTCAATGACGGCTCCAGCGGGATCACGACCAAGCTTTCCTGAAGGAATGCTGATTCTTGTAGACCCAGAAGATCCTGTAGACCCAGGCGATTTTTGCATTG
CAAGGTTATGTGGTGATGAGTTCACTTTTAAGAAGCTCATCAAAGACAGCGGACAAGTATTCCTACAACCGCTAAACCCTCAGTTCCCAATAATGCCGTGTAACGAACAATGCAGGGTTGTA
GGTAAGGTTGTAGCCAGCCAGTGGCCTGATGAGATATTTGGGTGA
>EGKIKD_10670 HTH cro/C1-type domain-containing protein
ATGAATAATATTCGCAATTTTCGCGAGCGCTTCGGTTTAACGCAGGAAGATCTTGCGAAAGTACTCGGTTGTACGCGTGGTGCAGTTTGTCATTACGAGACAGGCAGAAGGGGAATGGATAT
CAATCTTTGTCGCGCTTTTATCAATGCGTTCAAAGAATACGGTTATGAACTAACCATAGACGATCTTTTTCCACCAAAGGCCGCGTAA
>EGKIKD_10675 Bacteriophage CII protein
ATGGAACTCACAAATCACAGCAAAAAGATACGCGAAGTGGAAACAGAGCTTCGCGCCCGACTCGTATCAATGGGGCAGACAAATTTCGCAAAGATGGCGGGATGGTCTGATTCAAAAGTA
AGCCGCCTGAACATTCAGGATATGGCGGTGACGTTCGTTCTTCTGGAGAAGGTATGGGAGACGAGCTTAATCAGGGAAGTGGCAAGACAGGCAGTGGAAGCTGTTATGCCGGGAAATAA
AAAACGCCCGGCGTGCAAGACCGGGCGTTCTGAGCAAATCGATATGCAATTCTGA
>EGKIKD_10680 hypothetical protein
ATGCCAAAACGTCGTAAGAAATACCAGGAAAAAGAAGAGATTCGACACCCTGATTCACCTGAGGGATTAGTGGTAGCCGCAGCAAATAACAGGGCGTTCGCAGAGCGCCTTGTTGGTGTT
TACAGACTAGCCAAAGCAGGAGTGAAACATGGGCGTCGTTAA
>EGKIKD_10685 18 protein
ATGGGCGTCGTTAAGTTAGCAGACTACAGGCCGTCAGAGCCGGTCGTGGAGCGTAATGTGGCAGATCTCGATGATGGTTACGCCAGACTATCAAATATGCTGCTTGAGGCTTATTCAGGCGC
AGATCTGACCAAGCGACATTTTAAAGTGCTGCTTGCCATTCTGCGTAAAACCTATGGGTGGAATAAACCAATGGACAGAATCACAGATTCTCAACTTAGCGAGATTACAAAGTTACCCGTCAA
ACGGTGCAATGAAGCCAAGTTAGAACTCGTCAGAATGAATATTATCAAGCAGCAAGGCGGCATGTTTGGACCAAATAAAAACATATCAGAATGGCGCATCCCTCAAAATGAGGGAAAATCC

CCTAAAACGAGGGATAAAACATCCCTCAAATTGAGGGAGTGCTATCCCTCAAAACAGGGGGACACAAAAGACACTATTCAAAAGAAAGAAATACAAGATAAAAACATTATGTCCGAAAGTG
TTCGGACGAAGTGTGAAAAATCATCTGGCCATCACGAAGAAACCGACAAGGCATTCGAGGAAATTTTCTGGTGTGCTGGAATGCGGAAAGCCGGGAAGAAAAACGCAGCTTCGGCATTC
AGAACACAGTTCAGGGAGTGGCGTAAAACCACCAGGGGGACGGCAAGCGAGTTTGCCACAATGCTGGCAGAAGATATCGCGTGCAGGAACGGTAAGCAGTTCGGATTCGACAGGTTGT
TACCATCGAGCTACCTGAACGGTCAACGCTGGAACGACGAGAAGCCAGAAACCATTCAACCACAATCCAAACCATCATCCGCAATCACCGTATCGAAAACTGGCTACGTGTTTTTCGACAGG
TGA
>EGKIKD_10690 HTH-48 domain-containing protein
ATGAAATCAAAAATCAAATCGCTACTGGTCTCTGGTTATAACCACGGCTGGTTAGGTATTTCGTTTGTAGATTTCTGGTTTAAAAATCTCAATCTGAGGGAATCATGA
>EGKIKD_10695 Replication protein P
ATGACGCCAAGTGAACTTAGCGACCTGCTATGGTCGCAGGTTGACAGGGTGGCTCCGCACCTGTTGCCAAACGGCAAGAAAGACGGGCATGAATGGGTTGCTGGTAACGTCAACGGCGA
CAAGGGGAACAGTCTGAAGGTTAACCTTAGCGGAAAGAAAAAATGGGCTGATTTCGCTGAGGGAGACGGCGGTGACATGCTTGATTTGTGGATGGCGTGTCGTGGAATTAACCTGCATCA
GGCCATGCAGGAAGCGAAGGCATTTCTCGGCATCAGGGAGGACGATCACCATTTCGACGCCAGACGTGAGAAGAGATTCTCCAGACCTGACCGCAAGAAAATAGCCCGCTACGTTACCAG
AACAGAATCACATCTTGAGTACCTGCAATCGCGTGGCATATCGCCTGAAGTCGCGAAGCGATACGAGGTTGTCAGCGGAAAGGTCTGGAATGGCGAACGTGAACTGAGTGCCCTGGTGTT
TCCGTACAAACGCGATGGCGAGCTGCTGCAGGTCAAGCGAATCAGTACTGAACGTCCGGACGGGAAGAAAGTCATCATGGCAGAAGGTGACTGTGAACCTTGCCTGTTCGGGTGGCAGG
CTCTCGATGCTGGCGTGAGGGCGGTTGTACTTTGCGAAGGCGAAATTGATTGCATGAGCTATGCGCAATACGGAATACCGGCGCTATCTGTCCCTTTCGGTGGCGGGAAAGGCGCCAAGCA
ACAGTGGATTGAGTTCGAATACCACAACCTCGACAGGTTTGAAGAAATATTCATTTCGATGGACGTTGACGATGTCGGGCGTGAAGCAGCAAGGGAAATCGCAAGCCGACTTGGTGAGCA
TCGCTGCCGTCTGGTTACACTGCCACACAAAGATATCAACGAATGCCTGATGAACGGCGTCACCGAGGATGAAATCTGGCAGTACATCGGAACAGCGTCATATTTCGACCCGGAAGAGCTTT
ACAGCGCCCGTGAGTTTTATCAGGACACCATCAATGCTTTTTACGGCAAGCAGCAGTATCTGTTTAACCCACCGTGGGAAACGCTGGCTTACAACTTCCAGTTCCGTGAGGCGGAGTTAAC
GCTAGTCAATGGCGTGAACGGTCACGGGAAAACGGAGGTTGTCGGGCATATGGCACTTGAGGCCATGAGGCAGGGGGTAAAAACATGCGTCGCATCACTTGAACTGAAGCCCGGGGTTT
TGCTTAAACGCCTGACCAGGCAGTCAACATGCTGCAAAATGCCACCAGTACTGGAAATCGAATCAGCATTTAAGTTTTACGATGACCGGCTCTGGTTATTTGGCCTGACAGGTACAGCCAAG
GCTGAACGCCTGATTGAAATTTTCACATACGCCAGACGGCGATACGGCATCCAGTTATTCATCATCGACAGCCTCATGAAGTGCGGTATTGGCGATGACGATTACAACGGGCAAAAGGCGTT
TGTTGATTCGCTGTGCGACTTCAAGAATAAAACCAACTCTCACATTATCCTCGTCACTCACTCCAGAAAGGGAGACAGCGAGGAGAAACCTACCGGAAAGATGGACGTAAAAGGCTCAGG
AGCGATTACAGACCTGACGGATAACCTGTTTATCATCTGGCGCAATAAAGCTCGCGAGAGAGCGTTACAGCGCGTTCATGCTGGAGAGCAGATTAACGATAAAGACCAGCAGCTTCTTGCA
GCACCCGCATCTGTTTTGATGCTTGAGAAGCAGCGAAACGGGGAAGGGTGGGAAGGCGGCGTGCCGTTATTTCTTGACGAGCAGTCTCACCAGTTCCTGCAAATGGAAGGTGCATCACCA
TACAACTACATCGCCAACATGCCGAAGTCGGAGTATGACGAAGTGTGGCGGCAGGAAAACGTTACGGAGTACTGA
>EGKIKD_10700 Transposase
ATGCTTAACCGAATAATGCCAGAAATGCTTTTGAATCCACGGTTCATTGCTGTTTTGAACCGATGTATCGACGAAGAAGAATTAATTATTCAATTCGAAAGGCTGTCAGGAGTAAGCCGACCA
CCAAAGAGGCAGCATCCAGTAGAGCTGATGGTTGACAAAGCGACCGGATTTTATGATGAGCAGTGGAAACTGTTTTTTGAAGCATTTATCCCGTTCGTCTATGAGTTTATATGGCTCACATGG
GAAGACCGTGACAATGAGGAGTGCTGGCAATGA
>EGKIKD_10705 Prophage protein
GTGGCTGACTGGCAAATTCCAATCATCATTCTTGCCGGAGCTTCGCTGGTTGCTGGCTTTATCCTGCTGAAAAAGCATAAAGACCGTGATCAAAAAGTCGAAGTTCTCTATGGGTATCCAGCG
AACAGCACAACATGGCTGACCATTTACCACTACCGAAAATCAGGCCGCTGGGTATTCGAATGGGATGATCTGTTCGCTGAAAAGCGACCAAAGTCATGGGGAGACATCAGCGAATGCATGA
TGTTTGAAGAAAGAAAATCCGGCGCAACCCGAGAAGAGTTTAACGAAGCGTGGGCGCGATTAAGTGAGAGAGGGTATTTGTGA
>EGKIKD_10710 hypothetical protein
GTGAGCAAGTACGAAAAATTAGATCAAAACATTCTTTCAATGCTGAGTGAAAGACCAACACCTGTTTTTGATATCTGGCTTAAATGGCGGAGCAATGGAATGTATATCGAAACCATCGATCGC
CGTATGCAATACCTGAGAAAGAAAGGGCTTGTTGCAAATGTGCGTGGGAAGGGTTGGGTGAAAATTAACCTGTCATAA
>EGKIKD_10715 hypothetical protein
ATGGACGAATCAAGAAAGCAGTTTTTGGAGTGGTTTGGTGAAGAGTTCGAGTCTATTAACAACAGCGGAGAACTTCACGTTCAGGCCATCAAGATGATTGCTTGGCAGTCATGGGTTAAGT
CTCGCGCAGCTATCGAAATTAAATGGCCATCTCATGGAATAGACGCTTCTGGCGGGACTTACCACCGCAGAGATGACATGAGAAAAGAGCCAAAGCGCCGCTGGAATCAAATTGAAGGAG
TGAGTATGAGCGCATACGAAGAAATCATGTTAGCCCTGCGATTCTTTTTCGATGTGGAAGAAGATGAAAACGTAAAAAAGATTATCGGCTACGACCGTGACCCGATAGGCACTATTGCTGCC
GCACTTGACGATTACAGGAGCGTGGGAGATGAAGCAAACATACCTGCTTCGCAACGAAGCAATCAGAAATAA
>EGKIKD_10720 Recombination protein NinB
ATGAAGCAAACATACCTGCTTCGCAACGAAGCAATCAGAAATAACGCCATAGACGCCATTCTCTCACTTCCCATCGACGACAAGTCACCTCACGAAGTCCACGTCAAAGAACCCAAGCGCAC
GAAAGCGCAGAACGACCGTATGTGGCCGATGCTACAGGACGTCTCCCGTCAGGTGCTATGGCATGGTCAACGGCTGTCTCCGGAAGACTGGAAAGACCTCTTCACTGCGCTGTGGCTTAA
GACCAAAAAACTGGAGCAAAGGAGCGTACCCGGTATTGACGGCGGTGTTGTTCTTCTTGGGGTACGTACCAGCAAAATGAGGAAGGCCAGCATGACAGAGCTTATCGAAATCATGTTCTG
GTTCGGATCAGAACGTAACGTGAGATGGAGTGATGATTCCCGGCGAGAGTACGAGTGGTCAAAACGAACAGGGAGAGTTGCATGA
>EGKIKD_10725 hypothetical protein
ATGATTGACCCCAATCGAAGTTATGAGCAAGAGAGCATAGCAAGGGCAATGTGCGCAGGATGTAACAAGCAACTGGCACCTGATGAAATTTACGCCTGTTCCGAATGTGTTAACGAATGGC
TGGTATATCGCGATCCGAATGGAGATATGTCGAATGAGGAAGGTAAGGAGGCGTTGTAA
>EGKIKD_10730 Protein ninG
ATGAGGAAGGTAAGGAGGCGTTGTAAGAACGAAGAGTGCAGGGAATGGTTCTTCCCCGCAATTTCAGAACCAACAGTGGTGTTGTGTTGATTGTGGTACGAAGTTAGCACTCGAACGACG
AAGCAAAGAACGCGAAAAAGCGGAAAAAGCAGCAGAGAAGAAACGACGACGAGAGGAGCAGAGACAGAAAGATAAACTGAAGATTCGAAAGCTCGCCTTAAAACCCCGCAGTTACTG
GATTAAACAAGCCCAACAAGCCGTAAACGCCTTCATCAGAGAAAGAGACCGCGACTTACCATGTATCTCATGCGGAACGCTCACGTCTGCTCAGTGGGATGCCGGACATTACCGGACAACT
GCTGCGGCACCTCAACTCCGATTTGATGAACGCAATATTCACAAGCAATGCGTGGTGTGTAACCAGCATAAAAGCGGAAATCTCGTTCCGTATCGCGTCGAACTGATTAACCGAATCGGGCA
GGAAGCAGTAGACGAAATCGAATCAAACCATAACCGCCATCGCTGGACTGTCGAAGAGTGCAAGGCGATCAAGGCAGAGTACCAACAGAAACTCAAAGACCTGCGAAATAGCAGAAGTG
AGGCCGCATGA
>EGKIKD_10735 Protein ninH
ATGACGTTCTCAGTAAAAACCATTCCAGACATGCTCGTTGAAGCATACGGAAACCAGACAGAAGTGGCACGCAGACTGAAATGTAGTCGCGGCACGGTAAGAAAATACGTTGATGATAAAG
ACGGGAAAATGCACGCCATCGTCAACGACGTTCTTATGGTTCATCGCGGATGGAGTGAAAGAGATGCGCTATTACGAAAGAATTGA
>EGKIKD_10740 Serine/threonine-protein phosphatase
ATGCGCTATTACGAAAGAATTGATGGCAGCAAATACCGAAATATTTGGGTAGTTGGCGATCTGCACGGATGCTACACGAACCTGATGAAAAAACTGGAGACGATAGGATTCGACACCAAAA
AAGACCTGCTTATCTCGGTTGGCGATTTGGTCGATCGCGGTACAGAGAACGTCGAATGTCTGGAATTAATCACATTCCCCTGGTTCCGAGCTGTACGTGGAAACCATGAGCAAATGATGATT
GATGGCTTATCAGAGCGTGGAAACGTTAATCACTGGCTGCTTAATGGCGGTGGCTGGTTCTTCAATCTCGATTACGACAAAGAAATTCTGGCTAAAGCTCTTGCCCATAAAGCAGATGAACT
TCCGTTAATCATTGAACTGGTGAGCAAAGGTAAAAAATATGTCATCTGCCACGCCGATTATCCTTGTGACGAATACGAGTTTGGAAAGCCAGTTGATCATCAGCAGGTAATCTGGAACCGCG
AACGAATCAGCAACTCACAAGACGGGATCGTGAAAGAAATCAAAGGAGCGGACACGTTCATCTTTGGTCATACGCCAGCAGTGAAACCACTCAAGTTTGCCAACCAGATGTATATCGATAC
CGGCGCAGTGTTCTGCGGAAACCTCACATTGATTCAGGTACAGGGAGAAGGCGCATGGGCATAA
>EGKIKD_10745 Antiterminator
ATGGGCATAAGAGAACTAAACCTCACCAAAGAGCAGCACGAGTGGCTGAATGGCTGGCTTGAACTGTGGGGCGCATGGGTTTATTCAGGTCGTCTGGAAAAGCGCATGAGCAGCGTAATA
GCGAAGTTCATGGAGAGCGTAGAGCCGGGAAGAGTTATGACAAGGCCAATGTGCAATGATGATGATGGAATGTTGATTTCTCAGGTCGTCGATTCCGTCATGTACATTGACAAGAAAGCCT
TTGGCATCCTCCTCAGCTACTACGCTCATGGTTCATCTAAGCGAGCAATTGCATCCTACTATCACGCGACTGCAAAGCCACGCAAGATGTGTGGACGTGGTGGCGATGGATGGAGAAAACCT

TCACTGGCAACCTGTAGAAACGAAATTGACGACATCCTGAAAGCGTCGTTATTTGTTTTATACCAGCCAATGCAAAATGCTTTCAAAATGCGTAAACGTGTTGAGAAAGTTAAGCATGTTGCT
GTTAAAAACCTTGACATGCAATTATCCATTTAG
>EGKIKD_10750 HNH endonuclease
ATGAAAGAAATAAAATTAACGCCAGAAATGGTGCTTTCTGTTGTTGATTACAATCCATCATCAGGCGACTTTCACTGGAGATGGAGGCAGGGGAGAGAGAGGACCACTTTGACATGGAACT
CTCGTTTTGCTTTCAAGAAATGCTCATCAATAAATTCTGATGGGTATTTAATGATTATGATTAATGGTAAAGCATACCCTGCTCACAGACTGGCATGGTTGATTGTTTATGGCACCATGCCCGAT
GGTTTTATTGATCACATCAACAGGGTAAGAACAGATAACCGGATATCAAATCTTCGTCTTGTCACTCATTCCGAAAATATGCAGAACAGGAAAATTCAGAAGAATAATAAATCTGGATACCGTG
GCGTGTCTTGGGATGCTAAGTACGGGAAATGGAGAGCAAGAATTAATGCGTCTGGAAAGTGTATTAACCTTGGATACCATGACACTGCCGAACTTGCCGCTGCGGCTTTTGAGGCCGCCAG
AATTAAATATCATACCGTTTAA
>EGKIKD_10755 Holin
ATGTACCGTATGGACAAAATCAGAGAATGGTTCAGTTACAGCTTCGGAGGACTGACTGCGATGGGTGGCATTCTCTCCCTGAATGACTGGGCTGTCATCATTGGTATTCTTTGTACTGTCGGC
ACATTTGGCATCAACTGGTACTACAAGCGCAAAGAGCGCGAGGACAGATTGAATGGCAATGTCACCGGCACTACGAAATAG
>EGKIKD_10760 lysozyme RrrD
ATGGCAATGTCACCGGCACTACGAAATAGCGTAATAGCGGCGATAAGTGGCGGGGCTATTGCTATAGCATCTGTGTTAATCACTGGACCAAGTGGTAACGATGGTCTGGAAGGTGTCAACTA
CATACCATACAAAGATATTGTTGGCGTATGGACTGTATGTCACGGACACACCGGAAAAGACATCATGCTAGGTAAAACGTATACCGAAGCAGAATGCAAAGCCCTCCTGAATAAAGACCTTG
CCACGGTCGCCAGACAAATTAACCCGTACATCAAAGTCGATATACCGGAAACAACGCGCGGCGCTCTTTACTCGTTCGTCTACAACGTTGGTGCTGGCAATTTCAGAACATCGACGCTTCTTC
GCAAAATAAACCAGGGCGATATCAAAGGTGCATGTGATCAGTTACGTCGTTGGACATATGCTGGCGGTAAGCAATGGAAAGGGCTGATGACTCGCCGTGAGATTGAGCGTGAAGTCTGTTT
GTGGGGGCAACAATGA
>EGKIKD_10765 Spanin, inner membrane subunit
ATGAGCAGAGTAAGCGCGATTATCTCCGCTCTGGTTATCTGCATCATCGTCTGCCTGTCATGGGCTGTTAATCATTACCGTGATAACGCCATCGCCTACAAAGAACAGCGAGATAAAAAAGTCA
GTGAGCTGAAGCAGGCGACCGCCACCATTACTGACATGCAGCAGCGCCAGCGTTCTGCTGATGCACTCGATGCTAAATACACGAAGGAGTTAGCTGATGCGAAAGCTGAAAATGATGCTCT
TCGGCGCAAGCTTGATAATGGCGGCAGGGTGCTCGTCAAAGGAAAATGCCCTGTGCCATCCTCAGCCGAAACCTCCGGCGCCTCCGGCATGGGCAATGATGCCACCGTCGAACTCTCTCCA
GTTGCTGGACGAAACGTTCTCAGTATCAGATCTGGAATCATCAGCGACCAAACATCACTGAGAACGCTTCAGGAATACATCAACACGCAGTGCCTGAAATGA
>EGKIKD_10770 hypothetical protein
ATGTCATCTCCAATCATGAAGTATTTCGCTTATCAACACCTCCCTGCGCATCTGCAGGAAGTAAGTAAGCCAATCGGTGAACTTGCGACACTGATGGATGAATCACTGCCGGACAGTGCTGAA
AAGTCAGCAGGTCTCCGCAAGCTGCTCGAAGCTAAAGATGCGCTGGTGCGAGCAAAGCTGGGGTAA
>EGKIKD_10775 Uncharacterized protein in gp15-gp3 intergenic region
ATGGCAGAGATTATTTCCATGACTGAAGAACAGAAATTCCAGTTAGAGATTTACAAACTGGTCATGAACCAGAACGCAGCCGCAGAGGAAGCATTTCAATTCATTGGCACTGACGAGCTGA
AGCTTGAACTATTCAAAATTCACTTCCAGTCAGGCGGCGCTAATTCGGATATCACGATCCGCACATTCGAAGCAGTGCGTAAATCTAAGGAAGCGTTAGACCTGTTCACCACCGGAGCATGA
>EGKIKD_10780 Transcriptional regulator
GTGGGTTACGTAATCAATTTGGGTAAGGAGAAGAAATTCCCAATTACTCAAGAGCTATACGAGCGGCTGGAAAGCGCCATTCATGATTACGATGGTGAAATCAGTTTATGCGAGGCGATTGG
CACACTCGAATTGCTGAAGCAGTCATTGATTGAAGGCGCGAAAGAGCCATCAACCTGA
>EGKIKD_10785 hypothetical protein
ATGGCGACTGAACCAAAAGCTGGTCGCCCCTCTGATTATATGCCGGAGGTGGCTGACGATATCTGCTCGTTGCTTTCTTCTGGCGAAAGTTTGCTGAAAGTATGTAAGCGTCCTGGTATGCCG
GATAAGTCCACTGTTTTCCGCTGGTTGGCAAAGCATGAGGATTTTCGCGACAAGTACGCGAAGGCAACTGAGGCACGAGCTGATTCTATTTTCGAAGAGATATTCGAAATTGCTGACACTG
CGATTCCAGATGCTGCTGAGGTGGCAAAGGCAAGACTTCGCGTTGATACCCGCAAATGGGCGCTGGCCCGAATGAATCCCCGTAAGTATGGCGACAAGGTAACTAACGAGCTTGTCGGCA
AAGACGGCGGCGCAATCCAGATTGAAACATCACCGATGAGCACTCTATTCGGAAAATGA
>EGKIKD_10790 PBSX family phage terminase large subunit
ATGACCTCGATTAATCCTATCTTTGAACCGTTCATTGAGGCGCATCGCTACAAAGTCGCCAAAGGCGGTCGAGGTAGCGGTAAGTCATGGGCAATTGCGAGACTGCTTGTTGAAGCGGCGC
GTCGGCAGCCGGTGCGTATTCTCTGCGCTCGCGAACTGCAAAACAGTATCAGCGATTCGGTAATCCGGTTGCTTGAAGATACCATCGAGCGTGAAGGGTATTCGGCTGAGTTTGAAATTCA
GCGTTCCATGATTCGTCATCTCGGAACGAATGCTGAATTCATGTTCTACGGCATCAAAAACAACCCGACGAAGATTAAATCGCTCGAAGGTATTGATATCTGCTGGGTGGAGGAAGCGGAAG
CGGTAACGAAGGAATCATGGGATATTCTGATACCAACCATCCGCAAGACGTTTTCCGAAATATGGGTGAGCTTCAACCCTAAGAACATCCTCGACGATACCTATCAGCGATTCGTTGTAAATCC
TCCCGATGATATTTGCCTGCTGACGGTGAACTACACCGACAACCCGCACTTTCCTGAAGTTCTCCGTCTGGAGATGGAAGAGTGTAAACGCAGAAACCCGACACTGTATCGTCACATCTGGC
TTGGTGAGCCAGTGAGCGCAAGTGATATGGCAATCATCAAACGTGAATGGCTTGAAGCTGCTACCGATGCGCACAAGAAACTCGGATGGAAAGCGAAAGGCGCGGTTGTTTCTGCTCATG
ACCCATCAGATACAGGGCCAGATGCTAAAGGTTACGCATCGCGTCACGGTTCGGTGGTTAAGCGCATTGCCGAAGGCCTGCTGATGGACATCAACGAGGGGGCTGACTGGGCTACTTCTCT
TGCGATTGAAGACGGCGCTGACCACTACTTGTGGGATGGTGATGGCGTCGGTGCGGGGCTACGCAGACAGACAACGGAAGTGTTCTCCGGCAAGAAAATCACCGCCACGATGTTCAAGG
GCAGCGAATCGCCATTCGATGAAGATGCACTGTATCAGGCCGGAGCATGGGCCGATGAAGTCGTACAGGGCGACAACGTTCGCACTATTGGCGATGTGTTCCGCAATAAGCGAGCACAATT
CTATTACGCGCTGGCTGACAGGCTGTATCTGACATATCGGGCGGTTGTTCACGGTGAGTATGCAGACCCAGACGACATGCTGAGTTTCGACAAAGAAGCGATAGGCGAGAAGATGCTGGA
GAAGTTGTTTGCAGAACTGACGCAGATTCAGCGCAAATTCAATAACAACGGGAAGCTGGAGCTAATGACTAAGGTCGAAATGAAGCAGAAGCTCGGTATTCCATCTCCTAACCTGGCTGAT
GCGTTGATGATGTGTATGCATTGTCCGGAGTCGGCTGCGCAACCCGACTATTCCAGTTACTCAATTCCTTGTGGTGTAGGTTGA
>EGKIKD_10795 Portal protein
ATGGCAGAAAAAAAGATGACTGACTGGCATCGCAAGGTGCTGTGCAACTTTGATAATGCCTGGTCAGCAACGCAGGATATGCGTGAGCAGATTATTGAGGCTCAACGTTTCGTCCGGGTGT
CCGGCGCACAGTGGGAAGGCAGCACAAACGCTGGTTATTCATTTGATGAAGGCAGGTTTGAGCATTACCCGCGCTTTGAACTGAATAAGATTGCCCGTGAATGTGATCGCATCATTGGCGA
GTATCGACAGAATCGCATCAGCGTTAAATTCAGGCCGAAGGATGACAAGGCATCGGAAGCGTTGGCCGAAAAGATGAACGGCAAATTCCGCGCTGACTATCAGGAAACATCCGGTGGTGA
AGCGTGTGATAACGCATTTGATGATGCTGTAACGGGCGGATTCGGTTGTTTCCGCATGTGTGCCGATTACGAAGATGAAATGGATCCGAGTAACGAGCAGCGACGCATCAGCCTTCTTCCTG
TTTACGACCCAGCGACATGCGTCTTCTTCGATCAGGACAGCAAGCAATATGACCGCTCTGATGCTATGTGGGCTATGGAAATGTTTTCCATGACGCCTAAAGCGTTCGAAGCTGAATACCCTG
ATTCCATCGCTGCAAGTCTTTCTCGTGATGACACTGGCACTCAATATGACTGGTCAACTCCTGATGCTATCTATGTTGGTCGCTACTATGAAGTCCGCATAGAGAAGGTGAAGCTCACGGCGTG
GCGCAACCCTGTCAGCGGAGAAACGGCAATCTATGATGAAGAGCAAATCAAAGATATTGTCGACGAACTGACCGATGGTGCATTCGAACTGATTGGCGAGCGAACGGTGAAGAAGCGCC
GCGTTTATTGCGGTCTTCTGTCTGGCGCTGAATGGCTGGAAGAACCGAAGCGTATTCCGGGTGAACATATTCCTCTCATCCCGGTATATGGGCGTCGCTCATTTGTTGATAATCAGGAGCGAA
TCGAAGGCCACGCAGCAAAAGCGATGGATGCACAGCGTCTTGAGAACCTGATGGTTTCCATGATTGCAGATAACGCCACTCAGGCTGGCGGTGATGGCATTCCTATCGTGGATGTTGATTTC
ATTCCCGGCCCATTAATGAATCACTGGGCAGAGAGGAATAAGAAAAGACCTGCAGTTCTTCCTATGACCAGTAAGAAGGACAAAAACGGAACAGTCATTTCAGAAGCTCAGGTTGCTGGCT
GGACACCTCCGACACAAATGCCGCCAGCTCTTGCCGGGCTATTGCAGTACACCGGAACGGCTATTCAGCAAATTACAGGTGCGTCGCAGCTTGAGAACATGCCGAGCAACGTCGCCACCG
ATACCGTTGATAGCATTTTTAACCGGATGGATACGCAGTCCTATATCTACATGGACAACATGGCTAAATCCATGCGCCGCGCTGGCGTCGTGTGGCTTTCTATGGCGCGTGAGGTCTATGGCAG
TGATACTCCGATGCGTATCGTTAATGAGGACGGCAGCGATGACGTGGCGCTGATGACTGGTGAAGTGGTTGACCGTCAGACAGGGCAGGTTATCGCGCTTAATGACCTTTCGCAGGGCAAC
TATGAAGTGACTGTCGATGTCGGTCAGTCGTTCGCTACTCGCCGTGATGCAACGGTTAAGTCGTTACTTTCCATGCTGGCACTTATCCCACCAGGAACGCCGAAGCACGACCTTGTATCGTCG
ATGATTCTCGACAATATGGACGGCGAAGGGATGGACGACCTTAAAGAATACAACCGCAATCAGTTGCTTCTGTCTGGAGTTATCAAGCCGAGAACGCCAGAAGAGCAGCAAATGGTTGAG
CAGGCGAAACAACAACAGGCGAGTCAGCCAGATCCGGCTATGGTTGCTGCGCAAGGTCAGCTTCTTGCTGGTCAGGCTGAATTGCAGAAAGCGCAGAACGAACAAGCAGCCATTCAGGT
TAAGGCATTCCAGGCACAGACGGATGCTCAGGTTGCTGCGGCAAATGTTGTGAAAATCCTCGCATCTGCCGATAGCCAGCAAAAATCTGATATCCGTGAGGCGCTGAAACTGCTCGGACAG
TTCCAGCAACAGCAAGGAGATAATGCCCGTGCTGATGCAGAGCTTGTCCTGAAAAGTCAGGCACAGGGCCATGCGCAGCGCATGGACATCGGCAGCATCCTGCAAAAATCAACTCAGCAA
CAACCACAGCAGTAA
>EGKIKD_10800 Scaffolding protein

ATGGACCAGATGGCAGAAAACACACCAGAAGTTGAAATCGAAACCGATACATCAGAGCAGATTCCTGATGATGTCGAACTGGCTGAAGAAGTCGAAACAGAAGATGGCAGTGAGTCCTCC
GGCAATGATGCAGAGGAAGATACTGAAACTGATGACGACGAATCAGAACAGGAATTCTACTTTGGTGACGAAAAGCTGGATTCGCCAACCAGCGAAGATGGCGCAGAGCATGGACTGGT
AAAACACCTGCGCAAGACGATTAAAGAGAAAGACCGCGAGCTGAAAGAGTTGATGCGTCAGTCTCAGAAACCCGTCGAGCAGCAGCCGGTAATCACTCAACCACCGCGAATGCCAAAAC
TGGATGATGAGGACATCGGTTTCGATGAAGAAATCTATCAGCAACGTATGGCTAAGTGGGCAGAGGAAAACGGCAAATACCAGGAGCAAGTACGAGAGCGGAAACGAGAGGAAGAGGC
GCGTACCGCAACGCTTCAGCAGAAAGCAGCCAATTACATGCAGAGAGTAAAAGCACTGAAAGTGGCTGGCTACCAGGATGCAGAGCAGGCTGTACGCGAAGATGTTCCTGTTCATATTCA
GGACATGATCCTTCTTGAGTCAGAGAAGCCGGAAATCGTTGTTCTGGCACTCGGTCGCAACGCTGAACTGCGCAAGCAACTGGCAGAAGCTACCAACCCCGTAGCAATTGGTCGTCTGCTG
GAACGTATCGAATCGAAGGCCAGAATCATGCCAAAAGCAAAAACCACGGCAGCCACAACCCCGACAGTTAAGGGGAGCAACGGCGCAGTAATCAACAACCTCGACAAACTGAAAGCCAA
GGCGCTGGAAACTGGTGACTGGACGCCGTATTTCGCCGCTAAAAAGGCAAAAAAATAA
>EGKIKD_10805 Coat protein
ATGGCTAACCAATTAGCAAAAGACCTTGAAATCATGTTCGAAAACTACGTTGAAGGCTTTGAGGCCGCCTGCGTAGTTTCCCGTAACGCTAAAAAATTCCGTCCCGGTGATACAGCAATGCA
GCGAGCAGGTGATGTTCTGTATCGTCCGCAGCATTACCACATGAACATTGAGGAAGGCCTCGACCTCAGCAACAAAACGCCAACAGCACTGGTTCAGCGCCTTGTTCCTTCTGTGTTCAAG
GAGCCGAAAAACATTCTGTACACTCTGGATGCGCGTGAAATGCGTGACCCGGAACATAAAACTGAAGCTGGTCGCGCCGCAGGTATGCGCCTTGCTGCACAGATTGACTCTGACCTGATTT
CCATGGTTACGCAGCGTGCTACTAACGTGATCACGATGGCTGATTCAACCACAGGCACACAGGGCCGTGATTTGTGGAACTGTGCGGCAGGTATTGATGCCACCATGACGGCGATTGGTGT
ACCGCAGGGTATCAACCGTCGCTCTTTCTGGAACCCATTCAACTACAAAGACCTTGCTGGCGAGCTTGGTCACCGTGCCTATGCTCAGGGTGCAACCCTGACAGCATACGAAAAAGCGCAG
ATCCCTCCGGTTGCGTCCTTCGATAGCTACAAGACCGATATTTCTGGTCGTGTTCCGAAGGGTACAGCAACTTCCATTACGCTGGCAGCAGCACCTGCGCACAAGGTTGAAGCGAAAGATGC
TAACGATATGCCAGTGGATAACCGACAGGGGACCATTACGGTATCTGCTGAAGGTTTGCAGGTTGGCGATGCGTTTACCATCGCAGGGGTGAATTCCGTACACCAGATCACCAAAGATACCA
CCGGGCAGCCGCAGGTATTCCGCGTTCTGGCAGTTAGCGGAACGACAGTAACTATCTCCCCGAAAATTCTGCCGCCTGACAACGCGGATGTCGCCAGCCGTCCATATGCAAACGTTGATGCT
AATGCGGCAAGTAGCGCAGCAATCACCATTCTCAACAAAAATGCCGCACCGGCTAACCTGTTCTGGGCTGATGGTTCTGTTGAACTGATGTACGGCAAACTGGCGTTCCCAACTGGTCAGG
GTCCACAGGTAATGACAGCAACCACCGAGCAGGGCGCTACGCTGATCATGTCTTACGCCTTCGACCACATCAAAGGCGTAACCACTGCGCGTTTCACCACTCTGTACGGTTGCTCTGTACTG
GTTCCTGAATATACGGGCATCGTTATTGCCGGGCAGTAA
>EGKIKD_10810 hypothetical protein
ATGGCACGAACAATGCTCTATAAGCCTGGCAACATGATCACCTGTGGTCAGTTTGCTGTCGATTACGTCATTGTTGATGACGAAGAAGTTAAATCTCACCTGAAAAAAGGTTGGGTAAAAAC
TCCTGAAGAAACCGCAACGAAGCAAAAAGTGGCTAAGGCGGAAGAAGATGGCGAAAACGAAGGGTGA
>EGKIKD_10815 DNA stabilization protein
ATGGCGAAAACGAAGGGTGATCTCGTTCTAAAGGCTTTACGAAAAGCCGGGCTGTATTCCAATGCCACGTTGACAGATGCTGACCCTCAGGCAATTGAAGATGCCATTAATGACCTCGAAG
ACATGATGGCAGCATGGCAGGCTAAAGGTATCGAGCTTGGATATCAGTTTGCTGATACAGAAAACGGCATCATGCCGTTACCTGACGATGATTCAGGTATCCCTGCATGGGCAAATGATGGC
GTCGCTTTGAAACTCGCTGTGCAAGTGTGCATGGATAACGTCATTCAGCCGTCAGACGCTCTCCTTACCGCTGCTGACAGTGCATATCAGACAATCTGTATCGCTTTAACCAAAATACCACCAC
TTGAGCGGCGAAATGACATGCCTCGCGGTAGTGGTAACAAAAGCGCGTTTACGTGGAATCGGTTTTACATCGAGAAAGATGATCCGAGTACGTGA
>EGKIKD_10820 Phage head DNA stabilization protein
ATGCCGATTCAGCAACTTCCGCTCATGAAAGGCGTCGGCAAAGACTTCCGAAACGCCGACTATATCGACTATCTGCCAGTGAATATGCTGGCAACACCCAAAGAAATCCTTAACAGCAGCGG
CTATCTCCGCTCATTCCCTGGCATTACCAAACGTTATGATATGAACGGCGTATCGCGTGGAGTTGAGTACAACACCGCTCAGAATGCTGTTTATCGTGTTTGTGGTGGCAAGCTCTACAAAGG
AGAAAGCGAGGTTGGTGATGTTGCCGGAAGTGGTCGCGTATCAATGGCACATGGTCGCACATCACAGGCGGTAGGCGTTAATGGTCAACTGGTCGAGTATCGCTATGATGGTACGGTTAAA
ACCGTCTCAAACTGGCCTGCAGACAGCGGATTCACGCAGTATGAGTTAGGCTCGGTCCGCGACATTACGCGCTTACGTGGGCGTTATGCGTGGTCAAAAGACGGAACTGATTCATGGTTTG
TCACTGACCTTGAAGACGAATCGCATCCTGACCGATACAGTGCACAATATCGCGCAGAATCGCAGCCGGACGGCATCATCGGTATCGGGACATGGCGAGACTTCATCGTCTGCTTTGGTTCG
TCAACGATAGAGTATTTCTCTCTGACAGGCGCAACCACCGCTGGCGCTGCGTTGTATGTCGCACAGCCATCGCTGATGGTACAGAAGGGCATTGCCGGAACATACTGTAAAACGCCATTCGC
TGATTCATATGCATTCATCAGTCACCCGGCTACTGGCGCACCTTCCGTCTACATCATCGGGTCAGGGCAGGCTTCACCAATTGCGACGGCCAGTATTGAGAAAATTATCCGCTCATACACGGCT
GATGAACTGGCGACGGGTGTAATGGAGACTTTGCGCTTCGATTCTCATGAGCTTCTGATTATTCATCTCCCTCGTCATGTTCTGGTTTACGACGCATCGTCAAGTCAGAACGGACCACAATGG
TGCGTGCTGAAAACCGGGCTTTACGATGATGTATATCGTGCTGTCGACTTCATGTATGAAGGCAACCAGATTACGTGCGGCGATAAATCAGAAGCGGTGACAGGACAACTGCAATTTGACAT
TAGTAGCCAGTACGAAAAACAACAAGAGTTCATCTTGTTCACGCCAATCTTCAAAGCAGAGAACGCCAGATGCTTCGACCTCGAAGTTGAATCATCCACTGGTGTTGCTCAATACGCCGACC
GCCTGTTCCTGTCTGCAACAACTGACGGTATCAATTACGGTCGTGAACAGATGATTGAGCAGAACGAGCCGTTTGTGTACGACAAGCGCGTTTTATGGAAACGTGTTGGGCGCATTCGTCG
ATTAATCGGATTCAAACTGCGGGTAATCACCAAATCGCCAGTAACACTATCCGGGTGTCAAATTCGTCTGGAGTAA
>EGKIKD_10825 Phage tail protein
ATGGCAGACCCGTCACTTAATAAGCCTGTCGTGGTTCAGGCTACACGCATTGATGCTTCAATTCTTCCCCGTAACGTCTTTAGCCAGTCTTATTTGCTCTATGTAATCGCGCAGGGAGCTGACG
TTGGTGCTATTGCAGGCAAGGCAAATGAGGCAGGGAGTGGCGCTTACGACGCACAGGTTAAAAATGATGAGCAGGATGTAATTCTTGATGAGCACGAAAAAAGAATTGCAAAAACAGAA
GAGGATATTTCAGGAATAAAAGTAAAGCTTCTTGAAATAGAGAATGATGTTAATGGTCTGAAAATAAAAGTTGAGGATATCGACGGTAAAGTATCAGAGATAATCGTTGATTATGTTTCACTC
AGCAGAACAGGAACTCAAACTCTTGCCTCATCCCTTAACGTATCAGGAAGTTATTCTGTTAACGGTACAAAAGTTGTTGGCGCTCGCCAGACTGGATGGACCGCGGCAACAGGTACGGCGA
ATAAAGGCGTATTCGATGCTGACCTGACATTCGCCGTTAGCGATACTTACACGCAATCTGAAATCCAGGCTATAGCCAATGCTCTAATTACTGAGCGTCGGCGCACTAAGGCTTTGGAAGACG
CCTTGCGTGCACATGGGTTGATTGATTAA
>EGKIKD_10830 Protein gp14
ATGATTACATTCACTCCAACACGCAACATCGACCTGATAGAAATGGTTGGCAACCACCCCGACATCATTGCCGGAAGCAACAACGGTGACGGATACGACTACAAGCCTGAGTGTCGTTACTT
TGAAGTGAACGTACATGGTCAGTTCGGTGGCATCGTGTATTACAACGAGATTCAGCCGATGACCTTTGACTGCCACGCCATGTACCTGCCTGAGATTCGCGGATTCAGTAAGGAAATCGGAC
TGGCGTTCTGGCGATACATTCTCACCAATACCACCGTTCAGTGCGTTACATCATTTGCTGCACGCAAATTTCGCCACGGTCAGATGTACTGCGCAATGATTGGCCTTAAGCGTGTAGGAACCA
TCAAGAAATACTTCAAAGGCGTGGATGACGTGACTTTTTACAGCGCCACACGCGAAGAACTAATCGACTTCCTGAATCACGGGAGATAG
>EGKIKD_10835 Internal virion protein gp7
ATGTTATATGCATTTAAGCTGGGCAGAAAACTGCGCGGCGAGGAACCTTATTGCCCTGAAAAAGGCGGGAAAGGTGGCAGTTCTGATAAAAGTGCAAAGTATGCCGCAGAAGCTCAGAAG
TATGCCGCAGACCTGCAAAATAAGCAGTTCAACACCATCATGAACAACCTGAAGCCGTTTACTCCTCTGGCTGATAAGTATCTCGGCAGCCTCGAGAACTTATCGTCTCTGGAGGGGCAAGG
TCAGGCGCTAAACCAGTATTACAACTCTCAGCAGTACAAAGATCTTGCTGGTCAGGCTCGCTATCAGAGTCTGGCTGCAGCGGAAGCAACAGGTGGATTGGGTTCCACCGCAACCAGTAAT
CAGTTAGCAACAATCGCACCAACGCTTGGTCAGCAATGGCTATCTGGACAAATGAACAATTACAACAACCTGGCAAATATCGGTCTTGGCGCTCTTCAGGGGCAGGCAAACGCCGGGCAAA
CATATGCCAACAACATGAGTCAGATTTCACAGCAAAGCGCGGCGCTGGCGGCGGCAAACGCCAACCGACCGTCAGCATTGCAGCAGGGTGTTAGTGGTGCTGCATCCGGTGCGCTTTTGG
GTGGTGGCATAGCCAGTGCTCTCGAGCTATCAACTCCGTGGGGTGCTGGTATCGGTGCTGGTCTTGGTCTGCTTGGCTCGTTGTTTTAA
>EGKIKD_10840 Internal virion protein gp20
ATGGCTACGTGGCAACAGGGTATTAATTCTGGTGGTTTTCTGGCTGGAATTGGTGCGCAAAATGAGAATGCGCCAAAGGCAAGCGACATTAACGCAACGCTTGGTCTGATCCGCGAAAACA
ATGAACTGGCTCGCTCAGGTGCAAATAACGTTGGTCTGACCGCGTTACGTGGTCTGGCTGGAGTTGCTGATATTTATAAGCAGGAACAGCAACAGAAAGCGATTAATGCGTTCAATAAGGTT
CATGCTGATGCATGGGCTTCTGGTGATCCATCGGGACTATTTAAGTTTGCCAAGGAAAATCCAGCGTTTGTTGCGCAGGCACAACAGGCGTTTTCCGGTCTTAATGAGCAGCAACGCAACG
ATATGGGCGATTTAGCCATGAGGGCTAACGTCGCTCTTTCTCAGGGACCGGAAGCCTACAGTAAATTCATTACTGACAACAAGGACAGGTTAAATCGCGTTGGTGCTAATGCTGACTGGATG
ATTCAGACAGGTATCCAGAATCCAGAGCAGCTATCACACATGCTGACTACTATGTCTCTCGGTGCGCTTGGACCAGAAAAGGCGTTTGCTGTTCAGGACAAGATGGCTGGTCGTGAGATTGA
TCGCGGAAAACTTGCAGAGACAATCCGCAGCAATCAGGCTGGCGAGGCGCTAACAGCGCGTGGTCAGAATATCACGATGCGCGGTCAGGATTTATCGATGCAGAGAGCATCAATGAAAGG
GTCGGTTGGGAATAATGAGCGTACAGTTCAGTTAGCAGATGGCAGAACTGTAACGGTAGGCGGGAAGCTTCACGGCGCTGGGGCTAATGCGTTCTACGAAGGTATCGACAACGAGGGGA
ATATGGTTCGCGTCCCTGCTGGCTCTATTGCCGCTCCGGCTACATCGGCAGCAAGCGCACAGAATTACGCAATGAAGAAAGATCTTGATGCAATTTCTGGTGCATCAATTGACGATCTTGGCT
TCATGACTGGCATTACAGGCTCTTCAGGTTCTCCTGCTCTTGGTGCAGATATTCGTAGCCGTGCATCTGGTGGTGATCAGAGGAAACTATACAACGCGGCACAGCGAATCCAAGGAAAGATG

CAGAATCAGGGCATTGCAGCAGCCAGAGACATGGGGGCATCCGGTATCAACACCGTTGCAGAAGCAAAGATGTATTTTCAAGGTATGCCACAGGTTGATTTCTCAAGCCCTGAAGCACTGC
AACAATCAATGCGCGACATTCAGCAATATACCGACAATTACAACCAGCAATATAACGTTAATGTTGGTAAATCTCAGCGGCAGCAATCTCAACCTGCACAGGTATCACAGCCAGCAGCCAGCA
GTAACTTTTCTTCACTATGGGGTGATTAA
>EGKIKD_10845 DNA transfer protein
ATGGCTAAAGCATGGAAAGATGTTATCGCTTCTCCACAGTATCAGGCGTTAACTGAAGAACAGAAAGCACAGGCTCAAGCGCAATATTTTGATGAGGTTGTTGCCCCTAAGGCTGGTGACA
AATGGGCTGAAGCAAGAGATCAGTTTTATGCAGCATACCCTCCGCCTCAGCAGCAGAAAGAAGAACCATCATTGATGCAACAAGCTGGCGATTGGCTCACTGGTGATCAAAGTGCAGGGC
AAATTGCAGAACAGGCTGGTCGTGGTCTGGTAAACATACCATTTGACGTATTGCAGGGTGGCGCAAGTCTGATTAATTCAATCAGTCAGGGGCTTGGTGGGCCAAAAGTTTTGGATGATGT
TTATCGTCCAGTAGATAAACCGACAGACCCATACGCACAAGCCGGTGAAACAATTGGTGGGTATTTAGTTCCAGGAGTTGGAACGGCAGGAAGCATAGCTATTGGATCGCTGGCAGAGGCC
GCAAATCAGAAAGGCGATTTCGCACAAAATGCAGCTAAAAATGCCGGAGTTAACCTTGCCGCTCAGGGTGTTCTTTCCGCAGCAGCAAAGGGAATAGGGCGTGGAATAACGGCTATAAAA
GGTGATATTGCGCCAGAAGTGGCGAAGAAAATTGCCACATCAGAATCGATGGGCGTGACACCAATGACATCTGATGTTATCCCGCCGAAAAATGCTTTCACTCGCGGCCTTACTCAGGATGC
CGAGGGGGCTTTGCTCGGGACGGGCTCAAAGCGAGCAGAGCAATATGCAACGCGTAGTAAGCTGGTAAGCAATTATTTTGACCGTTTTGGTGAGTACAACCCGGATGATGTGGTGAAATC
TCTGACCACCACGTTAAGGGGACGGAAGGATGCCGCTGGCGCTGTTATCAATGACGTCACCAATAAAATGGGGAATGCCGCAGTTGATACCACAAACACTATGAATGCTCTGAATACAGCG
ATCGCAAGACAGGAACGGCTTGGGACGTCTGCCAATCAAAGCCTGCTTACATCCTTGCGTAACCTACGCGAAGAATTAGCAAACCCTGCAACTGATTTGGATGTTACGTTTGATCTCTTGCG
TCAGCACAGAACTGCATTTAGATCTAATGTTCAGGGAGATGCTATGGTCTTCCCCAACCAGGCAAAAGCAGCTACCAATATGGTAGAGAATGCAATGTCAAAAGACCTTCGTAACGCAGTTG
CAAAAAACCTCGGTGCGTCAGACGCAGCAAAATACCTTAAAGCAAATTCCGATTATGCAAACGTTTATAATAAGGTGCTTAATAAAAACATTGCTAACAAGCTCAACAAGGCAAGCAGTGAA
GCCAGTCCTGAACTTATAAATACCGTTGTATTAAGCAGAAAACCATCTGACGTGAAACGAATCTGGAGCGCATTGGATGATAAAGGGAAAGATGCTATGCGTGCAGCTTACGTCAGCAAAAT
AGCGGAAAAGGCCGGTGACTCTCCAGCCAAGTTCATCACTGAAGTTAATAAGCTGAAATCTCAGTCAGGCGGTGAAATTTACAACACTATTTTTTCTGGAAAGCACATGAAAGAGCTTGAT
TCTCTTCATGAAGTTCTACAGCAAACAGCAAGGTCAGACACCGCAAATGTAGTAACTCAGACGGGGCAATCGCAAGCCAACCGGATAAGGACGATTGGCGCAACTGCGACTCTTGGCGTAT
CAATGGGGCTTGAGGCTGGCTTTGGTGCAATGATGCGTTTGTATGAGTCTAAAGCAGCAAGAAACATGCTTCTCCGCCTTGCAAACGTCAAGCCTGGAACTCCGGCATATGAGCGAGCGTT
AAATCAGGCTGCTAACGCCGTTCGCCCTCTCCTAACTAACGAAGCTACCAGGCAGTAG
>EGKIKD_10850 hypothetical protein
GTGTTAGAAATAGTCGTACTCGCTCTTGGGATATCCTGTTGCGTACTATATGCAGGGTTAGCTGCCCTCAAGAAACAGGTTAAGGAATTAGATCGCTCACATGAAATTGATACAAAAATTGCG
CGATTAACAGAAGAGAATAAACACTTAAAAAATTCCATAAGGGCACTAACTGATGACAACTACAAACTGTCCCATGCATTGGCTAAGTGGGAAATAGTAAGTTATGAAAGAATGACCGACAT
GATTTTTTCGTCTTATATGGCTACAAAATCTCCTGAAACATCAGGAAAAGCAATAATTGCAGCCATTGAAAAGAGAATTAAATAG
>EGKIKD_10855 PEGA domain-containing protein
ATGAAGAGGATTATCGGCGTTGTTGCTGGCGCTATATTGTTATCTGGGTGCGCAACTATTGTTGGTGACGAAACACAGCTCGTGCAAGTGAACAGCAATCCTTCCGGTGCGAGCTTTAAAGT
AAAAGACGAATCAGGCGTGATTGTTGCGCAAGGCAAGACCCCGCAAGGAGTAACTCTTGCCAAGTCAGATGGTAGTTATTTTGGCAAAAAGAGCTACCAGATCACTATGGAAAAGGATGG
GTACGAACCAGTTACCCTGCCAATCAAAGCCAATGCTAATGGTTGGTATATTGGTGGAAACCTTGTGTTTGGTGGGTTAATTGGTTGGCTTGCTGTAGATCCTTTTAATGGTGGGATGTATACC
TTGAAGCCAAAAGAGGCAAACGCATCTCTTGTACCATCAACAAAGCAAGACTAA
>EGKIKD_10860 Regulatory protein mnt
ATGGCTAGAGATGATCCGCACTTTAACTTCCGTATGCCTATGGAAGTAAGGGAGAAATTAAAATTCAGGGCGGAGGCGAATGGGAGATCAATGAACTCCGAGTTGTTACAAATCGTCCAAG
ATGCTCTATCAAAACCATCGCCTGTGACTGGATATCGCGACGATGCAGAACGACTCGCTGATGAGCAGTCAGAGCTTGTTAAGAAGATGGTGTTTAATACGCTGAAGGATTTGTACAAAAAA
CCCACCTGA
>EGKIKD_10865 Transcriptional regulator
ATGAAAGGAATGAGCAAGATGCCGCAGTTCAATTTGCGGTGGCCTAAAGAAGTATTGGATTTGGTACGCAAGGTGGCGGAAGAGAATGGTCGGTCTGTTAACTCTGAGATTTATCAGAGA
GTAATGGAAAGTTTTAAGAAGGAAGGACGCATTGGCGCGTAA
>EGKIKD_10870 sar RNA
TCAATATTTCCTAAGACGATTTGTTGATACCGAAGCCCTGACTGTTCCCGCAGTTGGGGCTTCAACT
>EGKIKD_10875 Bro-N domain-containing protein
ATGAAAAGTATAGCAAAGGCACAAAACGATTTCACCATCTTCAAATTTGGCGACAGTGAAATCCGCGTAATTAACAAGTGCGGCGAGCCGTGGTTTGTAGCTAAAGATGTTTGTGATGCTTT
AGCTTTGACTAACTCACGCAAGGCGCTTACTGCACTTGATGACGATGAAAAGGGAGTAACTTTAAGTTACACCCTTGGTGGTGAGCAGAATCTAAGCATTGTGAGCGAGTCAGGTATGTATA
CATTGGTTCTGCGCTGCCGCGATGCCGTCAATAAAGGTTCAGTCCCGCACAAATTCCGCAAGTGGGTAACAGCAGAAGTTCTGCCTTCAATTCGCAAACATGGCGAGTATGTGAAAGGCAA
GAAAACCACTGTTGAGGAAAGAACACCGCTACGCGATGCAGTAAACATGCTGGTAGGAAAGAAAGGACTTCGCTATGACGATGCATACAATATGGTTCATCAGCGTTTTGGTATTGACAGC
ATTGATGAACTTTCAATTGAACAAATCCCGCTGGCAGTAGAGTACATCCACAGGGTAGTGCTTGAAGGTGAGTTCATTGGCAAACAAGAGAAGAAAACCGACGAGCTTTCTGCAAAAGAA
GCAAACAGCCTTGTATGGTTATGGGATTATGCCAACCGCTCTCAGGCATTATTCCGCGAATTGTATCCGGCGCTAAAACAAATTCAATCGAACTATTCCGGCAGATGTCATGACTGCGGTTATG
AGTTCTCCCGTATTATCGATATGGCGAGAGACGTTTTAATCAATCACACACGAGATGTTGATATCAATGAGCCAGACGGACCAACGAATCTTTCCGCATGGATGAGACTTAAGAATAAAGAAT
TACCTCCTTCAGTACATAACTACTGA
>EGKIKD_10880 hypothetical protein
ATGACAGACATTACAGCCAATGTGATCGTATCGATGCCTTCGCAACTCTTCACTATGGCTCGTTCTTTTAAAGCCGTTGCCAATGGCAAAATTTATATCGGTAAAATTGACACTGACCCGGTAA
ATCCTGAAAACCAGATTCAGGTTTATGTAGAGAATGAAGACGGTTCTCACGTTCCTGTTTCGCAACCAATCATCATTAACGCCGCTGGATATCCGGTATATAACGGACAGATTGCTAAGTTTGT
AACTGTGCAGGGACATTCAATGGCTGTATACGATGCCAACAATGCTCAGCAGTTTTACTTCCCAAACGTGCTTAAGTACGACCCTGATCAGCTTGAGTACAGACTTAGTCAGCCTGATGGGTA
TCTATTAGTTGGTGGGCTAGCAGAACATTATAGCCTTCCTGTTAAGTTCGTTGTTGTAGATAACGCACCTTATAATGGAGATTTAAAAGCAGCACTAACAGCAGCAACATCTGGTAGTGTTTTT
TGGTTGGGTAAGAAAACGTATAACATTACGGGGTTATATGGTGTAAATAGAAACACCGTTGAGAATATTACTATTGTTGGCGCTGGAATGCCTCAGCTGTCATCTGACAAAAGATATCTCATGG
ACGGAACAGGAACCATCATTCAAGGTACAATTAAAAATCAGGCCAAGGGATTCAAAATATTTAATTTAGGTATTGACGTTGGTGATTATGTTTCTCAAAACGTTTATCCGTCGGTTACTTATGA
AGATGGCCTGCAACATTATGGCGCTGGCAGTAATGCCAACCTTGAAATAAATAATGTAAAGCTCCTTAACACCGTGACAGACCCGTCAAAACCTGGCACTCACAGCTTACTGCTTGAACAGT
TGTCAGGTGTTAAATTAGGGTACGTGGAATGTATCGGCGGTTTTCATGGTTTTACAGTTAAATGCCAGGGTTTGCAGGGTGGAATTGCACACTGCTACGGTCAGTATGGTGATGCATTTATTT
TCAAATCTGATTCTGGCGGGGCGTGTGCCGATAACTACATGGAAAGAATCGCAGTTGGTCTTTATGACAACTCCGGATGGCCTGATGTCACTATGGGCGGGATTTATGATGCTCATGATAACG
TAACAATCGACAGAATTGGTATTGGTGAGTTAATTGTACAGAATGCATCATGGGGATTGATACCGTCTGATGCTAATACCGGATTCATAACAAACGTCAGCATTGGTAGGTACTCTGCATTTAAT
GTCTATGGGAACTATTATTCATTAACCATTGATAATAAATGTGTTGGTTGGACTATCGGTGAGCATAGGATTAGTAATGCTTCTGGCGGTATTCGTGTTCATCCTGATTCAGTTGAGATAAATATA
GGAACAGGATCGTCAAAAGGTAACACAAAAAGTGGTTATGCACTCGGTGGGAATAGCTTGACCCATGGAAAGCTATTTGCAAATGAGAATGGGGAGGCAGGAGTTGATTATCTTGGTGGC
CTGGGTCTTGATGCTTCTCTCATTAATGGATACATCAACGGTACTGTTCTTATTTCAGGTTATCCAGGTGTAAAAGACGGAAATCCATTAAATGGATGGGCAGATACCGGAGCTTTCGACATGA
TTTTAACTGGCAAGACAGTGCAGGTCACAGGGTCGCTGACGCGCGGAACAGCAGCAGTTGCATATAACACTATTTCTGCATGCAGACCTATAAAACGAGTTCCCATTCCTGCGTGGGGGGT
TAGTGCATCAAGCACAATGATTCCTGTCGAATGTTATATCGAAACAAACGGACAGTTAAACGTAGCTGGGTTCGCATCAATACCAGTCGGTGGTACTGTGAATTTTAACGGAAATTATTTAACT
AAATAG

Prophage 4.2

>OCONKI_07660 hypothetical protein
ATGAGCACCTTCACTAAAGAGCAGTTAATAGAATACCTGAAGGAATCGACGCAGAACGCTAGTGGTATGTTTGAAATCAGCGAGGACACCATCTGCGCGTTGATGTCCATGCTCGCCTCCCC
GCCAGCGCCGGTATCTGTGCCCGCTGCGATGGAAATTGATGATGACTTTGACAGCGCGTTTGAACACGGAAAAGCTGTCGGCTGGAACGCCTATCGCGCCGCCATGCTTCAGGCCGAACCT
GTAAGTAATAGTGATGAGTTACCGCTGGACTATCTGCAAGGACACAAAGACGGCCTGGAGTGGGCTGCACAATTGGCAGAAGCCAATCATCCGCAAACAGGTGACTGGTTGTACGACGAC
CCAATCGATCTTGCCAGGGCGATTCGCAAAGGTCCGGATATGCCTACTGTTCAGGGTGGCAACTCTCCGGTAACTCCGGATGGTTGGATAAGCTGTAGTGAGCGAATGCCGGATAAGTTAAT
TCCGGTAATGGTCATGTATGAAGACGGTGAGATGTGGTCTGCAATGTGGAATGGCAATCGCTGGGATGATGGCACCGAATATCCGAATCCGCACTCAGTTACGCACTGGCGTGAAATGCCAG
CAGCACCGAAGCAGGAGGTTAACCGTGGCTAA
>OCONKI_07665 SlyX protein
ATGAAATACGAAATCCCGGAATCAGAAGATATTGAATGGCAGCAGGCTATGCTCCGTGAAATAGACAGCGCCCTTGACGTCTTGCGTGATGAGCATGAGCACGCAGTGGTGGTAGAAGAAA
TCATCAATGATATCACCGCGAGAATAGCATCACTCCGCGCGTACTCTGGATACTGA
>OCONKI_07670 dATP-dGTP-PPHyd domain-containing protein
GTGAGCCAGATTGATTATCAGGCACTGCGTGCTAAGGCAGAAAAAGCAACGTGTGGCGAGTGGTCGCTCGAATATGGAGAGGGCCGATTTGATGGTGATGATGCGCTAATTCATCGTGAA
GTTGCTGGATATATTCCCATTTGCAGAATTGAAGGAGCGCATCCAGAAAGCGGTTTCGATGAAGATTTCCAAATGGAACAGCAGGCCAATGCTGAATTCATCGCCGCAGCCAATCCGGCTAC
CGTACTGGCGCTGCTGGATGAGCTGGAATCCAAAGACAGGCGCAACGCTGAGCTGGAGGCGAAACTCCAAGCCACTGACAAATTGCATGATAGCGCGTTCCGTCATGGTCTCCAGCATGG
TTTTAGTTACGGTCAGACAGATGATCAAGCAGGATTTGAGAAAGCCATCCAAGCGTATGGGCAGCAGTGA
>OCONKI_07675 WWE domain-containing protein
GTGAGTAATTCAGCACGACTACAGCTTGGTTTTTCACCGCTATCAAAAACTATCATGCTGGCAAAAATGCGCGATGTTGAAGGTGGACGTATGCGCGTTGGCAATGATCCAGGTCGTGATGT
TACCAATGAGGCTGCTCAATTGGTGTGGCGACTGGTCATGGCTGAAGGTGGTGAGATCGCGTGGGAGCTGGATGATGGTTCTCGCATGGTGTTGAAGGCAGAAAAGCAGGAGGCAACCA
GTGAGCCAGATTGA
>OCONKI_07680 Uncharacterized protein in eae-abc2 intergenic region
ATGAGAGGACTTGCATACAATCCCGGCATTCTTCCGGCAGAAATGATTATTCGCCAACGCGTAAAGCCAATGCCATCGAGAGAGGAATTGCTTAAGAGAAATTCTTTTCCGTCAGTGAATCA
AAACAAATATCTGAATGCGATGTGGCGTAAAGGAGGCAACCAGTGA
>OCONKI_07685 Anti-RecBCD protein 2
ATGCCAGCGCCTATGTATGGTGCGAATGACCCGCGCCGCTGTTCCGGCAATTCCGTCTCGGAGGTGCTGGATAAATTCAGAAAAAACTACGACCTGATAATGTCACTACCGCAGGAAACGAA
AGAGGAAAAGGAATTTCGCCACTGTATATGGCTTGCAGAGAAAGAAGAACGCGAGCGAATTTACCAGACATCAATCCGACCATTCCGCAAAGCCACATATACCCACTTCCCTGAATATATCG
ACCCGCGCCTGCGTAATTATCGATCACGCTATGGCGCTATCAGTAATGACTGA
>OCONKI_07690 3'-5' exoribonuclease
ATGAATCATTTAATGGTTGACCTTGAAACAATGGGCAACGGGCCATACGCGCCCGTTATTTCGATTGGGGCAGTATTCTTTGATCTGAAAACTGGAGAAACAGGAGAAGACTTCTCGGTTAA
TATCTCGCTCGAGTCATCAATGCGATACCGGGCGCGTCCTGATGCTTCAACCATTTTATGGTGGATGGAACAGGGAGAAGATGCCAGAAAATCGCTAACCAATGACACTCAAGAGCTTTCAA
CGGCTCTTTCATGGTTATCAGACTTTATCGCAAAGCACGCCAACCCTAAATTCGTTCAGGTTTGGGGAAATGGCGCATCATTTGACTGTGTGATTCTACGAAATAGTTATGCTCTGGCCGGGC
ACCAAGCGCCCTGGCAGTGGTGGAATGACCGCGACGTCCGAACCATCGTCGAGCTTGGAAAGGCAATTGGGTTCGACCCTAAACGAGATATGCCATTCGAAGGAACTCGACACAACGCGC
TTGATGATGCCATTCACCAAGCCAAATACGTTTCAGCAATCTGGAAAAAGTTAGCTAAATAA
>OCONKI_07695 Single-stranded DNA-binding protein
ATGGCTAGTAGAGGCGTAAATAAGGTGATCATCTTAGGCCGGGTCGGACAAGACCCGGAGGTTCGTTATTCACCATCAGGGACGGCGTTCGCTAACCTGACAGTCGCTACATCAGAGCAGT
GGCGAGATAAACAGACTGGCGAACAAAAGGAGCAGACTGAATGGCATCGTGTTGCCGTAGTCGGGAAACTTGCTGAAGTCGTAGGGCAGTATGTGAAAAAAGGTGATCAGGTTTATTTC
GAGGGAATGCTGAGAACCAGAAAGTGGCAAGACCAGACAGGGCAAGACCGCTACACCACTGAGATTAATGTTGGAATTAATGGTGTGATGCAAATGCTTGGAGGCACTGGCGACAGCAA
ACAACAAGCAGCCGACAGGCAGTCACAGAAACCACAGCAGCAACCATCACCAACACAACATAACGAGCCACCGATGGATTTTGATGATGATATACCCTTTGCACCAGTAACTCTCCCCTTCC
CTCGTCACGCTATTCACGCAATTTAA
>OCONKI_07700 Recombinase
ATGGATTTGAATAAATTCGACGACCCATTCAGTCCTGAAGATATCGAATGGCGAATACAGCAAAGCGGTAAAACACGCGATGGCAAAGTGTGGGCTATGGTGCTGGCTTATGTCACGAACA
GGGCAATCATGAAACGCCTTGACGATGTTTGCGGCAAAGCAGGATGGCGCAATGAATACCGCGATATTCCCAACAACGGCGGCGTTGAATGCGGAATATCAATCAAGATTGATTCCGAATG
GGTCACCAAATGGGATGCTGCTGAAAACACGCAGGTAGAAGCCGTCAAAGGTGGTCGTTCCGGTGCAATGAAGCGCGCTGCCGTTCAGTGGGGAATCGGTCGGTATCTGTATAACCTTGA
GGAAGGTTTCGCACAAACATCACTCGATAAAAAGCAGGGATGGCACAGGGCAAAACTGAAGGATGGAACAGGATTTTACTGGCTCCCTCCATCGCTGCCGGACTGGGCCATGCCAGCATC
AGGCAATCAACCATCACCAGAAAATACCAACCAGAAATCACCATCGTTTGACTGCGAACAAATCCTGAAAGACTTCAGAGATTATGCAGCAACAGAAACTGACAAGAAAAAGCTAATTGAG
AGATATCAGCATGACTGGCAATTATTGGCTGGTCACGATGATGCGCAGACAAAATGCGTTCAGGTAATGAACATCAGAGTTAACGAACTAAAACAGGCGGCATAA
>OCONKI_07705 Host cell division inhibitory peptide Kil
ATGGATAAATCACTTATGGCTATTCAGTCTAAATTCGCAATTGCTGTTTATCTTGGTGACAAAATAATGTATCGCGAAGCTGTAGAAGCCTTTCGCGAATGGAGGTTGAAATGA
>OCONKI_07710 hypothetical protein
ATGACATTTGCTATCGCGGGCGGTGCCGTCATGGGTATCGCACACCTTAATGAATCACTTTTAGAGCGTATCACCAGAAAATTACGGGCCGGATGGAAACGTCTGGTCGATATCCTGAATCAA
CCAGGAGTGCCGTGTAATGGATAA
>OCONKI_07715 hypothetical protein
ATGCCCTGGCTTTTCCTTCTATTCCGAGTGATGCCGCTACGGATTGCGATCCAGACACTTCCGCTGCTGACTGGTAGCCAGTGTTGGTTGCTGCTGACCTGTCGCCAGTGTTGGTTGCTGCTG
ACCAGTCGCCAGTGTTGGTTGCTGCTGAACAGTCGCCAGTGTTGGTTGCTGCTGACCAGTCGCCAGTGTTGGTTGCTGCTGACCGGTCGCCAGTGTTGGTTGCTGCTGACCAGTCGCCAGT
GTTGGTTGCTGCTGAACAGTAGCCAGTGTTGGTTGCTGCTGACCAGTCGCCAGTGTTGGTTGCTGCTGACCAGTCGCCAGTGTTGGTTGCTGCTGAACAGTCGCCAGTGTTGGTTGCTGCT
GAACAGTCGCCAGTGTTGGTTGCTGCTGATCGGTTACCAGTGTTGGTTGCTGCTGAACAGTCGCCAGTGTTGGTTGCTGCTGAACAGTCGCCAGTGTTGGTTGCTGCTGACCGGTCGCCAG
TGTTGGTTGCTGCTGACCAGTCGCCAGTGTTGGTTGCTGCTGAACAGTAGCCAGTGTTGGTTGCTGCTGACCAGTCGCCAGTGTTGGTTGCTGCTGACCAGTCGCCAGTGTTGGTTGCTGC
TGAACAGTCGCCAGTGTTGGTTGCTGCTGAACAGTCGCCAGTGTTGGTTGCTGCTGATCGGTTACCAGTGTTGGTTGCTGCTGAACAGTCGCCAGTGTTGGTTGCTGCTGATCGGTTACCA
GTGTTAG
>OCONKI_07720 Antitermination protein
ATGACGCGCAGAACAGCTTTCAATGGTTCAGCAGCAGGTCGTCGTCGTGAGCGCCGTGCAGCGCTTCAGAATGCGGTAACGGCAAGCTCAGAAGTATTACACCGCCCCACCCTTAGCCGC
GCCCAGATTCAGGCCAAAGGAAAACATGAAACGCCAAAACGTATTGAAGACGCAAAATCACTTCAGTTCATGGCGAAAGATGCATTCTGGCAACTGGAAGAATACAGACGCCATCTGGAG
CGGGCAGCCATTGTGTACGCAAATGAGTTTGGTCATAAGCCACCAGAAACCGGTGTATGTTTGCCAGACGTAGCGCTTTACGCAGCAGGTCATCGTAAATGTAGACAAGTTACCGCTAGATA
A
>OCONKI_07725 Fumarase D
ATGAAAAGCGAAGAGCTGGCGCAGTTGCGCTATCAGGAAATGTGCAGGATTGTGGGTGATGTCGTTTTTGCTATGGTTGCTGAGGGTCATGAGACCAAAAGAGTCGCTATAGCTGACGTG
ATAAGAACGGAGCTATCAAAGGGGCTGGATAAGTGGGATGTTGACCAGATTCAGGTTATGGAACTAGCGGTGAAGCTACTGGAAGAGTAG
>OCONKI_07730 DUF1828 domain-containing protein

ATGATGTGCTCAACGATAATTTCCAGACTCGGCTATGAATGCCTACCCATAGGTGAAGAGTCCTTGAGAATAATCAGTCCGTTTCCCTATTGTGACGATGGGGAGCATGTTGGCGCATTCGTAC
AGCATATTAATGGGAGTTTCAAAGTAACTGATCGATGCGACGCACTTATGAACATGGAGGCTCGTGGAATTTCACTTAACCAAAGTCGCCTTGATGTGATCAGGCAGGCACTCGCGCGAGAA
GGCGCTGAGCTAAATGAAAGAGGCGAAATTCTTAAGTGGGCTCATGATGAAGGTGAGCTTGGAAAGGTAACATCAGATGTTATTAGGGCTGGTATTCTTGCTTCAGCTATGTCCATCGATTG
GTATTCATCTAACCAATCTAAAAGATTTGAAGCTGAAGTGATTGATTTCATCTCAAAAAGTTCACTATCAAATTTAATGTCCCTTCGAGAGGAAGTCAGTGGCATGAGTGGTCACAATATTGTC
ATACCTGTTACCATTAAAACCGCAATGCCCAAATACATATTTACCTCAAGCATTAAAGAAGGTGGATCATGGAACAGCGCATATTCATTGCTTGGAAAGCTGATGGATCTTTATCAGGCCAACA
ACGCTGTTAACAATCGATACGTAGTTATTGACAATGAATCTATCGGCTCGCAAATGCAGCAACTGATACTGCTGTTCAACGACGTAAGCCAAGTTCTTCCATTCGAAAGCAGAAGTTTATGGC
TTCCAAAATTAGCTGCATAA
>OCONKI_07735 hypothetical protein
GTGGCAAAGAAAAACGAGAGTCCTCTGTTACAGTCGGATGTTGATCGCGTCATAAACTCCAAGAAAATAGTGGATGTTAGCAGCATAAAATGGGGTAAGAAGCCGCCACCTGGCAGGTCA
CCGATGTGGTTACAAACGGCTATCACACCTTATGAGGATGGCTCACCTTTGCCTGGCCTAAAGTTTGTTTTACAGTGGCGACCAGCTGATGAATACGGAGATTCCCCTAAAATTCAGATGGTG
GCGCTTTACTTTGGTCGAAGAATTTTTGGTGTGGACTCTTACCCAAATGATAGACATACTAACAGAGTCCGGGTATGCCATCCTGACTATGCTGAAAGCATTCTTGGCCCACATTATCACCTTT
ACTTTGAATCAGCGTTGCCGTATGAAATAGGTCTGATCATACGCGAGAAGATAGCCCCGGATGACTTGTTAGGACACTGGCGCTTTTTCTGCTATAAACTGAATGTTACATGCAAAGGAATAC
TGCCTTTGCCGACCCAAGAAGATTCGGGACAAATTCCATTGCTATGA
>OCONKI_07740 Repressor
ATGAACTGGTATGACATAGCGAAGCAAAGGATTGATCAGCTTGGATTGAGTCAGGATAAAGTTGCTGAACACCTTGGTGTAACCAAAGGTGCTGTTAGTCATTGGCTTAACGGGAGAAGGA
ACCCATCAATACAAGAAATTGGAGCTATATTTCAATATCTTGGCGTTACAGACGCGAGGTTCAACTCTGACGGAACCTTTAGCGTTGGCGAGTCAACAGAACAAAAGCCTGTTAAACCTCAA
TTTGAATACCCATTCTTCTCTCATGTTCAGGCTGGAATGTTTACACCAGAATTTCGAACCTTCACTCAACTAGATGCTGAGGGATGGGTAAGTACAACCAAAAAGGCCAGTGAGGCAGCTTTC
TGGCTTGAAGTTGAAGGCCACTCAATGACGGCTCCAGCGGGATCACGACCAAGCTTTCCTGAAGGAATGCTGATTCTTGTAGACCCAGAAGATCCTGTAGACCCAGGCGATTTTTGCATTG
CAAGGTTATGTGGTGATGAGTTCACTTTTAAGAAGCTCATCAAAGACAGCGGACAAGTATTCCTACAACCGCTAAACCCTCAGTTCCCAATAATGCCGTGTAACGAACAATGCAGGGTTGTA
GGTAAGGTTGTAGCCAGCCAGTGGCCTGATGAGATATTTGGGTGA
>OCONKI_07745 HTH cro/C1-type domain-containing protein
ATGAATAATATTCGCAATTTTCGCGAGCGCTTCGGTTTAACGCAGGAAGATCTTGCGAAAGTACTCGGTTGTACGCGTGGTGCAGTTTGTCATTACGAGACAGGCAGAAGGGGAATGGATAT
CAATCTTTGTCGCGCTTTTATCAATGCGTTCAAAGAATACGGTTATGAACTAACCATAGACGATCTTTTTCCACCAAAGGCCGCGTAA
>OCONKI_07750 CII
ATGAACGTAGTTGCAACTAAAAGCAAGAAGGCGGCTCGCATTGAGTCCACTTTACTCAACAAGTTAGCCATGATGGGCCAGAAGACATTCGCTAAAGCTATGGGTGTTCCTGAATACCAGG
TAAGCCGATGGAAGAACGGTTTCTTCTCTCAGGTCAGCATGATGCTTGCGGTTCTGGAGTATGGAATCGAAGACGAGGAAATGGCAGAGCTCACCAGGCGACTTGCTACCTACCTGACAAA
AGAAAAAGCCCCGAAGAACGGCGAATTCTTCGATGCCTGA
>OCONKI_07755 hypothetical protein
ATGCCAAAACAACTCAGTCCTGACCAGGACAAATTACACAAAAACATACTACGTGATCGGTTCTTATCCAGCTTCAAACAGCCTGGTCGATTTCGGGCTGAGTTGGAGAAAGTGAAGCTAAT
ACTGAAGAGGAAAGGTCATGAGTAA
>OCONKI_07760 DNA replication protein
ATGAGTAACATATCCAATCTAGCCGAAGCCAGAGAGGCCAGAAGGATCCAGAAGCCGCGTACAAATGGCGGCAAGGGGTTTGCCTTGATTCACCGCCAATTCATGGATAGCAAGCTATACA
AGGATTCTCAGGCTGTACATCTTTTCCTGCATCTGATACTGAAAGCCAATCACTCTCCGGCAGTCGTAAATACCGACATTGGTGAGATGTTGGTTGAGCGAGGACAGCTAATTACCGGACGG
CCAAAACTGGTAAGTGAAACATTCATCCCGGATAACAAAGTGAAAAGTTTGCTTCGTTCTTTTGAAGGGAATGGAATGATTTGTATCGAGTCGAAAGGGAGAAAATTCAGCCTGATAACAG
TGTTGAAATATGATGATTTTCAGGCTCCAAATTGTCCAACGGATGTCCAACGGATGTCCAACGCAAACACCAGTAATGACGCGGCTCACAGCAAATGTTATCCAACGGATGTCCAACGATTGT
CCATAAACAATAATATAAATAATATCTCTAATACTAACGTATTAGAGAGTACCGCAGCAGACGAAAATCCTGACAAGAAAAAATCGGCTCTCAGTTGTCAGGATGTTGTCGATGCTTACCACGA
ATTACTTCCTGAAGCTTCCAGGGTTCGCGCACTGAATGACAAACGTAAAAACCAGATCCGAACTTTCTGGCGAAAAGCCGGAGTGATAACACGCCAACTTGACGGGCATGGGTTCACGATG
CAGGACTGGAGAAATTATCTGAGCTACGTAGGCGAAAATTGCCGATGGATGTTCGAAGAGCGCCAAAACCATCAACGCGGAACCGTCTGGCACAAAAAGGGATTTGATTTCCTGCTTAAC
GATAATACCTACCTGAAAGTTCGTGAGGGTGAACACGATGACCGATAA
>OCONKI_07765 DNA helicase
ATGACCGATAATTTTTATGCGCCGCCCCATAGCATCGAGGCAGAGCAGGCTGTGATTGGCGGATTGCTTCTGGATGATGACAGCAGTGAGCGCGTCCAGAAAGTTCTGGCGATGCTGAAGC
CTGATTCATTTTACAGCCGACCACACAAAATCATTTTCGAAGAAATAACCAGAATGCACCGGGAGCAAAAGCCAGTAGATGGCCTGACGCTTTTCGATGAACTGGAGCGCAAATCGTTAAC
GGCGTCTGTTGGCGGTTTTGCTTATATCGCTGAGATCGCAAAGAACACGCCAAGCGCAGCAAACATCGTTGCCTATGCAATGCAGGTTCGTGAAACCGCAATGGAACGCTACGCCATCAAC
CGCATGACTGAAGCGACGGAATTGCTCTATTCCCGCAACGGAATGACTGCAACGCAGAAGTACGAAGCTATTCAGGCGATTTTCACGCAACTGACAGACCATGCAAAAACCGGATCGCGTC
GAGGCCTTCGCTCATTTGGTGAGGTCATGGAAGACTGGGTTAGCGACCTTGAGAAGCGATTTGACTCATCAGGCGAACAACGGGGAATGAGCACAGGGATCCCATCGCTGGACAGGATG
CTGTCACCGAAAGGTCTGGTGAAAGGCTCTCTGTTTGTCATTGGCGCTCGCCCTAAGATGGGGAAAACGACGCTATACAGCCAGATGGCAATCAACTGCGCAGTGCATGAGAAAAAGGCT
GCCCTGATGTTCAGCCTTGAAATGCCAGGTGACCAGATACTGGAAAAACTGGTAGGGCAGAAGTCAGGTGTTAACCCGAATATTTTTTACCTTCCGGCGACAAATGACGCTGATGACGGCT
ATCAGGGTGATTACGATGGTGACTTCAACAGGGCGATCGAAACAGCCAATCGCTTGAGTGAAATCGACCTGCTTTACATCGACGACACGCCGGGATTATCTCTGGCTCAAATCGTCAGCGAA
AGCCGTCGAATCAAGCGAGAAAAGGGGGGTGTTGGCATGATTCTGGTCGATTACCTGACACTAATGACCGCTGAAAAGGCCGATCGTAACGACCTTGCTTACGGCATGATTACTAAGGGGC
TGAAGAACCTTGCCAAAGAGCTTGATTGCGTTGTTGTGCTTCTGACGCAGCTTAACCGCGCACTGGAAAGCCGAACTAATAAACGCCCTTTACCAAGCGACTCCCGAGATACAGGGCAGAT
TGAACAGGATTGCGATTATTGGGTTGGTATCCATCGTGAAGGTGCTTTTGATGACAGCGTTCCGCCTGGTGAAACCGAACTAATCCTTCGTCTAAATCGCCATGGAAATACCGGCACGGTGTA
TTGCATTCAGGCAAATGGCGCTATTTATGACACAGACCAACAGTCTGCTGAAATGCGCCGCCGTGAACGCGAGGAACCGCAGTCCAAGAAGAAAGGAGGATTCTGA
>OCONKI_07770 Prophage protein
GTGGCTGACTGGCAAATTCCAATCGTCATTCTTGCCGGAGCTTCGCTGGTTGCTGGCTTTATCCTGCTGAAGAAGCATAAAGACCGTGATCAAAAAGTCGAAGTTCTCTATGGGTATCCAGC
GAACAGCACAACATGGCTGACCATTTACCACTACCGAAAATCAGGCCGCTGGGTATTCGAATGGGATGATCTGTTCGCTGAAAAGCGACCAAAGTCATGGGGAGACATCAGCGAATGCATG
ATGTTTGAAGAAAGAAAATCCGGCGCAACCCGAGAAGAGTTTAACGAAGCGTGGGCGCGATTAAGTGAGAGAGGGTATTTGTGA
>OCONKI_07775 hypothetical protein
GTGAGCAAGTACGAAAAATTAGATCAAAACATTCTTTCAATGCTGAGTGAAAGACCAACACCTGTTTTTGATATCTGGCTTAAATGGCGGAGCAATGGAATGTATATCGAAACCATCGATCGC
CGTATGCAATACCTGAGAAAGAAAGGGCTTGTTGCAAATGTGCGTGGGAAGGGGTGGGTGAAAATTAACCTGTCATAA
>OCONKI_07780 Phage protein
ATGGACGAATCAAGAAAGCAGTTTTTGGAGTGGTTTGGTGAAGAGTTCGAGTCTATTAACAACAGCGAAGAACTTCACGTTCAGGCCATCAAGATGATTGCCTGGCAGTCATGGGTTAAGT
CTCGCGCAGCTATCGAGATAAAACTCGATGACAAAGTGATGGCTGAGGATGATTTCGACAAAGGACACAACTGCGCTATCGATTATTGCGCTGACGCCATCCGCGCCGCTGGAATCAAAGT
GAAGGAGTGA
>OCONKI_07785 hypothetical protein
ATGTGTGTATATCACCTGAAACAATGCTACGGATGCGGAATGCCTCTTCGGTTTAATGGATTCCAGAGTATTCCTGATGTGCCTATGTGTAGTTCTTGTCGGGATAAAGGAATTAAGCCGAGAT
ACGTTTATGTTTATTCCGCCAACAAGATATTGCCGAAGTATGAATACAGCACGGAAATAATGAGGTCAAAAACAAATATCTAA
>OCONKI_07790 Protein ninB
ATGAAAAAACTAACCTTTGAAATTCGATCTCCAGCGCATCAGCAAAACGCCATTCACGCAGTACAGCAAATCCTTCCAGACCCAACCAAACCAATCGTAGTAACCATTCAGGATCGCAACCG
CAGCTTAGACCAAAATCGGAAGCTTTGGGCTTGCCTTGGGGATGTCTCGCGTCAGGTCGAATGGCATGGTCGCCGGCTGGATGCAGAAAGCTGGAAGTGCGTGTTTACCGCGGCATTAAA

GCAGCAGGACGTTGTTCCTAACCTTGCCGGTAATGGCTTTGTGGTAATAGGCCAGTCAACCAGCAGGATGCGTGTAAGCGAGTTTGCGGAGCTATTAGAGCTTATACAGGCATTCGGTACA
GAGCGCGGCGTTAAGTGGTCAGACGAAGCCCGGTTAGCACTGGAATGGAAAGCGAGGTTTGGAGACGCCGCATGA
>OCONKI_07795 hypothetical protein
ATGATTGACCCCAATCGAAGTTATGAGAAAGAGAGCATAGCAAGGGCAATGTGCGCAGGATGTAACAAGCAACTGGCACCTGATGAAATTTACGCCTGTGCAGAATGTGTTAACGAATGGC
TGGTATATCGCGATCCGAATGGAGATATGTCGAATGAGGAAGGCAAGGCGGCGTTGTAA
>OCONKI_07800 Protein ninG
ATGAGGAAGGCAAGGCGGCGTTGTAAGAACGAAGAGTGCAGGGAATGGTTCTTCCCGCAATTTCAGAACCAACAGTGGTGTTGTGTTGATTGTGGTACGAAGTTAGCACTCGAACGACG
AAGCAAAGAGCGCGAAAAAGCAGAAAAAGCAGCAGAGAAGAAACGACGACGAGAGGAGCAAAAACAGAAAGATAAACTGAAGATTCGAAAACTCGCCTTAAAGCCCCGCAGTTACTG
GATTAAACAAGCCCAACAAGCCGTAAACGCCTTCATCAGAGAAAGAGACCGCGACTTACCATGTATCTCGTGCGGAACGCTCACGTCTGCTCAGTGGGATGCCGGGCATTACCGGACAACC
GCTGCGGCACCTCAACTCCGATTTGATGAACGCAATATTCACAAGCAATGCGTGGTGTGCAACCAGCACAAAAGCGGAAATCTCGTTCCGTATCGCGTCGAACTGATTAACCGCATCGGGCA
GGAAGCAGTAGACGAAATCGAATCAAACCATAACCGCCATCGCTGGACTGTCGAAGAGTGCAGGACCATCAAGGCGGAGTATCAACAGAAACTTAAAAAACTGCGAAACAGCAGAAGTG
AGGCTGCATGA
>OCONKI_07805 Serine/threonine-protein phosphatase
ATGAATATCTACGAAAGAATTGATGGCAGCAAATACCGAAATATTTGGGTAGCTGGCGACCTGCACGGATGCTACACGAACCTTATGAACAAACTGGATACGATTGGATTCGACAACAAAAA
AGACCTGCTTATCTCGGTGGGCGATTTGGTTGATCGTGGTGCAGAGAACGTTGAATGCCTGGAATTAATCACATTCCCCTGGTTCAGAGCTGTACGTGGAAACCATGAGCACATGATGATTG
ATGGCTTATCAGAGCGTGGAAACGTCAATCACTGGCTGCTTAATGGCGGTGTCTGGTTCTTTAATCTCGATTACGACAAAGAAATTCTGGCTAAAGCTCTTGCCCATAAAGCAGAAGAACTTC
CGTTAATCATCGAACTGGTGAGCAAAGGTAAAAAATATGTCATCTGCCACGCCGATTATCCTTGTGACGAATACGAATTTGGAAAGCCAGTTGATCATCAGCAGGTAATCTGGAACCGCGAA
CGAATCAGCAACTCACAAGACGGGATCGTGAAAGAAATTAAAGGCGCGGACACGTTTATCTTTGGTCATACGCCAGCAGTGAAACCACTTAAATTTGCCAACCAAATGTATATCGATACCGG
CGCAGTGTTCTGCGGAAACCTCACATTGATTCAGGTACAGGGAGAAGGCGCGTGGGCATAA
>OCONKI_07810 Antiterminator
GTGGGCATAAGAGAACTAAACCTCACCAAAGAACAGCATGAGTGGCTGAATGGCTGGCTTGAACTGTGGGGCGCATGGGTTTATTCAGGTCGTCTGGAAAAGCGCATGAGCAGCGTAATA
GCGAAGTTCATGGAGAGCGTAGAGCCGGGAAGAGTTATGACAAGGCCAATGTGCAATGATGATGATGGAATGTTGATTTCTCAGGTCGTCGATTCCGTCATGTGCATTGACAAGAAAGCCT
TTGGCATCCTCCTCAGCTACTACGCTCATGGTTCATCTAAGCGAGCAATTGCATCCTACTATCACGCGACTGCAAAGCCACGCAAGATGTGTGGACGTGGTGGCGAGGGATGGAGAAAACCT
TCACTGGCAACCTGTAGAAACGAAATTGACGACATCCTGAAAGCATCGTTATTTGTTTTGTACCAGCCAATGCAAAATGCTTTCAAAATGCGTAAACGTGTTGAGAAAGTTAAGCATGTTGCT
GTTAAAAGCCTTGACATGCAATTATCCATTTAG
>OCONKI_07815 tRNA-Asn(gtt)
GGGTCAGTCGTATAAAGGTGATTACGGAAGGCTGTTAACCTTCTTATCGTGGTTCGAGTCCACGCTGTCCCGCCA
>OCONKI_07820 tRNA-Thr(tgt)
GCTGGTTTAGCTCTAATGGTAGAGCAGTCGCCTTGTAAGCGAATGGGTAGCGGTTCAAGTCCGTTAACCAGCACCA
>OCONKI_07825 Holin
ATGTACCGTATGGACAAAATCAGAGAATGGTTCAGTTACAGCTTCGGAGGACTGACTGCGATGGGTGGCATTCTCTCCCTGAATGACTGGGCTGTCATCATTGGTATTCTTTGTACTGTCGGC
ACATTTGGCATCAACTGGTACTACAAGCGCAAAGAGCGCGAGGACAGATTGAATGGCAATGTCACCGGCACTACGAAATAG
>OCONKI_07830 lysozyme RrrD
ATGGCAATGTCACCGGCACTACGAAATAGCGTAATAGCGGCGATAAGTGGCGGGGCTATTGCTATAGCATCTGTGTTAATCACTGGACCGAGTGGTAACGATGGTCTGGAAGGTGTCAGCTA
CATACCATACAAAGATATTGTTGGTGTATGGACTGTATGCCACGGACACACCGGAAAAGACATCATGCTCGGTAAAACGTATACCGAAGCAGAATGCAAAGCCCTCCTGAATAAAGACCTTG
CCACGGTCGCCAGACAAATTAACCCGTACATCAAAGTCGATATACCGGAAACAACGCGCGGCGCTCTTTACTCGTTCGTCTATAACGTGGGCACAGGCAATTTCAGAACATCGACGCTTCTTC
GCAAAATAAACCAGGGCGATATCAAGGGAGCATGTGATCAGCTACGGCGCTGGACATACGCTGGCGGTAAGCAATGGAAAGGGCTGATGACCCGTCGTGAGATTGAGCGTGAAGTCTGTT
TGTGGGGGCAACAATGA
>OCONKI_07835 hypothetical protein
ATGAGCATGATTTGCTTTTTCATGGCAGCGTTGCTCGCATTGAATGGCAACGATGCGTGGCCGTGGTTTCTGGCCGTTGGGGTGTTGATGTCATGA
>OCONKI_07840 Outer membrane lipoprotein Rz1
ATGCTAACGCGACTATCGAAAGTCTCCGTGCTGATGTTTCTGCTGGTCGTAAGCGCCTGCAAGTCGCCGCCACCTGTGCAAAGTCAACGACCGGAGCCAGCGGCATGGGCGATGGAGAAA
GCCCAAGACTTACAGCAGATGCTGAACTCAATTATTACCGTCTCCGAAGTGGAATCGACAGGATAA
>OCONKI_07845 DUF2829 domain-containing protein
ATGACCCAGCATATTGGCGTAAAACTGATTAACGCCTTTCCGATGACGAGACAGGCATATAACGATTTTCGTGGCTGGCAGCTTCCAGCCGGAGAAAACGGCGAGGACGAAGGCTATCTGG
TTGAATATCTGGATGGCGGAAAACCTAACACCGATCGCTTTGATGGCTACGTTATCTGGAGTCCAAAAGAAGTATTCGAAAAGGCTTATCGTCCGGTATCAGGGCTAAGTTTCGGCCTTGCC
ATTGAAGCGCTCAAGTTGGGGAATAAAGTTGCCCGTGCTGGCTGGAATGGTAAGGGTATGTGGCTGGCATACGTTAAACCGTACACTGAGGCTGTTCATACTGGCAGTACGCCTTGCTTTTG
CAGTCGCGTCTTTGAGTTGCCTGAAGGTACGCATGGAGAACCGAAACGATCTCCGAAACAACTTCCGTATATCGCCATGAAAACAGCGGACGATAAATTAGTGCCGTGGCTGGCTAGTCAG
ACTGATGTTCTAGCAGAAGACTGGCAAATCATTACCATGTAG
>OCONKI_07850 Uncharacterized protein in gp15-gp3 intergenic region
ATGGCAGAGATTATTTCCATGACTGAAGAACAGAAATTCCAGTTAGAGATTTACAAACTGGTCATGAACCAGAACGCAGCCGCAGAGGAAGCATTTCAATTCATTGGCACTGACGAGCTGA
AGCTTGAACTATTCAAAATTCACTTCCAGTCAGGCGGCGCTAATTCGGATATCACGATCCGCACATTCGAAGCAGTGCGTAAATCTAAGGAAGCGTTAGACCTGTTCACCACCGGAGCATAA
>OCONKI_07855 Decoration protein
ATGGCAAATCCAAATTTCACGCCATCCTGGCCTCTCTACAAAGATGCTGACGGTGTATATGTGTCTGCTCTTCCGATTAAAGCTATCAAATACGCTAATGACGGAAGTGCAAACGCAGAATTCG
ACGGTCCGTATGCTGACCAGTACATGTCAGCGCAAACAGTAGCCGTATTCAAGCCGGAGGTCGGTGGATATCTGTTCCGAAGCCAGTACGGAGAGCTGCTCTATATGAGCAAGGCCGCATTT
GAAGCTAAGTACACTTCTGCAAGCGGTTCAGTAACGAATGCAGAGACGGCGGATAAGTTATCTACTGCTCGCACTATCTCACTAACTGGCGCGGTCACAGGTTCGACGTCATTTGATGGTTC
GGCTAACGTGACTATCGCAACTACCCAAGGAAGCTAA
>OCONKI_07860 Terminase
ATGGCGGCTGAAGATAAGAAAATTGGTCGCCCATCGGCTTACAAACCAGAGTATGCCGAGCAGGCGAGAAAACTTTGTCTGTTAGGGCATACAGATGCGGAACTGGCTTCTTTCTTTGATG
TTAGCGAGCAAACAATCAATGCGTGGAAGCACGCGCATCCTGATTTTCTTGAGTCCATAAAAAAGGGTAAGGCTGTTGCGGACAGTGAAGTCGCCGCAAAGTTATTCCACCGCGCCACCGG
GTACGAACACCCAGAAGATGATATTCGCGCTGTCGATGGTTCGATCGTCATAACTCCTACTGTGAAACATTATCCACCCGATACGACTGCTGCCATTTTCTGGCTTAAGAACAGGCAGCGAGA
TAAATGGCGTGACAAACAGGAAGTAGAACACACCGGAGAGGTTAGCCTGATTCAGCGCATTCAGGAGGCCCGTAAACGCGCAAGGGGTGAGTGA
>OCONKI_07865 Terminase
ATGTCATCAGAATTTGAGGCAATGCTTGCCGACGATATGGGGCGATTCTTCTATGACCCACTCGGATTTGTGATGTATGCATTTGAGTGGGGAGCTGGCGATCTTGATGGCTTCGACGGTCCA
GATGAGTGGCAGAAAGAGTTTCTCACTGATTGGGGTGAAGCAATTCGCACTAACAACTTTGATGGTGTAAAGCCGGTAGAAGCATACCGATGCGCTACAAGTTCCGGCCACGGTATCGGG
AAAAGTGCGCTTACCGCCTGGGTAATTCTCTACATCATGAGCACTCGCCCATTCTGTAAAGGCGTCGTAACTGCCAATACATCAGAGCAGCTTCGCACCAAAACGTGGGGCGAGCTTGGAAA
ATGGAAGAAGCGTTGTATCACCGGGCACTGGTTCGAGTACAACAACGGAAAAGGCAACATGAACATATACCATGTAGATCACATGGAGTCATGGCGTTGTGACGGGCAAACCTGCCGTGA
GGAAAACAGCGAATCATTTGCAGGACTGCACTCCGCTAACTCAAGCCCGTTCTATATATTCGATGAAGCCTCAGCGGTGCCAGACAAGATATGGGAAGTCGCAGAAGGCGGACTGACTGAC
GGCGAACCTTTCTGGTTCGCTTTCGGCAACCCGACACGTAACACCGGACGATTCCGTGAGTGCTTCCGTAAATTCAAGCATCGCTGGCGTCGGCGTCAGATTGACAGCCGAAAAGCTAAGA
TGACAAACAAAGAGCTTATCGAAGAATGGCGCAACGACTACGGCGAGGATAGCGACTTCTTCAAAGTGCGCGTTCGTGGCCTCTTCCCGTCTGCTTCAGACCTTCAGTTTATTCCTCAAAGC

TATGCCGATGCTGGGATGTCCAGAAAGCTGGAGCACAGTCAGTATGGATTCGCTCCAAAGATTATCGGCGTTGACCCGGCATACTCTGGGAGCGACGAGGCATGTATCTATCTCAGGCAAG
GCCTGCACTCAAAGCTTTTAGGTTCCTACCCAAAAACAGACGACGATGTGAAATTTGCTCAGGTTGTTGCTGCTATCGAGGATGAACATAAAGCTGACGCGGTATTCATCGATTTCGGTTAC
GGAACGGGTATTCATTCTGTAGGTAAGTCATGGGGAAGAAAATGGCAACTTGTAAGCTTCGCAGGTGAATCGAAAGACCCGGCAATGCTCAACAAGCGCGGAGAAATGTGGAATGCAATG
AAATCCTGGCTGAATGAGGGAGGCAGCATTGACGACCAGCAGACCGCCGATGAGATTGTCGCCCCAGAATACAAAGTGAAGCTGGACGGTAAGATTGTTCTTGAGTCGAAAGACGACATG
AAACGCCGCGGTGTTCCCTCGCCAAACAGGGCCGATGCGCTGGCGCTGACATTTGCATTCCCGGTAGTCAAAAACAAACCTTCAAAAGCAATCCCCGCACCGATTAGACCAGTACGCAGAG
GACGATAA
>OCONKI_07870 Portal protein
ATGGCCGACAATGAAAACAGGCTGGAGAGTATCCTGTCGCGCTTTGATGCGGACTGGACAGCCAGCGATGAAGCCAGAAGGGAGGCCAAGAATGATCTCTTCTTCTCCCGTGTATCTCAGT
GGGATGACTGGCTATCACAATACACAACCCTGCAGTATCGCGGGCAGTTCGATGTTGTACGTCCAGTGGTGCGCAAGCTCGTTTCTGAGATGCGTCAGAACCCTATTGATGTTCTGTATCGTC
CAAAGGACGGAGCAAGCCCTGATGCCGCTGATGTGCTTATGGGTATGTATCGCACAGACATGCGACACAATACGGCAAAAATCGCGGTCAACGTCGCTGTTCGTGAGCAGATTGAAGCAG
GTGTAGGTGCGTGGCGTCTGGTCACTGACTACGAAGACCAAAGTCCAACGAGCAACAATCAGGTTATCCGTCGAGAGCCTATCCATAGTGCCTGTTCCCATGTTATATGGGACAGCAACAGC
AAGCTGATGGATAAGTCTGATGCCCGTCACTGCACAGTTATCCACTCAATGAGCCAGAATGGTTGGGAGGATTTCGCAGAAAAATACGACCTCGATGCGGATGATATTCCATCATTCCAGAAC
CCCAACGATTGGGTATTTCCATGGCTGACGCAGGACACAATTCAGATCGCTGAGTTTTACGAAGTGGTCGAGAAGAAAGAGACGGCGTTTATCTACCAAGACCCGGTTACGGGTGAGCCG
GTAAGCTACTTTAAGCGCGATATTAAAGACGTCATCGATGACCTGGCTGATAGTGGATTTATCAAAATTGCAGAGCGCCAGATTAAGCGTCGCCGGGTATACAAATCGATTATCACCTGCACT
GCTGTACTCAAAGACAAGCAGCTCATTGCTGGCGAGCATATCCCCATTGTTCCGGTGTTCGGAGAGTGGGGCTTCGTTGAAGATAAAGAAGTGTATGAGGGTGTCGTCCGCCTGACAAAAG
ACGGCCAGCGTCTGCGCAACATGATTATGTCGTTCAACGCCGACATCGTGGCCCGCACTCCGAAGAAGAAGCCGTTCTTCTGGCCTGAGCAGATTGCAGGCTTTGAGCATATGTACGACGG
TAACGACGATTACCCATACTACCTGCTCAATCGCACTGACGAAAATAGTGGAGACCTTCCGACTCAGCCGCTGGCATATTATGAAAACCCGGAAGTGCCGCAAGCCAACGCCTACATGCTGG
AAGCAGCAACCAGCGCAGTAAAAGAGGTTGCCACTCTCGGAGTTGATACAGAAGCGGTAAATGGCGGACAGGTTGCGTTTGATACCGTCAATCAACTGAATATGAGGGCTGACCTTGAGA
CATACGTGTTTCAGGATAATCTGGCTACCGCCATGCGCCGTGACGGAGAGATTTACCAGTCGATAGTTAATGACATCTACGATGTTCCTCGCAACGTTACGATTACCCTTGAGGATGGCAGCG
AGAAAGATGTTCAGCTAATGGCTGAGGTTGTTGACCTTGCTACTGGAGAAAAGCAGGTACTAAACGATATCAGGGGGCGCTATGAGTGCTACACGGATGTTGGACCATCATTCCAGTCCAT
GAAGCAGCAAAACCGCGCAGAAATTCTTGAGTTGCTCGGCAAGACGCCACAGGGAACGCCAGAATATCAACTGCTGTTGCTTCAGTACTTCACCCTGCTTGATGGTAAAGGTGTTGAGATG
ATGCGTGACTATGCCAACAAGCAGCTTATTCAGATGGGCGTTAAGAAGCCAGAAACGCCCGAAGAGCAGCAATGGTTAGTAGAGGCGCAACAAGCCAAACAAGGTCAACAAGACCCGGC
AATGGTTCAGGCTCAGGGCGTACTCCTGCAGGGGCAGGCTGAACTGGCTAAAGCTCAGAACCAGACGCTGTCCCTGCAAATCGATGCAGCTAAAGTCGAAGCGCAGAACCAGCTTAACG
CTGCCAGAATCGCAGAAATCTTCAACAACATGGACCTCAGTAAACAATCTGAGTTTAGAGAGTTCCTTAAAACCGTTGCTTCATTCCAGCAGGACCGCAGCGAAGACGCTCGCGCAAATGC
TGAGTTACTCCTTAAAGGCGATGAACAGACGCACAAGCAGCGAATGGACATTGCCAACATCCTGCAATCGCAGAGACAAAATCAACCTTCCGGCAGTGTAGCCGAGACACCTCAATAA
>OCONKI_07875 Scaffolding protein
ATGGAACCAACCACCGAAATTCAGGCAACTGAAGACTTAACCCTGTCCGGCGATCATGCAGCGGCATCTGCTGATAGCTTAGTTGTCGATAATGCCAACGACAATGCAGGTCAGGAAGAGG
GCTTTGAGATTGTCCTGAAGGACGATGAGACAGCACCAAAACAAGACCCGGCAAAGAACGCAGAATTCGCCCGCCGCCGCATCGAGCGCAAACGACAGCGCGAGCTTGAGCAGCAGAT
GGAGGCAGTTAAACGCGGAGAATTGCCGGAGAGTTTACGGGTAAACCCTGACCTTCCACCTCAGCCGGATATTAATGCCTATCTGTCAGAAGAAGGCCTGGCTAAATATGACTACGACAAC
AGCCGTGCGCTTGCCGCTTTCAATGCTGCTAATACCGAATGGCTAATGAAAGCGCAGGACGCCCGCAGCAATGCCGTAGCAGAACAGGGCCGCAAGACTCAGGAGTTTACCCAGCAATCA
GCGCAATACGTCGAAGCTGCCCGCAAACACTATGACGCGGCGGAAAAGCTCAACATCCCTGACTATCAGGAGAAAGAAGACGCATTTATGCAACTGGTTCCGCCTGCGGTTGGGGCCGAC
ATTATGCGCCTGTTCCCGGAAAAGTCCGCCGCGCTCATGTATCACCTGGGGGCAAACCCGGAGAAAGCCCGCCAGTTACTGGCGATGGATGGGCAGTCCGCGCTGATTGAACTCACTCGAC
TATCCGAACGCTTAACTCTCAAGCCTCGCGGTAAACAAATCTCTTCCGCTCCCCCTGCTGACCAGCCGATTACCGGTGATGTCAGCGCAGCAAATAAAGATGCCATTCGTAAACAAATGGATG
CTGCTGCGAGCAAGGGAGATGTGGAAACCTACCGCAAGCTAAAGGCAAAACTTAAAGGAATCCGATAA
>OCONKI_07880 Major capsid protein
ATGGCTTTGAACGAAGGTCAAATTGTTACACTGGCGGTAGATGAAATCATCGAAACCATCTCCGCAATCACTCCAATGGCGCAGAAAGCCAAGAAATACACCCCGCCTGCTGCTTCTATGCA
GCGCTCCAGCAATACCATCTGGATGCCTGTAGAGCAAGAGTCACCCACTCAGGAGGGCTGGGATTTAACTGATAAAGCGACAGGGTTACTGGAACTTAACGTCGCGGTAAACATGGGAGA
GCCGGATAACGACTTCTTCCAGTTGCGTGCTGATGACTTGCGAGACGAAACTGCGTATCGTCGCCGCATCCAGTCTGCCGCTCGCAAGCTGGCGAACAACGTTGAGTTGAAAGTCGCAAAC
ATGGCCGCCGAGATGGGTTCGCTGGTTATCACCTCCCCTGATGCCATCGGCACTAATACCGCAGACGCCTGGAACTTTGTGGCCGACGCAGAAGAAATCATGTTCTCCCGCGAACTTAACCG
CGACATGGGGACATCGTACTTCTTCAACCCTCAGGACTACAAAAAAGCGGGTTACGACCTGACCAAGCGTGACATCTTTGGGCGTATTCCTGAAGAAGCATACCGAGATGGCACCATTCAG
CGGCAGGTTGCAGGCTTCGATGATGTCCTGCGCTCTCCGAAACTTCCTGTGCTGACCAAATCTACTGCAACTGGTATCACTGTATCCGGTGCTCAGTCCTTCAAGCCTGTCGCATGGCAACTG
GATAACGATGGCAACAAAGTTAACGTTGATAACCGTTTTGCTACCGTCACCCTGTCTGCAACTACCGGCCTGAAACGCGGCGACAAAATTTCGTTTGCTGGCGTGAAGTTCCTTGGTCAGAT
GGCTAAGAACGTACTGGCGCAGGACGCGACTTTCTCCGTAGTTCGCGTTGTTGATGGTACTCACGTTGAAATCACGCCGAAGCCTGTAGCACTGGATGATGTTTCTCTTTCTCCTGAGCAAC
GGGCATACGCCAATGTTAACACCTCGCTGGCTGATGCGATGGCGGTGAACATCCTGAACGTTAAAGACGCTCGCACTAATGTGTTCTGGGCTGACGATGCTATTCGTATCGTGTCTCAGCCG
ATTCCGGCTAACCATGAACTTTTTGCAGGTATGAAAACTACCTCATTCAGCATCCCTGATGTTGGCCTGAACGGTATCTTCGCTACGCAGGGTGATATTTCCACCCTGTCCGGCCTGTGCCGTA
TTGCGCTGTGGTACGGCGTAAACGCGACACGACCGGAGGCAATCGGTGTTGGCCTGCCTGGTCAGACTGCGTAA
>OCONKI_07885 hypothetical protein
ATGAAAATAGCAATCTATAAGCCCGGTGGAAGCATCATGGTATGGGGCGTCATGGCTCAGATGAAGGTCATCGACTCCAGCGAACTTCCGGAATATGTCAAAGATGGCTGGCTTGATCATCC
GTCAAAGTTGCTTCCCGCGGAAGCAGATGATGTTAAGCCACGCAAAGGCCGCAAGCCTAAGGCGGTAAGCGATGCAGATAAAGACTAA
>OCONKI_07890 hypothetical protein
ATGCAGATAAAGACTAAAGGCGATCTGGTCAGGGCTGCGCTTCGTAAGTTGGGCGTGGCATCAGATGCAACCCTTACCGATGTCGAACCTCAGTCTATGCAGGATGCCGTAGATGACCTCG
AAGCGATGATGGCGGAGTGGTATCAGGACGGGAAAGGCATCATTACCGGCTATGTATTCTCAGATGATGACAATCCTCCTGCTGAAGGTGACGACCACGGCCTTCGCTCTAGTGCAGTCAG
CGCGGTATTCCACAATCTGGCTTGCCGGATTGCCCCGGATTATGCGCTTGAGGCTACTGCCAAAATTATCGCCACTGCTAAATATGGGAAGGAGCTTCTCTATAAGCAGACCGCCATCGCCAG
AGCAAAAAGAGCTCCTTACCCGTCACGCATGCCAACAGGCAGCGGTAATAGTTTCGCCAATCTGAACGAATGGCATTATTTCCCCGGAGAGCAGAATGCCGATTCAACAACTCCCCATGATG
AAGGGAATGGGTAA
>OCONKI_07895 Phage head DNA stabilization protein
ATGCCGATTCAACAACTCCCCATGATGAAGGGAATGGGTAAAGACTTCAAGAACGCCGATTATATCGACTATCTGCCAGTGAATATGCTGGCAACACCCAAAGAAATCCTTAACAGCAGCGG
CTATCTCCGCTCATTCCCTGGCATTACCAAACGTTATGATATGAACGGCGTATCGCGTGGAGTTGAGTACAACACCGCTCAGAATGCTGTTTATCGTGTTTGTGGTGGCAAGCTCTACAAAGG
AGAAAGCGAGGTTGGTGATGTTGCCGGAAGTGGTCGCGTATCAATGGCACATGGTCGCACATCACAGGCGGTAGGCGTTAATGGTCAACTGGTCGAGTATCGCTATGATGGTACGGTTAAA
ACCGTCTCAAACTGGCCTGCAGGCAGCGGATTCACGCAGTATGAGTTAGGCTCGGTCCGCGACATTACGCGCTTACGTGGGCGTTATGCGTGGTCAAAAGACGGAACTGATTCATGGTTTG
TCACTGACCTTGAAGACGAATCGCATCCTGACCGATACAGTGCACAATATCGCGCAGAATCGCAGCCGGACGGCATCATCGGTATCGGGACATGGCGAGACTTCATCGTCTGCTTTGGTTCG
TCAACGATAGAGTATTTCTCTCTGACAGGCGCAACCACCGCTGGCGCTGCGTTGTATGTCGCACAGCCATCGCTGATGGTACAGAAGGGCATTGCCGGAACATACTGTAAAACGCCATTCGC
TGATTCATATGCATTCATCAGTCACCCGGCTACTGGCGCACCTTCCGTCTACATCATCGGGTCAGGGCAGGCTTCACCAATTGCGACGGCCAGTATTGAGAAAATTATCCGCTCATACACGGCT
GATGAACTGGCGACGGGTGTAATGGAGACTTTGCGCTTCGATTCTCATGAGCTTCTGATTATTCATCTCCCTCGTCATGTTCTGGTTTACGACGCATCGTCAAGTCAGAACGGACCACAATGG
TGCGTGCTGAAAACCGGGCTTTACGATGATGTATATCGTGCTGTCGACTTCATGTATGAAGGCAACCAGATTACGTGCGGCGATAAATCAGAAGCGGTGACAGGACAACTGCAATTTGACAT
TAGTAGCCAGTACGAAAAACAACAAGAGTTCATCTTGTTCACGCCAATCTTCAAAGCAGAGAACGCCAGATGCTTCGACCTCGAAGTTGAATCATCCACTGGTGTTGCTCAATACGCCGACC
GCCTGTTCCTGTCTGCAACAACTGACGGTATCAATTACGGTCGTGAACAGATGATTGAGCAGAACGAGCCGTTTGTGTACGACAAGCGCGTTTTATGGAAACGTGTTGGGCGCATTCGTCG
ATTAATCGGATTCAAACTGCGGGTAATCACCAAATCGCCAGTAACACTATCCGGGTGTCAAATTCGTCTGGAGTAA
>OCONKI_07900 Phage tail protein

ATGGCAGACCCGTCACTTAATAAGCCTGTCGTGGTTCAGGCTACACGCATTGATGCTTCAATTCTTCCCCGTAACGTCTTTAGCCAGTCTTATTTGCTCTATGTAATCGCGCAGGGAGCTGACG
TTGGTGCTATTGCAGGCAAGGCAAATGAGGCAGGGAGTGGCGCTTACGACGCACAGGTTAAAAATGATGAGCAGGATGTAATTCTTGATGAGCACGAAAAAAGAATTGCAAAAACAGAA
GAGGATATTTCAGGAATAAAAGTAAAGCTTCTTGAAATAGAGAATGATGTTAATGGTCTGAAAATAAAAGTTGAGGATATCGACGGTAAAGTATCAGAGATAATCGTTGATTATGTTTCACTC
AGCAGAACAGGAACTCAAACTCTTGCCTCATCCCTTAACGTATCAGGAAGTTATTCTGTTAACGGTACAAAAGTTGTTGGCGCTCGCCAGACTGGATGGACCGCGGCAACAGGTACGGCGA
ATAAAGGCGTATTCGATGCTGACCTGACATTCGCCGTTAGCGATACTTACACGCAATCTGAAATCCAGGCTATAGCCAATGCTCTAATTACTGAGCGTCGGCGCACTAAGGCTTTGGAAGACG
CCTTGCGTGCACATGGGTTGATTGATTAA
>OCONKI_07905 Protein gp14
ATGATTACATTCACTCCAACACGCAACATCGACCTGATAGAAATGGTTGGCAACCACCCCGACATCATTGCCGGAAGCAACAACGGTGACGGATACGACTACAAGCCTGAGTGTCGTTACTT
TGAAGTGAACGTACATGGTCAGTTCGGTGGCATCGTGTATTACAACGAGATTCAGCCGATGACCTTTGACTGCCACGCCATGTACCTGCCTGAGATTCGCGGATTCAGTAAGGAAATCGGAC
TGGCGTTCTGGCGATACATTCTCACCAATACCACCGTTCAGTGCGTTACATCATTTGCTGCACGCAAATTTCGCCACGGGCAGATTTACTGCGCAATGATTGGCCTTAAGCGTGTAGGAACCA
TCAAGAAATACTTCAAAGGCGTGGATGACGTGACGTTTTACAGCGCCACACGCGAAGAACTAATCGACTTCCTGAATCACGGGAGATAG
>OCONKI_07910 Internal virion protein gp7
ATGTTATATGCATTTAAGCTGGGCAGAAAACTGCGCGGCGAGGAACCTTATTGCCCTGAAAAAGGCGGGAAAGGTGGCTCCGATAAAAGTGCAAAGTATGCCGCAGAAGCTCAGAAGTAT
GCCGCAGACCTGCAAAATCAGCAGTTCAACACCATCATGAATAACCTGAAGCCGTTTACTCCTCTGGCTGAGAAGTATGTCGGCAGCCTCGAGAACTTATCGTCTCTGGAGGGGCAAGGTC
AGGCACTTAACCAGTATTACAACTCTCAGCAGTACAAAGACCTTGCAGGTCAGGCTCGCTATCAGAGTCTGGCGGCAGCGGAAGCAACAGGTGGATTGGGTTCCACTGCAACCAGTAATCA
GTTAGCAACAATCGCACCAACGCTTGGTCAGCAGTGGCTGTCTGGCCAGATGAACAACTACCAGAATCTGGCAAATATTGGTCTTGGCGCTCTTCAGGGGCAGGCAAACGCCGGGCAAAC
ATATGCCAACAACATGAGCCAGATTTCACAGCAAAGCGCGGCACTGGCGGCGGCAAACGCTAATAGACCTTCCGGCCTTCAATCTGCAATAGGCGGGGCTGCCTCTGGAGCAATTGCTGG
AGCACAGCTTGGCAGCATTGTTCCCGGTATTGGTACTGGTATTGGTGCCGCTGTTGGCGGCGGTCTTGGTCTTCTTGGTTCACTGTTTTAA
>OCONKI_07915 DNA transfer protein
ATGACTACGTGGCAACAGGGTATTAATTCTGGTGGGTTTCTTGCTGGCATCGGTGCGCAAAATGAGAATGCGCCAAAGGCAAGCGACATTAACGCAACGCTTGGTCTGATCCGCGAAAACA
ATGAACTGGCTCGATCAGGTGCAAATAACGTTGGTCTGACCGCGTTACGTGGTCTGGCTGGAGTTGCTGATATTTATAAGCAGGAACAGCAACAGAAAGCGATTAGTGCGTTCAATAAGGT
TCACGCTGATGCATGGGCTTCTGGTGATCCATCGGGACTATTTAAGTTTGCCCAGGAAAATCCAGCGTTTGTTGCACAGGCACAACAGGCGTTTTCCGGTCTTAATGAGCAGCAACGCAAC
GATATGGGCGATTTAGCCATGAGGGCTAACGTCGCTCTTTCTCAGGGACCGGAAGCCTACAGTAAATTCATTACTGACAACAAGGACAGGTTAAATCGCGTTGGCGCTAATGCTGACTGGAT
GATTCAGACAGGTATCCAGAATCCAGAGCAGCTATCACACATGCTGACTACTATGTCTCTCGGTGCGCTTGGACCAGAAAAGGCGTTTGCTGTTCAGGATAAGATGGTTGGTCGGGATATTG
AGCGTGGCGAGTTGGCAGAGACGATTCGCAGCAATAAAGCTGGGGAAGGTTTACAGGCGCAAAGCATTGCTGTTAGCCGTGAAAACTCTCTGCGCACTGCTGGAGGTGCTGTTCCTGCAT
CTGTTAAAGAATATCAATATTTCAACAGCCTGTCTCCAGAGCAACAAAAGACATATCTTCGTGTTCGAGGCCGTCCTGCTGCTGGCGGGGAGAATGTTGTGCAACTGGCGGATGGTAGAACG
GTAACGGTTGGTAGGAAGCTTCACGGCGCTGGTGCTAATGCGTTCTACGAAGGAATAGACAACGAGGGCAATATGATTCGCGTCCCCGCAAGTTCTATTGCTGCTCCGGCTACATCGGCAG
CTAATGCGCAGAATTACGAAATGAAGAAAGATCTTGATGCAATTTCTGGCGCATCAATTGACGATCTTGGCTTCATGACAGGTATTACAGGTTCTTCAGGTTCTTTTGCTCTTGGTGCAGATAT
TCGTAGCCGTGCATCTGGTGTTGAGGAGAGGAAACTATACAACGCTGCACAGCGAATCATGGGCAAAATGCAAAATCAGGGTATTGCGGCAGCCCGAGATATGGGGGCATCTGGCATCAA
CACCGAAGCAGAGGCCAATAGGTATTTTCAGGGTATGCCAAAGCCTGATTTCTCAAGCCCTGAAGCACTACAACAATCAATGCGCGACATTCAGCAATATACCGACAATTACAACCAGCAAT
ATAACGTTAATGTCGGTAATGGCGGGAAGAAATCATCAAGGCAACAGCCAGCTACTCAGCAATCAGCAGGAGGTAGCTACACGTCAAAATCAGGCATTCAATTTACGGTGGAATGA
>OCONKI_07920 DNA transfer protein
ATGATGAAAGTAACTGCAAATGGTAAGACATTTACCTTTCCTGATGGTACGAGCACGGAAGATATTGGGGCCGCCATTGATGAGTATTTTGCTGGTCAGGCTGTTCAGCAACAAACAGTTAA
TCAGGCCAATAATGCACCAACACGGGAAGAACCATCATTGATGCAACAAGCTGGCGATTGGCTCACAGGTGGTCAAAGTGCAGGGCAAATTGCAGAACAGGCTGGTCGTGGTCTGGTAAA
CATACCATTTGACGTATTGCAGGGCGGCGCAAGTCTGATTAATGCAATCAGCCAGGGGCTTGGTGGACCCAAGGTTTTGGATGATGTTTATCGTCCAGTAGACAGACCGACAGACCCCTACG
CGCAAGCCGGTGAAACAATTGGTGGGTATTTAGTTCCAGGAGTTGGAACGGCAGGAAGCATGGCTATTGGATCACTGGCAGAGGCCGCAAATCAGAAAGGCGATTTCGCACAAAATGCA
GCTAAAAATGCCGGAGTTAACCTTGCCGCTCAGGGTGTTCTTTCCGCCGCAGCAAAGGGAATAGGGCGTGGAATAACGGCTATAAAGGTGATATTGCGCCAGAAGTGGCGAAGAAAATT
GCCACATCAGAATCGATGGGCGTGACACCAATGACATCTGATGTTATCCCGCCGAAAAATGCTTTCACTCGCGGCCTTACTCAGGATGCCGAGGGGGCTTTGCTCGGGACAGGCCCAAAGC
GAGCGGAGCAATATGCAACGCGTAGTAAGCTGGTAAGTAATTATTTTGACCGTTTTGGTGAGTACAACCCTGATGATGTGGTGAAATCTCTGACCACCACGTTAAGGGGGAGGAAGGATGC
CGCTGGCGCTGTTATCAATGACGTCACCAATAAAATGGGTAATGCCGCAGTTGATACCACAAATACCATGAATGCTCTGAATACAGCGATCGCAAGACAGGAACGACTTGGGACGTCTGCCA
ATCAAAGCCTGCTTACATCCTTGCGTAACTTACGTGAAGAATTAGCAAACCCTGCAACTGATTTGGATGTTACGTTTGATCTCTTGCGTCAGCACAGAACAGCATTTAGATCTAATGTTCAGGG
AGATGCTATGGTCTTCCCCAACCAGGCAAAAGCAGCTACCAATATGGTAGAGAATGCAATGTCAAAAGACCTTCGTAACGCAGTTGCTAAAAACCTCGGTGCATCAGACGCAGCAAAATACC
TTAAAGCAAATTCCGATTATGCAAACGTTTATAATAAGGTGCTTAATAAAAACATTGCTAACAAACTCAACAAGGCAAGCAGTGAAGCCAGTCCTGAACTTATAAATACCGTTGTATTAAGCAG
AAAACCATCTGACGTGAAACGAATCTGGAGCGCACTGGATGATAAAGGGAAAGATGCTATGCGTGCAGCTTACGTCAGCAAAATAGCGGAAAAGGCCGGTGACTCTCCAGCCAAGTTCAT
CACTGAAGTTAATAAGCTGAAATCTCAGTCAGGCGGTGAAATTTACAACACTATTTTTTCTGGAAAGCACATGAAAGAACTTGATGCTCTTCATGAAGTTCTGCAGCAAACAGCAAGGTCAG
ACACCGCAAATGTAGTAACTCAGACGGGGCAATCGCAAGCCAACAGGATAAGGACGATTGGCGCAACTGCGACTCTTGGCGTATCAATGGGGCTTGAGGCTGGTTTCGGTGCAATGATGC
GCTTGTATGAGTCCAAAGCAGCAAGGAATGCGCTCTTACGTCTGGCAAACACCAAAGCAGGAACGCCAGCCTATGAAAGAGCATTAAGTAACGCTGCAAATGCCATCAGACCGCTGCTTGC
CACTGAGGCAACACAGCAGTGA
>OCONKI_07925 P63C domain-containing protein
ATGACTGAAAAGAAAAGTGGCGAAGGGAAAGCTAAGGGCGGGATCGCTCGCGCAAAGTCGCTGACTAAAGAGCAGCGTTCTGAAATAGCAAAGAAAGCAGCTGCTGCAAGATGGAAAA
GTAAGATTCTCAGGGCAACTCATCGTGGTAACTTTTTAGATGATTTTGGCATTGATGCTGAATGTTATGTACTGGATGACGAGTCGAAAACTGTCGTTGTTACGAAAACTGGATTGTCTCAGTT
GCTAGGGATTGGTGAACATGCCAGGGATTTAGATCAACTGCTTGGCGCTCAGTATATGAGCAAATACCGAGATCTAGAATTGCAGCGAAAAATGGAAAATCCCTATAAATTTCAACTTACTTC
GAAGTCTAAAACCGTTCATCAAGCGTTAGGTTATGACATTACAGCAATTGTTGATATTGGTAGGGCACTAATAGAAGCCAAAGATAATGACGATCTACCACAATCACGGTTAAAGGCAGCCGC
CGCAGCACAGAGACTTATTAATGCCTCCGCTAAGGCGGGAATTAAGGGGGTTGCGTATGCGCTTGCTGGTTATCGTCCAGAAGTTCAGGCTGTCATTGACGAGTTCAAAGCGTTTGTTCGT
GAAGAGGCTCGTCAATATGAAAAGGAATTTCCAGATGAGCTATACGAGGAGTGGTATCGACTGTACGGCCTGAATAGGCCAGAGAAAGGACGGCCTATTCGTTTTGGGCAGCTAACCAAC
ATGCAGATATACACCCCGCTAGCAAAGAGTAAAGGTAAAATCCTTGAACAAATTCGAGCCAGCCGAGACGAGAACGGAAAACAATCTGATAAGTTGCATCTGTTCCTTTCTGAAATTGGTGT
CAAGGCTTTGCGTCAGCATATCGGTAAGCTTCTTGGTGTCGCAGCGATGAGTGAGACAAGAGAAGAATACGAAAAAGGAATAGAAAAGGTTTTCGGAAGAATGAAACCAGAAATCTAA
>OCONKI_07930 hypothetical protein
ATGCTGTTTCTTTCCATCGATAGTCCATGTGAAGTACTTTTTCTTAAATCCAGCACCACAGATATCGCAACTTCTTGGTTTAAACATACTGGCTATGAAGACAAGAACAACAAATCCTATGATCC
ATTCCATATCATGTACCTGTTTTAA

Prophage 4.3

>PNPANE_15620 hypothetical protein
ATGACAGACATTACAGCCAATGTGATCGTATCGATGCCTTCGCAACTCTTCACTATGGCGCGTTCTTTTAAAGCCGTAGCTAAGGGCAAGATTTATATCGGAAAAATTGACACTGACCCCGTAA
ATCCTGAAAACCAGATTCCTGTATATCTGGAACGAGAAGATGGTACTCATATTCAGGTGCCACAGCCAATCGTTATCAACGCTGCTGGATATCCGGTATACAACGGACAGATTGCCAAGTTTGT
AACTGTGCAGGGACATTCAATGGCTGTATACGATGCCAACAATGCTCAGCAGTTTTACTTCCCAAACGTGCTTAAGTACGACCCTGATCAGCTTGAGTACAGACTTAGTCAGCCTGATGGGTA
TCTATTAGTTGGTGGGCTAGCAGAACATTATAGCCTTCCTGTTAAGTTCGTTGTTGTAGATAACGCACCTTATAATGGAGATTTAAAAGCAGCACTAACAGCAGCAACATCTGGTAGTGTTTTT
TGGTTGGGTAAGAAAACGTATAACATTACGGGGTTATATGGTGTAAATAGAAACACCGTTGAGAATATTACTATTGTTGGCGCTGGAATGCCTCAGCTGTCATCTGACAAAAGATATCTCATGG

ACGGAACAGGAACCATCATTCAAGGTACAATTAAAAATCAGGCCAAGGGATTCAAAATATTTAATTTAGGTATTGACGTTGGTGATTATGTTTCTCAAAACGTTTATCCGTCGGTTACTTATGA
AGATGGCCTGCAACATTATGGCGCTGGCAGTAATGCCAACCTTGAAATAAATAATGTAAAGCTCCTTAACACCGTGACAGACCCGTCAAAACCTGGCACTCACAGCTTACTGCTTGAACAGT
TGTCAGGTGTTAAATTAGGGTACGTGGAATGTATCGGCGGTTTTCATGGTTTTACAGTTAAATGCCAGGGTTTGCAGGGTGGAATTGCACACTGCTACGGTCAGTATGGTGATGCATTTATTT
TCAAATCTGATTCTGGCGGGGCGTGTGCCGATAACTACATGGAAAGAATCGCAGTTGGTCTTTATGACAACTCCGGATGGCCTGATGTCACTATGGGCGGGATTTATGATGCTCATGATAACG
TAACAATCGACAGAATTGGTATTGGTGAGTTAATTGTACAGAATGCATCATGGGGATTGATACCGTCTGATGCTAATACCGGATTCATAACAAACGTCAGCATTGGTAGGTACTCTGCATTTAAT
GTCTATGGGAACTATTATTCATTAACCATTGATAATAAATGTGTTGGTTGGACTATCGGTGAGCATAGGATTAGTAATGCTTCTGGCGGTATTCGTGTTCATCCTGATTCAGTTGAGATAAATATA
GGAACAGGATCGTCAAAAGGTAACACAAAAAGTGGTTATGCACTCGGTGGGAATAGCTTGACCCATGGAAAGCTATTTGCAAATGAGAATGGGGAGGCAGGAGTTGATTATCTTGGTGGC
CTGGGTCTTGATGCTTCTCTCATTAATGGATACATCAACGGTACTGTTCTTATTTCAGGTTATCCAGGTGTAAAAGACGGAAATCCATTAAATGGATGGGCAGATACCGGAGCTTTCGACATGA
TTTTAACTGGCAAGACAGTGCAGGTCACAGGGTCGCTGACGCGCGGAACAGCAGCAGTTGCATATAACACTATTTCTCCATGCAGACCTATAAAACGAGTTCCCATTCCTGCGTGGGGGGT
TAGTGCATCAAGCACAATGATTCCTGTCGAATGTTATATCGAAACAAACGGACAGTTAAACGTAGCTGGGTTCGCATCAATACCAGTCGGTGGTACTGTGAATTTTAACGGAAATTATTTAACT
AAATAG
>PNPANE_15625 Bro-N domain-containing protein
ATGAAAAGTATAGCAAAGGCACAAAACGATTTCACCATCTTCAAATTCGGCGACAGTGAAATCCGCGTCATCAACAAATGCGGTGAGCCGTGGTTTGTAGCAAAAGATGTTTGTGATGCTTT
AACCCTGACTAACTCACGCAAGGCGCTTACTGCACTTGATGACGATGAAAAGGGAGTAACTTTAAGTTACACCCTTGGCGGTGAGCAGAATCTAAGCATTGTTAGCGAATCAGGTATGTATA
CATTGGTTCTGCGCTGCCGCGATGCTGTCAATAAAGGTTCGGTCCCGCACAAATTCCGCAAGTGGGTAACAGCAGAAGTTCTACCTTCAATTCGCAAACATGGCGAGTATGTGAAAGGCAA
GAAAACCACTGTTGAGGAAAGAACGCCGCTACGCGATGCAGTAAACATGCTGGTAGGAAAGAAAGGACTTCGCTATGACGATGCATACAATATGGTTCATCAGCGTTTTGGTATTGACAGC
ATTGATGAACTTTCAATTGAACAAATCCCGCTGGCCGTAGAGTACATCCACAGGGTAGTGCTTGAAGGTGAGTTCATTGGCAAACAAGAGAAGAAAACCAACGAGCTTTCTGCAAAAGAA
GCAAACAGCCTTGTATGGTTATGGGATTATGCCAACCGCTCACAGGCATTATTCCGCGAACTGTATCCGGCGCTAAAACAAATTCAATCGAACTATTCCGGCAGATGCTACGACTACGGTCAT
GAGTTCTCGTATGTTATCGGAATGGCGAGAGACGTTTTAATCAATCACACACGAGATGTTGATATCAATGAGCCAGACGGACCAACGAATCTTTCCGCATGGATGAGACTTAAGAATAAAGA
ATTACCTCCTTCAGTACATAACTACTGA
>PNPANE_15630 sar RNA
TCAATATTTCCTAATCCGATTTGTTGATACCGAAGCCCTGACTGTTCCCGCAGTTGGGGCTTCAACT
>PNPANE_15635 hypothetical protein
ATGAAAGGTGCAAGAGATATCAGTCCCTTGGGAATTAGGATTCCAGACTATTTAAAGAAACGCATTCAGCAGGAAGCGGATAAAAACGGCAGATCAATGAACTCAGAGATTGTGCTTATCCT
TCAAAAACATGTTGATAATCTTGATGGCCCTCGCTCACTTGAAGGATTCGCTAACCAAGAAGCTGATAAATTCAAAGAGGCGCTGCTTGAGACGCTGAAAACCATGTATGGTAAGGATGAAA
AATGA
>PNPANE_15640 GRIP domain-containing protein
ATGAACAAAAAACAGTTTATTAAGTCAACAACGTCAAGCAAAGAAGAGCTAGAGAAGGAGCTAAACTCCCTGAAATATGCTCTGTGCCTGGTTTACTCAAGACTGCCAATGGAAGATAAGA
ACGCCATTTACAATGAAATGATTAGCAGCCTTGATTTTAACGATAGAGACCTAGCATCCCACCTCAACAGCTTCCGCGTCCCTGAGTAA
>PNPANE_15645 Cytoplasmic protein
ATGACCATAGAAGAACGCCTGAACAACATTGAGTTGAACCAAACCCTGCTTGACCAGCGACTTTCAGATCTTGAGCTTAAAGATCTTGATGCGCAAATATCAGAAGCAGAAGCCAAGCTCTC
CAGCTTAAACCACCGTAAGAAGCAAATCCGCAATAGAATTACTCAGGGACGCGGAAGCTGTTGA
>PNPANE_15650 hypothetical protein
ATGCTGTTTCTTTCCATCGATAGTCCATGTGAAGTACTTTTTCTTAAATCCAGCACCACAGATATCGCAACTTCTTGGTTTAAACATACTGGCTATGAAGACAAGAACAACAAATCCTATGATCC
ATTCCATATCATGTACCTGTTTTAA
>PNPANE_15655 P63C domain-containing protein
ATGACTGAAAAGAAAAGTGGCGAAGGGAAAGCTAAGGGCGGGATCGCTCGCGCAAAGTCGCTGACTAAAGAGCAGCGTTCTGAAATAGCAAAGAAAGCAGCTGCTGCAAGATGGAAAA
GTAAGATTCTCAGGGCAACTCATCGTGGTAACTTTTTAGATGATTTTGGCATTGATGCTGAATGTTATGTACTGGATGACGAGTCGAAAACTGTCGTTGTTACGAAAACTGGATTGTCTCAGTT
GCTAGGGATTGGTGAACATGCCAGGGATTTAGATCAACTGCTTGGCGCTCAGTATATGAGCAAATACCGAGATCTAGAATTGCAGCGAAAAATGGAAAATCCCTATAAATTTCAACTTACTTC
GAAGTCTAAAACCGTTCATCAAGCGTTAGGTTATGACATTACAGCAATTGTTGATATTGGTAGGGCACTAATAGAAGCCAAAGATAATGACGATCTACCACAATCACGGTTAAAGGCAGCCGC
CGCAGCACAGAGACTTATTAATGCCTCCGCTAAGGCGGGAATTAAGGGGGTTGCGTATGCGCTTGCTGGTTATCGTCCAGAAGTTCAGGCTGTCATTGACGAGTTCAAAGCGTTTGTTCGT
GAAGAGGCTCGTCAATATGAAAAGGAATTTCCAGATGAGCTATACGAGGAGTGGTATCGACTGTACGGCCTGAATAGGCCAGAGAAAGGACGGCCTATTCGTTTTGGGCAGCTAACCAAC
ATGCAGATATACACCCCGCTAGCAAAGAGTAAAGGTAAAATCCTTGAACAAATTCGAGCCAGCCGAGACGAGAACGGAAAACAATCTGATAAGTTGCATCTGTTCCTTTCTGAAATTGGTGT
CAAGGCTTTGCGTCAGCATATCGGTAAGCTTCTTGGTGTCGCAGCGATGAGTGAGACAAGAGAAGAATACGAAAAAGGAATAGAAAAGGTTTTCGGAAGAATGAAACCAGAAATCTAA
>PNPANE_15660 DUF4359 domain-containing protein
GTGAAGCGATGGGTTATAGGTGCATTCTGTTTTCTAATTAGTGGTCTTGCACAATCGCAAGATAAGGATCTGAAATTTGCTAACGACATGTTAGTTACAGCTAAAGTTGCTGGTATGTGTGGA
ACATTTAAACAAATGTTTGCTTTTCAAGAAGCAACCCAGATGCCAGGCGGAGATGAATTTATTGAACGTTTTTTGAATACGGAAATTTCTCGCCTAGGAATGTCACTTCAAGAGTTCATGAAG
TTGTGCACAGATTCTATAGAGTCATACAATAAATTAAAAAGGATGTCAGAATAG
>PNPANE_15665 DNA transfer protein
ATGGCAACCAATGTAGGTTTACCAGAGGGTTTTGTTCTGGATGAACAGCCTGATAACTCACAACTTCCTGATGGCTTTGTGCTTGATTCCCAACCAGAACAGCAGCAATCTCCTTTGGTTTCA
CCAGAGGAAAATTCCAGACAGGAAAATGTTGTTAATAATGCTAACGGCTTCGACCGTTTTATGTATGGCGTTCTCAGTGGATTGATGGATGTTGGTAAAGGTGTTGGTCTATTTCAGGATATG
ACACCAGAAGAGCAAGCCGCAATTCAGTCTCTACAGCAGAAGTTAGCGGCAAAACCATCAACCGCACAAGATGTTGGTGAGTTCGTTGGACAAGCAGCACCATTTGTTAGTGGTGGTGGG
ATTATTTCTCAGGTTCCGAAAGGGGCGGCAAGGCTGGCTGCCGCCGCAGGGCTTGGTGCGGTTGAAGGTGGTGTTGTTGCTAACGGAACCGGTGGCAATGTAACTACAGGGGCGGCTAT
AGGTGCTGCCGCTGGTCCAATTGCTGAATTGGCATCTCCAGTGATTAGCAAAGCAGCCAGTGCGGTTACGAAGAGGTTTAGGCCTCCATTGGCAGAAATGTCTCAGGCATCTGAACTTAGT
GGAATGGCGAGGGTTGCAACGAAAGCTACAGATGTGAATCCAATAACAGGAAACAGATCTGGTTTAAATGCTCTCGCGGAAGCTGTTTCACCAGATGATTCTGTGCTACAGGCCGCCCGCA
ATCTTGGGATGGAGGATGCCTTAACTCCCGGCATGTACTCCAGCAACCCTTCTTACCGCGCGTTTGAAAATGCTTTAGCTGCAACCCCAGGGAATAAACTGTTCCACGCACAGCGAGAGGCC
ATTAGTAGGCTTGGAGAGCAGGCAGATAAATTTATTAATGATTTTGGTGGGAGCGTTGATAAGGACTTTATCAATCAAAAGGTAAAAAGCTCTTATGAGGAGTTAAGATCAAACCTGAAGCG
CCAAGAAGACACTCTATACAATCAAATTAGAGCAAAAATTCCAACAAGAGCTACCGTAGATACCACCCATACCACTAACGCCATTGAGGATATTGCAGATGATGTCGGTGGGCTAGAAAAACT
AAAATCGCTATATCCTCAACTGTCAAAGACCTTGGAACAAATAGACCCAAACACCGCCCCGACTTATGGTCTTCTTGATGCAGTTCGACGACAGGTTGGAAGGGCTCTTGGCAGAGAGATG
GATAAAGGGCCATATAGCGGCATCGACGCTCGGCACCTTGGAATTATTGAAAGCGCACTGATACAAGATCAGGGAGCCGCTGCTGCCAAATATGGTGCAGAGGATACATGGAATTTAGCTA
GAGAGATCGGGAGAAAGAGATTCGCAGTACAGGCAGCCGCTGTTCAAAATCTTGGCAGAGACTTGGATAAAGGAATAGTGCCGCAATTACAGCAAGCTATCGTCGATATGTCTCAAGGAA
AGGGCGGTGACTTCCGTCGCCTAGTTTCAAATCTTCCTGATGACATGGTGCAACCAGCTGTTGCCACCGCAATGAATAGGGCTTTCACTACGTTCGCTAAATCTCCCGGGCAGTCACTTGGT
GTTCCTGGATTTGTTAAGTGGTATAAAGGGCTATCAAGAAACAAATCAAATATGCAGGCATTGAGCCGGGCAATTGGCTATCCGGCTACGCGTAGGTTGAGAAACATCTATGAGGTGGCTTC
TGGCATGCAAAGAGTGGGCAGCGAGAAGGTGTATTCCTCCTCTCAAATTGACCGGATGATGAATCAGTTCGCAGGAGAGAAGGGAATGCTTGGGCGTATATATGGAATAGGAAAAGACGT
GGCTAAAGCAGAAGGGCTAACCACAGCCATCGGAGCGCCTGGAGCCGGTACGGCAGGGGTACTCTTTAGCCTCCTCCGTTCTGGTAAAAACTCAAGAATGGCTGCCGCTGATGAATTAATA
GCATCTCCGGCTTTTAAAAATCTCTCAAAAAGGATTGCCGCAGGTCACGCCAGCACCCCAGCCTCTAGGAAATCGGTGGATGCTTCAATGCAGAAAGTGAAACCATATAGGGATTGGGTAA
ATACGCTATCAGCGGACGAGAAGAAAAATCTAGCCCGCGTTGGTATTGTCGCTTTTCTCAGTAGTAACGATTCTTTGCCCACAGAGCAACAAGCAGGGCAACAATAA
>PNPANE_15670 Gp protein
ATGGCTACTTGGCAACAGGGTATTAATTCTGGTGGTTTTCTGGCTGGCATCGGTACGCAAAATGAGAATGCGCCAAAGGCAAGCGACATTAACGCAACGCTTGGTCTGATTCGCGAAAACA
ATGAACTGGCTCGCTCAGGTGCAAATAACGTTGGTCTGACCGCGTTACGTGGTCTGGCTGGAGTTGCTGATATTTACAATCAGGAACAGCAACAGAAAGCTATTAGTGCGTTCAATAAGGTT

CACGCTGATGCATGGGCTTCTGGTGATCCATCGGGACTATTTAAGTTTGCCCAGGAAAATCCAGCGTTTGTTGCACAGGCACAACAGGCGTTTTCCGGTCTTAATGATCAGCAACGCAACGA
TATGGGCGATTTAGCCATGAGGGCTAACGTCGCTCTTTCTCAGGGACCGGAAGCCTACAGTAAATTCATTACTGACAACAAGGACAGGTTAAATCGCGTTGGTGCTAATGCTGACTGGATGA
TTCAGACAGGTATCCAGAATCCAGAGCAGCTATCACACATGCTGACCACTATGACGCTAGGGGCTGTTGGTCCGGATAAAATGCTGGATTATCAGGATAAGATGGTTGGTCGCCAACTTGAG
AAAGGGCGATTGGATGAAAGCATCCGTCAGGCTGACATGGAGAACGCGAGAGGATGGGCAAATATCCAAAACGCTCAACTAGACAGGGCTCAGCGGGCACAAATGCATAATGATAACGTA
GCCCTGAAGTTGCAGGAATTAGGGATGAAACAACAGGAAAGCGGAAAGATTGACCCAAAACTTGTTCGAGATCTGAATAGTGATATTAATGGGTTCTCAAAGAATTATTCTGCAATGCGCA
GTGCTTCTGACAACCTACAAGCCCTTGGGAAGCGCAACACTCCAGCCGCGCAGTTGGGAATGATTTTCAACTATATGAAATCGCTGGATCCGCAATCTGTGGTACGCGAAGGTGAGCAAGT
GCAGGTAAAACGCACTGATGGAATATTCGGCACACTTGGTAACTATGTTAGCCAATTATCTAACGGCAAGATGCTGAATAATGAGCAAGTCCAAGACTTAATCAACACCTCAAAACTGATGGC
AAATACTGAAGGCGAAAAGTTTAACCAGCAAATGGATGATTATCTTTCAACTTATGGAGATTCTCTCCCCAGCGGACTAACTAAGCAATTGCAATCCAGAAAAGCCAAGCTGTATGAAGATAT
TCAGCAGCCTGCGCAACAACAGACACAACAAGCAACATCTGGCGGTCAAACATTTCGAGAAGGTATGACCGCGAAAAATCCTAAAACTGGTCAGAAAATTATTTACAGGAACGGACAATG
GCAACCAATGTAG
>PNPANE_15675 Internal virion protein gp7
ATGTTATATGCATTTAAGCTGGGCAGAAAACTGCGCGGCGAGGAACCTTATTGCCCTGAAAAAGGCGGGAAAGGTGGTAGCTCTGATAAAAGCGCAAAGTATGCCGCAGAAGCTCAGAAG
TATGCAGCAGACCTGCAAAATCAGCAGTTCAACACCATCATGAACAACCTGAAGCCGTTTACTCCTCTGGCTGATAAGTATGTCGGCAGCCTCGAGAACTTATCGTCTCTGGAAGGGCAAGG
TCAGGCACTTAACCAGTATTACAACTCTCAGCAGTACAAAGATCTTGCTGGTCAGGCTCGCTATCAGAGTCTGGCGGCAGCGGAAGCAACAGGTGGATTGGGTTCCACTGCAACCGGTAAT
CAGTTATCAACAATCGCACCAACGCTTGGTCAGCAGTGGCTGTCTGGTCAGATGAACAACTACCAGAATCTGGCAAACGTTGGGCTTGGTGCGCTGCAAGGTCAGGCAAACGCCGGGCAG
ACGTACGCCAACAACATGATCAGCATTGCACAGCAAAGCGCAGCTCTCGCCGCTGCTAATGCCAATAAGCCATCCGGTCTTCAGACAGCAATTAGCGGAGGAGCTTCAGGAGCTATGACTG
GTGCCGCTCTTGGTTCTATTGTTCCCGGTCTTGGTACTGGATTAGGCGCCGCAATCGGTGGCGGACTTGGCCTGCTTGGCTCGTTGTTTTAA
>PNPANE_15680 Protein gp14
ATGATTACATTCACTCCAACACGCAACATCGACCTGATAGAAATGGTTGGCAACCACCCCGACATCATTGCCGGAAGCAACAACGGTGACGGATACGACTACAAGCCTGAGTGTCGTTACTT
TGAAGTGAACGTACATGGTCAGTTCGGTGGCATCGTGTATTACAACGAGATTCAGCCGATGACCTTTGACTGCCACGCCATGTACCTGCCTGAGATTCGCGGATTCAGTAAGGAAATCGGAC
TGGCGTTCTGGCGATACATTCTCACCAATACCACCGTTCAGTGCGTTACATCATTTGCTGCACGCAAATTTCGCCACGGGCAGATTTACTGCGCAATGATTGGCCTTAAGCGTGTAGGAACCA
TCAAGAAATACTTCAAAGGCGTGGATGACGTGACGTTTTACAGCGCCACACGCGAAGAACTAATCGACTTCCTGAATCACGGGAGATAG
>PNPANE_15685 Phage tail protein
ATGGCAGACCCGTCACTTAATAAGCCTGTCGTGGTTCAGGCTACACGCATTGATGCTTCAATTCTTCCCCGTAACGTCTTTAGCCAGTCTTATTTGCTCTATGTAATCGCGCAGGGAGCTGACG
TTGGTGCTATTGCAGGCAAGGCAAATGAGGCAGGGAGTGGCGCTTACGACGCACAGGTTAAAAATGATGAGCAGGATGTAATTCTTGATGAGCACGAAAAAAGAATTGCAAAAACAGAA
GAGGATATTTCAGGAATAAAAGTAAAGCTTCTTGAAATAGAGAATGATGTTAATGGTCTGAAAATAAAAGTTGAGGATATCGACGGTAAAGTATCAGAGATAATCGTTGATTATGTTTCACTC
AGCAGAACAGGAACTCAAACTCTTGCCTCATCCCTTAACGTATCAGGAAGTTATTCTGTTAACGGTACAAAAGTTGTTGGCGCTCGCCAGACTGGATGGACCGCGGCAACAGGTACGGCGA
ATAAAGGCGTATTCGATGCTGACCTGACATTCGCCGTTAGCGATACTTACACGCAATCTGAAATCCAGGCTATAGCCAATGCTCTAATTACTGAGCGTCGGCGCACTAAGGCTTTGGAAGACG
CCTTGCGTGCACATGGGTTGATTGATTAA
>PNPANE_15690 Phage head DNA stabilization protein
ATGCCGATTCAACAACTCCCCATGATGAAGGGAATGGGTAAAGACTTCAAGAACGCCGATTATATCGACTATCTGCCAGTGAATATGCTGGCAACACCCAAAGAAATCCTTAACAGCAGCGG
CTATCTCCGCTCATTCCCTGGCATTACCAAACGTTATGATATGAACGGCGTATCGCGTGGAGTTGAGTACAACACCGCTCAGAATGCTGTTTATCGTGTTTGTGGTGGCAAGCTCTACAAAGG
AGAAAGCGAGGTTGGTGATGTTGCCGGAAGTGGTCGCGTATCAATGGCACATGGTCGCACATCACAGGCGGTAGGCGTTAATGGTCAACTGGTCGAGTATCGCTATGATGGTACGGTTAAA
ACCGTCTCAAACTGGCCTGCAGGCAGCGGATTCACGCAGTATGAGTTAGGCTCGGTCCGCGACATTACGCGCTTACGTGGGCGTTATGCGTGGTCAAAAGACGGAACTGATTCATGGTTTG
TCACTGACCTTGAAGACGAATCGCATCCTGACCGATACAGTGCACAATATCGCGCAGAATCGCAGCCGGACGGCATCATCGGTATCGGGACATGGCGAGACTTCATCGTCTGCTTTGGTTCG
TCAACGATAGAGTATTTCTCTCTGACAGGCGCAACCACCGCTGGCGCTGCGTTGTATGTCGCACAGCCATCGCTGATGGTACAGAAGGGCATTGCCGGAACATACTGTAAAACGCCATTCGC
TGATTCATATGCATTCATCAGTCACCCGGCTACTGGCGCACCTTCCGTCTACATCATCGGGTCAGGGCAGGCTTCACCAATTGCGACGGCCAGTATTGAGAAAATTATCCGCTCATACACGGCT
GATGAACTGGCGACGGGTGTAATGGAGACTTTGCGCTTCGATTCTCATGAGCTTCTGATTATTCATCTCCCTCGTCATGTTCTGGTTTACGACGCATCGTCAAGTCAGAACGGACCACAATGG
TGCGTGCTGAAAACCGGGCTTTACGATGATGTATATCGTGCTGTCGACTTCATGTATGAAGGCAACCAGATTACGTGCGGCGATAAATCAGAAGCGGTGACAGGACAACTGCAATTTGACAT
TAGTAGCCAGTACGAAAAACAACAAGAGTTCATCTTGTTCACGCCAATCTTCAAAGCAGAGAACGCCAGATGCTTCGACCTCGAAGTTGAATCATCCACTGGTGTTGCTCAATACGCCGACC
GCCTGTTCCTGTCTGCAACAACTGACGGTATCAATTACGGTCGTGAACAGATGATTGAGCAGAACGAGCCGTTTGTGTACGACAAGCGCGTTTTATGGAAACGTGTTGGGCGCATTCGTCG
ATTAATCGGATTCAAACTGCGGGTAATCACCAAATCGCCAGTAACACTATCCGGGTGTCAAATTCGTCTGGAGTAA
>PNPANE_15695 hypothetical protein
ATGCAGATAAAGACTAAAGGCGATCTGGTCAGGGCTGCGCTTCGTAAGTTGGGCGTGGCATCAGATGCAACCCTTACCGATGTCGAACCTCAGTCTATGCAGGATGCCGTAGATGACCTCG
AAGCGATGATGGCGGAGTGGTATCAGGACGGGAAAGGCATCATTACCGGCTATGTATTCTCAGATGATGACAATCCTCCTGCTGAAGGTGACGACCACGGCCTTCGCTCTAGTGCAGTCAG
CGCGGTATTCCACAATCTGGCTTGCCGGATTGCCCCGGATTATGCGCTTGAGGCTACTGCCAAAATTATCGCCACTGCTAAATATGGGAAGGAGCTTCTCTATAAGCAGACCGCCATCGCCAG
AGCAAAAAGAGCTCCTTACCCGTCACGCATGCCAACAGGCAGCGGTAATAGTTTCGCCAATCTGAACGAATGGCATTATTTCCCCGGAGAGCAGAATGCCGATTCAACAACTCCCCATGATG
AAGGGAATGGGTAA
>PNPANE_15700 hypothetical protein
ATGAAAATAGCAATCTATAAGCCCGGTGGAAGCATCATGGTATGGGGCGTCATGGCTCAGATGAAGGTCATCGACTCCAGCGAACTTCCGGAATATGTCAAAGATGGCTGGCTTGATCATCC
GTCAAAGTTGCTTCCCGCGGAAGCAGATGATGTTAAGCCACGCAAAGGCCGCAAGCCTAAGGCGGTAAGCGATGCAGATAAAGACTAA
>PNPANE_15705 Major capsid protein
ATGGCTTTGAACGAAGGTCAAATTGTTACACTGGCGGTAGATGAAATCATCGAAACCATCTCCGCAATCACTCCAATGGCGCAGAAAGCCAAGAAATACACCCCGCCTGCTGCTTCTATGCA
GCGCTCCAGCAATACCATCTGGATGCCTGTAGAGCAAGAGTCACCCACTCAGGAGGGCTGGGATTTAACTGATAAAGCGACAGGGTTACTGGAACTTAACGTCGCGGTAAACATGGGAGA
GCCGGATAACGACTTCTTCCAGTTGCGTGCTGATGACTTGCGAGACGAAACTGCGTATCGTCGCCGCATCCAGTCTGCCGCTCGCAAGCTGGCGAACAACGTTGAGTTGAAAGTCGCAAAC
ATGGCCGCCGAGATGGGTTCGCTGGTTATCACCTCCCCTGATGCCATCGGCACTAATACCGCAGACGCCTGGAACTTTGTGGCCGACGCAGAAGAAATCATGTTCTCCCGCGAACTTAACCG
CGACATGGGGACATCGTACTTCTTCAACCCTCAGGACTACAAAAAAGCGGGTTACGACCTGACCAAGCGTGACATCTTTGGGCGTATTCCTGAAGAAGCATACCGAGATGGCACCATTCAG
CGGCAGGTTGCAGGCTTCGATGATGTCCTGCGCTCTCCGAAACTTCCTGTGCTGACCAAATCTACTGCAACTGGTATCACTGTATCCGGTGCTCAGTCCTTCAAGCCTGTCGCATGGCAACTG
GATAACGATGGCAACAAAGTTAACGTTGATAACCGTTTTGCTACCGTCACCCTGTCTGCAACTACCGGCCTGAAACGCGGCGACAAAATTTCGTTTGCTGGCGTGAAGTTCCTTGGTCAGAT
GGCTAAGAACGTACTGGCGCAGGACGCGACTTTCTCCGTAGTTCGCGTTGTTGATGGTACTCACGTTGAAATCACGCCGAAGCCTGTAGCACTGGATGATGTTTCTCTTTCTCCTGAGCAAC
GGGCATACGCCAATGTTAACACCTCGCTGGCTGATGCGATGGCGGTGAACATCCTGAACGTTAAAGACGCTCGCACTAATGTGTTCTGGGCTGACGATGCTATTCGTATCGTGTCTCAGCCG
ATTCCGGCTAACCATGAACTTTTTGCAGGTATGAAAACTACCTCATTCAGCATCCCTGATGTTGGCCTGAACGGTATCTTCGCTACGCAGGGTGATATTTCCACCCTGTCCGGCCTGTGCCGTA
TTGCGCTGTGGTACGGCGTAAACGCGACACGACCGGAGGCAATCGGTGTTGGCCTGCCTGGTCAGACTGCGTAA
>PNPANE_15710 Scaffolding protein
ATGGAACCAACCACCGAAATTCAGGCAACTGAAGACTTAACCCTGTCCGGCGATCATGCAGCGGCATCTGCTGATAGCTTAGTTGTCGATAATGCCAACGACAATGCAGGTCAGGAAGAGG
GCTTTGAGATTGTCCTGAAGGACGATGAGACAGCACCAAAACAAGACCCGGCAAAGAACGCAGAATTCGCCCGCCGCCGCATCGAGCGCAAACGACAGCGCGAGCTTGAGCAGCAGAT
GGAGGCAGTTAAACGCGGAGAATTGCCGGAGAGTTTACGGGTAAACCCTGACCTTCCACCTCAGCCGGATATTAATGCCTATCTGTCAGAAGAAGGCCTGGCTAAATATGACTACGACAAC
AGCCGTGCGCTTGCCGCTTTCAATGCTGCTAATACCGAATGGCTAATGAAAGCGCAGGACGCCCGCAGCAATGCCGTAGCAGAACAGGGCCGCAAGACTCAGGAGTTTACCCAGCAATCA
GCGCAATACGTCGAAGCTGCCCGCAAACACTATGACGCGGCGGAAAAGCTCAACATCCCTGACTATCAGGAGAAAGAAGACGCATTTATGCAACTGGTTCCGCCTGCGGTTGGGGCCGAC
ATTATGCGCCTGTTCCCGGAAAAGTCCGCCGCGCTCATGTATCACCTGGGGGCAAACCCGGAGAAAGCCCGCCAGTTACTGGCGATGGATGGGCAGTCCGCGCTGATTGAACTCACTCGAC

TATCCGAACGCTTAACTCTCAAGCCTCGCGGTAAACAAATCTCTTCCGCTCCCCCTGCTGACCAGCCGATTACCGGTGATGTCAGCGCAGCAAATAAAGATGCCATTCGTAAACAAATGGATG
CTGCTGCGAGCAAGGGAGATGTGGAAACCTACCGCAAGCTAAAGGCAAAACTTAAAGGAATCCGATAA
>PNPANE_15715 Portal protein
ATGGCCGACAATGAAAACAGGCTGGAGAGTATCCTGTCGCGCTTTGATGCGGACTGGACAGCCAGCGATGAAGCCAGAAGGGAGGCCAAGAATGATCTCTTCTTCTCCCGTGTATCTCAGT
GGGATGACTGGCTATCACAATACACAACCCTGCAGTATCGCGGGCAGTTCGATGTTGTACGTCCAGTGGTGCGCAAGCTCGTTTCTGAGATGCGTCAGAACCCTATTGATGTTCTGTATCGTC
CAAAGGACGGAGCAAGCCCTGATGCCGCTGATGTGCTTATGGGTATGTATCGCACAGACATGCGACACAATACGGCAAAAATCGCGGTCAACGTCGCTGTTCGTGAGCAGATTGAAGCAG
GTGTAGGTGCGTGGCGTCTGGTCACTGACTACGAAGACCAAAGTCCAACGAGCAACAATCAGGTTATCCGTCGAGAGCCTATCCATAGTGCCTGTTCCCATGTTATATGGGACAGCAACAGC
AAGCTGATGGATAAGTCTGATGCCCGTCACTGCACAGTTATCCACTCAATGAGCCAGAATGGTTGGGAGGATTTCGCAGAAAAATACGACCTCGATGCGGATGATATTCCATCATTCCAGAAC
CCCAACGATTGGGTATTTCCATGGCTGACGCAGGACACAATTCAGATCGCTGAGTTTTACGAAGTGGTCGAGAAGAAAGAGACGGCGTTTATCTACCAAGACCCGGTTACGGGTGAGCCG
GTAAGCTACTTTAAGCGCGATATTAAAGACGTCATCGATGACCTGGCTGATAGTGGATTTATCAAAATTGCAGAGCGCCAGATTAAGCGTCGCCGGGTATACAAATCGATTATCACCTGCACT
GCTGTACTCAAAGACAAGCAGCTCATTGCTGGCGAGCATATCCCCATTGTTCCGGTGTTCGGAGAGTGGGGCTTCGTTGAAGATAAAGAAGTGTATGAGGGTGTCGTCCGCCTGACAAAAG
ACGGCCAGCGTCTGCGCAACATGATTATGTCGTTCAACGCCGACATCGTGGCCCGCACTCCGAAGAAGAAGCCGTTCTTCTGGCCTGAGCAGATTGCAGGCTTTGAGCATATGTACGACGG
TAACGACGATTACCCATACTACCTGCTCAATCGCACTGACGAAAATAGTGGAGACCTTCCGACTCAGCCGCTGGCATATTATGAAAACCCGGAAGTGCCGCAAGCCAACGCCTACATGCTGG
AAGCAGCAACCAGCGCAGTAAAAGAGGTTGCCACTCTCGGAGTTGATACAGAAGCGGTAAATGGCGGACAGGTTGCGTTTGATACCGTCAATCAACTGAATATGAGGGCTGACCTTGAGA
CATACGTGTTTCAGGATAATCTGGCTACCGCCATGCGCCGTGACGGAGAGATTTACCAGTCGATAGTTAATGACATCTACGATGTTCCTCGCAACGTTACGATTACCCTTGAGGATGGCAGCG
AGAAAGATGTTCAGCTAATGGCTGAGGTTGTTGACCTTGCTACTGGAGAAAAGCAGGTACTAAACGATATCAGGGGGCGCTATGAGTGCTACACGGATGTTGGACCATCATTCCAGTCCAT
GAAGCAGCAAAACCGCGCAGAAATTCTTGAGTTGCTCGGCAAGACGCCACAGGGAACGCCAGAATATCAACTGCTGTTGCTTCAGTACTTCACCCTGCTTGATGGTAAAGGTGTTGAGATG
ATGCGTGACTATGCCAACAAGCAGCTTATTCAGATGGGCGTTAAGAAGCCAGAAACGCCCGAAGAGCAGCAATGGTTAGTAGAGGCGCAACAAGCCAAACAAGGTCAACAAGACCCGGC
AATGGTTCAGGCTCAGGGCGTACTCCTGCAGGGGCAGGCTGAACTGGCTAAAGCTCAGAACCAGACGCTGTCCCTGCAAATCGATGCAGCTAAAGTCGAAGCGCAGAACCAGCTTAACG
CTGCCAGAATCGCAGAAATCTTCAACAACATGGACCTCAGTAAACAATCTGAGTTTAGAGAGTTCCTTAAAACCGTTGCTTCATTCCAGCAGGACCGCAGCGAAGACGCTCGCGCAAATGC
TGAGTTACTCCTTAAAGGCGATGAACAGACGCACAAGCAGCGAATGGACATTGCCAACATCCTGCAATCGCAGAGACAAAATCAACCTTCCGGCAGTGTAGCCGAGACACCTCAATAA
>PNPANE_15720 Terminase
ATGTCATCAGAATTTGAGGCAATGCTTGCCGACGATATGGGGCGATTCTTCTATGACCCACTCGGATTTGTGATGTATGCATTTGAGTGGGGAGCTGGCGATCTTGATGGCTTCGACGGTCCA
GATGAGTGGCAGAAAGAGTTTCTCACTGATTGGGGTGAAGCAATTCGCACTAACAACTTTGATGGTGTAAAGCCGGTAGAAGCATACCGATGCGCTACAAGTTCCGGCCACGGTATCGGG
AAAAGTGCGCTTACCGCCTGGGTAATTCTCTACATCATGAGCACTCGCCCATTCTGTAAAGGCGTCGTAACTGCCAATACATCAGAGCAGCTTCGCACCAAAACGTGGGGCGAGCTTGGAAA
ATGGAAGAAGCGTTGTATCACCGGGCACTGGTTCGAGTACAACAACGGAAAAGGCAACATGAACATATACCATGTAGATCACATGGAGTCATGGCGTTGTGACGGGCAAACCTGCCGTGA
GGAAAACAGCGAATCATTTGCAGGACTGCACTCCGCTAACTCAAGCCCGTTCTATATATTCGATGAAGCCTCAGCGGTGCCAGACAAGATATGGGAAGTCGCAGAAGGCGGACTGACTGAC
GGCGAACCTTTCTGGTTCGCTTTCGGCAACCCGACACGTAACACCGGACGATTCCGTGAGTGCTTCCGTAAATTCAAGCATCGCTGGCGTCGGCGTCAGATTGACAGCCGAAAAGCTAAGA
TGACAAACAAAGAGCTTATCGAAGAATGGCGCAACGACTACGGCGAGGATAGCGACTTCTTCAAAGTGCGCGTTCGTGGCCTCTTCCCGTCTGCTTCAGACCTTCAGTTTATTCCTCAAAGC
TATGCCGATGCTGGGATGTCCAGAAAGCTGGAGCACAGTCAGTATGGATTCGCTCCAAAGATTATCGGCGTTGACCCGGCATACTCTGGGAGCGACGAGGCATGTATCTATCTCAGGCAAG
GCCTGCACTCAAAGCTTTTAGGTTCCTACCCAAAAACAGACGACGATGTGAAATTTGCTCAGGTTGTTGCTGCTATCGAGGATGAACATAAAGCTGACGCGGTATTCATCGATTTCGGTTAC
GGAACGGGTATTCATTCTGTAGGTAAGTCATGGGGAAGAAAATGGCAACTTGTAAGCTTCGCAGGTGAATCGAAAGACCCGGCAATGCTCAACAAGCGCGGAGAAATGTGGAATGCAATG
AAATCCTGGCTGAATGAGGGAGGCAGCATTGACGACCAGCAGACCGCCGATGAGATTGTCGCCCCAGAATACAAAGTGAAGCTGGACGGTAAGATTGTTCTTGAGTCGAAAGACGACATG
AAACGCCGCGGTGTTCCCTCGCCAAACAGGGCCGATGCGCTGGCGCTGACATTTGCATTCCCGGTAGTCAAAAACAAACCTTCAAAAGCAATCCCCGCACCGATTAGACCAGTACGCAGAG
GACGATAA
>PNPANE_15725 Terminase
ATGGCGGCTGAAGATAAGAAAATTGGTCGCCCATCGGCTTACAAACCAGAGTATGCCGAGCAGGCGAGAAAACTTTGTCTGTTAGGGCATACAGATGCGGAACTGGCTTCTTTCTTTGATG
TTAGCGAGCAAACAATCAATGCGTGGAAGCACGCGCATCCTGATTTTCTTGAGTCCATAAAAAAGGGTAAGGCTGTTGCGGACAGTGAAGTCGCCGCAAAGTTATTCCACCGCGCCACCGG
GTACGAACACCCAGAAGATGATATTCGCGCTGTCGATGGTTCGATCGTCATAACTCCTACTGTGAAACATTATCCACCCGATACGACTGCTGCCATTTTCTGGCTTAAGAACAGGCAGCGAGA
TAAATGGCGTGACAAACAGGAAGTAGAACACACCGGAGAGGTTAGCCTGATTCAGCGCATTCAGGAGGCCCGTAAACGCGCAAGGGGTGAGTGA
>PNPANE_15730 Decoration protein
ATGGCAAATCCAAATTTCACGCCATCCTGGCCTCTCTACAAAGATGCTGACGGTGTATATGTGTCTGCTCTTCCGATTAAAGCTATCAAATACGCTAATGACGGAAGTGCAAACGCAGAATTCG
ACGGTCCGTATGCTGACCAGTACATGTCAGCGCAAACAGTAGCCGTATTCAAGCCGGAGGTCGGTGGATATCTGTTCCGAAGCCAGTACGGAGAGCTGCTCTATATGAGCAAGGCCGCATTT
GAAGCTAAGTACACTTCTGCAAGCGGTTCAGTAACGAATGCAGAGACGGCGGATAAGTTATCTACTGCTCGCACTATCTCACTAACTGGCGCGGTCACAGGTTCGACGTCATTTGATGGTTC
GGCTAACGTGACTATCGCAACTACCCAAGGAAGCTAA
>PNPANE_15735 Uncharacterized protein in gp15-gp3 intergenic region
ATGGCAGAGATTATTTCCATGACTGAAGAACAGAAATTCCAGTTAGAGATTTACAAACTGGTCATGAACCAGAACGCAGCCGCAGAGGAAGCATTTCAATTCATTGGCACTGACGAGCTGA
AGCTTGAACTATTCAAAATTCACTTCCAGTCAGGCGGCGCTAATTCGGATATCACGATCCGCACATTCGAAGCAGTGCGTAAATCTAAGGAAGCGTTAGACCTGTTCACCACCGGAGCATAA
>PNPANE_15740 DUF2829 domain-containing protein
ATGACCCAGCATATTGGCGTAAAACTGATTAACGCCTTTCCGATGACGAGACAGGCATATAACGATTTTCGTGGCTGGCAGCTTCCAGCCGGAGAAAACGGCGAGGACGAAGGCTATCTGG
TTGAATATCTGGATGGCGGAAAACCTAACACCGATCGCTTTGATGGCTACGTTATCTGGAGTCCAAAAGAAGTATTCGAAAAGGCTTATCGTCCGGTATCAGGGCTAAGTTTCGGCCTTGCC
ATTGAAGCGCTCAAGTTGGGGAATAAAGTTGCCCGTGCTGGCTGGAATGGTAAGGGTATGTGGCTGGCATACGTTAAACCGTACACTGAGGCTGTTCATACTGGCAGTACGCCTTGCTTTTG
CAGTCGCGTCTTTGAGTTGCCTGAAGGTACGCATGGAGAACCGAAACGATCTCCGAAACAACTTCCGTATATCGCCATGAAAACAGCGGACGATAAATTAGTGCCGTGGCTGGCTAGTCAG
ACTGATGTTCTAGCAGAAGACTGGCAAATCATTACCATGTAG
>PNPANE_15745 Outer membrane lipoprotein Rz1
ATGCTAACGCGACTATCGAAAGTCTCCGTGCTGATGTTTCTGCTGGTCGTAAGCGCCTGCAAGTCGCCGCCACCTGTGCAAAGTCAACGACCGGAGCCAGCGGCATGGGCGATGGAGAAA
GCCCAAGACTTACAGCAGATGCTGAACTCAATTATTACCGTCTCCGAAGTGGAATCGACAGGATAA
>PNPANE_15750 hypothetical protein
ATGAGCATGATTTGCTTTTTCATGGCAGCGTTGCTCGCATTGAATGGCAACGATGCGTGGCCGTGGTTTCTGGCCGTTGGGGTGTTGATGTCATGA
>PNPANE_15755 lysozyme RrrD
ATGGCAATGTCACCGGCACTACGAAATAGCGTAATAGCGGCGATAAGTGGCGGGGCTATTGCTATAGCATCTGTGTTAATCACTGGACCGAGTGGTAACGATGGTCTGGAAGGTGTCAGCTA
CATACCATACAAAGATATTGTTGGTGTATGGACTGTATGCCACGGACACACCGGAAAAGACATCATGCTCGGTAAAACGTATACCGAAGCAGAATGCAAAGCCCTCCTGAATAAAGACCTTG
CCACGGTCGCCAGACAAATTAACCCGTACATCAAAGTCGATATACCGGAAACAACGCGCGGCGCTCTTTACTCGTTCGTCTATAACGTGGGCACAGGCAATTTCAGAACATCGACGCTTCTTC
GCAAAATAAACCAGGGCGATATCAAGGGAGCATGTGATCAGCTACGGCGCTGGACATACGCTGGCGGTAAGCAATGGAAAGGGCTGATGACCCGTCGTGAGATTGAGCGTGAAGTCTGTT
TGTGGGGGCAACAATGA
>PNPANE_15760 Holin
ATGTACCGTATGGACAAAATCAGAGAATGGTTCAGTTACAGCTTCGGAGGACTGACTGCGATGGGTGGCATTCTCTCCCTGAATGACTGGGCTGTCATCATTGGTATTCTTTGTACTGTCGGC
ACATTTGGCATCAACTGGTACTACAAGCGCAAAGAGCGCGAGGACAGATTGAATGGCAATGTCACCGGCACTACGAAATAG
>PNPANE_15765 tRNA-Thr(tgt)
GCTGGTTTAGCTCTAATGGTAGAGCAGTCGCCTTGTAAGCGAATGGGTAGCGGTTCAAGTCCGTTAACCAGCACCA

>PNPANE_15770 tRNA-Asn(gtt)
GGGTCAGTCGTATAAAGGTGATTACGGAAGGCTGTTAACCTTCTTATCGTGGTTCGAGTCCACGCTGTCCCGCCA
>PNPANE_15775 Antiterminator
GTGGGCATAAGAGAACTAAACCTCACCAAAGAACAGCATGAGTGGCTGAATGGCTGGCTTGAACTGTGGGGCGCATGGGTTTATTCAGGTCGTCTGGAAAAGCGCATGAGCAGCGTAATA
GCGAAGTTCATGGAGAGCGTAGAGCCGGGAAGAGTTATGACAAGGCCAATGTGCAATGATGATGATGGAATGTTGATTTCTCAGGTCGTCGATTCCGTCATGTGCATTGACAAGAAAGCCT
TTGGCATCCTCCTCAGCTACTACGCTCATGGTTCATCTAAGCGAGCAATTGCATCCTACTATCACGCGACTGCAAAGCCACGCAAGATGTGTGGACGTGGTGGCGAGGGATGGAGAAAACCT
TCACTGGCAACCTGTAGAAACGAAATTGACGACATCCTGAAAGCATCGTTATTTGTTTTGTACCAGCCAATGCAAAATGCTTTCAAAATGCGTAAACGTGTTGAGAAAGTTAAGCATGTTGCT
GTTAAAAGCCTTGACATGCAATTATCCATTTAG
>PNPANE_15780 Serine/threonine-protein phosphatase
ATGAATATCTACGAAAGAATTGATGGCAGCAAATACCGAAATATTTGGGTAGCTGGCGACCTGCACGGATGCTACACGAACCTTATGAACAAACTGGATACGATTGGATTCGACAACAAAAA
AGACCTGCTTATCTCGGTGGGCGATTTGGTTGATCGTGGTGCAGAGAACGTTGAATGCCTGGAATTAATCACATTCCCCTGGTTCAGAGCTGTACGTGGAAACCATGAGCACATGATGATTG
ATGGCTTATCAGAGCGTGGAAACGTCAATCACTGGCTGCTTAATGGCGGTGTCTGGTTCTTTAATCTCGATTACGACAAAGAAATTCTGGCTAAAGCTCTTGCCCATAAAGCAGAAGAACTTC
CGTTAATCATCGAACTGGTGAGCAAAGGTAAAAAATATGTCATCTGCCACGCCGATTATCCTTGTGACGAATACGAATTTGGAAAGCCAGTTGATCATCAGCAGGTAATCTGGAACCGCGAA
CGAATCAGCAACTCACAAGACGGGATCGTGAAAGAAATTAAAGGCGCGGACACGTTTATCTTTGGTCATACGCCAGCAGTGAAACCACTTAAATTTGCCAACCAAATGTATATCGATACCGG
CGCAGTGTTCTGCGGAAACCTCACATTGATTCAGGTACAGGGAGAAGGCGCGTGGGCATAA
>PNPANE_15785 Protein ninG
ATGAGGAAGGCAAGGCGGCGTTGTAAGAACGAAGAGTGCAGGGAATGGTTCTTCCCGCAATTTCAGAACCAACAGTGGTGTTGTGTTGATTGTGGTACGAAGTTAGCACTCGAACGACG
AAGCAAAGAGCGCGAAAAAGCAGAAAAAGCAGCAGAGAAGAAACGACGACGAGAGGAGCAAAAACAGAAAGATAAACTGAAGATTCGAAAACTCGCCTTAAAGCCCCGCAGTTACTG
GATTAAACAAGCCCAACAAGCCGTAAACGCCTTCATCAGAGAAAGAGACCGCGACTTACCATGTATCTCGTGCGGAACGCTCACGTCTGCTCAGTGGGATGCCGGGCATTACCGGACAACC
GCTGCGGCACCTCAACTCCGATTTGATGAACGCAATATTCACAAGCAATGCGTGGTGTGCAACCAGCACAAAAGCGGAAATCTCGTTCCGTATCGCGTCGAACTGATTAACCGCATCGGGCA
GGAAGCAGTAGACGAAATCGAATCAAACCATAACCGCCATCGCTGGACTGTCGAAGAGTGCAGGACCATCAAGGCGGAGTATCAACAGAAACTTAAAAAACTGCGAAACAGCAGAAGTG
AGGCTGCATGA
>PNPANE_15790 hypothetical protein
ATGATTGACCCCAATCGAAGTTATGAGAAAGAGAGCATAGCAAGGGCAATGTGCGCAGGATGTAACAAGCAACTGGCACCTGATGAAATTTACGCCTGTGCAGAATGTGTTAACGAATGGC
TGGTATATCGCGATCCGAATGGAGATATGTCGAATGAGGAAGGCAAGGCGGCGTTGTAA
>PNPANE_15795 Protein ninB
ATGAAAAAACTAACCTTTGAAATTCGATCTCCAGCGCATCAGCAAAACGCCATTCACGCAGTACAGCAAATCCTTCCAGACCCAACCAAACCAATCGTAGTAACCATTCAGGATCGCAACCG
CAGCTTAGACCAAAATCGGAAGCTTTGGGCTTGCCTTGGGGATGTCTCGCGTCAGGTCGAATGGCATGGTCGCCGGCTGGATGCAGAAAGCTGGAAGTGCGTGTTTACCGCGGCATTAAA
GCAGCAGGACGTTGTTCCTAACCTTGCCGGTAATGGCTTTGTGGTAATAGGCCAGTCAACCAGCAGGATGCGTGTAAGCGAGTTTGCGGAGCTATTAGAGCTTATACAGGCATTCGGTACA
GAGCGCGGCGTTAAGTGGTCAGACGAAGCCCGGTTAGCACTGGAATGGAAAGCGAGGTTTGGAGACGCCGCATGA
>PNPANE_15800 hypothetical protein
ATGTGTGTATATCACCTGAAACAATGCTACGGATGCGGAATGCCTCTTCGGTTTAATGGATTCCAGAGTATTCCTGATGTGCCTATGTGTAGTTCTTGTCGGGATAAAGGAATTAAGCCGAGAT
ACGTTTATGTTTATTCCGCCAACAAGATATTGCCGAAGTATGAATACAGCACGGAAATAATGAGGTCAAAAACAAATATCTAA
>PNPANE_15805 Phage protein
ATGGACGAATCAAGAAAGCAGTTTTTGGAGTGGTTTGGTGAAGAGTTCGAGTCTATTAACAACAGCGAAGAACTTCACGTTCAGGCCATCAAGATGATTGCCTGGCAGTCATGGGTTAAGT
CTCGCGCAGCTATCGAGATAAAACTCGATGACAAAGTGATGGCTGAGGATGATTTCGACAAAGGACACAACTGCGCTATCGATTATTGCGCTGACGCCATCCGCGCCGCTGGAATCAAAGT
GAAGGAGTGA
>PNPANE_15810 hypothetical protein
GTGAGCAAGTACGAAAAATTAGATCAAAACATTCTTTCAATGCTGAGTGAAAGACCAACACCTGTTTTTGATATCTGGCTTAAATGGCGGAGCAATGGAATGTATATCGAAACCATCGATCGC
CGTATGCAATACCTGAGAAAGAAAGGGCTTGTTGCAAATGTGCGTGGGAAGGGGTGGGTGAAAATTAACCTGTCATAA
>PNPANE_15815 Prophage protein
GTGGCTGACTGGCAAATTCCAATCGTCATTCTTGCCGGAGCTTCGCTGGTTGCTGGCTTTATCCTGCTGAAGAAGCATAAAGACCGTGATCAAAAAGTCGAAGTTCTCTATGGGTATCCAGC
GAACAGCACAACATGGCTGACCATTTACCACTACCGAAAATCAGGCCGCTGGGTATTCGAATGGGATGATCTGTTCGCTGAAAAGCGACCAAAGTCATGGGGAGACATCAGCGAATGCATG
ATGTTTGAAGAAAGAAAATCCGGCGCAACCCGAGAAGAGTTTAACGAAGCGTGGGCGCGATTAAGTGAGAGAGGGTATTTGTGA
>PNPANE_15820 DNA helicase
ATGACCGATAATTTTTATGCGCCGCCCCATAGCATCGAGGCAGAGCAGGCTGTGATTGGCGGATTGCTTCTGGATGATGACAGCAGTGAGCGCGTCCAGAAAGTTCTGGCGATGCTGAAGC
CTGATTCATTTTACAGCCGACCACACAAAATCATTTTCGAAGAAATAACCAGAATGCACCGGGAGCAAAAGCCAGTAGATGGCCTGACGCTTTTCGATGAACTGGAGCGCAAATCGTTAAC
GGCGTCTGTTGGCGGTTTTGCTTATATCGCTGAGATCGCAAAGAACACGCCAAGCGCAGCAAACATCGTTGCCTATGCAATGCAGGTTCGTGAAACCGCAATGGAACGCTACGCCATCAAC
CGCATGACTGAAGCGACGGAATTGCTCTATTCCCGCAACGGAATGACTGCAACGCAGAAGTACGAAGCTATTCAGGCGATTTTCACGCAACTGACAGACCATGCAAAAACCGGATCGCGTC
GAGGCCTTCGCTCATTTGGTGAGGTCATGGAAGACTGGGTTAGCGACCTTGAGAAGCGATTTGACTCATCAGGCGAACAACGGGGAATGAGCACAGGGATCCCATCGCTGGACAGGATG
CTGTCACCGAAAGGTCTGGTGAAAGGCTCTCTGTTTGTCATTGGCGCTCGCCCTAAGATGGGGAAAACGACGCTATACAGCCAGATGGCAATCAACTGCGCAGTGCATGAGAAAAAGGCT
GCCCTGATGTTCAGCCTTGAAATGCCAGGTGACCAGATACTGGAAAAACTGGTAGGGCAGAAGTCAGGTGTTAACCCGAATATTTTTTACCTTCCGGCGACAAATGACGCTGATGACGGCT
ATCAGGGTGATTACGATGGTGACTTCAACAGGGCGATCGAAACAGCCAATCGCTTGAGTGAAATCGACCTGCTTTACATCGACGACACGCCGGGATTATCTCTGGCTCAAATCGTCAGCGAA
AGCCGTCGAATCAAGCGAGAAAAGGGGGGTGTTGGCATGATTCTGGTCGATTACCTGACACTAATGACCGCTGAAAAGGCCGATCGTAACGACCTTGCTTACGGCATGATTACTAAGGGGC
TGAAGAACCTTGCCAAAGAGCTTGATTGCGTTGTTGTGCTTCTGACGCAGCTTAACCGCGCACTGGAAAGCCGAACTAATAAACGCCCTTTACCAAGCGACTCCCGAGATACAGGGCAGAT
TGAACAGGATTGCGATTATTGGGTTGGTATCCATCGTGAAGGTGCTTTTGATGACAGCGTTCCGCCTGGTGAAACCGAACTAATCCTTCGTCTAAATCGCCATGGAAATACCGGCACGGTGTA
TTGCATTCAGGCAAATGGCGCTATTTATGACACAGACCAACAGTCTGCTGAAATGCGCCGCCGTGAACGCGAGGAACCGCAGTCCAAGAAGAAAGGAGGATTCTGA
>PNPANE_15825 DNA replication protein
ATGAGTAACATATCCAATCTAGCCGAAGCCAGAGAGGCCAGAAGGATCCAGAAGCCGCGTACAAATGGCGGCAAGGGGTTTGCCTTGATTCACCGCCAATTCATGGATAGCAAGCTATACA
AGGATTCTCAGGCTGTACATCTTTTCCTGCATCTGATACTGAAAGCCAATCACTCTCCGGCAGTCGTAAATACCGACATTGGTGAGATGTTGGTTGAGCGAGGACAGCTAATTACCGGACGG
CCAAAACTGGTAAGTGAAACATTCATCCCGGATAACAAAGTGAAAAGTTTGCTTCGTTCTTTTGAAGGGAATGGAATGATTTGTATCGAGTCGAAAGGGAGAAAATTCAGCCTGATAACAG
TGTTGAAATATGATGATTTTCAGGCTCCAAATTGTCCAACGGATGTCCAACGGATGTCCAACGCAAACACCAGTAATGACGCGGCTCACAGCAAATGTTATCCAACGGATGTCCAACGATTGT
CCATAAACAATAATATAAATAATATCTCTAATACTAACGTATTAGAGAGTACCGCAGCAGACGAAAATCCTGACAAGAAAAAATCGGCTCTCAGTTGTCAGGATGTTGTCGATGCTTACCACGA
ATTACTTCCTGAAGCTTCCAGGGTTCGCGCACTGAATGACAAACGTAAAAACCAGATCCGAACTTTCTGGCGAAAAGCCGGAGTGATAACACGCCAACTTGACGGGCATGGGTTCACGATG
CAGGACTGGAGAAATTATCTGAGCTACGTAGGCGAAAATTGCCGATGGATGTTCGAAGAGCGCCAAAACCATCAACGCGGAACCGTCTGGCACAAAAAGGGATTTGATTTCCTGCTTAAC
GATAATACCTACCTGAAAGTTCGTGAGGGTGAACACGATGACCGATAA
>PNPANE_15830 hypothetical protein
ATGCCAAAACAACTCAGTCCTGACCAGGACAAATTACACAAAAACATACTACGTGATCGGTTCTTATCCAGCTTCAAACAGCCTGGTCGATTTCGGGCTGAGTTGGAGAAAGTGAAGCTAAT
ACTGAAGAGGAAAGGTCATGAGTAA
>PNPANE_15835 CII

ATGAACGTAGTTGCAACTAAAAGCAAGAAGGCGGCTCGCATTGAGTCCACTTTACTCAACAAGTTAGCCATGATGGGCCAGAAGACATTCGCTAAAGCTATGGGTGTTCCTGAATACCAGG
TAAGCCGATGGAAGAACGGTTTCTTCTCTCAGGTCAGCATGATGCTTGCGGTTCTGGAGTATGGAATCGAAGACGAGGAAATGGCAGAGCTCACCAGGCGACTTGCTACCTACCTGACAAA
AGAAAAAGCCCCGAAGAACGGCGAATTCTTCGATGCCTGA
>PNPANE_15840 HTH cro/C1-type domain-containing protein
ATGAATAATATTCGCAATTTTCGCGAGCGCTTCGGTTTAACGCAGGAAGATCTTGCGAAAGTACTCGGTTGTACGCGTGGTGCAGTTTGTCATTACGAGACAGGCAGAAGGGGAATGGATAT
CAATCTTTGTCGCGCTTTTATCAATGCGTTCAAAGAATACGGTTATGAACTAACCATAGACGATCTTTTTCCACCAAAGGCCGCGTAA
>PNPANE_15845 Repressor
ATGAACTGGTATGACATAGCGAAGCAAAGGATTGATCAGCTTGGATTGAGTCAGGATAAAGTTGCTGAACACCTTGGTGTAACCAAAGGTGCTGTTAGTCATTGGCTTAACGGGAGAAGGA
ACCCATCAATACAAGAAATTGGAGCTATATTTCAATATCTTGGCGTTACAGACGCGAGGTTCAACTCTGACGGAACCTTTAGCGTTGGCGAGTCAACAGAACAAAAGCCTGTTAAACCTCAA
TTTGAATACCCATTCTTCTCTCATGTTCAGGCTGGAATGTTTACACCAGAATTTCGAACCTTCACTCAACTAGATGCTGAGGGATGGGTAAGTACAACCAAAAAGGCCAGTGAGGCAGCTTTC
TGGCTTGAAGTTGAAGGCCACTCAATGACGGCTCCAGCGGGATCACGACCAAGCTTTCCTGAAGGAATGCTGATTCTTGTAGACCCAGAAGATCCTGTAGACCCAGGCGATTTTTGCATTG
CAAGGTTATGTGGTGATGAGTTCACTTTTAAGAAGCTCATCAAAGACAGCGGACAAGTATTCCTACAACCGCTAAACCCTCAGTTCCCAATAATGCCGTGTAACGAACAATGCAGGGTTGTA
GGTAAGGTTGTAGCCAGCCAGTGGCCTGATGAGATATTTGGGTGA
>PNPANE_15850 hypothetical protein
GTGGCAAAGAAAAACGAGAGTCCTCTGTTACAGTCGGATGTTGATCGCGTCATAAACTCCAAGAAAATAGTGGATGTTAGCAGCATAAAATGGGGTAAGAAGCCGCCACCTGGCAGGTCA
CCGATGTGGTTACAAACGGCTATCACACCTTATGAGGATGGCTCACCTTTGCCTGGCCTAAAGTTTGTTTTACAGTGGCGACCAGCTGATGAATACGGAGATTCCCCTAAAATTCAGATGGTG
GCGCTTTACTTTGGTCGAAGAATTTTTGGTGTGGACTCTTACCCAAATGATAGACATACTAACAGAGTCCGGGTATGCCATCCTGACTATGCTGAAAGCATTCTTGGCCCACATTATCACCTTT
ACTTTGAATCAGCGTTGCCGTATGAAATAGGTCTGATCATACGCGAGAAGATAGCCCCGGATGACTTGTTAGGACACTGGCGCTTTTTCTGCTATAAACTGAATGTTACATGCAAAGGAATAC
TGCCTTTGCCGACCCAAGAAGATTCGGGACAAATTCCATTGCTATGA
>PNPANE_15855 DUF1828 domain-containing protein
ATGATGTGCTCAACGATAATTTCCAGACTCGGCTATGAATGCCTACCCATAGGTGAAGAGTCCTTGAGAATAATCAGTCCGTTTCCCTATTGTGACGATGGGGAGCATGTTGGCGCATTCGTAC
AGCATATTAATGGGAGTTTCAAAGTAACTGATCGATGCGACGCACTTATGAACATGGAGGCTCGTGGAATTTCACTTAACCAAAGTCGCCTTGATGTGATCAGGCAGGCACTCGCGCGAGAA
GGCGCTGAGCTAAATGAAAGAGGCGAAATTCTTAAGTGGGCTCATGATGAAGGTGAGCTTGGAAAGGTAACATCAGATGTTATTAGGGCTGGTATTCTTGCTTCAGCTATGTCCATCGATTG
GTATTCATCTAACCAATCTAAAAGATTTGAAGCTGAAGTGATTGATTTCATCTCAAAAAGTTCACTATCAAATTTAATGTCCCTTCGAGAGGAAGTCAGTGGCATGAGTGGTCACAATATTGTC
ATACCTGTTACCATTAAAACCGCAATGCCCAAATACATATTTACCTCAAGCATTAAAGAAGGTGGATCATGGAACAGCGCATATTCATTGCTTGGAAAGCTGATGGATCTTTATCAGGCCAACA
ACGCTGTTAACAATCGATACGTAGTTATTGACAATGAATCTATCGGCTCGCAAATGCAGCAACTGATACTGCTGTTCAACGACGTAAGCCAAGTTCTTCCATTCGAAAGCAGAAGTTTATGGC
TTCCAAAATTAGCTGCATAA
>PNPANE_15860 Fumarase D
ATGAAAAGCGAAGAGCTGGCGCAGTTGCGCTATCAGGAAATGTGCAGGATTGTGGGTGATGTCGTTTTTGCTATGGTTGCTGAGGGTCATGAGACCAAAAGAGTCGCTATAGCTGACGTG
ATAAGAACGGAGCTATCAAAGGGGCTGGATAAGTGGGATGTTGACCAGATTCAGGTTATGGAACTAGCGGTGAAGCTACTGGAAGAGTAG
>PNPANE_15865 Antitermination protein
ATGACGCGCAGAACAGCTTTCAATGGTTCAGCAGCAGGTCGTCGTCGTGAGCGCCGTGCAGCGCTTCAGAATGCGGTAACGGCAAGCTCAGAAGTATTACACCGCCCCACCCTTAGCCGC
GCCCAGATTCAGGCCAAAGGAAAACATGAAACGCCAAAACGTATTGAAGACGCAAAATCACTTCAGTTCATGGCGAAAGATGCATTCTGGCAACTGGAAGAATACAGACGCCATCTGGAG
CGGGCAGCCATTGTGTACGCAAATGAGTTTGGTCATAAGCCACCAGAAACCGGTGTATGTTTGCCAGACGTAGCGCTTTACGCAGCAGGTCATCGTAAATGTAGACAAGTTACCGCTAGATA
A
>PNPANE_15870 hypothetical protein
ATGCCCTGGCTTTTCCTTCTATTCCGAGTGATGCCGCTACGGATTGCGATCCAGACACTTCCGCTGCTGACTGGTAGCCAGTGTTGGTTGCTGCTGACCTGTCGCCAGTGTTGGTTGCTGCTG
ACCAGTCGCCAGTGTTGGTTGCTGCTGAACAGTCGCCAGTGTTGGTTGCTGCTGACCAGTCGCCAGTGTTGGTTGCTGCTGAACAGTCGCCAGTGTTGGTTGCTGCTGACCGGTCGCCAGT
GTTGGTTGCTGCTGACCAGTCGCCAGTGTTGGTTGCTGCTGAACAGTAGCCAGTGTTGGTTGCTGCTGACCAGTCGCCAGTGTTGGTTGCTGCTGACCAGTCGCCAGTGTTGGTTGCTGCT
GAACAGTCGCCAGTGTTGGTTGCTGCTGACCTGTCGCCAGTGTTGGTTGCTGCTGACCAGTCGCCAGTGTTGGTTGCTGCTGAACAGTCGCCAGTGTTGGTTGCTGCTGACCGGTCGCCAG
TGTTGGTTGCTGCTGACCAGTCGCCAGTGTTGGTTGCTGCTGAACAGTAGCCAGTGTTGGTTGCTGCTGACCAGTCGCCAGTGTTGGTTGCTGCTGACCAGTCGCCAGTGTTGGTTGCTGC
TGAACAGTCGCCAGTGTTGGTTGCTGCTGAACAGTCGCCAGTGTTGGTTGCTGCTGATCGGTTACCAGTGTTGGTTGCTGCTGAACAGTCGCCAGTGTTGGTTGCTGCTGATCGGTTACCA
GTGTTAG
>PNPANE_15875 hypothetical protein
ATGACATTTGCTATCGCGGGCGGTGCCGTCATGGGTATCGCACACCTTAATGAATCACTTTTAGAGCGTATCACCAGAAAATTACGGGCCGGATGGAAACGTCTGGTCGATATCCTGAATCAA
CCAGGAGTGCCGTGTAATGGATAA
>PNPANE_15880 Host cell division inhibitory peptide Kil
ATGGATAAATCACTTATGGCTATTCAGTCTAAATTCGCAATTGCTGTTTATCTTGGTGACAAAATAATGTATCGCGAAGCTGTAGAAGCCTTTCGCGAATGGAGGTTGAAATGA
>PNPANE_15885 Recombinase
ATGGATTTGAATAAATTCGACGACCCATTCAGTCCTGAAGATATCGAATGGCGAATACAGCAAAGCGGTAAAACACGCGATGGCAAAGTGTGGGCTATGGTGCTGGCTTATGTCACGAACA
GGGCAATCATGAAACGCCTTGACGATGTTTGCGGCAAAGCAGGATGGCGCAATGAATACCGCGATATTCCCAACAACGGCGGCGTTGAATGCGGAATATCAATCAAGATTGATTCCGAATG
GGTCACCAAATGGGATGCTGCTGAAAACACGCAGGTAGAAGCCGTCAAAGGTGGTCGTTCCGGTGCAATGAAGCGCGCTGCCGTTCAGTGGGGAATCGGTCGGTATCTGTATAACCTTGA
GGAAGGTTTCGCACAAACATCACTCGATAAAAAGCAGGGATGGCACAGGGCAAAACTGAAGGATGGAACAGGATTTTACTGGCTCCCTCCATCGCTGCCGGACTGGGCCATGCCAGCATC
AGGCAATCAACCATCACCAGAAAATACCAACCAGAAATCACCATCGTTTGACTGCGAACAAATCCTGAAAGACTTCAGAGATTATGCAGCAACAGAAACTGACAAGAAAAAGCTAATTGAG
AGATATCAGCATGACTGGCAATTATTGGCTGGTCACGATGATGCGCAGACAAAATGCGTTCAGGTAATGAACATCAGAGTTAACGAACTAAAACAGGCGGCATAA
>PNPANE_15890 Single-stranded DNA-binding protein
ATGGCTAGTAGAGGCGTAAATAAGGTGATCATCTTAGGCCGGGTCGGACAAGACCCGGAGGTTCGTTATTCACCATCAGGGACGGCGTTCGCTAACCTGACAGTCGCTACATCAGAGCAGT
GGCGAGATAAACAGACTGGCGAACAAAAGGAGCAGACTGAATGGCATCGTGTTGCCGTAGTCGGGAAACTTGCTGAAGTCGTAGGGCAGTATGTGAAAAAAGGTGATCAGGTTTATTTC
GAGGGAATGCTGAGAACCAGAAAGTGGCAAGACCAGACAGGGCAAGACCGCTACACCACTGAGATTAATGTTGGAATTAATGGTGTGATGCAAATGCTTGGAGGCACTGGCGACAGCAA
ACAACAAGCAGCCGACAGGCAGTCACAGAAACCACAGCAGCAACCATCACCAACACAACATAACGAGCCACCGATGGATTTTGATGATGATATACCCTTTGCACCAGTAACTCTCCCCTTCC
CTCGTCACGCTATTCACGCAATTTAA
>PNPANE_15895 3'-5' exoribonuclease
ATGAATCATTTAATGGTTGACCTTGAAACAATGGGCAACGGGCCATACGCGCCCGTTATTTCGATTGGGGCAGTATTCTTTGATCTGAAAACTGGAGAAACAGGAGAAGACTTCTCGGTTAA
TATCTCGCTCGAGTCATCAATGCGATACCGGGCGCGTCCTGATGCTTCAACCATTTTATGGTGGATGGAACAGGGAGAAGATGCCAGAAAATCGCTAACCAATGACACTCAAGAGCTTTCAA
CGGCTCTTTCATGGTTATCAGACTTTATCGCAAAGCACGCCAACCCTAAATTCGTTCAGGTTTGGGGAAATGGCGCATCATTTGACTGTGTGATTCTACGAAATAGTTATGCTCTGGCCGGGC
ACCAAGCGCCCTGGCAGTGGTGGAATGACCGCGACGTCCGAACCATCGTCGAGCTTGGAAAGGCAATTGGGTTCGACCCTAAACGAGATATGCCATTCGAAGGAACTCGACACAACGCGC
TTGATGATGCCATTCACCAAGCCAAATACGTTTCAGCAATCTGGAAAAAGTTAGCTAAATAA
>PNPANE_15900 Anti-RecBCD protein 2

ATGCCAGCGCCTATGTATGGTGCGAATGACCCGCGCCGCTGTTCCGGCAATTCCGTCTCGGAGGTGCTGGATAAATTCAGAAAAAACTACGACCTGATAATGTCACTACCGCAGGAAACGAA
AGAGGAAAAGGAATTTCGCCACTGTATATGGCTTGCAGAGAAAGAAGAACGCGAGCGAATTTACCAGACATCAATCCGACCATTCCGCAAAGCCACATATACCCACTTCCCTGAATATATCG
ACCCGCGCCTGCGTAATTATCGATCACGCTATGGCGCTATCAGTAATGACTGA
>PNPANE_15905 Uncharacterized protein in eae-abc2 intergenic region
ATGAGAGGACTTGCATACAATCCCGGCATTCTTCCGGCAGAAATGATTATTCGCCAACGCGTAAAGCCAATGCCATCGAGAGAGGAATTGCTTAAGAGAAATTCTTTTCCGTCAGTGAATCA
AAACAAATATCTGAATGCGATGTGGCGTAAAGGAGGCAACCAGTGA
>PNPANE_15910 WWE domain-containing protein
GTGAGTAATTCAGCACGACTACAGCTTGGTTTTTCACCGCTATCAAAAACTATCATGCTGGCAAAAATGCGCGATGTTGAAGGTGGACGTATGCGCGTTGGCAATGATCCAGGTCGTGATGT
TACCAATGAGGCTGCTCAATTGGTGTGGCGACTGGTCATGGCTGAAGGTGGTGAGATCGCGTGGGAGCTGGATGATGGTTCTCGCATGGTGTTGAAGGCAGAAAAGCAGGAGGCAACCA
GTGAGCCAGATTGA
>PNPANE_15915 dATP-dGTP-PPHyd domain-containing protein
GTGAGCCAGATTGATTATCAGGCACTGCGTGCTAAGGCAGAAAAAGCAACGTGTGGCGAGTGGTCGCTCGAATATGGAGAGGGCCGATTTGATGGTGATGATGCGCTAATTCATCGTGAA
GTTGCTGGATATATTCCCATTTGCAGAATTGAAGGAGCGCATCCAGAAAGCGGTTTCGATGAAGATTTCCAAATGGAACAGCAGGCCAATGCTGAATTCATCGCCGCAGCCAATCCGGCTAC
CGTACTGGCGCTGCTGGATGAGCTGGAATCCAAAGACAGGCGCAACGCTGAGCTGGAGGCGAAACTCCAAGCCACTGACAAATTGCATGATAGCGCGTTCCGTCATGGTCTCCAGCATGG
TTTTAGTTACGGTCAGACAGATGATCAAGCAGGATTTGAGAAAGCCATCCAAGCGTATGGGCAGCAGTGA
>PNPANE_15920 SlyX protein
ATGAAATACGAAATCCCGGAATCAGAAGATATTGAATGGCAGCAGGCTATGCTCCGTGAAATAGACAGCGCCCTTGACGTCTTGCGTGATGAGCATGAGCACGCAGTGGTGGTAGAAGAAA
TCATCAATGATATCACCGCGAGAATAGCATCACTCCGCGCGTACTCTGGATACTGA
>PNPANE_15925 hypothetical protein
ATGAGCACCTTCACTAAAGAGCAGTTAATAGAATACCTGAAGGAATCGACGCAGAACGCTAGTGGTATGTTTGAAATCAGCGAGGACACCATCTGCGCGTTGATGTCCATGCTCGCCTCCCC
GCCAGCGCCGGTATCTGTGCCCGCTGCGATGGAAATTGATGATGACTTTGACAGCGCGTTTGAACACGGAAAAGCTGTCGGCTGGAACGCCTATCGCGCCGCCATGCTTCAGGCCGAACCT
GTAAGTAATAGTGATGAGTTACCGCTGGACTATCTGCAAGGACACAAAGACGGCCTGGAGTGGGCTGCACAATTGGCAGAAGCCAATCATCCGCAAACAGGTGACTGGTTGTACGACGAC
CCAATCGATCTTGCCAGGGCGATTCGCAAAGGTCCGGATATGCCTACTGTTCAGGGTGGCAACTCTCCGGTAACTCCGGATGGTTGGATAAGCTGTAGTGAGCGAATGCCGGATAAGTTAAT
TCCGGTAATGGTCATGTATGAAGACGGTGAGATGTGGTCTGCAATGTGGAATGGCAATCGCTGGGATGATGGCACCGAATATCCGAATCCGCACTCAGTTACGCACTGGCGTGAAATGCCAG
CAGCACCGAAGCAGGAGGTTAACCGTGGCTAA

Prophage 5

>EHGDEF_01245 tRNA-Xxx
GGCCCTTAGTTGGTCAAGAGTGGCCCTTTATGTCTATGATTTATAAAGCAACCTATTGATTTTCGGTTGTTCTGTTAGGAACTCATAATCGCTTGGTCGCTGGTTCAAGTCCAGCAGGGGCCAC
CA
>EHGDEF_01250 hypothetical protein
ATGAGAATAGATTCTTTAGGATGGAGCAACGTTGATGTACTGGATCGCATCTGCGAGGCTTACGGGTTTTCACAGAAAATTCAGCTAGCTAACCATTTCGATATTGCATCGAGCTCCCTCTCTA
ACAGATATACCCGAGGCGCTATTTCGTATGACTTTGCGGCACACTGCGCTCTTGAAACAGGGGCCAATCTGCAGTGGTTACTTACAGGAAAAGGGCAACCGTTCACATCTTCTGCGACAGCC
GAGGACACAATGAGCATCGAGCTATTCACATTAAGTGAAGAAATACTCAAAAGTGATGGTTCTATAACAGTCGACGCTCATTTTTTCACAAAGCCGCTTACAGATGCGATGGCTATACGAACG
GAAGGAAAACTCCATTTCATTGATAAGCAGGCATCACTCTCTGACGGCCTTTGGCTGGTCGACATAGAGGGTGGAATTAGTATTCGAGAGCTAACAAAACTCCCGGGTAGAAAATTGCACG
TTACTGGTGGAAAGGTTCCTTTTGAGTGCGGTATTGATGACATAAAGACGCTGGGTAGAGTGGTAGGTGTGTACAGCGAGGTTAATTGA
>EHGDEF_01255 Prophage excisionase
ATGGCTTCTGAAATCGCAATCATCAAAGTGCCTGCCCCCATCGTCACCCTGCAACAGTTTGCAGAACTTGAGGGGGTGTCATACCGCACCGCGCGCCCGCTGGACTACCGGAGATAACCCGC
GTTTACCTATCGAACCTCGCGTTATCCGTAAGGGCTGTAAACGTGCTGGCGGTCAAGTTCGTATCTACTATGCCCGCTGGAAAGAGGAACAGATGCGTAAGGCATTGGGCCATTCCCGTTTTC
AACTCGTTATTGGTGCGTAA
>EHGDEF_01260 Regulatory protein CII
ATGTTTGATTTTCAGATTTCCAAACATCCCCACTATGACGAAGCGTGCCGGGCTTTTGCGCAGCGTCACAACATGGCGAAGCTTGCCGAGCGTGCGGGTATGAATGTTCAGACGTTACGTAA
CAAGCTCAACCCGGAACAGCCTCACCAGTTCACGCCGCCTGAATTGTGGCTGCTGACTGACCTGACCGAAGATTCAACCCTCGTTGATGGTTTTCTGGCGCAGATTCATTGCCTGCCATGCG
TGCCGGTTAATGAGCTGGCTAAAGACAAATTGCAGTCTTATGTCATGCGTGCAATGAGTGAACTCGGCGAGCTGGCGAGCGGTGCAGCCTCTGACGAGCGCCTGACCTCTGCCCGTAAGTA
CAACATGATTGAAAGCGTTAACGCTGGCATTCGCATGTTGTCATTGTCGGCGCTGGCGCTGCATGCACGTCTGCAGACTAATCCCGCTATGTCGAGCGTGGTCGATACCATGAGCGGTATTG
GCGCATCCTTCGGGCTGATTTGA
>EHGDEF_01265 Protein fil
ATGCTGAAAAGTGAACCGTCATTCGCGTCTCTGCTCGTTAAACAAAGCCCCGGCATGCACTACGGCCACGGCTGGATCGCAGGTAAGGAAGGCAAGCGCTGGCTGCCGAGCCACTCACAG
TCCGAATTATTAAAAGGGCTGAAAACAAAGCCGCCGAAATCGTCAGGTTTTTTAATTATTCGTATTGTCCACTTTATTATTAAAGGGGTTAAACATGTCACGCGATGA
>EHGDEF_01270 Protein dhr
ATGTCACGCGATGAATTAAGGATTGTTTTAGGTGCCATGATTCCGAACATGGAAGACGGTTTTGAAATTAAAACCCGCGATGGGATAATTTTACGAGTTGACCCGGAATGGGAGTGCTGCAA
GCAGTTTAAAGATAGCCTGAAAGCTGAAATTATCAGCCAATTGAAAAGTAAACCAGCCGTCGTATTTGGTTATAGCTAA
>EHGDEF_01275 DUF2732 domain-containing protein
ATGCGTAATACCGAAATCCGTAGTTTTAACACTGATAGTGATGCGCTGGCCGTATTGCTGACCGATGCAAAAAAAGAAGAGCGTAAAGACCGCGCGCTCGCTGTTTCCATCCGCCTTGAGGC
GCTGGCTATCCATATCACCAAAGAGGGTATGAGCGGCACCGAAGCTGCCGAACTGCTGCGTCGTGAAGCAACCCGCTTTGAGAATGAATCACAGGAGCTGCACTAA
>EHGDEF_01280 hypothetical protein
ATGGCCGACGCAATGGATTTAGCACAACAGCGCGAGCAGGAAGACCGCGAGCGCCACATCAGCAACGCGCGCAGCCGTATCGCTGCGCCTTCCCGTTTTCTTTGCGAAGAATGTGACGCA
CCAATCCCGGAAGCTCGCCGCGCTGCGATTCCGGGCGTGGCTTTTTGCGTGACCTGCCAGCAAATCGCCGAACTTAAATTAAAACACTATCGGGGTGCGATATGA
>EHGDEF_01285 hypothetical protein
ATGACTTTATTCACTGAAGAAGATCCCACCCAACGCCGCTATGTTGATTTGATTACAGAGGTGACAGCTTTCGAAGCAACGAAAGAACCAATGAAGATCATTTCCGCATCCGACATGCTGGG
CATTTGCGATGCTTTTCTTTCTGAGACTCAATGCCACATAGCAGACCGGCTACCTCTCTTTCAATAGCTGGTCGGCATGAGCTAGGTCGGAAAATTGAGTGGAATTGTCCACATAGCTGGAAACC
ACGCGAAGAATGGTCAACACATGTTCGGCACGCCTTACAAATTTTAAATCGACGTTATTTGGATACCCCAGTATCTCAACTGGATGACTGGCAGACCTGGGAAGAACTATCGACAGATATTCA
TGTGTCGGCTCGCTGTACTCGGCGGACTGTTGAGTTTTATCGCTCTGGAAATCCCCAGCATTTACCGATGTCGACTGAGCTTTTTGCTGTTCCCGAAGTTTTTTCAAAATTTGTAGCTTCGATT
CTTTCGGGTGATATTCACCCTGTTTGGATGTGGCATGCTGACGCAGCCAAAACGCCGCGATGCCTCGATGGGCTTTATCCCAAATACGCGCCGCTTTCTTGA
>EHGDEF_01290 Replication endonuclease

ATGGGTGAGATTGATTATGCCTTTGTATGGAATTTTCCAAAACAGGCAATAGCCAGCCCATATCTTACCTATGACCAACAACATCGCCGCGACCGTATGCTCGCGGCTTTGCTGCATGCGAGA
AAGGTGCTTTCTCTCCAGCCTGAATGCGTGCGTTTTGATGTTTATCGCACCGCTGCTGTGCTGGAGAAAAATCAGGGCAGTCAACGAGCCAATGCCTTTTTAATAAGTTTCTGCAAAAGGGC
ATTGCCACGTCTTGAACTGGTCGCAAAAAAATACGAGTGCGCGGGTATCAACAGCAATGTATCAGCCGCTGTTTTTGGCAGTCATTTTGATACTGAACTTATGCAATATCTGGCGTCACGCAT
GGTCAATATGGTTGCCAGATATAACCGACTCCCGGACATGTCACGCGCCGATATCGACCTTTTGGCCGCTGATATCGCGAATTTCATTCGCGCTGAACTGGCTGACATTGATGACACCGGATT
TAGCGAGCTTAAAACGCTGTACACCTGGTACATGCGCGCCGGTTTTATTTCTCTGCAATTCAATGTTACCCCTCCGCATTGGGAGCGAGTGACAAAGAAATATGTCGGAGAAGATGAAATCG
CCCCGGCCATCGCTCGCATGTTTAACGATATGTGGTGGCGTGGTCGTCTGCGTCGCATTGCGGCTGCATGGCGCGAACATCTGCAAATTACTGTCGGTAACGTCAGTAAGAAAAAGCATGCC
TATGCGAGTAAAAACTGCGTGACTGACTGGCGTGAACAAAAGCGCCGCACTCGTGAATTTCTCAAAGGTCTGGATCTCGAAGACGAAGACGGCAACCGTATCAGCCTGATTGAAAAAATTTG
ACGGCTCGGTCGCTAACCCTGCGATACGCCGCTGCGAGCTGATGACCCGCATCCGTGGGTTTGAAAATATCTGCAATGAACTCGGATACGTCGGGGAGTTTTACACCCTGACCGCACCGTCT
AAATATCATGCCACGACTAAAGCGGGATACCGTAACAGCAAATGGAACGGCGCCAGCCCGTCGGACACGCAGAGCTACCTAACCGGCCTTTGGGCGCGCATTCGTGCCAAGCTACATCGG
GAAGAAATTCGCATTTTCGGCATACGTGTTGCCGAGCCTCATCACGACGGAACGCCGCATTGGCACATGCTTATGTTCATGTTGCCAGAAGACGTCGAGCGCGTGCGCCTCATCATCCGCGA
TTATGCATGGGAGGAAGACCGCCACGAACTTAAAAGTGATAAGGCTAAAAAAGCCCGCTTTCATGCTGAGGCTATCGACCCGGAGAAAGGCAGTGCTACCGGCTATGTCGCGAAATACATT
TCTAAAAACATCGACGGTTATGCTCTCGATGGTGAAACCGATGACGAAAGCGGTGAGCTGCTGAAAGAGACAGCCCCCGCTGTATCAGCATGGGCGGCGCGCTGGCACATCCGTCAGTTTC
AGTTTATCGGCGGTGCGCCGGTGACGGTCTACCGTGAATTGCGTCGTCTCGCTGATACCGAGGCCGCGCACGGTCTGAGCGTTGAGTTTGCCGCCGTCCATGATGCCGCTGACGCCGGTGA
CTGGGCTGGTTACGTTAATGCGCAGGGTGGGCCGTTTGTCCGTCGCGATGATTTGCAGGTGCGCACACTGTATGAACCGCGCACCGAGTTTAATCAGTATGGTGAGGAAACCGTCTGCATT
CGTGGCGTATACGATTCCGCTATTGGTGCTGGCACTCCGATTTTAACCCGGCTAACGCAGTGGAAAATTGTGCCGAAGCGTGCCGTTGATTTGGCCGTTGACGTTAAGGGCGCTCCTGCGCC
CTCTCGGAGTTCTGTCAATAACTGTACGGGAAGCGAAAGCGATCCACCGGAACTCGATTTATCTAAGCCCCTAAGTCGACGTGAAAAACGAGAGCTGACAAACCGACTCAGGAAGCAAAA
GCCAGCAATACGGCGAAAATTTATCCACGGAACGGATGAGCAAAACGCAGCTATAGCGAAAACTATCGACGAGATACATCTGACTACCGGCATCACAATCAGCCGGGGCGAAGCCCTGCAC
CTGATGGCCGGTGGTAAAAGTTGCTTTAACGGTAAATGGTTGAGGGGAACGGCCAAGGGAGAAATATTTACCGCAGCACCATCGTATCAGGCTAAGGCTAGGATTATCCTCAATCGTGTTG
CGGCTTTAGCTGAACTCGCAACGAAAATATAA
>EHGDEF_01295 DNA damage-inducible protein DinI
GTGGATAGAGAGCTAAACGAGCACGTTATGATTGAGCGGGTCGAAATGATTGCGCGTCTGACTGCTGAAGGTACTTGTAAGGAAAGAGATCGTGAAATTGCATTGAATTTAATCGCGGAGA
TAGCAAGAGGCAACCTAATGAAAAATAATAATTTTTCTGTTGTTTTTTCCGCGCCACCTGTTGATGAAACCTTTGCAAAGGAGGGCAAGGTGAAAGTAAATATCACGTTGGATAAAGACCAA
AAAATCGGCCAGCCGGTAATTGATGCTTTTCAATGCGAATTGACCAAGCGAATACAGTCTGTTTTCCCCTCAACGCGCGTTACTGTTAAAAAGGGATCGATGACCGGTGTCGAGCTGATGGG
ATTCGATAAAGATTCAGACCGCGAAGCGCTGGATAGCATCCTTAAGGAAGTGTGGGAAGATGAGAGCTGGCGTTAA
>EHGDEF_01300 DUF4309 domain-containing protein
ATGGATACCGTAATAGCATTTTTATCTCTGGCTCTCTTTATTGCTTTTATCGTGGGGTTAATCAAGCCGTCGCTGGTTCGAATGCCGAACCGTAAGCGCTCCAGTGCGGTTTATCTTGGTGGCT
GTCTGGCGCTGGGTGTTATTGGCTCAATCTTATGGCCGACTGAAAAAAGTCAGCCTGTGGCAAAAGCTGACGTGCAGGAGGTTAAAGCGGAACCGACTACGCCAATCTTTGAGTACGCAG
ATAAGACCCTCAAAGAATATCGCAATGAGCCAAAAGAAACCCGGCACGATATCGTTAAAGGTTATGTTGGCTTCAAAGGTGTACCGGTAAGCTCTGCTGATGCTTTTTATGCCTGTATGAGTG
AGTACACTTTTACGAAAGATGATACGTTAAAGCTCGGTGATGTGTTGGAGTGGTGTTTTAACGACTTCGAGAAGGATCCACAATCCCTAAATAATAAAATCAACCTTGACGCATTTCAGGGTA
ATTTTAGTGGTTGGGATGGCTCTTATCGCCCGTTAGAGAAGCTGATAAAAGCCAGCATGAATGATGATTCTTCTTATAAACATGTTTCAACGGTCTACCATCTGATTTTGAATAAAGACCCGCA
TGCCGTTGTAAAAACAACGTTCCGCGGCACTAATGCTTATGGTGGTGTGGTAAAACAGACCGTAGCGGCGCGCGTCGATGTTCGAACGGGTGAAGTAACTTCAATAATAGATAACTAA
>EHGDEF_01305 AbiV family abortive infection protein
ATGAACTCAAGAAAAAGGCAGAAATATCTAAAGCTAATGTTTGAATGTAGAAAACGAATTGAAACACTACATGATTTAGCTTCTGGAGAATTTGGAGGGGTTTATCTTCAAGTTTTAATTGAA
ACAGAAGCACTACAACTCAGGAAGTTATTGGAGCTTATAGCCTTTTCATCGTTGATTTCACATGAGGCAGCCTATAGAACTGTTACACAGAATATTGATGGTGTATGGAAAGCCAAGCGTATT
ATGTGTGAGTTGGGGAAAATAAACCAAAATTTTTACCCGCTACCTGTATCAGAGATTACAAATGATGGATGGAAAAAATTAAAAGGTGGGTATTTAACAAAAAAACAATTTATTGCATTGTAT
GATGAGTGTTCAACTCATATTCATGTTAGAAATCCATACAGGGTGAGCGAAAATTCACTGGCCTTCCATAAAAAAGTTCCTGCATATTTATCAAGGATTGAATCTTTATTATCTAGGCATATTGTT
TCACTTGTAGATGACATGGAACTTCTTTTTGTAGAGGTCTTGCCTCATTTTGAAGATTCTACTAGCCCTATAAGGATTAGGTATTTGGTTAAAGACGATGGATGA
>EHGDEF_01310 hypothetical protein
ATGAAATTTATTGAGGGCGATAATATGCATCGACTACCGGGCGAAATTCCGCAGCACAAAACTAAAAGCATAAAACTTATGGCTATCGTTCATCGTCTGCAGACGATAATGGTCAATGAGAAC
CTGACTCCAGCAGAGTTGGTCGGGTGTGCAGAAATCGTCAGGGATAACTATGGCAAACTGGATAATATCAGCAGACAGGCACATTACGCACCGCCACCACGTCGACGATAG
>EHGDEF_01315 Transcriptional regulator
GTGCTGACGGGGTTGAACAACGAGCTACGCGAGGCGTTAGCCGGTCATAGCGTCGCAAATAATTCCGCCGTATGCTGGTTAAATAATTCGGGGTTTTTGGACATGCAAAATCCCATCTTATC
GAAAAAAGACCCGTTTGCAGATTGGGCAAAGAATCTGACTTTAATGGCATTAAATAACGACCTGAGCTCTCGCGAGGTGGAAAGCTACACCGCAAAAATGGTCGAAAAGGCTAATAAAGA
TGAGCTTTCAGTCGTTATCAAACACCTGTTAAATCACATCAGAATGCGCAAATAA
>EHGDEF_01320 hypothetical protein
ATGTTATCTCTCGTTTATGAAAATCCGTGGACAACCGTTTTTCTGCTGATTGTTGCTAGTTATTGTCTCAACAGTATTATTGGCGCATTGCGCGGCCAGTAA
>EHGDEF_01325 hypothetical protein
ATGCTTACCCCTCAAAACTTTACGGTCAACGAGTATGGCAGCGCGCTTCTTATCTCAGCCACGCGTGCATGGTCTGAATATCTCGCTAATTACATGTCTTCAGGGTCAGCCCCTCAAGAGACTA
TCGATTTATTTCTAGAGAATCTTGATACCGTTGTAAATGAAGTATCTGACGAGATTGGAAAACTCATCTGGGACTATAAATGCTATAATACGCCTCTGAGTGAAATGTATCTCGAAGTAAAAAAA
CGCATCAGGCTTATCATCAACTCTTATGCTTATGCGATGGGTTATGTCCACTCTCTAAATATCAATATTAAAGAATACGATCCAAAGCTATCCCTTACACTATCCAACTCAAAGATAAGATATCAAT
TATCAGAATTAGGCATTGCTTTTCAGAATCTTTATGGCAAGTTTAATGACCAGCACATTACAGGTTTTGATGATTATCGGGAAATAACAATAGTCATTAGTGAAATATTCAAGCAGTTTGGGCTT
GTTTTAGAGTGTCCCGATTGGTCTAGTGACTGTGAACTCTACATACATGTAAATTAG
>EHGDEF_01330 hypothetical protein
ATGGATGCGGAGATTAATATTTTGGAAACTAATTTTGAAATAACCCTGGAAGGTTTCGCTACTGAAGAAGAAGCAAATAATATCGGTAGGTTCACGCTAGAAGCCATCCGGGCTTTAAATAAC
AATCTCAACCTTGAAATCTCAAAATTAAAATGCATTGTTATTTCCTATAACTTTAGCGAAGCCCTTCAAAAGATAACATCCACTTACCAACACAAATCTCCTAGTTCATACACCAATAGCAAACA
GGGTGCAGCCGTCGGACAATTAGTATCAAAAATTGGCAATGATGGGCTATGTGAAGAATATACCCTTGTACTATCTATTGAATTTTTTGCTGAGCCTTTTCAATGA
>EHGDEF_01335 Phage portal protein
ATGGGTAAGCAAAAATCCCGTAAAGCCGCCGCGCAGAAAGCCCGCGCACCACAGCAACTAAAAGCCAGCGCACCGCAAAAATGGAAGCGTTCACCTTCGGTGAGCCGGTCGCCGGTACT
CGATAAGCGCGACATTCTGGATTACGTCGAGTGCATCAGTAACGGCAAATGGTACGAGCCGCCGGTCAGCTTCTCCGGGCTGGCAAAAAGCCTGCGCTCTGCAGTGCATCACAGCTCACCG
ATTTACGTTAAACGCAACGTGCTCGCGAGCACCTACATTCCGCACCCGCTGCTGTCCCGTCAGGATTTCAGCCGCTTTGCGCTCGACTATCTGGTATTCGGTAACGCCTTTCTTGAGCAGCGC
CACAGCGTCACCGGACAGTTAATAAAACTGCTGACTTCACCGGCAAAATACACCCGACGCGGGGTCGATGACTCGGTTTTCTGGTTTGTGGAAAACTTCACTCAGCCGCATGAATTCGCAC
CCGATACCGTGTTCCACCTGCTGGAGCCTGATATTAATCAGGAGATTTACGGCCTGCCTGAATATCTCAGCGCACTTAATTCCGCCTGGCTGAATGAATCCGCAACGCTGTTCCGCCGCAAGT
ATTACCAGAACGGCGCGCACGCAGGTTACATCATGTATGTGACTGACCCGGCGCAAAGCGCGACTGACGTCGAATCGCTGCGCGATGCAATGCGTAACTCTAAAGGGCTCGGCAACTTTAA
AAACCTGTTTTTCTACTCACCGAACGGGAAACCGGACGGCATAAAAATCGTGCCATTGAGCGAAGTCGCCACAAAGGATGACTTTTTCAACATCAAGAAAGCCAGCGCCGCTGACCTCATG
GATGCGCACCGCGTACCGTTCCAGCTCATGGGCGGCAAGCCTGAGAATATCGGCTCAATGGGTGATGTTGAGAAGGTGGCAAAGGTCTTTGTGCGTAACGAGTTATCACCCTTACAGGACA
GATTCAGGGAGGTAAACGACTGGCTCGGCATGGAGGTCATCAGGTTCAAAGAGTACACCCTCGACAACCCGGAATAA
>EHGDEF_01340 Terminase-6C domain-containing protein
GTGTTCCGGGAATACATCATCCAGTTTGCGCGACTGGTTGACGTCGACCTGACCGGCGACCCGATTGTCATCGGCAACAACGGCGCAAAGCTGATTTTTCTCGGCACCAATTCCAACACCG
CACAAAGCCATAACGGCGACCTGTATGTCGATGAAATATTCTGGATCCCGAATTTTCAGAAGCTGCGCAAAGTCGCATCGGGCATGGCCTCGCAAAAGCATCTGCGCTCAACTTACTTTTCG
ACACCTTCCACGCTGGCGCACGGCGCTTACCCCTTCTGGTCTGGCGAGCTGTTCAACAAGGGGCGCGCCAGTGCCGCTGACCGCATCGAAATCGACATCAGTCACAGCGCGCTCGCCGGT

GGGCTTCTTTGCGCTGACGGACAGTGGCGGCAGATTGTCACTATTGAGGACGCCCTTGCCGGTGGCTGCACCCTGTTCGACCTCGACCAGCTCAGACGCGAAAACAGTGATGAGGACTTT
AAGAACCTGTTTATGTGCGAGTTTGTCGACGATAAGGCATCGGTATTCCCGTTCGAGGAGCTGCAGCGCTGCATGGTCGACGTGATGGAAACATGGGAGGACTTCGCCCCGTTCGCCGACC
ATCCATTCGGCTCGCGACCGGTCTGGATTGGCTACGACCCGTCCCACACCGGCGACAGTGCCGGATGTGTCGTACTCGCGCCGCCGGTGGTTTCGGGTGGCAAGTTTCGCATGCTGGAGC
GTCACCAGTGGAAAGGCATGGACTTTGCCGCGCAGGCAGAAGGCATCCGCAGGCTCACTGAGAAATACAACGTCGAATACATCGGCATTGACGCAACAGGCCTCGGTCTCGGCGTATTCC
AGTTGGTGCGCTCATTCTACCCGGCGGCACGCGGTATCCGTTACACGCCAGAAATGAAAACCGCAATGGTGCTCAAGGCGAAAGACACGATTCGCCGTGGCTGTCTGGAGTACGACGCCG
GAGCAACTGATGTCACACAGTCGTTTATGTCCATCCGCAAAACCATGACCAGCAGCGGGCGCAGCGCCACCTATGAGGCCAGCCGCACCGAGGAAGCCAGTCACGCTGATATCGCATGGG
CTACCATGCACGCCCTGTTAAACGAACCGCTTTCTGCCGGTAGCGGCATGCAGCCTAAATCTATTCTGGAGTTCAACTAA
>EHGDEF_01345 hypothetical protein
ATGACCATTTCCACCGATACAACCTTATTGCATGACCCGCGACGACAGGCATCGCTGCTTTACTGGCAGGGCTTTTCCGTGCCACAGATTGCCGAAATGCTGCAGGTCAAGCGCCCGACCGT
GCAGAGCTGGAAACAGCGCGACGGCTGGGACGGCATCGCACCGATTTCCCGTGTCGAAAGCAGCCTTGAGGCGCGCCTGATTCAGCTCATCGCCAAGCCGCAAAAGTCAGGCGGCGACT
TCAAAGAGATTGACCTGCTCGGGCGGCAGATTGAGCGACTGGCGCGCGTCAACCGCTACAGCCAGACCGGCAACGAGGCCGACCTTAACCCCAACGTTGCCAACCGTAACAAGGGGGA
GCGTAAGAGGCCGAAAAAGAACTTTTTCAGCGATGAGGCTGTCGCAAAGCTGGAAGAAATTTTCTTCGACCAGTCTTTCGAATACCAGTTGCAGTGGTGCGGGCAGGACTGGCGCACCG
TATTCGCGATATTCTCAAATCCCGCCAGATTGGCGCGACGTTCTACTTTTCCCGCGAGGCACTGCTGCGCGCGCTCAAGACCGGCCATAACCAGATTTTTCTGTCGGCCAGTAAAACGCAGG
CTTACGTGTTCCGGGAATACATCATCCAGTTTGCGCGACTGGTTGACGTCGACCTGA
>EHGDEF_01350 GPO family capsid scaffolding protein
ATGGCAAAAAAAGTCTCAAAATTCTTTCGCATCGGCGTCGAGGGTGATACCTGCGACGGGCGCATTATCAGCGCCAGCGATATTCAGGAAATGGCCGAAACCTATGACCCGCGCGTCTACG
GTTGCCGTATCAACCTTGAACACATTCGCGGCCTTTTGCCCGACGGCATGTTTAAACGTTATGGCGATGTGGTTGAGCTGAAAGCCGAAAAGATTGACGACGATTCTGCGCTGAATGGCAA
ATGGGCGTTGTTCGCCAGAATCACCCCAACCGATGACCTTATCGCGATGAATAAAGCCGCGCAGAAGGTCTATACCTCTATGGAAATTCAGCCGAATTTTGGTAACACCGGCAAATGCTATCT
TGTCGGCCTTGCGGTCACTGATGACCCTGCGAGCCTCGGCACTGAATACCTCGAATTCTGCCGTAAGGCGAAGCACAACCCGCTGCAGCGCTTTAAGGCCAGTCCTGAAAATGTCTTTTCA
GTCGCCACGCTGGCCGAACTGGAATTTGAAGATGTTCCCGACACAGTGCTTAACAGCCTGGCCGATAAGGTGAAAGCCATTTTCAGCCGTAAGCAGGTCAGCGACGATGCGCGCATGAGT
GATGTGCATGAGGCGGTGACCGCCGTCAGCGAACATGTGCAGACCAGTCTCACTGCGCAGGATAAGCGTCTTTCCGATATGGAAACCGCGCTAGCCACCTTTAAACAGGAACTGACCGGC
AAGGTTGAAGAAACCAGCCAGGCATTTTCCGCCCTGAAAACCACCCTCGACAAAACCGAAAGTTTCAGCCAGCCGCGACGCACGAAAGCCAGCGGCGGCGGTGGCGACGAGCTGCTGA
CCGACTGCTGA
>EHGDEF_01355 Phage major capsid protein, P2 family
ATGCGTCAGGAAACCCGTTTTAAGTTCAATGCCTATCTGACCCAGCTCGCCAAACTGAACGGCATCAGCGTTGATGACGTTAGCAAAAAATTCACCGTCGAGCCGTCCGTCACGCAAACGCT
GATGAACACCGTGCAGGCGTCATCCGCATTTCTGCAGATGATTAACATTTTTCCGGTTGATGAGCTGAAAGGTGAAAAAATCGGTGTGGGTGTGGATGGCACTATTGCCAGCACGACCGAC
ACCTCAGGTGACGATGAGCGTGAAACTGCTGACTTTGTCGCGCTTGAGTCCAATTTGTACGAGTGCAACCAGGTCAACTTTGACTTCCACCTGCGCTACAAGACGCTCGACCTGTGGGCGC
GTTTTCAGGACTTCCAGCGCCGCATCCGTGACGCGATTATCCAGCGTCAGGCACTGGATTTCATCATGGCCGGATTTAACGGTACTCATCGTGCGCCGACCTCGAATCGCAAAACTAATCCAA
TGTTGCAGGATGTAGCGGTCGGCTGGCTGCAGAAGTACCGTAACGAAGCACCGGCGCGCGTGATGAGTAAAATCACCGACGATGAGGGTCAAGTCGTTTCCGATGTGATTCGTGTCGGTA
AAAACGGCGACTATGAGAATCTTGACGCGTTGGTAATGGATGCGACAGGCACCCTGATTGATGAGGTTTATCAGGACTCAACCAAACTGGTTGCCATCGTTGGCCGTAAGCTGCTGGCCGA
TAAATATTTCCCGCTGGTTAACAAGCAGCAGGAAAACAGCGAGTCGCTCGCTGCGGATACCATCATCAGCCAGAAACGCATCGGCAACCTGCCTGCCGTGCGCGTGCCGTACTTCCCTGCG
AATGCTGTACTGGTGACCACGATGGAAAATCTCTCAATCTACTTCATGGATGATGGTCATCGGCGTTCAGTCATCGAAAACCCCAAGCGTGACCGCGTGGAAAACTACGAGTCGATGAATATC
GACTATGTGGTCGAGGCGTATGCCGCCGGGTCGCCTGCTGGAAAACATCACCCTGGGCGATTTCACCGCACCTGCAGCACCGGAAAGCGGAGTCTAAACCATGACGAGCCCCGCACAGCG
TCACATGATGCGGGTCTCGGCCTCTCAAGCCGCGCAGCGGGAGCAAGCCCCACTGCGCCATGCAACCGCCTATGA
>EHGDEF_01360 Small terminase subunit
ATGCTGGTTAAGCTGGCCGATGACCGCCGCACGTTAAAAAACATCCGTTCAAACGAACGTAAAGCCGAGAAAAAGCGCGAGCTGCTGCCGTTCTATGCGCCGTGGGTCGCCGGTGTGCTG
GCTGATGGTCGCGGTGCGCAGGATGACATTGTCATGACCGTCATGCTGTGGCGTCTTGATGCCGGTGATATCGCTGGCGCGCTGGAAATTGCGCCCTATGCGCTGAAATACGGCCTCACCAC
TGACCATCGCCGCACGACGCCTTACATGCTGGTTGAGGAGGTGGCGCTTGCCGCACTGCGCCTGCGCGATGCCGGTGAGCCTGTCGACCTCGCATTACTGCTGACCACCCTCAGCCTGACC
GACGGCGCTGACGTTCCCGATATGGTGCGCGCCCGTCTGCATAAGGTGACCGGCCTGACCCTGCGCGATATCGGTCAGAATGCTGAGGCACTGGCTCAGTTTCAACGTGCGATGCAGCTCG
ACCGCAATGCCGGTGTGCGCAAAGAGATTGAGCGGCTGGAACGCGCACTGAAGCCAAAAGCGGAAAGCACCACCCCGTAA
>EHGDEF_01365 Capsid completion protein
ATGACGACAGTGATTCTGAACCAGCCCGACGAACCGCAGGACGTACCGGGCGTGGTGATTCCCGTACCGGAGACGGGCGATGCAGTAATTAAAAACACGTTCTTTTTCCCTGATGTGGATC
CGAAGCGTGTGCGCGAGCTGATGCGGCTTGAACAGACGGTTTCCGATGCGCGCCTGCGCCATGCCATCAGAACTGGCATGGCGGAAACCAATGCGGAGCTTTACGACTACCGGCTGCGCC
AGACTGCCGCCGGGTTTAAGCATCTGGCCGACGTGCCTGCTGAGGAAATCGATGGCGAGAATGTGCGTATTTTCCACTATCTGAGAGCCGTAACGGCGATGGCAACCGCCACCCTGTATGA
GCGCTATCGCGGTGTTGAAGCCACCGGCAAGGGTGACAAAAAAGCCGACAACGTCGAAACCACCATTGATGACCTGTGGCGGGATATGCGCTGGTCGGTCGCGCGTCTGCAGGACAAAC
CGCGCTGCATCGTGGGCCAGCTCTGA
>EHGDEF_01370 Tail X family protein
ATGCAGGGCGACACCCTCGACGTGATTTGCGCCCGGTATTACGGGCGCACTGAGGGCGTTGTTGAAACTGTGCTGCAGGCTAATCCCGGCCTGTCTGAGCTGGGCGTCATTCTGCCGCATG
GAACGGCAATTGACCTGCCCGATGTGCCGTCTTCACCCGTAACTGAAACTATCAATCTTTGGGAGTAA
>EHGDEF_01375 Holin
ATGACAGAAGGGGAAAAAGGCGTCCTGTCACTGTTTGTGATTGGGGCACTGATTGTGGTCGGAAAAGTGCTGGCAGGTGGTGAGCCCATCACCCCGCGCCTGTTTGTCGGACGCATGTTG
CTCGGCGGTTTTGTCTCAATGGTCGCCGGTGTTGTTCTGGTGCAGTTTCCTGATATGTCACTGCCCGCTGTGTGCGGTATTGGATCCATGCTCGGTATTGCCGGTTATCAGGTGGTGGAAATC
GCCATTCAGCGCCGCTTTAAGTCACAGAAGGGGGAAGGCGATGCCGGTCATTAA
>EHGDEF_01380 Lysozyme
ATGCCGGTCATTAATACTCACCAGAATATCGCCGCCTTTCTGGACATGCTGGCGTATTCCGAAGGAACGGCGAACCATCCGCTGACGAAAAACCGTGGCTACGACGTCATTGTTACCGGCCT
TGATGGCAGGCCAGAGATTTTCACCGATTACAGCGACCACCCTTTCGCACATGGCCGACCCGCGAAAGTGTTTAATCGCGGTGGCGAGAAATCCACGGCATCGGGGCGTTACCAGCAGCT
TTATATGTTCTGGCCGCACTATAAAAAACAGCTCGCATTGCCTGATTTCAGCCCACTGTCGCAGGACAAGCTCGCGATCCAGTTAATCCGGGAGCGCGGTGCTATTGACGATATCCGGGCGG
GGCGTATTGAGCGTGCTGTTTCCCGTTGCCGGAATATCTGGGCGTCATTGCCGGGCGCCGGTTACGGCCAGCGCGAGCACAGTCTCGAAAAGCTGGTTACCGTCTGGCGCACGGCTGGCG
GGGTGGTGGCATGA
>EHGDEF_01385 LysB family phage lysis regulatory protein
ATGAAAGTCCTGATAACGCTGTTTGTGCTGGCCGTGCTCGGTCTGATGTGGTTGCGCCATGAGAATGGCAATTTATCCCGCTCCTTTGAGACGGCAAACCGCGTTGCGAGCGAGCAAAAGG
CGACGATTGGCATGCTGAAAAATCAGCTCAGTGTCGCCGGCCAGCTCGCCCGACGTAATGAATCCGCTCAGGTGGCACTGCGCGAACAGCTCGCAAAGGCAGGCGCAGAAGCAAACCGC
CGCGAGCAGACGATAACGAGGTTACTTGATGAAAATGAAGCCTTTCGCCGCTGGTATAACGCTCCTCTGCCTGATGCTGTGCGCAGGCTGCACACCCGCGCCGCCTGCGCCAGCGCCGGTG
ATTGTGGTCAGCGGATGCCCGAGGGTGAGCCTTTGCCCGATGCCGGGAAGTGA
>EHGDEF_01390 hypothetical protein
ATGCCGGGAAGTGATCCGAAAACTAATGGTGACCTGAGCGCTGATATCCGCCGCCTTGAGGGCGCGCTGACCGCCTGCGCGCTGCAGGTCAAAATCGTCAAACACTGTCAGGATGAACTC
GATGCAGAAGCACAAAAGCCTGCGCAAGGCGCTGATTAA
>EHGDEF_01395 N protein
ATGCAGAAGCACAAAAGCCTGCGCAAGGCGCTGATTAACGCCGTGCCGCAGCTCCGAAACAACCCCGATATGCTGCGCCTGTTCGCTGATAACGGGCATACGGATTCCCGACTGGAGAGC
TCGCTGTCGTTTGAAAAGGTGTACGTGCTTAACGTGGTGGTGACTGACTTTACCGGCGACCTCGATTTGATATTTGTGCCGGTACAGGCATGGCTGCGTGAGCATCAGCCGGACATTATGAC

CACCGACGACGGGCGGGAGAAAGGATTCACATGGATGATTGATATCAATAACGACGATTCGCTCGATATCAGTATCAGCCTGAGACTCACCGAGCGCACGCTCGTCAAAGAGGTCGACGGC
GCATTGCATGTCAGCTATGCCCCTGAGCCACCGCTGCCTGAGCCAGTGACACGCCCGGTCGAGCTGTACGTTAACGGCGAGCTGGTGAGTAAGTGGGATGAGTGA
>EHGDEF_01400 Phage tail protein
ATGAGTGAGTTAACCGCGCTGCAGGAGCGCCTTGCCGGTCTGATTGCCAGCCTGTCACCGGCGGCGCGTCGTCAAATGGCGGCTGACATTGCAAAAAAACTGCGCGCCAGTCAGCAGCA
GCGCATCAGGCGACAGCAGGCACCAGATGACACCCCGTATGCCGCCCGAAAGCGCCAGCCGGTGCGAAGTAAGAAAGGCCGTATCAGGCGCGAAATGTTCGCCAGACTGCGCACTAACC
GCTTTATGAAAGCCAAAGGCAGCGACAGTGCGGCGGTGGTGGAATTTACCGGCAGGGTACAGCGCATGGCGCGGGTGCATCAGTATGGCCTCAAAGACCGGCCAAATCGTCACAGCCGG
GATGTGCAGTACGCGGCGCGCCCGTTGCTCGGTTTCACCCGCGACGATGAGCAGATGATTGAAGACATCATTATCAGACATCTCGGTAAATAA
>EHGDEF_01405 Spike protein
ATGAATACGTTATCCACAATACAGGAGCTCGCGCGCGCGATTCGCAACCTCATCCGCTCAGGTGTGGTGACTGAGGTCGATACCGCGCAGGGGCTGTGCCGCGTACAAAGCGGCGGGATCC
AGACTGCATGGCTGAACTGGCTGACCACCCGCGCCGGTCGTTCGCGGACATGGTGGGCTCCCTCGGTCGGTGAGCAGGTGCTGCTGCTGGCAATTGGTGGCGAGCTTGATACTGCTTTCG
TGCTGCCGGGGATTTTCTCCGACGATAACCCTGCCCCGTCAGCCTCGGCGGATGCGTGGCATATGGTGTTCCCCGACGGCGCTGTTATGGAGTATGAGCCGGAAACCGGTGCACTGACGGT
CAGCGGCATCAAGACTGCCGATGTGACGGCATCGGAGTCCATTACCGCCACCGTGCCGGTGGTACTGGTAAAAGCGGCAGAACGTATCACCCTCGACACCCCGGAGGTGGTATGCACCAA
CAAACTGACGACGGCGACGCTTGAGGTGCAGAAAGGCGGCGCCATGCGGGGAAACATCGAACATACCGGTGGCACGTTGAAATCAAACGGCGTGCAGGTCGATAACCACGGTCACGGC
GGCGTACAACGGGGCGGGAACTGGACGGAGGGCACAAAATGA
>EHGDEF_01410 GPW-gp25 domain-containing protein
ATGACAGCGCGTTATCTGGGGATGAACCGCAATACCGGCATAGGTATCAGTGACAGTGAGCATATCAGCCAGAGCATGCGCGACATTCTGCTGACGCCGGTCGGCTCGCGGGTAATGCGTC
GTGAATATGGCTCGCTCCTGTCTGCGCTGATTGACATGCCGCAAAACCCGGCGCTCAGGCTGCAAATCATGGTTGCGTGCTATTCCGCGATCCAGAAATGGGAACCACGCATCAGGCTTATC
TCCATCAGCTTTGAGCGCGGCGACACTGGCGAAATGTATGTCGATATTACCGGGATGCGTACCGATACCGGTGCGTCAGTTTCAACCACTGTTTCACTGAGTTAA
>EHGDEF_01415 Baseplate-J domain-containing protein
ATGGCAACCGTTGACCTGAGTCAGTTACCCGTTCCAGACGTGGTTGAGGAACTGGACTATGAAACCATCCTTGCGGAACGCATTGCGACGCTGATTTCGCTCTATCCCGAAGACCAACAGG
AAGCCGTCGCCCGGACGCTCGCACTTGAGTCTGAGCCAATTGTTAAATTGCTGCAGGAAAACGCCTACCGAGAGGTTATCTGGCGTCAGCGTGTCAATGAAGCTGCACGCGCAGTGATGCT
GGCTTATGCCATAGACAGTGACCTCGATAATATCGGGGTAAATTTCAGTGTTGAGCGTCTTGTCGTCACGCCTGCTGATGACACCACCATTCCACCCACCCCGGCAGAAATGGAACTCGACG
CCGATTATCGTCTGCGTATACAGCAGGCTTTTGAGGGGCTGAGCGTGGCGGGGCCTGTCGGGGCGTACCAGTATCATGGCCGTAGTGCTGACGGGCGCGTCGGCGATATTTCAGTTATCAG
CCCGTCGCCAGCCTGTGTGACGATTTCCGTGTTGTCACGTGAAAACAACGGCGTCGCATCTGAGGAACTGCTTGCAATTGTGCGCAATGCCCTGAACGCAGAAGATGTCAGGCCGGTCGCT
GACCGGGTGACGGTACAGTCAGCCGAAATTGTTAACTACCAGATTAACGCCACGCTTTATCTTTATCCCGGCCCGGAAAGTGAACCCATCAGGGCGGCGGCTGAGGCAAAGCTGAAAGCC
TATATCAGCGCGCAGCACCGCCTCGGGCGCGATATCCGTAAATCAGCGATTTATGCCGCCCTGCATGTTGAGGGGGTTCAGCGGGTGGAGCTGGCGGCACCGGTCGCAGATATTGTTCTCG
ATAACACACAGGCATCCTTTTGCACTGACTACAGCCTAGTAATCGGGGGATCTGATGAATGA
>EHGDEF_01420 Phage tail protein I
ATGAATGACTCACGATTATTGCCGGTGGGCTCATCGCCACTGGAAGTCGCCGCCGCAAGGGCATGTGCAGAGATTGAAAGGACGCCGGTCAACATCCGCGCGTTGTGGAATATTGACACCT
GCCCGGAAAATTTGTTGCCGTGGCTGGCGTGGGCGTTTTCTGTCGACCGGTGGAATGAGAACTGGCCGGAGGGAACAAAACGTGCCGTTATCCGTGATGCATATTTCATTCACTGCCACAA
GGGGACTATCGGCGCAATCCGTCGGGTAGTGGAGCCTCTCGGCTATGTCATCAATGTAACGGAATGGTGGGAAAGCGGCGACCCGCCAGGTACATTCCGGCTTGATATCGGGGTGCTTGAA
AGTGGCATTACCGAAGAAATGTATTTCGAAATGGAACGACTGATTGCGGATGCAAAACCAGCCAGTCGTCATCTGACTGGCCTGAATATTGTCCAGGACATTCCCGGTTATTTGTATACCGGC
GGCGTGTCCTGCGACGGCGATATTATTACGGTTTACCCGGGATAA
>EHGDEF_01425 Collar domain-containing protein
ATGAGCACGAAATTTAAAACCGTTATCACCACTGCCGGAGCCGCTAAGCTTGCTGCGGCCACCATGCCGGGCGGTAAGAAAATAAATCTTAACGTTATGGCTGTTGGTGACGGCGGCGGAA
AGCTGCCGGATCCTGAAGCTGGTCAGACGCAGCTTGTTAATGAGGTCTGGCGTCACACTCTGAATAAAATCAGCCAGGACAACCGGTACAGTAATTACATTGTGGCCGAGCTGGTTATTCCG
CCGGAGGTGGGCGGCTTCTGGATGCGTGAGCTTGGCCTTTACGACGATGAAGGGACGCTGATTGCTGTTGCCAATATGGCCGAAAGCTACAAGCCAGAACTGGCCGAGGGCTCAGGGCG
TGCGCAGACATGCCGCATGGTCATCATTGTCAGCAGTGTCGAGTCTGTGGAGCTTTTCATTGACTCAACGATGGTGATGGCGACGCAGGATTATGTCGACGACAGGCTCGCCGAACATGAA
AAATCCCGTCGTCATCCTGATGCCACTCTTAAAGAAAAAGGGTTTACTCAGCTCAGTAACGCGACAGACAGCGAGTCTGAAACGCTCGCAGCGACGCCGAAAGCTGTCAAGACAGCGTATG
ACCTTGCTAACGCGAAATATACAGCTCAGGACGCCACCACAACGCGTAAAGGCATTGTGCAACTCAGCAATGCCACTGACAGTACGTCTGAGACACTTGCCGCGACACCGAAAGCGGTCAA
GGTGGCATATGACCTTGCTAACGCGAAATATACAGCTCAGGACGCCACTACAGCGCGTAAAGGGATTATCCAGCTCAGCAATGCCACTGACAGCACGTCTGAGACGCTGGCCGCAACGTCG
AAAGCGGTTAAAGCGGCTATGGATAATGCGAACGGGCGGCTGGCGAAAAACAGTAATGGCGGCGATATTCCTGACAAAAAACAATTTGCGAGAACTATCGGTGCCGTGACCTCTACCAATA
TCACATTTAATAATGCTTCTGGATGGTACAAAATCGCCACAGTTGTAATGCCGCAGGCTACATCAACTGCGGTGATTAAATTATATGGAGGGGCGGGGTTTAACGCTGGTTCATCTGAACAAG
GGGCAATCAGTGAACTGGTACTGCGTGCCGGTAATGGTTCACCTGCTGGAATAACTGCCACGTTGTGGAGACGCTCGCCTGCTGCGGCTAACGAGGTCGCATGGGTTAATACATCAGGCGA
CACCTACGATATTTATATTAATATCGGCCAGTATGCGTACTGGTTAATTGCGCAATATGACTACACCGGTAATGCAAATGTCACGTTGCACAGTACGCCTGAATATTCATCAGTACAGCCGGGGA
ACTCAACCAGCGGTCAGACATATACAATTTACAGTAGTCTGATGAAACCAACAGCCGGTGATGTGGGGGCATTGCCGATTACAGGGGGGCAGCTTAACGGACCGCTGAGTATTGGTACTGA
CAATGCACTGGGAGGTAATTCGATTGTGCTCGGTGATAACGATACAGGGTTTAAACAGGATGGCGACGGCATTCTGGGTATTTACGCCAATAATGCCCGGGTCGGTTATATCGACAATTCCG
GGTTACACATGTCAGTAGATGTTCTCACTAATGGTGGCATACGAGCAGGTGACGGGAAAAGGCTGTCACTGACGAGTAATAATAATTCGACAATGACAGCCACGTTTAATTTATGGGGCGAC
GCAAACAGGCCAACAGTTATTGAACTGGACGACGATCAGGGATGGCATCTGTACAGCCAGCGAAATCCTGATGGTTCGATTGTCTTTACGGTCAATGGCGATATCACCGCTAACACGCTTCG
TGCAGGCGGGGCTATCTATCAGAATAACGGCGACATCTTTGGTTCGCTATGGGGAAATGGCTGGTTAAGTACCTGGATTAATAATAATCTCGTCTTAGATGTTCAGTTAGGGGCTGGCACATC
AGTGACTACCTGGAACAATGCAGGTTCCTGGCCTAACACTCCCGGATATGTAGTTACCTCCGTCTGGAAAGATTATCAGGGCGAAAATATTGATGGTATTAATTATGCGCCTTTGCAAAAACG
AGTCGGGAGTCAGTGGTATACCGTACAAGGGGGAACGGTATAA
>EHGDEF_01430 Tail fiber assembly protein
ATGAAAAAATATCAGAATATCAAAAATTTCAGACTTATTGACGCGCCCGTAAACAGGGATAAAACTCAGGCTGAAATAAATATAGGTGCATATTTTCTGAAGTCGGACGATGGACAGGACTG
GTATGAGTGTCAGTCATTATTTTCTGATGATACTGCAAAAATAATGTACGACCATGAAGGGGTTATCTGGGGTGTCGTTAATAAGCCAGTCCCGCAACGTGGCAACACATATTCTGTATCAATG
TTGTGGCCGGTTAATATGTCTGTTGCGGAAATAGACGCTGCTGACTGCCCTGATGATTGCCGTGGTGATGGTACGTGGTTATATCAGGACGGTAAAGTCGTTCAACGGGGTTATTCTCCGGA
AGAGCTGCGTAAAAATGCCGAGGCTGAAAAAATTCGTCGCCTTTTTGAAGCTGAATCAGCCATCGCACCACTGGCGCGGGCAGTAAAACTAAAAATTGCCACAGATGAAGAGATTAAACG
GCTTGAAGCATGGGAACTCTACAGCGTAATGGTAAACCGGGTGGATACATCTGCGCCTGACTGGCCGGATATACCACGCTAA
>EHGDEF_01435 Phage tail sheath protein
ATGAGTGATTTTCACCACGGCACGCAGGTCATCGAAATTAATGACGGTACGCGTGTCATTTCCACAGTAGCGACTGCAGTCGTTGGCATGGTCTGTACAGCCAGTGATGCAGATGCCACGCT
GTTTCCCCTCAATGAGCCGGTGCTGATTACCAATGTGCAAAGCGCCATTGCGAAAGCCGGTAAAAAAGGCACGCTGGCTGCATCACTGCAGGCCATCGCAGACCAGTCAAAACCCGTCACT
GTTGTTGTACGTGTTGAGGATGGAACCGGCGATGACGAGGAAGCTGCGCTCGCACAGACTGTTTCCAACATTATCGGAGGTACGGATGAGAACGGTAAATACACCGGTATCAAGGCTCTCC
TGACTGCTCAGGCCGTCACCGGCGTCAAGCCGCGTATTCTTGGGGTGCCGGGGCTGGATACTAAAGAGGTCGTGGTCGCGCTTGCGTCGGCTGCCATTAAGTTACGTGCATTTGCTTACGT
CAGCGCGTGGGGATGTAAGACTATTTCCGAAGCGATGGAATATCGTAAAAATTTCAGCCAGCGCGAGCTGATGGTTATCTGGCCTGATTTCCTCGCGTGGGACACCGTCAAAAATACCACCG
CAACGGCTTACGCCACTGCGCGTGCACTCGGCCTGCGTGCTTACATCGACCAGACTGTCGGCTGGCACAAAACCCTGTCTAACGTTGGTGTACAGGGCGTTACCGGCATCAGCGCCTCAGT
GTTCTGGGATTTACAGGCATCCGGCACCGATGCTGACCTGCTCAACGAGGCCGGGGTTACAACGCTGGTACGCAAGGACGGTTTCCGCTTCTGGGGTAACCGCACCTGCTCGGATGACCC
GCTTTTTCTGTTTGAGAACTACACCCGCACCGCGCAGGTACTGGCCGACACGATGGCGGAGGCGCACATGTGGGCGGTCGACAAGCCCATTACCGCCACGCTCATTCGTGACATTGTTGAC
GGCATTAACGCCAAATTCCGCGAGCTGAAATCAAACGGCTACATCGTGGAGGGTAAATGCTGGTTCGATGAGGAATCGAACGACAAGGAAACCCTCAAGGCCGGGAAACTGTATATCGAC
TACGACTATACACCGGTTCCGCCACTGGAAAGCCTGACCCTGCGCCAGCGTATTACCGATAAATATCTGGTGAATCTGGCCGAATCGGTCAACAGCTAA
>EHGDEF_01440 Bacteriocin serracin-P subunit

ATGGCACTACCCCGTAAACTCAAATATCTGAATATGTTCAATGACGGCCTCAGCTACATGGGTGTTGTTGAATCCGTGACGCTGCCGAAACTGACCCGCAAGCTCGAAAACTATCGCGGCGG
CGGTATGAATGGCGCGGCAGCGATTGACCTCGGCCTCGACGATGATGCGCTCACCGTCGAATGGTCTGTCGGCGGCCTGCCTGATGTGGCTCTGTGGGCGCAGTACGCCGCCCCGGGCGC
TGATGCTGTGCCGCTGCGTTTTGCTGGTTCTTACCAGCGCGACGACACCGGCGAAATCGTGGCGGTCGAGGTGGTCATGCGTGGCCGTCATAAAGAAATCGACGGCGGCGAGAATAAGCA
GGGTGAAAACACCTCGACCAAACTGTCGACTGTTTGCACCTACTACCGCCTCACGATTGATGGCAGCGACGTCATCGAAATCGACACCGTCAACATGGTCGAGAAGGTGAACGGCGTCGAC
CGTCTGGAGCAGCACCGCCGCGCAATCGGGCTGTAA
>EHGDEF_01445 hypothetical protein
ATGGCAAAAGCACCACGTAAAACCGCTGAATTTATTGATACGGCTGGCAATGAAATTGACACCGTGAATCCGAATGTCGTGACCCTGGACAAACCAATTAAGCGCGCCGGTCAGACGATTG
ATAAAGTCACCCTGATTGAGCCGAACGCCGATGCCTGCGCGGCGTCAGTCTGGCAGCGGTGGCGCAGTCCGAAGTCGACGCCCTGA
>EHGDEF_01450 putative tape measure protein
ATGAGTAATAACCTGAGGCTTGAGGTATTGCTGAAAGCGGTCGACCAGGCGACCCGACCGCTTAAATCTATCCAGACCGCGAGTAAAATCCTGTCGGGTGATATTCGCAACACGCAAAAGG
GTCTGCGCGACCTGAACGGTCAGGCGTCGAAAATCGACGGCTTTCGTAAGGCAAGCGCGCAACTGGCCGTAACTGGTCAGGCGCTTGACAAGGCGAAGCGTGAAGCCGGTGAGCTGGC
TGTGCAGTTTAAAAACACCACCAGTCCGACCCGCGCGCAGGCGCAGGCGCTCGACGCGGCAAAGCGTGCCGCCTCTGAGCTGCAGACGAAATATAACAGCCTGAGAACATCGGTACAGC
GCCAGCGCTCCGAGCTGATGCAGGCTGGTATTAATACCCGTACCCTGTCTGCCGATGAGCGTCGACTCAAAACTTCCATCAGCGAAACGACGGCGCAGCTTAACCGCCAGCGTGATGCACT
GGCGCGCGTCAGTGCGCAGCAGGCGAAATTAAGCCGGGTGAAAGAACGATATAAATCAGGTAAAGAGCTTGCCGGTAACATGGCCGCAGCAGGCGCTGCCGGGGTAGGTATTGCGACAG
CGGGAACGATGGCCGGGGTTAAATTGCTGATGCCCGGTTATGACTTTGCGCAGAAAAATTCCGAGCTGCAGGCTGTGCTCGGGGTCGAAAAGCAGTCGCCAGAAATGCAGGCGCTACGC
AAACAGGCGCGCCAGCTCGGCGATAATACTGCAGCCTCTGCAGATGATGCAGCGAGCGCGCAAATCATCATTGCGAAAAGCGGCGGTGACGCTGCTGCCATTCAGGCAGCGACGCCAGTC
ACGCTGAATATGGCGCTGTCAAACCGGCGCTCAATGGAGGAAAACGCTGCGCTGCTGACCGGGATGAAATCAGCGTTTCAACTTTCAAACGACAAGATTGCTCACATTGGCGACGTTCTCT
CGATGACGATGAACAAAACCGCCGCCGATTTTGACGGACTGAGCGACGCGCTGACCTATGCCGCGCCGGTGGCGAAAAATGCCGGGGTGAGCATCGAGCAAACCGCCGCAATGGTCGGT
GCGCTGCACGACGCCAAAATCACCGGGTCAATGGCGGGCACGGGTAGCCGCGCCATTCTCAGCCGCCTGCAGGCTCCCACCGGAAAAGCGTTTGAGGCCATTAAGGAACTCGGCGTCAA
AACGTCAGACAGCAAGGGGAACACGCGCCCGATATTCTCCATCCTGAAAGAAATGCAGCGCAGCTTTGAGAAAAACAACCTCGGGACAAGCCAGCGCGGCGAGTACATGAAAACCATTT
TCGGCGAGGAGGCCAGCTCGGCGGCGGCGGTACTGATGGAGGCAGCCTCAAGCGGCAAACTTGACCGGCTCACTGCCGCGTTTAAAGCCTCGGACGGTAAAACCGAGGAACTGGTTAA
GGTTATGCAGGATAACCTCGGCGGTGACTTTAAAGAGTTCCAGTCGGCTTATGAGGCTGTAGGCATCGACCTTTTTGACCAGCAAGAGAGTTCGCTGCGTAAGCTCACCCAAACCGCCACA
CAATACGTGTTAAAGCTCGATGGCTGGATCCAGAAAAACAAAGGTCTGGCGACAACTATCGGCATTATTGCCGGTGGCGCACTTGCTCTGATTGGTATCATCGGCGGCATTGGTCTCGTTGC
GTGGCCGGTTGTCATGGGGATTAACGCCATCATTGCCGCCGCTGGCGTTATGGGTACGGTCTTTACTGTGGCCGGTAGCGCCATTGTGACAGCTCTCGGTGCGATTACCTGGCCGATTCTGG
CTGTGGGGGCGGCGATTGTGGCTGGTGCGTTACTCATCCGTAAATATTGGGAGCCCATCAGCGCATTTTTCTCGGGGGTGATTGAGGGCATCATGAGTGCCTTTGCACCGGTCGGGGAAAT
GTTCGCTCCATTAGCTCCAATCTTTGACGGACTCGGTGAGAAGCTGCGCGGCGTCTGGCAGTGGTTTAAAGACCTGATTGCACCAGTCAAGGCCACGCAGGAGACGCTCGATAGCTGCAA
AAATGTCGGCGTCATATTTGGTCAGGCACTGGCCTCTGCCTTGATGGCTCCGCTCAATGTTTTTAACAAGCTGCGCAGCGGTGTCGACTGGCTTCTCGAAAAACTCGGCATCATCAACAAAG
AATCGGACAGCCTCGACCAGACTGCCGCCAAAACCAATGCCGCCAAGCAGGGTAATTCCTGCATCCCGGCAACCAGCACATATGGCGGCTATCAGGCTTATCAGCCCGTTACCGCACCGGC
GGGGCGCTCTTACATTGACCAGAGCAAAAGCGAATACAACATCACTCTGCCGGGTGGTGTTGCGCCGGGGCATCAGCTTGACCGCCAGTTACGCGACACGCTCGAACAGATTGAACGTGA
TAAACGTGCGCGCCAGCGTGCCAGTATGACCCACGATTTCTGA
>EHGDEF_01455 Phage tail protein
ATGATGCTTGCGCTGGGAATGTTTGTTTTTGAACTCCGTACTCTGCCTTATCAGTCAATGCAGCATTCGAAAGATTACCGCTGGGCGTCTAATGACCGGGTAGGTAAACCGCCTGCATATCAG
TTTCTCGGCGAGGGGGAAACCTCAATACAGCTTGCCGGTACACTTTACCCTGCCATTACCGGCGGTCATATATCCCTGCTGGCTGTGGAACTGATGGCCGATGAGGGCAGGGCGTGGCCGC
TGATTGAGGGGACTGGCAAAATCCTCGGGATGTATATCATCGATAAGGTGTCGACCACGCACGCCGAGTTTTTCAGCGATGGCGCGGCAAGAAAGATTGATTTCACGCTTTCGCTAAAACG
GGTCGATGAATCACTGACGGCAATGTTTGGCGACCTGAATAAACAGGCGAGCGAGCTTCTCGGCTCTGCCGGTAATCTGACCGATAAGCTGCAGGGTGCGCTCGGAGGGCTGACCACATG
A
>EHGDEF_01460 Phage late control D family protein
ATGATTACGGGCATGACCATTGACGCCGGTGCCAGTCTTGCACCGGCATTTATGCTAACGCTGAACAGCCAGGACATTACCAGCAATTTTAGTGACCGGCTGATTTCTCTCACCATGACCGAC
AACCGGGGTTTTGAGGCTGACCAGCTCGACATTGAGCTCGACGATACCGACGGAAAAGTCGAGTTACCCCTGCGCGGGGCGGTGCTGACGCTGTGGCTTGGCTGGCAGGGTTCGGCGCT
ATTGAATAAAGGTGATTTTACGGTCGATGAGATTGAGCACCGGGGCGCGCCTGATACCCTGACCATCCGGGCGCGTAGTGCAGACTTTCGCGGAACGCTCAATTCACGACGTGAAGAATCA
TGGCACGATACCACCCTCGGTGAGCTGGTCAGCACCATTGCAAAGCGTAACAAACTGACGGCCAGCGTCGCGGATTCGCTGAAACAAATCCCGATACCGCATATCGACCAGTCGCAGGAAT
CCGACGCGGTATTTCTTACCCGGCTGGCTGACCGAAACGGAGCGACTGTATCGGTTAAAGCGGGAAAACTACTGTTTCTGAAAGCCGGTAGTGCGCTGACGGCCAGCGGTAAGCCCATTC
CACAAATGACGCTGACCCGCAGTGACGGCGACCGTCATCAGTTTGCCATTGCCGACCGCGGAGCTTATACCGGTGTAACAGCTAAATGGTTGCACACCAAAGACCCGAAGCCGCAAAAAC
AGAAAGTGACGCTGAAACGCCAGCCAAAAGAGAAGCACCTTCGCGCACTGGAACACCCGAAAGCAAAGCCGGTCAGCAAAAAGACGAAGTCCAGAAAAGAGCCGGAGGCTCGTGAAG
GTGAGTATATGGTCGGTGAGGCAGATAACGTGCTGGCACTGACGACGGTCTACGCTTCCAGAACGCAGGCGATGCGCGCCGCTCAGGCTAAGTGGGATAAGCTGCAGCGAGGCGTTGCG
GAGTTTTCAATTACGCTGGCGCTTGGTAGGGCTGATTTATTCCCTGAGACACCGGTGCGCGTGTCAGGCTTTAAGCGCGTCATAGACGAGCAATCTTGGTTAATCAGTAAGGTGACTCACAA
TATGAATAATAGTGGCTTCACGACGGGCTTAGAGCTTGAGGTTAAACTCTCTGATGTGGAGTACAGTGCGGAATCGGATGATGAATAA
>EHGDEF_01465 hypothetical protein
ATGTTTCATTGTCCGAAGTGCCATCATGCCGCACATGCGCGAACAAGCCGCTATCTAACCGAAAATACGAAAGAGCGCTACCACCAGTGCCAGAATATCAACTGCAGTTGTACGTTTATGACA
ATGGAAACGATAGAACGCTTTATTGTTACTCCGGGATCCATCGACCCTAAGACCGCCGCACCCGACTGTTGGTGGTCAGCGACCATTGTGGCTCTGATAAATTTTCGTTAA
>EHGDEF_01470 tRNA-Ile2(cat)
GGCCCCTTAGCTCAGTGGTTAGAGCAGGCGACTCATAATCGCTTGGTCGCTGGTTCAAGTCCAGCAGGGGCCACCA

Prophage 6.1

>EHGDEF_18515 hypothetical protein
ATGTCAGACATAACTGCAAATGCTGTTGTAAGCATGCCTTCTCAGTTGTTCACGATGCCAAGGTCATTTAAGGCTGTGGCTAATGGTAAAATCTATATCGGACAGATTGATACCGATCCGGTAA
ATCCTGCAAATCAGGTTCAGGTTTATCTTGAGAATGAGAATGGAACTCACGTTCCTGTTCCTCAGCCAATTAACATAAACGCTGGAGGTTTCCCTGTTTATAACGGACAGATTGCTAAGTTTGT
AACTGTGCAGGGACATTCAATGGCTGTATACGATGCCAACAATGCTCAGCAGTTTTACTTCCCAAACGTGCTTAAGTACGACCCTGATCAGCTTGAGTACAGACTTAGTCAGCCTGATGGCTA
CTTATTGGTTGGTGGACTGGCTGAGCATTATAACCTTCCGTCATCTGTAATTGTAGTTGATAACGCACCCTATAACGGCGACTTAAAGGCAGCATGGAACGCAGCGCCGGAAGGGGCAACAC
TTCTACTTGGTAAAAAAGATTACAACATCACAGGCTTATGGGCGTCAGGTAGAAATACCAAGAAAAACATCATGATTGTTGGCATGGGCATGCCGGAATATGCGTCGGACTGGAGCAGATTT
GTCAGCGGTTCCGGGACGGTAATTCAGGGTGCAGTGAAGAACCAGGCCAAAGGATTCAAACTTTTCAACCTAGGCGTTGACTGTGGTAATTATGTGTCAACTACGCTGTATAGCACGACAA
CCTATGAAGATGCGGTGCAGATCTATGGAGTCGGTGCGAAAGCAAATATCGGAATTGACAACATCAGGACGCTTAACTCTCTCGGTGTTTCAAGCAATCCTGGCACCCACAGCATTCTGCTT
GAACAACTTGAAGGCGTTACGCTTGGCTACGTAGAGTGTTGCGGTGGTTTCCATGGGCTGACAATCAAGTGTAAGAACTTGCGCGGTGGTCGCGCCCATGTCTATGGACAGTATGGTGACG
GTTTTATTCTAAAATCTGATTCAGGCGGACCATGCAGCGATATCAGAATGGACAGTATCACTATTGGTCTGATTGATAGTTCACTGCTGCCGTCAGTATCTCTCGGTGGAATTTACGATGCTCAC
GACGGGGTTTCCATCGACAACATCAGTATTGGTGACCTTAGAGTGCAAAATGCTTCATGGGGGTTTATTCCTGCCATCGGGGCGGATGGTTATACCAGCCATGTCACAATAGGAAATTATTAT
GCGTCACAGGTTTATGGAAACTATTACTCTCTGGAGGTGGGTAATCAGTGCGTTAACTGGAATATCGGTTCTCACCAGTGTTCAGGCGTTTCAGGGGGCATCAAAATTAACGGATCGGCTCA

ATACATAACATTGGGCGAGGGTTCAGTAACAGGCAGCACAAGATGGGGTTATTCATTTGCTGCATCGACATTTACACATAGCTCACTGATATCAAATGGCAACTATGGTGGGGTTGAGTATCT
CGGTGGTACGGGATTTAACCCAGCTAATGTAATCGCGTATTACAATAACAACGGAAACTTTAGCGCGCTCCCTTCGGTGCTTAACGGGAATGCTTTAAATGGATGGGTGGCGCTATCAGACTT
TAAGGCAACACCAAATGCTCATCAGGTATTCATTAGCGGATCGCTGACAAATGGTACGGCAGCGAATGCGTGGCTTATCGCAGAAAATCTTAGACCGTCTGTTGACACACCTATTTCTGCATG
GGGGGTATCTAGCGGTGGTAGCCTTGTGCCTGTTGAGGCGTATGTGCGAGCAACTGGTTATATTGAGATTACAGGTTATGCATCTCTTGGGGCATCGCAAGCCGTGAGAATCAACGGATCAT
ATCTGATCGCTTAA
>EHGDEF_18520 Lytic transglycosylase domain-containing protein
ATGAAAGTTACAGCCAACGGTAAGACATTCACATTCCCAGAAGGAACAAGCACTGAGGATATTGGATCGGCTATCGATGAGTATTTTGCTGGACAGTCTGCACAGCAGGAACAGCAGGGC
ACATCTACGCCCCCAGAAAGCCAGCCACAGCAACAAGGTGGCTTCATTTCTGACCTTGGCAATGCTGCTGCAGAGACTGGGCGTGGATTACTACAGGCTGGCGTTAATCTGGCAAATATCCC
GGCATCAATGGCTGATGCTGTCGCCAGCGCCGGGGCATGGGCTGGTCAGAAGCTTGGCATTGGTGACGGAACTTATCAGCCAGCGCCTCGTGTCACGACACAAGGACTTGAGCAGGACTT
TGGCTTGCAACAAGGTGCGCTCACTCCACAGACGACAGAAGGTAAAATCTTCTCTGAAGCACTGCCATATTTGACTCCTGTTGGGGCAGAGAGAATTGCAACGCAGGCACCATCTATTGCC
GGTCGAGTTGCTCAGGGTGCATCACGCTTGCTGGCGGAGAACGCTGTTGGTTCATTGGCTGCAAACAGTGAGCGTGATAATCCAGAAACACTGGCAACAGACTTAGGAACTGGTGTTGCA
TTAGGCGGGGCAATCAATCAGTTAGGCCGTGCCGCTGGCGCTGCTTATCGTGGGATTCGCGGGACGATCGCACCAGAAGCGCAACAGGCTATTGAGTTCGCTAATGCTGCTGATGTTCCTT
TGCACACCACTGACGTTTTGCAGCCAAATTCACGCGTCGGCCGCATGGCGCAAACCACAGCTGAAAATATCCCATTTGCTGGGACAAGTACTATGCGAGCTAATCAGCAAGAAGCTCGCAG
TCAGTTGGTAGATGAGTTTGCATCGCGATTTGGTGAGTATGATCCGTCGATTGTGGTTGGTAGTCTGAAAGCAAAAACGTCAGGAATAAAGCGCGCAGCGGGGAATAGGCTGGAACAAGT
GCAAAACGCAATGGCAGGCGTAAACATTCAGCCGTCAAAGGCAATTCAGCAGATTGATACTGAAATAGCCAGTTTGCAGAAACTTGGAAAGGTTGCGGATAACGATACGATTTCTAAGCTT
CAGGCCTATCGCGATGAGCTTACCCGCAATGCTGGAGCAAACGGGCCAATGGCAATGGATTTGCAGCAGTTAAGCGGATTGAGAAGCCAGTTTAGGCAGGATGTTAAAGGAGAGAGAAC
AGTCTTGCCAAACCGATCTGATGCAGCCATTCAGCGTATTTACAATGCAATGACAGGTGATATCGACAGCGCTATCGGACAGAATCTTGGTAATGACACCCTGCGTCGTTATAAGCAAGCTAA
CGCCATATACGCTGACGAAGCTAACAAACTCCAGAATACTCGACTAAAGAACGTGATCATGAAAGGAGACTTAACTCCTGAAGTGGTCAACAACATGCTGTTCAGCAAGAACAAATCTGAG
ATTCAGAATCTATACCGGTCAGTTGGGCAGGTTGGCCGTGCACAAATGCGTAACGGCATCATCGGAAAGGCTATGGAGAAATCAGGCGGTTCTCCTGATCAATTCCTGCGCCAGGTAAATCT
GATGTCTAACCAGACTGGTATAGCTTTTAAAGGCCGTGATGCTGCATATTTGAAAGGGCTGAAAAACTATCTTGAGTCAACCAAGCGTGCGGGGCAGGCAGGTGTAACGACGCCAACCGG
ACAGCAGACAATCCCATTCATCTTAGGAATTGGGTCTGCAACTAACCCAGCGCTAGTTGGGGTCGGTGGTGGTTATGGATTGCTGGCGAGAATGTATGAAAGTGAACCGGCACGAAACGCA
ATGCTTCGCCTGGCTAACACGCCACGTGGTTCTACCGCATTCGAGAAAGCGTTATCTGATGTTGAGCGGGCAGTTAACTCATTCGCTCAGGGTGTAAAATCAGAATCCTTAAGCGAATAG
>EHGDEF_18525 DNA transfer protein
GTGGCTACATTTCAACTCGCCGGACTGCCATCAATGCAGGTGGCAAACCAGAACGCGCCCGGACAACCATCATTATCCAGTTACGACTTCAGCCAGCGTCCAAACGTTGGCGTTCAGCTTG
CTCAAGGTATTGGCTCAGTTGGCCAGGCAATGAGGCTTTCTGACTTTCAAAAAGCTTTCGGTCAGGCTTATGCGGCAGGTGATCGCGACGCCTTGCGTCAACTTGCAGCCACCAATTCAGA
CCAGATTGAAACAATTCGTCAGGGCATGGGGTTTGTTGATGCTGATCGCAATCAGGCGATGGGCGATATGTCTGCACGATTGAACATTGCCGCCGCTCAGGGGCCAGAAGCGGTGATGCG
AGAGCTTGCCACTCACCAGAATACGCTGCAGCAAATTGGCGTATCTCCTGAGCAGGCGTGGCAGACATATCAACAAAGCCCTGAAGGCTTCACGCAGTTAACAGACCTTATTGGAATGCAC
GCGGTAGGACCAGAAAAGTATTTTGATATTCAGGATAAGTTGACAGGTCGCGACATTGACCGAGGTCGCCTTGCTGAAACAATCCGCAGCAATAAAGCCGGTGAGGGGCTTCAGGCTCGC
GGGCAGAATATAACAATGCGTGGACAAGATATGTCAGCGGCAACAGCACGACGCGGTCAAGATTTGGCAACGCAAAGAGCAAACGCCAGAACGATATCAGGCAGCGAAGGAAATCGTGT
CGTTCAGCTTGCAGACGGGCGAACAGTCAGCGTCGGTGGAAAACTTCACGGCGCAGGGGCGAATGCGTTTTACGAAGGTATTGACGATAACGGCAATATGGTTCGTGTCCCGGCAAGCG
CCATTGCCGCACCTCCAACGTCTGCGGCAAGCGCACAGAACTACGCAATGAAGAAAGATATTGATGCAATCGCAAATGCAGATGCTTCTGCTCTCGATTTCATGACTGGAATGACTGGCGGA
GCAGGAAATCCGGCAATTGGTGCAGATGTTCGCAGCCGACTCACAGGCAAAGAGCAACGCCAGTTATATAACTCCGCACAACGTATTCAGGGAAGAATGCAGAATCAGGGCGTGGCAGCA
GCAAGAGATATGGGCGCTAGCGGTATCAACACCATTGCAGAAGCGAAGATGTATTTTCAGGGGATGCCGCAGGTTGACTACTCAAGCCCGGAGGCTATGCAGCAGTCTATTCGTGAGATTC
AGGAATACACCAACAATTATAACCAGCAGTACAACGTTAATGTTGATAATGGTGGGCAGAAATCATCAAGGCAGCAGCCAGCGACTCAGCAATCAGTCGGAGGAAGCTACACGTCTAAATC
CGGCATTCAATTCACGGTGGAATAA
>EHGDEF_18530 hypothetical protein
ATGTTATATGCATTTACGCTGGGCAGGAAACTGCGCGGTGAGGAACCTTCTTATCCTGAAAAAGGCGGTAAAGGCGGCTCATCAAGCAGCGGGGCAAAAGAAGCGGCAAAAGCAACACA
GTATGCAGCAGACCTGCAAAACCAACAATTCAATCGTGTGATGGAACAGTTGGCGACCTTACGCCGCCGCAGGTTTGCCGGCTCTCCAGCAGATTCAGCAACTATCAACACTGGAAGGGCA
GAACAGCGCTCTCAATCAGTATTACAATTCAGACCAGTATAA
>EHGDEF_18535 Protein gp14
ATGATTACATTCACTCCAACACGCAACATCGACCTGATAGAAATGGTTGGCAACCACCCCGACATCATTGCCGGAAGCAACAACGGTGACGGATACGACTACAAGCCTGAGTGTCGTTACTT
TGAAGTGAACGTACATGGTCAGTTCGGTGGCATCGTGTATTACAACGAGATTCAGCCGATGACCTTTGACTGCCACGCCATGTACCTGCCTGAGATTCGCGGATTCAGTAAGGAAATCGGAC
TGGCGTTCTGGCGATACATTCTCACCAATACCACCGTTCAGTGCGTTACATCATTTGCTGCACGCAAATTTCGCCACGGTCAGATGTACTGCGCAATGATTGGCCTTAAGCGTGTGGGAACCA
TCAAGAAATACTTCAAAGGCGTAGATGACGTGACGTTTTACGCCGCCACCCGAGAAGAGTTAACCGAATTACTGAATAACGGGAGATAA
>EHGDEF_18540 Phage tail protein
ATGGCAGACCCGTCACTTAATAAGCCTGTCGTGGTTCAGGCTACACGCATTGATGCTTCAATTCTTCCCCGTAACGTCTTTAGCCAGTCTTATTTGCTCTATGTAATCGCGCAGGGAGCTGACG
TTGGTGCTATTGCAGGCAAGGCAAATGAGGCAGGGAGTGGCGCTTACGACGCACAGGTTAAAAATGATGAGCAGGATGTAATTCTTGATGAGCACGAAAAAAGAATTGCAAAAACAGAA
GAGGATATTTCAGGAATAAAAGTAAAGCTTCTTGAAATAGAGAATGATGTTAATGGTCTGAAAATAAAAGTTGAGGATATCGACGGTAAAGTATCAGAGATAATCGTTGATTATGTTTCACTC
AGCAGAACAGGAACTCAAACTCTTGCCTCATCCCTTAACGTATCAGGAAGTTATTCTGTTAACGGTACAAAAGTTGTTGGCGCTCGCCAGACTGGATGGACCGCGGCAACAGGTACGGCGA
ATAAAGGCGTATTCGATGCTGACCTGACATTCGCCGTTAGCGATACTTACACGCAATCTGAAATCCAGGCTATAGCCAATGCTCTAATTACTGAGCGTCGGCGCACTAAGGCTTTGGAAGACG
CCTTGCGTGCACATGGGTTGATTGATTAA
>EHGDEF_18545 Phage head DNA stabilization protein
ATGCCGATTCAACAACTCCCCATGATGAAGGGAATGGGTAAAGACTTCAAGAACGCCGATTATATCGACTATCTGCCAGTGAATATGCTGGCAACACCCAAAGAAATCCTTAACAGCAGCGG
CTATCTCCGCTCATTCCCCGGCATTACCAAACGTTATGATATGAACGGCGTATCGCGTGGAGTTGAGTACAACACCGCTCAGAATGCTGTTTATCGTGTTTGTGGTGGCAAGCTCTACAAAGG
AGAAAGCGAAGTTGGTGATGTTGCCGGAAGTGGTCGCGTATCAATGGCACATGGTCGGACATCACAGGCGGTAGGCGTTAATGGCCAACTGGTCGAGTATCGCTATGATGGCACGGTTAA
AACCGTCTCAAACTGGCCTGCAGACAGCGGATTCACGCAGTATGAGTTAGGTTCAGTGCGTGACATTACGCGCTTACGTGGGCGTTATGCGTGGTCAAAAGACGGCACTGATTCATGGTTT
ATCACTGACCTCGAAGATGAATCGCATCCTGACCGCTACAGCGCACAATATCGCGCAGAGTCGCAGCCTGACGGCATCATCGGCATCGGAACATGGAGAGACTTCATCGTCTGCTTTGGTTC
GTCAACGATAGAGTATTTCTCACTGACAGGCGCAACCACCGCTGGCGCTGCGTTGTATGTCGCACAGCCATCGTTGATGGTACAGAAGGGCATTGCCGGAACATACTGTAAAACGCCGTTCG
CTGATTCATACGCCTTTATCAGTCATCCGGCTACTGGCGCACCTTCCGTCTACATCATCGGCTCAGGGCAGGCATCGCCAATTGCGACCGCCAGTATTGAGAAAATTATTCGCTCATATACCGCT
GAAGAAATGGCGACGGGTGTGATGGAGACTTTGCGCTTCGATTCTCATGAGCTTCTGATTATTCATCTCCCTCGCCATGTTCTGGTTTACGACGCATCGTCCAGCCAGAACGGACCTCAGTG
GTGTGTGCTGAAAACCGGGCTTTACGATGATGTATATCGCGGCGTCGACTTCATGTACGAAGGAAACCAGATAACGTGCGGCGACAAATCAGAAGCGGTGGTCGGACAATTGCAATTCGAC
ATCAGCAGCCAGTACGACAAACAACAAGAACACCTACTGTTTACGCCCCTTTTCAAAGCAGATAACGCCAGATGCTTCGACCTTGAGGTTGAATCATCCACTGGTGTTGCTCAATACGCTGA
CCGCCTGTTCCTGTCTGCAACAACTGACGGCATCAATTACGGTCGTGAACAGATGATTGAGCAGAATGAGCCGTTTGTGTACGACAAGAGAGTTTTATGGAAGCGTGTAGGGCGTATTCGTA
GATTAATCGGATTCAAACTGCGGGTAATCACCAAATCACCAGTAACACTATCCGGGTGTCAAATTCGTCTGGAGTAA
>EHGDEF_18550 hypothetical protein
ATGCAGATAAAGACTAAAGGCGATCTGGTCAGGGCTGCGCTTCGTAAGTTGGGCGTGGCATCAGATGCAACCCTTACCGATGTCGAACCTCAGTCTATGCAGGATGCCGTTGATGATCTGGA
AGCGATGATGGCGGAGTGGTATCAGGACGGGAAAGGCATCATTACCGGCTATGTATTCTCAGATGATGACAATCCTCCTGCTGAAGGTGACGACCATGGCCTTCGCTCCAGCGCAGTCAGC
GCCGTATTCCATAATCTGGCCTGCCGCATTGCTCCTGATTATGCGCTTGAGGCTACTGCCAAAATTATCGCCACTGCTAAATACGGAAAAGAGCTTCTCTATAAGCAAACCGCCATTTCCAGAG
CCAAACGAGCACCTTACCCGTCACGCATGCCGACAGGCAGTGGAAACAGTTTCGCCAATCTGAAAGAATGGCATTATTTCCCCGGAGAACAGAATGCCGATTCAACAACTCCCCATGATGA
AGGGAATGGGTAA

>EHGDEF_18555 hypothetical protein
ATGAAAATAGCAATCTATAAGCCCGGTGGAAGCATCATGGTATGGGGCGTCATGGCTCAGATGAAGGTCATCGACTCCAGCGAACTTCCGGAATATGTCAAAGATGGCTGGCTTGATCATCC
ATCAAAGCTGCTGCCCGTGGAAGCAGATGATGTTAAGCCACGCAAAGGCCGCAAGCCTAAGGCGGTAAGCGATGCAGATAAAGACTAA
>EHGDEF_18560 hypothetical protein
GTGCTGACCAAATCTACTGCAACTGGTATCACTGTATCCGGTGCTCAGTCCTTCAAGCCTGTCGCATGGCAACTGGATAACGATGGCAACAAAGTTAACGTTGATAACCGTTTTGCTACCGTC
ACCCTGTCTGCAACTACCGGCCTAAAACGCGGCGACAAAATTTCGTTTGCTGGCGTGAAGTTCCTTGGTCAGATGGCTAAGAACGTACTGGCGCAGGACGCGACTTTCTCCGTAGTTCGCG
TTGTTGATGGTACTCACGTTGAAATCACGCCGAAGCCTGTAGCACTGGATGATGTTTCTCTTTCTCCTGAGCAACGGGCATACGCCAACGTTAACACCTCGCTGGCTGATGCGATGGCGGTG
AACATCCTGAACGTTAAGGATGCTCGCACCAACGTGTTCTGGGCTGATGACGCCATCCGTATTGTGTCTCAGCCGATTCCGGCCAACCACGAACTGTTTGCAGGCATGAAAACTACCTCATTC
AGCATCCCGGATGTCGGCCTTAACGGTATCTTCGCGACGCAGGGGGATATTTCCACCCTGTCCGGCCTGTGCCGTATCGCGCTGTGGTACGGCGTAAACGCGACACGACCGGAAGCAATAG
GTGTTGGCCTGCCTGGTCAGACTGCGTAA
>EHGDEF_18565 hypothetical protein
ATGGCTTTGAACGAAGGTCAAATTGTTACACTGGCGGTAGATGAAATCATCGAAACCATCTCCGCAATCACTCCAATGGCGCAGAAAGCCAAGAAATACACCCCGCCTGCTGCTTCTATGCA
GCGCTCCAGCAATACCATCTGGATGCCTGTAGAGCAAGAGTCACCCACTCAGGAGGGCTGGGATTTAACTGATAAAGCGACAGGGTTACTGGAACTTAACGTCGCGGTAAACATGGGAGA
GCCGGATAACGACTTCTTCCAGTTGCGTGCTGATGACTTGCGAGACGAAACTGCGTATCGTCGCCGCATCCAGTCTGCCGCTCGCAAGCTGGCGAACAACGTTGAGTTGAAAGTCGCAAAC
ATGGCCGCCGAGATGGGTTCGCTGGTTATCACCTCCCCTGATGCCATCGGCACTAATACCGCAGACGCCTGGAACTTTGTGGCCGACGCAGAAGAAATCATGTTCTCCCGCGAACTTAACCG
CGACATGGGGACATCGTACTTCTTCAACCCTCAGGACTACAAAAAAGCGGGTTACGACCTGACCAAGCGTGACATCTTTGGGCGTATTCCTGAAGAAGCATACCGAGATGGCGCACCATTC
AGCGGCAGGTTGCAGGCTTCGATGATGTCCTGCGCTCTCCGAAACTTCCTGTGCTGA
>EHGDEF_18570 Scaffolding protein
ATGGAACCAACCACCGAAATTCAGGCAACTGAAGACTTAACCCTGTCCGGCGATCATGCAGCGGCATCTGCTGATAGCTTAGTTGTCGATAATGCCAACGACAATGCAGGTCAGGAAGAGG
GCTTTGAGATTGTCCTGAAGGACGATGAGACAGCACCAAAACAAGACCCGGCAAAGAACGCAGAATTCGCCCGCCGCCGCATCGAGCGCAAACGACAGCGCGAGCTTGAGCAGCAGAT
GGAGGCAGTTAAACGCGGAGAATTGCCGGAGAGTTTACGGGTAAACCCTGACCTTCCACCTCAGCCGGATATTAATGCCTATCTGTCAGAAGAAGGCCTGGCTAAATATGACTACGACAAC
AGCCGTGCGCTTGCCGCTTTCAATGCTGCTAATACCGAATGGCTAATGAAAGCGCAGGACGCCCGCAGCAATGCCGTAGCAGAACAGGGCCGCAAGACTCAGGAGTTTACCCAGCAATCA
GCGCAATACGTCGAAGCTGCCCGCAAACACTATGACGCGGCGGAAAAGCTCAACATCCCTGACTATCAGGAGAAAGAAGACGCATTTATGCAACTGGTTCCGCCTGCGGTTGGGGCCGAC
ATTATGCGCCTGTTCCCGGAAAAGTCCGCCGCGCTCATGTATCACCTGGGGGCAAACCCGGAGAAAGCCCGCCAGTTACTGGCGATGGATGGGCAGTCCGCGCTGATTGAACTCACTCGAC
TATCCGAACGCTTAACTCTCAAGCCTCGCGGTAAACAAATCTCTTCCGCTCCCCCTGCTGACCAGCCGATTACCGGTGATGTCAGCGCAGCAAATAAAGATGCCATTCGTAAACAAATGGATG
CTGCTGCGAGCAAGGGAGATGTGGAAACCTACCGCAAGCTAAAGGCAAAACTTAAAGGAATCCGATAA
>EHGDEF_18575 Portal protein
ATGGCCGACAATGAAAACAGGCTGGAGAGTATCCTGTCGCGCTTTGATGCGGACTGGACAGCCAGTGATGAAGCCAGACGAGAGGCAAAGAATGATCTCTTCTTCTCCCGTGTATCTCAGT
GGGATGACTGGCTATCACAATACACAACCCTACAATATCGCGGGCAGTTCGATGTGGTACGTCCTGTGGTGCGCAAGCTCGTTTCTGAGATGCGTCAGAACCCTATTGATGTTCTGTATCGTC
CAAAGGATGGAGCAAGTCCTGACGCTGCTGATGTGCTGATGGGCATGTATCGCACCGACATGCGGCACAATACAGCGAAAATTGCTGTCAACATAGCCGTTCGTGAGCAGATTGAAGCAGG
CGTGGGTGCGTGGCGTCTGGTCACTGACTACGAAGACCAAAGTCCAACGAGCAACAATCAGGTTATCCGTCGAGAGCCTATCCATAGTGCCTGCTCCCATGTTATATGGGACAGTAACAGCA
AACTGATGGACAAGTCTGACGCCCGTCACTGCACAGTTATCCACTCAATGAGCCAGAATGGCTGGGAGGATTTCGCAGAAAAATACGACCTCGATGCTGATGATATTCCATCATTCCAGAAC
CCCAACGATTGGGTATTTCCATGGCTGACGCAGGACACAATTCAGATCGCTGAGTTTTACGAAGTGGTCGAGAAGAAAGAGACGGCGTTTATCTACCAAGACCCGGTTACGGGTGAGCCG
GTAAGCTACTTTAAGCGCGATATTAAAGACGTCATCGATGACCTGGCTGATAGTGGATTTATCAAAATTGCAGAGCGCCAGATTAAGCGTCGCCGGGTATACAAATCGATTATCACCTGCACT
GCTGTACTCAAAGACAAGCAGCTCATTGCTGGCGAGCATATCCCCATTGTTCCGGTGTTCGGAGAGTGGGGCTTCGTTGAAGATAAAGAAGTGTATGAGGGTGTCGTCCGCCTGACAAAAG
ACGGCCAGCGTCTGCGCAACATGATTATGTCGTTCAACGCCGACATCGTGGCCCGCACTCCGAAGAAGAAGCCGTTCTTCTGGCCTGAGCAGATTGCAGGCTTTGAGCATATGTACGACGG
TAACGACGATTACCCATACTACCTGCTCAATCGCACTGACGAAAATAGTGGAGACCTTCCGACTCAGCCGCTGGCATATTATGAAAACCCGGAAGTGCCGCAAGCCAACGCCTACATGCTGG
AAGCAGCAACCAGCGCAGTAAAAGAGGTTGCCACTCTCGGAGTTGATACAGAAGCGGTAAATGGCGGACAGGTTGCGTTTGATACCGTCAATCAACTGAATATGAGGGCTGACCTTGAGA
CATACGTGTTTCAGGATAATCTGGCTACCGCCATGCGCCGTGACGGAGAGATTTACCAGTCGATAGTTAATGACATCTACGATGTTCCTCGCAACGTTACGATTACCCTTGAGGATGGCAGCG
AGAAAGATGTTCAGCTAATGGCTGAGATTGTTGACCTTGCTACTGGAGAAAAGCAGGTACTAAACGATATCAGGGGGCGCTATGAGTGCTACACGGATGTTGGACCATCATTCCAGTCCATG
AAGCAGCAAAACCGCGCAGAAATTCTTGAGTTGCTCGGCAAGACGCCACAGGGAACGCCAGAATATCAACTGCTGTTGCTTCAGTACTTCACCCTGCTTGATGGTAAAGGTGTTGAGATGA
TGCGTGACTATGCAAACAAGCAGCTTATTCAGATGGGCGTTAAGAAGCCAGAAACGCCCGAAGAGCAGCAATGGTTAGTAGAGGCGCAACAAGCCAAACAAGGTCAACAAGACCCGGCA
ATGGTTCAGGCTCAGGGCGTACTCCTGCAGGGGCAGGCTGAACTGGCTAAAGCTCAGAACCAGACGCTGTCCCTGCAAATCGATGCAGCTAAAGTCGAAGCGCAGAACCAGCTTAACGCT
GCCAGAATCGCAGAAATCTTCAACAACATGGACCTCAGTAAACAATCTGAGTTTAGAGAGTTCCTTAAAACCGTTGCTTCATTCCAGCAGGACCGCAGCGAAGACGCTCGCGCAAATGCTG
AGTTACTCCTTAAAGGCGATGAACAGACGCACAAGCAGCGAATGGACATTGCCAACATCCTGCAATCGCAGAGACAAAATCAACCTTCCGGCAGTGTAGCCGAGACACCTCAATAA
>EHGDEF_18580 Terminase
ATGTCATCAGAATTTGAGGCAATGCTTGCCGATGATATGGGGCGATTCTTCTATGACCCACTCGGATTTGTGATGTATGCATTTGAGTGGGGAGTTGGCGAGCTTGATGGCTTCGACGGTCCA
GATGAGTGGCAGAGAGAATTTCTCACTGATTGGGGTGAGGCGATTCGCACTAATAACTTTGATGGTGTAAAGCCGGTAGAAGCATACCGATGCGCAACAAGCTCAGGCCACGGCATCGGG
AAAAGTGCGCTCACTGCATGGGTAATTCTCTACATCATGAGCACCCGCCCGTTCTGCAAGGGCGTTGTAACAGCTAATACCTCAGAGCAGCTTCGAACCAAAACATGGGGCGAGCTTGGAA
AATGGAAGAAGCGTTGTATCACCGGGCACTGGTTCGAATACAACAACGGCAAAGGCAACATGAACATCTACCATGTAGATCACATGGAGTCATGGCGTTGTGACGGCCAGACCTGCCGCGA
GGAAAACAGTGAATCATTTGCTGGTCTTCATGCTGCAAACTCAAGCCCATTCTACATCTTCGATGAAGCTTCTGCAGTCTCTGACAAGATTTGGGAGGTGGCAGAGGGCGGACTGACAGAC
GGAGAGCCTTTCTGGTTCGCGTTCGGCAACCCGACACGTAACACCGGGCGTTTCCGCGAATGTTTCCGTAAATTCAAGCATCGCTGGAGACGCAAGCAAATTGATAGCCGTCTGGCGAAG
ATGACGAACAAAGAGCTTATTGAAGAATGGCGCAACGATTACGGTGAGGATAGCGACTTCTTTAAAGTACGCGTTCGTGGCCTCTTCCCGTCTGCGTCTGACCTGCAATTTATTCCCCAAAG
CTATGCTGATGCCGGTATGTCAAGAAAGCTGGAGCACAGTCAATATGGATTCGCTCCAAAGATTATCGGCGTTGACCCGGCATACTCAGGCAGTGATGAGGCATGTATCTATCTGCGGCAGG
GGCTTTATTCAAGGCTTTTAGGCTCTTACCCTAAAACAGACGACGATGTGAAGTTTGCTCAGGTAGTGGCTGCTATCGAGGATGAACACAAAGCTGACGCGGTGTTCATTGATTTCGGTTAC
GGCACGGGTATTCATTCTGTTGGTAAGTCGTGGGGCAGAAAGTGGCAACTTGTGAGCTTCGCGGGAGAATCGAAAGACCCGGCAATGCTCAATAAGCGCGGCGAGATGTGGAACGCAAT
GAAATCCTGGCTGAATGAAGGCGGAAGCATTGATGACCAGCAGACCGCTGATGAGATTGTCGCCCCTGAATACAAAGTAAAGCTAGACGGTAAGATTGTTCTGGAGTCGAAAGACGATATG
AAACGCCGTGGCGTTCCCTCTCCAAACAGGGCCGATGCGCTGGCGCTGACATTTGCATTCCCGGTAGTCAAAAACAAACCATCAAAAGCAATCCCCGCACCGATTAGACCAGTACGCAGAG
GACGATAA
>EHGDEF_18585 Terminase
ATGGCGGCTGAAGATAAGAAAATTGGTCGCCCATCGGCTTACAAACCAGAGTATGCCGAGCAGGCGAGAAAACTTTGTCTGTTAGGGCATACAGATGCGGAACTGGCTTCTTTCTTTGATG
TTAGCGAGCAAACAATCAATGCGTGGAAGCACGCGCATCCTGATTTTCTTGAGTCCATAAAAAAATGGTAAGGCTGTTGCGGACAGTGAAGTCGCCGCAAAGTTATTCCACCGCGCCACCGG
GTACGAACACCTAGAAGATGATATTCGCGCTGTCGATGGCTCGATCGTCATAACTCCTACTGTGAAACATTATCCACCCGATACGACTGCTGCCATTTTCTGGCTTAAGAACAGGCAGCGAGA
TAAATGGCGTGACAAACAGGAAGTAGAACACACCGGAGAGGTTAGCCTGATTCAGCGCATTCAGGAGGCCCGTAAACGCGCAAGGGGTGAGTGA
>EHGDEF_18590 Decoration protein
ATGGCAAATCCAAATTTCACGCCATCGTGGCCTCTCTACAAAGATGCTGACGGTGCATATGTGTCTGCTCTTCCGATTAAAGCTATCAAATACGCTAATGACGGAAGTGCAAGCGCAGAATTC
GATGGTCCGTATGCTGACCAGTACATGTCAGCGCAAACAGTGGCCGTATTCAAGCCGGAGGTCGGTGGATATCTGTTCCGAAGCCAGTACGGCGAGCTGCTCTATATGAGCAAGACAGCAT
TTGAAGCTAAGTACACTTCCGCAAGCGGTTCAGTAACGAATGCAGATACGGCGGATAAGTTGTCAACGGCCCGTACTATCACACTAACCGGCGCTGTCACAGGTTCAACGTCATTTGATGGT
TCGGCTAACGTGACTATCGCAACTACCCAAGGAAGCTAA
>EHGDEF_18595 DUF2560 domain-containing protein

ATGGCAGAGATTACAGCATTGACAGAATTACAGCAGATGAACCTCGATATCCTCCGTTTAGTTCAAAGCGATACCGCAGCAGCAGAGAAAGCGATCGCATTCGTTGCTGGAAGTAAGCTGA
ACTTCGAACTGTTCAAAGACCAACTGGTTTTGGCGCAGGGTGAAGGAACGGCATTAGCTCGCGCAGAAAAGGCTATTCGTGAGGCAAAAGAAGCGTTAGACCTGTTCACTGCCGGAGTAT
AA
>EHGDEF_18600 DUF2829 domain-containing protein
ATGACCCAGCATATTGGCGTAAAACTGATTAACGCCTTTCCGATGACGAGACAGGCATATAACGATTTTCGTGGCTGGCAGCTTCCAGCCGGAGAAAACGGCGAGGACGAAGGCTATCTGG
TTGAATATCTGGATGGCGGAAAACCTAACACCGATCGCTTTGATGGCTACGTTATCTGGAGTCCAAAAGAAGTATTCGAAAAGGCTTATCGTCCGGTATCAGGGCTAAGTTTCGGCCTTGCC
ATTGAAGCGCTCAAGTTGGGGAATAAAGTTGCCCGTGCTGGCTGGAATGGTAAGGGTATGTGGCTGGCATACGTTAAACCGTACACTGAGGCTGTTCATACTGGCAGTACGCCTTGCTTTTG
CAGTCGCGTCTTTGAGTTGCCTGAAGGTACGCATGGAGAACCGAAACGATCTCAGAAACAACTTCCGTATATCGCCATGAAAACAGCGGACGATAAATTAGTGCCGTGGCTGGCTAGTCAG
ACTGATGTTCTAGCAGAAGACTGGCAAATCATTACCATGTAG
>EHGDEF_18605 Lysis protein
ATGAGCAGATTAACCTCGATTATCTCCGCTCTGGTTATCTGCATCATCGTCTGCCTGTCATGGGCTGTTAATCATTATCGTGATAACGCCATCACCTACAAAGTCCAGCGCGACACTGTTACTCA
AAAGCTGGCGCTGGCGAACGCGACAATTACCGATATGCAAACGCGCCAGCGTGATGTAGCAGAACTTGACGCCAGATACACAAAGGAGCTTGCTGATGCTAACGCGACTATCGAAAGTCTC
CGTGCTGATGTTTCTGCTGGTCGTAAGCGCCTGCAAGTCGCCGCCACCTGTGCAAAGTCAACGACCGGAGCCAGCGGCATGGGCGATGGAGAAAGCCCAAGACTTACAGCAGATGCTGA
ACTCAATTATTACCGTCTCCGAAGTGGAATCGACAGGATAACCGCGCAGGTTAACTACCTGCAGGAGTACATCAGGACGCAATGCCTGAAATAA
>EHGDEF_18610 Lysozyme RrrD
ATGGCAATGTCACCGGCACTACGAAATAGCGTAATGGCGGCGATAAGTGGCGGGGCTATTGCTATAGCATCTGTGTTAATCACTGGCCCCGGTGGTAACGATGGTCTGGAAGGTGTCAGATA
CAAACCATATAAGGACGTAGTCGGTGTGTTGACTGTGTGTTATGGCCACACCGGAAAAGACATCATGCCTGGTAAAACGTATACCGAAGCAGAATGCAAAGCCCTCCTGAATAAAGACCTTA
TCACTGTCGCCAGACAAATTAACCCGTACATCAAAGTAGATATACCGGAAACAACGCGCGGCGCTCTTTACTCGTTCGTTTACAATGTGGGCGCAGGCAATTTCAGAACATCGACGCTTCTTC
GCAAAATCAATCAGGGTGATATCAAAGGTGCATGTGATCAGCTACGTCGCTGGACATACGCTGGCGGTAAGCAATGGAAAGGCCTGATGACTCGTCGTGAGGTTGAGCGTGATGTCTGTTT
GTGGGGTAAGCAATGA
>EHGDEF_18615 Holin
ATGTACCGTATGGACAAAATCAGAGAATGGTTCAGTTACAGCTTCGGAGGACTGACTGCGATGGGTGGCATTCTCTCCCTGAATGACTGGGCTGTCATCATTGGTATTCTTTGTACTGTCGGC
ACATTTGGCATCAACTGGTACTACAAACGCAAAGAGCGTGAGGACAGATTGAATGGCAATGTCACCGGCACTACGAAATAG
>EHGDEF_18620 tRNA-Thr(tgt)
GCTGGTTTAGCTCCAATGGTAGAGCAGTCGCCTTGTAAGCGAATGGGTAGCGGTTCAAGTCCGTTAACCAGCACCA
>EHGDEF_18625 tRNA-Asn(gtt)
GGGTCAGTCGTATAAAGGTCATTACGGAAGGCTGTTAACCTTCTTATCGTGGTTCGAGTCCACGCTGTCACGCCA
>EHGDEF_18630 Antiterminator
ATGAGTATACGAGAATTGAACCTCACTAAAGAGCAGCATGACTGGCTTAATGGGTGGCTTGAGCTATGGGGGGCATGGGTTTATTCAGGAAGACTCGAAAAACGCATGAGCAGCGTTATAG
CGCAGTTTATGGAGAGGGTAGAACCATCAAGAGTGATGACAAGGCCAATGTGCAATGATGATGACGGAATGTTGATTTCTCAGGTCGTAGATTCCGTTATGCGCATCGACACAAAGGCCTTT
GGCATTCTGCTTACCTATTACGCACATGGTTCCTCTAAGCGAGCAATTGCATCCTACTATCACGCGACTGCAAAGCCACGCAAGATGTGTGGACGTGGTGGTGAGGGATGGAGAAAACCTTC
ACTGGCAACCTGTAGAAATGAAATTGACGACATCCTGAAAGCGTCATTATTTTTTTTATACCAGCCAATGCAAAATGCTTTCAAAATGCGTAAACGTGTTGAGAAAGTTAAGCATATTGTTGTT
AAAAGTCTTGACATGCAATTATCCATTTAG
>EHGDEF_18635 hypothetical protein
ATGACCGTAACCAGCATTAACCAGGCGAAACAGCAGCGTGAACGTGACGAGGCTGAATTACGCAGCGTCAGAGAGATGACGGAGCAACACCAGAAGGCGATGGATTATCTGCATGAGCG
AGAGCGTGAACTGGTGAACCGGCTTGGATTGAACAAGCCGTCGGGAGGCAATGCTGCATGA
>EHGDEF_18640 hypothetical protein
ATGACGTTCTCAGTAAAAACCATTCCAGACATGCTCGTTGAAGCATACGGAAACCAGACAGAAGTAGCACGGCGCTTATCGTGCCACCGCAACACAGTCAGGCGTTATCTGTACGACAAAG
AAGCCAGGCATCACGCCATCGTTAACGGCGTTTTAATGATTCATCAGGGCGGGAGAGGTGTCTATGACCGTAACCAGCATTAA
>EHGDEF_18645 hypothetical protein
ATGAGGAAGGCAAGGCGGCGTTGTAAGAACGAAGAGTGCAGGGAATGGTTCTTCCCGCAATTTCAGAACCAACAGTGGTGTTGTGTTGATTGTGGTACGAAGTTAGCACTCGAACGACG
AAGCAAAGAGCGCGAAAAAGCAGAAAAAGCAGCAGAGAAGAAACGACGACGAGAGGAGCAAAAACAGAAAGATAAACTGAAGATTCGAAAACTCGCCTTAAAGCCCCGCAGTTACTG
GATTAAACAAGCCCAACAAGCCGTAAACGCCTTCATCAGAGAAAGAGACCGCGACTTACCATGTATCTCGTGCGGAACGCTCACGTCTGCTCAGTGGGATGCCGGGCATTACCGGACAACC
GCTGCGGCAGACCTCAACTCCGATTTGATGAACGCAATATTCACAAGCAATGCGTGGTGTGCAACCAGCACAAAAGCGGAAATCTCGTTCCGTATCGCGTCGAACTGA
>EHGDEF_18650 hypothetical protein
ATGATTGACCCCAATCGAAGTTATGAGCAAGAGAGCATAGCAAGGGCAATGTGCGCAGGATGTAACAAGCAACTGGCACCTGATGAAATTTACGCCTGTGCAGAATGTGTTAACGAATGGC
TGGTATATCGCGATCCGAATGGAGATATGTCGAATGAGGAAGGCAAGGCGGCGTTGTAA
>EHGDEF_18655 Recombination protein NinB
ATGAAGCAAATATACATGCTTCGCAACGAAGCAATCAGAAATAACGCCATAGACGCAATACTATCACTACCCATCGACGACAAGTCACCCCACGAAGTCCACGTCAAAGAACCTAAGCGAAC
CAAAGCGCAGAACGACCGTATGTGGCCGATGCTTCAGGACGTTTCCCGTCAGGTGCTATGGCATGGTCAACGACTGTCTCCAGAAGACTGGAAAGACATCTTCACCGCGCTGTGGCTCAAG
ACTAAAAAGCTGGAGCAAAGAAGCGTACCCGGTATTGACGGCGGTATTGTTCTTCTTGGGGTACGTACCAGCAAAATGAGGAAGGCCAGCATGACAGAGCTTATCGAAATCATGTTCTGGT
TCGGATCAGAACGTAACGTGCGATGGAGTGATGATTCCCGGCGTGAGTATGAATGGTCACAACGAACAGGCAGGGCAGCATGA
>EHGDEF_18660 Phage-ABA-S domain-containing protein
ATGGACGAATCAAGAAAGCAGTTTGAAGAGTGGTTTAAAAACAAATATCACGTTTCAAGTGACGTGATGAAGATTATGCATATCAAGGTCGAGATTGCATGGGAGGCGTGGCAGGCATCGC
GAGCAGCTATTGAACTGGATATCGACTGGCCCGAATCGAATGACGACTTTTGGAGAGATGGTGAAGAAGGTGCTTATGCGATGGGTTATGAGGATGGGCGTGACAAAACGGTAATTGCAG
TAATGAAAGCTATCAGAGCCGCTGGAATTAAAGAGAAGAATTTCGATGAAGCAAATATACATGCTTCGCAACGAAGCAATCAGAAATAA
>EHGDEF_18665 hypothetical protein
GTGAGCAAGTACGAAAAATTAGATCAAAACATTCTTTCAATGCTGAGTGAAAGACCAACACCTGTTTTTGATATCTGGCTTAAATGGCGGAGCAATGGAATGTATATCGAAACCATCGATCGC
CGTATGCAATACCTGAGAAAGAAAGGGCTTGTTGCAAATGTGCGTGGGAAGGGGTGGGTGAAAATTAACCTGTCATAA
>EHGDEF_18670 Prophage protein
GTGGCTGACTGGCAAATTCCAATCGTCATTCTTGCCGGAGCTTCGCTGGTTGCTGGCTTTATCCTGCTGAAGAAGCATAAAGACCGTGATCAAAAAGTCGAAGTTCTCTATGGGTATCCAGC
GAACAGCACAACATGGCTGACCATTTACCACTACCGAAAATCAGGCCGCTGGGTATTCGAATGGGATGATCTGTTCGCTGAAAAGCGACCAAAGTCATGGGGAGACATCAGCGAATGCATG
ATGTTTGAAGAAAGAAAATCCGGCGCAACCCGAGAAGAGTTTAACGAAGCGTGGGCGCGATTAAGTGAGAGAGGGTATTTGTGA
>EHGDEF_18675 Transposase
ATGAACAACCAAATAATACCTGAAATGCTTTTGAATCCCCGCTTCATTGCTGTTTTGAACAGATGTATCGACGAAGAAGAGCTCATTATGCAATTTGAAAGGTTGTCAGGTGTCACTCGACCA
CCAAAGGGGCAACATCCAATAGAGCTGATGGTTGATAAAGCGACAGGATTTTCTGATGAGCAGTGGAAACGGTTTTTTGAAGCATTTATCCCGTTCGTCTATGAGTTTATATGGCTCACATGG
AGAGACCGTGACAATGAGGAGTGCTGGCAATGA
>EHGDEF_18680 Replication protein P
ATGACGCCAAGTGAACTTAGCGACCTGCTATGGTCGCAGGTTGACAGGGTGGCTCCGCACCTGTTGCCAAACGGCAAGAAAGACGGGCATGAATGGGTTGCTGGTAACGTCAACGGCGA
CAAGGGGAACAGTCTGAAGGTTAACCTTAGCGGAAAGAAAAAATGGGCTGATTTCGCTGAGGGAGACGGCGGTGACATGCTTGATTTGTGGATGGCGTGTCGTGGAATTAACCTGCATCA

GGCCATGCAGGAAGCGAAGGCATTTCTCGGCATCAGGGAGGACGATCACCATTTCGACGCCAGACGTGAGAAGAGATTCTCCAGACCTGACCGCAAGAAAATAGCCCGCTACGTTACCAG
AACAGAATCACATCTTGAGTACCTGCAATCGCGGGGCATATCGCCTGAAGTCGCGAAGCGATACGAGGTTGTCAGCGGAAAGGTCTGGAATGGCGAACGTGAACTGAATGCCCTGGTGCT
TCCGTACAAACGCGATGGCGAGCTGCTGCAGGTCAAGCGAATCAGTACTGAACGTCCGGACGGGAAGAAAGTCATCATGGCAGAAGGTGACTGTGAACCTTGCCTGTTCGGGTGGCAAG
CTCTCGATGCTGGCGTGAGGGCGGTTGTGCTTTGCGAAGGCGAAATTGATTGCATGAGCTATGCGCAATACGGGATCCCGGCGCTATCTGTCCCGTTCGGTGGCGGGAAAGGCGCCAAGC
AACAGTGGATTGAGTTCGAATACCACAACCTCGACAGGTTTGAAGAAATATTCATTTCGATGGACGTTGACGATGTCGGGCGTGAAGCAGCAAGGGAAATCGCAAGCCGACTTGGTGAGC
ATCGCTGCCGTCTGGTTACACTGCCACACAAAGATATCAACGAATGCCTGATGAACGGCGTCACCGAGGATGAAATCTGGCAGTACATCGGAACAGCGTCATATTTCGACCCGGAAGAACTT
TACAGCGCACGTGAGTTTTATCATGACACCGTCAATGAGTTCTACGGCAAGCAGCAGTATCTGTTTAACCCACCGTGGGAAACGCTGGCTTATAACTTCCAGTTCCGTGAGGCGGAGTTAAC
GCTGGTCAATGGCGTGAACGGTCACGGAAAAACGGAGGTTGTCGGGCATATGGCACTTGAGGCCATGAGGCAGGGGGTAAAAACATGCGTCGCATCACTTGAACTGAAGCCCGGGGTTT
TGCTTAAACGCCTGACCAGGCAGTCAACATGCTGCAAAACGCCACCAGTACTGGAAATCGAATCAGCATTTAAGTTTTACGATGACCGGCTCTGGTTATTTGGCCTGACAGGTACAGCCAAG
GCTGAACGCCTGATTGAAATTTTCACATACGCCAGACGGCGATACGGCATCCAGTTATTCATCATCGACAGCCTCATGAAATGCGGGATTGGCGATGACGATTACAACGGGCAAAAGGCGTT
TGTTGATTCGCTGTGCGACTTCAAGAATAAAACCAACTCTCACATTATCCTCGTCACTCACTCCAGAAAGGGAGACAGCGAGGAGAAACCTACCGGAAAGATGGACGTAAAAGGCTCAGG
AGCGATTACAGACCTGACAGATAACCTGTTTATCATCTGGCGCAATAAAGCTCGCGAGAGAGCGTTACAGCGCGTTCAGGCTGGCGAGCAAATTAACGAGAAAGACCAGCAACTTCTTGCT
TCGCCCGCATCTGTTTTAATGCTTGAGAAGCAGCGGAACGGGGAGGGGTGGAAGGTGGCGTGCCGTTATTTCTTGACGAACAGTCTCACCAGTTCCTGCAAATGGAAGGTGCATCACCA
TACAACTACATAGCTAACATGCCTAAGTCGGAGTATGACGAAGTGTGGAGGCAGGAGAATGTTACGGAGTACTGA
>EHGDEF_18685 HTH-48 domain-containing protein
ATGAAATCAAAAATCAAATCGCTACTGGTCTCTGGTTATAACCACGGCTGGTTAGGTATTTCGTTTGTAGATTTCTGGTTTAAAAATCTCAATCTGAGGGAATCATGA
>EHGDEF_18690 18 protein
ATGGGCGTCGTTAAGTTAGCAGACTACAGGCCGTCAGAGCCGGTCGTGGAGCGTAATGTGGCAGATCTCGATGATGGTTACGCCAGACTATCAAATATGCTGCTTGAGGCTTATTCAGGCGC
AGATCTGACCAAGCGACATTTTAAAGTGCTGCTTGCCATTCTGCGTAAAACCTATGGGTGGAATAAACCAATGGACAGAATCACAGATTCTCAACTTAGCGAGATTACAAAGTTACCCGTCAA
ACGGTGCAATGAAGCCAAGTTAGAACTCGTCAGAATGAATATTATCAAGCAGCAAGGCGGCATGTTTGGACCAAATAAAAACATATCAGAATGGCGCATCCCTCAAAATGAGGGAAAATCC
CCTAAAACGAGGGATAAAACATCCCTCAAATTGAGGGAGTGCTATCCCTCAAAACAGGGGGACACAAAAGACACTATTCAAAAGAAAGAAATACAAGATAAAAACATTATGTCCGAAAGTG
TTCGGACGAAGTGTGAAAAATCATCTGGCCATCACGAAGAAACCGACAAGGCATTCGAGGAAATTTTCTGGTGTGCTGGAATGCGGAAAGCCGGGAAGAAAAACGCAGCTTCGGCATTC
AGAACACAGTTCAGGGAGTGGCGTAAAACCACCAGGGGGACGGCAAGCGAGTTTGCCACAATGCTGGCAGAAGATATCGCGTGCAGGAACGGTAAGCAGTTCGGATTCGACAGGTTGT
TACCATCGAGCTACCTGAACGGTCAACGCTGGAACGACGAGAAGCCAGAAACCATTCAACCACAATCCAAACCATCATCCGCAATCACCGTATCGAAAACTGGCTACGTGTTTTTCGACAGG
TGA
>EHGDEF_18695 hypothetical protein
ATGCCAAAACGTCGTAAGAAATACCAGGAAAAAGAAGAGATTCGACACCCTGATTCACCTGAGGGATTAGTGGTAGCCGCAGCAAATAACAGGGCGTTCGCAGAGCGCCTTGTTGGTGTT
TACAGACTAGCCAAAGCAGGAGTGAAACATGGGCGTCGTTAA
>EHGDEF_18700 Bacteriophage CII protein
ATGGAACTCACAAATCACAGCAAAAAGATACGCGAAGTGGAAACAGAGCTTCGCGCCCGACTCGTATCAATGGGGCAGACAAATTTCGCAAAGATGGCGGGATGGTCTGATTCAAAAGTA
AGCCGCCTGAACATTCAGGATATGGCGGTGACCTTCGTTCTTCTGGAGAAGGTATGGGAGACGAGCTTAATCAGGGAAGTGGCAAGACAGGCAGTGGAAGCTGTTATGCCGGGAAATAA
AAAACGCCCGGCGTGCAAGACCGGGCGTTCTGAGCAAATCGATATGCAATTCTGA
>EHGDEF_18705 Transcriptional regulator
ATGAATTTAGTTATTCAGCGAGCCTTAAATATCGTCGGAAGCCAAAAGCGGTTAGCCGATGAATGTGGTGTAACGCAGCCAGCAGTTCATAAATGGCTTAAAGGTGGGATGGTATCTCCTGA
GAAGGTATCCGCCATTGTTAACGCCACTGGTGGGCAGATCAAGGCATATGAGATACGCCCTGACCTACCACACTTGTTTCCTCATCCGAATCAGGCCGCGTAA
>EHGDEF_18710 Transcriptional regulator
ATGCATATGAAAACTTTTGCTGACAGACTTAACGCCGCCATGTCTGCATCTGGGCTATCGCAAGCTCAGTTGGCAGAAAAGGTAGGAATATCGCAACCAGCTATCCAAAAAATGTCCTCAGG
CAAAACCAATGGGACAAGAAAGATGGTTGAGTTAGCTAATGCCTTAAAAGTACGCCCTGAATGGTTAAGCTCAGGGGTTGGTGATATGAGATATCCAGAAGGACCAGAACCATCAAATATC
CGAGAATCTTCCCTTAAGGCTACTATTTGGGAGGATATGAACAGGGACTCTGAGGAGTTTGTTGAAATCCCTTTATTAAATGTTTCTCTTTCTGCCGGGAATGGGAGTTGCGAGTTGGAAGA
GTCTTCAGATTTTGCTTTGGTATTCCGTCGATATTACCTCAAAAAAATGGGCGTTCCAGAGAGCGCTGCAAAATTAGTTCGGGTAAGCGGTCAAAGCATGGAGCCAACTTTAAACGATGGAG
ATGTTGTTGGGGTAAACACGCAGGACACAACCATACGCGATGGCAAGACCTACGCAATTTGTCAATCTGATCTGCTGCGAGTAAAGACACTGATAGCTACTCCAACATCAGTAATAATCAGAT
CGATAAATCGCGAGGAATACCCTGATGAAGTGCTGGAGAGAGAAGACTTCCACAAAAACGTAAGAGTCATTGGAAGAGTGTTCTGGTCATCTCATAGCTGGTAG
>EHGDEF_18715 hypothetical protein
GTGGCAAAGAAAAACGAGAGTCCTCTGTTACAGTCGGATGTTGATCGCGTCATAAACTCCAAGAAAATAGTGGATGTTAGCAGCATAAAATGGGGTAAGAAGCCGCCACCTGGCAGGTCA
CCGATGTGGTTACAAACGGCTATCACACCTTATGAGGATGGCTCACCTTTGCCTGGCCTAAAGTTTGTTTTACAGTGGCGACCAGCTGATGAATACGGAGATTCCCCTAAAATTCAGATGGTG
GCGCTTTACTTTGGTCGAAGAATTTTTGGTGTGGACTCTTACCCAAATGATAGACATACTAACAGAGTCCGGGTATGCCATCCTGACTATGCTGAAAGCATTCTTGGCCCACATTATCACCTTT
ACTTTGAATCAGCGTTGCCGTATGAAATAGGTCTGATCATACGCGAGAAGATAGCCCCGGATGACTTGTTAGGACACTGGCGCTTTTTCTGCTATAAACTGAATGTTACATGCAAAGGAATAC
TGCCTTTGCCGACCCAAGAAGATTCGGGACAAATTCCATTGCTATGA
>EHGDEF_18720 hypothetical protein
ATGAGTGGTCACAATATTGTCATACCTGTTACCATTAAAACCGCAATGCCCAAATACATATTTACCTCAAGCATTAAAGAAGGTGGATCATGGAACAGCGCATATTCATTGCTTGGAAAGCTGA
TGGATCTTTATCAGGCCAACAACGCTGTTAACAATCGATACGTAGTTATTGACAATGAATCTATCGGCTCGCAAATGCAGCAACTGATACTGCTGTTCAACGACGTAAGCCAAGTTCTTCCATT
CGAAAGCAGAAGTTTATGGCTTCCAAAATTAGCTGCATAA
>EHGDEF_18725 HEAT repeat domain-containing protein
GTGAAAAGTGAGGAAGAGTACTTTGCGGAGCTTCACCCGCAGGTGGTGGAGGTTCTGGGAACGGCAGTTATGCAGGTACTGGTAGAGCAGCGCGAACCTTCGCGTGAAGCCCTGATTGA
GATGATTCAGGTACTGTGGCAGGAAGAGGATGTGGACTTGGCTGTAGAACTGGCTATTGATGTTCTGAGGCTGCCGAAAGAGTAG
>EHGDEF_18730 Antitermination protein
ATGACGCGCAGAACAGCTTTCAATGGTTCAGCAGCAGGTCGTCGTCGTGAGCGCCGTGCAGCGCTTCAGAATGCGGTAACGGCAAGCTCAGAAGTATTACACCGCCCCACCCTTAGCCGC
GCCCAGATTCAGGCCAAAGGAAAACATGAAACGCCAAAACGTATTGAAGACGCAAAATCACTTCAGTTCATGGCGAAAGATGCATTCTGGCAACTGGAAGAATACAGACGCCATCTGGAG
CGGGCAGCCATTGTGTACGCAAATGAGTTTGGTCATAAGCCACCAGAAACCGGTGTATGTTTGCCAGACGTAGCGCTTTACGCAGCAGGTCATCGTAAATGTAGACAAGTTACCGCTAGATA
A
>EHGDEF_18735 Phage tail protein
ATGACCAAAGAAATTGTGACATTCAAGGGATTTAACAAAGACCTAAAGTGCCGTGGCTTTCAGTTTGCAATCGGTGAAACCTTCCATCACGATGGAAAAGTAGAGGCTTGCGGTTCTGGAT
TTCACGCCTGTGAATGTCCTTTCGATGTTTTCAGTTATTATCCGCCGGCAGAAAGCCGCTATGCGGAAACAATATCTTTTGGTATTACAGACAGTGAAGAAGGAGGTGACACTAAAATAGCCA
GTTCCAGTATCACAATTAAGGATGAGTTAACGCTTCCTCAGTTCATTCAACGTGGTATCGAATGGATTTGGAGCAAGATAGATAAGTCTCTTGAGCAGCAGATCATGTGTGGCAGCTGGTCAG
CGGCAACCAACACTGGTAACCGATCAGCAGCAACCAACACTGGCGACTGTTCAGCAGCAACCAACACTGGCGACTGGTCAGCAGCAACCAACACTGGCGACTGTTCAGCAGCAACCAAC
ACTGGCGACTGGTCAGCAGCAACCAACACTGGCGACCGGTCAGCAGCAACCAACACTGGCGACTGTTCAGCAGCAACCAACACTGGCGACTGGTCAGCAGCAACCAACACTGGCGACTG
TTCAGCAGCAACCAACACTGGCGACTGTTCAGCAGCAACCAACACTGGCGACTGGTCAGCAGCAACCAACACTGGCGACTGTTCAGCAGCAACCAACACTGGCGACTGGTCAGCAGCAA
CCAACACTGGCGACTGTTCAGCAGCAACCAACACTGGCGACTGTTCAGCAGCAACCAACACTGGCGACTTGTCAGCAGCGGAAGTGTCTGGATCGCAATCCGTAGCGGCATCACTCGGAA
TAGAAGGAAAAGCCAGGGCATCTGAAGGCGGAGCTATTGTGCTTTGCTATCGAGATGAAGATGGCGAGTTAATTCATATCCGCGCCAGTAAGGTTGGCGAGAACGGTATTATGCCGGATAT
ATGGTATCAACTGAATGAAGATGGTGAGTTTGTAGAGTGTGAGTGA

>EHGDEF_18740 hypothetical protein
ATGCCAATAAATAAACATAAGGAACTTCCCATGATGCAACTCAGCTTTGCGGGAAGCGGCGTCATGTCCGCTTTTTACCCCGCTGAATCCGAATTATCAAAACGTATTCGTCGCCTTATTCGCT
CTGTCCGTAAGCAACTGGAGGCGTTATGCAAAGCCAAATAA
>EHGDEF_18745 hypothetical protein
ATGCAAAGCCAAATAACAATCAATCACCAGAAGTTAATTGCAGCACAAAGCAAGGCTGTTATCGCTCGTTTTCTTGGTGACGGACACATGTGGAAACAAGCTACCGAGGAAATGAAATCAG
CAATCAACTTTCCATGA
>EHGDEF_18750 hypothetical protein
ATGATACCGCAAGAAATGAGCATCGCTGACGCATGGACAGATGATGCTTTTATCCGATTAATGCAGGACATGCTCAATCAGCAGAAAGAACAGGAGAACGATGATGATTCTGACTCTGAATG
A
>EHGDEF_18755 Prophage protein
ATGATTCTGACTCTGAATGATAAGCGTGAAATATCGCAAATCATCGCAAGTTTTACCGATGATGATTATGAACGAATTAACAGTGAAGTTGATCGCCTTTGCAAACGTTGCGACCCAATAAGCG
AAATGCTTCGCTCATATAAACCAGATGAGCACACTAAGGACGCTATCGACTGGCTGGAAGATGATGATTGTAACTACCAGGAAAAAGCCGCTGAATGGTTCTGGGATGCAATAACCGATAGA
GTTAAGGCTGAATATGCCTTCGCAATATTCAAACGCAGACACATTTATGGAGAAGCAGCATGA
>EHGDEF_18760 DNA recombinase
ATGAGCAATATCGTTGAATTCGTTAAACAGCAGGAGCACTTATTCTGCGGGGCATTGACTGAACAGACGGTAACATGGGCTAAGGAAAGCCAGTTTGCAATTCAGTATTTCCAGAAAAACG
ATTACCTGGCTAAAACGGCACTGGCAAATCCAACCAGCGCACAGAACGCCATCATCAATGTTGCCGCCATAGGAATCACATTAAACCCGGCGAGCAAACTTGCTTACCTTGTGCCACGTGAT
GGCATGGTTTGCCTTGATATCAGTTACATGGGATTACTTCACCTTGCACAGTCGACAGGCTCAATTAAGTGGGGGCAATGCAAACTGGTGTACTCAAACGACACCTATGAATCAAACGGCCT
TGATTCAGCACCAACCCACAAATACAACGCATTTGGTGAGCGAGGCTCTATTGTTGGTGGTTATTGCACGGTTAAAACAGCAGATGGTGACTACCTGACTGAAGAAATGAGTCTGGCAGAA
ATTAAAGCTGTGGAAGCAACGAGCAAGGCAAAGAATGGACCGTGGAAGACATTCTGGGAAGAGATGGCGCGCAAAACAATAGTTAAACGCGCCAGCAAATACTGGCCTAAAGCCCAGCG
ACTGGATAATGCCATTCACCTGCTTAACGAAGATGAAGGTATGCATCAGGAACCGGTTATGCCGCACAAATCAGAGGAAGATATCCGCGAAGATGAACGGAAACGCCAGCAGGAAATAATG
GATAAAGCACAACTTCTTTGTAATGAAATGGCTCAGGCTGAAAACATGGATGATTTGAAGCGATATTTTGCAGAAGCATATCGCCTGACATCTGGAATGAAATTGCAGCAGAACGTACAAGC
AATTTACGCAGAATGCAAAGCGAAACTGGAGGTTGCCAGTGAGCAAACTGTATGA
>EHGDEF_18765 hypothetical protein
GTGAGCAAACTGTATGAAATAGCTAATGAATACGCAAAATTGATGGATTCAGATTTAGAACCAGAGATGATTGCTGACACAATAGAAGGCATGGAAGGAGAATTTACCGATAAAATAGAGCA
ACTTCTTGCCATTATTAAAAATGAGTCTGGCTATGCTTTGAGCGCCTCAAGGAAGAGGCAAAGTCACTGA
>EHGDEF_18770 hypothetical protein
ATGAGAGGTTTGTCCTACGACCCCGGAATCCTTCCATCGGAAATGATTATTCGACACCGCTTCAAGCCCATCAACGATATTCCACGCGAAGAAATGCTTAAGCGAAATAGTTTCGGTTATGTTA
ATGAAAACAAATATCTGAATGCAATGTTGCGGAGTGGGAAGAAATGA
>EHGDEF_18775 hypothetical protein
ATGAAAGAAGTGAAAATATACACGATTGTCAGTGACCAGTTATCACCACCAATAACAGGAGAATCATTCTGTACTGACATGGTGCGTCATAGTGATTATGCGGAGCTTGAGGATAAATACGCG
GCGTTGGCTGCGGATAACGATAAAGCAATGGAGTCACTTAAGCAGGCTGATGCAGTTGTTAAGTTGGCACACGAGAAGTTTTCGGCGCTGGCTTCGGAGAATGCGGCACTGAAAAAATCA
GAGGTCGAATTCAACGAATATTGTCGTCGCGAGTGCGAGGACGTTGGCGATACGTGGGTGGACGATTTCACTGAGACCCCAGCCACCGACACTTTTCTGGCTGAGGTGACACAAGAAGCA
GTAGAGTCACTCAAGAAGGAGATTGAATGGCTCAAAAAACAGTTGCTTATGTCTGTCGGTGATATTCGAGACCTTATTGATTACAACCCTGAAAATGGCGTTCTTACAGCGAAAGTTAACTTC
AGCGGAAGGCAGGCAGGTTCTGTCATTGGCTCTCAAACTTGGCAGGGATATTACGCATTCTCACTGTTCGGTAAGAAGTGCTTTGCTCACAGACTTGCATGGCTACTGCATTACGGTGAGTG
GCCTTCACAACCTATCGATCACATAAACGGAATTAAGACGGACAACAGCATAAGAAATCTGCGCCTTTGTTCTCTATCGCAGAACCAATTCAACAAGCCTACTCAGAAAAACAACACTACAG
GTGTGAAGGGTGTGTATTGGAACAAGCGAGATAAACGCTACGTTGCCAGCGTCCAATTTAACGGAAAGAAATACAGCGCGGGACATCACAAGGATATTGATAGCGCTAAAGAGGCTGTAAT
GAAACTTCGGGAAAAACTGGCTGGAGAATTCACTAACCACGGCGAATTCGAACTCGCCGCCCAACTTCGCAAAGGAGCCTCGCTATGA
>EHGDEF_18780 ClpB-D2-small domain-containing protein
ATGAGCAATACAGCAAAACTTCAACTCGGATTCTCTCCTTTATCAAAAACCATCATGCTCGCAAAAATGCGCGATGTGGAAGGTGGCCGCCTGCGCGTCGGTAATGATCGTGGTCGTGATGT
TACCAATGAGGCTGCGCAGCTAGTGTGGCAGCTTGTCATGGCGGAAGGTGGTGAAATCGGCTGGGAGCTTGATGATGGTTCTCGCATGGTGCTGAAAGCTGAGAAGCAGGAGGCAACCA
GTGAGCAAAATTGA
>EHGDEF_18785 Ead/Ea22-like family protein
GTGAGCAAAATTGACCATCAGGCACTGCGTGAGGCAGCAGAACGTGCAACTCCAGCAATGGAACGCCTGTTAATGTTGCCTGTTGATGATGATTTGTTAAGTGAACAGGAACTTAAAGATT
ACGGTGTGGATATTGATGCGCTCAACGCCTTCAAATTTCTGACCGGACCAGAAACCGTGCTGGCGCTGCTGGATGAGAATATTCAACTCCAGCGGGGAAAAGACGCAATAGAGGCCGTAG
CGCTGGTGCTGCGTGATGATATGCGACAGGCGCGAGAGCAACTGGCAGCCGCAGAAAAGCGGAACTCCGAACAGCGTGAGTATTACGAGGGCGTTATTGCTGATGGAGGTAAGCGCATA
GCAGAACTGGAGGCGAAACTTGAAACTGCTGACAAGTTGCAGGATAGCGCATTCCGTGACGGCCTGAAAGCCGGGTTCAGTTATGGGCAGACAGATGACCAATCCGGGTTCGCGCAGTG
CATGTCCGCATATAGCACACGCGCTGGCATTGGCGTGAAGCAGCAGGAAGATAGCGTCGATTCTGATGTGGGAAGGAATCAGCCTGGAATGGTTGTCGCGGTTCATATCTGCGCCGGTGAC
TTTGTGAAAGTCAAAGGACAAGTGTTTGAGGTCGAGGAAACCGACTTTGACGACCACGACGTTACATTATGGTTTGTGGGTGGCAACGCATTGAAATGTGAAGCTGGTTGTCAGGTTGAG
GTGGTATCTGCGCCAGTTGCCTCTGGCATCAAGGTTAAGGGGAGTGAGCATGACCGTAGCACTAAGAGATAA
>EHGDEF_18790 Phage portal protein
ATGACCGTAGCACTAAGAGATAAACGTCGTAGCGGGCAACGCATCCCAGGACTCGGAATGTCAAATGGGACATGGTTCGCGGTGCTGGATATTCCAGGCATGGGAAAACTTGTTAACCAGC
AACATACCAATGACCCGCTCGATGTGACTCCAGCCAAAGCGAAAAAGATGGCTGACATCGTAGAAGCATGGACACCACCTGAAGGATGGTCCGGTGATATGGCTGAAAAAATGAAGGGCT
ATATCGTCGAATTTCTTCGCGGTTGCAACGGATTCAGGAGCCACTAA
>EHGDEF_18795 hypothetical protein
ATGACCACTATTACCAAAGAACGTATCGAATTATTCATTAAAAGTCCTCTTGAAAACGGGCTTACCCGTGGCGAACAAATGGAGCTGGCGCGTATCGCGCTGGTATCGCTGGACGCTGGAGC
CTGTAAGCCAGCCTTACAAGTTGCCAGAAGAGAAAGGCGCATCACTACAATTGCGAAATCTCATTCGCAAACGACACGCGGAGTGGTCAGATTCAACGTTCGGTAA
>EHGDEF_18800 DUF551 domain-containing protein
ATGCAATTCCTGTTATGGGATGCGCAACGCCGCGCCGGTATCAGCGATGGTGAAATCACAGCTGCGATGGAAGAAAAGCTGAAGGTGAATATGGCGCGCCAGTGGCCTGAACCGAAAGAC
GGCGAGCCGCGGCTACATATCAAAGAGCAGCCGGCGCCGGTAGTGCCAGCGGAAATGCCAAAAGGTCTGGCTGGTCAAATTGTCAGTCTGCTGGCCCACAACATTGGCGATAAATTCCTG
GCACAGAAAATATGGTATGCCTGCCGCGCCGCCATGCTTCAGAGAGTCGAAAACGCCGAGTCGCGATGTGGCAACCCTAAGGTATCTCCGGATGGTTGGATTCCGGTAAGCGAAAGGATGC
CGGAAGAAAGTGGACGTTACTGGTGCTACGTGGAAGAGCAAAATAGCCTGGGTAAAAGTCACTATCAATGGAACTGCTCATGGAATGGTCAGGCATGGTCAGACAAGGCGCTATCTGGTC
GAGTGACTCACTGGATGCCACTGCCAGAACCACCGCAGGAGGAAAAGTAA
>EHGDEF_18805 hypothetical protein
ATGCAACCATTTGGGAAATTCTATTCGGTTGATGGTTGTACCTGTTCGTTGTGTCGATCACGAGGCTACAGAAAAGGAAACGGATATGATGCTGAATTGAGAACCTGTAAACATCGAGCTCG
TCAGCATAGTAAACGCCTGATTGATAAGGAGTTAATACAGTTCGAAAGCATTCGCGATTATTGA
>EHGDEF_18810 LF subunit
ATGGGTGGCAAAGTACCTAACTACCAAATCGTTTATAGAGACGAGACACTCAATTATTTCAAGCCTGGAGGATATGTTTTCTTTCAAAGGCTTAAAGAATATGGCGGCGGTTATTGGTTAGGC
AAAATTTACGAGGATGGATTCGAGTTTGTGCTTGAAAGGCCAACCTCATTAAGTGAGGGAATTAAGCATTTACTTGTTTTAAAAAGCGTTGAAGATGGGTATCTGGAATTTGTAGATGATATC
GACAACTTCAAACTTCAATGA
>EHGDEF_18815 Prophage integrase IntD

ATGTCCATCTTCCGCAGAGGTGAAATATGGTACGCGTCCTACTCGACACCGGGCGGGAAGCGAATTAAGGAAAGCCTTGGGACTTCCGACAAGCGGCTCGCTACTGAGCTACATGACAAGC
GCAAGGCTGAATTGTGGCGAGTAGACAGGCTTGGCGATTTTCCTGACGTAACGTTTGATGATGCCTGCATGCGCTGGCTGGAGGAAAAGGCAGAGAAGAAATCACTGAAGGATGACCGC
AGTCGTATGGCTTTCTGGCTGGCGCATTTTGAGGGAGTACGGTTAAAGGATGTAACCGAGCAAAAGATTTACTTAGCAGTAAACAAGATGAGCAACCGCAAACAGCTTGAGATATGGAAAA
TCAAAGCTGCCGCGGCGCAGAAGAATGGAGAACCTGCACCAATCTATTCAGCTAAACCGGTCACAACCTCCACTAAGGCCAAACACCTGGCATTAATGAAGGCTATTCTGCGTGCAGCAGA
ACGTGACTGGAAATGGCTGGAGAAAGCGCCTGTAATCAAGGTTCCAGCCGTCAGAAACAAACGCGTCAGATGGCTGGAGAAAGAAGAGGCAAAACGCCTGATTGATGAATGCCCTGAAC
CGTTGAAATCTGTTGTTAAATTTGCGCTGGCAACAGGACTTAGGCGGTCTAACATCATCAATATGGAATGGCAACAGATTGACATGCAACGTCGTGTTGCCTGGGTGAACCCTGAAGACAGC
AAGTCAAACCGCGCTATTGGCGTAGCGCTAAATGACACTGCCTGTAAGGTATTGCGTGACCAGATTGGTAAGCATCATAAATGGGTGTTCGTGCATACGAAAGAAGGCATCCGGCCCGATGG
CTCAAAGACTCCGACTATCAGAAAGATGCGCGTCGATGACCAGCGAGCATGGAATGCAGCTTGCCGCCGGGCTGGAATTGAGGATTTCCGTTTCCACGATCTGAGGCACACGTGGGCCAG
TTGGCTAATTCAGTCCGGAGTGCCGCTTTCTGTTCTGCAGGAAATGGGAGGATGGGAGAGCATCGAGATGGTGCGCCGATATGCTCACCTTGCGCCGAATCATTTAACGGAACACGCGAAG
CAAATTGACTCGATTTTCAGTGATGATGTCCCAAATATGTCCCATATGGAAAATAAGGAGGGAATTAAAGAGGCGTAA

Prophage 6.2

>DOCPEC_16590 hypothetical protein
ATGTCAGACATTACCCCTAATATTGTAGTGAGTATGCCTTCGCAACTCTTCACTATGGCGCGTTCTTTTAAAGCCGTAGCTAAAGGAAAAATTTATATCGGAAAAATTGACACTGACCCGGTAA
ATCCTGAAAACCAGATTCAGGTTTATGTGGAGAACGAAGACGGTTCTCACGTTCCTGTTGCACAGCCAATCATCATTAATGCTGCCGGATACCCTGTATATAACGGACAGATTGCCAAGTTCG
TAACTGTGCAAGGTCATTCTATGGCTGTTTATGACGCATATGGGACTCAGCAGTTTTACTTCCCAAACGTGCTTAAGTACGACCCTGATCAGCTTGAATACAGACTTAGTCAGCCTGATGGCTA
CTTATTGGTTGGTGGACTGGCTGAGCATTATAACCTTCCGTCATCTGTAATTGTAGTTGATAACGCACCCTATAACGGCGACTTAAAGGCAGCATGGAACGCAGCGCCGGAAGGGGCAACAC
TTCTACTTGGTAAAAAAGATTACAACATCACAGGCTTATGGGCGTCAGGTAGAAATACCAAGAAAAACATCATGATTGTTGGCATGGGCATGCCGGAATATGCGTCGGACTGGAGCAGATTT
GTCAGCGGTTCCGGGACGGTAATTCAGGGTGCAGTGAAGAACCAGGCCAAAGGATTCAAACTTTTCAACCTAGGCGTTGACTGTGGTAATTATGTGTCAACTACGCTGTATAGCACGACAA
CCTATGAAGATGCGGTGCAGATCTATGGAGTCGGTGCGAAAGCAAATATCGGAATTGACAACATCAGGACGCTTAACTCTCTCGGTGTTTCAAGCAATCCTGGTACCCACAGCATTCTGCTT
GAACAACTTGAAGGCGTTACGCTTGGCTACGTAGAGTGTTGCGGTGGTTTCCATGGGCTGACAATCAAGTGTAAGAACTTGCGCGGTGGTCGCGCCCATGTCTATGGACAGTATGGTGACG
GTTTTATTCTAAAATCTGATTCAGGCGGACCATGCAGCGATATCAGAATGGACAGTATCACTATTGGTCTGATTGATAGTTCACTGCTGCCGTCAGTATCTCTCGGTGGAATTTACGATGCTCAC
GACGGGGTTTCCATCGACAACATCAGTATTGGTGACCTTAGAGTGCAAAATGCTTCATGGGGGTTTATTCCTGCCATCGGGGCGGATGGTTATACCAGCCATGTCACAATAGGAAATTATTAT
GCGTCACAGGTTTATGGAAACTATTACTCTCTGGAGGTGGGTAATCAGTGCGTTAACTGGAATATCGGTTCTCACCAGTGTTCAGGCGTTTCAGGGGGCATCAAAATTAACGGATCGGCTCA
ATACATAACATTGGGCGAGGGTTCAGTAACAGGCAGCACAAGATGGGGTTATTCATTTGCTGCATCGACATTTACACATAGCTCACTGATATCAAATGGCAACTATGGTGGGGTTGAGTATCT
CGGTGGTACGGGATTTAACCCAGCTAATGTAATCGCGTATTACAATAACAACGGAAACTTTAGCGCGCTCCCTTCGGTGCTTAACGGGAATGCTTTAAATGGATGGGTGGCGCTATCAGACTT
TAAGGCAACACCAAATGCTCATCAGGTATTCATTAGCGGATCGCTGACAAATGGTACGGCAGCGAATGCGTGGCTTATCGCAGAAAATCTTAGACCGTCTGTTGACACACCTATTTCTGCATG
GGGGGTATCTAGCGGTGGTAGCCTTGTGCCTGTTGAGGCGTATGTGCGAGCAACTGGTTATATTGAGATTACAGGTTATGCATCTCTTGGGGCATCGCAAGCCGTGAGAATCAACGGATCAT
ATCTGATCGCTTAA
>DOCPEC_16595 Lytic transglycosylase domain-containing protein
ATGAAAGTTACAGCCAACGGTAAGACATTCACATTCCCAGAAGGAACAAGCACTGAGGATATTGGATCGGCTATCGATGAGTATTTTGCTGGACAGTCTGCACAGCAGGAACAGCAGGGC
ACATCTACGCCCCCAGAAATCCAGCCACAGCAACAAGGTGGCTTCATTTCTGACCTTGGCAATGCTGCTGCAGAGACTGGGCGTGGATTACTACAGGCTGGCGTTAATCTGGCAAATATCCC
GGCATCAATGGCTGATGCTGTCGCCAGCGCCGGGGCATGGGCTGGTCAGAAGATTGGCATTGGTGACGGAACTTATCAGCCAGCGCCTCGCGTCACGACACAAGGACTTGAGCAGGACT
TTGGCTTGCAACAAGGCGCGCTTACTCCACAGACGACAGAAGGTAAAATCTTCTCTGAAGCACTGCCATATTTGACTCCTGTTGGGGCCGAGAGAATTGCAGCGCAGGCACCATCTATTGC
CGGTCGAGTTGCTCAGGGTGCATCACGCTTGCTGGCGGAGAACGCTGTTGGTTCATTGGCTGCAAACAGTGAGCGTGATAATCCAGAAGCACTGGCAACAGACTTAGGAACTGGTGTTGC
ATTAGGCGGGGCAATCAATCAGTTAGGTCGTGCCGCTGGCGCTGCTTATCGTGGGATTCGCGGGACGATCGCACCAGAAGCGCAGCAGGCTATTCAGTTCGCTAATGCTGCTGATGTTCCTT
TGCATACAACTGACGTTTTGCAGCCAAATTCCCGCGTCGGGCGCATGGCACAAACCACAGCTGAAAACATCCCATTTGCCGGGACAAGTTCAATGCGAGCTAATCAGCAAGAAGCTCGCAG
TCAGTTGGTAGATGAATTTGCATCACGGTTTGGTGAGTATGATCCGTCAATTGTTATTGGCAGCCTGAAGGCAAAAACATCAGGAATTCGGAAAGCAGCAGGGAACCGTCTTGAGCAAGTT
CAGAGCGCAATGACAGGAGTCAACATTCAGCCAACGCGAGCAATTCAGCAGATAGATGATGAGATTGGAAAACTGCAAAAATTAGGACAAGTTGCTGACACGGATACAATTAGCAAACTT
CAGGCATACAGGAATGAATTGGCTAAAGGTGATGTTAACCTGGAACAGTTAAGCAGACTGAGAACGCAGTTTAGGATGGATGTCAGAGGAGAAAGGACACAAATGCCACCGCCAGCTGA
GGCGGCAGTGCAGCGTGTATACAGGGCAATGACAGGAGACATTGATAACTCCATTGGCCAGAACCTTGGAAACGACACTCTGCGCAGATACAAGCAGGCCAATGCGGTATACGCAGATGA
GGCTAGTAATCTCCAGAATACCCGCTTGAAGAACGTTCTGATGAAAGGGGATCTAACCCCTGAAGTTGTCAACAACATGTTGTTCAGCAAGAACAAATCAGAAGTTCAGAATCTGTACCGGT
CAGTCGGTCATGTGGGGCGCGCTCAGATGCGTAACGGCATCATCGGAAAGGCTATGGAGAAATCAGGCGGTTCTCCGGATCAGTTCCTGCGCCAGGTTAATTTAATGTCTACCCAGACGGG
AATCGCTTTTAAAGGCCGTGATGCTGCATATTTGAAAGGGCTGAAAAACTATCTTGAGTCAACCAAGCGTGCGGGGCAGGCAGGTGTAACGACGCCAACCGGACAGCAGACAATCCCATT
CATCTTAGGAATTGGGTCTGCAACTAACCCAGCGCTAGTTGGGGTCGGTGGTGGTTATGGATTGCTGGCGAGAATGTATGAAAGTGAACCGGCACGAAACGCAATGCTTCGCCTGGCTAAC
ACGCCACGTGGTTCTACCGCATTCGAGAAAGCGTTATCTGATGTTGAGCGGGCAGTTAACTCATTCGCTCAGGGTGCAAAATCAGAATCCTTAAGCGAATAG
>DOCPEC_16600 DNA transfer protein
GTGGCTACATTTCAACTCGCCGGACTGCCATCAATGCAGGTGGCAAACCAGAACGCGCCCGGACAACCATCATTATCCAGTTACGACTTCAGCCAGCGTCCAAACGTTGGAGTTCAACTTG
CTCAGGGTCTTGGTGCAGTTGGTCAGGCAATACAGCAGAATGAGGCTGCTCAGAGGCTTTCTGAATTTCAAAAAGCTTTCGGTCAGGCTTATGCGGCAGGTGACCGCGATGCCTTGCGTC
AACTTGCGGCCACCAATCCAGAACAGATTGAAACAATTCGTCAGGGAATGGGGTTTGTTGATGCTGACAGAAATCAGGCGATGGGCGATATGTCTGCACGATTGAATATTGCCGCCGCTCA
GGGGACTGAAGCGGTGATGCGAGAGCTTGCCACCCACCAGAATACACTGCAGCAAATTGGCGTATCTCCTGAACAGGCGTGGCAGACATATCAACAAAGCCCTGAAGGCTTCTCACAGTT
AACAGACCTTATTGGGATGCACGCGGTAGGACCAGAAAAGTATTTTGATATTCAGGACAAGTTGACAGGTCGCGAGATTGACCGAGGTCGACTTGCTGAAACAATCCGCAGCAATAAAGC
AGGGGAAGGACTTCAGGCTCGCGGGCAAAATATTACTATGCGCGGACAAGACATGTCAGCCTCTACAGCCCGCCGCGGCCAGGATTTGGCAATGCAAAGGGCAAACGCCAGAACGATATC
AGGAGTCGACGGGAATCGTGTCGTTCAGCTTGCAGATGGTAGAACAGTCAACATTGACGGAAAACTTCACGGCGCAGGGGCTAATGCATTTTACGAAGGTATTGACGATAACGGCAATATG
GTTCGTGTCCCGGCAAGTGCTATTGCAGCACCTCCAACGTCTGCGGCAAGCGCACAGAACTACGCGATGAAGAAAGATATCGATGCAATCGCAAACGCAGATGCTTCTGCTCTTGATTTTAT
GACTGGAATGACTGGCGGAGCAGGAAATCCGGCAATTGGTGCAGATGTTCGCAGCCGACTCACAGGAAAAGAGCAACGCCAGTTATATAACTCCGCACAACGTATTCAGGGAAGAATGCA
GAATCAGGGCGTGGCAGCAGCAAGAGATATGGGCGCTAGCGGTATCAACACCATTGCAGAAGCGAAGATGTATTTTCAGGGGATGCCGCAGGTTGACTACTCAAGCCCGGAGGCTATGCA
GCAGTCTATTCGTGAGATTCAGGAATACACCAACAATTATAACCAGCAGTACAACGTTAATGTTGATAATGGTGGGCAGAAATCATCAAGGCAGCAGCCAGCGACTCAGCAATCAGTCGGAG
GAAGCTACACGTCTAAATCCGGCATTCAATTCACGGTGGAATAA
>DOCPEC_16605 Internal virion protein gp7
ATGTTATATGCATTTAAGCTGGGCAGGAAACTGCGCGGTGAGGAACCTTATTATCCTGAAAAAGGCGGTAAAGGTGGCTCATCAAGCAGCGGGGCAAAAGAAGCCGCAAAAGCAACACA
GTATGCAGCAGACCTGCAAAACCAACAATTCAATCGTGTGATGGAACAGTTGGCACCTTACGCCGCCGCAGGTTTGCCGGCTCTCCAGCAGATTCAGCAGCTATCAACGCTGGAAGGTCAG
AACAGCGCTCTCAATCAGTATTACAACTCAGACCAGTATAAACAGTTGGCTGATCAGGCTCGCTATCAAAGCCTGAATGCAGCGGAAGCCACCGGAGGTCTTGGCTCTACAGCAACATCAAA
CCAAATTGCATCCATTGCACCAACGCTCGGGCAGAACTGGTTGTCAGGGCAGATGCAAAACTATGGCAACCTGTTAAACGTTGGTCAGTCTGCGGCAGCAGGCCAGGCATCGGCAGGACA
GAACTATGCAAATAACGCAGGTAATCTTGCGCAACAGATGGCGGCTATCCGCTCTCAGGGTTCTGGTCAATCCACGCTTGGAAGTGCCATTAGCGGGGGTACGAGTGGTGCGCTTGCAGGA
GCTGGTCTTGCCGGGATGCTTGGTACATCGACACCGTGGGGGGCAGGCATTGGTGCAGGTATCGGATTGCTTGGCTCACTCTTCTAA
>DOCPEC_16610 Protein gp14
ATGATTACATTCACTCCCACCCGAAACATCGACCTGATAGAAACGGTCGGCAACCATCCCGACATCATCGCCGGGAGTAACAACGGTGACGGATACGACTACAAGCCTGAGTTCCGCTATTTC
GAAGTGAACGTACATGGTCAGTTCGGTGGCATCGTGTATTACAACGAGATTCAGCCGCTGACCTTTGACTGCCACGCCATGTATCTGCCTGAGATTAGAGGATTCAGTAAGGAAATCGGGCT
GACGTTCTGGCGATACATTCTCGCCAACACCACCGTTCAGTGCGTTACATCATTTGCTGCACGCAAATTTCGCCACGGTCAGATGTACTGCGCAATGATTGGCCTTAAGCGTGTAGGAACCAT
CAAGAAATACTTCAAAGGCGTGGATGACGTGACGTTTTACAGCGCCACACGCGAAGAACTAATCGACTTCCTGAATCACGGGAGATAG
>DOCPEC_16615 Phage tail protein

ATGGCAGACCCGTCACTTAATAATCCCGTTGTTATCCAGGCCACTCGTCTTGATGCATCTATCCTCCCCCGCAACGTCTTCAGCCGGTCCTATCTGCTCTACGTAATCGCGCAGGGGACTGACG
TTGGCGCTATTGCGGGAAAGGCAAACGAAGCAGGACAAGGTGCCTATGACGCGCAGGTGAAGAACGATGAGCAGGATGTTGAGCTTGCAGACCACGAAGCGAGAATTCAGCAGTTACG
CATCGACGTAGACAACCATGAAATCCGTATTACTGCAAATACCAATGCAATTGCAGCGCTGGATGTCAGACTAACCACGGCTGAAGGCGAAATAGTCACCTTGCAGGCTGATGTCAGTGCTC
TTGATGGTAGAGTGACGACTGCCGAGAACAATATTTCGGCATTGCAGGCTGACTACGTATCTAAAACTGCCACTACATCTCAATCGCTGGCTTCACCCCTCAACGTGACAACGTCATATTCAGT
CGGCGGAAAGAAGGTTGTCGGCACCCGACAGACTGGATGGACAGCAGCTACGGGTACGGCGAATAAAGGCGCATTTAACGCTGACCTGACATTTGCCGTTAGCGATACTTACACGCAATC
TGAAATCCAGGCTATAGCCAATGCTCTAATTGCTGAGCGTCGGCGCACTAAGGCTTTGGAAGACGCCTTGCGTGCACATGGGTTGATTGATTAA
>DOCPEC_16620 Phage head DNA stabilization protein
ATGCCGATTCAACAACTCCCCATGATGAAGGGAATGGGTAAAGACTTCAAGAACGCCGATTATATCGACTATCTGCCAGTGAATATGCTGGCAACACCCAAAGAAATCCTTAACAGCAGCGG
CTATCTCCGCTCATTCCCCGGCATTACCAAACGTTATGATATGAACGGCGTATCGCGTGGAGTTGAGTACAACACCGCTCAGAATGCTGTTTATCGTGTTTGTGGTGGCAAGCTCTACAAAGG
AGAAAGCGAAGTTGGTGATGTTGCCGGAAGTGGTCGCGTATCAATGGCACATGGTCGGACATCACAGGCGGTAGGCGTTAATGGCCAACTGGTCGAGTATCGCTATGATGGCACGGTTAA
AACCGTCTCAAACTGGCCTGCAGACAGCGGATTCACGCAGTATGAGTTAGGTTCAGTGCGTGACATTACGCGCTTACGTGGGCGTTATGCGTGGTCAAAAGACGGCACTGATTCATGGTTT
ATCACTGACCTCGAAGATGAATCGCATCCTGACCGCTACAGCGCACAATATCGCGCAGAGTCGCAGCCTGACGGCATCATCGGCATCGGAACATGGAGAGACTTCATCGTCTGCTTTGGTTC
GTCAACGATAGAGTATTTCTCACTGACAGGCGCAACCACCGCTGGCGCTGCGTTGTATGTCGCACAGCCATCGTTGATGGTACAGAAGGGCATTGCCGGAACATACTGTAAAACGCCGTTCG
CTGATTCATACGCCTTTATCAGTCATCCGGCTACTGGCGCACCTTCCGTCTACATCATCGGCTCAGGGCAGGCATCGCCAATTGCGACCGCCAGTATTGAGAAAATTATTCGCTCATATACCGCT
GAAGAAATGGCGACGGGTGTGATGGAGACTTTGCGCTTCGATTCTCATGAGCTTCTGATTATTCATCTCCCTCGCCATGTTCTGGTTTACGACGCATCGTCCAGCCAGAACGGACCTCAGTG
GTGTGTGCTGAAAACCGGGCTTTACGATGATGTATATCGCGGCGTCGACTTCATGTACGAAGGAAACCAGATAACGTGCGGCGACAAATCAGAAGCGGTGGTCGGCCAATTGCAATTCGAC
ATCAGCAGCCAGTACGACAAACAACAAGAACACCTACTGTTTACGCCCCTTTTCAAAGCAGATAACGCCAGATGCTTCGACCTTGAGGTTGAATCATCCACTGGTGTTGCTCAATACGCTGA
CCGCCTGTTCCTGTCTGCAACAACTGACGGCATCAATTACGGTCGTGAACAGATGATTGAGCAGAATGAGCCGTTTGTGTACGACAAGAGAGTTTTATGGAAGCGTGTAGGGCGTATTCGTA
GATTAATCGGATTCAAACTGCGGGTAATCACCAAATCACCAGTAACACTATCCGGGTGTCAAATTCGTCTGGAGTAA
>DOCPEC_16625 hypothetical protein
ATGCAGATAAAGACTAAAGGCGATCTGGTCAGGGCTGCGCTTCGTAAGTTGGGCGTGGCATCAGATGCAACCCTTACCGATGTCGAACCTCAGTCTATGCAGGATGCCGTTGATGATCTGGA
AGCGATGATGGCGGAGTGGTATCAGGACGGGAAAGGCATCATTACCGGCTATGTATTCTCAGATGATGACAATCCTCCTGCTGAAGGTGACGACCATGGCCTTCGCTCCAGCGCAGTCAGC
GCCGTATTCCATAATCTGGCCTGCCGCATTGCTCCTGATTATGCGCTTGAGGCTACTGCCAAAATTATCGCCACTGCTAAATACGGAAAAGAGCTTCTCTATAAGCAAACCGCCATTTCCAGAG
CCAAACGAGCACCTTACCCGTCACGCATGCCGACAGGCAGTGGAAACAGTTTCGCCAATCTGAAAGAATGGCATTATTTCCCCGGAGAACAGAATGCCGATTCAACAACTCCCCATGATGA
AGGGAATGGGTAA
>DOCPEC_16630 hypothetical protein
ATGAAAATAGCAATCTATAAGCCCGGTGGAAGCATCATGGTATGGGGCGTCATGGCTCAGATGAAGGTCATCGACTCCAGCGAACTTCCGGAATATGTCAAAGATGGCTGGCTTGATCATCC
ATCAAAGCTGCTGCCCGTGGAAGCAGATGATGTTAAGCCACGCAAAGGCCGCAAGCCTAAGGCGGTAAGCGATGCAGATAAAGACTAA
>DOCPEC_16635 Major capsid protein
ATGGCTTTGAACGAAGGTCAAATTGTTACACTGGCGGTAGATGAAATCATCGAAACCATCTCCGCAATCACTCCAATGGCGCAGAAAGCCAAGAAATACACCCCGCCTGCTGCTTCTATGCA
GCGCTCCAGCAATACCATCTGGATGCCTGTAGAGCAAGAGTCACCCACTCAGGAGGGCTGGGATTTAACTGATAAAGCGACAGGGTTACTGGAACTTAACGTCGCGGTAAACATGGGAGA
GCCGGATAACGACTTCTTCCAGTTGCGTGCTGATGACTTGCGAGACGAAACTGCGTATCGTCGCCGCATCCAGTCTGCCGCTCGCAAGCTGGCGAACAACGTTGAGTTGAAAGTCGCAAAC
ATGGCCGCCGAGATGGGTTCGCTGGTTATCACCTCCCCTGATGCCATCGGCACTAATACCGCAGACGCCTGGAACTTTGTGGCCGACGCAGAAGAAATCATGTTCTCCCGCGAACTTAACCG
CGACATGGGGACATCGTACTTCTTCAACCCTCAGGACTACAAAAAAGCGGGTTACGACCTGACCAAGCGTGACATCTTTGGGCGTATTCCTGAAGAAGCATACCGAGATGGCACCATTCAG
CGGCAGGTTGCAGGCTTCGATGATGTCCTGCGCTCTCCGAAACTTCCTGTGCTGACCAAATCTACTGCAACTGGTATCACTGTATCCGGTGCTCAGTCCTTCAAGCCTGTCGCATGGCAACTG
GATAACGATGGCAACAAAGTTAACGTTGATAACCGTTTTGCTACCGTCACCCTGTCTGCAACTACCGGCCTAAAACGCGGCGACAAAATTTCGTTTGCTGGCGTGAAGTTCCTTGGTCAGAT
GGCTAAGAACGTACTGGCGCAGGACGCGACTTTCTCCGTAGTTCGCGTTGTTGATGGTACTCACGTTGAAATCACGCCGAAGCCTGTAGCACTGGATGATGTTTCTCTTTCTCCTGAGCAAC
GGGCATACGCCAACGTTAACACCTCGCTGGCTGATGCGATGGCGGTGAACATCCTGAACGTTAAGGATGCTCGCACCAACGTGTTCTGGGCTGATGACGCCATCCGTATTGTGTCTCAGCCG
ATTCCGGCCAACCACGAACTGTTTGCAGGCATGAAAACTACCTCATTCAGCATCCCGGATGTCGGCCTTAACGGTATCTTCGCGACGCAGGGGGATATTTCCACCCTGTCCGGCCTGTGCCG
TATCGCGCTGTGGTACGGCGTAAACGCGACACGACCGGAAGCAATAGGTGTTGGCCTGCCTGGTCAGACTGCGTAA
>DOCPEC_16640 Scaffolding protein
ATGGAACCAACCACCGAAATTCAGGCAACTGAAGACTTAACCCTGTCCGGCGATCATGCAGCGGCATCTGCTGATAGCTTAGTTGTCGATAATGCCAACGACAATGCAGGTCAGGAAGAGG
GCTTTGAGATTGTCCTGAAGGACGATGAGACAGCACCAAAACAAGACCCGGCAAAGAACGCAGAATTCGCCCGCCGCCGCATCGAGCGCAAACGACAGCGCGAGCTTGAGCAGCAGAT
GGAGGCAGTTAAACGCGGAGAATTGCCGGAGAGTTTACGGGTAAACCCTGACCTTCCACCTCAGCCGGATATTAATGCCTATCTGTCAGAAGAAGGCCTGGCTAAATATGACTACGACAAC
AGCCGTGCGCTTGCCGCTTTCAATGCTGCTAATACCGAATGGCTAATGAAAGCGCAGGACGCCCGCAGCAATGCCGTAGCAGAACAGGGCCGCAAGACTCAGGAGTTTGCCCAGCAATCA
GCGCAATACGTCGAAGCTGCCCGCAAACACTATGACGCGGCGGAAAAGCTCAACATCCCTGACTATCAGGAGAAAGAAGACGCATTTATGCAACTGGTTCCGCCTGCGGTTGGGGCCGAC
ATTATGCGCCTGTTCCCGGAAAAGTCCGCCGCGCTCATGTATCACCTGGGGGCAAACCCGGAGAAAGCCCGCCAGTTACTGGCGATGGATGGGCAGTCCGCGCTGATTGAACTCACTCGAC
TATCCGAACGCTTAACTCTCAAGCCTCGCGGTAAACAAATCTCTTCCGCTCCCCCTGCTGACCAGCCGATTACCGGTGATGTCAGCGCAGCAAATAAAGATGCCATTCGTAAACAAATGGATG
CTGCTGCGAGCAAGGGAGATGTGGAAACCTACCGCAAGCTAAAGGCAAAACTTAAAGGAATCCGATAA
>DOCPEC_16645 Portal protein
ATGGCCGACAATGAAAACAGGCTGGAGAGTATCCTGTCGCGCTTTGATGCGGACTGGACAGCCAGTGATGAAGCCAGACGAGAGGCAAAGAATGATCTCTTCTTCTCCCGTGTATCTCAGT
GGGATGACTGGCTATCACAATACACAACCCTACAATATCGCGGGCAGTTCGATGTGGTACGTCCTGTGGTGCGCAAGCTCGTTTCTGAGATGCGTCAGAACCCTATTGATGTTCTGTATCGTC
CAAAGGATGGAGCAAGTCCTGACGCTGCTGATGTGCTGATGGGCATGTATCGCACCGACATGCGGCACAATACAGCGAAAATTGCTGTCAACATAGCCGTTCGTGAGCAGATTGAAGCAGG
CGTGGGTGCGTGGCGTCTGGTCACTGACTACGAAGACCAAAGTCCAACGAGCAACAATCAGGTTATCCGTCGAGAGCCTATCCATAGTGCCTGCTCCCATGTTATATGGGACAGTAACAGCA
AACTGATGGACAAGTCTGACGCCCGTCACTGCACAGTTATCCACTCAATGAGCCAGAATGGCTGGGAGGATTTCGCAGAAAAATACGACCTCGATGCTGATGATATTCCATCATTCCAGAAC
CCCAACGATTGGGTATTTCCATGGCTGACGCAGGACACAATTCAGATCGCTGAGTTTTACGAAGTGGTCGAGAAGAAAGAGACGGCGTTTATCTACCAAGACCCGGTTACGGGTGAGCCG
GTAAGCTACTTTAAGCGCGATATTAAAGACGTCATCGATGACCTGGCTGATAGTGGATTTATCAAAATTGCAGAGCGCCAGATTAAGCGTCGCCGGGTATACAAATCGATTATCACCTGCACT
GCTGTACTCAAAGACAAGCAGCTCATTGCTGGCGAGCATATCCCCATTGTTCCGGTGTTCGGAGAGTGGGGCTTCGTTGAAGATAAAGAAGTGTATGAGGGTGTCGTCCGCCTGACAAAAG
ACGGCCAGCGTCTGCGCAACATGATTATGTCGTTCAACGCCGACATCGTGGCCCGCACTCCGAAGAAGAAGCCGTTCTTCTGGCCTGAGCAGATTGCAGGCTTTGAGCATATGTACGACGG
TAACGACGATTACCCATACTACCTGCTCAATCGCACTGACGAAAATAGTGGAGACCTTCCGACTCAGCCGCTGGCATATTATGAAAACCCGGAAGTGCCGCAAGCCAACGCCTACATGCTGG
AAGCAGCAACCAGCGCAGTAAAAGAGGTTGCCACTCTCGGAGTTGATACAGAAGCGGTAAATGGCGGACAGGTTGCGTTTGATACCGTCAATCAACTGAATATGAGGGCTGACCTTGAGA
CATACGTGTTTCAGGATAATCTGGCTACCGCCATGCGCCGTGACGGAGAGATTTACCAGTCGATAGTTAATGACATCTACGATGTTCCTCGCAACGTTACGATTACCCTTGAGGATGGCAGCG
AGAAAGATGTTCAGCTAATGGCTGAGATTGTTGACCTTGCTACTGGAGAAAAGCAGGTACTAAACGATATCAGGGGGCGCTATGAGTGCTACACGGATGTTGGACCATCATTCCAGTCCATG
AAGCAGCAAAACCGCGCAGAAATTCTTGAGTTGCTCGGCAAGACGCCACAGGGAACGCCAGAATATCAACTGCTGTTGCTTCAGTACTTCACCCTGCTTGATGGTAAAGGTGTTGAGATGA
TGCGTGACTATGCAAACAAGCAGCTTATTCAGATGGGCGTTAAGAAGCCAGAAACGCCCGAAGAGCAGCAATGGTTAGTAGAGGCGCAACAAGCCAAACAAGGTCAACAAGACCCGGCA
ATGGTTCAGGCTCAGGGCGTACTCCTGCAGGGGCAGGCTGAACTGGCTAAAGCTCAGAACCAGACGCTGTCCCTGCAAATCGATGCAGCTAAAGTCGAAGCGCAGAACCAGCTTAACGCT
GCCAGAATCGCAGAAATCTTCAACAACATGGACCTCAGTAAACAATCTGAGTTTAGAGAGTTCCTTAAAACCGTTGCTTCATTCCAGCAGGACCGCAGCGAAGACGCTCGCGCAAATGCTG
AGTTACTCCTTAAAGGCGATGAACAGACGCACAAGCAGCGAATGGACATTGCCAACATCCTGCAATCGCAGAGACAAAATCAACCTTCCGGCAGTGTAGCCGAGACACCTCAATAA
>DOCPEC_16650 Terminase
ATGTCATCAGAATTTGAGGCAATGCTTGCCGATGATATGGGGCGATTCTTCTATGACCCACTCGGATTTGTGATGTATGCATTTGAGTGGGGAGTTGGCGAGCTTGATGGCTTCGACGGTCCA
GATGAGTGGCAGAGAGAATTTCTCACTGATTGGGGTGAGGCGATTCGCACTAATAACTTTGATGGTGTAAAGCCGGTAGAAGCATACCGATGCGCAACAAGCTCAGGCCACGGCATCGGG
AAAAGTGCGCTCACTGCATGGGTAATTCTCTACATCATGAGCACCCGCCCGTTCTGCAAGGGCGTTGTAACAGCTAATACCTCAGAGCAGCTTCGAACCAAAACATGGGGCGAGCTTGGAA
AATGGAAGAAGCGTTGTATCACCGGGCACTGGTTCGAATACAACAACGGCAAAGGCAACATGAACATCTACCATGTAGATCACATGGAGTCATGGCGTTGTGACGGCCAGACCTGCCGCGA
GGAAAACAGTGAATCATTTGCTGGTCTTCATGCTGCAAACTCAAGCCCATTCTACATCTTCGATGAAGCTTCTGCGGTGCCTGACAAGATTTGGGAGGTGGCAGAGGGCGGACTGACAGAC
GGAGAGCCTTTCTGGTTCGCGTTCGGCAACCCGACACGTAACACCGGGCGTTTCCGCGAATGTTTCCGTAAATTCAAGCATCGCTGGAGACGCAAGCAAATTGATAGCCGTCTGGCGAAG

ATGACGAACAAAGAGCTTATTGAAGAATGGCGCAACGATTACGGTGAGGATAGCGACTTCTTTAAAGTACGCGTTCGTGGCCTCTTCCCGTCTGCGTCTGACCTGCAATTTATTCCCCAAAG
CTATGCTGATGCCGGTATGTCAAGAAAGCTGGAGCACAGTCAATATGGATTCGCTCCAAAGATTATCGGCGTTGACCCGGCATACTCAGGCAGTGATGAGGCATGTATCTATCTGCGGCAGG
GGCTTTATTCAAGGCTTTTAGGCTCTTACCCTAAAACAGACGACGATGTGAAGTTTGCTCAGGTAGTGGCTGCTATCGAGGATGAACACAAAGCTGACGCGGTGTTCATTGATTTCGGTTAC
GGCACGGGTATTCATTCTGTTGGTAAGTCGTGGGGCAGAAAGTGGCAACTTGTGAGCTTCGCGGGAGAATCGAAAGACCCGGCAATGCTCAATAAGCGCGGCGAGATGTGGAACGCAAT
GAAATCCTGGCTGAATGAAGGCGGAAGCATTGATGACCAGCAGACCGCTGATGAGATTGTCGCCCCTGAATACAAAGTAAAGCTAGACGGTAAGATTGTTCTGGAGTCGAAAGACGATATG
AAACGCCGTGGCGTTCCCTCTCCAAACAGGGCCGATGCGCTGGCGCTGACATTTGCATTCCCGGTAGTCAAAAACAAACCATCAAAAGCAATCCCCGCACCGATTAGACCAGTACGCAGAG
GACGATAA
>DOCPEC_16655 Terminase
ATGGCGGCTGAAGATAAGAAAATTGGTCGCCCATCGGCTTACAAACCAGAGTATGCCGAGCAGGCGAGAAAACTTTGTCTGTTAGGGCATACAGATGCGGAACTGGCTTCTTTCTTTGATG
TTAGCGAGCAAACAATCAATGCGTGGAAGCACGCGCATCCTGATTTTCTTGAGTCCATAAAAAAATGGTAAGGCTGTTGCGGACAGTGAAGTCGCCGCAAAGTTATTCCACCGCGCCACCGG
GTACGAACACCTAGAAGATGATATTCGCGCTGTCGATGGCTCGATCGTCATAACTCCTACTGTGAAACATTATCCACCCGATACGACTGCTGCCATTTTCTGGCTTAAGAACAGGCAGCGAGA
TAAATGGCGTGACAAACAGGAAGTAGAACACACCGGAGAGGTTAGCCTGATTCAGCGCATTCAGGAGGCCCGTAAACGCGCAAGGGGTGAGTGA
>DOCPEC_16660 Decoration protein
ATGGCAAACCCAAACTTCACGCCATCATGGCCTCTATACAAAGATGCTGACGGTGTATATGTGTCTGCTCTTCCGATTAAAGCTATCAAATACGCTAATGACGGAAATGCAAACGCAGAATTCG
ACGGTCCGTATGCTGACCAGTACATGTCAGCGCAAACAGTAGCCGTATTCAAGCCGGAGGTCGGTGGATATCTGTTCCGAAGCCAGTACGGAGAGCTGCTCTATATGAGCAAGGCAGCATT
TGAAGCTAAGTACACTTCTGCAAGCGGTTCAGTAACGAATGCAGAGACGGCGGATAAGTTATCTACTGCTCGCACTATCACACTAACCGGCGCTGTCACAGGTTCAACGTCATTCGATGGTT
CTGCTAACGTGACTATCGCAACTACCCAAGGAAGTTAA
>DOCPEC_16665 hypothetical protein
ATGGCAAAGACGAAGTGGCCTAAACTTCCCCGGTTCTTCGTGCCATTGTTCCATAGCGCCAATGTCTACCTGTGTCGTTCAAAGGAAGAGTGGGATCAGGCTTGCATTCATCTTGGAGTTGA
TAGCGGCGGGAATGAGATGCTGGCGGGGCAACACAGTCATATTGCAATACCGAAACAGGCGAGAGCCTTTACCTGCTTGGGGTATTCAATGGAGATGCTGCCACACTGGTTCATGAGTGTG
CTCACGTCGCATTCTATGTCTGCCGAGATGTTGGTGTAA
>DOCPEC_16670 DUF2560 domain-containing protein
ATGGCAGAGATTACAGCATTGACAGAATTACAGCAGATGAACCTCGATATCCTCCGTTTAGTTCAAAGCGATACCGCAGCAGCAGAGAAAGCGATCGCATTCGTTGCTGGAAGTAAGCTGA
ACTTCGAACTGTTCAAAGACCAACTGGTTTTGGCGCAGGGTGAAGGAACGGCATTAGCTCGCGCAGAAAAGGCTATTCGTGAGGCAAAAGAAGCGTTAGACCTGTTCACTGCCGGAACA
TAA
>DOCPEC_16675 KilA-N domain-containing protein
ATGGTCAATGGTGTGTCCGTTCGTGTTGATGAGGACGGACGCTACAACTTAAACGATCTCCATGCAGCTGCAGTTGCAAACGGAGAGGCTACAGAACAACAGCGCCCAAGCCAGTTTTTGC
GTAGTGCGCAGATAAAACGCTTCATAAAAGCACTAGAGGTCAAAGTGCAAAAAAGCACTTTGAAACAAATTCAACCACTTAAAATCATTAAAGGTGGTGCCGAACCTGGTGTGTGGGGCG
TTGAACTACTGGCAATCAGATATGCAGCATGGATTAAGCCGGAATTTGAAATCGAAGTTTATGAAGTTTTCAAAACGATTGTCCGTCTCGGCGTTGGTGCCATGTCTCGCCTGAACAAAATTG
ACCACATCATCAACACTGAAACCAAAGCGATAAGCCAGTGCGCAAGTCAAATGGCTAAGTGGGGAATTGGTGGTCGAAAAAGATTGCTTCATGTTGCACGTGAGAGAGCGGCAAATGAAG
TGCAAATGTATTTGCCCGACATGGTGTGA
>DOCPEC_16680 Rz
ATGACCAACCTAATCGCATCAGGCTGGCAATACATCTTAGGCATCGTTGGTATTGTCGTTGCTGTTCTGGCTGCATGGTTCTCTGGAAGGAGCAAGGGGACAACAGAGGCCAAAGCTAAGG
CTGATGTTGAGTCAGCTCACCAGACCGTTAAGCAATCACAAGCCGCCAGTAATAAGCAAGCCTCAATCATTAAGGTTGCCAAAGATGCAGACCAAACGAATCAGTCTCTTTCTGATAACGCT
GCTCGTGACCGCATGCGGAAATCAAAGTACCACTCCGACGATTAA
>DOCPEC_16685 hypothetical protein
ATGAATGGCTGGCTCTCGGCGGTTTCGTTCTGGCAGTATTTACATTCGCCATTAATCTCCATTACCAACGAAAACGAGATCGCAGAGAAGAAAACGCTCTACGGATGCAGCATGGAGACCAG
CGCAATGAGTCAAATAATCCCCCTGCTTAACTTCGAAGAGGGTTATCGTGAGAAACCCTACATTGATACTGAAGGCTATCCGACAGTGGCTTGTGGTATCAGAATTGGCCCTAAAGGCGCTTC
GTTAAACAATTACACTTTCACCGTTCCTCGAGATGTTGGCGATGCATGGCTTGAATCATTCGTCAAAACCACCATCATTAAGATGAACACCAACCCCTCCATTGTAGCTGCAATGAAATCCTGC
AACCCAGCACGCCGAGACATCCTGATAAGCATGGCTTACCAGATGGGGGTAAGTGGTCTTGCTGGATTCAAGAATACGCTGGCAATGGTCGCAGAAGGAAACTATGCAGGCGCAGCTAAC
GGAATGCTATCAAGCCTGTGGGCTAAGCAAACACCTGAGCGCGCTAAACGTCATGCTGAGGTGATGCGCACTGGTGAGATGGCTGCATATGCGGGGCTGCTATGA
>DOCPEC_16690 Antiterminator
GTGGGCATAAGAGAACTAAACCTCACCAAAGAGCAGCACGAGTGGCTGAATGGCTGGCTTGAACTGTGGGGCGCATGGGTTTATTCAGGTCGTCTGGAAAAGCGCATGAGCAGCGTAAT
AGCGAAGTTCATGGAGAGCGTAGAGCCGGGAAGAGTTATGACAAGGCCAATGTGCAATGATGATGATGGAATGTTGATTTCTCAGGTCGTCGATTCCGTCATGTACATTGACAAAAAAGCC
TTTGGCATCCTCCTCAGCTATTACGCACATGGTTCCTCTAAGTACGCCATATCATCCTACTATCACAAGACTGCAAGTCCCCGCAAAATGTCAGGCCGCGGCGGAGAAAGGATGCGCAAGCCA
TCTCTTATTACCTGTCGGAGAGAAGTAGATGACGTACTCAAGGCATCCCTGTTCATGCTTTATCAACCGATGCTAAATGCCTTCAATAGTCGTAAACGTGTGGATAAAATTAAACATGTTGCATA
G
>DOCPEC_16695 Serine/threonine-protein phosphatase
ATGCGCTATTACGAAAGAATTGATGGCAGCAAATACCGAAATATTTGGGTAGTTGGCGACCTGCACGGATGCTACACGAACCTGATGAAAAAACTGGAGACGATAGGATTCGACACCAAAA
AAGACCTGCTTATCTCGGTTGGCGATTTGGTCGATCGCGGTACAGAGAACGTCGAATGCCTGGAATTAATCACATTCCCCTGGTTCAGAGCTGTACGTGGAAACCATGAGCAAATGATGATT
GATGGCTTATCAGAGCGCGGAAACGTCAATCACTGGATGCTTAATGGCGGTGGCTGGTTCTTTAATCTCGATTACGACAAAGAAATTCTGGCTAAAGCTCTTGCCCATAAAGCAGATGAACT
TCCGTTAATCATCGAACTGGTGAGTAAAGGTAAAAAATATGTCATCTGCCACGCCGATTATCCTTGTGACGAATACGAGTTTGGAAAGCCAGTTGATCATCAGCAGGTAATCTGGAACCGCGA
ACGAATCAGCAACTCACAAGACGGGATCGTGAAAGAAATCAAAGGAGCGGACACGTTCATCTTTGGTCATACGCCAGCAGTGAAACCACTCAAGTTTGCCAACCAGATGTATATCGATACC
GGCGCAGTGTTCTGCGGAAACCTCACATTGATTCAGGTACAGGGAGAAGGCGCGTGGGCATAA
>DOCPEC_16700 Protein ninH
ATGACGTTCTCAGTAAAAACCATTCCAGACATGCTCGTTGAAGCATACGGAAATCAGACAGAAGTAGCACGCAGACTGAAATGTAGTCGCGTTACGGTCAGAAAATACGTTGATGATAAAG
ACGGGAAAATGCACGCCATCGTCAACGACGTTCTCATGGTTCATCGCGGATGGAGCGAAAGAGATGCGCTATTACGAAAGAATTGA
>DOCPEC_16705 Recombination protein NinG
ATGGAAAAAGCAGAAAAAGCAGCAGAGAAGAAACGACGACGAGAGGAGCAAAAACAGAAAGATAAACTGAAGATTCGAAAACTCGTCTTAAAGCCCCGCAGTTACTGGATTAAACAAG
CCCAACAAGCCGTAAACGCCTTCATCAGAGAAAGAGACCGCGACTTACCGTGTATCTCGTGCGGAACGCTCACGTCTGCTCAGTGGGATGCCGGGCATTACCGGACAACCGCTGCGGCAC
CTCAGCTCCGATTTGATGAACGCAATATCCATAAGCAATGCGTCGTGTGCAATCAACACAAGAGCGGGAACCTGGTTCCTTATCGCGTGATGCTCATCGAGCGCATAGGGATTGCAGCAGTA
GACGAAATCGAATCAAACCATAGCCGCCATCGCTGGACTGTCGAAGAGTGCAGGTCCATCAAGGCAGAGTACCAACAGAAACTCAAAGACCTGCGAAATAGCAGAAGTGAGGCCGCATG
A
>DOCPEC_16710 DNA helicase
ATGACCGATAATTTTTACGCTCCGCCACATAGCATTGAGGCGGAGCAGGCGGTGATTGGTGGATTGCTTCTGGATGATGACAGCAGTGAGCGCGTCCAGAAAGTTCTGGCGATGCTGAAGC
CTGATTCATTTTACAGCCGACCACACAAAATCCTTTTCGAAGAAATAACCAGAATGCACCGGGAGCAAAAGCCAGTAGATGGCCTGACGCTTTTCGATGAACTGGAGCGTAAATCGTTAACG
GTGTCAGTTGGCGGTTTTGCTTATATCGCTGATATCGCAAAGAACACGCCAAGCGCAGCAAACATCGTTGCCTATGCAATGCAGGTTCGCGAAACCGCAATGGAACGCTACGCCATCAACCG
CATGACTGAAGCGACGGAATTGCTCTATTCCCGCAACGGAATGACTGCAACGCAGAAGTACGAAGCTATTCAGGCGATTTTCACGCAACTGACAGACCATGCAAAAACCGGATCACGTCGC
GGCCTTCGCTCATTTGGTGAGGTCATGGAGGACTGGGTTGGTGACCTTGAGAAGCGATTTGACCCGTCAGGCGAACAACGAGGAATGAGCACAGGGATCCCATCGCTGGACAGGATGCT
GTCACCGAAAGGTCTGGTGAAAGGCTCTCTGTTTGTCATTGGCGCTCGCCCTAAGATGGGGAAAACGACGCTATACAGCCAGATGGCAATCAACTGCGCAGTGCATGAGAAAAAGCCCGC
TCTGATGTTCAGCCTTGAAATGCCAGGTGACCAGATACTGGAAAAACTGGTAGGGCAGAAGTCAGGTGTTAACCCGAATATTTTTTACCTTCCGGCGACAAATGACGCTGATGACGGCTATC
AGGGTGATTACGATGGTGACTTCAACAGGGCGATCGAAACAGCCAATCGCTTGAGTGAAATCGACCTGCTTTACATCGACGACACGCCGGGATTATCTCTGGCTCAAATCGTCAGCGAAAG
CCGTCGAATCAAGCGAGAAAAAGGATGTGTTGGCATGATTCTGGTCGATTACCTGACACTAATGACCGCTGAAAAGGCCGATCGCAACGACCTTGCCTACGGCATGATCACCAAAGGACTG
AAGAACCTTGCCAAAGAGCTTGATTGCGTTGTTGTGCTTCTGACGCAGCTTAACCGCGCACTGGAAAGCAGAACCAATAAACGCCCATTACCAAGTGACTCACGTGATACAGGGCAGATTG

AACAGGATTGCGATTATTGGGTGGGGATCCATCGTGAAGGTGCTTTTGATGACAGTGTTCCACCTGGTGAAACCGAACTAATCCTTCGTCTCAATCGTCATGGCAATACCGGCACGGTGTATT
GCATTCAGGCAAATGGCGCTATTTATGACACAGACCAACAGTCTGCTGAAATGCGCCGCCGTGAACGCGAGGAACCGCAGTCCAAGAAGAAAGGAGGATTCTGA
>DOCPEC_16715 DNA replication protein
ATGAGTAACATATCCAATCTAGCCGAAGCCAGAGAGGCCAGAAGGCTCCAGAAGCCGCGTACAAATGGCGGTAAGGGGTTTGCCTTGATTCACCGCCAATTCATGGATAGCAAGCTATACA
AGGATTCTCAGGCTGTGCATCTTTTCCTGCATCTGATACTGAAAGCCAATCACTCTCCGGCAGTCGTAAATACCGACATTGGTGAGATGTTGGTTGAGCGAGGACAGCTAATTACCGGACGG
CCAAAACTGGTAAGTGAAACATTCATCCCGGATAACAAAGTAAAAAGTTTGCTTCGTTCTTTTGAAGGGAATGGAATGATTCGTATCGAGTCGAAAGGGAGAAAATTCAGCCTGATAACAG
TGTTGAAATATGATGATTTTCAGGCTCCAAATTGTCCAACGGATGTCCAACGGATGTCCAACGCAAACACCAGTAATGACGCGGCTCACAGCAAATGTTGTCCAACTGATGTCCAACGATTGT
CCATAAACAATAATATAAATAATATCTCTAATACTAACGTATTAGAGAGTACCGCAGCAGACGAAAATCCTGACAAGAAAAAATCGGCTCTCAGTTGTCAGGATGTTGTCGATGCTTACCACGA
ATTACTTCCTGAAGCTTCCAGGGTTCGCGCACTGAATGACAAACGTAAAAACCAGATCCGAACTTTCTGGCGAAAAGCCGGAGTGATAACACGCCAACTTGACGGGCATGGGTTCACGATG
CAGGACTGGAGAAATTATCTGAGCTACGTAGGCGAAAATTGCCGATGGATGTTCGAAGAGCGCCAAAACCATCAACGCGGAACCGTCTGGCACAAAAAGGGATTTGATTTCCTGCTTAAC
GATAATACCTACCTGAAAGTTCGTGAGGGTGAACACGATGACCGATAA
>DOCPEC_16720 hypothetical protein
ATGCCAAAACAACTCAGTCCTGACCAGGACAAATTACACAAAAACATACTACGTGATCGGTTCTTATCCAGCTTCAAACAGCCTGGTCGATTTCGGGCTGAGTTGGAGAAAGTGAAGCTAAT
ACTGAAGAGGAAAGGTCATGAGTAA
>DOCPEC_16725 Transcriptional regulator
ATGGAACTCACAAGCACTCGCAAGAAAGCCAACGCAATTACCAGCAGCATTCTGAACCGGATAGCTATTCGTGGTCAGCGGAAAGTCGCTGATGCGTTAGGCATTAACGAATCTCAAATTTC
ACGATGGAAAGGCGATTTCATCCCGAAGATGGGAATGTTATTGGCGGTTCTGGAGTGGGGTGTCGAGGATGAGGAGTTGGCAGAACTGGCAAAGAAAGTTGCGCATCTGCTGACAAAAG
AAAAAGCCCCGAAGAACGGCGAATTCTTCGAGGCCTGA
>DOCPEC_16730 HTH cro/C1-type domain-containing protein
ATGAGCAATCTTCGGAAAATCCGGGAAACCATGAAGGTATCCCAGGCCGTTCTGGCCGAAAAAGTTGGGTGTACTCAGGGAGCAATTGGTCATTACGAATCAGGGCGACGCCATCCGGAT
TTGAGAATGTGCCGCCAGCTCGTAGAGGCGCTCAACAGTTTTGGCGCGAATGTTCAGCTAGACGATGTGTTCCCACCTGAACTTAATGCTGCCTAA
>DOCPEC_16735 Repressor protein CI
ATGTGTGTTGTCACAGTCAATACATCTCGTAATAATCGGCGCATGAAAACACCGTGGAATGAGCTGGCGAAAGCCAGAATGAAACAAATAGGCCTAACCCAGGATAAACTTGCTGAAGCTCT
CGGTAAGACTCAGGGGGCGATAGGTCATTGGCTTAATGGCCGCCGCGAACCAAGTATTGAAGATATTGCAGCGATCATGAAGCAGCTAGGATTGAAGGAGCTAGTATTAAGTTCTGATGGG
ATGGTTGATTATCCAGACTCCAACCTGAACAATGTTTCAAGTCCTCGTCCACACACAGAAATAAGGAGGTTTCCCCTGATTAGCTGGGTGAGCGCAGGTAACTGGTGTGAGGCTGTTGAACC
TTACCAACTCCGAGAAATAGAGGTGTGGCCTGAGACAACTGCACATGCAAGCGAAAGGTCATTCTGGCTAACCGTTCGTGGCGACTCTATGACATCTCCTACAGGATTAAGCATACCGGAAG
GAATGCAAATTCTTGTTGATCCGGCTATCGAACCGACTAATGGAAGACTCGTGGTGGCAAAGCTTGAGTCTGAAAACGAGGCAACCTTCAAGAAATATATTGTTGACGCTGGACAGAAATAT
CTTAAACCGTTAAACCCCAGCTATCACATGATCCCCATAAACGGAAATTGCCGCATTATCGGTGTTGTCATTGAAGCAAAATGGCAAGGCCTCTAA
>DOCPEC_16740 24 protein
ATGACGGTAATCGTGTACGGAAAATCAACATTTGCAGGAAATGCCAAAACTCGCCGTCATGAGCGGCGCAGAAAGCTGGCTATCGAGCGTGATGCTATATGCAACATAATCGATTCGATCTTC
GGCACAGACAGTGAGGAAGCTGTTCAGGAAGAACCGAGAAAGCGTTTAAGTCTTTCTGAAAAAGCAATATCACTCGGCATCATCAGTAAATCAAATACAGATGCGAATGGCGGAAGCGTT
TGTTTGCCTGAAGTTGCACTGTATGCTGCTGGTTACAGGTCAATGAAATCAATTACGGCGAGATAG
>DOCPEC_16745 Transposase
ATGACAAAATCATGGAGCGTACCTTTTCCTGAATCAGAAACTGAACATGATGGAATGCCTGTTTTCTGGAGATTCCAAGCGACAGTTGAAGAAGATGGAATCAAAATATTCGCACTTCAATAT
ATAGCTTTTCATCAGACAGAGCATTATGCATGGTTGGTTCCTGCACATTGGATTGTTAATTTTAAACCAGCACCAAATCAGTGGTTACAGGAATGGAAACAAAGGAGAAATAGATATGCAATT
AAGAAAGTAGCAAAAAATGCAGAAAGATCTTTTGCATTCCCGACGAAGAAACTTGCCATTGAAAGTTTATTGCGCCGAAAGAAATACCATTTGATGAGAATCAAACAAGATTTGGCTGTTG
TATCAACTCTTGTTGATGGGATGAAGAATATTGATACATCAACACCAGATATTGAATATAACTTTGGACACAACCAAGAAACAGAAAATTGGGTATTTTATTAG
>DOCPEC_16750 Superinfection exclusion protein
ATGAAATTACGTGTCTGGCATATCCCGCAAGTACCTATGAAACCGTTCATTGTAGAAGTGGCAAGTGTTGAAGAGGGTGTTCGCCTGATGGATGCACTGGCTGATTATGACGCCTTTCAGTAT
GACAACAACATCAAGCCTGACTACTGCAATGCTAACGGCCTTGAGATGTGGGATGAGAGTCTTACCGATGAAGATTTATCAGAGATGGGGTTTACGGATCGCTGGGTGGATTGGTACAGCG
AATGTCAATGTTACGACGACCCACGTAAATATATCGAAAGCCTGAAAGAAGAAATCTCAGCCGCCTGA
>DOCPEC_16755 CIII family protein
ATGATGCACTTTCAGCTCGCGGGTAGCGGCGTCATGTCCGCTTTCTACCCGCACGAATCTGAATTATCACGCCGAGTTAAACAATTAATCAGAGCAGCAAAGAAACAACTGGAGGCGTTATG
CGCAATGAAATAG
>DOCPEC_16760 hypothetical protein
ATGCGCAATGAAATAGTCATTAATCACCAGATGCTTCGTGCAGCACAAAACAAAGCAGTAATAGCCAGATTTATTGGTGATTCAAAAATGTGGCTTGAAGCAAATAAAGCGATGAAATCAGC
TATCAACCTTCCGTGGTATCGCAGGAAATGA
>DOCPEC_16765 TPR-REGION domain-containing protein
ATGTCACTTGCAACCACAGTGAAGGAGAGCAAGTTACAGAGACGCATGTACACGCAGCAGGCGTTAATGTATCGCCAGAAGGGAGATCGTGAAGGTGTTCGCGTATTTTTGAATGCGGCA
AAGACTGAAGTATTAAATCAGCGTTATTTCCTTGGGCCATGTCCATTCTGA
>DOCPEC_16770 ERF family protein
ATGAGCAAAGAATTTTACGCAAGACTGGCAGCTATTCAGGAGAATCTGAACGCGCCAAAGAATCAGTACAACTCATTCGGCAAATATAAATACAGAAGCTGCGAAGACATTCTTGAAGGCG
TTAAGCCGTTACTGAATGGCCTGTTTTTATCAATCAGCGATGAAGTTGTGTTGATTGGTGATCGGTATTACGTGAAAGCCACGGCAACTATTACCGATGGCGAAAACAGTCATACGGCAACCG
CTCTTGCACGAGAGGAAGAAAGCAAGAAAGGAATGGATTCTGCACAAGTTACGGGAGCTACAAGCTCTTATGCACGCAAGTATTGCCTCAATGGTTTGTTCGGCATTGATGATGCGAAAGA
TGCAGATACAGACGAGCATAAACATCAGCAGAACGCAGCAGCAAAGCAATCAAAACCATCACCTACACCTGAACAGGTTCTAAAAGCATTCACTGACGCAGCAATGCAGAAAAACACCGTA
GAAGAGCTTAAACAGGCGTTCGCCAAAGCGTGGAAGATGCTCGAAGGCACACCGGAGCAGCACAAAGCGCAGGACGTTTACAACATCAGACGAGACGAATTAGAAGGAGCGGCTGCTT
AA
>DOCPEC_16775 Bacteriophage HK97 gp40
ATGGCACATTCGATTACTGTAAGACTAAACAAGCCCGCAAGAGAGTTTCAGGCCGGGGAAAATATCGGATTCAACATCCGTGCTGGCGTTCAGTATTACGATCGCCAGACAAAAAAGAAAG
AATGGACAAACTACAGCGCCGTTGTATTTGCCAAGCCGGGAGCGCAAGCGGATTACTACCGTAGTGTTCTTGTTGAAGGTGGCATTGTTGAAATTACCGGAGAAAACATCAGGGTTGATGT
TTATCAGGGGCAAAATGGTCAATCAATCAATCTTGAATTACTGAATGCAAAGATTGGATTTGCAACTTCAGGAAACAGCCAACAGCAGCAAAGTAGCAATCATCAAAATCATCCTGAATACG
ACGATTCAATTCCCTTCTAG
>DOCPEC_16780 Anti-RecBCD protein 2
ATGCCAGCGCCTCTGTATGGTGCGGATGACCCGCGCCGCTGTTCCGGCAATTCCGTATCGGAGGTGCTGGATAAATTCAGAAAAAACTACGATCGGATAATGTCGCTACCGCAGGAAACGA
AAAAGGAAAAGGAATTTCGCCACTGTATATGGCTTGCAGAGAAAGAAGAACGCGAGCGAATTTACCAGACATCAATCCGACCATTCCGCAAAGCCACATATACCCACTTCCCTGAAATTGAC
CCGCGCCTGCGTAATTACCGATCACGCTATGGCGCTATCAGTAATGACTGA
>DOCPEC_16785 hypothetical protein
ATGAAAGAAGTGAAAATATACACGATTGTCAGTGACCAGTTATCACCACCAATAACAGGAGAATCATTCTGTACTGACATGGTGCGTCATAGTGATTATGCGGAGCTTGAGGATAAATACGCG
GCGTTGGCTGCGGATAACGATAAAGCAATGGAGTCACTTAAGCAGGCTGATGCAGTTGTTAAGTTGGCACACGAGAAGTTTTCGGCGCTGGCTTCGGAGAATGCGGCACTGAAAAAATCA
GAGGTCGAATTCAACGAATATTGTCGTCGCGAGTGCGAGGACGTTGGCGATACGTGGGTGGACGATTTCACTGAGACCCCAGCCACCGACACTTTTCTGGCTGAGGTGACACAAGAAGCA
GTAGAGTCACTCAAGAAGGAGATTGAATGGCTCAAAAAGCAGTTGCTTATGTCTGTCGGTGATATTCGAGACCTTATTGATTACAACCCTGAAAATGGCGTTCTTACAGCGAAAGTTAACTTC
AGCGGAAGGCAGGCAGGTTCTGTCATTGGCTCTCAAACTTGGCAGGGATATTACGCATTCTCACTGTTCGGTAAGAAGTGCTTTGCTCACAGACTTGCATGGCTACTGCATTACGGTGAGTG
GCCTTCACAACCTATCGATCACATAAACGGAATTAAGACGGACAACAGCATAAGAAATCTGCGCCTTTGTTCTCTATCGCAGAACCAATTCAACAAGCCTACTCAGAAAAACAACACTACAG
GTGTGAAGGGTGTGTATTGGAACAAGCGAGATAAACGCTACGTTGCCAGCGTCCAATTTAACGGAAAGAAATACAGCGCGGGACATCACAAGGATATTGATAGCGCTAAAGAGGCTGTAAT
GAAACTTCGGGAAAAACTGGCTGGAGAATTCACTAACCACGGCGAATTCGAACTCGCCGCCCAACTTCGCAAAGGAGCCTCGCTATGA

>DOCPEC_16790 ClpB-D2-small domain-containing protein
ATGAGCAATACAGCAAAACTTCAACTCGGATTCTCTCCTTTATCAAAAACCATCATGCTCGCAAAAATGCGCGATGTGGAAGGTGGCCGCCTGCGCGTCGGTAATGATCGTGGTCGTGATGT
TACCAATGAGGCTGCGCAGCTAGTGTGGCAGCTTGTCATGGCGGAAGGTGGTGAAATCGGCTGGGAGCTTGATGATGGTTCTCGCATGGTGCTGAAAGCTGAGAAGCAGGAGGCAACCA
GTGAGCAAAATTGA
>DOCPEC_16795 hypothetical protein
GTGAGCAAAATTGACCATCAGGCACTGCGTGAGGCAGCAGAACGTGCAACTCCAGCAATGGAACGCCTGTTAATGTTGCCTGTTGATGATGATTTGTTAAGTGAACAGGAACTTAAAGATT
GCGGTGTGGATATTGATGCGCTCAACGCCTTCAAATTTCTGACCGGACCAGAAACCGTGCTGGCGCTGCTGGATGAGAATATTCAACTCCAGCGGGGAAAAGACGCAATAGAGGCCGTAG
CGCTGGTGCTGCGTGATGATATGCGACAGGCGCGAGAGCAACTGGCAGCCGCAGAGAAGCGTATTGCTGAACAGCGTGAGTATTACGAGGGCGTTATTGCTGATGGAAGTAAGCGCATAG
CAGAACTCGAACACAGCGAGACGCAGCTTATCAATGAGCGTGATAGTGCTGAATCTGCACTGGCCGATATGTACCAGGCCGCAACAGGAGAGCGTCCAGAATGGAGCAATATGTTTGGTTT
CGCTGACGCCGTTGATGTGGTGGAAGAACGACTGGCGACGCTGGAGGCCAACCAAAGCCAAACCACGCCAACGGGAATTCAGCTCATCACAGAAGCCATAGGTGCGCACGGCTATATCG
TTGGCTGCCTGTTGCAAGGTCGCCCTGATTTGGCGCTGGAAGAATCGAGAAAGTGGGTATCCGCTTTCGGTCAGGCGGCGGAAATAGTTAGTGCACAAGACGCCGATGACATCAAGGTTA
AGGGGGAGTGA
>DOCPEC_16800 Prophage protein
ATGACCACTATTACCAGAGAACAGCAAAAACAGATTTTAATTGATACAGCGAACCACGTAATCAGTCGTGATAACACGTCACCGTATAGCGAAAACCTGCGTGAACTGGCGCGTATCGCGCT
GGCATCGCTCGAAGCGGAGAAAGGTGCCGACCCCGTTGTGTTCACCGACGAACGAAATCTTCATCATATTGCCAGGGGTCGAGAAACCTCTTTGATTTGGGGTAAACAAAACCAGGAGGT
GGGGGATATCCCGCTCTATCGTCACGCCCAGCCAGTGCCGGTAGTGCCTGATGAAATGGCGACATCTGATGACATGAATCTTTATCAAAAGAGCTTTGCGCAAGGCTATAACGCCTGCCGCA
ATGCCATGCTCAACGGAGGTAAATCGTGA
>DOCPEC_16805 DUF551 domain-containing protein
GTGAAAGAGAATCAAATCCGGGAGCTTGTAAACGAGCTGCATGATATTGCTATTGAGTATCACGGCACACAACAGTTACGTGAACGAATTGCGCGTACAGTTCGCGCCGCCCTGCATCATGA
CTTAGAAAAACTAAACCAACCTGTAAGCCAAACTTACGAGTTGCCAGAATTAATCGAAGGCATGGAGGTGTCCATTGATGTCAGCACTTGTGATGCTGATGCCGGGAATCGCTATTTCGGTA
CTGTCACCGAGGTATCAGAACTGGACACAGCAAAGAATGGCTACATTCTTCTGGTTCAGGACGCTGAACCAAATTTCGATGTGAATGGCAACTCTCCGGTAATTCCGGATGGCTGGATAAGC
TGTAGTGAGCGAATGCCGGATAATGATGAATCTAAACCCATCGCAATTTTTACCGGAAAATGTCTGGGTCAGGGGATGTTCGTTGCTACATACGACGATGATGGGTTCTTTGACTATTGGGAG
GGTATGGAAATTATCGGTGTAACCCACTGGATGCCGCTACCGGAGCCACCGCAGGAGGTGAACCGTGGCTAA
>DOCPEC_16810 ASCH domain-containing protein
GTGGCTAACCTGCAACTTGCCGTCAAAGGTGAATACTTCGATGCCATGATTCGCGGGGAGAAAACGGAAGAGTATCGCTTGTGTAATGACTACTGGAATAAGCGAATTATGTTCCGCGAGTA
TGACCGACTGATTATCACAAAGGGATATCCAAAGCGCGACGATTCCAGTCGCAGAATTGATGTTCCGTACGACGGATATGAAATCAAGACAATCACACATCCCCACTTCGGCGATAAACTGGT
GAAGGTATTCGCGATAAAGGTGAATATCGGCAATGAATAA
>DOCPEC_16815 Eac protein
ATGTCAGACCAGAGCAAGTATTACGATTACTACATGGTTGAAGGTGATGATGTTAAGGAACTTATCAGTTCATACGATACCATTAACGAACAACGTAATTCTATCCTCCCAGCCGCAGCAGAAC
AGGTTGGTGCTATAGCATGGACAACAACTCGTAATTGGGGTGGTGGAGGTGGCTTGCTGCAAAGTTTCGTTTGGGAAAAAGGATATGAATTCCCATGCCAGATAACAATCAAACGCGAGG
ATTTTTGGGACGGGAAGAGAGTTGTGATAGCGCGAGGAAAGGGAAACACAAAGGAAGGCCGCGCATACAACAAAGAGCTGGATGCAATCATGCATAACGCTAACGCCAAGCTAAAATCC
TTGCCTGAATGGAATTACTACATAACCAACCACTACGGGATTATGCGTACAGGAATTGGTGGTCAATCGGGCCGTGGACTTGGTTTCGTTATGTTATCAACGTATGGCGGTAAGCACCCGAAG
CGCAATGATTGTCTTATTTTTGCAATACCAAATAACAAAGAAGAGAGGCATGGCGAAGTTGTTATCCCTGACAGCTTCAAGAAAATAACTTACGGGAAATTCTACGACATCGCGAATGAAGTT
GAAGAAGAAGCTGTGGAGTAA
>DOCPEC_16820 Prophage integrase IntD
ATGTCCATCTTCCGCAGAGGTGAAATATGGTACGCGTCCTACTCGACACCGGGCGGGAAGCGAATTAAGGAAAGCCTTGGGACTTCCGACAAGCGGCTCGCTACTGAGCTACATGACAAGC
GCAAGGCTGAATTGTGGCGAGTAGACAGGCTTGGCGATTTTCCTGACGTAACGTTTGATGATGCCTGCATGCGCTGGCTGGAGGAAAAGGCAGAGAAGAAATCACTGAAGGATGACCGC
AGTCGTATGGCTTTCTGGCTGGCGCATTTTGAGGGAGTACGGTTAAAGGATGTAACCGAGCAAAAGATTTACTTAGCAGTAAACAAGATGAGCAACCGCAAACAGCTTGAGATATGGAAAA
TCAAAGCTGCCGCGGCGCAGAAGAATGGAGAACCTGCACCAATCTATTCAGCTAAACCGGTCACAACCTCCACTAAGGCCAAACACCTGGCATTAATGAAGGCTATTCTGCGTGCAGCAGA
ACGTGACTGGAAATGGCTGGAGAAAGCGCCTGTAATCAAGGTTCCAGCCGTCAGAAACAAACGCGTCAGATGGCTGGAGAAAGAAGAGGCAAAACGCCTGATTGATGAATGCCCTGAAC
CGTTGAAATCTGTTGTTAAATTTGCGCTGGCAACAGGACTTAGGCGGTCTAACATCATCAATATGGAATGGCAACAGATTGACATGCAACGTCGTGTTGCCTGGGTGAACCCTGAAGACAGC
AAGTCAAACCGCGCTATTGGCGTAGCGCTAAATGACACTGCCTGTAAGGTATTGCGTGACCAGATTGGTAAGCATCATAAATGGGTGTTCGTGCATACGAAAGAAGGCATCCGGCCCGATGG
CTCAAAGACTCCGACTATCAGAAAGATGCGCGTCGATGACCAGCGAGCATGGAATGCAGCTTGCCGCCGGGCTGGAATTGAGGATTTCCGTTTCCACGATCTGAGGCACACGTGGGCCAG
TTGGCTAATTCAGTCCGGAGTGCCGCTTTCTGTTCTGCAGGAAATGGGAGGATGGGAGAGCATCGAGATGGTGCGCCGATATGCTCACCTTGCGCCGAATCATTTAACGGAACACGCGAAG
CAAATTGACTCGATTTTCAGTGATGATGTCCCAAATATGTCCCATATGGAAAATAAGGAGGGAATTAAAGAGGCGTAA

Prophage 7.1

>EHGDEF_26235 Phage late control D family protein
ATGACAGCCAGCGGTAAACCCGTCCCGCAAATGACCCTGACCCGCAGCGATGGCGACCGTCATCAGTTTGCCATTGCCGACCGTGGAGCTTATACCGGAGTAACGGCAAAATGGTTGCACA
CCAAAGACCCGAAGCCGCAAAAGCAAAAAGTGACGCTGAAACGTAAGCCAAAAGAGAAGCACCTGCGCGCACTGGAGCACCCGAAAGTAAAGCCGGTCAGCAAAAAGACGAAGTCCA
GAAAAGAACCGGAAGCGCGCGAGGGTGAGTATATGGCCGGTGAGGCCGATAACGTGCTGGCGCTGACGACGGTCTACGCTTCTAAGGCGCAGGCGATGCGCGCCGCTCAGGCTAAGTG
GGATAAGCTGCAGCGAGGCGTTGCGGAGTTTTCAATTACGCTGGCGCTTGGCAGGGCTGATTTATTCCCTGAGACACCTGTGCGTGTGTCGGGCTTTAAGCGCGTCATAGACGAGCAGAC
ATGGTTAATCAGTAAGGTGACTCACAGCCTGAATAATAGCGGCTTCACGACGGGCTTAGAGCTTGAGGTTAAGCTCTCTGATGTAGAGTATAAAGCGGAAGATGATGATGGGTGA
>EHGDEF_26240 Phage late control D family protein
ATGATTACGGGTATGACTATTGATGCCGGTACCAGCCTTGCACCGGCGTTTATGCTGACACTGAACAGCCAGGACATTACCAGCAATTTTAGTGACCGGCTGATTTCTCTTACCATGACCGAC
AACCGGGGTTTTGAGGCTGACCAGCTCGACATTGAGCTAGACGACACCGACGGCAAAGTCGAGTTACCCCTGCGCGGGGCGGTGCTGACGCTGTGGCTTGGGTGGCAGGGTTCCGCGC
TTCTGAATAAGGGCGATTTCACGGTCGATGAGATTGAGCACCGGGGTGCGCCTGATACCCTGACCATCCGGGCGCGTAGTGCAGACTTTCGCGGAACGCTCAATTCACGGCGTGAAGAATC
ATGGCACGACACCACCCTCGGTGAGTTAGTCAGTACCATCGCAAAGCGCAATAAACTGACGGCCAGCGTCGCGGATTCACTGAAAAAAGTCCCGGTCCGCATATCGACCAGTCACAGGAGT
CTGACGCCGTATTTCTGA
>EHGDEF_26245 Phage tail protein
ATGATGCTTGCGCTGGGAATGTTTGTTTTTGAACTCCGTACTCTGCCTTATCAGTCAATGCAGCATTCGAAAGATTACCGCTGGGCGTCTAATGACCGGGTCGGTAAACCGCCTGCATATCAG
TTTCTCGGCGAGGGGGAAACCTCAATACAGCTTGCCGGTACACTTTACCCTGCCATTACCGGCGGTCATATATCCCTGCTGGCTGTGGAACTGATGGCCGATGAGGGCAGGGCGTGGCCGC
TGATTGAGGGGACTGGCAAAATCCTCGGGATGTATATCATCGATAAGGTGTCGACCACGCACGCCGAGTTTTTCAGCGATGGCGCGGCCAGAAAGATTGATTTCACCCTTTCGCTGAAACG
GGTCGACGAATCACTGACGGCAATGTTTGGCGACCTGAATAAACAGGCGAGCGAGCTTCTCGGTTCTGCCGGTAATCTCACTGATAAGCTGCAGAGTGCGCTCGGAGGGCTGACAGCATG
A
>EHGDEF_26250 hypothetical protein
GTGCAGGACCTTTTTGACCAGCAAGAGAGTTCGCTGCGTAAGCTCACCCAAACCGCCACACAATACGTGTTAAAGCTCGATGGCTGGATCCAGAAAAACAAAGGTCTGGCGACAACTATC
GGCATTATTGCCGGTGGCGCACTTGCTCTGATTGGTATCATCGGCGGCATTGGTCTCGTTGCGTGGCCGGTTGTCATGGGGATTAACGCCATCATTGCCGCCGCTGGCGTTATGGGTACGGT
CTTTACTGTGGCCGGTAGCGCCATTGTGACAGCTCTCGGTGCGATTACCTGGCCGATTCTGGCTGTGGGGGCGGCGATTGTGGCTGGTGCGTTACTCATCCGTAAATATTGGGAGCCCATCA
GCGCATTTTTCTCGGGGGTGATTGAGGGCATCATGAGTGCCTTTGCACCGGTCGGGGAAATGTTCGCTCCATTAGCTCCAATCTTTGACGGACTCGGTGAGAAGCTGCGCGGCGTCTGGCA

GTGGTTTAAAGACCTGATTGCACCAGTCAAGGCCACGCAGGAGACGCTCGATAGCTGCAAAAATGTCGGCGTCATATTTGGTCAGGCACTGGCCTCTGCCTTGATGGCTCCGCTCAATGTTT
TTAACAAGCTGCGCAGCGGTGTCGACTGGCTTCTCGAAAAACTCGGCATCATCAACAAAGAATCGGACAGCCTCGACCAGACTGCCGCCAAAACCAATGCCGCCAAGCAGGGTAATTCCT
GCATCCCGGCAACCAGCACATATGGCGGCTATCAGGCTTATCAGCCAGTTACCGCACCGGCGGGGCGCTCTTACATTGACCAGAGCAAAAGCGAATACAACATCACTCTGCCGGGTGGTGT
TGCGCCGGGGCATCAGCTTGACCGCCAGTTACGCGACACGCTCGAACAGATTGAACGTGATAAACGTGCGCGCCAGCGTGCCAGTATGACCCACGATTTCTGA
>EHGDEF_26255 Phage related tail protein
ATGAGTAATAACCTGAGGCTTGAGGTATTGCTGAAAGCGGTCGACCAGGCGACCCGACCGCTTAAATCTATCCAGACCGCGAGTAAAATCCTGTCGGGTGATATTCGCAACACGCAAAAGG
GTCTGCGCGACCTGAACGGTCAGGCGTCGAAAATCGACGGCTTTCGTAAGGCAAGCGCGCAACTGGCCGTAACTGGTCAGGCGCTTGACAAGGCGAAGCGTGAAGCCGGTGAGCTGGC
TGTGCAGTTTAAAAACACCACCAGTCCGACCCGCGCGCAGGCGCAGGCGCTCGACGCGGCAAAGCGTGCCGCCTCTGAGCTGCAGACGAAATATAACAGCCTGAGAACATCGGTACAGC
GCCAGCGCTCCGAGCTGATGCAGGCTGGTATTAATACCCGTACCCTGTCTGCCGATGAGCGTCGACTCAAAACTTCCATCAGCGAAACGACGGCGCAGCTTAACCGCCAGCGTGATGCACT
GGCGCGCGTCAGTGCGCAGCAGGCGAAATTAAGCCGGGTGAAAGAACGATATAAATCAGGTAAAGAGCTTGCCGGTAACATGGCCGCAGCAGGCGCTGCCGGGGTAGGTATTGCGACAG
CGGGAACGATGGCCGGGGTTAAATTGCTGATGCCCGGTTATGACTTTGCGCAGAAAAATTCCGAGCTGCAGGCTGTGCTCGGGGTCGAAAAGCAGTCGCCAGAAATGCAGGCGCTACGC
AAACAGGCGCGCCAGCTCGGCGATAATACTGCAGCCTCTGCAGATGATGCAGCGAGCGCGCAAATCATCATTGCGAAAAGCGGCGGTGACGCTGCTGCCATTCAGGCAGCGACGCCAGTC
ACGCTGAATATGGCGCTGTCAAACCGGCGCTCAATGGAGGAAAACGCTGCGCTGCTGACCGGGATGAAATCAGCGTTTCAACTTTCAAACGACAAGATTGCTCACATTGGCGACGTTCTCT
CGATGACGATGAACAAAACCGCCGCCGATTTTGACGGACTGAGCGACGCGCTGACCTATGCCGCGCCGGTGGCGAAAAATGCCGGGGTGAGCATCGAGCAAACCGCCGCAATGGTCGGT
GCGCTGCACGACGCCAAAATCACCGGGTCAATGGCGGGCACGGGTAGCCGCGCCATTCTCAGCCGCCTGCAGGCTCCCACCGGAAAAGCGTTTGAGGCCATTAAGGAACTCGGCGTCAA
AACGTCAGACAGCAAGGGGAACACGCGCCCGATATTCTCCATCCTGAAAGAAATGCAGCGCAGCTTTGAGAAAAACAACCTCGGGACAAGCCAGCGCGGCGAGTACATGAAAACCATTT
TCGGCGAGGAGGCCAGCTCGGCGGCGGCGGTACTGATGGAGGCAGCCTCAAGCGGCAAACTTGACCGGCTCACTGCCGCGTTTAAAGCCTCGGACGGTAAAACCGAGGAACTGGTTAA
GGTTATGCAGGATAACCTCGGCGGTGACTTTAAAGAGTTCCAGTCGGCTTATGAGGCTGTAGGTGCAGGACCTTTTTGA
>EHGDEF_26260 hypothetical protein
ATGGCAAAAGCACCACGTAAAACCGCTGAATTTATTGATACGGCTGGCAATGAAATTGACACCGTGAATCCGAATGTCGTGACCCTGGACAAACCAATTAAGCGCGCCGGTCAGACGATTG
ATAAAGTCACCCTGATTGAGCCGAACGCCGATGCCTGCGCGGCGTCAGTCTGGCAGCGGTGGCGCAGTCCGAAGTCGACGCCCTGA
>EHGDEF_26265 Bacteriocin serracin-P subunit
ATGGCACTACCCCGTAAACTCAAATATCTGAATATGTTCAATGACGGCCTCAGCTACATGGGTGTTGTTGAATCCGTGACGCTGCCGAAACTGACCCGCAAGCTCGAAAACTATCGCGGCGG
CGGTATGAATGGCGCGGCAGCGATTGACCTCGGCCTCGACGATGATGCGCTCACCGTCGAATGGTCTGTCGGCGGCCTGCCTGATGTGGCTCTGTGGGCGCAGTACGCCGCCCCGGGCGC
TGATGCTGTGCCGCTGCGTTTTGCTGGTTCTTACCAGCGCGACGACACCGGCGAAATCGTGGCGGTCGAGGTGGTCATGCGTGGCCGTCATAAAGAAATCGACGGCGGCGAGAATAAGCA
GGGTGAAAACACCTCGACCAAACTGTCGACTGTTTGCACCTACTACCGCCTCACGATTGATGGCAGCGACGTCATCGAAATCGACACCGTCAACATGGTCGAGAAGGTGAACGGCGTCGAC
CGTCTGGAGCAGCACCGCCGCGCAATCGGGCTGTAA
>EHGDEF_26270 Phage tail sheath protein
ATGAGTGATTTTCACCACGGCACGCAGGTCATCGAAATTAATGACGGTACGCGTGTTATTTCCACAGTAGCGACTGCGGTCGTCGGCATGGTTTGTACAGCCAGCGATGCAGATGCCACGCT
ATTTCCCCTCAATGAACCGGTACTGATTACCAATGTGCAAAGCGCCATTGCGAAAGCCGGTAAAAAAGGCACGCTGGCTGCATCACTGCAGGCCATCGCAGACCAGTCAAAACCCGTCACT
GTTGTTGTACGTGTTGAGGATGGAACCGGCGATGACGAGGAAGCTGCGCTCGCACAGACTGTTTCCAACATTATCGGAGGTACGGATGAGAACGGTAAATACACCGGTATCAAGGCTCTCC
TGACCGCTCAGGCCGTCACCGGCGTCAAGCCGCGTATTCTTGGGGTGCCGGGGCTGGATACTAAAGAGGTCGCGGTCGCGCTTGCGTCGGCTGCCATTAAGTTACGTGCATTTGCTTACGT
CAGCGCGTGGGGATGTAAGACTATTTCCGAAGCGATGGAATATCGTAAAAATTTCAGCCAGCGCGAGCTGATGGTTATCTGGCCTGATTTCCTCGCATGGGACACCGTCAAAAATACCACCG
CAACGGCTTACGCCACTGCGCGTGCACTCGGCCTGCGTGCTTACATCGACCAGACTGTCGGCTGGCACAAAACCCTGTCTAACGTTGGTGTACAGGGCGTTACCGGCATCAGCGCCTCAGT
GTTCTGGGATTTACAGGCATCCGGCACCGATGCTGACCTGCTCAACGAGGCCGGGGTTACAACGCTGGTACGCAAGGACGGTTTCCGCTTCTGGGGTAACCGCACCTGCTCGGATGACCC
GCTTTTTCTGTTTGAGAACTACACCCGCACCGCGCAGGTACTGGCCGACACGATGGCGGAGGCGCACATGTGGGCGGTCGACAAGCCCATTACCGCCACGCTCATTCGTGACATTGTTGAC
GGCATTAACGCCAAATTCCGCGAGCTGAAATCAAACGGCTACATCGTGGAGGGTAAATGCTGGTTCGATGAGGAATCGAACGACAAGGAAACCCTCAAGGCCGGGAAACTGTATATCGAC
TACGACTATACACCGGTTCCGCCACTGGAAAGCCTGACCCTGCGCCAGCGTATTACCGATAAATATCTGGTGAATCTGGCCGAATCGGTCAACAGCTAA
>EHGDEF_26275 tail fiber assembly protein
ATGACTTTTAAAATGAGCAGCAAAGCGCAGACAATTAAAATTTTCAATCTGCGTTCAGATACTAACGAATTTATTGGTGCGGGCGATGCGTATATCCCGCCGCACACTGGATTACCGGCTAATT
GTACTGATGTCGCCCCTCCTGAAATTCCGACCAGCCATATTGCGATATTTGATGCTGAAACACAGACGTGGACTCTGCATGAAGACCACAGAGGTGAAACGGTTTATGACACAACAACCGGT
AATCAGGTTTATATCTCCGAACCCGGGCCGTTGCCTGAAAATGTCACATCAGTTTCGCCAGAGAGTCAATACCAGAAATGGGATGGTAAGGCATGGGTGAAAGATGAAGTTGCGGAAGCTG
CAGCACGACTTATTGAAGCGGAAGGGACAAAAAACAACCTTCTGCAAATGGCTTTGACGAAAATAGCGCCATTACAGGATGCAGTTGAGCTTGATATCGCTACGAGTGAAGAGAGAGCGC
AGCTCAACGAGTGGAAAAAATACAGGGTACTGGTAAACAGGGTGGATACATCTGCGCCTGACTGGCCGGATATACCACGCTAA
>EHGDEF_26280 Collar domain-containing protein
ATGAGCACGAAATTTAAAACCGTTATCACCACTGCCGGAGCCGCTAAGCTTGCTGCGGCCACCATGCCGGGCGGTAAGAAAATAAATCTTAACGTTATGGCTGTTGGTGACGGCGGCGGAA
AGCTGCCGGATCCTGATGCCGGTCAGACGCAGCTTGTTAATGAGGTCTGGCGTCACACTCTGAATAAAATCAGCCAGGACAACCGGTACAGTAATTACATTGTGGCCGAGCTGGTTATTCCG
CCGGAGGTGGGCGGCTTCTGGATGCGTGAGCTTGGCCTTTACGACGATGAAGGGACGCTGATTGCTGTTGCCAATATGGCCGAAAGCTACAAGCCAGAACTGGCTGAGGGCTCAGGGCG
TGCGCAGACATGCCGCATGGTCATTATTGTCAGCAGTGTCGAGTCTGTGACGCTGTCCATTGACTCAACGATGGTGATGGCGACGCAGGATTATGTCGACGACAGGCTCGCCGAACATGAA
AAATCCCGTCGTCATCCTGATGCCACTCTTAAAGAAAAAGGGTTTACTCAGCTCAGTAACGCGACAGACAGCGAGTCTGAAACGCTCGCAGCGACGCCGAAAGCTGTCAAGGCAGCATATG
ACCTTGCTGACGCGAAATATACGGCTCAGGACGCCACCACAACGCGTAAAGGGATTGTACAACTAAGTAGTGTAACCGACAGTAATGATGAAAATCAGGCTTCCACGCCAAAAGCGGTCAA
AATTGCGATGGACAACGCCAACAAGCGCCTTGCCAAAGAACGCAACCTCGCTGACCTGACAAACATCCAGAAGGCCCGTCAGTCCCTCCAGCTTGGCAACAGCGCTACACTCAATGTCGG
CACCACACCAGACACTGTAGCCGCAGGTGACGACGCCCGCATTATCACCACCAAAAAAGCCATTGACGACACCCAGAACGGTCTTGGTGCTCAGCCCGTTATGTGGGTAAGCTCCGCCGAT
GATTTGAGCAGCCTGCCGTCTGGCGCACGGCGGTTTGCCAGCAATAAAGCTCCGGCAACAATATTGCCGGTAAACGATTATGTTTTCCTGGAAGTGATTGCCAAACGCGATTGCGTAGACG
GCTGCGCCGTTCTGATAACAGACTCAATTGGTAACACCTGGATTGGCGCGCGCTGGGACGCAACCAATGGTTCCGGTTTTACCTGGCGTCCCCTGATGTCGTGTCCGCCCGGTGTTCCCCTT
CCGTGGCCGTCTGACACTATCCCTGCCGGTTATGCCCTGATGCAGGGGCAGGCATTTGATAAGAACGTTTATCCCTTACTGGCAATAGCATATCCATCCGGCACTATTCCGGACATGCGAGGC
TGGACAATCAAAGGCAAGCCTGTCAGTGGACGCGCTGTGTTGTCGCAAGAACTGGACGGCAATAAATCGCACAGTCACAGCGCCAGGGCGCAGGATACCGACTTAGGGATGAAAACTAC
CTCATCCTTTGATTACGGCACGAAGAGTTCCGATACAACAGGCGGTCATAACCATTCGGCTGGCGGACTATATGGCGGTGACTCAATCGGTGGAAAAACTCGCGTTCAGCGTGACGGAAAT
AATCAGTTAACAAGCTGGAATGGCGATCACGCACATACCACATGGATTGGCCCGCATGAACACTCCGTATACATCGGTCCACACGGACACGTCGTTATAGTGGACGCAGATGGTAATGCGGA
AACCACGGTTAGAAACATTGCATTTAACTACATAGTGAGGCTGGCATAA
>EHGDEF_26285 Phage tail protein I
ATGAATGACTCACGATTGCTGCCGGTAGGCTCATCGCCACTGGAGGTTGCCGCCGCAAGGGCATGTGCTGAGATTGAAAGAACGCCGGTCAACATCCGCGCGTTGTGGAACCCTGACACC
TGTCCGGAAAATTTGCTGCCGTGGCTGGCGTGGGCATTTTCTGTCGACCGGTGGGATGAGAACTGGCCGGAGGGAACAAAACGTGCCGTTATCCGTGATGCATATTTCATTCACTGCCACA
AGGGGACTATCGGCGCAATCCGTCGGGTAGTGGAGCCACTCGGCTATGTCATCAATGTAACGGAATGGTGGGAAAGCGGCGACCCGCCAGGCACATTCCGGCTTGATATCGGGGTGCTTG
AAAGTGGCATTACCGAGGAAATGTATTTCGAAATGGAGCGACTGATTGCGGATGCAAAGCCAGCCAGTCGTCATCTGACTGGCCTGAATATTGTCCAGGACATTCCCGGTTATTTGTATACCG
GCGGCGTGTCCTGCGACGGCGATATTATTACGGTTTACCCGGGATAA
>EHGDEF_26290 Baseplate-J domain-containing protein
ATGGCAACTGTTGACCTGAGTCTGCTACCTGTTCCTGATGTGGTCGAGGAACTGGACTATGAAACTATCCTTGCGGAGCGCATTGCAACGCTGATTTCGCTCTATCCGGAAAACCAGCAGGA
AGCCGTCGCCCGGACGCTCGCACTTGAGTCTGAGCCAATTGTTAAATTGCTGCAGGAAAACGCCTACCGCGAGGTTATCTGGCGTCAGCGTGTCAATGAAGCTGCACGCGCAGTGATGCTG
GCTTATGCCATAGACAGTGACCTCGATAATATCGGGGCGAATTTCAGTGTTGAGCGCCTTGTCGTCACGCCTGCTGATGACACCACCATTCCACCCACTCCGGCAGAAATGGAACTCGACGC

AGATTATCGTCTGCGTATACAGCAGGCTTTTGAGGGACTGAGCGTGGCGGGGTCTGTCGGATCGTACCAGTATCACGGCCGTAGTGCTGACGGGCGCGTCGGCGATATTTCAGTTATCAGCC
CGTCGCCAGCTTGTGTGACGATTTCCGTGCTGTCTCGTGAAAACAACGGCGTCGCATCTGAGGAACTGCTTGCAATTGTGCGCAATGCCCTGAACGCAGAAGATGTCAGGCCGGTCGCTGA
CCGGGTGACGGTACAGTCAGCCGAAATTGTTAACTACCAGATTAACGCCACGCTTTATCTTTATCCCGGCCCGGAAAGTGAACCCATCAGGGCGGCGGCTGAGGCAAAGCTGAAAGCCTAT
ATCAGCGCGCAGCACCGCCTCGGGCGCGATATCCGTAAATCAGCGATTTATGCCGCCCTGCATGTTGAGGGTGTTCAGCGGGTGGAGCTGGCGGCACCGGTCGCGGATATTGTTCTCGATA
ACACACAGGCGTCCTTTTGCACTGACTACAGCCTTGTAATCGGGGGCTCTGATGAATGA
>EHGDEF_26295 GPW-gp25 domain-containing protein
ATGACGGCGCGTTATCTGGGGATGAACCGCAATACCGGCCTCGCTATCAGTGACAGTGAGCATATCAGCCAGAGCATGCGCGACATTCTGCTGACGCCGGTCGGCTCGCGGGTAATGCGTC
GTGAATATGGCTCGCTCCTGTCTGCGCTGATTGATATGCCGCAAAACCCGGCGCTCAGGCTGCAAATCATGGTGGCGTGCTATTCCGCGATCCAGAAATGGGAACCACGCATCAGGCTTACC
TCAATCAGCTTTGAGCGTGGCGACACTGGCGAAATGTATGTCGATATTACCGGGATGCGTACCGATACCGGTGCGTCAGTTTCAACCACTGTTTCACTGAGTTAA
>EHGDEF_26300 hypothetical protein
ATGCGGGGAAACATCGAACATACCGGTGGCACGTTGAAATCAAACGGCGTACAGGTCGATGACCACGGTCACGGCGGCGTGCAACGGGGCGGGAGCTGGACGGAGGGCACCAGATGA
>EHGDEF_26305 Baseplate assembly protein V
ATGAATACGTTATCCACAATACAGGAGCTCACGCGCGCGATTCGCAACCTCATCCGCTCAGGTGTGGTGACTGAGGTCGATACCGCGCAGGGGCTGTGCCGCGTACAAAGCGGCGGGATCC
AGACTGCATGGCTGAACTGGCTGACCACCCGCGCCGGTCGTTCGCGGACATGGTGGGCTCCCTCGGTCGGTGAGCAGGTGCTGCTGCTGGCAATTGGTGGCGAGCTTGATACTGCTTTCG
TGCTGCCGGGGATTTTCTCCGACGATAACCCTGCCCCGTCAGCCTCGGCGGATGCGTGGCATGTGGTGTTCCCCGACGGCGCTGTTATGGAGTATGAGCCGGAAACCGGTGCACTGACGGT
CAGCGGCATCAAGACTGCCGATGTGACGGCATCGGAGTCCATTACCGCCACCGTGCCGGTGGTACTGGTAAAAGCGGCAGAACGTATCACCCTCGACACCCCGGAGGTGGTATGCACCAA
CAAACTGACGACGGCGACGCTTGAGGTGCAGAAAGGCGGCGACCATGCGGGGAAACATCGAACATACCGGTGGCACGTTGAAATCAAACGGCGTACAGGTCGATGA
>EHGDEF_26310 hypothetical protein
ATGTTCGCCAGACTGCGCACTAACCGCTTTATGAAAGCCAAAGGCAGCGACAGTGCGGCGGTGGTGGAATTTACCGGCAGGGTACAGCGCATGGCGCGGGTGCATCAGTATGGCCTCAAA
GACCGGCCAAATCGTCACAGCCGGGATGTGCAGTACGCGGCGCGCCCGTTGCTCGGTTTCACCCGCGACGATGAGCAGATGATTGAAGACATCATTATCAGGCATCTCGGTAAATAA
>EHGDEF_26315 hypothetical protein
ATGAGTGAGTTAACCGCGCTGCAGGAGCGCCTTGCCGGTCTGATTGCCAGCCTGTCACCGGCGGCGCGTCGTCAAATGGCGGCTGACATTGCAAAAAAACTGCGCGCCAGTCAGCAGCA
GCGCATCAGGCGACAGCAGGCATAA
>EHGDEF_26320 N protein
ATGCAGAAGCACAAAAGCCTGCGCAAGGCGCTGATTAACGCCGTGCCGCAGCTCCGAAACAACCCCGATATGCTGCGCCTGTTCGCTGATAACGGGCATACGGATTCCCGACTGGAGAGC
TCGCTGTCGTTTGAAAAGGTGTACGTGCTTAACGTGGTGGTGACTGACTTTACCGGCGACCTCGATTTGATATTTGTGCCGGTACAGGCATGGCTGCGTGAGCATCAGCCGGACATTATGAC
CACCGACGACGGGCGGGAGAAAGGATTCACATGGATGATTGATATCAATAACGACGATTCGCTCGATATCAGTATCAGCCTGAGACTCACCGAGCGCACGCTCGTCAAAGAGGTCGACGGC
GCATTGCATGTCAGCTATGCCCCTGAGCCACCGCTGCCTGAGCCAGTGACACGCCCGGTCGAGCTGTACGTTAACGGCGAGCTGGTGAGTAAGTGGGATGAGTGA
>EHGDEF_26325 hypothetical protein
ATGCCGGGAAGTGATCCGAAAACTAATGGTGACCTGAGCGCTGATATCCGCCGCCTTGAGGGCGCGCTGACCGCCTGCGCGCTACAGGTCAAAACCGTCAAACACTGTCAGGATGAACTC
GATGCAGAAGCACAAAAGCCTGCGCAAGGCGCTGATTAA
>EHGDEF_26330 hypothetical protein
ATGCCGGTCATTAATACTCACCAGAATATCGCCGCCTTTCTGGACATGCTGGCGTATTCCGAAGGAACGGCGAACCATCCGCTGACGAAAAACCGTGGCTACGACGTCATTGTTACCGGCCT
TGATGGCAGGCCAGAGATTTTCACCGATTACAGCGACCACCCTTTCGCACATGGCCGACCCGCGAAAGTGTTTAATCGCGGTGGCGAGAAATCCACGGCATCGGGGCGTTACCAGCAGCT
TTATATGTTCTGGCCGCACTATAAAAAACAGCTCGCATTGCCTGATTTCAGCCCACTGTCGCAGGACAAGCTCGCGATCCAGTTAATCCGGGAGCGCGGTGCTATTGACGATATCCGGGCGG
GGCGTATTGAGCGTGCTGTTTCCCGTTGCCGGAATATCTGGGCGTCATTGCCGGGTCGCCGGTTACGGCCAGCGCGAGCACAGTCTCGAAAAGCTGGTTACCGTCTGGCGCACGGCTGGC
GGGGTGGTGGCATGAAAGTCCTGATAACGCTGTTTGTGCTGGCCGTGCTCGGTCTGATGTGGTTGCGCCATGAGAATGGCAATTTATCCCGCTCCTTTGAGACGGCAAACCGCGTTGCGAG
CGAGCAAAAGGCGACGATTGGCATGCTGAAAAATCAGCTCAGTGTCGCCGGCCAGCTCGCCCGACGTAATGAATCCGCGCAGGTGGCACTGCGCGAACAGCTCGCAAAGGCAGGCGCA
GAAGCAAACCGCCGCGAGCAGACGATAACGAGGTTACTTGATGAAAATGAAGCCTTTCGCCGCTGGTATAACGCTCCTCTGCCTGATGCTGTGCGCAGGCTGCACACCCGCGCCGCCTGCG
CCAGCGCCGGTGATTGTGGTCAGCGGATGCCCGAGGGTGAGCCTTTGCCCGATGCCGGGAAGTGA
>EHGDEF_26335 Holin
ATGACAGAAGGGGAAAAAGGCGTCCTGTCACTGTTTGTGATTGGGGCACTGATTGTGGTCGGAAAAGTGCTGGCAGGTGGTGAGCCCATCACCCCGCGCCTGTTTGTCGGACGCATGTTG
CTCGGCGGTTTTGTCTCAATGGTCGCCGGTGTTGTTCTGGTGCAGTTTCCTGATATGTCACTGCCCGCTGTGTGCGGTATTGGATCCATGCTCGGTATTGCCGGTTATCAGGTGGTGGAAATC
GCCATTCAGCGCCGCTTTAAGTCACAGAAGGGGGAAGGCGATGCCGGTCATTAA
>EHGDEF_26340 Tail X family protein
ATGCAGGGCGACACCCTCGACGTGATTTGCGCCCGGTATTACGGGCGCACTGAGGGCGTTGTTGAAACTGTGCTGCAGGCTAATCCCGGCCTGTCTGAGCTGGGCGTCATTCTGCCGCATG
GAACGGCAATTGACCTGCCCGATGTGCCGTCTTCACCCGTAACTGAAACTATCAATCTTTGGGAGTAA
>EHGDEF_26345 Capsid completion protein
ATGACGACAGTGATTCTGAACCAGCCCGACGAACCGCAGGACGTACCGGGCGTGGTGATTCCCGTACCGGAGACGGGCGATGCAGTAATTAAAAACACGTTCTTTTTCCCTGATGTGGATC
CGAAGCGTGTGCGCGAGCTGATGCGGCTTGAACAGACGGTTTCCGATGCGCGCCTGCGCCATGCCATCAGAACTGGCATGGCGGAAACCAATGCGGAGCTTTACGACTACCGGCTGCGCC
AGACTGCCGCCGGGTTTAAGCATCTGGCCGACGTGCCTGCTGAGGAAATCGATGGCGAGAATGTGCGTATTTTCCACTATCTGAGAGCCGTAACGGCGATGGCAACCGCCACCCTGTATGA
GCGCTATCGCGGTGTTGAAGCCACCGGCAAGGGTGACAAAAAAGCCGACAACGTCGAAACCACCATTGATGACCTGTGGCGGGATATGCGCTGGTCGGTCGCGCGTCTGCAGGACAAAC
CGCGCTGCATCGTGGGCCAGCTCTGA
>EHGDEF_26350 Small terminase subunit
ATGCTGGTTAAGCTGGCCGATGACCGCCGCACGTTAAAAAACATCCGTTCAAACGAACGTAAAGCCGAGAAAAAGCGCGAGCTGCTGCCGTTCTATGCGCCGTGGGTCGCCGGTGTGCTG
GCTGATGGTCGCGGTGCGCAGGATGACATTGTCATGACCGTCATGCTGTGGCGTCTTGATGCCGGTGATATCGCTGGCGCGCTGGAAATTGCGCCCTATGCGCTGAAATACGGCCTCACCAC
TGACCATCGCCGCACGACGCCTTACATGCTGGTTGAGGAGGTGGCGCTTGCCGCACTGCGCCTGCGCGATGCCGGTGAGCCTGTCGACCTCGCATTACTGCTGACCACCCTCAGCCTGACC
GACGGCGCTGACGTTCCCGATATGGTGCGCGCCCGTCTGCATAAGGTGACCGGCCTGACCCTGCGCGATATCGGTCAGAATGCTGAGGCACTGGCTCAGTTTCAACGTGCGATGCAGCTCG
ACCGCAATGCCGGTGTGCGCAAAGAGATTGAGCGGCTGGAACGCGCACTGAAGCCAAAAGCGGAGGCATGCCACCCCGTAAAACGACTAAACCGCGCACGCGCAAACCTGTCGCCAGA
CCGGCAGCAAAGCGCGGGCGTCCACCAAAGGCGGTAA
>EHGDEF_26355 Phage major capsid protein, P2 family
ATGCGTCAGGAAACCCGTTTTAAGTTCAATGCCTATCTGACCCAGCTCGCCAAACTGAACGGCATCAGCGTTGATGACGTTAGCAAAAAATTCACCGTCGAGCCGTCCGTCACGCAAACGCT
GATGAACACCGTGCAGGCGTCATCCGCATTTCTGCAGATGATTAACATTTTTCCGGTTGATGAGCTGAAAGGTGAAAAAATCGGTGTGGGTGTGGATGGCACTATTGCCAGCACGACCGAC
ACCTCAGGTGACGATGAGCGTGAAACTGCTGACTTTGTCGCGCTTGAGTCCAATTTGTACGAGTGCAACCAGGTCAACTTTGACTTCCACCTGCGCTACAAGACGCTCGACCTGTGGGCGC
GTTTTCAGGACTTCCAGCGCCGCATCCGTGACGCGATTATCCAGCGTCAGGCACTGGATTTCATCATGGCCGGATTTAACGGTACTCATCGTGCGCCGACCTCGAATCGCAAAACTAATCCAA
TGTTGCAGGATGTAGCGGTCGGCTGGCTGCAGAAGTACCGTAACGAAGCACCGGCGCGCGTGATGAGTAAAATCACCGACGATGAGGGTCAAGTCGTTTCCGATGTGATTCGTGTCGGTA
AAAACGGCGACTATGAGAATCTTGACGCGTTGGTAATGGATGCGACAGAATGCCTGATTGATGAGGTTTATCAGGACTCAACCAAACTGGTTGCCATCGTTGGCCGTAAGCTGCTGGCCGA
TAAATATTTCCCGCTGGTTAACAAGCAGCAGGAAAACAGCGAGTCGCTCGCTGCGGATACCATCATCAGCCAGAAACGCATCGGCAACCTGCCTGCCGTGCGCGTGCCGTACTTCCCTGCG
AATGCTGTACTGGTGACCACGATGGAAAATCTCTCAATCTACTTCATGGATGATGGTCATCGGCGTTCAGTCATCGAAAACCCCAAGCGTGACCGCGTGGAAAACTACGAGTCGATGAATATC

GACTATGTGGTCGAGGCGTATGCCGCCGGGTCGCCTGCTGGAAAACATCACCCTGGGCGATTTCACCGCACCTGCAGCACCGGAAAGCGGAGTCTAAACCATGACGAGCCCCGCACAGCG
TCACATGATGCGGGTCTCGGCCTCTCAAGCCGCGCAGCGGGAGCAAGCCCCACTGCGCCATGCAACCGCCTATGA
>EHGDEF_26360 GPO family capsid scaffolding protein
ATGGCAAAAAAAGTCTCAAAATTCTTTCGCATCGGCGTCGAGGGTGATACCTGCGACGGGCGCATTATCAGCGCCAGCGATATTCAGGAAATGGCCGAAACCTATGACCCGCGCGTCTACG
GTTGCCGTATCAACCTTGAACACATTCGCGGCCTTTTGCCCGACGGCATGTTTAAACGTTATGGCGATGTGGTTGAGCTGAAAGCCGAAAAGATTGACGACGATTCTGCGCTGAATGGCAA
ATGGGCGTTGTTCGCCAGAATCACCCCAACCGATGACCTTATCGCGATGAATAAAGCCGCGCAGAAGGTCTATACCTCTATGGAAATTCAGCCGAATTTTGGTAACACCGGCAAATGCTATCT
TGTCGGCCTTGCGGTCACTGATGACCCTGCGAGCCTCGGCACTGAATACCTCGAATTCTGCCGTAAGGCGAAGCACAACCCGCTGCAGCGCTTTAAGGCCAGTCCTGAAAATGTCTTTTCA
GTCGCCACGCTGGCCGAACTGGAATTTGAAGATGTTCCCGACACAGTGCTTAACAGCCTGGCCGATAAGGTGAAAGCCATTTTCAGCCGTAAGCAGGTCAGCGACGATGCGCGCATGAGT
GATGTGCATGAGGCGGTGACCGCCGTCAGCGAACATGTGCAGACCAGTCTCACTGCGCAGGATAAGCGTCTTTCCGATATGGAAACCGCGCTAGCCACCTTTAAACAGGAACTGACCGGC
AAGGTTGAAGAAACCAGCCAGGCATTTTCCGCCCTGAAAACCACCCTCGACAAAACCGAAAGTTTCAGCCAGCCGCGACGCACGAAAGCCAGCGGCGGCGGTGGCGACGAGCTGCTGA
CCGACTGCTGA
>EHGDEF_26365 Terminase, ATPase subunit
ATGACCATTTCCACCGATACAACCTTATTGCATGACCCGCGACGACAGGCATCGCTGCTTTACTGGCAGGGCTTTTCCGTGCCACAGATTGCCGAAATGCTGCAGGTCAAGCGCCCGACCGT
GCAAAGCTGGAAACAGCGCGACGGCTGGGACGGCATCGCACCGATTTCCCGTGTCGAAAGCAGCCTTGAGGCGCGCCTGATTCAGCTCATCGCCAAGCCGCAAAAGTCAGGCGGCGACT
TCAAAGAGATTGACCTGCTCGGGCGGCAGATTGAGCGACTGGCGCGCGTCAACCGCTACAGCCAGACCGGCAACGAGGCCGACCTTAACCCCAACGTTGCCAACCGTAACAAGGGGGA
GCGTAAGAGGCCGAAAAAGAACTTTTTCAGCGATGAGGCTGTCGCAAAGCTGGAAGAAATTTTCTTCGACCAGTCTTTCGAATACCAGTTGCAGTACCGGGCAGGACTGGCGCACCGTAT
TCGCGATATTCTCAAATCCCGCCAGATTGGCGCGACGTTCTACTTTTCCCGCGAGGCACTGCTGCGCGCGCTCAAGACCGGCCATAACCAGATTTTTCTGTCGGCCAGTAAAACGCAGGCTT
ACGTGTTCCGGGAATACATCATCCAGTTTGCGCGACTGGTTGACGTCGACCTGACCGGCGACCCGATTGTCATCGGCAACAACGGCGCAAAGCTGATTTTTCTCGGCACCAATTCCAACACC
GCACAAAGCCATAACGGCGACCTGTATGTCGATGAAATATTCTGGATCCCGAATTTTCAGAAGCTGCGCAAAGTCGCATCGGGCATGGCCTCGCAAAAGCACCTGCGATCAACTTACTTTTC
GACACCTTCCACGCTGGCTCACGGCGCTTACCCCTTCTGGTCTGGCGAGCTGTTCAACAAGGGGCGCGCCAGTGCCGCTGACCGCATCGAAATCGACATCAGTCACAGCGCGCTCGCCGG
TGGACTTCTTTGCGCAGACGGACAGTGGCGGCAGATTGTCACTATTGAGGACGCCCTTGCCGGTGGCTGCACCCTGTTCGACCTCGACCAGCTCAGACGCGAAAACAGTGATGAGGACTT
TAAGAACCTGTTTATGTGCGAGTTTGTCGACGATAAGGCATCGGTATTCCCGTTCGAGGAGCTGCAGCGCTGCATGGTCGACGTGATGGAAACATGGGAGGACTTCGCCCCGTTCGCCGAC
CATCCATTCGGCTCGCGACCGGTCTGGATTGGCTACGACCCGTCCCACACCGGCGACAGTGCCGGATGTGTCGTACTCGCGCCGCCGGTGGTTTCGGGTGGCAAGTTTCGCATGCTGGAGC
GTCACCAGTGGAAAGGCATGGACTTTGCCGCGCAGGCAGAAGGCATCCGCAGGCTCACTGAGAAATACAACGTCGAATACATCGGCATTGACGCAACAGGCCTCGGTCTCGGCGTATTCC
AGTTGGTGCGCTCATTCTACCCGGCGGCACGCGGTATCCGTTACACGCCAGAAATGAAAACCGCAATGGTGCTCAAGGCGAAAGACACGATTCGCCGTGGCTGTCTGGAGTACGACGCCG
GAGCAACTGATGTCACACAGTCGTTTATGTCCATCCGCAAAACCATGACCAGCAGCGGGCGCAGCGCCACCTATGAGGCCAGCCGCACCGAGGAAGCCAGTCACGCTGATATCGCATGGG
CCACCATGCACGCCCTGTTAAACGAACCGCTTTCTGCCGGTAGCGGCATGCAGCCTAAATCTATTCTGGAGTTCAACTAA
>EHGDEF_26370 Phage portal protein
ATGGAAGCGTTCACCTTCGGTGAGCCGGTGCCGGTACTCGATAAGCGCGACATTCTGGATTACGTCGAGTGCATCAGTAACGGCAAATGGTACGAGCCGCCGGTCAGCTTCTCCGGGCTGG
CAAAAAGCCTGCGCTCTGCAGTGCATCACAGCTCACCGATTTACGTTAAACGCAATGTGCTCGCAAGTACCTACATTCCGCATCCACTGCTGTCCCGTCAGGATTTCAGCCGCTTTGCACTCG
ACTATCTGGTATTCGGTAACGCCTTTCTTGAGCAGCGCCACAGCGTCACCGGCCAGTTAATCAAACTGCTGACTTCACCGGCAAAATATACCCGGCGCGGGGTCGATGACTCGGTTTTCTGG
TTTGTGGAAAACTTCACTCAACCGCATGAGTTCGCACCCGATACCGTGTTCCACCTGCTGGAGCCTGATATTAATCAGGAGATTTACGGCCTGCCTGAATATCTCAGCGCGCTTAATTCCGCAT
GGCTGAATGAATCCGCGACGCTGTTCCGCCGCAAGTATTACCAGAACGGCGCGCACGCAGGTTACATCATGTATGTGACTGACCCGGCGCAAAGCGCGACTGACGTCGAATCGCTGCGCG
ATGCAATGCGTAACTCTAAAGGGCTCGGCAACTTTAAAAACCTGTTTTTCTACTCACCGAACGGGAAACCGGACGGCATAAAAATCGTGCCATTGAGCGAAGTCGCCACAAAGGATGACTT
TTTCAACATCAAGAAAGCCAGCGCCGCTGACCTGATGGATGCGCACCGCGTACCTTTCCAGCTCATGGGCGGCAAGCCTGAGAATATCGGCTCAATGGGTGATGTTGAGAAGGTGGCAAA
GGTCTTTGTGCGTAACGAGTTATCGCCCTTACAGGACAGATTCAGGGAGGTAAACGACTGGCTCGGCATGGAGGTCATCAGGTTCAAAGAGTACACCCTCGATAACCCGGAATAA

Prophage 7.2

>PELPCK_26010 Ecotin
ATGTTCCACTTCAAACCGACAATTGCAGTAGCGGCTATCCTGCTCTCAGTCAGCACATACGCCAATGCTAACGACACAAAAACACTTCAAAAGCAGTTAACACCTTGGCAGCCAATGGAAAT
CACAACCCGAGAAAACTCACTAACGGTGGTGCTTACCGGCAAAGAAATCACGTCAGAAGCCTATAGCCAATTAATTATGTCAGGTGTATGCACACCGATATGGACGCATGATGCACCAGCGT
CATACCTTAAAAATACAAAAGAAATAAGTGTGCTTAACGAATACAAATCTTTTGGCTATACATTTGAAGACCCATTAGCAACATGTAATGAAATTGCACCACTGGCAGACAAAGCCGCACAGA
CCATGCTGCTATCGAAAACCCATACCTACACAGGAAAATAG
>PELPCK_26015 DNA-binding transcriptional regulator
ATGTTTCATTGCCCGTTATGCCAGCATGCAGCTCATGCACGTACAAGCCGTTACATGACCGATACGACAAAGGAGCGTTATCATCAGTGTCAGAACGTGAATTGTAGCGCCACGTTCATCACA
TTTGAGTCGGTGCAGCGTTACATTGTTAAGCCGGGTGAGGTTAATGCCGTCAGGCCTCATCCGTTGCCATCAGGTCAACAAACTATGTGGATGTAA
>PELPCK_26020 Phage late control D family protein
ATGACAGCCAGCGGTAAACCCGTCCCGCAAATGACCCTGACCCGCAGCGATGGCGACCGTCATCAGTTTGCCATTGCCGACCGTGGAGCTTATACCGGAGTAACGGCAAAATGGTTGCACA
CCAAAGACCCGAAGCCGCAAAAGCAAAAAGTGACGCTGAAACGTAAGCCAAAAGAGAAGCACCTGCGCGCACTGGAGCACCCGAAAGTAAAGCCGGTCAGCAAAAAGACGAAGTCCA
GAAAAGAACCGGAAGCGCGCGAGGGTGAGTATATGGCCGGTGAGGCCGATAACGTGCTGGCGCTGACGACGGTCTACGCTTCTAAGGCGCAGGCGATGCGCGCCGCTCAGGCTAAGTG
GGATAAGCTGCAGCGAGGCGTTGCGGAGTTTTCAATTACGCTGGCGCTTGGCAGGGCTGATTTATTCCCTGAGACACCTGTGCGTGTGTCGGGCTTTAAGCGCGTCATAGACGAGCAGAC
ATGGTTAATCAGTAAGGTGACTCACAGCCTGAATAATAGCGGCTTCACGACGGGCTTAGAGCTTGAGGTTAAGCTCTCTGATGTAGAGTATAAAGCGGAAGATGATGATGGGTGA
>PELPCK_26025 Phage late control D family protein
ATGATTACGGGTATGACTATTGATGCCGGTACCAGCCTTGCACCGGCGTTTATGCTGACACTGAACAGCCAGGACATTACCAGCAATTTTAGTGACCGGCTGATTTCTCTTACCATGACCGAC
AACCGGGGTTTTGAGGCTGACCAGCTCGACATTGAGCTAGACGACACCGACGGCAAAGTCGAGTTACCCCTGCGCGGGGCGGTGCTGACGCTGTGGCTTGGGTGGCAGGGTTCCGCGC
TTCTGAATAAGGGCGATTTCACGGTCGATGAGATTGAGCACCGGGGTGCGCCTGATACCCTGACCATCCGGGCGCGTAGTGCAGACTTTCGCGGAACGCTCAATTCACGGCGTGAAGAATC
ATGGCACGACACCACCCTCGGTGAGTTAGTCAGTACCATCGCAAAGCGCAATAAACTGACGGCCAGCGTCGCGGATTCACTGAAAAAAGTCCCGGTCTGCATATCGACCAGTCACAGGAGT
CTGACGCCGTATTTCTGA
>PELPCK_26030 Phage tail protein
ATGATGCTTGCGCTGGGAATGTTTGTTTTTGAACTCCGTACTCTGCCTTATCAGTCAATGCAGCATTCGAAAGATTACCGCTGGGCGTCTAATGACCGGGTCGGTAAACCGCCTGCATATCAG
TTTCTCGGCGAGGGGGAAACCTCAATACAGCTTGCCGGTACACTTTACCCTGCCATTACCGGCGGTCATATATCCCTGCTGGCTGTGGAACTGATGGCCGATGAGGGCAGGGCGTGGCCGC
TGATTGAGGGGACTGGCAAAATCCTCGGGATGTATATCATCGATAAGGTGTCGACCACGCACGCCGAGTTTTTCAGCGATGGCGCGGCCAGAAAGATTGATTTCACCCTTTCGCTGAAACG
GGTCGACGAATCACTGACGGCAATGTTTGGCGACCTGAATAAACAGGCGAGCGAGCTTCTCGGTTCTGCCGGTAATCTCACTGATAAGCTGCAGAGTGCGCTCGGAGGGCTGACAGCATG
A
>PELPCK_26035 putative tape measure protein

ATGAGTAATAACCTGAGGCTTGAGGTATTGCTGAAAGCGGTCGACCAGGCGACCCGACCGCTTAAATCCATCCAGACCGCGAGTAAAACCCTGTCGGGTGATATTCGCAACACACAAAAGG
GTCTGCGCGACCTGAACGGTCAGGCGTCGAAAATCGACGGCTTTCGTAAGGCAAGCGCGCAACTGGCCGTAACTGGTCAGGCGCTTGACAAGGCGAAGCGTGAAGCCGGTGAGCTGGC
TGTGCAGTTTAAAAACACCACCAGTCCGACCCGCGCGCAGGCGCAGGCGCTCGAAGCGGCAAAACGTGCCGCCTCTGAGCTGCAGACGAAATATAACAGCCTGAGAACATCGGTACAGC
GCCAGCGCTCCGAGCTGATGCAGGCCGGCATCAATACCCGCACCCTGTCTGCTGATGAGCGTCGGCTCAAAACCTCCATCAGCGAAACGACGGCGCAGCTTAATCGCCAGCGTGAGGCAC
TGGCGCGCGTCAGTGCGCAGCAGGCGAAATTAAGCCGGGTGAAAGAACGATATAAATCAGGTAAAGAGTTTGCCGGTAATGCAGCGGCGGCTGGTGCTGCAGGTGTTGGCATTGCGGCT
GCGGGAACGATGGCCGGGGTTAAATTACTGATGCCCGGTTATGACTTTGCGCAGAAAAAATTCCGAGCTGCAGGCCGTGCTCGGGGTCGACAAGCAGTCGCCAGAAATGCAGGCGCTACG
CAAACAGGCGCGCCAGCTCGGCGATAATACTGCAGCCTCTGCAGATGATGCAGCGAGCGCGCAAATCATCATTGCGAAAAGCGGCGGTGACGCTGCTGCCATTCAGGCAGCGACGCCAGT
CACGCTGAATATGGCGCTGTCAAACCGGCGCTCAATGGAGGAAAACGCTGCGCTGCTGACCGGGATGAAATCAGCGTTTCAACTTTCAAACGACAAGATTGCTCACATTGGCGACGTTCTC
TCGATGACGATGAACAAAACCGCCGCCGATTTTGACGGACTGAGCGACGCGCTGACCTATGCCGCGCCGGTGGCGAAAAATGCCGGGGTGAGCATCGAGCAAACCGCCGCAATGGTCGG
TGCGCTGCACGACGCCAAAATCACCGGGTCAATGGCGGGCACGGGTAGCCGCGCCATTCTCAGCCGCCTGCAGGCTCCCACCGGAAAAGCGTTTGAGGCCATTAAGGAACTCGGCGTCA
AAACGTCAGACAGCAAGGGGAACACGCGCCCGATATTCTCCATCCTGAAAGAAATGCAGCGCAGCTTTGAGAAAAACAACCTCGGGACAAGCCAGCGCGGCGAGTACATGAAAACCATT
TTCGGCGAGGAGGCCAGCTCGGCGGCGGCGGTACTGATGGAGGCAGCCTCAAGCGGCAAACTTGACCGGCTCACTGCCGCGTTTAAAGCCTCGGACGGTAAAACCGAGGAACTGGTTA
AGGTTATGCAGGATAACCTCGGCGGTGACTTTAAAGAGTTCCAGTCGGCTTATGAGGCTGTAGGTACCGACCTTTTTGACCAGCAAGAGAGTTCGCTGCGTAAGCTCACCCAAACCGCCAC
ACAATACGTGTTAAAGCTCGATGGCTGGATCCAGAAAAACAAAGGTCTGGCGACAACTATCGGCATTATTGCCGGTGGCGCACTTGCTCTGATTGGTATCATCGGCGGCATTGGTCTCGTTG
CGTGGCCGGTTGTCATGGGGATTAACGCCATCATTGCCGCCGCTGGCGTTATGGGTACGGTCTTTACTGTGGCCGGTAGCGCCATTGTGACAGCTCTCGGTGCGATTACCTGGCCGATTCTG
GCTGTGGGGGCGGCGATTGTGGCTGGTGCGTTACTCATCCGTAAATATTGGGAGCCCATCAGCGCATTTTTCTCGGGGGTGATTGAGGGCATCATGAGTGCCTTTGCACCGGTCGGGGAAA
TGTTCGCTCCATTAGCTCCAATCTTTGACGGACTCGGTGAGAAGCTGCGCGGCGTCTGGCAGTGGTTTAAAGACCTGATTGCACCAGTCAAGGCCACGCAGGAGACGCTCGATAGCTGCA
AAAATGTCGGCGTCATATTTGGTCAGGCACTGGCCTCTGCCTTGATGGCTCCGCTCAATGTTTTTAACAAGCTGCGCAGCGGTGTCGACTGGCTTCTCGAAAAACTCGGCATCATCAACAAA
GAATCGGACAGCCTCGACCAGACTGCCGCCAAAACCAATGCCGCCAAGCAGGGTAATTCCTGCATCCCGGCAACCAGCACATATGGCGGCTATCAGGCTTATCAGCCAGTTACCGCACCGG
CGGGGCGCTCTTACATTGACCAGAGCAAAAGCGAATACAACATCACTCTGCCGGGTGGTGTTGCGCCGGGGCATCAGCTTGACCGCCAGTTACGCGACACGCTCGAACAGATTGAACGTG
ATAAACGTGCGCGCCAGCGTGCCAGTATGACCCACGATTTCTGA
>PELPCK_26040 hypothetical protein
ATGGCAAAAGCACCACGTAAAACCGCTGAATTTATTGATACGGCTGGAAATGAAACTGACACCGTAAACCCGAACGTCGTGACCCTGGACAAACCGATTAAGCGCGCCGGTCAGACAATTG
ATAAAGTCACCCTGATTGAGCCGAACGCCGATGCCTGCGCGGCGTCAGTCTGGCGGCGGTGGCGCAGTCCGAAGTCGACGCCCTGA
>PELPCK_26045 Bacteriocin serracin-P subunit
ATGGCACTACCCCGTAAACTCAAATATCTGAATATGTTCAATGACGGCCTCAGCTACATGGGTGTTGTTGAATCCGTGACGCTGCCGAAACTGACCCGCAAGCTCGAAAACTATCGCGGCGG
CGGTATGAATGGCGCGGCAGCGATTGACCTCGGCCTCGACGATGATGCGCTCACCGTCGAATGGTCTGTCGGCGGCCTGCCTGATGTGGCTCTGTGGGCGCAGTACGCCGCCCCGGGCGC
TGATGCTGTGCCGCTGCGTTTTGCTGGTTCTTACCAGCGCGACGACGACACCGGCGAAATCGTGGCGGTCGAGGTGGTCATGCGTGGCCGTCATAAAGAAATCGACGGCGGCGAGAATAAGCA
GGGTGAAAACACCTCGACCAAACTGTCGACTGTTTGCACCTACTACCGCCTCACGATTGATGGCAGCGACGTCATCGAAATCGACACCGTCAACATGGTCGAGAAGGTGAACGGCGTCGAC
CGTCTGGAGCAGCACCGCCGCGCAATCGGGCTGTAA
>PELPCK_26050 GpF1
TTGGGGTCGGGGCTGGATACTAAAGAGGTCGCGGTCGCGCTTGCGTCGGCTGCCATTAAGTTACGTGCATTTGCTTACGTCAGCGCGTGGGGATGTAAGACTATTTCCGAAGCGATGGAAT
ATCGTAAAAATTTCAGCCAGCGCGAGCTGATGGTTATCTGGCCTGATTTCCTCGCATGGGACACCGTCAAAAATACCACCGCAACGGCTTACGCCACTGCGCGTGCACTCGGCCTGCGTGCT
TACATCGACCAGACTGTCGGCTGGCACAAAACCCTGTCTAACGTTGGTGTACAGGGCGTTACCGGCATCAGCGCCTCAGTGTTCTGGGATTTACAGGCATCCGGCACCGATGCTGACCTGCT
CAACGAGGCCGGGGTTACAACGCTGGTACGCAAGGACGGTTTCCGCTTCTGGGGTAACCGCACCTGCTCGGATGACCCGCTTTTTCTGTTTGAGAACTACACCCGCACCGCGCAGGTACT
GGCCGACACGATGGCGGAGGCGCACATGTGGGCGGTCGACAAGCCCATTACCGCCACGCTCATTCGTGACATTGTTGACGGCATTAACGCCAAATTCCGCGAGCTGAAATCAAACGGCTA
CATCGTGGAGGGTAAATGCTGGTTCGATGAGGAATCGAACGACAAGGAAACCCTCAAGGCCGGGAAACTGTATATCGACTACGACTATACACCGGTTCCGCCACTGGAAAGCCTGACCCTG
CGCCAGCGTATTACCGATAAATATCTGGTGAATCTGGCCGAATCGGTCAACAGCTAA
>PELPCK_26055 hypothetical protein
ATGAGTGATTTTCACCACGGCACGCAGGTCATCGAAATTAATGACGGTACGCGTGTTATTTCCACAGTAGCGACTGCGGTCGTCGGCATGGTTTGTACAGCCAGCGATGCAGATGCCACGCT
ATTTCCCCTCAATGAACCGGTACTGATTACCAATGTGCAAAGCGCCATTGCGAAAGCCGGTAAAAAAGGCACGCTGGCTGCATCACTGCAGGCCATCGCAGACCAGTCAAAACCCGTCACT
GTTGTTGTACGTGTTGAGGATGGAACCGGCGATGACGAGGAAGCTGCGCTCGCACAGACTGTTTCCAACATTATCGGAGGTACGGATGAGAACGGTAAATACACCGGTATCAAGGCTCTCC
TGACCGCTCAGGCCGTCACCGGCGTCAAGCCGCGTATTCTTGGGGTCGGGGCTGGATACTAA
>PELPCK_26060 tail fiber assembly protein
ATGACTTTTAAAATGAGCAGCAAAGCGCAGACAATTAAAATTTTCAATCTGCGTTCAGATACTAACGAATTTATTGGTGCGGGCGATGCGTATATCCCGCCGCACACTGGATTACCGGCTAATT
GTACTGATGTCGCCCCTCCTGAAATTCCGACCAGCCATATTGCGATATTTGATGCTGAAACACAGACGTGGACTCTGCATGAAGACCACAGAGGTGAAACGGTTTATGACACAACAACCGGT
AATCAGGTTTATATCTCCGAACCCGGGCCGTTGCCTGAAAATGTCACATCAGTTTCGCCAGAGAGTCAATACCAGAAATGGGATGGTAAGGCATGGGTGAAAGATGAAGTTGCGGAAGCTG
CAGCACGACTTATTGAAGCGGAAGGGACAAAAAACAACCTTCTGCAAATGGCTTTGACGAAAATAGCGCCATTACAGGATGCAGTTGAGCTTGATATCGCTACGAGTGAAGAGAGAGAGCGC
AGCTCAACGAGTGGAAAAAATACAGGGTACTGGTAAACAGGGTGGATACATCTGCGCCTGACTGGCCGGATATACCACGCTAA
>PELPCK_26065 Collar domain-containing protein
ATGAGCACGAAATTTAAAACCGTTATCACCACTGCCGGAGCCGCTAAGCTTGCTGCGGCCACCATGCCGGGCGGTAAGAAAATAAATCTTAACGTTATGGCTGTTGGTGACGGCGGCGGAA
AGCTGCCGGATCCTGATGCCGGTCAGACGCAGCTTGTTAATGAGGTCTGGCGTCACACTCTGAATAAAATCAGCCAGGACAACCGGTACAGTAATTACATTGTGGCCGAGCTGGTTATTCCG
CCGGAGGTGGGCGGCTTCTGGATGCGTGAGCTTGGCCTTTACGACGATGAAGGGACGCTGATTGCTGTTGCCAATATGGCCGAAAGCTACAAGCCAGAACTGGCTGAGGGCTCAGGGCG
TGCGCAGACATGCCGCATGGTCATTATTGTCAGCAGTGTCGAGTCTGTGACGCTGTCCATTGACTCAACGATGGTGATGGCGACGCAGGATTATGTCGACGACAGGCTCGCCGAACATGAA
AAATCCCGTCGTCATCCTGATGCCACTCTTAAAGAAAAAGGGTTTACTCAGCTCAGTAACGCGACAGACAGCGAGTCTGAAACGCTCGCAGCGACGCCGAAAGCTGTCAAGGCAGCATATG
ACCTTGCTGACGCGAAATATACGGCTCAGGACGCCACCACAACGCGTAAAGGGATTGTACAACTAAGTAGTGTAACCGACAGTAATGATGAAAATCAGGCTTCCACGCCAAAAGCGGTCAA
AATTGCGATGGACAACGCCAACAAGCGCCTTGCCAAAGAACGCAACCTCGCTGACCTGACAAACATCCAGAAGGCCCGTCAGTCCCTCCAGCTTGGCAACAGCGCTACACTCAATGTCGG
CACCACACCAGACACTGTAGCCGCAGGTGACGACGCCCGCATTATCACCACCAAAAAAGCCATTGACGACACCCAGAACGGTCTTGGTGCTCAGCCCGTTATGTGGGTAAGCTCCGCCGAT
GATTTGAGCAGCCTGCCGTCTGGCGCACGGCGGTTTGCCAGCAATAAAGCTCCGGCAACAATATTGCCGGTAAACGATTATGTTTTCCTGGAAGTGATTGCCAAACGCGATTGCGTAGACG
GCTGCGCCGTTCTGATAACAGACTCAATTGGTAACACCTGGATTGGCGCGCGCTGGGACGCAACCAATGGTTCCGGTTTTACCTGGCGTCCCCTGATGTCGTGTCCGCCCGGTGTTCCCCTT
CCGTGGCCGTCTGACACTATCCCTGCCGGTTATGCCCTGATGCAGGGGCAGGCATTTGATAAGAACGTTTATCCCTTACTGGCAATAGCATATCCATCCGGCACTATTCCGGACATGCGAGGC
TGGACAATCAAAGGCAAGCCTGTCAGTGGACGCGCTGTGTTGTCGCAAGAACTGGACGGCAATAAATCGCACAGTCACAGCGCCAGGGCGCAGGATACCGACTTAGGGATGAAAACTAC
CTCATCCTTTGATTACGGCACGAAGAGTTCCGATACAACAGGCGGTCATAACCATTCGGCTGGCGGACTATATGGCGGTGACTCAATCGGTGGAAAAACTCGCGTTCAGCGTGACGGAAAT
AATCAGTTAACAAGCTGGAATGGCGATCACGCACATACCACATGGATTGGCCCGCATGAACACTCCGTATACATCGGTCCACACGGACACGTCGTTATAGTGGACGCAGATGGTAATGCGGA
AACCACGGTTAGAAACATTGCATTTAACTACATAGTGAGGCTGGCATAA
>PELPCK_26070 Phage tail protein I
ATGAATGACTCACGATTGCTGCCGGTAGGCTCATCGCCACTGGAGGTTGCCGCCGCAAGGGCATGTGCTGAGATTGAAAGAACGCCGGTCAACATCCGCGCGTTGTGGAACCCTGACACC
TGTCCGGAAAATTTGCTGCCGTGGCTGGCGTGGGCATTTTCTGTCGACCGGTGGGATGAGAACTGGCCGGAGGGAACAAAACGTGCCGTTATCCGTGATGCATATTTCATTCACTGCCACA
AGGGGACTATCGGCGCAATCCGTCGGGTAGTGGAGCCACTCGGCTATGTCATCAATGTAACGGAATGGTGGGAAAGCGGCGACCCGCCAGGCACATTCCGGCTTGATATCGGGGTGCTTG

AAAGTGGCATTACCGAGGAAATGTATTTCGAAATGGAGCGACTGATTGCGGATGCAAAGCCAGCCAGTCGTCATCTGACTGGCCTGAATATTGTCCAGGACATTCCCGGTTATTTGTATACCG
GCGGCGTGTCCTGCGACGGCGATATTATTACGGTTTACCCGGGATAA
>PELPCK_26075 Baseplate-J domain-containing protein
ATGGCAACTGTTGACCTGAGTCTGCTACCTGTTCCTGATGTGGTCGAGGAACTGGACTATGAAACTATCCTTGCGGAGCGCATTGCAACGCTGATTTCGCTCTATCCGGAAAACCAGCAGGA
AGCCGTCGCCCGGACGCTCGCACTTGAGTCTGAGCCAATTGTTAAATTGCTGCAGGAAAACGCCTACCGCGAGGTTATCTGGCGTCAGCGTGTCAATGAAGCTGCACGCGCAGTGATGCTG
GCTTATGCCATAGACAGTGACCTCGATAATATCGGGGCGAATTTCAGTGTTGAGCGCCTTGTCGTCACGCCTGCTGATGACACCACCATTCCACCCACTCCGGCAGAAATGGAACTCGACGC
AGATTATCGTCTGCGTATACAGCAGGCTTTTGAGGGACTGAGCGTGGCGGGGTCTGTCGGATCGTACCAGTATCACGGCCGTAGTGCTGACGGGCGCGTCGGCGATATTTCAGTTATCAGCC
CGTCGCCAGCTTGTGTGACGATTTCCGTGCTGTCTCGTGAAAACAACGGCGTCGCATCTGAGGAACTGCTTGCAATTGTGCGCAATGCCCTGAACGCAGAAGATGTCAGGCCGGTCGCTGA
CCGGGTGACGGTACAGTCAGCCGAAATTGTTAACTACCAGATTAACGCCACGCTTTATCTTTATCCCGGCCCGGAAAGTGAACCCATCAGGGCGGCGGCTGAGGCAAAGCTGAAAGCCTAT
ATCAGCGCGCAGCACCGCCTCGGGCGCGATATCCGTAAATCAGCGATTTATGCCGCCCTGCATGTTGAGGGTGTTCAGCGGGTGGAGCTGGCGGCACCGGTCGCGGATATTGTTCTCGATA
ACACACAGGCGTCCTTTTGCACTGACTACAGCCTTGTAATCGGGGGCTCTGATGAATGA
>PELPCK_26080 GPW-gp25 domain-containing protein
ATGACGGCGCGTTATCTGGGGATGAACCGCAATACCGGCCTCGCTATCAGTGACAGTGAGCATATCAGCCAGAGCATGCGCGACATTCTGCTGACGCCGGTCGGCTCGCGGGTAATGCGTC
GTGAATATGGCTCGCTCCTGTCTGCGCTGATTGATATGCCGCAAAACCCGGCGCTCAGGCTGCAAATCATGGTGGCGTGCTATTCCGCGATCCAGAAATGGGAACCACGCATCAGGCTTACC
TCAATCAGCTTTGAGCGTGGCGACACTGGCGAAATGTATGTCGATATTACCGGGATGCGTACCGATACCGGTGCGTCAGTTTCAACCACTGTTTCACTGAGTTAA
>PELPCK_26085 hypothetical protein
ATGCGGGGAAACATCGAACATACCGGTGGCACGTTGAAATCAAACGGCGTACAGGTCGATGACCACGGTCACGGCGGCGTGCAACGGGGCGGGAGCTGGACGGAGGACGCCAGATGA
>PELPCK_26090 Baseplate assembly protein V
ATGAATACGTTATCCACAATACAGGAGCTCACGCGCGCGATTCGCAACCTCATCCGCTCAGGTGTGGTGACTGAGGTCGATACCGCGCAGGGGCTGTGCCGCGTACAAAGCGGCGGGATCC
AGACTGCATGGCTGAACTGGCTGACCACCCGCGCCGGTCGTTCGCGGACATGGTGGGCTCCCTCGGTCGGTGAGCAGGTGCTGCTGCTGGCAATTGGTGGCGAGCTTGATACTGCTTTCG
TGCTGCCGGGGATTTTCTCCGACGATAACCCTGCCCCGTCAGCCTCGGCGGATGCGTGGCATGTGGTGTTCCCCGACGGCGCTGTTATGGAGTATGAGCCGGAAACCGGTGCACTGACGGT
CAGCGGCATCAAGACTGCCGATGTGACGGCATCGGAGTCCATTACCGCCACCGTGCCGGTGGTACTGGTAAAAGCGGCAGAACGTATCACCCTCGACACCCCGGAGGTGGTATGCACCAA
CAAACTGACGACGGCGACGCTTGAGGTGCAGAAAGGCGGCGACCATGCGGGGAAACATCGAACATACCGGTGGCACGTTGAAATCAAACGGCGTACAGGTCGATGA
>PELPCK_26095 Phage tail protein
ATGAGTGAGTTAACCGCGCTGCAGGAGCGCCTTGCCGGTCTGATTGCCAGCCTGTCACCGGCGGCGCGTCGTCAAATGGCGGCTGACATTGCAAAAAAACTGCGCGCCAGTCAGCAGCA
GCGCATCAGGCGACAGCAGGCACCAGATGGCACCCCGTATGCCGCCCGAAAGCGCCAGCCGGTGCGAAGTAAGAAAGGCCGTATCAGGCGCGAAATGTTCGCCAGACTGCGCACTAACC
GCTTTATGAAAGCCAAAGGCAGCGACAGTGCGGCGGTGGTGGAATTTACCGGCAGGGTACAGCGCATGGCGCGGGTGCATCAGTATGGCCTCAAAGACCGGCCAAATCGTCACAGCCGG
GATGTGCAGTACGCGGCGCGCCCGTTGCTCGGTTTCACCCGCGACGATGAGCAGATGATTGAAGACATCATTATCAGGCATCTCGGTAAATAA
>PELPCK_26100 N protein
ATGCAGAAGCACAAAAGCCTGCGCAAGGCGCTGATTAACGCCGTGCCGCAGCTCCGAAACAACCCCGATATGCTGCGCCTGTTCGCTGATAACGGGCATACGGATTCCCGACTGGAGAGC
TCGCTGTCGTTTGAAAAGGTGTACGTGCTTAACGTGGTGGTGACTGACTTTACCGGCGACCTCGATTTGATATTTGTGCCGGTACAGGCATGGCTGCGTGAGCATCAGCCGGACATTATGAC
CACCGACGACGGGCGGGAGAAAGGATTCACATGGATGATTGATATCAATAACGACGATTCGCTCGATATCAGTATCAGCCTGAGACTCACCGAGCGCACGCTCGTCAAAGAGGTCGACGGC
GCATTGCATGTCAGCTATGCCCCTGAGCCACCGCTGCCTGAGCCAGTGACACGCCCGGTCGAGCTGTACGTTAACGGCGAGCTGGTGAGTAAGTGGGATGAGTGA
>PELPCK_26105 hypothetical protein
ATGCCGGGAAGTGATCCGAAAACTAATGGTGACCTGAGCGCTGATATCCGCCGCCTTGAGGGCGCGCTGACCGCCTGCGCGCTACAGGTCAAAACCGTCAAACACTGTCAGGATGAACTC
GATGCAGAAGCACAAAAGCCTGCGCAAGGCGCTGATTAA
>PELPCK_26110 hypothetical protein
ATGCCGGTCATTAATACTCACCAGAATATCGCCGCCTTTCTGGACATGCTGGCGTATTCCGAAGGAACGGCGAACCATCCGCTGACGAAAAACCGTGGCTACGACGTCATTGTTACCGGCCT
TGATGGCAGGCCAGAGATTTTCACCGATTACAGCGACCACCCTTTCGCACATGGCCGACCCGCGAAAGTGTTTAATCGCGGTGGCGAGAAATCCACGGCATCGGGGCGTTACCAGCAGCT
TTATATGTTCTGGCCGCACTATAAAAAACAGCTCGCATTGCCTGATTTCAGCCCACTGTCGCAGGACAAGCTCGCGATCCAGTTAATCCGGGAGCGCGGTGCTATTGACGATATCCGGGCGG
GGCGTATTGAGCGTGCTGTTTCCCGTTGCCGGAATATCTGGGCGTCATTGCCGGGTCGCCGGTTACGGCCAGCGCGAGCACAGTCTCGAAAAGCTGGTTACCGTCTGGCGCACGGCTGGC
GGGGTGGTGGCATGAAAGTCCTGATAACGCTGTTTGTGCTGGCCGTGCTCGGTCTGATGTGGTTGCGCCATGAGAATGGCAATTTATCCCGCTCCTTTGAGACGGCAAACCGCGTTGCGAG
CGAGCAAAAGGCGACGATTGGCATGCTGAAAAATCAGCTCAGTGTCGCCGGCCAGCTCGCCCGACGTAATGAATCCGCGCAGGTGGCACTGCGCGAACAGCTCGCAAAGGCAGGCGCA
GAAGCAAACCGCCGCGAGCAGACGATAACGAGGTTACTTGATGAAAATGAAGCCTTTCGCCGCTGGTATAACGCTCCTCTGCCTGATGCTGTGCGCAGGCTGCACACCCGCGCCGCCTGCG
CCAGCGCCGGTGATTGTGGTCAGCGGATGCCCGAGGGTGAGCCTTTGCCCGATGCCGGGAAGTGA
>PELPCK_26115 Holin
ATGACAGAAGGGGAAAAAGGCGTCCTGTCACTGTTTGTGATTGGGGCACTGATTGTGGTCGGAAAAGTGCTGGCAGGTGGTGAGCCCATCACCCCGCGCCTGTTTGTCGGACGCATGTTG
CTCGGCGGTTTTGTCTCAATGGTCGCCGGTGTTGTTCTGGTGCAGTTTCCTGATATGTCACTGCCCGCTGTGTGCGGTATTGGATCCATGCTCGGTATTGCCGGTTATCAGGTGGTGGAAATC
GCCATTCAGCGCCGCTTTAAGTCACAGAAGGGGGAAGGCGATGCCGGTCATTAA
>PELPCK_26120 Tail X family protein
ATGCAGGGCGACACCCTCGACGTGATTTGCGCCCGGTATTACGGGCGCACTGAGGGCGTTGTTGAAACTGTGCTGCAGGCTAATCCCGGCCTGTCTGAGCTGGGCGTCATTCTGCCGCATG
GAACGGCAATTGACCTGCCCGATGTGCCGTCTTCACCCGTAACTGAAACTATCAATCTTTGGGAGTAA
>PELPCK_26125 Capsid completion protein
ATGACGACAGTGATTCTGAACCAGCCCGACGAACCGCAGGACGTACCGGGCGTGGTGATTCCCGTACCGGAGACGGGCGATGCAGTAATTAAAAACACGTTCTTTTTCCCTGATGTGGATC
CGAAGCGTGTGCGCGAGCTGATGCGGCTTGAACAGACGGTTTCCGATGCGCGCCTGCGCCATGCCATCAGAACTGGCATGGCGGAAACCAATGCGGAGCTTTACGACTACCGGCTGCGCC
AGACTGCCGCCGGGTTTAAGCATCTGGCCGACGTGCCTGCTGAGGAAATCGATGGCGAGAATGTGCGTATTTTCCACTATCTGAGAGCCGTAACGGCGATGGCAACCGCCACCCTGTATGA
GCGCTATCGCGGTGTTGAAGCCACCGGCAAGGGTGACAAAAAAGCCGACAACGTCGAAACCACCATTGATGACCTGTGGCGGGATATGCGCTGGTCGGTCGCGCGTCTGCAGGACAAAC
CGCGCTGCATCGTGGGCCAGCTCTGA
>PELPCK_26130 M protein
ATGACGAGCCCCGCACAGCGTCACATGATGCGGGTCTCGGCCTCTCAAGCCGCGCAGCGGGAGCAAGCCCCACTGCGCCATGCAACCGCCTATGAGCAGATGCTGGTTAAGCTGGCCGAT
GACCGCCGCACGTTAAAAAACATCCGTTCAAACGAACGTAAAGCCGAGAAAAAGCGCGAGCTGCTGCCGTTCTATGCGCCGTGGGTCGCCGGTGTGCTGGCTGATGGTCGCGGTGCGCA
GGATGACATTGTCATGACCGTCATGCTGTGGCGTCTTGATGCCGGTGATATCGCTGGCGCGCTGGAAATTGCGCCCTATGCGCTGAAATACGGCCTCACCACTGACCATCGCCGCACGACGC
CTTACATGCTGGTTGAGGAGGTGGCGCTTGCCGCACTGCGCCTGCGCGATGCCGGTGAGCCTGTCGACCTCGCATTACTGCTGACCACCCTCAGCCTGACCGACGGCGCTGACGTTCCCGA
TATGGTGCGCGCCCGTCTGCATAAGGTGACCGGCCTGACCCTGCGCGATATCGGTCAGAATGCTGAGGCACTGGCTCAGTTTCAACGTGCGATGCAGCTCGACCGCAATGCCGGTGTGCGC
AAAGAGATTGAGCGGCTGGAACGCGCACTGAAGCCAAAAGCGGAGGCACCACCCCGTAAAACGACTAAACCGCGCACGCGCAAACCTGTCGCCAGACCGGCAGCAAAGCGCGGGCGT
CCACCAAAGGCGGTAAAAACCGCCGGTTAA
>PELPCK_26135 Major phage capside protein
ATGCGTCAGGAAACCCGTTTTAAGTTCAATGCCTATCTGACCCAGCTCGCCAAACTGAACGGCATCAGCGTTGATGACGTTAGCAAAAAATTCACCGTCGAGCCGTCCGTCACGCAAACGCT
GATGAACACCGTGCAGGCGTCATCCGCATTTCTGCAGATGATTAACATTTTTCCGGTTGATGAGCTGAAAGGTGAAAAAATCGGTGTGGGTGTGGATGGCACTATTGCCAGCACGACCGAC
ACCTCAGGTGACGATGAGCGTGAAACTGCTGACTTTGTCGCGCTTGAGTCCAATTTGTACGAGTGCAACCAGGTCAACTTTGACTTCCACCTGCGCTACAAGACGCTCGACCTGTGGGCGC

GTTTTCAGGACTTCCAGCGCCGCATCCGTGACGCGATTATCCAGCGTCAGGCACTGGATTTCATCATGGCCGGATTTAACGGTACTCATCGTGCGCCGACCTCGAATCGCAAAACTAATCCAA
TGTTGCAGGATGTAGCGGTCGGCTGGCTGCAGAAGTACCGTAACGAAGCACCGGCGCGCGTGATGAGTAAAATCACCGACGATGAGGGTCAAGTCGTTTCCGATGTGATTCGTGTCGGTA
AAAACGGCGACTATGAGAATCTTGACGCGTTGGTAATGGATGCGACAGGTGCCTGA
>PELPCK_26140 GPO family capsid scaffolding protein
ATGGCAAAAAAAGTCTCAAAATTCTTTCGCATCGGCGTCGAGGGTGATACCTGCGACGGGCGCATTATCAGCGCCAGCGATATTCAGGAAATGGCCGAAACCTATGACCCGCGCGTCTACG
GTTGCCGTATCAACCTTGAACACATTCGCGGCCTTTTGCCCGACGGCATGTTTAAACGTTATGGCGATGTGGTTGAGCTGAAAGCCGAAAAGATTGACGACGATTCTGCGCTGAATGGCAA
ATGGGCGTTGTTCGCCAGAATCACCCCAACCGATGACCTTATCGCGATGAATAAAGCCGCGCAGAAGGTCTATACCTCTATGGAAATTCAGCCGAATTTTGGTAACACCGGCAAATGCTATCT
TGTCGGCCTTGCGGTCACTGATGACCCTGCGAGCCTCGGCACTGAATACCTCGAATTCTGCCGTAAGGCGAAGCACAACCCGCTGCAGCGCTTTAAGGCCAGTCCTGAAAATGTCTTTTCA
GTCGCCACGCTGGCCGAACTGGAATTTGAAGATGTTCCCGACACAGTGCTTAACAGCCTGGCCGATAAGGTGAAAGCCATTTTCAGCCGTAAGCAGGTCAGCGACGATGCGCGCATGAGT
GATGTGCATGAGGCGGTGACCGCCGTCAGCGAACATGTGCAGACCAGTCTCACTGCGCAGGATAAGCGTCTTTCCGATATGGAAACCGCGCTAGCCACCTTTAAACAGGAACTGACCGGC
AAGGTTGAAGAAACCAGCCAGGCATTTTCCGCCCTGAAAACCACCCTCGACAAAACCGAAAGTTTCAGCCAGCCGCGACGCACGAAAGCCAGCGGCGGCGGTGGCGACGAGCTGCTGA
CCGACTGCTGA
>PELPCK_26145 hypothetical protein
ATGACCATTTCCACCGATACAACCTTATTGCATGACCCGCGACGACAGGCATCGCTGCTTTACTGGCAGGGCTTTTCCGTGCCACAGATTGCCGAAATGCTGCAGGTCAAGCGCCCGACCGT
GCAAAGCTGGAAACAGCGCGACGGCTGGGACGGCATCGCACCGATTTCCCGTGTCGAAAGCAGCCTTGAGGCGCGCCTGATTCAGCTCATCGCCAAGCCGCAAAAGTCAGGCGGCGACT
TCAAAGAGATTGACCTGCTCGGGCGGCAGATTGAGCGACTGGCGCGCGTCAACCGCTACAGCCAGACCGGCAACGAGGCCGACCTTAACCCCAACGTTGCCAACCGTAACAAGGGGGA
GCGTAAGAGGCCGAAAAAGAACTTTTTCAGCGATGAGGCTGTCGCAAAGCTGGAAGAAATTTTCTTCGACCAGTCTTTCGAATACCAGTTGCAGTGA
>PELPCK_26150 Terminase-6C domain-containing protein
GTGTTCCGGGAATACATCATCCAGTTTGCGCGACTGGTTGACGTCGACCTGACCGGCGACCCGATTGTCATCGGCAACAACGGCGCAAAGCTGATTTTTCTCGGCACCAATTCCAACACCG
CACAAAGCCATAACGGCGACCTGTATGTCGATGAAATATTCTGGATCCCGAATTTTCAGAAGCTGCGCAAAGTCGCATCGGGCATGGCCTCGCAAAAGCACCTGCGATCAACTTACTTTTCG
ACACCTTCCACGCTGGCTCACGGCGCTTACCCCTTCTGGTCTGGCGAGCTGTTCAACAAGGGGCGCGCCAGTGCCGCTGACCGCATCGAAATCGACATCAGTCACAGCGCGCTCGCCGGT
GGACTTCTTTGCGCAGACGGACAGTGGCGGCAGATTGTCACTATTGAGGACGCCCTTGCCGGTGGCTGCACCCTGTTCGACCTCGACCAGCTCAGACGCGAAAACAGTGATGAGGACTTT
AAGAACCTGTTTATGTGCGAGTTTGTCGACGATAAGGCATCGGTATTCCCGTTCGAGGAGCTGCAGCGCTGCATGGTCGACGTGATGGAAACATGGGAGGACTTCGCCCCGTTCGCCGACC
ATCCATTCGGCTCGCGACCGGTCTGGATTGGCTACGACCCGTCCCACACCGGCGACAGTGCCGGATGTGTCGTACTCGCGCCGCCGGTGGTTTCGGGTGGCAAGTTTCGCATGCTGGAGC
GTCACCAGTGGAAAGGCATGGACTTTGCCGCGCAGGCAGAAGGCATCCGCAGGCTCACTGAGAAATACAACGTCGAATACATCGGCATTGACGCAACAGGCCTCGGTCTCGGCGTATTCC
AGTTGGTGCGCTCATTCTACCCGGCGGCACGCGGTATCCGTTACACGCCAGAAATGAAAACCGCAATGGTGCTCAAGGCGAAAGACACGATTCGCCGTGGCTGTCTGGAGTACGACGCCG
GAGCAACTGATGTCACACAGTCGTTTATGTCCATCCGCAAAACCATGACCAGCAGCGGGCGCAGCGCCACCTATGAGGCCAGCCGCACCGAGGAAGCCAGTCACGCTGATATCGCATGGG
CCACCATGCACGCCCTGTTAAACGAACCGCTTTCTGCCGGTAGCGGCATGCAGCCTAAATCTATTCTGGAGTTCAACTAA
>PELPCK_26155 hypothetical protein
ATGGGTAAGCAAAAATCCCGTAAAGCCGCCGCGCAGAAAGCCCGCGCACCACAGCAACTGAAAGCCAGCGCACCGCAAAAATGGAAGCGTTCACCTTCGGTGAGCCGGTCTGCCGGTAC
TCGATAA
>PELPCK_26160 Phage portal protein
GTGCATCACAGCTCACCGATTTACGTTAAACGCAATGTGCTCGCAAGTACCTACATTCCGCATCCACTGCTGTCCCGTCAGGATTTCAGCCGCTTTGCACTCGACTATCTGGTATTCGGTAACG
CCTTTCTTGAGCAGCGCCACAGCGTCACCGGCCAGTTAATCAAACTGCTGACTTCACCGGCAAAATATACCCGGCGCGGGGTCGATGACTCGGTTTTCTGGTTTGTGGAAAACTTCACTCAA
CCGCATGAGTTCGCACCCGATACCGTGTTCCACCTGCTGGAGCCTGATATTAATCAGGAGATTTACGGCCTGCCTGAATATCTCAGCGCGCTTAATTCCGCATGGCTGAATGAATCCGCGACG
CTGTTCCGCCGCAAGTATTACCAGAACGGCGCGCACGCAGGTTACATCATGTATGTGACTGACCCGGCGCAAAGCGCGACTGACGTCGAATCGCTGCGCGATGCAATGCGTAACTCTAAAG
GGCTCGGCAACTTTAAAAACCTGTTTTTCTACTCACCGAACGGGAAACCGGACGGCATAAAAATCGTGCCATTGAGCGAAGTCGCCACAAAGGATGACTTTTTCAACATCAAGAAAGCCAG
CGCCGCTGACCTGATGGATGCGCACCGCGTACCTTTCCAGCTCATGGGCGGCAAGCCTGAGAATATCGGCTCAATGGGTGATGTTGAGAAGGTGGCAAAGGTCTTTGTGCGTAACGAGTTA
TCGCCCTTACAGGACAGATTCAGGGAGGTAAACGACTGGCTCGGCATGGAGGTCATCAGGTTCAAAGAGTACACCCTCGATAACCCGGAATAA
>PELPCK_26165 Helicase ATP-binding domain-containing protein
ATGTCTGGTTTTTTCCTTGATTTAACCAAGCTTTCGCCCAGTGTAAATAGCGACACAGCGATTCCACCGCGAGATATATTTACAGCTCTACCGAGCAAAGACACCAAATTTCAATACCCTCGAG
ATGTGCAATCAGAGGTCTGGGAAAAATGGTATGAAGCAAGGAGAAGCCCAACCAATGTAATAAAAATGAATACCGGAAGCGGTAAAACTTCAGTTGGGTTAATCATTCTTAAAAGCTGTATA
AATGAAGGAGAAGGTCCCGCTGTATATGTTGTTCCTGACAACTATCTAGTTGAGCAAGTGGTTTTGGAGGCTAATCAACTTGGTATACCTGTAACTCAGGATGAGAGGTCCCCCAAATTTATT
GCAGGTAAAGAAATTTTAGTTACAAATATATTCAAAGTGGTTAATGGACGTTCTGCATTTGGCGTTGGAGATGATGGACAAAAAATACCAATAGGTTCTATAATAATTGACGATGCTCACGCTT
GCCTTTCTTCAATTGAAGAACAATTCTCCATTAGCATTCAAAAAAATAACCCTGTCTATGATAAATTATTCAGAATTTTTGAAAATTCACTAATGACACAAGCTGGTGCAAAAACACTAGAGATA
AAATCCGGCGATAGAAATGCTTATGTGAGAGTTCCATTTTGGAAATGGCAAGAAAGCATAAATGATATAATGACAATCTTGTTAGAAAATAAAGACCATGATGACTTGAGTTTCAAATGGCCA
TTAATAAAAGACAATCTAATATTATCGAAATGCGTTGTAAGTGCTTCCAAAATAGAGATATCACCTCATTGCATACCAATCCAAATGATCCCAAGCCTATCAAACGCAAGAAGAAAAAATATTCAT
GACAGCTACTCTTGTCGATGAATCCATTCTAGCGAGCCACTTCGGAATAACGGATGAATCACTCTCTAATCCGATAACACCAAAAACAATTGGTGATATTGGTGATAGAATGATACTAATGCCT
CAGGTTATAAACCCTAGTCTTTCTGATGTAGATATCAAAGCTATGTGCAAGGAGATATCCTCCAAACATAATGTTGTTGTAATTGTACCATCCGATTACAGAACTAGATTTTGGCAAGATGTTGC
AGACAGAATATTAGATAAAAATACAATTTATCAAGGTGTACAAGAATTAAGGACACAACATGTTGGCTTAGTAGTCCTAGTTAATAGATATGATGGTATTGATTTACCAAATACCGCATGTCGTC
TATTAGTAATAGATGGACTTCCTGACGTTCGTAGACTAATAGATAAAGTCTCACAAAGCTTATTGCTTGGAAGCGAAAAAAACCAAAGATGAAATTATCCAGAAAATTGAACAAGGAATGGGG
CGAGGAGTGAGATCAAGCGACGACTTCTGCGGAGTGATCTTACTTGGCAAAGCATTAAATGGCGCTGTTTTCCTTGGCTCCTCCTTAGAAAGGTTTTCTCCAGCAACGAAAGCACAAATTC
AACTTTCACAGCAATTGGTATCGATACTGCCGGATACAACTATTGATTCAATCAAAGGTGCTCTTGATTATTGTCTGTTACGCAATTCTGACTGGGTCTCAAAAAGCAAAGGTATATTGACTGG
CTTAACATTGGAAAATAAGCAGATTGACCAGCATACTATAAACAAAAGATTGGCATATGACTTAGCATCACGAAATATGTTTCAACAAGCCGCTTTAACTCTAAAAAATGATAGTAGTACAGCT
GACAAAGTTTATAAGGGTTATCTAAAAGAACATGCTGCGGAATATGTAAACTTATATGACAAATCTGAAGCTCAGATATTGCTTCAGTCTGCATCAAACGATAACTATCGGGTATTAAAACCAC
TAATCGGCGTAACATATAATAGACTTAATGGTGCGGCACTTGAACAGGCCAGGGAATGTAGCTCCTATCTCAGAAGTAATTTTGAATCGGCGAATCAAGTCGTTGTGCATACTAATTCAATAAT
AGAAAACCTTATTTTCTCCGAGGGCACATCGAATCCCTTCGAAGATGCTATCGAAAAAGTTGCATATTTGGTCGGTTTTCGTAGCCAACGGCCAGAAAATGATACAGGTAAAGGACCTGACA
ATTTATGGGCAATGGGAGAAAACAATTATCTAGTAATTGAATGCAAAAATGGAGCAACCGCAGAAAGAATAAGTAAGCATGACTGCAATCAGTTAAATGGGTCGGGTGCATGGTTCAGAAA
CATGTATGATCAAACTGCTACCGCCACACCTATAATGATACATCACTCTAACATGCCTGAATATGCCGCAACACTAAACGAAGGGTCTCGAATAATGACCATAAATGATCTTGAACGTTTTAAAG
CATCTATCCTCAGTTTTATCACGGCCATTTGTACAAGTGATAAACGACACGATGAGATATTTATAAGAGAACAATTGATTACATGTAAATTACGTGCATCAGACATAGTTGAAACCTATACGCGA
AATCCTCGTTAA
>PELPCK_26170 Replication gene A protein
TTGTCCCTCCGTCAGCATTTGCTGCAGGCGACCGGTAAGGCGTTTACCGGTGCTTATGCATGGAACGCGCCACGCGAGGCCGTCGGGCGCGAAAGACCCCTTACACGTGACGAGATGCGT
CAGGTGCAAGGTGTTTTATCCACGATTAACCGCCTGCCTTACTTTTTGCGCTCGCTGTTTACTTCACGCTATGACTACATCCGGCGCAATAAAAGCCCGGTGCACGGGTTTTATTTTCTCACAT
CCATTTTTCAGCGTCGTTTATGGCCGCGCATTGAGCGTGTGAATCAGCGCCATGAAATGAACACCGACGCGTCGTTACTGTTTCTGGCAGAGCGTGACCACTATGCGCGCCTGCCGGGAATG
AATGACAAGGAGCTGAAAAAGTTTGCCGCCCGTATCTCATCGCAGCTTTTCATGATGTATGAGGAACTCTGCGATGCCTGGGTGGATGCGCATGGCGAAAAAGAATCGCTGTTTACGGATG
AGGCGCAGGCTCACCTGTATGGTCATGTTGCTGGCGCTGCACGTGCTTTCAATATTTCCCCGCTCTACTGGAAAAAAATACCGTAAAGGACAGATGACCACGAGGCAGGCATATTCTGCCATT
GCCCGTCTGTTTAACGATGAGTGGTGGATTAGTCAGCTTAAAGGCCAGCGTATGCGCTGGCATGAGGCGTTACTGATTGCTGTCGGGGAGGTCAATAAAGACCGTTCTCCTTATGCCAGTAA
ACACGCCATTCGTGATGTGCGTGCACGCCGCCAGGCAAATCTGGAATTTCTTAAATCGTGTGACCTTGAAAACAGGGAAACCGGCGAACGCATCGACCTTATCAGTAAGGTGATGGGCAGT
ATTTCTAATCCTGAAATTCGCCGGATGGAGCTGATGAACACCATTGCCGGTATTGAGCGTTACGCCGCCGCAGAGGGTGATGTGGGGATGTTTATCACGCTGACCGCGCCGTCAAAGTATCA

CCCGACACGTCAGGTCGGAAAAGGCGAAAGTAAAACCGTCCAGCTTAATCACGGCTGGAACGATGAGGCATTTAATCCAAAGGATGCGCAGCGTTATCTCTGCCGTATCTGGAGCCTGATG
CGCACGGCATTCAAGGATAATGATTTACAGGTCTACGGTTTGCGAGTCGTCGAGCCACACCATGACGGAACGCCGCACTGGCATATGATGCTTTTTTGTAATCCACGCCAGCGTAACCAGAT
TATTGAAATCATGCGTCGCTACGCGCTCAAAGAGGATGGCGACGAAAGAGGAGCCGCGCGAAACCGTTTTCAGGCGAAACACCTTAACCGGGGCGGTGCTGCGGGGTATATCGCGAAATA
CATCTCAAAAAATATCGACGGATATGCACTGGATGGTCAGCTCGATAATGATACCGGCAGACCGCTGAAAGACACTGCCGCGGCTGTTACCGCATGGGCGTCAACGTGGCGCATCCCGCAAT
TTAAAACGGTTGGTCTGCCGACAATGGGGGCTTACCGTGAACTACGCAAATTGCCTCGCGGCGTCAGCATTGCTGATGAGTTTGACGAGCGCGTCGAGGCTGCACGCGCCGCCGCAGACA
GTGGTGATTTTGCGTTGTATATCAGCGCGCAGGGCGGGGCAAATGTCCCGCGCGATTGTCAGACTGTCAGGGTCGCCCGTAGCCCGTCGGATGACGTTAACGAGTATGAGGAAGAGGTCG
AGAGAGTGGTCGGCATTTACGCGCCGCATCTCGGCGCGCGTCATATTCATATCACCAGAACGACGGACTGGCGCATTGTGCCGAAAGTTCCGGTCGTTGAGCCTTTGACTTTAAAAAGCGG
CATCGCCGCGCCTCGGAGTCCTGTCAATAACTGTGGAAAGCTCACCGGTGGTGATACTTCGTTACCGGCTCCCACACCTTCTGAGCATGCCGCAGCAGTGCTTAATCTGGTTGATGACGGTG
TTATCGAATGGAATGACCCGGAGGTCGTGAGGGCGCTCAGGGGTGCATTAAAACACGGTCGGAGAACGCCAAGTCGTCAGCAAAGAAACGGAAGCCCGTTAAAACCACATGAAATTGCA
CCATCGGCCAGACTGACCAGGTCGGAACGAATGCAAATCACCCGTATCCGCGTTGACCTTGCTCAGAACGGTATCAGGCCGCAGCGATGGGAGCTTGAGGCGTTAGTACGCGGTGCGATC
GTCAATTACAATGAAAAGCAATTTTCATATCCTAGTGTAGATGAGTGGAGTGGGTTTTCTGGTTATTCAGAGTAA
>PELPCK_26175 Uncharacterized protein in GpA 5'region
ATGAGTATTCGTATTGAAATTGGCGAACGTTATGTCGTTACCAGTGACAGCTTTCAGTTTATTCTCCACGAGAAAAAGAGAGCGGAAAGCGGTAAAAACGCCGGTCAGGAATGGCTGGCGG
TGGTTGGTTATTATCCGAAATTAAGCCAGCTCGTTTCCGGCCTGATGCATCACGATATTCTGACCGGAAGCGCAAAGTCTTTTGCTGATTTAAACGCGCAGGTTGAGCAACTCAGCAGGCGT
TGTTCAGAGGCTTTTGGCTCATATGGCCGTTAA
>PELPCK_26180 Uncharacterized protein in gpA 5'region
ATGCCGGACAACGTAGATTTTATTCAGGAACAACAGGCTGAATTACTGGAGCGTCAGATTAACGCGGCAAGGGTAAAACATTGCGGTGCTTCTGCGCTGGTTTGCGAAGAGTGTGACGCG
CCAATACCTGCTGCCCGTCGTGCGGCTTATCCGTCAGCCACGCGTTGTGTTTCCTGCCAGTCAGTCTTTGAAGCAAAAAACAAACATTACCGGAGAACGGCATGA
>PELPCK_26185 Uncharacterized protein in GpA 5'region
ATGTCCTGTTCTCATTCAGTTGTATTACTGAATAACGCCTTAAAAATCGCCGTTATGAAAAATGGCGATTTATCTCTTATTCAACTTGGTCTTGATAAAGAAAAACGCGAAATAACTGAGTCTGT
TATCGCGATTTATCAGAACGAATTAAATCTCCTGTCTGATGTGGTCAATTTACTTGTTAAACGCGCTGTATTTCACAAGCAAATCTCCTCCGTGGATGAACTGACGAAATTAACGACAGAAGTT
GCCAGCTATTGCGCTGATGAATTTAAAAAACTTAACGACAAAAGGAGCTGGTAA
>PELPCK_26190 Uncharacterized protein in GpA 5'region
ATGCATACAGTTTCTGAAAATCAGTGCGGTAAATACGCATTACTGCTGCAACAGGCCAGAACCGAAGCACAGGCCGACGCTGCGACGCGCTTTTCTTCTCATCTTGACGCCATGATTCGCCA
CATAACAAAGGCGGAGTTATCCCGCGTGGAGATAGTCGAGCTGCTCAGTCAGGAGTCGGAAAAAATTTCACAATATCGGATTGTCTCGCGGGGAGGTGCTTTGA
>PELPCK_26195 Replication gene B protein
ATGACAGTGATGACGCTCAATCTCGTTGAAAAACAGCCAGCAGCTATGCGCCGGATAATTGGTAAGCATCTGGCCGTCCCTCGCTGGCAGGATACATGTGATTATTATAATCAGATGATGGAG
CGCGAACGGCTAACAGTTTGCTTTCATGCGCAGTTAAAACAACGTCACGCAACGATGCGTTTTGAAGAAATGAACGACGTCGAACGTGAACGGCTGGTTTGTGCAATTGATGAATTGCGTG
GGGCATTCTCAAAACGCCGTCAGGTTGGCGCAAGTGAGTATGCATATATTAGTTTTTTAACAGTCAGTCAGCGTCGTACTTTATTTATGCATGCCGGATTGACTGAAAAAGAATTCAACCAGC
CATACTGGCGAATTAATGAAGAGTCATGTTACTGGCGTGATGCTTTATTCCGTGCATTACGTGAATTATTCAGTCTGTTTGAGTATGCACCGACAATTCTGACGTCGGTAAAACCAGAGCAATA
TCTGCATTAA
>PELPCK_26200 hypothetical protein
ATGGCTTTTGTGGATGGGGTTGTGATTATGAATGAGCCGCGTTGTATTGCTCAGTTACTGCGTAACGAAAGCCCCAGGGCGATTGACTTCACCATCACCCACGGGAAGGGATGCAAGGGAA
TCATTATCCGCACCAAAAAACAGAGTCCGTTAAAAAAGGCTCTGACCTTTCTGAAAAGCCGGAGGGTATGGAAATGA
>PELPCK_26205 Cox protein
ATGGAAGTTAATGATTATGTGATTAAGTACCCGCTTGATGCGGTACATGCGGAAAAGTTTGCAGATTTATTGGGTAAACCAAAAACCGCAGTTACAGAAATGATAAAGGCTAACAAGCTACC
GGTTATCGAGCTTCGAGATCCAAATAAGCCGAAGGCCCGCGCCGGTGAGAAATGGGTTTTCATTCCAGAGTTTAATCGCGCTGTACGTGAGGCGTTTTATAACCGACCGGTTGAACAGCGT
GATGCATGGCTTTTGTGGATGGGGTTGTGA
>PELPCK_26210 Helix-turn-helix transcriptional regulator
ATGAATATCGCACAGAAATTAAGAGCAATAAGACAATCGGAAGGGCTAACTCAAGCAAAATTCAGTGAAATCAGCGGCATAGCGTTAGGAACGCTAAAGAATTACGAAGGAGGCCATCAA
GACCCTGGCATCCAAGTTGTGTTGCAGGTCACCAATGCACCTCAGTTCCAAAAATACACACTATGGCTCATGACAGATAAAACAGCGCCGGAAGCGGGACAAATCGCACCGGCTCTCGCGC
ACAGTGGGCCAGAGTCAACAGAATCCAACCACTCCGCGAAAAGGATTGGCTAA
>PELPCK_26215 Integrase
ATGGCAATTAAGAAGCTCGATGATGGTCGCTATGAAGTGGACATTAGACCTCGCGGTCGCGACGGAAAACGCATCCGCAGGAAATTTGAAAGAAAAGCTGAAGCACTAGCATTTGAGCGA
TACACAATCGCCAATGCCAGTCAGAAAGAATGGGGAGGCCAGCGAGCAGACCGCCGAACTTTGACAGAATTGCTCGACATCTGGTGGAAATACCACGGGCAAAACCACGAGCATGGGAC
AAAAGAGTTTAATCATCTGCTCAAAACCATCAGCGGCATAGGTGATATACCAGTGAGTCGGATGAACAAAAGGGCTTTGATGGATTATCGCTCCATGCGACTACGTGATGGCATCAGTGCTG
CAACGATAAACCGCGACATGTACCGATTATCCGGCATGTTCACAAAATTAATTCAATTGGATGAATTTTCCGGGCAACACCCAATTCACGGACTGCCGCCACTGACGGAGGCTAACCCTGAA
ATGACGTTCCTGGAAAAAGCAGAAATCGAAAAACTGCTAAATGTTTTGACTGGTGATGACTTACTTGTCGCGCTTTTATGTCTGAGCACTGGAGGAAGGTGGACGGAAGTTGCCACGCTAA
AACCAGCACAGATTACAAGTTGCAGGGTTACCTTCCTGAAAACCAAAAACGGTAAAAAGCGAACAGTGCCGATTTCTGAGGAACTGGAGAAAAAAGTTAAAGAGGAGGCCAGCGCCAA
ATTGTTCAAGGTAGATTATGAGAAGTTTTGCGAGATTTTACGCAGAGTGAAACCTGATATACCTCCCAATCAGGCAACCCACATTCTGCGGCATACATTCGCAAGCCATTTCATGATGAATGG
GGGCAACATAATTGCACTGCAACAGATTCTGGGGCATGCGAGCATTCAGCAGACAATGACCTATGCGCACCTTGCGCCTGACTACCTGCAGAACGCCGTCGCTCTGAATCCACTAAAAGGC
GGAGTGACGTTATAA

Prophage 7.3

>PNPANE_05960 Phage late control D family protein
ATGATTACGGGCATGACCATTGACGCCGGTGCCAGCCTTGCACCGGCATTTATGCTGACGCTGAACAGCCAGGACATTACCAGCAATTTTAGTGACAGGCTGATTTCTCTCACCATGACAGA
TAACCGGGGATTTGAAGCTGACCAGCTCGACATTGAGCTCGACGATACTGACGGAAAAGTCGAGTTACCCCTGCGCGGGGCGGTGCTGACGCTGTGGCTTGGCTGGCAGGGTTCGGCGC
TTCTGAATAAGGGCGATTTCACGGTTGATGAAATTGAGCACCGGGGCGCGCCTGATACTCTGACCATTCGTGCGCGTAGCGCAGACTTTCGCGGCACGCTCAATTCACGACGTGAAGAATC
ATGGCACGATACCACCCTCGGTGAGCTGGTAAGCACCATTGCAAAGCGTAACAAACTGACGGCCAGCGTCGCGGATTCGCTGAAACAAATCCCGGTACCGCATATCGACCAGTCGCAGGAA
TCCGACGCGGTATTTCTTACCCGGCTGGCTGACCGCAACGGGGCGACTGTATCGGTTAAAGCGGGAAAGCTCCTGTTTCTGAAAGCCGGTAGTGCGCTGACGGCCAGCGGTAAGCCCATT
CCACAAATGACGCTGACCCGCAGTGACGGCGATCGTCATCAGTTTGCCATTGCCGACCGCGGAGCTTATACCGGTGTAACAGCTAAATGGTTGCACACCAAAGACCCGAAGCCGCAAAAAC
AGAAAGTGACGCTGAAACGCCAGCCAAAAGATAAGCACCTTCGCGCACTGGAACACCCGAAAGCAAAGCCGGTCAGCAAAAAGACGAAGTCCAGAAAAGAGCCGGAGGCTCACGAAG
GTGAGTATATGGTCGGTGAGGCAGATAACGTGCTGGCACTGACGACGGTCTACGCTTCCAGAGCGCAGGCGATGCGCGCCGCTCAGGCTAAGTGGGATAAGCTGCAGCGAGGCGTTGCG
GAGTTTTCAATTACGCTGGCGCTTGGTAGGGCTGATTTATTCCCTGAGACACCGGTGCGCGTGTCAGGCTTTAAGCGCGTCATAGACGAGCAATCTTGGTTAATCAGTAAGGTGATTCACAA
TCTGAATAATAGTGGCTTCACGACGGGCTTAGAGCTTGAGGTTAAACTCTCTGATGTGGAGTACAGCGCTGACAAAAGCGAGAACTGA
>PNPANE_05965 Phage tail protein

ATGATGCTTGCGCTGGGAATGTTTGTTTTTGAACTCCGTACCCTGCCTTATCAGTCAATGCAGCATTCGAAAGATTACCGCTGGGCGTCTAATGACCGGGTCGGTAAACCGCCTGCATATCAG
TTTCTCGGCGAGGGGGAAACCTCAATACAGCTTGCCGGTACGCTTTACCCTGCCATTACCGGCGGACATATATCCCTGCTGGCTGTGGAACTGATGGCCGATGAGGGCAGGGCGTGGCCGC
TGATTGAGGGGACCGGCAAAATCCTCGGGATGTATATCATCGATAAGGTGTCGACCACGCACGCCGAGTTTTTCAGCGATGGTGCGGCAAGAAAGATTGATTTCACGCTTTCGCTAAAACG
GGCCGATGAATCACTTACGGCAATGTTTGGCGACCTGAATAAACAGGCGAGCGAGCTTCTCGGCTCTGCCGGTAATCTGACCGATAAGCTGCAGGGTGCGCTCGGAGGGCTGACCACATG
A
>PNPANE_05970 putative tape measure protein
ATGAGTAATAACCTGAGGCTTGAGGTATTGCTGAAAGCGGTCGACCAGGCGACCCGACCGCTTAAATCCATCCAGATCGCGAGTAAATCCCTGTCGGGAGATATTCGCGACACACAAAAAG
GGCTGCGTGACCTGAACGGTCAGGCGTCGAAAATCGACGGATTTCGTAAGGCAAGCGCGCAACTGGCCGTGACTGGTCAGGCGCTTGACAAGGCAAAGCGCGAAGCCGGTGAGCTGGC
TGTGCAGTTTAAAAACACCACCAGTCCGACCCGCGCGCAGGCGCAGGCGCTCGACGCGGCAAAGCGTGCCGCCTCTGAGCTGCAGACGAAATATAACAGCCTGAGAACATCGGTACAGC
GCCAGCGCTCCGAGCTGATGCAGGCCGGTATTAATACCCGCACCCTGTCTGCCGATGAGCGTCGGCTCAAAACCTCCATCAGCGAAACGACGGCGCAGCTTAATCGCCAGCGTGAGGCACT
GGCGCGCGTCAGTGCGCAGCAGGCGAAATTAAGCCGGGTGAAAGAACGATATAAATCAGGTAAAGAGCTTGCCGGTAACATGGCCGCAGCAGGCGCTGCCGGGGTCGGTATTGCGACA
GCGGGAACGATGGCCGGGGTTAAATTGCTGATGCCCGGTTATGACTTTGCGCAGAAAAACTCCGAGCTGCAGGCTGTGCTCGGGGTCGATAAGCAGTCGCCAGAAATGCAGGCGTTACG
CAAACAGGCGCGCCAGCTCGGCGACAATACTGCAGCCTCTGCTGACGATGCTGCCAGTGCGCAGATTATTATCGCAAAAGGTGGTGGTGATGCTGAAGCTATAGCAGCAATGACACCTGTG
ACACTTAATCTGTCACTTGCTAACCGGAAAACAATGGAGGAAAACGCTCAACTGCTGATGGGGACAAAAGCCGCCTTTCAGCTCTCTAATGATGCGGCCGCACATATTGGTGATGTTCTCTC
AACCACTATGAATAAAACCACCGCTGATTTTCAGGGGTTAAGTGACTCATTAAGTTACCTTGCTCCTGTTGCGAAGAATGCTGGAGTAAGCCTTGAGCAAGCAGCAGCGATTACCGGGACG
CTGCATGACAACAATATCAGGGGGTCAATGGCTGGGACGGGCGGCGCGGCTGTTATTACGAGATTACAGGCACCCACAGGGAAAGCATACGATGCGCTCAAAGAGTTGGGAGTTAAAACC
TCGGATAGCAAAGGCAATACGCGCCCTCTATTTACCATCCTGAAAGAAATGCAGGCCAGCTTTAAGCGCAACAATCTTGGTACCTCACAAAAGGCCGAGTACGTGAAAACGATATTCGGCGA
GGAGGCCATGAAGTCTGCAAGTGTACTTATGGCGGCAGCAGCAAGCGGAAAACTCGATAAGCTAACTGCCACGATAAAGGATTCAGACGGTAAAACCGAGGAGTTGGTCAAGGTTATGCA
GGATAACCTCGGCGGCGACTTTAAAGAGTTTCAATCCGCTTATGAGGCCGTCGGTACCGACCTCTACGACCAACAAGATAGCTCGTTGCGTCAGCTAACTCAGACAGCAACGCGGTATGTGC
TAAAGCTTGATGACTGGATAAAAAACAACAAGGAGTTAGCGGAAACCATCGGCATCATCGCCGGTGGCGCTCTGGCTCTGATTGGTATCATCGGCGGTATTGGTCTTGTGGCGTGGCCGGT
AGTTATGGGGATTAACGCCATTATCGCTGCTGCTGGCGTGCTGGGTTCCGTCTTTACTGTCACCAGTAGTGCCATTGTGACAGCGCTCGGTGCGATTACCTGGCCGATTGTGGCTGTCGGTGC
GGCGATTGTGGCCGGGGCGCTACTTATCCGTAAATACTGGGAGCCCATCAGCGCATTTTTCTCGGGGGTGATTGAGGGCATCATGAGTGCCTTTGCGCCGGTCGGGGAAATATTTGCGCCA
CTGGCTCCCATCTTTGACGGACTCGGTGAGAAACTGAGCGGCGTCTGGCAGTGGTTTAAAGACCTGATTGCGCCGGTCAAGGCCACGCAGGAGACGCTCGATAGCTGCAAAAATGTCGGC
GTTATATTTGGTCAGGCACTGGCCTCGGCCTTGACGGCTCCGCTCAATGTTTTTAACAAGCTGCGCAGCGGTGTCGACTGGCTTCTCGAAAAGCTCGGCATTATCAACAAAGAGTCAGACA
GCCTGGACCAGACCGCCGCCAGAACCAACGCCGCCACGCAGGGTAATTCCTACATCCCGGCAACCAGCACATATGGCGGCTATCAGGCTTATCAGCCCGTTACCGCACCGGCGGGGCGCTC
TTACATTGACCAGAGCAAAAGCGAATACAACATCACTCTGCCGGGTGGTGTTGCGCCGGGGCATCAGCTTGACCGCCAGTTACGCGACACGCTCGAACAGATTGAACGTGATAAACGTGC
GCGCCAGCGTGCCAGTATGACCCACGATTTCTGA
>PNPANE_05975 Phage tail assembly protein
ATGGCAAAAGCACCACGCAAAACCCCGGAATTTATTGATACGGCTGGCAATGAAACTGACACCGTGAACCCGAACGTCGTGACCCTGGACAAACCGATTAAGCGCGCCGGTCAGACGATT
GATAAAGTCACCCTGATTGAGCCGAGCGCCGGTACCCTGCGCGGCGTCAGTCTGGCAGCGGTGGCGCAGTCCGAAGTCGACGCCCTGATTAAGGTGCTGCCCCGCATGACCTACCCCGCG
CTCACCGCGCAGGAGCTTACCGCGATGAACCTGCCCGATATGTTGTCGCTGGCCGCTAAGGTGATTGGTTTTTTGTCACCGGCTTCGGCGGAATAG
>PNPANE_05980 Bacteriocin serracin-P subunit
ATGGCACTACCCCGTAAACTCAAATATCTGAATATGTTCAACGATGGCCTCAGCTACATGGGAGTTGTTGAATCCGTGACGCTGCCGAAACTGACCCGCAAGCTCGAAAACTATCGCGGTGG
CGGTATGAATGGCGCGGCGTCTATTGACCTCGGCCTCGACGATGATGCGCTCACCGTCGAATGGTCTGTCGGTGGCCTGCCGGATGTGGCTCTGTGGGCGCAGTACGCCGCCCCGGGTGCT
GATGCTGTGCCGCTGCGTTTTGCTGGTTCTTACCAGCGCGACGACACCGGCGAAATCGTGGCGGTCGAGGTGGTCATGCGTGGCCGTCATAAAGAAATCGACGGCGGCGAGAATAAGCAG
GGTGAAAACACCTCGACCAAACTGTCGACTGTCTGCACCTACTACCGCCTCACGATTGATGGCAGCGACGTCATCGAAATCGACACCGTCAACATGGTCGAAAAGGTGAACGGCGTCGACC
GTCTGGAGCAGCACCGCCGCGCAATCGGGCTGTAA
>PNPANE_05985 Phage tail protein
ATGAGTGATTTTCACCACGGCACGCAGGTCATCGAAATTAATGACGGTACGCGTGTTATTTCCACAGTAGCGACTGCAGTCGTTGGCATGGTCTGTACAGCCAGCGATGCAGATGCCACGCT
GTTTCCCCTCAATGAGCCGGTGCTGATTACCAATGTGCAAAGCGCCATTGCGAAAGCCGGTAAAAAAGGCACGCTGGCTGCTTCCCTGCAGGCAATCGCCGACCAGTCAAAGCCTGTCACC
GTTGTCGTGCGTGTTGCTGAAGGTACCGGAGACGACGCAGAAGCGCAGACCACGTCCAACATTATCGGCGGCACGGATGAAAACGGTAAGTACACCGGTATCAAGGCGCTGTTAACTGCC
GAAGCGGTCACCGGTGTTAAGCCGCGCATTCTCGGTGTGCCGGGTCTCGATACGCAGGAGGTTGCAACTGCACTTGCGTCGATCTGTATCAGCCTGCGCGCCTTTGGTTATGTCAGCGCAT
GGGGCTGTAAAACCATTTCCGAGGCGATAGCCTATCGTGAGAATTTCAGTCAGCGTGAGCTGATGGTTATCTGGCCTGATTTCCTCGCGTGGGACACCGTCAAAAATGCCACCGCAACGGC
TTACGCCACTGCACGTGCACTCGGCCTGCGTGCTTACATCGACCAGACTGTCGGCTGGCACAAAACCCTGTCTAACGTTGGTGTACAGGGCGTTACCGGCATCAGCGCCTCAGTGTTTTGG
GATTTGCAGGCATCCGGCACCGATGCTGACCTGCTCAACAAGGCCGGGGTTACGACACTGGTACGCAAGGACGGTTTCCGTTTCTGGGGTAACCGCACCTGCTCAGATGACCCGCTTTTTC
TGTTTGAGAACTACACTCGTACCGCGCAGGTACTGGCCGACACGATGGCTGAGGCGCACATGTGGGCGGTTGATAAGCCCATTACCGCCACGCTCATTCGTGACATTGTTGACGGCATCAAT
GCCAAATTCCGCGAACTGAAAACAAACGGCTACATCGTGGACGGTGAATGCTGGTTCGACGAGGAATCGAACGACAAGGAAACCCTCAAGGCCGGGAAACTGTATATCGACTACGACTAT
ACACCGGTTCCGCCACTGGAAAGCCTGACCCTGCGCCAGCGTATCACCGACAAATATCTGGTGAATCTGGCCGAATCGGTCAACAGCTAA
>PNPANE_05990 Phage tail protein
ATGACAGCGGCAAAAAATAAAAAGAACAAGCAGTTTTTAAATATTAAAAATTTCATTCCGTATACACCGGAACCAGACGACACATTATTCGCCGGTGCGGCGCATCTACAATCAGAGGATGG
TCAGGACTGGTATGCATGCCAGCAATTATTTTCAGAAGACACGCTGAAAATTACCTACGACGATAACGATGTTATTACGTGTATCACGCGCGATATTTCCGGTTTATGGCCTGCAGGCCAGAG
CGTGGCGGAGCTACCTGATACGGATGAAAACCGTCGCGCTGATATTTCAGGCGGCTGGCAGTTTAAAGACGGTAAAGTCGTTCAACGGGTTTATTCGCCGGAAGAGCTGCGTAAAAAGGC
GGAAGCTGAAAAAGTTCGCCGCCTTGCTGAGGCTGAATCAGCCATTGCACCACTGGCGCGGGCAGTAAAACTAAAAATCGCCACAGATGAAGAGATTAAACGGCTGGAAGCATGGGAAC
TCTACAGCGTAATGGTAAACCGGGTGGATACATCTGCGCCTGACTGGCCGGATATACCACGCTAA
>PNPANE_05995 Collar domain-containing protein
ATGAGCACGAAATTTAAAACCGTTATCACCACTGCCGGAGCCGCTAAGCTTGCTGCGGCCACCGTGCCGGGCGGTAAGAAAATAAATCTTAACGTTATGGCTGTTGGTGACGGCGGCGGAA
AACTGCCGATGCCTGATGCCGGTCAGACGCAGCTTGTTAATGAGGTCTGGCGCCACACTCTGAATAAAATCAGCCAGGACAACCGGTACAGTAATTACATTGTGGCCGAGCTGGTTATTCCG
CCGGAAGTAGGTGGCTTCTGGATGCGTGAGCTTGGCCTTTACGACAATGAGGGGACGCTGATTGCTGTTGCCAATATGGCCGAAAGCTACAAGCCAGAACTGGCCGAGGGCTCAGGGCG
TGCGCAGACATGCCGCATGGTCATTATTGTCAGCAGTGTTGAGTCTGTGGCGCTGTCCATTGACTCAACGATGGTGATGGCGACGCAGGATTATGTCGACGACAGGCTCGCCGAACATGAG
AAATCACGTCGTCATCCTGACGCCACACTGAAAGAAAAAGGGTTTACTCAGCTCAGTAACGCGACAGACAGCGAGTCTGAAACACTCGCAGCGACACCGAAAGCTGTTAAGGCAGCATAC
GACCTTGCTGACGGGAAATATACAGCTCAGGACGCCACCACAACGCGAAAAGGCCTTGTGCAACTCAGTAATGCTACTGACAGTGTGTCTGAGACGCTTGCCGCGACACCGAAAGCGGTC
AAGGTGGCCTATGACCTTGCTAACGCAAAATATACAGCTCAGGACGCCACTACAGCGCGTAAAGGGATTATCCAGCTCAGCAATGCCACTGACAGCACGTCTGAGACGCTGGCCGCAACGC
CGAAAGCGGTTAAAGCGGCTATGGATAATGCGAACGGACGCGTCCCGTCAGACCGTAAGGTCAATGGGCATCCATTATCCGGGGATATCACCCTGTGGGCGTCAGATGTGAAGGCTATTTC
CGCAGATGCCATTGGACAGATTACCGATAACGGCACGATGGCATCAGCTAATACTCCAGGATGGTGGCGGGTGGCGGTGTCGAATTCTGATACGGTCGCTGATTTTCCGACCTATCCGGATG
GCAGCAAGCTGTACAGCTACGGATATATGTTTGTTGAGAAAATTGGAGAAGTCTGGTTTCAGCACTATTACGCGCATATGGGCGCGAACGCAAAGCGCCAGGACTGGGGAACGGAACCGA
ATACCAGCCGCCAGTGGGTTATTGACTACAACACCGTAAATAAACCTTCAGCCAGTGATGTGGGTGCATTGCCGATTACCGGGGGGCGGCTTAACGGCCCACTGGGTATTGGTACTGACAAT
GCACTGGGCGGTAATTCGATTGTATTCGGCGATAACGATACAGGGTTTAAGTGGCACAGTGACGGCGTTCTGGGTATTTATGCCAATAGTGCCCAGGTCGGTTATATCGACAATTCCGGGCT
GCACATGCTGGCAGATATTCGCGCTACTGGTGTCGTGCGCACCGGCAACGGAAAAACACTGACGTTATCGAGTGGTAACAATTCTGCACTGAATGCTGGTTTAAGTCTGTGGGGAGGTGGA
GAACGTCCAACAGTCATTGAGCTGAGCGATGAACAGGGATGGCATTTATACAGTCAACGAAATACGGACGGAAGTATTTCATTTACTGTTAACGGTATCGTCTACTGTAACGCGTTAAATATA
GGTGGCGCTATTTATCAGAATAACGGTGATATCTTTGGTTCGTTATGGGGAAATGGCTGGTTAAGTACATGGATTCACAACAATGTAGTAAAAGCGGTCAGACTTGGCCCCGTGGCGCTTTCT

GGCGGTCTGTGGCGTGATTTTCAGCTTGGCGGCGGACAGGTGGTGACGGGGTTCCATACTGACGGTAGCTGGGAAATGGAAGGTAACGATGACAAGGTTTATTACCGTCCCATTCAGTATC
TGGTTGGTGATACGTGGGTAACAGCCCCAAGTGTATAA
>PNPANE_06000 Phage tail protein I
ATGAGTGATTCACGATTGCTGCCGGTGGGCTCATCGCCACTGGAAGTCGCTGCCGCAAGGGCGTGTGCTGAGATTGAAAGGACGCCGGTCAATATCCGCACGTTGTGGGATCCTGACACCT
GCCCGGAAAATTTGCTGCCGTGGCTGGCGTGGGCGTTTTCTGTCGACCGGTGGGATGAGAACTGGCCGGAGGGAACAAAACGTGCCGTTATCCGTGATGCATATTTTATTCACTGCCACAA
GGGGACTATCGGCGCAATTCGTCGGGTAGTGGAGCCACTCGGCTATGTCATCAATGTAACGGAATGGTGGGAAACCAGCGACCCGCCGGGCACATTCCGGCTTGATATCGGGGTGCTTGA
AAGTGGCATTACCGAGGAAATGTATTTCGAAATGGAGCGACTGATTGCAGATGCAAAACCAGCCAGTCGTCATCTGACTGGCCTGAATATTGTCCAGGACATTCCCGGTTATTTGTATACCGG
CGGCGTGGCCTGCGACGGCGATATTATTACGGTTTACCCGGGATAA
>PNPANE_06005 Baseplate-J domain-containing protein
ATGGCAACTGTTGACCTGAGTCTGCTACCTGTTCCTGATGTGGTCGAGGAACTGGACTATGAAACTATCCTTGCGGAGCGCATTGCGACGCTGATTTCGCTCTATCCGGAAGACCAGCAGGA
AGCTGTCGCACGGACGCTCGCGCTTGAGTCTGAGCCAGTTGTTAAATTGCTGCAGGAAAACGCCTACCGTGAGGTTATCTGGCGTCAGCGTGTCAATGAAGCTGCACGCGCAGTGATGCT
GGCTTATGCCATAGACAGTGACCTCGATAATATCGGGGCGAATTTCAATGTTGAGCGTCTTGTCGTCACACCTGCTGATGACACCACCATTCCACCCACCCCGGCAGAAATGGAACTCGACG
CCGATTATCGTCTGCGTATACAACAGGCTTTTGAAGGGCTGAGCGTGGCGGGGTCTGTCGGGTCGTACCAGTATCATGGCCGTAGTGCTGACGGACGCGTCGGCGATATTTCAGTTATCAGC
CCGTCGCCAGCCTGTGTGACGATTTCCGTGTTGTCACGTGAAAACAACGGCGTCGCATCTGAGGAGCTGCTCGCAATTGTGCGCAATGCCCTGAACGCAGAAGATGTCAGGCCGGTCGCT
GACCGGGTGACGGTACAGTCAGCCGAAATTGTTAACTACCAGATTAACGCCACGCTTTATCTTTATCCCGGCCCGGAGAGTGAACCCATCAGGGCGGCGGCTGAGGCAAAGCTGAAAGCC
TATATCAGCGCGCAGCACCGCCTCGGGCGCGATATCCGTAAATCAGCGATTTATGCCGCCCTGCATGTTGAGGGTGTTCAGCGGGTGGAGCTGACGGCACCGGTCGCGGATATTGTTCTCGA
TAACACTCAGGCGTCTTTTTGCACTGACTACAGCCTTGTAATCGGGGGATCTGATGAGTGA
>PNPANE_06010 GPW-gp25 domain-containing protein
ATGACGGCGCGCTATATGGGGATGAACCGCAATACCGGCCTCGCTATCAGTGACAGTGAGCATATCAGCCAGAGCATGCGCGACATTCTGCTGACGCCGGTCGGCTCGCGGGTAATGCGTC
GTGAATATGGCTCGCTCCTGTCTGCGCTGATTGATATGCCGCAAAACCCGGCGCTCAGGCTGCAAATCATGGTGGCGTGCTATTCCGCGATCCAGAAATGGGAACCACGCATCAGGCTTACC
TCAATCAGCTTTGAGCGTGGCGACACTGGCGAAATGTATGTCGATATTACCGGGATGCGTACCGATACCGGTGCGCCAGTTTCAACCACTGTTTCACTGAGTTAA
>PNPANE_06015 Spike protein
ATGAATACGTTATCCACGATACAGGAGCTCGCGCGCGCAATTCGCAACCTCATCCGCTCAGGTGTGGTGACTGAGGTCGATATTGTGCAGGGGCTGTGCCGCGTACAAAGCGGCGGGATCC
AGACTACATGGCTGAACTGGCTGACCACCCGCGCCGGTCGTTCGCGGACATGGTGGGCCCCCTCGGTCGGTGAGCAGGTGCTGCTACTGGCGATTGGGGGCGAGCTTGATACCGCTTTCG
TACTGCCGGGGATTTTTTCCGACGATAACCCAGCCCCGTCAGCCTCGGCGGATGCGTGGCATGTGGTGTTCCCCGACGGCGCTGTTATGGAGTATGAGCCGGAAACCGGTGCACTGACGGT
CAGCGGCATCAAGACTGCCGATGTGACGGCATCGGAGTCCATTACCGCCACCGTGCCGGTGGTACTGGTAAAAGCGGCAGAATGTATCACCCTCGACACCCCGGAGGTGGTATGCACCAAC
AAACTGACGACGGCGACGCTTGAGGTGCAGAAAGGCGGCACCATGCGGGGAAACATCGAACATACCGGTGGCACGTTGAAATCAAACGGCGTACAGGTCGATGACCACGGTCACGGCG
GCGTGCAACGGGGCGGGAGCTGGACGGAGGGCACCAGATGA
>PNPANE_06020 Phage tail protein
ATGAGTGAGTTAACCGCGCTGCAGGAGCGCCTTACCGGTCTGATTGCCAGCCTGTCACCGGCGGCGCGTCGGCAAATGGCGGCTGACATTGCAAAAAAACTGCGCGCCAGTCAGCAGCA
GCGCATCAGGCGACAGCAGGCACCCGACGGCACCCCGTATGCCGCCCGAAAGCGCCAGCCGGTGCGAAGTAAGAAAGGCCGTATCAGGCGCGAGATGTTCGCCAGACTGCGCACTAACC
GCTTTATGAAAGCCAAAGGCAGCGACAGTGCGGCGGTGGTGGAATTTACCGGCAAAGTGCAGCGTATGGCGCGGGTGCATCAGTATGGCCTCAAAGACCGGCCAAACCGCAACAGCCGG
GAGGTGCAGTACGAGGCGCGTCCGCTACTCGGTTTCACCCGCGACGATGAGCAGATGATTGAAGACGTCATTCTCAGTCACCTCGGCAAATAA
>PNPANE_06025 N protein
ATGCAGAAGCACAAAAGCCTGCGCAAGGCGCTGATTAACGCCGTGCCGCAGCTCCGAAACAACCCCGATATGCTGCGCCTGTTCGCTGATAACGGGCATACGGATTCCCGACTGGAGAGC
TCGCTGTCGTTTGAAAAGGTGTACGTGCTTAACGTGGTGGTGACTGACTTTACCGGCGACCTCGATTTGATATTTGTGCCGGTGCAGGCATGGCTGCGTGAGCATCAGCCGGACATTATGAC
CACCGACGACGGGCGGGAGAAAGGATTCACATGGATGATTGATATCAATAACGACGATTCGCTCGATATCAGTATCAGCCTGAGACTCACCGAGCGCACGCTCGTCAAAGAGGTCGACGGC
GCACTGCATGTCAGCTATGCCCCTGAGCCACCGCTGCCTGAGCCGGTGACGCGCCCGGTCGAGCTGTACGTTAACGGCGAGCTGGTGAGTAAGTGGGATGAGTGA
>PNPANE_06030 hypothetical protein
ATGCCGGGAAGTGATCCGAAAACCAATGGCGACCTGAGCGCAGATATCCGCCGTCTTGAGGGCGCGCTGACCGCCTGCGCGCTGCAGGTCAAAACCGTCAAACACTGTCAGGATGAACTC
GATGCAGAAGCACAAAAGCCTGCGCAAGGCGCTGATTAA
>PNPANE_06035 LysB family phage lysis regulatory protein
ATGAAAGTCCTGATAACGCTGTTTGTGCTGGCCGTGCTCGGTCTGATGTGGTTGCGCCATGAGAATGGCAATTTATCCCGCTCCTTTGAGACGGCAAACCGCGTTGCGAGCGAGCAAAAGG
CGACGATTGGTATGCTGAAAAATCAGCTCAGTGTCGCCGGCCAGCTCGCCCGACGTAATGAATCCGCGCAGGTGGCACTGCGCGAACAGCTCGCAAAGGCAGGCGCAGAAGCAAACCGC
CGCGAGCAGACGATAACGAGGTTACTTGATGAAAATGAAGCCTTTCGCCGCTGGTATAACGCTCCTCTGCCTGATGCTGTGCGCAGGCTGCACATCCGCCCCGCCTGCGCCAGCGCCGGTG
ATTGTGGTCAACGGATGCCCGAGGGTGAGCCTTTGCCCGATGCCGGGAAGTGA
>PNPANE_06040 Lysozyme
ATGCCGGTCATTAATACTCACCAGAATATCGCCGCCTTTCTGGACATGCTGGCGTATTCCGAAGGAACGGCGAACCATCCGCTGACGAAAAACCGTGGCTACGACGTCATTGTTACCGGCCT
TGATGGCAGGCCAGAGATTTTCACCGATTACAGCGACCACCCTTTCGCACATGGCCGACCCGCGAAAGTGTTTAATCGCCGTGGCGAGAAATCCACGGCATCGGGGCGTTACCAGCAGCTT
TATATGTTCTGGCCGCACTATAAAAAACAGCTCGCATTGCCTGATTTCAGCCCACTGTCGCAGGACAAGCTCGCGATCCAGTTAATCCGGGAGCGCGGTGCTATTGACGATATCCGGGCGGG
GCGTATTGAGCGTGCTGTTTCCCGTTGCCGGAATATCTGGGCGTCATTGCCGGGTGCCGGTTACGGCCAGCGCGAGCACAGTCTCGAAAAGCTGGTTACCGTCTGGCGCACGGCTGGCGG
GGTGGTGGCATGA
>PNPANE_06045 Holin
ATGACAGAAGGGGAAAAAGGCGTCCTGTCACTGTTTGTGATTGGGGCACTGATTGTGGTCGGAAAAGTGCTGGCAGGTGGTGAGCCCATCACCCCGCGCCTGTTTGTCGGACGCATGTTG
CTCGGCGGTTTTGTCTCAATGGTCGCCGGTGTTGTTCTGGTGCAGTTTCCTGATATGTCACTGCCCGCCGTGTGCGGTATTGGATCCATGCTCGGTATTGCCGGTTATCAGGTGGTGGAAATC
GCCATTCAGCGCCGCTTTAAGTCACAGAAGGGGGAAGGCGATGCCGGTCATTAA
>PNPANE_06050 Tail X family protein
ATGAAGGTCAGGTCGATGCAGGGCGACACCCTCGACGTGATTTGCGCCAGGTATTACGGGCGCACTGAGGGCGTGGTTGAAACGGTGCTGCAGGCTAATCCCGGCCTGTCTGAGCTGGG
CGTCATTCTGCCGCATGGCACGGCAATTGACCTGCCCGATGTGCCGTCTTCACCCGTAACTGAAACTATCAATCTTTGGGAGTAA
>PNPANE_06055 Capsid completion protein
ATGACGACAATAATTCTGAACCAGCCCGACGAACCACAGGACGTACCGGGCGTGGTGATTCCCGTACCGGAGACGGGCGATGCAGTAATTAAAAACACGTTCTTTTTCCCTGATGTGGATC
CGAAGCGTGTGCGCGAGCTGATGCGGCTTGAGCAGACGGTTTCCGATGCGCGCCTGCGCCATGCCATCAAAACCGGCATGGCGGAAACCAATGCGGAGCTTTACGACTACCGGCTGCGCC
AGACTGCCGCCGGGTTTAAGCATCTGGCCGACGTGCCTGCTGAGGAAATCGACGGCGAGAATGTGCGTATTTTCCACTATCTGAGCGCCGTAACGGCGATGGCAACCGCCACCCTGTATGA
GCGCTATCGCGGTGTTGAAGCCACCGGCAAGGGTGACAAAAAAGCCGACAGCGTCGAAACCACCATTGATGACCTGTGGCGGGATATGCGCTGGTCGGTCGCGCGTCTGCAGGACAAAC
CGCGCTGCATCGTGGGCCAGCTCTGA
>PNPANE_06060 M protein
ATGACGAGCCCCGCACAGCGTCACATGATGCGGGTCTCGGCCTCTCAAGCCGCGCAGCGGGAGCAAGCCCCGCTGCGCCATGCAACCGCCTATGAGCAGATGCTGGTTAAGCTGGCCGAT
GACCGCCGCACGTTAAAAAACATCCGTTCAAACGAGCGTAAAGCCGCGAAAAAGCGCGAGCTGCTGCCGTTCTATGCGCCGTGGGTCGCCGGTGTGCTGGCTGATGGCTGCGGTGCGCA
GGATGACATTGTCATGACCGTCATGCTGTGGCGTCTTGATGCCGGTGATATCGCTGGCGCGCTGGAAATTGCGCCCTACGCGTTGCAATACGGCCTCACCACTGACCATCGCCGCACGACAC
CTTACATGCTGGTTGAGGAGGTGGCGCTTGCCGCACTGCGTCTGCGCGATGCCGGTGAATCTGTCGACCTTTCCTGGCTGCAGACCACTATCGACCTGACCGACGGCGCTGACGTTCCCGA

TATGGTGCGTGCCCGTCTGCATAAGGTGACAGGCCTGACCCTGCGTGATGCCGGTATGAATGCAGAGGCGCTGGCGCAGTTTCAGCGCGCGATGCAGCTCGACCGCAATGCCGGTGTGCG
CAAAGAGATTGAGCGGCTGGAACGCGCACTGAAGCCAAAAGCGGAGGCACCCCCCCGTAAAACGACTAAACCGCGTACGCGCAAACCTGTCGCCAGACCGGCAGCAAAGCGCGGGCGT
CCACCAAAGGCGGTAAAAACCGCCGGTTAA
>PNPANE_06065 Phage major capsid protein, P2 family
ATGCGCCCGGAAACCCGTTTTAAGTTTAATGCCTATCTGACCCGCGTCGCTGAGCTGAACGGCATCAGCACTGATGACGTCAGTAAAAAATTCACCGTCGAGCCGTCCGTCACGCAAACGCT
GATGAACAAAGTGCAGGAGTCATCCGCGTTTCTGCAGACGATTAATATTCTGCCGGTCGCAGAAATGAAGGGTGAGAAAATCGGCGTCGGTGTGACCGGTACTATCGCCAGCACGACTGAT
ACCTCGGGCGATGATGAGCGTAAGACCGCAGACTTCACCGCGCTTGAATCCAACAAGTACGAGTGCGACCAGATTAACTTTGACTTCCACCTGAAATATAAAACCCTCGACCTGTGGGCGC
GTTTTCAGGACTTCCAGCGCCGCATCCGCGACGCCATTGTCAAGCGTCAGGCGCTCGATTTCATCATGGCCGGTTTTAACGGTACCACCCGCGCCGCCACCTCTGACCGCACCAAAAATCCG
ATGCTGCAGGATGTGGCCGTCGGCTGGCTGCAGAAATACCGCAATGAAGCCCCGACGCGTGTGATGAGCAATATCACCGATGCTGACGGTAAGGTCGTTTCGGCAGTGATTCGCGTCGGTC
GAAACGGCGACTATGAGAACCTCGACGCGCTGGTGATGGATGCGACCAATAACCTGATTGACGAGGTTTATCAGGATGACCCGAAACTCGTTGCCATCGTTGGCCGTAAGCTGCTGGCCGA
CAAATATTTCCCGCTGGTGAACAAGCCGCAGGAAAACAGCGAGGCGCTCGCGGCAGATATCATCATCAGCCAGAAGCGAATCGGCAACCTGCCTGCTGTGCGCGTGCCGTACTTCCCGGC
GAATGCCGTACTGGTAACTACTCTGGAAAACCTCTCTATCTATTTCATGGATGAGAGCCACCGCCGCAGCATTGATGAAAACCCGAAAAAAGACCGCGTTGAAAACTACGAGTCGATGAATA
TCGACTATGTGGTCGAGGCGTATGCCGCCGGGTGCCTGCTGGAAAACATCACCCTGGGCGATTTCACCGCACCTGCAGCACCGGAAAGCGGAGCCTAA
>PNPANE_06070 GPO family capsid scaffolding protein
ATGGCAAAAAAAGTCTCAAAATTCTTTCGCATCGGCGTCGAGGGTGATACCTGCGACGGGCGCATTATCAGCGCCAGCGATATTCAGGAAATGGCCGAAACCTATGACCCGCGCGTCTACG
GTTGCCGTATCAACCTTGAACACATTCGCGGCCTTTTGCCCGACGGCATGTTTAAACGTTATGGCGATGTGGTTGAGCTGAAAGCCGAAAAGATTGACGACGATTCTGCGCTGAATGGCAA
ATGGGCGTTGTTCGCCAGAATCACCCCGACCGATGACCTTATCGCGATGAATAAAGCCGCGCAGAAGGTCTATACCTCTATGGAAATTCAGCCGAATTTTGGTAACAGCGGCAAATGCTATCT
TGTCGGCCTTGCGGTCACTGATGACCCTGCGAGCCTCGGTACTGAATACCTCGAATTCTGCCGCAAGGCGAAGCACAACCCGCTGCAGCGCTTTAAGGCCAGTCCTGAAAATGTCTTTTCA
GTCGCCACGCTGGCCGAACTGGAATTTGAAGACGTTCCCGACACGGTGCTCAACAGCCTGGCCGACAAGGTGAAAGCCATTTTCAGCCGTAAACAGGTCAGCGACGATGCGCGCCTGAAT
GATGTGCATGAAGCGGTTACCACCGTCAGCGAACATGTGCAGACCAGCCTCACTGCGCAGGATAAGCGTCTTTCCGATATGGAAACCGCGCTAGCCACCTTTAGACAGGAACTGACCGGCA
AGGTTGAAGAAACCAGCCAGGCATTTTCCGCCCTGAAAACCACCCTCGACAAAACCGAAAGTTTCAGCCAGCCGCGACGCACGAAAGCCAGCGGCGGCGGTGGCGATGAGCTGCTGAC
CGACTGCTGA
>PNPANE_06075 Terminase, ATPase subunit
ATGACCATTTCCACCGATACAACCTTATTGCATGACCCGCGACGACAGGCATCGCTGCTTTACTGGCAGGGCTTTTCCGTGCCACAGATTGCCGAAATGCTGCAGGTCAAGCGCCCGACCGT
GCAAAGCTGGAAACAGCGCGACGGCTGGGACGGCATCGCACCGATTTCCCGCGTCGAAAGCAGCCTTGAGGCCAGGCTGATTCAGCTCATCGCCAAGCCGCAAAAGTCAGGCGGCGAC
TTCAAAGAGATTGACCTGCTCGGGCGGCAGATTGAGCGACTGGCGCGCGTCAACCGCTACAGCCAGACCGGCAACGAGGCCGACCTTAACCCCAACGTTGCCAACCGTAACAAGGGGGA
GCGTAAGAGGCCGAAAAAGAACTTTTTCAGCGATGAGGCTGTCGCAAAGCTGGAAGAAATTTTCTTCGACCAGTCTTTCGAATACCAGTTGCAGTGGTACCGGGCAGGACTGGCGCACCG
TATTCGCGATATTCTCAAATCCCGCCAGATTGGCGCGACGTTCTACTTTTCCCGCGAGGCACTGCTGCGCGCGCTCAAGACCGGCCATAACCAGATTTTTCTGTCGGCCAGTAAAACGCAGG
CTTACGTGTTCCGGGAATACATTATCCAGTTTGCGCGACTGGTTGACGTCGACCTGACTGGCGACCCGATTGTCATCGGCAACAACGGCGCAAAGCTGATTTTTCTCGGCACCAATTCCAAC
ACCGCGCAGAGCCATAACGGCGACCTGTATGTCGATGAAATATTCTGGATCCCGAATTTTCAGAAGCTGCGCAAAGTCGCATCGGGCATGGCCTCGCAAAAGCATCTGCGCTCAACTTACTT
TTCGACACCTTCCACGCTGGCGCACGGCGCTTACCCCTTCTGGTCTGGCGAGCTGTTCAACAAGGGGCGCGCCAGTGCCGCTGACCGCATCGAAATCGACATCAGTCACAGCGCGCTCGCC
GGTGGGCTTCTTTGCGCTGACGGACAGTGGCGGCAGATTGTCACTATTGAGGACGCCCTTGCCGGTGGCTGCACCCTGTTCGACCTCGACCAGCTCAGACGCGAAAACAGTGATGAGGAC
TTTAAGAACCTGTTTATGTGCGAGTTTGTCGACGATAAGGCATCGGTATTCCCGTTCGAGGAGCTGCAGCGCTGCATGGTCGACGTGATGGAAACATGGGAGGACTTCGCCCCGTTCGCCG
ACCATCCATTCGGCTCGCGCCCGGTCTGGATTGGCTACGACCCGTCCCACACCGGCGACAGTGCCGGATGTGTCGTACTCGCGCCGCCGGTGGTTTCGGGTGGCAAGTTTCGCATGCTGGA
GCGTCACCAGTGGAAAGGCATGGACTTTGCCGCACAGGCAGAAGGCATCCGCAGGCTCACTGAGAAATACAACGTCGAATACATCGGCATTGACGCAACAGGCCTCGGTCTCGGTGTATT
CCAGTTGGTGCGCTCATTCTACCCGGCGGCACGCGGTATCCGTTACACGCCAGAAATGAAAACCGCAATGGTGCTTAAGGCGAAAGACACGATTCGCCGGGGCTGTCTGGAGTACGACGC
CGGAGCAACCGACGTCACACAGTCGTTTATGTCCATCCGCAAAACCATGACCAGCAGCGGGCGCAGCGCCACCTATGAGGCCAGTCGCACCGAGGAAGCCAGTCACGCTGATATCGCATG
GGCCACCATGCACGCCCTGTTAAACGAACCGCTTTCTGCCGGTAGCGGCATGCAGCCTAAATCCATTCTGGAGATTAACCAATGA
>PNPANE_06080 Terminase-6 domain-containing protein
ATGAAAAATAATGTTTTCTCACAAAGCCAGATTCAGGCAATGGCCGATATTCTGCACAATGACAGTTTTGACTATCAGGCAACATGGTTGCGGGTCGGAAAACTCAATATCGACCGCAGCATT
ACTAAATCGCGCCAGATTGGCGCAACGCTGCTATTTAGTCGTGAGGCACTGCTCGATGCGCTGACAACGGGCGATAATCAAATCTGGTTTGCTCATACAGTTGAGCATGCGCGCGTGGCGCT
GATGTACATGAACAATCTTTCAGCACGCGTCGGTGTCCGTCTGGCGAGCAACGGTTACAGCGTACAGCTTGACAGCGGTGCGACCATCAACCTTGTCGGCGAGGAATCCCATTGCGCCGCG
CTGGCGGGTAATGTCTACCTTGATGAGTTCGGATGGTTCAATAACCCGCTAAGAGCGGCAAAAGTCGCAGCGGCTATAGCCTGCCATAATCGCCACAGCCTGACGATGTTCACCTCTCCCTCT
GATAATTATGACGCTTTCCGGGTGTGGAACGGCACGTTCCGCAGGCATCGACCGACACCGCTAATCAATACCGGCGACAGCGTATTCTGTACAGATGGCGTCTGGCGCCAGTCGGTCACACT
GGATGCAGCATGCCAGCGCGGGTGCAATCTCTTTGCGCCTGAGGAAATTAAACACGAATACAGCGACGATGATTATCGCATGTTATTTGGTTGCGACTGGTCTTTCGCTGTTGCAGCGGGCG
AGGTGGCAGCATGA
>PNPANE_06085 Phage portal protein
ATGAGCAAGCGCAAGCCACGCAAAGCAGTCGCCATGACCGCCAGTGCACCGCAAAAAATGGAGGCGTTCACCTTCGGTGAGCCGGTGCCGGTGCTCGATAAGCGCGATATTCTGGATTAC
GTCGAGTGCATCAGTAACGGCAAATGGTACGAGCCGCCGGTCAGCTTCTCCGGGCTGGCAAAAAGCCTACGCTCTGCAGTGCATCACAGCTCACCGATTTACGTTAAACGCAACGTGCTCG
CGAGCACCTACATTCCGCACCCGCTGCTGTCCCGTCAGGATTTCAGCCGCTTTGCGCTCGACTATCTGGTATTCGGTAACGCCTTTCTTGAGCAGCGCCACAGCGTCACCGGACAGTTAATCA
AACTGCTGACTTCACCGGCAAAATACACCCGGCGCGGGGTCGATGACTCGGTTTTCTGGTTTGTGGAAAACTTCACTCAGCCGCATGAGTTCGCACCCGATACCGTGTTCCACCTGCTGGA
GCCTGATATTAATCAGGAGATTTACGGCCTGCCTGAATATCTCAGCGCGCTTAATTCCGCATGGCTGAATGAATCCGCGACGCTGTTCCGCCGCAAGTATTACCAGAACGGCGCGCACGCAG
GTTACATCATGTATGTGACTGACCCGGCGCAAAGCGCGACTGACGTCGAATCGCTGCGCGATGCAATGCGTAACTCTAAAGGGCTCGGCAACTTTAAAAACCTGTTTTTCTACTCACCGAAC
GGGAAACCGGACGGCATAAAAATCGTGCCATTGAGCGAAGTCGCCACAAAGGATGACTTTTTTAACATCAAGAAAGCCAGCGCTGCCGACCTGATGGATGCGCACCGCGTACCGTTCCAG
CTCATGGGCGGCAAGCCTGAGAATATTGGCTCAATGGGTGACGTTGAGAAAGTGGCAAAGGTCTTTGTGCGTAACGAGTTATCGCCGCTACAGGACAGGTTCAGGGAGGTAAACGACTG
GCTCGGCATGGAGGTCATCAGGTTCAAAGAGTACACCCTCGACAACCCGGAATAA
>PNPANE_06090 hypothetical protein
ATGTTATCTCTCGTTTATGAAAATCCGTGGACAACCGTTTTTCTGCTGATTGTTGCCAGTTGTTGTCTCAACAGTATTATTGGCGCATTGCGCGGCCAGTAA
>PNPANE_06095 Transcriptional regulator
ATGCAAAATCCCATTTCATCGAAAAAAGACCCGTTTGAAGATTGGGCAAAGAATCTGACTTTAATGGCATTAAATAACGACCTGAGCTCTCGCGAGGTGGAAAGCTACACCGCAAAAATGG
TCGAAAAGGCTAATAAAGATGAGCTTTCAGTCGTTATCAAACACCTGTTAAATCACATCAGAATGCGCAAATAA
>PNPANE_06100 hypothetical protein
ATGCATCGACTACCGGGCGAAATTCCGCAGCACAAAACTAAAAGCATAAAACTTATGGCTATTGTTCATCGTCTGCAGACGATAATGGTCAATGAGAACCTGACTCCAGCAGAGTTGGTCGG
GTGTGCAGAAATCGTCAGGGATAACTATGGCAAACTGGATAATATCAGCAGACCGGCACATTACGCACCGCCACCACGTCGACCATAG
>PNPANE_06105 DinI family protein
ATGAGAATCAATATCACATTGGATAAAGAGCAAAAAATTAGTCAGGCAACGTTGGATGCACTTGAGGCTGAGCTGTACCGCAATCTTCAACCTATTTACCCAAAGACTGCTATCCGCATTCGC
AAGGGCTCCGCAAATGGTGTTGAGCTAAGCGGTTTGAAACTGGACGAAGATAAAAAAAGAGTAATGGAAATCATGCAGCAGGTATGGGAGGACGATAGCTGGCTGCATTAA
>PNPANE_06110 TumA
ATGGTTGGCGAACATTTCAGCCGAACGCAGCAAAAGTGGGCTTGTGTGCAATTTATCGCCGAGGTATCTCTGATTGCAAACTGCAAGCCATCAGACTTAAAGCTCGCGCTCACTCTCATTGC
AGACCTAGCAAACAGCGAAAATAACGAAACCGAAGATGATATTTTTTATAAAGCTGATTAG

>PNPANE_06115 Replication endonuclease
ATGACGAAATATACGTTTGCATACCCGTGGAATGCTCCACGGTCGGCAATAGCCAGCCCATATCTTACTTATGACCAACAGCATCGCCGCGACCGTATGTTTGCGGCTTTGCTACATGCGAGA
AAAGTGCTTTCTCTCCAGCCCGAGTGCGTGCGTTTTGACGTTTATCGCACCGCTGCTGTGCTGGAGCAAAATCAGGGCAGTCAACGAGCCAATGCCTTTTTAATCAGCTTCTGCAAAAAGG
CATTGCCACGTCTTGAACTGGTCGCAAAAAAATACGAGTGCACGGGTATCAACAGCAATGTATCAACCGCTGTTTTTGATGGTCATTTTGATACCCAGCTTATGCAATATCTGGCGTCACGGAT
GGTTAATATGGTTGCCAGATATAACCGCCTCCCTGATATGTCGCGCGCCGATATTGACTTGCTGGCTGCTGATATCGCTAATTTCATTCATGCTGAACTGGCTGACATTGATGACACCGGATTTA
GCGAGCTTAAAACGCTGTACGCCTGGTACATGCGCGCTGGGATTATTTCCCTGCAATTCAACGTTACCCCGCCGCACTGGGAGCGGGTGATAAAGAAATATGTTGGCGAAGATGAAATCGC
CCCGGCTATCGCCCGAATGTTTAACGATGTGTGGTGGCGTGGCCGCCTGCGTCGCATTGCGGCTGCATGGCGCGAACATCTGCAAATTGCTGTTGGCAACGTCAGTAAGAAAAAGCATGCC
TACGCGAGTAAAAGCTGCGTGACTGACTGGCGCGAGCAGAAGCGCCGCACACGCGAATTTCTCAAGGGACTGGATCTCGAAGACGAATATGGTAATCGCATCAGCCTGATTGAAAAATTC
GACGGCTCGGTCGCTAATCCAGCAATACGCCGCTGCGAGCTGATGGCTCGTATCCGTGGGTTTGAAAATATCTGTAATGAGCTCGGTTATGTCGGGGAGTTTTATACTCTGACTGCACCGTCT
AAATATCACGCTACCACCAAAGCGGGCTACCGTAACAGCAAATGGAACGGAGCAAGCCCGTCAGACACGCAGAGCTATCTCACCGGTCTTTGGGCGCGTATTCGCGCCAAGCTACACCGA
GAAGAAATCCGCATTTTTGGCATACGTGTTGCCGAGCCTCATCACGACGGAACGCCGCATTGGCACATGCTTATGTTCATGTTGCCGGAAGACGTCGAGCGTGTGCGCCTCATCATCCGTGA
TTATGCGTGGGAGGAAGACCGCCACGAACTGAGAAGCGATAAAGCCAAAAAAGCGCGCTTTCATGCCGAGGCCATTGACCCGGAAAAAGGCAGCGCTACCGGCTATGTTGCTAAATACAT
TTCGAAAAAATATCGACGGCTATGCTCTTGATGGTGAAACCGATGACGAAAGCGGTGAGCTGCTGAAAGAGACAGCCCCCGCTGTTTCTGCATGGGCGGCGCGCTGGCACATCCGTCAGTTT
CAGTTTATTGGCGGTGCGCCGGTGACGGTATACAGGGAACTACGCAGAATGGCTGACCCTGAAACAGCCAGGGCGCTTAGTGTTGAATTCGCCGCCGTGCATGATGCAGCCCACTATGGCC
GCTGGGCTGATTATGTGAATGCTCAAGGCGGACCATTCGTTCGCCGTGACGATTTACAAGTACGTACATTGTATGAGCCTCGAACTGAATTTAATCAGTATGGCGAAGAAACTGTGTGTATCA
AAGGTGTCTACGATGCCTCGATAGGTGCTGGCTCTCCTATTCTAACCCGGCTTACGCAGTGGAAAATTGTTCCGAAGCGTGCCGTTGATTTGGCCGTTGACGTTAAGGGCGCTCCTGCGCCC
TCTCGGAGTTCTGTCAATAACTGTACGGGAAGCGAAAGCGATCCACCGGAACTCGATTTATCTAAACCCCTAAGTCGACGTGAAAAACGAGAGCTGACAAACCGACTCAGGAAGCAAAAG
CCAGCAATACGGCGAAAATTTATCCACGGAACGGATGAGCAAAACGCAGCTATAGCGAAAACTATCGACGAGATACATCTGACTACCGGCATCACAATCAGCCGGGGCGAAGCCCTGCACC
TGATGGCCGGTGGTAAAAGTTGTTTTGATGGCAAATGGCTACGCGGAACGGCCAAAGGTGAAATATTTTCCGCAGCGCCATCGCATCAGGCTAAAGCCAGGAAAATCCTTAATCGTGTTGC
TGCGATGGCTGAAGCAGCAAAAACCAATATCTGA
>PNPANE_06120 Oxidoreductase
ATGGTTAAGGGCGTGGCAGATGTTACTCATTACCACGGAACACCTGTATGGGGTGACGCCGGTAATGTTCACCGTATTGCGGTCACCGGTGCTGGCGCATTTGTATCGTATGCACGTCCTGAC
CAATTAGCTGCATCAATTAAATACGCCCTCTCTGTTGCTATAGATAATGGCGCTTTTTCAGCGTGGAAGCGTGGCCTTGTTATTGACTGGCAGCAATTCTATCAATGGCTGATTCCTTATTATCAC
CATCCCAAGCTGTCATTTTTTGTTATCCCTGATGTGGTTGAAGGTGGCGAAGCTGACAATGACGCACTTATCGCCAAATTACCACGCTGTTTCAAAGACAAAGCTGCGCCAGTCTGGCATTTG
CATGAATCATTGCACCGTCTGGTTGAGCTTTGCCGGGAGTGGCCGCGTGTGTGCTTTGGGTCGTCAGGTGAGTACGCAACTATCAGAACCCAGCTCTGGCATCGCAGGATGTCAGAAGCGT
TTGAAACCATTTACTGCAAACATAGTTTCTCAACGCAGGTGCACGGGCTCCGCATGCTCGATGGTAGGGTTTTGGGTAACTATCCTTTGGCGACTGCCGATAGCACAAACCTTGCCTGTAATG
TGCCGAAATTCGAAGTCAAATACCCGGAGCTCACAAAAGCAATTCGCGAAGCCGATTATGCGAAAAACCTTTCTGAAGATGAACTTAAAGCCGTCATTCTCAAACGCCGTTGCGCCATTCTG
AAAAATACTATTGAGGCCGTATCCCCTCCATCGATTGAGAATTGGCTATCAAAAGGGTTGGCTCCGCTTCAACTGGAGTTGGCTATCGCATGA
>PNPANE_06125 Uncharacterized protein in GpA 5'region
ATGAGTATTCGTATCGAAGTTGGCGACAAATGGGTTGTTACCAGCGACCAATATCAATTCATCCTGAATGAAAAGAAAATCGCTCAATCAGGGAAGAAAGCTGGCGAGGAATGGCTCGACA
CCATCGGCTATTACCCGAAGATTAACCAGCTTATTTCCGGTCTGGCACATCACTACATCCATAACTCGGAGCTTCAAACTCTGAATGACATTGCCTCAGAAATTGAGAGGTTGGGGATGTTATG
TGAGTCAGCCTTTCATACTGGGATTGCGAAATAA
>PNPANE_06130 hypothetical protein
ATGGCCGACGCAATGGATTTAGCACAACAGCGCGAGCAGGAAGACCGCGAGCGCCACATCAGCAACGCGCGCAGCCGTATCGCTGCGCCTTCCCGTTTTCTTTGCGAAGAATGTGACGCA
CCAATCCCGGAAGCTCGCCGCATTGCGATACCGGGCGTGGCCTTTTGCGTGACCTGTCAGCAAATAGCAGAGCTCAAATCTAAACATTACAGGGGAGTTTAA
>PNPANE_06135 DUF2732 domain-containing protein
ATGCGTAATACCGAAATCCGTAGTTTTAACACTAATAGTGATGCGCTGGCCGTATTGCTGACCGATGCAAAAAAAGAAGAGCGTAAAGACCGCGCGCTCGCTGTTTCCATCCGCCTTGAGGC
GCTGGCTATCCATATCACCAAAGAGGGTATGAGTGGCACCGAAGCTGCCGAACTGCTGCGTCGTGAAGCAATCCGCTTTGAGAATGAATCACAGGAGCTGCACTAA
>PNPANE_06140 Protein dhr
ATGTCACGCGATGAATTAAGGATTGTTTTAGGTGCCATGATTCCGAACATGGAAGACGGTTTTGAAATTAAAACCCGCGATGGGATAATTTTACGAGTTGACCCGGAATGGGAGTGCTGCAA
GCAGTTTAAAGATAGTCTGAAAGCTGAAATTATCAGCCAATTGAAAAGTAAACCAGCCATCGTATTTGGTTATAGCTAA
>PNPANE_06145 Protein fil
ATGCTGAAAAATGAACCGTCATTCGCGTCTCTGCTCGTCAAGCAAAGCCCAGGCATGCACTACGGCCACGGCTGGATCGCAGGTAAGGACGGCAAGCGCTGGCACCCGAGCCACTCACAG
TCCGAATTATTAAAAGGGCTGAAAACAAAGCCGCCGAAATCGTTAGACTTTTTAATTATTCGTATTGTCCACTTTATTATTAAAGGGGTTAAACATGTCACGCGATGA
>PNPANE_06150 Regulatory protein CII
ATGTTAGATTTTCGTGTTTCGTCACATGCGCACTTTGACGAGGCCTGCAGAAAATTCGCAGCTACTCATAACGTGAAAGAACTAGCGAATAAAGCCGGAATCAAGCCGCATACGCTTTACAA
CAAACTCAACCCAGAACAGCCGCACCAGTTAACGCCGCGTGAAATCTGGACGCTGACAGACATGACCGAAGACTCGACCCTCGTCGATGGTTTTCTGGCGCAGATCCATTGTCTGCCATGC
GTACCGGTCAACGAACTGGCAAAAGAGAAACTGCAATCCTACGTCATGCACGCAATGAGTGAACTTGGCGAACTGGCAAGCGGTGCAGTTTCCGGCGACCGCCTCACGCCAGCCAAAAA
ACAAAACATGATTGCTAGCGTAAATGCGGGGATTCGCATGTTGTCATTATCGGCAATGGCGCTGCATGCACGTCTGCAGACTAATCCCGCTATGTCGAGCGTAGTCGATACTATGAGCGGTAT
TGGCGCATCGTTTGGTCTGATTTGA
>PNPANE_06155 Cro/Cl family transcriptional regulator
ATGTCAGATGCAAAATCAATCCCGCTGCATGACGCACAAAACTTACAAAATCAAACTGTATTGCTAGACGCTGGTCAGTTCAACGCGCTTGTAACCATGATGCAGCTATCCATGCAAAACATG
ATTCGTACAGCGATGTTAGACACCATGTCAGTAAAAGACTTCGCCGCCGCCCGTGGCGTTAGCGAGCGTCTGGTCTGGCAATGGATTGATGAGGGCGTCCTTCTCAAAGCTCCCACCAAAG
ATGTTACCAGCAAAGAAAAAGCCGCTAAGCGTAGCCGCACCCTTATCAACGTCAAAGCATGGCGTGACAAACTGACCCAGCAAGCTATCGATTGCCGTTATATCAACTAA
>PNPANE_06160 hypothetical protein
ATGTCTGTAATGTTGCATTCAAATTCAGCAGACTTATTCGATAGTCTAACTTTCCCACCCGGCAATCTGATTACGTCAAAAACATCAAGAGCACCATCAATACCAATAAGCCAGCGGCCATTTCC
TATTTTTGAGCTGGAGCAGTCGACAAGCCAAGATACACTCACGCCATCAATAAACACTAAATCATCAGTGTTTGCCGGAACCATAGAGACATCAGGAGTCCAATGCCCGATATCTTTGAGCTC
GCCAGCTTCAAGGCGGCATTTCCTGATGGTTAAAGCATTAGACGGTTCCCCATCTTTGCTTTTACGCATCTGGCCTTTTCCCGTAGACAACCATCCTAACGAAACACCAGTATCGAGGGCGCA
GGTCACAACCACGTCACCAGGGAAAAAATCGCGTCTAACCCAAGTACTTATAGTGCCAGAAGATATGCCTAATAAATCCCCAAGCTCTTTTTGCATCGTAAAACCATAG
>PNPANE_06165 DUF2511 domain-containing protein
ATGAAACGTAAACTTTTTTTAACTCTGCTTCTTACTCTTTCGTTTGGTGTCGCTGCTGCTGAAAAAACCAAAGAGATAGACGGATCTACCTATGGCGATAAGTGGCCTCTAACCTTTGAAAAG
GCGAAAGTATCATGTGTTAACCGTGCTTATGCTTTTGTGTACGACATTAAAACTGATGACAGATATCCATTGAATGGCATGGCTGTTGATGCTGTTAAGTCAGGAAAGATGGAAGGATCTAAC
TTAGATGACGTATGGAAGGATGACCCTGATTATGACGGTGTTAAAATTTCCATTTCACCGGTGATTGATGCCGCTACAGCCCTTTGTAATTAA
>PNPANE_06170 Tyr recombinase domain-containing protein
ATGACTGTAAGTAAACAAAAAAGTGGCAAATGGTTATGTGAACTCTATCCAAATGGTCGAGAAGGGCGGCGTATACGTCGGCAATTCAATACCAAAGGTGAGGCCGAGGCATTCGAAGCAT
TTACCAAAAGCGAGAGTGAAGATAAGCCGTGGCTCGGCAAGAAAGAAGACCGCCGACGCTTAAGCGAAATTATTCAGCTTTGGCACAATTTGCACGGTCAAGCGTTAGTCGCCAGTAAGT
CGCGGTTAGCTAAGCTTCAAATTGTATGTAACGGGTTGGGCGACCCTATTGCATCTCGTCTTACCGCTAAAGATTGGGCTCATTACCGCGACCGTCGATTACGTGGCGAAATAGACAACGGCT
ATCACAAAGACCCGGCGAAGTGGATCGCCAAACCTATAACAGTCAATCGCGAGCAGCAATACCTCGAAGCGGTGTTCAATGAACTGCGACGATTAGGAGAGTGGAGTTTACCCAATCCACT
GGACGGGATTCGCGTATTCAAAGAAGCTGAGAAAGAAATGTCGTGGCTAACTTTGTCTCAACTCGCGGAGCTGTTCCGAGCCTGTGAACAATATGGCAAAGAAAATCTTACGATGATTGTT
AAGGTGTGCCTAGCTACTGGCGCACGATGGGGAGAAGCAGAGAGACTTACACGCCCCCAACTTTCTCCATACAAGCTGACCTTCACCAAAACCAAAGGTAAGAAGAATCGCACGGTTCCG

ATTCCTAAATGGCTTTACGACGAGTTATCCGAACGTCAGGGCAGAATGTTTAAGCCCTGCTATCAGGAGTTCAAGAAGATGCTCAAACTAACGAACATTGAGCTGACGGAAGGGCAGAAGA
CTCACGTTTTGCGTCATACTTTTGGTGCGCATTTTATGATGAACGGCGGAAATATACTGGTGTTACAGAAAATTCTCGGACACGCCAATATTCGAGAAACAATGAAGTATGCGCACTTTGCTCC
TGACCACCTTGAACAAGCTGTAACTCTCAATCCGTTATCGCTGTATGTTGGCGACAATATGGCGGCAGAGGTTGCATAA

Prophage 7.3

>PNPANE_05960 Phage late control D family protein
ATGATTACGGGCATGACCATTGACGCCGGTGCCAGCCTTGCACCGGCATTTATGCTGACGCTGAACAGCCAGGACATTACCAGCAATTTTAGTGACAGGCTGATTTCTCTCACCATGACAGA
TAACCGGGGATTTGAAGCTGACCAGCTCGACATTGAGCTCGACGATACTGACGGAAAAGTCGAGTTACCCCTGCGCGGGGCGGTGCTGACGCTGTGGCTTGGCTGGCAGGGTTCGGCGC
TTCTGAATAAGGGCGATTTCACGGTTGATGAAATTGAGCACCGGGGCGCGCCTGATACTCTGACCATTCGTGCGCGTAGCGCAGACTTTCGCGGCACGCTCAATTCACGACGTGAAGAATC
ATGGCACGATACCACCCTCGGTGAGCTGGTAAGCACCATTGCAAAGCGTAACAAACTGACGGCCAGCGTCGCGGATTCGCTGAAACAAATCCCGGTACCGCATATCGACCAGTCGCAGGAA
TCCGACGCGGTATTTCTTACCCGGCTGGCTGACCGCAACGGGGCGACTGTATCGGTTAAAGCGGGAAAGCTCCTGTTTCTGAAAGCCGGTAGTGCGCTGACGGCCAGCGGTAAGCCCATT
CCACAAATGACGCTGACCCGCAGTGACGGCGATCGTCATCAGTTTGCCATTGCCGACCGCGGAGCTTATACCGGTGTAACAGCTAAATGGTTGCACACCAAAGACCCGAAGCCGCAAAAAC
AGAAAGTGACGCTGAAACGCCAGCCAAAAGATAAGCACCTTCGCGCACTGGAACACCCGAAAGCAAAGCCGGTCAGCAAAAAGACGAAGTCCAGAAAAGAGCCGGAGGCTCACGAAG
GTGAGTATATGGTCGGTGAGGCAGATAACGTGCTGGCACTGACGACGGTCTACGCTTCCAGAGCGCAGGCGATGCGCGCCGCTCAGGCTAAGTGGGATAAGCTGCAGCGAGGCGTTGCG
GAGTTTTCAATTACGCTGGCGCTTGGTAGGGCTGATTTATTCCCTGAGACACCGGTGCGCGTGTCAGGCTTTAAGCGCGTCATAGACGAGCAATCTTGGTTAATCAGTAAGGTGATTCACAA
TCTGAATAATAGTGGCTTCACGACGGGCTTAGAGCTTGAGGTTAAACTCTCTGATGTGGAGTACAGCGCTGACAAAAGCGAGAACTGA
>PNPANE_05965 Phage tail protein
ATGATGCTTGCGCTGGGAATGTTTGTTTTTGAACTCCGTACCCTGCCTTATCAGTCAATGCAGCATTCGAAAGATTACCGCTGGGCGTCTAATGACCGGGTCGGTAAACCGCCTGCATATCAG
TTTCTCGGCGAGGGGGAAACCTCAATACAGCTTGCCGGTACGCTTTACCCTGCCATTACCGGCGGACATATATCCCTGCTGGCTGTGGAACTGATGGCCGATGAGGGCAGGGCGTGGCCGC
TGATTGAGGGGACCGGCAAAATCCTCGGGATGTATATCATCGATAAGGTGTCGACCACGCACGCCGAGTTTTTCAGCGATGGTGCGGCAAGAAAGATTGATTTCACGCTTTCGCTAAAACG
GGCCGATGAATCACTTACGGCAATGTTTGGCGACCTGAATAAACAGGCGAGCGAGCTTCTCGGCTCTGCCGGTAATCTGACCGATAAGCTGCAGGGTGCGCTCGGAGGGCTGACCACATG
A
>PNPANE_05970 putative tape measure protein
ATGAGTAATAACCTGAGGCTTGAGGTATTGCTGAAAGCGGTCGACCAGGCGACCCGACCGCTTAAATCCATCCAGATCGCGAGTAAATCCCTGTCGGGAGATATTCGCGACACACAAAAAG
GGCTGCGTGACCTGAACGGTCAGGCGTCGAAAATCGACGGATTTCGTAAGGCAAGCGCGCAACTGGCCGTGACTGGTCAGGCGCTTGACAAGGCAAAGCGCGAAGCCGGTGAGCTGGC
TGTGCAGTTTAAAAACACCACCAGTCCGACCCGCGCGCAGGCGCAGGCGCTCGACGCGGCAAAGCGTGCCGCCTCTGAGCTGCAGACGAAATATAACAGCCTGAGAACATCGGTACAGC
GCCAGCGCTCCGAGCTGATGCAGGCCGGTATTAATACCCGCACCCTGTCTGCCGATGAGCGTCGGCTCAAAACCTCCATCAGCGAAACGACGGCGCAGCTTAATCGCCAGCGTGAGGCACT
GGCGCGCGTCAGTGCGCAGCAGGCGAAATTAAGCCGGGTGAAAGAACGATATAAATCAGGTAAAGAGCTTGCCGGTAACATGGCCGCAGCAGGCGCTGCCGGGGTCGGTATTGCGACA
GCGGGAACGATGGCCGGGGTTAAATTGCTGATGCCCGGTTATGACTTTGCGCAGAAAAACTCCGAGCTGCAGGCTGTGCTCGGGGTCGATAAGCAGTCGCCAGAAATGCAGGCGTTACG
CAAACAGGCGCGCCAGCTCGGCGACAATACTGCAGCCTCTGCTGACGATGCTGCCAGTGCGCAGATTATTATCGCAAAAGGTGGTGGTGATGCTGAAGCTATAGCAGCAATGACACCTGTG
ACACTTAATCTGTCACTTGCTAACCGGAAAAACAATGGAGGAAAACGCTCAACTGCTGATGGGGACAAAAGCCGCCTTTCAGCTCTCTAATGATGCGGCCGCACATATTGGTGATGTTCTCTC
AACCACTATGAATAAAACCACCGCTGATTTTCAGGGGTTAAGTGACTCATTAAGTTACCTTGCTCCTGTTGCGAAGAATGCTGGAGTAAGCCTTGAGCAAGCAGCAGCGATTACCGGGACG
CTGCATGACAACAATATCAGGGGGTCAATGGCTGGGACGGGCGGCGCGGCTGTTATTACGAGATTACAGGCACCCACAGGGAAAGCATACGATGCGCTCAAAGAGTTGGGAGTTAAAACC
TCGGATAGCAAAGGCAATACGCGCCCTCTATTTACCATCCTGAAAGAAATGCAGGCCAGCTTTAAGCGCAACAATCTTGGTACCTCACAAAAGGCCGAGTACGTGAAAACGATATTCGGCGA
GGAGGCCATGAAGTCTGCAAGTGTACTTATGGCGGCAGCAGCAAGCGGAAAACTCGATAAGCTAACTGCCACGATAAAGGATTCAGACGGTAAAACCGAGGAGTTGGTCAAGGTTATGCA
GGATAACCTCGGCGGCGACTTTAAAGAGTTTCAATCCGCTTATGAGGCCGTCGGTACCGACCTCTACGACCAACAAGATAGCTCGTTGCGTCAGCTAACTCAGACAGCAACGCGGTATGTGC
TAAAGCTTGATGACTGGATAAAAAAACAACAAGGAGTTAGCGGAAACCATCGGCATCATCGCCGGTGGCGCTCTGGCTCTGATTGGTATCATCGGCGGTATTGGTCTTGTGGCGTGGCCGGT
AGTTATGGGGATTAACGCCATTATCGCTGCTGCTGGCGTGCTGGGTTCCGTCTTTACTGTCACCAGTAGTGCCATTGTGACAGCGCTCGGTGCGATTACCTGGCCGATTGTGGCTGTCGGTGC
GGCGATTGTGGCCGGGGCGCTACTTATCCGTAAATACTGGGAGCCCATCAGCGCATTTTTCTCGGGGGTGATTGAGGGCATCATGAGTGCCTTTGCGCCGGTCGGGGAAATATTTGCGCCA
CTGGCTCCCATCTTTGACGGACTCGGTGAGAAACTGAGCGGCGTCTGGCAGTGGTTTAAAGACCTGATTGCGCCGGTCAAGGCCACGCAGGAGACGCTCGATAGCTGCAAAAATGTCGGC
GTTATATTTGGTCAGGCACTGGCCTCGGCCTTGACGGCTCCGCTCAATGTTTTTAACAAGCTGCGCAGCGGTGTCGACTGGCTTCTCGAAAAGCTCGGCATTATCAACAAAGAGTCAGACA
GCCTGGACCAGACCGCCGCCAGAACCAACGCCGCCACGCAGGGTAATTCCTACATCCCGGCAACCAGCACATATGGCGGCTATCAGGCTTATCAGCCCGTTACCGCACCGGCGGGGCGCTC
TTACATTGACCAGAGCAAAAGCGAATACAACATCACTCTGCCGGGTGGTGTTGCGCCGGGGCATCAGCTTGACCGCCAGTTACGCGACACGCTCGAACAGATTGAACGTGATAAACGTGC
GCGCCAGCGTGCCAGTATGACCCACGATTTCTGA
>PNPANE_05975 Phage tail assembly protein
ATGGCAAAAGCACCACGCAAAACCCCGGAATTTATTGATACGGCTGGCAATGAAACTGACACCGTGAACCCGAACGTCGTGACCCTGGACAAACCGATTAAGCGCGCCGGTCAGACGATT
GATAAAGTCACCCTGATTGAGCCGAGCGCCGGTACCCTGCGCGGCGTCAGTCTGGCAGCGGTGGCGCAGTCCGAAGTCGACGCCCTGATTAAGGTGCTGCCCCGCATGACCTACCCCGCG
CTCACCGCGCAGGAGCTTACCGCGATGAACCTGCCCGATATGTTGTCGCTGGCCGCTAAGGTGATTGGTTTTTTGTCACCGGCTTCGGCGGAATAG
>PNPANE_05980 Bacteriocin serracin-P subunit
ATGGCACTACCCCGTAAACTCAAATATCTGAATATGTTCAACGATGGCCTCAGCTACATGGGAGTTGTTGAATCCGTGACGCTGCCGAAACTGACCCGCAAGCTCGAAAACTATCGCGGTGG
CGGTATGAATGGCGCGGCGTCTATTGACCTCGGCCTCGACGATGATGCGCTCACCGTCGAATGGTCTGTCGGTGGCCTGCCGGATGTGGCTCTGTGGGCGCAGTACGCCGCCCCGGGTGCT
GATGCTGTGCCGCTGCGTTTTGCTGGTTCTTACCAGCGCGACGACACCGGCGAAATCGTGGCGGTCGAGGTGGTCATGCGTGGCCGTCATAAAGAAATCGACGGCGGCGAGAATAAGCAG
GGTGAAAACACCTCGACCAAACTGTCGACTGTCTGCACCTACTACCGCCTCACGATTGATGGCAGCGACGTCATCGAAATCGACACCGTCAACATGGTCGAAAAGGTGAACGGCGTCGACC
GTCTGGAGCAGCACCGCCGCGCAATCGGGCTGTAA
>PNPANE_05985 Phage tail protein
ATGAGTGATTTTCACCACGGCACGCAGGTCATCGAAATTAATGACGGTACGCGTGTTATTTCCACAGTAGCGACTGCAGTCGTTGGCATGGTCTGTACAGCCAGCGATGCAGATGCCACGCT
GTTTCCCCTCAATGAGCCGGTGCTGATTACCAATGTGCAAAGCGCCATTGCGAAAGCCGGTAAAAAAGGCACGCTGGCTGCTTCCCTGCAGGCAATCGCCGACCAGTCAAAGCCTGTCACC
GTTGTCGTGCGTGTTGCTGAAGGTACCGGAGACGACGCAGAAGCGCAGACCACGTCCAACATTATCGGCGGCACGGATGAAAACGGTAAGTACACCGGTATCAAGGCGCTGTTAACTGCC
GAAGCGGTCACCGGTGTTAAGCCGCGCATTCTCGGTGTGCCGGGTCTCGATACGCAGGAGGTTGCAACTGCACTTGCGTCGATCTGTATCAGCCTGCGCGCCTTTGGTTATGTCAGCGCAT
GGGGCTGTAAAACCATTTCCGAGGCGATAGCCTATCGTGAGAATTTCAGTCAGCGTGAGCTGATGGTTATCTGGCCTGATTTCCTCGCGTGGGACACCGTCAAAAATGCCACCGCAACGGC
TTACGCCACTGCACGTGCACTCGGCCTGCGTGCTTACATCGACCAGACTGTCGGCTGGCACAAAACCCTGTCTAACGTTGGTGTACAGGGCGTTACCGGCATCAGCGCCTCAGTGTTTTGG
GATTTGCAGGCATCCGGCACCGATGCTGACCTGCTCAACAAGGCCGGGGTTACGACACTGGTACGCAAGGACGGTTTCCGTTTCTGGGGTAACCGCACCTGCTCAGATGACCCGCTTTTTC
TGTTTGAGAACTACACTCGTACCGCGCAGGTACTGGCCGACACGATGGCTGAGGCGCACATGTGGGCGGTTGATAAGCCCATTACCGCCACGCTCATTCGTGACATTGTTGACGGCATCAAT
GCCAAATTCCGCGAACTGAAAACAAACGGCTACATCGTGGACGGTGAATGCTGGTTCGACGAGGAATCGAACGACAAGGAAACCCTCAAGGCCGGGAAACTGTATATCGACTACGACTAT
ACACCGGTTCCGCCACTGGAAAGCCTGACCCTGCGCCAGCGTATCACCGACAAATATCTGGTGAATCTGGCCGAATCGGTCAACAGCTAA
>PNPANE_05990 Phage tail protein
ATGACAGCGGCAAAAAAATAAAAAGAACAAGCAGTTTTTAAATATTAAAAATTTCATTCCGTATACACCGGAACCAGACGACACATTATTCGCCGGTGCGGCGCATCTACAATCAGAGGATGG
TCAGGACTGGTATGCATGCCAGCAATTATTTTCAGAAGACACGCTGAAAATTACCTACGACGATAACGATGTTATTACGTGTATCACGCGCGATATTTCCGGTTTATGGCCTGCAGGCCAGAG

CGTGGCGGAGCTACCTGATACGGATGAAAACCGTCGCGCTGATATTTCAGGCGGCTGGCAGTTTAAAGACGGTAAAGTCGTTCAACGGGTTTATTCGCCGGAAGAGCTGCGTAAAAAGGC
GGAAGCTGAAAAAGTTCGCCGCCTTGCTGAGGCTGAATCAGCCATTGCACCACTGGCGCGGGCAGTAAAACTAAAAATCGCCACAGATGAAGAGATTAAACGGCTGGAAGCATGGGAAC
TCTACAGCGTAATGGTAAACCGGGTGGATACATCTGCGCCTGACTGGCCGGATATACCACGCTAA
>PNPANE_05995 Collar domain-containing protein
ATGAGCACGAAATTTAAAACCGTTATCACCACTGCCGGAGCCGCTAAGCTTGCTGCGGCCACCGTGCCGGGCGGTAAGAAAATAAATCTTAACGTTATGGCTGTTGGTGACGGCGGCGGAA
AACTGCCGATGCCTGATGCCGGTCAGACGCAGCTTGTTAATGAGGTCTGGCGCCACACTCTGAATAAAATCAGCCAGGACAACCGGTACAGTAATTACATTGTGGCCGAGCTGGTTATTCCG
CCGGAAGTAGGTGGCTTCTGGATGCGTGAGCTTGGCCTTTACGACAATGAGGGGACGCTGATTGCTGTTGCCAATATGGCCGAAAGCTACAAGCCAGAACTGGCCGAGGGCTCAGGGCG
TGCGCAGACATGCCGCATGGTCATTATTGTCAGCAGTGTTGAGTCTGTGGCGCTGTCCATTGACTCAACGATGGTGATGGCGACGCAGGATTATGTCGACGACAGGCTCGCCGAACATGAG
AAATCACGTCGTCATCCTGACGCCACACTGAAAGAAAAAGGGTTTACTCAGCTCAGTAACGCGACAGACAGCGAGTCTGAAACACTCGCAGCGACACCGAAAGCTGTTAAGGCAGCATAC
GACCTTGCTGACGGGAAATATACAGCTCAGGACGCCACCACAACGCGAAAAGGCCTTGTGCAACTCAGTAATGCTACTGACAGTGTGTCTGAGACGCTTGCCGCGACACCGAAAGCGGTC
AAGGTGGCCTATGACCTTGCTAACGCAAAATATACAGCTCAGGACGCCACTACAGCGCGTAAAGGGATTATCCAGCTCAGCAATGCCACTGACAGCACGTCTGAGACGCTGGCCGCAACGC
CGAAAGCGGTTAAAGCGGCTATGGATAATGCGAACGGACGCGTCCCGTCAGACCGTAAGGTCAATGGGCATCCATTATCCGGGGATATCACCCTGTGGGCGTCAGATGTGAAGGCTATTTC
CGCAGATGCCATTGGACAGATTACCGATAACGGCACGATGGCATCAGCTAATACTCCAGGATGGTGGCGGGTGGCGGTGTCGAATTCTGATACGGTCGCTGATTTTCCGACCTATCCGGATG
GCAGCAAGCTGTACAGCTACGGATATATGTTTGTTGAGAAAATTGGAGAAGTCTGGTTTCAGCACTATTACGCGCATATGGGCGCGAACGCAAAGCGCCAGGACTGGGGAACGGAACCGA
ATACCAGCCGCCAGTGGGTTATTGACTACAACACCGTAAATAAACCTTCAGCCAGTGATGTGGGTGCATTGCCGATTACCGGGGGGCGGCTTAACGGCCCACTGGGTATTGGTACTGACAAT
GCACTGGGCGGTAATTCGATTGTATTCGGCGATAACGATACAGGGTTTAAGTGGCACAGTGACGGCGTTCTGGGTATTTATGCCAATAGTGCCCAGGTCGGTTATATCGACAATTCCGGGCT
GCACATGCTGGCAGATATTCGCGCTACTGGTGTCGTGCGCACCGGCAACGGAAAAACACTGACGTTATCGAGTGGTAACAATTCTGCACTGAATGCTGGTTTAAGTCTGTGGGGAGGTGGA
GAACGTCCAACAGTCATTGAGCTGAGCGATGAACAGGGATGGCATTTATACAGTCAACGAAATACGGACGGAAGTATTTCATTTACTGTTAACGGTATCGTCTACTGTAACGCGTTAAATATA
GGTGGCGCTATTTATCAGAATAACGGTGATATCTTTGGTTCGTTATGGGGAAATGGCTGGTTAAGTACATGGATTCACAACAATGTAGTAAAAGCGGTCAGACTTGGCCCCGTGGCGCTTTCT
GGCGGTCTGTGGCGTGATTTTCAGCTTGGCGGCGGACAGGTGGTGACGGGGTTCCATACTGACGGTAGCTGGGAAATGGAAGGTAACGATGACAAGGTTTATTACCGTCCCATTCAGTATC
TGGTTGGTGATACGTGGGTAACAGCCCCAAGTGTATAA
>PNPANE_06000 Phage tail protein I
ATGAGTGATTCACGATTGCTGCCGGTGGGCTCATCGCCACTGGAAGTCGCTGCCGCAAGGGCGTGTGCTGAGATTGAAAGGACGCCGGTCAATATCCGCACGTTGTGGGATCCTGACACCT
GCCCGGAAAATTTGCTGCCGTGGCTGGCGTGGGCGTTTTCTGTCGACCGGTGGGATGAGAACTGGCCGGAGGGAACAAAACGTGCCGTTATCCGTGATGCATATTTTATTCACTGCCACAA
GGGGACTATCGGCGCAATTCGTCGGGTAGTGGAGCCACTCGGCTATGTCATCAATGTAACGGAATGGTGGGAAACCAGCGACCCGCCGGGCACATTCCGGCTTGATATCGGGGTGCTTGA
AAGTGGCATTACCGAGGAAATGTATTTCGAAATGGAGCGACTGATTGCAGATGCAAAACCAGCCAGTCGTCATCTGACTGGCCTGAATATTGTCCAGGACATTCCCGGTTATTTGTATACCGG
CGGCGTGGCCTGCGACGGCGATATTATTACGGTTTACCCGGGATAA
>PNPANE_06005 Baseplate-J domain-containing protein
ATGGCAACTGTTGACCTGAGTCTGCTACCTGTTCCTGATGTGGTCGAGGAACTGGACTATGAAACTATCCTTGCGGAGCGCATTGCGACGCTGATTTCGCTCTATCCGGAAGACCAGCAGGA
AGCTGTCGCACGGACGCTCGCGCTTGAGTCTGAGCCAGTTGTTAAATTGCTGCAGGAAAACGCCTACCGTGAGGTTATCTGGCGTCAGCGTGTCAATGAAGCTGCACGCGCAGTGATGCT
GGCTTATGCCATAGACAGTGACCTCGATAATATCGGGGCGAATTTCAATGTTGAGCGTCTTGTCGTCACACCTGCTGATGACACCACCATTCCACCCACCCCGGCAGAAATGGAACTCGACG
CCGATTATCGTCTGCGTATACAACAGGCTTTTGAAGGGCTGAGCGTGGCGGGGTCTGTCGGGTCGTACCAGTATCATGGCCGTAGTGCTGACGGACGCGTCGGCGATATTTCAGTTATCAGC
CCGTCGCCAGCCTGTGTGACGATTTCCGTGTTGTCACGTGAAAACAACGGCGTCGCATCTGAGGAGCTGCTCGCAATTGTGCGCAATGCCCTGAACGCAGAAGATGTCAGGCCGGTCGCT
GACCGGGTGACGGTACAGTCAGCCGAAATTGTTAACTACCAGATTAACGCCACGCTTTATCTTTATCCCGGCCCGGAGAGTGAACCCATCAGGGCGGCGGCTGAGGCAAAGCTGAAAGCC
TATATCAGCGCGCAGCACCGCCTCGGGCGCGATATCCGTAAATCAGCGATTTATGCCGCCCTGCATGTTGAGGGTGTTCAGCGGGTGGAGCTGACGGCACCGGTCGCGGATATTGTTCTCGA
TAACACTCAGGCGTCTTTTTGCACTGACTACAGCCTTGTAATCGGGGGATCTGATGAGTGA
>PNPANE_06010 GPW-gp25 domain-containing protein
ATGACGGCGCGCTATATGGGGATGAACCGCAATACCGGCCTCGCTATCAGTGACAGTGAGCATATCAGCCAGAGCATGCGCGACATTCTGCTGACGCCGGTCGGCTCGCGGGTAATGCGTC
GTGAATATGGCTCGCTCCTGTCTGCGCTGATTGATATGCCGCAAAACCCGGCGCTCAGGCTGCAAATCATGGTGGCGTGCTATTCCGCGATCCAGAAATGGGAACCACGCATCAGGCTTACC
TCAATCAGCTTTGAGCGTGGCGACACTGGCGAAATGTATGTCGATATTACCGGGATGCGTACCGATACCGGTGCGCCAGTTTCAACCACTGTTTCACTGAGTTAA
>PNPANE_06015 Spike protein
ATGAATACGTTATCCACGATACAGGAGCTCGCGCGCGCAATTCGCAACCTCATCCGCTCAGGTGTGGTGACTGAGGTCGATATTGTGCAGGGGCTGTGCCGCGTACAAAGCGGCGGGATCC
AGACTACATGGCTGAACTGGCTGACCACCCGCGCCGGTCGTTCGCGGACATGGTGGGCCCCCTCGGTCGGTGAGCAGGTGCTGCTACTGGCGATTGGGGGCGAGCTTGATACCGCTTTCG
TACTGCCGGGGATTTTTTCCGACGATAACCCAGCCCCGTCAGCCTCGGCGGATGCGTGGCATGTGGTGTTCCCCGACGGCGCTGTTATGGAGTATGAGCCGGAAACCGGTGCACTGACGGT
CAGCGGCATCAAGACTGCCGATGTGACGGCATCGGAGTCCATTACCGCCACCGTGCCGGTGGTACTGGTAAAAGCGGCAGAATGTATCACCCTCGACACCCCGGAGGTGGTATGCACCAAC
AAACTGACGACGGCGACGCTTGAGGTGCAGAAAGGCGGCACCATGCGGGGAAACATCGAACATACCGGTGGCACGTTGAAATCAAACGGCGTACAGGTCGATGACCACGGTCACGGCG
GCGTGCAACGGGGCGGGAGCTGGACGGAGGGCACCAGATGA
>PNPANE_06020 Phage tail protein
ATGAGTGAGTTAACCGCGCTGCAGGAGCGCCTTACCGGTCTGATTGCCAGCCTGTCACCGGCGGCGCGTCGGCAAATGGCGGCTGACATTGCAAAAAAACTGCGCGCCAGTCAGCAGCA
GCGCATCAGGCGACAGCAGGCACCCGACGGCACCCCGTATGCCGCCCGAAAGCGCCAGCCGGTGCGAAGTAAGAAAGGCCGTATCAGGCGCGAGATGTTCGCCAGACTGCGCACTAACC
GCTTTATGAAAGCCAAAGGCAGCGACAGTGCGGCGGTGGTGGAATTTACCGGCAAAGTGCAGCGTATGGCGCGGGTGCATCAGTATGGCCTCAAAGACCGGCCAAACCGCAACAGCCGG
GAGGTGCAGTACGAGGCGCGTCCGCTACTCGGTTTCACCCGCGACGATGAGCAGATGATTGAAGACGTCATTCTCAGTCACCTCGGCAAATAA
>PNPANE_06025 N protein
ATGCAGAAGCACAAAAGCCTGCGCAAGGCGCTGATTAACGCCGTGCCGCAGCTCCGAAACAACCCCGATATGCTGCGCCTGTTCGCTGATAACGGGCATACGGATTCCCGACTGGAGAGC
TCGCTGTCGTTTGAAAAGGTGTACGTGCTTAACGTGGTGGTGACTGACTTTACCGGCGACCTCGATTTGATATTTGTGCCGGTGCAGGCATGGCTGCGTGAGCATCAGCCGGACATTATGAC
CACCGACGACGGGCGGGAGAAAGGATTCACATGGATGATTGATATCAATAACGACGATTCGCTCGATATCAGTATCAGCCTGAGACTCACCGAGCGCACGCTCGTCAAAGAGGTCGACGGC
GCACTGCATGTCAGCTATGCCCCTGAGCCACCGCTGCCTGAGCCGGTGACGCGCCCGGTCGAGCTGTACGTTAACGGCGAGCTGGTGAGTAAGTGGGATGAGTGA
>PNPANE_06030 hypothetical protein
ATGCCGGGAAGTGATCCGAAAACCAATGGCGACCTGAGCGCAGATATCCGCCGTCTTGAGGGCGCGCTGACCGCCTGCGCGCTGCAGGTCAAAACCGTCAAACACTGTCAGGATGAACTC
GATGCAGAAGCACAAAAGCCTGCGCAAGGCGCTGATTAA
>PNPANE_06035 LysB family phage lysis regulatory protein
ATGAAAGTCCTGATAACGCTGTTTGTGCTGGCCGTGCTCGGTCTGATGTGGTTGCGCCATGAGAATGGCAATTTATCCCGCTCCTTTGAGACGGCAAACCGCGTTGCGAGCGAGCAAAAGG
CGACGATTGGTATGCTGAAAAATCAGCTCAGTGTCGCCGGCCAGCTCGCCCGACGTAATGAATCCGCGCAGGTGGCACTGCGCGAACAGCTCGCAAAGGCAGGCGCAGAAGCAAACCGC
CGCGAGCAGACGATAACGAGGTTACTTGATGAAAATGAAGCCTTTCGCCGCTGGTATAACGCTCCTCTGCCTGATGCTGTGCGCAGGCTGCACATCCGCCCCGCCTGCGCCAGCGCCGGTG
ATTGTGGTCAACGGATGCCCGAGGGTGAGCCTTTGCCCGATGCCGGGAAGTGA
>PNPANE_06040 Lysozyme
ATGCCGGTCATTAATACTCACCAGAATATCGCCGCCTTTCTGGACATGCTGGCGTATTCCGAAGGAACGGCGAACCATCCGCTGACGAAAAACCGTGGCTACGACGTCATTGTTACCGGCCT
TGATGGCAGGCCAGAGATTTTCACCGATTACAGCGACCACCCTTTCGCACATGGCCGACCCGCGAAAGTGTTTAATCGCCGTGGCGAGAAATCCACGGCATCGGGGCGTTACCAGCAGCTT
TATATGTTCTGGCCGCACTATAAAAAACAGCTCGCATTGCCTGATTTCAGCCCACTGTCGCAGGACAAGCTCGCGATCCAGTTAATCCGGGAGCGCGGTGCTATTGACGATATCCGGGCGGG

GCGTATTGAGCGTGCTGTTTCCCGTTGCCGGAATATCTGGGCGTCATTGCCGGGTGCCGGTTACGGCCAGCGCGAGCACAGTCTCGAAAAGCTGGTTACCGTCTGGCGCACGGCTGGCGG
GGTGGTGGCATGA
>PNPANE_06045 Holin
ATGACAGAAGGGGAAAAAGGCGTCCTGTCACTGTTTGTGATTGGGGCACTGATTGTGGTCGGAAAAGTGCTGGCAGGTGGTGAGCCCATCACCCCGCGCCTGTTTGTCGGACGCATGTTG
CTCGGCGGTTTTGTCTCAATGGTCGCCGGTGTTGTTCTGGTGCAGTTTCCTGATATGTCACTGCCCGCCGTGTGCGGTATTGGATCCATGCTCGGTATTGCCGGTTATCAGGTGGTGGAAATC
GCCATTCAGCGCCGCTTTAAGTCACAGAAGGGGGAAGGCGATGCCGGTCATTAA
>PNPANE_06050 Tail X family protein
ATGAAGGTCAGGTCGATGCAGGGCGACACCCTCGACGTGATTTGCGCCAGGTATTACGGGCGCACTGAGGGCGTGGTTGAAACGGTGCTGCAGGCTAATCCCGGCCTGTCTGAGCTGGG
CGTCATTCTGCCGCATGGCACGGCAATTGACCTGCCCGATGTGCCGTCTTCACCCGTAACTGAAACTATCAATCTTTGGGAGTAA
>PNPANE_06055 Capsid completion protein
ATGACGACAATAATTCTGAACCAGCCCGACGAACCACAGGACGTACCGGGCGTGGTGATTCCCGTACCGGAGACGGGCGATGCAGTAATTAAAAACACGTTCTTTTTCCCTGATGTGGATC
CGAAGCGTGTGCGCGAGCTGATGCGGCTTGAGCAGACGGTTTCCGATGCGCGCCTGCGCCATGCCATCAAAACCGGCATGGCGGAAACCAATGCGGAGCTTTACGACTACCGGCTGCGCC
AGACTGCCGCCGGGTTTAAGCATCTGGCCGACGTGCCTGCTGAGGAAATCGACGGCGAGAATGTGCGTATTTTCCACTATCTGAGCGCCGTAACGGCGATGGCAACCGCCACCCTGTATGA
GCGCTATCGCGGTGTTGAAGCCACCGGCAAGGGTGACAAAAAAGCCGACAGCGTCGAAACCACCATTGATGACCTGTGGCGGGATATGCGCTGGTCGGTCGCGCGTCTGCAGGACAAAC
CGCGCTGCATCGTGGGCCAGCTCTGA
>PNPANE_06060 M protein
ATGACGAGCCCCGCACAGCGTCACATGATGCGGGTCTCGGCCTCTCAAGCCGCGCAGCGGGAGCAAGCCCCGCTGCGCCATGCAACCGCCTATGAGCAGATGCTGGTTAAGCTGGCCGAT
GACCGCCGCACGTTAAAAAACATCCGTTCAAACGAGCGTAAAGCCGCGAAAAAGCGCGAGCTGCTGCCGTTCTATGCGCCGTGGGTCGCCGGTGTGCTGGCTGATGGCTGCGGTGCGCA
GGATGACATTGTCATGACCGTCATGCTGTGGCGTCTTGATGCCGGTGATATCGCTGGCGCGCTGGAAATTGCGCCCTACGCGTTGCAATACGGCCTCACCACTGACCATCGCCGCACGACAC
CTTACATGCTGGTTGAGGAGGTGGCGCTTGCCGCACTGCGTCTGCGCGATGCCGGTGAATCTGTCGACCTTTCCTGGCTGCAGACCACTATCGACCTGACCGACGGCGCTGACGTTCCCGA
TATGGTGCGTGCCCGTCTGCATAAGGTGACAGGCCTGACCCTGCGTGATGCCGGTATGAATGCAGAGGCGCTGGCGCAGTTTCAGCGCGCGATGCAGCTCGACCGCAATGCCGGTGTGCG
CAAAGAGATTGAGCGGCTGGAACGCGCACTGAAGCCAAAAGCGGAGGCACCCCCCCGTAAAACGACTAAACCGCGTACGCGCAAACCTGTCGCCAGACCGGCAGCAAAGCGCGGGCGT
CCACCAAAGGCGGTAAAAACCGCCGGTTAA
>PNPANE_06065 Phage major capsid protein, P2 family
ATGCGCCCGGAAACCCGTTTTAAGTTTAATGCCTATCTGACCCGCGTCGCTGAGCTGAACGGCATCAGCACTGATGACGTCAGTAAAAAATTCACCGTCGAGCCGTCCGTCACGCAAACGCT
GATGAACAAAGTGCAGGAGTCATCCGCGTTTCTGCAGACGATTAATATTCTGCCGGTCGCAGAAATGAAGGGTGAGAAAATCGGCGTCGGTGTGACCGGTACTATCGCCAGCACGACTGAT
ACCTCGGGCGATGATGAGCGTAAGACCGCAGACTTCACCGCGCTTGAATCCAACAAGTACGAGTGCGACCAGATTAACTTTGACTTCCACCTGAAATATAAAACCCTCGACCTGTGGGCGC
GTTTTCAGGACTTCCAGCGCCGCATCCGCGACGCCATTGTCAAGCGTCAGGCGCTCGATTTCATCATGGCCGGTTTTAACGGTACCACCCGCGCCGCCACCTCTGACCGCACCAAAAATCCG
ATGCTGCAGGATGTGGCCGTCGGCTGGCTGCAGAAATACCGCAATGAAGCCCCGACGCGTGTGATGAGCAATATCACCGATGCTGACGGTAAGGTCGTTTCGGCAGTGATTCGCGTCGGTC
GAAACGGCGACTATGAGAACCTCGACGCGCTGGTGATGGATGCGACCAATAACCTGATTGACGAGGTTTATCAGGATGACCCGAAACTCGTTGCCATCGTTGGCCGTAAGCTGCTGGCCGA
CAAATATTTCCCGCTGGTGAACAAGCCGCAGGAAAACAGCGAGGCGCTCGCGGCAGATATCATCATCAGCCAGAAGCGAATCGGCAACCTGCCTGCTGTGCGCGTGCCGTACTTCCCGGC
GAATGCCGTACTGGTAACTACTCTGGAAAACCTCTCTATCTATTTCATGGATGAGAGCCACCGCCGCAGCATTGATGAAAACCCGAAAAAAGACCGCGTTGAAAACTACGAGTCGATGAATA
TCGACTATGTGGTCGAGGCGTATGCCGCCGGGTGCCTGCTGGAAAACATCACCCTGGGCGATTTCACCGCACCTGCAGCACCGGAAAGCGGAGCCTAA
>PNPANE_06070 GPO family capsid scaffolding protein
ATGGCAAAAAAAGTCTCAAAATTCTTTCGCATCGGCGTCGAGGGTGATACCTGCGACGGGCGCATTATCAGCGCCAGCGATATTCAGGAAATGGCCGAAACCTATGACCCGCGCGTCTACG
GTTGCCGTATCAACCTTGAACACATTCGCGGCCTTTTGCCCGACGGCATGTTTAAACGTTATGGCGATGTGGTTGAGCTGAAAGCCGAAAAGATTGACGACGATTCTGCGCTGAATGGCAA
ATGGGCGTTGTTCGCCAGAATCACCCCGACCGATGACCTTATCGCGATGAATAAAGCCGCGCAGAAGGTCTATACCTCTATGGAAATTCAGCCGAATTTTGGTAACAGCGGCAAATGCTATCT
TGTCGGCCTTGCGGTCACTGATGACCCTGCGAGCCTCGGTACTGAATACCTCGAATTCTGCCGCAAGGCGAAGCACAACCCGCTGCAGCGCTTTAAGGCCAGTCCTGAAAATGTCTTTTCA
GTCGCCACGCTGGCCGAACTGGAATTTGAAGACGTTCCCGACACGGTGCTCAACAGCCTGGCCGACAAGGTGAAAGCCATTTTCAGCCGTAAACAGGTCAGCGACGATGCGCGCCTGAAT
GATGTGCATGAAGCGGTTACCACCGTCAGCGAACATGTGCAGACCAGCCTCACTGCGCAGGATAAGCGTCTTTCCGATATGGAAACCGCGCTAGCCACCTTTAGACAGGAACTGACCGGCA
AGGTTGAAGAAACCAGCCAGGCATTTTCCGCCCTGAAAACCACCCTCGACAAAACCGAAAGTTTCAGCCAGCCGCGACGCACGAAAGCCAGCGGCGGCGGTGGCGATGAGCTGCTGAC
CGACTGCTGA
>PNPANE_06075 Terminase, ATPase subunit
ATGACCATTTCCACCGATACAACCTTATTGCATGACCCGCGACGACAGGCATCGCTGCTTTACTGGCAGGGCTTTTCCGTGCCACAGATTGCCGAAATGCTGCAGGTCAAGCGCCCGACCGT
GCAAAGCTGGAAACAGCGCGACGGCTGGGACGGCATCGCACCGATTTCCCGCGTCGAAAGCAGCCTTGAGGCCAGGCTGATTCAGCTCATCGCCAAGCCGCAAAAGTCAGGCGGCGAC
TTCAAAGAGATTGACCTGCTCGGGCGGCAGATTGAGCGACTGGCGCGCGTCAACCGCTACAGCCAGACCGGCAACGAGGCCGACCTTAACCCCAACGTTGCCAACCGTAACAAGGGGGA
GCGTAAGAGGCCGAAAAAGAACTTTTTCAGCGATGAGGCTGTCGCAAAGCTGGAAGAAATTTTCTTCGACCAGTCTTTCGAATACCAGTTGCAGTGGTACCGGGCAGGACTGGCGCACCG
TATTCGCGATATTCTCAAATCCCGCCAGATTGGCGCGACGTTCTACTTTTCCCGCGAGGCACTGCTGCGCGCGCTCAAGACCGGCCATAACCAGATTTTTCTGTCGGCCAGTAAAACGCAGG
CTTACGTGTTCCGGGAATACATTATCCAGTTTGCGCGACTGGTTGACGTCGACCTGACTGGCGACCCGATTGTCATCGGCAACAACGGCGCAAAGCTGATTTTTCTCGGCACCAATTCCAAC
ACCGCGCAGAGCCATAACGGCGACCTGTATGTCGATGAAATATTCTGGATCCCGAATTTTCAGAAGCTGCGCAAAGTCGCATCGGGCATGGCCTCGCAAAAGCATCTGCGCTCAACTTACTT
TTCGACACCTTCCACGCTGGCGCACGGCGCTTACCCCTTCTGGTCTGGCGAGCTGTTCAACAAGGGGCGCGCCAGTGCCGCTGACCGCATCGAAATCGACATCAGTCACAGCGCGCTCGCC
GGTGGGCTTCTTTGCGCTGACGGACAGTGGCGGCAGATTGTCACTATTGAGGACGCCCTTGCCGGTGGCTGCACCCTGTTCGACCTCGACCAGCTCAGACGCGAAAACAGTGATGAGGAC
TTTAAGAACCTGTTTATGTGCGAGTTTGTCGACGATAAGGCATCGGTATTCCCGTTCGAGGAGCTGCAGCGCTGCATGGTCGACGTGATGGAAACATGGGAGGACTTCGCCCCGTTCGCCG
ACCATCCATTCGGCTCGCGCCCGGTCTGGATTGGCTACGACCCGTCCCACACCGGCGACAGTGCCGGATGTGTCGTACTCGCGCCGCCGGTGGTTTCGGGTGGCAAGTTTCGCATGCTGGA
GCGTCACCAGTGGAAAGGCATGGACTTTGCCGCACAGGCAGAAGGCATCCGCAGGCTCACTGAGAAATACAACGTCGAATACATCGGCATTGACGCAACAGGCCTCGGTCTCGGTGTATT
CCAGTTGGTGCGCTCATTCTACCCGGCGGCACGCGGTATCCGTTACACGCCAGAAATGAAAACCGCAATGGTGCTTAAGGCGAAAGACACGATTCGCCGGGGCTGTCTGGAGTACGACGC
CGGAGCAACCGACGTCACACAGTCGTTTATGTCCATCCGCAAAACCATGACCAGCAGCGGGCGCAGCGCCACCTATGAGGCCAGTCGCACCGAGGAAGCCAGTCACGCTGATATCGCATG
GGCCACCATGCACGCCCTGTTAAACGAACCGCTTTCTGCCGGTAGCGGCATGCAGCCTAAATCCATTCTGGAGATTAACCAATGA
>PNPANE_06080 Terminase-6 domain-containing protein
ATGAAAAATAATGTTTTCTCACAAAGCCAGATTCAGGCAATGGCCGATATTCTGCACAATGACAGTTTTGACTATCAGGCAACATGGTTGCGGGTCGGAAAACTCAATATCGACCGCAGCATT
ACTAAATCGCGCCAGATTGGCGCAACGCTGCTATTTAGTCGTGAGGCACTGCTCGATGCGCTGACAACGGGCGATAATCAAATCTGGTTTGCTCATACAGTTGAGCATGCGCGCGTGGCGCT
GATGTACATGAACAATCTTTCAGCACGCGTCGGTGTCCGTCTGGCGAGCAACGGTTACAGCGTACAGCTTGACAGCGGTGCGACCATCAACCTTGTCGGCGAGGAATCCCATTGCGCCGCG
CTGGCGGGTAATGTCTACCTTGATGAGTTCGGATGGTTCAATAACCCGCTAAGAGCGGCAAAAGTCGCAGCGGCTATAGCCTGCCATAATCGCCACAGCCTGACGATGTTCACCTCTCCCTCT
GATAATTATGACGCTTTCCGGGTGTGGAACGGCACGTTCCGCAGGCATCGACCGACACCGCTAATCAATACCGGCGACAGCGTATTCTGTACAGATGGCGTCTGGCGCCAGTCGGTCACACT
GGATGCAGCATGCCAGCGCGGGTGCAATCTCTTTGCGCCTGAGGAAATTAAACACGAATACAGCGACGATGATTATCGCATGTTATTTGGTTGCGACTGGTCTTTCGCTGTTGCAGCGGGCG
AGGTGGCAGCATGA
>PNPANE_06085 Phage portal protein
ATGAGCAAGCGCAAGCCACGCAAAGCAGTCGCCATGACCGCCAGTGCACCGCAAAAAATGGAGGCGTTCACCTTCGGTGAGCCGGTGCCGGTGCTCGATAAGCGCGATATTCTGGATTAC
GTCGAGTGCATCAGTAACGGCAAATGGTACGAGCCGCCGGTCAGCTTCTCCGGGCTGGCAAAAAGCCTACGCTCTGCAGTGCATCACAGCTCACCGATTTACGTTAAACGCAACGTGCTCG
CGAGCACCTACATTCCGCACCCGCTGCTGTCCCGTCAGGATTTCAGCCGCTTTGCGCTCGACTATCTGGTATTCGGTAACGCCTTTCTTGAGCAGCGCCACAGCGTCACCGGACAGTTAATCA

AACTGCTGACTTCACCGGCAAAATACACCCGGCGCGGGGTCGATGACTCGGTTTTCTGGTTTGTGGAAAACTTCACTCAGCCGCATGAGTTCGCACCCGATACCGTGTTCCACCTGCTGGA
GCCTGATATTAATCAGGAGATTTACGGCCTGCCTGAATATCTCAGCGCGCTTAATTCCGCATGGCTGAATGAATCCGCGACGCTGTTCCGCCGCAAGTATTACCAGAACGGCGCGCACGCAG
GTTACATCATGTATGTGACTGACCCGGCGCAAAGCGCGACTGACGTCGAATCGCTGCGCGATGCAATGCGTAACTCTAAAGGGCTCGGCAACTTTAAAAACCTGTTTTTCTACTCACCGAAC
GGGAAACCGGACGGCATAAAAATCGTGCCATTGAGCGAAGTCGCCACAAAGGATGACTTTTTTAACATCAAGAAAGCCAGCGCTGCCGACCTGATGGATGCGCACCGCGTACCGTTCCAG
CTCATGGGCGGCAAGCCTGAGAATATTGGCTCAATGGGTGACGTTGAGAAAGTGGCAAAGGTCTTTGTGCGTAACGAGTTATCGCCGCTACAGGACAGGTTCAGGGAGGTAAACGACTG
GCTCGGCATGGAGGTCATCAGGTTCAAAGAGTACACCCTCGACAACCCGGAATAA
>PNPANE_06090 hypothetical protein
ATGTTATCTCTCGTTTATGAAAATCCGTGGACAACCGTTTTTCTGCTGATTGTTGCCAGTTGTTGTCTCAACAGTATTATTGGCGCATTGCGCGGCCAGTAA
>PNPANE_06095 Transcriptional regulator
ATGCAAAATCCCATTTCATCGAAAAAAGACCCGTTTGAAGATTGGGCAAAGAATCTGACTTTAATGGCATTAAATAACGACCTGAGCTCTCGCGAGGTGGAAAGCTACACCGCAAAAATGG
TCGAAAAGGCTAATAAAGATGAGCTTTCAGTCGTTATCAAACACCTGTTAAATCACATCAGAATGCGCAAATAA
>PNPANE_06100 hypothetical protein
ATGCATCGACTACCGGGCGAAATTCCGCAGCACAAAACTAAAAGCATAAAACTTATGGCTATTGTTCATCGTCTGCAGACGATAATGGTCAATGAGAACCTGACTCCAGCAGAGTTGGTCGG
GTGTGCAGAAATCGTCAGGGATAACTATGGCAAACTGGATAATATCAGCAGACCGGCACATTACGCACCGCCACCACGTCGACCATAG
>PNPANE_06105 DinI family protein
ATGAGAATCAATATCACATTGGATAAAGAGCAAAAAATTAGTCAGGCAACGTTGGATGCACTTGAGGCTGAGCTGTACCGCAATCTTCAACCTATTTACCCAAAGACTGCTATCCGCATTCGC
AAGGGCTCCGCAAATGGTGTTGAGCTAAGCGGTTTGAAACTGGACGAAGATAAAAAAAGAGTAATGGAAATCATGCAGCAGGTATGGGAGGACGATAGCTGGCTGCATTAA
>PNPANE_06110 TumA
ATGGTTGGCGAACATTTCAGCCGAACGCAGCAAAAGTGGGCTTGTGTGCAATTTATCGCCGAGGTATCTCTGATTGCAAACTGCAAGCCATCAGACTTAAAGCTCGCGCTCACTCTCATTGC
AGACCTAGCAAACAGCGAAAATAACGAAACCGAAGATGATATTTTTTATAAAGCTGATTAG
>PNPANE_06115 Replication endonuclease
ATGACGAAATATACGTTTGCATACCCGTGGAATGCTCCACGGTCGGCAATAGCCAGCCCATATCTTACTTATGACCAACAGCATCGCCGCGACCGTATGTTTGCGGCTTTGCTACATGCGAGA
AAAGTGCTTTCTCTCCAGCCCGAGTGCGTGCGTTTTGACGTTTATCGCACCGCTGCTGTGCTGGAGCAAAATCAGGGCAGTCAACGAGCCAATGCCTTTTTAATCAGCTTCTGCAAAAAGG
CATTGCCACGTCTTGAACTGGTCGCAAAAAAAATACGAGTGCACGGGTATCAACAGCAATGTATCAACCGCTGTTTTTGATGGTCATTTTGATACCCAGCTTATGCAATATCTGGCGTCACGGAT
GGTTAATATGGTTGCCAGATATAACCGCCTCCCTGATATGTCGCGCGCCGATATTGACTTGCTGGCTGCTGATATCGCTAATTTCATTCATGCTGAACTGGCTGACATTGATGACACCGGATTTA
GCGAGCTTAAAACGCTGTACGCCTGGTACATGCGCGCTGGGATTATTTCCCTGCAATTCAACGTTACCCCGCCGCACTGGGAGCGGGTGATAAAGAAATATGTTGGCGAAGATGAAATCGC
CCCGGCTATCGCCCGAATGTTTAACGATGTGTGGTGGCGTGGCCGCCTGCGTCGCATTGCGGCTGCATGGCGCGAACATCTGCAAATTGCTGTTGGCAACGTCAGTAAGAAAAAGCATGCC
TACGCGAGTAAAAGCTGCGTGACTGACTGGCGCGAGCAGAAGCGCCGCACACGCGAATTTCTCAAGGGACTGGATCTCGAAGACGAATATGGTAATCGCATCAGCCTGATTGAAAAATTC
GACGGCTCGGTCGCTAATCCAGCAATACGCCGCTGCGAGCTGATGGCTCGTATCCGTGGGTTTGAAAATATCTGTAATGAGCTCGGTTATGTCGGGGAGTTTTATACTCTGACTGCACCGTCT
AAATATCACGCTACCACCAAAGCGGGCTACCGTAACAGCAAATGGAACGGAGCAAGCCCGTCAGACACGCAGAGCTATCTCACCGGTCTTTGGGCGCGTATTCGCGCCAAGCTACACCGA
GAAGAAATCCGCATTTTTGGCATACGTGTTGCCGAGCCTCATCACGACGGAACGCCGCATTGGCACATGCTTATGTTCATGTTGCCGGAAGACGTCGAGCGTGTGCGCCTCATCATCCGTGA
TTATGCGTGGGAGGAAGACCGCCACGAACTGAGAAGCGATAAAGCCAAAAAAGCGCGCTTTCATGCCGAGGCCATTGACCCGGAAAAAGGCAGCGCTACCGGCTATGTTGCTAAATACAT
TTCGAAAAATATCGACGGCTATGCTCTTGATGGTGAAACCGATGACGAAAGCGGTGAGCTGCTGAAAGAGACAGCCCCCGCTGTTTCTGCATGGGCGGCGCGCTGGCACATCCGTCAGTTT
CAGTTTATTGGCGGTGCGCCGGTGACGGTATACAGGGAACTACGCAGAATGGCTGACCCTGAAACAGCCAGGGCGCTTAGTGTTGAATTCGCCGCCGTGCATGATGCAGCCCACTATGGCC
GCTGGGCTGATTATGTGAATGCTCAAGGCGGACCATTCGTTCGCCGTGACGATTTACAAGTACGTACATTGTATGAGCCTCGAACTGAATTTAATCAGTATGGCGAAGAAACTGTGTGTATCA
AAGGTGTCTACGATGCCTCGATAGGTGCTGGCTCTCCTATTCTAACCCGGCTTACGCAGTGGAAAATTGTTCCGAAGCGTGCCGTTGATTTGGCCGTTGACGTTAAGGGCGCTCCTGCGCCC
TCTCGGAGTTCTGTCAATAACTGTACGGGAAGCGAAAGCGATCCACCGGAACTCGATTTATCTAAACCCCTAAGTCGACGTGAAAAACGAGAGCTGACAAACCGACTCAGGAAGCAAAAG
CCAGCAATACGGCGAAAATTTATCCACGGAACGGATGAGCAAAACGCAGCTATAGCGAAAACTATCGACGAGATACATCTGACTACCGGCATCACAATCAGCCGGGGCGAAGCCCTGCACC
TGATGGCCGGTGGTAAAAGTTGTTTTGATGGCAAATGGCTACGCGGAACGGCCAAAGGTGAAATATTTTCCGCAGCGCCATCGCATCAGGCTAAAGCCAGGAAAATCCTTAATCGTGTTGC
TGCGATGGCTGAAGCAGCAAAAACCAATATCTGA
>PNPANE_06120 Oxidoreductase
ATGGTTAAGGGCGTGGCAGATGTTACTCATTACCACGGAACACCTGTATGGGGTGACGCCGGTAATGTTCACCGTATTGCGGTCACCGGTGCTGGCGCATTTGTATCGTATGCACGTCCTGAC
CAATTAGCTGCATCAATTAAATACGCCCTCTCTGTTGCTATAGATAATGGCGCTTTTTCAGCGTGGAAGCGTGGCCTTGTTATTGACTGGCAGCAATTCTATCAATGGCTGATTCCTTATTATCAC
CATCCCAAGCTGTCATTTTTTGTTATCCCTGATGTGGTTGAAGGTGGCGAAGCTGACAATGACGCACTTATCGCCAAATTACCACGCTGTTTCAAAGACAAAGCTGCGCCAGTCTGGCATTTG
CATGAATCATTGCACCGTCTGGTTGAGCTTTGCCGGGAGTGGCCGCGTGTGTGCTTTGGGTCGTCAGGTGAGTACGCAACTATCAGAACCCAGCTCTGGCATCGCAGGATGTCAGAAGCGT
TTGAAACCATTTACTGCAAACATAGTTTCTCAACGCAGGTGCACGGGCTCCGCATGCTCGATGGTAGGGTTTTGGGTAACTATCCTTTGGCGACTGCCGATAGCACAAACCTTGCCTGTAATG
TGCCGAAATTCGAAGTCAAATACCCGGAGCTCACAAAAGCAATTCGCGAAGCCGATTATGCGAAAAACCTTTCTGAAGATGAACTTAAAGCCGTCATTCTCAAACGCCGTTGCGCCATTCTG
AAAAATACTATTGAGGCCGTATCCCCTCCATCGATTGAGAATTGGCTATCAAAAGGGTTGGCTCCGCTTCAACTGGAGTTGGCTATCGCATGA
>PNPANE_06125 Uncharacterized protein in GpA 5'region
ATGAGTATTCGTATCGAAGTTGGCGACAAATGGGTTGTTACCAGCGACCAATATCAATTCATCCTGAATGAAAAGAAAATCGCTCAATCAGGGAAGAAAGCTGGCGAGGAATGGCTCGACA
CCATCGGCTATTACCCGAAGATTAACCAGCTTATTTCCGGTCTGGCACATCACTACATCCATAACTCGGAGCTTCAAACTCTGAATGACATTGCCTCAGAAATTGAGAGGTTGGGGATGTTATG
TGAGTCAGCCTTTCATACTGGGATTGCGAAATAA
>PNPANE_06130 hypothetical protein
ATGGCCGACGCAATGGATTTAGCACAACAGCGCGAGCAGGAAGACCGCGAGCGCCACATCAGCAACGCGCGCAGCCGTATCGCTGCGCCTTCCCGTTTTCTTTGCGAAGAATGTGACGCA
CCAATCCCGGAAGCTCGCCGCATTGCGATACCGGGCGTGGCCTTTTGCGTGACCTGTCAGCAAATAGCAGAGCTCAAATCTAAACATTACAGGGGAGTTTAA
>PNPANE_06135 DUF2732 domain-containing protein
ATGCGTAATACCGAAATCCGTAGTTTTAACACTAATAGTGATGCGCTGGCCGTATTGCTGACCGATGCAAAAAAAGAAGAGCGTAAAGACCGCGCGCTCGCTGTTTCCATCCGCCTTGAGGC
GCTGGCTATCCATATCACCAAAGAGGGTATGAGTGGCACCGAAGCTGCCGAACTGCTGCGTCGTGAAGCAATCCGCTTTGAGAATGAATCACAGGAGCTGCACTAA
>PNPANE_06140 Protein dhr
ATGTCACGCGATGAATTAAGGATTGTTTTAGGTGCCATGATTCCGAACATGGAAGACGGTTTTGAAATTAAAACCCGCGATGGGATAATTTTACGAGTTGACCCGGAATGGGAGTGCTGCAA
GCAGTTTAAAGATAGTCTGAAAGCTGAAATTATCAGCCAATTGAAAAGTAAACCAGCCATCGTATTTGGTTATAGCTAA
>PNPANE_06145 Protein fil
ATGCTGAAAAATGAACCGTCATTCGCGTCTCTGCTCGTCAAGCAAAGCCCAGGCATGCACTACGGCCACGGCTGGATCGCAGGTAAGGACGGCAAGCGCTGGCACCCGAGCCACTCACAG
TCCGAATTATTAAAAGGGCTGAAAACAAAGCCGCCGAAATCGTTAGACTTTTTAATTATTCGTATTGTCCACTTTATTATTAAAGGGGTTAAACATGTCACGCGATGA
>PNPANE_06150 Regulatory protein CII
ATGTTAGATTTTCGTGTTTCGTCACATGCGCACTTTGACGAGGCCTGCAGAAAATTCGCAGCTACTCATAACGTGAAAGAACTAGCGAATAAAGCCGGAATCAAGCCGCATACGCTTTACAA
CAAACTCAACCCAGAACAGCCGCACCAGTTAACGCCGCGTGAAATCTGGACGCTGACAGACATGACCGAAGACTCGACCCTCGTCGATGGTTTTCTGGCGCAGATCCATTGTCTGCCATGC
GTACCGGTCAACGAACTGGCAAAAGAGAAACTGCAATCCTACGTCATGCACGCAATGAGTGAACTTGGCGAACTGGCAAGCGGTGCAGTTTCCGGCGACCGCCTCACGCCAGCCAAAAA
ACAAAACATGATTGCTAGCGTAAATGCGGGGATTCGCATGTTGTCATTATCGGCAATGGCGCTGCATGCACGTCTGCAGACTAATCCCGCTATGTCGAGCGTAGTCGATACTATGAGCGGTAT
TGGCGCATCGTTTGGTCTGATTTGA

>PNPANE_06155 Cro/Cl family transcriptional regulator
ATGTCAGATGCAAAATCAATCCCGCTGCATGACGCACAAAACTTACAAAATCAAACTGTATTGCTAGACGCTGGTCAGTTCAACGCGCTTGTAACCATGATGCAGCTATCCATGCAAAACATG
ATTCGTACAGCGATGTTAGACACCATGTCAGTAAAAGACTTCGCCGCCGCCCGTGGCGTTAGCGAGCGTCTGGTCTGGCAATGGATTGATGAGGGCGTCCTTCTCAAAGCTCCCACCAAAG
ATGTTACCAGCAAAGAAAAAGCCGCTAAGCGTAGCCGCACCCTTATCAACGTCAAAGCATGGCGTGACAAACTGACCCAGCAAGCTATCGATTGCCGTTATATCAACTAA
>PNPANE_06160 hypothetical protein
ATGTCTGTAATGTTGCATTCAAATTCAGCAGACTTATTCGATAGTCTAACTTTCCCACCCGGCAATCTGATTACGTCAAAAACATCAAGAGCACCATCAATACCAATAAGCCAGCGGCCATTTCC
TATTTTTGAGCTGGAGCAGTCGACAAGCCAAGATACACTCACGCCATCAATAAACACTAAATCATCAGTGTTTGCCGGAACCATAGAGACATCAGGAGTCCAATGCCCGATATCTTTGAGCTC
GCCAGCTTCAAGGCGGCATTTCCTGATGGTTAAAGCATTAGACGGTTCCCCATCTTTGCTTTTACGCATCTGGCCTTTTCCCGTAGACAACCATCCTAACGAAACACCAGTATCGAGGGCGCA
GGTCACAACCACGTCACCAGGGAAAAAATCGCGTCTAACCCAAGTACTTATAGTGCCAGAAGATATGCCTAATAAATCCCCAAGCTCTTTTTGCATCGTAAAACCATAG
>PNPANE_06165 DUF2511 domain-containing protein
ATGAAACGTAAACTTTTTTTAACTCTGCTTCTTACTCTTTCGTTTGGTGTCGCTGCTGCTGAAAAAACCAAAGAGATAGACGGATCTACCTATGGCGATAAGTGGCCTCTAACCTTTGAAAAG
GCGAAAGTATCATGTGTTAACCGTGCTTATGCTTTTGTGTACGACATTAAAACTGATGACAGATATCCATTGAATGGCATGGCTGTTGATGCTGTTAAGTCAGGAAAGATGGAAGGATCTAAC
TTAGATGACGTATGGAAGGATGACCCTGATTATGACGGTGTTAAAATTTCCATTTCACCGGTGATTGATGCCGCTACAGCCCTTTGTAATTAA
>PNPANE_06170 Tyr recombinase domain-containing protein
ATGACTGTAAGTAAACAAAAAAGTGGCAAATGGTTATGTGAACTCTATCCAAATGGTCGAGAAGGGCGGCGTATACGTCGGCAATTCAATACCAAAGGTGAGGCCGAGGCATTCGAAGCAT
TTACCAAAAGCGAGAGTGAAGATAAGCCGTGGCTCGGCAAGAAAGAAGACCGCCGACGCTTAAGCGAAATTATTCAGCTTTGGCACAATTTGCACGGTCAAGCGTTAGTCGCCAGTAAGT
CGCGGTTAGCTAAGCTTCAAATTGTATGTAACGGGTTGGGCGACCCTATTGCATCTCGTCTTACCGCTAAAGATTGGGCTCATTACCGCGACCGTCGATTACGTGGCGAAATAGACAACGGCT
ATCACAAAGACCCGGCGAAGTGGATCGCCAAACCTATAACAGTCAATCGCGAGCAGCAATACCTCGAAGCGGTGTTCAATGAACTGCGACGATTAGGAGAGTGGAGTTTACCCAATCCACT
GGACGGGATTCGCGTATTCAAAGAAGCTGAGAAAGAAATGTCGTGGCTAACTTTGTCTCAACTCGCGGAGCTGTTCCGAGCCTGTGAACAATATGGCAAAGAAAATCTTACGATGATTGTT
AAGGTGTGCCTAGCTACTGGCGCACGATGGGGAGAAGCAGAGAGACTTACACGCCCCCAACTTTCTCCATACAAGCTGACCTTCACCAAAACCAAAGGTAAGAAGAATCGCACGGTTCCG
ATTCCTAAATGGCTTTACGACGAGTTATCCGAACGTCAGGGCAGAATGTTTAAGCCCTGCTATCAGGAGTTCAAGAAGATGCTCAAACTAACGAACATTGAGCTGACGGAAGGGCAGAAGA
CTCACGTTTTGCGTCATACTTTTGGTGCGCATTTTATGATGAACGGCGGAAATATACTGGTGTTACAGAAAATTCTCGGACACGCCAATATTCGAGAAACAATGAAGTATGCGCACTTTGCTCC
TGACCACCTTGAACAAGCTGTAACTCTCAATCCGTTATCGCTGTATGTTGGCGACAATATGGCGGCAGAGGTTGCATAA

Prophage 8

>DLDGEG_10095 SraC/RyeA RNA
AAATAAAAAGAGACCGAATACGATTCCTGTATTCGGTCCAGGGAAATGGCTCTTGGGAGAGAGCCGTGCGCTAAAAGTTGGCATTAATGCAGGCTAAATCGCCTTGCCCTTTAAGAATAGAT
GACGACGCCAGGTTTTCCAGTTT
>DLDGEG_10100 SdsR_RyeB RNA
AGGCGATTTAGCCTGCATTAATGCCAACTTTTAGCGCACGGCTCTCTCCCAAGAGCCATTTCCCTGGACCGAATACAGGAATCGTATTCGGTCTCTTTTT
>DLDGEG_10105 Integrase
GTGAGTCGAAAAAAGTACGATGCTAACCTTCCGAGGAACCTCACCTACCGTAAGGCCAGTAAATCTTTTTTCTGGCGTAACCCGGTAACTGACAAGGAATTTCCGCTCGGTCAGATCGCCCG
CAGGGACGCTATCACACAGGCCATAGAGGCAAACAACTTCATAGCGCAAAACCACACGCCCGTGGCGCTTATTGAAAAGCTAAAAGGAACTGACTCATTCACGGTGTCCGCATGGATTGAT
CGCTATGAGGTTTTATTACAGCGCCGGAGTCTGTCGGTTAATACCTACAAGATTCGCGGTAATCAATTAGCGACCGTACGCGAAAAAATGGGGGAAATAATACTGGCAGAAGTAACAACCAG
GCACATTGCCAAGTTTCTTGAGTCGTGGATAACCGAGGGAAAAAACACTATGGCGGGAGCAATGAGATCAGTTCTATCTGACATGTTCAGAGAGGCTATTGTCGAAGGGCATATTGTGAAA
AACCCGGTGGAAGCAACCCGGATACCAGAGATTAAGGTGGCCAGGGAACGCCTGCAACTGGAAACGTATAACGCCACACGAGCGGCAGCAGAGCATATGCCTGCATGGTTCCCTCTCGCG
ATGGATTTAGCGCTCGTTACTGGTCAACGTAGGGAGGATATCGTAAATATGAAATTTAGTGATGTTTTTGACAACCGCTTATACGTCACTCAGATTAAAACCGGAATGAAAATAGCCATTCCCC
TCTCCCTGACACTTCGGGCGACGGGGTTACGTCTGGGAACGGTAATCGATCGCTGCCGACTTGTAAGCCGCACTGATTTCATGATCAGTGCCGGAATCAGGAAAAATAGCCCGACCGGGAA
TATTCATCCGGATGGATTGACAAAGACATTTGTAAAAGCAAGAAAAGCCTCCGGTGTTAACTTCAGCAATAATCCACCGACATTTCACGAGATCCGAAGTCTGGCCGGGCGGCTGTACAAAA
ATGAGCACGGCGAGGTGTTCGCCCAAAAACTCCTGGGCCACACATCAGCGAACACCACGAAACTCTATCTCGATGAGCGTGATGATAAAGCTTATATGATGCTCTAA
>DLDGEG_10110 Histidine kinase
ATGAAGAAGCCTGTCTGTATGTTCTGCGGCGCCCCTGCCACCCTGCTTTGTGACGGGATCATCGGCTGGGATGCCGATGAGGATGAACACGGGCACATGACAAAATGTCGAGCCATGTTCA
CCTGCGATGCGCCCGTGTGCCGGAACTGCGCTACATGGCATGGCAACATATTTTTCGATGGAAAAATCCGGATGATGGATACACGCGACCTTTGCCCCCTGTGCCAGAAGTTACACGAAGCC
GGCAAATCCATACGCGTTGCAGAGCACCGGAAAAACTCCGCCCTGCCGCAACCCTGCCTGACTGAAGAGCAGGCTGACAGGATACGCGCCGCGCATTGGGCAGGATTTACAGGACGGCG
CGCCGGAGATGTAAAAGTTTTACCGGGCGGCGGTCAGCAGTCCTTTGAATTTTAG
>DLDGEG_10115 DNA (cytosine-5-)-methyltransferase
GTGAATAACGAAATCATTGTGGATAATTTTGCTGGTGGTGGTGGCGCCAGTACCGGAATAGAAATGGCGATCGGTCACAGCGTTGATATTGCCATCAACCACGACCCGAATGCTGTTGCAAT
GCATACAACTAATCACCCCGACACGCTGCATTATTGTGAGTCGGTTTTTACCGTTAAACCCAAAATAGTGACCGCTGGCCGTCCCGTTGCGCTGGCGTGGTATTCCCCTGATTGCCGCCATTTC
TCCAAAGCGAAGGGCGCAAAACCTGTTGAGAAATCAATTCGCGGACTGGCGTGGATCGAGCTTCGCTGGGGGCTGGAGGTAAAGCCACGGGTAATGATGCTGGAAAACGTGGGCGAGT
TCAGAACGTGGGGGCCACTGCTTGCCGGGGAAATGCGTCCTGATCCTGAGCGTACCGGAGAAACTTTTGAGGCGTTTATCGGCATGTTAACTACTGGCATTCCGGTGGATCATCCTGCGCT
GGCGGAATGCTGTGAGTTTCTGGAAATCGATATTCACAGTGAGCAGGCAAAGCGACTGATCAACGGTCTGGGTTATACCGTTGAATACCGGGAATTACGCGCCTGCGATTATGGCGCGCCG
ACAATCAGAAAGCGTTTTTTCATGGTGGCACGCTGCGATGGTCAGCCGGTTGTATGGCCGGAACCAACCCACGGCGATCCGAAATCAGAAGCGGTGAAATCGGGTCGCCTGAAGCCCTGG
AGAACGGCAGCAGAATGTATTGACTGGTCAATTCCGGCACCGTCAATTTTCGGGCGCAAAAAGCCGCTGGCTGAAAATACGCTCAGGCGTATAGCGCGGGGTATCCAGCGATTTGTCATCG
ACAGTGAACAACCGTATATCGTGCCATTCATTGTGAAATGCAATCACACCAGCAGCAAGAGTGAATACGAGTGTTTTCGCGGTCAGGGTTTACAGGAACCGCTACAGACAATCACAAAAAA
ACACGGTTATGCCGTGGCAGTCCCTCATCTTACAAAATTCCGTACCGGCGCTACGGGGCAAAATGTCACCGACCCCGCGCCCACCATCACCGCAGGTACATCGACGCGACCGGGTGGAAAC
GGTCATGCCCTGGGTATTGTTGAGGCCGCATTAACACCTTTTCTTGCTGGAAATGGTGGCAGCGAGTACCAGGCAAAACCGAGACCACTGGATAAACCCTCGCATACCATTCTGAAGCAGTC
CCGCGCCTGCCTGGTCGCCCCGGTCATTACCCGCCAGTTCGGGGCCAGTATCGGTCATCGGGCAGACGAACCTGGCGCCACTGTCACCGCTGGCGGTGGCGGGAAAAGCCAGTTAACGA
CCGCCACGCTTATTCAGATGGGCTATGGAGAACGTCCCGGCCAACAGCCTCGCGTACTCCAGCTCGGAAAACCGCTGGGCACGGTCACTGCCGGCGGCGGTAAGTTTGCTATCACCAGCG
CATTCCTGGCGAAACACTACGGCGGGAATTATACGGGGCCAGGTGTAGGACTGGAGGAACCTGCGCACTCAATAACAACGGTCGATCATCACGCTGTTGTATCCGCGCACCTTATGGTCAAT
AACACAGGACATGCTGGCGGCGCTGCTGATTCACCTGCTCACACCGTCACAACGGGAAACCATCATGCTGTAGTCGCATCCCACATGGTCAAATTGCGCGGCACCTGCCGGGATGGGCAGC
GCGTTGACGAGCCGATGCCGACGATCACGGCAGGAGGCCTGCATGTAGGTAACATCGAGACCAGCCTTGCTGTCGATGAGTACGACGAAGAGCGCGCACAACAGGTACTGTCGTTCCTGC
GGAAATATTGCGGAGAAGACTGTACTGGTCTGGTCACTGTCGGCGGCGTGGTTTACCGCATCGTAGATATCGGAATGCGGATGCTGCAACCGCGCGAACTCTACCGCGCACAGGGATTTCC
GGACTGGTACATCATTGAGCATGATTTTCGTGGTGTGAAATATGCGAAGGACAAGCAAGTAGCGCGCTGTGGTAATGCCGTGCCACCACAGTTTGCTGAGGCGCTGGTGCGCGCAAATCTG
CCGGAATTGTGCGTCAACGGGGAGGTAATTGCAGCATGA
>DLDGEG_10120 DNA repair protein

GTGGAAAAAATATTGTGTTACGCCTTAAACCGCATCGCCGAACTGGAAAATATGCTGCTGCCGGCAATTCCGGAAACCGTCTGGCCTGCTGAGGTGGAGCTTATTTTTTCCCATACTGAACG
GGCCGGAGATTTGCCAGTACATCACCAGCACAGACTGAAGCACCACATTAACCGTATGTGGCTGGAACACCTGCCTGTCCCGTCAATTGTTACCGCCGCTGAGGTGTTGTGTAAGGAAATG
GAGAGATACGCGTGA
>DLDGEG_10125 hypothetical protein
ATGTACAAAATAACCGCCATCGTCAAAAAGCCGGGTAATTCCCCGACAAACTGGGTTCGTTTTTCTGACAAAAAAATGAATAAAGCCGAGTGTGAAAAAATGCTGTCCGGCAGAACTGAAG
CCGGAAAATCACGCGAAGAGAAAGTCACGCTGGAAGAGTTTAAATGTATTAAGGAATAA
>DLDGEG_10130 site-specific DNA-methyltransferase (adenine-specific)
ATGAGCTTTGCTATCAAACATCCGGCGATCCGTTATCACGGCGGTAAATTCCGGCTGGCATCCTGGATTATTAGCCGTTTTCCGGCACACCGCTGCTACGTGGAACCATTCGGCGGCGGCGC
ATCAGTGTTACTGAAAAAAGAGCCATCAGAAGCGGAAGTCTATAACGACCTTGATGGCGATGTGGTAAATCTGTTCCGCGTGCTTCGTAACCCTGAAAGCAGTCAGGCACTTATCGCTGCGT
GCGCCCTTACACCGTATTCACGCGAGGAATTTACTCATGCTTATGGACATAGCGAAGACCCGGTAGAACGGGCCCGCCGTCTGGTTGTTCGGGCAACGATGGGCTTCGGCAGCGCAGGTGC
CACAAAGGGCAAAACTGGTTTCCGCCTCGATACCCGGCGTAACAGCGCGACTGCTCAGGCTATCTGGGCACGTCAGCCTGATAACCTGGCGGCGGTGGCCAGCCGTTTTACTGGGGTACT
TGTTGAGAACCGCGACGCTGTGACATGCATGAAAGACCACGATACACCTTCAACGCTGCATTTTGTTGATCCGCCTTACATACACGATACGCGTGTCGAAGTAGCAAAAAACAGCGCCTACC
GCTTCGAAATGACTGATGCAGAACATGTTGCCCTGCTGGATTGCCTCAGGCAATTAAGTGGCATGGTTATTGTCTGCGGCTACGACAGCAAACTTTATAACGATGCTTTATCAGACTGGAAAT
GTATTACAAGAACCACGTCTGCTAATGGCCGGGCGGGTTCAGTACAACGAACAGAATGCCTGTGGATTAATCCGGCAGCACAGAAAAAGGAGACCGGGCCATGTACAAAATAA
>DLDGEG_10135 Phage protein
ATGAGCAATCCATTTTTTATCAAATGCCTCAAAGATACAGAAGGCTGGTGGACTGAAGGCGAGATTTACGAAGCTCGCAGGGTTGCCGGTGGTTTTGTGCAGTTTGGTGATGATAACCAAC
CAAACGGTGAGGACTGGAGCGCTTCTCCGATTCAGTACCGGGAGGATGGTTCGATCCTGTATCAGGTCGGTGGTCTGGATGGTGAAGTCATTTTTGAGGAGGCAGGACAATGA
>DLDGEG_10140 Recombination and repair protein RecT
ATGCCTAAACAGCCACCTATTGCAAAAGCCGACCTGCAAAAAACACAGGGAGCACGCACCCCGACGGCAGTGAAAAATAACAACGATGTGATCAGCTTTATCAACCAGCCTTCCATGAAAG
AACAACTGGCGGCGGCCCTGCCCCGCCACATGACAGCGGAACGCATGATCCGGATAGCCACAACGGAAATCCGAAAAGTTCCGGCGCTGGGTGACTGTGACACCATGAGTTTTGTCAGCG
CCATCGTTCAGTGTTCCCAGCTTGGGCTGGAGCCCGGCGGCGCGCTCGGTCATGCCTATCTGCTACCGTTCGGAAACAGAAACGAAAAGTCAGGCAAAAAAAACGTTCAGTTAATTATTGG
CTACCGGGGAATGATCGACCTTGCCCGCCGTTCCGGACAGATTGCAAGTCTTTCCGCGCGCGTCGTCCGCGAAGGTGACGATTTCAGCTTCGAGTTTGGTCTGGAAGAGAAGCTGGTACA
CCGTCCGGGTGAGAACGAAGATGCACCAGTTACTCATGTCTATGCCGTTGCCCGCCTTAAAGATGGCGGCACACAGTTTGAGGTAATGACCCGTAAACAGATAGAGCTGGTACGGGCACAG
AGCAAAGCCGGTAACAACGGCCCGTGGGTTACTCACTGGGAGGAAATGGCAAAAAAAACCGCCATACGCCGCCTGTTCAAATACCTGCCTGTATCCATTGAGATCCAGCGCGCGGTATCAA
TGGACGAAAAGGAGACGCTGACTATCGATCCGGCTGATGCGTCTGTCATCACAGGTGAGTACAGCGTCGTCGAAAACGCTGGCGTGGAAGAGAACGTGACCGCATAA
>DLDGEG_10145 DNA breaking-rejoining protein
ATGAGCATTAAGCAGGAAGAATATTCATTTTATTACAAGGTTAAAAATGAAAGTGCCAGGAAACGCCTCGGCTTTAAAGCCGGTTTTTTCTGGTGTACAGCTAAAAAGCAGTCACTCGCCCT
CTCCCGTGGCGAACTGGCTATGGATGCTGCCGGATTTGATGAAGCTGATTTTGCCAGACCTGTACGCGTACATTTTCCGGTAGAAAATGACATTCCGCCCGAGGGGGCCTTTGATACTAAATT
TTGTGAAAACCGCGAACCCGGTGGCGAAGACGGCAAAACCCTGACACTTATCCCCGGCGCAGCTTCTGCTGTTAAATCAGATGAAACAGAACGCGCCGACGGTGCTGGCACTCCTGCCGG
AGAAAACGGGATACAGGAATCTCATAACCCGCCAGCAAACCCTCAACTGACCGTGGTTGCGACACTGCCGTTCCGCCATCGCGTTCTGGCACAGTATATTGGCGATGGAGAATATCTTTATC
ACGTCGATACAGACCAGAAAAAAGAAATCGCGTGTCTGGAGATGGATACTCAGAATACCACTGTCCAGAACCTGATACTCGCAGCAGAAAATGTAGAGCCGTTCAAAAAAGCTATCGAGCA
CGATATTCACAAAGCAGTGAATGCGTATAAACAGGTATTTCCTGTCGATGGAAAAGTGCCTGAGTTATGCACCACTATTAAGTTTTTTAAGGAATGGTTCAGTGCTGAACACATTAACCGCGG
CCTGCTGATTAAGGAATGGGCTGAACGCCTGAAGAATAAACCGGCACCTGTTAAAAAAACCGGGCCACATAAAGTAATTGTCGACGACGTAAATAAGCCAGAACGTCCACGCCGTAGCGA
AAAACCGACACACAGAACGATTAACTATGAGCTCGCTTGTGGTTTCTGTGAGGAGCTGGATCTGAATAACCTGCGTCCTGCAATGGATTTTGCAAAACGTATCATCGCCGAAGACCGGGAA
GACTGGAAACAAATGTCGATGACAGTGGGCATTATTCCCGACATCAAAGGCTACGACCGACAGACCATTATTGACCTGGTACGCAAAGCGCCAAAGGCCGTACATAACGGTAATCCTGATCT
TCGCCGGACGTGGTGCGAAAGCTTTCTTGCCGTTCATGGTGTTCGCGATCCGGACTGGTACGAATATGTGCCTGATAACACCCCAACAACCCATGAAGAAAATGTAGCAAGGCTTCGTCAG
GCGGGCAAATGTCTGCAGGATATTGAGGCAGGGAGATTTCAGTGTGATGAAGAAAAACCGCAACCGACAGGCGAACTGGCAGATGAACCAGCAACGCCTGAAACAATGGAACAGGACA
CAACTGAACATCATCCGGACCCGCAGCCGCTGGAGAATGAGCCACCTGTAAGCCAGACAGAAGCAGGCTACCAGAAAATACGGGCAGAACTGCACGAAGCACGTAAAAACATTCCACCC
AAAAACCCGGTTGATGTTGGTAAACAACTGGCAGCCGCGCGCGGTGAATATGTCGAAGGCATCAGCGACCCGGACGATCCGAAGTGGGTTCATAACAATTACAGCGCCTCAAATCAGGGT
GAAAAAGAAGAAGTGGTGCCGGAGGAAAAACAACCAGCAGCAGAGCCGGAGGCTGTCACCAGAAACGCGGACGGGACTTTCGATGTATCAGCGCTATTCCCGCCCCCCTCAAACCAGA
CCGAAAAAACGGAAGCCAGAACAGAAAGAGATGGAGAGATGCGGAAAGAAAGCAACCAGCAGGAAACGGCTGGCGATACAGGGCAGGAAATTACAACGGACGGTGGATCAGGTACT
GGCGGTGATGAAGCTGGCGAAGCGGCAGATCCCGTAGAAAACGGAAATTTCACTGTCCCTGATGATATACAGCCAGGCATTTACTATGACATCCCTAACGAGGCGTATCACGCTGGCCCGG
GGGTCAGTAAATCACAGCTTGATGATATCGCAGATACACCAGCAATTTATCTTTGGCGCAAAAATGCCCCCGTGGACACGGAGAAAACAAAATCTCTCGATACAGGAACGGCTTTTCACTGC
CGGGTACTGGAACCAGAGGAATTCAGTAAACGCTTCATCATCGCACCGGAGTTTAACCGCCGTACCAGTGCAGGAAAAGAAGAAGAGAAAACCTTTCTGGAAGAGTGCGCCCGGACAGG
AAGAACCGTGCTTACGGCAGAAGAAGGCCGGAAAATCGAACTTATGTACCAGAGTGTGATGGCGTTACCGCTGGGGCAGTGGCTGGTTGAAAGCGCCGGATATGCTGAATCATCAGTCTA
CTGGGAAGATCCGGAAACAGGAATTTTGTGTCGGTGCCGTCCGGACAAAATCATCCCTGAATTTCACTGGATCATGGATGTGAAAACCACTGCTGATATCCAGCGGTTCAGGACAGCTTATT
ATGATTATCGCTACCACGTACAGGACGCTTTCTACAGCGACGGTTATCGGGCGCAGTTCGGTGAGATACCCACCTTCGTCTTCCTTGTTGCCAGTACAACCGCCGAATGTGGGCGTTACCCGG
TTGAGATTTTCATGATGGGTGAAGACGCAAAACTGGCAGGTCAGCGGGAATATCGTCGCAATCTGCAAACCCTGGCCGAATGCCTTAATAACGATGAATGGCCTGCCATTAAAACTTTATCA
CTGCCCCGCTGGGCGAAGGAGAATGCAAATGCCTAA
>DLDGEG_10150 hypothetical protein
ATGAAAAATACAACGCCTGATGCAGCAGTATTACAGGAACTAAAAGAACTCACCAGCCGTATATTTAAAATATGCGAGCAAAACAATATGCCGGTAGTTATTGGCTATTCATACGAGTTAAGCA
GAAACGAAGATGGCTATTCAATAAATAAATCAATAACTGCATATGCAGATGAAAAAACAGGAGCATGGGACTCCACTATAGCCGCAGCAGCCATGTTGCTCAAAGTGAAAGACGTCCCCAG
GGAGGTTATTGGTGCATTGAAGAGCTTGTCTGTTGCAAGTGATTTTGCGCGGGCGATGTCTGAGGCCTCAAAAGAAAAAAGCCTGCATTAA
>DLDGEG_10155 Gifsy-1 prophage protein
ATGATACCTGTCATTACACCTCGTTCCGACTGGATGCGCAGTCCGGCTAAACAGCAGACTGCAATAAACAGAAAACCGGGCTTGATTCGTAAAATTTATACTCTACTCACCCAGAAAGGAGAC
CCGACATTAATTAACTGCGCATATTGTCAGAAAGCAATACCGGAAGAGACCGCATACGAATATGAACTGATATATATGCACGGAACGCTTATTTCACGTAAAAATCAAAAATATTGCAGTAAAC
GCTGTGCCAGCCATGACCAGATGGCACATGAACTTTAA
>DLDGEG_10160 DUF1391 domain-containing protein
ATGAAAACAATCGACTTAGGCAACAATGAATCTGTGGTTTATGGCGTATTTCCCAATAATGACGGTACGTTTACCGCCATGACGTTTACCAGAAGCAAAACTTTCAAAACTGAAGCTGGCGCA
CAACGCTGGTTAACAAGAAACCATTGTGAGTAA
>DLDGEG_10165 Putative regulator
ATGAGAAAAGAAAATGAAAAAATTGCCGCCAGTCGGCTTAATGATGAGATCGCAATGCGCCTCAAGGGGCGCAGACAAAAGCTCGGCCTGTCTCAAGGTAAACTGGCTGAGATTTGTGGA
TGGACTCAGTCACGCATAGGAAACTATGAAGCAGGAAGTAGAAATGTTGGGGTGTATGATGCAGTTGTACTTGGTGAAGCACTAGGTATTTCCCCACCCGAACTTCTGTTTGGTGAAAAGG
ACTCCTCGCAGGCATGGCTAAGTGATCAACATAAAAAATTGCTTGAGTTATTCAATCAGTTACCAAGCTCAGAGCAACAACGAATGATTGATCTCTTTGAGGTTCGTTTAAAAGAGATTGATG
ACTATGTTGAAACGTATCTAAGAAATCGCCTTAAAAACTCAACTCAACCACCAGAAAACTAA
>DLDGEG_10170 hypothetical protein
ATGAACAAAATTTCAACATACAGAAAGCAACTGGGGCTATCTCAAAGGCAGCTTGCGACTCATTTGGGATGGATACAGAGCCGTCTGGCGAACTACGAAGCAAATTTTCGCACACCCGGAC
TGGAGGAGTGCCGAAAAATTGTTGCCACACTTAACCATCTGGGATCTCGCTGTGTTCTTGATGATGTTTTCCCGCCTCATGTGAACGATAGCAGAACCATATTAGCGAAGGTGAACAACCATG
ATCACCCCTGA
>DLDGEG_10175 YdaT-toxin domain-containing protein

ATGATCACCCCTGAAACAGCCAGTCAGGCGTTATCGTCATGGCTGGCATATCTACAGATAACCCAGGAAACCGCCACGCAGCTGATCACCCGTGCATTCCTGGAGCAGCCGGCGCGACCGG
AAATAGCGGTTCACCGTATCGAGCGTGACGACGGAACGGTGGATTACGACGCATGGCGCCGTAACCGGATAAACATTTTTCAGCGCTGGCGGAAACGGAAACGGCGGAACACTGCGAG
AAATTCTCTGCGCTGATCCCCGCTATTCTGGAGGCGATCCGCAAAAGTGCGCCGGAACTGCATAAACGAATAACGGCAGGGCAGAGCATTGAATACCTGCTTTCACAGCTTTTAAAAAAACC
GCAGTGGCAAGCGCGGTACTTCTTGGCGCGCCGCTGGCGGATTTTGAGCGAAAGTGTGACGAGGCCATATATGCGTTACAGGCGTTACGTAGCGGTTATCGCCAGCAGTACCAGAGACAT
GACCAGTGAGTAA
>DLDGEG_10180 DnaT domain-containing protein
ATGGCCGCACTTCCATACATGCAGCTTTACATCGCTGATTATCTGGCGGACACCATGCACCTTTCTGCCGAGGAACATGGAGCCTATTTGCTGTTGATGTTCAATTACTGGCAGACCGGAAGA
GCTATCCCGAAAAGCAGGCTGGCAAAAATTGCTCGGATTAGCAATGAACGCTGGGGGGCTGTGGAAGAGTCCCTGAGAGAGTTTTTCATTGATAACGGTACTGAATGGGTTCATGAGCGT
ATCGAAAATGATCTCGCTGCGGTCAGGGATGTTCTGGCGAAAAAGTCGGCAGCAGGAAAAGCATCTGTTCAGTCCAGAAGGAACAGGAAGAAAACGCAGGCTGCCAGTGGAAGTAACA
CATGTTCAACAGGTGTTGGTTCGGTGTTTAAACAGGAAGCCAACAAAAAGGGAACTAATAAAGATATAGATCTAAAAGAATTAAACCCCACACATAACGCGTGCGCGCGCGCGAGTGCTCC
GGTTAGTCAGCCTGGAATTATGGAACAGCCTGTCGTGACTGAACCGGAATACCGGGAAGGCCTGAACGAGCCGATCGGGAAATTCTCAATGATGGATGACTGGCATCCCTCGCTGGATTTC
CGACAACGGGCCGCCCAGTGGGGCGTTGCGTTACCAGAGCCGGAGTATTTACCTACGGAGCTTGTCGCGTTCAGGGATTACTGGACGTCGGAGGGAAAGGTGTTCACACAAATCCAGTG
GGAACAAAAATTCGCCCGTCACGTAAACCACGTCAGGGCAAAGGCGAAACCAGCCAGCAGGGGAGAAAGCCATGCAGAAATCCAGCCAGACAGCACCGCATCGCGGGCAGTACAGCAA
ATCAGGGCAGCCCGCGTGCAGTGGGAACGCGAAAACGGGATCGTCAGCGACGCAGACGGCCTGGCGACTCTGGGAAGTCATGGGGGAAATTTATTCGAACCGATGGACGCAGAAGAAC
GGCGCGGCACCTTCAAAGCTGTGGGTGGCCCAGATTGGGGCGATGACTGA
>DLDGEG_10185 Replication protein
ATGACTGAGCGCCAAATCCGGCTGATTTGTCAGCAGTGTATGGAGCGATGCCGGGCGGCTGAGACATGGCCGCCGGACCTGGCTGAGTTTATTTCGCTGGTTTCTGAAAGCGGAGCTAAT
GCGTTTGGTCTCACAGCCGATGCGGTGCTGGCGGAATATCGTCACTGGCGTAACGAGTCCTGGCGCTACTCCGGCAGTGATAAATATCCGTGGCCTCAGCCGGTTCTGTATCACATCTGCAC
CGAGATGCGCAGAACGGGCGTTGAGCACCAGATGACGGAAGGCGAACTGAAACGACTTGCAGAACGGTTACTGGCGAAGTGGACAAAACACGTCGGTAATGGTTTCAGCATACCGCCG
GTACGCCGTCAACTGGCAGCGCCGCGTCATCCGGCAGGGCCAACCCCGGCACAACTGATGATGGAAGAATTCAGACGGCGTAAGGCGGCGGGAAGGCTTTAA
>DLDGEG_10190 DNA-binding protein
ATGAGCAGAAATTACACACCGGCGCAGAAAGCTGAAATACAGAAGCGCCTGACGGAACTGGTACGAACCCACGGTCGGATGACGTTTGGAGAACTGCGGAAGATAACGGGGTTAACCAT
TTTTACAGCCCGCCACTACCTGGAAAAGGCGGAAAGTTGTGGGGATCTGTATCAGGCCGGGAGAAGCGGTATTTTCCCTTCGGAACGGGCTTTCCGGCTTTGGAAGCAGAAACGTGAAG
ATGCCAGGATTACCCGCTTTCTGAAAACGCCGGAAGGTGTGGTGAGTTCCTACGACCGGACCAGAAACGTTATCTGTACGGAGTGCCGGAACAGCGTGACGATGCAAAGGGTACTGGCAT
TTTATCGGGGACATTACCGGGAGGCGAAATCTGCATGA
>DLDGEG_10195 Cytoplasmic protein
ATGAAAATCGAATACCAGGAAGGAGGAGCTGAGTCCCGTCTGGTTATCACCAGCGGTTTCCTTTGGTGGAGAAAACATATCCATCTGGTTGATGAAATTTTGTTGCGTGCGCCGCAACTGC
GGGCGGTGAGTGAGGGATTTTTTATCGTGACGACGACCGTCAGTGGATTTACAGCGGATGTGCTACGGGCGGAAATGATTGTTGAAGGTATGGGGTACAAGGTGATGAACACCGAAATGA
TACATAACAGTTGCATGGAGGCAGACAAATAG
>DLDGEG_10200 sok antitoxin (CssrC)
GGGTGCTTGAGGCTGTTTGCCTGGAGCATTCGTGAAAGGCAGACAGAGAAAAGCCCCAGTTAACATTCGGCGTCTTGCAGGACGCTTAACATTAAACTGAGGCCACATCTATGCTCTACAC
ACGTAGATTAGCCTCTTACGGACCGAAAGGTCAAGGAGAAGCAGGCTATG
>DLDGEG_10205 Type I toxin-antitoxin system toxin HokD
ATGAAGCAGCAAAAGGCGATGTTAATCGCCCTGATCGTCATCTGTATCACCGTTGTAATGGCGGTGCTGGTAACGAGGAAAGACCTCTGCGAGGTACGCATCCGAACCGGCCAGACGGAG
GTCGCTGTCTTCACGGCTTACGAATCTGAGGAGTAA
>DLDGEG_10210 Phage protein
ATGCTCAAGTTCGTTATCTATGGAGACACCGTGAAAAAATTAAATAAAACATTTAATTGTAAATACGCTGTTATTCGCCGTGATGACATGACAGTAATTGCTGAAATGGATTTTTTTCCTGACTG
CAACAGGTCATTGATGTATCGGGATGGCCGCTATGTCCGGTTTCTGCCGTTGTTGCAAAATGACATCATGGGGAGCGATACCCTGATTAATGAGCTGACTATCAGGGCCGGTTATCATGAATA
A
>DLDGEG_10215 DUF1367 domain-containing protein
ATGGCGCATTTACAACTGGTCAAGCAAACCTCATCAGGGCTTCTGCTCCCGGCGACGCCGGAGAGTGGGGATTTCCTGCGCTCAGTAAAAATCGGTGAGTGGATACACGCCGATTTTAAGC
GTGTCCGCAACTACGCCTTTCATAAACGATTTTTTAAACTCCTTCAGCTTGGTTTCGACTACTGGACGCCAAAGGGCGGCACGGTCACATCGCGGGAACAGAAACTTATCTCCGGATTCGTT
AATTTTCTTTGCGACTCCGCAGGCCAGGAATATACCCCGGCCCTTAATGAGGCGGCGGAACAGTACCTCCATAACGTAGCTACCCTGCGAACCGGGGACGTTGCCCTTCTTAAATCTTTCGAT
GCCTTCCGGGAATGGGTAACCGTTCAGGCCGGGTTTTATACCGAGCATTTTTATCCGGATGGCAGCCGCGGGCGCCGGGCGAAATCCATAGCGTTCGCCAGTATGGACGAAACCGAGTTTC
AACAGGTCTATAAGGCTGTGCTGAACGTCCTGTGGAACTGGATTCTGTTTCGTAAATTTTCCTCTCCGGAAGAAGTTGAAAACGTGGCCGCGCATCTGCTGGAGTTCGCATGA
>DLDGEG_10220 hypothetical protein
ATGAAAATGACATGGTTTCAGCATCCGGCGTGTACCACCGAAGAGGCGGATGAGCTGGTGAAGCAGTACCGGCGCAGGGGGGTAAAGACGGAGCGCAGCCTGAATCATGACTGTATTCA
CTGGACGGTAAGTGCCCTGTTACCGGAATTCGGGCATGTGCCAGTACGGAGGCGTGCGTGCTCTTATCTGAAATGA
>DLDGEG_10225 DUF1364 domain-containing protein
GTGCTCTTATCTGAAATGAAAACTTACCGCAGTAAAAAATGGCTGGCAGCCGTCGGGCAGATTGAGCAGTGCGTGCTGTGTGGTCGGTGGGGAACGCAGGTTGCGCACATGAATGAAGGC
AAAGGCATGGGAATGAAAACGGATGACTGCGCCACGGCGGCTATTTGTCAGGAATGCCATCATGAAATCGATAACGGCAGTCACCTGAGCAGGGAAGAACGCCGGTGTCTGATGAACAG
GGCGATCGTACTGACAGTGATTAAACTTGTACGCATGGGAAAGGTGGTACCGAAATGA
>DLDGEG_10230 DUF1133 domain-containing protein
ATGATTTATCCAACCAGTACCGGAAAACCGGGCGAATATTTTCGACTGAATACACTGGAAAGCGTGTGGATTCAGGGAAAACTCCGTATGTGGGGACGATGGTCATACATCGGCAGCGGTA
AACCCGGCAATATGTTTAACCAGTTACTGGCCTCCAGAAAACTGACAAAAACAGCCATCAATGAGGCTTTACGCCGTCTGAAAAAATCAGGAACAAGCAAGCCAGAGCTGGAGGCCTTTCT
TCGTGAAATGATGAACGGGAAACAAAAAAGCTGGCTGGCGCATTGTACTGATTCCGAGGCCATGTTGATTGACCGCGTGATTGGTACTGTATTAGCTGAGTATCCGGCGCTGAAAAAGTTG
ATTCACCAGCGTTACGAAGGACGGGGAATGAGTAAGCGCAAAATGGCAGAACAGCTAAATGAGCTGCATCCAGATTGGTGCCTGAGGACCTGCAAAAATCGTATTGATCAATGGTTATGTA
CGGCTGAGAACGCGCTCTATGTTCCGCTTTGTGAGGCATATGGTCTGGATGTTACGAGATTTGGAAATTGA
>DLDGEG_10235 HicB-like domain-containing protein
ATGCGATATCCAGTAGTATTAACGCCAGACAGCGGCGGATATGTTGTCTCGTTCCCGGATATACCGGAAGCCCTTACTCAGGGTGATTCGCGGGAGGAGGCGTTGAAAAACGCGCTTGATGC
GCTTGTTACGGCCTTCGAATTTTATTTCGAAGACGGGGAGCGCATACCAGAACCGGGTAACGTGACAGATGATTTTGTCGAAGTACCGGCAAGTGTGGTAGCGAAGGTGATGCTTTTGAAC
GCCTGGATTAGTTCCGGCTTAACTCAGGTTGAGCTGGCGCAACGTATGGGTATCAAAAAACAGGAAGTGACCAGATTGTTTGATCTGAAGCACTCGACGAAAATCGACACGATACAGAAAG
CGCTGGCAGCGCTTGGAAGACGGCTTGAAATATTAGCTGCGTAA
>DLDGEG_10240 Alpha/beta hydrolase
ATGACAATGGATAAACATACGACATGGCTGGCCTACATCTGGGCATTAATCAGCGGCATATGCGCCCAGTGGACGTTAAACGACTATGGCGCGCTGATAGGTATTGTTCTGGGTATTGGTACG
TTTCTGGTTAATAAGCATTACAAAAAAAAATCAGAGCAGGCTCAGGCAAGGCAGGCTGCCGCGATGGAAGAGCGTAACAGGCTAATCGCCCGGATTCTGGAAAAAAACGACCATGACAGC
ACGTTAAAGATGCTGGCGGTATCTGAAATGCCGGAGGGCAGTAATGGCGCTCAGGACAAAAGTTAA
>DLDGEG_10245 Endolysin
ATGGCGCTCAGGACAAAAGTTAAATACGGTCTTTCCGCCGCCATGCTGGCGCTGATTGCCGCCGGTGCCAGCGCACCGCAACTACTCGACCAGTTTTTGCAGGAGCGGGAAGGAAATACG
CTGGTGGCCGTTCGTGATAACGGCGGCGTCTGGTCAGTATGCCGTGGCGTGACTCGTATCGATGGTAAACCCGTTGTGAAAGGTCAGCGACTGACGCAAAGCCAGTGCGACCATTACAACG

CCATCGAGCGGGATAAAGCGCTGGCATGGGTAAATAAACATGTTCACATACCGCTGACCGAACCGCAGAAAGCCGGTATTGCGTCGTTCTGTCCGTATAACATCGGTCCCGGTAAATGTTTTC
CGTCCACGTTTTACCGGAAGCTCAACGCAGGAGATCGTAAGGGAGCGTGTGCAGAAATCCGCCGTTGGGTATATGACGGCGGCAAAGATTGCCACAACAGGGAAAATCAGTGTTACGGCC
AGGTGATACGCCGCGACCAGGAATCAGCGCTGACGTGTTGGGGGATAGACCAGTGA
>DLDGEG_10250 Spanin, inner membrane subunit
ATGAACCGTATAACCTTTACTGCCATCATCCTTCTGCTGATAGTTGCCATAGCGCTGGCGTGGACGACTGACCACTACCACGGTAACGCGGTGCGCTATAAAGACCAGCGCGATACCGCCACT
CACAATCTGAAGCTGGCGAACGAGACAATTACCGACATGACGAAGCGCCAGCGTGACGTTGCCGCCCTCGATGAAAAATACACGAAGGAATTAGCTGATGCACAGACCAGGAATACTGAT
TTGCAGCGCCGCCTTGCTGCTGGTGGCCGGGTGCGCGTCGAAGGACGATGTTCAGTGCCCACCGAGACCGAAACCGCCAGCACCAGCCGCGTGGGCAATGCTGCCACCGTCGAACTCTC
TCCAGGTGCTGGACAAAACGTTCTCAATATCCGCGCCGGGATCATCAGCGACCAGGAAAAACTGAAGTATTTGCAGGAGTACGTTCGCACGCAGTGCAGATAA
>DLDGEG_10255 hypothetical protein
ATGCCGGATATGAAAGATATCGTCACCGACGACATGGTGAAAAACGCCCTCAAATCAGACGCCGTTACCATCGCAGTTAAAACGCAGATTAAATCCACTCTGGATCAGCAGATTGACGCCGC
TGTCGATACCGCATTGACCGATATTCTCGGTAGTGATGCTGATAATACGGTTATGCAGTAG
>DLDGEG_10260 HTH-7 domain-containing protein
ATGGCAAAACCGGACTGGGAGGCCATCGAGACGGCATACCGGGCCGGAGTGATGTCCCTCCGTGAAATTGCGTCACATCATGGTATTAGTGAAGGTGCTATCCGCAAGCGCGCAAAGCGT
GATGACTGGTCCCGTGATCTTAACGCCAGGATTCAGCAAAAGGCTGACGATCTGGTACGCAAACAGGAAGTACGCAAAACGGTACGCACCAAAACGGAACTTACAGAACGCGTACTGATA
GAAGCCACAGCGGAGGTAATAGCCTCGGTACGCATGGAACACCGGGGCGATATTCGCCGGGCCCGGGAACTCACAAACACGCTTTTTGATGAACTTGGTGCGCAGTGTGCTGATGTGGG
GGCGCTGGAGCAACTGGGTAACATCATGTTCGATCCTGACGATAAAGGCCGCGACCGGCTCAATGAAACTTATCAAAAAGTCATCAGTCTGCCTTCCCGTGTGAAATCTCTGAAAGACCTGA
GCGCCAGTCTGAAAACGCTGATCGGCCTGGAGAGGGAAGCCTGGAGTATAGGTACTACCAGTGAACCAGAAAAAACGCCTCTACCAGGAAAAGATACTGATCTGACAACTGATCAGGCAG
CGGAATTGTACAAAAAAATGATGAGTTGA
>DLDGEG_10265 TerL protein
ATGCCTTTACCATTCCCCTTTGACTTTAAAAATCCTGATTATGTTCAGGTTTTTGAATGGCGAATGGAGCGTCTGCAACGTATCAGGAAGGCTCCCGAAACTCTCCCTGCTCTCAGGCAGTTTT
ACCGTACAAACCCGGCGCAGTTCATCATCGACTGGGGCATGACTACTGACCCGCGCAATCTCGATTATGGTCTTCCGGTCACCATTCCTTTTTTGCTGTTTCCACGGCAGGAGGAATGGATCG
ACTGGATTATGGAACGCTCGCGTAACCATGAGAATGGTCTGACTGAAAAAAGCCGCGAAATGGGGCTGAGCTGGACATCTGTCGGTCTGGCCAGTGCGTTATGTCTGTTTAACCGTGAAAT
GGTTATAGGGTTTGGTTCCCGTAAAGAGGAGTATGTCGATAGCACGGTTGATCCAAAAGCGCTGTTCTGGAAAGTACGCAAATTTATAGCAACTCTTCCTGCCGAGTTTCGGGGAGGCTGG
GACGAGAGAAAGCATTCACGTTTTATGAGCGTGGAGTTTCCTGACACTGGCGCGGTAATTAAAGGAGAAGCTGGCGATAATATCGGGCGCGGTGACCGTACTACGCTTTATTTTGTGGATG
AGGCCGCCTTTCTCCAGCGGCCATTACTTATTGATGCCGCGCTTTCCCAGACAACTCGTTGCCGTATCGATCTCTCATCGGTTAATGGCATGAATAACCCCTTTGCGCAGAAGCGGCACAGCG
GAAAAATCCCTGTGTTTACGTTTCACTGGCGTAGCGACCCGCGTAAGGATGATGAGTGGTACCGCAAGGAGTGCGAGAAAATTGATAACCCGATCATCGTTGCTCAGGAGCTGGATCTTAAT
TACCAGGCATCGGCAGAGGGTATCCTGATCCCATCAGAATGGGTACAGGCTGCGGTTGACGCACATATCAAACTGGGGATTCAGCCCAGCGGTCAACGGCTCGGTGCAATGGATGTCGCCG
ACGAGGGGCGGGATAAAAACGCCTGTTCCCTTCGTTACGGCTTCCTGTTGAGTGATGTCCAGGAATGGTCGGGTAAGGGTAGTGACATCTATGACTCCGTGGTTAAGGTCTTCGGCCTGTG
CGATGACTTTGGCGCCGATGAGTTCCGCTTTGACGAGGACGGGTTAGGCGCTGGCGTTCGTGGTGATGCACGCGCTATCAACGAACTGCGGGAAGCTGAGGGTACAGATCAAATTACTGC
CACACCATTCCGGGGGAGTGGAAGCGTTTTTTATCCTGAAAATGAAGCTGTTCCCGGTGATAACGGCAAACCGTCACGTCTGAATAAGGACTTTTTCGCCAATGCCAAAGCTCAGGGCTGG
TGGCATCTTCGCAAATTATTCCGCAATACATTTCGTGCGCTAAAGGGCATGGAGTATGACCCGGATGAGATTATTTCCATCAGCAGCACGATGGAAAATAAAGACAGGCTTTTGATGGAACTG
TCACAACCCACCTGGTCGAAAAATGCCGTCGGAAAAATTCTTGTTGATAAGCAACCTGACGGGACGAAATCTCCTAACCTGGCAGACTCAGTGATGATTGCTTATGCCCCGATGGAAATGCC
CGTCGTAATTTCTGATGATTTTATGGAGTGGATTTGA
>DLDGEG_10270 hypothetical protein
ATGTGGCTTTTTAAACGTAAAAAAACGGTGACACCGCCAGAAAGTCCGCCTGAACCACATCCGATGACGATCAGCGATGAGGTGGTTGCTGAGGCCGGACAAAAACCGCAGCGTGAATTT
GTTCGCCATGAGCCACCGCCGGGAGTCATTCCCGAAGACATACGCAATGCTGTACTGGCAATGGACTCGACCCCCTACGATACACTGAACAGCCAGTATCCTGATTTTGTGTACGGAGGATT
TCCGGGCTATCCGTATCTGGCACTTCAGGCGCAGTTACCAGAGTACCGGCGCATGGTCAGTGTGATTGCCGAGGAGATGACCCGCAAATGGATAAAGGTTAAGGCGGTCGGGGTAGGGGA
CGACAGCCGCGCGCCGCGCATAGCGCAGCTTACTGATGCACTGGAGCGCTATAACGTACGGGATGCCTTCAGGCTGGCGGTGGAGCATGACGGCTTTTTCGGGCGAGGGCAAATTTATATC
GATGTGCGTTCGCCATCGGGTATGTCGGCCTGGACTGACCCGGCGGAGCTGGAGTCCAGGCTGTTTATTTCCGACAAAAAAATCCCGAAAGGTTCCCTGCTGGGGCTTCGTGTTATTGAAC
CCGTCTGGACGTATCCGGGTATGTATAACTCGGATAATCCGCTGAGTGATGATTTTTACCGTCCGTCCGAATGGTACGTAATGGGAAAAACGGTTCACGCCAGCCGCATGATTGATCTGATTTC
TCGCCCGGTTCCGGACATGCTGAAGCCGGCCTATAACTTTAGCGGCCTGTCACTGGTTCAGATTGCCGAACCTTACGTCAACAACTGGCTGCGTACACGCGACAGCGTGGGCGATATGCTG
CATTCGTTTTCGCTGAGCGGGATCATGACGGACATGAGCCAGGCGTTAACGGGGAAAAGGGACTCGAATTACGCAAAACGCGCGGAGCTGTTTAACCGTACCCGTGATAACCGCGGGTTG
TTGATGCTGGACAAGCAGAAAGAAGAGTTTTTCCAGTTCAACACCCCTCTGAGCGGCCTCGACACCCTTCAGGCGCAGGCACAGGAACACATGTTCTTTGTCAGTGCCATACCATCAGTAA
AGTTCGCCGGGCTGAGTCCTACGGGACTGAACGCGTCGAGTGAGGGTGAAATCCGTGTGTTTTACGACACCATCGCTGCACTTGCCACTCGCCTTCTGAAGAAACCGCTGAAAAAGGTAC
TGGATATTATTCAGTTGTCTGAGTTCGGCGATATCGATCCTGATATCACTTTTGAATTTGAACCCCTGCATGAACTGACGCGCGAGCAACTGGCAAATATCCGTAAAACTGAAGCGGAAACAG
ATCAGATTTACGAGAGCGCCGGAGCGGTGACCAATAACGAGGTACGCGAACGGCTGGCTACTGCACCGGACAGCCCGTACAGCGGTATTGACCTGAGCGGAGAAATCGAAATTGACGAC
ACCGAAGAAAATCCGCCGCAAGACCCGAACGCAGACCCTGAGACGGATTTCACCCAACGCGGGGATTGA
>DLDGEG_10275 Putative phage putative head morphogenesis protein, SPP1 gp7 family
ATGCACAACAGCGTGCTTTACTGGCTGCGGGCTGAGTACCGTAAAACAGACCTCGCGCAGGATGCGTCCCCCGTTAACCTGATGCGTGGAGCCATGCAACAACTTGCCAGGCGCTGGCAG
AAAAAGTTTGACGAAATGGCCCTGCGGCTGGCGAGGCGGTTTGCCGGTGATGTTCTGAAAAACAGCGATGCGTCACTGTCCACTGCGCTCCGTGATGCCGGGTTTACGGTTCCTTTCCGTA
TGACAGCGGAGATGAACACCGCACTTCAGGCCAGCATCACGGAGAATGTGAACCTCATTCGCTCCATCCCGCAGCAACATCTCACCCAGGTGGAAACACTGGTCATGCAGTCTGTTGGCCG
GGGGCGTGACCTGAAAACTCTGACCGATGAACTGGAAAAACGCTACGGCATCACACGACGGCGCGCGGCGCTGATTGCCCGCGACCAGAACAATAAAGCGACCTCGGTAATGCAGTCGG
CCAGACAACGCTCGGTGGGCATCACTGAAGGTATATGGCGGCATTCCCGCGCGGGTAAAACATGGCGCCCGTCGCATGTGAAGGCGAACGGTAAACGGTTTGATCTGCGAAAGGGGATGT
TTCTGGATGGTAAGTGGGTACTGCCGGGCGAAGAAATCAACTGCAAGTGCGGCTGGGAGGCCGTTATTCCCGGACTGGAGAAAAGATGA
>DLDGEG_10280 Phage protein
ATGATTATTACCGAAATGCTGGCGTTTGACCGGGCATCGGTAAGGCAGTTCGATAAAGTAGGTCGCCTCCAGATTGAGCGCAGTAATCTCAGCAAGGCGAACGTCTGCGGTTATTTCGGGC
ATGAAATACCGGGGGCGGAAGCGCTGGGACTCGACCCTCAAAAACTTTATCAGCTTTACCGTGACCCCGATGAACTGCGCAAGGCAGTTTCAACCTTCAACAATATTCCCGTCCTGTGCCG
ACACAAACCCGATTATCCGGGCGCGCCCGCGCGCGAGTACCGGGTGGGGACGACTCATGCCAACAGCGAGTTTGACGGTACCTATCTGGTTAACGGCATGTCCATCTGGGACAACTCCGCC
ATCGCGGGGATAGAAACGGATGAACAACGGGAAATCTCATCGTCATATGCCTATGTGGCAGATATGACGCCGGGAACCACCCCCGACGGTGAACCGTATGACGGCGTTATGCGGAATATCGT
GGGAAATCATGTGGCGCTGGTCGGCGATGGCCGGGCGGGGCCGGACTGTCTTGTTATGGACTCTCTCCCTCAGGAGCTAAAACGCATGAAACTGAGTAAAAAAGAAGTGGCGGTGCTTA
CCGCGCTGGGAACCTATCTTGCGCCGCGTCTGGCACAGGATGCGGCTCCTAAGGATTTGTTACGCCTGATGGCGCAGCATAAGCGCCCGGCAGCTATCGCCAGCGCGGTAAAAACTGCCTA
CAGCGAACGGCTGGCACAGGATATGGATATTGAACCGGCGGAGCTGGCGCAACTGATGGAATCAGCAGAAGCCGTGCCGGAGCTGGCCGGGGACGATGATACCGGGTTAACTGACGAG
CCGAAGGCATTTGATACCGACAGCCCGATGGAAAGTGTACTGGCGTTGCTGTCCGGCAAAGTTCCTGATGATGTGCTGGAAAAAATTAAATCCGCACTGGCTCCGGCAACTGACGAAGACC
CCGAAATAAAAGAGGCTGATGTGAAACCCGACGATGTGAAAGTCGATAAACCCGCGATGGATGCGGCAATCAGGCTGGCAACTGACCAGGCAACGAAACGGGCTGCTGAAAATTTCCGC
GCCGTTCGTGTGGCTGAAACCGAGGTGCGGCCGCTGATTGGCGATGTGGTGGCGATGGACTGCGCCGAAGAGGTTTACCGTACCGCGCTGGAACAGACGGGGATCGATATCCAGGGCAT
TCACCCCAGCGCGTACCGCAGCATGGTGAAGTTTGCCGTTGAGCAGAAACAGACGGCTAAAGGTCCGCGTGTTGCGATGGACCAGGCCAGCGCATCGACGTTTGCGGCAGATTTCCCCG
GTGCAAAACTGAAACGAGGTTACTGA
>DLDGEG_10285 DUF2190 domain-containing protein
ATGAATACTTTTCAGACACACATGAACCAGTACCCGGCACCGGGGATTCCGGGGGCATTTGCCAGTGATAACCCTCACGCCTCGTATGTGGCGGGAGAAGGCGCGCTGATTACCGGCCCTG
ACGGACTGGTTATTGCCCGGTTTGCCTGGGTAACCAAAGGCGTTGCCGCCAATGAGGGAACCGGTGCGCCGGCGGGTTTTGTTCCGCGCGACGGGCAGGCTTCTGTTGTGGAATGGCTG

GCTGGCGACTCGAACACTATTTACCCGGGACGTGAATGTACCCTGATGGTATCGGGGGACTTCTGGGCGCTGACCACCACCGCTGCGACGGTCGGGCAGAAAGTTTTTGCCTCCCTGACCA
CCGGGGAGATAGCCACAGGGGCGGCAGGCACCACGATGGCGGGTTTTGTCGAAACCGGGTTTTCCGTTGCCAGCGCTGCGGCGGCGAAAGAAGTTATTAAGATCAGCACCTGGAGCAA
ATGA
>DLDGEG_10290 DUF2184 domain-containing protein
ATGAATAAATTTAAACAGCATTATGCGACGGTAAGCCGCGACTACGGGATTATCCTTCCCGGTGCGCAGGCTTATTTGCCCCCGGAATACGCCGCCGATTACGGACTGGCGATGGACGCGCA
GCCTGCGCTGGTTACCGCGGCTAACAGTGGTATCCCTGCATATTTCACCAATTACGTTGAGCCAGAACTGATCCGCGTGCTGGTGACGCCGATGAAAGCCTCTCAGATTCTGGGCGAAACCA
AAAAAGGTGACTGGACGACACTGTCGGCACAGTTCCCGATTGCAGAATCTGCCGGGGAGGTGAGTTCCTACGGGGATTACAGCAACAACGGTGTTGTGACGTCTAACGTCAACTGGGTAC
CGCGCCAGAGCTATCACTTCCAGACGTTTACCCGCTGGGGCGAGCGAGAGCTGGATATGTACGGCGCAGCCCGTATTGGCTGGGCGGCAGAGCTGAACGTGGCATCGGCACTGACGCTG
AATAAGTTCCAGAATAAGTCCTACTTCTATGGTATTGCCGGACTGGCGAACTACGGTTTGCTGAATGACCCGTCGTTATCCGCACCGATAACCCCGGATACCGTGGACGGTAAGCTCAAGTGG
GACGACAAGGACGGACAGGGCGTGTATGACGATGTCGTGAAGCTCTTTAAACAACTGGTGAAACAGACTAACGGCCATATTGAGCGTACCGACAAAATGAAGCTGTGCATGTCGCCGCTG
GCGGAGGTGAACCTCACCAAGACTAACCAGTACAAGGTTAACGTGTCCGATCTGCTGGCGAAAAACTTCCCGGCGATGACCATTGAAACGGCGGTTGAATACACCTCTGACGCTGGCGAG
CTGGTACAGCTTATCGCGGAGCGTCTGGGGGAACAGGATACAGGCTATTGCTCTTTCACTGAAAAAATGCGCGCCCATGCGGTAGTGACTGAATCATCTGCCTGGAAACAAAAAAAATCTG
CCGGTACCTGGGGGGCGATTATTCGCCAGCCGCTGGCGTATGCACAAATGCTGGGGGTGTGA
>DLDGEG_10295 Peptidyl-prolyl cis-trans isomerase
ATGGCTGAAATGGTAACAGTGGGCTGCAAATTGCCGAATGGTCTGGTGTTGGAAGTGGGACCGGAGCGGGTACAGGTAGCAGGCTGGCGAAATAACGCCGTTAAAATCGTTGGGGGTTA
TGGCCTGACGCAGGTTGAAAAGGCGTTCTGGGAAGCCTGGCTGGCGGAGCACTGCCAGCAACCTTATGTGAAAAACGGCGTTATTTTTGCGCAGGACAAGGCGAACAGCGCTGCCGCG
CAGGCTACGGAGCAGAAAACCGTGAAATCCGGCCTTGAACCGCTGCCGCAGAAAAATCCGGCTCCGGGCATTAACCGCGATGATGAAGTGATGGACAAACCTCAGGAGTAA
>DLDGEG_10300 DUF4054 domain-containing protein
ATGGGTACGGTAACGTTTGACTGGCAGGCATTTTCGGCCCTTTACCCGGAGTTTTCCGCTGTTGGTCAGGTTTCCGCAGCCGCCATGTTTGGTAAAGCGACCACGTTATACCTGGATAATAC
GGACGACAGTCCGGTTACCGACCTGAACGAGCGGGAACAGCTTTTGTTCCTGCTGGTTGCGCATCTGTGCTCGTTACGGGGACTGGGGAGCGGGAAAGATGGACAGGCCGGACTGGTG
GGACGTATCACCAGTGCGTCGCAGGGTTCAGTTTCCGTCTCCGTGGATAATAGCGGCAGTAACGATGCGTCGTGGTGGTATCTCCAGACACCTTACGGCGCTGATTACTGGCAGGCGACGG
CGCCGTACCGTTCAATGGAGTATGTACCGGGCGGTTCACCTTCGCGTTATCCGGGGCATTATTACCGGGGATACGGGAGGGGGCGTCGATGGTAA
>DLDGEG_10305 Ald-Xan-dh-C domain-containing protein
ATGGTAAACAAAGTTACGGGCGGCAGACAGTTCCGGCAGAAGCTGAAACAGGCCGCAGATAACCTTAAATCGGGCAAAAGCCTCAAAGTGGGTTTTCTTGAAGGGGCAACCTACCCCGA
CGGTACGCCGGTGGCGTATATCGCCGCCATTAACGAGTTTGGCGGTAGTGCGATTATACCCGCTCGCGAGCAGACGCTTCACTTTCGCTATAACGAAAAAACGGGAGAAATCGGGCACCGC
TTTGTCAAAGCCGGTAAGGGTAATTTTGCTCAGGATGTGGTTATTCCTGAGCACACGGTCACCATTCCACCCCGTCCTTTCTTCCGTAAGATGATCGAGCATAAAAGCCCCGAATGGGGCGA
AAAAATGGCGACGCTTTTACGGGCGAATGATTTTGATACCGCGACCGCGCTGGTGTACATGGGGGAGCATATCAAAGGGCAGTTGCAGATGTTTATTCGCGACTGGAAAAGGCCGCCCAA
CGCCGCATCCACTGTCCGGCAAAAGGGCTTTAACAACCCGCTTATTGAAACCGGTCATATGGTGAACAGTGTCGATTATTCTGTTGACGGGGCCAAAAAATGA
>DLDGEG_10310 Phage protein
ATGAACCTCCACGGTATTGTTTCCGGCGCGGTACGCCGGGTAAATCCTTATACGGACGCGCTGGTTTATCGCTCGCGTGGGAGTACACAGCAGGCGGACTATTCCCGCGTGCCTGAGTATGA
TGATCCGGTTCCCGTCAGGGTACAAAAACAGGCCGTCACCCAGGAGGATTTACGTCATCTCGACAATCTGAACCAGCAGGGTGTTTTCGCCACACTGTATACCGACGGTAACTGGTGCGGG
CTTAACCGTACCCGGCAACAGGGTGGCGATAAATTTGTCATTGGAGATGAAACGTGGCTGGTGGTTAAGGTACCGGAAATCTGGCCGGACTGGACGAGGGTTATTGTATGTCTTCAGGTGT
GA
>DLDGEG_10315 Tail completion protein
ATGTCTTCAGGTGTGACCCTCTCCGTTACGGAAAGCGATCTTTATCAGGCCCTCGGTGATTATCTCCGGGGGCTTTTTTCTGATGCCGGGATTGAACGAACACAGCAGAACCGGGTCCCGAT
GCCTCAGGGGGACTTCATCACCATGACAGGTATTGATGTTACCGGATTATCCACTGCGGTAGTGACATACTCTGCGCCGGAACAGGCCGGTGAAGGCTCTCAGCATATCACCCGTACCACAA
AATGGCGTTGCCAGCTTGATTTCTACGGGCCTCATGCGGCGGATAACGCGCAGGCGCTGGCAACGCTTTTCCGGTCTGAATTTTCCGTGCAGCTTTTCCGGCAGACCGGCGGGCTGATTTC
CCCGCTGTATTGCTCAGATCCCCTTAACACCACGTTTGTCAACGGCCAGCAGCAGTATGAACCGCGCCGGACGCTTGATATTCAGATGCAGATTAACCCTGTGGTCACAACACCCCTGATGTT
TTTTGACAACGTGATCACCCGGACAACGGAGGCTGATAATGCCAATCCCACTCAGTAA
>DLDGEG_10320 DUF3383 domain-containing protein
ATGCCAATCCCACTCAGTAAAGATGTACAGATAAATCCCGGTGTGCTGGCTGTGGCGGGTAATGCCGTCGATCTTAATGGCCTGTTGCTGACCGGAAATCCACTACTCCCGGTCGGCGGTGT
GGTTCCGTTTTCCTCCCCGGATGATGTGGCCGCGTATTTTGGTGCATTATCCGATGAGTACGCACGCGCGCAGCTTTATTTTCAGGGCTTCAAAAATGCCACTAAAACGCCGGGACAATTGTT
GTTTTCCCGTTTCAATCTTGCCGCATCGGCGGCCTGGTTACGTAGTGGTTCGTTTAAGGGCGTGACTATTGAACAGCTACAAAAACTTTCCGGTACGCTGACGCTGAGTATTAACGGGAAAA
GCGCCAGCGCTGAGGTGAATTTTAACGGTGTCACCAGCTTCGCTGCTGCTGCAACGGCACTACAGACAGCGCTGACCGCGGCGGTGGCAACAGTGGTATTCGATACCACACAGAATGCTT
TCGTCATTACTGCCGCCGGGGCGAAACCGGAGAGCACCACGATAACGTTCGGCAGTGGATCGGCTGCGGAACCCCTGAAGATGACCAGTAATACGGGCGCGGTGATATCCCAGGGCGCG
CCTGTATCTGATGTACCTGACACGATGGTAGCCATTAAGGACGCTTCCCAGCAATGGGCGGGATTTTCCACAGTATCTGAAGTCACTGACGAGCAACACCTGGCGTTTTCTGCCTGGGCAAA
CGGGCAGGGCAAGCGTTACTTTTATGTGGCATGGACAACCAGTGGTAAGGCCAAAGTAAAAGGGGATACCAGTCATATCGCATACCAGATAATCACCGTCAATAACTACAGTGCTGTTGTAC
CGGTTTTCGCGTCTGATGGTAACCGGGCGGCTGCGGTACTGGGGTATGCGGCGTGCCTTGATTTTGTCCGACCAGAGGGGCGCGTGCCGTTCAAGTTCCGCGAGTATGAAGGTCTGGCCG
CTGATGTTACCAGTGGCAGCGATTACGATGCACTGATAGCCGCAGGTTACAACTTCTATGGGAAATATGCGGAAAACAGTGTGGTGGAAGATTACTGGGCGGATGGCACCATTACCGGCGA
TTTTAAATGGCTGGACAGCTTCTGCGGGCAAATCTGGCTGAATGCCAATTTGCAGGGAGCTGTGATCTCGTTATTCAAGTCAAACCAGACTATCCCCTACAACAATGAAGGGCGGGCGCTG
GTTGCGGCATCAATGAGTGACGTTATCCAGCAGTACAAACGCTGGGGCGGTATCCGTGAAGGGGTGACACTGACGGAGGCGCAGAAGAAGCAGATCAACAATGTTGTGGGGGAGGATGT
TTCTTCAACGTTGTTTGCCACCGGCTACTACCTGTATATCGGCGATATGCTGCCTTCTCTGCGGGCAACACGTAGCAGCCCGTCCTGTACGCTCTGGTACTGTGACGGCGGCAGTATCCAGAA
ACTTGTTATTGCATCCACGGAGGTCCAGTAA
>DLDGEG_10325 DOMON domain-containing protein
ATGTCAGGTAACAACAACACCATCACTGCGGCTGATGCCATTATCACGCTGACAGTGAATAACCTGTATCCCTCCGGCGTACAACTTCAGGGATTTGCCGCAGATAACGTTTATGGCACCGAT
CCGCTGGTACTGGCGGAAACCGTCCGCGGTATTGACGGTAAACTGTCTGCGGGATTTGTGTACAGCAACATTATCCAGACGTTTCACATCATGCCGGACTCACCCAGCCGGGATATTTTTGAT
ACCTGGTCAACCACATCCCGGACCAGCCGGGCTGTATTCCGTTGTAATGCTGTCGTGCTGCTTCCGGCGATAGGCCGTAAATATACCTGCGTAAATGGCGTACTCAAACAATGGAAAGCGCT
GCCTGACGCGGCGCGTACATTGCAGCCAGGACAGGCGGTTATCGAGTGGGAAACTATCACTCCGGAGGTTTTTAACTGA
>DLDGEG_10330 SH3 domain-containing protein
ATGGCCCGTAAAGAGAAATTTATCACTATTGATGGTCAGGGGCGGGATAACGGCAAGGTATTTCACCTTACCGAAATGTCTGCCTCGCAGGCGGAATGGTGGGCGATGCGCGCCATTATGG
CGATGGGGCGTGGCGGCGTGGAGTTACCGGATGATGTTCGCAGTATGGGGATGGCTGCGCTGGCGCTGGAAGGGCTGAAAGCGTTGTCAAAAATCCCGCCGGAAGAAGCCCGTCCACTG
CTGGATGAAATGATGGAATGTATACAGTTTGTTCCCGATCCGAAAAATCGTGGTATACGGCGACCTCTTATTGAAGACGATATAGAGGAAATCACCACCAGGCTTAATTTACGTGCGGAGGTA
TTCAGACTGCATGTGGATTTTTTCAGTCCCGCCGCCAGCTAG
>DLDGEG_10335 peptidoglycan lytic exotransglycosylase
ATGGCAGCGGTACTTGATGAGCTGGTTCTGGCACTGGATATAGAAAGTAAGGACTTTACTGCCGGGGAACAGGCTGCGCACGCTGCACTGGACCGACTGACCGCCGCAATGGAGCGGGT
GGCGGATGTTTTCGAACTGGGGCAAAAACAGGCCAGTAATGCCCTGGCGAAAACAGGCAGTGATGCGGATAAAGCTGCACGTGAGACGGAAGCCGCCGGTGAGCGCACGGGTAAGGCC
CTGAAGAAAACAGGCTCTGACGCTGATAAAACTGCCGCGAGTATGGAACAGGCGGGGAAGCGAACCGGTGATGCCATCGCGAATACCGGAAAAAAGGCCGAAAAAACCGCTAAGAAAA
TGGAGGCAGCAGGTAAACGGGCATCAACGTTTTTTTCCGGCATACGTACTCAGATACTGGCGCTGGCAGGCGTCACCCTGACACTGGGGGGAATTAAAAGCCTGGTCACGGGGTTTGCCG
GTGATCTTAACCGGCTGTCAATTTCCTCCGATGCCTTTGGCATGAAAGCGAAACATCTGGACGGCTGGATACGCGCAGGGCAGGCGAATGGCGCTGACGCTGGCGAGATCACCGGGGCGT
TTTCCCGGATTACGGATGCAAAAGCCGCATTCAAAGCCGGAAAGTCCTTTGATCCTGTGTTGCAGGATTTGTTTCAGGTTGCAGCCCGTGCGGGTGTCAGTGTTGATTTAAATACCGACAGT

ACCGAAGTCATCATGCGCAAGCTGGCGTCTGCCTTTCCTCGACTGACAAAGTCAGAACAGACAGCCTACGGTAATGCGCTGGGGTTCAGTTATGCCGGGCAGCAGTTTCTTGGCTCAGGCC
ATGCTCTTCAGGATGTGGATGACTTTACATCCCGTTCGCAGGTCTCCGACGATAAAATCCGGAAAGCCCGCAAATTGCGGGAAGCCCTTGCAGAACTGGACCAGGTATGGACAACAATTGG
TCTGACTATAGGTACGGCACTGATGCCGTATGCCACGGAATTCAGCAAATGGCTGGAGAAACTCGGTGACTGGATGCAGCAACATCCGGAGGAAGTGAACAAGTTTATCACCACATTTCTG
AATAAAGTTGAGTCAGTGGCCTCCTGGGTGAATAAGGCTGCCGGAGAAATGGGGGGCTGGCAGAATGTCATTATTACGCTGATCGGGCTGAAAGTGGCGTCATGGGTACTGGGGCTGACT
AAGGCCCTCAACGGTCCCGGCGGCCTTCTTTTTGCGATAACGGCGCTTTACCCGGTTGTTGACGGGTTAATGACATCCATCGTTGGCAGGAAGAATAAGGACTGGCTGGATTCGCATGGTT
TTTTCTGGGCTTCAGACGGGACTTTCTTTTTCAATAAGAAAGAGATGGAGGAATACCAGGCAAAACTGGATGCCGGAGAAAAGCCTGGAAACATCACCCATGCACAATCACCTACAGTATG
GCAGCAGGGAATGCTGGATACTCAGGCTTCTCTGGCAACCGGGAGGGGAGCAGCCTCCGGGGCATCCTGGCTACAGGATATGCGTGCGACGCAGGAAAAACTCGGTAATGCCATGCAAA
ACCGCCCGCGTCCGACGAAGGCCGGGGAGGCTCTGTTAGGCTGGCTGCAACCGAAACTGTCCCAACTGGAGGCAAAATATAACCTGCCGACCGGACTGCTGCGCAGTGTTGCGATCACC
GAATCCGGTGGTAATCAGTTTGCCGTCTCACGCGCTGGTGCGATGGGACTGTTTCAGTTCATGCCGCAGACGGCTAAGGAATTTGGTCTGAGGGGAAACGATGCCTTTGATCCTGCAAAAT
CCGCTGATGCCGCCGCGAGAAAACTTGGTGGCCTGCTGCGGTTTTTTCATGGCGATCTGGCTAAGGCTTTGGCGGCATACAACTGGGGTGAGGGAAATGTTCAGCGTAAGGGGCTGGCTG
CTGCTCCGGAGGAGACCCGTAACTATATTCCCCGCGTTCTGGCGAATCTGCCCCATCCAGGGGCGGCAATGGCCGTACAGTCGCGTCATCCGGCGCCTGTATCTCAGTCCACCGTAACGGAA
ACCACGCATATCGGGACGCTGAATGTCACTACAACCTCGGACAATGTGAAGGGCATTACCGATGATGCGCGTAGGCGTATCAGGAATTCGGCGCTTGTTTCAGTTTATTCCAGCGGGGTAAC
AGGATGA
>DLDGEG_10340 Phage tail protein
ATGAGTTTCTCTTTCGATAATCTTTCCCTGAATAACTTTTCGCTCAATGAAAGTAACGTACTGAGTGCCGTTCGTGGCGGCGGTGTCCTGGGACTCATTAACAGTGTACTGGCACCGTCATTCG
GTATTTATTACGCATGGAATGATCCGGCTGGTGTTCACCTGAAGGGCGGGAGGCCTTTCTCCCCGGATTCTTTTGTTGTCGTTGAGGTGGGAGCGGAGGCTTCTGTTTCCACCGCCCCCGTC
GAACAGGGAGCCTATACCACCTTTAATAAAATCCAGCGACCGCCGGAGCTGCATGTGACTTTCACTGTAGAGGGGTGGACGGCGTTTTCCGGGGCCGTCCCGAACCTGACAAATTTTTCCA
CCACCTCGCGATCGAATGTGCTGGAAACGCTTGAAATGATGCGTACCACAGCAGGACTTTACGATATTGAGACGCCGGACAAGACATGGACATCCTACGACCTGGTGAAATACGACTACCG
AACGCGAAGTAATAATGGACCGACATTACTGACGGTCAGCGCAGTATTCCAGGCGGTAATGAATACTGGAGAGGTGTCAGTGGGAAGTACGGATAACCAGTCTCCCACGGACAACGATAAA
GCAAAAGGGGCAGCATCGGTTAAAACTCAGCCAGTTACGGCGTCGGTGGCACAACCGTCAGACGCTGACAGACGGAGCGTCACGAACAGGGGGATCACCTGA
>DLDGEG_10345 hypothetical protein
ATGCTGGAAATTGTTTTATCTCCCGTCAAAGCCCAGCAGTTTACGGTGACACTGGGTGCTCAGGTCTGCACCATTCGCCTGAATCAGCGTACTACGGGGATGTATATCGATATTACCGTTAACG
GTGAACCGTGCCTGTATGGCGTGTTGTGCCTGAACAATAACCGGATTGTCCGGTACGGATACCTGCCGTTTCAGGGCGATCTGTTTTTTTCCGACACGGAGGGGAACCATGATCCCGACTGG
CGGGGGCTTGGTTCACGGTACCGGCTCTACTGGCTGTCGCCTGAGGATCTGACATGA
>DLDGEG_10350 Minor tail protein
ATGAGCTATGTACAGCGTGACATTACCGTGGAGTTCACCCTGTCAGACGGGCGGACGTTCGACAATGGTAAGGGCAATATTCTGACTGTTTCAGGAGCTAAATGTTTTGCCACTGTCACGGT
ATATGGCGGAACTGCCGGAACGCAGATAACCCTGTATATCTGGGGGCTGTCTCCGGCGCATATGGCCGACCTGAGTTATCGGGGCGTGTGGCGACCCGCTCAAAGTACGGCCAATGAAATG
CGGGTACGGGCTGGTGGTCGGCTTATTTTCGAGGGAGATATTACCGATGCGTATGCGGACTACAACCAGGCGCCGGATATACCCCTTATTCTGACCGGGCAGGTTAGTTTCAACCTGCGTAA
TCAGACAGCGGCCGATTTCAGCGCGAAAGGTGATGTGCCGGTTGCAGATATCATCCGTGCGCTGGCGTCATCTGCCGGGCTGAAATTTGAAAATCAGGGCGTCAGTCGCAGCCTGTCGAAT
CCACATTTTTCCGGAAACCTAGTACAACAAATGCTGGATGCCGCTTCAGCCGCCGATATTAACATCGACCTGGGGGACGCGGAGAAAGTCACCATCTGGCCGAAGGACAAAGCCCTGGATA
TTCCGGCTGTGCATATTTCGCCGGACCACGGGCTTATCGGATATCCGGTCTATACCATGACCGGCCTCAGCGCCACCACGACATTCTGCCCCGATCTTTTCATCGGTCGGCGGGTCCATCTGG
AATCGTCACTACCTAACGTGACAGGCGATTACCAGTTAACCGGAGTGATACACACCATTACCTCGCGAACCGTGGGCGGTCCGTGGAGCTCCAACTGTACCATGACAAGGCTTAACGATAAT
GGCACAACCACTCAGTAA
>DLDGEG_10355 Gp138-N domain-containing protein
ATGGCACAACCACTCAGTAATCCGACGGACGTAAACAGTGAAATCAATGCGCAGGACTTTATGCTGCGGCAGTTTCTCGGGAAACACGTATTTATCACTCTGGGGCAGGTAGTGGCGGTGG
AGGGGGAGTTTATTGATGTCCGACCGATGGTAATGGGCGTTGCAGCAGACGGTTCCCCGGTTGAGCATGAGGTGATTTATAACCTTCCCGTATGGCGGCTACAGGGGGGCAGCAATGCGG
TGATTATGCCGCCACATGTGGGCGATATTGGTTTCCTCGGCATCTGCGACCGGGATATCAGTGCGGTAAAAGCCACGCGTCAGGCCGCGATGCCGGGATCAAAACGCACTCATAACTACGCC
GATGCCATCTGGTTTGGCGGTGTGCTTAACGGTGCGCCCGTACAGTTCGTGGAATTTGCTGACAACCAGATACGGGTTATTTCCCCCTGGAAAGTGGAGATTTCTGCGCCGGAAGGCATCG
TGAACGCCTCGAAAAGTTTCACTGTTAACTCGCCAAAAATCGCGCTTAACGGGGATGCTGCCGTCAGCCAGGGGCTTAATGTTACCGGACAGTCTGAACTTTCCGGTGGCGCGCAGATTGG
CGGTATTGATTTTGGATACCATGTTCACAGTGGTGTTAAGTCCGGCGGTTCGACCACGCAGGGACCGCAGTAA
>DLDGEG_10360 hypothetical protein
ATGCAGTCACGATCGCTTCTTCTCGACACCGGGACATGGGACATCCTGCTGGATGATACCGGAAATCTTGCCATTACTGATAATCCCCATGCGGTAGCACAGGATGTGGCGTGTGCGTGCAG
TACCTTTCTGGGGGAGTGCTGGTACGACTCAACGTCCGGCATACCTTACTGGTCACGCATCCTCGGACACTGGCCCGGCACGCAACTGGTGAATGCCACCCTGCAACAGGAAGCACTTAAA
CTGCCGACAGTGAGCGCCGCAATTTGCCAGGTCACTGTTGATAAAGCCCGGACAGTAACGGGAGTGCTGCGTATTACAGATACCAATAACGACATTTTTACGGTACTGCTATGA
>DLDGEG_10365 Baseplate-J domain-containing protein
ATGAGTGAAAATAAATCTTTTTCTACCGCAGTACCCGCTGTACGTATTACGGACAGCGGGCTGAACGTGCCGGATGAAGCGGATATTCTGAGCGGCAGGCTCAACGATTTTTCTGGTGCGCT
GGGCGGCGCAATGAGTACCAGTCTGAGCAGTCCGCAGGGGCAGCTTGCATCAAGCGAAAGTGCCATTATCGCGGATAAAAACGATCAGTTGCTGTATATCGTTAACCAGGTAAACCCTGAC
TTTTCCAGTGGACGATTTCAGGACGCAATAGGAAAGATTTATTTCCTGGAACGACGCGGGGCTACAGGTACGACAGTAACGGCAACCTGTACCGGGCTGGTTGGTACGCTGATTCCGGCG
GGCAGTATGGCGCAGGATGAGGCCGGCTATAAGTACGTCAGTCTGTCAGACGCCACAATCGGCGCATCAGGGAAGGTTGATGTGGTATTCCTGAATTTGTCCACCGGGCCTGTCGGCTGTC
CGGCGGGATCTCTGAATAAAATTTATAAGGCAATACCCGGCTGGTCAGGTGTCACTAACGCCAGTGCAGGTGTACCGGGCAGCGACGAGGAAACCCGCGCGGACTTTGAAAATCGTCGGC
GTAATTCAGTTGCCCGTAATGCCCGTAATATTCTGGAAGCCATCCGGGGTGAAATACTCTCTACGGTAGAAAACGTGGTGGATGTTTACGTCACCCATAATCCGAAAAAAACGGAACAAAAA
GCCGGGGTCAGTCAGTATCCGTTAACACCCGGTTCGTTTTATGTTGGCGTGTACGGCGGCAGTCCGGCAGATATCGCGGCGGCCATCTGGCGTAAGGCTCCGCCGGGTATTGATATGAACG
GCGACACAACGTTCACCGTTGCGGATAAGGAGTACGATCCGCCGTATCCTGAATACGTGATCACCTGGCAGACACTCAAACCTGTCAGTCTGCATGTCAGTGTGACGCTGAAAAAAAGTGA
CTATCTGCCCTCAGATATTACCCAACAGGTACAGCAATCTGTGTTGTCCGCGTTTAACGGTACAGATGGTGGTCTGCGGGCAAGGGTAGCCTCTGTTGTCTCCGCAGGGCGCTACTATGCCG
GCGTTTACAAAACCGATCCGGAAAATATCGATATTCTGGGCCTTACTGTGAGTCGTGACGGCTCGTCATGGACAACTGCTGTCACTTTCGGGATAGATGAGATTCCGGTTCTGGATGTGTCGA
ATATCGGTGTGAAACTACAGGAGGCGTAA
>DLDGEG_10370 DUF2612 domain-containing protein
GTGCAGAATGTGGCTGCAACCGTGCTTGCACAGTATGCCGCCAGCCCCCGACTCAATGCCCTCATTAACAGCTTTAACGCAGCACTTTCCCCCGACAGTTTTATCAATGATTTTTATGACCTTA
TCTGGAACATCGATACCGCAGAAAAGTACGGTCTTGATGTCTGGGGAAAGATTGTGGGCGTCAGTCGCCGGCTGACGGTAAAGGACGATTTTAATTACCTGGGCTTCAGCGAGGCCCGGA
TGGACAACCCGGTAATGGATGACCCGCGTCCGTTTAATCAGGCACCGTTTTACAGCGGAAAATCGGTTACCCGGACCGTTGACCTGTCTGATGAGATATACCGGCGGCTGATACTGATGAAA
GCCATGTCGAATATTACTGACTGCTCTGTGCCGGATATTAACCGGATGCTGCGGTTTATGTTCGGAAAAAAACGCCGGGCTTATGTTCTGAATAATGGTGGACTGAGGATGAGTTACATCTTT
GAGTTTGCTCTCTCGTCGGCAGAACTGGCGATTATCCAGTCATCGGGAGCACTGCCGTCCCCGCCGGGTGTTTATGTCTCAGTGGTTTTAAAGGAGACCAGTAATGAAGCTTAA
>DLDGEG_10375 hypothetical protein
ATGAAGCTTAACGATAAACCCCGTCAACTGGCAGTACCCTTTGCGAGTACCGGGGATAAAAATAATATCCCGGACAAGGCGACGCAGCAGACCAAAGAGAGCGGTAACGCGGCGTATGAT
TCGGGTTTTCCTCCGGTAACCATGACCCCTATTTCAGCGGGAGGTATACCGCCACACGGCAAGGATTTTAACGGTCTGATGCACGATATTACCGCAGCAATACGGTACGTCCAGGCTGGCGG
TTTGTACACGTATAATGCCGATTTCGCCGGGGCCATTGGTGGATATGCAAAAGATGCCATTCTCGCCGGAGTCTCAACAACAGCGGTCTGGCTGAATACCATTGACGATAACCTGACCGATCC
GGAAGGTGCCGACAGCGCAGGCTGGGTAAACCTGCTGGCAGATCCCCTGAAGCTGTTTCTGTGGCAGAAAAACAATCTGTCAGACCTTCAGAATAAAGGAACGGCACGAGATAATCTTCA
GGTCTACAGTCAGGATCAGACGGATCTTAAATACCTCGCCAAAGACCAGAACGGTAGCGATATTCCGGAAAAGCCGCTGTTTGTACAAAATATCGGAGCGCTTCCTGCCAACGGTACGGCT
GTTGCAGCGAACAGACTGGCATCACGCGGCGCGCTTCCGGCACTGACTGGTACGACAAGAGGCAGTGATAGCGGCCTGATAATGGGCGAGGTTTACAGTAATGGCTATCCGACAGAGTAT
GGAAATCTGTTACATCTAACCGGAACTGGCGAGGGGGAGATTCTCATTGGCTGGAGCGGGACAAGCGGCGCGCCAGCACCCGCCTATATCCGTAGCCTTCGTGATACCTCTGACGCTGAGT

GGTCCGAATGGGCGATGCTCTACACCTCACTAAATCCGCCACCGGATTCGCATCCAGTAGGGGCGGCGATAGCATGGCCGTCTGATGCTACTCCGGCAGGTTACGCTCTGATGCAGGGGCA
GTCCTTCGATAAATCTGCTTACCCGTTACTGGCTATAGCGTATCCGTCCGGCGTTATTCCTGACATGCGAGGCTGGACAATCAAAGGTAAACCCGCCAGCGGTCGAGCCGTGCTCTCACAGGA
AATGGACGGCAACAAGGCGCACGGACACACCGCGCGGGCGCAGGATACTGATTTAGGGACAAAATCTACCTCATCCTTTGATTACGGTACGAAATCGACCAATACCACGGGCAACCATACT
CACCAGTTCGGCGGTTATATCAATTCATACTGGGGAGATTCCAATCACACCTCATTTCAGCCTGGAGGTGGTGCGTGGACACAGGCCGCTGGCGACCATGCACATACAGTTTATATCGGAGG
ACATGAGCACACCATGTATATCGGTCCACACGGACACGTCGTTATTGTGGACGCAGACGGTAATGCGGAAACCACGGTTAAAAACATTGCATTTAACTATATTGTGAGGCTGGCGTGA
>DLDGEG_10380 Phage tail protein
GTGATTAAATTAATTCTTTCAGCACCCGTGCCAGCAATGGCCGTGGCTTTTGAACATTCTTTTCAGAATACCGAAAATGTGGAAATTATCCCAGGACCGTTTGAAACCATACCGGAATTTGAC
TGCATGGTCAGTGCGGCGAACTCTTTCGGTTTGATGGATGGCGGCGTGGATGCTGCGATAACAGCATATTTTGGGCCGCAGTTACAGGAACGTGTACAGCAAAATATCATCCGTGAATATCT
GGGAGAACAGCCTGTCGGCAGCGCCTTTGTTATTGAAACGGGTAACAGTAAACATCCGTGGTTGGTTCACGCTCCGACGATGCGCGTTCCGCTGATAATCGACGGCACCGATGCGGTTTAT
AACGCAACACGGGCAGCGTTACTGGCAATTTTTCAGCACAATAAAAGCGCCGGGGAAGGCCGGAAAATCAAATCGGTTGTGTTCCCTGCGATGGGGGCCGGGTGTGGTAAGGTATCACC
GGATAGTGTCGCCCGGCAAATGAAGCTGGCGTGGGATGGTTTTATTAACTGTGCCTCGGAAATTAACTGGCAATACGCCAGCGCCCGCCAGGATGCTGTATTCAGCACAACGGCATACTGTC
CGTCAAAGGCGCTTTGTCCGAACGCCAGAACGGAATATATCGGTTTTGGTGATTACAGAACGTATTGCAAAAAATCAGGTAACACCTGCATTAGTCCCCGTCATCAGGTTGATGATATTTATAT
TGGTGCGCATAGCCATGCTGTTTCCCCCGGTACTTATCCCCACAGCCATCACCTAAATACAGAATATTTATCCGGAGTAAAAAATGACGTTTAA
>DLDGEG_10385 tail fiber assembly protein
ATGACGTTTAAAATGAGCGACACCCCGCAGACAATTAAAATTTTTAATCTTCGTTCAGATACAAACGAATTTATTGGCGCAGGTGATGCATATATCCCGCCGCACACTGGATTACCGGCAAACT
GTACTGATATCGCCCCTCCTGATATTCCCTCCAGTCATATTGCTGTATTTGACGCTGAAACCCAAACATGGAGTCTGCAGGAGGATCACCGCGGCGAGACGGTTTACGACACAACAACCGGC
AATCAGATTTATATCTCCGAACCCGGCCCGTTGCCCGAAAATGTCACATCAGTTTCACCAGACGGTGAATACCAGAAATGGGATGGTAAGGCGTGGGTGAAGGATGAAGCTGCGGAAACAG
CGGCCAGACTTCGTGAAGCTGAAGGGACCAAAAGCCGTCTTTTGCAAATGGCATCGGGGAAAATCGCGCCGCTTCAGGATGCGGTTGATCTTGGACTCGCAACAGATGAAGAGAAAAGC
CAGTTCGCCGAGTGGAAAAAAATACAGGGTACTGGTAAATCGTGTTGATACCTCAAGCCCCATCTGGCCGGAAATACCATCATGA
>DLDGEG_10390 Protein HtrL
ATGACCACTATAACAATTGTTACTGCTTATTTTGATATAGGCAGAAGTCAATGGACATCGCAAAATGGGTTTGCTCCACGCATTGAGCGAACTACTGATGAATATATGGACTGGTTTTCCAATCT
TGCTCAACTTGAAAATGATATGGTTATTTTCACTTCACCTGACCTCAAATCCAGAATTGAGGAAATCCGGAGAGGAAAACCCACAACTATTGTTACATTAAATTTCAATAAAAAATTTCGTCAT
ATCAGGAGCCGGATCGCTTCTATACAGTCAGATGTAGCGTTTAAGTTCAGAACTCCCGTAGAGCAGCGGGGGAATCCAGAGTATCTGTCGGCTGATTACGTTTTACTCTGCAATCTGAAAAC
ATACTTTGTAAATCAGGCTATCAGGCAGGGGTTGATCAAAGACGAGATGGCTGCCTGGATTGATTTTGGATATTGTAGAGATTCTGATACCACTAATGGAATAAAAAAGTGGTCCTGGCCCTT
CAATAAGGAAAAAATGAATTTTTTTACGATCAGAAGAGGGCTTAAACTTGAAACACTGGAGTCAGTATTCAACTGTATGTCAGGTAACCATGTGTATATCATCGGTGGTGTTCTGGTCGGAAC
GCTGGAAAAGTGGCAAGAATTTTACCGACTGGTGTGGTGTTGCCAAAAGAAGGTGTTGAGAGAGAATATTGTGGATGATGATCAGGGTATATTTCTGATGTGCTATTATTACAGGCCTGACA
TGATAAAGTTAAACTATCTCGGTAAAAACAAGTGGTTTGACTTGTTCAAGTGCAAGGGAAAGCGAACGATTCGCACTTTTTCTCACAGGATGAGAATATTATGTCTTCACAAATAA
>DLDGEG_10395 Phage-like protein
GTGAAACAGAGACAACATAGCTTAATAATTATCATCGGTTTAATAATAGTCTCATATGGTGTTAATAAAGTTGTTTTCGCGAGAGACTCATCTATCCCATTTCTTTCTACTTTGTCTTTTTTATTAA
TTTCTTTTTATTTATTGAGATGTAAAAATCTGGTACCACGTATTAGTGGTTACTTTCTCATATTTTTACTTTCATCGGAAATTTCATACTTCATCGTTTTCAATGAACAGATATCTTTTGACGTTATC
AGTTCCGTTGTGGAAACAAACCTTATTGAAGCGAAAGGAATGTTTTTAAGTGATGGCATTAAAATTTTTGGAATAGCAATTCTATTAACCTTAGCAATTAGTTATGGAATAACTAAGCTTTATA
AAAACCAGGATGACTTCAAGTGGATTCCAAAACTATCAATCCTCCTGTATTTATTAATAGCGATAATGATAGTAAATGATTTGCGGCCACAAATAAACGATATCAAAATGAGCATGAATGAATCG
CGCTCAACAATAGGCAAACTTATTAAAAGCTATTTCCCCGCCGTTATAGGTGACGTGGCGTATTTCGCCAGTACAATGCTATTAAATGACCGTTACTCGAATACATCAATAATACCAGACTTCAA
CGAGTCTATAACAGGTAAAGCAGAAAGCGGTAATAATACCATTGTTATTGTAATGGGAGAATCTTCGCTATTTTCAAGGTATAGCATTTACGGGTATCCTAAGCTGACAAGTCCGGATTTACAA
AAAATATTCACACAACCCAAATCATGCATTGTCAGAAACGTTCATTCAAGTGCTCCTGAAACAAGGGATTCTCTTGCAATGACATTCTCATTCAGTACGCCAGAAAGTGATACTAACCTCTTCA
AGAATAAATCCATTATAGAAATGGCAAAAGCTAACGGATATAAAACCTGGTGGATTGGTTCTCAGGAACTTGAGGGATTATTCAGTTCAAAATATGGATTTATTGCAAGGAAAAGCGACGTT
GTCAGACTGACGAATGGACACGATGAACATCTGGTCTCAATGCTGACTGACGCACTGGAAGATACGTCTGCCCCCAAAAAATTTATAATAGTTCACCTGCTCGGCAACCATAAGCCTTACCAT
AACTACGATGCAGAAGATAAGAAAGCATTACCCGGAGCTGAAGAATACGATTTAACTATTCATAAAACAGACAGAGTTGTTTCTTCATTGTTCAACGATGTAGCAAAACACAGTAAAAACTAT
ATATTCCTGTATACCTCAGACCACGGAGAAGTAGTCAATAAAGGTCATGGACTTATGAAAGGTAAGGATCAATGGTACATACCTTTTCTGTATAAATCTACCAATGACAAATTCGATTGTTCGTT
CATTGAACAATTCAGAAACAAGGATGGATGGCTGAGTGGCCTTATGAATAAGTATATCCTTTCACGTCTTATTGGTTATACGCTCGATAAAAACATTGTTAATAACGAAATGAATAACGACAGA
GTAAAGGCGGCAAATGAAAAACCCGTTTTATTTAAAGATACAGAATAA
>DLDGEG_10400 hypothetical protein
ATGACAAAATCAGCATCAACCAACAAGAAGCCCCGTAAACAACACACTCCTGAGTTTCGCAAGGAAGCCCTGAGGCTCAATAAACATATTGCGTTGCCGCTCTCCAGTTCAGCCTATATGAA
TCACAGCCTTATGCCTGGCACAACAAGCGACATCTTCTGA
>DLDGEG_10405 Integrase
ATGATCAGTGCCGGAATCAGGAAAAATAGCCCGACCGGGAATATTCACCCGGATGGGCTGACAAAGAAATTTGTAAAAGCCAGAAAAATTTCAGACGTTAAATGTAGTGATAACCCACCGA
CATTTCACGAGATCCGTAGCCTGCTCGGGCGGTTGTACAAAGACGAACGCGGCGAGGAATTCGCTCAAAAACTCCTGGGCCACACCTCAGAGAACACCACGAAACTCTATCTCGATGAAC
GCGATAATAAAGCTTACGTGATGCTCTGA
>DLDGEG_10410 hypothetical protein
ATGGCGCTTATTGAAAAACTAAAAGGAACGGACTCATTCACTGTGTCCGCATGGATTGATCGCTATGAGGTTTTATTACAGCGCCGGAGTCTGTCGGTTAATACCTACAAGATTCGCAGTAAT
CAATTAGCGACCGTACGCGAAAAAATGGGGGAAATGATACTGGCAGAGGTGACCACGCGGCATATTGCTGAGTTTCTGGAGTCATGGATCGCGGAAGGTAAAAACACGATGGCGGGGGC
GATGAGGTCTGTACTATCTGACATGTTTCGTGAGGCAATTGTGGAAGGAAGAATAACAACGAATCCGGTTGAGCCAACCCGAGCACCTGAAATTAAGGTTGCCAGGGAACGCCTGCAACT
GGAAACATATAACGCCACGCGGACGGCGGCAGAATATCTGCCTGTGTGGTTTCCTCTTGCGATGGATCTCGCGCTGGTTACTGGCCAACGCCGTGAGGATATAGTTAACATGAAGTTTAGTG
ATATCGTTGATGGTCGCCTACACGTAACCCAGATAAAGACAGGAATGAAGATAGCATTCCCCCTATCCCTGACCCTTCAGGCGCCAGGGTTACGTCCGGGACGGTTATCGATCGCTGCCGACT
GGTAA
>DLDGEG_10415 hypothetical protein
ATGAAGAAAAAAAAAGCAACCGACAGGCGAACTGGCAGATGAACCAGCAACGCCTGAAGCAGTGGAACAGGACACAACTGAACATCATCCGGACCCACAGCCGCTGGAGAATGAACCAC
CTGTAAGCCAGACAGAAGCAGGCTACCAGAAAATACGGGCAGAACTGCACGAAGCACGTAAAAACATTCCACCCAAAAACCCGGTTGATGTTGGTAAACAACTGGCAGCCGCGCGCGGT
GACTATGTCGAAGACATCAGCGACCCGAACGATCCGAAGTGGGTTCATAACAATTACAGCGCCTCAAATCAGGGTGAAAAAGAAGAAGTGGTGCCGGAGGAAAAACAACCAGCAGCAGA
GCCGGAGGCTGTCACCAGAAACGCGGACGGGACTTTCGATGTATCAGCGCTATTCCCGCCCCCCTCAAACCAGACCGAAAAAACGGAAGCCAGAACAGAAAGAGATGGAGAAACGCCG
AAAGAGAGCAACCAGCAGGAAACGGCTGGCGATACAGGGCAGGAAATTACAACGGACGGTGGATCAGGTACAACTTCATAG
>DLDGEG_10420 Glycoside hydrolase family 19 protein
ATGAACGGCAAGAAAGCTTTCGCACCACGTTCGGCGAAGATCAGGATTACCGGGCTGGAGAATTACACCAGATGTGGCGTTGCACTGAAACTGGATCTAGTGGCGAATCCAGGACAGCTT
GAGCTAGAACGTCATGCCGCCCGATCCGCAGCGTGGCTTTTTGTGACTAAAGGGTGTCTGAAATATTCCGGCGACCTGGTACGAGTTACGCAGATCATCAACGGAGGGTAG
>DLDGEG_10425 Uncharacterized protein RzpQ
ATGAATCTGTTATCTGCTCTTCTGAAAAGATACTGGTTGCAGCTGGTGTTTATTTTGCTGATGGCTGGTGCGTTTATCGCCGGTAATGTCTGGAGTGACAGGGGCTGGCAAAAAAAAATGGGC
AGATCGCGACAGCGCTGAATCCTCTCAGGAAGTCAACGCCCAGACCGCCGCCCGTATTATTGAACAGGGCCGCGTTATTGCCCGTGATGAGGCTGTGAAAGATGCACAAGCGCAAGCCGC
TAAATCTGCTGCCACTGCTGCTGGCCTGTCTGCCACTGTTAGCCAGCTGCGTACCGAAGCAAAAAAACTTGCCACCCGCCTGGACGCCGCAAAGCACACCGCAAATCTTGCCGCTGCAGTC
AGAAGCAAAACAACCAACGCCGACGCCAGAATGCTTGCCAACATGCTCGGAGATATTGCAGAAGAAGCTAAACATTATGCTGGAATTGCTGACGAGCGCTACCGGGCAGGAATGACGTGT
GAACGAGTATATGATTCGGTGAGAGAGTCAAATAATTACAGGAGGCATTGA

>DLDGEG_10430 Lytic enzyme
ATGAAAAACATCATCACTATTATCGTAGCCATTATTATCGTTTTTTATGCAGGTATGTGGTCGCAGAAATTCCTGATGGAAGATGAGTGCCTTGATTCAGGTGGTTCATACAATGAAAATGGAAT
TTGCAATATTGCAGGCAGTCATCAGGATGTTCCCCCTAAGTAA
>DLDGEG_10435 DUF1441 domain-containing protein
ATGGACGGCGAACTGAAGAACCTCAAATGCAATATCTGTCAGCTTGCCGCTATTACAGGGTTACATCGACAGACGTTTGTCAGTCGCCTCTCGGGCGTTCCCCTGGCACTGGGAAGCAATG
AAAAAAACAAGCTGTATCTCCTGACGGATGTGATCCGCGTACTGATGGAAACGCCCGTTTCCCAGGCTGCTGAACATCAGGACCCGAATAAAATGACTCCAAAAGAGCGTAAGAACTGGTT
TGACTCCGAAAAGGGGCGTCTCTGGCTGGAAAAAGAGATGAAGCAGGTCGTCCCGTTGTCGGAAGTCCGTCAACAAATGGCGGCGATAGTCAAGGCCATTACGCAGGTACTTGAAGTCT
GGCCGGATAAACTGGAAAGGGATAAGGGATGGTCTGCGGATCAGCTAAACGAGGCCCAGGATGTGGTGGATGAGGTCAGAATACTGTTAGTTAAGGCAATACAGGAGACCGCAGACGAT
GACGGGGAATAA
>DLDGEG_10440 Phage tail protein
ATGACGGGGAATAAATATGGCTCCGCAGCGGCAGTACGCCGGGAGGTTGCTGAATATCTCAGGCCTCCACGCAGAATGCCGGTAGCGGAAGGAACAAAACAATTTATGTTTGTTCCCCGCG
GTACCAATACGGCGGTTCCTTGGGATGACACGTTAGCGTCTCAGTCCTTCCCGAAATGA
>DLDGEG_10445 hypothetical protein
ATGGGGGAGGTACAGACCAAAGCCCCGCTGGACAGTCCGGCACTGACCGGTACGCCAACGGCACCAATGCCGGAAACCACAGCTGCAGGTATTGAAATTGCCACGGCAGCGTTTGTGGT
TGCGAAAGTGGCGCAGTTGGTTGGTTCTGCGCCGGAAGCGCTGGACACCCTGCAGGAACTGGCTGACGCTTTGGGAAACGATCCGAACTTTGCCATCACGGTACTGAATAAACTGGCGG
GCAAGCAGCCGCTGGACGAAACCCTGACGGCGCTGTCAGGAAAAAGCGCTGATGGTTTTATCGAATACATTAGTTTACGGGAAACGATAAATCACGCCGCCGATGCGTTACATAAATCACA
GAACGGTGGCGATATTCCGGAAAAGCCGCTGTTTGTACAAAATATCGGAGCGCTCCCTGCATCAGGTACGGCTGTTGCAGCGAACAGACTGGCATCACGCGGCGCGCTTCCGGCACTGAC
TGGCACGACAAGAGGCAGCGATAGCGGCCTGATAATGGGCGAGGTTTACAATAACGGTTATCCAACGCAATACGGGAATATTTTGCGTCTGACCGGAACCGGTGATGGGGAAATTCTCATT
GGCTGGAGCGGGACAAACGGTGCGCCAGCGCCCGCATATATTCGCAGCCATCGAGATACCGCCGAGGCTGAGTGGTCCGAATGGGCAATGCTCTACACCACACTAAATCCGCCACCGGATT
CGCATCCAGTAGGGGCGCCGATAGCATGGCCGTCTGATGCTACTCCGGCAGGTTACGCTCTGATGCAGGGGCAATCGTTTGATAAATCTGCTTACCCGTTACTGGCTATAGCGTATCCGTCCG
GCGTTATCCCTGACATGCGAGGCTGGACAATCAAAGGTAAACCCGCCAGTGGGCGAGCTATACTGTCGCAAGAAATGGACGGCAACAAATCGCACAGTCACAGCGCCAGAGCGCAGGATA
CCGACTTAGGGACAAAAACTACCTCATCCTTTGATTACGGCACGAAATCGACCAATACCACGGGCAATCATACTAACCAGTTCGGCGGTTATATCAATTCATACTGGGGAGATTCCAATCACAC
TTCATTTCAGCCTGGAGGTGGTGCGTGGACACAGGCCGCTGGCGACCATGCGCATACAGTTTATATCGGAGGACACGAGCACACCATGTATATCGGTCCACACGGACACGTCGTTATTGTGG
ACGCAGACGGTAATGCGGAAACCTTTGGTCTTATGGACGGCGGTGTGGATGCTGCTATTACGGCATATTTCGGGTCGCAATTACAGGAACGGGTACAGCAAAATATCATCCGTGAATACCTG
GGGGAACAGCCCGTCGGCACAGCCTTTGTTATTGAAACGGGTAACAGTAAACATCCGTGGCTGGTTCACGCCCCGACGATGCGCGTTCCGCTGATTATTGACGGCACCGACGCGGTTTATA
ATGCAACACGGGCTGCGTTACTGGCAATTTTTCAGCACAATAAAAGCGCCGGAGAAGACCGGAAAATTACATCTGTTGCATTACCTGCAATGGGGGCCGGATGTAGTCAGGTCCCCCCGGA
CAGCGTCGCCCGGCAAATTGTACTGATATAG

Prophage 9

>DLDGEG_15945 hypothetical protein
ATGACCTTTCAAGTTATTGCCCTTGACCTCGACGGCACGTTACTTACCCCACAGAAAACAATCCTGTCTGAATCCATACTAGCCCTTCAGAATGCCCGTAAATCTGGTGCAAAGATAGTGATAG
TAACCGGACGACATCATGTAGCAATCCATCCCTTTTATCAGGCATTGGATTTAGATACACCTACTATATGTTGTAACGGGGCATTGCTTTACGATTACGTAGGAAAACGGGTGTTGGCCTCTGA
CACCCTACATCCAGAACAAGCTACGCAATTAGTATCACTTTTGGATGCACACAATGTTCATGGCTTGATGTATGCAGACGATGTAATGTTTTACAGTCAAGTAACTGGACACATTACCCGTACT
GAAACGTGGGGCGGGCTTAGGTGTAGCTATGGGTAA
>DLDGEG_15950 hypothetical protein
ATGGGTAACGCCGTCGATGAAGTAAAAGCCTGTGCCGATCTGGTAATTGGTTCTAATACGGAACCTAGTATTGCTGAAATATTAAATGCTCATTATAACAATGAAACATTGAAGTTTATTCCGTA
A
>DLDGEG_15955 AI-2E family transporter
ATGGTAAGCATACTTTATGACTTAATAAAATCTGGTATTAATTTTTTCACTAAAACTAAAACCATAGAACAGGAAGTAAAATCCGAAAACGCCGAAGGGCAAATAGAAGTTAATAAGATTGAG
ATAGAAAAAACTACAATTCACTGGCGTAATGTATTGGGACTAGTCCTAACACTTATCGTACTATATAGTTATATCATTATTCCATTCTTTGATTATTTCGGGATTGTGTTAATAAATTTACCACTGGA
ACCGATTTTCAAGCTACTTTTTGTACTATTGGGTACACCAATGTAA
>DLDGEG_15960 DUF2642 domain-containing protein
ATGCAGACAGCAATTAACCAAATGAGCCAACATTACGACACACAAACACCATACATTCTAGTAGATAACGTTACGCCTATTATGAATAGTCTACCGTTTCCACGTGCATTGATGGGTAATAAGA
AACTTAAAAAGATTCTCAAAGCACATCCATATAATGACAAAGTAGATAGCATTATGAATATTGCTTTCGAACGTCCACAGCTAGGTGAAGTAGGTGAAATTATCGAATGGTCATTACGTGATAC
TTCTATTCACGTGGTTGTACTTTCGAATGAAAAGGCATTCGTAAAAGGTACTTATATTTGGTTAATGGTAGTAGGTATCATCGAATAA
>DLDGEG_15965 PBECR3 domain-containing protein
ATGGCAAAAAATAATTTAATTCGTGTCAAAAACACACAAGCAGTACAGAAGTACTTAAACAAAGAAGGGAAGAATTTTAATAATGATTTCCGCAATGAAATGATCGTAAAGATGCGAGACAT
TAGCAAGGAACTACAGACGGGTATCAACAATGCCGCCGCAGGTGGTGTAGTGCCATTTACGGGCAATGCAATGTTATATTTCTTCAATAAACGTGTAACGGGCGTTACCTGTACTATTCAGGT
AAAAGACATACAAGCCCAATACCTATACGGATCATTAGTAAAACAAGAAAGTTTAGATAAGTTTATTCCTACCAGTAAGACCAAACTAACAAAGCAAGGTAACATTACGGGACTAAAATCTAA
TCTTAAGAGTGGTAAATATAAGGTAGTCGAATCAAAGGGCGTTAAACGTATCGTAGATACACGCAAAAAGAAAGATCGCGTAATTGCGACCAAAGACACTAAGACACGTAAAATAATTTTTG
ATTTCTATAAGGAAGCAGATAGCCGTATATTGAAAGTAATTAATAATATGCGTGGAGAATATAGCATAAGGAAAAAAATAA
>DLDGEG_15970 hypothetical protein
ATGTGGGATATGTTTTTCAAGGATGATTGGGATATAAGCGAAGTAACAGACGGTAACTATAGTGCATTTGTTTATGTAATTCAGTTCCCAGACGATGGTAGTTTTTATTTCGGTTTTAAACAGAT
TTTTCGACGTATCAAAGATGCAAAGAAAATAAAGGGAAGTACCGTACTAAATGAGTCCGATTGGAAAACATATTCCAGTAGCTCTAAGACAGTACAACAACGTATAGACAATGGCGAACACC
ATACAAAACATATTCTATGGTGTTTTGCCAGTAATACCGAAGCGACGTTAGTAGAGACTGCATTGATTGCGTTATACGGTACGCGTTACGATTGCTTGAATAAAGCGATTATGGCAAAGACCAA
ACTACGTAAAGACAAGGGATTACAACTGGACGTTATACGCCGTATTATGGAGTGTTTTTAA
>DLDGEG_15975 Lysozyme
ATGGACTTAATCACACAATTAAAAATATTCGAAGGTACTAAAGAGTATCAAAAGTATATCGGTTATTACCGAAATGGACGCTTTCAAGTTTATAAAGATCATTTGGGTTATCCAACAATTGGATA
CGGACACCTAATTAAAAAGGGTGAATCATTTCCCAATGGACTAACGGACGAAGAAGCCGAAGCATTGCTAATTAAAGATATTGCAATTGCGGAAAATGATTACCGTACTTTAAATCTTAATCT
ACCTTCCGTTAGTCGATGGCATGACTTTATGATCATGATGTTGTTTCAAGTGGGACTAACTAAAACGCGTGGCTTTAAAAAAGCTTTACAGGCGTTACGTGATGGGCGTTATAACGACGCCAT
TGCAGAATTTAAAAATAGTAACTGGTACAGACAGACACCCAATAGAGTGAATGAAATGATTAGTTACGTAACTAAGGGCTAA
>DLDGEG_15980 DUF2730 domain-containing protein
ATGGAATGGTTAGCAATTGGAGCACTTGCCGCCACCGTAATTGGGCTAATTGGTAAGTTTACCAATTGGACTTTTTCGGGCGTCAAACGCTACGAACATAGGATCGGTATGATTGAATCCAAA
TTAAAATTATTGGAACAACGGACGGAGACTATCGAAGACGATATTTTAGAACTTAAGTTAGTAGTTAAGGAAATTAGCGATATTAAGGTAGAGCTAGCACAAATTAAAACTATCTTAGAGGTT
TTAGAAAGGAAGATTTAA
>DLDGEG_15985 Bulb-type lectin domain-containing protein

ATGGCCGCAGGCTTTCAGGCATTTAATGCACGTGGTGCATTAACAATTGATAGTACCAATAAATCTATTGTTACTAGTCAGGTATTAGGAATGCAGCGTTTAATTGACGTTGGATATTATATTTTC
GGTAATAACAGTATCGGCAACGGTCAAACATTGGGTTTTACTGGTCTTAATCAATGGCCTACTAAAGAAGGTATTCGCTGGTGCCAACTTCTAGTAGATGGTACTTACTGTTTTCCAGGAGCA
GAATTATACGAACAAGACCGAGCAAGATTTATGATTTCCAGTAATACAACGCCGCTACAGTCGGGATATTTGGACGTATTCAATGCAAGCGGTCAATTGGTATGGAGTGCCGCAAGTGCCGG
TACTATGCCCCGTATACAAGATTTCTTTAATGTGCCTGCCGGACACGATCTAGGAACCGCAATTACATTAAATACTAGCTTTCCTAATCCGTGGTTTTGCGTTTCTCAGTGTCCCGGCAATATTTC
GGACGATGGAACGGTAGCGGGATATTCAGGAATATTGATTAGGCGAAACAATGCACAATCTTTTACCCTGCAATATATAAACCGAAACCAAAAAAACTATACTCAAGCAATGGGGAATAATG
GTATCAGAATTGCCCTAGCAAGTGTAACGGGTTACTAA
>DLDGEG_15990 hypothetical protein
ATGGCAAGTGGTTTACAGTGCTGGAATGCTAGCGGTGTATTGGTCGCAGATCTCACAGATTATAATATGCGTTATGTAGGGACTACTACATTAGGAATTGGTACAGGAACTACTACATCATGGA
ATGTTGGGTGGGGCGGTATGCGTCCCACTGGATGGTTAGCAATCGTCCGACAAACTTATAATTCAAATGATTTTTATTGTATTCCCTATAATGATTCATTTGTCGTGCAATATTTGCCAGTCAGT
GGAGTCTATGCACAAACATTAATAATTGATATATACACTTTCGAATAA
>DLDGEG_15995 Fibronectin type-III domain-containing protein
ATGGGTAGTAAAGGTGGTGGAATTTTCGGAGCCATTATTACGGCGGTCGCAGTATTCGCAGCAGCGTATACGGGCGGTGCATCACTCTATGCCGCTGCCGCATGGGGTGCAGCCGCAGGTG
CACTAAGTTTTGTTGCATCTAGTGCATTGGCACAATTGGGTACTACGGGCTATGACGACGCAGCAACGAGCGTAAGCCGCAGTACAAGTCCCGTAACAGGTCTACCCGTTCTATTGGGTGGT
GAATTGCCCCACAAGAACGGCGTAAGCAATGGTAGCTTTATTATGTGCGGTACGGTCGTACCGTGGTACAACGTCAAGGATTCAGAATCACAATATCTTTTCACCGAACACGTAGTAGCATTG
GGCGGTACTGAAAAATGGATCGAACAGATTTATATTGACGATGAACCTGTACTAGTAAATCCAATTAAAGCCGATGGACGCGTAGCAACCGAATCTATTGTTGCAAAATATCAAAAGTACTTA
CAATTGGAAGTACGTTTTGGGGGATCATATAGTAATAGTAAATCCCTACCAATGGAATATGCCGGATCTCGCTGGAATAATAATTTTCGTGGCGATGGCGTAGTTAGTATTAGTTGCGTTATCAA
GAAAACCCAAGATTCATTGGAAGACAGTATTTTAGTCAATGACAACTATATTATGAAAGTTGAAATGAAAGGACTAATGATTACTGACCTTACCGATATGACCCGTAAAGCGTCCAGTAATCCC
CCGTCACAGATCTATGAACTATTGACAAATACACTTTGGGGAATGGGCTTAGACCCTGCATTAATTGATCTAGATAGTTTCCGTACTACGGCACAGTATTGTAAGGATATGGAATTTTATAGCAA
TGGCAACATGTCCTATAACGATACCTATAAACAAACTATCGAAGCCATTCTACAGACGTTTAGCGGATTGCTTTATATCAATGCAGGTAAAATTTGTTGTGGTGCGGATCGTAAATCACTTTCAG
TACATACATTCGACGAAACCAATATTACGGGATCATTGAAAGTAACAACCAGTGGTAACACGGACTATGCCAATACAATCGATGCAAAATATACTGCCGTAGGTAATAACTACGGAAATGATGT
AGTACGTTTTCCAAGTGACATTAGCAATGACGATGTAATTAGAAGCGACGGTAGAGTAATTACACAGGCACTAGATTTTACATGGATCTATGACCAATCGCAGTTAGCATTTTTGGCAAACCG
TGAATTACTTAAAATGAAGTACGCACAAAATACCATTACCTTTACTACTCCCGATGCATGGGATCTACAGGTATGGGATTGCGTAGATATTAATGTTAAAGAATTTGATATTAGCGGAAAGTACC
GAATAGTAAGTAAAGAAATTAGCACAGCACAAGATACATTAGGTTTTATCAATCTAGTATGCGTCCAGACCAATGACGGCATTTATGACGGCAAAGATCCCGGTATTTGGACACCAGACGGAT
CTATTAGTAGCGTGATTGGTGTACTACCGCCAAGCAATCTAGAAGTAGTTCGCCGTGGGAACGTTGTAAGCGGGAACATTGTAGATCTAACGTGGACAGCTAGCCCCGATGGTAATTTACGT
GGCTATTACATCTACTACCGCAAATCTGGTAACAATGCGTGGACGTTTGCAGGAACTACGACCAAGTACGAAATAAGCTACGCTCTATATAGTCTACAGTCTGACCAGAAATACGACTTTGCA
GTAGCGGCCTATAACAACTTAGGATTCGTAAGTAGTAAAGTAACGGTAGATGGTTTAATTCCACAATATGATTTTGCATTACCTGCCGTAACGGGTATCAAACTTTCGAATAGTACAGAATCGG
CCTATGTAACCGATGCACCAGATTTTAACATCGCATGGGACGATCAAAGCAATCTAGTTATTAATGGGCGTCCATTTAGTGAGTACTTAAAATACTATGAGATTCGTCTATATGACGGTGAAGCA
TATATTAAATCTTTCTATTCACCTACCAATGCATTTAATCTAACACTTGCACTAAACGAAACAAAGATTCGTAAACCAACGTTAGGTGTAATTGCACAGGGATATAGTACAGGTACATACTCACCA
GAAGTTAAAATTACCGTAGAAAATAAGCAATGTAAATTAGCCACTGGCTTTACTATTGCAGGTGGATTCGGGAACTTATTCTGTAGTTGGAAACAATCGGACGAACGCGACTATGCAGGTGC
AGTTATTCAATTAACAAGTGGTTTAGTAAATACTCAGTACATTTCCAATAAACCAGAATTCGATAGTATTCCAAATATTCCCGACGGTGAATATAAAGTTAAGATGGGCTTTTTCGATGTATTTGG
CATGGATAACATACAATATACTGCCGAACAAACAATTAGTATCAATAGTAAGTATCAATTTACCGAAGAAGATGCAGCCGATATTAACGATATTCTTGATTTAAATAATCGTCTAGATGATATTCTT
TCGAATGCAGTTAATGAAAGTAATGAATATACCAATACAAAGATTGCGGTACTACAAAATACTATCGACAATGACGTAACGGCAAAGATCACCGAACTAAATCAAACCATCGTCAAGAACGAA
GAAGCCACAGCACAGCAAATTACACAAATCAAATCGACCGTAGACGGGAACCAAGCCAGCGTAAACCAACAACTAATTACAAAGGCAGATAAAGGTACAGTAGACGCACAATACAGCCTT
TCAGTGCAGGCTAACGGTACTGTAGCGGGTATTCGTCTAGTAGCGTCCGAAGGTGCAAGTAATAACAGTGCAATCTATTTTGCCGCCGATAAATTTGTTATCAGTGGATCGGATACCGCGACT
GTAGGAGGACAAGCACCATTTGCATTAATCAATGGTACTACTTATCTAAAAACGGCAATGATCCAGCAAGGTAGTATAGGTACAGCTTATATTTCCGATCTAAGTGTTACTAACTCTAAGATTGC
AAATGCCAGTATTAACAGTGCCAAGATTATAGATGGCGAAATTACAAACGCGAAAATCGGGAACGAAATTTATAGTAATAACTATGTATGGCAACAATCAGGATGGTATATAGGCAAAAACGG
CGAAATGTACATTAACGGTAGTGGCGGTACTGGACGTATGACAATCAATAATAACTTGATTCAGATATTCGACCAGAACGGAACACTACGCGTAAGAATGGGCTTATGGTAA
>DLDGEG_16000 Ornithine carbamoyltransferase
ATGAATTTTAAAAACAGTAATGAACTATATGAAATATGCGAAAACTCACTTAATAAAGAATATCGTTTCGGTAGCAATGATTGCAATATTCTATGCCTACGTGTACTTGATCTAAAGCATGGTACA
GATTGGGCAGAAGTAGCTAACTACAATTCCGTAATTGACGGTGTACGACAATTAAATGAATTAGGTTTTAAAAGTACACAAGAAATTATTAAACAATATGCCGATGAAACAAAACACCCAATC
GATGGTGATATTTGGCTAGATACTGAAAACCCCTTACTAATGGCAGTGGTTTTTAGTAATCGCTTGATTGGCGTAAATGATGAACATACACAATTTAAATTAATTCCCCTGAAGGATCACGGGA
AGTATTACAGAATAAGGAAATAA
>DLDGEG_16005 DUF2163 domain-containing protein
ATGAATGCAGGAGTTTTTACCAATCCCGATCTATTGGAATATTGGAATGTTTTTAGAGGTGGCAATAAAAAACAACTAACATTAACAGAAGTACTTTCTATGGGTATACATGTTAAATGTTTCGA
CGTTATTCCAAAGGCAATTGATTCTATTCACTGGACAGATGGACTAGGGGAAGTAACGCTAGGTGGTACTTTATATGTACCTTTTCCCGACTTAATCACCGATTCATTACCATCATTTACCGAAG
AAAAACAAATTACCAATACAAATATTAATTTCAAGATCAGTAATGTTTCCAATACTACACGTATTCTTGCATTGGGCGGGGCGTTTAAAGATGCGAAAGTAAATATCTATCTTGCGATTCTTAAC
CCCGCTACGGGCGATGTACTGGACTACGATCTAATGTATTCGGGCTTTATCGATTACATCGAAGCCGAAGCCGATCCAATGAATGAAAAGAATGAATTAACAGTACAGTTAAATAGTGTATATA
AACAACTAGACCTACAGACCCGTACACTATGTGCAAATAGTGTCTATCAATCTTATTTTCCGGGCGATCAAATTATGTCCCTATTGGGCGTAGTTAATAGTGGTCAAACATGGAAGTACAAATAA
>DLDGEG_16010 Structural protein
ATGGCAAACTTAACATTTTCAAATAATATTAAATTATCGGACTTTACGTTATCGAGTAAATCACCACAATATAGTAATCAATCGTGGACGGGTGCACTTATTCAACGTAGTACGGGCGTACAGTG
GTATACATTTAATTTCACACTTAATTTCAACCAACGCGATAGACAGGAAGTTCTTGCATTTATTGCGGAGTATTCACAGGGCAAGCTGTTTACTATTCCATTGGGACACCTATCGACCTATAAAG
GAAAACAAACAGGTGCAGTAAGTGTAAAAAATGATGTAAAACGTGGTGTATATAAATTTACTACGGCATCGGCACAACAACTTGAAGTAGGAACGATGATTCAATTTGGTAATCATAAAAAG
ATTTACCAGATCGTAGCCAATACAGGTACAGAAGTTTCTATTTTTCCCGCACTACAGGCCAATATTCAAGCCAATGAAACAGTTTTCTACAATGGCCTTGTAATTGAAGCACGTCTAGACGTAG
ATAACGATTTTCAAATGCCCGTAACGAATCTAGTAGCAATTACTTTTAAATGTACGGAGGTTGTACGCTAA
>DLDGEG_16015 Tail tape measure protein
ATGGCAAGAAAAAAATGAAAGAATTATAGTAGAGGTTGACGGTGATACTACTGGCTTGCAAAAGAAACTAAATCAAGCCGAAGATTCTATAGGTAAATTTGGTAAAAAAGCGGGTGGATCAA
TTGGTGGTGCTGCCGATTCTATTGCAAGTGGTTTAGGCCGTATTAGTGGTGGTTTCGTTGGTATTGTAGGTGCAGTGGGTGTAGCTGGTACTGCATTAGCTGGATTGGCAATAAAACTAAATG
ACAACGTAAGACAACTTAATCAATTAAGTATGCAAAGCGGATTGACCGTTACCGAGTTACAGAAACTTGATAAGGCATTCTATCGTACTGGTGTTAATGCCGAGAAACTAGCAGATATAAACC
AAGATACCTTAGATAAATTGGGCGATGCATTTGCAAATGGTGGTGCTATTTCCGATGATATTAAATCCGTAGGATTGGATATTAATGATTTTACCAAATACCTAAATCAAGCCGATGGTGGTATTA
AAGCCGTAATCGATACTTTCTATAAATTGAAAGATGCAGGAAGGACTACGGGCGAAATTACATTCTTATTAGAAAGTATGGGTAGTGATGCTAGCCGCCTAGTATCAGTACTTAATGAAAGTGC
CAATAGTCAAGAAGCATGGAATAAGATTAATAATGAATCTGTAATTATCAGTGAAGATACCGCCGCAGCATTCCAGAAATTTGACGATAATTTGCAGAGTGCAACCAGAACGGGGCAATCGTA
TCTCTATGACTTCATGACACCAGTTATAAAGGAATTTAATGATCTTTATGAATTGGTTAATAAGGATTGGACGGGAAGCGATTTTATGCAAATGCTATACCGTGGTGCAGATAATTTCGTATTGG
GTGGTAAAGGTGCTATTCCAGACTTTCTAAAGAAATTGTTAGGTAAAGATTCATTAGATTCTGCATTTATTGCCGGATCAAATGAATATGATTTCGTAAATGCTTCACGTGCAGGATATGAAGC
CAATAAGGGTAAATGGAAAACTAACCCCCGTAATACTGGTACAGCAGATCCAAAAAGTGGATGGGGTGCTAAACAGGAAGCAGAGAAGGCCGCAGCAGCCGCTAAGAAAGCACAGGAA
GCCGCAGCACGTAAAGCCGAGGCCGCAGCCAAACAAGCAGCAGCGAAGCGTATACAGGCCGAGAGAGCGTTAGAAGTGGCATTGTCGCAAGTAGCAGAGAACGCCGGAGAACAACGC
CTACAGGTATTCGACCGCCAACAAAACGAACTATTGAAGACAATCGAAACTACTGCAAAGACTGTAGGTAAAACTGCATCCGAAATTGAAGCATTACTAGATCGTGCTAGAAAGTCCGGTGC
ACGTGCACGTACTGAATTAATCGATAGCATGATTGGATATACTGACCCCAATCAAGATTTAAAAGACCAAGTTAATATATCTGGTATGGGTATTAATAAAACCCAATCTAATTTTTTATCTGACCA
ACAAGCACAGCGTTTAAATGGTGATAATCCTTTTGCCTATGACAATACACAAAAGCTATTGGAAGAAAATAAAAAACGTGAAGAATTAGAACTACAGTTAAATGCCAATCTATTACAAAGTAC
AGAAGACTTTGAAAAACGTAAGGCCGATATACAGGCACGTTATGCAGCACAGGCAATGCAGATTGCACAGCAAAACACCAACGCACAGATGAATATATTAGCTACTGCCGCTGGTGATATG
GGTAATGTACTTGCAGGTGTATTTGGTGAAGGTAATAAAGCCGCTGCCGCAGCCTTTGCGATCCAGAAAGGGATCAATATGGCACAAATTATGATGAATATGCAGGTAGCACTATCCGAAGC

ACTTGCTACGCCATTCCCCGCAAATATTGCCGCATACGGTCAAATATTGGGTATGGGTGCACAGCTAATTAGTACAGCACGTGGTACTAACATCAGTGGACAATTCCACGGCGGGGTAGATAG
TCTAGATCCGCGATGGATAATAAATCATTTGTACTAA
>DLDGEG_16020 Phage protein
ATGCTACATAAGCGTGGACTATCCCTAGAAGAAATAGACACCATTGACCCTGATATTTTTAATGCCCTTTATATATACGACACCTTAATAGAGCCAAACGGGGCAAGAATGGAAATGATCAAGT
ACGCAAATCTTTGTAACTTACTCTTAATGACTTCCCAATCAATAACCCCCGAAGCACGTAAAAAAGCAAAGGTTAGTGATTGGGATTTCGCAGATCTTCTAAGTGATGTTTCGTTAACAATGCG
AGAAAAAGCATTAAAAAGGGAAGAACAAGAAATAGAAAACAGTCGCAATAATATTAAATCTATTGGCGATATGATCAAACGTCAAATAAGCAATGAGGGCAAAAATGGCAAGAAAAAAATGA
>DLDGEG_16025 hypothetical protein
ATGAATCTACAGGCACTAAAACAAGCATTAAAACCAAAACTAAATGAAACTAAAATTGACGGATTCACGGTATATATTCATCGTCCATCAATTAAAGATGCAAGTAATTGTACTACCCTAGAAG
CCGTTCTAGTTTATTGTGTTAAGGATGAAAACGGTAAACCTATTTTTGCACTAGAAGATGATAACGATCTAATTAATGTTTCGGAGATTGATCAAGTGCTCGCCAATGAAATTTACATTAAGGTA
CTTGCCCTAATTTCTACCGAAGACGAAGTAGATTCTACCGAAAAAAAAATAA
>DLDGEG_16030 phage tail protein
ATGGCAACTATGGATATTTTTGCAGGTGCTAACCTTACTGTAGAAGTAGGCACACATACCCCAGGATCTAAAACCCCCGCTACTGATTTCGAAGAAATTCCAGAAATTGGTGCATTCCCTACCG
TTGGTGCGGAAAACGTAGTAATCGATGTAGTTACTTATAATAACGCATATAACCGTAAACTATTGGGTACTAAATCCGTACCAGATATTACGCTAACCGTTAACTACCTACCCGATAATAAGATTC
ACCAAAAACTATTGGAACTAGAAGAAAACCCAACAACGTGCACAATTCCGTATTACTTATTATGAAGATGCAACGCATACCCAATCATATAGTATTACCTACGTAGCTTTCGTTTCTTCTTCCGTA
ACTAGTGGTGATAAAGACCAAGTAGTAACACGTGATTTCGTTCTAGCCGTCGATGGTGGCCCGATTGAATCTAAAGTAACAGTAGTACCAACACCATAA
>DLDGEG_16035 Tail fiber domain-containing protein
ATGGCAGACATTTTTAACGGTAAAGACGTTAAGGTATATTACAACGATGATACGGGAAATAGAGCCGTAATTACTGGTGGTAATATTCAGATTAACGAGTTAGCCGCATACCCATCTTTTAGTA
TGGGAACCGAAGTACAGAAGATCGAAACATATAATGATGAATATAGCAATGCCATCGAAGGACAGAAAACCGTAGATAATGTGACGCTAGTAGTTAATTATATTCCTACCGATCCAACGCATG
AGTACTTAGATAAAAAATACGATAACCAAGAAGAATTCCAAATTACTATTTTTGTCAATGAAAATAACGAAGCCGGACGTTTAGAAAGTGTAATGCTAAATGGTGTACTAGGTAGTCGTATGCT
AAATGGTGATAAAGATTCTGTAGTTACTATGACCTACGATTTTGTACCTACCGAAGTAATTAGCTATGCACCCCGTGATATTCCCCCAGTATTACGCCGTGGTGATTTCGGCGTAGGATCGGACG
GTAGTATAGACTATCCGCAATATCAACCAGATCAAGCAACGGGCAACGCCTTTGTAAAAATTTCTGCATCCGATATTGATAACCCAAGTGGTGTAGATCTAATGGGTATTGAACTAGTAGATCA
ACAAGCAGAAAACACCAATATCATGATGACAACAACGGGCGACCTACGTTTATATGCACGTAATGCTACTACGCCGTGGACACGTCTATATACCAGTGGTGAAGCCGATACACGTTATCTAATT
AAGTCCAATAACCTAAGCGATTTAACTAACTTTGAATCAGCACGTAGTAATTTAAGCGTATACAGTAAATCCGAAACTGATAATAAATTCATGATCGGCCCGAATAACTTAAGTGAATTAACTAA
CGTTGTAGTAGCTCGCCAGAAATTGGAAGTATATAGCCAATCGGAAACGGATGCTAAATTTCTACAGGCTGCAAATAATCTAAGTGATATTGTCGATACTGCAATTGCCCGTACCAATTTAGGA
ATTAATAGTACTCTTGAAAATGATGCAAAGTACTTACAAGTAGAAAATAATCTATCCGATGTTAAGGACGTTGCAACGGCACGTACTAATCTAGGTATTAACAGTACAATCGAAAACGATGCAA
AGTACTTGCAGGTATCAAATAACCTAAGTGATGTATCCGACGCAGCCGCAGCACGTACCAATATTAGTGTTTATAGCAAGGCCGAAAGCGATGCTACATACGTCCCTAAAACAACCACAGTCA
ACGGACACGCCCTAGACTCTAATGTAACCGTATCTAAGGCCGATGTAGGCTTAGGAAGCGTTACAGACGACCCACAGCTAAAGATCAGTGCAAACCTAAGCGATCTATCGAACGTTGCAAAG
GCACGTACTAATTTAGGTATTAACAGTACAATCGAGAACGATGCAAAGTACTTGCAGGTATCAAATAACCTAAGTGATGTAGCGGACGTTGATACTGTAAAACAAAACATTGGTTTAGACAGA
TTTAAACAGTCGCCATCCGAAACTATGATATATCCATCTAGTGGGCAAAATCCTTATCGTATCACTATCAGACCTAACGGTGATTGGGGAACTTGGCGTGATGATACTGGAACATGGGAACCAT
TAAAGATTGTTGCCGGTGGTACTGGTGCAACAAATAAAAAAGACGCCAGATTAAATCTTAATATCCCAGCAGCTTATAAAATAATTCCTGATGGAACTAATATTCTTGGTTGGTTTATAGAAAA
CAATGAATCAGGATTTTTCTCATCTGGAGAAAATGTAATTAATAAGCCCGCCGACGGTCATGGTTGGTGGACTTATAATTTTAAGATACATCTAAGGAATCAAGAAGGAAAACCAGAATTCGG
AGTTGTAGAAGCAACGTCGGCAGCAAATATCATGTACATCATTGTGCTTACAAATGGTCAATGGCCGCATGGATGGTTTAAAATTGTTCGTGAGAATGATAATGTTCAATTGCGAGACTTGAA
ATTAACACAATATGATACTGGGTTTAGCGGTCATCTTGAACTTTATAACATACAAAACAATAATCCAAAAGGTCTTACGCAACTTTATAATGAATTTCAAGACGGTGTGCTAAAGACTACCCTTC
GCACTGGAAACCTAGAAAATAACAGGAACTCGTATTTGCAGTGGGATGAAAACGGATCTCTTTGGGGTGTTGTTAACATCCTTTCCAATGATTTGTATTTCGGCCCGCATAGCGTTAGAAAG
CTTCACACTCGACACGGAACTATGCTTGTTGATGGTACGGCAACTCCATATTATTACACATTTGGCAATCCTGATGGTAGACGTAGTGTAACTGAGTTCGGAACTGTAGAAGATGGATGGATAT
TTTACGGACAAGTAAACAGAGATCTATCTAAACAGCTCGATGTAAATGGTGTTGTAAATGCATCGGCATTTAATCAGGCATCCGATAGAGATCTAAAAGAAAATATCGAGGTTATTAGTAATGC
TATAGATAGAGTGAGAGCTATTGGTGGATATACTTATACATTGAAGGAAAATGGTATGCCACATGCTGGTGTTATAGCTCAAGAAGTAAGAGATGTATTACCAGAAGCAAGTGGTTCTTTTACT
AAATATGTTGATTTACCGGGGCCAACACAAGATGGTACTCCACTTCGTGAAGAGGAAAGATTCTATTCAGTTGACTATGCCGGAATAACGGCACTTCTTGTTCAAGCCTTCAAGGAAATGGA
CGAAAAGATAACCAAACTTGAGGAACAACAAAAACAAATCGATGAACTAAAAGAATTAGTACAGAAACTTTTGGACAATAAATAA
>DLDGEG_16040 Phage tail protein
ATGAATCTTACCGAAAAGATCAAGGCAAAAACGATAGAAGTACTTGAAAATATCCAGCCCGTCACACCAAGCCTAACTACAGGCCGTCAACGTGATGTATTTTCCCTTGTCACATATGACTATC
AATTAGAATCCGCAAACTATGCAGGGAATAGCCGCCTAAAGGGGCAATTCGATATGCAATTCCTAGTTAGCCCATATACAGGTCAGAAACAGCCGCTATCAAGCTACGACGAGATTGTAACGT
ACTTTAATAGTAACTATGCACCAGTTTTTAAAGCCTCTGGTATAACCGTTCTATGGGTTAATTTCGGTAACAGTACATTGGTAACGGATAAAACTACAGGAAGTAGTTCTATTGTTTTCACAATA
AATATTGAAGCAATAGAGAAAACCAGATAA
>DLDGEG_16045 Nuclear transport factor 2 family protein
ATGAGAGCATTTAATAGTATCCAGCAGGATATTTTCTTAAATGCTTTCAGTCAAAATATAATTCTAATCTCTAATGGGGCGGGCTTTCGCGGGATCGTGGAATCTCGCCCCATTGTCATTGATGG
ATCGGACGGACTAATAGAAAGTACAGAATTATATTTCACTGCAAAGAAACAAGAAGATATTGTTTTAGGTAGTCAAGTTTCATTAGTAATTGACGATGCACAGGCCGAAGTAATACCAAGCCC
ACTAAATACCTATACCATTTATAACATTGAAAACGATTTAAGCGGGATGATTAATTATTATTTCCGCATGGAGTAA
>DLDGEG_16050 HK97 family phage prohead protease
ATGAGCAAGGAAGCGAAACCGAAACTAATCAAACCCCAGACGAGGGAAATTAATGTAACACAAATCAGTAATGCAATTGATGATAGTACACGTACCGTTGAAATTGCATTCATGAGTGAAG
AACCAGTAGTACGCGATATTGACGGTACTCTATATAACGAAATTATTCTAACTACTCCCGACAATGTAGATCTATCGCGACTAAATAACGGCGGTGCACTTCTATATAACCATAATATGGATTGGT
TAATCGGTGTAGTAGAAAACGCCCGTATCGACGAAGACCGCGTAGGACGTGCATTAGTACGATTCAGTACCGGAACATTTGCCGACCAGAAATATAAACAAGTACAGGAAGCCGTACTAACT
AAGATTTCATTTGGATATAACATCCCTAGATTACTATTTCGAAGGTAATAACTTAATCGTTACGCGAGTACAGCCATATGAAATTAGTTTCGTGTCTGTACCTGCCGACGATTCTGTAGGCGTAGG
TCGATCACTAAATACAGAAGAAAACAACGCCTATAAGGATTATTTAAAAATGGATGAAAAGGATATTCAAGAAGTACAGGAACCAGTAGAGCAAGAAGCCGTACTAGAACCAGTAGAAGAA
ACCGTAGAAGCAGAAACAGAAACTAAAGCCGAAGAATCTGTAGAAGAAATTGTAGTACCAGAAGTAGAAGTAGAACTAGAAGTAGAACGCCAGCTACAGGAAGCAAAGGACGCTTTAAA
AGTAGCTCAACGTACTATTGAAGTTGAAAGCGAGCTAGAAGAAGTACGCAAACAACTACAGGCAATTGAAGACGCAAAAGCCGAACAACTAAATAAAGAAGAATTCGCGAAATTGAATC
TATTGCAAAAGTATTTGAAGTAGATCCGAGCGAAGCAATAAATACAAATGTGAGTGTAGAAGATTTCAAACGTGGAATTGAAACACAAAAAGAAAAATCAAATAAAAATCTTAACGTTAAGG
ATGATAATAAGATGAAACAAAAACGTACTATCGACGCACTAGTAGAATTTGCAAAAGGCGATCACAATGCACTAAATGATCTAGAAATGGGATCACGTGGTTATGCAATCGCAGAAGGTGAA
TTCGCACTAGCACGTGCAAACACTACTACCGTCACAGCAGCAGGTGCAATTCAAACTGAATATTCAGATGATTTTATTCGCCCACTACTAGCAGAATCTATTTTAGGCCGTCTAGGTCTAGAAG
TGCTAACTGGCATGAATAACCGTGAATTTACCATTCCTCGCCTAACTTCCCTAGATGATAAAGCTAGCTTTAAATTCTATAACGAAGGTGAAGCAATTGCAGAAACCGTAGCTAATTTTGATAAC
GTAGTTCTAAAGCCACGTCTATTTGCTGGTGCAATTCCCGTAACTAAATCTCTAATGCTATCTAGCCCAGATATTGGTACATGGGTACAACGTGCACTACTTGAAAACGTTGCTAACTCCCTAGA
AAAAGAAGTAATTGCAGAAGTAGAAGCAACTGCTACTAAAATGGAAACTGCCGCTTCCGGCACTCTATCCGATGCAGATATTGAAGCAGCACTACAAAAACTTCTAGAAGCAAATATCCGCA
GCCGTGAATGTGTTGCAATTGTTTCCCCACAAATGTATGCACGTCTACGTCAAACTGCATTCCTATCTAACGTAGCTGGTGTTGCACTTGCTCAAGGGATGCGTTTTTCAGATAGCCAATGGTT
GTGTGAAGAAATGCCGCTAATCGTTAGCACTTTCGTAGCAGATGATACTATTCTAATGGGTAATTTCTCATTCGTTACTATTGCTAACTGGATTGGAAGTACTCTAGATGTGGATACCACTACTTA
CCGTAGTTCACTAACTACAGTATTCCGTAACTATCATTATCTTGATATTGTACAGAAACATCCAGAATCTGTAATTCAACTAAAAGTTAAAGCGTAA
>DLDGEG_16055 Phage portal protein
ATGTGGTTTAAGAATAAAAAAGCCGAACCAAAAACCAAAAAGGAAGTACCGACAATTAAAGGTGATATTCGTACAAAAGAACGTCTAATCAAAAATCCTACTTTCAAACGATCATTGGGATC
GGCAATGACAACAAAACAGGCAATCGACACGGCACTAGGACTAGGGGCAAGTTTCGTAACTGCCGATGTAAACGGACTAATTAACCGTAGTCTAGCGTCAATGGTAATAGCTAGCCGTGAC
TTAAGCATTAATAACCCAATTGCTAAAAAGTACTTTACCGAAACCGCTAGCGGCGTAGTAGGTAGTCAGGGTATCTATGTTCGTCCCGATGTACATCTATACGAAGACGAACAAGAAAACATCC
GAATCAGTCAGGAACTAGAATCACGTTTCTATGACTGGTGCGACAATCCCGACCGTTTTGATATTAATGGTGTACTAGATTTAGTACATTCCAAAACCTTGTAGAAAAAGAACGAGCTATCG

GCGGTGAAATTTTCATTCGTATTCATAATATCAATGGCAATATTAAAATTGAATTAATCAATGGTATCCGTTGCCCGACCAATAACAATATGTTATTTGCCGACGGTCACTATATTTCAAACGGCAT
TGAATATAAAGATGGTAAACCTATTGCGTACTATTTCATGCGTTATAATCCAATTACCTATAGCTATGATTATGCAAACCCCGAACGCGTAGACGCAAGCGAAATACTTCATTATTTTCAACAAGA
ATTTCAATGTAACCAAGAACGCGGAATACCCGATCTAGTATGTGCCGAACCATTACTAAGTGAATTAGATAGTTTCCTAGAAGCAAGTCTAGTTACTAAGAAAGTAGGATCGGCGGTAATGGG
CTTTATCGAAAATGATGCATCCGAAAGTGATGATATTTCCCTAGACGATGGAACACCAAACTATTTCGATAACGAAGCCTTAGAACCGGGGGCACTTGTAGAACTACAGCCAGGACAACGCC
TAAAAGATTTCAGCCCAAAGGCCGCTACTGACGGTATTAGTGAGTATGTAGATCAACAAATGACACTAATTTCTATGGGACTAGGAATTACCCGCCAAACATTAACGGGCGATACTTCATCGG
CTAGTTATTCGGCAAGCCGTCTAAGTGACAAGATTCAACAAAATACATATGCATCCCGTACAAATCTATTAAAAGTAAAAGTACTTAAACCGTTATATCGTTTATGGTTGCGTCAAGAATTACTA
AATAATAGTAATGAGATGGGATTAAAGTTTAGTGATTTTAATGCACTTCTAGAAGCACAATATCATACACAACGTCCCGTTAGTTTAGATCCGCTAAAAGATGCACAGTATGCCGTAATGATGCT
AGATGCAGGATTAGCAAGTAAAGCCGAAATTATTGCAGAGTCTGGTAGAGATCCCGCCGTAGTATTAGCCCAAATTGCAAAAGAAAAAGAGGACGCACAATTACAGGAAGTAAAACAGGA
TGAGCAAGGAAGCGAAACCGAAACTAATCAAACCCCAGACGAGGGAAATTAA
>DLDGEG_16060 DUF4369 domain-containing protein
ATGGCAAGAAGCAGAAGTACGAAAGCACAGAACGCCAGACCAGTAGATTTTAAATCTGCAATTTATATCGGTGAGGACGTAATGCCAGCATTCCCCGAAGGATCTAGTATTGAAATTATTCT
ACCCAATGGAAATAAAGAATCATATGCATATCCATATGATCCAGTTAGTACCGCTACATGGGTAGAAGGTATGGCAACCGTAATTATCAAAGACGGTAATCAATCGGTAGAACATTTCCAGATC
GTTGACCCTACGAACATTGCAAATAACTATAACCAGTTAATGGGAATTCTCAAAGACATTGACGCCGTAATAGAATCACGTCTAACTGGTGGTGGCATTCTACAAACTACTATAAATAACAAAA
CATTGATTAGTGAATCTCTAAGCTCACTCTATGCCATTCGTGCGGCATACGTAAAAAGAATCAATGCAGAATTAGCCAAGATTAATAAGACCAATCCCAATAATCCGATTAAGTCTATTAGTCGT
TTCAATAGAGGATATAAATTTTAA
>DLDGEG_16065 Terminase
ATGGCAAATAATCCCGCCAATATGCTACATGCATCAATTACGGGACAAATGCTAAAGAAATTTAAAACAGGTAATTTCGAACAAGGAATTAAAGCAAGTCCTACACTACAGGAACTAGTAACA
AAGAAAACCGATAAGACTAGTACAAACGACCAGTATCAAACAGAATGCCGCGACGGGACGATGATCTACTATGCGTCATTGGGTGCACCTTCACAGCTACGTAGTGTAACGGCAAAGTACTT
ATTCTTAGATGAAATATCAGGAGCCACAGAAACCGACGAGGGCGACCCGATTGCATTGGTAGAACAACGTGCAACTACATTTGCCGACAGTCTAATAATGATGAGTAGTACGCCCGTCACGC
CCGACGATCCCGTAATGGTTGAATTTAATAAAAGTGATCAACGTCATTTCTATGTACCGTGTCCCCATTGTGGCGAGTACCACACCCTAGAATGGAAGAATGTAAAATTCGATTGGGTAATTGT
CGATAACGGACGACGCAAGCGAGCCGACCCCGAAACGGCAAAGTTACATTGTCCACACTGCAACCACGCAATAACAGACGTAGAACGAAACCGAGCCGTAACAAAGGGATATTGGAAAG
CAGACCGACCAGAAATTACGGACGTGGTAGGATTTCAGATTAGCCGCCTTTATTCACCACTAACTACGATTAAGAAAATAGTACAAGACTTTGCCGACGCACATTATAACTTTGACCTACGAT
CATTCTATAACCTTAGTCTAGGACTACCATATGAAGATGAACACAATAAACAAATTGATCTAACACTATTAGAAAACCAACGCGACTATTCAATCAATATTAAAAAAATACCGGACGATTGTTTA
GGGTTATTTGTGGGAATTGACCAACAATTAGATAGATTGGAATGTACTACAATCGGTATTACGGACAATGAGAAAAAGATATATGTACTAGATCATAGAACCTTTTCGGCAATCGATACGACGA
AAGTAGAATCACCTGCATATAAAGAGTTAGTACAATTCCTTAAGTACCCATTCAAGACAATGAACGGCAAACCCCTACGCCGCTTTGCTGCATTTATCGATAGTGGTAACGGTAACGCAACTTC
TACGGTATATCGCGTATGCGGTCAATATCGACAGAATGGCGAGAAGATATTAACGGCAGTTAAAGGTAGTGGACAACCACACACCGAACTATTCCGCAAATCTAAAACAGCAGGTAAAGAAT
TTATTCTACTAAACGTCAATGAAGGTAAAAACTATATAGCCAAATTGATTTCACAATCTGTTAGTGCCGAGCATGAAAACTTTGCCAATAACATTTATTTCAGTGGCGATCTACCGGACGATTAT
TTTATTCAGTTAACTAGTGAACGTAGAGTATTCAGGGGCGGTAATTACGTATGGGAAAAAAGAACCAAATCAAATAATGACCGTAACGAAATGTTAGATACATTGAACTACGCATTAATTAGC
GTCAAGTGGGTACTAAGTCGATTGGGTGCACATCCATTTAAGGAACTACGAATATATAACGCCAATCAATTACGTAAAGTACAATCTGAATTCGACAACGATATAGATTTTAGTAACATCGATAC
CGAAGATACACAACCACAACCAATAAATACTAAAGCAACTACACAACCAATAAATAAAAGAAGGCGTACTAGTTTTATTGCTGGTGGACGTAGTTGTAAATTTAAATAA
>DLDGEG_16070 Terminase small subunit
ATGAATACATTAAGTCTTAAAACAATCCAAGACCGCTACGGCTATGAACTATCGCAGATTAGCCGCTGGCAAGACGAGGGATTAGATCTTACATGGCCTGAGAATCAGATCAATAACTGGATT
GTAACAAATAAAATTAATCCCATGAGAAAAGGCGACCCCGCATTAAAGGCCGAAAAGATGCAGGAAGAAATACGCCTAACACGTGCAAGAGCCGACCAACAAGAAATAGACACAATGAAA
GCTAGTGGCGAAATGGTACTAGTAAATGACGTTGCCGGCGAACTAGCAAAATATTGCTTACAGCTTAAAACAGCAATTAGAAATATTCCCCTGAATGTATATTTGCAACTTGCCGAAATTGCC
GATGATCCGTTAGCAATGAAAAACTTATTAAGTACAAAAATCGATGAGGTACTATTAGAATTAGGAACGATGAAATATGAAGAAGGATTTACACCTACACCTACAGAACAAACTACAGAAGAT
ACTACGAGCAGGAAACGAAGCACTAAAGCCACCAAGAAAAATAAAGCCGAGTAA
>DLDGEG_16075 hypothetical protein
ATGCAAACATATATTCATTTCAGCAATGGAACTACTACAATTGACACGGACGGCAACAAATCAGGATATGAATATACCCGCCACGATCTAAATAACATATTAGATATTCATCTAGTATTGGGTAGT
CCCTATTACGTACACGTACACATAGGTAATGACTATCTAATACTACGACATAAATACATTACATTTAACTATCACAATAATTCAGTACAGATCGGTAATAAAACCTACGCAATGGAACGATACCGA
GGAACGGAACAAACACACATCCAGCAATTGCAATCACTCGCCATTGAAATGACCGACAACGGGACGATATACGGGCAACGTTTACCCTATGACATATGCATAACAATTCATTTTAATCATGAA
TGCACAATTCTGTACTGA
>DLDGEG_16080 methyltransferase
ATGGCAAGAGTAACTAAAAAAGAATCGGCAAATCATAATAAGGTAATGGATCTAATTCATTCCGACCGTGAACTAACATTCGATGATAAAGAATTCATATTCAAAAATTATAGAGGCGATGGCA
TTGGTGCAACGGGTGCATTTTTTACGCCCGATGGGTTAGCGTGTGATTTTGTATTAGATGCTGGCTGTACTGGACAATGCATAGAACTATGTGCAGGTATCGGTATTCTAAGCTACTATCAGTA
CCGCCGAAATAAACCCAAACATATTACTTGCATAGAAATAAATCCCGAATACGTTCAAATCGGTAAACGTATCCTACCACAAGCCGAATGGATCACAGGAGACGCCCTAGAATACACTCCTACG
CGTTTTTATGATGTAGCCTATGGAAACCCACCATTTGGTAATATAAAGACGTCACAGAGCGTTACAGGCCGTTATAGAGGATCTGAATTCGAATACAAGATCATCGACAGGGCAAAAGACTAT
GCATCATACGGGGCGTGGATAGTCCCACAGTCAAGTGCAGGCTTTAAGTATTCAGGTCATACCTTCTATGACCGTTCGATACAGTCTACTAAGTACGGACGATTTGAAAGGGAAACGGGGCT
TGTACTACACGGAGGTGTAGGAATAGACACGAGCATATACAAGGATCAATGGAACGGTACGACCGTTATATGTGAATCCGTGATAGTGGATTATGACGGTACTAATTTATAG
>DLDGEG_16085 MliC domain-containing protein
ATGAAAAAAATATTACTCGCACTATTATTAATATCGTCCTATAGCTATGCCGATCGCTATGATAACTGCAATGCACAGGTGTATAGAGACGGTAAACCCGTAGATAAAGAATTTCAGGTAGATGT
ACTGGACTATAAAAACCGTTTTGAATTAAGACAGGGAAGTACTTTTATCGGTGCATCGCCAGTACTTGAATCTACCAATAGATATAACGGCAATATGAAACGCATCACCAATGAGGCCGAATG
GCGTATAGATGATAACGGTACTAAAACCTATTCCATCGAATACTACGCCACTAAATCAACATTAAAAGTAACCTGCAAATAA
>DLDGEG_16090 Lipoprotein
ATGAAAAAGATCATTGCAATGGGTATTGCTGTACTAATTACTGGCTGTAGCAATAATCATTACGCATTCATGGACGATGCAGTAGTAGGAGATATAAACGGGCGTACCCTAGTTATGTTCCCGAT
TGGTAATGATCAGTACCGTCCACAGTTCGCTAGTGATTGTCCACCCGTACTATTGGGTGAAGATCGTCCTAGCACTGGTGGCTGTATCATCGACGGATACCGCCTAGCTAATGTTCACTCATTC
AATGAACACAAAGCCCACGGTAGCCACACGAACGCCACCACAGCACCACAACGCCCCAATATGATTGGGTTAGATGTGCCAGCGTCTACTATTTCAATTCCCGATAACATCTAA
>DLDGEG_16095 Tail assembly chaperone
ATGAAAACCATTACTACTAAGTTTATCGCTACTGTACTGAATGATGACTATACCCCCGCAATGTTACAGGCGGACGGCGACCGCCGTAATGAGCTGGCGAACATGGACACGAACGAACTACA
GACGCTGATAGATCGCCTTGCAGACGTCATTCCAATGGTCAACGAGATACGCGGCCAGAAGCGACAAGAAGCAATGAAACTCTTAGAAGGTGTGGTAGGGACGGGATCTTTTGCAAGCGT
ACAGGAGCTACTACAGGCCGTTACAGGCGGTACTACTAGTGCTACCGAAGAACCACGGACAAAGGCAAAGACCAAACAAAAGACTACGACCAATAACAACCGAGTCTACAGGGTGGAGA
TCTACGACAATGCAGCAGACGAACGCCGTACATATACCATCGTGAATAATGTACTACCGCCGACACTACAGAAAGATCCGGCATACATGGCATTGGTCGCAAAGAATCCAGAACTTGCCGACC
CGACAGAATTACTTATGGCATACAGTACCGAATATCGTGATACACATCCGATCAATGCTAAATATGAAAAGGTAGAATTTCATATCAATGAACGCGGTCGCCTGAATGCAAAGGCAATGAAATT
TTACGAACAATATAAAAAGAAAAACCCAAACGCAACTACGAGCGAATTTAAAACCGTAGTAATGGATAACTATAAGAAGGTGTAA
>DLDGEG_16100 ATPase
ATGTTTTCTGATCCAGCTATGGAAAAAGTACGGCAAGAAGTAAGGGAACTAAACGAAATAGTAATTACTGTTATACGTTCCCTTTCTACCGAAGATGTACTAATACTATTTGGTGATAGTATTAT
TGGAGATACTATTAATCTAGTCAAATCCATTAAAGAGGAAAATCTATATAAACTAATAGAAACTAATCAAACACTACTTTCATTAAATTATGAGGTACTGAAAAACATGTTACGGGGTAGCTAA
>DLDGEG_16105 DUF2969 domain-containing protein
ATGAGACTCAATATAATTGCTGGTGGTCTAATTACCGAAGTGTACGACGATCACGATAAAAAGGTAGGTAGCGTTTCAATGAGTAAACAGGAACGCCACCGCAATAAACCATATACCGATGGT
AAGCAATTCTACGCATCATTTAACGAGGCATTACTTGCACTACTAACACGTAAGAAGGTTAACATCCATAATGTTTTCTGA
>DLDGEG_16110 DUF3799 domain-containing protein

ATGAAAGCAATCGATGTAGTTTATTCTGTTTTAGCACAATCGAATATTTCTACCATCGTTAGGGAAGTGATCGCGTCACCGCTTCCCGCCTACCAACGCCACGCACTGGTACAGAATATTGAAC
AATGTATCAGTACATGCGAGGGGGCGTTAGACGATGGCTATCAAGTACAAGACTTGCCCCGCGTATTGGGTGGTAAAGGTACATTGTTAGATGTTTTCAAAAGCTATGCACGTACATTAGAG
GAACAATATAAAATGACTTTACAAGAATATATCCCCCTGCAAAATGCCGTAGATGCCGCGACCTATGGTTATGCCGAACGCCGTAACATTACTATCGAAGAATCGATGGTAGATGGAATGCACG
TAGTTGTATTTGGCATGAAAGGTTATATTCAGCCTTTATTTGTACTCTATGCATGTCAGGACGGATCATTTAGTTGGAAACAAAATATCAGCCTTGCACCGAGTGTTAAGGAAGAATTACCCGC
ATATTATCAAAACTGCAAAATGCTTAGGGTAATGATTGAGTACGTAGCCAAAGAACATAGCTACGAAATTAATGCTAAAAAATGA
>DLDGEG_16115 Phage protein
ATGAAGAAGTACAAAAGCTTCACTGCTAACTACGTAAAGGATTTAAATGAATCCTCAATGGGTTTTTATGCCAACGTAGAAGTACAGACCCACGACAACGGTACTACGGCACAAATTAGTACC
TATGCAATGGATAATCCAAACTACGCATTCTTTGCATTTGAATCGGGTTTACAGGTATCCGTAGACTATAACAACGGGATCATGAAAATCGGTACTACCGATAATCATGTAGAACGTGATTTACG
TACTCACCCTTGCTATGCAGGTAAACAACGTAATGCACTTATTCGCGTATACATTCGTTTCATATTGGGACGCATCGCCAGCCACGCCGAACAAGAAGCATTATCCGATTTCGTAATTAATGAAT
TGGCAGGTGATCATGATCATTACATTTAA
>DLDGEG_16120 Acetyltransferase
ATGAAACTTAAAGAAGAAGACGACAGGATCACGCACAAGATACGCAAGTACGAAAGCGGCGAAACCATCACCGTATACCCACAGGTACTAATATACTACGGACAGGACGATCTAAGGATCG
TCTACCATATGTACAGCAAAGTGTTTAGCCAGGAGTACATAGAAGAAGGCCGAAGCCGCCATTTACCTACATTCGTAGGTATGAGTGAGGCCGAATTGTTCCAACAATCGGTACTATATGACG
TAGAAAAAAATCATTACTATTCAACAAGAAATGATTTTTATAGAAAATAGTAGTTGCAAGCCAATATAG
>DLDGEG_16125 DNA-binding protein
ATGAAAAAGAATTGGCACAAAGGAAACATAACCAATAACTACCGTAAATTTGGTGCAGATAAGAATGGGATGTACTCAAAGCCAGTACAAACCCCATTGACCGATACACCCCCCGTAGGTGA
GGAACTACAGGAATGCATATGCCAGTACATAAGCACCTACCGCAAAGAACACGCCCAGACACCCGCCTATACGCATCTAAACCGCCGCTATGGGCTTAGAGCAACAAAGGCATACTACAGAG
GACTAGACAATGAAACTTAA
>DLDGEG_16130 Longin domain-containing protein
ATGAATAACCAACAACTTTACATTATGGCAGTAGAAGGAAGTAACACACTTTTAAAAGTAGGACTACATAATGATCCAGTAGAACAACTTTTCGAGTACCAAAGCAATGCAACCAGTACAATA
ACATTGATGGTAGTAAGTGACAGTATGACTTTTGATAAAGCATATGGTTTATATGAATCCATAATGAATACTTTACCAACTATACCGCCAGAATTGGGAACTAAATTTCATAATCAACAAAACCA
ATCACTTTTAGAACTAACACAATTAGAGAACATCATGACAATGATCGGTAATGTATATGAAATAATGAAACGCACAATAGACTATTCACCAATCCAACAAACTTACTACAATAACAAGATTATAC
AAAAACAATGCAATACGCCAGTAAGTGAAATAGCAAATACTATGCCCGAATTTAAATCTACGGGAAATAAATCTGTCGATGATGTGATGATGAAAATTCGTGGTGGTCGTACTGAATCATTTAA
ACTATAG
>DLDGEG_16135 hypothetical protein
ATGGAAAAACTATTGGAAAAATCAACGGTAGAATGCGGCTTACAGGGGTGTTTTTGGGGTTTTTCCAACTATATTAATATTATTTTGAGTTTTAATGGAATAACAAAGTTTTTTCTTTAG
>DLDGEG_16140 Phage protein
GTGAGTAGACCCCGTAAGATTTACGACAATAGCGAGTTAGTACAGATCATGAAAGGTTATTCGTACTTGAACCAATTGACCAACGAAGGACAGAAGATCATTAGTGATGCAATAGATTCGGT
ACTTTCAAGTAGTAGAAACAAAGTAAGTAAGAAAGTAATATTCAAGATGGTATGTAAAATAGAAAGTCTCTCTACCAGTGAAGTAGAATCATTCTTAAACTTTGAAAAACAATTTAAGGGTGA
AAAGAAGTTGGCTAAATCATCCATCTACAATTACCGCAATATAGCCCATCGTGCAGCCGTCGAACTACTGGAAGCATACAACCACGGAGTAATGATTAAGTACACTCTCAATGGTGATGCACG
TAACCTAACGAGCGACGAAACGAACAAACTAAAACAGATGCTACATGACGGTACGAGCCTAATGCGTATCAAGGCATATATTAATAGCTTATGA
>DLDGEG_16145 CopG family transcriptional regulator
ATGGGAAATAAATATAATAAATCAGACGTAGTTAAACACGCCTCGCAAGGGGCGGTTTTTTTGCACGTAAAAAACATAAAAATGAATGTCAAAGGAGTATTCATGAAAGACACTAAAGCAAA
ACAAATAACAGTACGCATTACCAAAACACAAGAAGATACTTTACAAAGAATGGTAGATAGTGGTGAACATAAATCTATCGCCGCCGCCGTACAGTACCTTATCAATAAAGAAGCAGCCTTAAA
AAGCAATTAA
>DLDGEG_16150 hypothetical protein
ATGGAATGGCTAATTACAATACTTGTACTTTTAATCGGATTTACCATTATGGTAACTGGATTCATTAAGTACTTGTTTATTTTATTCATGATTTGGCTTATTTGGGACGGTTTTTTACGGATTTTGA
TAAATAATCGTAGAAAGTTGGGACAAAATGCGATTCATGAGCTATTATAG
>DLDGEG_16155 Recombinase family protein
ATGATAAAAACCTTCATATATAGCCGTGTTTCTAGCCAAGAACAGTTAAGTGGGGGCGGGTTAGACCGTCAAGAAACGGACGTTCGCGAGTACGTCACCCGTAACGGATTAGACCAGTACG
AAATAGTGGCAATGGTAGATCGCGGCATATCTGGCTATGACGGTAGTAATATGCGTGATGGTGAATTAGGTAAATGGTACAAAGATGCGATTACGGGTATGTATTGGGGTAGTCATTTAGTATT
AGAAGCGATTGACCGTTTTAGCCGCCGTGATCCTATGCTGGCAATGGAAGATTTTACCGTATTAGTTAATAAGGGCGGTATTAAAGTACATATAGTAAAATTTAATACATTCATCAATGGCGATA
ACTTGCCTATGTTATCTATGATGATGAATCTTGCACATAGTGAATCTAAACAGAAAAGTGATCGTATTAGTAAAGGTTGGCAACGTCGCCGCCGGATGGCATTAGATAACGGTACTGCTATTAC
CGCACGTACACCATATTGGATCGATGTAAGGGATAATGAGTACCGTCTTAATAATAGTTCTATTGCAGTAGTAGAAGCGTTTGCAATGTACAAACAAGGTTTATCATGTGGATTAATTGCAAAG
AAACTAAATGAATTGAAACATTACTTTCCGTATATGTGGACTAAAACCGCAGTACAACATATTGTCGATGGACTTAATACATCGGGTATCATCGATGCACCACGTGCCATTAAAACTATTAAGGC
ATTTTATAAATCTGGTTTTAGTGTCGAAGATATTGCAAAGAAAATGAATGCAGAATTCCAATTCATGAAGGAATGGAACGCACCCGCAGTACACAAACTACTACAAAATAAATCGGTAACTGG
ATTAGTAGAGATTCAGAAACGCAAACTTATTCAGGAGTTTGATAGCGACGGGAAGCCACTAGAATTACGTACTAAAAGCGAAGGTGAAAAGTACTTTGAATCTATATATCCACAGATCATTAG
TATCGATGATTTCGAACTAATTCAAAAATTATTGGAATCAAATCGTAAAGTAGATACCAAGACCGGAAAGGGACAAACAACGGGCGGTACTGTTACGCAAAATGAAGACGGTGAATTAGTTA
AGCAATTCATTCTTTCGTCATTATGCCGTTGCTATAAATGTGGTGGTGCAATGTTCAATAACATTGTGCGGTCGAAACGTAAGAACGGCAAAGTAGATACCTATTCGTATTTGCGTTGCCTCAAT
GAACGCGATGCACACTGCGACGTTAAAGCATTAAACTACCCCGTAATCGAAAGGGCGATTATAGAACACGTCAAGGGATTGAATTTTGATACCATGTTCAAGGGTACTAATAACAGTGAACT
AGAATTACTCAAAACACGAGAAGTAGAACTACTACGCGATATAGCCACGTATGAAGACGCTATAGCCGCTAGGAAGGCCGCAGGAAAAAAATACTAGTCCTGTACTGGTTACATCATTAGTCG
ATGCACAAGACGATTTAGAGGCGTTACAGAAGAAGATTAGAGCCATATCGGGCGTTACCGTAAACGCCGAGGAACTAACGAGCCTGAATGAATCCGTATTCGATGTAGCAGAGTATGAATTA
CGTAACATGGTAGAACTAGAACTACAGAAGATCATTAAACGCATTACCTTTAATTGTACTGAAAAGAATATCTACTTTATTACCATTCAGTACAATACAGGTACAGTTCTACAGCATGGACTTAA
AGTAGATAAGAAGAAAGGCGTAATAGAAACTTATGAACTTCATGAAGGGAATAAGGGTTATGTAAGTAATGGAGAAGTAATTACACCTGCATTAATAGAAGCCGCAGAAAGTAAAAATATTG
GAATTTTCGAAGGTAAGGTTATGTAA
>DLDGEG_16160 Phosphotransferase
ATGCCCGTGGATAAGGCGTTGCAGTTGATTGATTTACTGGATGAGCATCAGATTCACGGCCTGATGTATGTTGATGACGCTATGCTTTACGAACACCCAACCGGCCACGTCGTGCGTACCTCC
CGGTGGGCGCAGACCTTGCCGCCGGAACAACGTCCGACCTTTACACAGGTCTCTTCGTTAGCGCAGGCGGCGCGCGACGTGAATGCCGTGTGGAAGTTTGCGCTTACCGATGAAGATATT
CCCAGACTACAGCGGTTCGGTCAGCATGTTGAACAGGCGCTTGGCCTGGAGTGCGAATGGTCATGGCACGATCAGGTGGATATCGCGCGCAAAGGCAACAGTAAAGGCAAGCGCCTTACC
CAGTGGATAGAAGCGCAGGGAGGGTCAATGAAAAATGTGATCGCTTTCGGCGATAACTACAACGACATCAGTATGCTGGAGGCGGCAGGCACCGGCGTTGCGATGGGCAACGCCGATGA
GGCGGTGAAAGCGCGCGCTGACGTTGTGATCGGCGATAACACTACCGATAGCATCGCCAAATTTATTTACACCCACCTGCTATAG
>DLDGEG_16165 Molybdenum import ATP-binding protein ModC
ATGCTGGAGCTTAATTTTTCCCAGACGCTGGGAACGCACTGTTTAACCCTCAACGAAACGCTGCCAGCCAGTGGGATCACGGCTATCTTTGGCGTCTCCGGCGCGGGAAAAACCTCGCTGA
TTAATGCCATCAGCGGTTTGACTCGCCCGCAAAAAGGACGCATCGCGCTGAATGGCCGGGTGTTGCATGACGCGGAAAACGGTATCTGTTTAACGCCGGAAAAGCGCCGCATCGGTTATGT
TTTCCAGGATGCGCGCCTGTTCCCCCATTACAAAGTACGCGGCAATTTGCGCTACGGCATGGCGAAAAGCATGACCGGTCAGTTTGATAAGCTGGTGTCGCTGCTGGGAATTGAAGCGTTG
CTTGATCGCCTGCCGGGGAGCTTGTCCGGCGGAGAAAAACAGCGCGTAGCGATAGGCCGGGCATTGCTCACCGCCCCGGAGTTGCTGCTGTTGGACGAACCGTTGGCCTCGCTGGATATC
CCGCGTAAACGCGAATTACTGCCGTACCTGCAACGGCTGGCGCGGGAAATCAATATTCCCATGCTGTATGTCAGCCATTCTCTCGATGAAATTCTCCACCTGGCGGATAAAGTCATGGTGCTG
GAGGATGGACAGGTTAAAGCGTTTGGCCCGCTGGAAGAAGTCTGGGGAAGCAGCGTTATGCACCCGTGGCTGCCGAAAGAGCAACAGAGCAGTATTCTGAAAGTCAGCGTGCTGGAGC
ATCACCCGCATTATGCCATGACCGCCCTGGCGCTTGGCGACCAGCACCTGTGGGTAAATAAACTCAATCAGCCGCTGCAATCCACACTGCGTATTCGTATACAGGCGTCGGATGTCTCGCTGG

TACTACAGCCGCCGCAGCAGACCAGTATCCGCAACGTATTGCGGGCAAAAGTGGCGAATTGTTATGACGATAACGGGCAGGTGGAAGTCCAGCTTGAGATAGGCGGCAGAACGCTGTGG
GCGCGAATAAGCCCGTGGGCCAGGGATGAGCTGAATATCAAGCCTGGCCTGTGGCTTTACGCGCAGGTGAAAAGCGTTTCTATCACCGCCTGA
>DLDGEG_16170 Molybdenum transport system permease protein ModB
ATGATACTGACCGATCCAGAATGGCAGGCTGTCCTTCTTAGCCTGAAAGTGTCTTCCCTGGCCGTGCTGTTTAGCCTGCCGTTTGGGATCTTTTTTGCCTGGTTACTGGTGCGATGCCGGTTT
CCGGGCAAAGCCTTACTTGATAGCGTCCTGCATCTGCCGCTGGTGCTGCCGCCGGTGGTGGTCGGCTATCTGCTGCTGGTGTCGATGGGGCGACGCGGATTTATTGGCCAGTGGCTGTATG
ACTGGTTCGGTCTCACGTTTGCCTTTAGCTGGCGCGGCGCCGTGCTGGCGGCGGCGGTGATGTCCTTCCCGCTGATGGTGCGGGCCATTCGGCTGGCGCTGGAGGGGGTGGATATCAAGC
TCGAACAGGCCGCCAGAACGCTGGGCGCCGGGCGCTGGCGCGTTTTTCTGACTATCACCTTACCGCTTACCCTGCCAGGCATTATTGTCGGTACGGTGCTGGCGTTTGCCCGTTCGCTTGG
CGAATTTGGCGCGACCATTACCTTTGTTTCCAATATTCCGGGCGAAACCCGCACCATACCTTCCGCCATGTATACCTTGATCCAGACGCCGGGCGGGGAGAGCGCGGCGGCGAGGCTGTGC
GTGATTTCGATTGTATTAGCGCTGATTTCGCTGTTGATTTCTGAATGGCTGGCGCGGATTAGCCGTGAACGGACGGGGCGCTAA
>DLDGEG_16175 Molybdate transporter
ATGGCGCATTCCTGGTTACGCCTGGTCGCAGGGGCAACATTATCTTTCGTCATCGCCGGTCATGCGCTGGCGGATGAAGGCAAGATCACCGTATTTGCCGCCGCGTCGCTAACGAACGCGAT
GCAAGACATTGCGGCAGAATATAAAAAAGAGAAAAATGTGGATGTGGTCTCCTCATTCGCCTCCTCTTCAACGCTGGCGCGCCAGATAGAAGCGGGCGCGCCGGCGGATCTCTTCATCTCT
GCTGATCAGAAATGGATGGATTACGCAGCCGATAAAAAAGCGGTGGATACCACGACGCGCGAAACATTGCTTGGCAATAGCCTGGTGGTGGTAGCGCCGAAAGCCAGCGAGCAGAAACC
GTTTACCATCGACAACAAAACGGACTGGATTCGTCTGCTGAACGGCGGACGTCTGGCGGTGGGCGATCCGCAACACGTACCGGCGGGAATTTATGCCAAAGAAGCGCTGCAAAAGTTGG
GCGCATGGCAAACGCTTGAACCGAAACTGGCGCCGGGCGAGGATGTGCGCGGCGCGTTGGCGCTGGTCGAACGTAACGAAGCGCCTTTGGGCATTGTATACGGTTCTGATGCGGTCGCC
AGCAAGGGGGTCAACGTGGTGGCGACGTTCCCGGAAGATTCACATAAAAAAGTGGAATACCCCATCGCTATCGTGGAGGGGCATAAAAATGCGACCGTCAGCGCGTTCTATGATTACCTGA
AAGGACCGCAGGCCTCGGCAATCTTTAAACGTTATGGATTTACGACGAAGTAA
>DLDGEG_16180 Multidrug efflux pump accessory protein AcrZ
ATGTTAGAGTTATTAAAAAGTCTGGTATTCGCCGTCATCATGGTACCGGTAGTGATGGCCATCATCCTCGGTCTGATTTACGGTCTGGGTGAGGTGTTCAACATTTTTTCCGGTATCGGTCAGA
AAGACCAGTCCAGACAGAACCGCTGA
>DLDGEG_16185 DNA-binding transcriptional regulator ModE
ATGCAGGCCGAAATCCTTCTTACCCTGAAACTTCAGCAAAAGCTTTTTGCCGATCCCCGACGTATCTCTCTGCTGAAACACATTGCGCTTTCCGGCTCGATTAGTCAGGGCGCGAAAGACGC
GGGAATCAGCTATAAGAGCGCCTGGGATGCGATAAACGACATGAACCAGCTTAGCGAACATATGCTGGTTGAACGCGCAACGGGCGGCAAAGGCGGCGGCGGCGCGGTATTAACCCGCT
ATGGTCAGCGTCTGATCCAGCTTTACGATTTGCTGGGCCAAATTCAGCAAAAAGCGTTCGATGTGTTAAGCGACGATGACGCCCTGCCGCTTGACAGTCTGCTGGCCGCTATTTCCCGCTTT
TCACTGCAAACCAGCGCCCGAAATCAGTGGTTCGGCACCATTACCGCCCGCGATCGCGATCAGGTGCAACAGCATGTCGATGTGCTGCTGGCCGACGGCAAAACGCGGCTCAAAGTCGCG
CTGACGGCGCAAAGCGGCGAGCGTCTCGGCCTGGAGGAAGGTAAAGAAGTGCTGATCCTGCTAAAAGCGCCGTGGGTTGGCATTACCCGGGATGCAGCCGTTGCGCGCGCCGCTGACA
ATCAGTTGTCGGGAACGATCAGCCATATCGAGCGCGGCGCGGAACAGTGTGAAGTGTTGATGGCGCTGCCGGACGGCCAGACGCTGTGCGCCACGATACCGACTTCAGATGCCGCCACGT
TAAAGGAAGGCGATGACGTGATTGCCTGGTTTAATGCGGACAGAGTGATTATTGCAACGTTGTGCTAA
>DLDGEG_16190 ABC transporter ATP-binding protein ModF
ATGTCATCGTTGCAAATTTCGCAAGGCACGTTTCGTCTGAGCGATACAAAAACACTTCATCTGGATTCGCTGACGCTCAATGCGGGCGATAGCTGGGCATTCGTTGGGGCTAACGGGAGCG
GGAAGTCGGCGCTCGCCCGCGCGCTGGCGGGCGAACTGCCGTTATTAACAGGCGAGCGCCAGTGTCGGTTTACCCGCATTACTCGTCTGTCCTTTGAACAATTGCAAAAGCTGGTCAGCG
ATGAGTGGCAACGTAATAACACCGATATGCTCAGTCCTGGCGAAGACGATACCGGGCGCACTACCGCGGAAATTATTCAGGATGAAGTTCACCATCCTGCCCGCTGTGCGATGCTGGCGCA
GCAGTTCGGCATTTCCGCTTTACTCGACAGGCGTTTTAAATACCTTTCTACCGGCGAGACGCGAAAAGCGCTGCTGTGTCAGGCGCTGATGTCTGAGCCGGAGTTATTAATCCTTGATGAAC
CGTTCGACGGACTTGACATGACCGCCCGTCAACAACTGGCGCAACGCCTGACGGCGTTGAACCAGGCGGGTATGACCCTGGCACTGGTACTCAACCGTTTTGATGAAATCCCGGACTTCGT
GCAGTTTGCCGGCGTGCTTGCCGACTGTACGCTGGCGGAGACCGGAACGACGGCGGAGTTGCTGCAACGGGCGTTGGTTGCTCAACTCGCGCACAGCGAGCGTCTGACCGACGTACAAT
TACCCGAACCGGACGAACCTTCCGCCCGCCATGCGTTACCCGATGGCGAACCACGGATCGTACTTAACGATGGCGTAGTCTCGTATAACGATCGCCCTATCCTCCACCACCTAAGCTGGCGAG
TCAATCCGGGCGAACACTGGCAGATCGTCGGTCCTAACGGAGCGGGTAAGTCTACGTTGCTTAGCCTTATTACTGGCGATCATCCGCAAGGCTACAGCAACGATCTTACGCTGTTCGGGCGG
CGGCGAGGAAGCGGCGAAACAATCTGGGATATCAAAAAACATATCGGCTACGTCAGCAGCAGTTTACATCTGGATTACCGCGTCAGTACCACGGTGCGCAATGTCATTCTCTCCGGCTATTT
CGATTCTATCGGCATTTACCAGGCCGTTTCAGACAGGCAACGAAAACTGGCGCAACAGTGGCTGGATATTCTGGGAATCGATAAGCGTACGGCGGATGCCCCCTTCCACAGTCTCTCCTGG
GGACAGCAGCGGCTGGCGCTGATCGTGCGCGCGCTGGTTAAGCATCCGACAGTGTTGATTCTGGATGAGCCGCTGCAAGGGTTGGACCCGCTGAACCGCCAACTGGTACGCCGTTTTGTA
GATGTATTAATTCGCCAGGGAGAGACCCAACTGCTGTTCGTTTCCCATCATGCAGAGGATGCGCCTGCCTGTATTACCCACCGGCTGGAATTTGTACCAGAGGGCGAACGTTATAAGTACGTT
TCCGGACGATGCAATAATTAA
>DLDGEG_16195 hypothetical protein
ATGGCTTTTCGTTTTCCACAAATAATACTATTTTTACTGGCAGCCATGTTATTTTGCCCCGGCAGTTATGCTGAGCAAAAGCCGACCGCTGCGCAGGAAGCGCGTAAAACCGCCGTTGAGGTC
GCTGTTGAAGGCATGAGTCGCGCCGCCGTTGCGGGTCCGACAAAGATTAGTTTAGGTGATAAAGCCACGCTTAATCTGCCGGAAGGATTTACCTGGATACCCGCAAAAGAAGCGGCGGTA
TTTATGCGTGAAATTGGTAACTATGTTGACGATGAATATTTTTACGGCCTCGTATTTAAAAAGGAAATGAATGGATTTATTTCTATCGAATATGATGATTCTGGTTATGTAAAAGATGATGATGCA
AAAAACTGGGATGCCGATGAATTAATGGATAACCTGCGCAAAGGCACAAAAGAAGCGAATAAAGATCGTATTGCAAAAGGCATTGAGCCGATAGAAATTATTGGCTGGATCGAGAAGCCTA
CCTACGATGCAACCAATCATCGTCTGATCTGGTCCGCCGCCATCCAGGATATCGGCACCAACGAACCACTGAATGAACAAGGCGTGAACTACAACACCTATCTGCTGGGACGTGAAGGCTAT
TTTTCTTTAAATCTGGTGACCGACAGAGGTTCTGTTGACCATGAAATCCCTCTGGCGAAAAGAATTCTCAGTTCCGTCAAGTTCAACGCTGGACAACGCTACGCTGACTTCAACGAATCAAC
GGACAAAATAGCGGAGTATGGGCTCGCCGCGCTGATTGGCGGTATCGCGGCCAAGAAAGTAGGTCTGCTTGCTATGCTCGGTATCGCGTTGCTTAAATTCTGGAAAGTGACCGCCATCGGC
GTCGTCGCTGTCGGGGCGCTCGCCAGGAAGCTACTCTCGCGTAAAAAAGACTGA
>DLDGEG_16200 UDP-glucose 4-epimerase
ATGAGAGTATTGGTTACAGGTGGTAGCGGTTACATTGGAAGTCATACCTGCGTACAATTGCTGCAAAATGGTCATGACGTCGTCATCCTCGATAACCTCTGCAACAGCAAGCGCAGCGTGCT
GCCCGTGATTGAACGTCTGGGCGGTAAGCACCCGACCTTTGTCGAAGGCGATATTCGCAACGAAGCGCTTATAACCGAAATTCTGCACGATCACGCGATTGATACCGTGATTCACTTTGCCG
GCCTGAAAGCCGTCGGCGAATCGGTCGCCAGGCCGCTGGAGTACTATGACAACAACGTCAACGGTACGCTGCGGTTGGTCAGCGCCATGCGTGCGGCCAACGTTAAAAACCTTATTTTTAG
CTCCTCCGCCACCGTTTATGGCGATCAGCCCAAAATTCCTTACGTCGAAAGTTTTCCTACCGGCACGCCGCAAAGCCCCTACGGCAAAAGTAAATTGATGGTAGAACAGATCCTCACCGATCT
GCAAAAAGCCCAGCCGGAGTGGAGTATTGCGCTGCTGCGTTATTTCAACCCGGTCGGCGCGCACCCGTCGGGCGACATGGGAGAAGATCCGCAAGGTATTCCGAATAACCTGATGCCCTAT
ATCGCCCAGGTCGCCGTGGGTCGTCGCGAATCGCTCGCCGTTTTCGGCAACGATTACCCGACCGAGGATGGCACCGGCGTACGCGATTACATTCACGTTATGGACTTAGCCGACGGGCACG
TCGTGGCGATGGAAAAACTGGCGGACAAATCCGGCGTACATATTTATAACCTCGGCGCGGGTGTCGGCAGTAGCGTCCTGGATGTGGTCAACGCCTTTAGTAAAGCCTGCGGTAAACCCAT
TAATTACCACTTCGCGCCGCGCCGCGACGGCGATCTCCCGGCGTATTGGGCGGATGCCAGCAAAGCCGATCGCGAGCTGAACTGGCGCGTCACCCGCACGCTTGACGAAATGGCGCAGGA
CACCTGGCACTGGCAGTCACGCCATCCGCAGGGATACCCAGATTAA
>DLDGEG_16205 Galactose-1-phosphate uridylyltransferase
ATGACCCCATTTAATCCCATCGATCATCCGCATCGCCGTTATAACCCGCTTACCGGCCAGTGGGTTCTGGTTTCGCCGCATCGCGCAAAACGCCCCTGGCAGGGGGCGCAGGAAACGCCGTC
TCAGCAAATGCTGCCGGCCCACGATCCGGACTGCTTCTTGTGTGCGGGCAATACGCGTGTCACCGGCGATAAAAATCCCGATTACAAAGGGACTTACGTCTTTACTAACGATTTCGCGGCGC
TGATGGCCGATACGCCGGATGCGCCGGACAGCCACGATCCGCTGATGCGCTGTCAGAGCGCGCGCGGCACCAGCCGCGTGATCTGCTTTTCTCCCGATCACAGTAAAACGCTGCCGGAGT
TGAGCCTGCCCGCGCTGACGGAAATCGTCAGAACCTGGCAGACGCAGACCGCCGAGTTAGGCAAAACCTATCCGTGGGTGCAGGTCTTTGAAAATAAAGGCGCGGCGATGGGCTGTTCG
AACCCGCATCCGCATGGACAGGTCTGGGCCAACAGCTTCCTGCCGAATGAGGCCGAACGCGAAGATCGTTTACAAAAAGCCTACTTCGCCGAGCAGCGCTCGCCAATGCTGGTGGATTATG
TCCAGCGAGAACTGGCGGACGGCAGCCGCACCGTGGTCGAAACCGAACACTGGCTGGCGGTGGTGCCTTACTGGGCGGCGTGGCCGTTCGAAACGCTCCTGTTACCCAAAACGCATGTC
CTGCGCATTACCGATTTAAACGACGAGCAGCGCGACTCTCTGGCGCTGGCGTTGAAAAAGCTGACCAGTCGTTATGACAACCTGTTCCAGTGTTCCTTCCCCTACTCAATGGGCTGGCACGG

CGCGCCGTTTAACGGTGAAGAAAACGCACACTGGCAGTTGCACGCGCACTTTTATCCGCCGCTGCTGCGCTCTGCGACCGTCCGTAAATTTATGGTCGGCTATGAAATGCTGGCGGAAACC
CAGCGTGATTTAACCGCAGAACAAGCGGCTGAACGCCTGCGCGCGGTCAGCGACATCCATTTTCGCGAATCCGGAGTATAA
>DLDGEG_16210 Galactokinase
ATGAATCTGAAAGAGAAAACGCGCGCGCTGTTTGCTGAAATTTTCGGCTACCCTGCCACCCACACGATTCAGGCGCCAGGCCGCGTCAATCTGATCGGCGAGCACACTGATTACAATGATG
GTTTTGTTCTGCCCTGCGCTATTGATTACCAGACCGTAATTAGCTGTGCGCCGCGCGACGATCGTACCGTACGGGTGATTGCCGCCGATTACGACAATCAGGCGGACGAATTTTCACTGGATG
CGCCGATCGTGACCCACGATAGCCAGCAGTGGTCAAACTATGTGCGCGGCGTAGTGAAACACCTGCAGCAGCGTAACAACGCGTTTGGCGGCGTGGATATGGTCATCAGCGGCAATGTGC
CGCAGGGCGCCGGGTTAAGCTCCTCCGCCTCGCTGGAAGTGGCGGTGGGCACCGTCTTCCAGCAGCTTTATCACCTGCCGCTGGACGGCGCGCAAATTGCGCTCAACGGACAAGAGGCC
GAGAACCAGTTTGTTGGCTGTAACTGCGGCATTATGGATCAGCTCATCTCTGCGCTCGGCAAAAAAGATCATGCGTTGCTGATTGATTGCCGTACGCTCGGCGCCAAAGCGGTTTCCATGCC
GAAAGGTGTCGCCGTGGTGATCATCAATAGTAACTTTAAGCGCACGCTGGTGGGCAGCGAGTATAATACCCGCCGTGAACAGTGCGAAACCGGCGCCCGTTTCTTCCAGCAGCCGGCCCTG
CGCGATGTCAGCCTTGAGGCGTTCAATGCCGTTGCCAGCGAACTGGACCCGGTAGTCGCAAAACGCGTTCGCCATGTATTGAGCGAAAATGCGCGCACCGTTGAAGCGGCAAGCGCGCTG
GAGAAAGGTGATTTGCAACGTATGGGCCAACTGATGGCGGAGTCCCATGCCTCAATGCGCGATGATTTCGAAATTACCGTCCCGCAGATAGACACGCTGGTAGACATTGTCAAAGCGACCA
TCGGCGATCGAGGCGGCGTGCGCATGACCGGCGGCGGCTTTGGCGGGTGTGTTGTCGCACTGATCCCGGAAGATCTGGTTCCCGCTGTTCGGCAGGCCGTTGCGCAACAGTACGAAGCG
AAAACCGGAATCAAAGAAACCTTTTATGTATGCAAACCGTCACAAGGAGCAGGACAGTGCTAA
>DLDGEG_16215 hypothetical protein
GTGCTAAATGAAACGCCCGCACTGGCCCCCGATGGTCAACCGTACCGCCTGCTAACCCTGCGCAACAGCGCAGGGATGGTGGTAACGCTGATGGACTGGGGAGCCACTCTTCTTTCCGCCC
GTATTCCGCTGTCTGACGGCAGCGTTCGGGAAGCATTGCTGGGCTGCGCCAGCCCGGAACACTACCCTGAACAGACGTCATTCCTCGGCGCCTCTATTGGCCGTTACGCGAACCGTATCGC
CAATAGCCGCTATACGTTTGCCGGCGAAACCGTACAGTTAAGCCCAAGCCAGGGCGAGAACCAGCTACACGGCGGACCGGAAGGCTTTGACAAGCGCCGCTGGCAAATTGTGAACCAAA
ACGATCGCCAGGTATTATTCGCGCTCACCTTCTGA
>DLDGEG_16220 hypothetical protein
GTGAATTTGACCAACCACGTCTATTTTAATCTGGACGGCGATCGGACCGACGTGCGCCAACATAAGCTGCAAATTCTGGCGGATGAGTATCTGCCGGTAGATGAATCCGGTATTCCACGCCA
GGGGCTAAAATCGGTAGCCAATACCTCGTTTGACTTCCGGATGCCAAAAGTCATTGCCAGCGAGTTTCTCGCCGATGACGATCAGCGCAAGGTTAAAGGCTACGATCATGCTTTCCTGCTAC
AGACGCAGGGTGATGGCAAAAAGCCGGCGGCCCGTCTGTGGTCACAGGATGGAAAGCTACAAATGATGGTTTACACCACCGCCCCGGCGCTACAGTTTTATTCCGGCAATTATCTGGCGG
GTACACCTTCCCGCGGACCGGAACCTTATGCCGACTGGCAAGGGCTGGCGCTGGAAAGCGAACTGCTGCCGGACAGCCCGAATCATCCTGAATGGCCGCAGCCTGACTGTATCCTTCGCC
CTGGCGAGGAGTACGCCAGCCTGACCGAGTATCAGTTTATTCCGTTCTAA
>DLDGEG_16225 2,3-bisphosphoglycerate-dependent phosphoglycerate mutase
ATGGCTGTAACTAAGCTGGTTCTGGTACGTCACGGTGAAAGTCAATGGAACAAAGAGAACCGTTTCACCGGCTGGTATGATGTTGACCTGTCTGAAAAAGGCGTAAGCGAAGCAAAAGCA
GCGGGTAAACTGCTAAAAGAAGAAGGTTTCAGCTTTGATTTTGCCTACACTTCCGTACTGAAACGCGCTATCCATACGCTGTGGAACGTACTGGATGAACTGGATCAGGCGTGGCTGCCGG
TGGAAAAATCCTGGAAACTCAATGAACGTCACTATGGCGCGTTGCAGGGGCTGAATAAAGCGGAAACGGCAGAAAAATATGGTGACGAGCAGGTTAAACAGTGGCGCCGCGGTTTTGCC
GTGACGCCGCCGGAACTGACCAAAGATGATGAGCGTTATCCGGGTCACGATCCACGTTATGCCAAACTGAGCGAGAAAGAGCTGCCGTTGACCGAAAGTCTGGCGCTGACCATCGACCGC
GTTATTCCTTACTGGAACGACACCATCCTGCCGCGTATGAAGAGCGGTGAGCGCGTGATCATCGCCGCTCACGGTAACTCATTGCGTGCGCTGGTGAAATACCTGGATAACATGAGTGAAGA
TGAGATCCTTGAGTTAAACATCCCGACCGGCGTGCCGCTGGTGTATGAGTTCGACGAAAACTTCAAGCCGCTCAAACATTACTATCTGGGCAACGCTGACGAAATCGCCGCGAAAGCGGCT
GCCGTGGCGAATCAGGGTAAAGCGAAGTAA
>DLDGEG_16230 ABC transporter ATP-binding protein
ATGCGCGGGTTAATGCTCCGCTCATTTAGCGCCGGCTATTCCACTCAGCCGGTTATTGCAGATCTCAACGTTCCCTTACTGCCGCGCGGGAAAATTACCATTTTGCTGGGGCCGAACGGCTGT
GGTAAATCGACGTTACTACGTTCGCTGGCCGGGCTGAATAACGCCGACGGCGAGGCGCTGCTTGATGGCGAGGATCTCATGTCGTTATCGTTTGCCGAGCGGGCGCAGAAAGTTGTGTTTT
TGCCGCAGTCATTACCGCAGGGCGTACATCTGCATGTACTGGAGTCGATTATCGTGGTACTGCGGGCATCAGGCGGCAGAGACAATGCGCAGGGCAGAGCGCAGATACTGGCTATCCTTGA
GCAATTGGGCATAACGCATCTGGCGTTGCAGTATCTCGACCAGCTTTCCGGCGGTCAGCGGCAGTTAGTGGGTCTGGCGCAATCGCTGATTCGTCGCCCTGAACTTCTGTTGCTGGATGAG
CCGCTCAGCGCGCTGGATTTAAACTATCAGTTTCACGTAATGGATCTCGTGCGTCGGGATACCCAGGCGCAAAATAGGGTGACGATAGTCGTGGCGCATGACATTAATATTGCACTGCGTCAT
GGTGACCATGTCCTGATGCTAAAAGACGGCAGGCTGGTCGCCAGCGGCGCGCCGGAGACGGTGATTACTGCGGAGCGTCTGGCTGAAGTGTACCGGGTACGAGGACGCGTGGAGCGCT
GTTCGCAGGGGAAATTGCAGGTGGTGCTGGACGGCGTGATCGCTGTTTAA
>DLDGEG_16235 Iron ABC transporter permease
ATGACCTGTCAGGACAAATCACTTTTAGCCCCCGCTCTCGCTTCCACGCCCGTCATTCCGCGCTATCGCCAGATCATACGCAAACGGGCCGTGCTCATGTTAGCTATTGCGCTGGCGATGATG
GCTTCACTGATGGTGGATGTAACCTGTGGGTCTTCTGGTTTACCGCTCAGTGCGCTGTGGCAGGCGCTTTTCCAGCCGGAAAAGGTGAATGCGGGCATACACGTTATTGTCTGGGATATCCG
CTTACCCTATGCCCTGATGGCCCTACTGGTGGGCATGGCGTTAGGGTTAGCGGGCGCCGAAATGCAAACCATTTTAAATAATCCGCTGGCGACGCCGTTTACCCTCGGCGTATCGTCCGCGG
CGGCTTTTGGCGCTGCGCTGGCGATTGTGTTGGGGATCGGTATTCCCGGTATTCCTGCCGCCTGGTTTATCCCGGCCAATGCGTTCATCTTTGCGCTTTTATCCGCGTTACTCCTGGACGGTAT
TACCCGTTGGACAGGCGTCGCCGCATCCGGAGTGGTGTTGTTCGGTATTGCGCTGGTATTCACCTTTAACGCGTTGGTCGCCATTATGCAGTTTGTGGCTGATGAAGATACGCTGCAGGGGC
TGGTATTCTGGACGATGGGGAGCCTGGTCCGCGCGTCGTGGGAAAAATTGGGCGTACTGGCCGTGGTGTTTATTGCTGTGCTTTTTTGCGCGTTACGAAGCGCCTGGCAACTCACGGCATT
ACGTCTGGGCGAAGAGAGGGCGATGAGCTTTGGGATTCATGTCCGCCGTTTACGCCTGCTGTCATTGTTGCGTATCAGCTTGTTATCAGCGCTGGCGGTAGCTTTCGTCGGGCCGATTGGTT
TTATTGGTCTCGTTGCGCCGCACATTGCGCGAATACTTTTGGGGGAAGATCACCGTTTCTACCTTCCGGGCAGCGTGCTGATAGGCGGGCTGGTGTTATCGCTGGCCTCTATTGCGGCGAAA
AATAGTATTCCGGGCGTCATGGTGCCGGTGGGGATTGTCACCTCGCTGGTCGGGGTTCCGTTCTTTTTAAGCATTGTATTGCGACATCGGGGGAGTCTCTGA
>DLDGEG_16240 2-(5''-triphosphoribosyl)-3'-dephosphocoenzyme-A synthase
ATGAATGAAACCATAGCATTTCGTCCGGAAGGCCGGGCGTCGACAAAAGATGTGGTTGTCAATTATACGCTGGCGTCACTGGACGAGCTTGAACAACTGCACCCGGCGCTGAATCTGCTGG
CGCACCCGTCAATCGTCCGTGGCAGCCATGATTTCTTTGTCCGTTGTTATGACGCGGGCGTACAGCATGCGCAGGCCGGGGCGGCTGAAACGGTAGCGGTTTTGCCTGATTCGCTGCCACA
AGAACGGCAAAGGAGCGGGCTGCTGTGTGCCGCAGCAGGTCGTCTGGATGCCCTGCGCCAGCCGCTGACACACAATCGTTTATGTGACCTGGCCAGTCAGTTCTGTGCTGGCATGGCAGA
TGTTGACAGCGAAACCCGCAGCGGTTTTTATACCGTGCGCAGCATATCGCTGCCGGTTTACCGGCGGCTGTTGCGGGATCAACATTCTCACGGTGTCTGCCTGCAGCAGGCTCTGCTGCACC
TGCTGGCCTGGAAGAGCGATTCTCCCTGGGCGCGGCAGCAGGCGCAGCGGCTGCTCTGGCAGGGCGGCGTGCTGGGCGATAAAGGTGAGTTTGCGCTTATGACGCTGGATGATGAACTG
CGGGAGCGCCAGATTGAGTGGCCCGGGCTGTGGTCGCTGCTGGCCGTCACCGGGTTTCTGGCGAAATTCCCTGCCGGGCCGATATTTGCTGACTGA
>DLDGEG_16245 Oxaloacetate decarboxylase beta chain
ATGGAAAGTCTGAACGCCCTGCTTCAGGGCATGGGGCTGATGCACCTGGGTACCGGCCAGGCCATCATGCTGCTGGTGAGCCTGCTGCTGCTGTGGCTGGCGATAGCGAAGAAGTTCGAG
CCGTTGTTGCTGCTGCCGATTGGCTTCGGCGGTCTGCTGTCGAACATCCCGGAAGCGGGCATGGCGCTGACCGCGCTGGAGAGCCTGCTGGCGCATCACGACGCCGGGCAGCTGGCGGT
GATTGCCGCGAAGCTTAACTGCGCGCCGGACGTGCACGCGATTAAAGAGGCATTAGCGCTGGCGCTGCCGTCGGTGCAGGGACAGATGGAGAACCTGGCGGTGGACATGGGCTACACGC
CGGGGGTGCTGGCGCTGTTCTATAAAGTGGCGATTGGCTCCGGCGTCGCGCCGCTGGTCATCTTTATGGGCGTCGGCGCGATGACCGACTTCGGCCCGCTGCTGGCCAACCCGCGCACCC
TGCTGCTCGGCGCGGCGGCGCAGTTCGGCATCTTCGCCACCGTGCTGGGGGCGCTGACGCTGAACTACTTCGGCCTTATCAGCTTCACCCTGCCGCAGGCGGCGGCCATCGGCATTATCGG
CGGCGCGGACGGCCCGACGGCGATTTATCTGTCGGGCAAGCTGGCGCCGGAGCTGCTGGGGGCCATCGCGGTGGCGGCGTACTCGTATATGGCGCTGGTGCCGTTAATCCAGCCGCCGAT
TATGAAAGCGCTGACCACGGAGACGGAGCGGAAAATCCGCATGGTGCAGCTGCGCACGGTGAGTAAGCGGGAAAAAATCCTCTTCCCGGTGGTGTTGCTCATGCTGGTGGCCCTGCTGTT
GCCGGACGCCGCACCGCTGCTGGGGATGTTCTGCTTTGGCAATCTGATGCGTGAAAGCGGGGTGGTGGAGCGTCTGAGCGACACGGTACAGAACGGTCTGATTAATATCGTGACCATCTT
CCTCGGGCTGTCGGTGGGCGCGAAGCTGGTGGCGGACAAGTTCCTGCAGCCGCAGACGCTGGGCATTCTGCTGCTGGGTGTGATTGCCTTTGGTATCGGGACGGCGGCCGGGGTACTGA
TGGCGAAGCTGCTGAACCTGTGCAGTAAGAACAAAATCAACCCGCTTATCGGTTCGGCGGGGGTGTCGGCGGTGCCGATGGCGGCCAGGGTGTCGAACAAAGTGGGGCTGGAATCAGA
CCCGCAGAACTTCCTGCTGATGCACGCGATGGGCCCGAACGTGGCGGGGGTTATCGGTTCGGCCATTGCGGCGGGCGTGATGCTGAAGTACGTGCTGGCGATGTAA
>DLDGEG_16250 Oxaloacetate decarboxylase subunit alpha

ATGACCGTTGCCATTACCGATGTCGTCCTGCGCGACGCCCACCAGTCCCTGTTCGCCACCCGCCTGCGCCTTGACGATATGCTGCCGATTGCCGCCGCGCTCGATGACGTGGGCTACGGCTC
GCTGGAGTGCTGGGGCGGCGCCACTTTTGACGCCTGTATCCGTTTTCTCGGCGAAGACCCGTGGCTGCGCCTGCGCGAGCTCAAAAAAGCCATGCCCAAAACCCCGTTGCAGATGCTGCT
GCGTGGTCAGAACCTGCTCGGTTATCGCCACTACGCCGATGACGTGGTGGAACGCTTCGTTGAGCGGGCGGTGAAAAACGGCATGGACGTGTTCCGCGTCTTCGATGCCATGAACGACCC
GCGCAATATGAAAGCCGCCCTGCAGGCGGTGCGCAGCCACGGCGCGCACGCCCAGGGCACGCTCTCGTACACCACCAGCCCGGCGCACACCCTGCAGACCTGGCTGGATTTAACGGAGC
AACTGCTGGAAACCGGCGTCGATTCCATCGCCATCAAGGATATGTCCGGCATTCTCACGCCGATGGCGGCGTATGAGCTGGTCAGCGAAATCAAAAAACGTTTTGAGGTACGCCTGCATCTG
CACTGTCACGCCACCACCGGGATGGCGGAGATGGCCCTGCTGAAGGCCATTGAAGCGGGCGTCGACGGCGTGGACACGGCGATTTCCTCCATGAGCGCCACCTACGGCCACCCGGCCAC
CGAGGCGCTGGTGGCGACGTTGGCAGGTACTGAGCACGACACCGGGCTGGATATCCTGAAGCTGGAAAACATCGCCGCCTACTTCCGCGAGGTGCGCAAAAAATACCACGCCTTTGAAG
GCCAGCTGAAAGGCTATGACAGCCGTATTCTGGTGGCCCAGGTGCCGGGCGGAATGCTCACTAACCTCGAAGGCCAGCTGAAGCAGCAGAACGCGGCGGACAAGCTGGACCAGGTGCT
GGCGGAAATCCCCCGCGTGCGCGAGGACCTCGGCTTTATCCCGCTGGTGACCCCGACCTCGCAGATTGTCGGCACCCAGGCGGTGCTCAACGTCCTGACCGGGGAACGCTACAAAACCAT
CGCCAAAGAAACCGCCGGCATTCTGAAAGGCGAGTACGGCCACACCCCGGTGCCGGTGAACGCCGCGTTACAGGCCCGCGTGCTGGAAGGGGGCGCTCCGGTGACCTGCCGTCCGGCG
GATTTACTCAAACCGGAACTGGCTGAACTGGAAGCGGACGTCAGGCGCCAGGCGCAGGAGAAGGGGATTACTCTGGCGGGAAACGCCATCGACGACGTGCTCACCGTGGCGCTGTTCCC
GCAAATCGGCCTCAAATTCCTCGAAAACCGCCACAACCCGGCGGCGTTTGAGCCGGTACCGCAGGCGGAAGCCGCGCAGCCGGTGGCAAAAGCAGAAGAAGCCTGCCGCTTCCGGTATC
TACACCGTGGAAGTGGAAGGCAAAGCCTTTGTGGTGAAGGTCAGCGACGGCGGCGATATCAGCCAGCTCACTGCTGCCACCCCGTCTTCCGCACCGGTTCAGGCCACCGCCCCGGCAGG
CGCAGGCACACCGGTCACCGCGCCGCTGGCGGGCAATATCTGGAAGGTGATTGCCGCTGA

Prophage 10

>DOCPEC_14870 Zinc ribbon domain-containing protein
ATGGCACTTACCAAATGTAAAGAATGCAAAAAAGAAGTCTCAACCTCTGCAAAAACGTGTCCCCACTGTGGCGTTAAAGATCCGGGGTTTGGTGCAAAACAGAAGCTTGGCGGATGCCTTA
TCTTGATAATCATCGTTGCAGCAGTCATGTACTTTGTTGGTGGAAGCGATGACAAGCAAACGGCGGAAGCGCCAAAAGTATGCTCTAACACGGATACCCAGTGTAACTTTGACAAGAACATG
GTCGATGCAGTGACCAAATGCAAACCACTGGTAGAACATGCAGCAAAATATGAATTTGAATGGACTGACGGCCTTTTAGATCCAATGTTCTCTCATGCACGAATCGACAGCAAAAAGAATCA
GCTAACGTTCATCGGGGATAAGGTCAAATTTACAAATGGCTTTAATGCAAAAATGACCATGACTTACGCTTGCACTATGGACCTGAAAACCAAAGAAATAGTGGATTTCAAGATTTCAGAAG
GCAAATTGTAA
>DOCPEC_14875 Phage protein
ATGAAACCAGTAAAACGCCTTTACCTTTCAACGGATGAAATACACCTGGCTGACGCCAGTCTGGTGCTGGAGCTGAACAGCTGTGGACGTGGCTTTATTACGGCACAGACGACCACAGACT
ACACCGGGAAACTGGTACGGCTGGATGTAGGGTATTCCGGTTTACTTCTGCGCTGGTTTACCGGCTATGTGGAGCGCTCACAGCCTGCCGAAAACGGTTATCAGCGTCTGTTCGTCCGCGA
GCTGGCTGGCGTATTTGAGCGGATGTGGCCATGCTCATTCCAGCATCCAACACTGCGCGATGTGGCCGGATGGCTGGAGGAAAACAGCGGGATCAGCATTGCGGTACCGGATGTGCCGTA
CAGTGATAAACCGATCCCCCATTTCACCCATAACGGGACGGGATACCAGCTGCTGAATAATCTGGGCAGGGCATTCAGCATCACGGATTACATCTGGTATCCATTGCCGGATGGTTCGCTGTA
TGTCGGCGGCGCAGAAAAGGCGCTGTTTGCCGGACGTCCGGTAGAAATCCCGGCAGAGTTCAGCCAGGGAACGGCGGGTGGTAATTCCATGACATTGCCGGTGATCCAGAGTCTTCGTC
CGGGCGTGGACGTGAACGGGGAACGCGTGACCAAAGTTCATCTGACGAATGACACAATGACCATCACGTGGACACCACGGAACCGCGCCACAGGTCAGCCATTACAGAAAACACCGGCG
CAGCGTCAGATAGAAAGCCATTACCCGGAACTGGCTTCAGGTCTTCACCTGCCCAAACTGGCCAGGGTAGTGGCACCCAGCGAGGCCGTAAAAAGCGGTAATTTTGCCGACCCGTTCCGG
CCACGGTACGCTGTTGACGTGCAGCTGCTTGACGCGGACGGCAACCCGGACAACCAGACGCCGGTATATTCCGCCGTACCGCTGCCGGTGCCAATGGCCGGTAACGATTCGGGAATGTTC
CAGTTTCCACCGGAAGGAACGCTGGTAGAAGTGGCGTTTACGGGCGGCAGACCGGACAAGCCCTTTATCAGGCAGACGCTGCCGGATGGCACCAGTCTGCCGGACATTAAGCCCGGCGA
ACAGCTGCAACAGCAGCGCGCGGAAGTATCGCAACGCGTGACACAGGCAGGAGACTGGGTACGCCAGACGGATCAGACCATCAGTGAAACATCGATGGCGCGGACGGTGAAAGCCGAT
ACGGAACGGCGCGAACTGGTCAGCCGTGAAACCACGGTGAAAGCCACGGATAAAATCACAGTACTGGGTACCGCCACGCTGATGGCCGGAGCCATACAGCAGGTCAGTGCTGGCGACTT
CAGCCAGGCGGTAAAAGGAAACCGGCTGGCCAGTATTACAGGAAATGAAGAAACCGAAATCGCCGGGCAGCAATCCACGAAAGTGGCCGGTGCCATGAATGTTGATATAGGGGGAACCC
TGACAGAAAAGATTGCCGCATTACGTAAATCCGTGGCAGCGGGCGGTCAGCAGATTATGGGGCCAACCGTGCATATCGGTAGCGAGGACGTAAATACGCTAACCATGATGCTGGACACCAT
TGATTTACTGGCAGAGCTGGCGCAGCAGTGCGCGAGCCATTCACACCCCAGTGTTGGTACGCCGACCAATGCCGGAGCATTCAACCAGACGGCAGTAAAGGCCGGGCAGACCCGGAGCA
AGTACCAGAACATCATCGCCTGA
>DOCPEC_14880 Phage protein
ATGACGTCGATCCCGACACTGGCGCTTAATGGTGAGGCCATACTGCTGAAAAACATGCGCGTGACCGTTTCCCAGCAGTTTCAGGATAAAGACCAGTCCGGTCAGACCAGCGCGACCACCA
AATCAGAGCAGGGGGCAAAGGGCAAAGAACTGCGTATCAGCGGCGAAATACCCTTTAAAAACCCGGAGATCCTGAAGCGTATTTTTGAACTGGCCAGCACCACTGATGCAGACGGGAAA
CGCATGAAATACCGCGTTGCGCATGAGGTGGCCAGAGCGGTGAATTTTCGTGAGGCCACCTTCAGCGGAATGCTGGATGCACCGCCGCAGGACGGGAAAATGGCCTGGCTGGTCACGTT
CACCCTGGCGGAACATGTCAGCGTACAGGAGAAGCGGGAAGCCAGGGCAACCGGTAAAACAACGGCAAAAAAACAGACGGCCAGCAGTACGGGACAATCCGGTGGCCAGAGTGCCGG
AGAGGATGAAGAAAAACTGACGTGGTTTGAACGCAGGGTGCTGAAGCCCGTCAATGATGCTTTAGGTTAA
>DOCPEC_14885 Phage protein
ATGTGGCGTGAAGCGCGTCTGGCTTTTACGGATTCTCTGGCTGCGCTGGATTGTTCTGTCGTTCCGGCGCATCCGTGGATTCACGGTCTGGGGCAGCAGACAGATAACGGGGCATACCTGA
GTCCGGTCAATGCAATCCATTATCTGGCGGAAAGGCTGGCCGGAACGGGAGGCAATACCGATGTGGTGATCATGATGGTAACGGGACAGACCCATGAAAACTTCATGAAGGGGCTTAACA
GCCTGGTGGATGTTTTCCCCGCGCCCGCATTCACCCAGGTTAGACGTCTGGCTGAGTCTGCGGCGACACTGGCTACTGAGAAAATGCAGATCCCCGCGAAAGCCGGGGCAGGACTGCCGG
TTGCCATTCCGCTGTCCGTTCCGACCAGCAGGGCAGCGTTATCCGCTGCCGCTATCAGTGAGGCGCAGAAAGCGGCTGGCGCCGGATTCAGCCTGGACGGGCTGAAACAACAGCTGGGG
GAATTTACGCAACTGCGTGACAGCCTGATTAACGATGTGGCCAGCGGCCTTGCTGATTTGCAGGGGAAAAGCGCCAGGGCATGGGTATTTACGGCCAGTGGCGACACCGCCGCCACCCTT
CTGGCGCTGGTAAAGGACATTCCGCAGCCTTCAGCCGTTTATACTGCGGCAATCATGCTGGCCGGAAAAAATCTTGATGGAATAAGGGGCATGATTCATGACGTCGATCCCGACACTGGCGC
TTAA
>DOCPEC_14890 Phage tail protein
ATGAAAGCGGAAAGAAACAAAAAGAGCGAACAGTTTTTAAATATTAAAAATTTCATCCCTTATACACCGGATGCATCATTCCCCGGTGCGGCGCATCTTAAATCAGAGGATGGTCAGGACTG
GTACGGGTGCCAACAATTATTTTCAGCAGACACGCTGAAAATTACCTACGACGATAACGATGTTATTACGTGTATCACGCGCGATATTTCCGGTTTATGGCCTGCTGGCCAGAGCGTGGCGGA
GCTACCTGATACGGATGAAAACCGTCTCGCTGATATTTCAGGCGGCTGGCAGTTTAAAGACGGTAAAGTCGTTCAACGGGCTTATTCGCCGGAAGAGCTGCGTAAAAAGGCGGAGGCTGA
AAAAGTTCGTCGTCTTGCTGAGGCTGAATCAGCCATTGCACCACTGGCGCGGGCAGTAAAACTAAAAATTGCCACAGATGAAGAGATTAAACGGCTTGAAGCATGGGAACTCTACAGCGT
AATGGTTAACCGTGTGGATACAGATTCCCCTGACTGGCCGGAGGTACCGGATGTGGCGTGA
>DOCPEC_14895 Phage tail protein
ATGTCACAGACAACAATTACACTGGCATTTGAACAGTGGAAAGCGCAACAGGGTGCCACGGGGGAGCCTGTCTTGCTGGATGAATTTGTGTTCGCTAACGTACCGGGACTTGATCCGGATC
AGCCGGTTGACCGCAATGAAACCCTGCCACCGGCTGAACAGATTGTTCACCGGCAGGCCGTCAGCCGTAAGGGTGTGGTGAATGACAACGCCGTGGTGCATTCCGTCGTACTGGGGGCG
GACGTGGGGGATTTTTCCTTTAACTGGATTGGATTGCTGAATAAAGCCAGCGGTACGCTGGCAATGATTGTTCATGCGCCATTACAGCAAAAACTTAAAACAGCTGAAGGGCAACAGGGGA
ACGTGCTTACGCGCTCGTTTCTGATGGAATATAACGGCGCACAGGCTGAAACCGGAATTAATACGCCTGCTGAGACCTGGCAGATTGACTTTACCGCACGTATGGCCGGAATGGACGAGCG
CCAGCGCCTGGAAAATATCGACATCTTCGGGGCGGCGGCGTTTTTTGGTGACGGCTATCTGGTCGGGAAAAGCGGGAATCAGTTTTATGTGACCAAAGGTACCGGCTATGTGGCAGGGCT
GCGCACAACGCTTGCAGAAAACCTGAATATTACCGTGACAACCAGGCCGGTCAAAGTCTGGCTGGATGTATGCTGGACAGGAACGCTTACCAGCGTGTGGGGTGTGCAGTCCCGTATTACG
GTTGCTGACAACCTGGCGGATTATGTGCAGAACGGCGTACAGCATTATGTGTTTGCGGTGGCGGGTATTGATGAAAACGGCAATATTACGGATTTACGCCCGAAAGGGACGCTGAATGAGC
AGCAGGCCAGCGATGCGCTGAGAAAACATGAACAATCCCGTAATCATCCGGACGCCACAACCCGCGAAAAAGGGTTTGTGCAGTTAAGCAGTGAAACAAACAGCGATTCGGAGATGCTG

GCCGCAACGCCGAAAGCGGTTAAAGCGGCTATGGATAATGCGAACGGACGACTGGAGAAAAACAGTAATGGCGGCGATATTCCGGACAAAAAACAATTTGCGAGAACTATCGGCGCGGT
AACGTCGACCACCATTACACTTGGCGAATCAGGCTGGTTCAAAATCGCCACGGTTGTAATGCCGCAGTCCACATCAACAGCGGTAATTAAGCTTTATGGAGGTTCGGGGTATAACGTTGGAT
CATTTGAGCAAGCGGCAATCAGTGAGCTGGTACTGCGTGCCGGTAATGGTTCACCTGTTGGAATAACCGCCACATTATGGAGGCGTTCACCTTCTGCTGCTAACGAGGTCGCATGGGTTAAT
ACATCAGGCGACACCTACGATATTTATATTAATATCGGCCAGTATGCGTACTGGTTAATTGCGCAATATGATTACACCGGTAATGCAAATGTCACGCTACACAGTACGCCTGAATATTCATCAGTA
CAGCCGGGAAACTCAACCAGCGGTCAGACATATACGCTGTATAACAGCCTGATGAAACCTACTCCCGAAGATGTCGGAGCGCTGTCAGTTAATGGAGGGAGGCTAAACGGTCCGTTAGGCA
TTGGTACTGATAATGCGCTGGGCGGTAATTCGATTGTATTCGGCGATAACGATACAGGGTTTAAGTGGCACAGTGACGGCGTTCTGGGGATTTATGCCAATAATGCTCTGGTTGGTTATATCG
ACAATTCCGGGCTGCACATGTCAGTAGATGTTCTCACTAATGGTGCCGTACGCGCAGGCAACGCAAAAAAACTGTCACTGACGAGTAATAATAATTCAACAATGACTGCCACGTTTAATTTGT
GGGGTGACGGAGGAAACCGCCCAACGGTTATTGAACTGGATGACGACCAGGGATGGCATCTGTACAGCCAGCGAAATCCCGATGGTTCGATTGTCTTTACGGTCAATGGAGATATCAATGC
TAACACACTTCGTGCAGGCGGGGCCATCTACGCCAATAACGGAGACGTATCAGGCACCGTGTGGGGGGGAGGGAATGCAGCCTGGTTGAGCGGCTACCTCTACTCGAATATGGTCAAAGC
GATCAGACTCGGCCCGGTGGCGCTTTCTGGCGGTCTGTGGCGTGATTTTCAGCTCGGCGGCGGACAGGTGGTGACGGGGTTCCATACTGACGGTAGCTGGGAAATGGAAGGTGATGATG
ACAAGGTTTATTACCGTCCCATTCAGTATCTGATTGGTGATACGTGGGTAACGGCACCAAGTGTATAA
>DOCPEC_14900 Phage tail protein
ATGAATGAGTTCATGAAAAAACTGGCCGGAATGGTACTTCCCTCCTGGATGGACAGGGGCGAGCCGCGAAAACTGCTGCAAACGGCGCGGCGATTCTGGGCGGAGGTGTACGGCTGGGT
GACGTGGCCACTGAACCAGTTTGATCCGCTGACCTGTACACCGGCGTTACTTAACCTGCTGGCGTATGACCGGGACATTTCCCGCTTTGACGGGGAGCCGCTGGAACTGTTCCGCAGGCGT
GTGGCGTATGCCTTCGTGAATGCGCGTGACGCCGGTTCTGTTGAGGGATTTATCAGTATCTTTGAGCGGCTGGGGATCGGGTACGTTGAACTGATGGAGCGCCAGCCGGGCATTGACTGG
GATGTGATTCAGGTTCGCGTCACGGACAGCCAGATTGCGACTAACACGCAGTTGATGATCCAGATTATCCGGCAGTACGGGCGGACATGCCGCCGTTACCAGTTTGAAGTGATCACGTCCG
AACGGCTGACTATCCGGGCGGGATGGGATCAGGGGGAATATGTGGTTTATCCGGCAGCACTGAGCGGTACGGAAACCAGCAACGCGACGTACAGCGCAGGGTTATAA
>DOCPEC_14905 Putative baseplate protein gp29
ATGACGGAAAAGCCACAGGTTGACTTTGAAGAGGTGGTGAAAGCCAGCGGTATGCCGGTGACGGAAGAAGAGATTCGCGATCGCTTTAATGCCATTGCGACGGAGGAGGGAATTATCAC
GAATACCTCCCGTATGTCTCCGTTCTGGCGACTGGTCACGGCCATTGTAACCGCGCCGGTGATGTGGCTGAAGGAGGTTCTGATCTCCACCGTACTGGCCAATATGTTTGTGGCCACGGCCA
GTGGAAGCATGTTACGGCTGCTGGCATGGGCGGTGAATATCACGCCGAAGCCCGCCAGCGCTGCACAGGGCGTTATCCGTTTTTACAAGGAAGACGCCAGCGCCGTGGTGACGGTGAAG
GCCGGAACGGTGATACAGACAGAACGTATTAACGGCAGGGTGTATGAACTGGCCATCACGGAAGATGTGGTGATCGCCTCCGGTACCGCCAGCGCACTGCTGCCGGTAAAGGCAACGGG
AACGGGCGGCGCATATAACCTTGCGCCGGGATATTACCGCATTCTGCCGGTGGCCGTGGACGGCATCAGCCATGTGGCCAGTGAAGAGAACTGGCTGACCGTACCGGGCGCGGATGAGG
AAAGCGATGATGAGCTGCGTGAGCGTTGCCGTAACCAGTTTAACCTGGTGGGCAACTACCACACGGACGCGGTGTACCGGTCGATGATAGCCGGTGTTGCCGGACTGAGCATTGACCGGA
TTTTCTTTGAGCACGAAGCACCGAGGGGGCCGGGGACAGCCAACGCCTATTTATTGCTGGACAGCGGCGTGGCTTCTGCGCCGTTTGTGGATGCCGTGAATGACTATATCAACACGCAGG
GGCATCACGGCCACGGGGACGATATGCAGTGTTATGCCATGCCGGAAACCCTGCACGATCTGGCGGTCACTGTCTGGGTCAGGAACCTGAACAACATCAGTGATGATGAACAGAAGCGCC
TGAAGGACGGTATTGAAAACCTGATCCGGTGCGCCTTCCGGGAAAATACGGACTATGACGTCAGAAGGACGTGGCCGTATTCACGGTTCTCCTTCTCGCAGCTGGGGCGCGAAATCCATAA
AAACTTTCCGGTAACGGAATCGCTGAATTTTTCGCTGGATGACATTGCCAGTGAGCTGAATGTGCCGCGCCTGAAATCGCTTGTGGTGAGTATTGAGAATGAATGA
>DOCPEC_14910 DUF2590 domain-containing protein
ATGAATGAAATCCTGTATGTTGATTTATTAATTCAGGGGAATGACTTTGTCCTGAATACCGGTAATGAACCTGAATTATGTAATAACCGTAAAAGTATCGGGCAGGACATTATTCATTCCATTATT
GAAAGCGGTCTGGCGACGGAATTAATTGCCGAGAGAAGCCCGACCATGCGGGCAGATATTTTTACCCGTATGGAATTACTGATTGAGGATGATGAACGTATCGTTCCGGGAACAGTGGAAA
TCGGTGAAGAAAGCCGGACACGGTTGTGGATCACGGCCAGCACTTACGACTTCGGCGGAATATCGGTACAGGTGGATTTATGA
>DOCPEC_14915 putative tape measure protein
ATGAAACAGTTAGATTTTACATTAAGCCTGATTGATAAGTTGTCCCGCCCGTTAAAACAGGCACAGAGCAGCGTCACCGGCTTTGCGGAAAAATCAAAAGCGGCCTTTATGCAGATTGGCG
GTGGTGTGCTGGCTTTAGCGGGTACAGGAATGGCCATACGGGGGGCGTTATCACCGGCAATTGAAATGTATGATGCGCTGAATGATGCAGCATCAAAAGGGATTGATGATCAGGCATTAAA
AGCCGTACAGCGGGATGCGCTGCGCTTCAGTACAACTTATGGTGCCAGTGCGGTGGAATTTGTTCAGTCCACTGAAAGTATTAATTCCGCCATTGCCGGGCTGACCGGTAATGAACTGCCGA
AAGTGACAAAAGTTGCTAATACCCTGGCGTTTGCCCTGAAATCCACCGCCGCAGAAACGGCAGAATTTATGGGGCAGATGTTTGGTAATTTTTCCGCCGATGCGGAGCGTCTGGGCAAGGT
TCAGTTCGCTGAACAGCTGGCCGGAAAAATGGTGTATATGCGCAAGGTCTTCGGTACCGAAATGGGCACTATCAAAGACCTGATGGAAGGGGCGCGGGGCGTCGGTACCAACTACGGCGT
CGGACTGGATGAACAGCTGGCCGTACTGGGGCAGCTTAACCGCACGCTGGGAACGGAAGCCAGCAGCGCTTACGAAGGCTTCATGACCGGAGCCATTGAGGGCGGTAAAAAGCTGGGG
CTGTCCTTTACGGATGCCACCGGCAAAATGCTGTCCATGCCTGAGATGCTGATTAAATTGCAGGGCAAATACGGCAAGAGCCTGGAAGGGAATCTGAAAGCCCAGGCGGAACTGGATGCG
GCATTCGGTGACAGTTCGGCTGTGGTCAAACACCTTTACGGTAATGTGGCGCTTCTCCAGAGGAACATCACCGAACTGGGCGGATCTGACGGTCTGAAACGTACGCAGGAGATGGCCAGT
AAACTGGTGAAACCGTGGGATCGGTTTGTACAAATCCTGAAAGCTATTCAGACCGTAATAGGGCTGACACTAATCCCGGTATTGTATCCGGTGCTGAATCGTCTGGCGGATATGGGACAGAC
CTTTGCCAGATGGATGCAGCTATTTCCCAACATTGCCCGTGTTATCGGCTACGCCGCTATGGCGTTGCTGGGGTTTGCGGCAGTGGGCGCGGTTGCCAATATTTTGATGGGCGCTTCTAAGTT
CATCATGGCAGGTTTACGCGGGATCTGGGTTGCCATGACCGCCGTCACGAAAGCATATACGGCAATGGTATGGCTGGCACAAATTGCTGTTATCGCCTGGAATGCGACGCTTAAATTTTTGC
GCGGAGCGTTGCTGGCCGTTCGTATGGCGGCAATCATGGCCGGAATCGGTATTAATCTTATGAGCTGGCCGGTCTTGCTTGTGATCGGGGCGATAGCGTTGCTTGCGGCGGGTTGCTGGTT
GCTGATTAAACACTGGGATACGGTGAAAGCAGCTGTTATGGAAACATCCGCGTTTCAGGCATGTGCCAGGGTGGTGGCGTGGCTGGCCGGGGTGTTTTCCACAGCATGGCAATCTATCAGT
GAAGGCTGGAACAGTTTTATTGCGCTATTAACAGGGTTTTCACCCTCACAGGCATTAAGTGGACTGGCGTCGGGTATTGTATCCATGTTTGATAATGTCTGGCAGTCCGTTAAAGGTGGTTTT
CTGAAATCGTGGAACTGGATTGTTGAGAAGCTGAATAAAATACCCGGCGTTGATATCTCAATGGCTAATGAAACCTCTTCGCCACCATTAACAGTAAATAATTTATCTACAGGTGGCGAGCTA
AAAGGAATTGATAAAGGTGGTATCAGTAAATCTGTCAGTAATAACTCAAGGTCTGTGACGGATAACAGTCGGAAAATTAATACTGTCAATATCTATCCAAAAGAAATGATAACGCCGGGGCAG
TTAATGGAGTTTCAGGAGCTGGGCGTATGA
>DOCPEC_14920 Phage protein
ATGGAAAAAATTAAACTGTGTGTCTGTGGGACTGATATTATTTTTGAACCAAATCAGACCGCCTATAATAAGTTTATTAATGAAATGGCAATGGACAATAAAGTGGCACCTGCGCATAACTACC
TGACGCGTATTGTTGCAACGGAAAGCAAGGAAGCTTTAGCTGAAATATTAAAACGTCCGGGCGCTGCGCTTCAGCTTGTCAGTAAGGTTAATGATATTTACGCCCCTGAACTGGAAATTGAA
GTAAAAAAACTGA
>DOCPEC_14925 Phage spanin Rz
ATGGATCGTGTGGTGGCGGGCTGGCTTATAACGGTTGTTCTGGCCTTCTGGGCAGGCTGGAAGGCGGCTAACTGGCAGCGTGACAGTATCGATCTGGCCATCAGCCGGTCAGCCAGCGCT
ACCGGGGAAACGCTGGCGAGCATGGCCAGTGAATCCGGGCGAAAACTGGAAGAACAACTGGAGGCTTTGAAAAATGCACCGCCGCGTGAAATTCGTACGGAGGTGGTTAAGCCGGTGT
TTACTAACGTGTGCCTGTCTGACGACTTTGTCCGCATGTACAACGACGCCGCCGCCAGTACCGAACGTGCGTTATCAGGAAAACCTGAAAACTAA
>DOCPEC_14930 Peptidase-M15-4 domain-containing protein
ATGACACTGAGTGAAAAACAGCAGCTGTTTACCGTTATGGTGGCAAATCTGATCCACTGGGCAGAAGAACACGGCTACCGGCTGACGTTCGGGGAGGCGTACCGCATGCCGGAACAGGC
GGCGCTGAACGCGAAAAAGGGCAGCGGTATTACCAACAGTCTGCATACACGGCGTCTGGCAGTGGATTTTAACCTGTTTGTTAACGGCCAGTACCAGACCAGGACAGAGGATTACCTGCC
GCTGGGCGAATACTGGGAGTCACTGGGCGGCAGCTGGGGCGGGCGCTTCAAATCCAGGCCGGATGGTAATCATTTCAGTCTGGAACATGACGGGGTTCGCTGA
>DOCPEC_14935 Holin
ATGCAGGACTACGAAAAAGGGTTTATTGCGCTGGCAATTATGGGGGCGCTGATTGCCCTGGGCAAGATGCTGAACAGTGACGAGCCGATCACGGCCCGTCTGGTGCTGGGGCGTGTCATT
GTGGGCAGCGCGTTATCAGTAGCAGCAGGGGTGGCGCTTTACTTCGTACCGGATATCCATCCGCTTGCGCTTGCCGGCATTGGTTCAGCGTTGGGGATTCTTGGCCTTAATGGTGTTGAAGC
TTGGCTACGTAAGAAAGGGATCGGTTTTCTGGGGAAGGGGGCTGATAAATGA
>DOCPEC_14940 DUF2597 domain-containing protein
ATGACCAAACGTATTTCAGGCATGTCATTTGATGCCTACATCGATGGTGAACTGATCCATATAGAAAAAATTTCGCTGGATATCACGGATAACAGCGCCGCCGCCCAGACCCGTGGTGTGCCG
GACGGCCATGTTGATGGTGATGTGGCCGCAGAGGGAGAAATTGAAGTCAGTTCTAAAGTGCTTCAGGTACTGACAGCCAAAGCCCGCGCCGCAGGTTCGTGGCGAGGTATTGAACCGCT

GGATTTTCTTTTCTACGCCAAAGCAGGCAGTGAAGAAGTGAAGGTCGAGACGTTCGGCAATAAATTGCAGTTAAGCAATCTGCTGGATATTGATCCAAAAGGCGGCAGCGTATCCACGCAC
AAAATTAAATACTTCGTGACCAGTCCGAAGTTCGTCAACATCAACGGGGTTCCGTATCTGGAAGCGGAAGCTACGGAAAATCTGATCGGGTAA
>DOCPEC_14945 Putative tail sheath protein
ATGACCTGGCCAACCGTGACCGTTAACCAGGTAAACCAGTTACTGGGTGAAACCAACGAGGTGGAGCGCACGTTGCTGTTTATCGGTACGGGTACCAAAAATGTGGGTAAAACGCTGGCT
GTTAATGCACAGAGTGACTTTAACGCACTACTGGGCGAGGGGAACAGCCCGTTAAAAAGCGATGTACTGGCGGCAATGGCGAACGCCGGCCAGAACTGGTGGGGATTTGTTCATGTACTG
GCCGCAGACAGTGAGCCGGGCGCGTGGGTGGATGCCGTCAAAGCTGCACAGGTTTCCTGCTCGGTGGAAGGCGTGGTGCTGTCGGATGATGTGGCGGCAAAAGAACAGATTAACCAGG
CGGCAACGCTGAGATCTGAACTGATTGCGCAATACGGGCGCTGGGTATGGTTCATCCTGGCCGTACAGGGTATGCAGGAGGATGAAGCCCAGGCGGATTATCTGAAACGTCTGTCCACCCT
TCAGCAGGGTATTGCAGAGAAAGCGGTTCAGCTGGTTCCGCGTCTGTGGGGGAATGAACCGGGCGTGCTGGCCGGTCGCCTGTGTAACCGGGCGGTGACGGTTGCTGACAGTCCGGCG
AGGGTGAAAACCGGGGCGTTGCTTAATCTGGGCAGCGATGAACTGCCGGAAGATGGCACCGGGAAAACACTGGAGCTGGCCACCCTTAAAGCGCTGGAAGCGCAGCGCTACAGCGTGC
CGATGTGGTATCCGGATTATGACGGCTTTTACTGGGCTGACGGACGTACGCTGGATGTGGAAGGGGGTGATTATCAGTCCATTGAGACGCTACGTATTGTGGATAAGGCCGCCCGTCGTGTC
CGCCTGCTGGCTATCGGTAAAATTGCCGATCGTTCGCTGAACAGTACGCCGGGCAGCATCGCGGCACACCAGACGTTGTTTGCCCGCCCACTGCGCGAAATGTCCACGGCGGCCAACATTA
ACGGTGTGTCGTTTCCGGGAGAGGTGAAGCCGCCGCAGGATGGAGATGTCTCTATTGTCTGGAAGAGCAAGAAGGCGGTGGATATTTACATTGTGGTACGCACGTATGAAGTGCCGCTGC
AAATCACTATCAGCCTGTTACTGGATGCCAGTCTGGAGGCCGCAGCATGA
>DOCPEC_14950 Phage virion morphogenesis protein
ATGATAGCCGGTGGCGAGCTGAATAAAAAACAGCTGACTGAATTACGTAAGGCACTGGCCAGTATGGAGTTGCCACCGCAGAAGCGCCAGCGGCTGATCTGGCGTCTGGCGAAATATGGC
GTGATTGCTGCTGCAAAAAGACATGTTCGTAACCAGGAATCCCCGGACGGCCAGAAATGGCCGGGACGTAAGACAAAACGCAAAGGGAAGATGCTGCGTAACCTGCCAAAGCTGCTTCAT
ATCCGCGAAATGCCTGAGATTCAGGCCGTACGGATCTATTTGCAGGGCGGCGGGTACCGGAACGGGGAAACGCCGGTTCCGGCAGGAACCGTAGGTTATGCGCAACAAAACGGAATGCG
GGTAAAGGTCAGCCGCAGCAGTCAGCCACGTAAGGCGGACGCCGGAAAAATGGCGACACCTGCCCAGGCTAAAAAACTGCGTGCGCTGGGGTATCGGGTGAGAACCGGAAAACGCTGG
AAAAAGCCCACGCTGGGCGATATCACGCGGACGATGCCATACAGCCAGGCCGGATTACTGATTCGAAAGCTGAGTGGTAAAGCAGTGAAAACCAGCTGGACTGTGGATCTTCCTGCCCGC
GTATTTCTGGGCATGAATGACGATGAATTTGATAAAGCGCTGGCGCGTCAGCTTCAGGCTATAGGCTTTGGCTGGAATGTAAAGGCGCAGGATATTAAGGGGAAAACATGA
>DOCPEC_14955 Phage tail protein
ATGAGCCAGACGCAGATCCAAAGCCTGACCGCTTTTTTTCAGGAGAACGTTCCGCCGCGGGCGATGCAGTCATTTGACAGCGTACTGGATGAAATGAAGTTCATCCCCGCCGCGAAGGATT
ACGGGCTGGGGCAATATCGCCAGGCGGTTATTCGGTATGACGCAGTTCTGAGCTGGGCGCGTTTTCCCTATCGCCTGTGTCCGCCGCAGTTACTTATGTCCTTACTGGCGGCGTGGCTGGAC
GATGCAGACAGAGACCTGCTGGATGAAGTCGGGCTGAGTGAAGCCGAACCTGACTGGGATGTGTCGGTGGAAGATGAGGAAACCGCCACTGTGGTGCTGACCGTTCCGATGGTGGAAG
AACTGGTGATCAGGCAGGACGAAAACGGGGCTATTCCGTGGCGTGGTGAACGCTGGTCACTGGCAGATCCTGAAATCTGGACGGCGTTAACTGCCAGCATTTTCAGTGTGGATGAAACCG
GGGCGCCGGTGAGCGGGGAAATATGA
>DOCPEC_14960 Head completion/stabilization protein
ATGTTTAGTGGAAAACCGCTGGATTATCAGGACGAGCCGCTGGCCAATAATGGTTTCTGGCCGGATCTGAATCTGAAGGATTTTCAGGTGCAGCGGTCACTACCGCCAGATATTGACGCTGA
CACCATCAGCCAGGCGCTGCTTGCCGCTGTCGCGGAGGTGAATGCCGAGCTGGAAAACGTGGAGGCCAGCTGGAAAGCGAAAGGCCATACGCTGGCGGCAGATGTGCCGGGTGTAAAG
ATGGGCGGACTTAACAGCCTGTGCGCCCAGTACATGAAAGCCGTTTTTGCCAGGGCAAAAGCGGATCTGTTGGGTGAGTTCGCCACTATCGGACGGCGTGATACCCATCCGGGGCAGGAA
AGCCAGGAGACACGGGCCGGGTTACTGGCTGAGGCGTCCGTGGTGATCCGTCGCATGAAGGGGCTTAAACGGGCAACGGTGAAAAAAGTATGA
>DOCPEC_14965 Terminase
ATGGCACTTTCTCCCGCGCAGCGTCACAGCCAGCGCATTGCGATGGAACAAAAGCTGAAGCGAAGCCAGGCGCTGGAAACCACGGAAAGTATGCACCTTCTGGTCAAAGCGCTGGAAAC
GGATGTGGAACACGTACGCAGCCTGCCGACAATCGCGGATCGCATTGAGTTTAAAAGGGATGTGTTGCTGCCGCGCTGGGTACCGACTGTTGAAGCGTATCTGGAAAGCAAGCAGGTGTA
CGCCAATCCGGTATTTGCCTGGTGTGTTATCTGGCTGTTTGACGTGGGCGAGCTGGATCAGGCGCTGGAATGGGCTGATATCGCAATCAGTCAGCAACAGGCCACACCGGATCAGTTACGC
AGCAATTTTCCCACGTTTGTGGCCGATACGATGCTGGCATGGGCGCAGGAAAGCGCCGGGCGCGGAGAAAGTATTGAGCCGTATTTCTCCCGTACGTTTGAACGTGTGGCAGGGGTATGG
CGACTGCATGAGCAGGTAACAGCCAAGTGGTACAAATTTGCGGGGCTGGAGCTGCTGCGCAATGAGGATGGCCAGCAAACTGCTGCGGGTGTGGATGATATTGAAACGCTGGAAAAAGC
CGATCATTTGCTGGCCATCGCAGAAAAACACTACTCAAAAATTGGCGTCAGAACAGCGCGGCAGACCATTGCCGCCCGTGTCCGAAAACTGACGCAGGGGTAA
>DOCPEC_14970 Phage major capsid protein, P2 family, putative
ATGCACCTTAATAACCGTGCGCGGGAATTACTGGACGGATATTCGGCGGGCATGGCGCAGCAGTTTGGGGCGCGTGATGCCAGCCGTTATTTTTCCCTGAATAACCCGCAGGAAAATGCGC
TGCGTCTTGCGCTGCTGGAATCCGTCGAATTCCTGGACATGCTTACCTGTCTGGATGTTGATCAGCTGAGTGGCCAGGTGATTTCCGTTGGTTCTTCCGTATTACACACAGGACGTAGTGAAA
GTGGCCGTTTTATTCGCCAGGTTGGTGTGGACGGAAACGACTATTCACTGGTTGAAACAGACAGCTGCGCCGCGTTGCGCTGGGATCTGCTTTCGGTCTGGGCAAACGCCGGTAAGGATG
AAAACGAGTTTTACAACCTTGTCCAGGCATTTACCACGCAGGCTTTTGCACTGGATATGTTGCGTATCGGCTTTAACGGTAAGAGCCGCGCAAAAACCACTGATCCCGAAGCTAACCCGAAC
GGTGAAGATGTGAATATCGGCTGGCATGAGCGCATGAAAACGCTGCTGGGCGGCAATCAGATTATGACCGATCCGGTGGTGCTGGATGCAGCCGGGGATTACAAATCACTGGATGCAATG
GCGTCAGACCTGATTAACGCCAAAATTCCGGCGCAGTTCCGCAATGACCCGCGTCTGGTGGTTCTGGTGGGGGCTGATCTGGTTGCTGCTGAACAGTATCGCCTGTATCAGGCCGCAGACC
GTCCGACTGAAAAAATCGCAGCGCAGTTGCTGGGGAATACCATTGCTGGCCGTCCGGCCATTATCCCGCCTTTTATGCCGGGAAAACGCATGGTGGTGACGCCGCTGAAAAATCTGCACAT
CTATACCCAGCGCAATACCCGTATGCGTAAGGCGGAGTTTGTGGAAGACCGTAAGCAGTTCGAAAACAAATACCTGCGCAATGAAGGATATGCGGTGGAAGTGCCGGAACTGTATGCGGCC
ATTGATGAATCCGCCGTAACTATCGGCAAGGTTTCCGAACCAGCGGAGGGCTGA
>DOCPEC_14975 Phage capsid scaffolding protein
ATGTCACATTTAAAAACTGACTGGCTGTGTGTTGCTACTGAAGGGGATACCGTTGACGGACGGATTATTGAACGTCAGTGGATTATCGATATGGGGGAAACCTATGACTATAACCACTATGTC
GCTTTAATCTGGCCGGAACACCAGAAAGGCGGGGGTAATTTCGGTGAAGTACTGGAAGCCACCTGGCGGGACGGGGATGACGGGCTGGCGCGGTTGTATGTCAGTCTTTGCCCGAATAT
GCGGCTGATATACGCTAACGAAGAAGACCAGCTTCTTTTCTTCTCCATAGAGCCGGAGGAAGACTGGCGCGGAACAGGACGAACATACCTCAAGGGACTGGCAGTGACGGATACTCCTGC
CAGCATTGGCACCACACGGCTGCGCTTCAGTCGGCAGCGCAAATTATCTGAACAGGGTTATTACCGTTATGTAATTTCCCGCGATGGCAAAATTAAGCAGGAAGGAAAAATGAAGAACTGG
CAAAATTTGTTTGGTATTAAACCGAAGTTTGAAGATGAAACGCCGCCAGATAATACCGCGCAGGGTGATGATAAGTTACAGGCACTGGCAAACGCGGTAAACGAGCTGGAAGGCCGTGTG
GCCAAAATTGAAAATCAACTGAATGATGTTCAGGGTGATGTGGATACTATTGCGGAAGTGGTGGATACAGAAGAATTTGCAGCCATTCGTGATAATGCAAAAGATATCGTTAAGCGTTTTAAC
GATTTGGGAAATAAATCAGTCCGTACACCAGGACGCAAAATTTCAGAGAAAGCCGGGAAGTTTAATTTCCTGTAA
>DOCPEC_14980 Terminase-6C domain-containing protein
ATGGCTAAATACTCTGAAGAATTAAAAGGCGTTGTCCGCGCACTTTATCTGCGCCGCTATACGCCAAAAGAAATTGCATCTGAATTAAATCTGCCGAATGCGCGGATAGTTTACTACTGGGCT
GAAAAATACAGCTGGGCTGATTTGCTCAGTTTTGAAAGCACTGAAGAGGCTATCGAACGCCGCTACCAGCTACTGGCCAGCCGGGATAACAAAACCGATCTCGACCTGAAAGAAATGGAC
ATGCTCATTGCTCACGCCACAAAACTGCGTGCGCAAAGCAATAAGCATAAAGAAAAGATGGCCAGCGGTCAGAACTCCGGGCAGGCAGATGCGCGGGACAGCAATGATGACGAACCCCG
CCGCAAACGGAAATACAAGAAAAACGATATTTCCTCGCTGACGCAGGAGGATTTTGACACCTGGGCGGAAGAACATCTTTTTGAATACCAGAAACACCTGCGCCGGAACATTGGCCAGCT
GGTCAGAAACATCCTCAAAAGCCGCCAGATAGGTGCGACCTGGTATTTTGCATTTGAAGCCTTTGAAAACGCGGTCATGACAGGCGATCCGCAAATCTTCCTGTCTGCCTCCAAAGTCCAG
GCGGAATACTTCCGGTCTTACATCGTAAATATTGCTGAACAGTATTTCGGGATCACGCTGACCGGCAACCCCATCCGCTTGTCCAACGGCGCGGAGCTGCGCTTCCTGTCCACCAACAAGAA
CACCGCCCAGTCCTACAGCGGCCACCTGTACTGTGATGAATATTTCTGGGTTCCAAATTTCACAAAACTTAATGAAGTGGCCAGTGCAATGGCCACACATGACAAGTGGCGTACCACCTACTT
TTCAACACCGTCAGCTAAAACTCACCAGGCGTACCCGTTCTGGACAGGCGATGAATGGAAACAGGGCAGTAAAAAACGTACTGCCATTAAGTTTCCGACCTTTGATGAATTGCGCGACGGC
GGGCGGGTTTGTCCGGATGGCCAGTGGCGCTACGTCATTACTATGGAGGATGCTATTGCGGGCGGCTTCAATCTGGCCAACATCGAGAAGCTGCGCAACCGCTACAACACAGCCACTTTCA
ACATGCTCTATATGTGCGTGTTCGTGGACAGTAAAGATTCCGTTTTCAGCTTTTCCGACCTGGAAGCCTGCGGCGTGGAAGTGGATACCTGGCAGGATCATAACCCGGACGCCGCCCGGCCA
TTTGGTGACAGGCCAGTATGGGGCGGCTTTGATCCGGCTCGCAGCGGGGATTTGTCCTGTTTTGTGATTGTGGCGCCGCCGATGTTCGCCGTGGAGAAATTCCGCGTTCTGAAGGTGATTT
ACTGGAAAGGAATGAACTTCCGGTACCAGGCAAAGCAGATCGAGCAGCTGTTTAAAAAATACAACTTCACCTATCTGGGCGTGGACGTTACCGGTATTGGCCAGGGCGTTTTTGACAACAT
TCAGCATTTTGCCATGCGTGTGGCCGTCGCCATTCGTTACGACCTGAACACAAAAAATCAGCTGGTACTGAAGGCGGCTGACGTGGTCGAAAGTCAGCGAATTGAATGGGACAAAAACCT

GAAAGAGATCCCGGCCAGCTTTATGTCCGTACGCCGCACAACCACGCAAAGCGGTAACGCCATGACCTTTGTTGCAGACCGCAGCCAGGACACAGGACACGCCGAAGCGTTCTGGGCGAT
AACCCACGCCCTGCATAACGAACCTCTGAACTATGAAAATAAACCTAAATCACGCTGGAATTTAAGGAACAAGGCAGCATGA
>DOCPEC_14985 Phage portal protein
ATGAGTAAAAAGAAACACTTCGTTAAGCGCGACCAGCGCGGCGATAAGTCAAAAAAAATGAGCATCATTACGTTCGGCAAACCGGAACCGGTTCTGACCACTGGCACCGACTACCGGGAT
ATCTGGTACGACAATGCCGCCGATCATTTTACTCAGCCAATTGACCGGCTGGCACTGGCACAACTGATTAACCTTAACGGTCAACATGGCGGTATCATCCACGCCCGTAAAAACATGATTGTG
TCTGATTATCTGTCTGGCGGCCTGACTTACGACCAGCTGGAAGCCGCAGCTTTTGACTACATCACATTTGGGGATATTGCGCTTGGAAAAATTCGTAACGGATGGGGAGATGTGATCGGACT
GGAACCCTTACCCGGCCTCTATATCCGACGCAGGAAAGACAGGAACAACGCAACTGATCAACCTGGTGATTACGTGGTGCTACAGGAAGGCGAACCGCAGATATGGCCTGAAGAAGATATT
ATCTTCATCAAAATGTATGACCCGCAACAGCATATTTACGGACTGCCGGACTACATCGGCGGCGTACATTCTGCATTACTCAACAGTGAAGCGGTCATTTTCCGTCGCCGTTACTACCACAATG
GCGCCCACACTGGCGGCATTCTCTACACGCGCGATCCCAGCATGACGGATGAAATGGAAGAGGAAATTGAACAGCAGCTGCGTGACAGCAAAGGGATCGGCAACTTCTCCACCATCCTGG
TAAACATTCCCGGCGGAGACGGTGACGCCATCAAATTCATTGAAATGGGGGATATTTCCGCTAAGGATGAATTCGCCAACATCAAAAATATCAGCGCCCAGGATATTCTGAACGCGCACCGT
TTTCCTGCCGGGCTTGCCGGCATTGTCCCGCAAAATACTGCCGGACTTGGTGACGTAGAAAAGGCCGAACGGATTTATAAAAAAAGCGAAGTCGCCCCTGTTCAGCGCCGGTTTATGATGG
CCGTAAACAATGATCCAGAAATACCGGAAAACCTGCACCTGAACTTTGATTTAAGTTACACAGAATCAACGGATAAGGGTGCGGTATGA
>DOCPEC_14990 Transcriptional regulator
ATGCGAGTTCTGAAAATCGAATGCCCGGAATGCGGCTCAAAAGCTGTTATTCGTAAAACGAACCGGAAACACCGGCAGATTGCCGATATTTACTGCGCCTGTTCAGATGTTGAGTGTGGCC
ACACGTTTGTCATGAATCTGACGTTCTCCCACACTCTCAGCCCAAGCGCTAAAACAGGTGATGCTATGGTACAAAAAATACTGAATGCCCTTTCACCCGATCAGCGTCAGATGGCATTAGACC
TACTGAAAGCGACTCCCGCCGCCTGA
>DOCPEC_14995 hypothetical protein
GTGCAGGATTTGTTTTTGGAAACCATCGCATTGCAACGGATTGCTTTGTTTACCAGGTTGATTGCAAACAGTAAGTGCACTGGCTGTGAAAAAGATATAGCACTGGCTTGGCTGAGCGAATT
GACTGCGGATCTGGAAAGTAAGCTTGATGAATATGAAGGCAAAAGCCCCCAAAAAGGGGGCTTATCAGGCGGCGGGAGTCGCTTTCAGTAG
>DOCPEC_15000 hypothetical protein
ATGACGCTGGCAATAAACGAAGATTGCTACGCCGTTGATGCCTGGCGGCGTGAAACCTTCGCGCCAGGTACACCGGCAGACGTAACAATCACGGAGCGCCGCCTGTGGGCTGTAAACCCG
CAGGATCATAAATGGCGTGCTCAATACCTGCATGAAATACCCGACTGGTTAGCAGGGTATTTTGGCCGTCGTTACGAAAAGCTTTTTACTGGCCCTGACAGGCGTCGCCGTGCCAATACATTC
CTGCGTCAGACTATTGGAGGGAATGTATTGCCACGTCTGCGCAAAGTGGCTGCTCGTTATAAGCTGGCCGCTGATGCAATAGACCTTCCTTTTGGCAAGTCGCTGGAACGCCTGCCGTCACT
TGACCGCCCGGAACTTAAAAAACTGGCTGGCCAGATATCTGGCTGGATCTCCCAGTCGCTGTATGACTTCACAGAACGGTTTGATTCCGGCACTGACGACGCTAAAGAGCTGCACCGCCGA
ACGATGGAGTCTTATCGCTATCTTTGTGCGTGCAGTCGGATGCTGAATAATCAGCCGCCATACTGGGCAGAACATGAAGCCAATGCCGGGCAACTGGAAACACGTAAGGCTGAATCCGGCA
TTCTTCGCATGATGGCACCTGAATGGTGGTATCTGCGCCTGAAGCGAGCGCGTGACGTACAGCGTGAGCATATGGCCATAGCCGTGGGGCAGGTGCAGAAAGCGGCCAGCGCTTATGTATC
CCGTAAAACGCTGGGGGAATGGATAGAACAGAAAAAACGAAATCTGGAGTTCTTTAAAAAGTTTGATCTGCTGAATGATGAAGGGTTGCGCATTGCACTGGACAGCATGGTACACCGCAG
TGTTGCAAATCCGGCGATCCGTCGCTGTGAGCTAATGGTAAGAATGCGAGGATTTGAAGATATGGCCAATGAAGAAGGGCTGGCCGGTGAGTTTTACACTATCACCGCGCCATCTCGTTTCC
ATGCGGTACACAGCAAAGGGGGCTTTGTATCGCAATGGGATGGAAGTACGCCGCAGGATACCCAGCGCTATTTATGTGGCGTATGGGCAAAAGCCCGCGCCGCGATCTCGCGTGCGGGTAT
CCATGTATTTGGGTTCAGGGTTGTCGAACCTCACCATGACGGGACACCGCACTGGCATATGTTGCTGTTCATGCGCCCGCAGGACGTGGACACGGTGCGAGATATTCTTTGTTATCACGCCA
GAATTACCGATTCCGAAGAACTGCAAACGCCAAATGCGCTAAAGGCACGTTTCCATGTTGAAGCCATCGATCCCGCTAAAGGGTCAGCGACGGGCTACATCGCCAAATACATTTCCAAAAAC
ATTGATGGATTCGCGCTGGATGGCGAGCAGGACGAAGAAACCGGCGAAAACCTGCGGGATATGGCTAAATCTGTTTCTGCATGGGCTTCACGCTGGCGCATTCGCCAGTTTCAGCAGATTG
GCGGTGCGCCGGTGACAGTCTGGCGTGAGCTTCGTCGGTTGCGGGATCAGGTGCTGACCGATCGCAGAATGGATGCGGTTCTGGCTGCTGCTGATGTCGGGGACTGGGCTGCATATACCC
AGGCGCAGGGCGGCGCACTGGTTGCCCGCCGTGATCTGGTTGTTCGTCTGGCCTATGAAATTACGGAACAGGGTAACGAATATGCGGAAGACGTGCAGCGCGTACAGGGTGTTTATTCTC
CTTTGGTTCCAGATTCAGAGGTCTGCACCCGTCTGGTTAAGTGGCAGAAGGTTGCGAAGTTGACCGAAGCGTCAGCGGAGGCGGGTTTTTCTGGCGGCAACGCCGCCCCTTGGAGTTCT
GTCAATAACTGTACGGAGGGTGGAACCCGCAGACGGTTAAAACTGGAATTAAGAAGCCGGGGGTTCGATGGTTCTGATGAAGAAATAGACATACTGAAACGGAGGGGGAGGGCTTCGTTT
CGGTCAGTCAGCACTCATATACCGGAACGGACGACTACAGGAGAAGCAAAACGAGCCAATGCAGGAGCTGTGGCCGGGATGGTTTTAG
>DOCPEC_15005 Site-specific DNA-methyltransferase (adenine-specific)
ATGGCAGTAAAAACTCCTCTTAAATGGGTGGGCAGCAAAGCCCGCCTTATGCCGAAGTTGCGCCAGCATCTTCCAGAAGGTAAACGCCTGGTTGAACCGTTCGCCGGTTCCTGTGCCGTCA
TGATGAATACGGATTATGACGAGTATCTGATTGCAGATGTGAATCCTGATCTGGTTAATCTTTATAAAGCGATGGCATATCACACAGATGCGTTGCTTAATGAGCTGGAGAGTCTGTTTACTGC
CGGTTCGTTAGGTGACGAAGAAAGCCGAGCGGTTTTCTATTACGCGGTGCGTGATGCGTTCAACCTGTCAGGTAAATCCTTTGGTTCGGAATCCGTAGAAGCTGCTGCGCGTTTCCTGTACC
TGAACCGGCACTGCTTTAATGGCCTGTGCCGGTACAATCGCCGCGGTCAGTTCAATGTTCCGTTTGGTAAGTACAAAAAGCCTTATTTCCCTGCTGATGAAATCTGCGCCTTTGCTGAAAAAG
CAAAGCGCGCAACATTCATTACTGCCCACTATTCAGAAACGCTCGATTTGGTTCGGGACGGGAATGACGTTGTTTACTGCGATCCGCCTTATCTGACAGATAGCGATAATTTCACCGCTTACC
ATGAGCGTGGTTTTTCGCACATGGATCAGGGGCGGCTGGCGCGTAAGCTGCGGCGCCTGGCTGAACGTGGAGTTCAGGTAGTAGCGTCAAACAGCGATCTGGAAATGGTGCATTACCTTT
ATGCAGGGTTTGAAGCCTTAAAGGTTAATGCGCCGCGTAGTGTTGGTGCGGCAGCTGCAAGCCCGAAAATGGCGGCAGAACTGATACTTAAATGGCCTTTACCGACGAATCCTGAGGCCA
GCGCATGA
>DOCPEC_15010 DUF2732 domain-containing protein
ATGGCACAAATTAGCTCTGAAGTAAACGTTACTACTCCTCTTTCAAACTATCAGGAAATCTTAAAAAAAGCCGTAGATGAGGGAAAGGCAGCAGCCGCCGTTCTGTTTTCCTCTCGTCTGGAT
AAGCTGGCCACCCATGCGGCCACCGAGGGGCTTAATGCTGCTGAAATAATTGAACTGTTGCGCGAAGAATCGGTGATTTTTGGTAAGGGCGGTGCCGCATGGCAGTAA
>DOCPEC_15015 Phage protein
ATGAGCATTATTTCTGTAGAGGGTAAATCGTTGGGGGCTGAACTGGCTGTGTGGGGAGTCCCGCATAACTACGCGGTAGCGTTTGCAGAGAAAAGCGCCAGTAAAAATGGCCGCATTGCG
TTGCATCCGTTCTTCTTCAATGACACCGAACACATGACTAACCAGCGCCACTGGCTGGCGATCAATGCCGCTTTCTGGTGCTGCGTGTACCGCGAAGCTGAGAGCAAAGAGGCACAGATTG
AAGCACTGGCGGGGATTCGCGCAATTTTCTATACAGCCGGGGCGCTGGGTGTTGGCGAGATAAAGGCGCTGATTCAGGAGTGGTGGCGGACAACCTATGAGCTTCACCTTATTCCGGCAC
CGAATTATTCAGCCGTCACTACACAACCCGCTTTTCACTAA
>DOCPEC_15020 hypothetical protein
ATGATACAGCTGCGCCAGGGAATTTCCCGTGCTCGTTTAACTTTTAGCGGATTCGGGAGTTTACGGGACAGTGATTTCTCCAGACGAACCGCTGAACGTCCAGTAAAAATCGTCCATCCCAT
TAATACCAGGAACAAAGTGCAGATGATTATCGCACCTCATATTGTCCATAAATTGCAGCAGGGGAATCACTGA
>DOCPEC_15025 DUF2724 domain-containing protein
ATGAAAGCGTTTGTGACTTACCTGAAAAAAGAATCACCGGCTATGCAGTTACCCAGTGGTTCAACCGGTTGGATAGAACTGCCGAACGGCCAGCGCTGGAATCCTGGTCACACATATAAATT
TAATGCACATGAGTCTGTCCAGATGAAGGGCGGTTCTGTTCTGCGTTTCCTGACGACTAAAACCCGCCGCCTGCTGGGAATGGTTGGGGGGCGTTATGTCAATTAA
>DOCPEC_15030 Phage regulatory CII family protein
ATGTTTGATTATCAGACCTCTAAACATGCGCACTTTGATGCGGCTTGCCGAGCATTTGCGGTTGAGCACAATCTGGAGGATGTGGCCGCTGCCGTTGGTATGAGACCACAGATCCTGCGTAA
CAAACTGAACCCAGCACAACCGCACCGCTTAACCTGTGACGAGCTTTTAGCCATTACGGATTACACCGAAGACGCGCGTTTACTGGATGGGATGCTGGGGCAGATTAACTGCCTTCCATCCG
TGCCGGTGAACAATGCTACAGAAGCAAACATGCAACTGTGTGCTCTTAGCGCCACCGCCAGTGTGGGCGCAATTGCTGGGGAAGCCGTATCAACTGGTCATATGACCGCCGCCCGCCGTAC
ACAAATTCTTGATCGCGCTCGCGATGCTATCCGTAGCCTTTCCGTGTTGGCTTACACCGTTGAAAGCCGTATCCATTCTGCGCCGGTTTTAGCCGCAGCGGTGGATCTGGTCACGACGAATGC
CACTGGCCTGATGTGA
>DOCPEC_15035 Regulator
ATGAAACCTAATATTTCAATATCGTTGGCCGTACCTTCGATTTCAATTGAAAAGTACAGTGAACTCACAGGGTTATCTATAGACACAATCAATGACATGTTAGCGGATGGGCGTTTGATACGGC
ACCGCTTACGCAAAGATAAAAAGCGTGAAAAAGTTATGATTAATATCGCCGCTATGACTATCGATGCGCTCTCAGATTGCAATGTGTCCATCAATTAG
>DOCPEC_15040 hypothetical protein

ATGTCAACAACAAAGTACCCAAATGAGATCTCGCTAAATTTTGAGAGCGGAGGGAGAGAAGCCATCGAAAGGCTAGTTTCAGCATATGGCTTTACAACGCGCCAAGCTCTAGCTGATCACC
TGAAGGTATCTAAAAGTACTTTGGCTAATCGATATCTTAGAGATACCTTCCCTTCTGACTGGATCATCCAATGTGCCATCGAAACCGGAGCAACCCTCACTTGGTTAGCTACTGGGCACGGACA
AGTATTTGAAAGTGAAGAAAGTGAAGTTTTAAAAATTTCTAAAGTAAAACTTATTGACAATCAAATTTTCGATGCTGGTCATTTATTTATAGATAAGACTCTGCTTTCTCATTTTAATGATTTACT
CGCAATTGAGGACGAATATTGCACAAATATTGCACATATAAATAAGTCTAATATTGAAGATGGCAGATGGCTTGTTCAATACCAAAATAATTTTAAGTTAAAACAAATCTTTCTATTGCCTGGAG
GTAATCTTCGGGTAACAGATGATGATTTAACATTTGATTGTAAAAAAGAAGACATAAAATTAGTTGCAAAAATCTTCTCACAGTACCGTACTGTTTAA
>DOCPEC_15045 TAFII55-N domain-containing protein
ATGGATTTTGTTAAAAAACTCGAGTTTGGAAATTACACTCTCCGTTTTGGTGATGATGTTTTATTAGACTACTATGATGAAATCGTTTTCCCATCTTTTTTAGAAATGGCAAACATTCGCAGAAT
ATCTGATAAATCAGAGTTCTTTTTCATTGATACTGAATGTGTCATACTTGATGAAGAAGCAACGCCCCCAGTACTAGGAATTAAAGGGAGGATTATTAAAAACACATTACTAACCAGAGAACA
GGTATTTGATGGTGCCGATTTAGTCGAAGATCATGATGAACTTGAAACAGCACCCAGCTCTTTCTTTCTTCTAATATTAAATACGCACAGGTTAATTCTTTGCAAGGAAGTTAGTGGTGCACCT
ACAATTCAAAACTTCCAGTCAACAAGTCAATGCTTCTTAAATATAGAATATGAAAAATACATATCTCATTTGTACGAAACTGCACAAAGAGAACGCGAAGAAAACCCTGACTTGCCTCGAGTT
ACTAAAAAAAGCTTACGGAGTGAAATCGAACGGCCTAAGCTAAGAATTACGCCGTTAACTGATGAACAAAGCTTGGAGCAATTCATTGAAAATTTTAGAAAAATTCAAAATGTTTCTGTAAA
ACTGCTCCCTACAAATGAAGAGGAAATTGATAACGACGAATTTTGGGAGTCACTTGAAAGCGCTGGAGACGAAATGGGCAGCATTTCTACGTCAATTCGTTTTTCAAACACCGATTCAGGT
TTGAACCATGATGCTGTTTTAGAACAACTCACATCAGCAACTAGGTTAGCAAATTCAGGAATAAATATTAAAGGATATGATGACGAAGGTGATATAATCAAAGGCAGTAATGATGATTTTGTTT
TATTAAGTGAGATGAATGAATTATCAAAAAACACGGAAGTGGCTGCAAATGAAAGTTATGAACGATATGAAAACCTTGTAAACGAAGGTAAGATATCTCTACCCAGATCTCTTTCACAAAAGA
CGATTAGCATTATTAGCAATATATATGAAAGGTTTGGCAGATGA
>DOCPEC_15050 ABC transporter permease
ATGATAAATAAAATAGATGCTAAAGAGATAACCAAAGAAAAAAACCTTTGGGATGTTTACTTGCTTTGTAAACGGATTACTATTAGTACATTCCATGTTTGTATTTTGCTCACAGCATCTATTTTT
TTATTGACAAACTCTTTTTTTCTTGAGAGGGACATGTCACATTTAGTATCTGATATTAGAAACTGGGCTTTGATTGGTTTCAACTTTGCAGTGACAACACTAGGTTTTCTTATTGCAGGGTTCA
CTATATTTGCAACACTATCCAAACCCGATATGTTTCTTCAAATGATGTCAGTACAACATAAAAAAACACAGATGCCCACGTTAAAATATAACTTCATGGCATTTATGAAAGTTTTCATCTCTTTTAT
TACATTTACTTTCATTTATCTAGTTATAATTCTTTTTTGTCAGACAAATGGAATGATAGGAAATATGGTTGATTTAGTTCCATATTCAACAGTGATAAAAGAATTAATTATCAAACTCGGATATTGG
GTTATAGGGACTAGCCTTATTTACTTAGTATTAGTAGTAAAGACTTTTATTTTTAATATTTATGCCATCATAATGAATAATATCCGTTGGGAATTATATATTAAAAGAAAAGAACAAAAGTTTTCCA
GTAACAAAGGGAAAATAGATAAAAATATAAATGTTACTAAAGTGCACTAA
>DOCPEC_15055 Integrase
ATGGCAGTACGAAAACTCACAACCGGAAAATGGCTTTGCGAGTGTTACCCCATGGGACGCACTGGTAGGCGTGTTCGCAAGCAGTTTGCCACTAAAGGTGAGGCTCTAGCTTTTGAGCGT
CACACGATGGAGGAAACCGAAGCAAAGCCCTGGCTGGGCGAATCAGTGGATCGTCGGACACTGAAAGATATGGTTGAGCTATGGTTCAAATTACATGGCAAATCTCTTACTGCCGGACAGC
ATGTCTACAACAAGCTGCTGTTGATGGTTGACGCCTTGGGAAATCCCCTTGCAACTGATCTCACCTCAAAAATGTTTGCTCACTATCGCGATAAACGCCTTACTGGTGAAATCTACTTCAGTGA
AAAATGGAAGAAAGGAGCAAGCCCAGTTACTGTTAACCTGGAGCAAAGCCATCTAAGCAGCGTTTTCAGAGAACTTTCCCGCCTTGGTGAATGGACACTTCCAAACCCAATAGAGAAGAT
GCGCAAATTCACTATCGCAGAAAAGGAAATGGCATGGCTTACACATGAGCAGATCATCGAATTACTGTCTGACTGCAAACGTCAGAACCCAATTCTGGCACTGGTGGTAAAAATATGTCTAA
GTACAGGCGCACGTTGGCGAGAAGCAATCAATCTTACCCGCTCACAGGTCACCAAGTATCGAATCACCTTTGTGAGAACGAAGGGGAAGAAAAACAGAAGCATACCTATCAGTAAAGAGC
TTTATGAAGAGATCATGGCGCTCGATGGGTTCAATTTCTTCACAGACTGCTATTTTCAATTTTTATCCGTGATGGAAAAAACGTCTATCGTTCTCCCTCGCGGTCAACTGACACACGTTCTGCG
CCATACGTTTGCGGCGCACTTCATGATGTCGGGTGGAAACATCTTGGCCTTACAAAAAATTCTCGGACATCACGATATAAAAATGACTATGCGTTATGCGCATCTGGCACCGGATCATTTGGA
AACGGCGCTCCGTTTCAATCCACTGGCAACGTTACCAAATGGCGACAAAGTGGCGGCAGCGGTTGGCATTATCCCGTAA

Prophage 11

>CFICBL_16480 Integrase
ATGCGTTACCGTTTCGACGGCAAGGAAAAGACACTGGTCATCGGACCGTACCCGCAAATCTCTCTTACCGAAGCCAGGGCAAAACAATCTGACGCGAAAATGAAGCTGCTTGCTGGCGTG
GACCCATCAGAACAGAAACAGGCTATAAAGAAGAAGGAAAAGGAAGAAGTAGCTGATTCGTTCGGTGATATCTTCAGGGAGTGGCATGCTCATAAATCGAAGGTATGGTCGAAAGGATAT
GCTGACGAAATGATGAACATGTTCACTGGCGATATATTGCCACTCATCGGACATCTGAGAATGGAAGAGGTGGAGCCGATGATGCTACTGAAGGTGATCAGGCTATTTGAGGACAGAGGGG
CGATGGAACGTGCTGATAAGGCTCGTCGCAGGTGTGGCGAGGTTTTCAGCTACGCAATAGTAACCGGAAGAGCTAAATATAATCCGGCTCCAGACCTTGTTGGGGCAATGAAGGGTTACAG
AAAAAACAACTACCCTTTCCTACCTATGCATCGCATTCACGAATTCCAGAGGGCGCTGAATGGGTATGGAGGCTGGGTTATAGGTAAGATTGCTGCTCAAGTTCTTCACTATACAGCAATGCG
AACAGTGGAGTTACGTTCGTTGGTATGGTCAGGAATTGACTTTGAAAACAGGCTGATCACCGTTGACCCTGAAGTCATGAAAGGAAGAAAACTGCATGTCGTTCCAATGTCAGAGCAAGTT
ACAGCGCTTTTCAAATTCCTGCAACAAATCACCGGACAGTACGAACTTTGCTTCCCGGGAAGGAATGACAGGAAGAAGCCAATCAGCGAAAATGCCGTCCTTGGTGTAATCCGCGGCATAG
GATATGAAGGGCAGACATGCGGACACGGTTTCAGACATCAATTCAGCACGGTACTCAACGAGAAGCACTGGAACAGCGACGCAATAGAGATGCAGCTGGCACACGTAAGCGGCGGGACG
CGCTCAGTTTACAACCATGCTGCATATCTGGCTACCCGCAGAGAAATGATGCAATTTTGGGCGGACTGGCTTGATGAGAAGGTGTCGTAG
>CFICBL_16485 hypothetical protein
ATGACAGATATCACTGCCAACGCAGTTGTTTCTAACCCTCGCCCAATCTTTACTGAATCCCGTTCGTTTAAAGCTGTTGCGAATGGGAAAATTTACATTGGTCAGATTGATACAGATCCGGTTA
ATCCTGCCAATCAGATACCCGTATACATTGAAAATGAGGATGGCTCTCACGTCCAGATTACTCAGCCGCTAATTATCAACGCAGCCGGTAAAATCGTATACAACGGCCAACTGGTGAAAGTTG
TCACCGTTAAGGGTCATAGCATGGCTATCTATGATGCCTATGGTTGTCAGGTTGACTATATTGCTAACGTATTGAAGTATGACCCAGATCAGCTTGAATACAGGCTGAGCCAACCAGACGGTTA
TCTTTTGGTTGGTGGACTGGCCGAGCATTATAACCTTCCGGCTAAATTTGTCGTCGTCGACAACGAGCCATATAACGGTGATTTGAAAGCAGCACTTTCTGAGGCTGAAGCCGGTACTGTGT
TTTGGCTTGGTAAAAAAACATACAACATTACCGGCCTGTACGGAACTGGCAGGAACACAGTTGAAAATATCTCTATTGTTGGCACTGGGATGCCTCAGTTATCTGATGATAAAACCAGATTTA
TCGATGGAACTGGGACAGTTATTCAAGGGGCGGTAAAGAATCAAGCGAGAGGGTTTAAAACCTACAACCTTGGGATCGATGTTGGTGCTTATGTGTCTCAGAATGTGTATACAACGGAAAC
TTACGAGGACGCTCTTGTGCACTATGGTGTTGGTTCCAACGCCAATATAGAAATTGACAATGTCAAGACGCTGAGTTCAGTTAACGTTGCCAGTAAGCCAGGAACGCACAGCATTCTTCTTG
AGCAATTATCCGGTGTAACCTTGGGTTACGTTGAGTGCATTGGTGGTTTTCATGGTCTAACGATTAAGTGTCAAAATTTGCAGGGCGGCATTGCTCATTGCTACGGCCAATATGGTGATGCAT
TTATCTTCAAATCAGATTCTGGTGGCGCATGCGCCAGTAACTATATGGAAAGAATTGCAGTTGGTCTTTATGACAACGCTGGCTGGCCTGACGTCACCATGGGTGGTATTTACGACGCCCACG
ATGATGTAACAATCGACAGAATTGGCATTGGTGAGTTAATTGTGCAGAACGCGTCATGGGGATTTATACCATCTGATGCCAATACCGGTTTCATAACAAACGTCAGCATTGGTAGATACTCTGC
ATTCAATGTCTATGGGAACTATTATTCATTAACCATTGATAATAAATGTGTTGGTTGGACTATTGGTGAGCACAGAATTAGCAATGCATCTGGCGGCATTCGTGTTCACCCGGACTCTGCAGAA
ATCAATATTGGAACCGGATCCGCAAAGGGGAATACTGAGAGTGGCTATGCGTTGGGAGGCAACAGTTTAAGTCACGGCGTGCTCTTTGCTAATGAGAATGGGAAGGCTGGAGTTGATTAC
CTCGGTGGTATTGGTTTTGATGCCTCTCTTGTCCGTGGTTATGTTAACGGAACGGTTCTTGTTTCAGGATATCCAGGCGTAAAAGACGGTAATCCTGTAAATGGGTGGGCTGATACTGGTGAT
TTTGACATGATGCTAACCGGCAAGACTGTGCAGATCACAGGTTCACTGACTCGCGGAACAGCTGCGGTTGCGTATAACACTATCGCTGCGTGCAGGCCTTTGAAGCGAGTGCCTGTCCCGG
CATGGGGTGTTAGCGCGACAAGCTCTATGATCCCAGTTGAGTGTTATATTGAAACTAACGGTCAATTAAACGTAGCTGGGTTCGCCTCGATACCAACTGGCGGAACTGTATATTTCTCTGGAC
AATATTTAACAAAGTAA
>CFICBL_16490 Phage antirepressor Ant
ATGTCAAGCTTAGCAAAGTCAACTGTAAATTGCACTAATAGCATCATCATTTCTGACGTCAAGATTCATATGGATTCAGAGGGGCGTTACTCGCTTAATGACCTTCATGTAGCGTCTGGAAAGG
AGGAAAAACATCAGCCAGCTTTCTTCATGCGTAGAAATGAAACTATTGAATTGATTAATGAAATTTTTAATTCTGCGGATATGCAGAATAAGAATCCCGTCATTTCTAAGAAAGGTCGATATGG
TGGAACCTACGTGTGCAAGGAGCTTGTTTACTCCTACGCCATGTGGATTAGCGCAGCATTTGCGCTGAAGGTTATCCGTGCATATGACGCAATGGTTACTGCCACACAAGAGAGGAAGACTA
TTGGCGGTAAAACTTCAGTAGCTGAACGCACACCGCTACGCGATGCAGTAAACATGCTGGTAGGAAAGAAAGGACTTCGCTATGACGATGCATACAATATGGTTCATCAGCGTTTTGGTATT
GACAGCATTGATGAACTTTCAATTGAACAAATCCCGCTGGCCGTAGAGTACATCCACAGGGTAGTGCTTGAAGGCGAGTTCATCGGCAAACAAGAGAAGAAAACCAACGAGCTTTCTGCA

AAAGAAGCAAACAGCCTTGTATGGCTATGGGATTATGCCAATCGCTCACAGGCATTATTCCGCGAACTCTATCCGGCATTAAAACAAATTCAATCGAACTATTCCGGCAGATGCTACGACTAC
GGTCATGAGTTCTCGTATGTTATCGGAATGGCGAGGGACGTTTTAATTAATCACACACGAGATGTTGATATCAATGAGCCAGACGGACCAACGAATCTTTCCGCATGGATGAGACTTAAGAAT
AAAGAATTACCTCCTTCAGTACATAACTACTGA
>CFICBL_16495 sar RNA
TCAATAATACCTCGTAAGTTTTGTTGATACCGAAGCCCTGACTGTTCCCGCAGTTGGGGCTTCACT
>CFICBL_16500 DNA-directed RNA polymerase
ATGAGGTTTATTGAGGTTGATGAGATTGGCTATGAGAATGCACTTGGTAGAGGTAAATCTTTAGTTAACATTGATGGCATTGAATCGATAAGGAGTGGTGAATTTACGGAGATCAGAATGATG
TCAGGCGGAATCATTCCTTGCCGTGACTCAGTAGAGTCAATCAAATCAAAAATGAGTGGTCAGTGGCTTTATAGCTACAGTGACTTACAAAAACAGTGA
>CFICBL_16505 Arc family DNA-binding protein
GTGGCTAGAAATGATCCACAATTCAACGTAAGAATGCCTGATGAGATAAAACAGCAACTTACGCATATTGCTGCAACAAATCGCCGCTCGATAAACGCAGAGATTATTTCAGCTATCGAACTA
TGGATAAAAATTCATAAAGGGCAGCTAATACCTTCAAGTAGTCGAATCCTGACAAAGTCTGAGCAAGAGGCGTTTGATGTAGCTATTGATGTGTTAAAACGTGTAAGAGATGGAGGATAA
>CFICBL_16510 Signal peptidase I
ATGGAGCAACAACAAGGGTCGCCATTATCATTGATTATTATGGTGATTTTTTTCGTTTTTATCTTTTTTATACCTGCGTTAAAAATATCCAAAAAGGCCGGTTTTGACTGGAAGATGGCTGTATG
TTTAACAATCCCTGGCTTTAACGTGGTGGCGTGGCTCGCGCTTGCGTTCATGGATTGGCCAATTCACAAGTATCTACCAAAAGATGTGAATCGTAAGGATGCAAAATGA
>CFICBL_16515 GRIP domain-containing protein
ATGAATAAAAAACAGCTTATTAAGTCAAAAACGTCAAGCAAGGAAGAATTAGAGAAGGAGCTAAACTCCCTGAAATATGCTCTGTGTCTGGTTTACTCAAGACTGCCAATGGAAGATAAAA
ACGCCATTTACAATGAAATGATTAGCAGCCTTGATTTTAACGATAGAGACCTAGCATCCCACCTCAACAGCTTCCGCGTCCCTGAGTAA
>CFICBL_16520 Cytoplasmic protein
ATGACCATAGAAGAACGTCTGAACAACATTGAGTTGAATCAAACCCTGCTTGACCAGCGACTTTCAGATCTTGAGCTTAAAGATCTAGATGCGCAAATATCAGAAGCAGAAGCCAAGCTCTC
CAGCTTAAACCACCGCAAGAAGCAAATCCGAAACAGAATTACTCAGGGACGCGGAAGCTGTTGA
>CFICBL_16525 FERM domain-containing protein
GTGGTTCTGAAATTAGTTCTAAGGAGATGGCAGAGTACTTTGCAACTCGGATTAACTAACGAGATGGCAAGAAAATCTAAAAGGATTATCCCCCCTGCTGAATCTACAGGTAAGTTAAAGTT
AGGGCCGCAGTCAGGGGGGAATTCAGATCAAAAGAAACCGAAATTTTCATTCTGTTACATCCAAAGCTCTCACTGCATAACGAAATGCCAAAAAGATGAGAAAGCTGGCTTAGCTGATAAA
TTATACAGATTAAGTCAACTAACATGGGCAGATATTAAGCAGCAAGGTAGGCATAAGCTTGGATTTGAGAAGATTGCAAGGGGTGCAATTAAAGCAGGTATCCCCAGTCATATAACGGAAGA
CGTAGATCATTTCTTGGCTTTTAGATTTGATGATTTGAAAGCCATGGTTGGTTATCGTCTTGGATCGACATTTTTTGTTATATGGCTTGATAGAGAGTTCAACCTATACAAGCACTAA
>CFICBL_16530 DUF4065 domain-containing protein
ATGCTTACTTGTTTTGATGTCGCCGACTACTTCCTGTCGCGGTGTGACGAGGATAGCGGTGACGCAATCTCTAATTTAAAATTACAAAAGCTTGTTTACTATGCTCAGGGTTTTTCATTGGCGT
TGCTTGGCGAGCCTCTTTTTCAGAATAAGATGGAAGCTTGGATGCATGGCCCTGTTGTCCCTGAGCTTTATCACCGTTATAAACAATATGGTAATGGTGCTATCCCTTCTCCAGAGTCTTTTGAT
GCTGAAAAATTCAGCGAAGAACAGTTAGAGCTATTAGAAGAAGTCTGGGATGTTTTTGGGCAATTTTCTGCCTGGAAACTGAGGAACATGACACACGAAGAGTCACCATGGAGATCAAAC
TATATTGAGGGTGTTGGTGGTTCTGAAATTAGTTCTAAGGAGATGGCAGAGTACTTTGCAACTCGGATTAACTAA
>CFICBL_16535 DUF2846 domain-containing protein
GTGAAGCAATTTCTTGCTGCTATGTTCTTATTCATATCTTTTGGGGCTACAGCAGAGTGCTGGGTCGTTGGAGATATGCGCGGAATAAGCTATTCAGAACGAAATAATTTCCATCCGGAAGAA
GATGGTTTTAGTGGAACATTCATCATTAAGACAAGCGGTGAAGATGCTAGCATCACATATTCTGGGACAGATGCGGGCGGCATGGCTTACAAAGTATTGTCTAAAAACTCCATCATAGGAATC
GGCGCGAATGGCGAAACTCAACGCGTTATCGACTCATGGGTAATACATCCTACTGGAACAGTTTTAATGTCAAAAACCATTTCCGGTTATGGAAATATGGATTCAACCAAAGCTTTTGTTGGA
AAAGTAAAAAGAAAATGTTAG
>CFICBL_16540 hypothetical protein
ATGGCCTATAGTCGTGAACAATTGATGACGGCGTTAAGGAATGCTGATGCTGCTGGTGATACTGAGGGAGCACGTCGCATCGCTCAGATTCTGTCCTCCAGTAATCAACCAACTCAAAACCA
AAAGCAGCCTGTAGAACAGCAAGATGGATTTATGTCTGACCTCGGCGAAGCAGTAAAAGAAACTGGTCGCGGACTGGTGCAGGCTGGTGTAAATGTGGCAAATATACCAGCATCAGTTGC
CGATGCTGTAACAAGCGCGGCGGCATGGGCTGGCGGTAAACTCGGTATTGGCGATGGAACATATCAACCAGCGCCACGAGTAACAACTAAGGGATTAGAGCAGGCGTTTGGACTTCAGCA
AGGTGCTCTGACTCCACAAACGACAGAAGGTAGGGTGTTTGCGGAGGCATTGCCTTACCTGGCTACTGTTGGCGTTGGCGGCGCTTCAACTCAGGCGCCAACACTTGCTGGTCGAATTACT
CAAGGCGCAGCCCGTCTTCTGGCAGAAAACGCAGTCGGATCGCTCGCCGCAAACAGTGAGAAAAATGATGCGGGAAAACTGGCAACAGATATAGGTGTTGGCATGCTAACAGGTGGTGC
TGTTAATACTGTTGCAAAAGGGCTTGAGCGTGGAATAACTGCCTTTAAAGGTGATATTGCACCAGAAGTGGCGAAGAAAATTGCCACATCAGAATCGATGGGCGTGACACCAATGACATCT
GATGTAATCCCGCCGAAAAATGCTTTCACTCGTGGTCTTACTCAGGATGCTGAGGGGGCTTTGCTGGGAACAGGCTCAAAGCGAGCTGAGCAGTATGCAAAAAGAAGTCAGCTAGTTAAA
AAACAGCTTGAGAAATATGGTGAATATAGCCCATCAGTTGTTGTTGACGATCTGTATGGCTCTCTGAAGTCAAGGAAGGATTCGGCCGGAAGCGTTATTGAAGACATTACAGCCAAAATGGG
AGACACACCAGTTGACACATCAAAATCTATTAAGGTTATCGACAACGTACTTACCAGAGCTAATAGGCTTGGGAAAGTGGCAAATAAGGATTTGATTCGCGGGTTATCCGATTTGCGAGAAG
AACTTGCTAAACCAGATATAGATTTTGGTCTATTGAGAGAGTTGCGGTCAGCCTTGAGGGAAAGTATTCAGGGAGATGCCATGGTTTTTCCTAATAGCGCGAAAGCCGCAACTGATGCCGTG
GAGAGGGCAATGGGGGCGGATTTGAGGAATAACGCAGCGCGGTATTTGGGGGCTGGAGAGGCCGCCAGATACGTCAAAGCAAACTCTGACTACTCCAACGTCTTCAATAAGGTTCTTAAT
AAAAGGATTGCGAATAATCTCAACAAAGCTAAAAAAGAGTTTACTCCAGAGCTAATAAACAGCGTTGTATTCAGCAGAAAACCATCAGATATTAAGAGGATATGGCCAGCTCTTAGTGAAGA
TGGAAAGAACGCTATGCGTGCTGCTTATATCAGCAAGATTGCAGAAAAAGCAGGAGACTCGCCAACAAAATTTCTTACCGAGTTAAATAAGTTGAAGTCGCAATCTGACGGTCAGATCTATA
ACACGATATTCAGTGGGAGACACATGAAAGAGCTTGATGCTCTTCATGAAGTTCTACAGCAAACAGCAAGGTCGGACGCCGCAAATGTAGTAACACAGACAGGACAATCGCAAGCTAACA
GGATTAGAACGATTGGCGCAACTGCAACCCTTGGTGTATCACTGGGACTTGAGGCTGGTTTCGGTGCAATCATGCGCTTGTATGAGTCCAAAGCAGCAAGGAATGCTCTCTTACGTCTGGC
AAACACTAAAGCAGGAACGCCAGCCTATGAAAGAGCATTAAGTAACGCTGCAAATGCCATCAGACCGCTGCTTGCCACTGAGGCAACACAGCAGTAA
>CFICBL_16545 Acyltransferase
ATGGCTACGTGGCAACAGGGTATTAATTCTGGTGGTTTTCTGGCTGGCATCGGTACGCAAAATGAGAATGCGCCAAAGGCAAGCGACATTAACGCAACGCTTGGTCTGATCCGCGAAAACA
ATGAACTGGCTCGCTCAGGTGCAAATAACGTTGGTCTGACCGCGTTACGTGGTCTGGCTGGAGTTGCTGATATTTACAATCAGGAACAGCAACAGAAAGCGATTAGTGCGTTCAATAAGGT
TCACGCTGATGCATGGGCTTCTGGTGATCCATCGGGACTATTTAAGTTTGCCCAGGAAAATCCAGCGTTTGTTGCACAGGCACAACAGGCGTTTTCCGGTCTTAATGATCAGCAACGCAACG
ATATGGGCGATTTAGCCATGAGGGCTAACGTCGCTCTTTCTCAGGGACCGGAAGCCTACAGTAAATTCATTACTGACAACAAGGACAGGTTAAATCGCGTTGGTGCTAATGCTGACTGGATG
ATTCAGACAGGTATCCAGAATCCAGAGCAGCTATCACACATGTTGACTACTATGTCTCTCGGTGCGCTTGGACCAGAAAAGGCGTTTGCTGTTCAGGACAAGATGGCTGGTCGTGAAATTGA
CCGAGGCAGGCTGGCAGAGACAATCCGCAGCAATCAGGCAGGTGAAGCACTTCAGGCGAGAGGGCAAAACCTTTCCTATCAGTCTGCAATGACCGGACACGGACTTGCAGCAGAAAGA
CTGGCACTTGATAAGCAGAAATTCGGTTTTGAAGTACAACAGGCACAAAAAAAGGCCGAGGAACTTATTAATGCTGCGCCAAAATTATCAGTGAACATGGAAAAGGCTATAGAAAAATCAG
CAGGTGATGCGGCAGCTAGTCGTAATGCTGCCGATTCAATGACAACGCTCGCTGACACGCTGGAGAGGGAGAAGCCAACTCCTGGTTTGTTCGGTAACGCTGAAAATATGTTCTCTAAGCT
TACGGGGCAAGATAACTACCTCCGAGATATGCGGATTAGATTCAACCAACTAGCCAATGCGCAGGCAACCAAGCTTCTCCCTCCCGGCCCTGCATCTGATAAGGATATTGAGTTTGCAAGGA
AAGGCATTCCAAGCGAAACGGATAATCCAATGGTCATGGCTCGATGGTTAAGGGGTATGGCAAAAATGGAAAGTAATAACGCGAAGTTCAACGAGTTTAGGTCAGAGTGGATGAGTGCAA
ACGGCAGCCCTGGACAATCTGATCGCAACCGAAACATCATGGGGATGGATGTTAAGAAGGGTGAATCATTGAACTCTGCGGCAAAACGTTTTCTTTCCTCAAGTTATGGAGATAGCCAACCT
CAACAGCAATTGTCCGATGACGAATTAATTGGCAAATATCTCGGAGGGCAGTAA
>CFICBL_16550 Internal virion protein gp7
ATGTTATATGCATTTAAGCTGGGCAGAAAACTGCGCGGCGAGGAACCTTATTGCCCTGAAAAAGGCGGGAAAGGTGGCAGTTCTGATAAAAGCGCAAAGTATGCAGCAGAAGCTCAGAA
GTATGCCGCAGACCTGCAAAATCAGCAGTTCAACACCATCATGAACAACCTGAAACCGTTTACTCCTCTGGCTGAGAAGTATGTCGGCAGCCTCGAGAACTTATCGTCTCTGGAGGGGCAA
GGTCAGGCACTTAACCAGTATTACAACTCTCAGCAGTATAAAGACCTTGCAGGTCAGGCGCGCTATCAGAGTCTGGCGGCAGCGGAAGCAACAGGTGGATTAGGTTCCACCGCAACCAGT
AATCAGTTAGCAACAATCGCACCAACTCTTGGCCAGCAATGGCTATCTGGACAAATGAACAACTACCAGAATCTGGCAAATATTGGTCTTGGCGCTCTTCAGGGACAGGCAAACGCCGGGC

AAACATATGCCAACAACATGAGCCAGATTTCACAGCAAAGCGCGGCACTGGCGGCGGCAAACGCTAATAGACCTTCCGGCCTTCAATCTGCAATAGGCGGGGCTGCCTCTGGAGCAATTGC
TGGAGCACAACTTGGCAGCATTGTTCCTGGTATTGGTACTGGTATTGGTGCCGCTGTTGGTGGCGGTCTTGGTCTGCTTGGCTCGTTGTTTTAA
>CFICBL_16555 Protein gp14
ATGATTACATTCACTCCAACACGCAACATCGACCTGATAGAAATGGTTGGCAACCACCCCGACATCATTGCCGGAAGCAACAACGGTGACGGATACGACTACAAGCCTGAGTGTCGTTACTT
TGAAGTGAACGTACATGGTCAGTTCGGTGGCATCGTGTATTACAACGAGATTCAGCCGATGACCTTTGACTGCCACGCCATGTACCTGCCTGAGATTCGCGGATTCAGTAAGGAAATCGGAC
TGGCGTTCTGGCGATATATTCTCACCAATACCACCGTTCAGTGCGTTACATCATTTGCTGCACGCAAATTTCGCCACGGTCAGATGTACTGCGCAATGATTGGCCTTAAGCGTGTGGGAACCAT
CAAGAAATACTTCAAAGGCGTAGATGACGTGACGTTTTACGCCGCCACACGCGAAGAACTAATCGACTTCCTGAATAACGGGAGATAG
>CFICBL_16560 Phage tail protein
ATGGCAGACCCGTCACTTAATAAGCCTGTCGTGGTTCAGGCTACACGCATTGATGCTTCAATTCTTCCCAGTAACGTCTTTAGCCAGTCTTATTTGCTCTATGTAATCGCGCAGGGAGCTGACG
TTGGTGCTATTGCAGGCAAGGCAAATGAGGCAGGGAGTGGCGCTTACGACGCACAGGTTAAAAATGATGAGCAGGATGTAATTCTTGATGAGCACGAAAAAAGAATTGCAAAAACAGAA
GAGGATATTTCAGGAATAAAAGTAAAGCTTCTTGAAATAGAGAATGATGTTAATGGTCTGAAAATAAAAGTTGAGGATATCGACGGTAAAGTATCAGAGATAATCGTTGATTATGTTTCACTC
AGCAGAACAGGAACTCAAACTCTTGCCTCATCCCTTAACGTATCAGGAAGTTATTCTGTTAACGGTACAAAAGTTGTTGGCGCTCGCCAGACTGGATGGACCGCGGCAACAGGTACGGCGA
ATAAAGGCGTATTCGATGCTGACCTGACATTCGCCGTTAGCGATACTTACACGCAATCTGAAATCCAGGCTATAGCCAATGCTCTAATTACTGAGCGTCGGCGCACTAAGGCTTTGGAAGACG
CCTTGCGTGCACATGGGTTGATTGATTAA
>CFICBL_16565 Phage head DNA stabilization protein
ATGCCGATTCAGCAACTTCCGCTCATGAAAGGCGTCGGCAAAGACTTCCGAAACTCCGACTATATCGACTATCTGCCAGTGAATATGCTGGCTACACCCAAAGAAATACTCAACAGCAGCGG
ATATCTTCGCTCATTCCCAGGCATTGCCAAACGTTCTGATGTAAACGGTGTATCTCGCGGCGTCGAGTACAACATGGCGCAGAATGCTGTTTATCGCGTATGTGGTGGCAAGCTGTACAAAGG
AGAAAGCGAGGTCGGTGATGTTGCCGGAAGTGGTCGCGTATCAATGGCGCATGGTCGAACATCACAGGCGATAGGCGTTAACGGGCAACTGGTCGAGTATCGCTATGATGGCACGGTTAA
AACCGTCTCAAACTGGCCTGCGGACAGCGGATTCACGCAGTATGAGTTAGGTTCAGTGCGTGACATTACGCGCTTACGTGGGCGTTATGCGTGGTCAAAAGACGGCACTGATTCATGGTTT
ATCACTGACCTCGAAGATGAATCGCATCCTGACCGCTACAGTGCACAATATCGCGCAGAATCGCAGCCGGACGGCATCATCGGTATCGGGACATGGCGAGACTTCATCGTCTGCTTTGGTTC
GTCAACGATAGAGTATTTCTCTCTGACAGGCGCAACCACCGCTGGCGCTGCGTTGTATGTCGCACAGCCATCGCTGATGGTACAGAAGGGCATTGCCGGAACATACTGTAAAACGCCATTCG
CTGATTCATATGCATTCATCAGTCACCCGGCTACTGGCGCACCTTCCGTCTACATCATCGGGTCAGGGCAGGCTTCACCAATTGCGACGGCCAGTATTGAGAAAATTATCCGCTCATACACGGC
TGATGAACTGGCGACGGGTGTAATGGAGACTTTGCGCTTCGATTCTCATGAGCTTCTGATTATTCATCTCCCTCGTCATGTTCTGGTTTACGACGCATCGTCAAGTCAGAACGGACCACAATG
GTGCGTGCTGAAAACCGGGCTTTACGATGATGTATATCGTGCTGTCGACTTCATGTATGAAGGCAACCAGATTACGTGCGGCGATAAATCAGAAGCGGTGACAGGACAACTGCAATTTGACA
TTAGTAGCCAGTACGAAAAACAACAAGAGTTCATCTTGTTCACGCCAATCTTCAAAGCAGAGAACGCCAGATGCTTCGACCTCGAAGTTGAATCATCCACTGGTGTTGCTCAATACGCCGAC
CGCCTGTTCCTGTCTGCAACAACTGACGGTATCAATTACGGTCGTGAACAGATGATTGAGCAGAACGAGCCGTTTGTGTACGACAAGCGCGTTTTATGGAAACGTGTTGGGCGCATTCGTC
GATTAATCGGATTCAAACTGCGGGTAATCACCAAATCGCCAGTAACACTATCCGGGTGTCAAATTCGTCTGGAGTAA
>CFICBL_16570 DNA stabilization protein
ATGGCGAAAACGAAGGGTGATCTCGTTCTAAAGGCTTTACGAAAAGCCGGGCTGTATTCCAATGCCACGTTGACAGATGCTGACCCTCAGGCAATTGAAGATGCCATTAATGACCTCGAAG
ACATGATGGCAGCATGGCAGGCTAAAGGTATCGAGCTTGGATATCAGTTTGCTGATACAGAAAACGGCATCATGCCGTTACCTGACGATGATTCAGGTATCCCTGCATGGGCAAATGATGGC
GTCGCTTTGAAACTCGCTGTGCAAGTGTGCATGGATAACGTCATTCAGCCGTCAGACGCTCTCCTTACCGCTGCTGACAGTGCATATCAGACAATCTGTATCGCTTTAACCAAAATACCACCAC
TTGAGCGGCGAAATGACATGCCTCGCGGTAGTGGTAACAAAAGCGCGTTTACGTGGAATCGGTTTTACATCGAGAAAGATGATCCGAGTACGTGA
>CFICBL_16575 hypothetical protein
ATGGCACGAACAATGCTCTATAAGCCTGGCAACATGATCACCTGTGGTCAGTTTGCTGTCGATTACGTCATTGTTGATGACGAAGAAGTTAAATCTCACCTGAAAAAAGGTTGGGTAAAAAC
TCCTGAAGAAACCGCAACGAAGCAAAAAGTGGCTAAGGCGGAAGAAGATGGCGAAAACGAAGGGTGA
>CFICBL_16580 Coat protein
ATGGCTAACCAATTAGCAAAAGACCTTGAAATCATGTTCGAAAACTACGTTGAAGGCTTTGAGGCCGCCTGCGTAGTTTCCCGTAACGCTAAAAAATTCCGTCCCGGTGATACAGCAATGCA
GCGAGCAGGTGATGTTCTGTATCGTCCGCAGCATTACCACATGAACATTGAGGAAGGCCTCGACCTCAGCAACAAAACGCCAACAGCACTGGTTCAGCGCCTTGTTCCTTCTGTGTTCAAG
GAGCCGAAAAACATTCTGTACACTCTGGATGCGCGTGAAATGCGTGACCCGGAACATAAAACTGAAGCTGGTCGCGCCGCAGGTATGCGCCTTGCTGCACAGATTGACTCTGACCTGATTT
CCATGGTTACGCAGCGTGCTACTAACGTGATCACGATGGCTGATTCAACCACAGGCACACAGGGCCGTGATTTGTGGAACTGTGCGGCAGGTATTGATGCCACCATGACGGCGATTGGTGT
ACCGCAGGGTATCAACCGTCGCTCTTTCTGGAACCCATTCAACTACAAAGACCTTGCTGGCGAGCTTGGTCACCGTGCCTATGCTCAGGGTGCAACCCTGACAGCATACGAAAAAGCGCAG
ATCCCTCCGGTTGCGTCCTTCGATAGCTACAAGACCGATATTTCTGGTCGTGTTCCGAAGGGTACAGCAACTTCCATTACGCTGGCAGCAGCACCTGCGCACAAGGTTGAAGCGAAAGATGC
TAACGATATGCCAGTGGATAACCGACAGGGGACCATTACGGTATCTGCTGAAGGTTTGCAGGTTGGCGATGCGTTTACCATCGCAGGGGTGAATTCCGTACACCAGATCACCAAAGATACCA
CCGGGCAGCCGCAGGTATTCCGCGTTCTGGCAGTTAGCGGAACGACAGTAACTATCTCCCCGAAAATTCTGCCGCCTGACAACGCGGATGTCGCCAGCCGTCCATATGCAAACGTTGATGCT
AATGCGGCAAGTAGCGCAGCAATCACCATTCTCAACAAAAATGCCGCACCGGCTAACCTGTTCTGGGCTGATGGTTCTGTTGAACTGATGTACGGCAAACTGGCGTTCCCAACTGGTCAGG
GTCCACAGGTAATGACAGCAACCACCGAGCAGGGCGCTACGCTGATCATGTCTTACGCCTTCGACCACATCAAAGGCGTAACCACTGCGCGTTTCACCACTCTGTACGGTTGCTCTGTACTG
GTTCCTGAATATACGGGCATCGTTATTGCCGGGCAGTAA
>CFICBL_16585 Scaffolding protein
ATGGACCAGATGGCAGAAAACACACCAGAAGTTGAAATCGAAACCGATACATCAGAGCAGATTCCTGATGATGTCGAACTGGCTGAAGAAGTCGAAACAGAAGATGGCAGTGAGTCCTCC
GGCAATGATGCAGAGGAAGATACTGAAACTGATGACGACGAATCAGAACAGGAATTCTACTTTGGTGACGAAAAGCTGGATTCGCCAACCAGCGAAGATGGCGCAGAGCATGGACTGGT
AAAACACCTGCGCAAGACGATTAAAGAGAAAGACCGCGAGCTGAAAGAGTTGATGCGTCAGTCTCAGAAACCCGTCGAGCAGCAGCCGGTAATCACTCAACCACCGCGAATGCCAAAAC
TGGATGATGAGGACATCGGTTTCGATGAAGAAATCTATCAGCAACGTATGGCTAAGTGGGCAGAGGAAAACGGCAAATACCAGGAGCAAGTACGAGAGCGGAAACGAGAGGAAGAGGC
GCGTACCGCAACGCTTCAGCAGAAAGCAGCCAATTACATGCAGAGAGTAAAAGCACTGAAAGTGGCTGGCTACCAGGATGCAGAGCAGGCTGTACGCGAAGATGTTCCTGTTCATATTCA
GGACATGATCCTTCTTGAGTCAGAGAAGCCGGAAATCGTTGTTCTGGCACTCGGTCGCAACGCTGAACTGCGCAAGCAACTGGCAGAAGCTACCAACCCCGTAGCAATTGGTCGTCTGCTG
GAACGTATCGAATCGAAGGCCAGAATCATGCCAAAAGCAAAAACCACGGCAGCCACAACCCCGACAGTTAAGGGGAGCAACGGCGCAGTAATCAACAACCTCGACAAACTGAAAGCCAA
GGCGCTGGAAACTGGTGACTGGACGCCGTATTTCGCCGCTAAAAAGGCAAAAAAATAA
>CFICBL_16590 Portal protein
ATGGCAGAAAAAAAGATGACTGACTGGCATCGCAAGGTGCTGTGCAACTTTGATAATGCCTGGTCAGCAACGCAGGATATGCGTGAGCAGATTATTGAGGCTCAACGTTTCGTCCGGGTGT
CCGGCGCACAGTGGGAAGGCAGCACAAACGCTGGTTATTCATTTGATGAAGGCAGGTTTGAGCATTACCCGCGCTTTGAACTGAATAAGATTGCCCGTGAATGTGATCGCATCATTGGCGA
GTATCGACAGAATCGCATCAGCGTTAAATTCAGGCCGAAGGATGACAAGGCATCGGAAGCGTTGGCCGAAAAGATGAACGGCAAATTCCGCGCTGACTATCAGGAAACATCCGGTGGTGA
AGCGTGTGATAACGCATTTGATGATGCTGTAACGGGCGGATTCGGTTGTTTCCGCATGTGTGCAGATTACGAAGATGAAATGGATCCGAGTAACGAGCAGCGACGCATCAGCCTTCTTCCTG
TTTACGACCCAGCGACATGCGTCTTCTTCGATCAGGACAGCAAGCAATATGACCGCTCTGATGCTATGTGGGCTATGGAAATGTTTTCCATGACGCCTAAAGCGTTCGAAGCTGAATACCCTG
ATTCCATCGCTGCAAGTCTTTCTCGTGATGACACTGGCACTCAATATGACTGGTCAACTCCTGATGCTATCTATGTTGGTCGCTACTATGAAGTCCGCATAGAGAAGGTGAAGCTCACGGCGTG
GCGCAACCCTGTCAGCGGAGAAACGGCAATCTATGATGAAGAGCAAATCAAAGATATTGTCGACGAACTGACCGATGGTGCATTCGAACTGATTGGCGAGCGAACGGTGAAGAAGCGCC
GCGTTTATTGCGGTCTTCTGTCTGGCGCTGAATGGCTGGAAGAACCGAAGCGTATTCCGGGTGAACATATTCCTCTCATCCCGGTATATGGGCGTCGCTCATTTGTTGATAATCAGGAGCGAA
TCGAAGGCCACGCAGCAAAAGCGATGGATGCACAGCGTCTTGAGAACCTGATGGTTTCCATGATTGCAGATAACGCCACTCAGGCTGGCGGTGATGGCATTCCTATCGTGGATGTTGATTTC
ATTCCCGGCCCATTAATGAATCACTGGGCAGAGAGGAATAAGAAAAGACCTGCAGTTCTTCCTATGACCAGTAAGAAGGACAAAAACGGAACAGTCATTTCAGAAGCTCAGGTTGCTGGCT
GGACACCTCCGACACAAATGCCGCCAGCTCTTGCCGGGCTATTGCAGTACACCGGAACGGCTATTCAGCAAATTACAGGTGCGTCGCAGCTTGAGAACATGCCGAGCAACGTCGCCACCG
ATACCGTTGATAGCATTTTTAACCGGATGGATACGCAGTCCTATATCTACATGGACAACATGGCTAAATCCATGCGCCGCGCTGGCGTCGTGTGGCTTTCTATGGCGCGTGAGGTCTATGGCAG
TGATACTCCGATGCGTATCGTTAATGAGGACGGCAGCGATGACGTGGCGCTGATGACTGGTGAAGTGGTTGACCATCAGACAGGGCAGGTTATCGCGCTTAATGACCTTTCGCAGGGCAAC

TATGAAGTGACTGTCGATGTCGGTCAGTCGTTCGCTACTCGCCGTGATGCAACGGTTAAGTCGTTACTTTCCATGCTGGCACTTATCCCACCAGGAACGCCGAAGCACGACCTTGTATCGTCG
ATGATTCTCGACAATATGGACGGCGAAGGGATGGACGACCTTAAAGAATACAACCGCAATCAGTTGCTTCTGTCTGGAGTTATCAAGCCGAGAACGCCAGAAGAGCAGCAAATGGTTGAG
CAGGCGAAACAACAACAGGCGAGTCAGCCAGATCCGGCTATGGTTGCTGCGCAAGGTCAGCTTCTTGCTGGTCAGGCTGAATTGCAGAAAGCGCAGAACGAACAAGCAGCCATTCAGGT
TAAGGCATTCCAGGCACAGACGGATGCTCAGGTTGCTGCGGCAAATGTTGTGAAAATCCTCGCATCTGCCGATAGCCAGCAAAAATCTGATATCCGTGAGGCGCTGAAACTGCTCGGACAG
TTCCAGCAACAGCAAGGAGATAATGCCCGTGCTGATGCAGAGCTTGTCCTGAAAAGTCAGGCACAGGGCCATGCGCAGCGCATGGACATCGGCAGCATCCTGCAAAAATCAACTCAGCAA
CAACCACAGCAGTAA
>CFICBL_16595 PBSX family phage terminase large subunit
ATGACCTCGATTAATCCTATCTTTGAACCGTTCATTGAGGCGCATCGCTACAAAGTCGCCAAAGGCGGTCGAGGTAGCGGTAAGTCATGGGCAATTGCGAGACTGCTTGTTGAAGCGGCGC
GTCGGCAGCCGGTGCGTATTCTCTGCGCTCGCGAACTGCAAAACAGTATCAGCGATTCGGTAATCCGGTTGCTTGAAGATACCATCGAGCGTGAAGGGTATTCGGCTGAGTTTGAAATTCA
GCGTTCCATGATTCGTCATCTCGGAACGAATGCTGAATTCATGTTCTACGGCATCAAAAACAACCCGACGAAGATTAAATCGCTCGAAGGTATTGATATCTGCTGGGTGGAGGAAGCGGAAG
CGGTAACGAAGGAATCATGGGATATTCTGATACCAACCATCCGCAAGACGTTTTCCGAAATATGGGTGAGCTTCAACCCTAAGAACATCCTCGACGATACCTATCAGCGATTCGTTGTAAATCC
TCCCGATGATATTTGCCTGCTGACGGTGAACTACACCGACAACCCGCACTTTCCTGAAGTTCTCCGTCTGGAGATGGAAGAGTGTAAACGCAGAAACCCGACACTGTATCGTCACATCTGGC
TTGGTGAGCCAGTGAGCGCAAGTGATATGGCAATCATCAAACGTGAATGGCTTGAAGCTGCTACCGATGCGCACAAGAAACTCGGATGGAAAGCGAAAGGCGCGGTTGTTTCTGCTCATG
ACCCATCAGATACAGGGCCAGATGCTAAAGGTTACGCATCGCGTCACGGTTCGGTGGTTAAGCGCATTGCCGAAGGCCTGCTGATGGACATCAACGAGGGGGCTGACTGGGCTACTTCTCT
TGCGATTGAAGACGGCGCTGACCACTACTTGTGGGATGGTGATGGCGTCGGTGCGGGGCTACGCAGACAGACAACGGAAGTGTTCTCCGGCAAGAAAATCACCGCCACGATGTTCAAGG
GCAGCGAATCGCCATTCGATGAAGATGCACTGTATCAGGCCGGAGCATGGGCCGATGAAGTCGTACAGGGCGACAACGTTCGCACTATTGGCGATGTGTTCCGCAATAAGCGAGCACAATT
CTATTACGCGCTGGCTGACAGGCTGTATCTGACATATCGGGCGGTTGTTCACGGTGAGTATGCAGACCCAGACGACATGCTGAGTTTCGACAAAGAAGCGATAGGCGAGAAGATGCTGGA
GAAGTTGTTTGCAGAACTGACGCAGATTCAGCGCAAATTCAATAACAACGGGAAGCTGGAGCTAATGACTAAGGTCGAAATGAAGCAGAAGCTCGGTATTCCATCTCCTAACCTGGCTGAT
GCGTTGATGATGTGTATGCATTGTCCGGAGTCGGCTGCGCAACCCGACTATTCCAGTTACTCAATTCCTTGTGGTGTAGGTTGA
>CFICBL_16600 hypothetical protein
ATGGCGACTGAACCAAAAGCTGGTCGCCCCTCTGATTATATGCCGGAGGTGGCTGACGATATCTGCTCGTTGCTTTCTTCTGGCGAAAGTTTGCTGAAAGTATGTAAGCGTCCTGGTATGCCG
GATAAGTCCACTGTTTTCCGCTGGTTGGCAAAGCATGAGGATTTTCGCGACAAGTACGCGAAGGCAACTGAGGCACGAGCTGATTCTATTTTCGAAGAGATATTCGAAATTGCTGACACTG
CGATTCCAGATGCTGCTGAGGTGGCAAAGGCAAGACTTCGCGTTGATACCCGCAAATGGGCGCTGGCCCGAATGAATCCCCGTAAGTATGGCGACAAGGTAACTAACGAGCTTGTCGGCA
AAGACGGCGGCGCAATCCAGATTGAAACATCACCGATGAGCACTCTATTCGGAAAATGA
>CFICBL_16605 Transcriptional regulator
GTGGGTTACGTAATCAATTTGGGTAAGGAGAAGAAATTCCCAATTACTCAAGAGCTATACGAGCGGCTGGAAAGCGCCATTCATGATTACGATGGTGAAATCAGTTTATGCGAGGCGATTGG
CACACTCGAATTGCTGAAGCAGTCATTGATTGAAGGCGCGAAAGAGCCATCAACCTGA
>CFICBL_16610 Uncharacterized protein in gp15-gp3 intergenic region
ATGGCAGAGATTATTCCCATGACTGAAGAACAGAAATTCCAGTTAGAGATTTACAAACTGGTCATGAACCAGAACGCAGCCGCAGAAGAAGCATTTCAATTCATCGGCACGGACGAACTGA
AGCTTGAGCTATTCAAAATTCACTTTCAGTCAGGCGGCGCTAATTCTGATATCACGACCCGCACTATCGAAGCGGTGCGTAAATCGAAGGAAGCGTTAGACCTGTTCACCACCGGAGCATGA
>CFICBL_16615 KilA-N domain-containing protein
ATGAAGTATCCAACTGTCATTGTCAATGGTGTGTCCGTTCGTGTTGATGAGGACGGACGCTACAACTTAAACGATCTCCATGCAGCAGCAGTTGCAAACGGAGAGGCTACAGAGTCTCAAC
GCCCCAGTAATTTTCTGAGGAGTGCGCAGATTAAACGGTTCATTTCAGCACTAAAAGCCAAAGCTCAAAAAAGAGCTTTGGAAGAAATTCAACCACTTAAAGTAATAAAGGGTGGGGTTG
ATTCTGGTGTGTGGGGTGTTGAACTACTGGCAATCAGATATGCAGCATGGATTAAGCCGGAATTTGAAATCGAAGTTTATGAAGTTTTCAAAACGATTGTCCGTCTCGGCGTTGGTGCCATGT
CTCGCCTGAACAAAATTGACCACATCATCAACACGGAAACCAAAGCGATAAGCCAGTGCGCAAGCCAAATGGCTAAGTGGGGCGTTGGTGGGCGTAAAAGATTGCTTCACGTTGCTCGTG
AGCGAGTAGTGAATGAAGTGCAAATGTATTTGCCCGGAATGGTGTGA
>CFICBL_16620 Spanin, inner membrane subunit
ATGAGCAGAGTAACCGCGATTATCTCCGCTCTGGTTATCTGCATCATCGTCTGCCTGTCATGGGCTGTTAATCATTACCGTGATAATGCAATCGCCTACAAAGAGCAGCGCGATAAAAAAGTCA
GTGAACTGAAGCAGGCGACCGCCACCATTACTGACATGCAGCAGCGCCAGCGTGATGCTGATGCACTCGATGCTAAATACACGAAGGAGTTAGCTGATGCGAAAGCTGAAAATGATGCTCT
TCGGCGCAAGCTTGATAATGGTGGTCGGGTGCTCGTCAAAGGAAAATGCCCTGTGCCATCCTCAGCCGAAACCTCCAGCGCCTCCGGCATGGGCAATGATGCCACCGTCGAACTCTCTCCA
GTTGCTGGACGAAACGTTCTCGGTATCCGGGATGGAATCATCAGCGACCAAACAGTACTGAGAACGCTTCAGGAATACATCGTGACGCAATGCCTGAAATAA
>CFICBL_16625 Lysozyme RrrD
ATGGCAATGTCACCGGCACTACGAAATAGCGTAATAGCGGCGATAAGTGGCGGGGCTATTGCCATAGCATCTGTGTTAATCACTGGCCCCGGTGGTAACGATGGTCTGGAGGGTGTCAGATA
CAAACCATATAAGGACGTAGTTGGTGTGTTGACTGTGTGTTATGGCCACACCGGAAAAGACATCATGCTCGGTAAAACGTATACCGAAGCAGAATGCAAAGCCCTCCTGAATAAAGACCTTG
CCACGGTCGCCAGACAAATTAACCCGTACATCAAAGTCGATATACCGGAAACAACGCGCGGCGCTCTTTATTCGTTCGTCTATAACGTGGGCGCAGGCAATTTCAGAACATCGACGCTTCTTC
GCAAAATCAACCAGGGCGATATCAAGGGCGCATGTGACCAGCTACGTCGCTGGACATACGCTGGCGGTAAGCAATGGAAAGGGCTGATGACTCGCCGTGAGATTGAGCGTGAAGTCTGTT
TGTGGGGGCAGCAATGA
>CFICBL_16630 Holin
ATGTACCGTATGGACAAAATCAGAGAATGGTTCAGTTACAGCTTCGGAGGACTGACTGCGATGGGTGGCATTCTCTCCCTGAATGACTGGGCTGTAATCATTGGTATTCTTTGTACTGTCGGC
ACATTTGGCATCAACTGGTACTACAAACGCAAAGAGCGTGAGGACAGATTGAATGGCAATGTCACCGGCACTACGAAATAG
>CFICBL_16635 Antitermination protein Q
ATGAGACTCGAAAGCGTAGCTAAATTTCATTCGCCAAAAAGCCCGATGATGAGCGACTCACCACGGGCTACGGCTTCTGACTCTCTTTCCGGTACTGATGTGATGGCTGCTATGGGGATGGC
GCAATCACAAGCCGGATTCGGAATGGCTGCATTCTGCGGTAAGCATGAACTCAGCCAGAACGACAAACAAAAGGCTATCAACTATCTGATGCAATTTGCACACAAGGTATCGGGGAAATAC
CGTGGTGTGGCAAAGCTCGAAGGAAATACTAAGGCAAAGGTACTGCAAGTGCTCGCAACATTCGCTTATGCGGATTATTGCCGTAGTGCCGCGACGCCGGGTGCAAGATGCAGAGATTGC
CACGGTACAGGCCGTGCGGTTGATATAGCCAAAACAGAGCAGTGGGGGAGAGTTGTTGAGAAAGAGTGCGGAAGATGCAAAGGTGTCGGCTATTCAAGAATGCCAGCAAGCGCCGCATA
TCGCGCTGTAACGATGCTAATCCCAAACCTTACCCAACCCACCTGGTCACGCACTGTTAAGCCGCTGTATGACGCTTTGGTGGTGCAATGCCACAAGGAAGAGTCAATCGCAGACAATATTTT
GAATGCGGTCACGCGTTAA
>CFICBL_16640 Protein ninH
ATGAACGCCACAATTCAAACGATACCAGAACTGCTTATACAGACACGAGGCAATCAGACCGAAGTGGCGAGGATGCTTTCCTGCGCAAGAGGAACAGTGCTCAAGTACAACCGAGACAGC
AAAGGCGAGCGTCACGTAATAGTTAACGGCGTCCTGATGGTCAAACAGGGCAAGAGGGGAAGACGATGA
>CFICBL_16645 Crossover junction endodeoxyribonuclease rusA
ATGAACGAATATCAGTTTGTGCTTCCATACCCGCCGTCGGTGAATACCTACTGGCGAAGACGTGGAAGCCAATATTACATCAGCGATAAAGGCCAGAAATACCGAAAAGACGTTCAGCAAGT
CATCCTCCAACTTAAGTTAGACATTTTCACCAAATCACGACTTCGCATCAAAGTCATCGCAGACGTTCCAGACTCCCGCCGCCGCGACCTCGACAACATCCTGAAAGGTTTACTCGACTCCCT
TATCCACGCCGGATTTGCGGAAGACGACGAGCAATTCGATGACATTCGCGTAATTCGTGGTGTGAAAGTACCAGGCGGACGGCTTGGAATAAAAATCACCGAACTGGAGAACGTATGA
>CFICBL_16650 DUF1364 domain-containing protein
ATGGCTAACTTACGCAAAGAAGCGCGCGGCAGAGAATGCCAGGTACGTATTTATGGCGTATGTAATGGCAATCCTGAAACTACAGTTCTGGCACATTACCGGATGGCTGGAATTTGCGGAAC
GGGGATGAAGCCTGACGACCTGATCGGTGCATGGGCTTGTAGCGCGTGTCACGATGAAATCGATCGACGAACCCATATTCTCGACAACAAAGACGCCAGACTTTACCACCTCGAAGGCGTG
ATCAGGACGCAGGCGATACTGCTGAAGGAGGGGAAGATTAAGCCATGA
>CFICBL_16655 Site-specific DNA-methyltransferase (adenine-specific)

ATGAGTGATTCGTTTAGTAGCTCTGATTATTTGTATTTTGTTTTAGTGCCTGTGGCAGAAGTATTCCGCTCTCGATTTCCTGAAGGCTCAGCACCATTTAATGCCATTCGCACTTACTCAAAATG
CAGAGTGAAATTTACAGGTAAGCGGCTGGAAAGAGAGTGGCAGGAATTCTGCAAAAAACATGACCTAAAAAATGACCCTGAACTGGAGTATTAA
>CFICBL_16660 Protein ninF
ATGCTTAGCCCATCCCAATCCATTCAATACCAGAAAGAAAGCGTCGAGCGGGCTTTAACGTGCGCTAACTGCGGTCAGAAGCTGCATGTGCTGGAAGTTCATGTATGTGAAGCGTGCTGCGC
AGAACTGATGAGCGATCCGAATAGCTCAATGTACGAGGAAGAAGACGATGAGTGA
>CFICBL_16665 DUF2591 domain-containing protein
ATGGATTATTCACAGTTAAGTGATTTTGAAATTAACAAGCGAGTGGCAATTTGCTGTGGATTTGCTCCCGAAGATTGCGAAATCGCAAAGTTGGGAACATCAATCGTTGGTGTTGAGTGGGA
TGACGAAACTGGTTATGCAATAAAAACGGTTGATTACTGTAAAAGCCCATCAGACGCAGAGCCGATTATCGTAGAGAACAGAATTGGCATTATTCCAGCGCCAGAAAATGGATTATGGAAGG
CAGCGCATAGAAAAGTTGGCAGTGATAGTACCCCATATCATATGACTCAAGATGAAAACCCACTCCGCGCTGCCATGATTGTCTTTCTCATGATGCAGGACGCCAATAATGCTTAG
>CFICBL_16670 Recombination protein NinB
ATGAAGCAAACAATCTTCCTCCGTGGTAAACAACAACAGCAAGCCGCAATCAACGCCATCCTCGCAACACCACTCGATAAAGACAAGCCAGTTACCATCCGCATTACTGACTACAAGCGCAA
CCTTGACCAGAACGCAAAATTTCACGCGATGCTGGCGGATATCGCTCGTCAGGTTCAATGGTGCGGCAAATGGTTAAAACCGGAACAATGGAAGGTTTTGTTAATTAGCGGTCATGCAGTG
GCAACAAAACAGGAAGCTGATGTTTTGCCCGGCCTTGAAGGTGAATACGTCAACATTCGCGAAAGCAGCGCACAGATGAGCGTGAAGCGCATGGCAAGCCTGATTGAGTACACGACAGC
ATGGGCTATTGGTCAGGGTGTCAGATTTACCGACAGGAGGTACGAATGA
>CFICBL_16675 hypothetical protein
GTGAGCAAGTACGAAAAATTAGATCAAAACATTCTTTCAATGCTGAGTGAAAGACCAACACCTGTTTTTGATATCTGGCTTAAATGGCGGAGCAATGGAATGTATATCGAAACCATCGATCGT
CGTATGCAATACCTGAGAAAGAAAGGGCTTGTTGCAAATGTGCGTGGGAAGGGTTGGGTGAAAATTAACCTGTCATAA
>CFICBL_16680 Prophage protein
GTGGCTGACTGGCAAATTCCAATCATCATTCTTGCCGGAGCTTCGCTGGTTGCTGGCTTTATCCTGCTGAAGAAGCATAAAGACCGTGATCAAAAAGTCGAAGTTCTCTATGGGTATCCAGC
GAACAGCACAACATGGCTGACCATTTACCACTACCGAAAATCAGGCCGCTGGGTATTCGAATGGGATGATCTGTTCGCTGAAAAGCGACCAAAGTCATGGGGAGACATCAGCGAATGCATG
ATGTTTGAAGAAAGAAAATCCGGCGCAACCCGAGAAGAGTTTAACGAAGCGTGGGCGCGATTAAGTGAGAGAGGGTATTTGTGA
>CFICBL_16685 Transposase
ATGCTTAACCGAATAATGCCAGAAATGCTTTTGAATCCACGGTTCATTGCTGTTTTGAACCGATGTATCGACGAAGAAGAATTAATTATTCAATTCGAAAGGCTGTCAGGAGTAAGCCGACCA
CCAAAGAGGCAGCATCCAGTAGAGCTGATGGTTGACAAAGCGACCGGATTTTATGATGAGCAGTGGAAACTGTTTTTTGAAGCATTTATCCCGTTCGTCTATGAGTTTATATGGCTCACATGG
GAAGACCGTGACAATGAGGAGTGCTGGCAATGA
>CFICBL_16690 Replication protein P
ATGACACCGAGTGAACTCAGCGACCTGCTTTGGGCGCAGGTTGACAGGGTGGCTCCGCACCTGTTGCCAAACGGCAAGAAAGACGGGCATGAATGGGTTGCTGGTAACGTCAACGGCG
ACAAGGGGAACAGTCTGAAGGTTAACCTTAGCGGAAAGAAAAAATGGGCTGATTTCGCTGAGGGAGACGGCGGTGACATGCTTGATTTGTGGATGGCGTGTCGTGGAATTAACCTGCATC
AGGCCATGCAGGAAGCGAAGGCATTTCTCGGCATCAGGGAGGACGATCACCATTTCGACGCCAGACGTGAGAAGAGATTCTCCAGACCTGACCGCAAGAAAATAGCCCGCTACGTTACCA
GAACAGAATCACATCTTGAGTACCTGCAATCGCGTGGCATATCGCCTGAAGTCGCGAAGCGATACGAGGTTGTCAGCGGAAAGGTCTGGAATGGCGAACGTGAACTGAGTGCCCTGGTGT
TTCCGTACAAACGCGATGGCGAGCTGCTGCAGGTCAAGCGAATCAGTACTGAACGTCCGGACGGGAAGAAAGTCATCATGGCAGAAGGTGACTGTGAACCTTGCCTGTTCGGGTGGCAG
GCTCTCGATGCTGGCGTGAGGGCGGTTGTACTTTGCGAAGGCGAAATTGATTGCATGAGCTATGCGCAATACGGAATACCGGCGCTATCTGTCCCTTTCGGTGGCGGGAAAGGCGCCAAGC
AACAGTGGATTGAGTTCGAATACCACAACCTCGACAGGTTTGAAGAAATATTCATTTCGATGGACGTTGACGATGTCGGGCGTGAAGCAGCAAGGGAAATCGCAAGCCGACTTGGTGAGC
ATCGCTGCCGTCTGGTTACACTGCCACACAAAGATATCAACGAATGCCTGATGAACGGCGTCACCGAGGATGAAATCTGGCAGTACATCGGGACAGCGTCATATTTCGACCCCGAAGAACTT
TACAGCGCCCGTGAGTTTTATCAGGACACCATCAATGCTTTCTACGGCAAGCAGCAGTATCTGTTTAACCCACCGTGGGAAACGCTGGCTTACAACTTCCAGTTCCGTGAGGCGGAGTTAAC
TCTTGTCAATGGCGTGAACGGTCACGGGAAAACGGAGGTTGTCGGGCATATGGCACTTGAGGCCATGAGGCAGGGGGTAAAAACATGCGTCGCATCACTTGAACTGAAGCCCGGGGTTT
TGCTTAAACGCCTGACCAGGCAGTCAACATGCTGCAAAACGCCACCAGTACTGGAAATCGAATCAGCATTTAAGTTTTACGATGACCGGCTCTGGTTATTTGGCCTGACAGGTACAGCCAAG
GCTGAACGCCTGATTGAAATTTTCACATACGCCAGACGGCGATACGGCATCCAGTTATTCATCATCGACAGCCTCATGAAGTGCGGCATTGGCGATGACGATTACAACGGGCAAAAGGCGTT
TGTTGACGCGCTGTGCGACTTCAAGAATAAAACCAATTCCCACATCATTCTCGTTACTCACTCAAGGAAAGGAGACAGCGAGGAGAAACCTACCGGAAAGATGGACGTAAAAGGCTCAGG
AGCGATTACAGACCTGACAGATAACCTGTTTATCATCTGGCGCAATAAAGCTCGCGAGAGAGCGTTACAGCGCGTTCATGCTGGCGAGCAGATTAACGATAAAGACCAGCAGCTTCTTGCA
GCACCCGCATCTGTTTTGATGCTTGAGAAGCAGCGAAACGGGGAAGGGTGGGAAGGCGGCGTGCCGTTATTTCTTGACGAGCAGTCTCACCAGTTCCTGCAAATGGAAGGTGCATCACCA
TACAACTACATCGCCAACATGCCGAAGTCGGAGTATGACGAAGTGTGGCGGCAGGAAAACGTTACGGAGTACTGA
>CFICBL_16695 HTH-48 domain-containing protein
ATGAAATCAAAAATCAAATCGCTACTGGTCGCTGGTTATAACCACGGCTGGTTAAGTATTTCGTTTGTCGATTTCTGGTTTAAAAATCTCAATATGAGGGAATTATGA
>CFICBL_16700 18 protein
ATGGGCGTCGTTAAGTTAGCAGACTACAGACCGTTAGAGCCGGTCGTGGAGCGTAATGTGGCAGATCTCGATGATGGTTACGCCAGACTATCAAATATGCTGCTTGAAGCTTATTCAGGCGC
AGATCTGACCAAGCGACATTTTAAAGTGCTGCTTGCCATTCTGCGTAAAACCTATGGGTGGAATAAACCAATGGACAGAATCACAGATTCTCAACTTAGCGAGATTACAAAGTTACCCGTCAA
ACGGTGCAATGAAGCCAAGTTAGAACTCGTCAGAATGAATATTATCAAGCAGCAAGGCGGAATGTTTGGACCAAATAAAAACATATCAGAATGGCGTACCCCTCAAAATGAGGGAAAATCC
CCTAAAACGAGGGATAAAACATCCCTCAAATTGAGGGAGTGCTATCCCTCAAAACAGGGGAACACAAAAGACACTATTCAAAAGAAAGAAATACAAGATAAAAACATTATGTCCGAAAGTG
TTCGGACGAAGTGTGAAAAATCATCTGGCCGTCACGAAGAAACCGACAAGGCATTCGAGGAAATATTCTGGTGTGCTGGAATGCGGAAAGCCGGGAAGAAAAACGCAGCTTCGGCATTC
AGAACACAGTTCAGGGAGTGGCGTAAAACCACCAGGGGGACGGCAAGCGAGTTTGCCACAATGCTGGCAGAAGATATCGCGTGCAGGAACGGTAAGCAGTTCGGATTCGACAGGTTGT
TACCATCGAGCTACCTGAACGGCCAGCGCTGGAACGACGAAAAGCCAGAAACCATTCAACCACAATCCAAACCATCATCCGCAATCACCGTATCGAAAACTGGTTACGTGTTTTTCGACAGG
TGA
>CFICBL_16705 hypothetical protein
ATGCCAAAACGTCGTAAGAAATACCAGGAAAAAGAAGAGATTCGACACCCTGATTCACCTGAGGGATTAGTGGTAGCAGCAGCAAATAACAGGGCGTTCGCAGAGCGCCTTGTTGGTGTT
TACAGACTAGCCAAAGCAGGAGTGAAACATGGGCGTCGTTAA
>CFICBL_16710 Transcriptional regulator
ATGGAACTCACAAGCACTCGCAAGAAAGCCAACGCAATTACCAGCAGCATCCTGAACCGGATAGCTATTCGTGGTCAGCGGAAAGTCGCTGATGCGTTAGGCATTAACGAATCTCAAATTTC
ACGATGGAAAGGCGATTTCATTCCGAAGATGGGGATGTTATTGGCGGTTCTGGAGTGGGGTGTCGAGGATGAGGAGTTGGCAGAACTGGCAAAGAAAGTTGCGCATCTGCTGACAAAAG
AAAAGCCCCAAGACTGCGGGAACAGTTTTGAGGCCTGA
>CFICBL_16715 HTH cro/C1-type domain-containing protein
ATGAATAATATTCGCAATTTTCGCGAGCGCTTCGGTTTAACACAGGAAGATCTTGCGAAAGTACTCGGTTGTACGCGTGGTGCAGTTTGTCATTACGAGACAGGCAGAAGGGGAATGGATAT
CAATCTTTGTCGCGCTTTTATCAATGCGTTCAAAGAATACGGTTATGAACTAACCATAGACGATCTTTTTCCACCAAAGGCTGCGTAA
>CFICBL_16720 Repressor
ATGAACTGGTATGACATAGCGAAGCAAAGGATTGATCAGCTTGGATTGAGTCAGGATAAAGTTGCTGAACACCTTGGTGTAACCAAAGGTGCTGTTAGTCATTGGCTTAACGGGAGAAGGA
ACCCATCAATACAAGAAATTGGAGCTATATTTCAATATCTTGGCGTTACAGACGCGAGGTTCAACGCTGACGGAACCTTTAGCGTTGGCGAGTCAACAGAACAAAAGCCTGTTAAACCTCAA
TTTGAATACCCATTCTTCTCTCATGTTCAGGCTGGAATGTTTACACCAGAATTTCGAACCTTCACTCAACTAGATGCTGAGGGATGGGTAAGTACAACCAAAAAGGCCAGTGAGGCAGCTTTC
TGGCTTGAAGTTGAAGGCCACTCAATGACGGCTCCAGCGGGATCACGACCAAGTTTTCCTGAAGGAATGCTGATTCTTGTAGACCCAGAAGATCCTGTAGACCCAGGCGATTTTTGCATTG
CAAGGTTATGTGGTGATGAGTTCACTTTTAAGAAGCTCATCAAAGACAGCGGACAAGTATTCCTACAACCGCTAAACCCTCAGTTCCCAATAATGCCGTGTAACGAACAATGCAGGGTTGTA
GGTAAGGTTGTAGCCAGCCAGTGGCCTGATGAGATATTTGGGTGA

>CFICBL_16725 MG2 domain-containing protein
ATGAGATATTTGGGTGATGATGGACTGAAGGGATTTTTGGGTGATATACAAATGATTAAAGAACGTATTTCTTATGTTATCCCGATCGCGATAGATGGCAGCAAATCAGGAACCCCAGTCCTTA
TCTATGAGATGGCAAAAGACTCACATGAAGTGGATTTGTCATTCGGTATTTTTTTTATCGGTCTTAGAGCAGCCAAGAAATACTCCGTTGGCATCGAGGTGTTCAATGACAATGAGACACCAA
TTCCAATTGATACAAAGAAATTTTCCAACCATATGTTTTTCACCGTTGCAGAAGCTGGTGATGGAGAAACTGTTGTGTCGGCATCCATGAAAACAACGTTTCCTAAGGTTGAAATTATTAACC
CTGGAATATTTGAAGTTAGAGCGTCACTGGTTAATCCAGACACCAAAGAAATCATTGATGTGAAAAGCTCTTTTTTCGATATTAAGCGTGCTGGAGTGGTTCGCAATGAGTTCCAATAA
>CFICBL_16730 hypothetical protein
ATGAGTTCCAATAACACCGTTACTCAGCTTCGTCCAAATCAAGACATCTCTCGCCAAATTGGACACCACTTCACTGATGATGCATACTCACGGCATGGTGGTGGAAATGGCGGGGGTAACATG
CTTGAGGCTAGAGTAGCGAAGCTTGAAGCTGACGTTGAAAACATCAAAACAAATCTCGCAGAAGCAAGAGCTGACATCCGTGAACTAAATAAAAATTACGCTTCAATAAAAACGGATGTTT
CTGTTTTGCTGCAAAAAACCGTTGAAATAGACACTGCTTTATCTAAAAAGCCAACAGCAGATTCATTGAAAGTCTGGTTTTTAACCATTCTTCTCTTTTCTGTCGCAATGCCAGTGATTACTTTT
TTGATTAATCTCTATATGAAAAAACCGTAA
>CFICBL_16735 HEAT repeat domain-containing protein
GTGAAAAGTGAGGAAGAGTTCTTTGCGGAGCTTCACCCGCAGGTAGTTGAGGTTCTCGGTACTGCGCTGATGCAGGTACTGGTAGAGCAGCGCGAACCTTCGCGTGAAGCTTTGATAGAA
ATGATTCAGGTACTGTGGCAGGAAGAGGATGTGGACTTGGCTGTAGAACTGGCTATTGATGTTCTGACACTGCCGAAAGAGTAG
>CFICBL_16740 Antitermination protein
ATGACACGCAGAACAGCTTTCAATGGCTCAGCGGCAGGCCGTCGCCGAGAACGTCGCGCAGCGCTTCAGAATGCGGTAACGGCAAGCTCAGAAGTAATGCACCGCCCCACCCTTAGCCGT
GCGCAGATTCAGGCCAAAGGAAAACATGAAACGCCAAAACGTATTGAAGACGCAAAATCACTTCAGTTCATGGCGAAAGATGCATTCTGGCAACTGGAAGAATACAGACGCAATCTGGAG
CGGGCAGCCATTGTGTACGCAAATGAGTTTGGTCATAAGCCACCAGAAACCGGTGTATGTTTGCCAGACGTAGCGCTTTACGCAGCAGGTCATCGTAAATGTAGACAAGTTACCGCTAGATA
A
>CFICBL_16745 Phage tail protein
ATGACCAAAGAAATTGTGACATTCAAGGGATTTAACAAAGACCTAAAGTGCCGTGACTTTCAGTTTGAAATTGGCAAGACCTTCCATCACGATGGAAAAGTGGAGGCTTGCGGTTCTGGAT
TTCACGCCTGTGAATGTCCTTTCGATGTTTTCAGTTATTATCCGCCGGCAGAAAGCCGCTATGCGGAAACAATATCTTTTGGTATTACAGACAGTGAAGAAGGAGGTGACACTAAAATAGCCA
GTTCCAGTATCACAATTAAGGATGAGTTAACGCTTCCACAGTTCATTCAGCGTGGTATTGAGTGGATTTGGAGCAAGATTGATAAATCGCTGGAACAGCAGATCATGACTGGCGACTGGTCA
GCAGCAACCAACACTGGCGACTGGTCAGCAGCAACCAACACTGGCAACCGGTCAGCAGCAACCAACACTGGCGACTGGTCAGCAGCAACCAACACTGGCGACTGGTCAGCAGCAACCA
ACACTGGCGACTGGTCAGCAGCAACCAACACTGGCAACCGGTCAGCAGCAACCAACACTGGCAACCGGTCAGCAGCAACCAACACTGGCGACTGGTCAGCAGCAACCAACACTGGCAA
CCGGTCAGCAGCAACCAACACTGGCGACTGGTCAGCAGCAACCAACACTGGCGACTGGTCAGCAGCAACCAACACTGGCGACTGGTCAGCAGCAACCAACACTGGCAACCGGTCAGCA
GCAACCAACACTGGCAACCGGTCAGCAGCAACCAACACTGGCGACTGGTCAGCAGCAACCAACACTGGCAACCGGTCAGCAGCAACCAACACTGGCGACTGGTCAGCAGCAACCAACA
CTGGCGACTGGTCAGCAGCGGAAGTGTCTGGATCGCAATCCGTAGCGGCATCACTCGGAATAGAAGGAAAAGCCAGGGCATCTGAAGGCGGAGCTATTGTGCTTTGCTATCGAGATGAAG
ATGGCGAGTTAATTCATATTCGAGCAAGCAAGGTTGGCGAGAACGGTATTATGCCGAATACATGGTATCAACTGGATAAAGATGGTGAGTTTGTAGAGTGTGAGTGA
>CFICBL_16750 hypothetical protein
ATGACATTTGCTATCGCGGGCGGTGCCGTCATGGGTATCGCACACCTTAATGAATCACTTTTAGAGCGTATCACCAGAAAATTACGGGCCGGATGGAAACGTCTGGTCGATATCCTGAATCAA
CCAGGAGTGCCGTGTAATGGATAA
>CFICBL_16755 Host cell division inhibitory peptide Kil
ATGGATAAATCACTTATGGCTATTCAGTCTAAATTCGCAATTGCTGTTTATCTTGGTGACAAAATAATGTATCGCGAAGCTGTAGAAGCCTTTCGCGAATGGAGGTTGAAATGA
>CFICBL_16760 Recombinase
ATGGATTTGAATAAATTCGACGACCCATTCAGTCCTGAAGATATCGAATGGCGAATACAGCAAAGCGGTAAAACACGCGATGGCAAAGTGTGGGCTATGGTGCTGGCTTATGTCACGAACA
GGGCAATCATGAAACGCCTTGACGATGTTTGCGGCAAAGCAGGATGGCGCAATGAATACCGCGATATTCCCAACAACGGCGGCGTTGAATGCGGAATATCAATCAAGATTGATTCCGAATG
GGTCACCAAATGGGATGCTGCTGAAAACACGCAGGTAGAAGCCGTCAAAGGTGGTCGTTCCGGTGCAATGAAGCGCGCTGCCGTTCAGTGGGGAATCGGTCGGTATCTGTATAACCTTGA
GGAAGGTTTCGCACAAACATCACTCGATAAAAAGCAGGGATGGCACAGGGCAAAACTGAAGGATGGAACAGGATTTTACTGGCTCCCTCCATCGCTGCCGGACTGGGCCATGCCAGCATC
AGGCAATCAACCATCACCAGAAAATACCAACCAGAAATCTCCATCGGTTGACTGCGAACAAATCCTGAAAGACTTCAGCGATTATGCAGCAACAGAAACTGACAAGAAAAAGCTAATTGAG
AGATATCAGCATGACTGGCAATTATTGGCTGGTCACGATGATGCGCAGACAAAATGCGTTCAGGTAATGAATATCAGAATAAATGAGCTTAAACAGGTGGCTTAA
>CFICBL_16765 Anti-RecBCD protein 2
ATGCCTGCACCTCTATATGGTGCGGATGACCCGCGCAACTGCTCCGGTAGCTCCAAGTCGGAGGTGCTGGAAAATATCAAAAACAATCTCGACGCGTTTCTTGCTCTGCCACCGGAAACAAA
AGCAGAACGGAAGTACCGACGCGATATACAACTCGCAGAAAAACAGGAAAAAGACCGAATAAACGAAACAGCAATCCGACCATTCCGAAAAGCCACTTACACCAAATTCATTGAAATAGA
CCCGCGCCTTAAAAATTACCGTTCGCGTTACGGCGCTATCAGCAATAACTGA
>CFICBL_16770 hypothetical protein
ATGAGAGGTTTGTCCTACGACCAAGGAATCCTTCCATCGGAAATGATTATTCGACACCGCTTCAAGCCCATCAACGATATTCCACGCGAAGAAATGCTGGCGAGAAAGAGTTTTCCATCAGT
GAATCAAAACAAATATCTGAATGCGATGTGGCGGAGTGGGAAGAAATGA
>CFICBL_16775 Eae-like protein
ATGAAACAAATGACACTAATTGAGATGGATGGATTTCTGAAAGGTAAATGCATCCCACGAGATTTAAAGGTTAACGAAACAAACGCTGAATATCTGGTGCGTAAATTTGCTGAAGCGGAGGC
CAAGATTTCCGCGCTGGCCGAAGACCACCAGAGAGCGATTGAGTCAATTAAGCAGGCTGATTCGGCTGTTAAGTTGGCACACGAGAAGTTTTCGGCGCTGGCTTCGGAGAATGCGGCACT
GAAAAAATCAGAGGTCGAATTCAACGAATATTGTCGTCGCGAGTGCGAGGACGTTGGAGATACGTGGGTGGACGATTTCACTGAGACCCCAGCCACCGACGCGTTTCTGGCTGAGGTGCG
GGCGCAGGCTCACAAGGAAGGCGCTTACTTTGTTGCTAACCGAATGCTGGCCGCATGGGATGCAGGATTTATCGACGACACAGCAAAGAACGCTGCGGACATCGCACGAATGATACTGAC
CTCCACAGAATTTATGGCTGATGCGCCGGAAGGCGATTTTGTTCGCTCATTCGCCGATGGCGTTCTCGAAGGTATCGCCGCCCAGCTTCGCAAAGGAGTGCAGTCATGA
>CFICBL_16780 NinF family protein
ATGATTACGGGAACCTCAAATTACGACGAAGTGCCGACGATACCCTGCAAAATCTGCGGCGGTTATTTCAAAGCCGATGATCCAGAAAGTCACAAATGCGAGGAGTCAGCCCAATGA
>CFICBL_16785 Ead/Ea22-like family protein
ATGAGCAACATCGACAAACAGGCAGTAACAGCAAAAACAAAAGAACTGGCATCCTTCATGGTTGAGCGATTCAGTATGAATCCTGTCAGCTGCAAATTGCTGAATGAGGCATGGGAAAAA
GAATTCCCTGACGAAGTGGCTATCGCTGAGCGAATGCTGGCGCTACTGGATGAGCTGGAGCATTACAAATCACGCGAAGAGCGAGTTACAAAGCTGGTTCTGGACAACTCGACAAGCTGG
GATGCTCTCTACAAGAAGCTGGAGGCCGCAGAGAAGCGTATTGCTGAACAGCGTGAGTATTACGAGGGCGTTATTGCTGATGGAAGTAAGCGCATAGCAGAACTCGAACACAGCGAGACG
CAGCTTATCAATGAGCGTGATAGTGCTGAATCTGCACTGGCCGATATGTACCAGGCCGCAACAGGAGAGCGTCCAGAATGGAGCAATATGTTTGGTTTCGCTGACGCCGTTGATGTGGTGG
AAGAACGACTGGCGACGCTGGAGGCCAACCAAAGCCAAACCACGCCAACGGGAATTCAGCTCATCACAGAAGCCATAGGTGCGCACGGCTATATCGTTGGCTGCCTGTTGCAAGGTCGCC
CTGATTTGGCGCTGGAAGAATCGAGAAAGTGGGTATCCGCTTTCGGTCAGGCGGCGGAAATAGTTAGTGCACAAGACGCCGATGACATCAAGGTTAAGGGGGAGTGA
>CFICBL_16790 Prophage protein
ATGACCACTATTACCAGAGAACAGCAAAAACAGATTTTAATTGATACAGCGAACCACGTAATCAGTCGTGATAACACGTCACCGTATAGCGAAAACCTGCGTGAACTGGCGCGTATCGCGCT
GGCATCGCTCGAAGCGGAGAAAGGTGCCGACCCCGTTGTGTTCACCGACGAACGAAATCTTCATCATATTGCCAGGGGTCGAGAAACCTCTTTGATTTGGGGTAAACAAAACCAGGAGGT
GGGGGATATCCCGCTCTATCGTCACGCCCAGCCAGTGCCGGTAGTGCCTGATGAAATGGCGACATCTGATGACATGAATCTTTATCAAAAGAGCTTTGCGCAAGGCTATAACGCCTGCCGCA
ATGCCATGCTCAACGGAGGTAAATCGTGA
>CFICBL_16795 DUF551 domain-containing protein
GTGAAAGAGAATCAAATCCGGGAGCTTGTAAACGAGCTGCATGATATTGCTATTGAGTATCACGGCACACAACAGTTACGTGAACGAATTGCGCGTACAGTTCGCGCCGCCCTGCATCATGA
CTTAGAAAAACTAAACCAACCTGTAAGCCAAACTTACGAGTTGCCAGAATTAATCGAAGGCATGGAGGTGTCCATTGATGTCAGCACTTGTGATGCTGATGCCGGGAATCGCTATTTCGGTA

CTGTCACCGAGGTATCAGAACTGGACACAGCAAAGAATGGCTACATTCTTCTGGTTCAGGACGCTGAACCAAATTTCGATGTGAATGGCAACTCTCCGGTAATTCCGGATGGCTGGATAAGC
TGTAGTGAGCGAATGCCGGATAATGATGAATCTAAACCCATCGCAATTTTTACCGGAAAATGTCTGGGTCAGGGGATGTTCGTTGCTACATACGACGATGATGGGTTCTTTGACTATTGGGAG
GGTATGGAAATTATCGGTGTAACCCACTGGATGCCGCTACCGGAGCCACCGCAGGAGGTGAACCGTGGCTAA
>CFICBL_16800 ASCH domain-containing protein
GTGGCTAACCTGCAACTTGCCGTCAAAGGTGAATACTTCGATGCCATGATTCGCGGGGAGAAAACGGAAGAGTATCGCTTGTGTAATGACTACTGGAATAAGCGAATTATGTTCCGCGAGTA
TGACCGACTGATTATCACAAAGGGATATCCAAAGCGCGACGATTCCAGTCGCAGAATTGATGTTCCGTATGGCGGATATGAAATCAAGACAATCACACATCCCCACTTCGGCGATAAACTGGT
GAAGGTATTCGCGATAAAGGTGAATATCGGCAATGAATAA
>CFICBL_16805 DUF3800 domain-containing protein
ATGTCTGATTTAGCAATGAAGGTATTGAAGTGGCAAACGAAAGGCCACGTTGGCATAAGTAGCGCAACTATGGCTTCTATTGCTCTTGGGCTGGAAAAGAGCTTCTACCACGGACGGTTTG
ACGCACCAAGCGATCCTGCCGATTTGCGAAGATGCATGATGCTCGTAGATGAAATACCTGAAATTAAAGATAGCTTTCCGCTCATAGCGAAAAAGGTAAAGCGGTTTTCTCCGATTTTACGTG
AGTGGGATTCACTTATTGCTCTGCTTAAGCTTGAGCTTAAGAGGCCAGATAAGCGAGCACCAAAAACATATAAATGGATAAAAGAGCTTCTTTCTGACCAGGAGTAA
>CFICBL_16810 hypothetical protein
ATGGAATCACACAGTCTCACACTCGATGAGGCCTGTGCATTTCTCAAGATATCCCTTCCCTCCAAGTTCGATTCCCAAACCGGAGATAAAACCTATGCGCGAATTACGCGATGA
>CFICBL_16815 AlpA family phage regulatory protein
ATGCGCGAATTACGCGATGACTCGCTTGTTGACTTGAAGTTCATGATGGAGGATTCTGGAATGGGCAAGACCTTCATTTACTCAGAAATTAAGAAAGGCCGGTTGCCTGTCCCTCACAAAAT
CGGTAGCGCATCCAGATGGGTTTATGCCGACTACCAAAACTGGAAACGCAGCCACTTCTCCCCTCTCCAAAATGCATCATGA

Prophage 12

>PNPANE_22705 Phage protein
ATGAAACCAATTAAACGCCTGTACCTTTCCACTGATCCGGTCCATCTGATTGACTGCAATATCGTGCTGGAGCTGAACGCGTGCGGTCGGGGGTTTATTACTGCGGGGACAGAGACAGATTA
CACTGGCAAAATGGTGCGTATCGATGTTGGCTATGATGGTCTGGTCCTGCGCTGGTTTACCGGGTATGTCGAACGGTCACAGCCTGCTGATAATGGAACATGCCGGTTGTTCGTGCGTGAGC
TTGTCGGCATCTTTGATAAATTGTGGCCGTGTTCTTTCCAGCATCCAACGCTTCGCCAGATTACTGACTGGATAAGTGAGCAAAGCGGGCTGACCGTCACAACGCCGGTCGGCGCTGCTTAT
GCAGATAAACCGATCCCCCACTTTACGCATAGCGGCACGGGCTATCAGCTTCTTGCCAGTCTGGGCCGCGCATTTACAGTGACGGATTATCTTTGGTATCAGCTGCCGGACGGGGATGTCTT
CGTCGGCGCTGCGGAGCATAGTCTTTTTGCGGGTAAACCAGTAGAGATCCCGCACGAATTTAGCCAGGCATCGGCAGGCGGCAATTCAATGGTTGTGCCGATGATTCAGAGCCTGCGCCCT
GGTGCTGAGGTGAACGGCCAGCGGTTGAACCAGGTCCGGCTAAATAACGACGATATGGCAATCACCTGGCAGCCCCGCAACAAAGCCAACGGCCAGCCGTTGCAAAAATCACCGATTCA
GCGGCAGATTGAAAACGCATTCCCGGAGCTGGCAAGCGGCCTGCATCTTCCAAAGTTCGCCAGGGTGGAAGCACCAAGCGAGGATGTTTCACGGGGAAATATTGCCGATCCATTCCGCCC
GCGCTATGCCGTGGATCTCCAGCTGCTTGACGAAGACGGCAAGCCTGCCGCAAATACGCCGGTTTATTCTGCCGTTCCACTACCTGTACCAATGGCGGGCAGTGAGTCGGGAATGTTTCAG
TTTCCGCCCCCTGGCACGCTGGTTGAAGTAGGATTCGCGGAGGGGCGGCAGGATAAGCCATTTGTGCGCCAGATTATGGCGGAGGGTCATAACCTGCCAGCAGTTAAACCCGGCGAGCA
GTTGCAGCAACAGCGCGATGGTGTATCTCAGCGCGTGACGGTTGCCGGAGACTGGGAACGCCAGACAGACCAGACAATCCGCGAAAACTCCATGATTCGCGAAGTCACCACCGACGAAG
AGATCCGCAAAGTGGTTGTCCGTGAAACTACGGTCCAGGCAACGGATAAAACAACCGTGCTTGGCACGGCCACACTTCTGGCCGGGGCGGTCGTTCATATCAGCGAGGGGGATTACAGCG
TCGGCACATCCGGCAACCTGACAGTAACCTGCAGCAAGGACAATTCCGTCAGTGTTGGCCGGAACGTGAAACGGGACGTCGCGGGCAATGTTACAGACGACGTTAAAGGGAATGTCACG
TCAAACGTCAGCGGCGCACTGACTGAAAAAATCAGCGGCATCCGCCGAAGCGTGGCCCAGGCGCAACAGCTGATTGCCCCGGTGGTAAAACTGGGAAGCGAAGAGATTAACGTCCTGAC
ACTACTCACCGACACCCTGGACGTGGTGAGGGAACTGGCAGAAGACTGCCGCGTCACATACTCACCCCAATACGGGGGCCAGCGGGCAGGCTGCGCAGTTCAGCGCAACGGCCACTAA
>PNPANE_22710 Nucleoid DNA-binding protein
ATGAGCCAGACCGCATTACTCGCGCTTGATGGTGAAGGGATCGCCATGCAGAACATGCTGGTTTCACCTTCCATGCAGTTTCAGGAAAAGGACCAGTCGGGCCAGACATCGAGCACGGCC
AATGCTGAACAGGGTATCAAGGCCAAAGAGTTGCGTGTGTCGGGTCTGGTGACATTCGACGACGAAGCCGTTTTACAGCGGCTTTTCCAGTTGGCATCCGCAACCGAAGCCAGCGGAGCA
CTGAAAAAGTACCGCGTCGCCAATGCGACGGCAACGGCTATTAATCTTCGTGAAGCCACGTTCACCGGTCAGATTGATGCCGTACCGCAGGAGGATCGTCTTGCCTGGCAGGTAAGTTTCA
CCCTGCGTGAAAAAGGCAGCGTCCCGGAAAAACGACAGGCCAGAAAAGGTAACGCGACGGCCAGCACGAAGCAAACGGGGTCAAAGGGCGCGGGTTCGGCTGCCGGTGCTGATGAG
CCAGCCGACAAAATGAGCTGGTTTGAAGAAAAGGTCTTAAAGCCGGTCAACGATGCGCTGGGGTAA
>PNPANE_22715 Phage protein
ATGTGGCGTGAATCGGTTGTAAAGATTGCTGACGATATGGTGGCGCTGGCCTGTTCCATTGTGCCAGCGCATCCTTGGGTTTACGGGCTGGGACAGAACACGGATTCAGGCGGTTATCTCA
GTCCGGCCAATGCGATGGGATACCTTGCTAAAAAGCTGTTATTCGCTGGCGGTCGCGGTGATGTTATCGTCATGATGGTGGCGGAGAATACCCATGATACTTTTATGCAGGGGCTGAATAAAC
TGTCCACCGTATTTCCGGCCCCCGCATTTACGCAGGTAAGCCGCATGGCAGCCGCCGCCGCAGAACTCAGCACGGTAAAAATGCAGTTGCCGGTTAAAGACGATGTATTGCCTGCCAGTGC
GCCGTTATCAGTCTCAACCAACCGGCTGGCGCTGAATGCCCAGCGTGTTGCCGCCGCGCAGCTGGCCGCCGCAGTCAGTACCACCACAACAGATCTAAAAAATCAGATTACGGGATTTATTC
AGGAACGGGCCGGTTTGCTGTCCTCACTCAGCCAGGGACTGGAAGACTTGAAAGCCGCCAGTGCGAATATTTTTTCATTCAGTTACAGCGGGAGTTATGCCGTTGCTGCCGCTGAATTGTT
GAAAGGCATCCCGCAAACAACGGCAGTGCATACCGCCGCGATGATGTTTATCGGGGATTCGTTATCTGACTTAGGGAAGATGCTACATGAGCCAGACCGCATTACTCGCGCTTGA
>PNPANE_22720 Tail fiber assembly -like protein from lambdoid prophage Fels-1
ATGACTTTTAAAATGAGCAGCAAAGCGCAGACAATTAAAATTTTCAATCTGCGTTCAGATACAAACGAATTTATTGGGGCAGGTGATGCATATATTCCGGCAAATACAGGATTGCCCGCGTAC
AGTACAGATATTGCACCGCCAGCAGCGAAGGATGGATTCGTCGCTGTATTTAATTCTGAGTCAGAAAAATGGTCACTTGTTGAAGACCATCGAGGGAAAGTTGTCTACAACATCAAAACGG
GGGAAGCCATCACAATTAACCAGCTAGGCGCACTACCTGATGATGTTGTTTCCATTGCGCCCGAAGGCCATTTTGTGAAATGGGACGGGAAAAAATGGGTACACGACACGGAGGCGGAAA
AAGTGGCGCAGGTTACTCAGGCGACGCAGCAAAAAGAAGGTCTTCTGACGCTGGCTGCATCAAAGATTGGACCGCTACAGGATGCTGTTGATTTGGGTATTGCGACAGATTCGGAAGCGG
CGCTTTTGCTGGAGTGGAGAAAACACCGCGTTCTGATCAATCGTATTAATCCTGCCGATGCGCCAGATATTAACTGGCCGGAGGTTCCGGGCGATGTGGCGTGA
>PNPANE_22725 Phage tail protein
ATGTCACAGGCTGTTATTACAAAAGCATTTACAGAGTGGAAAGCCCAGCAGGCAATTAATAATCAGCCCGTCACGCTGGATGAATTTATTTTCGCGTATATTCCGGGACTGGATGCTGATAAA
CCGATTGATAATACCGAGACAATGCCCGCAGCGGATAAAATTGTTGATCGCCTGCCGGTAAGTAAAACCGGCGTTGTGAATGAAAATTCTGTCGTTTATTCCGTCACGCTGGGGGCGGATGT
GGGCGATTATAATTTCAACTGGATCGGGCTGGCAAATAAAGCCACCGGCACACTGGCGATGATTATTCACGCCCCGACTCAGCGCAAAATTAAAAATGCGAACGGCCAGCAGGGAAACGT
GTTGGTTCGTTCCATGCTGATGGAATACAGCGGAGCCAGGGAAGCAACAGAAATTACCACCCCGGCAGAGACATGGCAGATTGATTTCACTGCCCGCCTTTCGGCAATGGATGAACGCCA
GCGCCGCGAAAATATCGATCTGTATGGTGCCGCGGCATTCTTTGATTCGGGTTATCTGGTCGCAAAGTCCGGTAATCAGTTCTTTGTCACAAAAGGGGTGGGATACGTCGCCGGGTTACGCA
CTGAATTGCCCGCAGACCTGAACATCAACGCATCTGCGAAGCCGACAAAAGTCTGGCTTGATGTCAGCTGGACCGGGACGTTAACGAGCGAATGGGCAGTACAGAGCAAAATCACCGTTG
CCGCAGATCTTGCCGATTATGTGCTGGGCGGTGTACAGCATTATGTCTTTGCGGTGGCGAGTATTGATGCTGCCGGAAATATCACAGACCTGCGCCCGAAAGGCACGCTTAACGACCAGGC
GGCCAGCAATGCGCTGAGTGAGCATGAAAAATCGCGCAATCACCCTGATGCATCAACCGATGAAAAAGGATTTGTGCAGTTAAGCAGTGCAATAGACAGCGATTCCGAAAAGCTGGCCGC
CACGCCAAAGGCAGTAAAAGCGGCTAATGACAACGCAGATAAGCGACTGGTTAAAGACCAGAACGGCGATGACATTCCGGACAAAAGTTTATTTGTGCGTAATATCGGAGCACTTCCTGCC
AACGGTACGGCTGTTGCAGCGAACAGACTGGCATCACGCGGTGCGCTTCCGGCACTGACTGGTGCGACAAGAGGCAGCGATAGCGGCCTGATAATGGGCGAGGTTTACAACAATGGCTAT
CCAACACAATACGGGAATATTTTACGTCTGACCGGAACCGGTGACGGGGAGATATTGATCGGATGGAGCGGGGTTAATGGTGCTCCTGCGCCTGCATATATTCGCAGCCACAGAGATAACGC
CGAGGCTGAGTGGTCCGAATGGGCAATGCTCTACACCACACTAAACCCGCCACCGGATTCGCATCCAGTAGGGGCGGCGATTGCATGGCCGTCTGATGCTACTCCAGCCGGTTACGCCCTG
ATGCAGGGGCAAGCATTTGATAAATCTGCTTACCCGTTACTGGCTATAGCGTATCCGTCCGGTGTCATCCCTGACATGCGAGGCTGGACAATCAAAGGTAAGCCCGCCAGCGGGCGGGCTGT
ACTGTCTCAGGAAATGGACGGTAACAAATCGCACAGCCACGGCGCGCGGGCGCTGGATACCGATCTGGGAACGAAAGGCACGTCGTCATTTGATTACGGTACGAAATCGACCAATACCAC
GGGCAACCATACTCACCAGTTCGGCGGTTATATCAATTCATACTGGGGAGATTCCAATCACACCTCATTTCAGCCTGGAGGTGGTGCGTGGACACAGGCCGCTGGCGACCATGCGCATACAG

TTTATATCGGAGGACACGAGCACACGATGTATATAGGTCCACACGGACACGTCGTTATTGTGGACGCAGACGGTAATGCGGAAACCACAGTGAAGAATATCGCGTTTAACTATATTGTGAGG
CTGGCATAA
>PNPANE_22730 Phage tail protein
ATGACTGAGTTTTCAAAGTTGCTTGCCGGTCTGAAATTGCCGTCGTGGCTGAACAAAGGCGACCCCACCAGGCTGCTGCGGGCCTGCGTGAAGTTCTGGTCGCAGGTGTACGGGTGGATC
ACCTGGCCGTTAAAGCAGTTTGACCCGCTGGTCTGCCCCGAACCGCTGTTAAACCTGATTGCCTGGGAACGCGACATCGATCGGTTTAAGGGGGAACCGCTCGACATCTTTCGCAAACGG
GTGAATTACGCATTTATCAATGCGCAGCAGGCTGGAGAGGTGGCGGGCTTTATTGCCATTTTTGAGCGACTGGGGATTGGTTACGTTGAATTACTGGAACGACAGGACGGACTCGACTGG
GACGTGATCGTCGTTCGGGTGACAAACAGCCAGATTGCGGAAAATGGCGATCTCCTGCTGGAAATCATCCGCAAATACGGGCGCACATGCCGCCGTTACCAGTTTGAAGTGATCACCGCCC
TGCCGCTGAATATCAATATTGGCTGGTATCAGGGGGAATATATTTGCTGGCCTGCCACCCTGGGTGATGTGAATAACCAGCCAGAAGCAACATACAGCGCAAGTTTGAAGTAG
>PNPANE_22735 Putative baseplate protein gp29
ATGACTGAGAAACCCGACGTTGATTTTGAAAAAGTGCTGAATGACAGCGGGATGCCCGCGACAGAGGCCGAAATTACGGCGGCATTTAAAGCCACCGTGCAGGCGGAAGGGTTCGTCAC
AAATACGTCGAGAATGTCACCTTTCTGGCGGCTGATTTCGAAAATTGTCACCACGCCGGTGTTATGGCTGCGGGCGGCGCTGATCGATGTGGTTCTGCGCAATATGTTTGTCGCGACGGCCA
CCGGTCCCATGCTGCGCCTGCTGGCCTGGGCGGTCCATATTGTGCCTAAACCGGCCAGCGCTGCCGCTGGCGTGCTGCGATTCTACAAGCTGAACGCGGCGGATGTGGTCGTCGTGCCTGC
CGGAACCCTGGTGCAAACCGAGCGTATTAACGGCGTGGTTTACGTGCTGGCGGTGAATGAAGACGTGACGCTGCCAGCCGGGGTTGAAAGCGGGCTGGTTCCCGTCACGGCGACCGGC
ACCGGCAGCGGCTATAACCTTGCGCCCGGCTATTACCGGATCTTACCCGTTGCGGTGGCTGGGATCGCCAGTGCGGTCAATGAGGACGAATGGCTGATTACGCCAGGGGCTAACGAGGAA
AGCGACGACGAGCTGCGCGACCGCACCCGCAACCAGTTTAATCTGGTAGGCAATTACCATTCTGACGCTATCTACCGCAGCATGATTGCCAGCGTGCTGGGCCTGAGCGTTGATCGCATTTA
CTTTTTGCACGATGCCCCGCGTGGGCCGGGTACGGCAAATGCTTACCTGTTACTGGACAGCGGCGAAATATCACAGCCCTTTATTGATGCGGTTAACGACTATGTGAATACCCAGGGCCACC
ACGGACACGGTGATGATCTGCAGTGTTATGCCATGCCGGAAACCAGCCACACCCTGGCGGTTACGGTCTACGTCAAAAGCGTGGAAAACATGGAGGCGGAAGACCTGAGCGCGTTAAAA
ACCGGTATTACCGACCTGATTCGTTGCGCGTTTCGCGAGAACGCCAATTACGACGTTAAAAAGACGCAGCCCTATTCGCGCTATTCCTTTTCGAATCTGGGCCGCGAGATCCACAAGGCTTTT
CCGGTTGTCGATTCACTGCATTTTTCACTGACGGATATTGTCAGCGAACTGTCAGTCCCGCGCCTGTCAGGGTTAACGGTGGAGATTGAAAATGACTGA
>PNPANE_22740 DUF2590 domain-containing protein
ATGAGTACCGAACCGTTATACATCGACCTTTTGATCACTGACGGCGATTTCACGCTGGACAGCGGCAACGAGCCGCGCCGTTGCGATAACCGCGACAGCATCACCCAGGACATTATTCACAG
CATTCTGGAAAGCGGTATCACCACCCGTCTGATTGGTGAACGTAGCCCGACAATGCGCGGTGACGTGCTGACGCAGCTGTCCTTACTGGTAGAAAGCGACGAACGTCTGGTCCCCGGCACC
ATAGTGATCACCGAAGAAACCCTTTCGCGATTGTATGTCACGGCGGAAACCTACGATTTTGGCCCTGTCGGTACAGAGGTTAACTATGACTGA
>PNPANE_22745 PhageMin-Tail domain-containing protein
ATGAGCCACCTGGATTTTACCCTGAGCCTGATCGATAAGCTGACGCGGCCATTAAAAACGGCCCAGTCTTCGCTGTCCGGCTTTGCTGAAAAATCGCAGGCGTCTTTTACAAAAATCGGGAT
CGGTGCGGCGGCTGTCTGGGGCGTGGCACAGTCCATCGCGGGCGTGGTGGGTCCGGCGTATGAGATGAACGCCGCGCACTTGCAGAAGTGGGTTCCAAAGGCGTGGCAGAGGATGCGCTG
AAACGTCTGTCCGGCGAAGCCATGCGATTCAGTATGCGTTACGGCAAAGGGGCCGTTGATGTGGTCCGGTCAAGTTACGCGATGAAAGGCGCAATGGCGGGCCTGTCCGATATGGACCTG
CCCCGCGTCACCATCGCGGCCAATACCCTGGCGGCAGGCGTCAAGGCCAGCGGCGAAGAGGCGGGCGAATACATCGGCGCAATGGCGTCACGTTTCAACGCGGAGTTGTCCAGTCTGGG
CCATGTGCGTTTTGCCGAAGAACTGGCAGGAAAAAACGGCGTACATGGTGCAAAACTTCGGCGTGAAAATGCAGACCATGCAGGAGCTTATCGAGGGGGACGAAAAGCGCCGGTGCTGACT
TTGGCGTCAGCCTGGATGAACAGTTCGCCGTACTGGGTACGCTTTCGCGCACGTTGGGTACTGAGGCCAGCGGGATCTACGAGCAGTTTTTACGCAGTGCCCCGGCTGCCGCTGAAAAGC
TGGGTATGAGCTTTGTCGATGCCACCGGCAAAATGCTGCCGATGGGCGACATTCTGCAAAAACTCCAGAGCAAATACGGGCAGAGCATTGAAGGGAACGTCAAGGCACAACAGGCGCTG
GACGCCGCGTTCGGTGGTGGTGCTGACGTTATCAAAAAGCTGTACGGCCAGCAGGATAAATTAAACCGCAGCATCACCGAGCTGGGCAGAAATGACGGGATGAAACGCGCCCAGGAAAT
GGCCGAACGAATGGCCGAGCCGTGGGAGCGTATCAAAGCGACATTCTTTGCCATTCGCGTGGCGATTGGTAACACGCTGATCCCTATCCTGTCGCCGCTGATGAACCGTATTGCCGACGTG
GGGACTAAATTTGCCCGCTGGCTGGATATGTTCCCGAATATTGCCCGCTGGCTGGGTTACATCACCCTGGGCGTGCTGTCCTTCGGGCTGGCCGGGGCGGCGGTCAATATCGTGATGGGGG
TCTTTGGCTTCACCATGACGGGGCTGGCCGCAATCGCTAAGGTGCTGGGTGGCGCATGGAAACCCCTGTTATGGACGCTCAACCTGTTGCGTCCGTCCCTGCTGACAACGCGCATTGGCCT
GGCTGCATTGTGGATCCAGTCAAAATTGCTGGCGCTGTGGACGGGTGTCTGCCGCATCGCGCTGGCTGCATGGAATATCGCGTTAAAGACCGGGGCCATTGCCATGCGGGTTTACGGTGTG
GCGACCATGTTTGCCGGAGCGGCAATGCAATTCCTGATGAGTCCGATCACCCTGATTATTGCCGGGCTGGCGCTTCTGGCCGTGGGGGTCTGGTACGTCATCACTCACTGGAAAGAACTGG
CGGCAGCGATCATGAATACGGCGGCTTTTGCCTGGGTGATGTCAGTCGCGGAACAGGTAGGCCAGGTGTTTGCGCGGGTCTGGCAGTCCATCACTGACGGCTGGGCCGTGGTGGTTGATT
TCTTTGCCGGTCTTTCCCCGCTTGCCACCTTTGAAGGGTTCGCACAGATCATCGGCGGGGTATTCAGCAAACTTTTTGACGTCCTCAAAAGCACCTTTGCGTCTACCTATAACTGGATTGTTG
AGAAGTTAAACAAGATCCCCGGCGTCAATATCGACCTGAAAACCGTTTCACCACCGGCAGCGGCTGCCGTTCCGGCAAACGCGGTCATTCCAGACAGTGCTGCAGGTTCGTCGAAGCTGA
ACAGCCCGTCCGTGCTGACGGGGAACCGAATTAATGCAGACATCCCACGCGGCGGCCTGATGAGTCAGGTTAAAACCGACCGCAAAACCACCGTGGATAACCGTAAATCGTGGGGCGATA
CCTACATCAACGCCCCCAATGGAATCACCCCGGCCCAGCTGGCTGAATGGCAGGAGCTTAACGCAGGATGA
>PNPANE_22750 Phage protein
ATGAGTAAACCGAAAAAAATCGCCCTGACCGTGGCGGGCGTAAATCTGAGCTTTGAGCCGAATAAAACCGCGTTTAACAACCTGCTTAACGAAATGACCATGACCAATAAGGTTGCCCCTAT
GGTGACTTATCTGGGCCGAATTGTTGATGCCGAGTGTAAAGAGTCGCTCAACAAGCTGATGGAAGATTATCCGGGTTGTGAAATGCAGATCGTCGAGAAGGTTAACGAGATTTACTCCCCA
AAACTTGAGATCGAAGTAAAAAACTGA
>PNPANE_22755 Phage spanin Rz
ATGCGCAATTTGCTGGGTCTTTTGCTGATTCTGGTCGCTGCAATGTCAGCGGGCTGGCAGGCGCATGACTGGCACGACGCAAAGCTGCAACTCGCTGCCAGTGAAGCGGCAGAACAAACA
CGCCAGATTGTCGTTGAAGTGACGCAACAGTCTGGCGAAGCGCTGGAAGCAAAACTCGCGGGGCTAAAAGCCAATGAAAGGCACACGGAACGGGTTATTCGTACAGAAATCATTAAGCC
GGTTTTTAGCAACGTTTGCGCTTCTGATGATTACGTCCGGTTGTTCAACGAAAGTGCAGATCAAGCCGAACGAGAATTATCAGGAAAACCAGCTGACACTTTGCCCGGTCACGCTGCCACG
TCTGGCCGGACCGACCGGAAATGA
>PNPANE_22760 Peptidase-M15-4 domain-containing protein
ATGAAACTGAGTGAAAAACAGCAACTTTTCACGGTGATGATCGCCAGCCTGATTCATTTTGCCGAAGAAAAGGGCTATCGCCTGACGTTTGGCGAAGCGTACCGCACGCCGGAACAGGCC
GCGCTTAACGCAAAAAAAGGGAGCGGCATTGCTAACAGCCTGCATACCCAGCGCCTGGCGGTGGATTTTAACCTGTTTATTGACGGCGAATACCAGACCGACAGCGCCGCATATCGCCCAC
TGGGCGAATACTGGGAATCTATCGGCGGATCGTGGGGTGGCCGTTTCAGTAAGCCGGACGGGAACCATTTCAGTCTTGAGCATAACGGGGTGCGCTGA
>PNPANE_22765 Holin
ATGATGAACGGAGAAACGTCACTGCTTGAAAAACTGTTGCTTATCGGGGCCGTGATCGGTCTGGGGCAACTGATGGTCAGTAATGAGCGAATCACAACCCGCCTGCTGGTCGGGCGGATG
ATTCTGGGATCTGCGGTCGCACCACTGGCCGCAATCCCGCTGCTGAAATTCCCCGATATGCCGGAACTGGTCGTCATTGGGCTGGCCTGCGCCCTGGGCATTCTGGGAAGTGCGTTTATTGA
GGCGGGGTTAAAGCGCTGCCTGGACATGTATATCAAGCGATGGGGGAGCAAGCGCAATGAAACTGAGTGA
>PNPANE_22770 DUF2597 domain-containing protein
ATGACTGAACGTATCAGCGGCGGATCGTTCGATGTGAACTACGACAGCATCATGATTCACGTAGAAAACGCCACCGTCACCATTACGGACAACAGCGCCGTTGCGCAGTCGCGTGGCATCC
CTAACGGCCACACGAAAGGGTCCGTTTCGGCGGATGTGGAAGTCGAAGTCGATTCCCAGAACTTTAAAAAGTTTACCGCTGTGGCCCGCGCCGCAGGTTCCTGGCGGGCCATTCCGGCAA
AGGACTTTTTGTTCTATGCCAACGCCGGGGACGACGAAGAAAAAATCGAGGTGTTTGGCTGTGTTCCGACGCTGTCCGACATCGTCAACATCAACCCCAACGAGGCCAGCAAAACCACGA
AGAAAATTAAATTCATGGTGACAAGCCCGGACTTTGTCGCGATTGACGGCGTGCCGTACCTGTCAGCCCGTGATACTCGCGATCTGAAAGGCTGA
>PNPANE_22775 Putative tail sheath protein
ATGACCTGGCCGAATGTCAACGTCAGTCAGAAAAACCGCTTCAACGGCACAACGAACGACGTCGAGCGCGTCATCCTCTTTGTGGGTTACGGCGACACTAACATCGGGAAAACCCAGTCG
CTGAATACCGGCAGCGATCTGGATAAAGCCCTGGGAGACAAAGACAGCCCGTTAAAAAATATGGTGGCCGCAGCGGCCAATAACGCCGGTCAGAACTGGTTTGCTTACGTGCATGTGCTG
GCAGAGCCAGACAAGGACGCCGAGGGCTACAAACCAGATGAAGACTGGATGAACGCGGTCAAACAGGCCCAGAGCGTGGCATCCGTGGAAGGGGTTGTCCTGACATTTGATACCGCCG
ACGCAGCCACCATTAACCGCGCAACGGAAATGCGCGTCACCTTACAGGCCAGTTTTGGGCGTTTTATCTGGTTTGCCCTTGCTGTGGGCGGCCGGAAAAGGACGAAGCGTGGAGCGAC
TATGTGACGCGCCTGGCAAAACTCCAGGACGGTATTGCATCGCCTGGGGTGCAACTGGTCCCGCGTCTGTGGGGCAACGAACCCGGCGTCCTGGTTGGTCGCTTGTGTAACCGTTCGGTG

ACGGTGGCAGACAGCCCCGCCCGCGTTGCAACCGGTGCAGTCACCGCGCTGGGGCGCGATGGCCTGCCGGTCGACGGGACGGGGGCCGAGATTGATCTGGCCGTGTTGCAGTCTTTGC
AGGCGAACCGCTACAGCGTGCCGATGTGGTATCACGATTATGACGGCATCTACTGGGCTGACGGTCGCACTCTGGACGTTGAAGGCGGTGATTATCAGGTGATTGAAAACGTGCGCGTGGT
TGATAAAGCCTCCCGCCGTGTCCGTCTGCGTGCAATCCCCAAAATTGCCGATCGTTCGCTGAACAGCACACCGGGCAGCATCGCCGCGCATGAAACCTATTTCGGCAAGCCGCTGCGTGAA
ATGGCGATTTCAACCCAGATCAACGGCATCGAATTTCCGGGCGAAGTGAAGCCACCAAAGGACGGTGACATCACCATCACCTGGACCAGTAGCGAAGCGGTACAGATTTACCTTGTGGTTC
GACCATATGAGAGCGCGAAAGAAATCAGCGTCAGCATCGAACTGGACACCTCACTGGAGAGCTAA
>PNPANE_22780 Phage virion morphogenesis protein
GTGATCCGTGGGGAGCTGAACCAACAACAGCTAAAGCAGATGCGGGAAACGCTGGCAAAAGCTGACCTTCCCCCGCGTAAGCGCCAGCGCCTTTTATGGCGTATTGCAAAGCTGGGCATT
GTCACAGCGGCAAAACGTCACCAGCGCCAGCAGGCGGCCCCGGACGGTACGCCGTGGGAGCCGCGCAAGCGTGGCAAAGGGAAGATGTTAAAAGGGCTGCCCAAACTGCTGGCCGTG
CGTGAAATGCCGGAGATTCAGGGGGTAAGAATTTACCTCAAGGGCGGGAACTACCGGAACGGGACGAAGCCCATTGCGGCGGGTCTGGTCGGCGCGGTCCAGCAGGACGGCGCAAGG
ATCCAGATGAAAGCCAGTAACGCCCCGCGCAAGCCGCAGGCTGACAAGCCAGCGCTACCGAGACAGGCCAAGCGCCTGCGGGCGCTGGGCTACAAAACCCGCAAGGGCAAGCGCTGG
GTTAAGCCGTCCAGCAAGCAAATCATGGAAACCATGAGCATGGCCCAGGCGGGATTACTGATTCGAAAACTGAAAGGCACACCCTCAAAACGCACATGGACCATTGATATTCCAGGGCGC
GTTTTTCTGGGGGTGAGCAACGACGAATTTAACCAAATTATTGCGCGGCAAATGCAGGCAATCGGCTTCGGCTGGGACGTCAACGCGCAGCAAATCAGGGGGTAA
>PNPANE_22785 Phage tail protein
ATGAGCCAGCTTGAAAGTCTGACGGCGTTTATTACGGCAAATCTGCCGCCTGATGCCATGCAGATGTTTTCCAGTTCAATGGAGGATTGCGAGCTGGTACGCAACGCCAAAGCACTGGGGA
ACAACCAGCGCCGGATCGGAGTGCTGACTTACACCGCCCGTTTGTCGTGGGATGACTTCCCCTACCGCAAATATTCGCCGGGGCTGATTTATGCCCTGGTGCTGGCCTGGGTGGATGAGTTC
GCCAACGAGCTGCGCGACGAGCTGAAGTTAGCCGATCCCACCGTGGACCCGGAGTTCGACGACGAAGGGTCATGCATTCTGGATGTGGTTGTCCCGCTGGTTGATCCGCTGGTCCTGCGT
GAAGTGGAAAAAGGGCCGATCCCCTTCAAGGGTAAGAAATGGGACATCGTAAATCCCGAAATCTGGGAGGCGTCGCAACTGGAATTTATTGTCCAGCGTGGTGACGCGTCGTGA
>PNPANE_22790 Head completion/stabilization protein
ATGAGTGGCCCAAGTTTCAGTATCAGCGGTAAGCCGGTGATGGTGACGCCAACAGCGATCACCAACGGCGTGACGTTCTGGCCCGATCTCGATCTGGCCGAGTTTCAGAAGGTGCGCACG
CTGCCCGCTGATCTGCCGCCAGAAACCGCAGGCGTGGCCCTGCTGGCTGCTATTGCGGAGGTAAACGACGCGCTGGCCGACGTGGTGACGTACTGGAACGCGAAAGCCTGCGAGCGGGC
CGCAGATGTACCGGGTGCAAAGATGGGCGACGAAAGCCAGTTAACAGCCCAGTACAAAAAAGCGGTCTACGCCCGCGCAAAGGCCGATTTACTGGGGGAGTTCGCCACCATCGGGCGGC
GTGAATCGCATCCGGGGCAGGAAAGCCAGGACACCCGCGCCAGCCTGCTGGCCGAGGCGGCCAACGTGATGCGAAACATGCTGCGACAACCACGCGTTGGGGTGCATCTGATATGA
>PNPANE_22795 Terminase
ATGGCTATGACCCCGTGTCAGCGACACCGAGCACGCGTGAAAGCCGCAAAGGCGCTAGATAAGTGCGAAGCCCTGACGGCATCGCCGGTCAGCTTTCACATTCAAATGCTGGAGCTGGAA
AAGGATGTTGAACGGCTTCGTAGTCTGACGCGTGCAGAGCGCATGGACATGAAGCGCGATGTCCTTTTGCCGCGCTGGATGCCGACCGTTGAAGCATATCTTGCCGGTGATGCCCGCTTTG
CCAATCCGGCCCTGGTTTACTGCGTGATCTGGCTGTTCGATACGGGGGAAATGGGCAAGGCGCTGGACTGGGCTGACGTGGCTATCAGTGAGAACCAGGCCACGCCGGAAAACTTCAAA
AGCAACCTGCCCGCCTTTGTGGCCGATACGGTGCTGGGGTGGGCGATCATGCAGGCGGAAGCCGGTCACAGCATCGAACCCTATTTCAGCCGCACGTTTGAAAACATCCGCGAAAAGTGG
CGTTTGCACGAAGACATTAACGCGAAGTGGTTCAAGTTCGCCGGTCTTTACCTGCTGCGCGACGAGAAGGGCCAGCCGCGTGCCACTGCCGTGGATGATGTGAATACGCTGGAACAGGCC
GACGCCCTGCTGGCCCAGGCGGCGGCGTACAACAAAAACGCCGGAGTTAAGACCATGCGCGAAAAAATCCGCGCCCGGATTAACGGCCTGACCCAGCTTTAA
>PNPANE_22800 Phage major capsid protein, P2 family
ATGCAATTAACCCCAAAAGCAGAGCAGATGCTGCGTAAGTTTACTGCTGGCCTGGCAAAAGCTAACGGCCAGGTGGACACGTCGCGCTACTTCTCGCTGACCAATCCGAAAGAAACCCAG
CTGCGCAATGCTCTGCTGCAACAATCTGAGTTCCTGCGCCTGCTGCCTAACGTGCTGGATGTGGATCAGATCACCGGCCAGGTGGTCAGTACTGGTAAGCCGGGTATTTATACCGGGCGCAA
AAAGGATGGTCGTTTCTCTCGCCCCCTGGGTGTTACCGGGAATGAATACAAGCTTGTAGAAACGGATTCCGGTTCGTATCTGCCTTACTCCCTGTTGGTTGTCTGGGCTAACGCGGGCAGTG
AAGAGGAATTCTTCCAGCGTATTCAGGCATTCAGCAACGAGTCATTCGCGCTGGATATGCTGCGCGTGGCGTTTAACGGTACAAGTGTTGCAGACGACACCGACCCGGAAACCAACCCGAA
CGGCGAAGACGTTAACATCGGCTGGCACCAAATCGTTAAGGCCCGTTCGTCTGAACAGATTATTTCTGATGCCGTCACTATCGGCGCGTCGGGTGCTGACTTTATTGGTCTGGATGCGGCAG
TCACTGACCTGGTACACACCTGCATTTATGAGCCATTCCGCAATGACCCGCGCCTGGTTGTGCTGGCTTCCGCTGACCTTATCGGCAACGACGCCACCACGATGATGAATAAGATTGATCGCC
CGACTGAGAAAGTCGCCGCGCAGCTTATTGGCCGCCAGATTGCTGGCCGTACCGTGTACACCCCGCCGTTTATGCCGGAGGGCCGCCTTATCGTCACCACGCTGGACAACCTGCATATCTAC
ACCCAGCAGGGGACACGTAAGCGTAAAGCGGAATGGAACGACGACCGCAAGCGCTTCGAGAATAACTATCTGCGCATGGAAGGTTACGGCGTCGAGCATGATGAGCTGTACGCGGCATT
CGACAAAATCACCCTTGCGACCGGCGAAGTTGCACCAGGAGGGGGCGCGTAA
>PNPANE_22805 Phage capsid protein
ATGCCGCAATCTCATTACCGCACGGATTGGCTATGTATTGCCACATCTGGAAAGGCTGTGGACGGTCGCACCATTGAACCGCAATGGTTGATTGATGCGGCAGAAACCTACACCCGCAAAAC
CTACACGGCCATGATTTGGCCGCACCATCCGCAATGCGATATCAGCGAGCGTGAATTTACCTGCAATCTGGGGGAAGTGGACGCGCTGAAAGTGGAAACGGAAGGTGATGTCACGAAGTT
ATATGCCCAGTTAATCCCGAATCAATTTTTAATTGAAGCCAATCGGCAGGGGCAAAAGTTATTTACGTCAGCAGAATTTGTCACTGATTTTGCTGGCAGCGGTCGTGAATATCTTTTTGGACTG
GCTGTGACGGATATTCCGGCAAGTCTGGGAACGGAAAAACTTAAATTCGTTTTAGCCGGTGAAGAAAAGGACGCCGAGCGCGGGAGTCTGGAAACATTCAGCTTAGGGAAATTACAGAC
AAGTAAACCAGATAAAAAAGATTCTTTCTGGTCGCGTTTATTTTCGGCCAGTAAAGATTTTACGCCAACGCCAGAGCCAAACACTGATAAGCCCACCGAGGGCGACGGAGAAAAAATGGA
AGAATTAAAAGCGCTCATTCAGCAAATGCTGGATCTGCTTAAAAGCGGTAAAGACGCCGCAGCGGGTGACGCTGACACGGTTGATACGCCAGAACAGGCCGCCGATGTGGTGGCAGATGT
TGCCGCCCAGATCGCTGATGCTGCCGACGAGGTGGCCGAGCTGGCACAGGACGTTATCGAAAACCCGGAAGACGAAGTCAAAGCGGAAGAATTCAGCGCCGCCAAAGCTAACCTGGCA
AAAGTCATGAAGTCATTCAATGTGACGCCAGCGAAGCGCCCACGCGCCAGCCGTCGCCGTGACTTTTCCGCCCGTCGCCAGCCAGCTGGCAACCAGATGGACAACCTTGCCACGCAGCTG
ACTACCGTCCTGACCAAGTTGTCAGCGATGGAAAACGGTAATACGCGCCGCCCTGGCAGTGCGCCAGGCGGAAGCAAAGAACCGTTTGAATTCATGTAA
>PNPANE_22810 Terminase-6C domain-containing protein
ATGGCTAAATATTCCGATGAATTAAAAGAAGCGGCCCGCACGCTTTATATAAAAAGCTGGACGCCGAAAGATATTGCGCAGGAACTGAATATTCCACCGCGTACAATTTACCACTGGGCTGAC
GTCGGGAAGTGGGCATCACTGCTGCCTGTTGAATCGGTGGAAAATGTCATCGCCCGCCGTATCGACCAGCTCTCCCGCCGCGAGAAAAAAACAGCGCTGGAACTGGAAGAACTGCGCGAT
CTGATTGCTCACCATGTGAAACTCATGGCGCAGCGCAACAAGCACGCCGAAAAGCTGGCAGAAATTCAGGCCCAGAAAGCGGCTTATGATGGTGAAGGGTACTGCCTCAGCAGCGCAGG
CGGGGAACAAGGGGAAAGAAAGCGCCGGTATAAGAAAAACGACGTTTCCGGTATTACAGCGGAAATGCTCGACACCTGGGCGCGGGAACATCTCTTCGAGTACCAGCTGCACTGCCGCG
AACATAAAGGCGAAGACTGGCGCTTCATTCTGAAAAGCCGCCAGGTGGGTATGACCTATTATTTCGCCTGGGAAGCATTCGAAGACGCCGTCATTACCGGTGATAACCAGGTCTTTTTCTCT
GCAAGCCGATCCCAGTCGGAAATCTTCCGCGAATACATCGTCCAGATCGCCCAGAACCATTTCGGCGTGACGCTTACGGGTAAAAATATCCGCCTCAGTAACGGCGCAATACTGCGCTTTTT
GTCCACGAACGCCAGCACTGCGCAGGGCTTTAACGGCCACCTGTATGGCGATGAGGTTTTCTGGATCCCGAAATTCACGCGCCTGCATGAAGTTGCCAGCGCAATGGCAACGCATAACAAA
TACCGCACGACCTACTTTTCGACGCCCAGCGCGAAAACGCACCAGGCTTACCCGGTCTGGACTGGCGAAGCCTGGCGCGGGGACGACCCGAAACGCAAAGGCGTGAAGTTCCCGAACG
AAAGCGACATGCGCCAGGGCATTCTCTGCCCGGATCAGATCTGGCGCTACATCATCACGATGGAAGACGCTATCGAAGGTGGCCTCGGTGCGCTTGTCGATATTGAACGGCTGCGCAACAA
ATACAGCCCGACCGCGTTCGCCATGCTCTACATGTGCCAGTTTGTTGACAGTAAAGACGCGGTTTTCAAGTTCTCCGCACTTGTTGGCTGTGAAGTGGACCGGGCCACATGGGGCGACTTT
GATCTGACTGCTGCGCGGCCCTTCGGCAATCGCGAAGTATGGGCAGGTTTTGACCCGTCGCGCTCCGGCGACAACTCAACATTTGTGTTAATCGCGCCCCCTATAGAGGACGGCGAGCGCT
TCCGCGTGCTGGCCGTCTGGCAATGGCAGGGTTTTAACTTCAGCTGGCAGGCCGACCAGATAAAGCAGCTTATGCGCCGCTTTAACATCACTTACATCGGGATCGATACAACCGGCATAGGT
AAAGGCGTTTATGACCTTGTCAGCAAATTTGCGCCACGCGAAGCCACACCAATTCTTTACAGCGTCGAAAGCAAAAACCGCCTGGTGATGAAGATGATCGACGTTGTCGAGCGTAAGCGCA
TCGAATGGGCAAAAGACGCCGTAGACGAAACGAACAAGGAGCGCGTCGAAATTCCGGCCAGCTTTATGGCTATCCGGCGCACGACAACCAACAGCGGCAACGCGCTGACGTTTGTTGCC
GAACGTTCAGACGCGACCGGCCATGCAGATGTTTTCTTCGCTATCTCGCACGCCGTAATTAACGAACCTATCGATCACGAATATGACCGCCCATCGGGCTGGTATTTTGGGAAAGCAGCATGA
>PNPANE_22815 Phage portal protein
ATGACAAAGAAACAGCGTAATAATAAAAAATTCAGGTCCATGATCGGCAACAAGGTTGAAACCTTCACGCCAGGGCGCGGAAGCGTGATCACATTTGGCGAACCCGAACCCATCCTGACG
ACCGGCACGGATTATCACAATATCTGGTATGACAATGAGTATGACCACTGGCGACTTCCGATTGATCGCCTGGCGCTGGCCCAGTTGCCGAACCTTAACGGCCAGCACGGCGGGGTACTGTA
TGCGCGGCGCAATATGGTGGCCGGTGGCTACATCGGCGGCGGCCTGACGCCGGACCAGGTCGAACAGGCTGTCTTTGATTACCTGCTGTTTGGTGACGTCGCTATCCTGAAAATTCGTAAC
GTATTCGGGGAAGTGATCGACCTGCTACCGCTGCCGTCGCTGTATCTGCGCTGCCGGAAAGACGGCTCGTTTGCTGTCCTGCAGGAAGGGCCAGCGCTTATCTATGACCCGGAAGACATCG

TATTCTTTAAAATGTACGACCCTCGCCAGCAGGTGTATGGCCTGCCTGACTATATCGGCGGCATCCATTCGGTGTTACTCAACAGTGAGGCAACCATCTTCCGGCGCCGTTACTACAATAACGG
GGCGCACATGGGCTTTATTCTCTATACCAGCGATCCGAATCTGACGCTGGAAATGGAAAACGAAATCAAAGAGAAGATCGCCCAGTCCAAAGGGCTGGGTAACTTCCGCAATATGTTTATCA
ACATCCCGAAAGGCGACCCGGAAGGGGTTAAAATCATGCCAGTGGGCGAAGTCAGCGCAAAGGATGAATTTGCCAACATCAAAGGGATCACCGCGCAGGATATCTTTACCGCTCACCGTT
TCCCCGCAGGTCTGGCGGGTATTATCCCGACCAACGGCGCGATAATGGGTAATCCTGAAACGGCCCGCGCAACCTATCGCAAGGATGAAGTTATTCCGTTGCAGCGCAAGCTAATGAACGG
CGTCAACAATGACCCAGAGATCCCCCCACATTTGCACCTGCATTTTGATATAGATATCCCGGCAATCACCGCCGAAAAGGGCGAAAAATGA
>PNPANE_22820 Transposase
ATGAGCGCAATTAAGTTAAAATCACCCCCATTGTTAGCAATGGCGTGCGGGATGGTGAACATGCGAGTTTTTAAAATTAAATGTCCTGAATGCGGCTCACCGGCCATCATTCGCAAATCAGAC
TGGAAAGATAAAAAGCTGGCCGATTTATACTGCGCCTGCACGGAAGTTGAATGCGGCCACACCTTCGTTTTTAACGCCCAGTTTTCACATACCCTCAGCCCCAGCGGGCTGACCGGCAACA
AGCTGGTTAAATTTCTGATTGACCGGCTCAAGCCAGACGAAAGACAATTCGCTTTAGATTTGCTAAACAACCATATTAATTAA
>PNPANE_22825 NERD domain-containing protein
ATGAGCAATTCAAAATATATTGATTTAGATTCGAGGATAAAAAAATTAAAAGAAGTTGTTAAAGATTTAGATACAAACACTTTAGCAACCTACTCCTTTTGGGAAATGTACAAGTTTGGCACAT
TAGGTCAAAAAATTGAACTACAATCACCCGCTCGACAAATCTTATATCTTTTTGGGATTGCATGTGCGTCTGTTGAACCAACCGAGCCAACTGATAATGTTGATGGTAAGATAAAACAAACTGT
AGAGATCCTAAATGATATTTTTACAAAATATATGCTGGCGTACTTTCCAACCAAAGATGATTTAAAATCTGGATTAGAAGATGAATGGCATAAAGTTAGAGAAGTTGCTATGCCAATGTTTTCCA
ACTATTTCTTTGAAGGATTAAAAGTTTCCACTGAAAACTTCAAAGAGATCATAAAAAAATTACTTCGATGGTTTCGAAAATGAAATAAAAACATATTTTGGCTTAGATCACCATGAAATGGTGGA
AATTTTAAATCTCATTGGTGAGCTAATTCAGAGCAAATATGATAGATACCGTGAAATCATGGATACATTAAAAAATGAACATGAGCGGTTTAAAGAAAATAATTTCGAAAAAGTTGAAGATTTT
AAATCATACATGGATGACCTGAGGGAGCGTACAAAGCATTTAGCACAAGAATATTATGACTTCTACAATGGGAGTGTATCATTCAGATTTGATTCAATAAGATATAAACTTGGTGACGATATCG
TGGATAGTTTTATAAAAACTTTCGTTACTGAGCGTGGAAATTCACCTGAAATTAAATACATTACTGATGAGAATCCATTTTCATATAAACCGATAGTAACTGTAAATAATCTGGATTATATGCTCCC
TTCTGCTAACGCAGCTTATGAAGCTATCATACTTAATCTTGAAAAGTTTTTTAAAGAGTCAAAATACAATGATAAATTTAGAAAAGCCAGAGACAACAGGCTAGAACATGAAACTTTTTGCAC
CTTTAGAGATTTCTTCCCTGAATCAACTACAATACTTGAGAGTGTATTTGAAACAAACAAATCTCATAATGAACATGATCTAATTATTTTCCATAATAAAACCATTCTTATTGTAGAGGCGAAAGC
ATCACCACGCCGCGCTCCATTGAGAGAACCAGCCAGAGCATACATTAGAATTAGAGATGATTTCAAACGGAAATCAGGAATACAAAGTGCATCTGAGCAAGCAAATAACTTAAGAAATTTAA
TAATTAATAATGAAAAAACACCATTATATGATAAAAAAGGTAATTTATTATATACCATAAATCGTTGTGACTATGACAATATATATTGTATATGTGTCACTAAAGATGATTTCGGAATGCTTGCTACA
AATTTAACTTTAATGTTAGAAAAAAAAGAAGATGAACCTTACCCTTGGGTTATTTGTATTCAAGACCTGAAATTTTATTTTGATTGCCTTAAGGAAATAGATTTGGATCATGACTATTTCTTAAA
TTACATTCATGAAAGAATAAAAATACACGGCAAAGCAACAGCATGTGATGAACTTGAATATGCAGGAGCGTACTTAAATTATGGTGGTTTCGATTTCATTAATAAAGAAGTAAACTCTAACGTT
TTTCTTGATATATCTGAATCACGAGTATTCGATGAAATCCATCTCGAAAAGATACATGGTAGGAAATATGTTCACCAGATTAAAAAACCTACTTACAGCATTTTAAATAGAGATAAGTTATTTTCT
TCTTTAAATACTAAGCACTCAAGGAAAGATGCAAAAAAAAATAAAAGCTAAAAGAAAGGAACAGAAAAAATCCAGAAAAAGAAACCGTTAA
>PNPANE_22830 Replication endonuclease
ATGTCTGACGCCGCTTTTGCATGGCCCTGGAACGCGCCGCGCCCAGCCGTTGGTCTGTACACCTACGAACCGAAAAAAATCGCCCCGCTTGCCGGGGCGGTGGTGCATCATCCTGCCGTAA
AAAAACACATCGATCACATCTTCAAACGCGCCGGTTACAACCCCGACGACGTTCGCGACCGTGACGCGCTGATCCAGTCGCTGGACAGGTACGAACCGTGCGGCCTGCCACTGGCCGCCC
AACAAAATATCATCCGGCAGGAAAGTGCAGCCGCCAAAGCAGCGGCGGCTACCTGGGCCAATACGCCGGAAGGTGTCGAAGCGCGTTTGTTGTCTGAGCCGTTCTTCATTCGCGAAGTCT
GGCGCAAAAAAATTGAATGGTTACGGGCCAACCGTGAAACCAGACACACCAATGATTTTCTTATGGGGACCGTGAAAAAATCATTGCTGCGTCTTGATGTTGTGCGCACAAGGCAAGGCGT
TTCGCCTGATATCACCGGCGAACTGGCCGCGTACTGGTTCGGGCGCTGGCAACGGCTGGCTGATTTCACAAAGCGGGAAGCCCTAAGCGCCGCTAATGAGATCGCCAGCCGCATGGCTGA
AATGCTGGGGACGGAATGCGAAGCCCTGGGACGGAATGTTTCAGACATGAACGTCGAAGAACTGGACTGGCTTTATTGCCACCTGGGCCGCGAAATGCTGGCGCTTCGCATTGTGCCGCC
TGCATGGTATGCGCCGTGGGAACGCGAGCGCATATGCACGGCCATTTTGCGTATGGCTTCGCCTGACTGGTGGGGGCGCAAAATCTGGCGCCTGCGTTGTGACTGGCGCGAAAACCAGCT
GCGTGCTGTTGGTGCGGTAAATAAAAAAGCGCATCCGTATATCAGCGCATCAAGCCTGATGGAATGGCAGGAACAGCGACGTAAAAACCGTAATTTCTTCAAAAGTCATGAACTGGTAGAC
GAAGACGGCAACGTTTCGTCGCTTGAGGACATGATTAACAAATCCACGTCTAACCCTGCAATTCGTCGTCATGAGCTTATGGCCCGTATGGCTGGCGTGGAGCTTGTCGCCCAGAGTCGTGG
CGATGTTGGCATCTTCCTGACCATCACTTGCCCGTCGAAATATCACGGCAATATTGCGTCCGGCCACCATAACGCAAAATGGAACTACGACACGGTTGCACAGGCGCAACGCTATTTATGCCG
TGTATGGAACCGGGCAACCGCCAAACTGAAACGCGAAGATTTGCGCCCTTATGGCTTCCGCGTCGCCGAGCCGCATCACGATGGGACACCACATTGGCACGCGTTGCTATTTATGCCACAA
GAGCAGGTTAAAGCCACGGTTGCGATCCTTCGCGCCTACTTTATCGCGGAAGACCGCGACGAGCTGGGCCGTAATACCGGTGCTCGTTTCAAGTCAAAAAAAATGGACCCACGGAAAGGG
TCAGCAACGGCGTATATCGCCAAATACATTTCGAAGAATATCGACGGCCACGCGCTGGCCGGTGAACTGGACGACGAAAGCGGCAAGCCGCTGAATGAAACAGCCAAATATGCAATGGCC
TGGGCGTCACTTCACCGCATCCGCCAGTTTCAGCCAATCGGACAGCCGCCCATATCGGTTTACCGCGAGCTGCGCAAACTGAGCAATCAGATCACGACCCGCCAGAAAATTGACAATACCTT
CAAGCGCGGTGCGCCGTTGCTTGTGGATCCTGCAATGGATGCGGTTTGCGCCGCTGCCGATGTCGGATGCTTTGCTACCTACATCATCCGCCAGGGTGGTGTTTTGATCCCGCGTGAAAACT
ATGTCGTCCGTCTGGCCTATCAGCCAGCTGATGAAATGAATGCTTATTGTGAGATCCCCGAAAAGGTTTTTGGGGTCTGGTCGCCGCGTCTGGGTGATGCCTCCCGTATTTGCACCCGTCTGG
TTAAGTGGAAAATCCGCGCCAAATCTAAAGCCGCCACCGGGGCCAAAAACGGCCCCGGTTTGGGGGTTGACCTTTTGCCGTCGCCAACCGGCGACGCTTGGAGTTCTGTCAATAACTCTA
CGGAAGACGAAAAAATCACCGATTTTTCACTGTCAGAAATGGCTGTGCCAGCTGATGGAGAGTTAAAGACAGAAGATTCAGACGATAAAATCGTCGATTTTGAAAATATGGACCAGCCAAC
GCGGCGCAAATTGATGCGGCGACTACGTGAAACACCATTCAAAAGGCGGCAAAGCGGATCACCTTATGAGCCAGGGAGCGAATTAGATGTTGCCTGGCGTTCTGCCGTTGAAAAAACCGA
GGCCAGATCGGCGGCTGATAAAGCCAGGCGGGCAGCGCTTGCCCCTGCGGTTGCCAGTCTGCTGGCTGATGCGGAGTTGTGTTCGGTTGAGATTTCGGAGATTCAGGCCGTTTCGCTGTT
AATGGGGAGCCGTCTGGAGATTGGCGGCAAAGTTTATCGGGCCAGTGCTGGCGGCCAGTTGATAACGCGTCAATTACCCGATGAGTCACGGACGGTGAATAAATTATGGGACCGTCTGCG
GGATAACCACGGCATTGATGCCACGCGCCTGCGGTTTGATCCGGTCGGAGAGTATCAAAAAATGCTGGCGGGTGTGGAAAGAAGTCACTCAAAAAAATGA
>PNPANE_22835 Site-specific DNA-methyltransferase (adenine-specific)
ATGATTCGATCTCTCGTTAAATGGCCCGGTGGTAAAGGCCGCGTTATGTCTGATTTGCTGCCGATTCTGCCGAAAGCCGATTGTCTGGTGGAACCGTTTGTCGGTGGTGCTTCTGTTTTCCTC
AATACTGAATATCGCCGTTATATCTTGGGTGATATCAACCCAGATTTAATTAACCTGTATCGCCAGATAACCCGCTGGCCTGATGCGGTGATCGACGCTGCTCGCCCGCTGTTTAAAGTGTACG
GCGATAAAGACGGCTATAAGTGGATCCGCGGCGATTTCAACGCCCGCGCCCATGACCTTCTGTCATCGCGCAATGTGTTTGAGGATGGACCGGACACGGGCAAGATTCTTCGTGCAGCACA
ATTTCTTTACCTGAACCGTCACGGATATAACGGCGTAGTACGCTACAACCAACAGGGTGGATATAACGTTCCCTTTGGGCGACACAAAACCCCGCCTTACTTCCCGGAAGAACAGATCCGTTT
ATTCTCTGAAAAAGCTAACGACACGAAAGCTATTTTCGTGTGCTGCGATTTCCAGAGCACGTTAAAAATCATGATTGGTAGTGACGCGGTTATCTACTGCGATCCGCCATACCTGCCAGCAAG
CGACACCGCTAATTTCACCCAATACCACACCGCCCCGTTTGGCATTAAAGAGCATCGCCAGTTAGCTGCCGCCCTGCTGGATATTAACCGCCTTACTGGTTCGCCGGTGATCTTGTCCAACAG
CGACACCCCAGCTACCCGCGAGATTTATCACTCTTTCAACTTCCAGGAAATCAGCGTTAACCGTTCTGTCAGCGCGAACGCCATTACCAGAGGGGCCGCCAGTGAAGTGATAGGCGTGCTG
CCTGTCTGTGATGGCTGCGGACGCTATGAAGGTGGTCACTGTGCGAAATGTTGCCCAGGTATCGCCGCATGTGAGGAGGGATGGTCGTTTGATTGTCGAGGCAATTGCGATATTTGCGAGC
CGTCCGATAACCCGGAAACGTGGTAA
>PNPANE_22840 Phage protein
ATGTTGATGGATAAGACAGGACAACAGCCGGGCCGCCGCAAGTTCTTAGAGCAACGGGCGCGGCTGCAAGCCAGTTTGAACGCTTCACGCGTAAATGACACTGCAACCCGTTTTAACCGC
CTGGATGATGCCTGCAAAAAGGTGATTTTCATCCTGGCAAACGATGCGTCCAGATACATAGCCGGAATGCCGAAACTGACCGCCAAACAGTTGGGTTGCACTTATGAAAATCTAACTGAAAA
GGAGCAAACGTGCCTTTTGATGGGCATTAAGCGCCTTTCAGAATTTGCAGCATCAATGCCGTGGGAATTTGAGGACTACGCCGCACCACGCGCCGAAATTCAGGCGATACGCGACAAACC
ACCCGCGCCAGATAACGCAGTCAATTAA
>PNPANE_22845 DUF2724 domain-containing protein
ATGGACTTAACACAATGCCCTTCACTAGCCAGCCTGCTCACCCACGGCCAGCAGATCACCCACCGTCAGCATCAACGCGGCTGGATTGAAACCCCGGACGGGCGTTTCTTCCAGCCTAAAG
CGGCAGATGTTCAATTTGTTAAAAACTGCCGTTTACCGTTTATGTCGCGCCCGCGTAATAAGCGCCGCTGGTTTTCCCGCTTAATGGGCATCTTTGCGTAG
>PNPANE_22850 TerB domain-containing protein
ATGAACAAACAAGAAATTGCTGAGTTCTTCGAAGAAGCAAAAGCCTCTCGCACAAAACATTCAGTTAATGATCGCCTGGTAGCTATTGAATTCATGGTCTGTTCAATTGCAGCTGCCCTTGAA
GGAAGCGCGAAAGAAACACACCTTAAAACGATGAATTCTTTCTCTGAAAACTTCGACCCATTGAAGGATCCAACCCTTAAAGCAATTGCTGACCTGAATGTTTTAAGCGCTGACTTTAAAAA
TCTCGCTAATCAACTTGCAGATTCGAAATAA

>PNPANE_22855 Thymidylate synthase
ATGAAAACACAAAGATTCTTGATTGCCACCGAAGGCACATTCGATGGACGCCAAATTAGTGCGGAAGCCATTCGCTCTGTTTTGGCTAATTTCATTGCAGGCGGCATGACTCTTCCGGTTGT
GTATGACTTCTACGATTGGAGTCCTGCTTTAAGTGAAGTTGTCGCCCTGGCCGTCGATTCTGATGGAGAGAAAATGCACCTTTATGCAGAGGTGTTTGCTACCGAAGAACTTCAACAAATGG
CCGCGCGGCATGTCGATTACTTTCTGGTTCCTGCCATTCTCAGCATTAATGAATGTGACACAACCGCGTGTTTAGTCTCTGTTGGTTTAACGAAAGATCCACTAATACCCGGTCTGGATAAAAT
GCAGTTTTGTTCGGCTAACCATTCCTACCGCGTTTTTTCTAAACCCAAAAATGAGGGTGGTATTTGCAGTCAGCAGAAAGTAAATGCCGGAAAAAAAATGCATCTGGCCCGTTATTACCATCA
TGGCGCACATGCCGGAAATGTTCGTTATGAGATTGAGGGATGGGGAATGAACAGCCCCGTAAGAGAGGCTGAAATTAAGCGTCGATTACATGAGTTTATTTCGAATCTGCAAGTTGATTAG
>PNPANE_22860 Cox protein
ATGGAAGCTAATGATTACGTGATCCAGTACCCACTGGACGCGGTGCATACAGACAAGTTCGCCGAACTTCTCGGTAAACCAAAAACCGCTGTCGAAGCGATGGCAAAAGCCAATAAGCTGC
CATTGATCGAATTGCGCGACCCTTCAAAACCTGCCGCTCGCGCTGGTGATAAGTGGGTTTTCATTCCTGAGTTTAATCGTGGTGTTCGTGAGGCGTTTTATAACCGCCCAGTAGAACAGCGC
GATGCTTGGCTTTTATGGATGGGGTTATAG
>PNPANE_22865 Integrase
ATGTCAATTAAGAAGCTCGATGATGGTCGCTATGAAGTGGACATAAGACCGCGCGGGCGCGAAGGACGTCGCATTCGCCGGAAGTTTGAAAGGAAAGCTGAGGCGGTCGCATTTGAACG
CTATACGCTGGCAAACGCGAACCAGAAAGAATGGGCTGGACAGCGAGCAGATCGCCGCACGCTAAAAGAACTGCTGGATTTATGGTGGAAGTATCACGGTCAGAATCATGAACACGGCCA
AAAGGAATTTAATCACCTGCTCAAAACTATCAGCGGGCTGGGTGATGTTCCGGTTAGCAGGATGAACAAAAGAATGTTGATGGATTACCGTTCCGCAAGACTTCGCGAGGGGATCAGCCCA
TCAACAATTAACCGGGATATGTACCGTTTTTCCGGCATGTTCACAAAGCTGATTCAGCTGGAAGAGTTTGTCGGAGCACATCCAGTACATGGATTGCCGCCGCTGGCCGAGGCTAACCCAGA
AATGACCTTCCTGGAGAAAGCCGAAATAGCCAGCCTGCTGGATGCCTTAACAGGTGATGCCCGATTAATTGCATTGTTGGGGGTTAGCACTGGCGGGAGGTGGTCAGAACTAGCAACGCT
AAAACCTTCGCAGATAGCAAACTGCCGGGTCACATTCCTTAAAACGAAGAACGGGAAAAAACGAACAGTTCCCATTTCGTCAGAACTGGAAAAGATGATCAGGAAGGAAGCCAGCGCGA
AACTGTTTAAGGTGGATTATGAGAAGTTTTGCAAGACTCTGCGGATGGTCAAACCCGACATCCCGCCGAACCAGGCAACGCATATTTTGCGCCATACCTTCGCCAGTCATTTTATGATGAACG
GGGGGAACATAATTGCCTTACAACAAATACTGGGACACGCCAGCATTCAACAAACGATGGTCTATGCGCATTTGTCGCCAGATTATCTGCAAAATGCTGTCGCACTGAATCCGCTTGCGGGT
GGAACGTCGATTTAA

Prophage 13

>AONIPM_23605 Prophage integrase IntD
ATGTCCATCTTCCGCAGAGGTGAAATATGGTACGCGTCCTACTCGACACCGGGCGGGAAGCGAATTAAGGAAAGCCTTGGGACTTCCGACAAGCGGCTCGCTACTGAGCTACATGACAAGC
GCAAAGCTGAATTGTGGCGAGTAGACAGGCTTGGTGATTTCCCTGATGTAACGTTTGATGATGCCTGCATGCGCTGGCTTGAGGAAAAAGCGGAGAAGAAATCACTGAAAGATGACCGTA
GCCGTATGGCTTTCTGGCTGGCGCATTTTGAGGGAGTACGGTTAAAGGATGTAACCGAGCAAAAGATTTACTTAGCAGTAAACAAGATGAGCAACCGCAAACAGCTTGAGATATGGAAAAT
CAAAGCTGCCGCGGCGCAGAAGAATGGAGAACCTGCACCAATCTATTCAGCTAAACCGGTCACAACCTCCACTAAGGCCAAACACCTGGCATTAATGAAGGCTATTCTGCGTGCAGCAGAA
CGTGACTGGAAATGGCTGGAGAAAGCGCCTGTAATCAAGGTTCCAGCCGTCAGAAACAAACGCGTCAGATGGCTGGAGAAAGAAGAGGCAAAACGCCTGATTGATGAATGCCCTGAACC
GTTGAAATCTGTTGTTAAATTTGCGCTGGCAACAGGACTTAGGCGGTCTAACATCATCAATATGGAATGGCAACAGATTGACATGCAACGTCGTGTTGCCTGGGTGAACCCTGAAGACAGCA
AGTCAAACCGCGCTATTGGCGTAGCGCTAAATGACACTGCCTGTAAGGTATTGCGTGACCAGATTGGTAAGCATCATAAATGGGTGTTCGTGCATACGAAAGAAGGCATCCGGCCCGATGGC
TCAAAGACTCCGACTATCAGAAAGATGCGCGTCGATGACCAGCGAGCATGGAATGCAGCTTGCCGCCGGGCTGGAATTGAGGATTTCCGTTTCCACGATCTGAGGCACACGTGGGCCAGT
TGGCTAATTCAGTCCGGAGTGCCGCTTTCTGTTCTGCAGGAAATGGGAGGATGGGAGAGCATCGAGATGGTGCGCCGATATGCTCACCTTGCGCCGAATCATTTAACGGAACACGCGAAGC
AAATTGACTCGATTTTCAGTGATGATGTCCCAAATATGTCCCATATGGAAAATAAGGAGGGAATTAAAGAGGCGTAA
>AONIPM_23610 DUF3800 domain-containing protein
ATGTCTGATTTAGCAATGAAGGTATTGAAGTGGCAAACGAAAGGCCACGTTGGCATAAGTAGCGCAACTATGGCTTCTATTGCTCTTGGGCTGGAAAAGAGCTTCTACCACGGACGGTTTG
ACGCACCAAGCGATCCTGCCGATTTGCGAAGATGCATGATGCTCGTAGATGAAATACCTGAAATTAAAGATAGCTTTCCGCTCATAGCGAAAAAGGTAAAGCGGTTTTCTCCGATTTTACGTG
AGTGGGATTCACTTATTGCTCTGCTTAAGCTTGAGCTTAAGAGGCCAGATAAGCGAGCACCAAAAACATATAAATGGATAAAAGAGCTTCTTTCTGACCAGGAGTAA
>AONIPM_23615 ASCH domain-containing protein
GTGGCTAACCTGCAACTTGCCGTCAAAGGTGAATACTTCGATGCCATGATTCGCGGGGAGAAAACGGAAGAGTATCGCTTGTGTAATGACTACTGGAATAAGCGAATTATGTTCCGCGAGTA
TGACCGACTGATTATCACAAAGGGATATCCAAAGCGCGACGATTCCAGTCGCAGAATTGATGTTCCGTACGACGGATATGAAATCAAGACAATCACACATCCCCACTTCGGCGATAAACTGGT
GAAGGTATTCGCGATAAAGGTGAATATCGGCAATGAATAA
>AONIPM_23620 DUF551 domain-containing protein
GTGAAAGAGAATCAAATCCGGGAGCTTGTAAACGAGCTGCATGATATTGCTATTGAGTATCACGGCACACAACAGTTACGTGAACGAATTGCGCGTACAGTTCGCGCCGCCCTGCATCATGA
CTTAGAAAAACTAAACCAACCTGTAAGCCAAACTTACGAGTTGCCAGAATTAATCGAAGGCATGGAGGTGTCCATTGATGTCAGCACTTGTGATGCTGATGCCGGGAATCGCTATTTCGGTA
CTGTCACCGAGGTATCAGAACTGGACACAGCAAAGAATGGCTACATTCTTCTGGTTCAGGACGCTGAACCAAATTTCGATGTGAATGGCAACTCTCCGGTAATTCCGGATGGCTGGATAAGC
TGTAGTGAGCGAATGCCGGATAATGATGAATCTAAACCCATCGCAATTTTTACCGGAAAATGTCTGGGTCAGGGGATGTTCGTTGCTACATACGACGATGATGGGTTCTTTGACTATTGGGAG
GGTATGGAAATTATCGGTGTAACCCACTGGATGCCGCTACCGGAGCCACCGCAGGAGGTGAACCGTGGCTAA
>AONIPM_23625 Prophage protein
ATGACCACTATTACCAGAGAACAGCAAAAACAGATTTTAATTGATACAGCGAACCACGTAATCAGTCGTGATAACACGTCACCGTATAGCGAAAACCTGCGTGAACTGGCGCGTATCGCGCT
GGCATCGCTCGAAGCGGAGAAAGGTGCCGACCCCGTTGTGTTCACCGACGAACGAAATCTTCATCATATTGCCAGGGGTCGAGAAACCTCTTTGATTTGGGGTAAACAAAACCAGGAGGT
GGGGGATATCCCGCTCTATCGTCACGCCCAGCCAGTGCCGGTAGTGCCTGATGAAATGGCGACATCTGATGACATGAATCTTTATCAAAAGAGCTTTGCGCAAGGCTATAACGCCTGCCGCA
ATGCCATGCTCAACGGAGGTAAATCGTGA
>AONIPM_23630 hypothetical protein
GTGAGCAAAATTGACCATCAGGCACTGCGTGAGGCAGCAGAACGTGCAACTCCAGCAATGGAACGCCTGTTAATGTTGCCTGTTGATGATGATTTGTTAAGTGAACAGGAACTTAAAGATT
ACGGTGTGGATATTGATGCGCTCAACGCCTTCAAATTTCTGACCGGACCAGAAACCGTGCTGGCGCTGCTGGATGAGAATATTCAACTCCAGCGGGGAAAAGACGCAATAGAGGCCGTAG
CGCTGGTGCTGCGTGATGATATGCGACAGGCGCGAGAGCAACTGGCAGCCGCAGAAAAGCGGAACTCCGAACAGCGTGAGTATTACGAGGGCGTTATTGCTGATGGAGGTAAGCGCATA
GCAGAACTCGAACACAGCGAGACGCAGCTTATCAATGAGCGTGATAGTGCTGAATCTGCACTGGCCGATATGTACCAGGCCGCAACAGGAGAGCGTCCAGAATGGAGCAATATGTTTGGTT
TCGCTGACGCCGTTGATGTGGTGGAAGAACGACTGGCGACGCTGGAGGCCAACCAAAGCCAAACCACGCCAACGGGAATTCAGCTCATCACAGAAGCCATAGGTGCGCACGGCTATATCG
TTGGCTGCCTGTTGCAAGGTCGCCCTGATTTGGCGCTGGAAGAATCGAGAAAGTGGGTATCCGCTTTCGGTCAGGCGGCGGAAATAGTTAGTGCACAAGACGCCGATGACATCAAGGTTA
AGGGGGAGTGA
>AONIPM_23635 ClpB-D2-small domain-containing protein
ATGAGCAATACAGCAAAACTTCAACTCGGATTCTCTCCTTTATCAAAAACCATCATGCTCGCAAAAATGCGCGATGTGGAAGGTGGCCGCCTGCGCGTCGGTAATGATCGTGGTCGTGATGT
TACCAATGAGGCTGCGCAGCTAGTGTGGCAGCTTGTCATGGCGGAAGGTGGTGAAATCGGCTGGGAGCTTGATGATGGTTCTCGCATGGTGCTGAAAGCTGAGAAGCAGGAGGCAACCA
GTGAGCAAAATTGA
>AONIPM_23640 hypothetical protein
ATGAAAGAAGTGAAAATATACACGATTGTCAGTGACCAGTTATCACCACCAATAACAGGAGAATCATTCTGTACTGACATGGTGCGTCATAGTGATTATGCGGAGCTTGAGGATAAATACGCG
GCGTTGGCTGCGGATAACGATAAAGCAATGGAGTCACTTAAGCAGGCTGATGCAGTTGTTAAGTTGGCACACGAGAAGTTTTCGGCGCTGGCTTCGGAGAATGCGGCACTGAAAAAATCA

GAGGTCGAATTCAACGAATATTGTCGTCGCGAGTGCGAGGACGTTGGCGATACGTGGGTGGACGATTTCACTGAGACCCCAGCCACCGACACTTTTCTGGCTGAGGTGACACAAGAAGCA
GTAGAGTCACTCAAGAAGGAGATTGAATGGCTCAAAAAGCAGTTGCTTATGTCTGTCGGTGATATTCGAGACCTTATTGATTACAACCCTGAAAATGGCGTTCTTACAGCGAAAGTTAACTTC
AGCGGAAGGCAGGCAGGTTCTGTCATTGGCTCTCAAACTTGGCAGGGATATTACGCATTCTCACTGTTCGGTAAGAAGTGCTTTGCTCACAGACTTGCATGGCTACTGCATTACGGTGAGTG
GCCTTCACAACCTATCGATCACATAAACGGAATTAAGACGGACAACAGCATAAGAAATCTGCGCCTTTGTTCTCTATCGCAGAACCAATTCAACAAGCCTACTCAGAAAAACAACACTACAG
GTGTGAAGGGTGTGTATTGGAACAAGCGAGATAAACGCTACGTTGCCAGCGTCCAATTTAACGGAAAGAAATACAGCGCGGGACATCACAAGGATATTGATAGCGCTAAAGAGGCTGTAAT
GAAACTTCGGGAAAAACTGGCTGGAGAATTCACTAACCACGGCGAATTCGAACTCGCCGCCCAACTTCGCAAAGGAGCCTCGCTATGA
>AONIPM_23645 hypothetical protein
GTGAGCAAACTGTATGAAATAGCTAATGAATACGCAAAATTGATGGATTCAGATTTAGAACCAGAGATGATTGCTGACACAATAGAAGGCATGGAAGGAGAATTTACCGATAAAATAGAGCA
ACTTCTTGCCATTATTAAAAATGAGTCTGGCTATGCTGAGCGCCTCAAGGAAGAGGCAAAGTCACTGAATGAACGAGCAGCAGTAATTCAAAATAAGATTGACAGCATTATGGCATATATAGC
GTCATCGCTTGAAATGGTTGGCAAGAAAAAGATTCGAGCTGGTATTCACCAGGTGACAATCCGCAAACCGACAGAAACTGTAGAAATCATCGATTCCAGCGCCCTTCCTCCAGAATACGTTG
AGTTTGAAACAACAATTAAAGCCGACAAACTGGCAATTAAACACCAACTAAAAGCTGGAATAAATATCCCCGGCGCTTACCTCAAAGTTGGAAAAACTTCACTTCTTATCAAATAA
>AONIPM_23650 DNA recombinase
ATGAGCAATATCGTTGAATTCGTTAAACAGCAGGAGCACTTATTCTGCGGGGCATTGACTGAACAGACGGTAACATGGGCTAAGGAAAGCCAGTTTGCAATTCAGTATTTCCAGAAAAACG
ATTACCTGGCTAAAACGGCACTGGCAAATCCAACCAGCGCACAGAACGCCATCATCAATGTTGCCGCCATAGGAATCACATTAAACCCGGCGAGCAAACTTGCTTACCTTGTGCCACGTGAT
GGCATGGTTTGCCTTGATATCAGTTACATGGGATTACTTCACCTTGCACAGTCGACAGGCTCAATTAAGTGGGGGCAATGCAAACTGGTGTACTCAAACGACACCTATGAATCAAACGGCCT
TGATTCAGCACCAACCCACAAATACAACGCATTTGGTGAGCGAGGCTCTATTGTTGGTGGTTATTGCACGGTTAAAACAGCAGATGGTGACTACCTGACTGAAGAAATGAGTCTGGCAGAA
ATTAAAGCTGTGGAAGCAACGAGCAAGGCAAAGAATGGACCGTGGAAGACATTCTGGGAAGAGATGGCGCGCAAAACAATAGTTAAACGCGCCAGCAAATACTGGCCTAAAGCCCAGCG
ACTGGATAATGCCATTCACCTGCTTAACGAAGATGAAGGTATGCATCAGGAACCGGTTATGCCGCACAAATCAGAGGAAGATATCCGCGAAGATGAACGGAAACGCCAGCAGGAAATAATG
GATAAAGCACAACTTCTTTGTAATGAAATGGCTCAGGCTGAAAACATGGATGATTTGAAGCGATATTTTGCAGAAGCATATCGCCTGACATCTGGAATGAAATTGCAGCAGAACGTACAAGC
AATTTACGCAGAATGCAAAGCGAAACTGGAGGTTGCCAGTGAGCAAACTGTATGA
>AONIPM_23655 Prophage protein
ATGATTCTGACTCTGAATGATAAGCGTGAAATATCGCAAATCATCGCAAGTTTTACCGATGATGATTATGAACGAATTAACAGTGAAGTTGATCGCCTTTGCAAACGTTGCGACCCAATAAGCG
AAATGCTTCGCTCATATAAACCAGATGAGCACACTAAGGACGCTATCGACTGGCTGGAAGATGATGATTGTAACTACCAGGAAAAAGCCGCTGAATGGTTCTGGGATGCAATAACCGATAGA
GTTAAGGCTGAATATGCCTTCGCAATATTCAAACGCAGACACATTTATGGAGAAGCAGCATGA
>AONIPM_23660 hypothetical protein
ATGAGCATCGCTGACGCATGGACAGATGATGCTTTTATCCGATTAATGCAGGACATGCTCAATCAGCAGAAAGAACAGGAGAACGATGATGATTCTGACTCTGAATGA
>AONIPM_23665 hypothetical protein
ATGCAAAGCCAAATAACAATCAATCACCAGAAGTTAATTGCAGCACAAAGCAAGGCTGTTATCGCTCGTTTTCTTGGTGACGGACACATGTGGAAACAAGCTACCGAGGAAATGAAATCAG
CAATCAACTTTCCATGGTACCGCAAGAAATGA
>AONIPM_23670 hypothetical protein
ATGCCAATAAATAAACATAAGGAACTTCCCATGATGCAACTCAGCTTTGCGGGAAGCGGCGTCATGTCCGCTTTTTACCCCGCTGAATCCGAATTATCAAAACGTATTCGTCGCCTTATTCGCT
CTGTCCGTAAGCAACTGGAGGCGTTATGCAAAGCCAAATAA
>AONIPM_23675 hypothetical protein
ATGGACAAGAAAATTAAATCATTGAAGCCAGGAATTGTTATCAGAGACATAAGTGGTGATTACGATACAGAGACATATGATATTTTATATGTGCATGCAGATGGAAAATGTCAGTATTCGAACG
ACATTTTTAATAATAAAGGTGGTGCCGAAATTGCTGCCACCACAGTTAATAAAGAGTTAGTAGCTAACGAGTCGTGGGATTATTTTATGCCATCATCCACTTCTATGAACTGGAAAGAGGTTTT
GTACATTCCATCATAA
>AONIPM_23680 Persistence and stress-resistance toxin PasT
TTGAAGAGTGCTACAGTTTACACGGCAATGAGAGTTAAGCAATATGGCGGCGTACCTTCGGTAGAGGTAAGGTGTGATGATAAAAGAACCAAAGTTGTTACTGATTGCCTTCTGCTTTTTAA
AAGCATAGATGAGGTAATTTTTATTGGCGCTGAAAATCTTCACCCTTTGATAAAAGCAGAGATGAAGCAATGTGCAATCGATGCATTGGGAGTTGGGCAAGGCAAGTCTCAGATCGAAGCA
AAGCAAATGCTTACAGAGCTTGAAAGCAGCAGACGTAGACAAGCAAGCAGGGCAAGACAATTTCACGATGCGATCGCAGGATGTAGCAGAGAGCTTATGTCTTTAAATGTAGACATACAG
AGAGGAATTGATATTCCAACAATAAGGTCAAGAATAGGTACGATCGCAGAGAACATGGAAAAGCTAAATCCAAAGAAATAA
>AONIPM_23685 HTH-type transcriptional regulator RdgA
ATGAAACTAGATACTTTTTCTCAAAGGCTTACATACGCGATGGATCAGGCTGGGTTTACTCAGGCTTCTCTTGGCAATGCTGTTGGCATGTCTCAGCCAAGCGTCTGGAAACTTACGTCTGGA
AAAACACGCAATACGCGCAAACTTTTTGAAATATCAAAAGTGCTTGGAGTTCGTACGGAATGGCTTTCCGATGGAACTGGGCCAATGCGTGATGAGGGAGTTGAACCTTATAATCCAAAATC
TTCTATTCCTCATGAAAGCACGTGGGGATATTTGGACCCATGGGATGGAGGAACGCCTTTAAGAGGTGATGAAGTTGAAATTCCTTACCTTAAAGATATTGAGTTTGCATGCGGGGATGGTC
GGGTGATTGATGAAGATCACAACGGCTTTATGTTGCGCTTCTCCAAATCAACCCTTCGCAGAGTTGGCGCGAACAGTGATGGAAGCGGTGTTGTTTGTTTTCCGGCTCGTGGCAACAGCAT
GGAGCCAAACATTCCTGATGGAACAACAGTTGCTGTTAACACCAACGATAAGAAAATAGTTGACGGAAAGATTTACGCCATTAACGAGAACGGTTGGAAACGCATTAAGATTCTCTTTCGAT
CAGGGCCTGACAAGGTAAGCATTAGAAGCTTTAACTCACTGGAATACCCACAAGAAGAAAAGAATCTAAGCGATATCGAGATCATCGGAAGAATCTTCTGGTGGTCTGTAGTTGACTACTAA
>AONIPM_23690 Transcriptional regulator
ATGGTCAACGAGGCTATTAAAGCGGCTATTGACTCAGTAGGAAGTCAGCAAAAGTTAGCTGATGCCTGCGGTGTTAAGCAGCCGTCTGTATGGGCTTGGTTGCATGGGAAGAAAAGGGTAT
CCGCTGAAAATGCCAAGCGCATTGAAATGGCTACCAATGGAAGCGTCCCTGCATACCTGATTCGCCCTGATTTATCCGCTTTGTTCCCCAATCCGAACAAAGCAGCTTAA
>AONIPM_23695 Bacteriophage CII protein
ATGGAACAAACAAGTTACAGCAAACTATCACAGCGCGATGTTGATCGCGCAGAAACCGATTTACTCATCAACCTGTCAACACTTACCCAGCGCGGTCTGGCAAAGATGATTGGCTGTCATGA
ATCGAAGATAAGCAGAACGGACTGGAGATTTATTGCTTCGGTCTTGTGTGCTTTCGGAATGGCATCAGACATCAGTCCGATTAGCAGGGCTTTTAAGTATGCGCTTGATGAAATCACAAAGA
AAAAATCCCCGGCCGCCACCGAGGATTTTAAGCAAATTGATATGCAATTCTGA
>AONIPM_23700 hypothetical protein
ATGACAAAACGTCGTAAGAAATACCAGGAAAAAGAAGAGATTCGACACCCTGATTCACCTGAGGGATTAGTGGTAGCCGCAGCAAATAACAGGGCGTTCGCAGAGCGCCTTGTTGGTGTT
TACAGACTAGCCAAAGCAGGAGTGAAACATGGGCGTCGTTAA
>AONIPM_23705 Phage-rep-O domain-containing protein
ATGGGCGTCGTTAAGTTAGCTGATTACAGGCCTCAACTGGAGGTCGTGGAGCATCGCGTGGCAGATACCGAAGATGGTTTCATGCGCGTTGCTAACGAGATTACCGACAGTCTGCTGATGG
CTGATTTAACCGTCCGGCAGTTGAAGGTGATGCTCGCTATCATGCGCAAGACATACGGATTCAATAAGCCGATGGATCGACTCACAAACACGCAGATAGCAGCCATGACAGGTATTCATCAC
ACTCATGTTTGCGCTGCCAAGCGCCAGCTTATTGAGCGTAAATTCCTCATTGCTGATGGCGTGAAAATCGGAGTGAACAAGGTGGTTTCTCAGTGGATTAGCCAGGACAGCTTAACATTAGC
TAAAACAGCTAATAAAACATTAGCCGACTCGGCTAATGGGTATAAGCCAAGTCAGCTAAACACAAAAGACAATATACAAAAGACAATAAATACAAATACCCCCTTACCCCCTAACGGGGGTG
GCGATGGGCAGGTTAAACCTGAACGTCGCAAGGCAGAACGAATCGACTACGAATCCTTCCTGAACGCCTACAACACCGAAGTCGGTGACAGACTTCCACACGCTGTTGCGGTCAACGAGA
AACGAAAACGCCGCCTGAAGAAAATCATCCCGCAACTGAAAACGCCAAACGTGGACGGTTTCAGAGCGTATGTCAGGGCGTTTGTGCATCAGGCCAAGCCGTTTTACTTCGGAGACAACG
ACACGGGCTGGACGGCAGATTTTGATTACCTGCTGAGGGAAGATTCGTGTTAA
>AONIPM_23710 hypothetical protein
ATGAGACAGGATATCGAAGCGAGCGTTATCGGTGGCCTGCTGATTGGTGGATTAACTCCAACCGCCAGCGACGTTCTGGCAACTCTGGAGCCGGAAGCGTTTTCAATTCCGCTCTACCGGA
AAGCCTTCGAGGTTATCCGCAAGCAGGCGAGAAACAGAAACCTAATCGACGCGCTGATGGTTGCCGAGGCGTGCGGAGAGGAGCATTTCACGTCAATCCTGATGACCAGTAAGAACTGCC

CGAGTGCCGCAAACCTGAAGGGATATGCCGGAATGGTCGCGGATACTATCACCGCCGCCTGGTGCTGGAAATCATGGATGAAATGCGTGAACCAATTCAGAACGGACCATCGATACATCGA
GTCAGGCGATGGACGAGCTGGTAAAGCGTCTTTCAGCCATCAGAAAGCCACGTGA
>AONIPM_23715 hypothetical protein
ATGGACGAGCTGGTAAAGCGTCTTTCAGCCATCAGAAAGCCACGTGACGAGGTAAAACCTGTACGGTTAGGGGAAATCATCACCGACTACACTGACACGCTTGCAGGCGTCTGA
>AONIPM_23720 hypothetical protein
ATGAACGCGGAAGACCTGGTGATAATCGCTGCTCGTCCTGGTATGGGGAAAACCGAACTGGCGCTGAAGATTGCCGAAGGCGTTGCAAGCCGCGTTATTCCTGGTTCTGACGTCCGGCGC
GGAGTGTTGATTTTCTCGATGGAAATGAGCGCATTGCAGATTGCAGAGCGAAGCATTGCCAACGCCGGGAGGATGTCGGTTAGCGTGCTGCGAAATCCTGCATCGATGGATGACGAAGGC
TGGGCGCGCGTTGCTAACGGCATGAGTCAGCTTGCAGATTTGGATGTATGGGTATCGATGCCTCGCGGTTATCGGTCGAAGAAATACGCTCAATCGCAGAACGGCACAAACAGGAAAATCC
AAACCTGTCACTCATCATGGCGGATTATCTTGGCCTGA
>AONIPM_23725 Phage protein
GTGGCTGAGTTAATTTTCTCTGCATTGATGATTCTCGGTGCCATGTGGATGGTGGCGACGTTCATTGTTGTTGCCAGCAGTTTTGTCCGGTTGGTAGGCGAAGGTAAAGACTCGGTGGGTGT
GCTTTTCGGTAGCATTTTCCTGTGGGTGATTATCGGTGTTATGCCTGTTGTCGTAGCAAAAGTGGCGTGGCGTTTTGTGAGTTGA
>AONIPM_23730 Prophage protein
GTGGCTGACTGGCAAATTCCAATCATCATTCTTGCCGGAGCTTCGCTGGTTGCTGGCTTTATCCTGCTGAAGAAGCATAAAGACCGTGATCAAAAAGTCGAAGTTCTCTATGGGTATCCAGC
GAACAGCACAACATGGCTGACCATTTACCACTACCGAAAATCAGGCCGCTGGGTATTCGAATGGGATGATCTGTTCGCTGAAAAGCGACCAAAGTCATGGGGAGACATCAGCGAATGCATG
ATGTTTGAAGAAAGAAAATCCGGCGCAACCCGAGAAGAGTTTAACGAAGCGTGGGCGCGATTAAGTGAGAGAGGGTATTTGTGA
>AONIPM_23735 hypothetical protein
GTGAGCAAGTACGAAAAATTAGATCAAAACATTCTTTCAATGCTGAGTGAAAGACCAACACCTGTTTTTGATATCTGGCTTAAATGGCGGAGCAATGGAATGTATATCGAAACCATCGATCGT
CGTATGCAATACCTGAGAAAGAAAGGGCTTGTTGCAAATGTGCGTGGGAAGGGTTGGGTGAAAATTAACCTGTCATAA
>AONIPM_23740 hypothetical protein
ATGCTGGCGGATATCGCTCGTCAGGTTCAATGGTGCGGCAAATGGTTAAAACCGGAACAATGGAAGGTTTTGTTAATTAGCGGTCATGCAGTGGCAACAAAACAGGAAGCTGATGTTTTGC
CCGGCCTTGAAGGTGAATACGTCAACATTCGCGAAAGCAGCGCACAGATGAGCGTGAAGCGCATGGCAAGCCTGATTGAGTACACGACAGCATGGGCTATTGGTCAGGGTGTCAGATTTA
CCGACAGGAGGTACGAATGA
>AONIPM_23745 Protein ninE
ATGAGACGACAGCGACGAAGTATCACCGACATCATCTGCGAAAACTGCAAATACCTTCCAACGAAGCGCTCCAGAAATAAACGCAAGCCAATCCCAAAAGAATCTGACGTAAAAACCTTCA
ACTACACGGCTCACCTGTGGGATATCCGGTGGCTAAGACATCGTGCGAGGAAATGA
>AONIPM_23750 DUF2591 domain-containing protein
ATGGATTATTCACAGTTAAGTGATTTTGAAATTAACAAGCGAGTGGCAATTTGCTGTGGATTTGCTCCCGAAGATTGCGAAATCGCAAAGTTGGGAACATCAATCGTTGGTGTTGAGTGGGA
TGACGAAACTGGTTATGCAATAAAAACGGTTGATTACTGTAAAAGCCCATCAGACGCAGAGCCGATTATCGTAGAGAACAGAATTGGCATTATTCCAGCGCCAGAAAATGGATTATGGAAGG
CAGCGCATAGAAAAGTTGGCAGTGATAGTACCCCATATCATATGACTCAAGATGAAAACCCACTCCGCGCTGCCATGATTGTCTTTCTCATGATGCAGGACGCCAATAATGCTTAG
>AONIPM_23755 hypothetical protein
ATGGCTAACTTACGCAAAGAAGCGCGCGGCAGAGAATGCCAGGTACGTATTTATGGCGTATGTATGGCAATCCTGAAACTACAGTCTGGCACATTACCGGATGGCTGGAATTTGCGGAACG
GGATGAAGCCTGACGACCTGATCGGTGCATGGGCTTGTAGCGCGTGTCACGATGAAATCGATCGACGAACCCATATTCTCGACAACAAAGACGCCAGACTTTACCACCTCGAAGGCGTGAT
CAGGACGCAGGCGATACTGCTGAAGGAGGGGAAGATTAAGCCATGA
>AONIPM_00005 hypothetical protein
GTGAGCAAACTGTATGAAATAGCTAATGAATACGCAAAATTGATGGATTCAGATTTAGAACCAGAGATGATTGCTGACACAATAGAAGGCATGGAAGGAGAATTTACCGATAAAATAGAGCA
ACTTCTTGCCATTATTAAAAATGAGTCTGGCTATGCTGAGCGCCTCAAGGAAGAGGCAAAGTCACTGAATGAACGAGCAGCAGTAATTCAAAATAAGATTGACAGCATTATGGCATATATAGC
GTCATCGCTTGAAATGGTTGGCAAGAAAAAGATTCGAGCTGGTATTCACCAGTGA
>AONIPM_00010 hypothetical protein
ATGCCGCACAAATCAGAGGAAGATATCCGCGAAGATGAACGGAAACGCCAGCAGGAAATAATGGATAAAGCACAACTTCTTTGTAATGAAATGGCTCAGGCTGAAAACATGGATGATTTGA
AGCGATATTTTGCAGAAGCATATCGCCTGACATCTGGAATGAAATTGCAGCAGAACGTACAAGCAATTTACGCAGAATGCAAAGCGAAACTGGAGGTTGCCAGTGAGCAAACTGTATGA
>AONIPM_00015 hypothetical protein
ATGAGCAATATCGTTGAATTCGTTAAACAGCAGGAGCACTTATTCTGCGGGGCATTGACTGAACAGACGGTAACATGGGCTAAGGAAAGCCAGTTTGCAATTCAGTATTTCCAGAAAAACG
ATTACCTGGCTAAAACGGCACTGGCAAATCCAACCAGCGCACAGAACGCCATCATCAATGTTGCCGCCATAGGAATCACATTAAACCCGGCGAGCAAACTTGCTTACCTTGTGCCACGTGAT
GGCATGGTTTGCCTTGATATCAGTTACATGGGATTACTTCACCTTGCACAGTCGACAGGCTCAATTAAGTGGGGGCAATGCAAACTGGTGTACTCAAACGACACCTATGAATCAAACGGCCT
TGATTCAGCACCAACCCACAAATACAACGCATTTGGTGAGCGAGGCTCTATTGTTGGTGGTTATTGCACGGTTAAAACAGCAGATGGTGACTACCTGACTGAAGAAATGAGTCTGGCAGAA
ATTAAAGCTGTGGAAGCAACGAGCAAGGCAAAGAATGGACCGTGGAAGACATTCTGGGAAGAGATGGCGCGCAAAACAATAGTTAAAACGCGCCAGCAAATACTGGCCTAA
>AONIPM_00020 Prophage protein
ATGATTCTGACTCTGAATGATAAGCGTGAAATATCGCAAATCATCGCAAGTTTTACCGATGATGATTATGAACGAATTAACAGTGAAGTTGATCGCCTTTGCAAACGTTGCGACCCAATAAGCG
AAATGCTTCGCTCATATAAACCAGATGAGCACACTAAGGACGCTATCGACTGGCTGGAAGATGATGATTGTAACTACCAGGAAAAAGCCGCTGAATGGTTCTGGGATGCAATAACCGATAGA
GTTAAGGCTGAATATGCCTTCGCAATATTCAAACGCAGACACATTTATGGAGAAGCAGCATGA
>AONIPM_00025 hypothetical protein
ATGAGCATCGCTGACGCATGGACAGATGATGCTTTTATCCGATTAATGCAGGACATGCTCAATCAGCAGAAAGAACAGGAGAACGATGATGATTCTGACTCTGAATGA
>AONIPM_00030 hypothetical protein
ATGCAAAGCCAAATAACAATCAATCACCAGAAGTTAATTGCAGCACAAAGCAAGGCTGTTATCGCTCGTTTTCTTGGTGACGGACACATGTGGAAACAAGCTACCGAGGAAATGAAATCAG
CAATCAACTTTCCATGGTACCGCAAGAAATGA
>AONIPM_00035 hypothetical protein
ATGCCAATAAATAAACATAAGGAACTTCCCATGATGCAACTCAGCTTTGCGGGAAGCGGCGTCATGTCCGCTTTTTACCCCGCTGAATCCGAATTATCAAAACGTATTCGTCGCCTTATTCGCT
CTGTCCGTAAGCAACTGGAGGCGTTATGCAAAGCCAAATAA
>AONIPM_00040 hypothetical protein
ATGGACAAGAAAATTAAATCATTGAAGCCAGGAATTGTTATCAGAGACATAAGTGGTGATTACGATACAGAGACATATGATATTTTATATGTGCATGCAGATGGAAAATGTCAGTATTCGAACG
ACATTTTTAATAATAAAGGTGGTGCCGAAATTGCTGCCACCACAGTTAATAAAGAGTTAGTAGCTAACGAGTCGTGGGATTATTTTATGCCATCATCCACTTCTATGAACTGGAAAGAGGTTTT
GTACATTCCATCATAA
>AONIPM_00045 Persistence and stress-resistance toxin PasT
TTGAAGAGTGCTACAGTTTACACGGCAATGAGAGTTAAGCAATATGGCGGCGTACCTTCGGTAGAGGTAAGGTGTGATGATAAAAGAACCAAAGTTGTTACTGATTGCCTTCTGCTTTTTAA
AAGCATAGATGAGGTAATTTTTATTGGCGCTGAAAATCTTCACCCTTTGATAAAAGCAGAGATGAAGCAATGTGCAATCGATGCATTGGGAGTTGGGCAAGGCAAGTCTCAGATCGAAGCA
AAGCAAATGCTTACAGAGCTTGAAAGCAGCAGACGTAGACAAGCAAGCAGGGCAAGACAATTTCACGATGCGATCGCAGGATGTAGCAGAGAGCTTATGTCTTTAAATGTAGACATACAG
AGAGGAATTGATATTCCAACAATAAGGTCAAGAATAGGTACGATCGCAGAGAACATGGAAAAGCTAAATCCAAAGAAATAA
>AONIPM_00050 HTH-type transcriptional regulator RdgA

ATGAAACTAGATACTTTTTCTCAAAGGCTTACATACGCGATGGATCAGGCTGGGTTTACTCAGGCTTCTCTTGGCAATGCTGTTGGCATGTCTCAGCCAAGCGTCTGGAAACTTACGTCTGGA
AAAACACGCAATACGCGCAAACTTTTTGAAATATCAAAAGTGCTTGGAGTTCGTACGGAATGGCTTTCCGATGGAACTGGGCCAATGCGTGATGAGGGAGTTGAACCTTATAATCCAAAATC
TTCTATTCCTCATGAAAGCACGTGGGGATATTTGGACCCATGGGATGGAGGAACGCCTTTAAGAGGTGATGAAGTTGAAATTCCTTACCTTAAAGATATTGAGTTTGCATGCGGGGATGGTC
GGGTGATTGATGAAGATCACAACGGCTTTATGTTGCGCTTCTCCAAATCAACCCTTCGCAGAGTTGGCGCGAACAGTGATGGAAGCGGTGTTGTTTGTTTTCCGGCTCGTGGCAACAGCAT
GGAGCCAAACATTCCTGATGGAACAACAGTTGCTGTTAACACCAACGATAAGAAAATAGTTGACGGAAAGATTTACGCCATTAACGAGAACGGTTGGAAACGCATTAAGATTCTCTTTCGAT
CAGGGCCTGACAAGGTAAGCATTAGAAGCTTTAACTCACTGGAATACCCACAAGAAGAAAAGAATCTAAGCGATATCGAGATCATCGGAAGAATCTTCTGGTGGTCTGTAGTTGACTACTAA
>AONIPM_00055 Transcriptional regulator
ATGGTCAACGAGGCTATTAAAGCGGCTATTGACTCAGTAGGAAGTCAGCAAAAGTTAGCTGATGCCTGCGGTGTTAAGCAGCCGTCTGTATGGGCTTGGTTGCATGGGAAGAAAAGGGTAT
CCGCTGAAAATGCCAAGCGCATTGAAATGGCTACCAATGGAAGCGTCCCTGCATACCTGATTCGCCCTGATTTATCCGCTTTGTTCCCCAATCCGAACAAAGCAGCTTAA
>AONIPM_00060 Bacteriophage CII protein
ATGGAACAAACAAGTTACAGCAAACTATCACAGCGCGATGTTGATCGCGCAGAAACCGATTTACTCATCAACCTGTCAACACTTACCCAGCGCGGTCTGGCAAAGATGATTGGCTGTCATGA
ATCGAAGATAAGCAGAACGGACTGGAGATTTATTGCTTCGGTCTTGTGTGCTTTCGGAATGGCATCAGACATCAGTCCGATTAGCAGGGCTTTTAAGTATGCGCTTGATGAAATCACAAAGA
AAAAATCCCCGGCCGCCACCGAGGATTTTAAGCAAATTGATATGCAATTCTGA
>AONIPM_00065 hypothetical protein
ATGACAAAACGTCGTAAGAAATACCAGGAAAAAGAAGAGATTCGACACCCTGATTCACCTGAGGGATTAGTGGTAGCCGCAGCAAATAACAGGGCGTTCGCAGAGCGCCTTGTTGGTGTT
TACAGACTAGCCAAAGCAGGAGTGAAACATGGGCGTCGTTAA
>AONIPM_00070 hypothetical protein
ATGGGCGTCGTTAAGTTAGCTGATTACAGGCCTCAACTGGAGGTCGTGGAGCATCGCGTGGCAGATACCGAAGATGGTTTCATGCGCGTTGCTAACGAGATTACCGACAGTCTGCTGATGG
CTGATTTAACCGTCCGGCAGGTTTGA
>AONIPM_00075 hypothetical protein
ATGAGACAGGATATCGAAGCGAGCGTTATCGGTGGCCTGCTGATTGGTGGATTAACTCCAACCGCCAGCGACGTTCTGGCAACTCTGGAGCCGGAAGCGTTTTCAATTCCGCTCTACCGGA
AAGCCTTCGAGGTTATCCGCAAGCAGGCGAGAAACAGAAACCTAATCGACGCGCTGATGGTTGCCGAGGCGTGCGGAGAGGAGCATTTCACGTCAATCCTGATGACCAGTAAGAACTGCC
CGAGTGCCGCAAACCTGAAGGGATATGCCGGAATGGTCGCGGATAACTATCACCGCCGCCTGGTGCTGGGAAATCATGGATGA
>AONIPM_00080 hypothetical protein
ATGGAAATGAGCGCATTGCAGATTGCAGAGCGAAGCATTGCCAACGCCGGGAGGATGTCGGTTAGCGTGCTGCGAAATCCTGCATCGATGGATGACGAAGGCTGGGCGCGCGTTGCTAAC
GGCATGAGTCAGCTTGCAGATTTGGATGTATGGGTAGTCGATGCCTCGCGGTTATCGGTCGAAGAAATACGCTCAATCGCAGAACGGCACAAACAGGAAAATCCAAACCTGTCACTCATCAT
GGCGGATTATCTTGGCCTGATTGAGAAGCCGAAAGCAGATCGCAACGACCTTGCAATTGCTCACATCTCCGGAAGCCTGAAGGCGATGGCGAAAGACCTGAAAACGCCTGTTATCTCCCTG
AGTCAGCTTTCGCGCGATGTTGAGAAGCGACCAAACAAACGCCCGACAAACGCAGATTTGCGTGATTCAGGAAGCATTGAACAGGACGCAGACTCAATCATCATGCTCTATCGGGAAGCT
GTATATGACGAGAACAGTAGCGCCGCGCCATTTGCTGAAATCATTGTGACGAAAAACCGTTTTGGCTCGCTTGGTACGGTTTACCAGCGGTTCTGCAACGGACACTTTGTTGCATGTGACCA
GGATGAAGCCAGACAGATTTGCACAGCATCAAATGCACCTGCTGCGCGTGGCAGACGATATGCACAAGGGGCTGACGTATGA
>AONIPM_00085 Phage protein
GTGGCTGAGTTAATTTTCTCTGCATTGATGATTCTCGGTGCCATGTGGATGGTGGCGACGTTCATTGTTGTTGCCAGCAGTTTTGTCCGGTTGGTAGGCGAAGGTAAAGACTCGGTGGGTGT
GCTTTTCGGTAGCATTTTCCTGTGGGTGATTATCGGTGTTATGCCTGTTGTCGTAGCAAAAGTGGCGTGGCGTTTTGTGAGTTGA
>AONIPM_00090 Prophage protein
GTGGCTGACTGGCAAATTCCAATCATCATTCTTGCCGGAGCTTCGCTGGTTGCTGGCTTTATCCTGCTGAAGAAGCATAAAGACCGTGATCAAAAAGTCGAAGTTCTCTATGGGTATCCAGC
GAACAGCACAACATGGCTGACCATTTACCACTACCGAAAATCAGGCCGCTGGGTATTCGAATGGGATGATCTGTTCGCTGAAAAGCGACCAAAGTCATGGGGAGACATCAGCGAATGCATG
ATGTTTGAAGAAAGAAAATCCGGCGCAACCCGAGAAGAGTTTAACGAAGCGTGGGCGCGATTAAGTGAGAGAGGGTATTTGTGA
>AONIPM_00095 hypothetical protein
GTGAGCAAGTACGAAAAATTAGATCAAAACATTCTTTCAATGCTGAGTGAAAGACCAACACCTGTTTTTGATATCTGGCTTAAATGGCGGAGCAATGGAATGTATATCGAAACCATCGATCGT
CGTATGCAATACCTGAGAAAGAAAGGGCTTGTTGCAAATGTGCGTGGGAAGGGTTGGGTGAAAATTAACCTGTCATAA
>AONIPM_00100 Recombination protein NinB
ATGAAGCAAACAATCTTCCTCCGTGGTAAACAACAACAGCAAGCCGCAATCAACGCCATCCTCGCAACACCACTCGATAAAGACAAGCCAGTTACCATCCGCATTACTGACTACAAGCGCAA
CCTTGACCAGAACGCAAAATTTCACGCGATGCTGGCGGATATCGCTCGTCAGGTTCAATGGTGCGGCAAATGGTTAAAACCGGAACAATGGAAGGTTTTGTTAATTAGCGGTCATGCAGTG
GCAACAAAACAGGAAGCTGATGTTTTGCCCGGCCTTGAAGGTGAATACGTCAACATTCGCGAAAGCAGCGCACAGATGAGCGTGAAGCGCATGGCAAGCCTGATTGAGTACACGACAGC
ATGGGCTATTGGTCAGGGTGTCAGATTTACCGACAGGAGGTACGAATGA
>AONIPM_00105 Protein ninE
ATGAGACGACAGCGACGAAGTATCACCGACATCATCTGCGAAAACTGCAAATACCTTCCAACGAAGCGCTCCAGAAATAAACGCAAGCCAATCCCAAAAGAATCTGACGTAAAAACCTTCA
ACTACACGGCTCACCTGTGGGATATCCGGTGGCTAAGACATCGTGCGAGGAAATGA
>AONIPM_00110 DUF2591 domain-containing protein
ATGGATTATTCACAGTTAAGTGATTTTGAAATTAACAAGCGAGTGGCAATTTGCTGTGGATTTGCTCCCGAAGATTGCGAAATCGCAAAGTTGGGAACATCAATCGTTGGTGTTGAGTGGGA
TGACGAAACTGGTTATGCAATAAAAACGGTTGATTACTGTAAAAGCCCATCAGACGCAGAGCCGATTATCGTAGAGAACAGAATTGGCATTATTCCAGCGCCAGAAAATGGATTATGGAAGG
CAGCGCATAGAAAAGTTGGCAGTGATAGTACCCCATATCATATGACTCAAGATGAAAACCCACTCCGCGCTGCCATGATTGTCTTTCTCATGATGCAGGACGCCAATAATGCTTAG
>AONIPM_00115 Protein ninF
ATGCTTAGCCCATCCCAATCCATTCAATACCAGAAAGAAAGCGTCGAGCGGGCTTTAACGTGCGCTAACTGCGGTCAGAAGCTGCATGTGCTGGAAGTTCATGTATGTGAAGCGTGCTGCGC
AGAACTGATGAGCGATCCGAATAGCTCAATGTACGAGGAAGAAGACGATGAGTGA
>AONIPM_00120 Site-specific DNA-methyltransferase (adenine-specific)
ATGAGTGATTCGTTTAGTAGCTCTGATTATTTGTATTTTGTTTTAGTGCCTGTGGCAGAAGTATTCCGCTCTCGATTTCCTGAAGGCTCAGCACCATTTAATGCCATTCGCACTTACTCAAAATG
CAGAGTGAAATTTACAGGTAAGCGGCTGGAAAGAGAGTGGCAGGAATTCTGCAAAAAACATGACCTAAAAAATGACCCTGAACTGGAGTATTAA
>AONIPM_00125 DUF1364 domain-containing protein
ATGGCTAACTTACGCAAAGAAGCGCGCGGCAGAGAATGCCAGGTACGTATTTATGGCGTATGTAATGGCAATCCTGAAACTACAGTTCTGGCACATTACCGGATGGCTGGAATTTGCGGAAC
GGGGATGAAGCCTGACGACCTGATCGGTGCATGGGCTTGTAGCGCGTGTCACGATGAAATCGATCGACGAACCCATATTCTCGACAACAAAGACGCCAGACTTTACCACCTCGAAGGCGTG
ATCAGGACGCAGGCGATACTGCTGAAGGAGGGGAAGATTAAGCCATGA
>AONIPM_00130 Crossover junction endodeoxyribonuclease rusA
ATGAACGAATATCAGTTTGTGCTTCCATACCCGCCGTCGGTGAATACCTACTGGCGAAGACGTGGAAGCCAATATTACATCAGCGATAAAGGCCAGAAATACCGAAAAGACGTTCAGCAAGT
CATCCTCCAACTTAAGTTAGACATTTTCACCAAATCACGACTTCGCATCAAAGTCATCGCAGACGTTCCAGACTCCCGCCGCCGCGACCTCGACAACATCCTGAAAGGTTTACTCGACTCCCT
TATCCACGCCGGATTTGCGGAAGACGACGAGCAATTCGATGACATTCGCGTAATTCGTGGTGTGAAAGTACCAGGCGGACGGCTTGGAATAAAAATCACCGAACTGGAGAACGTATGA
>AONIPM_00135 Protein ninH
ATGAACGCCACAATTCAAACGATACCAGAACTGCTTATACAGACACGAGGCAATCAGACCGAAGTGGCGAGGATGCTTTCCTGCGCAAGAGGAACAGTGCTCAAGTACAACCGAGACAGC
AAAGGCGAGCGTCACGTAATAGTTAACGGCGTCCTGATGGTCAAACAGGGCAAGAGGGGAAGACGATGA
>AONIPM_00140 Antitermination protein Q

ATGAGACTCGAAAGCGTAGCTAAATTTCATTCGCCAAAAAGCCCGATGATGAGCGACTCACCACGGGCTACGGCTTCTGACTCTCTTTCCGGTACTGATGTGATGGCTGCTATGGGGATGGC
GCAATCACAAGCCGGATTCGGAATGGCTGCATTCTGCGGTAAGCATGAACTCAGCCAGAACGACAAACAAAAGGCTATCAACTATCTGATGCAATTTGCACACAAGGTATCGGGGAAATAC
CGTGGTGTGGCAAAGCTCGAAGGAAATACTAAGGCAAAGGTACTGCAAGTGCTCGCAACATTCGCTTATGCGGATTATTGCCGTAGTGCCGCGACGCCGGGTGCAAGATGCAGAGATTGC
CACGGTACAGGCCGTGCGGTTGATATAGCCAAAACAGAGCAGTGGGGGAGAGTTGTTGAGAAAGAGTGCGGAAGATGCAAAGGTGTCGGCTATTCAAGAATGCCAGCAAGCGCCGCATA
TCGCGCTGTAACGATGCTAATCCCAAACCTTACCCAACCCACCTGGTCACGCACTGTTAAGCCGCTGTATGACGCTTTGGTGGTGCAATGCCACAAGGAAGAGTCAATCGCAGACAATATTTT
GAATGCGGTCACGCGTTAA
>AONIPM_00145 Holin
ATGTACCGTATGGACAAAATCAGAGAATGGTTCAGTTACAGCTTCGGAGGACTGACTGCGATGGGTGGCATTCTCTCCCTGAATGACTGGGCTGTAATCATTGGTATTCTTTGTACTGTCGGC
ACATTTGGCATCAACTGGTACTACAAACGCAAAGAGCGTGAGGACAGATTGAATGGCAATGTCACCGGCACTACGAAATAG
>AONIPM_00150 Lysozyme RrrD
ATGGCAATGTCACCGGCACTACGAAATAGCGTAATAGCGGCGATAAGTGGCGGGGCTATTGCCATAGCATCTGTGTTAATCACTGGCCCCGGTGGTAACGATGGTCTGGAGGGTGTCAGATA
CAAACCATATAAGGACGTAGTTGGTGTGTTGACTGTGTGTTATGGCCACACCGGAAAAGACATCATGCTCGGTAAAACGTATACCGAAGCAGAATGCAAAGCCCTCCTGAATAAAGACCTTG
CCACGGTCGCCAGACAAATTAACCCGTACATCAAAGTCGATATACCGGAAACAACGCGCGGCGCTCTTTATTCGTTCGTCTATAACGTGGGCGCAGGCAATTTCAGAACATCGACGCTTCTTC
GCAAAATCAACCAGGGCGATATCAAGGGCGCATGTGACCAGCTACGTCGCTGGACATACGCTGGCGGTAAGCAATGGAAAGGGCTGATGACTCGCCGTGAGATTGAGCGTGAAGTCTGTT
TGTGGGGGCAGCAATGA
>AONIPM_00155 Spanin, inner membrane subunit
ATGAGCAGAGTAACCGCGATTATCTCCGCTCTGGTTATCTGCATCATCGTCTGCCTGTCATGGGCTGTTAATCATTACCGTGATAATGCAATCGCCTACAAAGAGCAGCGCGATAAAAAAGTCA
GTGAACTGAAGCAGGCGACCGCCACCATTACTGACATGCAGCAGCGCCAGCGTGATGCTGATGCACTCGATGCTAAATACACGAAGGAGTTAGCTGATGCGAAAGCTGAAAATGATGCTCT
TCGGCGCAAGCTTGATAATGGTGGTCGGGTGCTCGTCAAAGGAAAATGCCCTGTGCCATCCTCAGCCGAAACCTCCAGCGCCTCCGGCATGGGCAATGATGCCACCGTCGAACTCTCTCCA
GTTGCTGGACGAAACGTTCTCGGTATCCGGGATGGAATCATCAGCGACCAAACAGTACTGAGAACGCTTCAGGAATACATCGTGACGCAATGCCTGAAATAA
>AONIPM_00160 hypothetical protein
ATGCAATTAGTTGAAATCAAGAAGCTCGACTTGGTCACTAACTCCGCTGTAATCGCTACTGGCGTCAAAAAGGATCACAAGCCTGTGATTCAGCTCATCAGGAAGTACAAAAGCGACCTCGA
AGAGTTCGGAAGGGTGGAATTTGAAATGCGACCCTTTCAAACGGATGGGGGCATGCAGAAGCAGGAAATAGCACTGTTAAACGAACAGCAAACCACGCTGTTGATCACATACATGCGAAA
CAATGAAGTTGTGCGTGAATTCAAAAAGCGCCTGGTAGCTGAATTCTTCACTATGCGTAGCGCGCTGGCGAAAAAGAAAATGGATCGCAACTCTGCCTGGAGTACAAACCCATGA
>AONIPM_00165 hypothetical protein
ATGACCGACGCCATCAAACATGAGCGAGAGGCTCAGGGTAAGCAGATCTCCCCGCATCACTTCAGCAACGAAGCTGACCTGATTAACCGACTGGCGCTGGGCATGACGGCGGCCAAGTTC
CGCGTGCATCACGAAATCGGGAAGAAAGAGCCGATCCGCGATTACCTGACGCCGGAACAAATTCACTGCATCACCGAGCTACAGCGCGCCAACACGGTATTCATCAGCATGGGGTGGGAC
TTCGAACAACGCAAAGAAGTGCTGCGCGGCATGTTCGAGCGTAATCATCGTCAGCCGCTTATCGAAGAACAGCACCGCCTGGCGGCCTAA
>AONIPM_00170 Terminase small subunit
ATGACAGGTCTGACAATTAAGCAAGAAGCTTTCTGTCAGGCATACATCGAAACGGGTAATGCTTCTGAGGCTTATCGGACGGCGTATGCTGCTGACAAGATGAAGCCGGAGGCAGTACATG
TTCAAGCATGTAAGTTGCAGGATAACCCTAAGATAGCCCTAAGGATAAAAGAATTGAGGGGCGAGATTAAGCAACGCCATAACGTCACCGTCGATTCTCTCCTCGCTGAACTGGAAGAGGC
CAGACAAAAAGCCTTAAGCGCCGAGACGCCACAATCATCTGCAGCTGTAGCGGCGACAATGGGCAAGGCTAAGCTGGTCGGCCTTGATAAGCAGATTATCGATCACACCTCATCTGATGGA
ACTATGGCAACGAAGCCAACCACTATTCGCCTGGTAGGAGTTGACCCAGCAAATGGAAAGCCAAGTTGA
>AONIPM_00175 PBSX family phage terminase large subunit
ATGGAAAGCCAAGTTGACCTCCAGATACCTGCCAAGTTAGTTCCTGTATTCGCGACAGAAGGAATCCGTTATCGTGGCGCTCACGGCGGACGTGGATCTGCTAAGACGCGTACTTTTGCACT
AATGACTGCCGTCAAAGCGTACCAAGCGGCAGAAGCCAATATCAGTGGAGTTATCCTGTGCGCTCGAGAATACATGAACTCGCTGGAAGAATCCTCCATGGAGGAGGTAAAGCAGGCAATT
CGCTCCGTGGCGTGGCTTGATGATTACTTTGACATTGGAGATAAATACATCAGGACAAAGAACCGCAAGGTCAGCTATGTATTCTGCGGTCTTCGCCATAACCTAGACAGCATCAAATCAAAA
GCGCGAATTCTTGTAGCCTGGGTTGACGAGGCCGAGTCTGTTTCTTCGACTGCGTGGAAAAAGCTTCGCCCGACCGTTCGTGAAGAAGGCTCAGAAATCTGGGTTACGTGGAACCCGGAG
AAGGACGGCAGCGCCACCGACAAACTTTTCAGAAAGAATCCCCCAAAAAGCTCAATTATTGTCGAGATGAACTATGTCGACAATCCATGGTTCCCTGCTGTGCTTGAGGAAGAGCGACAGG
AAGACTTGGCAAACCTTGATTACGCAGATTACGCGTGGATATGGGAGGGGGCTTACCTCGAAAACTCAGATAAGCAGGTGCTGGCTAACAAATACATCGTGCAGAGCTTCGAAGACAATCT
ATGGAGGAAATCAGAGCGCTTGCTGTTCGGTGCTGACTTCGGATTCGCGAAAGACCCCAGCACGCTTATTCGCATGTTCATTCTGGATAACAACCTCTACATCGAATACGAGGCCTACGGCA
ATGGCGTAGAGCTCGACGATATGTGGAAGTTTTATGCAGGCAAAACCGATGCCACGCCGAAACAGCTTGAGGACTGGGAGGTCACTGACGATGCGAAATTCCCCGGTATCCCTGAAGCGC
GTAAATGGCCCATCAAAGCCGACAACTCTAGGCCAGAAACTATCAGCCATATCAAAGGGCAGGGATTCAATATCTCAGCTGCTCAGAAATGGCAGGGTAGCGTAGAGGACGGCATCACCTT
CCTACGTGGATTTAAGAAGATCATCATCCATCCTCGCTGTAAAGAAACGGCGAAAGAGGCGCGGCTTTACTCGTACAAAACAGACCGTATCACTGGCGAAGTCTTGCCGATTATCGAGGATA
AGAACAACCACTGCTGGGATGGAATCCGGTACGGTCTGGACGGGTATATCAAACGCAAACCTAAATCGATGGGGATGATGATTCCTAAGCGCCTTAGGGGGCAATAA
>AONIPM_00180 DUF1073 domain-containing protein
ATGACTGACAAATTAACACTAGCCGTCAATCACGCGCTGAATGACGTCAGGCTTGCTCGCGCCCGTATGGGGTTGATGGTGCCGACGATGGGGTTGGATAATAAGCGCCATTCCGCATGGTG
CGAATACGGCTTCCCTGAGCTGGTCACCTACGAAAACCTCTACTCCCTGTACCGGCGCGGTGGTATTGCCCACGGTGCTGTAGAGAAGCTTGTTGGCAAGTGCTGGCAGACCAACCCGGAA
ATCATTGAGGGTGACGATGCCGACGATAGCGAAGACGAAACCGCCTGGGAGAAAAAGTCCAAACAGGTATTCACCAACCGATTATGGCGCTCATTCGCTGAGGCGGATCGCCGTCGTCTT
GTCGGGCGATATGCCGGAATCCTGCTGCACATCCGAGATGGAAAAGACTGGAATCTGCCTGCCACTAAAGGTCGTGGGCTACAGAAGGTTTCCGTGTCATGGGCTGGGTCGTTGAAGGTT
GGCGAGTGGGACACTGGCATTAACTCGAAGACATACGGCCAGCCGAAGATGTGGAAGTACACCGAACGGTTGCCGAATGGTTCAAGCCGCCGTGTGGATATTCACCCTGACCGAGTATTC
ATCCTGGGGGATTACTCAGAAGATGCAATCGGATTCCTTGAACCAGCATATAACGCCTTTGTCAGCCTGGAGAAGGTGGAAGGTGGTTCAGGTGAGTCGTTCCTGAAGAACGCTGCACGCC
AGCTTAATGTCAACTTCGAAAAGGAAATAGACTTCACAAATCTGGCGTCGCTGTATGGCGTGAGTATCGATGAACTACAGGATAAGTTTAACGAAGTTGCCGGGGAAATTAACCGTGGTAAC
GACGTGCTAATGACCACACAGGGGGCGACAGTTACACCCCTCGTTACATCAGTTGCGGACCCTACAGCAACATACAACGTGAACCTCCAGACAGCAGCCGCAGGGGTAGATATTCCTACGC
GAATTCTGATTGGTAATCAGCAGGCAGAACGTTCAAGTACTGAAGACCAAAAATATTTCAACGCCCGATGTCAGTCTCGCCGCGTAGACCTGTCATTCGAGATAGAGGACTTCTGCGACAAG
CTCATTGAGCTGCAGATTATCGACTCCGTCAGCCAGAAAGCGGTGATATGGGATGACCTGAACGAACAGACCGGTACTGAAAAGCTCACCAACGCCAAGACCATGGGCGAGATTAACCAG
ACCATGCTGGGCAGCGGTGACAACCCGGCGTTTAGCCGTGAGGAAATTCGCACAGCTGCGGGCTATGACAATGATGACGAAGAGCCGTTAGGAGAAGAGGATGGCGACGAAGAGGACA
AGGCCACCGATTCTGCCGCGTAA
>AONIPM_00185 Phage-Mu-F domain-containing protein
ATGGCGACGAAGAGGACAAGGCCACCGATTCTGCCGCGTAACTATCAGGATCCGACCGGAGCCGATGCGCTGGAACGCCGGGCAATGAAAGACTTCTCCAGGCGGATGAATAAGATTGG
CAAAGCGTACAAATCAGCACTCGACAAAATACCTTCCTCCCTCGCAGTAAACGCCAGATATGAATACCAGCTAAACCCAACGCTGCTCTCCATCATCCTGAACGATGCCAGTTACCTGGTAGA
CCAGGTGCTGCTTGATGGTGACGAATACGACCTGTGGTTTTACGAGTACATCGACCTGGCTGCTGAGAAAGGGACAGGCCAAGCCTTCTACAACCTCAGCAAGCAATCACCTGTGTATGCA
GCAGGACGCGAGTCACTGGCGGCAATCCTCGCGAGTGACCCGTATCAGCAACGTATGGCGCTGGTACATGCGCGTGTGTTTGAGGAAATGAAAGGGCTGAGTGCTGATGTTAAGCGCGAC
ATGGCACGAGTGCTGACTGATGGCGTGGGGCGAGGTCTTAATCCCCGTGACATTGCTCGTAACCTGACTGCACAGGCTGGCATAGAGAAGCGCCGGGCGAACAGGATAGCGCGCACTGA
AGTGACTACCGCGCTGCGTCGAGCCAAGTGGGATGAAGACCAGGAGGCGAATGACCTCTTCGGCCTGAAAACGCTTCTGGTTCACATCTCGGCGCTGTCACCGACAACGCGACACACCCA
CGCCGTGCGCCATGCTCACCTCTACACAAATGAAGAGGTTCGTGAATGGTATGCAAAGGATGCGAACAGTATCAACTGCAAATGCAGCCAACAGTCAGTTCTGGTTGACGGCGATGGAAGG
CCTCAGTTCCCAGACGCCATTACCAAACTCAAACAGGAATATAAATCGATGCAGGCGCGTGGTTACGCCTGGGCGGAGAAATAA
>AONIPM_00190 Gp7
ATGCCAATTCAGATTCACGTCAACACAAAGGTAAACAGCCAGACCATCCGCCGTGAGATTTATAACGGGCGAGAGCATGTCGTTATTCCAAGTTACACGCTTCCTGCCAACGTGATTATGAAC
AGAGAGTTCTATCCAGAGGCAGAGATAACCGCGAACTATCAAAGCATGGAAGGGACAATAGCACCGCTAGGTCATCCAACAGTTGATGGGAGGAATGTTTCTGCATTCTCACCAGAAGGG

TTATGCACCAATTTCATCGGAGCGTGGAATCGCAATGTGAGCCTGAAAGGGAATCGCGTTTATTCAGAAAAGTGGGTAGACGTTGAGAGAGCGATGCAATCTCCAGGCGGTCAGCGTCTG
ATGGAGCGTATTTCGTCTTTGGAAAGCGGTGATTCATCGGAGCCTATCTGGTCAAGCGTTGCAGTGTACCGAGAGCAGATTCCTGCACCAGAAGAACTGAAAAAGCAGGGTGCTGATTGG
GTAGTAAAGATTCATTCAATTGATCATGACGCAATTCTTCTCGACGAACCACCGGCAGCCGGACCAGAAAAGGGCGTCGGCCTAATGGTGAACGCAGACCAGGCAATATCGCTACAGCCTA
ATAGTGGGGCGTTGATTGGCGAGTCCTACCGGGAGCGTGAACAACGCCTTGACCGTGCAGCCAAAGCAAAGTTCGCACCAGGTGAAAACGAATACGCCTGGGTAGCTGATTTCACTGACT
CGCAGGCGGTAATCGTCAGAAACGGCGGAGGCGCTGAGGTATTTGGCTACAAATCCGATGGCGGCGTAATCACATTCGACGATACCGGAACGGCAGTCGCACGACAGGAATCATGGGTG
GCTATCGTCGCCAACAAATTCAAATCTCTTTTCACACCGCAGGAGCAGCCTGCAACAAACCACAAAACGGAGGGCGACATGCCTTTAACCAAAGAAGAACTGGAACAACTCGGCAGCATG
GTTAGCGAGGCCGTTGCCACCAATACCGAAAAAGCGATCAAGCCTCTCGCAGAAAAGGTAGATGCGTTGCAGGCCAACCAGCAGCAATTGGCTGAAACTCTGACTGCCAACTCCCGCGCA
GAAGAGAAAGCCAAGCGTGAAGCGGTGGCGAAAGTTCACGGCGAGATCGTGGCTAACGCTCTTTCAGGCGAAGCACTGGATGCGATGTTTAAAACAATCGGTGAATCCGCGCCGCTGGG
TACCAACTCTGCGCAACAGCAGAAAGAAACCGGTGCACCTGCCGCATCTGAATACTTCAAATAA
>AONIPM_00195 Capsid decoration protein
ATGTCACGTTATCGTCGCGTTAATATCGACGGGAAATCGCTCTACAAGACCGAAACCCGTCTCACCGCCGCAGAACTACTGCCAGGCACCGCCGTCACTATTAACGGTGATGGTAAGTTCGC
ACAAGCCACTGCATTAACTGGCCGCATGTACATTATCGATTGTGCTTATCATCAGGGGCTTGGCATTCGTGATGCCGTTCCTGCTGGCGATTCTGCTGTTGGCAACTATGTCGAAGAAGGTCG
TGAACTTGCGCTTCTGTGTGTACCTGGTGCGTACAAGAAGGACAGCCCGATTAAGCTTGGCGCGGCTGGTCAATTCACACTGGCAACTGGCGACACCGATTCAGTAATCGGCTACAGCCAG
GACGAGTTCACCATCGCAGCCAGCACCACCGACTTCATTCGCGTTCGTATGCGCGTTGGCACTGCTGCCGCTGCAGGCGCGTAA
>AONIPM_00200 Phage coat protein
ATGTATTTCTCTAAAGAGACACTGGCGACTAACTCGCGCCTTGGTGGTCACTGGAATGAGCTTTGGGCAAACCGAAACATGTGGAACGCACAGCATGATGCTATGATTGCGGCAAATCGTTC
TAATATGACTCCTGAATGGCTGGCGGTTAATGCTGTAGGCGGTTTTACGCGTGATTTCTGGGCCGAGATTGACCGCCAAGTGCTGCAATTGCGTGATCAGGAAGTTGGCATGGAAATTGTCA
ACGACCTGATTGGTGTTCAGACGGTGCTGTCTGTAGGCAAAACCGCAAAGCTTTACAACGTTGTTGGCGACATTGCCGATGATGTGTCTGTGAGCATTGACGGTCAGGCTCCATTCTCATTT
GACCATACCGAATATGCGAGTGATGGCGATCCGATCCCGGTGTTCACTGCAGGTTACGGTGTGAACTGGCGTCATGCTGCTGGTCTTAATTCTGTGGGTATCGATCTGGTACTTGACTCGCAG
ATGGCGAAAATGCGCAAGTTCAACCAGAAGCGTGTTAACTACTACCTGAACGGCGACCCGAATATCAAGGTGCAGTCATACCCGGCGCAGGGTATCAAAAACCACCGCAACACCAAGAAG
ATTAATCTTGGCTCTGGGGCAGGCGGCGCAAACATCGATCTGACCACTGCAACCATGACTCAGTTGTTTGAGTTCTTCGGTAAAGGTGCATTCGGCACCCTGGCCCGTGCGAATAAGGTCG
CGGCATACGATGTGATGTGGGTATCGCCAGAAATCTGGGCTAACCTGGCTCAACCGTATGTAGTTAATGGCGTAGTTAGCGGCAATGTGCTGAATGCCGTGCTCCCATTTGCACCGGTACTTG
AAATCCGTCCTACCTTCGCGCTGAGTGGTAACGAGTTCATCGCCTATGTTCGCCGTCAGGATGTTATTTCCCCGCTGGTTGGCATGGCTGTTGGTGTTGTTCCTCTGCCGCGTCCACTGCCTAA
CGTTAACTACAACTTCCAGATTATGTCTGCTGAAGGTCTGCAAATTACCGCTGACGAGCAAGGACTTTCTGGTGTTGTCTACGGCGCTAACCTGGCGTAA
>AONIPM_00205 FluMu-N domain-containing protein
ATGGCTAAATACGAAGTAGTGCGTCCGTGGTTCGGCGTAAAGGTCGGCGATGTGGTGGAGTTAAAAGAGCTTCACCCGGCACTTAAGTCGAACGTTCGCCTGATGCGTGGCGAAGCTGGC
GGCGAGCTAAGACCGGCGACACCAGATGCTGGAACAGATGCCAAATCGCGCAAAGAGATTATCGCGGCACGACTGACGGAACTGGGCATTGAGTTTAAAGGCAATCTGGGAGCTGAAAA
GCTCAGTGAGCTGTTGCCAGATGGCGAACTCGAAAAGCTTTTCCCTGCTGAATAA
>AONIPM_00210 hypothetical protein
ATGAACCAGGAAGATATTAATCAACCACTGCCATATTCGCAGTTTTCTGAGATTTCATCGTTGAGCGTCACCAATGAGATCAGTCTGGCGATATCCAGCACCGCCGAACTGGTTAAAGACTCG
TCGAGCGTGGAACGGCTGATTCTCCTGAAGCATCTTCGCGCGCTTTGCGAACTGCAACTCAATAAATTATCAGGGCTGGAATAG
>AONIPM_00215 Gp11
ATGGTAACGCTGGAGAAGGCCAAAGAGTATTTGCAGAGCCAGGGAATAAATCTTCCTGACTTCATGCTTCAGGCGTTAGTGGACGATGTTAACAGCATTCAGGAATGCCTCGATGCGCATTA
CCCGGCATCAAAGGCGCTGGCAATCCAGATGTATTTGCTCGCATTGATGGGCCTGGCGCAAGGCGACAAGTATATCAGCTCACAGACTGGCCCTAATGGTGCATCTCGTTCATTCCGTTTTCA
GTCGTTTCCGGATCGCTGGAAAGGGGCGCTGGCACTGTTGCGCGTCACCGATAAACACGGCTGCGCTAATGACCTCATCCCTCCAGACCCGACCAATACAGCTTTTGCTGGCATATGGATTG
CCAGGGGTGGATGCATGTGTGGCGGGGGGCGGTGA
>AONIPM_00220 DUF551 domain-containing protein
ATGCATGTGTGGCGGGGGGCGGTGATGGGGTGGATATCGGTTAAGAAGCGGCTTCCGGAGCCTTTTGTCAAAGTCTGGGTGATGACCGACAGTGGTAAACGCGTTACCGGATACGTCAAA
AGCAACGGTGACTGGTATCTGCTGTGCCGGAAGGTTGCGGCGGAGAATCCGGAGGTGATCCGGTGGGAGGATAACGGTGTCTGA
>AONIPM_00225 Tail fiber protein
GTGTCTGAAACAGCTGCATGGAGCTATACCAATGTTGCCACTGTTTACCCGCGAGTCTACGACGACTGGAACAGCACCTGGACAACCGGAACCCCCTACCTGCTTGACTGCACCTGGACGG
CAAACAATGAGGTTGCGGTAGATGCCAGCGGGAAAGAGTTCACCACGAACCTGATTTTCTTCACTGAACTGAAGCGCAATGGCATCGATGCGACCATGCCGAAGCGTGACTGGTATATCGC
CAGAGGTGACACAACGGCACAGGCCGATCCGCTGAAAGCTGGTGCAAACGTCATCAAGGCGGTGACGGAATGGGATATGTCACCATTCGGCGAGGAGCCGGACTACAAAATTCTGACGT
GA
>AONIPM_00230 HK97 gp10 family phage protein
ATGCCCGTAAAAGGTCTCAAGCGTGTTCAAATGAACACCCGCAAGGTGCTGGCAGAAATTGCCGGGCCACGCACAGAAAGAGTGCTGACTGAGGTCATGATTGTCGGATCGTCTCACGCT
GCGCTACTTACTCCCATTGACACATCCACGCTTATCAACAGCCAGTACAGAAAGCTTGAGCCAATGCCAGGCGGGATGCAGGGAAAGGTCGGTTACACGGCTGCATACGCTGCCGCCGTTC
ACGGTATGTCCGGTAAGCTAAAAGGTCAGCCGCGTGGACACTTCGGAAGAACTCGCGCTGGAAAAGAATTCGGCGGCGGCACCGGGAAGGGGAATTACTGGGATCCCGATGCCGAGCC
GGGGTTCCTGACCAAAGGCTTTGAGCGTGACGGTCTCAACGAGATTAAGGCCATCATCAAGCAAGGGTACAAAGTATGA
>AONIPM_00235 Phage protein
ATGACGCGTAGCGAAGTGTATGACGCGCTGAGAGCGTGGTTGAAGTCGCATGGCTTTGATGTTGGTTATCGCGTCCAGAAGCGATTCTGGAATGAGTTGGAGAATACCGAGGGGGAAAGA
TACCTTGTCATCCAGCAGAACGGTGGCGGCAAGCCAGAAGAAGCGATAACCCGCGATTATTTCCGCATCCTCCTCCTGTCAGGTCAGAACGACAGCGATATTAACCAGGTTGAAGACCGCG
CCGACGCCATCCGCCAGGCGATGATCGACGACTACAAAACCGAATGCATCATTTCGATGCAGCCAATAGGCGGCATCACCGCCATCCAGACCGAAGAAGGCCGTTACCTCTTCGAGATTTCC
TTTCAAACCATCATTTCCAGATAA
>AONIPM_00240 Ig-like domain-containing protein
ATGGCCGGATGTGAGTCAGGTGCATTCACAGGGCGCGATGTCGTTGTTTATTACGCGATTGGCTGCCCGGAAGTACAACCTACCGCCAGCGCTTACCAGCGACTCGGCATGATGCGCGGTA
AAACAGTTAATGCAGAGTGGGAAACCGCAGATGCAACTGCCGATATGAGCGCCGCGTTTACGCAGGAAAATCTTGTTACCTATAAGAACATTTCGTTCTCAGGTGACGGCGTTACCCGCAA
AGAAGATGTTTATGCGCAGAACGCGCTTAAACGCCACGTCTACAACCCACCCGCAGAGACCAGTAACCAGCCGTATGTCTGGCTGAAGATCATCTCTCCAAACGATATTACTGAAGGGCCGT
TCATGGTGACCTCATGGCAGGATGAAGCGCCGCACGATGACGTTGCCACCTGGTCTATTGAGGCATCCAGTGCTGGTAGTGTGGACGTGCGCGACGTTGGTGCGGTCATTACTATCACCGCT
CAACCGCAGAATCGTACGCTGACCGTTGGTGATGCGCTGAATCTGTCAGTATCCGCAACGGTTTCTGATAGTTCTGCGCTGGCTTACCAGTGGAAGCATGATGGCGAAGATATCAGTGGCGC
CACGTCTCCCACTTACACCAAAGCGAGTGTTACAGAAGATGATGCCGGGGCTTATACCTGTCAGATCACTTCGACCACCGCTGGCAGTCTGACGTCTGGCGCGGCGACGGTTATCGTCAAC
GCAGAGTAA
>AONIPM_00245 Phage protein
ATGAAAGCAATAACCGATATCGGCCAGGCTGTCATTCGGGCCGGTAGCAAAGAGATATTTCTCAATCCGTCATTCCTCGCTATGTCGCGCATCGGCTCACCGGAACAGATTGTTGATGCTTTC
GTGAAAGTTCATGCAGGCCATTACCCAAAGCACAGAATTGCGGACCCGCAGATACTCAAAGCGGCTAATGCACGCTGCTTTGCAGACATGGCAGAAGTTGCAGCTAAAGTGGTCAGGCAT
TGCTCTGAAGGCGACGTTGCTGAAATCATCGGCTCTTACTCAGTGACTACGGCGGGACGCCTCCTGTTCAAGCCTGGCTACTTACCCATTGAAGACGTTATCCAACTGGCGCGCCACCTGAT
TCTGCATGGCGTGATGGGGGATCAGCCGCCCGAGGAGTTCGAGAGCAAGAAGGGGGAGTACAGCGATAAATTCGATGTACGGTCATTCGTCTACACCGCTGTTGCTCACCTCGGCATGAG
CGAGTCAGACGCCTGGAACATGACAATGACCAGCTTCCGCGCCGCCATGAACGCTAAATTTCCGCAGAAAGAGAAAGCCAGAGTGCCGACTCAGGAAAAATACGACGAAGTCATGGACT
GGGCAGAACAGATGCTGGCGATCGACGCGCAACGGAACGGGCCGCATTAA
>AONIPM_00250 KilA-N domain-containing protein

ATGCACTATCCAACCGTATCTGTAAACGGCGTATCCGTTCGTGTCGATAACGAAGGGCGATACAGTCTCAACGATCTTCATGCTGCTGCGGTAGCAAACGGAGAGGCCACCGAGTCACAAAG
ACCCAGCGTCTTTCTCCGCAGCGCCCAGATCAAACGTTTCGTAAAAGCCCTCAAATCCAAAGCACTAAAAAGTGCTTCGGAACAAAATCAACCGCTTAGGGTAATAAAAGGTGGTGATCAA
AGTGGGGCATGGGGCGTTGAACTTCTGGCTATTCGCTACGCGGCCTGGATTAAGCCCGAATTTGAAATTGAGGTATACGAGGTTTTCAGAACAGTAGTTCGCCTCGGCATTAATGCCATGTC
TCGCCTCAACAAAATCGACCATGTGATTAACACCGAAACCAAAGCGATTAGCCAGTGCGCCAGTCAGATGGCGAAGTGGGGAGTTGGTGGTCGCAAAAAGCTACTCCATGCAGCGCGCGA
AAGGGTTGCTGATGAAGTGCAGATGTATTTGCCTGGCATTGTGTAG
>AONIPM_00255 hypothetical protein
ATGGTTAATAAAAAAATAGATGATAATATTTATTTTGGAGACTTCCCTAAACATGGTGGCGGCGGAATCGGAGGTGGTGAAATGCTCGAAGTACGAGTGGCTAAGCTTGAATCCAACGTTGA
GGATATCAAAGCAAACCTGTCTGAGGCGCGGGTTGACATTCGAGACCTTCGTAACACGTCATCAGGAACAAGTAGAGATGTGGCGGTAATTCTTCAGAAACAGTTAGATATTGACGAAAAA
CTATCAAAAAAAACCAGTATCAGCGATATGGACAGAACCATATCAACTGCCGCAAACAAGCAAATAATCTGGACGGTTTCTGTCATGGTAGGAATTGCGGGCCTATCAATGGCTGTAGCTAAG
CTCATTTTCTAA
>AONIPM_00260 hypothetical protein
ATGAAGAAGGTAGTTGCTTTAGCTCTCGGGACTTTAATGCTGTCTGGCTGTACTGTTCGCGTTGCTGATATGACCGTAGGTAGTACCAAAAACTACAACCTGAACGCAGCTAAGTTTGAAAA
AGGGCAGCGCGTAACTGGTGAAGACAAAGCTCCGATTGTCATTTTCCCGCTGGGCATTCCTAGCGTTAAAACAGCAATGGATCGCGCTATTGAGAAAGATAAGTGCGCTGTTGGTCTGAGT
GATGTGGTCATTTACCAGCTCAACCATGCCTTCCTGTTCGGAACCTACGGTTTCCGAGTAGAAGGTACGCAGATCATTGATAAATCTCAGATGGGTTGCGAAAGCCGCTAA
>AONIPM_00265 TMP-3 domain-containing protein
ATGGCCGTGAACGTAGGTGATATTGAATATGTAATTAAGGCTGATACTGCTCAGCTGCTGCGCGCAGATAAGAAGGTTACATCTGTAACCAATAACATGGATACTGGGTTTAAGAAGGCTGAT
AATTCTGCGTCAAAGCTGTCTACGACAATAAGCAAGCTGTCTGCCGCCATTTCTGTTGCGTTAATTGTTGAGTGGGGGAAGAAGTTCCTTGAGCTTGCGGACAACATGACACAACTCCAGG
CAAGGATAGCAAGGCTTTCAACGGATGCCTCCACAGCAAAAGAAACGTTTCAGTCGTTGACGCAAATATCGTCAAAAACAGGCGCCAGTCTGTCGGATACAACCAAACTGTGGGAAACAT
TGACATCGTCTCTTAAAGAAGCTGGCGCATCGAATGCTCAGGTATTAAATATTACCGATACGCTGCAAAAAATTGGTCGCATAGGCGGAAACTCGACAGAAGAAATGTCGAATGCTCTTCGC
CAGTTTGGGCAGTCAATTGCGTCAGGCACTGTCAGAGCAGAAGAATTTAACTCAATCCTCGAACAGATGCCTGAGTTAGCAAGACAGATTGCTGCTGGCATGGGTATTAGCATGGGTGAGT
TGCGTCAGCGAATGCTCAATGGAAAGTTGACAGCGCAAGATGCTCTTAATGCTATACAGGACAGAACGACAGTTGTTAACGAGGAATTTAATAAGCTTCCACGTTCATTATCACAAGCTACC
GGCTCTCTCGAAACATCATTTGCAAAACTTGTCTCCTCAATAAATGACGCAACTGGAGCATCAAGTGCAGCAGTTACTGTCATTGATGGATTGGCAAAATACATCGAGTTACTCGGAGACTCG
TCAACATCAACTGCAGATAAACTATGGTCTCTCGCTGGTGCTATTTCAAAACTTAATCCTGGATATATTGGAAAACTTTTAGCTGACGGGCTTATAGGAGATGAACCTCCAAAACAAGTCGATG
TATTAAGCGAGAAAATTGCCAATCTTACCGGGGATTTAAAAGGGCTTTATGGGCAGGCTGAAAAAACAGCGCATGTGGAGATACCTCAGACCGATAACAATAAGGGAGGAAAAGGAGGTG
GTGGGAAGTCCACTAAGAAACAGGCTGATGAAGCAACAGAGTCTCTCGCCAGACAGCAAGCCGCACTCGATCGCCTGAACACTGGTTACGCCGATGGCTCTCTCGAATTAGCGAAATACG
ACGCTGTAGTTGCTCTTGGAAATAAAGCATCAGGAGAGCAGATTGCCAAAGCTGAACAGCAAGCGGAATCCATATGGAAAATACAGCAGGCAACCAAAGCGGCGGCGGAAGAGGAAAG
GAAGCGCACACAGGCGGGTCAAAACTTTACCGGGCTACAGGGGCAGGTATCACCAGTTGCCGCAGTAGATAACATCTACGCACAGCAAATGGCGCAGCTTGACGAGTATGTGCAACTCTA
CCCACAAAAAATTGCAGAAGCAGAAGCCGTCCGTGCAGGAATTGAAGATCAGTATCATCAGAAACGCATGGCCGCAATGTGGGAGGAATGGCAGCAGCAAAGCGAGATTAATAGCATGCT
TGGCGCGGCTGTAGATTCGCTTCAGGGCGGAGCTACCAATGCCATAACCGGGCTGATTAACGGCACTCAAAGCCTGCAGGAGTCATTCGCAAATATTGGTTCGACAATACTCAACAGCGTT
GTAAGCGCCATTGTGGATATGGGAGTTCAGTATGTTAAGAGCCTGATTATAGGTAAGGCCATGTCATCTGCTGCAACTGCCGCACAGATTGCTGAGGCTGGCGCTCTTGCAACAGCTTGGGC
TCCTGCGGCTATGGCAGCATCTATTGCGACCCAGGGCAAAGCATCTGCTATCGGTTTGGCTGCCTATAGTTCTTCCATGGCGGCAGGGCAGGCGCTTTCTATTGCTGGCGCTCGCCGTTACG
GCGGCACAGTATCAGCTGGCAACGCCTACCGCATCAACGAAGATGGACGCTCTGAAATCTTCCAGACTGCAGGTGGGCAGCAGGCATTCATCCCGAACCAGTCAGGGAAGGTGATATCTG
CTGATAAGGCGGGTGGGGGCGGAGTTCAAAACGTTTACTTCACCATTAACACTACAGGTGGAATAAGTGATGCTGAGTGGGCTCAAATCGAGGCTAAAGCAATTAACATCAGCAAGAAAA
TGGCGCTTTTCCAAATCAGTGACCAGGCCAACCGCCCAAATGGTATGATACAACCGCGTAGGAAATAA
>AONIPM_00270 hypothetical protein
GTGGAGATTGAAGTAACGAATATCACGGCAGCAGACAACGAGGTAGCTACGGGAATTAACGCTACTGTTACCTTTATTGATTACGAGAATAATAGTGGTGAAATTGTTGTTTACGTAAAGCT
GCCTTTAGAAAAGCAACTATCAATTTCTGATGTTGAGGAAAAGGCTCAGGAATTGGCGAAAAATAAATTGAAAGCGCTTGTGGCTGGCTTTTAA
>AONIPM_00275 Phage tail protein
ATGCCAGAAACATTCACATGGACACCGCAGCGAGCTTATCAGGTAGAACGTACCCCAAACGTAGCCGTTGTTAAGCTCGGTGATGGCTACGAGCAGCGACAGGTGAAGGGTATCAATCCGT
TAATGGATAAATATTCGCTCACCTTTCGCGGAGTCAGCGGCGCTTGCCGCAGCAACCCGGCAAAGGATGCTGAGGCATTTCTCAAGGCTCGAATGGCGGTAGAGTCATTCTACTGGACTCC
ATCCGATACGGGAGTGCAGGCATTGTTTGTCTGCCGCTCCTGGAATATGACAAAAACCGGGCCGCTGTTTGAACTGACGGCCACGTTTGAACAGGTGCCAAGATGA
>AONIPM_00280 Phage protein
ATGATTAAATTGCTGCATGGAGCTGTGATTTGCAGGCCTTTCGCAAAGCAACCTGTGCCAGGTGAAAAAGAAAAGAAGCAACGTGAAATTGATGATGTTGAAATGTGTTGCCGATTCGAGG
CGGAAGACTGGGATGCTCCAGACTTTAAAGCGGTTTCGGGGCCTCATAACTGGCGAAGCTACATTACGCCACAGCTCAAAGACGCATGGCCATCATTCACTGACTGGCAAAAGAAAGTGAT
AGCTCATGCGCTGGATGATGCTGCATCACACGAAGAATGGGATTAA
>AONIPM_00285 Minor tail protein L
GTGCGCGACATACCAGCAAATTTGATTATCGACAGCGTGGACGCCGGAGTTGGCGCATTTATCGACCTGTTCGAAGCTGACCTGCAACCATTTGGTGGTGACCTTATCCGTTTCCATTCCGG
CACAAACGGCTATTACGGCAATGTTATCTGGAAAGGTAACCAGTACCAGGCGTACCCGATAGCGGTTGAAGGATTCGAGTCAAAGAACGAAGGCACATATGCCCGCCCATCAATGTCGGTG
GCGAACGTTACCGGCCTGCTGACCGGGATTAACCATGACTTTGACGACATGCTGGGCGTGGTTATTACCCGGCGTCAGGTTCCGGTGAAATATCTGGACGCGGTGAATTTTCCGAACGGCA
ATCCTGATGCAGATCCGACACAGGAAGCCGTTTCCCGCTACGTTGTGGAAGAGATGACCGAAGAGACATTTGAGCAGGTCACTTACACGCTGGCGACGCCGATTGACTGCGATAACGCCAT
CATCCCGGCCAGAACTATCCTTGCAGACGTATGTCAGTGGCAGTATCGCGGCGTCGGGTGTGGATATGACGGCCCCCCTGTTGCAGACGAGCGCGACAACCCAACCACTGACCCGGCGAA
GGACAAATGCTCGCACCGTCGTACTGGTTGCCGGTTCCGCTATCCACGGCCTGAACCAATGCCAATAAGCAGTTTCCCCGGCTCTCAAAAGGTCTCCTGA
>AONIPM_00290 NlpC/P60 domain-containing protein
ATGCAAGAATTACTCGATTATGCGGCATCGTCGCAGGATGAAGTATGCGCGCTGATCCTGAATGGCGAGCGAGTGTTCCGCTGTAGGAATGTGCATCCAGATCCCTGGCATCACTTCCGCATA
AGTGACGACGACTGGCTTGCGGCGGAGGAAGAAGGGGAGATTATCGCAGTATTCCATTCGCATCCGCAAAGCCAGCCAGCGCTTTCTGGTGCTGACCGGCAGATGCAGGTAATGACGGG
GCTGCCATGGTGGCTTGCATCTGGCGGGAAACTGAGGAAATACAGGCCTGTGCCACTCCTGCTGGGGCGCAGGTTCGACCACGGCATTATGGATTGCTACACGCTTTTCCGGGACGCATAT
CATCTTTGCGGCATTGACCTGCCTGATTTTGAGCGCACTAACGGATGGTGGTTGCGCGGTGAAAACCTCTATCTGAGCAACATGCCGCTCAATGGATTCCGCCAGGTATCGCCGGGAGAGG
CACAACCGGGCGACGTCATTATCAGGCAGCCATTCCCGGGCGCCGACCCATGCCACTCAATGATTCTCCTTCATGACAACCTGGTGCTTCACCATGACCATGCAGGACACCTCAGCCGACGA
GAACAAATGCGCCCGGCATACATCAAGCAGACGCATTCAATCTGGAGACATGAACAGTGCTCATCTTTAAATTTGCAGGCAATTTACGCCGATTTTACCGCCAGATATCCCTGA
>AONIPM_00295 Tail assembly protein
GTGCTCATCTTTAAATTTGCAGGCAATTTACGCCGATTTTACCGCCAGATATCCCTGAATGTTGATACGCCAGCGCAGGGGTTGCGCCTTCTGCTGGCGCAGGATTTTGAATTTAAAAAAGCC
TTTCTCAACACAAAGCTGCGGGTTCGGGTGGCTGGCGAGGATGTCGAGGCATCTGCGATGCAATGGCATCTTGATCGCCACCTTAAAGACGGCTCGGTGGTCCTGTTTGTACCAATAGTGG
AGGGGGCGATCACCGCCGCCGCTGCCGCATGGATTGCGGTTGCCGTCAGCGTGGCTTCAATTGCGTACTCGGTCTACATGTCCCGCAACATGAAGACCAAAACCTCCGCAGAAGCCGCAG
AGACCAACACACTCACAAACAACTCATTTACCAGTGCTGAGAACAGAGTTGGGCAGGGTAGACCGGTGCCAATACTACTCGGTGAGATGGAAGTTGGCTCAAATGTTATTTCTCTTGGTATC
GATACCGCGAATAATGTTGATTGGGATGCTTCGATAGGGTGA
>AONIPM_00300 HNHc domain-containing protein
ATGGCTGAGCTAAGTCTACATGATTTATTTGCCTATGACGAAACGTCACCCACCTGCCTGATTTGGAAGATATCTCCTTCACGGCGCACAAAATGTGGTCACCAAGCCGGCACAATTAACAAG
GTGTCCGGTAAATACCCTCGAAACTATTACCGAGCAAGAGTGAATGGCGGGTATCAAAGCGTGCATCGCATAGTTTGGTTCCTGCATCACGGGGAAGTTCCCGAAGGGTTGGCAATCGATC
ATGTTGATGGAAATACATTAAACAACAAGATCACGAACCTCCGATTGGTAACACCATCCAAAAATGCCCGAAATTGCCGAAGGCAAAAGAATAATATTTCTGGTCAGACTGGAGTAAGGTTG

GCTATGGATAAAGGGAGATTTCCCTTCTATGAGGCCTATGTTCACATTGATGGCAAGCAGGTCCGTCGCCAATTTTATCCAAAAGGCGGAACACTTGAAGAGGCTAGGTCCCGAGCGGTTG
ACTGGCGAAAAGAGCAGATAAATCGACTTAACGAACATGGTGCCGGATACACCGAGCGCCACGGCAAATAA
>AONIPM_00305 Fibronectin type-III domain-containing protein
ATGTCTTCAGGCGGCGGTAAAGCATCAACCCCCAAACTCCTCGACGATAACCTCAAATCAAAACAATTTTACAGGGTGCTCGATCTTATTTCGGAAGGTCCAATATACGGGCCGGTAGACCA
GGAACACCTTTCTTCATTCAAACTGAACAAAACGTCTGTTACCGATGCAAACGGAAACGTCAGTGTGAATGGCATCAGTGTGGCATGGCGGCCTGGCTCAGAGAGTCAGTTACCGATTAAC
GGTTTCTCTGCAATTGAAGCAACAACCATCGTTAATACGGAAGTCACTTATGATACCCCCCTGGTACGCACCATAACCGATCAGGATGTAACCCGCGTTCGTTTCAACGTTGGCGTCACCGGT
CTGGTAGAGCAGGATACCAAAGGAAACCAGAAGAACACTTCAGTCACTATGGTTCTGGAGACCAGATCTGGAGCGAGCGGGTTTGCAATAGTAAAGACCGTCACCATTACAGGCAAAATC
TCAGGTGAATATCTTGAAGCACACCTGATTGACGCGCCGGAAACTAAACCGTTCGATATCCGTGTGCGTCGAATCACGCCGGACAGCACCAGTGACTTGCTGTCAAACGGCACTATCTGGAA
CAGCTACAGTGAGATCACCGACGATAACCTGAACTATCCGTTCTCCGCTATTGCGGGTGCGGTCATCGACCGCGACCAGTACACCGACACCCCAAGTCGCACATACCATCTTCGCGGCCTGAT
TGTGGACGTTCCTGACAACTACGATCCGATTGCCAGAACTTACTCGGGGTTGTGGACTGGCGGATTCAAAAAAGCGTGGACTAACAACCCGGCGTGGCTGTTCCGTGAACTGGCGAAGAA
TACGCGTTTTGGCCTGGCGAAACGTGCCGGTTATATCGATGTAGATGACGGTGCGTTGTACGTCCTCTCACAATATTGCGATCAGCTTGTTAATGATGGCTACGGCGGGCAGGAACCAAGGA
TGACGCTGAATGCCTATATTACCGAGCAGGAGAGTGCGCGAGACATTCTAGACAAGATAGCGAGCATGTTTCGAGGTATAGCGCTGTGGGACGGGATGCGACTGTCTGTCATGCTGGACGC
GCCACAAGACCCAATTGCGACAATCACGAATGCTAACGTGGTTGATGGCGAGTTCAAGCGCAGCTCCGTGAAGCGTTCAGAGAAATACAATGCCGTTGTTGTGTCCTGGACTGACCCGGAT
AACGGCTGGGAGCAGGTAAAAGAGTATGTTTCCGACGATGAGATGATCGCCCGCGGGAACTACAACGAAACAACAATTGAAGCATTCGGGTGCACGTCTCGTGGTCAGGCATGGCGCGC
TGGGAAATGGCTTCTTGAAACGGCGAAACGGGAAAGCAGCAGACTGTCTTTCCAGATGGCGCGCGATGCTATCCACTTCACGCCAGGTGACATCGTTGAAGTTATGGACAACAACTATGCT
GGTGCGCGTCTTGGTGGGCGCATCATGTCGCACGCGGGCAATAAGATTACCGTTGATGCTGTTGATTCGTCTCTGATATCAGAAGGCGACACCATGTCGATCATGGGTAGTGACGGGAAATT
CGTTAAGTACGTGATTGCCAGCATTGCCGACAACATCGTGACGCTGAAAACCACACCTGCATGGGTTCGTGACGGGACTGTATTCGCTATCTCTACCAGCAACGTTTCCACCAGACTATTCCG
CATCCTGAGCGTTGCAGAGACGGATAACAATTCTGTCTACAGCATCACCGCATCGCAGCATGATCCGAACAAACAGGCCATTGTTGATGAAGGCGCAGTGTTTGAAATCCCCAACGATACGC
TGAACGGTTACCGTGTACCGAACGTGGAGAACCTGCGCATCATCAACACCAACTCAGAGACTGTCCAGGTTACGGCCACGTGGGAGACGGCAACCACTACCAAAAAGCTGATGTTTGAAG
TGTATGTATACACCGATGACGGGAAAGTGGTTGCGCAATATGAAACAGACCAGTTCCGCTACGAGTTCTTTGGTCTGAACGCCGGCGGATACACGCTTGGCGTTCGCGGTCGCAATGAAAA
CGGAATGAAAGGCGCTGAGACGCAAATTAGTATGGTCATCGGTGCGCCACCTGCACCATCCAGTGTTATCTGGACGCCAGGCTTGTTCTCTGCTGACCTGGTCCCCGTCATGCGCATTACGG
CAACGACAGACACATCGTTTGAGTTCTGGTACTCCGGGCAGAACCAGATTGTCAATCCTGACGATATTGAAGACCAGACTCAGTTCCTCGGGCGCTCTAACCAGTGGACGCTTCATGGTCTA
CAGGCTGATAAGACGTATTACGTTTATGTCCGCACCAAAAATGCTTTCGGGGTATCGGAGTTCGTTGAGGCATCAGGTCAGGCGTCATCAGATATTCCTGGAATGATAGAACTCATTGATGAG
CAGATCCGCGAATCAGATGCGTTTAAAAATGTTCAGCAGGGTGTCAACACCAACCTGGACGGTATCATGTCGAACGCGCTGGCGAACCACGGAACCGTTGAGCATCAGTATCAGCAATATG
GGGAGGTTCGCGCCGACATCCTTGTTGTGAAAACAACTGTCGCTACTGCCGAGCAGGGGCTCGCCGATCTTTCCACTTACGTACAGGCACAAATAGGCCATGAAGGAGAGTTAACCTCAGC
CGTAAATCAGAAAATGACCGCTGAGGTAAATAGTGATGGGACTGCAAAAGCCTCTTACACACTCAATATGGGGATTGTCAGGAACGGTGTGAAATATAACACCGGATTCGGCATGTCTATCG
GGCCATCGGGGAATAGCTATAAATCTACCGTTGTATTTGCCGCGGATCAGTTCGGCATTTATTCCGGTAATAACCCCGGCAACTGGCAGGCTGCATTCTTCGTCTATAACGGACAGGTATTTAT
TCGTAGCGCATTAATTCAGGAAGCATCCATCGATTTTGCGAAAATTACCGATTCACTTCAGTCTGCAAACTTTATCCCCGGTGGTGGTGGACGCGGATGGAGTTTACCAAAATCTGGTAGCCC
AGAATTCCATGGGAAACTCTATGCCGACAGCGGTGAATTTGCATTTAACGGAGTGAATAACGTTACTCGCATTGACGGCAATGGGATCACAGTAAATCTCTCAGGAGGTGGTCGTGTTGTTG
TTGGACGATGGACATAA
>AONIPM_00310 Phage protein
ATGCCGGAAGGAATACTGATAGATTATAACGATGGCCGTCCTGCGATGGCGATTACAGCGGGGCTCCGTGCCCCGTCATTCTGCACAAGTTTTGCTGGTTACGGTACGGGGGCAAACCAGT
TTCAGGTTAATACTCCATTAACGTCAGGCTCCACAGTTTTTGTTTTACCGACACGTCCGGTTGACGTTCAGGAGTTCGCAGACAATCAGACATGGATAGTTTTACCGATATATATGACATCCGT
TACAAGAAACGGAGACAGCGGTGTGACTGTTAACGGTACAAACAGGGGAAACTACCAGCGAATACCAAACTGGGCAGGAACTGTATTCGAAATTCTCCCTGCAGCTACTTACAACGAAGG
ACTTCTCGTTTCCAACTCTACTGATTTCACTGCAATTTCGAATCAGGCAAGGTTAATGACATGTGCTTACGTTGGCACGGTGACTGTCAACGGCTCGATGGCGCTTCCCGTATCAGGAATACC
GTTCGGGAAATGGAGTAATAATAATGTGTCTGTAGGATTTGACGGAGCAAATATTATTGTAAGAGACATCAGTTACTCAGGACGTGATGATGTTCCCGCATCTGTAACAATGGACCTGGTAAT
TTTCAATAATATCGCGCCTGTAGCAGGTGATGGCATTACTATGACCAATCCATCAGGACAGGTTACGTTCTCTACAGTGAAGCGCCCATTTGTATATGACCAGCAACTAACGGTAACAGACAAT
AATCAATACATAGGTGATAAATATTGTCAGATAGTATTCACTGGCGCACAGTCAAGACGAGTGGATGGATATTTTAATATAAGGAAAAAAGGCGTGGTAATGTCAGGTGGAAACATCCGTTCA
GCGTATAACCAGGTTGTTGGTAATTACAATGACAACAGATTTGATATGACATTTAATCAAAATATTAATATGTCCATTCTTATTCTTCCGAACATGTACTGA
>AONIPM_00315 Peptidase S74 domain-containing protein
ATGTCAGCAGGAACCTTAACACTGACGAATAACTCTGCCCAGGTATCAGGGGCAGGGACTTCATTCACTACCGAACTGACGGATGGCGATTTTATTGTTGTCACTGTCGGCGGCGTTCCCTA
TACGCTCCCGGTTAAATCAGTGGAAAGCGGTACAGCGTTGACGCTGGTCAGTAATTTCACCGGGCCAACACAATCTGGTGCGGCCTGGTCAGCTGTTCCTCGTGTGGCGCTGAACATGGTT
ACTGCCGCGCTGGTGGCTCAGAGCGCAGAAGCGCTTCGCGGCCTGAACTACGACAAGCAAAACTGGCAGCAGTTTTTCACCGATGATGGTGATGTAACTATCACACTGCCTGACACCAGTC
AGACTACAGGTCCATCAGCGAAAAAGTTAATCAGTAGTGTGGCAAATAAAGCAGATAAGGTTAATGGCGTTGTTCCGAAAGAGCAGGGCGGTACCGGACTTTCTCAACCATTTGGCGATAA
AGCGGGACAATTTTGCCAGGGTAATGACCCCCGGCTCAATTCTGTTAATGGAAAGGCTGGGGGAAAAATAACATCAACGATATCAGTGATCGGAGAAAATAAGGCAGTAAAAGAGACAGC
TTCTGAACCCGATAATGGTGTGACGCTTAACGGCGCGTCGGTTCTTTCCGTGCATAATATTGCCGGGGTTGACCGGGCTGTAGCGAGCATTGAGGCGCGATACCAGTGGGGGCAACCCGTC
TCCGACGCATATATCAATTGCGGATTGCTCTCTGCGCAAGGAGGATGGGTCTCAGGATCATCGTATCGATTTAGTGGCAGTGGAGATGCGACCGCCCCTGGCTCCTGGGTCAATGGCGCCTC
AGACGTACGCGTTAAGCTTAATGTGAAGCCCATTGAAAACCCCAAATCGACCATGCGTAAGATAACGGCGGCGACCTGGAATCTTGACATTAAGGGGCAGGAAGGCAGATTCGGCATTGG
TGTTCTTGCCAACGGTCTTTATGATGATTACCCGGAAGCATCAATAAATGTAGGCGATAAAGAATTGTCTGACGGCACGATTATAAGTGATGTTCTTTCTGTGCAGGCTGGTGATTCTGGGGTA
CTGGCTGCGGTTCATCATGCAACGATACTTGAGTTGATGGATGACGTGGATGAGCTTAAAAAAATAATTTTATCTTTAAAGTCAAAATAG
>AONIPM_00320 Transposase
ATGCTAATCCTGAAAGCCTACAAATTCAGACTGGAACCAACGCCTGAACAGTCGCAGCGTTTGCGGCAGTTATGTGGTTGCGCCCGTTTTGTCTGGAATTTAGGTCTTGCGGAGACAAAGC
GCATCCTTGGCTCAGGCGAAAAGTTACCTTCGGCTTTCGAGTTGAACAGGATGCTTACAGTGTGGAAAAAAATGCCGGAACACATCTTTTTACAGGATGCTTATACCGACAATCTGCAACAA
AAGTTGAAAGACCTGCATGCTGCATGGAAACGTTGCTTTGATAAAAAGCTCGCAGCTAAGGCCCCGGTATGGAAACGAAAAAATGAGGGCAGAGACTCAATCCGTTTTGTGAACTTTGAG
AAATATTGCCGCCTTGAAAATCGCAGAGTGAAGCTACCGTCAGGTCTTGGGTGGGTAAAATTCCGGCAATCTCAACGTGTGAACGGTAAAATCAAAAATGCGACAATCAGTCAGTTAGCGG
GACAGTGGTATATCTCGTTTCAGGTTGAAGTTGAAACGGCAGAACCCAATCACACAAGCACAACGATAGTCGGTCTGGACGCAGGAGTAACAAAACTCGCCACGCTTTCAGACGGTACGG
TATACCAACCAGTCAACAGTTTTAAAGCAAGTCAGCGCAAGCTGGCAACACTCCAGCGGCAGTTAAGCCGTAAAGTCAAATTCAGCAGTAACTGGCAGAAACAAAAGCGAAAAGTCCAGC
GTCTGCACTCGCACATAGCCAATATCCGGCGCGACTACCTTCATAAAGTTACCAGTGAAATCAGCAAAAACCACGCGATGATAGTCATTGAGGATTTGAAGGTGAGTAACATGTCGAAATCG
GCAAAAGGTACAGCAGAACAGCACGGACGAAACGTCAAGGCCAAATCAGGTCTGAACCGTTCGATACTGGATCAGGGCTGGTATGAAATGCGCCGCCAGCTTGAGTACAAACAGCTCTG
GCGTGGCGGTCAGGTGCTTGCGGTTCCGCCAGCATACACAAGCCAGCGTTGCGCGTGCTGTGGTCATACCGCGAAAGAAAATCGCCTGTCACAAAGTAAATTTGTGTGTCAGGCATGCGG
ATATACAGCGAACGCTGATGTAAACGGCGCTCGTAACATTTTAGCGGCGGGGCACGCCGTTCTTGCCTGTGGAGGGATGGTGCAGTCAGGCCGCCCGTCTGAAACAGGAACCAGACGCTG
A
>AONIPM_00325 IS200/IS605 family ISSen6 transposase
ATGTTACTGTTTTTACTGTCAACTTCAATGCAAAAACATAATATTAATCGTTCAAGACATGCCGCCTTCCTTTTGCATGTTCATCTTGTCTTTGTAACAAAATACCGTCGAAAGATACTGGGTGA
GTCACACTATGCTGCATTCCATCAGTATGCAGCAGAAGTCTGCCATGATTTTGGTGCAGAACTTAAAGAAAGTAACGGAGACGTCGATCATGTCCACATGCTGATCGAATACCCACCAACAGT
GCAGCTTTCGGTACTGGTTAACTCACTGAAAGCTGTGACATCCCGCCGTCTGCGAAACGAATTTATTGATTTGCGGGGAGCATACGGAAAAGCCGTGCTCTGGTCGAGGTCATACTTTGCTG
GCTCGTGCGGAGGTGCGCCGCTGGAAGTTGTGAAGCAATACATTCAACATCAGCGTGGCTGA
>AONIPM_00330 hypothetical protein
ATGGTACGGCAGCGAATGCGTGGCTTATCGCAGAAAATCTTAGACCGTCTGTTGACACACCTATTTCTGCATGGGGGGTATCTAGCGGTGGTGTCCTTGTACCTGTTGAGGCGTATGTGCGA
GCAACTGGTTATATTGAGATTACAGGTTATGCATCTCTTGGGACATCGCAAGCCGTGA

Prophage 14

>AONIPM_09630 Integrase
ATGCGTTACCGTTTCGACGGCAAGGAAAAGACACTGGTCATCGGACCGTACCCGCAAATCTCTCTTACCGAAGCCAGGGCAAAACAATCTGACGCGAAAATGAAGCTGCTTGCTGGCGTG
GACCCATCAGAACAGAAACAGGCTATAAAGAAGAAGGAAAAGGAAGAAGTAGCTGATTCGTTCGGTGATATCTTCAGGGAGTGGCATGCTCATAAATCGAAGGTATGGTCGAAAGGATAT
GCTGACGAAATGATGAACATGTTCACTGGCGATATATTGCCACTCATCGGACATCTGAGAATGGAAGAGGTGGAGCCGATGATGCTACTGAAGGTGATCAGGCTATTTGAGGACAGAGGGG
CGATGGAACGTGCTGATAAGGCTCGTCGCAGGTGTGGCGAGGTTTTCAGCTACGCAATAGTAACCGGAAGAGCTAAATATAATCCGGCTCCAGACCTTGTTGGGGCAATGAAGGGTTACAG
AAAAAACAACTACCCTTTCCTACCTATGCATCGCATTCACGAATTCCAGAGGGCGCTGAATGGGTATGGAGGCTGGGTTATAGGTAAGATTGCTGCTCAAGTTCTTCACTATACAGCAATGCG
AACAGTGGAGTTACGTTCGTTGGTATGGTCAGGAATTGACTTTGAAAACAGGCTGATCACCGTTGACCCTGAAGTCATGAAAGGAAGAAAACTGCATGTCGTTCCAATGTCAGAGCAAGTT
ACAGCGCTTTTCAAATTCCTGCAACAAATCACCGGACAGTACGAACTTTGCTTCCCGGGAAGGAATGACAGGAAGAAGCCAATCAGCGAAAATGCCGTCCTTGGTGTAATCCGCGGCATAG
GATATGAAGGGCAGACATGCGGACACGGTTTCAGACATCAATTCAGCACGGTACTCAACGAGAAGCACTGGAACAGCGACGCAATAGAGATGCAGCTGGCACACGTAAGCGGCGGGACG
CGCTCAGTTTACAACCATGCTGCATATCTGGCTACCCGCAGAGAAATGATGCAATTTTGGGCGGACTGGCTTGATGAGAAGGTGTCGTAG
>AONIPM_09635 hypothetical protein
ATGACAGATATCACTGCCAACGCAGTTGTTTCTAACCCTCGCCCAATCTTTACTGAATCCCGTTCGTTTAAAGCTGTTGCGAATGGGAAAATTTACATTGGTCAGATTGATACAGATCCGGTTA
ATCCTGCCAATCAGATACCCGTATACATTGAAAATGAGGATGGCTCTCACGTCCAGATTACTCAGCCGCTAATTATCAACGCAGCCGGTAAAATCGTATACAACGGCCAACTGGTGAAAGTTG
TCACCGTTAAGGGTCATAGCATGGCTATCTATGATGCCTATGGTTGTCAGGTTGACTATATTGCTAACGTATTGAAGTATGACCCAGATCAGCTTGAATACAGGCTGAGCCAACCAGACGGTTA
TCTTTTGGTTGGTGGACTGGCCGAGCATTATAACCTTCCGGCTAAATTTGTCGTCGTCGACAACGAGCCATATAACGGTGATTTGAAAGCAGCACTTTCTGAGGCTGAAGCCGGTACTGTGT
TTTGGCTTGGTAAAAAAACATACAACATTACCGGCCTGTACGGAACTGGCAGGAACACAGTTGAAAATATCTCTATTGTTGGCACTGGGATGCCTCAGTTATCTGATGATAAAACCAGATTTA
TCGATGGAACTGGGACAGTTATTCAAGGGGCGGTAAAGAATCAAGCGAGAGGGTTTAAAACCTACAACCTTGGGATCGATGTTGGTGCTTATGTGTCTCAGAATGTGTATACAACGGAAAC
TTACGAGGACGCTCTTGTGCACTATGGTGTTGGTTCCAACGCCAATATAGAAATTGACAATGTCAAGACGCTGAGTTCAGTTAACGTTGCCAGTAAGCCAGGAACGCACAGCATTCTTCTTG
AGCAATTATCCGGTGTAACCTTGGGTTACGTTGAGTGCATTGGTGGTTTTCATGGTCTAACGATTAAGTGTCAAAATTTGCAGGGCGGCATTGCTCATTGCTACGGCCAATATGGTGATGCAT
TTATCTTCAAATCAGATTCTGGTGGCGCATGCGCCAGTAACTATATGGAAAGAATTGCAGTTGGTCTTTATGACAACGCTGGCTGGCCTGACGTCACCATGGGTGGTATTTACGACGCCCACG
ATGATGTAACAATCGACAGAATTGGCATTGGTGAGTTAATTGTGCAGAACGCGTCATGGGGATTTATACCATCTGATGCCAATACCGGTTTCATAACAAACGTCAGCATTGGTAGATACTCTGC
ATTCAATGTCTATGGGAACTATTATTCATTAACCATTGATAATAAATGTGTTGGTTGGACTATTGGTGAGCACAGAATTAGCAATGCATCTGGCGGCATTCGTGTTCACCCGGACTCTGCAGAA
ATCAATATTGGAACCGGATCCGCAAAGGGGAATACTGAGAGTGGCTATGCGTTGGGAGGCAACAGTTTAAGTCACGGCGTGCTCTTTGCTAATGAGAATGGGAAGGCTGGAGTTGATTAC
CTCGGTGGTATTGGTTTTGATGCCTCTCTTGTCCGTGGTTATGTTAACGGAACGGTTCTTGTTTCAGGATATCCAGGCGTAAAAGACGGTAATCCTGTAAATGGGTGGGCTGATACTGGTGAT
TTTGACATGATGCTAACCGGCAAGACTGTGCAGATCACAGGTTCACTGACTCGCGGAACAGCTGCGGTTGCGTATAACACTATCGCTGCGTGCAGGCCTTTGAAGCGAGTGCCTGTCCCGG
CATGGGGTGTTAGCGCGACAAGCTCTATGATCCCAGTTGAGTGTTATATTGAAACTAACGGTCAATTAAACGTAGCTGGGTTCGCCTCGATACCAACTGGCGGAACTGTATATTTCTCTGGAC
AATATTTAACAAAGTAA
>AONIPM_09640 Phage antirepressor protein
ATGAAAAGTATAGCAACAGCAGTATCTACTATCAACGTGCCATTCCACGGCGCAGAGCTTTATGTTGTCAATCACAACGGCGAACCGTATACCCCAATGAAACCTATCGTTGAAGGCATGGGT
ATGGATTGGGCTTCACAGTTTACAAAGTTAAAACAAAGATTTGCTAAAGGTATTGTGGAAATCGCAATACCTTCAGTTGGTGGTGTGCAGACCATGATTTGCCTTGCTTTGCGTAAACTGAAT
GGCTGGCTGCAAACCATCAGTCCTAACAAAGTCCGCCCTGAAATCCGCGACAAGGTAATCCAGTATCAGGAAGAGTGTGACGATGTGCTCTACGAGTACTGGACTAAAGGCCATGTAGTTA
ACCCACGCAAGGCTAAAAAAGCGTTGCCGGGTAAAATCACCACTGAACAGCAGGAAGCCATTAAACAACTCGTCATGAGTCGCGGTCAGTCTCTGCCAAAAGAAAAACAGGCTAAGGCG
ATGATCACCATGTGGTCGTCACTGAAATCCCATTTTGGATGTTCATACAAAGAAATCAGTGAGGGGCAGTTTACCGAAGCACTGTCACTTGCAGCTCGAGTTCCGCTTGAAGGTGAGTTCAT
TGGCAAACAAGAGAAGAAAACCAACGAGCTTTCTGCAAAAGAAGCAAACAGCCTTGTATGGTTATGGGATTATGCCAACCGCTCACAGGCATTATTCCGCGAACTGTATCCGGCGCTAAAA
CAAATTCAATCGAACTATTCCGGCAGATGCTACGACTACGGTCATGAGTTCTCGTATGTTATCGGAATGGCGAGAGACGTTTTAATCAATCACACACGAGATGTTGATATTAATGAGCCAGAC
GGACCAACGAATCTTTCCGCATGGATGAGACTTAAGAATAAAGAATTACCTCCTTCAGTACATAACTACTGA
>AONIPM_09645 sar RNA
TCGTTAATTCCTATGTGTGGTTTACGATACCGAAGCCCTGACTGTTCCCGCAGTTGGGGCTTCAACT
>AONIPM_09650 Transcriptional regulator
ATGAAAGGAATGAGCAAAATGCCGCAGTTCAATTTGCGGTGGCCTAAAGAAGTATTGGATTTGGTCCGCAAGGTGGCGGAAGAGAATGGTCGGTCTGTTAACTCTGAGATTTATCAGAGA
GTAATGGAAAGCTTTAAGAAGGAAGGGCGCATTGGCGCGTAA
>AONIPM_09655 Regulatory protein mnt
ATGGCTAGAGATGATCCGCACTTTAACTTCCGTATGCCTATGGAAGTAAGGGAGAAATTAAAATTCAGGGCGGAGGCGAATGGGAGATCAATGAACTCCGAGTTGTTACAAATCGTCCAAG
ATGCTCTATCAAAACCATCGCCTGTGACTGGCTATCGCGACGAAGCTGAACGCTTGGCTGATCAGCAGGCAGAGCAGTTCAAGACTGTTGTGTTTGAGACACTTAAAAAGATTTATGGCAA
GGATGATAAATAA
>AONIPM_09660 ATP-dependent Clp protease proteolytic subunit
ATGCTGCACACAATTCATTTCTTATGCCCCGTTAACACCGCCACTGTTGGGCAACTTCAGAACCACTGTCTCACCGCATTATCTCAAGGCGCAACTGAATTAAATATCCATATATCAAGTCAGGG
AGGGGAAACTGCCGCTGGTTTTACTGCGTATAACTTTCTTAAGTCACTCCCTGTTACCGTTAGAACTCACAACATAAGCAATGTTGAATCCATAGCTAATATCGTTTTCCTGGCTGGCTCAGAA
CGTTTCGCAAACCCATTATCAAGATTCCTGTTACATCCTCTATTATGGGGCTTTGCCACCCCAGCCGCCGACCATGCCAGATTGAGAGAGTACGGGAAATGCCTCGATAACGATCTTGATCGCT
TCGTTGAGACGTTCAATATCGACATCGGAACCCATATTAGGTGGGCATCCCTGATAGCAGACTCGACCATTTTGGATGCTAACAAGGCTCTTGAGCATGGCATAATTAATTCCATAAAAACTGC
AAGGCTGGTATCCAATCAGGCAAACTGGTGGGTTGTTTGA
>AONIPM_09665 Cytoplasmic protein
ATGACCATAGAAGAACGCCTGAACAACATTGAGTTGAACCAAACCCTGCTTGACCAGCGACTTTTAGAGCTTGAGCTTAAAGATCTAGATGCGCAAATATCAGAAGCAGAAGCCAAACTCT
CCAGCTTAAATCACCGTAAGAAGCAAATCCGCGACAGAATTACTCAGGGACGCGGAAGCTGTTGA
>AONIPM_09670 P63C domain-containing protein
ATGACTGAAAAGAAAAGTGGCGAAGGGAAAGCTAAGGGCGGGATCGCTCGCGCAAAGTCGCTGACTAAAGAGCAGCGTTCTGAAATAGCAAAGAAAGCAGCGGCAAAACGATGGGAA
GGTAAGCCACTAAGAGCGATCAGAAAGGGTAACTTCATTGATGATTTCGGCATTGATGCAGAATGTTACGTACTTGATGATAATGATAAAACGGTTGTAGTTAGTAAAACTGGTCTGGCTAAA
TTACTAGGAATCGGTGAGCATGGTAGGGATGTAGACCGTTTTCTAAATACGAATTATATGGCTGATTTTGTTGATCCATTTCTGCTAGAAAAATTCAAAAAACCTCTTATTTTTCAATGGAGAA
GTCCGGTCCCAAACTCGGCCCCAAATCCACTCGCTGATGAGGCTCACGGTTACGATATTGCCCTTATTGGTGATATAGCCACAGCAATGATTAATGCCGATCGAGCTGGCGCACTACCGCCTT
CAAGATCAAAATCTGCCAGCCTTGCCCAAAGACTTGTTACCGCATCAATGAAAGCAGGGCTTAAAGGACTTGGTTATGCTATTGCAGGATATCGACCTGAAGTACAGGAGGTTATTGATTCC
TTCAAGGCCTTTGTTCGTGAAGAGGCGAGGCAGTATGAAAAAGAATTTCCAGATGAGCTATATGAGGAGTGGTATCGATTGTACGGCCTGAACAGGCCAGAGAAAGGACGTCCAATTCGT
TTTGGGCAACTAACCAACATGCAGATATACACTCCGCTAGCAAAGAGTAAAGGTAAAATCCTTGAACAGATTCGAGCCAGCCGAGACGAGAACGGAAAACAATCTGATAAGTTGCACCTGT
TCCTTTCTGAAATTGGTGTCAAGGCTTTGCGTCAGCATATCGGTAAGCTTCTTGGTGTCGCAGCGATGAGCGAGACAAGAGAAGAATACGAAAAAGGAATAGAAAAGGTTTTTGGAAGAA
TAAAACCAGAAATCTAA

>AONIPM_09675 Lipoprotein
GTGAAGAAAAAATTTGTAAACGTTGCAAAGTGCGCAATTTTTATATTTTTTATGACCTTTTACGGATTTAGTGCCGGAGAAGGTTTCGATCGTGATGCCAATGCATTCAGTTTCTTTTGTCTGT
CTATAGTGGTCATAATTGTATGGGTGGAATTAAAGCAAACATTGTTTCATGTGCTAGGGAAATAA
>AONIPM_09680 DNA transfer protein
ATGGCTAAAGCATGGAAAGATGTTATCGCCTCTCCACAGTATCAGGCGTTAACTGAAGAACAGAAAGCACAGGCTCAAGCGCAATATTTTGATGAGGTTGTTGCCCCTAAGGCTGGTGACA
AATGGGCTGAAGCAAGAGATCAGTTTTATGCAGCATACCCTCCGCCTCAGCAGCAGAAAGAAGAACCATCATTGATGCAACAAGCTGGCGATTGGCTCACTGGTGGTCAAAGTGCAGGGC
AAATTGCAGAGCAGGCTGGTCGTGGTCTGGTAAACATACCATTTGACGTATTGCAGGGTGGCGCAAGTCTGATTAATGCAATCAGCCAGGGGCTTGGTGGCCCCAAGGTTTTGGACGATGT
CTATCGTCCAGTAGATCGACCAACAGACCCTTATGCGCAAGCTGGAGAAACAATTGGCGGGTATTTAGTTCCAGGAGTTGGAACGGCAGGAAGCATGGTTATTGGATCACTGGCAGAGGC
CGCAAATCAGGAAGGCGATTTCGCACAAAATGCAGCTAAAAATGCCGGAGTTAACCTTGCCGCTCAGGGGGTTCTTTCCGCAGCAGCAAAGGGAATAGGGCGTGGAATTACTGCTGTTCG
TGGCGAAATATCACCAGCAGATCAGCAATTGCTCAAGCGTGCCGCTGCGGCAGATGTACCAGTTATGACATCGGATGTAGTTCCTCCAAAAACAAAACTTGGCAATCAACTGCAGGGTTACT
CAGAAGGAGTCATAGCTGGTACTGGACCAATGAGAGCCGCACAGCAGGATGCCAGAACCAAGCTTGTTAATCGCTTCACCGAAAAATACGGCGACTACGATCCATCTGTAGTCGTTGATAG
TCTAAAGTCAGGCGTTGCAAGGGAAAAATCGTTAGCCAAGTCAAAACTAAACAGCCTGTCAGGAAGAATGGTTGGAAAGCCAGTTGATACAAGTGGCGCCATAAGAGCTATCGACGGAGC
AGTAAACGAACTTGGGAAACTTAAAGGTGTTTCTGACACCCAGACCATTTCTGCGCTTAATGATTATAAGAATGCCATTCAGGAGATAACAAATGGAGATGATGCCTTTGAGTTACTTGATAA
GCTGAGAACTCAGTTCCGCATTGACGTAAAAGGCGATCGTACAGTTCTGCCATCAATGTCGCAAACAATGGTCGACAGGGTCTATAACTCGCTAACCAATAGCCTTAGTAAATCTATAGCGAA
AGGACTTAGCCCAAAAGATGCTTCAGCATGGAGAGCGGGAAAAGCTGATTATGCAAAAATGGCAACACATGCAACTCAAACGCGCCTTAAAAACGTTCTAAACAAAGGAGATTTAACTCCC
GAGGCTGTAAATACCATTGTGTATGGACAATATGGGTCAGATATAGCTCGATTGTACGGGAAACTCGATCAAAAAGGTAAAGACATGCTAAGGGCGGCATATATCAGCAAAATAGCTGACAA
GGTAGGTGACAGCCCTCAGAAAATGATGACCGAGCTTGGCAAGCTGCAAAAACAAGCAAATGGTCAGGTGTTTAAAACTGTATTTGGTGGGAAGAACGGAAAAGAGATAGAGGGGATGT
TATCTATTCTCGATGCTACCAAAAGAGCATCTGAGGCTAATGTTGTGACGAAGACTGGCATGACACTCGCGCCTTTGGTAAGGGTTATTGGTAACCTAAAAACCGGAGGCGCGCTATTGGCT
GGGGAAACAGGGATTGGCCTTATGTCGAGGGTTTATGAAAGCCCTATGGCCAGGAATGCGCTCTTACGTCTGGCAAACACCAAAGCAGGAACGCCAGCCTATGAAAGAGCGCTGAATAAC
GCTGCGAATGCCATCAGACCGCTGCTTGCCACTGAGGCAACACAGCAGTGA
>AONIPM_09685 Internal virion protein gp20
ATGGCTACGTGGCAACAGGGTATTAATTCTGGTGGTTTTCTGGCTGGAATTGGTGCGCAAAATGAGAATGCGCCAAAGGCAAGCGACATTAACGCAACGCTTGGTCTGATCCGCGAAAACA
ATGAACTGGCTCGCTCAGGTGCAAATAACGTTGGTCTGACCGCGTTACGTGGTCTGGCTGGAGTTGCTGATATTTATAAGCAGGAACAGCAACAGAAAGCGATTAATGCGTTCAATAAGGTT
CATGCTGATGCATGGGCTTCTGGTGATCCATCGGGACTATTTAAGTTTGCCAAGGAAAATCCAGCGTTTGTTGCGCAGGCACAACAGGCGTTTTCCGGTCTTAATGAGCAGCAACGCAACG
ATATGGGCGATTTAGCCATGAGGGCTAACGTCGCTCTTTCTCAGGGACCGGAAGCCTACAGTAAATTCATTACTGACAACAAGGACAGGTTAAATCGCGTTGGTGCTAATGCTGACTGGATG
ATTCAGACAGGTATCCAGAATCCAGAGCAGCTATCACACATGCTGACTACTATGTCTCTCGGTGCGCTTGGACCAGAAAAGGCGTTTGCTGTTCAGGACAAGATGGCTGGTCGTGAGATTGA
CCGAGGCAGACTGGCAGAGACAATCCGCAGCAATCAGGCTGGCGAGGCGCTAACAGCGCGTGGTCAGAATATCACGATGCGCGGTCAGGATTTATCGATGCAGAGAGCATCAATGAAAG
GGGCGGTTGGGAATAATGAGCGTACAGTTCAGTTAGCAGATGGCAGAACTGTAACGGTAGGCGGGAAGCTTCACGGAGCTGGGGCTAATGCGTTCTACGAAGGTATCGACAACGAGGGG
AATATGGTTCGCGTCCCTGCTGGCTCTATTGCCGCTCCGGCTACATCGGCAGCAAGCGCGCAGAATTACGCAATGAAGAAAGATCTTGATGCAATTTCTGGTGCATCAATTGACGATCTTGGC
TTCATGACTGGCATTACAGGCTCTTCAGGTTCTCCTGCTCTTGGTGCAGATATTCGTAGCCGTGCATCTGGTGGTGATCAGAGGAAACTATACAACGCTGCACAGCGAATCCAAGGAAAGAT
GCAGAATCAGGGCATTGCAGCAGCCAGAGACATGGGGGCATCCGGTATCAACACCGTTGCAGAAGCAAAGATGTATTTTCAAGGTATGCCACAGGTTGATTTCTCAAGCCCTGAAGCACTG
CAACAATCAATGCGCGACATTCAGCAATATACCGACAATTACAACCAGCAATATAACGTTAATGTTGGTAAATCTCAGCGGCAGCAATCTCAACCTACACAGGTATCACAGCCAGCAGCCAGC
AGTAACTATTCTTCACTATGGGGTGATTAA
>AONIPM_09690 Internal virion protein gp7
ATGTTATATGCATTTAAGCTGGGCAGAAAACTGCGCGGTGAGGAACCTCTTTGCCCTGAAAAAGGCGGAAAAGGTGGCGCAGATAAAAGCGCAAAGTATGCAGCAGAAGCGCAAAAGTA
TGCCGCAGACCTGCAAAACCAGCAGTTCAACACCATCATGAACAACCTGAAGCCGTTTACTCCTCTGGCAGATAAGTATATCGGCAGTCTTGAAGGTTTATCCTCTCTCGAAGGTCAGGGGC
AGGCGCTTAACGATTATTACAACTCTCAGCAGTATAAAGATCTTGCTGGTCAGGCTCGCTATCAGAGTCTGGCGGCAGCGGAAGCAACAGGTGGATTGGGTTCCACCGCAACCAGTAATCA
GTTAGCAACAATCGCACCAACGCTTGGTCAGCAATGGCTATCTGGACAAATGAACAATTACAACAACCTGGCAAATATCGGTCTTGGCGCTCTTCAGGGACAGGCAAACGCCGGGCAAACA
TATGCCAACAACATGAGTCAGATTTCACAGCAAAGCGCGGCGCTGGCGGCGGCAAACGCCAACCGACCGTCAGCATTGCAGCAGGGTGTTAGTGGTGCTGCATCCGGTGCGCTTTTGGGT
GGTGGCATAGCCAGTGCTCTCGAGCTATCAACTCCGTGGGGTGCTGGTATCGGTGCTGGTCTTGGTCTGCTTGGCTCGTTGTTTTAA
>AONIPM_09695 Protein gp14
ATGATTACATTCACTCCCACCCGAAACATCGACCTGATAGAAACGGTCGGCAACCATCCCGACATCATCGCCGGGAGTAACAACGGTGACGGATACGACTACAAGCCTGAGTGCCGCTATTT
CGAAGTGAACGTACATGGTCAGTTCGGTGGCATCGTGTATTACAACGAGATTCAGCCGCTGACCTTTGACTGCCACGCCATGTACCTGCCTGAGATTCGCGGATTCAGTAAGGAAATCGGGC
TGACGTTCTGGCGATACATTCTCACCAACACCACCGTTCAGTGCGTTACATCATTTGCTGCACGCAAATTTCGCCACGGTCAGATGTACTGCGCGATGATTGGGCTCAAGCGAGTAGGAACC
ATCAAGAAATACTTCAAAGGCGTAGATGACGTGACGTTTTACGCCGCCACCCGAGAAGAGTTAACCGACTTCCTGAATAACGGGAGATAA
>AONIPM_09700 Phage tail protein
ATGGCAGACCCGTCACTTAATAATCCCGTCATCATTCAGGCTACTCGTCTTGATGCCTCAATCCTCCCACGCAACGTCTTCAGCCAGTCCTATCTGCTCTACGTAATCGCGCAGGGGACTGACG
TTGGCGCTATTGCAGGAAAGGCAAACGAAGCAGGACAAGGTGCCTATGACGCGCAGGTGAAGAACGATGAGCAGGATGTTGAGCTTGCAGACCACGAGGCGAGAATTCAGCAGTTACG
CATCGACGTAGACGACCATGAAATACGTATTACTGCAAATGCCAATGCAATTGCGGTACTGGATGTCAGACTAACCACGGCTGAAGGCAAAATAGTCACCTTGCAGGCTGATGTCAGTGCTC
TTGATGGTAGGGTTACGGCTGCTGAAAGCACTATTTCTTCATTGCAGGCTGATTACGTATCGAAGTCAGCAACTGCTTCTCAATCGCTGGCGTCACCTCTCAACGTGACAACGTCCTATTCAG
TTGGCGGCACTAAAGTTATCGGTGCGCGACAGACAGGATGGACAGCAGCAACAGGTGCTGCGCTTCTCGGCACATTCAACGCTAACCAGGCATACACGGTCAGTGCCACATATACGCAGTC
TGAGGTATCAGCTATGGCTACCGGATTGCAGCAGGCGCGGCAGCGTATCAAAGCTCTCGAAGATGCAATACGAACTCATGGATTGATCAACTGA
>AONIPM_09705 Phage head DNA stabilization protein
ATGGCAATACAACAACTACCACTAATGAAGGGATTAGGGAAAAGTGCGGTTAATGCTGATTATATAGACCAACTTCCAGTCAATCTTTTAGCTACGCCCAAGGAGGTGTTGAATTCATCGGGA
TATCTTCGCTCATTCCCGGGCATTGCCAAACGCTCTGATGTAAACGGTGTATCGCGCGGAGTCGAGTACAACATGGCGCAGAATGCTGTTTATCGCGTGTGTGGTGGCAAGCTCTACAAAGG
AGAAAGTGAAGTCGGTGATGTTTCCGGAAGTGGTCGTGTATCAATGGCGCATGGTCGGACATCTCAGGCGGTTGGCGTTAACGGGCAACTGGTCGAGTATCGCTATGATGGAACGGTTAA
AACCGTCTCAAACTGGCCTACAGACAGCGGATTCACTCAGTATGAGTTAGGCTCGGTCCGCGACATTACGCGCTTGCGTGGGCGTTATGCGTGGTCAAAAGACGGAACTGATTCATGGTTT
ATCACTGACCTTGAAGACGAATCGCACCCTGACCGATACAGTGCACAATATCGCGCAGAATCGCAGCCGGACGGCATCATCGGCATCGGCACATGGCGAGACTTCATTGTCTGCTTTGGTTC
ATCGACGATTGAATATTTTTCCCTGACTGGTGCAACCACCGCTGGCGCTGCGTTGTATGTCGCACAGCCATCGTTGATGGTACAGAAGGGCATTGCCGGAACATGCTGTAAAACGCCATTCG
CTGATTCATATGCATTCATCAGTCACCCGGCTACTGGCGCACCTTCCGTCTACATCATCGGGTCAGGGCAGGCTTCACCAATTGCGACGGCCAGTATTGATAAAATTATCCGCTCATACACGGC
TGATGAACTGGCGACCGGGGTGATGGAGGCGTTGAGGTTCGATTCGCACGAACTGCTGATTATCCATCTCCCGCGTCATGTGCTGGTTTACGATGCCTCATCAAGCCAGAACGGGCCGCAA
TGGTGCGTACTGAAAACCGGTTTATACGACGATGTTTATCGCGCCATCGATTTCATGTACGAAGGCAACCAGATTACGTGTGGCGACAAGTCAGAAGCGGTGACGGGGCAGTTGCAATTCG
ACATCAGTAGTCAGTACGACAAGCAGCAAGAACACCTGTTGTTTACACCCCTCTTCAAAGCGAACAATGCCAGATGCTTCGACCTTGAGGTTGAATCATCCACTGGTGTTGCTCAATACGCT
GACCGCCTGTTTCTGTCTGCAACGACTGACGGAATCAATTACGGTCGCGAACAGATGATTGAGCAGAATGAGCCGTTTGTGTACGACAAGCGTGTTATCTGGAAACGTGTAGGGCGTATTC
GTAGATTAATCGGCTTCAAACTGCGGGTAATCACAAAATCACCAGTAACACTATCAGGGTGTCAAATTCGTCTGGAGTAA
>AONIPM_09710 HNHc domain-containing protein
ATGCTTTCTGAGAATGCTAAAGATATACCTGGATTTGAAGGTGTTTATGCCGTAACAGAAGATGGCAGGGTGTATTCTCACTCACGTGTTGTTAAGGCTGCGCATGGCAGCACGCAACTCAG
AAAGGGGCGCTGGTTAAAGCCTAAAATAAATCAGGGAAGGGTGCTTTATAATATCGGAGCAAAATGGACTTTTGCCCATCGAATCGTTGCAATGACATTCCTGCCAAATCCTGAAAACAAGC
CTCAGGTAAATCATATTGATGGCAATCCACTCAATAATAACGTCAATAATCTTGAGTGGTGCACTCAAAGCGAAAACATCAAACATGCATACGCCACAGGATTAAAGAAACCAATCAAGTTTTT

CGGAACCAAGCACCCAAAACACAAGTTGAGTGATGACGATGTTCTTGCAATCAAGTCATCAAAAGAAAGCTTGTCAGTAATTGCGGCTAAGTACGGGATATCTAAGACTTGGGCAAGTAGG
CTAAAGCGTGATGCTAACTGGGTTCATATAAAGGTTGATTCCAATGGCAATACAACAACTACCACTAATGAAGGGATTAGGGAAAAGTGCGGTTAA
>AONIPM_09715 hypothetical protein
ATGCCGATTCAGCAACTTCCGCTTATGAAAGGTGTCGGCAAAGACTTTCGAAACGCCGACTATATCGACTATCTGCCAGTGAATATGTTGGCAATTTTGATATAA
>AONIPM_09720 DNA stabilization protein
ATGGCGAAAACGAAGGGTGATCTCGTTCTAAAGGCTTTACGAAAAGCCGGGCTGTATTCCAATGCCACGTTGACAGATGCCGACCCTCAGGCAATTGAAGATGCCATTAATGACCTCGAAG
ACATGATGGCAGCATGGCAGGCGAAAGGTATCGAGCTTGGATATCAGTTTGCTGATACAGAAAACGGCATCATGCCGTTACCTGACGATGATTCAGGTATCCCTGCATGGGCAAATGATGGC
GTCGCTTTGAAACTCGCTGTGCAAGTGTGCATGGATAACGTCATTCAGCCGTCGGATGCTCTCCTTACCGCTGCTGACAGCGCATATCAGACAATCTGCATCGCTTTAACCAAAATACCACCA
CTTGAGCGGCGAAATGACATGCCTCGCGGTAGTGGTAACAAAAGCGCGTTTACGTGGAATCGGTTTTACATCGAGAAAGATGAACCGAGTACGTGA
>AONIPM_09725 hypothetical protein
ATGGCACGAACAATGCTCTATAAGCCTGGCAACATGATCACCTGTGGTCAGTTTGCTGTCGATTACATCATTGTTGATGACGAAGAAGTTAAATCTCACCTGAAAAAAGGTTGGGTAAAAACT
CCTGAAGAAACCGCAACGAAGCAAAAAGTGGCTAAGGCGGAAGAAGATGGCGAAAACGAAGGGTGA
>AONIPM_09730 Coat protein
ATGCCTAACCAATTAGCAAAAGACCTTGAAATCATGTTCGAAAACTACGTTGAAGGCTTTGAGGCCGCCTGCGTAGTTTCCCGTAACGCTAAAAAATTCCGTCCCGGTGATACAGCAATGCA
GCGAGCAGGTGATGTTCTGTATCGTCCGCAGCATTACCACATGAACATTGAGGAAGGCCTAGACCTCAGCAGCAAAACGCCAACAGCACTGGTTCAGCGCCTTGTTCCTTCAGTGTTCAAG
GAGCCGAAAAACATTCTGTACACTCTGGATGCGCGTGAAATGCGTGACCCGGAACATAAAACTGAAGCTGGTCGCGCCGCAGGTATGCGCCTTGCTGCACAGATTGACTCGGACCTGATTT
CCATGGTCACGCAGCGTGCTACTAACGTGATCACAATGGCTGACTCAACCACTGGTTCACAGGGCCGTGATTTGTGGAACTGTGCGGCAGGTATTGATGCCACCATGACGGCGATTGGTGT
ACCGCAGGGTATCAACCGTCGCTCTTTCTGGAACCCCTTCAACTACAAAGACCTTGCTGGCGAGCTTGGTCACCGAGCCTATGCTCAGGGCGCAACCCTGACAGCATACGAAAAAGCGCAG
ATCCCTCCGGTTGCGTCCTTCGATAGCTACAAGACCGATATTTCTGGTCGTGTTCCGAAGGGTACAGCAACTTCCATTACGCTGGCAGCAGCACCTGCGCACAAGGTTGAAGCGAAAGATGC
TAACGATATGCCAGTGGATAACCGACAGGGGACCATTACGGTATCTGTTGAAGGTTTGCAGGTTGGCGATGCGTTTACCATCGCAGGGGTGAATTCCGTACACCAGATCACCAAAGATACCA
CCGGGCAGCCGCAAGTATTCCGCGTTCTGGCAGTTAGCGGAACGACAGTAACTATCTCCCCGAAAATTCTGCCGCCTGACAACTCGGATGTCGCCAGCCGTCCATATGCAAACGTTGATGCT
AATGCGGCAAGTAGCGCAGCAATCACCATTCTCAACAAAAATGCCGCACCGGCTAACCTGTTCTGGGCTGATGGTTCTGTTGAACTGATGTACGGCAAACTGGCGTTCCCAACTGGTCAGG
GTCCACAGGTAATGACAGCAACCACCGAGCAGGGCGCTACGCTGATCATGTCTTACGCCTTCGACCACATCAAAGGCGTAACCACTGCTCGTTTCACCACTCTGTACGGTTGCTCTGTACTTG
TTCCTGAATATACGGGCATCGTTATTGCCGGGCAGTAA
>AONIPM_09735 Scaffolding protein
ATGGACCAGATGGCAGAAAACACACCAGAAGTTGAAATCGAAACCGACGCGTCAGAGCAGATTCCTGATGATGTCGAACTGGTTGAAGAAGTCGAAACAGAAGATGGCAGTGAGTCCTC
CGGCAATGATGCAGAGGAAGCTACTGAAACTGATGACGACGAATCAGAACAGGAATTCTACTTTGGTGACGAAAAGCTGGATTCGCCAACCAGCGAAGATGGCGCAGAGCATGGACTGG
TAAAACACCTGCGCAAGACGATTAAAGAGAAAGACCGCGAGCTGAAAGAGCTGATGCGTCAGTCTCAGAAACCCGTCGAGCAGCAGCCGGTAATCACTCAACCACCGCGAATGCCAAAA
CTGGACGATGATGACATCGGTTTCGATGAAGAAATCTACCAGCAACGCATGGCTAAGTGGGCAGAGGATAACGGCAAGTACCAGCAACAGGAGATGGCTCGCAAGCAGAAGGAGCAGGA
GCTTCAGGCTGCCTATCAAGAGCGATTATCCAAATATCAGCAACGTGTTAAGGCTCTCAAAGTTCCTGGCTATCAGGAAGCAGAACAGGCTGTACTCGAGGAAATCCCCATCGAGACACAAA
ACGCGATCCTGTTTGAGTCAGAGAAGCCGGAAATCGTTGTTCTCGCGCTCGGTCGCAACGCTGAACTGCGCAAGCAACTGGCAGAAGCTACCAACCCAGTAGCAATTGGTCGTCTGCTGG
AACGTATCGAATCGAAGGCCAGAATCATGCCAAAAGCAAAAACCACGGCAGCCACAACCCCGACAGTTAAGGGGAGCAACGGCGCAGTAATCAATAACCTCGACAAACTGAAAGCCAAG
GCGCGGGAAACTGGTGACTGGACGCCGTATTTCGCCGCTAAAAAGGCAAAAAAATAA
>AONIPM_09740 Portal protein
ATGGCAGAAAAAAAGATGACTGACTGGCATCGCAAGGTGCTGTGCAACTTTGATAATGCCTGGTCAGCAACGCAGGATATGCGTGAGCAGATTATTGAGGCTCAACGTTTCGTCCGGGTGT
CCGGCGCACAGTGGGAAGGCAGCACAAACGCTGGTTACTCATTTGATGAAGGCAGGTTCGAGCATTATCCGCGTTTTGAATTGAATAAGATTGCCCGTGAATGTGATCGCATCATTGGCGA
GTATCGACAGAATCGCATCAGCGTTAAATTCAGGCCGAAGGATGACAAGGCATCGGAAGCGTTAGCCGAAAAGATGAACGGCAAATTCCGCGCTGACTATCAGGAAACATCCGGTGGCGA
AGCGTGTGATAACGCATTTGATGATGCTGTAACGGGCGGATTCGGTTGTTTCCGCATGTGTGCCGATTACGAAGATGAAATGGATCCGAGTAACGAGCAACGCCGTATAAGCCTTCTTCCTG
TTTACGACCCAGCGACATGCGTCTTCTTCGATCAGGACAGCAAGCAATATGACCGCTCTGATGCTATGTGGGCTATGGAAATGTTCTCCATGACGCCTAAAGCGTTCGAGGCTGAATACCCTG
ATTCCATCGCGGCAAGCCTTTCTCGTGATGACACTGGCACTCAATATGACTGGTCAACGCCAGATGCCATCTATGTTGGTCGCTACTACGAAGTTCGCATAGAGAAGGTGAAGCTCACGGCAT
GGCGCAACCCTGTTAGCGGAGAAACGGCAATCTATGATGAAGAGCAAATCAAAGATATTGTCGACGAGCTGACCGATGGCGCATTCGAACTGATTGGTGAGCGGACAGTGAAGAAACGCA
GAGTTTATTGCGGTCTTCTGTCTGGCGCTGAATGGCTGGAAGAACCGAAGCGTATTCCGGGCGAACATATTCCTCTCATCCCGGTATATGGGCGTCGCTCATTTGTTGATAATCAGGAGCGAA
TAGAAGGCCACGCAGCAAAAGCGATGGATGCACAGCGTCTTGAGAACCTGATGGTTTCCATGATTGCAGATAACGCTACTCAGGCTGGCGGTGATGGCATTCCTGTAGTTGATGTTGACAT
GATTCCTGGTCCTCTTGCCACTCATTGGGCGGAGCGCAACAAAAAGCGCCCGGCGTTCCTGCCGATGGTCAGTCTGAAAAACAAAAACGGAGATATTACTGCGCAGGCTCAGGTCAGCAG
TTATACACCTCCGACACAAATGCCGCCAGCTCTTGCCGGGCTATTGCAGTACACCGGAACGGCTATTCAGCAAATTACAGGTGCGTCGCAGCTTGAGAACATGCCGAGCAACGTCGCTACCG
ATACCGTTGATAGCATCTTTAACCGGATGGACACGCAGTCCTATATCTACATGGACAACATGGCTAAATCCATGCGCCGTGCTGGCGTCGTGTGGCTTTCTATGGCACGTGAGGTCTATGGTAG
TGATACGCCGATGCGTATCGTTAATGAGGACGGCAGCGATGACGTGGCGCTGATGACTGGTGAAGTGGTTGACCGTCAGACAGGGCAGGTTATCGCGCTTAACGACCTTTCTCAGGGTAAC
TATGAAGTGACTGTCGATGTCGGTCAGTCGTTCGCTACTCGCCGTGACGCAACGGTTAAGTCGTTACTTTCCATGCTGGCACTTATCCCGCCAGGAACGTCGAAACACGACCTTGTATCGTCG
ATGATCCTCGACAATATGGACGGCGAAGGGATGGACGACCTTAAAGAATACAACCGCAATCAGTTGCTTCTGTCTGGAGTTATCAAGCCGAGAACGCCAGAAGAACAGCAGATGGTTGAG
CAGGCGAAACAACAACAGGCCAGTCAGCCAGATCCGGCTATGGTTGCTGCGCAAGGTCAGCTTCTTGCTGGTCAGGCTGAATTGCAGAAAGCGCAGAACGAGCAGGCAGCCATTCAGGT
TAAAGCATTCCAGGCACAGACTGATGCTCAGGTTGCAGCGGCAAACGTTGTGAAAATCCTCGCATCTGCTGATAGCCAGCAGAAATCTGATATCCGCGAGGCTCTGAAACTGCTCGGACAG
TTCCAGCAACAGCAAGGAGACAATGCCCGTGCTGATGCAGAGCTTGTCCTGAAAAGTCAGGCACAGGGCCATGCGCAGCGCATGGACATCAGCAGCATCCTGCAAAAATCAACTCAGCAA
CAACCACAGCAGTAA
>AONIPM_09745 PBSX family phage terminase large subunit
ATGACCTCGATTAATCCTATCTTTGAACCGTTCATTGAGGCGCATCGCTACAAAGTCGCCAAAGGCGGTCGAGGTAGCGGTAAATCATGGGCAATTGCGAGACTGCTTGTTGAAGCGGCGC
GTCGGCAGCCAGTGCGTATTCTCTGTGCTCGTGAACTGCAAAACAGTATCAGCGATTCGGTAATCCGGTTGCTTGAAGATACCATCGAGCGTGAAGGGTATTCGGCTGAGTTTGAAATTCAG
CGTTCAATGATTCGTCATCTCGGAACGAATGCTGAATTCATGTTCTACGGAATAAAAAACAACCCGACGAAGATTAAATCGCTCGAAGGTATTGATATCTGCTGGGTGGAAGAAGCGGAGGC
GGTAACGAAGGAATCATGGGATATCCTGATACCAACCATCCGTAAGCCGTTCTCTGAAATATGGGTGAGCTTTAACCCGAAGAACATCCTCGACGATACCTATCAGCGATTCGTCGTAAATCCT
CCTGATGATATTTGTCTGCTGACGGCGAACTACACCGACAATCCGCACTTTCCTGAAGTTCTCCGTCTGGAGATGGAAGAGTGTAAACGCAGAAACCCGACACTGTATCGTCACATCTGGCTT
GGTGAGCCAGTAAGCGCAAGTGATATGGCAATCATCAAACGTGAATGGCTTGAAGCCGCAACCGATGCGCACAAGAAACTCGGATGGAAAGCGAAAGGTGCTGTTGTCTCTGCGCATGAC
CCGTCAGATACAGGGCCGGATGCTAAAGGTTATGCATCGCGTCACGGTTCGGTAGTTAAGCGCATTGCCGAAGGTCTGCTGATGGACATCAACGAGGGTGCTGACTGGGCTACTTCGCTGG
CGATTGAAGACGGCGCTGACCATTACCTGTGGGATGGCGATGGTGTCGGTGCAGGGCTACGCAGACAGACAACGGAAGCATTCTCCGGCAAGAAAATCACCGCCACGATGTTCAAGGGC
AGTGAATCGCCATTCGATGAAGATGCACCATATCAGGCCGGAGCATGGGCTGATGAAGTCGTACAGGGCGACAACGTTCGCACTATTGGCGATGTGTTCCGCAATAAGCGAGCGCAATTCT
ATTACGCGCTGGCTGACAGGCTGTATCTGACATATCGGGCGGTTGTCCACGGTGAGTATGCAGACCCCGACGACATGCTGAGTTTCGACAAAGAAGCGATAGGCGAGAAGATGCTGGAGA
AGCTGTTTGCAGAACTGACGCAGATTCAGCGCAAATTCAATAACAACGGGAAGCTGGAGCTTATGACTAAGGTCGAAATGAAGCAGAAGCTCGGTATTCCATCTCCTAACCTGGCTGATGC
GCTGATGATGTGTATGCATTGCCCAGAGTCGGCTGCGCAACCCGACTATTCCAGTTACTCAATTCCTTGTGGTGTAGGTTGA
>AONIPM_09750 Terminase
ATGGCACGCCCAACAAAGTATCAAGAGGCGTATGCCGAACAGGCACGCAAACTGTGCTTGCTGGGCTACACCGATGCAGAACTTGCTGATTTCTTTGAAGTCAGTGAGTCAACTATTAACA
AGTGGAAGCTTGATTATCCTGAGTTTTCGGAGTCCATAAAAAAGGGTAAGGCCGTCGCTGATGCAGAAGTTAGTGATCGTCTTTATCAACGCGCTATGGGCTTCGTGGCTCCAGATATCGATA

TTCGTGTTATTGAAAACAGAATTGTCGAAACTCCGCTTGAGAAGTATTACCCGCCTGATACAACCGCTGCCATTTTCTGGCTTAAGAACCGACAGAAGGATAAATGGCGCGACAAGGTTGAT
CACGAGCTAACAGGCAAAGACGGCGGCGCAATTCAGATTGAAACATCACCGATGAGCACTCTATTCGGAAAATGA
>AONIPM_09755 Uncharacterized protein in gp15-gp3 intergenic region
ATGGCAGAGATTATTCCCATGACTGAAGAACAGAAATTCCAGTTAGAGATTTACAAACTGGTCATGAACCAGAACGCAGCCGCAGAAGAAGCATTTCAATTCATTGGCACTGACGAGCTGA
AGCTTGAGCTATTCAAAATTCACTTCCAGTCAGGTGGCGCTAATTCAGATATCACGACCCGCACTATCGAAGCGGTGCGTAAATCGAAGGAAGCGTTAGACCTGTTCACTACCGGAGCATAA
>AONIPM_09760 hypothetical protein
ATGACCGACGCCATCAAACATGAGCGAGAGGCTCAGGGTAAGCAGATCTCCCCGCATCACTTCAGCAACGAAGCTGACCTGATTAACCGACTGGCGCTGGGCATGACGGCGGCCAAGTTC
CGCGTGCATCACGAAATCGGGAAGAAAGAGCCGATCCGCGATTACCTGACGCCGGAACAAATTCACTGCATCACCGAGCTACAGCGCGCCAACACGGTATTCATCAGCATGGGGTGGGAC
TTCGAACAACGCAAAGAAGTGCTGCGCGGCATGTTCGAGCGTAATCATCGTCAGCCGCTTATCGAAGAACAGCACCGCCTGGCGGCCTAA
>AONIPM_09765 hypothetical protein
ATGCAATTAGTTGAAATCAAGAAGCTCGACTTGGTCACTAACTCCGCTGTAATCGCTACTGGCGTCAAAAAGGATCACAAGCCTGTGATTCAGCTCATCAGGAAGTACAAAAGCGACCTCGA
AGAGTTCGGAAGGGTGGAATTTGAAATGCGACCCTTTCAAACGGATGGGGGCATGCAGAAGCAGGAAATAGCACTGTTAAACGAACAGCAAACCACGCTGTTGATCACATACATGCGAAA
CAATGAAGTTGTGCGTGAATTCAAAAAGCGCCTGGTAGCTGAATTCTTCACTATGCGTAGCGCGCTGGCGAAAAAGAAAATGGATCGCAACTCTGCACGCCTGTAG
>AONIPM_09770 Spanin, inner membrane subunit
ATGAGCAGAGTAACCGCGATTATCTCCGCTCTGGTTATCTGCATCATCGTCTGCCTGTCATGGGCTGTTAATCATTACCGTGATAATGCAATCGCCTACAAAGAGCAGCGCGATAAAAAAGTCA
GTGAACTGAAGCAGGCGACCGCCACCATTACTGACATGCAGCAGCGCCAGCGTGATGCTGATGCACTCGATGCTAAATACACGAAGGAGTTAGCTGATGCGAAAGCTGAAAATGATGCTCT
TCGGCGCAAGCTTGATAATGGTGGTCGGGTGCTCGTCAAAGGAAAATGCCCTGTGCCATCCTCAGCCGAAACCTCCAGCGCCTCCGGCATGGGCAATGATGCCACCGTCGAACTCTCTCCA
GTTGCTGGACGAAACGTTCTCGGTATCCGGGATGGAATCATCAGCGACCAAACAGTACTGAGAACGCTTCAGGAATACATCGTGACGCAATGCCTGAAATAA
>AONIPM_09775 Lysozyme RrrD
ATGGCAATGTCACCGGCACTACGAAATAGCGTAATAGCGGCGATAAGTGGCGGGGCTATTGCCATAGCATCTGTGTTAATCACTGGCCCCGGTGGTAACGATGGTCTGGAGGGTGTCAGATA
CAAACCATATAAGGACGTAGTTGGTGTGTTGACTGTGTGTTATGGCCACACCGGAAAAGACATCATGCTCGGTAAAACGTATACCGAAGCAGAATGCAAAGCCCTCCTGAATAAAGACCTTG
CCACGGTCGCCAGACAAATTAACCCGTACATCAAAGTCGATATACCGGAAACAACGCGCGGCGCTCTTTATTCGTTCGTCTATAACGTGGGCGCAGGCAATTTCAGAACATCGACGCTTCTTC
GCAAAATCAACCAGGGCGATATCAAGGGCGCATGTGACCAGCTACGTCGCTGGACATACGCTGGCGGTAAGCAATGGAAAGGGCTGATGACTCGCCGTGAGATTGAGCGTGAAGTCTGTT
TGTGGGGGCAGCAATGA
>AONIPM_09780 Holin
ATGTACCGTATGGACAAAATCAGAGAATGGTTCAGTTACAGCTTCGGAGGACTGACTGCGATGGGTGGCATTCTCTCCCTGAATGACTGGGCTGTAATCATTGGTATTCTTTGTACTGTCGGC
ACATTTGGCATCAACTGGTACTACAAACGCAAAGAGCGTGAGGACAGATTGAATGGCAATGTCACCGGCACTACGAAATAG
>AONIPM_09785 Antitermination protein Q
ATGAGACTCGAAAGCGTAGCTAAATTTCATTCGCCAAAAAGCCCGATGATGAGCGACTCACCACGGGCTACGGCTTCTGACTCTCTTTCCGGTACTGATGTGATGGCTGCTATGGGGATGGC
GCAATCACAAGCCGGATTCGGAATGGCTGCATTCTGCGGTAAGCATGAACTCAGCCAGAACGACAAACAAAAGGCTATCAACTATCTGATGCAATTTGCACACAAGGTATCGGGGAAATAC
CGTGGTGTGGCAAAGCTCGAAGGAAATACTAAGGCAAAGGTACTGCAAGTGCTCGCAACATTCGCTTATGCGGATTATTGCCGTAGTGCCGCGACGCCGGGTGCAAGATGCAGAGATTGC
CACGGTACAGGCCGTGCGGTTGATATAGCCAAAACAGAGCAGTGGGGGAGAGTTGTTGAGAAAGAGTGCGGAAGATGCAAAGGTGTCGGCTATTCAAGAATGCCAGCAAGCGCCGCATA
TCGCGCTGTAACGATGCTAATCCCAAACCTTACCCAACCCACCTGGTCACGCACTGTTAAGCCGCTGTATGACGCTTTGGTGGTGCAATGCCACAAGGAAGAGTCAATCGCAGACAATATTTT
GAATGCGGTCACGCGTTAA
>AONIPM_09790 Protein ninH
ATGAACGCCACAATTCAAACGATACCAGAACTGCTTATACAGACACGAGGCAATCAGACCGAAGTGGCGAGGATGCTTTCCTGCGCAAGAGGAACAGTGCTCAAGTACAACCGAGACAGC
AAAGGCGAGCGTCACGTAATAGTTAACGGCGTCCTGATGGTCAAACAGGGCAAGAGGGGAAGACGATGA
>AONIPM_09795 Crossover junction endodeoxyribonuclease rusA
ATGAACGAATATCAGTTTGTGCTTCCATACCCGCCGTCGGTGAATACCTACTGGCGAAGACGTGGAAGCCAATATTACATCAGCGATAAAGGCCAGAAATACCGAAAAGACGTTCAGCAAGT
CATCCTCCAACTTAAGTTAGACATTTTCACCAAATCACGACTTCGCATCAAAGTCATCGCAGACGTTCCAGACTCCCGCCGCCGCGACCTCGACAACATCCTGAAAGGTTTACTCGACTCCCT
TATCCACGCCGGATTTGCGGAAGACGACGAGCAATTCGATGACATTCGCGTAATTCGTGGTGTGAAAGTACCAGGCGGACGGCTTGGAATAAAAATCACCGAACTGGAGAACGTATGA
>AONIPM_09800 DUF1364 domain-containing protein
ATGGCTAACTTACGCAAAGAAGCGCGCGGCAGAGAATGCCAGGTACGTATTTATGGCGTATGTAATGGCAATCCTGAAACTACAGTTCTGGCACATTACCGGATGGCTGGAATTTGCGGAAC
GGGGATGAAGCCTGACGACCTGATCGGTGCATGGGCTTGTAGCGCGTGTCACGATGAAATCGATCGACGAACCCATATTCTCGACAACAAAGACGCCAGACTTTACCACCTCGAAGGCGTG
ATCAGGACGCAGGCGATACTGCTGAAGGAGGGGAAGATTAAGCCATGA
>AONIPM_09805 Site-specific DNA-methyltransferase (adenine-specific)
ATGAGTGATTCGTTTAGTAGCTCTGATTATTTGTATTTTGTTTTAGTGCCTGTGGCAGAAGTATTCCGCTCTCGATTTCCTGAAGGCTCAGCACCATTTAATGCCATTCGCACTTACTCAAAATG
CAGAGTGAAATTTACAGGTAAGCGGCTGGAAAGAGAGTGGCAGGAATTCTGCAAAAAACATGACCTAAAAAATGACCCTGAACTGGAGTATTAA
>AONIPM_09810 Protein ninF
ATGCTTAGCCCATCCCAATCCATTCAATACCAGAAAGAAAGCGTCGAGCGGGCTTTAACGTGCGCTAACTGCGGTCAGAAGCTGCATGTGCTGGAAGTTCATGTATGTGAAGCGTGCTGCGC
AGAACTGATGAGCGATCCGAATAGCTCAATGTACGAGGAAGAAGACGATGAGTGA
>AONIPM_09815 Putative protein ninX
ATGAGACGACAGCGACGAAGTATCACCGACATCATCTGCGAAAACTGCAAATACCTTCCAACGAAGCGCTCCAGAAATAAACGCAAGCCAATCCCAAAAGAATCTGACGTAAAAAACCTTC
AACTACACGGCTCACCTGTGGGATATCCGGTGGCTAAGACATCGTGCGAGGAAATGACAATGGATTATTCACAGTTAAGTGATTTTGAAATTAACAAGCGAGTGGCAATTTGCTGTGGATTT
GCTCCCGAAGATTGCGAAATCGCAAAGTTGGGAACATCAATCGTTGGTGTTGAGTGGGATGACGAAACTGGTTATGCAATAAAAACGGTTGATTACTGTAAAAGCCCATCAGACGCAGAGC
CGATTATCGTAGAGAACAGAATTGGCATTATTCCAGCGCCAGAAAATGGATTATGGAAGGCAGCGCATAGAAAAGTTGGCAGTGATAGTACCCCATATCATATGACTCAAGATGAAAACCCA
CTCCGCGCTGCCATGATTGTCTTTCTCATGATGCAGGACGCCAATAATGCTTAG
>AONIPM_09820 Recombination protein NinB
ATGAAGCAAACAATCTTCCTCCGTGGTAAACAACAACAGCAAGCCGCAATCAACGCCATCCTCGCAACACCACTCGATAAAGACAAGCCAGTTACCATCCGCATTACTGACTACAAGCGCAA
CCTTGACCAGAACGCAAAATTTCACGCGATGCTGGCGGATATCGCTCGTCAGGTTCAATGGTGCGGCAAATGGTTAAAACCGGAACAATGGAAGGTTTTGTTAATTAGCGGTCATGCAGTG
GCAACAAAACAGGAAGCTGATGTTTTGCCCGGCCTTGAAGGTGAATACGTCAACATTCGCGAAAGCAGCGCACAGATGAGCGTGAAGCGCATGGCAAGCCTGATTGAGTACACGACAGC
ATGGGCTATTGGTCAGGGTGTCAGATTTACCGACAGGAGGTACGAATGA
>AONIPM_09825 hypothetical protein
GTGAGCAAGTACGAAAAATTAGATCAAAACATTCTTTCAATGCTGAGTGAAAGACCAACACCTGTTTTTGATATCTGGCTTAAATGGCGGAGCAATGGAATGTATATCGAAACCATCGATCGT
CGTATGCAATACCTGAGAAAGAAAGGGCTTGTTGCAAATGTGCGTGGGAAGGGTTGGGTGAAAATTAACCTGTCATAA
>AONIPM_09830 Prophage protein
GTGGCTGACTGGCAAATTCCAATCATCATTCTTGCCGGAGCTTCGCTGGTTGCTGGCTTTATCCTGCTGAAGAAGCATAAAGACCGTGATCAAAAAGTCGAAGTTCTCTATGGGTATCCAGC
GAACAGCACAACATGGCTGACCATTTACCACTACCGAAAATCAGGCCGCTGGGTATTCGAATGGGATGATCTGTTCGCTGAAAAGCGACCAAAGTCATGGGGAGACATCAGCGAATGCATG
ATGTTTGAAGAAAGAAAATCCGGCGCAACCCGAGAAGAGTTTAACGAAGCGTGGGCGCGATTAAGTGAGAGAGGGTATTTGTGA
>AONIPM_09835 Phage protein

GTGGCTGAGTTAATTTTCTCTGCATTGATGATTCTCGGTGCCATGTGGATGGTGGCGACGTTCATTGTTGTTGCCAGCAGTTTTGTCCGGTTGGTAGGCGAAGGTAAAGACTCGGTGGGTGT
GCTTTTCGGTAGCATTTTCCTGTGGGTGATTATCGGTGTTATGCCTGTTGTCGTAGCAAAAGTGGCGTGGCGTTTTGTGAGTTGA
>AONIPM_09840 DNA helicase
ATGAGACAGGATATCGAAGCGAGCGTTATCGGTGGCCTGCTGATTGGTGGATTAACTCCAACCGCCAGCGACGTTCTGGCAACTCTGGAGCCGGAAGCGTTTTCAATTCCGCTCTACCGGA
AAGCCTTCGAGGTTATCCGCAAGCAGGCGAGAAACAGAAACCTAATCGACGCGCTGATGGTTGCCGAGGCGTGCGGAGAGGAGCATTTCACGTCAATCCTGATGACCAGTAAGAACTGCC
CGAGTGCCGCAAACCTGAAGGGATATGCCGGAATGGTCGCGGATAACTATCACCGCCGCCTGGTGCTGGAAATCATGGATGAAATGCGTGAACCAATTCAGAACGGAACCATCGATACATC
GAGTCAGGCGATGGACGAGCTGGTAAAGCGTCTTTCAGCCATCAGAAAGCCACGTGACGAGGTAAAACCTGTACGGTTAGGGGAAATCATCACCGACTACACTGACACGCTTGACAGGCG
TCTGAGGAACGGAGAAGAGTCAGATACCCTGAAGACCGGAATCGAAGAACTTGACGCCATCACCGGAGGGATGAACGCGGAAGACCTGGTGATAATCGCTGCTCGTCCTGGTATGGGGA
AAACCGAACTGGCGCTGAAGATTGCCGAAGGCGTTGCAAGCCGCGTTATTCCTGGTTCTGACGTCCGGCGCGGAGTGTTGATTTTCTCGATGGAAATGAGCGCATTGCAGATTGCAGAGC
GAAGCATTGCCAACGCCGGGAGGATGTCGGTTAGCGTGCTGCGAAATCCTGCATCGATGGATGACGAAGGCTGGGCGCGCGTTGCTAACGGCATGAGTCAGCTTGCAGATTTGGATGTAT
GGGTAGTCGATGCCTCGCGGTTATCGGTCGAAGAAATACGCTCAATCGCAGAACGGCACAAACAGGAAAATCCAAACCTGTCACTCATCATGGCGGATTATCTTGGCCTGATTGAGAAGCC
GAAAGCAGATCGCAACGACCTTGCAATTGCTCACATCTCCGGAAGCCTGAAGGCGATGGCGAAAGACCTGAAAACGCCTGTTATCTCCCTGAGTCAGCTTTCGCGCGATGTTGAGAAGCG
ACCAAACAAACGCCCGACAAACGCAGATTTGCGTGATTCAGGAAGCATTGAACAGGACGCAGACTCAATCATCATGCTCTATCGGGAAGCTGTATATGACGAGAACAGTAGCGCCGCGCCA
TTTGCTGAAATCATTGTGACGAAAAACCGTTTTGGCTCGCTTGGTACGGTTTACCAGCGGTTCTGCAACGGACACTTTGTTGCATGTGACCAGGATGAAGCCAGACAGATTTGCACAGCATC
AAATGCACCTGCTGCGCGTGGCAGACGATATGCACAAGGGGCTGACGTATGA
>AONIPM_09845 Phage-rep-O domain-containing protein
ATGGGCGTCGTTAAGTTAGCTGATTACAGGCCTCAACTGGAGGTCGTGGAGCATCGCGTGGCAGATACCGAAGATGGTTTCATGCGCGTTGCTAACGAGATTACCGACAGTCTGCTGATGG
CTGATTTAACCGTCCGGCAGTTGAAGGTGATGCTCGCTATCATGCGCAAGACATACGGATTCAATAAGCCGATGGATCGACTCACAAACACGCAGATAGCAGCCATGACAGGTATTCATCAC
ACTCATGTTTGCGCTGCCAAGCGCCAGCTTATTGAGCGTAAATTCCTCATTGCTGATGGCGTGAAAATCGGAGTGAACAAGGTGGTTTCTCAGTGGATTAGCCAGGACAGCTTAACATTAGC
TAAAACAGCTAATAAAACATTAGCCGACTCGGCTAATGGGTATAAGCCAAGTCAGCTAAACACAAAAGACAATATACAAAAGACAATAAATACAAATACCCCCTTACCCCCTAACGGGGGTG
GCGATGGGCAGGTTAAACCTGAACGTCGCAAGGCAGAACGAATCGACTACGAATCCTTCCTGAACGCCTACAACACCGAAGTCGGTGACAGACTTCCACACGCTGTTGCGGTCAACGAGA
AACGAAAACGCCGCCTGAAGAAAATCATCCCGCAACTGAAAACGCCAAACGTGGACGGTTTCAGAGCGTATGTCAGGGCGTTTGTGCATCAGGCCAAGCCGTTTTACTTCGGAGACAACG
ACACGGGCTGGACGGCAGATTTTGATTACCTGCTGAGGGAAGATTCGTTAACGGGAGTACGGGAAGGGAAGTTTGCAGACAGGGGGATTGCATGA
>AONIPM_09850 Transcriptional regulator
ATGGTCAACGAGGCTATTAAAGCGGCTATTGACTCAGTAGGAAGTCAGCAAAAGTTAGCTGATGCCTGCGGTGTTAAGCAGCCGTCTGTATGGGCTTGGTTGCATGGGAAGAAAAGGGTAT
CCGCTGAAAATGCCAAGCGCATTGAAATGGCTACCAATGGAAGCGTCCCTGCATACCTGATTCGCCCTGATTTATCCGCTTTGTTCCCCAATCCGAACAAAGCAGCTTAA
>AONIPM_09855 HTH-type transcriptional regulator RdgA
ATGGATCAGGCTGGGTTTACTCAGGCTTCTCTTGGCAATGCTGTTGGCATGTCTCAGCCAAGCGTCTGGAAACTTACGTCTGGAAAAACACGCAATACGCGCAAACTTTTTGAAATATCAAA
AGTGCTTGGAGTTCGTACGGAATGGCTTTCCGATGGAACTGGGCCAATGCGTGATGAGGGAGTTGAACCTTATAATCCAAAATCTTCTATTCCTCATGAAAGCACGTGGGGATATTTGGACC
CATGGGATGGAGGAACGCCTTTAAGAGGTGATGAAGTTGAAATTCCTTACCTTAAAGATATTGAGTTTGCATGCGGGGATGGTCGGGTGATTGATGAAGATCACAACGGCTTTATGTTGCG
CTTCTCCAAATCAACCCTTCGCAGAGTTGGCGCGAACAGTGATGGAAGCGGTGTTGTTTGTTTTCCGGCTCGTGGCAACAGCATGGAGCCAAACATTCCTGATGGAACAACAGTTGCTGTT
AACACCAACGATAAGAAAATAGTTGACGGAAAGATTTACGCCATTAACGAGAACGGTTGGAAACGCATTAAGATTCTCTTTCGATCAGGGCCTGACAAGGTAAGCATTAGAAGCTTTAACT
CACTGGAATACCCACAAGAAGAAAAGAATCTAAGCGATATCGAGATCATCGGAAGAATCTTCTGGTGGTCTGTAGTTGACTACTAA
>AONIPM_09860 Persistence and stress-resistance toxin PasT
TTGAAGAGTGCTACAGTTTACACGGCAATGAGAGTTAAGCAATATGGCGGCGTACCTTCGGTAGAGGTAAGGTGTGATGATAAAAGAACCAAAGTTGTTACTGATTGCCTTCTGCTTTTTAA
AAGCATAGATGAGGTAATTTTTATTGGCGCTGAAAATCTTCACCCTTTGATAAAAGCAGAGATGAAGCAATGTGCAATCGATGCATTGGGAGTTGGGCAAGGCAAGTCTCAGATCGAAGCA
AAGCAAATGCTTACAGAGCTTGAAAGCAGCAGACGTAGACAAGCAAGCAGGGCAAGACAATTTCACGATGCGATCGCAGGATGTAGCAGAGAGCTTATGTCTTTAAATGTAGACATACAG
AGAGGAATTGATATTCCAACAATAAGGTCAAGAATAGGTACGATCGCAGAGAACATGGAAAAGCTAAATCCAAAGAAATAA
>AONIPM_09865 hypothetical protein
ATGGACAAGAAAATTAAATCATTGAAGCCAGGAATTGTTATCAGAGACATAAGTGGTGATTACGATACAGAGACATATGATATTTTATATGTGCATGCAGATGGAAAATGTCAGTATTCGAACG
ACATTTTAATAATAAAGGTGGTGCCGAAATTGCTGCCACCACAGTTAATAAAGAGTTAG
>AONIPM_09870 hypothetical protein
ATGCGGCCTGTGATGTTAGATTTACTTTCATTTTTGAACGGCAGCTACAATTATGATGGAATGTACAAAACCTCTTTCCAGTTCATAGAAGTGGATGATGGCATAAAATAA
>AONIPM_09875 hypothetical protein
ATGCCAATAAATAAACATAAGGAACTTCCCATGATGCAACTCAGCTTTGCGGGAAGCGGCGTCATGTCCGCTTTTTACCCCGCTGAATCCGAATTATCAAAAACGTATTCGTCGCCTTATTCGC
TCTGTCCGTAA
>AONIPM_09880 hypothetical protein
ATGCAAAGCCAAATAACAATCAATCACCAGAAGTTAATTGCAGCACAAAGCAAGGCTGTTATCGCTCGTTTTCTTGGTGACGGACACATGTGGAAACAAGCTACCGAGGAAATGAAATCAG
CAATCAACTTTCCATGGTACCGCAAGAAATGA
>AONIPM_09885 hypothetical protein
ATGAGCATCGCTGACGCATGGACAGATGATGCTTTTATCCGATTAATGCAGGACATGCTCAATCAGCAGAAAGAACAGGAGAACGATGATGATTCTGACTCTGAATGA
>AONIPM_09890 Prophage protein
ATGATTCTGACTCTGAATGATAAGCGTGAAATATCGCAAATCATCGCAAGTTTTACCGATGATGATTATGAACGAATTAACAGTGAAGTTGATCGCCTTTGCAAACGTTGCGACCCAATAAGCG
AAATGCTTCGCTCATATAAACCAGATGAGCACACTAAGGACGCTATCGACTGGCTGGAAGATGATGATTGTAACTACCAGGAAAAAGCCGCTGAATGGTTCTGGGATGCAATAACCGATAGA
GTTAAGGCTGAATATGCCTTCGCAATATTCAAACGCAGACACATTTATGGAGAAGCAGCATGA
>AONIPM_09895 hypothetical protein
ATGAGCAATATCGTTGAATTCGTTAAACAGCAGGAGCACTTATTCTGCGGGGCATTGACTGAACAGACGGTAACATGGGCTAAGGAAAGCCAGTTTGCAATTCAGTATTTCCAGAAAAACG
ATTACCTGGCTAAAACGGCACTGGCAAATCCAACCAGCGCACAGAACGCCATCATCAATGTTGCCGCCATAGGAATCACATTAAACCCGGCGAGCAAACTTGCTTACCTTGTGCCACGTGAT
GGCATGGTTTGCCTTGATATCAGTTACATGGGATTACTTCACCTTGCACAGTCGACAGGCTCAATTAAGTGGGGGCAATGCAAACTGGTGTACTCAAACGACACCTATGAATCAAACGGCCT
TGATTCAGCACCAACCCACAAATACAACGCATTTGGTGAGCGAGGCTCTATTGTTGGTGGTTATTGCACGGTTAAAACAGCAGATGGTGACTACCTGACTGAAGAAATGAGTCTGGCAGAA
ATTAAAGCTGTGGAAGCAACGAGCAAGGCAAAGAATGGACCGTGGAAGACATTCTGGGAAGAGATGGCGCGCAAAACAATAGTTAAAACGCGCCAGCAAATACTGGCCTAA
>AONIPM_09900 hypothetical protein
ATGCCGCACAAATCAGAGGAAGATATCCGCGAAGATGAACGGAAACGCCAGCAGGAAATAATGGATAAAGCACAACTTCTTTGTAATGAAATGGCTCAGGCTGAAAACATGGATGATTTGA
AGCGATATTTTGCAGAAGCATATCGCCTGACATCTGGAATGAAATTGCAGCAGAACGTACAAGCAATTTACGCAGAATGCAAAGCGAAACTGGAGGTTGCCAGTGAGCAAACTGTATGA
>AONIPM_09905 hypothetical protein
GTGAGCAAACTGTATGAAATAGCTAATGAATACGCAAAATTGATGGATTCAGATTTAGAACCAGAGATGATTGCTGACACAATAGAAGGCATGGAAGGAGAATTTACCGATAAAATAGAGCA
ACTTCTTGCCATTATTAAAAATGAGTCTGGCTATGCTGAGCGCCTCAAGGAAGAGGCAAAGTCACTGAATGAACGAGCAGCAGTAATTCAAAATAAGATTGACAGCATTATGGCATATATAGC
GTCATCGCTTGAAATGGTTGGCAAGAAAAAGATTCGAGCTGGTATTCACCAGTGA
>AONIPM_09910 hypothetical protein

ATGAAAGAAGTGAAAATATACACGATTGTCAGTGACCAGTTATCACCACCAATAACAGGAGAATCATTCTGTACTGACATGGTGCGTCATAGTGATTATGCGGAGCTTGAGGATAAATACGCG
GCGTTGGCTGCGGATAACGATAAAGCAATGGAGTCACTTAAGCAGGCTGATGCAGTTGTTAAGTTGGCACACGAGAAGTTTTCGGCGCTGGCTTCGGAGAATGCGGCACTGAAAAAATCA
GAGGTCGAATTCAACGAATATTGTCGTCGCGAGTGCGAGGACGTTGGCGATACGTGGGTGGACGATTTCACTGAGACCCCAGCCACCGACACTTTTCTGGCTGAGGTGACACAAGAAGCA
GTAGAGTCACTCAAGAAGGAGATTGAATGGCTCAAAAAGCAGTTGCTTATGTCTGTCGGTGATATTCGAGACCTTATTGATTACAACCCTGAAAATGGCGTTCTTACAGCGAAAGTTAACTTC
AGCGGAAGGCAGTCGTTCATGGCTTAA
>AONIPM_09915 hypothetical protein
ATGGCTAACTTACGCAAAGAAGCGCGCGGCAGAGAATGCCAGGTACGTATTTATGGCGTATGTATGGCAATCCTGAAACTACAGTCTGGCACATTACCGGATGGCTGGAATTTGCGGAACG
GGATGAAGCCTGACGACCTGATCGGTGCATGGGCTTGTAGCGCGTGTCACGATGAAATCGATCGACGAACCCATATTCTCGACAACAAAGACGCCAGACTTTACCACCTCGAAGGCGTGAT
CAGGACGCAGGCGATACTGCTGAAGGAGGGGAAGATTAAGCCATGA
>AONIPM_09920 DUF2591 domain-containing protein
ATGGATTATTCACAGTTAAGTGATTTGAAATTAACAAGCGAGTGGCAATTTGCTGTGGATTTGCTCCCGAAGATTGCGAAATCGCAAAGTTGGGAACATCAATCGTTGGTGTTGAGTGGGA
TGACGAAACTGGTTATGCAATAAAAACGGTTGATTACTGTAAAAGCCCATCAGACGCAGAGCCGATTATCGTAGAGAACAGAATTGGCATTATTCCAGCGCCAGAAAATGGATTATGGAAGG
CAGCGCATAGAAAAGTTGGCAGTGATAGTACCCCATATCATATGACTCAAGATGAAAACCCACTCCGCGCTGCCATGATTGTCTTTCTCATGATGCAGGACGCCAATAATGCTTAG
>AONIPM_09925 Protein ninE
ATGAGACGACAGCGACGAAGTATCACCGACATCATCTGCGAAAACTGCAAATACCTTCCAACGAAGCGCTCCAGAAATAAACGCAAGCCAATCCCAAAAGAATCTGACGTAAAAACCTTCA
ACTACACGGCTCACCTGTGGGATATCCGGTGGCTAAGACATCGTGCGAGGAAATGA
>AONIPM_09930 hypothetical protein
ATGCTGGCGGATATCGCTCGTCAGGTTCAATGGTGCGGCAAATGGTTAAAACCGGAACAATGGAAGGTTTTGTTAATTAGCGGTCATGCAGTGGCAACAAAACAGGAAGCTGATGTTTTGC
CCGGCCTTGAAGGTGAATACGTCAACATTCGCGAAAGCAGCGCACAGATGAGCGTGAAGCGCATGGCAAGCCTGATTGAGTACACGACAGCATGGGCTATTGGTCAGGGTGTCAGATTTA
CCGACAGGAGGTACGAATGA
>AONIPM_09935 Phage protein
GTGGCTGAGTTAATTTTCTCTGCATTGATGATTCTCGGTGCCATGTGGATGGTGGCGACGTTCATTGTTGTTGCCAGCAGTTTTGTCCGGTTGGTAGGCGAAGGTAAAGACTCGGTGGGTGT
GCTTTTCGGTAGCATTTTCCTGTGGGTGATTATCGGTGTTATGCCTGTTGTCGTAGCAAAAGTGGCGTGGCGTTTTGTGAGTTGA
>AONIPM_09940 hypothetical protein
ATGAGACAGGATATCGAAGCGAGCGTTATCGGTGGCCTGCTGATTGGTGGATTAACTCCAACCGCCAGCGACGTTCTGGCAACTCTGGAGCCGGAAGCGTTTTCAATTCCGCTCTACCGGA
AAGCCTTCGAGGTTATCCGCAAGCAGGCGAGAAACAGAAACCTAATCGACGCGCTGATGGTTGCCGAGGCGTGCGGAGAGGAGCATTTCACGTCAATCCTGATGACCAGTAAGAACTGCC
CGAGTGCCGCAAACCTGAAGGGATATGCCGGAATGGTCGCGGATAACTATCACCGCCGCCTGGTGCTGGAAATCATGGATGAAATGCGTGAACCAATTCAGAACGGAACCATCGATACATC
GAGTCAGGCGATGGACGAGCTGGTAAAGCGTCTTTCAGCCATCAGAAAGCCACGTGACGAGGTAAAACCTGTACGGTTAGGGGAAATCATCACCGACTACACTGACACGCTTGACAGGCG
TCTGAGGAACGGAGAAGAGTCAGATACCCTGAAGACCGGAATCGAAGAACTTGACGCCATCACCGGAGGGATGAACGCGGAAGACCTGGTGATAATCGCTGCTCGTCCTGGTATGGGGA
AAACCGAACTGGCGCTGAAGATTGCCGAAGGCGTTGCAAGCCGCGTTATTCCTGTTCTGACTACGTCCCGGCGCGGAGTGTTGATTTTCTCGATGGAAATGAGCGCATTGCAGATTGCAGA
GCGAAGCATTGCCAACGCCGGGAGGATGTCGGTTAGCGTGCTGCGAAATCCTGCATCGATGGATGACGAAGGCTGGGCGCGCGTTGCTAACGGCATGAGTCAGCTTGCAGATTTGGATGT
ATGGGTAGTCGATGCCTCGCGGTTTATCGGTCGAAGAAATACGCTCAATCGCAGAACGGCACAAACAGGAAAAATCCAAACCTGTCACTCATCATGGCGGATTATCTGGCCATGA
>AONIPM_09945 HTH-type transcriptional regulator RdgA
ATGAAACTAGATACTTTTTCTCAAAGGCTTACATACGCGATGGATCAGGCTGGGTTTACTCAGGCTTCTCTTGGCAATGCTGTTGGCATGTCTCAGCCAAGCGTCTGGAAACTTACGTCTGGA
AAAACACGCAATACGCGCAAACTTTTTGAAATATCAAAAGTGCTTGGAGTTCGTACGGAATGGCTTTCCGATGGAACTGGGCCAATGCGTGATGAGGGAGTTGAACCTTATAATCCAAAATC
TTCTATTCCTCATGAAAGCACGTGGGGATATTTGGACCCATGGGATGGAGGAACGCCTTTAAGAGGTGATGAAGTTGAAATTCCTTACCTTAAAGATATTGAGTTTGCATGCGGGGATGGTC
GGGTGATTGATGAAGATCACAACGGCTTTATGTTTGCGCTTCTCCAAATCAACCCTTCGCAGAGTTGGCGCGAACAGTGATGGAAGCGGTGTTGTTTGTTTTCCGGCTCGTGGCAACAGCAT
GGAGCCAAACATTCCTGATGGAACAACAGTTGCTGTTAACACCAACGATAAGAAAATAGTTGACGGAAAGATTTACGCCATTAACGAGAACGGTTGGAAACGCATTAAGATTCTCTTTCGAT
CAGGGCCTGACAAGGTAAGCATTAGAAGCTTTAACTCACTGGAATACCCACAAGAAGAAAAGAATCTAAGCGATATCGAGATCATCGGAAGAATCTTCTGGTGGTCTGTAGTTGACTACTAA
>AONIPM_09950 hypothetical protein
ATGGACAAGAAAATTAAATCATTGAAGCCAGGAATTGTTATCAGAGACATAAGTGGTGATTACGATACAGAGACATATGATATTTTATATGTGCATGCAGATGGAAAATGTCAGTATTCGAACG
ACATTTTTAATAATAAAGGTGGTGCCGAAATTGCTGCCACCACAGTTAATAAAGAGTTAGTAGCTAACGAGTCGTGGGATTATTTTATGCCATCATCCACTTCTATGAACTGGAAAGAGGTTTT
GTACATTCCATCATAA
>AONIPM_09955 hypothetical protein
ATGCCAATAAATAAACATAAGGAACTTCCCATGATGCAACTCAGCTTTGCGGGAAGCGGCGTCATGTCCGCTTTTTACCCCGCTGAATCCGAATTATCAAAACGTATTCGTCGCCTTATTCGCT
CTGTCCGTAAGCAACTGGAGGCGTTATGCAAAGCCAAATAA
>AONIPM_09960 hypothetical protein
ATGCAAAGCCAAATAACAATCAATCACCAGAAGTTAATTGCAGCACAAAGCAAGGCTGTTATCGCTCGTTTTCTTGGTGACGGACACATGTGGAAACAAGCTACCGAGGAAATGAAATCAG
CAATCAACTTTCCATGGTACCGCAAGAAATGA
>AONIPM_09965 hypothetical protein
ATGAGCATCGCTGACGCATGGACAGATGATGCTTTTATCCGATTAATGCAGGACATGCTCAATCAGCAGAAAGAACAGGAGAACGATGATGATTCTGACTCTGAATGA
>AONIPM_09970 Prophage protein
ATGATTCTGACTCTGAATGATAAGCGTGAAATATCGCAAATCATCGCAAGTTTTACCGATGATGATTATGAACGAATTAACAGTGAAGTTGATCGCCTTTGCAAACGTTGCGACCCAATAAGCG
AAATGCTTCGCTCATATAAACCAGATGAGCACACTAAGGACGCTATCGACTGGCTGGAAGATGATGATTGTAACTACCAGGAAAAAGCCGCTGAATGGTTCTGGGATGCAATAACCGATAGA
GTTAAGGCTGAATATGCCTTCGCAATATTCAAACGCAGACACATTTATGGAGAAGCAGCATGA
>AONIPM_09975 DNA recombinase
ATGAGCAATATCGTTGAATTCGTTAAACAGCAGGAGCACTTATTCTGCGGGGCATTGACTGAACAGACGGTAACATGGGCTAAGGAAAGCCAGTTTGCAATTCAGTATTTCCAGAAAAACG
ATTACCTGGCTAAAACGGCACTGGCAAATCCAACCAGCGCACAGAACGCCATCATCAATGTTGCCGCCATAGGAATCACATTAAACCCGGCGAGCAAACTTGCTTACCTTGTGCCACGTGAT
GGCATGGTTTGCCTTGATATCAGTTACATGGGATTACTTCACCTTGCACAGTCGACAGGCTCAATTAAGTGGGGGCAATGCAAACTGGTGTACTCAAACGACACCTATGAATCAAACGGCCT
TGATTCAGCACCAACCCACAAATACAACGCATTTGGTGAGCGAGGCTCTATTGTTGGTGGTTATTGCACGGTTAAAACAGCAGATGGTGACTACCTGACTGAAGAAATGAGTCTGGCAGAA
ATTAAAGCTGTGGAAGCAACGAGCAAGGCAAAGAATGGACCGTGGAAGACATTCTGGGAAGAGATGGCGCGCAAAACAATAGTTAAACGCGCCAGCAAATACTGGCCTAAAGCCCAGCG
ACTGGATAATGCCATTCACCTGCTTAACGAAGATGAAGGTATGCATCAGGAACCGGTTATGCCGCACAAATCAGAGGAAGATATCCGCGAAGATGAACGGAAACGCCAGCAGGAAATAATG
GATAAAGCACAACTTCTTTGTAATGAAATGGCTCAGGCTGAAAACATGGATGATTTGAAGCGATATTTTGCAGAAGCATATCGCCTGACATCTGGAATGAAATTGCAGCAGAACGTACAAGC
AATTTACGCAGAATGCAAAGCGAAACTGGAGGTTGCCAGTGAGCAAACTGTATGA
>AONIPM_09980 hypothetical protein
GTGAGCAAACTGTATGAAATAGCTAATGAATACGCAAAATTGATGGATTCAGATTTAGAACCAGAGATGATTGCTGACACAATAGAAGGCATGGAAGGAGAATTTACCGATAAAATAGAGCA
ACTTCTTGCCATTATTAAAAATGAGTCTGGCTATGCTGAGCGCCTCAAGGAAGAGGCAAAGTCACTGAATGAACGAGCAGCAGTAATTCAAAATAAGATTGACAGCATTATGGCATATATAGC
GTCATCGCTTGAAATGGTTGGCAAGAAAAAGATTCGAGCTGGTATTCACCAGGTGACAATCCGCAAACCGACAGAAACTGTAGAAATCATCGATTCCAGCGCCCTTCCTCCAGAATACGTTG
AGTTTGAAACAACAATTAAAGCCGACAAACTGGCAATTAAACACCAACTAAAAGCTGGAATAAATATCCCCGGCGCTTACCTCAAAGTTGGAAAAACTTCACTTCTTATCAAATAA
>AONIPM_09985 hypothetical protein

ATGAAAGAAGTGAAAATATACACGATTGTCAGTGACCAGTTATCACCACCAATAACAGGAGAATCATTCTGTACTGACATGGTGCGTCATAGTGATTATGCGGAGCTTGAGGATAAATACGCG
GCGTTGGCTGCGGATAACGATAAAGCAATGGAGTCACTTAAGCAGGCTGATGCAGTTGTTAAGTTGGCACACGAGAAGTTTTCGGCGCTGGCTTCGGAGAATGCGGCACTGAAAAAATCA
GAGGTCGAATTCAACGAATATTGTCGTCGCGAGTGCGAGGACGTTGGCGATACGTGGGTGGACGATTTCACTGAGACCCCAGCCACCGACACTTTTCTGGCTGAGGTGACACAAGAAGCA
GTAGAGTCACTCAAGAAGGAGATTGAATGGCTCAAAAAGCAGTTGCTTATGTCTGTCGGTGATATTCGAGACCTTATTGATTACAACCCTGAAAATGGCGTTCTTACAGCGAAAGTTAACTTC
AGCGGAAGGCAGGCAGGTTCTGTCATTGGCTCTCAAACTTGGCAGGGATATTACGCATTCTCACTGTTCGGTAAGAAGTGCTTTGCTCACAGACTTGCATGGCTACTGCATTACGGTGAGTG
GCCTTCACAACCTATCGATCACATAAACGGAATTAAGACGGACAACAGCATAAGAAATCTGCGCCTTTGTTCTCTATCGCAGAACCAATTCAACAAGCCTACTCAGAAAAACAACACTACAG
GTGTGAAGGGTGTGTATTGGAACAAGCGAGATAAACGCTACGTTGCCAGCGTCCAATTTAACGGAAAGAAATACAGCGCGGGACATCACAAGGATATTGATAGCGCTAAAGAGGCTGTAAT
GAAACTTCGGGAAAAACTGGCTGGAGAATTCACTAACCACGGCGAATTCGAACTCGCCGCCCAACTTCGCAAAGGAGCCTCGCTATGA
>AONIPM_09990 ClpB-D2-small domain-containing protein
ATGAGCAATACAGCAAAACTTCAACTCGGATTCTCTCCTTTATCAAAAACCATCATGCTCGCAAAAATGCGCGATGTGGAAGGTGGCCGCCTGCGCGTCGGTAATGATCGTGGTCGTGATGT
TACCAATGAGGCTGCGCAGCTAGTGTGGCAGCTTGTCATGGCGGAAGGTGGTGAAATCGGCTGGGAGCTTGATGATGGTTCTCGCATGGTGCTGAAAGCTGAGAAGCAGGAGGCAACCA
GTGAGCAAAATTGA
>AONIPM_09995 hypothetical protein
GTGAGCAAAATTGACCATCAGGCACTGCGTGAGGCAGCAGAACGTGCAACTCCAGCAATGGAACGCCTGTTAATGTTGCCTGTTGATGATGATTTGTTAAGTGAACAGGAACTTAAAGATT
ACGGTGTGGATATTGATGCGCTCAACGCCTTCAAATTTCTGACCGGACCAGAAACCGTGCTGGCGCTGCTGGATGAGAATATTCAACTCCAGCGGGGAAAAGACGCAATAGAGGCCGTAG
CGCTGGTGCTGCGTGATGATATGCGACAGGCGCGAGAGCAACTGGCAGCCGCAGAAAAGCGGAACTCCGAACAGCGTGAGTATTACGAGGGCGTTATTGCTGATGGAGGTAAGCGCATA
GCAGAACTCGAACACAGCGAGACGCAGCTTATCAATGAGCGTGATAGTGCTGAATCTGCACTGGCCGATATGTACCAGGCCGCAACAGGAGAGCGTCCAGAATGGAGCAATATGTTTGGTT
TCGCTGACGCCGTTGATGTGGTGGAAGAACGACTGGCGACGCTGGAGGCCAACCAAAGCCAAACCACGCCAACGGGAATTCAGCTCATCACAGAAGCCATAGGTGCGCACGGCTATATCG
TTGGCTGCCTGTTGCAAGGTCGCCCTGATTTGGCGCTGGAAGAATCGAGAAAGTGGGTATCCGCTTTCGGTCAGGCGGCGGAAATAGTTAGTGCACAAGACGCCGATGACATCAAGGTTA
AGGGGGAGTGA
>AONIPM_10000 Prophage protein
ATGACCACTATTACCAGAGAACAGCAAAAACAGATTTTAATTGATACAGCGAACCACGTAATCAGTCGTGATAACACGTCACCGTATAGCGAAAACCTGCGTGAACTGGCGCGTATCGCGCT
GGCATCGCTCGAAGCGGAGAAAGGTGCCGACCCCGTTGTGTTCACCGACGAACGAAATCTTCATCATATTGCCAGGGGTCGAGAAACCTCTTTGATTTGGGGTAAACAAAACCAGGAGGT
GGGGGATATCCCGCTCTATCGTCACGCCCAGCCAGTGCCGGTAGTGCCTGATGAAATGGCGACATCTGATGACATGAATCTTTATCAAAAGAGCTTTGCGCAAGGCTATAACGCCTGCCGCA
ATGCCATGCTCAACGGAGGTAAATCGTGA
>AONIPM_10005 DUF551 domain-containing protein
GTGAAAGAGAATCAAATCCGGGAGCTTGTAAACGAGCTGCATGATATTGCTATTGAGTATCACGGCACACAACAGTTACGTGAACGAATTGCGCGTACAGTTCGCGCCGCCCTGCATCATGA
CTTAGAAAAACTAAACCAACCTGTAAGCCAAACTTACGAGTTGCCAGAATTAATCGAAGGCATGGAGGTGTCCATTGATGTCAGCACTTGTGATGCTGATGCCGGGAATCGCTATTTCGGTA
CTGTCACCGAGGTATCAGAACTGGACACAGCAAAGAATGGCTACATTCTTCTGGTTCAGGACGCTGAACCAAATTTCGATGTGAATGGCAACTCTCCGGTAATTCCGGATGGCTGGATAAGC
TGTAGTGAGCGAATGCCGGATAATGATGAATCTAAACCCATCGCAATTTTTACCGGAAAATGTCTGGGTCAGGGGATGTTCGTTGCTACATACGACGATGATGGGTTCTTTGACTATTGGGAG
GGTATGGAAATTATCGGTGTAACCCACTGGATGCCGCTACCGGAGCCACCGCAGGAGGTGAACCGTGGCTAA
>AONIPM_10010 ASCH domain-containing protein
GTGGCTAACCTGCAACTTGCCGTCAAAGGTGAATACTTCGATGCCATGATTCGCGGGGAGAAAACGGAAGAGTATCGCTTGTGTAATGACTACTGGAATAAGCGAATTATGTTCCGCGAGTA
TGACCGACTGATTATCACAAAGGGATATCCAAAGCGCGACGATTCCAGTCGCAGAATTGATGTTCCGTACGACGGATATGAAATCAAGACAATCACACATCCCCACTTCGGCGATAAACTGGT
GAAGGTATTCGCGATAAAGGTGAATATCGGCAATGAATAA
>AONIPM_10015 DUF3800 domain-containing protein
ATGTCTGATTTAGCAATGAAGGTATTGAAGTGGCAAACGAAAGGCCACGTTGGCATAAGTAGCGCAACTATGGCTTCTATTGCTCTTGGGCTGGAAAAGAGCTTCTACCACGGACGGTTTG
ACGCACCAAGCGATCCTGCCGATTTGCGAAGATGCATGATGCTCGTAGATGAAATACCTGAAATTAAAGATAGCTTTCCGCTCATAGCGAAAAAGGTAAAGCGGTTTTCTCCGATTTTACGTG
AGTGGGATTCACTTATTGCTCTGCTTAAGCTTGAGCTTAAGAGGCCAGATAAGCGAGCACCAAAAACATATAAATGGATAAAAGAGCTTCTTTCTGAACAGGAGTAA
>AONIPM_10020 hypothetical protein
ATGGAATCACACAGTCTCACACTCGATGAGGCCTGTGCATTTCTCAAGATATCCCTTCCCTCCAAGTTCGATTCCCAAACCGGAGATAAAACCTATGCGCGAATTACGCGATGA
>AONIPM_10025 AlpA family phage regulatory protein
ATGCGCGAATTACGCGATGACTCGCTTGTTGACTTGAAGTTCATGATGGAGGATTCTGGAATGGGCAAGACCTTCATTTACTCAGAAATTAAGAAAGGCCGGTTGCCTGTCCCTCACAAAAT
CGGTAGCGCATCCAGATGGGTTTATGCCGACTACCAAAACTGGAAACGCAGCCACTTCTCCCCTCTCCAAAATGCATCATGA

Prophage 15

>PNNMHK_01355 tRNA-Arg(tct)
GCGCCCTTAGCTCAGTTGGATAGAGCAACGGCCTTCTAAGCCGTGGGTCGCAGGTTCGAATCCTGCAGGGCGCGCCA
>PNNMHK_01360 Prophage integrase IntD
ATGTCCATCTTCCGCAGAGGTGAAATATGGTACGCGTCCTACTCGACGCCGGGCGGGAAGCGAATTAAGGAAAGCCTTGGGACTTCCGACAAGCGGCTCGCTACTGAGCTACATGACAAG
CGCAAGGCTGAATTGTGGCGAGTAGACAGGCTTGGCGATTTTCCTGACGTAACGTTTGATGATGCCTGCATGCGCTGGCTGGAGGAAAAGGCAGAGAAGAAATCACTGAAAGATGACCG
CAGCCGTATGGCTTTCTGGCTGGCGCATTTTGAGGGAGTGCGGTTAAAGGATGTAACCGAGCAAAAGATTTACTTAGCAGTAAACAAGATGAGCAACCGCAAGCAGCTTGAGATATGGAA
AATAAAAGCTGCCGCGGCGCAAAAGAATGGAGAGCCAGTGCCAGTTTATTCAGCTAAACCGGTCACTACATCGACAAAGGCAAAGCACCTCGCACTGATGAAGGCCATTCTCCGGGCGGC
AGAACGTGACTGGAAATGGCTGGAGAAAGCGCCTGTAATCAAGGTTCCTTCTGTCAGAAACAAGCGCGTCAGATGGCTTGAGCGTGATGAGGCAAAAAGACTTATTGAAGAATGTCCGG
AGCCGTTGAAATCTGTTGTTAAATTTGCGCTGGCAACGGGACTTAGGCGGTCTAACATCATCAATATGGAATGGCAACAGATCGACATGCAGCGTCGTGTTGCCTGGGTGAACCCTGAAGA
CAGCAAGTCAAACCGCGCTATTGGCGTAGCGCTAAATGACACTGCCTGTAAGGTATTGCGTGACCAGATTGGTAAGCATCATAAATGGGTGTTCGTGCATACTAAAGAAGGCATCCGGCCTG
ATGGTTCAAAGACGCCAACCGTGAGAAAGATGCGCGTCGATGACCAGCGAGCATGGAATGCGGCTTGCCGCCGGGCTGGAATTGAGGATTTCCGTTTCCACGACCTGAGGCACACATGG
GCCAGCTGGCTGATTCAGTCCGGAGTTCCGCTTTCTGTTTTGCAGGAAATGGGGGGATGGGAGAGCATCGAGATGGTGCGCCGATATGCCCACCTTGCGCCGAACCATTTAACGGAACAC
GCGAAGCAAATTGACTCGATTTTCAGTGATGATGTCCCAAATATGTCCCATATGGAAAATAAGGAGGGAATTAAAGAGGCGTAA
>PNNMHK_01365 Eac protein
ATGTCAGGCCAGAGCAAATATTACGACTACTACATGGTAGAGGGTGAGGATGTTAAGGAGCTTATCCAGTCATACGACACCATTAACGATCAACGAAATTCAATCCTAACCACAGCCGCTGAA
AAGGTTGGCGCAATCGCATGGACTACAGCCCGTAGCTGGGGTGGAGAAGGCGGACTTCTACAAAGCTTCGTTTGGGAAAAAGGATATGAATTCCCATGCCAGATAACAATCAAACGCGAG
GATTTTTTGGACGGGAAGAGAGTTGTGATAGCGCGAGGAAAGGGAAACACAAAGGAAGGCCGCGCATACAACAAAGAGCTGGATGCAATCATGCATAACGCTAACGCCAAGCTAAAATC
CTTGCCTGAATGGAATTACTACATAACCAACCACTACGGGATTATGCGTACAGGAATTGGTGGTCAATCGGGCCGTGGACTTGGTTTCGTTATGTTATCAACGTATGGCGGTAAGCACCCGAA
GCGCAATGATTGTCTTATTTTTGCAATACCAAATAACAAAGAAGAGAGGCATGGCGAAGTTGTTATCCCTGACTGCTTCAAGAAAATAACTTACGGGAAATTCTACGACATCGCGAATGAAG
TTGAAGAAGAAGCTGTGGAGTAA
>PNNMHK_01370 hypothetical protein

GTGAAAGAGAATCAAATCCGGGAGCTTGTAAACGAGCTGCGCGATATTGCTATTGAGTATCACGGCACACAACAGTTACGTGAACGAATTGCGCGTACAGTTCGTGCAGCGATAATTCAGG
CCGGCAATTCTCCGGTAACTCAGGATGGTTGGATAAGCTGTAGTGAGCGAATGCCGGATAGCAAAACTGGCGTTCTTGTTGCTTGCGAGTTTGACAGGAAAGGTGACTGGCGAATGAAAT
GGGGAACCTACATACCATGA
>PNNMHK_01375 Prophage protein
ATGACCACTATTACCAGTAAATTCACTAAAGAGCGCCTGATTGATTGGGCTGTGGTAGCTGTAGCTGAAAGGGGGCGAGATTTAAAGGATGCCCCGGAGTCAGTAGAAGCTGCGGCGAATT
TAAAATTAGCTGAAATAGCGTTGGCAGCGCTTACCGCTGAACCTGTGCGATATCTAAATAAATTTTCAGGTACATGCGTGACGTTAGAGCAGCAGTCAAACGCTGCTGATGATGTTGCCGTGT
ATATTCCGCTTTACGCCGCCCCGCCAGCGCCGGTAGTGCCGGAAGAAATGAACTTTTCCACCGCCTGCAACTTTGTGCAAATCAACGGAATGGCGAAGGAGGATCGGGCAACTCTTGCAAT
GAGAGCATGGAACGCCTGTCGCGCTGCCATGCTCAACGGAGGTAAATCGTGA
>PNNMHK_01380 Ead/Ea22-like family protein
ATGAACATCGACAAACGTGCGCTGCGTGAAGTGGCGGAGAAGGCGACAAAAGGCCCCTGGAAGGTATTTTCTGATATCGATACTAAAACTTTTTCTATCCACACCCCGCGAGATAAGCGCT
GTGAAAACGTTATTAAGTGGGGTGGGTTTGATTGTCAACCGAATGCTGAGGCCAACGCTGAATTTATTGCTGCATTTAACCCGAAAGTCGCGCTGGCGCCGCTGGATGAGTTGGACAGCGC
CAACGGTTACGCCAGCGCATACGAGGCTGAAAAGTGGCATTACCACGGATTAGCAGAATCAGAGGGCGAGCGAGCAGACCGGGCAGAAAAGCAAGTGGAAGAATTAACGATGTGGGTT
AAACGTCTGGCCCATTCACTAAGAATCGCCAAGCCGAACAGTAAGTTACACAGCGCAGCAATGGACTATCTGAGCCATAAAGGATTAATCAGTGTGGAGGATGTATTGAGATGA
>PNNMHK_01385 hypothetical protein
GTGAAACTATACGAAATGGAAGGATTTCTTCGCGGTAAGTGCATACCGGGAGACCTGAAGGTTAACGAAACCAATGCCGAGTATCTGGTGAGAAAATTTAGCGAGGCGGAAGAAAGATGC
GCGGTACTGTCTGCAAATCTGAGCATGATTAACGACCTTATGAAAGCCGCCGAACAAGCAAACAAACTGGCGCAGGAAGCAACGGAAAATCTGGTTCAGGAGCGTAATGCGTTGGCTGCG
GAAAATGTAGCACTGAAGTCTGCACTTAACGACATCCTCCAGCCTGACGCAGCAGTACTTGAGAGAAACCACCGTGTCCGCGCACTGGATGCAATGGAGACCCCAGCCACTGACGCTTTTC
TGGCTGAGGTGCGGGCAAGTGCAATCCCTGAAGGTTACGCGCTTGTCCCTCAACAAATTTTCCTTGAGCCATCGGACATTGAGTTAATTTGTTCACAATGTGGTGACGGTCATGAATCCGGG
TACGGTGATTTTACTGACGGACTGCTGTGGGTTGGCAACATTCAACGAGACGACGGCAGCATTGTTCATGGTCTGCATATCTCGTCAGCAGATTACACAGAGGAAGGCGGTGTAACAGTTT
GCGAGTTCGCCGCCCAACCTCGTAAAGGCGGTGCGGTATGA
>PNNMHK_01390 DUF2737 domain-containing protein
ATGAGAGGTTTGTCCTACGACCCCGGCATCCTTCCATCGGAAATGATTATTCGACACCGCTTCAAGCCCATCAAAGATATTCCACGCGAAGAAATGCTGGCGAGAAATAGTTTTCCATCAGTG
AATGAAAATCGCTTTCTCAATACATGGTTTAAAGAAAGGAGTAAGAAGTGA
>PNNMHK_01395 Anti-RecBCD protein 2
ATGCCTGCACCTCTATATGGTGCGGATGACCCGCGCAACTGCTCCGGTAGCTCCAAGTCGGAGGTGCTGGAAAATATCAAAAACAATCTCGACGCGTTTCTTGCTCTGCCACCGGAAACAAA
AGCAGAACGGAAGTACCGACGCGATATACAACTCGCAGAAAAACAGGAAAAAGACCGAATAAACGAAACAGCAATCCGACCATTCCGCAAAGCCACTTACACCAAATTCATTGAAATCGA
CCCGCGCCTTCGTGATTACCGTTCGCGTTACGGCGCTATCAGCAATAACTGA
>PNNMHK_01400 Recombinase
ATGGATTTGAATAAATTCGACGACCCATTCAGTCCTGAAGATATCGAATGGCGAATACAGCAAAGCGGTAAAACACGCGATGGCAAAGTGTGGGCTATGGTGCTGGCTTATGTCACGAACA
GGGCAATCATGAAACGCCTTGACGATGTTTGCGGCAAAGCAGGATGGCGCAATGAATACCGCGATATTCCCAACAACGGTGGCGTTGAATGCGGCATATCAATCAAGATTGATTCCGAATG
GGTAACTAAATGGGATGCTGCTGAAAACACACAGGTAGAAGCCGTCAAAGGTGGTCGCTCCGGCGCAATGAAGCGTGCTGCCGTTCAGTGGGGAATTGGTCGGTATCTCTATAACCTTGA
GGAAGGTTTTGCGCAGATATCCAGTGATAAGAAACAAGGATGGCACAGGGCCAAACTGAAGGATGGAACAGGATTTTACTGGCTCCCTCCATCGCTGCCGGACTGGGCCATGCCAGCATC
AGGCAATCAACCATCACCAGAAAATACCAACCAGAAATCTCCATCGGTTGACTGCGAACAAATCCTGAAAGACTTCAGCGATTATGCAGCAACAGAAACTGACAAGAAAAAGCTAATTGAG
AGATATCAGCATGACTGGCAATTATTGGCTGGTCACGATGATGCGCAGACAAAATGCGTTCAGGTAATGAATATCAGAATAAATGAGCTTAAACAGGTGGCTTAA
>PNNMHK_01405 Host cell division inhibitory peptide Kil
ATGGATAAATCACTTATGGCTATTCAGTCTAAATTCGCAATTGCTGTTTATCTTGGTGACAAAATAATGTATCGCGAAGCTGTAGAAGCCTTTCGCGAATGGAGGTTGAAATGA
>PNNMHK_01410 hypothetical protein
ATGACATTTGCTATCGCGGGCGGTGCTGTCATGGGTATCGCACACCTTAATGAATCACTTTTAGAGCGTATCACCAGAAAATTACGGGCCGGATGGAAACGTCTGGTCGATATCCTGAATCAA
CCAGGAGTGCCGTGTAATGGATAA
>PNNMHK_01415 Superinfection exclusion protein
ATGAAATTACGTGTCTGGCATATCCCGCAAGTTCCTATGAAGCCATTCATTGTAGAAGTAGCAAGTGTTGAAGAGGGTGTGCGAGTGATGGATGCACTGGCTGATTATGACGCATTTCAGTAT
GACAACAACATCAAACCTGATTACTGCAACGCTAACGGCCTTGAGATGTGGGATGAGAGCCTTACTGACCAGGATTTGGAAGAAATGGAGCTTACGGATCGCTGGGTGGATTGGTATAGCG
AATGCCAGTGTTACGACGACCCGCGTGAATATATCGAAAGCCTGAAAGAAGAAACAACAGCCGCCGCCTGA
>PNNMHK_01420 Pentapeptide repeat-containing protein
ATGAATTCAGCAGATTTATCGAAGATTCTTGAAGAACACAAAGTGTGGATTACCTCAATGCGTGAGAGCGGTTCGAGAGCCAACCTGCGCGGTGCCAACCTGCGCGGTGCCGACCTGCGC
GGTGCCGACCTGTGCGATGCCAACCTGCGCGGTGCCGACCTGTGCGATGCCAACCTGCGCGGTGCCGACCTGCGCGGTGCCGACCTGTGCGATGCCAACCTGCGCGGTGCCGACCTGTAC
GGTGCCAATCTGTGCGATGCCAACCTGCGCGGTGCCGACCTGTGCGATGCCAACCTGCGCGGTGCCAACCTGTACGGTGCCAATCTGCCTGATCTCACTTTCGTAATCCTGGGTGAGAAATA
CTTCATAAGTATAACGAACGGTGAATATGTACGAGCAGGATGCCAGAACCACACAGTTGAGGAATGGAGAAAATATAGTAAGCAGAAAATTGCTGAGATGGATGGTCGTAAAGCTCTTAAAT
TTTATCCACGTCTTCTGGACATTATCGACTTCTATATTGGTAAAGGTGAACGCCCGGATTGGTTAACAAGTAAAGAATATGCAGATGAAGTCACTGAGTAA
>PNNMHK_01425 Antitermination protein
ATGACACGCAGAACAGTTTTCAATGGTTCAGCAGCAGGTCGTCGCCGCGAACGTCGCGCTGCGCTTCAGAATGAGACTACAGCCAGTTCAGAAGTATTACACCGCCCCACCCTTAGCCGTG
CGCAGATTCAGGCCAAAGGAAAACACGAAACGCCAAAACGCATTGAAGACGCAAAATCACTTCAGTTCATGGCGAAAGATGCATTCTGGCAACTGGAAGAATACAGACGCAATCTGGAG
CGGGCAGCCATTGTGTACGCAAATGAGTTTGGACATAAGCCACCAGAAACCGGTGTATGTTTGCCAGACGTAGCACTTTACGCGGCTGGCCATCGTAATTGTGGGCAAATTACCGCTAGATA
A
>PNNMHK_01430 Fumarase D
GTGAAAAACGAGGAACTGGCGCAGTTGCGCTATCAGGAAATGTGCAGGATTGTCGGTGATGTCGTTTTTGCTATGGTTGCTGAGGGTCATGAGACTAAGCGAGTGGCTATAGCTGACGTG
ATACGAACTGAGTTAGCGAAGGGGCTGGATAAGTGGGATGGTGACCAGTTGCAGTGCATGAAACTTGCTGTGAAGCTACTGGAAGAGTAG
>PNNMHK_01435 hypothetical protein
ATGTTCACACACATGCTCAAGATTTCCATACCTGATCCGTTCATAGGCATTAACATGAACTTCTCGATCGGACTGGTCATTCAAATTGTATGTAGCCATCTGTTTTTTCTCATGTTAGCGGCAGCC
CTACAACCGCTTGAAAAAATGTACCAGACGCGCTAA
>PNNMHK_01440 hypothetical protein
ATGCTGTTTAACCAGAGCCTCCAAAAAGGAGCCCTTGTTAAGGTACACAACATTTGCAATGTAACCTTAGCGCGTCTGGTACATTTTTTCAAGCGGTTGTAG
>PNNMHK_01445 hypothetical protein
ATGGCAGAATCAAACGTTAGCGTACAGGCATTCAAGGGCTTCCTTGAAGAGCTTATGTCGCTGAACATAATGAAGGAGGCCACCGCTCGAAATTTAAAAAACTCATCTGCTCGCCTCTTAAC
GGTAGTCAAAGAAGAGGAAATGGATGATGTTACAAAGCTTGATGTGAATGAGCTTGCCGAGCGCTACATTAACGCAACTGAGCCAAAGCCTAGTGACAGCAGCATTACTGCGTATAAAAGC
CGTATGGAAAGTGCGATTAAAAAATTTGTGGCTTACCAAGCTGGAGAGACAATCCCTTACATTCCTGTAGAGCGCCAAGAGGAGGAAGAAGAATTGGTTGAGTTAAAACAGCCAGTAGAG
GTAAAGAGTGCTACGCCATCCTCTTACTCACTACCCGTAGTCATTCGCCCAGAATTAGGAGTCACAGTAACCATTAGCGGAATACCCACTGACCTTACAAGCGAAGAAGCCGAACGCATCTCT
TCAATACTGAAGGTTTACGTCCGGCCTCATTAA
>PNNMHK_01450 Transcriptional regulator

ATGCATATGAAAACTTTTGCTGACAGACTTAACGCCGCCATGTCTGCATCTGGGCTATCGCAAGCTCAGTTGGCAGAAAAGGTAGGAATATCGCAACCAGCTATCCAAAAAATGTCCTCAGG
CAAAACCAATGGGACAAGAAAGATGGTTGAGTTAGCTAATGCCTTAAAAGTACGCCCTGAATGGTTAAGCTCAGGGGTTGGTGATATGAGATATCCAGAAGGACCAGAACCATCAAATATC
CGAGAATCTTCCCTTAAGGCTACTATTTGGGAGGATATGAACAGGGACTCTGAGGAGTTTGTTGAAATCCCTTTATTAAATGTTTCTCTTTCTGCCGGGAATGGGAGTTGCGAGTTGGAAGA
GTCTTCAGATTTTGCTTTGGTATTCCGTCGATATTACCTCAAAAAAATGGGCGTTCCAGAGAGCGCTGCAAAATTAGTTCGGGTAAGCGGTCAAAGCATGGAGCCAACTTTAAACGATGGAG
ATGTTGTTGGGGTAAACACGCAGGACACAACCATACGCGATGGCAAGACCTACGCAATTTGTCAATCTGATCTGCTGCGAGTAAAGACACTGATAGCTACTCCAACATCAGTAATAATCAGAT
CGATAAATCGCGAGGAATACCCTGATGAAGTGCTGGAGAGAGAAGACTTCCACAAAAACGTAAGAGTCATTGGAAGAGTGTTCTGGTCATCTCATAGCTGGTAG
>PNNMHK_01455 Transcriptional regulator
ATGAATTTAGTTATTCAGCGAGCCTTAAATATCGTCGGAAGCCAAAAGCGGTTAGCCGATGAATGTGGTGTAACGCAGCCAGCAGTTCATAAATGGCTTAAAGGTGGGATGGTATCTCCTGA
GAAGGTATCCGCCATTGTTAACGCCACTGGTGGGCAGATCAAGGCATATGAGATACGCCCTGACCTACCACACTTGTTTCCTCATCCGAATCAGGCCGCGTAA
>PNNMHK_01460 Bacteriophage CII protein
ATGGAACTCACAAATCACAGCAAAAAGATACGCGAAGTGGAAACAGAGCTTCGCGCCCGACTCGTATCAATGGGGCAGACAAATTTCGCAAAGATGGCGGGATGGTCTGATTCAAAAGTA
AGCCGCCTGAACATTCAGGATATGGCGGTGACCTTCGTTCTTCTGGAGAAGGTATGGGAGACGAGCTTAATCAGGGAAGTGGCAAGACAGGCAGTGGAAGCTGTTATGCCGGGAAATAA
AAAACGCCCGGCGTGCAAGACCGGGCGTTCTGAGCAAATCGATATGCAATTCTGA
>PNNMHK_01465 hypothetical protein
ATGCCAAAACGTCGTAAGAAATACCAGGAAAAAGAAGAGATTCGACACCCTGATTCACCTGAGGGATTAGTGGTAGCCGCAGCAAATAACAGGGCGTTCGCAGAGCGCCTTGTTGGTGTT
TACAGACTAGCCAAAGCAGGAGTGAAACATGGGCGTCGTTAA
>PNNMHK_01470 18 protein
ATGGGCGTCGTTAAGTTAGCAGACTACAGGCCGTCAGAGCCGGTCGTGGAGCGTAATGTGGCAGATCTCGATGATGGTTACGCCAGACTATCAAATATGCTGCTTGAGGCTTATTCAGGCGC
AGATCTGACCAAGCGACATTTTAAAGTGCTGCTTGCCATTCTGCGTAAAACCTATGGGTGGAATAAACCAATGGACAGAATCACCGATTCTCAACTTAGCGAGATTACAAAGTTACCCGTCAA
ACGGTGCAATGAGGCCAAGTTAGAACTCGTCAGAATGAATATTATCAAGCAGCAAGGCGGCATGTTTGGACCAAATAAAAACATCTCAGAATGGCGCATCCCTCAAAATGAGGGAGTATCC
CTCAAAACAGGGGATAAAACATCCCTCAATTTGAGGGAGTGTTATCCCTCAAAACAGGGGGACACAAAAGACACTATTCAAAAGAAAGAAATACAAGATAAAAACACTATGTCCGAAAGC
GTTCGGACGGAGTGTGAAAAATCATCTGACCGTCACGAAGAAACCGATAAGGCATTCGAGGAAATATTCTGGTGTGCCGGTATGCGAAAAGCCGGGAAGAAAAACGCAGCTTCGGCATTC
AGAACACAGTTCAGGGAATGGCGTAAAACTACCAGGGGTACGGCAAGCGAGTTTGCCACGATGCTGGCAGAAGACATCGCATGCAGGAATGGTAAGCAGTTCGGATTCGACAGGTTGTT
ACCATCAAGCTACCTGAACGGTCAGCGCTGGAACGACGAAAAGCCAGAAACAATTCAACCACAATCCAAACCATCATCCGCAATCACCGTATCGAAAACTGGCTACGTGTTTTTCGACAGG
TGA
>PNNMHK_01475 Replication protein P
ATGACTCCAAGTGAACTGAGCGACCTGCTATGGGCGCAGGTTGACAGGGTGGCTCCGCACCTGTTGCCAAACGGCAAGAAAGAGGGGCATGAGTGGGTTGCCGGCAACGTCAACGGTG
ACAAGGGGAACAGTCTGAAGGTTAACCTTAGCGGTAAGAAAAAAATGGGCTGATTTCGCTGAGGGCGACGGCGGTGACATGCTTGATTTGTGGATGGCCTGTCGGGGAATAAATCTGCATC
AGGCTATGCAGGAGGCAAAGGCATTTCTCGGTATCAAGGATGATGATCACCATTTCGACGCCAGACGTGAGAAGAAATTCTCCAGGCCTGACCGCAAGAAAATCGCCCGTTACGTTACCAG
AACAGAATCCCATCTTGAGTACCTGCAATCGCGTGGCATATCTCCAGAAGTCGTAAAGCGGTACGAGGTTGTCAGCGGCAAGGTGTGGAATGGAGAGCGAGAACTGGATGCTTTGGTGCT
TCCGTACAAACGCGATGGTGAGTTGTTGCAGGTCAAGAGAATCAGCACCGAACGTCCGGACGGGAAGAAAGTCATCATGGCAGAAGGTGACTGTGAACCCTGTCTGTTCGGATGGCAGG
CTCTCGATGCTGGCGTGAGGGCGGTTGTGCTTTGCGAAGGCGAAATTGATTGCATGAGCTATGCGCAATACGGAATTCCGGCGCTATCTGTCCCTTTCGGTGGCGGGAAAGGCGCTAAGCA
ACAGTGGATTGAGTTTGAATACCATAACCTCGACAGGTTTGAAGAAATATTCATTTCGATGGACGGTGATGATGTTGGTCGTGAAGCTGCAAGGGAAATCGCAAGCCGACTCGGTGAACAT
CGCTGCCGTCTGGTTACACTGCCGCACAAAGATATCAACGAATGCCTGATGAACGGCGTCGCTGAGGATGAAATCTGGCAGTACATCGGTACAGCGTCATATTTCGACCCCGAAGAGCTTTA
CAGCGCCCGTGAGTTTTATCAGGACACCATCAATGCTTTCTACGGCAAGCAGCAGTATCTGTTTAACCCACCGTGGGAAACGCTGGCTTACAACTTCCAGTTCCGTGAGGCGGAGTTAACGC
TTGTCAATGGCGTGAACGGTCACGGAAAAACGGAGGTTGTCGGGCATATGGCACTTGAGGCCATGAGACAGGGGATAAAAACATGCGTCGCATCGCTTGAACTGAAGCCCGGGATTCTGC
TTAAACGCCTGACCCGGCAGTCTACATGTTGCAAAATGCCGCCAGTTCTGGAAATCGAATCAGCATTTAAGTTTTACGATGACCGGCTCTGGTTATTTGGCCTGACAGGTACAGCCAAGGCT
GAACGCCTGATTGAAATTTTCACATACGCCAGACGGCGATACGGCATCCAGTTATTCATCATCGACAGCCTCATGAAGTGCGGTATTGGCGATGACGATTACAACGGGCAAAAGGCGTTTGT
TGACGCGCTGTGCGACTTCAAGAATAAAACCAACTCTCACATTATTCTCGTCACTCACTCCAGAAAGGGAGACAGCGAGGAGAAACCTACCGGAAAGATGGACGTAAAAGGCTCAGGAGC
GATTACAGACCTGACAGATAACCTGTTTATCATCTGGCGCAATAAAGCTCGCGAGAGAGCGTTACAGCGCGTTCAGGCTGGCGAGCAAATTAACGAGAAAGACCAGCAACTTCTTGCTTCG
CCCGCATCTGTTTTAATGCTTGAGAAGCAGCGGAACGGGGAGGGGTGGGAAGGTGGCGTGCCGTTATTTCTTGACGAACAGTCTCACCAGTTCCTGCAAATGGAAGGTGCATCACCATAC
AACTACATAGCTAACATGCCTAAGTCGGAGTATGACGAAGTGTGGAGGCAGGAGAATGTTACGGAGTACTGA
>PNNMHK_01480 Transposase
ATGAACAACCAAATAATACCTGAAATGCTTTTGAATCCCCGCTTCATTGCTGTTTTGAACAGATGTATCGACGAAGAAGAGCTCATTATGCAATTTGAAAGGTTGTCAGGTGTCACTCGACCA
CCAAAGGGGCAACATCCAATAGAGCTGATGGTTGATAAAGCGACAGGATTTTCTGATGAGCAGTGGAAACGGTTTTTTGAAGCATTTATCCCGTTCGTCTATGAGTTTATATGGCTCACATGG
AGAGACCGTGACAATGAGGAGTGCTGGCAATGA
>PNNMHK_01485 Prophage protein
GTGGCTGACTGGCAAATTCCAATCGTCATTCTTGCCGGAGCTTCGCTGGTTGCTGGCTTTATCCTGCTGAAGAAGCATAAAGACCGTGATCAAAAAGTCGAAGTTCTCTATGGGTATCCAGC
GAACAGCACAACATGGCTGACCATTTACCACTACCGAAAATCAGGCCGCTGGGTATTCGAATGGGATGATCTGTTCGCTGAAAAGCGACCAAAGTCATGGGGAGACATCAGCGAATGCATG
ATGTTTGAAGAAAGAAAATCCGGCGCAACCCGAGAAGAGTTTAACGAAGCGTGGGCGCGATTAAGTGAGAGAGGGTATTTGTGA
>PNNMHK_01490 hypothetical protein
GTGAGCAAGTACGAAAAATTAGATCAAAACATTCTTTCAATGCTGAGTGAAAGACCAACACCTGTTTTTGATATCTGGCTTAAATGGCGGAGCAATGGAATGTATATCGAAACCATCGATCGC
CGTATGCAATACCTGAGAAAGAAAGGGCTTGTTGCAAATGTGCGTGGGAAGGGGTGGGTGAAAATTAACCTGTCATAA
>PNNMHK_01495 Phage-ABA-S domain-containing protein
ATGGACGAATCAAGAAAGCAGTTTGAAGAGTGGTTTAAAAACAAATATCACGTTTCAAGTGACGTGATGAAGATTATGCATATCAAGGTCGAGATTGCATGGGAGGCGTGGCAGGCATCGC
GAGCAGCTATTGAACTGGATATCGACTGGCCCGAATCGAATGACGACTTTTGGAGAGATGGTGAAGAAGGTGCTTATGCGATGGGTTATGAGGATGGGCGTGACAAAACGGTAATTGCAG
TAATGAAAGCTATCAGAGCCGCTGGAATTAAAGAGAAGAATTTCGATGAAGCAAATATACATGCTTCGCAACGAAGCAATCAGAAATAA
>PNNMHK_01500 Recombination protein NinB
ATGAAGCAAATATACATGCTTCGCAACGAAGCAATCAGAAATAACGCCATAGACGCAATACTATCACTACCCATCGACGACAAGTCACCCCACGAAGTCCACGTCAAAGAACCTAAGCGAAC
CAAAGCGCAGAACGACCGTATGTGGCCGATGCTTCAGGACGTTTCCCGTCAGGTGCTATGGCATGGTCAACGACTGTCTCCAGAAGACTGGAAAGACATCTTCACCGCGCTGTGGCTCAAG
ACTAAAAAGCTGGAGCAAAGAAGCGTACCCGGTATTGACGGCGGTATTGTTCTTCTTGGGGTACGTACCAGCAAAATGAGGAAGGCCAGCATGACAGAGCTTATCGAAATCATGTTCTGGT
TCGGATCAGAACGTAACGTGCGATGGAGTGATGATTCCCGGCGTGAGTATGAATGGTCACAACGAACAGGCAGGGCAGCATGA
>PNNMHK_01505 hypothetical protein
ATGATTGACCCCAATCGAAGTTATGAGCAAGAGAGCATAGCAAGGGCAATGTGCGCAGGATGTAACAAGCAACTGGCACCTGATGAAATTTACGCCTGTGCAGAATGTGTTAACGAATGGC
TGGTATATCGCGATCCGAATGGAGATATGTCGAATGAGGAAGGCAAGGCGGCGTTGTAA
>PNNMHK_01510 Protein ninG
ATGAGGAAGGCAAGGCGGCGTTGTAAGAACGAAGAGTGCAGGGAATGGTTCTTCCCGCAATTTCAGAACCAACAGTGGTGTTGTGTTGATTGTGGTACGAAGTTAGCACTCGAACGACG
AAGCAAAGAGCGCGAAAAAGCAGAAAAAGCAGCAGAGAAGAAACGACGACGAGAGGAGCAAAAACAGAAAGATAAACTGAAGATTCGAAAACTCGCCTTAAAGCCCCGCAGTTACTG
GATTAAACAAGCCCAACAAGCCGTAAACGCCTTCATCAGAGAAAGAGACCGCGACTTACCATGTATCTCGTGCGGAACGCTCACGTCTGCTCAGTGGGATGCCGGGCATTACCGGACAACT
GCTGCTGCACCTCAACTCCGATTTGATGAACGCAATATTCACAAGCAATGCGTGGTGTGCAACCAGCATAAAAGCGGAAATCTCGTTCCGTATCGCGTCGAGCTTATTAACCGTATCGGTCAG

GCCGCGGTAGACGAAATCGAATCAAACCATAACCGCCATCGCTGGACTATCGAAGAATGCAAAGCGATTAAGGCGGAGTATCAGCAGAAGCTTAAATACCTGCGTGACAGCAGAAGTGAG
GCAGCATGA
>PNNMHK_01515 Putative protein ninY
ATGAGCAAAATCCAATACCCAATGACCACTGCGGCAATTTTCGATGATGTTGTCTATCCGCTGCATTTCGACAATGCCGGCAAGGTCAGGCAAGAAATGGAAGGCGCTGTTAACTGGTTCTG
CAGGTGGCGCAACGAAGAGAAATCCGCTGTGAAAGCGAGATTGTTGGTCAGTTGCTGGGGTCAATATCTGAGTCATGAGCAGGTTATCCGGGAGGCCGCATGA
>PNNMHK_01520 hypothetical protein
ATGACACACACTATCAAAACCATTCCAGACATGCTCATAGAGACATATGGAAACCAGACAGAAGTAGCACGGCGCTTATCGTGCCACCGCAACACAGTCAGGCGTTATCTGTACGACAAAGA
AGCCAGGCATCACGCCATCGTTAACGGCGTTTTAATGATTCATCAGGGCGGGAGAGGTATCTATGACCGTAACCAGCATTAA
>PNNMHK_01525 hypothetical protein
ATGACCGTAACCAGCATTAACCAGGCGAAACAGCAGCGTGAACGTGACGAAGCTGAATTGCGCAGCGTCAGAGAGATGACGGAGCAACACCAGAAGGCGATGGATTATCTGCATGAGCG
AGAGCGTGAACTGGTGAACCGGATTGGATTGAACAAGCCATCGGGAGGCGATGCTGCATGA
>PNNMHK_01530 Antitermination protein Q
ATGAATTTGGAAAATGTAGTGAAGTTTCACTTCGCAAAATCTTCTCAGATAAACGATATCCCTCGCGCAACAGCTTCAGAAACGTTAACTGGCACTGATGTTATGGCAGCTATGGGTATGACT
CAAAGTCGCGCATCGTTGGGTTACAGCGCGTTTCTTGGGAAGATGGAAATCAGCAGCAATGACCGTGAGAAAGCTATTGAACTGCTGACTGCCTATGCTCTTAAGAACTGCGATAATGTTCC
TGCCTTACGCAAGCTAGAAAATGATATTAAGCCAAAGGTAATGCAAGTGCTCGCAACATTCGCATTTGCTGACTATTCACGAAGCGCTGCCAGTACCAGAACCTGTGATTGCTGCGGCGGGA
AGAAGTTTATCGATGCCGAGGTAATGACGATGAAAAGCATCGGGCAGCCGTACCTTTCAGAGCGCAAGGAGACGGTGAAAGTTTTGTGCAATAAGTGCAAAGGGAAGGGGGGTTCTGACC
AACGCATGCCAGTGCAATGGCAAAGGTGTTGTTATCGATAAAGAGAAAACTATTCTACAAGGAGGCGTCCCTGCATACAAAACATGCAGACGTTGTAATGGGCGAGGATATGCTCGGTTACT
GCCTGATAGCGTTCGAAAGTACATCTGCGCAACAGTGATTGATATTCCTGAAACCACATGGCGCAGGTCATATAAGGATTTCTTCGAAAGTCTGGTAGGTGAGTGTATTAAGCAGGAGGAAT
ATGCAAATCAGATGTTGAGCAAAGTCACGCAGTGA
>PNNMHK_01535 Antiholin
ATGAAAAAGATGCCAGAAAAACATGATCTGTTAACCGCCATGATGGCGGCAAAGGAACAGGGCATCGGGGCAATCCTTGCGTTTGCAATGGCGTACCTTCGCGGTCGGTATAATGGCGGTG
CGTTTAAGAAAACACTAATAGACGCAACGATGTGCGCCATTATCGCCTGGTTCATTCGTGACCTTTTAGTCTTCGCCGGACTGAGTAGCAATCTTGCTTACATAGCGAGTGTGTTTATCGGCTA
CATCGGCACAGACTCGATTGGTTCGCTAATCAAACGCTTCGCTGCTAAAAAAGCCGGAGTCGATGATGCAAATCAGCAGTAA
>PNNMHK_01540 Endolysin
ATGCAAATCAGCAGTAACGGAATCACCAGATTAAAACGTGAAGAAGGCGAGAGACTAAAAGCCTATCCAGATAGCAGGGGGATACCAACTATTGGGGTTGGGCACACCGGAAAAGTGGA
TGGTAATCCTGTCGTATCAGGGATGATAATCACAGCCGAAAAATCGTCTGAACTGCTTAAAGAGGATTTGCAGTGGGTTGAAGATGCGATAAGTAGTCTTGTTCGCGTCCCGCTGAATCAGA
ACCAATATGATGCACTATGTAGCCTGATATTCAATATAGGAAAATCAGCATTTGCCGGCTCTACCGTTCTGCGCCAGTTGAATTTAAAGAATTACCAGGCAGCAGCAGATGCTTTCCTGTTATG
GAGAAAAGCTGGTAAAGACCCTGATATTCTCCTTCCTCGGAGGCGGCGAGAAAGAGCGCTGTTCTTATCATGA
>PNNMHK_01545 Outer membrane lipoprotein Rz1
ATGCTAACGCGACTATCGAAAGTCTCCGTGCTGATGTTTCTGCTGGTCGTAAGCGCCTGCAAGTCTCCGCCACCTGTCCAAAGTCAACGACCGGAACCAGCGGCATGGGCGATGGAGAAAG
CCCAAGACTTACAGCAGATGCTGAACTCAATTATTACCGTCTCCGAAGTGGAATCGACAGGATAA
>PNNMHK_01550 Rha family transcriptional regulator
ATGCAATTAGTTGAAATCAAGAAGCTCGACTTGGTCACTAACTCCGCTGTAATCGCTACTGGCGTCAAAAAGGATCACAAGCCTGTGATTCAGCTCATCAGGAAGTACAAAAGCGACCTCGA
AGAGTTCGGAAGGGTGGAATTTGAAATGCGACCCTTTCAAACGGATGGGGGCATGCAGAAGCAGGAAATAGCACTGTTAAACGAACAGCAAACCACGCTGTTGATCACATACATGCGAAA
CAATGAAGTTGTGCGTGAATTCAAAAAGCGCCTGGTAGCTGAATTCTTCACTATGCGTAGCGCGCTGGCGAAAAAGAAAATGGATCGCAACTCTGCACGCCTGGAGTACAAACCCATGACC
GACGCCATCAAACATGAGCGAGAGGCTCAGGGTAAGCAGATCGCCCCGCATCACTTCAGCAACGAAGCTGACCTGATTAACCGACTGGCGCTGGGAATGACGGCGGCCAAGTTCCGCGT
GCATCACGAAATCGGGAAGAAAGAGCCGATCCGCGATTACCTGACGCCGGAACAAATTCACTGCATCACCGAGCTACAGCGTGCCAACACGGTATTCATCAGCATGGGGTGGGACTTCGA
ACAACGCAAAGAAGTGCTGCGCGGCATGTTCGAACGTAATCATCGTCAGCCCCTTATCGAAGAACAGCACCGTCTGGCGGCCTAA
>PNNMHK_01555 DUF2560 domain-containing protein
ATGGCAGAGATTACAGCATTGACAGAATTACAGCAGATGAACCTCGATATCCTCCGTTTAGTTCAAAGCGATACCGCAGCAGCAGAGAAAGCGATCGCATTCGTTGCTGGAAGTAAGCTGA
ACTTCGAACTGTTCAAAGACCAACTGGTTTTGGCGCAGGGTGAAGGAACGGCATTAGCTCGCGCAGAAAAGGCTATTCGTGAGGCAAAAGAAGCGTTAGACCTGTTCACCGCCGGAGTA
TAA
>PNNMHK_01560 Decoration protein
ATGGCAAATCCAAATTTCACGCCATCATGGCCTCTCTACAAAGATGCTGACGGTGTATATGTGTCTGCTCTTCCGATTAAAGCTATCAAATACGCTAATGACGGAAGTGCAAACGCAGAATTCG
ACGGCCCGTATGCTGACCAGTACATGTCAGCGCAAACAGTAGCCGTATTCAAGCCAGAGGTTGGCGGATATCTGTTCCGGAGCCAGTACGGCGAGCTGCTCTATATGAGCAAGACAGCATT
TGAAGCTAAGTACACTTCTGCAAGCGGTTCAGTAACGAATGCAGAGACGGCGGATAAGTTATCTACTGCTCGCACTATCACACTAACCGGCGCTGTCACAGGTTCAACGTCCTTTGATGGTT
CGGCTAACGTGACTATCGCAACAACATCAGGAAGTTAA
>PNNMHK_01565 Terminase, small subunit
ATGGCAGCACCAAAGGGCAACCGATTCTGGGAGGCCCGCAGTAGTCATGGGCGTAACCCGAAATTCGAGTCGCCTGAGGCGCTGTGGGCTGCTTGTTGTGAATACTTCGAGTGGGTGGA
GGCTAACCCACTATGGGAGATGAAGGCTTTCTCATATCAAGGAGAAGTTACACAAGAGCCTATCGCCAAGATGAGGGCGATGACCATCACTGGGCTAACGCTATTCCTCGATGTGACGCTTG
AGACATGGCGACAATACAGGGTGAGAGAAGACTTATCTGAGGTCGTTACGCGAGCAGAGCAAATCATCTACGACCAAAAATTCTCCGGCGCAGCCGCTGATCTTCTCAACGCTAACATCATC
GCCCGCGATTTGGGCCTCAAAGAGCAGTCGCAAGTTGAAGACGTGACACCTGATAAGGGAGATCGCGATAAGCGCCGCTCTCGTATCAAGGAGCTATTCAACCGTGGAACTGGACGCGAT
TCTTGA
>PNNMHK_01570 Terminase, large subunit
GTGGAACTGGACGCGATTCTTGATAACCTGAGCGACGAAGAGCAAATCGAATTGCTCGAGCTACTCGAAGAAGAAGAGAACTACCGAAATACACACTTGCTATATGAGTTTACGCCATACA
GCAAACAGCGTGAGTTCATCGACGCAGGTCATGACTATCCAGAGCGATGTTTTATGGCTGGTAACCAGCTTGGTAAGTCATTTACTGGCGCTGCTGAAGTCGCGTTTCACCTTACCGGGCGA
TACCCGGGAACGAAAGGTTATCCGGCTGATGGTAAATATGGCGGAGAGTGGAAAGGTAAGCGTTTCTATGAGCCAGTTGTCTTCTGGATTGGCGGTGAAACAAACGAGACTGTAACCAAA
ACGACTCAACGCATCCTGTGCGGGCGTGTCGAAGAGAATGATGAACCTGGCTATGGGTCAATCCCGAAAGAGGACATCATTAGCTGGAAGAAGTCTCCGTTCTTCCCTAATCTTGTTGATCA
CCTTCTTGTTAAGCACCACACGCCAGAAGGCGTCGAAGATGGCATCTCAATATGCTACTTTAAGCCTTACTCACAGGGCCGCGCCCGCTGGCAGGGCGACACAATTCACGGTGTCTGGTTTG
ACGAAGAGCCGCCATATAGCATCTATGGCGAAGGTCTTACCCGTACAAACAAATACGGGCAATTCTCAATTCTGACGTTTACCCCGCTGATGGGGATGTCTGACGTTGTTACCAAGTTCCTGA
AGAATCCCAGTAAGTCGCAGAAAGTGGTCAACATGACAATCTATGACGCTGAGCACTACACCGACGAGCAGAAAGAGCAAATCATCGCATCCTATCCTGAGCATGAGAGAGAGGCGCGTG
CTCGCGGTATTCCTACGATGGGTAGCGGTCGAATATTCCAGATACCGGAAGAGACGATTAAGTGCCAGCCGTTTGAGTGTCCCGATCACTTCTATGTTATCGACGCTCAGGACTTCGGCTGGA
ACCACCCGCAAGCTCACATTCAGCTTTGGTGGGACAAAGACGCAGATGTTTTCTATCTGGCGCGTGTATGGAAGAAATCAGAGAACACTGCCGTTCAGGCATGGGGTGCTGTTAAGTCGTG
GGCTAACAAAATACCTGTCGCGTGGCCTCATGACGGTCACCAACACGAAAAGGGCGGTGGTGAGCAACTTAAAACCCAATATGCGGACGCCGGGTTCTCTATGCTTCCCGAACACGCAAC
GTTCCCGGATGGCGGTAACTCAGTAGAGTCAGGCATTAGTGAACTTCGTGACCTGATGCTTGAAGGAAGATTCAAAGTATTCAACACATGCGAACCATTTTTTGAAGAGTTCCGCCTATATC
ATCGCGATGAGAACGGCAAGATTGTCAAGACCAACGATGATGTGCTCGATGCTACTCGCTACGGCTACATGATGCGCCGCTTCGCCAGGATGATGCGCGATATCAGAAAGCCGAAAGAAAA
GAAAATCCCCGCACCGATTAGACCAGTACGCAGAGGACGATAA
>PNNMHK_01575 Portal protein
ATGGCCGACAATAAAAACAGGCTGGAGAGCATCCTGTCGCGCTTTGATGCGGACTGGACAGCCAGCGATGAAGCCAGAAGGGAGGCCAAGAATGATCTCTTCTTCTCCCGCGTATCTCAG
TGGGATGACTGGCTATCACAATACACAACCCTGCAGTATCGCGGGCAGTTCGATGTTGTACGTCCAGTGGTGCGCAAGCTCGTTTCTGAGATGCGTCAGAACCCTATTGATGTTCTGTATCGT

CCAAAGGATGGAGCAAGTCCTGACGCTGCTGATGTGTTGATGGGCATGTATCGCACCGACATGCGGCACAATACGGCGAAAATTGCTGTCAACATAGCCGTTCGTGAGCAGATTGAAGCAG
GCGTGGGTGCGTGGCGTCTGGTCACTGACTACGAAGACCAAAGTCCAACTAGCAACAATCAGGTTATCCGTCGAGAGCCTATCCATAGTGCCTGCTCCCATGTTATCTGGGACAGCAACAGC
AAACTGATGGACAAGTCTGACGCCCGTCACTGCACAGTTATCCACTCAATGAGCCAGAATGGTTGGGAGGATTTCTCAGAAAAATACGACCTCGATGCTGATGATATTCCATCATTCCAGAAC
CCCAACGATTGGGTGTTTCCATGGCTGACGCAGGACACAATTCAGATCGCTGAGTTTTACGAAGTGGTCGAGAAGAAAGAGACGGCGTATATCTACCAAGACCCGGTTACGGGTGAGCCG
GTAAGCTACTTTAAGCGCGATATTAAAGACGTCATCGACGACCTGGCTGATAGTGGATTTATCAAAATTGCAGAGCGCCAGATTAAGCGTCGCCGGGTATACAAATCGATTATCACCTGCACC
GCAGTACTGAAAGACAAGCAGCTCATTGCTGGCGAGCATATCCCCATTGTTCCGGTATTCGGAGAGTGGGGCTTCGTTGAAGATAAAGAAGTGTATGAGGGTGTCGTCCGCCTGACAAAAG
ACGGTCAGCGTCTGCGCAACATGATTATGTCCTTCAACGCCGATATCGTGGCCCGTACTCCGAAGAAGAAGCCGTTCTTCTGGCCTGAGCAGATTGCAGGCTTTGAGCATATGTACGACGGT
AACGACGATTACCCGTACTACCTGCTCAATCGCACTGATGAAAACAGCGGAGAAATGCCAACTCAGCCGCTGGCATATTACGAAAATCCGGAAGTTCCACAGGCCAATGCCTATATGCTGGA
AGCTGCAACCAGTGCAGTAAAAGAGGTTGCAACGCTAGGTGTTGATGCAGAGGCGGTAAACGGTGGACAGGTAGCCTACGACACTGTTAACCAGCTAAACATGCGTGCTGACCTTGAGAC
ATACGTGTTTCAGGATAATCTGGCTACCGCTATGCGCCGTGACGGTGAGATTTACCAGTCGATAGTTAACGACATCTATGATGTCCCTCGCAACGTGACAATCACCCTTGAGGATGGCAGCGA
AAAAGAGGTTCAGCTAATGGCTGAGGTTGTTGACCTTGCTACTGGTGAACGACAGGTACTGAACGATATCAGGGGGCGCTATGAATGCTACACGGATGTTGGGCCATCATTCCAGTCCATG
AAGCAGCAAAACCGCGCAGAAATTCTTGAGTTGCTCGGCAAGACGCCACAGGGAACGCCAGAATATCAACTGCTGTTGCTTCAGTACTTTACCCTGCTTGATGGTAAAGGTGTCGAGATGA
TGCGTGACTATGCCAATAAGCAGCTTATTCAGATGGGCGTTAAGAAACCAGAAACGCCTGAAGAGCAGCAATGGTTAGTAGAGGCGCAACAAGCCAAACAAGGTCAACAAGACCCGGCA
ATGGTTCAGGCTCAGGGCGTACTCCTGCAGGGGCAGGCTGAACTGGCTAAAGCTCAGAACCAGACACTGTCCCTGCAAATCGATGCAGCTAAAGTCGAAGCGCAGAACCAGCTTAACGCT
GCCAGAATTGCAGAAATCTTCAACAACATGGACCTCAATAAACAATCTGAGTTTAGGGAGTTCCTTAAAACTGTTGCTTCATTCCAGCAGGACCGCAGCGAAGACGCTCGCGCAAATGCTG
AGTTACTCCTTAAAGGCGATGAACAGACGCACAAGCAGCGAATGGACATTGCCAACATCCTGCAATCGCAGAGACAAAATCAACCTTCCGGCAGTGTAGCCGAGACACCTCAATAA
>PNNMHK_01580 Scaffolding protein
ATGGAACCAACCACCGAAATTCAGGCAACTGAAGACTTAACCCTGTCCGGCGATTATGCAGCGGCATCTGCTGATAGCTTAGTTGTCGATAATGCCAACGACAATGCAGGTCAGGAAGGGG
GCTTTGAGATTGTCCTGAAGGACGATGAGACAGCACCAAAACAAGACCCGGCAAAGAACGCAGAATTCGCCCGCCGCCGCATCGAGCGCAAACGACAGCGCGAGCTTGAGCAGCAGAT
GGAAGCAGTTAAACGCGGAGAATTGCCGGAGAGTTTACGGGTAAACCCTGACCTTCCACCTCAGCCGGATATTAATGCCTATCTGTCAGAAGAAGGCCTGGCCAAATATGACTATGACAAC
AGCCGTGCGCTTGCCGCTTTCAATGCTGCTAATACCGAATGGCTAATGAAAGCGCAGGACGCCCGCAGCAATGCCGTAGCAGAACAGGGCCGCAAGACTCAGGAGTTTACCCAGCAATCA
GCGCAATACGTCGAAGCTGCCCGCAAACACTATGACGCGGCGGAAAAGCTCAATATCCCTGACTATCAGGAGAAAGAAGACGCATTTATGCAACTGGTTCCGCCTGCGGTTGGGGCCGAC
ATTATGCGCCTGTTCCCGGAGAAGTCCGCCGCGCTCATGTATCACCTGGGTGCAAACCCGGAGAAAGCCCGCCAGTTACTGGCGATGGATGGGCAGTCCGCGCTGATTGAACTCACTCGAC
TATCCGAACGCTTAACTCTCAAGCCTCGCGGTAAACAAATCTCTTCCGCTCCCCCTGCTGACCAGCCGATTACCGGTGATGTCAGCGCAGCAAATAAAGATGCCATTCGTAAACAAATGGATG
CTGCTGCGAGCAAGGGAGATGTGGAAACCTACCGCAAGCTAAAGGCAAAACTTAAAGGAATCCGATAA
>PNNMHK_01585 Major capsid protein
ATGGCAGGTCTTAACGAAGGTCAAATTGTTACACTGGCGGTGGATGAGATTATTGAAACCATCTCCGCAATCACTCCAATGGCGCAGAAAGCCAAGAAATACACCCCGCCCGCAGCTTCTAT
GCAGCGCTCCAGCAATACCATCTGGATGCCTGTAGAGCAGGAGTCCCCCACTCAGGAGGGTTGGGATTTAACTGATAAAGCGACAGGGTTACTGGAGCTTAACGTCGCGGTAAACATGGG
AGAGCCGGATAACGACTTCTTCCAGTTACGCGCAGATGACTTGCGAGACGAGACTGCGTATCGTCACCGCATCCAGTCCGCAGCTCGCAAACTGGCTAACAACGTTGAGCTGAAAGTCGCA
AACATGGCCGCCGAGATGGGGTCATTGGTTATCACTTCGCCGGATGCAATCGGCACTAATACCGCAGACGCATGGAACTTTGTGGCCGATGCAGAAGAGCTGATGTTCTCCCGCGAACTTA
ACCGCGACATGGGGACATCGTACTTCTTCAACCCACAGGACTACAAAAAGGCGGGTTATGACCTGACTAAGCGCGATATCTTCGGGCGCATTCCTGAAGAAGCGTACCGCGATGGCACTAT
CCAGCGTCAGGTTGCTGGCTTCGATGATGTCCTGCGCTCTCCGAAACTTCCTGTGCTGACCAAATCCACCGCAACTGGCATCACTGTATCCGGTGCGCAGTCCTTCAAGCCTGTCGCATGGC
AACTGGATAACGATGGCAACAAAGTTAACGTTGATAACCGTTTTGCTACCGTCACCCTGTCTGCAACTACCGGCCTGAAACGCGGCGACAAAATTTCTTTTACTGGCGTGAAGTTCCTTGGT
CAGATGGCTAAGAACGTACTGGCGCAGGACGCGACTTTCTCCGTAGTTCGCGTTGTTGATGGTACTCACGTTGAAATCACGCCGAAGCCTGTAGCACTGGATGATGTTTCTCTTTCTCCTGA
GCAACGGGCATACGCCAACGTTAACACCTCGCTGGCTGATGCAATGGCGGTGAACATCCTGAACGTTAAGGATGCTCGCACCAACGTGTTCTGGGCTGATGACGCCATCCGTATTGTGTCTC
AGCCCATTCCGGCTAACCACGAACTGTTTGCAGGTATGAAAACTACCTCATTCAGCATCCCGGATGTCGGCCTTAACGGTATCTTCGCTACGCAGGGTGATATTTCCACCCTGTCCGGCCTGT
GCCGTATTGCGCTGTGGTACGGCGTAAACGCGACACGACCGGAAGCAATCGGTGTTGGCCTGCCTGGTCAGACTGCGTAA
>PNNMHK_01590 hypothetical protein
ATGGGCGTAATGCTATATAAGCAGGGTCGTGGAACGAAGGTATGGGGCAAGGAAGTTCAGGCTAAAGTTGTCGATGACGGCGACGTAGAAGATCACCTTGCCGATGGTTGGGTTAAGCAT
CCAAATGAGGTGCCGGAGACTAATGACGAGCCAATCGGCGATTCAGGCGTGGTCAAGAAAGACATGGGTGAAGTATCTGATGGATACCACACCTTTAACGAACTATATGCACATCGAGTGC
GCCTGTTTTCAACACTAATGAATGCCTTCCGCGAAAGCGCATGGTGGAGCTTCCAGCATCATGACGGCGAGCAATGGGATGGATGGGTGTTAGCTGGCATCGACACCCCAGAAGGCGCGG
TAACATACCACCTCCCAGAGAGTGAAATTGAACATCTGCCTAAAGGCACGGAAATTGAGTTTGGCAAGGAATGGGACGGCCACACGGCAGATGATGTGTTGAATCGTCTGCTAAGCCTGCG
ACCGAAAGAACCGGCAACCAAAGAACGCAAAAAGCCAGGACCAAAGCCTAAGGCGGAAAGCGATGCAGATAAAGACTAA
>PNNMHK_01595 hypothetical protein
ATGCAGATAAAGACTAAAGGCGATCTGGTCAGGGCGGCGCTGCGTAAGCTTGGTGTAGCATCAGATGCAACTCTCACTGATGTTGAGCCACAGTCTATGCAGGATGCCGTAGATGACCTCG
AAGCGATGATGGCCGAATGGTATCAGGACGGAAAGGGCATCATCACCGGCTATGCATTCTCAGGTGATGATAATCCTCCCGCTGAAGGTGACGACCACGGCCTTCGCTCCAGTGCAGTCAG
CGCAGTATTCCACAATCTGGCCTGCAGAATTGCGCCGGATTATGCGCTTGAGGCTACCGCCAAAATTATCGCAACCGCTAAATATGGGAAGGAGCTTCTCTATAAGCAGACCGCCATCGCCA
GAGCAAAAAGAGCTCCTTACCCGTCACGTATGCCAACTGGCAGTGGAAACAGTTTCGCCAATCTGAACGAATGGCATTATTTCCCCGGAGAGCAGAATGCCGATTCAACAACTCCCCATGAT
GAAGGGAATGGGTAA
>PNNMHK_01600 Phage head DNA stabilization protein
ATGCCGATTCAACAACTCCCCATGATGAAGGGAATGGGTAAAGACTTCAAGAATGCCGACTACATTGATTACCTACCAATCAATATGTTGGCCACACCGAAAGAAGCCCTCAACTCATCTGGT
TATTTACGCTCATTCCCAGGCATAGCGAAGCGCAACGATGTAAATGGTGTATCGCGTGGTGTTGAATACAATACCGCTCAGAACGCCGTATATCGCGTCTGTGGTGGGAAGCTCTACAAAGGC
GAAACCGTAGTCGGAGACGTTGCCGGGGCAGGCCGCGTATCTCTTGCTCACGGTCGTACTTCTCAGGCGGTAGGTGTGAACGGTCAGCTCATCGAGTACCGATACGATGGCGCCGTTAAG
ACGATGGCAAACTGGCCTGCAGACAGCGGATTCACGCAGTATGAGTTAGGTTCAGTCCGTGACATTACTCGCTTACGTGGGCGTTATGCGTGGTCAAAAGACGGCACTGATTCATGGTTTAT
CACTGACCTTGAAGACGAATCGCATCCTGACCGATACAGCGCACAATATCGCGCAGAATCGCAGCCGGACGGCATCATCGGTATCGGCACATGGCGAGACTTCATTGTCTGCTTTGGTTCAT
CGACGATTGAATATTTTTCCCTGACTGGTGCAACCACCGCTGGCGCTGCGTTGTATGTCGCACAGCCATCGCTGATGGTACAGAAGGGCATTGCCGGAACATACTGTAAAACGCCATTCGCT
GATTCATATGCATTCATCAGTCACCCGGCTACTGGCGCACCTTCCGTCTACATCATCGGGTCAGGGCAGGCTTCACCAATTGCGACGGCCAGTATTGAGAAAATTATCCGCTCATACACGGCT
GATGAACTGGCAACCGGGGTGATGGAAGCGTTGAGGTTCGATTCGCATGAACTGCTGATTATCCATCTCCCGCGTCATGTGCTGGTTTACGATGCCTCATCAAGCCAGAACGGGCCGCAATG
GTGCGTACTGAAAACAGGTTTATACGACGATGTTTATCGTGCCATCGATTTCATGTACGAAGGAAACCAGATTGCGTGTGGCGACAAGTCAGAAGCGGTGACAGGGCAGTTGCAATTCGAC
ATTAGTAGCCAGTACGACAAGCAGCAAGAACACCTGTTGTTTACACCCCTATTCAAAGCGGACAATGCCAGATGCTTCGACCTCGAAGTTGAATCATCCACTGGTGTTGCTCAATACGCTGAC
CGCCTGTTCCTGTCTGCAACCACAGACGGAATCAATTACGGTCGCGAACAGATGATTGAGCAGAATGAGCCGTTTGTGTACGACAAGAGAGTTTTATGGAAGCGTGTAGGTCGTATTCGTC
GATTAATCGGATTCAAACTGCGGGTAATCACCAAATCACCTGTAACACTATCCGGGTGTCAAATTCGTCTGGAGTAA
>PNNMHK_01605 Phage tail protein
ATGGCAGACCCGTCACTTAATAAGCCTGTCGTGGTTCAGGCTACACGCATTGATGCATCTATTCTCCCTCGTAACATATTCAGTCGGTCTTACCTTCTGTATGTCATAAATCAGGGTACTGATGTT
GGCGCTATTGCGGGAAAGGCAAACGAAGCAGGGCAAGGCGCCTATGACGCGCAGGTAAAGAACGATGAGCAGGATGTTGAGCTTGCAGACCACGAAGAGAGAATTCAACAGTTACGCA
TCGACGTAGACGACCATGAAATACGTATTACTGCAAATGCCAATGCAATTGCGGTACTGGATGTCAGACTAACCACGGCTGAAGGCAAAATAGTCACCTTGCAGGCTGATGTCAGTGCTCTT
GATGGTAGGGTTACGGCTGCTGAAAGCACTATTTCTTCATTGCAGGCTGATTACGTATCGAAGTCAGCAACTGCTTCTCAATCGCTGGCGTCACCTCTCAACGTGACAACGTCCTATTCAGTT
GGCGGCACTAAAGTTATCGGTGCGCGACAGACAGGATGGACAGCAGCAACCGGAGCAGCACTTCTCGGCACATTCAACGCTAACCAGGCATACACGGTCAGTGCCACATATACGCAGTCT
GAGGTATCAGCTATGGCTAACGGATTGCAGCAGGCGCGGCAGCGTATCAAAGCTCTCGAAGATGCAATACGAACTCATGGATTGATCAACTGA
>PNNMHK_01610 Protein gp14

ATGATTACATTCACTCCCACCCGAAACATCGACCTGATAGAAACGGTCGGCAACCATCCCGACATCATAGCCGGGAGTAACAACGGTGACGGATACGACTACAAGCCTGAGTGCCGCTATTT
CGAAGTGAACGTACATGGTCAGTTCGGTGGCATCGTGTATTACAACGAGATTCAGCCGCTGACCTTTGACTGCCACGCCATGTATCTGCCTGAGATTAGAGGATTCAGTAAGGAAATCGGGC
TGACGTTCTGGCGATACATTCTCGCCAACACCACCGTTCAGTGCGTTACATCATTTGCTGCACGCAAATTTCGCCACGGTCAGATGTACTGCGCAATGATTGGCCTTAAGCGTGTAGGAACCA
TCAAGAAATACTTCAAAGGCGTGGATGACGTGACGTTTTACAGCGCCACACGCGAAGAACTAATCGACTTCCTGAATCACGGGAGATAG
>PNNMHK_01615 Internal virion protein gp7
ATGTTATATGCATTTAAGCTGGGCAGGAAACTGCGCGGTGAGGAACCTTATTGCCCTGAAAAAGGCGGGAAAGGTGGCAGCTCTGATAAAAGCGCAAAGTATGCAGCAGAAGCTCAGAA
GTATGCCGCAGACCTTCAAAATCAGCAGTTCAACACCATCATGAATAACCTGAAGCCGTTTACTCCTCTGGCTGATAAGTATGTAGGCAGTCTTGAAGGTTTATCGTCTCTCGAAGGTCAGGG
GCAGGCGCTTAATAATTACTATAACTCCCAACAATACCATGACCTTGCGGGGCAGGCTCGCTATCAGAATCTGGCAGCGGCAGAAGCAACAGGTGGCCTTGGTTCTACAGCGACCAGTAACC
AGCTTTCAGCAATCGCCCCAACACTTGGTCAGCAATGGCTGTCAGGTCAGATGAATAACTATCAGAACCTTGCAAATATTGGTCTTGGTGCGCTTCAGGGGCAGGCAAACGCCGGGCAGAC
ATATGCCAACAATATGAGCCAGATTTCACAGCAAAGCGCGGCTCTTGCAGCGGCAAATGCCAACAGGCCATCAGCTATGCAATCTGCTGTTGGTGGTGCTGCCTCCGGTGCATTGTTAGGTG
GTGGAATAGCCAATGCTTTGTCAATGTCAACCCCATGGGGAGCGGCTATCGGTGGTGGTATTGGTCTGCTTGGTTCTTTGTTTTAA
>PNNMHK_01620 Internal virion protein gp20
ATGGCTACGTGGCAGCAGGGTATTAATTCTGGTGGTCTTCTGGCTGGCATTGGTGCGCAAAATGAGAATGCGCCAAAGGCAAGCGACATTAACGCAACGCTTGGACTGATTCGCGAAAAC
AATGATTTGGCTCGATCAGGCGCTAATAATGTGGCTTTAACAGGGCTGCGTGGTCTGGCTGGAGTTGCTGATATTTATAAGCAGGAACAGCAACAGAAAGCGCTAAACGCATTCAATCAGGT
TCATGCCAACGCATGGGCTACTGGCGACCCGTCTGGCCTGTTTAAGTTTGCTCAGGAAAACCCGGCGTTTGTTGCGCAGGCACAGCAGGCGTTTTCCGGTCTTAATGAGCAGCAGCGTAAC
GATATGGGCGATTTGGCTATGAAGGCTAACGTCGCTCTTTCTCAGGGGCCGGAATCCTACAGTAAATTCATTACTGATAACAAGGACAGGTTAAACCGTGTTGGCGCTAATCCAGACTGGAT
GATACAGACTGGAGTACAGAATCCAGAACAGCTATCACACATGTTGACTACGATGTCTCTCGGTGCGCTTGGACCCGAAAAGGCGTTTGCTGTTCAGGATAAGATGGTTGGCCGTGAGATT
GACCGAGGTAGACTTGCTGAAACAATCCGCAGCAATAAAGCAGGGGAAGGACTTCAGGCTCGCGGGCAAAATATTACTATGCGCGGACAAGACATCTCAGCTTCTACAGCCCGCCGCGGC
CAGGATTTGGCAATGCAAAGGGCAAACTCCAGAACGATATCAGGAGTCGACGGGAATCGGGTCGTTCAGCTTGCAGATGGTAGAACAGTCAACATTGACGGAAAACTTCACGGCGCAGG
GGCTAATGCATTTTACGAAGGTATTGACGATAACGGCAATATGGTTCGTGTCCCGGCAAGTGCTATTGCAGCACCTCCAACGTCTGCGGCAAGCGCACAGAACTACGCGATGAAGAAAGATA
TCGACGCAATCGCAAACGCAGATGCTTCTGCTCTTGATTTTATGACTGGAATGACTGGTGGAGCAGGTGATCCGGCAATTGGTGCAGATGTTCGCAGCCGACTCACAGGCAAAGAGCAACG
CCAGTTATATAACTCAGCACAACGTATTCAGGGAAGAATGCAGAATCAGGGCGTGGCAGCAGCAAGAGATATGGGCGCTAGTGGTATCAACACCATTGCAGAAGCGAAGATGTATTTTCAG
GGGATGCCGCAGGTTGACTACTCAAGCCCGGAGGCTATGCAGCAGTCTATTCGTGAGATTCAGGAATACACCAACAATTATAACAAGCAGTACAACGTTAATGTTGGTAAATCGCAGTATCA
GCAATCCCAACCTGTACAGAAATCACAGCCTGCATCCAACAGCAACTTTTCTTCACTATGGGGTGATTAA
>PNNMHK_01625 DNA transfer protein
ATGGCTAAGGCATGGAAAGACGTTATTGCCTCTCAACAGTACCAGGCATTGGCACCAGAACAGAAGGCACAGGCTCAGGAGCAATACTTCAATGAAGTGGTTGCACCTCAGGCAGGCGAT
CAGGCTGAGCAGGCTAAACAGGCTTTCTATGCTGCGTATCCGGTGCCGACAACTCAGCAACAATCATCACAGCAGGAAGAACCACAGCAGCCTGAGCAATCCCTGATGCAACGGGCTGGT
GACTTCCTCACTGGCGGCCAGTCAGCAGGACAGATTGCAGAGCAGGCTGGGCGTGGGCTCGTAAATATCCCGTTTGATGTATTGCAGGGTGGGGCGAGTCTCATTAACGCAATTAGTCAG
GGGTTAGGCGGACCGAAAGTGCTTGATGACGTGTATCGCCCGGTAGCTCGCCCAACTGACCCATATGCGCAGGCTGGCGAGGATATCGGTGGGTATCTCATTCCAGGCGCTGGAGTCGCTG
GCAATATGGCGATCGGCTCAGTTGCTGAGGCTGCAAATCAGCAGGGTGATTTTGCTAGTAATGTTGCAAAGAATGCCGCCATCAACCTTGGGGCACAAGGGGCTTTATCGGCTGTTGCAAA
AGGGATAGGAAGAGGAGTTACAGCACTTAGAGGTGATATTTCTCCTGCAGATCAGCAATTGCTCAAGCGGGCTGCTGCGGCAGATGTGCCAGTTATGACATCAGATGTGGTTCCTCCTACA
ACAAAACTTGGCAATCAACTGCAGGATTACTCAGAGGGGGTCATCGCTGGAACTGGACCAATGAGAGCTGCTCAGCAGGATGCCAGATCCAAGCTTGTTAATCGTTTCACCGAAAAATACG
GCGACTATGATCCATCGGTAGTAGTTGATAGCCTAAAGTCAGGTGTTGCAAGGGAAAAATCGTTAGCCAAGTCAAAGCTAAACGACCTGTCAGGTAGAATGGTTGGTAAGACAGTTGATAC
AAGTGGTGCCATAAGAGCTATCGACGGCGCAGTAAACGAACTTGGGAGACTTAAAGGTGTTTCTGATACCCAAACCATTTCTGCGCTTAATGATTATAAGAAGGCTATTCAGGAGATATCTAG
TGGTGATGATGCCTTTGAGTTGCTTGATAAGCTAAGAACTCAGTTTCGCATTGACGTAAAAGGCGATCGCACAGTTCTTCCATCACAGTCGCAAACAATGATAGACAGGGTCTACAACTCGTT
AACCAATAGCCTTAGCAAATCTATAGCGAAAGGTCTTAGCCCAAAAGACGCTTCAGCATGGCGAGCAGGAAAAGCTGATTATGCAAAAATGGCAACACATGCAACTCAGACGCGCCTTAAA
AACGTTCTAAACAAAGGCGATTTGACTCCAGAGGCTGTAAATACCATTGTTTATGGACAATATGGATCAGATATAGCTCGATTGTATGGAAAACTCGACCAAAAAGGTAAAGACATGCTAAGG
GCTGCATATATCAGCAAAATTGCTGATAAGGTAGGTGACAGCCCTCAGCGAATGATGACTGAGCTTGGTAAGTTACAAAAACAAGCAAATGGTCAGGTATTTAAAACTGTATTTGGCGGGAA
GAACGGCAAAGAGATAGAGGGAATGCTATCTATTCTCGAAGCTACCAAAAGAGCATCTGAGGCCAATGTTGTGACAAAAACTGGCATGACACTTGCGCCTTTAGTAAGGGTTATTGGTAATC
TAAAAACTGGTGGCGCTCTCTTGGCCGGTGAAACAGGAATTGGCCTTATGTCTAGGGTTTATGAAAGCCCTATGGCAAGGAATGCGCTCTTACGTCTGGCTAACACAAAAGCAGGAACGCC
AGCCTATGAAAGAGCGCTAAATCAGGCTGCTACTGCCGTGCGCCCGCTCTTAGCTAACGAAGCTACCAGGCAGTAG
>PNNMHK_01630 Superinfection exclusion protein A
GTGTCTGATTCTATGAGTTACGCTGTGCTAGTTGCCGCAACTCTATTTCTGGGGATAGGGTTGCAGATTGCGTGGTGGTTTTTTTCTAATTTTATTAAACGTAAAAGAATTGAATCAAGGATAT
CTGAGATTTCTATTGCAATAGGGAAAAATGCTGAAAATCCAGAGAATGATGCTTGCGCACTCAATTACCTTAAAGAAAAGTTTTCCCCTGAAAGATTTGAAAACAGAATCACTGATGCTCTTG
GATTGGTAATATCAGTAATTCATATACCACTAAGTTTGCTGATAACAGTGTGGTACTTCGCCATGATCGCCGGAAGAATATTTGGTTTCATGAATATAGAGCCTGTAGTTCTTTGGGTTCCAATG
ATACTGCAATTGTTGTTAAGCGTTGCTATCTTTATTTTTTCTGTTTTTATAAAAAATTGTCTTTGGAAGATACCCCGGAGAAGCGAAGGGATTTAATAAAGAATTCATAAAAACTATAAAATAA
>PNNMHK_01635 PEGA domain-containing protein
ATGAAGAGGATTATTAGCATCGTTGCTGGCGCTATCATGTTATCTGGGTGCGCAACTATTGTTGGTGATGAAACGCAACTTGTGCAAGTGAACAGCAATCCTTCTGGTGCGAGCTTTAAGGTA
AAAGATGAATCAGGCGTGATTGTTGCGCAAGGTAAGACTCCACAAGGTGTAACACTCGCCAAGTCAGATGGTAGCTATTTTGGCAAAAAGAGCTACCAGATCACTATGGAGAAGGATGGG
TACGAACCAGTTACCCTGCCAATCAAAGCTAATGCTAATGGTTGGTATATTGGTGGAAACCTTGTGTTTGGTGGGTTAATTGGTTGGCTTGCTGTAGATCCATTTAATGGTGGGATGTATACCT
TGAAGCCAAAAGAGGCAAACGCATCTCTTATACCGTCAACAAAGCAAGACTAA
>PNNMHK_01640 Regulatory protein mnt
ATGGGAAGAGATGACCCGCAATTTAATCTGCGGCTACCTTACGAATTAAAGGAAAAGCTGAAACAGCGAGCTAAATCGAATGGCCGCTCACTGAATGCTGAATTAGTTCAGATAGTTACCGA
AGCTATATCTAAGCCATCTCCAGTATCAGGTTATCGCGACGAAGCAGAGCGCATTGCTGATGAGCAGTCCGAACTTGTTAAGAAAATGGTGTTTGATACGCTGAAGGATTTGTACAAAAAAC
CCACCTGA
>PNNMHK_01645 hypothetical protein
ATGACAGATATCACTGCAAACGTAGTTGTTTCTAACCCTCGTCCCATCTTCACTGAATCCCGTTCGTTTAAAGCTGTTGCTAATGGGAAAATTTACATTGGTCAGATTGATACCGATCCGGTTAA
TCCTGCCAATCAGATACCCGTATACATTGAAAATGAGGATGGCTCTCACGTCCAGATTACTCAGCCGCTAATTATCAACGCAGCCGGTAAAATCGTATACAACGGCCAACTGGTGAAAGTTGT
CACCGTTAAGGGTCATAGCATGGCTATCTATGATGCCTATGGTTGTCAGGTTGACTATATTGCTAACGTATTGAAGTATGACCCAGATCAGCTTGAATACAGGCTGAGCCAACCAGACGGTTAT
CTTTTGGTTGGTGGACTGGCCGAGCATTATAACCTTCCGGCTAAATTTGTCGTCGTCGACAACGAGCCATATAACGGTGATTTGAAAGCAGCACTTTCTGAGGCTGAAGCCGGTACTGTGTT
TTGGCTTGGTAAAAAAACATACAACATTACCGGCCTGTACGGAACTGGCAGGAACACAGTTGAAAATATCTCTATTGTTGGCACTGGGATGCCTCAGTTATCTGATGATAAAACCAGATTTAT
CGATGGAACTGGGACAGTTATTCAAGGGGCGGTAAAGAATCAAGCGAGAGGGTTTAAAACCTTCAACCTTGGGATCGATGTTGGTGCTTATGTGTCTCAGAATGTGTATACAACGGAAACT
TACGAGGACGCTCTTGTGCACTATGGTGTTGGTTCCAACGCCAATATAGAAATTGACAATGTCAAGACGCTGAGTTCAGTTAACGTTGCCAGTAAGCCAGGAACGCACAGCATTCTTCTTGA
GCAATTATCCGGTGTAACCTTGGGTTACGTTGAGTGCATTGGTGGTTTTCATGGTCTAACGATTAAGTGTCAAAATTTGCAGGGCGGCATTGCTCATTGCTACGGCCAATATGGTGATGCATTT
ATCTTCAAATCAGATTCTGGTGGCGCATGCGCCAGTAACTATATGGAAAGAATTGCAGTTGGTCTTTATGACAACGCTGGCTGGCCTGACGTCACCATGGGTGGTATTTACGACGCCCACGAT
GATGTAACAATCGACAGAATTGGCATTGGTGAGTTAATTGTGCAGAACGCGTCATGGGGATTTATACCATCTGATGCCAATACCGGTTTCATAACAAACGTCAGCATTGGTAGATACTCTGCAT
TCAATGTCTATGGGAACTATTATTCATTAACCATTGATAATAAATGTGTTGGTTGGACTATTGGTGAGCACAGAATTAGCAATGCATCTGGCGGCATTCGTGTTCACCCGGACTCTGCAGAAAT
CAATATTGGAACCGGATCCGCAAAGGGGAATACTGAGAGTGGCTATGCGTTGGGAGGCAACAGTTTAAGTCACGGCGTGCTCTTTGCTAATGAGAATGGGAAGGCTGGAGTTGATTACCT
CGGTGGTATTGGTTTTGATGCCTCTCTTGTCCGTGGTTATGTTAACGGAACGGTTCTTGTTTCAGGATATCCAGGCGTAAAAGACGGTAATCCTGTAAATGGGTGGGCTGATACTGGTGATTT
TGACATGATGCTAACCGGCAAGACTGTGCAGATCACAGGTTCACTGACTCGCGGAACAGCTGCGGTTGCGTATAACACTATCGCTGCGTGCAGGCCTTTGAAGCGAGTGCCTGTCCCGGCA

TGGGGTGTTAGCGCGACAAGCTCTATGATCCCAGTTGAGTGTTATATTGAAACTAACGGTCAATTAAACGTAGCTGGGTTCGCCTCGATACCAACTGGCGGAACTGTATATTTCTCTGGACAA
TATTTAACAAAGTAA
>PNNMHK_01650 Phage tail protein
ATGAACAGAAGAGATTTCCTAAGTCTTTCGGCATTTTCCATGAGTGGTTTACTGGGGTCAAAAATGGCGTTTGCAAATGATATCCTTGTTGCTCCTTCTTTAACCATAGTAGATAACCCTCCTTA
CAACGGGGATCTCAGGCAAGCGCTACAGGAAACTACGCCTGGAAGCGTCCTGGTTCTTGGCAAATCGAAGCCTTATGACCTTACTGGATACTTTGTTTCTCCCACGCATACAGATGGGGAG
CCCGTAACCAACACAATAGAAAACCTGACCATTATAGGGATGGGGATGCCAAGGCTTGCAGATGATAAATCGCACTTCATTCCTGGGTCAGGAACTATAATACTTGGTCTTATTATGAATAAG
GCCAAAGGATTTCATATAGAAAACCTTGGAATTGATTGCGGTAATTATGTCTCTCAGAATGTATTCCCCAATGTCACCTATGAAGATTGCCTTCATATTTATGAAGCTGGTGACAATTCCAATATC
TTCGTTAACAATATCAAGACGCTTAATTCTCTTGGGGTTTCTTCAAAGCCAGGGACTCATAGCATTCTGATTGAAAGAACCGGTGACGTTTACAAAGGATATGTAGAGTGCATAGGCGGGTTT
CATGGCCTAACTCTAAAAGGGTGGAATATCACCGGGGGATGGTCTCACTGCTATGCTCAATATGGTGATGGGTTTATTCTTAAATCTGATTCAGGGACTAAATGCAGGGATATTCATCTTGATG
GAATCAAAGTTGGTCTTATTGATAACACAGGATGGCCTGACATATCTATGGGGGGGATTTACGACCCGCATGACGGGCAGACTATTGACAGGGTGACCATAGGAGAACTCGCAATTCAGGG
TGCCGCTTGGGGTCTGGTGGCAGCATCATCTAGTGATGGATATACTACTAACATCAACATCGGCATATTCTCTGCCATTGAGGTTTATGGGAATTACTACGCACTTGAAATAAATGATAAGTGC
GTAGGGTGGAGTCTTGGACAGCATGCGATTTCTGCAGCATCTGGAGGTATAAGAGTTAATAAAAATGCAGCTTATATTGATATAGGTCACGGATACTCAAAGAACAATACAAGAAGCGGATAT
TCTCTTGGTGGCAACACTTTAAGTCATGGAAGGCTTTTAACTAATGAAAATGGTGAATATGGCGTTGAATATACTGGCGGTTATGGCTTTAACAAAGATGAAGTTATAGCATATGTCAATGGAC
TAGGAAGCTTTAATGATTTGCCATCAGCTATTCAGGGCAATCCAATAAATGGGTGGGCTAAAGACAAAAACTTTAAAGCTATAGTTTGCGGTCATCGCGTATTCATTAGCGGGCGATTGAGAA
AAGGAACCGCTTCACCTGCATTTTCTATTCCTGCTGCATTACTACCCAAAGAGGATGTGCCTGTGCATGGTGTAGGGGTAAATCTAGGTGTTAATGCTATCCCCGTGCAGGCTTGGGTTAGAT
CTTCAAACTCACCAACTAACCCAGGTATGTTCGATGTTTGGGGGTTTGCATCAACGGATTCATACGTAGATTTCAACAGTTCATATGACATTGCCTGA
>PNNMHK_01655 tRNA-Ser(cga)
GCGCAGGTGTGACGTGGTATGTTGTTGACTTAAAAGGTGGTTCTAGGAACTTCTAAGCCGTGGGTCGCAGGTTCGAATCCTGCAGGGCGCGCCA
>PNNMHK_01660 PMT-2 domain-containing protein
ATGACCAAAAAGGGGCTTTCGGTAATATTAGTTTTTTTGATATTTTCATATATTTTTACCGCATTAAGCTATAAATTTATCCCAAGCTCTGACAGCATGAGTGGTATTTTAGAGGCTGCTGACATT
GCAAACGGAAACATAACACTAAAAGGATGGTACTTATCTACAGTAACTTTCTATTTTACTGACTTAGTCTGGTTTGCTCTTGCTATAAAGCTTTTTGGTTATTCTGAATGGATAACATACGTTATA
CCTGGATTAATGGCTGGTAGCCTGTTCGCTTCATGCTATGCACTGGGAACAATTTCTGGCTACAAAAAAGCATGGGCTTTGCTACTGTTCCTTGCTTTCCCTGGTGCTGCTGTCAGTTACATGC
TTTCTGTAGCGATAATCCATGTCCCTACATATACTTATATCGTTGTTTCATATATATTAATTGATTTTTATTGTCGCAGAAGAAATAGATTATATTTATTTCTATCATCAATAATCGCATCTTTAACGATA
TTTAGCGATGATATAACAATATATTTATTTTTTTTGCCAATTGCATTGAGCTGTTTTATAGCCAATGAAAATGCAAAAGATAAATTTGTAATATTTTCGTCTTTGGTTTTTTCGTATTTTTTATTCAA
GTTAATCTTACATTTTACTAACTCGGCTGATTTTTTTTATTTGCCAGGGGTTGGTTCGCCTACATTTGTTAGTTATGACAAGTTAACTTTTAACATCTCGCTACTTTTTAAAGGGCTTTTGATATTA
TTCAACGCTGATTTTTTTAGTAAAATAATCAGTTCACCTGAAGGAATATTCTCTTCTTTAAAATTCACATCATTAGTTATATTTTTTATACTTTTAATTTCTTCGCTTATAAAAATAAGAAAGTTTAG
TCTCGTTGACGCCGCGCTATTGATAGCATCTCTTATTATGATTCCTGCATATGCCTTAAGCGATAAACCAGTGGATGAGGGTACAACAAGATATTTAATTCCTGTCATTATTTTTGGTTCAATTTTC
TTATGTCGAAATGCGAATGTACCAAAGATATCAAATATAGTTTTATGGTTTTTTTCAATTTCAATTTCTGCTTATTCATTAATATATGTAAATCAGCCTGATTTCTTATTTCGCAATGACAGAACCAC
ATCAAAATATAGGCTTATATCTAATTTTTTGACTCAACACAACTTATCTAATGGATATGCAACATTCTGGAATGCGGCAGCGGTGAGTGTGGAAAAGAAATTCAATATAGCCCCTGTTAACATCG
ACATAGAAAATAAAAAAGTTTTGCCATCTTTTTGGTTAACAAAAATATCATATTTTAACAATGGAAATAACTTTTTCATTGTTGATAATGACCAACAAAAAAAAGTCATAGAAGAATTATATGGC
AAACCAGAATTAACATATATGGTGTGGGATTCCCCATCCTGGTTTACAGTCATTCTATTAATATTTATGATGGCGATATAGAAGGAAGTGCCAATGTAGTAAAAAGTGACTTCAAGGTTGGGG
ACAATAATCAAATATGCAATGCTGGCGTACAAGGCATGGTTGCATATGGGCCCTATAAGACTCTTGGTCGTGGGTGGTATTCTTTAAAAATTAATGCACATGGCGATCAGTATGAAGCATTAAT
TTTTTCTTATATAACAGGAAAAAAAATCAAGATGTCTGAGAATAAATATAAAAATGGTTCTTATATTTTCGAAATAAACGAAGATATGCCATCTGCAGAAATACAGTTATTCGCTCAAAAAGATT
CAAATGTATGTTTTGAGTCATACTCACTTCAGCATATAAAATAA
>PNNMHK_01665 Bactoprenol glucosyl transferase
ATGAAAATCTCTCTTGTCGTTCCAGTTTTTAATGAAGAAGACACGATACCGATTTTCTATAAAACGGTACGTGAGTTTAATGAACTAAAAGAATATGAAGTGGAGATCGTTTTTATTAATGACG
GAAGCAAAGATGCAACTGAATCAATAATTAACAAAATCAGCTGCATCTGATCCGCTCGTTATTCCGCTTTCGTTTACGCGAAACTTCGGTAAAGAACCTGCTCTTTTCGCGGGGCTCGACCACG
CAACCGGGGATGCGGTCATTCCTATTGATGTCGATTTACAGGATCCGATAGAAGTTATCCCCCATCTCATTGAGAAGTGGCAGGCTGGCGCAGATATGGTGCTGGCTAAGCGCTCAGACCGC
TCAACTGACGGGCGCATGAAGCGTAAGACAGCTGAGTGGTTTTATAAGCTGCACAACAAAATCAGCAATCCGAAAATCGAAGAGAACGTTGGCGATTTTCGGTTAATGAGTCGTGAAGTT
GTCGAAAATATTAAATTAATGCCCGAACGCAACTTGTTCATGAAGGGTGTGCTGAGTTGGGTTGGGGGACACAGTGATGTTGTGGAGTATGCGCGCGCGCAACGTGTGGCTGGCAACACT
AAATTCAACGGATGGAAGCTATGGAATCTGGCGATAGAAGGTATAACTAGCTTCTCAACTTTTCCTCTGCGTATGTGGACCTATATCGGTCTGGTTGTCGCAGGAATGGCTTTTATTTACGGG
GCGTGGATGATTTTGGATACGCTCGCCTTCGGAAACCCAGTAAGAGGATATCCTTCATTACTCGTCTCCATACTTTTCCTTGGAGGGGTGCAGTTGATAGGGATAGGCGTGCTGGGAGAATAT
ATTGGCAGAATTTATATTGAAACCAAAAAACGTCCAAAATATTTACTTAAGGATATTAAAAAATGA
>PNNMHK_01670 Prophage bactoprenol-linked glucose translocase-like protein
ATGTTGAAGTTATTCGCTAAGTACACATCGATCGGTGTTCTTAACACGCTTATTCATTGGGGCGTATTTGCTTTTTGTGTGTATGGGATGCATACGCATCAGGCGTTGGCGAACTTTTCCGGTT
TTGTTATCGCCGTATCGTTCAGCTTCTATGCCAATGCGCGCTTCACCTTTAACGCCAGCACCACCACGCTTCGCTACATGATGTACGTGGGATTTATGGGAACACTGAGCGCTGTTGTTGGCTG
GATGGCTGACAAATGCTCCTTGCCACCACTCCTTACTCTTATCACCTTTTCAGCTATCAGCCTGATATGCGGATTCATTTATTCAAAGTTCATTGTCTTTAGGGATGCGAAATGA
>PNNMHK_01675 tRNA-Ser(cga)
GCGCCGTAGTAATGGGATAACTTGTTGTTAGCTCAGGTAGTTCCAGGAACATCTAAGCCGTGGGTCGCAGGTTCGAATCCTGCAGGGCGCGCCA

Prophage 16

>PNNMHK_03680 DUF3799 domain-containing protein
ATGGAATTTTTCTATGTAGTAAAAGCTACGCAGAAATCCGGAAAGCAAGATGCGACGGTCTGGTTCACTGCAAAATCAGAAGCGCGCGCCAACCTTATGCTGGATGTCGTTCTGGAAGATG
CTGAAATTGAAACCGGCCGCGGTAAGGATTATGCAAGGCCGATCCGCACCAATTTTCCGGTAGTCAACGAGCTGCCGCCGGAAGGTGAAATAAGTTTTACCTTCACTAATTATTATCACCTC
GGTGAAGATGGCATGACTTGGGAACAAATCCCCGGCGTCACCCTGCCATCATCTGAAGCCGCCGCCGTGGCCCGCCAGCACATCGTTGACGGTGTTGATACCGAAACAGGCGAAGTGCTG
GAAGACCACACCGAAAATTTTGGTAACGAAAGCAACAGCCCTGCCCAGGCAACAGCCCCAGCCCCCGAGCTGACTGTTGTCGCAACTATGCCTCTCCGTCACCGCGTTCTTGCTCAGTACA
TAGGTGAAGGTGAGTATCTTTATCACGTCGACGCCTCCCAGAAAAAAGAAATTCTGCGTCTCGAAATGGACACCGATAATTCATATGTCCAGAACCTGCTGCTTGCCGCCGAGAATGTTGAA
GCGTTCAAGAAAGCCATTGAACATGACATTCACAAAATAGTGAATGCCGTTAAAAAAGTATTCCCTGTCGATGGAAAAACTCCTGAACTGGCGACTGTTATCCAGTTCCTTAAAACATGGTTC
GAGACGGAGCATATCGATCGCGGTTTGCTCGTTAAGGAGTGGGCGAAAGGCAACCGTGTATCGGCTATTCAACGCACTGAAAGCGGCGCCAACGCTGGCGGTGGCAATAAGACTGACCG
TAACCCTGATTACGAACACACTCTCGATACTCTGGACGTAGAGATTGCAATGGCCACTTTGCCTATGGACTTTAATATCTATGAGCTACCTGGCAGCGTTTACCGTCGCGCAAAAGAAATCGTA
AAGAAAAAGGAAAGTCCGTTCAAAGAATGGTCCGCAGCACTTCGCGCAACGCCCGGTATCCTGGATTATTCCCGCGCCGCTATTTTCGCGCTGATCCGAAGCGCACCCCCTGAGTTTTATCA
CTACCCCGGACGCCTTCAGGGGTATATCAACGCCAACTTAACGGAGACTGATCACGAGAACCCCACCGAGGAAGCTCTCACGGCTGCCCGACACACTCCGGAAAAAGACGCGGTAGAAGA
AGCCAACCGACAGCTTGCCGCCGCGCGCGGTGAAATATGTGGAAGGCATCAGCGACCCGAACGACCCAAAATGGGTGAAGACCGGGACAAGCCAGCCGACCACCGAACCTGAACTGGTT
AAAAATGTTGGCAACGGTATTTTCGACGTGTCCGCTTTAATGCAGAACTCATCAACTCATGGCACAGAAACGAATCCGGAGACCACCAGCAATGTGCAGGTTCAAAAAGCTGACAGTGATG
AAAAACAGGCTGGTGATGCGGTGCAGGCAGGCGAAGGCGATCTGGGTACTGGTAAAGAAGCAGTTACCGTAGAGAACCAGAATCAGGCTGAGACGCACCAGAACAACGATTCTGTGAG
CCAATCTGAACCTGAGGCGCAACAAAACGTACCGGAATCGCAACAAGAAGAGCCAGAAGCAGCCTGGCCGGAATACTTCGAGCCGGGCCGCTATGAAGGTGTACCAAACGAGGTTTACC
ACGCCGCCAACGGGATCAGCTCAACTCAGGTGAAAGATGCTCGCGTGTCGCTGATGTACTTTAACGCGCGTCACGTAGAGAAGACTATCGTCAAAGAGCGCTCTCCAGTGCTTGATATGGG
CAACCTGGTACATGTTCTGGCTCTACAGCCGGAAAACCTCGAAGCGGAGTTCAGCGTAGAGCCGGAGATCCCTGAGGGTGCTTTCACCACCACCGCCACCCTGCGCGAGTTCATCGACGC

GCACAACGCCAGCCTGCCAGCGCTGCTGAGTGCTGACGATATCAAAGCGCTGCTGGAAGAGTACAACGCCACCCTGCCGTCGCAGATGCCGCTTGGAGCTTCGGTAGATGAAACCTATGC
ATCGTATGAGCAGCTTCCCGAAGAATTCCAGCGCATTGAAAACGGCACCAAACATACAGCCACGGCGATGAAAGCCTGCATCAAAGAGTACAACGCCACCCTGCCCGCGCCGGTTAAAACC
AGCGGCAGCCGTGACGCGCTGCTGGAGCAACTGGCAATAATCAACCCTGACCTGGTCGCTCAGGAAGCGCAAAAATCGTCGCCGTTGAAAGTCTCTGGCACAAAGGCCGATCTGATTCAG
GCCGTGAAATCAGTCAACCCGGCAGCGGTATTCGCCGACGAATTGCTGGATGCGTGGCGGGAGAACACCGAAGGGAAAGTGCTGGTCACCCGCCAACAGCTCAGAACCGCGCTGAACAT
TCAGAAAGCCCTGCTGGAGCACCCGACCGCCGGCAAATTGCTGACTCACCCAAGCCGCGCTGTCGAGGTTAGCTATTTTGGGATTGATGAGGAAACCGGGTTGGAAGTTCGGGTACGCCC
TGACCTTGAGCTCGATATGGGCGGCCTGCGCATTGGCGCCGACCTGAAAACTATCAGCATGTGGAACATCAAGCAGGAAGGCCTGCGTGCGAAGTTGCACCGGGAAATCATCGATCGGGA
CTATCACCTGAGCGCGGCCATGTACTGCGAAACTGCGGCGCTGGACCAGTTTTTCTGGATTTTCGTCAACAAAGACGAGAACTACCACTGGGTCGCCATCATTGAGGCGTCTACCGAGTTG
CTGGAACTTGGCATGCTGGAATACCGCAAAACAATGCGAGAGATAGCAAACGGCTTCGACACTGGTGAATGGCCAGCGCCTATCACAGAAGACTACACCGACGAACTGAACGATTTTGAT
GTGCGCCGCCTTGAAGCGTTGCGCGTACAGGCATAA
>PNNMHK_03685 hypothetical protein
ATGCTTAAACAATGCGGTTACTGCCGCAAATCCATTGATGAAGGCAAAGAAGTAAAAAACACCCTTCTCTATCGCAACGGCTCGCAACTGGCGCGCAAAGAAAAGGAATATCGTTCCAGGC
AGTGCGCTGAATACGACCAGATGGCGCACGAAAGTTAA
>PNNMHK_03690 Prophage protein
ATGCCTCAAAATAGAACTTTTAGTTGCGATTTTTACTTTACAAATCGAACCTTTGGTTTTATTGTTGATTTCATCGACAACAAGCGCATCGTTGTCAGGTGTAAAACGTTCCGCTGGCCGGCGA
TAAGGCAAACGAGGGTGAGAATGATTGATTTCGCACGTAAACCAGCTCGACAGCAGGCTGTCCCGCTCAACCGGATTGAGGTTTTAATCCGCCGCCTCTGCTACCTGCTGGCGCAGAAAG
GGGATCCGGATGCTTAA
>PNNMHK_03695 Helix-turn-helix domain-containing protein
ATGAACAAAAATCTTCATCCCATTTTCGCCAAGCGTATCCAGCAAGTTCTGGATGAGAACGGCTGGTCTATGGCTGACCTCTCACGGCGCGTAATGCTTTCTCACACATCTGTGAGAAAGTGG
GCCTCTGGCACATCTGTAGCCAGCGGAGAGCGCTTGAAAAGGTTATCGGCGGTGACCGGAAGGCCTGAATATTGGTTCTTCATGGAGCCAGGGGATGAAAGTGAAGGCGAAAGGGATGA
ACCTAAACCCAGAGTCCTTGATGAAAAAGAAGAAACATTGCTTTCTCTTTTCAATCAACTCCCGGAAGCAGAGAAACTGCGTGTTATCCTCCATACAAAAGCAGTCCTCCAAGAGATGGATC
TGCTGAAGAACAACGTTTTTGATCTAATTAACGACCTCAAAAAATAG
>PNNMHK_03700 Helix-turn-helix domain-containing protein
ATGGACAACCAATTAAAAATCAAAATCAGTAACCACATGACTCAAATGAGCATCGGCGAACATTTTGGTATCTCGTCTCAAGCTGTAGGCAAGTGGCTGAGAAAAGGAGTAATTCCTCCCCG
TCGCATCCTGCCGTTATGTGAAATTCTTGAATGGAAAGTCACTCCTCACGAGATTGACCCAGCAGCATACCCAAACCCAACCGATGGCTTACCAAGCCAAGAGGCATCAGCCAAATAA
>PNNMHK_03705 Regulatory protein CII
ATGGAGAACGCAATTGCACGAAAGTTAGACCCACCAGAAATCAACCCGGTTGAGATAGAGAGTGTCCTGCTCAACCGGCTTGCATCAGTAGGGCAGAAATCATACGCCGAGCATATGGGC
ATCAGCGAGTCGACAGTCAGCAGGCGTAAAGCTGAGGGATATTTCTGCAACATGGCGAAAGAGCTGGCTTTTCTTGGGATTCAGGCCGCGCCACCGGAGGCGGTACTGGTATCCAGAAAC
TATCTCACAGCCGTAGAGATTCTCGCTGATGCAGGGCTAAAGGCTGAACGAGCCAGGCCGGATGCGCTGGGGTGGGACTGA
>PNNMHK_03710 Phage replication protein O
ATGGCAAACACTGCCGAAGTAATCAATTTTCCTGTGCCTGACGTGGCACCTAAGGAGCCGCGCGTGGCAGATCTCGATGATGGCTATACGCGCCTGGCAAATGAACTTCTGGATGCCGTGAT
GTGTTCTGGTTTGCCGGAGACTGAGCTGTGCATCCTGATGGCCGTATGGCGCAAAACGTATGGATACAACAAGAAAATGGACTGGATCAGCAACGAGCAGTTAGAGGAGATGATTCAGAA
GCATCATACCCATTGCTCGACAGCAAAAAACAGTCTGATCAGGAAGAAGGTACTGATTCAGGAAGGCCGCAGGGTTGGTATGAATATCCATATTTCCGAGTGGCAAACTAAAATAACGGAT
TCTGCAAAACATTAGCTAAACCTGCTAAGAAAACCTTAGCGGAAGTTGCTAACGCACCTAAGCAGAAGTTGCTAACCACAAAAGACAAACTAACAAAAGACAATATTAAAAGATCTACGTCC
GAGAATTCTGACGAATCCTCTGACAAACCAAGAAAGAAACCTCATGTTCTAAAACCCGAAGCAGCGATTCAGAGAGGCAACAAGTGGGGAACTGCTGAAGACCTAACTGCTGCCGAGTG
GATGTTTGACCTGATAAAAACCATTTCTCCATCAGCCAGAAAACCTAACCTGGCAGGATGGGCTAACGATATACGCCTGATGCGTGAATGTGACGGACGAACACATCGCGACATGTGCGTGC
TGTTTCGCTGGGCGTGCCATGACAGCTTCTGGGCTGGCAACGTCATTAGCCCGGCAAAGCTCCGCGAAAAGTGGACTCAACTCGATATCAACCGCAACAAGCAACAGACTGGCACAACTG
CCTCTAAGTCAAAACTTGACCTGAATAACACTGACTGGATATACGGAGTGGAGCTATGA
>PNNMHK_03715 Replication protein P of bacteriophage
ATGAAAAACATTGCTGCGCAGATGGTTAATTTTGACCGTGAGCAGATGCGCCGTATTGCCAACAACATGCCGGAACAGCATAACGATAAGCCGCAAGTTGAGCAGGTTGCTAAGGTCATCA
ACAACGTGTTTAGTCAGCTTATGGCAGCGTTCCCTGCTACCACAGCTAATCGCAGCCAGGCTGAGATGAACGAAATCCGGCGTCAGTGGGTTCTGGCTTTCCGTGAGAACGACATCACCAC
CATGGAGCAAGTTGCGGCCGGAATGCGTGTCGCCCGCCGTCAGGAACGTCCGTTTCTTCCGTCTCCAGGCCAGTTCGTCGCATGGTGTAAGGCTGAATTGGCAACTGCTGCCGGGCTTCCT
GATGCCAATGAACTGGTCGATATGGTTTACCAGTATTGCCGCACGCGTGGTTTATACCCGGATGCAGAATCATATCCATGGGAATCCAAAGCCCATTACTGGCTTGTCACCACACTGTACTCAA
ACATGCGAGCTAACGCTCTTAGTGATACCGAGTTGCGCCGCAAGGCAGTCGAAGAACTCAACCACATGGTTACCAGGATAAACCGTGGAGAGGTGATCCCGGAGCCAGTTAAGCAGCTTC
CTGTTCTTGGTGGCAGACCACTAAACCGCGCGCAAAACCTCGCGAAGATTGCAGAAATTCGGGCCAAATTCGGACTAAAGGGAGTCAGATCATGA
>PNNMHK_03720 Chromosome partitioning protein ParB
ATGATAACTGCATTAGATATTGAAAAAGTAATCACCGATAAAGGGCCAATGAGCAACATCAAAGGTCCGCTTATCAGCAGTCAGCGCTACCTCGACAAGGCAAAGGTAAACGACAGAGCGG
CAAGATTTAAGCGTTTTATCGTATCTGTTTACCCGATAGTTCTGCGTGGGCAGCAATACACCATCCTGATGGATGGCCACCACAACTACGCGGCGGCAAAACTGGCTGGCATAGAACCTGATT
ACCGACCAATCACCAAAAAGGTGCAGCGTATTCTCGGTGAGATGTCATGGCGCGAGCGTGAGGCATTCTTCATCAACAACGTTACAGACAGCAACTACTACTTTGTTGAAACAGGCGAAGT
GGTTCATGAGTTGGTTATGCCTGACACGTCCTGCAAATTCCAGTCGCACGCAGGTAACCAATGGATTTTTGGAGGTGCAGCATGA
>PNNMHK_03725 hypothetical protein
ATGAACATCGACAAACGTGCGCTGCGTGAAGTGGCGGAGAAGGCTACGCCGGGGAATTGGCGCCGCACCTCATCACTGTTCAATGGCATCACGGTAACGCCGTTTTCTCTCTGCGGTGAA
GAAGTGACGTTGGCCCATACTGTTGAGAAACGTGACGCGGAATTTATCGCCGCAGCCAACCCCGCCACCATGCTGGCGCTGCTGGATGAACTGGAGCATTACAAATCACGCGAAGAGAAA
GTCACGCTGGAAGAGTTTAAATGTATTAAGGAATAA
>PNNMHK_03730 Nmad5 domain-containing protein
ATGAACCAGACACTGAATAAATCCATTAAAGAGAAAATCGTGGATAATGCACTGGCAAAAGCTGGTATTCCGCAACGGAAGAAAGCCCTGCGTGCTGCCCGGGCAGACTGGGCGGAACGT
GTAAGGCTGGCGGCAATTGGCGGACCGGAAGCAGAATCTGAGGTATTAAAAACCGAAAAGAAAATTGCAGCGCTTATTGCGAAACTGCCCGAAGAGCTGAGGACAAACTATACGTTTGTC
AGATATGACAGCGATATTTATCTGAATCTGGCGGGTTCGCGTGTTACTGCATACTTCAACGGCAATTACCGGCACAATGAGCCGGGTTCACCAGACCATATCCAAAAAATAACCCCCTATGAAT
ACACCTTACTGGCAGATGACCCACTCGTTACCGAGTTCTACAGCTTCGATACGCTTTACAGGGAAATAAAAAGCGACGAAACAGACATCCGCCAGAGCGTCACCGCCGCACTGGATAAAGC
CAGAACGGTTAAGCGACTGCTGGAACAGTGGCCTGAAGCCAAAGAACTGTTACCTGCTGAAGATGCGGTTGTTCCCCTGCCTCCTGCTATTCGCCGGGAAGCGCTGAACGAGATGATTGG
GCTACCTTCTGAAGCAATTCAGGAGTAG
>PNNMHK_03735 Bacteriophage protein
ATGCGAATAATAACCAGGAAGAAACCTGCGTTCACTGACCTGTACCAGACTGGTGTTCTGACGCGTATAGCAGCCGTTAAGACTGACAGTGGCGGCTGGCGCCTGTTTGGAGTGTGGCGTG
ATCAGGATATCGCTGTATTTGTGGAAGCGGCGCGCGGCGGCATCCGGGAATGGTCCGGTTTAAATTATCTGGCTGATTTTGTGTTCAGTTGCGGCATTAGTCTCTGGGAGGTTCACAACAAG
ACGGATCGGAAAACTCCGGCATGA
>PNNMHK_03740 DNA damage-inducible protein I
ATGAGAATTGAACTTGTTATCAGCCGGACAAAAACAGCTTCCGGAAGGTGCCGTTCCTGCACTTGAAAAAGAATTAATTACCCGTCTCCAGAATCAGTATGAAAACTGCAACTTAACCATCCG
TCGAGGCAGTCAGGATGGTCTGAGTATCGTCGGTGCTGCTGATGGCGATAAAAAACGTATACAGAGCATTCTGCAGGAAACGTGGGAAAGCGCTGACGACTGGTTTTATTAA
>PNNMHK_03745 DUF1367 domain-containing protein
ATGGCGCACGAATTACAACTCATCAAGCAGTCATCTGGAATTCTGATCCCCGCAACGCCGGAGACCAGTGATATTCTGCAATCAAAAATCAAACTCGGCGCCGTGCTGGTGGCTGAGTTCCG
TCAGGTGAGGAATCCTGCATTCCATCGCCGCTTTTTCGCGTTGCTTAATCTTGGGTTTGAATACTGGGAACCCACCGGCGGCGCCATTTCTGCCAATGAGCGCAAACTGGTAAACGGTTATG

CAAAGTTTCTCGCTGCATATGGCGGGAATGAAAGCGCATTACTGGATGCGGCTGAACAGTATCTGGAACAGATTGCAAACCGCCGGGTAACAAACGGGATTAGCCTGTGTAAATCATTCGAT
GCCTACCGCGCATGGGTGACGGTTGAGGCTGGTCACTATGACGCCATCCAGCTACCGGACGGCACCCTTCGCAAACATCCCCGCAGCATCGCTTTTTCCAGTATGAATGAGGTCGAATTTCA
GCAGTTGTATAAATCTGCGCTTGATGTTCTCTGGCGCTGGATTTTATCACGTACATTCCGTACTCAGCGCGAGGCCGAGAACGCCGCCGCCCAGCTCATGAGCTTTGCGGGGTGA
>PNNMHK_03750 Phage protein
ATGGCGATGAAATACTCCTGGTTCCATCATCATGACTGCACAACCGAGCAGGCCGACACGCTGATATCGGATTATCAGAAGCGGGGCGTAAGGACAGAAAAGAGCCTGAACCCTGACTTCA
TTACCTGGACTGTCAGCGCGAAATTACCTGAATATGCACACCGGGTGCGGACGCCAAAATCCTTACGCCAAAAGGTCTGGGGGTGA
>PNNMHK_03755 Protein NinG
ATGGCTAAATTACCGCGCCGTAAGTGCGCAAACAAAGAATGCCGCCAGTGGTTTCACCCGATACGCGAGGGGCAGATCGTTTGCTCGTACCAGTGCGCCAGCGCCGTCGGCAAAGAACAA
ACCAGAAAAGCTCGCGAAGCCGCGCAACGTAAGGCGCAATCCCTTCAGCGCGCCGCTGAGAAAAAAGAACGCGCCACCTGGCGCCAGCGGAAAGCCGCGGTTAAGCCGCTGAAGCACT
GGATTGACTTGACGCAGCGCGCCGTAAATGACATTTGCCGCGAAACCGAACTGGCAGAAGGACTCGGTTGCATCTCCTGTGGAACGAAGACGGCGTTCGCATGGCATGCAGGCCATTACA
GGACTACGGCCGCCGCGGGGCATCTGCGCTTCACTCGCTTCAACATCCATCTTCAGTGTGATGTCTGCAACGTCTACAAATCAGGGAACATCGAAGCATATCGTACCGCGCTGGTTGAGCGT
TACGGTGAGGCGGCGGTGCTGGCACTCGAGAACAATAACACCCCGCACCGCTGGACGGTCGAGGAGCTGAAGGAAATCAGGCTCGCGGCTCTGGCGGATCTGCGTGCGCTAAAAAAGC
TGGAGGCCGCATGA
>PNNMHK_03760 YlcG family protein
ATGAAACCAGAACTGATCGAGATACTCCGCATGCGCTGGCAGCGCCTCCGCATTTACCGCCGTCCGGGGTCGGTGTTGGTTGACTACCGCATCCTGCGCAATTTTGTTCGTATTTATCAGTTC
ACAGGATTTACTCAATGA
>PNNMHK_03765 Antiterminator
ATGAACACTCAATACCTCCAGTATGTACGTGAGCAGCTAATGGTAGCGACAGCCGATTTAAGCGGGGAGACTAAAGGGCAGCTTTTGGCCTGGCTGGAGAACGCGCAATTCGACACGAAA
AACTATCCCCGAAAAAAACAGCGTATCTGGAACGAGGAAACAGAAAGCTGGATAACGTTAAATAACCCGCCAATCCCCGGCAAGCAGTCGCTGGCGAAAGGAAGCGCTATCCCGCTGGTG
AAGCCTGTGGAATATTCCACTGCCTCATGGCGCCGGGCGGTTCTTTCACTCGATGAACACTACAAGGCGTGGTTGTTGTGGAATTACAGTGAGAATACCTGCTGGGAACACCAGGTCGAGA
TAACACAGTGGGCTTGGGAGCAATTCAGCCAGCAACTGGAGGGTAAGCGGGTAGCTAAAAAGACTATTGACCGCCTGCGCCAGCTTATCTGGCTTGCAGCGCAGGATGTTAAAGCTGATC
TGGCTGGTAAAGATACATACGAATTCCAGGCACTGGCAGAACTTGCAGGCGTGGCTAAATCAACTTGGACGGAGATATACCTACCACACTGGCTTGTAATGCGTAGCTGTTTTATTAAGCTCG
ACAGTAGTGCATTGATTGCGGTAACACGATCACGTTCACAACAAAAGGCGACAAATTATGTACAAAGTCTTGCAAAACCGAACTGA
>PNNMHK_03770 isrJ Hfq binding RNA
TGTAAATCTGATATCGTCGCCATAGTTTCAATCGTCGACCAAACAAATTCAAGCCTCGCCATCGTGCGGGGTTTT
>PNNMHK_03775 tRNA-Arg(tct)
CCGCCATTAGCTCAACTGGAAAGAGCACGGAGCTTCTACCTCTGTGGTTCGGGGTTCGAATCCTCGATGGCGGACCA
>PNNMHK_03780 Hydrolase
ATGAAAGAAGGCTTCTACTGGATACAGCACAACGGCAGGGTTCAGGTTGCTTACTACACCCACGGCGTAACCGAGGACCTGGAAACTGGTCAGACTATTATTGGTGTCTGGCATCTGACGC
AGGGCGATGACATTTGTCACAACGGAGAGGCTGAGATTCTGGCGGGACCGTTAGAACCTCCAATTTAA
>PNNMHK_03785 isrI Hfq binding RNA
ATTATTGGTGTCTGGCATCTGACGCAGGGCGATGACATTTGTCACAACGGAGAGGCTGAGATTCTGGCGGGACCGTTAGAACCTCCAATTTAATACAT
>PNNMHK_03790 Phage holin family protein
ATGTCCGAACCTGTATCCAGTGCGACAGTGTTGGCTGGTGGATTAATGGGGGCCAGTGTATTCGGTCTGGCAACCGGAACCGATTATGGTGTGGTATTCGGTGCTTTTGCCGGCGCGGTGTT
TTATGTCGCCACGGCAACCAACATCGGACGCATCAGGCTGGTCGCTTATTTTATTACATCATTTATTGTGGGAGTGCTTGGCGCCGGGCTGATAGGTACTAAGCTTGCGGCAATAACGCATTAT
GAAAAGCCACTGGATGCTCTTGGCGCAGTGATTATTTCTGCAATGTGTATAAAGTTTCTCACTTTTCTTAACAGTCAGGATCTGAACAGCCTGTTCAGTATTCTTTCTCGTATCAGGGGAGGG
GGAGCAAATGGTAGCAAATGA
>PNNMHK_03795 Phage holin family protein
ATGGTAGCAAATGACCCTTCTGCAGTTCTGAATGCCGTAATTTGTGGGGTAATAGTCATCGTTCTGATGTTTTACCGACGCGGTGATGCGACACACCGCCCCCTGATTTCGTTACTGGCCTATG
TCATGGTGCTGGTGTATGCCAGCGTCCCTTTCCGGTTTGTTTTCGGTTTATATGAATCATCCCACTGGCTGGTGGTGATGGTGAATATCCTTATCTGCGCCGCTGTGCTGTGGGCTCGCGGTAA
TGTGGCGCGTCTGGTCGATGCACTGAGGCACTGA
>PNNMHK_03800 Endolysin
ATGGATCAACAACAATTTCAGCAGGCGGCTGGTATTAGCGCCGGGCTTTCTGCGCGCTGGTATCCGCATATTACGGCGGCAATGAGCGAATTCGGTATTACTGCTCCACTGGATCAGGCCAT
GTTCATTGCTCAGGCGGGACATGAAAGCGCTGGTTTTACAAGGCTGGTGGAGAGCTTCAACTACAGTATCGCCGGGCTGACCGGATTCATCCGCGCCGGGAGAATCACTCCAGATCAGGC
CAGTACTCTTGGACGAAAAGTCTGTGAGAAGGCGCTTCCGCTCGAGCGACAGCGTGCAATAGCTAATCTGGTATACAGCAAGCGAATGGGTAACAACGGACCTGGCGACGGCTGGAACTA
CCGCGGGCGTGGACTTATCCAGATCACAGGTCTGAACAACTACCGTGATTGCGGTAACGGGATCAAAACTGAGCTCGTTGCCCATCCGGATCTACTGGCACAGGATACGTATGCTGCCCGTA
GTGCAGCGTGGTTTTTTGTGATTAAAGGGTGCCTGAAATACTCCGGCGATGTGGAGCGAATCACGCTCATTATCAACGGCGGCCTGAACGGTATCGACGATCGCCGGGCGCGCTACAACAA
GGCGCGGGCGGCGCTACTGTTATGA
>PNNMHK_03805 Rz1 lytic protein
ATGCTAAAAGCCAGAACGCACTGGAGTCACAGAGAGCCCAGGCAGATATCAAAGTGGCTGTTGCGGATGATGATTGCGCTCGTCGGCCTGTGCCTGCTGCAGCTGCTGACAGGCTGCGGG
AATACGCGGACAGTTTACGTGCCGGTTCCGCCGGTGCCGCTGCCGACCAGCCTGACCGCTGAGACGCCACAGCCGACAATTCCTGACCCGCTGACCTACGGTGCCAGTCTGGACCTGAAC
GTGAACCTGCTATCGGCGCTGGGGCAGTGCAATATCGACAAGGCCAGTATCAGGGCAATTGAGCAAGAACGGAAATGA
>PNNMHK_03810 Conjugal transfer protein
ATGTTAGATGGATATTTTAATCATGACGGCGATAGTGGTGACGAGTTCATAAACAAGCGCAAGCGTTTGTTGGCGGTACAGGCTGCACTCGAAATCGCAAAACAAAGTGTTGGAAATTATCA
TCAATCTACGGTCTCTCGAGTAAACGAAGATTTAGCAAAAACCGCTGATGGAATCGAAAGGTTAGCAGATGCGATCCAGGCAGCATTAGATAAATAA
>PNNMHK_03815 Bacteriophage protein
ATGGCAAAACCGGACTGGGGGGCCATCGAAGCGGCATACCTGGCCGGGGTTATGAGCCTCCGCGAAATAGGCGCCCAACACGGCGTAACTGAAGGGGCTATCAGAAAGAAAGCCAAAA
AGCTGGGATGGGTACGCAAAGGCGGTACGCAGGTACGCAAAAGTGGTACGCAAAAGAAAAGTACGCGTACCTCAGCAAAGCCAACGTACTCAGGAGCAGCGCAACAATGTACGCAGCC
AGGTGATGAACAGCCCACCGATACGAAACCGATTCGTGGCTCGCGTACCGCACCACCAGTAAACCCATTCCCGTCCGGTAATCAGCATGCACTTAAGCACGGTGGCTATGCCCGCCGCCTTC
TGCTGAAAGATGACGTAATCGAAGATGCCAGGGCGCTGACACTGGAGGATGAACTGTTTCGCCTCCGGGCAAGCAACCTAGTAGCGGCGGAGAATATCGGCCGCTGGATAACCATGCTCG
AAGATATTGGCATGGAGGAAGAGCGGACCCGAAAGGTGCTGATGGAAAACATCAGGGCTGCTGAGAACGCCATGATGCGAAACACCGTACGAATTGAATCGATCACCGGAACGCTGGCG
ACAGTTAATAAAATTTTCGCCGATACCGATTACCGTGTGGCGGCGACCGATAAAGTATCTCTGGAGGCTGATCGCTTACGCCGTGATGCTGGTATTGATGATGGCAACGGAGAACGTGACCT
CAATGACTTCTACTCTGACATCCAGGCCGACACTTAA
>PNNMHK_03820 Terminase-3 domain-containing protein
ATGACTTCTACTCTGACATCCAGGCCGACACTTAATCCAGTGCTTCGGAACTTCTGGACCACGCAGGCGCGAAATAAGGTGCTCTATGGCGGGCGGTCATCTTCTAAATCATGGGACGCTGC
CGGGTTCGCTATTTTCCTGGCTAACAAGTACACCCTGCGTTTCTGTTGCGCTCGCCAGATCCAGAACAAGATTGAGGAGTCGGTTTATACGCTGCTCAAAATCCAGATCGACAGATTTGGTTT
GCGGCATCGCTTCCGCATTCTGAACAACAAAATCATTAACCGGGTGACCGGTTCTGAATTCGTTTTTTACGGCCTCTGGCGAAATATCGAAGAGATAAAATCGCTGGAGGGTGTAAGCGTTC
TCTGGCTGGAGGAGGCTCATGCGCTAACGGAATACCAGTGGAAGATACTGGAGCCGACGATCCGTAAAGAGGGTTCAGAGTGTTGGTTTATTTTTAACCCCGGACTGGTCACTGATTTCGT
GTGGCGTAACTTTGTGGTCGATCCGCCGGAAGATACGCTGATTCGCAAAATCAACTACGACGAGAATCCATTTCTTTCAGACACCATGCTGAAGGTAATCGACGCCGCGCGCCGCCGTGACC
CGGACGGATTTAAGCACGTCTATGAAGGTGTACCAGAGTCAGATGACGACGCCGCCATTATTAAGCTGTCATGGATAGAGGCTGCGGTTGATGCGCACAAGGCTTTGAGTTTTGAGCCCAG

CGGGCGTAAGCGTATTGGCTTTGACGTTGCTGACAGTGGCACCGATAAATGCGCGAACATCTACCGGCACGGCTCTGTTGTCTACTGGGCTGATGAATGGAAGGCCAAAGAGGACGAATT
GCTGAAGAGTTGCCAGCGCACTTACCAGGCCGCAATGGAGCGCGATGCGGATATCGTTTACGACTCGATTGGGGTCGGCGCATCGGCGGGCGCAAAGTTCTCGGAAATTAACGATGACAG
GGAACGGGAAAACGTTTACTCCCGAAGAGTAAATTATCAACGTTTTAATGCCGGCGCAGGCGTCTATGAACCGGACGAAGAATATAACGGCATTCCGAATAAAGATTTTTTTGCCAACCTGA
AAGCGCAGGCATGGTGGCTGGTGGCCGATCGCTTCCGCAACACGTTCAATGCAGTTAATAACGGTGAGCAGTATCCGGTTGATGAACTGATAAGCATCGATTCCTCCTGTCCATTGCTGGAG
AAGCTCAAACTGGAGTTGACCACGCCGCACCGCGACTTTGACCGTAATGGCCGCGTGATGGTGGAAAGCAAAAAGGAGCTGGCGAAACGCGACGTTCCATCACCGAACGTGGCTGATGC
CTTCATCATGGCATTCGCGCCAACAGACACATCGCTGGACATCTGGAACAAGCTTGGGAGTCAGTAA
>PNNMHK_03825 DUF1073 domain-containing protein
ATGTCACGTAAACAAAAGGTCGCTACGGCTGACTCTTACGATAACTTTGTCGCGCGGGTTGGCCTGCAGCAACCAAACCAACACGCGGCGTCGACATATCGGGCCAATTACACCAGCCGAA
ACCGCCTGCTTATAGAGTGGGCGTATCGTTCGTCCTGGATCATCGGCGCAGCTGTTGATGCAAAGCCCGACGATATGACAAAAAAAGGGGCCAGAATCACCAGTGAAATTGACCCGAAGCG
TCGGGGAATTCTGGAAGCCAGATTTGAAGAACTGAAGCTCTGGGAGCGGCTGAACCTGATACTGAAATGGTCCAGGCTTTATGGCGGCGCTGTTGGGCTAATCCTCATTGAAGGGCAAGC
TCCGCTAACGCCACTCGTACTGGATAAAGTGGGTAAAGGCAGCTTTAAAGGTCTCGCCGTGCTTGACCGCTGGATGATTAATCCGAACCTCGGACGGCGTATCAGGACTCTGGGGCCTGAA
CTGGGGCGGCCTGAGACTTACGATATTGTCACCACGGCGCAGGGTATTCCACCGTGGACGGTGAACTACAGCCGGTTGATACGCATGGATGGCATTATTCTCCCGTATCAGCAGGCCCTCAC
AGAAAACGAATGGGGGATGTCGATCGTTGAGCGGATCTTTGACCGCCTGACTTCCTACGACAGTACGAGCGTTGGCGCGGCACAGCTGGCTTATAAAGCACATTTGCGTACAGTAAAAATT
AAGAAGCTGCGCGAAATCATCGCTATGGGTGGAAAGCCTTACGAAGCACTAATTAAACAAATGGATATGGTACGTCAGTTCCAGACCAACGAGGGGATGTCTCTCTTTGATGCTGAAGATAC
CTTCGAGACGCACTCTTACTCGTTCGCCGGTTTGTCAGACCTGCTGAGCGAGTTCAAAGAAGACATTGCCGGCGCCGTCGGTATTCCGCTGGTTCGCCTGTTTCGACAGTCGCCAAAGGGT
TTTTCAACGGGAGATTCCGATCTTGCTAACTACTACGACGATATTGGCGCGCTACAGGAAAACACCCTGAGGGCACCGGTGCGCCTGCTTTATGAGGTGCTTCATCGCTCGGAGTTCGGCGA
GCCGTTACCTGATGATTTTACTTTTGAGTTTAATCCTCTTTGGCAGATGTCAGACGTTGATCGCTCGACTGTGGCAACCAATACGACAACCGCACTGGCGACCGCTGTGCGTGAGCTGGGTAT
GCCTGCGCCCGCTGCACTAACTGACCTCAGGGAATCGGCGCGTGTGACAGGGATCGGCGCATCCATCACTGATGAGGATATTGAACATGCGAAGGCCCAGTGGTCGGAGGATGAATCTGA
AACCAGCCCTCCGCCGCCGTTCGGAAATACAGTACCGCAAAAGCCTGCTGGCGATAGCAAACCAGATCGGGGAAATCGTGGCGGGCTCTTACGATGGTTCACAGGCCAGCACCGATAA
>PNNMHK_03830 Phage-Mu-F domain-containing protein
GTGGCGGGCTCTTACGATGGTTCACAGGCCAGCACCGATAAAACCGTCAGTACCCTTGTTGACTATTCAGCGCTGTTAGATAACTGGGCCGAGATGGTAGGCCGCAAGATGTTCGCTCAGG
TTGAGCAGGAAGAATGGAATCAGTGGAAATCTGTATCTGAGGAAATCGGCGCAGGTCTGCGTGATGTGGTGGGTAATACTCCCGTCGGGCTGGTGGCACAGGATATTGTGTCCCGTCAAAT
CCAGTTGATGAAATCCCTGCCTCTGGAAGCTGCGCAACGGGTTACGGAAATCCAGCAGCGCGCAATGCAGGCGGTGATCAACGGAGAGCGGCCAGACCAGCTTTACGGCATGATCATGCA
ATCCGGAGATGTGGCCGCTGGCCGTGCTCGTACTATTGCACGAACTGAGATCGGCCGCGCCACTACCGCTTTGACACAGGCCCGCGCGCTGGCTGTTGGCTCGGAGGGCTACTGGTGGCG
TATTAAGGGCGCTGGCACCCGTGACTCACATTACAAAATGCGTAATAAGTTTGTACGCTGGGATAGCCCACCAACGCTCGATGGCATGACAGGGCATGCCGGATGCCTGCCTAACTGCCAGT
GCTGGCCTGAGGTTGATATTCCCGCACCAAGAAAATAA
>PNNMHK_03835 DUF2213 domain-containing protein
ATGAAATATTTCTTTAAAACCCGCCTAGGGAATACTCGTTTCCAGTTGGCCGATGGCTCGATCCTGTTCAAGGACGTCCCTATCGGACGAACCGGTGAACAGATTTACGGTGCGGAAGAGCT
ACCGGAGCTTACCCCTGACAGTGACGGGTTGATAGTTGTCCGCCGGACGCCTGAGGAAGTCTTCAGCGAACGCACGATCGCGTCATTCGAGGGGATGGCTGTAACCATTGGTCATCCTAAG
GACTTTGGCGGCAACATCATTTTTGTCACGCCAGAGAACTGGCGTCGGCTGGCAAATGGCCACATCCAGAACGTCAGGCGCGGAGAGGGTGCCAGTTCCGATCTGCTGCTGGCTGATGTC
ATTGTCAAAACACCGGAAGCCATTCAGGCGGTTGAAGACGGAGACGATGAGGTCAGTTGTGGTTATGACGCTGACTACCGACAAATCTCGCCGGGTGTCGCAGAGCAATATGCGATCACC
GGTAATCATCTGGCCCTAGTCCCTAACGGGCGGGCCGGTTCACGTTGTGCACTGGGAGACGCTATGCCGAGCACTACTAAAAACTGGTTTACCCGGCTTTTAAAGGCCCGTAAAACCAACG
ACGCCGCCGAAATGGCGAATCTTATCGACAATCCACCGGAGAATATGACCGGTGATAACGATGATGTTACAACCGCCTTAACCCCGGGAGGCGTGGTCATAAACCTTTCACCGCAGAATCCG
ATGCCAGCGCCGACGTTGCCTGTGACGACTGACTCGGAGGAAGAAATCCCCGCATGGGGCAAAGCGCTGATTGAGGCCGTGGCGAAACTCACACCGACTGAACCGGTCACGGGTGATGA
GGATGAAGACGAGAAGGGCGAAGAAGAAGGAGGCGTGACTGGTGATGCCGCGTATCGCGCTGATCTGATTCAGCCAGGTATTCAGCTGCCTGAGAAGGTGAAGCCGACGGCGTTTAAA
CGCCAGATCCTTGCCGCTGCCGATCAGACGCTGGTTCGGTCCATCATCGGTGATGCAGACCTGGCGAAGCTGAAAAAAGCCACAGTTGACATGGCGTTTAACGCTGTTTCTGAACTGGCAA
AAAACCGCAACACCGCAGCGAAAACTACCGACGGTTTTCGCTCCATGACCACCAACACCACCAAATCCATCGCGGACATTAACAAAGCCGCTAAAGAACTCTGGAAACGAGGCTAA
>PNNMHK_03840 Bacteriophage protein
ATGGCTAATACCATTCTTTTCCGGATGCCTTCTGGCATCGCCGGGGCAATTTCACGCCCGCAGAATTTAACCGTTGAGCCACATGTGCTCGATTCCACGAAACCGTTCGCAGCGTATGGTCTG
GGCGGGAAAATCTCTGGGGGTAAATTTGTCCCGGTTGAAGCTGGCGACACGGCGGACGTAATAGCGGGGATTTTTGTTCGGCCTTACCCGACAGCATCACAACCCGACAAGATTCGTCAG
ATCAGCTCTGGTTTCAACTTTACCGGCGATAACCTCAAACGCGGTTATGTCACGGTCAATATTGGTGGTAACGCGTCAGCGGTTGCTCTCAATGCTCCGGTCTACATGCGCGTCGATAACCCA
ACGGATTCCAGCCCCCTCGGCGCATTCCTTGCTGCTGCTGACGGTGATAACACCGTTCAGATAACCAATGCATATTTCAATGGTCCCGGCGATGCCGACGGGAATATTGAGCTGGCGTACAA
CATTTAA
>PNNMHK_03845 DUF2184 domain-containing protein
ATGCCAATGACTTTTGATCAGGCAACAGTCGACAGTACTGGCGCCTTCCTTGTTCATGAGCTGGAGCGACTTGACCAGACGCTGAACCTTCCGTTGACATCACAGACGTGGAGCCGGGATA
TCGAACTACGCGAAGATGTTTCAATTGCTGATGAGGTGAGTTCTTTCACCAACACAACCTTTGCGGCGCCCGGTTCACCGAATGCGAATGGAAAGAACTGGATTAACCAGCTTGCGACAGC
AATCGCCGGCCTGAATGTGGATATCACGAAAAAAGGATTTCCACTGGAGTTGTGGGGGATGGAGCTTGGCTGGACGGTAGTCGAGCTGGCTGCTGCTCAGCAGGTGGGCCGTCCTATCGA
CACCCAGAAATACGATGGCATGCAGTTGAAGTGGAATATGGATACTGACGAGCAGGTGTATATCGGTGATGAGGTAAAAGGTGCTAAAGGCCTGCTTAATCTGTCACAGGTCACCCCGACG
AATGCTGCTAAAACTTGGGCGACCTCCAGCCCGGATGAAATCCGTGCCAGCATTAACCAGGTACTGAGCAATGCCTGGGTTCGTTCCGCTTATTCGAAAGTACCGGAAGATTTGCTGATCCC
GCCGGAACAGTATTCCTTCCTGGCGAGTACGATCGTTTCTTCCGCGGGTAACCAGTCACTGCTTACCTACCTTGAAACCAACACGATTGCTTTCCACCAGAACGGTAAACCGCTGGGTATTCG
TCCTGTTAAATGGGCTATTGGGCGCGGTGTGGCGGGTAAAGACCGCATGGTCGCCTACACGAACGATAAAAAGTTTGTGCGCTTCCCGATGGTTCCCCTCCAGAGTGTGCCGATCCAGTATC
GCGGCATCTACCAGCTTGTAACCTACTACGGCAAGCTGGGTGCTGTTGAACCAGTCTATCCGGAAACCCTTAACTACATGGACGGTATTTAA
>PNNMHK_03850 hypothetical protein
ATGAAAAAAATCTATGTGCTGTCGGCGTTTAACTTTAACGACGGTGCCAGCATCAAGACATTTACTCCCGGCTTTCATGACGTCGAAAGCGATGTGGCTGATCACTGGTTTGTGAAGGCGCA
CTGTTCACCGGACGGCGAGGCTCCGTCCCCGGAAAATGATCCGCGTATTGCAGAGCTGGAAGCGCAGGTGGCGGAACAATCCACCCGTATTGCAGAGCTGGAAGCGCAACTTGCGGAGG
CTAAAGCCAGTGGCAAAAAACAAAAGCCTGCCGACGCCTGA
>PNNMHK_03855 DUF4054 domain-containing protein
GTGGCAAAAAACAAAAGCCTGCCGACGCCTGAGAAATTCCGCGCTGATTTCCCACAGTTTGCTGATGAAACAAAATACCCCACAACGATGATTCAGGCCCGTCTGGTCCTTGCTGATGTGC
TTATGAACGAGTCGCGATTCGGCGAGGACGTTTTCCCCTATGTCGTAGGGTTGTTCGTGGCGCACTACATGGCGCTTTACGCCGCTGATATGCGCGGCGTGGCGGTTGGTGGCGCTGGAGG
AGCTAATAGCGGGGTACAGACATCAAAATCGGTTGATAAGGTGTCTGTAAGCTACGACGCGAGTACCACTCTGAATCCTGACGCTGGTTTCTGGAATAACACCCGTTACGGTTCCGAGTTCT
GGGAATACCTGATGATTTTCGGCGCCGGGGCTATCCAGTTGGGGACTCCCTGA
>PNNMHK_03860 Bacteriophage protein
ATGAAAAGCGGGTTAACGGTTCGCGCTGATAATGCTGTTGCCATCCTGGAATCCCTCCGGCAGCTATCCGGAATGGATGTGCTGGTGGGAATACCAGAGGACAAGGCGGGGCGTGAGGAT
GGCTCACCGTTTAATAACGCTGAGCTGGGCTATCTCCATTCAACGGGCGCAACGGTGGAAATCGACGGCACGACAGTCACGCTTCCCCCTCGTCCTTTTCTTGATATGGGGATCGAGGATTC
AAAACGCCGAACTACTGCGCACCTGAAGGCTGCGGCTACCGCCGCACTTGAAGGGCAGTCTGAAACGGCACTGCGTGAGCTGGAGAGTGCAGGACAGATTGCCCGTGACGCTGCAAAA
GCCGTTATTGGTGCTGGCGATCGCCTTCACCCTCTTACAGAGAAAACCCTCGAACGCAGACGGGCTGAGGGCATTGCTGGTGATAAGCCGCTGTATGCCCACGGTTACCTGCTGCGCTCAAT
TAACTACGTCGTGAGGAAAATATAA
>PNNMHK_03865 Head-tail adaptor
ATGCCTTTTCTCGATGTGACGGACGTCCTTCTCGATCCGGATTTTTGCGACCCGTCACTGGTGTGCCTGCGGCAGGTACAAAGCCTGGATGAGGATAACTTTGCAACGAACACTACGCAGGA
AATACCTTTCGCTGGCGTCGTAACGGTGGATCGTTCGCTGGAGGCACAGAGGATGATTGCCGGGCAAAACATCGCTGGCGCAATTCTCATCGTGACACAATTCCGGTTAACCCGGGGTAAC

CGCGAAGGCCTGGACGCCGACATCGTGCCTTATGAAGGTTATAACTATCGCGTAACAAAAGTTGACCCGTACACACGTTATGGCGCCGGGTTTGTTCAGGCTCACTGCGAACTGCTTGACG
AGGTGTAA
>PNNMHK_03870 Bacteriophage protein
ATGAGCAACGACAGCACCGCGCGCGGTTATCTGACTTCCGTCGGTGACAGTCCCCAATATGATGAGGCGCTGGAGCGTGAAATCAGCCGGTGGATTCGCGGTGTTTCCGGGTTGCCGGCA
GAACTGGTTTTTCCACGCTGGACCGACCCGCAGCCGCAGATCCCCAAAAACGGAACGACGTGGTGTGGTTTCGGTATCACCACCGTTCCGCAGCCGCTGAGTCAGTCTGATGTTCAGGTAT
CGGAAGAACAATCCGAACAATGGACATGGGAACAGGTCACCGTCATTTGCTGCTTTTATGGCCCTCTGGGGGCAGGTGCCGCATCTGCTTTCCGGGCGGGAATTTTCGTCGAGCAGAATAA
TGCCGAACTGAACCGTTCGGGGCTTTCGCTGGTGGAGGCCGGGACTATCTACAACCTGCCAGAACTCATTAACAACCAGTGGGTGAGGCGCTATGACCTCACCATCACACTGTCCCGCAAA
AACATTCGTACCTACAACATCCGGACGCTGAAAGATGCGCCCGTCTCATTTCTCGGAGACTAA
>PNNMHK_03875 DUF3383 domain-containing protein
ATGCCGCAGGGATTACCTGTATCAAACGTCGTTAACGTCGACGTGATCATTGGGCCGCGCGCGGCTACTGGTCGAAACTTTGGTTCGCTGCTCATTCTCGGGAGCTCAACGGTTATCCCTGT
TTCTGAGCGCATTCGTCTCTACTCAGCGCCTGAGGCTATCGGTGATGATTTCGGCGTGGATAGCCCGGAATATGAAGCCGCTACGGTGTATTTCTCGCAGTCACCGAAGCCTCAGCAGGTGT
ATGTGGGCCGCTGGGTGAAAACACTGGAAGCCGCTGAAAGTGGTTCAACCGAAACGTTGCTGCAGGCCGTTAACGCCGTACTGAATTACACGAACTGGTATGGTCTGGCTGTGGCAGACG
ATGAAGACATCGATGATGCCGACTGGCTGAGCGTGGCCGCCGCGATCGAAGCTTCAAGCCTCAGTCGTATTCTGGCAATTACCACTCAAGACCCGGAAGCGATTAACACGACATCAACTACC
GATCTCGCCTATAAGCTGAAGGCGGCAAAATATGGTCGTACGTTTGTTCAGTATTCCACCAGCAGCAGATACGCGGCGCTGTCTGCGTTTGGTCGCGCGTTTACGGTGAATTTCAACGGCAG
CAACACCACCATTACCCTGAAATTTAAACAGGAGCCGGGGATCACGTATGAAACCCTGACCACCAATCAGGCGGCGGCGCTGGATGCCAAAAACTGTAACGTCTACGTGTATTACGAGAAC
GATACGGCAATTCTGCAGCAGGGCGTCATGTCCAGCGGCGATTTCTTTGATGAGCGCCACGGACTCGACTGGCTGCAGAACTACGTGCAGACCAATCTGTATAACCTGCTCTACACCAGCAC
GACCAAAGTTCCCCAGACCGATGCGGGGGTGACCCGCCTTCTGTCCAACGTGGAGAAATCAATGGATCAGTCTGTCACGAACGGGCTGGTGGCTGCCGGTGTCTGGAACGGTGGCCCGA
TTGGGCAACTGGATTCCGGTGACACGCTGACAAAAGGCTATTACGTCTACGCGCAGCCGCTTTCCGAGCAGGCACAGGCAGATCGTGAAGCACGTAAGGCGCCGGTTATTCAGGTGGCCT
GTAAGCTGGCGGGTGCGGTGCATTTCGCTGACGTACAGATCAACGTCGTTCGCTAA
>PNNMHK_03880 DUF3277 domain-containing protein
ATGGCTACTTATTCTTTTATGGATGTCACGGCGTCCCTCTCCGGCCCGACCGGAGAGATTGATCTGGGCTACGGCTCCGCCAGTTCAGAGGAAGGGATCACGGTTGCAATGGCCGGCCCTAA
AAACACCATGACTATTGGCGCTGACGGTGAAGTGATGCACAGCCTGCACGCGGACAAAAGCGGTACGGTAACCGTCAATCTTCTGAAGACCTCGCCGACAAACAAAAAGCTGTCGCTGGC
GTACAACGCGCAGAGTCAGTCTTCAGGCACCTGGGGGAACAACGTCATTGTGATCCGAAACAAGGTGAGCGGAGACATCATCACGGCGCGCAGCGTGGCGTTCCAGAAACAACCGGATA
ACGCCAACGCCAAAGCCGGTAATACCATGCCCTGGGTATTTGACTGCGGCAAAATCGACCAGATTCTCGGAGAGTTTTAA
>PNNMHK_03885 Bacteriophage protein
ATGGAATGCTCAATCAAAGGTCACGATTACCGCGTGGCAAAACTCAGCGTTTTTGATCAGCTGAAAGTGACCCGTAAACTGCTGCCGGTGCTGGCGGGCATGATGTCAGATTTCGGGAGCT
TTCGCTCCCTTCTGCCTGCAGATGGCAAAATCGATGGCGCAAAATTTGATGCGTTAAAGCCGGTATTTGAAACCCTGCTGCCGCGTATCGCCGATGAACTGTCTTCTCTGACCGAAGATGACA
CCAACGCGATTATTCATCCTTGCCTGGCTGTGGTGTCACGCAAACATATGGGTGGATGGACCCCGGTATTTAACAGCGGTCAGTTGGTGTTTGACGATATCGACCTGCTGACCATGCTGCAAC
TGGTGGCGCGGGTGGTCGCCGATTCGCTGGGAAATTTTTTGCAAGGACTCCCTACCAGCGGGACGCCCACCCAGCCAGCGGAATAA
>PNNMHK_03890 peptidoglycan lytic exotransglycosylase
ATGAACGCTGAGACTATTAAAGATTTTCTCGTCTCCCTTGGCTTTGATATTGATAAAGCCGGATACGAGAAATTTGAATCTGTTCTTGCTGGCGTCACCGCAAATACTATAAAAACAGGGCTGG
CGGTGGAAGGTGCGGCGCTGTCCGTTGTTGCGTTTACGGCGAAAATTGCCTCCGGTCTGGATAATCTCTACTGGGCATCTCAACGCACCGGCGCGACGGTTCAGGGGATTCAGTCGATTGG
CTACGCAGTTTCGCAGGTGGGCGGTAGTGTGGACGCGGCGCGGACTTCGCTGGAAAGTCTCTCCCGGTTTGTGCGTAATAATCCCGGCGCGGAAGGCTTTCTGAACCGCCTGGGCGTACA
GACCCGGGACGCCAGCGGGAATATGCGCGATATGGCCGCCATTTTTACGGGCGTCGGCCAGAAGCTCAGCAGCATGCCGTATTACCGGGCTAACCAGTATGCGCAGATGCTGGGCATTGAT
GAAAATACCCTTATGGCGATGCGCCGGGGTTTAGGGGGATTCTCCGGCCAGTACAGCGCGATGGCAAGGGCCATCGGTTTCAATGCTGACGAGGCGGCCAAAAGCTCCAACAGGTTCATG
ACCTCCCTGCGCGAGTTCGGCGCGATGGCAGGCTTGGCCCGTGACAAGATCGGCTCTAATCTTGCTGGTGGTCTGGCGGGTTCGCTGGACACGCTGCGCCGCCACATCCTGGATAACTTCC
CGCGCATCGAGCAGACCCTGACGAAAGCCATAAAAGGTATTCTGACGCTCGGGGATATCATCGGGCGCCTGTTCTTCAGGCTTATTGAGGGAACATCCAGCCTTATCACCTGGTGGCAATCA
CTGGATAAGCAAACGCGGGAACTGATCTCGCTGTTTGGCGCACTGACGATTGCGCTGCGCATTCTGAACAGCACGTTCTGGATGTCGCCGATTGGCCTCATTACCGCGCTGGCGGCGGGGA
TCGCCCTCCTGTGGGAAGACTATCAGACCTGGAAGGAAGGCGGCCAGAGCCTGATTGACTGGGAGAAATGGAAACCGGAGGTCGACGTCGCGCTGAAGATGGTTCGTGACCTGAAAGC
GACCGTTAACGACCTGGCGAAAGCACTGGCGAAACTGCTCAACATTGACCCCAAATCGTGGTCCCTGAAATGGGATTTCAGCAATTTCATCGACCAGATGGGGGAGTTCAGCAAAATGCTG
AACATGATCGCCGACCTGCTCAATGCCATTAAAGACGGTCGCTGGGCCGATGCCGCCAGCATCGGCAAACAGATGCTTAATCAGGGCAGCGAAAATTCGTCAGCGATGCCAATGGTAACAG
ACAGCGCCAACGGTGCCGCCGACTGGATTAAAGAGCACTGGGGATTCGATCCCCGCAGCGTAGGCCGAACGGTTCGCGGCTGGTTTGGGGGGGATGACCCTGAACAACACGGACAGTC
AGTTAAGCGGCCACAACCAACAAAAGCTGGCGCTGAGCTGCTGGGATGGATGCAGCCGATGCTAACCAATCTGGAACACCTATACCGGCTTCCGGAAGGTTTATTGCGCAGTGTGGCCATC
ACTGAATCAGGCGGGAATCAGTTTGCAGTTTCCGGCGCCGGCGCTAAAGGCCTGTTTCAGTTTATGGACGGCACTGCACGAGATATGGGGCTGCGCGGGAATGATGTTTTCGATCCGGAG
AAGGCTGCGCAGGCTGCAGCAAAATATCTTTCACAGTTGCTGCGGGCGAATGGCGGTGACCTGAGCAAGGCGCTGGCGTCATATAACTGGGGGATCGGGAATGTACAGAAACACGGGAT
GGCCCTTATGCCTCAGGAAACCCGCAACTACATTCCGAAGGTGTTAAGCAACATGCCCGCACCCGGAGCTCAGGTCCAGCAACAGAACACCTATCACATTTACGGTGGTGGTGATCCGCAC
TCCGTGGGGAATCAGGTAGAACGTCGGCAGCAGTCTGCAAATGCCCAGCTCATGCGCGGCAATCAAACGAAGGTGGGTTAA
>PNNMHK_03895 Bacteriophage protein
ATGGATATTCTCTCTACTCTCTTTCATCAGCAGTCCAGGAGAATTGGGGTGCTTATCCCCAGTGTGGTTGTTTCAGAGAAGCACACCGACACGCTAGAGATAACAGAGCACCCTGTCGAGGT
CGGTGCCGCCATCGCTGACCATGCCTACAAAAAACCGTCTGAAGTGGTGATGGAGGTCGGTTTCGCTGGTGGCGGATCGTTGCTGGATTTTGCCAGTAACCTGACGGCCACCAGCTTACTG
GGCCTGAGTCCTCAGCAGACGTATCAGGAGATACTCGACTTGCAGGAAAGCCGTATTCCCTTCGATGTGGTGACCGGCAAACGGCTGTACAGCAACATGCTGATCCGCGCACTGGAGGTG
ACGACCGACAGGACGACCGAAAATGTCTTGTCCGCCGTCCTCACCCTGAGGGAGGTTCTCATCTCGCAGACGCAGCAGGTAACCGTCGCGGATAAAACCGACATGAAGGACGGGGCCAG
CACGTCGCCAGTCCTCAATACCGGAACCAAAACAACTAAACCGCCAAACACTTCTTTATTGCAGAGTGGTGCGGCTTTTCTGGGGCTGGGTTAA
>PNNMHK_03900 Bacteriophage protein
ATGACTATTCAGGAAATTCCGCTGACCGCGGATAACCAGCAGTTCAGCATCATCCTGGCGGGTATCACGTGGCGGATTCGCATCATCTGGCGTGACCTGTACTGGATCATGGACCTGCAGAA
CGACAGGGGGGAGCCGGTAATCTCCGGCATTCCTTTGGTCACTGGCGCCGACCTGCTGGCACAGTACGCATACATGGGACTGGGTTTTAAGCTGGTGGTGATGTGTGACGACAGTACACA
GGATTATCCGACGAAAACCGACCTGGGCGGTCGCAGTCATTTACTGGTATTAACGGAGTAA
>PNNMHK_03905 Phage related protein
ATGTCACAGAACTGGATGAGGCATTTCGAGCTGCAGCTCGTGGGCGAAAACGGACAGGGTATTCAACTCAGCGATTTTAAAGTGACCTTTACGATCGACTGGTTCAACATCAGCAGCGCGT
CCCGGGTGGGAACGTTCAAAATCTACAACCTGTCAGCTGATACGGTGAACCGCATCACCGGCCAGGAGTTTTCGAAAGTGCGGCTGATTGCCGGTTACGACGGTATCGCGCCGGAGGTGG
CAGCCAGTGATGTCGGCATTGCGCGGGAAGTCGACGCCGACACGGTGGGCCAGAGCGACGGGCGCAACTACGGACTGATTTTTAGTGGGGAAATTCGCTACTCGGTCACAGGAAAAGA
CAGTCCCATTGACTCCTACGTCCTGATTCAGGCCGCCGATACGGATCTGGCATTTGCCACCAGCATTACCAATCAGACGCTGGCAGCGGGTTACACGACAGAAGATATGTTCAGGCTGTTGA
TGAAGGACTTCGAAGCCAAAGGCGCGACCGTTGGTCGCACTCCGGTATTCCCCCCGACTGTTTTCCCGCGGGGACGTGTGTTGTTTGGTATGACACGGCATCTTATGGATAACGTTGCTGCT
CAGTGCGGCACCACCTGGCAGTTCGTGGATGGTCAGCTTAATATGTTGCCCGAAGGTGAATACATGCACGACGCGATTGTGCTCAACAGCGCCACTGGGCTGATCGGCATGCCTCAGCAGA
CCATCGGTAATGGCGTTAACGTCCGCGCGCTGATTAACCCGAACATCCGGGTTAACGGGCTAATTCAACTGGATCAGGCTTCAGTGTACCGTACTGCATTGTCGAATAACGATATTGCTATGG
CTGGTGGCCGTATCACCGACCAGAACACTGACGGTAATATCACGCTAAGCGGCACCACGGCGCAGCCTGCCAGCATTGCAACGGATGGCGTTTATGTTGTGAAAGGCATTATGTATACTGGT
GACACAAGGGGCCAGCCGTGGTACATGGATATGATGTGCGAAGCGCGTGGGGCTCAAGATGTGAGAACTCAGGATTCGCTCAATCGGGGGTAA
>PNNMHK_03910 hypothetical protein
ATGGACATGATCAATGGCGAACGCGTCACCTCTCAAAAGATTACAAATTTGGGAGCGGACGGTATCAAAATTGATATGGGTCTGATGCCTGCCAAAGATGGCAACAACTATGGTTTTGAGTA
TATCCATCGCCCTGGTACCGAAACGCGTTTCCTGAACGTCCAACTGCTGCAGAACAGCATGGACGCGCCGAGGATCATTGGATCATTTCCGTGTAAGAAAGTGCCTGATTGA

>PNNMHK_03915 Cytoplasmic protein
ATGTCACTTGCACTGCATAGCAAACTGAATGAAACGCTTGAGATGCTTACTTCGCATATTGATGAAGGGCAACAGTTAGATGCTTTTACACTAAAGCGAGTGGTTTCTGGAGCAAGTAAAAT
CCCAGATGAGCCCGTGAAGCTTATGGTGTTGGCTCTGGCCTATGGAGCTGCGCATCAACACAGTGAGGCTGTCGGTTTTTTCCGGGAGGCAGTTGCATATCGTGACGAAGCAGTAGCCAG
GAATTATCTCTCTTATCTCAGTTATACCGGGCAGTATGAATTGTATCGTGAAGAGGCTGTGAGACTGGCTAGGGAAATTACTAGTCTCGCTTTGTGTGTACGAGCCAGGAATGCAGCCTATGC
GGACGGTGATGGTGAACTATCACTCTTTTTTGCTCGAAAAGCTCTGTCTATGATTGGAAGCGATAGCGAGCGAGAAAGCATGGAGTCAGATGTTATGGAAAAAAAACAGGCATTAGATGCA
TTCATAAGTGTGACTGATTTAAGTACAAATGAAATAAGTCTTCTTTCCAGAACAATTGCTAATGTCGCCAAAAATTATGGTGTTTTAGCAATTTCACATGACTACGTCGCAAGCCCTGAAGGGG
ATGCGGGCATAGTTTGTGACGTGTTATGCGAAGATGCTGATACGCTTTCCGATATGGATATTGACGTTGCTACTGAAATTGCAATGAATGAAGTCTTCGCTGGAAAAAATGTAACAGCTTGGT
TCCGCGGGCGTAATCGGCAGGAGATTCAATTCACAATATGA
>PNNMHK_03920 Gp138-N domain-containing protein
ATGGCAGTATCTGACCAGACCCGTAGCGGCGACCTTGCCGAAACATTCAAATCTGAGCGGGAAACAACAAAGAACCAAATCCGCGTCGCTTTGCCCGGCATTATTCAGTCATTCGACCCTG
ACGCGGTTACGGCGGTTGTGCAGCCTGCTATCCGTTCGGTTGAGACCGATAACGACGGCAACCGGGTGACAAAACCTTACCCGCTGCTGGTGGATGTGCCCGTGGTATTTCCTCGCGGCGG
CGGCTGCACGCTAACGTTCCCGGTGAAAGCCGGTGATGAATGCCTGGTGATTTTTTCCGATCGCTGCATCGATTTCTGGTGGCAGAACGGCGGGGTACAGGAGCCTGTCGACGATCGGGT
GCATGATTTATCGGATGCGTTCTGTATCGTCGGGCCGCAGTCGCAGGCGCAAAAAATAAGCGGAATAAGTACGGGGGCCGCGCAGCTCCGTAGCGACGACGGAAGTACGTTCTTTGAGCT
CAACCCTTCCACTCAGAAAATTAAAATCGTAGCGCCTGGCGGTCTGGATATAGTGACTCCGCTGGCCGACTTCTCGGCGAAAGTTACCATTCATGGGCTGCTGTCCTGGCTGGGCGGCATGG
TAGGGTCCGTTGTTTCTGGCGTTGCATCAAAAATCACCGGTGCTGTTGAGTTTATCGGTAGTGTGAAAGCGAACGGCAAGTTGATCGACAATACCCATACGCATGGCGGCGTTCAACGCGG
TGGAAGCAATACAGATGAGGTGAACTAA
>PNNMHK_03925 Bacteriophage protein
ATGCGGTACAGACGTGAAGACGGCGAAGGTGATTACACTTTTGGTAGTGGCGATGATACCTGGCTGATTAACTCGCCAGAAGCTGTCGCGCAGGCAGTAAAAACACGATTTGCATTGTGGT
ACGGGCAGTGGTTCCTCGATAAGACAGAGGGAACACCGTGGATTCAGTCTGTGCTCGGTAAGCAAAAGCCGGAAACCTACAATCTGGCGATCCGCAAGCGCATCCTCGAAACGCGGGGC
GTGAAATCCATCCTCTCTTTCAATACCACAGTGAACACGACGACGCGCCGCGTCCAGTTCTTCGCTGAAATCGACACTATCTACGGAACAACGACAGTAACCAGCGAGGCATAA
>PNNMHK_03930 Baseplate-J domain-containing protein
ATGGCCCTCAATTTGGACACACTCGGCTTATCGGCAACGGTAACCGCTGAGGGGATCAGTGCGCCTGATTACCAGACGATACTCGATACCCTGACGAGCTATTTCCAGCAGATTTATGGTAGT
GACGCTTATCTGGAGCCGGACAGCAAAGACGGCCAGATGGTGGCGCTGGTGGCGCTTGCTATTCACGATGCCAATAACACAGCCATTTCCGTCTATAACTGCTTTTCACCTGCTACGGGTTA
CGGCGCAGCGCTGACCAGTAACGTAAAAATTAACGGTATCGCGCGCAAAGGTGCAACGAACTCTACCGTGGATTTACTGCTCACTGGCACCGCAGGAACAACCATTACGAACGGCACCGT
GAAAGACACCAATAACGTGATCTGGCGTCTTCCGGATTCAGTGGTGATTGGTGTTGATGGCACCGTGACGGCAACTGCAATTTGTTCCAAAAGCGGAGCGGTTGCAGCTCCTGCCGGGAC
GATTACCACTATCAATACACCGACCCGTGGCTGGACGTCAGTAACCAACCCGGCAGCGGCCACCGTTGGCGCACCTGCAGAAACGGACGCAGAACTGCGCATCAGGCAGGGGCAGAGTG
TCGCGATACCATCCATCACACCATTTGAAGGCGTGGACGGGGCGATCGCTAATATTGCTGGTGTGACGCGCCACAAGCTCTATGAAAATGATACAGGAAAGACTGACGGTAACGGGCTTCCT
CCGCATTCCATCTCGGCCATTGTTGATGGTGGTGATGTGACCGAAATAGCCAGAACCATCCGGGGAAATAAAGGGCAGGGGGTCCGGACCTGGGGAAAAACATCCGTAACCGTACCGGAT
AAATATGGCAATCCTCACATAATCAGTTTTTCGCGACCAACTGATGTCCCTGTTTACGGAAAAATCACCTTAAAAGTTTTTGCCGGGTACACCTCTCAGATAGGTGTGCAGATTCAGCAGGCT
GTTGCGGATTACATTAACAGACTGATGATTGGTGACCAGGTACTGCTGAGCCGGATTTATTCTCCTGCTAACCTTGGGGTCGTCAGTGGTGGAAATGCGCGCTATTACGATATTCAGGAGTTG
CTGATCGGCAAATCTCCGGAAGCTGTTGCTGCGGCGAATATTAATATTGCTTACGACGAATCTGCCTCCTGTAAGCCGGAAAATATCATTATTACGGTGGAAGCATGA
>PNNMHK_03935 hypothetical protein
ATGAGCAAATATACGGACTTAATTACTAACTACCATGCGACAAAACCTAAATTCGTTGAACACATCGATTTAGTGACCAGGCCGTTAGCTGAAACCTCAGCCGCAATAAATGGGATAATAAAC
GCTTTTGATATTGATTATGCGACAGGAATACAACTCGATATTCTCGGCCAGTGGATAGGGTTAAGCCGGGTTGTAAGCCAGCCAATAAGCGGTGTCTATTTCAGCTGGGACACTGACGGACT
CGGATATGACCAGGGCGTCTGGCAGGGGCCATATGATCCGGATTCGGGTTATACCTCGCTGAGCGATGAAACCTATCGCATCGTTCTAAAAACGAAGATAGCAATTAACAACTGGGACGGA
AGAAACGACTCTCTGCCTCCCATTCTTGACGCTGCACTGGACGGGTCCGGTCTGAAGATGCAGATCGTCGATAACCAGGATATGACCATAGGTATCTGGGTTTTTCCTGAAACAGATATTTCA
TCGGTCTCTCTCGAACTTATTGCTGCGATACGACAAGGGTATCTGACGGTAAAGGCCGCTGGTGTATGGGGCGGAAGTATTGAAATTCCTTCGGTGGAAACGCCTTCTGAAGGAAACAGGT
TTTTTGGGTTTGATATGGATAACGAATATATCAGCGGGTTTGATGCCGGTTCATGGGGGACATTACTCTGA
>PNNMHK_03940 Phage tail protein
ATGGCTAAAAATGATTTTAAACCGTTTGCGACGGGTAAGGGTGCTAATGTTACATCGCAGCCTGACTGGGAGGCGCTGCCGGCGCTCCTGTCTGGTTTTACTACGGGCAAGGCATCAAGTG
CACAGGTAAATAAAGCGCTGCGTCAGGCGAGCTTCATCGCTGCAGCACTGGCACAGTACACAGCCAGCAAGAGCGGGCAGGATGTACTCGATGATGGTGACCTGAGCGGCTTTATCGCCA
AAATGTCCGCTGCGTTCGGTAAGGATTTTCAGACTCTTGATGCCACGCTGACGGCGCTCGCTGGTCTGGCTACTGGTGCAGATAAACTTCCGTATTTTACGGGGAATGATACAGCCGGACAG
ACAGATCTTACTTCTGTTGGGCGCGACATCATCGGAAAAGCCAGCATTGCGGATATTCTCACATACCTCGGTTTGGGAGAAACAGCAAAGCAAGCTGCTGGTGCCATGCAAAAATCGCAGA
ATGGCGGCGATATTCCGGACAAAAAACAATTTGCGAGAACTATCGGCGCTGTGACTTCTACCAGCGTTACATTTGGTGAATCGGGATGGTTCAAAATCGCCACGGTTGTAATGCCGCAGGCC
ACATCAACTGCGGTGATAAAACTGTACGGTGGGGCGGGGTTTAACGCTGGTTCACCTGAACAGGCGGCAATTAGCGAACTGGTATTGCGTGCCGGTAATGGTTCACCTGCTGGAATAACTG
CCACATTATGGAGACGTTCGCCTGCCGCGGCTAACGAGGTCGCATGGGTTAATACATCAGGCGACACCTACGATATTTATATTAATATCGGCCAGTATGCGTACTGGTTAATTGCGCAATATGAT
TACACCGGTAATGCAAATGTCACGCTGTACAGTACGCCTGAATATTCATCAGTACAGCCGGGAAACTCAACCAGCGGTCAGACATATACAATTTACAGTAGTCTGATGAAACCAACAGCCGGT
GATGTGGGTGCATTGCCGATTACAGGGGGACGGCTTAACGGCTCGCTGGGTATTGGTACTGATAATGCGCTGGGCGGCAATTCGATTGTATTCGGAGATAACGATACAGGAATTAAACAGA
ACGGTGACGGGATACTGGATACGTTTGCGAATAGCCAGCACACCGTTCGTGTCGCTCCCGGTGAAATGCAGATTCTGGGAGCCATTCGCGCAGGCGATGCCAAACGAATGACCATGACTAG
CTCAAATAACTCCGTGCTGAATGCTCAATTTCATTTGTGGGGTGACGGAAATCGACCAACGGTTATTGAGCTGGATGACGACCAGGGATGGCATTTATACAGCCAGCGTAATACCGATGGCA
GTATTCAGTTTGTTGTTAATGGACAAGTTATTCCGGATAATTACGGTAATTTCGACGCCCGTTATTTAACATCAGGAAACGTATATACAAAAGGCGAATCAGATAATCGTTATGTCCAGAATATC
CAGCGCGGTGCTCCTGTATGGCCTGGCAAAGTAGATGAATATGGACCAGCAGAAGCGCCTGCTGGTTGCTTTTTAACACAGGCCAGACATGACCCAACAACAGCATACGGTGTGACATTTG
CGTATAGACCGCTACAAATGTGGGTGGGTAATGGCTGGCGTACAATTAATGGATAA
>PNNMHK_03945 hypothetical protein
ATGGAATTAAAAAACGTTAACCGATACATTCCTGACGACCCGGATTATGATAGCAATTTTCTGTATTTTCGTAGTGAAGATGGTCAGGATTTTTATGAGTCGCTGAGTAAATTCACGAAAAAAT
ATAAGTTGTGCATTGATTCTGATAATATAATCCGTTCCGTATCAGAAGATGTGTCGCGACTCTATCCGGCTGGCTTTTCAGTTGTTGAGGTCAATAAACTACCAGCCGGATTTAATATCTATGGC
GACTGGAAATATTCGAACGGCACTGTTCTGGCTGTTCCCGTTGACTATCAGGCTAAGGCCGAAACCACCCGACAGAAACTACTGGATGCCGCTAACAGCACCATTGCTGACTGGCGAACCG
AACTGGCGTTGGGTGAAATCAATGATGACGATAAGGCCAGCCTGACTAAATGGATGGCGTATATCAGGGCGCTTAAAACGCTGGATTTGAGCGGCGTGAAAGACGCGGCCACCTTCACAG
CAATCAGGTGGCCTGCATTACCACAGTAA
>PNNMHK_03950 hypothetical protein
ATGCAATATTTCAAAAATAAAAGTAATGCAGTGTTTGCATATGAAGATGACTGTGATGAGGAATATATCCTGGAAGGGCTAATTAAAATATCGGAACCGGAAGCGATGGAGCTTCTTGCCCCA
ACGGCTGAGGAATTAACTGCTTCAGCTGAAGCAAATAAATCCAGACTGCTTTTAGAGGCGGATAGTGAAATAGACTGGCGTCAGGACGCAGTTGATCTTGGAATCGCAACAGAGGATGAG
AAAGCGCAGCTTGACGAGTGGAAAAAAATACAGGGTACTGGTAAACCGGGTGGACACATCAAAACCTGACTGGCCGGATAGACCAGCCAGTCAGTAG
>PNNMHK_03955 hypothetical protein
ATGCCATTTACGACCGTTTTCTGCATTTTCATAAACCTCGGTTTAGTAGATTTAGCAAAAACAAGCGGAAACAACACATTTACTGGAGAAGCGAAATTCACGTTAAGTGTTTCACTTCCAGCC
AATACAGCTTACAAAGGGATTGGTTTACAGGCTTGGTTTGATTATAAACAAGACAAAGACGATACATTAACAGCGTTATCAGGTAAGGATATTGCCGGATTACTTTTGTATCTCGGATTAACCA
GCGCCGCGAAAACGGATGCTAAAAATATATTCACTCAGAAGCAAACATTTTCAGGTTCAGTTGCGCTCCCGTCTGATACAACTCTGAACGGGACAGGGCTTGAATCTCTTTTTGACAGTAAG
TTAGATGCCTCTAATGCACTGGGAACGGGACAGAACTGGGTGGATGTTACCGCTTCACGAACTGCCGGAGTGACTTACACCAACACCACAACCAGACCGATCTGCGTATCTATTCCTGAAAC
GGCTGATGGTCAGCGTGGATTGTATGTTGGAGGTGTATTAACGGCATTTGCGCGGGGTTCGGACTCAGATTCAACGTTATTTGCCATTGTGCCACCTGGTGCGCAATACATATTCTCGGGTGC
ATTTTCTTCATGGGCGGAGTTGCGGTAA

>PNNMHK_03960 Resolvase/invertase-type recombinase catalytic domain-containing protein
GTGCAAATTGGCTATGTGCGGGTGTCAACAAATGACCAGAACACCGATTTACAGCGAAACGCGCTGAATTGCGCAGGATGTGAACTGATATTTGAAGACAAAATAAGCGGAACTAAAGCA
GAAAGACCAGGATTAAAAAAGTTACTCAGGACGTTATCAGAAGGGGACACGCTGGTTGTATGGAAGTTAGATCGCCTGGGTCGCAGTATGCGTCATCTGGTCGTTCTGGTAGAGGAGCTAC
GGGAGCGTGGCATTAATTTCCGCAGTCTTACGGATAGCATAGATACTTCCACACCAATGGGACGTTTCTTTTTCCATATCATGGGCGCACTCGCTGAAATGGAACGTGAATTAATTGTGGAAA
GAACCCGCGCCGGGCTGGCAGCGGCAAGGGAGCAGGGGCGAATTGGTGGCAGGCGTCCAAAGCTCACCCCGGAGCAATGGGCGCAGGCCGGGCGCCTGATTAGGGCAGGAGTACCG
CGGCGGCAGGTAGCGATTATTTATGATGTGGGAGTGTCCACCCTCTATAAAAAATTTCCGGTGGGTGGTGACTAA
>PNNMHK_03965 Helix-turn-helix domain-containing protein
ATGGACGTTTTCTTGGAAAAGCTAACATTGACACGAAAGGAAGCTGCTGAACTACTTGGTATCTCTACGGCTACCGTAACTCAGTGGGTTCTGGAAGGCAGGCTTAAGGCTTACCGTGTCA
GCAGCAGACCTAAATCACCGTATCTGTTCACCAGAGAAGATTGTCAGGCTGCGCTGTTAGCAGTGGAAGTCGAACCTACCAAGCTCAGGGACAAAGAGCGAAAAGTTGAAGCTGAGGTG
GCGTTCACTCGTAGCCAGAAAGAAGTGAATGAAAAATTGCGTCGGATGCTTAACATGCCTGAAAAAGAATGA
>PNNMHK_03970 Biopolymer transporter ExbB
ATGTCACAGAACCACAAAGTCATGCCAGGTGTAATTCTTTTTGCATTGCCATACTTTCCTGTTATTGCAGCTTATTTTGCATTCAGATACTTGTTTCCTGGCGTTTCGTTCTCTGAACATAGAGA
CGCGCTTGGAGCGGCTATAGCTTCATACGCAGGCACAACGATCGCAATTCTGATCGCTGCGTTAACGTTTGTAATAGGTATTGGTGGCAGAAACATGCATAAGGTAAAGTCTTATGGATACAT
GACAACTGTCATCATCATGTACGCTCTTACCTTTGCTGAGTTAGGGATTGTTTTCTTTATGGGGCTTTTTCTCATGGCTACCAGCAAAGAACCGATTAATTTTCTCCCGACTCTGTCAATTGGTT
TCGCGGCTACTTCATTGATGCACATTAGTTTGCTTTTAGTACAACTGTTCAACTTCTCCAAGAAAAAGTAA
>PNNMHK_03975 Protein rexA
ATGAAAATGAGTTTTTATGCTATTTATTATAGCAATGGAAAAGATAACTCACCATGTAACCTTTTTGAATCACTTTCTTCACAGGTAGTCGAAAAGAAAGGTAACGCCCTTGAAGTTGACGATT
ATTTCGTTTTCGCCCATCACATTGTTGACAAAAGCTTTTTATTAACAAAGACATATGATTCTGCCTTTGTAAAAAGGGTAAACAAGAAAACATTTTCTATTGATGAAATGAAGAATGCTTTGGG
TAATGATGAAACCCTTGCTTTCCCTTCCTTTCTTTTAGTGAAAGACAACATCATCGGTTATGCATCATCTCTACACGGCCCCAGAACCAGAGATCTGCAGATTTATCTTTCCAATAAATTGGATAT
TCCGATGGGCTTCAAACTTTGTATTGAACCTCTGATGCGTGATGTTAGTAAAGATGATGCTTTAGAAATGCAGTTCATCGGTAGAACCACGCTTAGGGTGGAAAGCGGGTCTAAGCTGTTCA
GCCCTTTGCTTAGAGCAACAGGTATTGAAACTATTGAAGAAGAACTTTTAGACGGTATTGAGATCACGCTAAAGCCGAAGAGAGCAAGAAACATTCGAGACATGGCGAAGGAACTCATTA
AAAACGCCGATGACAGCCATAGCGATATACTCATCAAAGGCAAAGAGCAAGCAGCCGATCTTCTTACGGAATACTACCTGTCTAATAAAGGACATCTGGCAGCTAATCTGTACAAATCGACAA
ACGAAGATATCGCTGAAGAGATGGAAACATGCTTCATAAGAATGAAGTCTGTTATAATGAAAAGCTATAACGATGCATTTGATAGTGAGCTTTAA
>PNNMHK_03980 DinI family protein
ATGTTCGTAGAACTGGTTTATGACAAGCGTAATGTTGAAGGACTCGAAGGGGCCAGCGAGATCATTCTGGCCGAACTGACGAAGCGGGTGCACCAGATTTTCCCTGATGCCGAAGTGAGG
GTGAAGCCGATGCAGGGGAATGCGTTAAATAGCGACGCCAGTAAAAGTGATCGGGAAAAGCTGAACCGCATGCTGGAGGAGATGTTTGAAGAGTCCGACATATGGCTGGTGGAAGACTA
A

Prophage 17

>HMDLIN_01680 DinI-like family
ATGTTCGTAGAACTGGTTTATGACAAGCGTAATGTTGAAGGACTCGAAGGGGCCAGCGAGATCATTCTGGCCGAACTGACGAAGCAGGTGCACCAGATTTTCCCTGATGCCGAAGTGAGG
GTGAAGCCGATGCAGGCAAACTGCTTGAATAGTGATGCCAACAAAAGCGATCACGAAAAGTTGAACAGATGCCTGGTTTCAGATTAA
>HMDLIN_01685 hypothetical protein
GTGGTCGTTTTTTTGACTTTTACTGATTCAAAAGGAATATCGGAATATATACTTGACCCTTGGCTGGATGCGCGCATTTTCAAAAAGGAAGAGTCATATGAAATTTATAAAAACAATAGCAGTG
AATATATCAATGAAAATCATTGCTTCGAAGCATACGATAAATACACTGCTATAATGAATAGCGCAGAATATATAGACGCCATAACAAAGACAATAAATCTTTTATATAGAGTTAATCTTGATGAAAT
TCAATTAACAAATCCATTTAAGTTTATATAA
>HMDLIN_01690 Ail/Lom family outer membrane beta-barrel protein
ATGAAAAAAGAAGCATTAGTACTTATCTTATCTGCAGGCATTTTTGCTATAAATACTGCACAGGCAGATACCAACTCACTTACTGCTGGATACTCTCAAGGCAAAATGAAAGATGGCGGTAATA
TCCGTGGGGTGAATGTTAAGTATCATTATCAGGGGGATTTTCCTGTAGGGATTATCACCTCTTTAACTTACATGTATGACAATGACAGGTCTTCTGGAACTGATGAGGATACCGGAGAAACCTA
CCATGATAAGTCGAATGTAAAATATGGCTCGTTAATGGTTGGCCCGACTTATCAAGTAACTGACTCTTTTTCTTTATATGCACTGGTAGGGGCTGCAATCCTTAAAGCAAGAGATAAAGAAAAC
GGTACCTGGGAGGATGGTAGTCCATACACGAATTCAGCGTCAATCAATGAAAAAGCGCTGGCATGGGGGGCTGGCGTACAAATGAATCCCACAAAGAATTTTGTTATTGATGTGGGATATG
AAGGAAGCCGTGAGCTCTTGACACAAATTAATGGATTTAATATTGGTGTTGGGTATCGCTTCTGA
>HMDLIN_01695 tail fiber assembly protein
ATGACGTTTAAAATGAGCGACACCCCGCAGACAATTAAAATTTTTAATCTTCGTTCAGATACAAACGAATTTATTGGCGCAGGTGATGCATATATCCCGCCGCACACTGGATTACCGGCAAACT
GTACTGATATCGCCCCTCCTGATATTCCCTCCAGTCATATTGCTGTATTTGACGCTGAAACCCAAACATGGAGTCTGCAGGAGGATCACCGCGGCGAGACGGTTTACGACACAACAACTGGCA
ATCAGGTTTATATCTCCGCTCCCGGCCCGCTGCCTGAAAATGTCACATCAGTTTCACCAGGCGGTGAATACCAGAAATGGGATGGTAAGGCGTGGGTAAAAGACGGAGCGGCTGAAAAAG
CAGCGCAACTTCGTCAGGAGGAAGAAACCAAAAGCAGGCTCCTGCAAATGGCATCTGAAAAAATCGCGCCGTTACAGGATGCTGTTGATCTTGATATCGCAACAGATGATGAGAAAGCGC
AGCTCGACGAATGGAAAAAATACAGAGTGTTGGTAAACCGGGTGGACACCACAAGTCCTGACTGGCCTGATGTGCCTGTAAGCCAGTAA
>HMDLIN_01700 Phage tail protein
ATGATTAAATTAATTCTTTCAGCACCCGTGCCAGCAATGGCTGCGGCTTTTGAACATTCTTTTCAGAATACCGAAAATGTGGAAATTATCCCAGGACCGTTTGAAACCATACCGGAATTTGACT
GCATGGTCAGTGCGGCAAACTCTTTCGGTTTGATGGATGGCGGCGTGGATGCTGCGATAACAGCATATTTGGGGCTGCAGTTACAGGAACGTGTACAGCAAAATATCATCCGTGAATATCTG
GGAGAACAGCCCGTCGGCAGCGCCTTTGTTATTGAAACGGGTAACAGTCAGCATCCGTGGCTGGTTCATGCCCCGACGATGCGCGTTCCTCTGATAATCGACGGCACCGACGCGGTTTATA
ATGCAACACGTGCAGCGTTATTAGCGATATTTCAGCACAATAAAAGCGCCGGGGAAGGCAGGAAAATTAAATCAGTGGTATTCCCTGCGATGGGGGCCGGGTGTGGTCAGGTATCTCCGGA
CAGTGTCGCCCGGCAAATGAAGCTGGCGTGGGATGGTTTTATTAACTGTGCCTCGGAAATTAACTGGCAATACGCCAGCGCCCGCCAGGATGCTGTATTCAGCACAACGGCATACTGTCCGT
CAAAGGCGCTTTGTCCGAACGCCAGAACGGAATATATCGGTTTTGGTGATTACAGAACGTATTGCAAAAAATCAGGTAACACCTGCATCAGTCCCCGTCATCAGGTCGATGATATTTATATTG
GTGCGCATAGCCATACTGTTTCCCCCGGTACTTATCCCCACAGCCATCACCTGAATACAGAATATTTATCCGGAGTAAAAAATGACGTTTAA
>HMDLIN_01705 Phage tail protein
ATGCCAGTACTTATTTCCGGCGTACTGAAAGATGGTACGGGAACGCCGGTACAGAACTGCACCATTCAGCTGAAGGCCTGCCGGACCAGTACGACGGTGGTCGTGAATACGGTGGCATCG
GAAAATCCGGATGACGCCGGGCGCTACAGCATGGATGTGGAGCAGGGGCAGTACACTGTCACGCTCCTGGTGGAAGGGTATCCCCCGTCACATGCCGGAGTTATTACGGTTTACGATGATT
CAAAGCCGGGCACCCTGAATGATTTTCTGGGGGCCATGACGGAAGACGACGTCCGCCCGGAGGCGCTGCGGCGTTTTGAGGCGATGGTGGAAGAAGTTGCCCGCCAGGCATCGGAGGC
ATCGCGGAATGCCACCGCCGCAGGGCAGGCATCTGAACAGGCGCAGACATCAGCAGGTCAGGCATCGGAAAGCGCCACGGCAGCAGTGAATGCAGCCGGAGCGGCAGAAGCATCAGCC
ACACAGGCAGCCTCATCCGCAGCATCTGCGGAGAGCAGCGCAGGTACGGCGACCACAAAAGCCGGGGAGGCATCAGCCAGCGCGGCGTCGGCTGACACAGCCAGAACGGCAGCAGCC
GCATCGGCAGCCGCAGCGAAAACATCTGAAGCGAATGCAGATGCCTCCCGTACTGCCGCCGGAGATTCAGCTGCTGCCGCAGCCGCCAGCGCGACGGCGGCGCAGACATCAGCAGAGCG
CGCCGGAGCATCCGAAACCGCCGCGAAGACGTCAGAAACGCAGGCGGCTTCCAGTGCCGGTGATGCAGGTGCGTCAGCCACTGCGGCGGCAGCGTCGGAAAAGGCGGCAGCCGCATC
GGCAGCCGCAGCGAAAACATCTGAGACAAATGCAGCAACGTCAGCAAGTACAGCAGCGGCCAGCGCAACAGCCGCCTCGTCATCAGCATCGGAGGCATCCACTCACGCCGCCGCATCTG
ATACTAGCGCATCACTGGCGGCGCAAAGCAGTACTGCTGCCGGAGCAGCAGCCACCAGAGCAGAAGATGCCGCAAAACGGGCAGAAGATATCGCGGACGTGATTTCCCTGGAAGATGCC
AGCCTGACGAAAAAAGGTATCGTTAAGTTAAGCAGCGCCACGGACAGTGACAGCGAAGCGCTGGCAGCCACGCCAAAGGCGGTCCATGCTGTCATGGACGAGGTACAGACCAAAGCGC
CGCTGGACAGTCCGGTATTCACTGGAACGCCGACCACACCGACGCCGCCAGATGACGCTAAGGGACTTCAGACTGCAAACGCTGAGTTTGTTCGTAAACTGATTGCTGCACTGGTCGGTTC

CGTACCTGAGTCGCTGGATACGCTGCAGGAACTGGCGGACGCGCTGGGTAACGATCCGAACTTTGCCACCACTGTACTGAATAAACTGGCGGGCAAGCAGCCGCTGGACGATACACTGAC
GGCGCTGTCAGGAAAAAGCATTGAAGGTCTTATCGAATACGTTGGTTTACGGGAAACGATAAATCACGCCGCCGATGCGTTACAAAAATCACAGAACGGCGGCGATATTCCGGAAAAGCC
GCTGTTTGTACAAAATATCGGAGCGCTTCCTGCATCAGGTACGGCTGTTGCAGCGAACAGACTGGCATCACGCGGCGCGCTTCCGGCACTGACTGGTACGACAAGAGGCAGCGATAGCGG
CCTGATAATGGGCGAGGTTTACAATAACGGTTATCCAACGCAATACGGGAATATTTTGCGTCTGACCGGAACCGGTGATGGGGAAATCCTCATTGGCTGGAGCGGGACAAACGGTGCGTCA
GCACCCGCATATATTCGCAGCCATCGAGATAACGCCGACGCTGAGTGGTCCGAATGGGCAATGCTCTACACCACACTAAACCCACCTCCGGATTCGCATCCAGTAGGGGCGGCGATTGCATG
GCCGTCTGATGTGCTCCCGGATGGTGGTTATGCTTTTATGTATGGGCAGTCCTTCGATAAATCTGCTTACCCGTTACTGGCTATAGCGTATCCGTCCGGCGTTATTCCTGACATGCGAGGCTGG
ACAATAAAGGGTAAGCCCATCAGTGGGCGCGCTGTACTCTCCCAGGAGATGGACGGCAACAAATCGCACTCGCACACCGCGCGGGCGCAGGATACTGACTTAGGGACAAAATCTACCTCA
TCCTTTGATTACGGTACGAAATCGACCAATACCACGGGCAACCATACTCACCAGTTCGGCGGTTATATCAATTCATACTGGGGAGATTCCAATCACACCTCATTTCAGCCTGGAGGTGGTGCAT
GGACACAGGCCGCTGGCGACCATGCGCATACAGTTTATATCGGAGGACACGAGCATACCATGTATATCGGTCCACACGGACACGTCGTTATTGTGGACGCAGACGGTAATGCGGAAACAAC
AGTGAGAAATATCGCATTTAATTATATTGTGAGGCTGGCATGA
>HMDLIN_01710 isrG Hfq binding RNA
GTTTTGCGGCATCTTCTGCTCTGGTGGCTGCTGCTCCGGCAGCAGTACTGCTTTGCGCCGCCAGTGATGCGCTAGTATCAGATGCGGCGGCGTGAGTGGATGCCTCCGATGCTGATGACGA
GGCGGCTGTTGCGCTGGCCGCTGCTGTACTTGCTGACGTTGCTGCATTTGTCTCAGATGTTTTCGCTGCGGCTGCCGATGCGGCTGCCGCCTTTTCCGACGCTGCCGCCGCAGTGGCTGAC
GCACCTGCATCACCGGCACTGGAAGCCGCCTGCGTTT
>HMDLIN_01715 Secreted protein
ATGACTATGTCGCGCGTAATTTCTCTGGCGGCAGGGCTTTCCCTGTCCGTTTTATTTTCCACTGCTGCCGTTGCCGATAACGGAAGAGGAAGCGGCAACAGCAATATTGAAAACCAGACCCG
GATTTATACCGGCACCGACCGTGGGCAGAAACAGCACCGCGAGGCAAAGGGAAAAACAATCACGCGGAGCGTCCAGTGTTCTCTGCCGGCATATTTACGTGACCCGGATAATCAGTGCTG
A
>HMDLIN_01720 Gifsy-1 prophage VhsJ
ATGAGCAAAGGTGGAGGGAAGGGGCATACACCACGTGAGGCGAAGGACGATCTGAAGTCCACACAACAACTGAGCGTGATTGATGCCCTCAGTGAGGGACCGATAGTCGGCCCGGTGA
ACGGTCTGCAGAGCGTGCTGATTAATAACACGCCGATGGTGGACGCGGACGGTAACAGTAATATTCACGGCGTGACCGTGGTATATCAGGTGGGGGAGACACCACAGGCACCGCTGGAAG
GTTTTGAGGCTTCCGGCGCGGAAACGGTGCTGGGTGTGGAAGTGAAACACGATAATCCCGTTACCCGTACTGTTGTCTCAGAGAATGTCGACCGGCTACGCTTCACCTTTGGTGTACAGAT
GCTGCAGGAGACCACGGACAAGGGGGACCGTAACCCGTCCTCCGTGAATCTGCTGATACAGTTTCAGCGTAGCGGGATCTGGAACACAGAATTTGATATCACTATTAACGGCAAGATCACA
ACACAATATCTGGCATCGGTAGTGGCTGATAATTTACCGCCGCGCCCGTTCAGTGTCCGCATGGTCAGGGTGACACCGGACAGCACCACCGACAGGCTTCAGAACAAAACGCTGTGGTCGT
CGTATACGGAAATCATCGATATCCGGCAGGGTTATCCTGGCACAGCGGTTGCCGGTCTGCTGGTGGATGCGGAACAGTTCGGCAGCCAGCAGGTCACGCGTAACTACCACCTGCGCGGACG
TATTTTTCAGGTCCCCTCAAACTATGACCCGGATACCCGCACATATACCGGCCTGTGGGACGGGGCGTTTAAACCGGCGTACACGAATAACCCGGCGTGGTGCACGATGGATAAACTGACCC
ACCCCCGTTACGGGCTGGGCAGGCGTATCGGGGGGGCGGATGTGGATAAATGGGCGCTGTACGCCATCGCGCAGTACTGCGATCAACCGGTGCCGGACGGATTTGGCGGCACGGAACCC
CGCATGACGCTTAATGCGTATATTACCACCCAGCGTAAGGCGTATGACGTTCTGGCGGATTTCTGCTCGGTGATGCGTTGTATGCCGGTATGGAATGGCCGCAAAATGACCTTCATCCAGGAC
CGCCCCTCCGATAAAGCATGGACCTACACCAACGGTAACGTGGTGGGCGGGCGCTTTAAATACAGCTTCAGTGCCCTGAAAGACCGCCATAACGCGATAGAAGTGAGATACACCGATCCGC
TGAATGGCTGGCAAACCTCCACGGAGCTGGTGGAAGACCATGCCTCACAGGCCCGTTATGGACGCAATCTGCTGAAAATGGACGCGTTCGGCTGTACCTCACGTGGACAGGCGCACCGGA
CGGGGTTGTGGGTGATGATGACGGAGCTGCTGGAAACGCAGACCGTGGATTTTTCTGTCGGTGCGGAAGGTCTGCGTCATCACACCGGGCGATATTATTGAGGTCTGCGACAACGATTACG
CCGGGGCGTCGGTCGGTGGGCGTATCACTGACCTGGATATTTCCACCCGCACGCTGACGCTTGACCGGGAAATAACACTACCGGAAAGCGGCGCCACCACGCTGAATATTGTCGGGCCTG
ACGGTAAGCCGTTCAGTACGGAGATTCAGTCGCAGCCCGCACCGGATCGGGTGGTAACGAAAGTCCTGCCGGAAACCGTGCAGCCGTACAGTATCTGGGGGCTGAAACTGCCCTCCCTGA
AGCGCCGCCTTTTCCGTTGCGTGCGTATTAAGGAGAATGACGACGGCACATACGCCATCACTGCCTTGCAGCACGTTCCGGAAAAAGAGTCCATTGTGGACAACGGGGCGCACTTTGACCC
GTTACCGGGGACCACCAACAGCATTATTCCGCCCGCTGTACAACATCTGACCGTCAGCACGGATAACGACAGCACCCTGTATCAGGCCAAAGCGAAATGGGATACGCCGCGGGTGGTAAAA
GGTGTGCGTTTTGTCGTGAGACTGACTATCAGAAACGGGAAAGATGACGACCCCGCGCGTCTGGTGACAACCGCCACAACCAGTGAGACAGAGTACGCTTTCCACGAATTACCGCTGGGT
GACTACACGCTGACAGTCAGGGCAATAAACGGATTCGGGCAGCAGGGGGAGCGTCATCGGTCACGTTCAGCATTCAGGCTCCGGCGGCACCGTCCACCATTGAACTGACGCCGGGCTAT
TTTCAGATAACGGTGACGCCGTACCAGGCCATTTATGATGCCAGTGTGCAGTATGAATTCTGGTACTCCGCAACACAGCTGACGACCGCAGCAGACATTCAGTCAAAAGCGCAGTATCTGGG
CATCGGGTCATTCTGGATAAAGGATGGACTGAAACCACTGCATGATGCCTGGTTTTACGTGCGTAGTGTAAATCTGGCTGGAAAATCAGTGTTTGCGGAAGTGTCCGGGCGTCCGAGTGAT
GACGCGAAAGGGTATCTGGACTTTTTTAAAGGACTGATTACGGAGGCGTATCTTGGTACAGAGTTGCTGAAAAAAAATTGACCTGACGGAGGATAACGCCAGCAAACTGCAACAGTTTTCG
AAGGAGTGGAAGGACGCTAACGATAAATGGAACGCCATGTGGGGCGTCAAAATAGAGCAGACCAAAGACGGCAAATATTATGTGGCCGGAATTGGACTGAGTATGGAAGACACGCCTGA
CGGGAAGATAAGCCAGTTCCTGGTGGCGGCGGATCGCATTGCTTATATTAACCCGGCAAACGGAAACCAGACGCCAGGATTCGTCATGCAGGGCGACCAGATAATCATGAACGAGGCGTT
CCTGAAATACCTGAGCGCGCCGACTATCACCAGTGGCGGTAATCCTCCGGCATTTTCCCTGACGCCGGACGGAAAGCTGACTGCGAAAAATGCGGATATCAGCGGCCATATTAACGCTGTGT
CTGGCTCGTTTACCGGAGAAATCAATGCCACCTCCGGTAAGTTTTCTGGCGTGATAGAAGCAAAAGAGTTTGTCGGTGATATCTGCGGCTCAAAAGTCATGCAGGGCGTGAGCATCAGAGC
GAAGAACGACGAACGCAGCACCTCAACACGGTATACCGACAGCGCCACCTATCAGATAGGGAAAACCATCACGGTGATGGCTAACTGTGAGCGTAACGGTGGCTCCGGTGCCATCACCGT
CACGATAAATATTAACGGCCAGGTGAAAACGGCGGAGGTTATCCCGTATACCGCAGGGCTTCCGGCCATGTATCAGACAGTTGTCTTTTCGGTCTACACCACTTCACCTGTCGTGGATATCAG
CGTTTCTCTGAGGGTCGGCGGGCAGTACACCACTGAAGCTTCCGTCTGGCCGCTGGTGATGGTTTCCCGGTCGGGGAGCAACTTCACAAACTGA
>HMDLIN_01725 Tail assembly protein
ATGAATGAGGGCTGGTATCAGGTCCGGATTGCCGGGAGCGATATGGCGCCGGATACCCTTACTGCCAGACTGAACGAATCGTTACCGCCGGGGGCAGTGGTTCATATTGTACCGCGTATGG
CGGGAGCGAAAAACGGTATCTGGCAGGTGGTAGCCGGGGCAGCGCTGATTGGCGCGTCATTTATTCCCGGTCTGAATGCTGTAGCGGCGGCAGTATTGTTTTCCGCAGGAACCAGTATGG
CGCTGGGTGGTGTGGCGCAGATGCTGACACCTGTACCCAAAACACCAACGGTGGGTCAGACAGATAACGGGAAACAGAACACGTACTTTTCTTCCCAGGAAAATATGGTGGCCCAGGGG
AACCCGGTGCCGGTGTTGTACGGTGAAATGAAAATCGGGTCACGGGTGATATCGCAGATGATGAGTACCCGGGATGAGAGCACGTCGGGAAAAGTTGTGGTGATCGGCTCCCCGTTACAG
GCAAACACCACGTCGCGGCAGGACGGCGGGATTACCAGACCGTCTGTCGTCATCCGGCAGTGA
>HMDLIN_01730 Phage tail assembly protein
ATGATTAACGATGACATTCTGGCACATGCCCGACAGTGTGCGCCTGCGGAATCGTGCGGTTATGTGGTCAGAACGGCACAGGGAGAGCGGTATTTTCCGTGTGAAAATCTGTCTGCTGAAC
CCACGATGTATTTTCGTATATCCCCGGAGGATTACCTGAATGCCCGGAACCGCGGCGACATCGTGGCGCTGGTACACAGCCATCCTGACGGTAAGCCCTGTCTCAGCAGTGCGGATCGTACC
CTCCAGATACAAAGCGGGCTGGACTGGTGGCTGGTCCGTGATAACAGGATACATAAATTCCGCTGCGTGCCACACCTGACCGGGCGGCAGTTTGAGCATGGCGTGACGGACTGCTACACG
CTGTTTCGTGATGCCTACCATCTGGCCGGGATTGATATGCCGGATTTCGATAGGGAAGATGACTGGTGGAGTCAGGGTAAAAGCCTCTATCTGGATCACCTGGAGGCGGCGGGATTTTACCG
GGTGAATCCGGAGGATGCGCAGCCCGGCGACGTGCTGATTTGCTGTTTTGGTTCACCGACGCCCAATCATGCGGCGATTTACTGCGGCAACGGTGAACTGTTGCACCATATTCCGGAGCAG
TTGAGTAAACGAGAGGGGTATAACGACAAATGGCAACGACGGACACACTCAATATGGCGGCACCGGCAATGGTGCGAATCTGCCTTTACGGGGATTTACAACGATTTGGAAAGCGCATCA
GCCTCAGCATAA
>HMDLIN_01735 Phage minor tail protein L
ATGCAGGATATACCACAGGAAACCCTGAGCGAGACCACCAAAGCGGAGCAGTCCGCGAAGGTGGATTTGTGGGAATTTGATTTAACCGCGATTGGCGGTGAGCGCTTTTTCTTCTGTAAC
GAACCGAACGAAAAAGGCGAGCCGTTAACCTGGCAGGGGAGGCAGTACGAACCGTACCCGATACAGGTACAGGATTTGAGATGAACGGGAAAGGCGCATCTCCCCGCCCGAACCTCGT
TGTTGCCAATCTCTTTGGTCTGGTCACGGGAATGGCGGAGGATTTGCAAAGTCTCGTCGGCGCGTCAGTGGTAAGGCATCAGGTTTACAGCAAGTTTCTTGATGCGGTGAATTTCAGTAAC
GGCAATCCGGGCGCTGACCCGGAGCAGGAGGCGGTAGCGCGCTATAACGTGGAGCAGTTGTCAGAACTGGATTCATCAACTGCTACCATTATTCTGGCATCACCGGCAGAAACCGACGGT
TCTGTGGTGCCGGGGCGTACCATGCTAGCGGACTCCTGTCCGTGGGATTACCGGGATGAAAACTGCGGATACGACGGCCCGCCCGTGGCCGATGAGTTCGATAAGCCCACCTCAGACCCG
AAAAAGGATAAATGCAGCCACTGCATGAAAGGCTGTGAAATGCGTAACAATCTGGTGAATGCCGGATTTTTCGCTTCCATCAACAAACTGTCTTAA
>HMDLIN_01740 superoxide dismutase [Cu-Zn] SodC1

ATGAAATACACAATATTGTCGCTGGTAGCTGGTGCGCTCATCAGTTGTTCAGCAATGGCAGAGAATACCCTGACTGTAAAGATGAACGATGCCCTGTCCAGCGGAACAGGAGAAAACATAG
GTGAAATCACAGTTTCAGAGACACCTTACGGTCTGCTTTTCACTCCTCACCTAAATGGTCTTACGCCAGGAATTCACGGCTTCCATGTCCACACAAACCCAAGTTGTATGCCGGGAATGAAAG
ACGGTAAAGAGGTTCCGGCGCTCATGGCCGGAGGACATCTTGACCCCGAAAAAACCGGGAAACATCTTGGCCCATATAATGACAAAGGGCATTTGGGGGATCTGCCTGGACTGGTGGTCA
ATGCAGATGGTACAGCCACGTATCCGTTACTGGCACCACGCCTTAAATCACTGTCAGAACTGAAAGGTCACTCATTGATGATCCATAAAGGCGGTGACAATTACTCCGATAAACCTGCTCCAC
TGGGTGGTGGCGGTGCACGTTTTGCCTGTGGTGTCATTGAGAAATAA
>HMDLIN_01745 hypothetical protein
ATGGAAAAGATTGCCGTGGCGGTTTTAGTTGGTCTGGCGTTAGGTAGCATCGGCGTGGCTAACGCAGCAGGGTATAAAAATACCGTTTCAATTGGATATGCCTACACAGATTTAAGCGGCTG
GCTTTCCGGTAATGCGAACGGTGCCAACATCAAATATAACTGGGAAGATCTGGACAGTGGATTCGGGGCGATGGGTTCAGTTACATACACCTCGGCTGATGTTAATAACTATGGGTATAAGG
TAGGTGATGCTGATTATACCTCCCTTCTTGTTGGTCCTTCATACCGTTTTAACGACTATCTGAATGCTTACGTGATGATTGGTGCAGCAAACGGACATATTAAGGATAACTGGGGAAATTCTGAC
AATAAAACCGCCTTTGCTTATGGGGCAGGTATTCAGCTTAACCCGGTTGAAAATATTGCCGTTAATGCGTCTTATGAGCATACAAGTTTTTCCACTGATGCTGACAGTGACGTCAAAGCTGGA
ACCTGGGTGCTTGGCGTAGGTTACAGCTTCTGA
>HMDLIN_01750 Phage tail protein
ATGATGAAAACCTTTCACTGGAAAGTTGACCCGGACATGGGGGTGGATTCGGAACCACAGGTGTCGGTGGTGAGGTTCGGTGATGGCTACGAGCAGCGGCGTGCATCCGGGATCAATAAT
GACCTGAAAAAAATACAGTGTGACTATCCGCGTTGACCGGGAGGATGGTCCGGCACTGGAGGGCTTTTTGTCACAGCATAACGGTGTGAAGGCGTTTTTGTGGACTCCGCCTTACGGATACC
GGCAGATTAAGGTTGTCTGCCGGAAATGGAGTGTGAAAGCGGGATTGCTGAAAACAACATTCACCGCGACATTTGAGCAGGTTATTTCTTAG
>HMDLIN_01755 Phage tail tape measure protein
TTGAGCCAGAAAGTCGGTGATATCGTCATCAACATGGATGTTGATACAGCTAAAGTTGCCGCCGGTCTTCAGACTGCCAGTAACGGGCTGGGGAAGCTGGTGGACAGCAGTGATCTCGTTG
AAAAACGCATCAAGCGATGTATGGAGTCCAGCGCCAGAAGTGTGGCGGCATCGGCAAAAAGTATCAGTGCCGCTATGGCGCAATCACAGGTTGCCACACGCACACAGAGTGACGCTATGG
CACAACTGGCGCGTGAGGCGAACGAGGCCAGAGAAAGGGCTGTCGACCTGAATCAGAAGTTAAGGGCGGAAGCTGCGCAGGCAGCGGCGGTTGCACAGGCTCAGGATGCAGCCGCAG
CGGCATTTTACCGTCAGATTGACAGTGTAAAACAGTTAAGCGGTGGTCTGCAGGAGTTACAGCGTATCCAGGCGCAGGTACGACAGGCGAAAGGACGCGGAGATATTTCACAGGGCGATT
ATCTGGCGCTGGTGTCTGAAGCTGCTGCAAAGACACGCGAACTTACCGATGCGGAGGCGCTGGCCACGCAGAAAAAAGCACAGTTTATACGTCGACTGAAAGAGCAGACGGCGGTACAG
GGCCTCTCCCGTACTGAGTTGCTGCGGGTGAAGGCGGCTGAACTGGGGGTTAGCAGTGCCGCCGATGTCTATATCCGCAAACTGGATACCGCAACAAAATCCACTCATGCACTGGGACTGA
AATCAGCAATGGCGCGCCGCGAGATAGGCGTACTGATTGGTGAACTGGCACGGGGAAATTTTGGCGCCCTTCGCGGTTCCGGTATCACGCTGGCCAACCGGGCCGGGTGGATTGAGCAA
CTGATGTCGCCGAAGGGCATGATGCTCGGCGGGCTGGTTGGCGGTGTGGCTGCGGCGGTTTACGGACTGGGTAAGGCGTACTATGAGGGGGCGAAAGAAAGTGAGGAGTTCAATAAAC
AGCTTATTCTGACCGGGAGTTATGCCGGAAAAACCACAGGCCAGCTTAATGCGATGGCGAAGTCGCTCGCCGGAAATGGCGTCACGCAGCACGATGCTGCAGGCGTGCTGGCACAGGTG
GTCGGTAGCGGAGCGTTTACCGGGCAGGCAGTGGCAATGGTATCCCGTACCGCGACCAGAATGCAGGAAAACGTGGGACAATCAGTGGATGAAACCATCCGCCAGTTTAAACGCCTGCG
GGATGATCCGGTGAATGCGGCGAAAGAACTGGACAGGACACTGCATTTTCTGACCGCCACCCAGCTTGAACAAATCAGGGTACTGGGCGAGCAGGGAAGAGTGGCTGATGCCGCGAAA
ATTGCCATGTCCGCGTATTCGGAAGAAATGAATAAGCGGATGGGGGACGTACACGACAATCTGGGCTGGATTGAAAGAGCATGGAATGCTGTCGGTGATGCGGCGAAGTGGGCATGGGAT
CGGATGCTGGATATCGGGCGGGAAGACACGCTCGATGAAAAGATCGCGACACTGCAGGAAAAAATCGCGCGCGGCAGAAAAACGCCCTGGACGGTGTCTTCCTCCCAGACTGAATACGA
TCAGCAGCAGCTGAACGAACTTCAGGAACAGAAACGCCAGAAGGACCTGCTGGATGCGAAGGCGCAGGCAGAGCGTAATTATCAGGAAACGCAGAAACGTCGGAACGAGCAGAACGC
CGCGCTGAACCGGGATAATGAAACTGAATCCCTGCGGCATCAACGGGAGGTGGCGCGCATTACCGCCATGCAGTATGCCGATGCTGCGGTACGCAATGCCGCGCTGGAGCGTGAAAACGA
ACGCCATAAAAAAGCAATGGCACGGCAGAAGGAAAAGCCAAAGGCTTACCACAACGACGAGGCCGGGCGACTGCTTTTGCAGTACAGCCAGCAACAGGCGCAGACTGAAGGGCTGATT
GCCGCCGCGAAGCTTTCCACGACCGAAAAAATGACGGAAGCGCATAAGCAGCTTTTGTCATTTCAGCAGCGCATCGCTGATTTGTCCGGTAAAAAACTGACGGCGGATGAACAAAGCGTA
CTGGCACATAAGGATGAAATAGCGCTTGCGCTACAGAAGCTGGATATCTCACAACAGGATTTGCAACACCAGAATGCCTTTAATGAACTGAAGAAAAAGACGCTCACATTAACCAGCCAGC
TCGCTGACGAAGAATCCCGCGTCAGGCAGCAGCACGCACTGGCGCTGGCCACAATGGGTATGGGCGATCAGCAACGTGGCCGGTACGAAGAGCATCTGAAAATTCAACAGCACTACCAG
GAACAACTGGAGCAGCTTAAGCGCGACAGCAAGGCAAAAGGGACATACGGTTCTGACGAATACCGTCAGGCGGAGCAGGAACTTCAGGCCAGTCTCGATCGCCGACTGGCTGAGTGGG
CGGATTATAACGCGAAAGTGGATGCTGCGCAGGGAGACTGGACGCAGGGCGCGTCGCGGGCGCTGGATAACTTTCTGGCGCAGGGGGGCAACGTGGCAGGCATGACGGAGAACGTTTT
CACAAACGCATTTAACGGCATGGCGGACAGTATCGCGAATTTTTCCGTGACCGGAAAGGGCAGTTTCCGGAGCCTGACGGTCTCCATCCTGGCTGACCTGGCAAAAATGGAGGCACGTATT
GCGGCTTCTAAACTGTTGGGTTCAGTACTGGGTATGTTCGGCTTTGGCGCATCAGCAGGCGGAAGTACACCATCCGGGGCATACAGTTCAGCGGCGCTGTCGGTCATTCCAAATGCGGACG
GCGGCGTGTACCGCTCAGCAGGACTCAGTCAGTACAGCGGCAGTATTGTTAACAGACCGACGTTCTTTGCATTTGCCAGAGGGGCGGCAGTAATGGGAGAGGCCGGTCCGGAGGCTATAC
TGCCGCTTCGTCGCGGTACTGACGGTAAGCTGGGGGTTGTGGCAGCAGGTTCCGGAGGGATGGCGATGTTTGCGCCGCAGTATCATATTGCAATCAGCAACACGGGGCCGGAGCTGACG
CCGCAGGCGCTGAAGGCGGTTTATGATCTGGGTAAAAAGGCGGCGGCTGATTTCGTGCAGCAGCAGGGGCGTGACGGCGGCAGGCTGAGCGGGGCATATCGATGA
>HMDLIN_01760 hypothetical protein
ATGAGTTCAACTGAACTTAGTGAGTGGGCTGATTTCTTTCGGGAAAACAGTTTCAGTGATGCCCTGCTGGATGCGGAGTTTTCCACGCTGAAAGCGCAGGTGTTCATGCTGGTGACGGGG
AAAGAAATCGATGCAGCGGACTTCAGTTTGCTGACATTACCCGGAGCGGTACAGAGCATGACGGAGCAGGATCTGCTTGAAGTGGCAGTCGGTATTCCGGGAGGAGTGAGATTTGAGCC
AGAAAGTCGGTGA
>HMDLIN_01765 hypothetical protein
ATGTTTTTAAAGAAAGAGACGTTTACCCGGGGGGATGCGTCGGTGGCATTGTTCGAGTTATCCGGCCTGCAACGTATTGAGTACCTGGAGTTCATCCAGAAACGTACTGCGAAATATGACAC
GGATATGGATGGTGCGACGGAAGCGGATAAGCGCGTGGCTTATATGCAAATGGCACTGGAAATTAATGCCTGGCTGGTATCCCGCTCGCTTCTGAATGGTGATTCTTCTCAGGATGCAGACA
CGCTTTATCAGTCAGTACAGGCGAAGTGGTCGTATGAGGCGCTGGACGCAGGCGCAGAAAGTGTTCTGATGCTCAGCGGGCTGTCTGCGGATAAGAAAGATAACGCCAGTGATTCAGGTA
ATGAATCAGAGGACATGACGCCGGAAAAGTCCTGA
>HMDLIN_01770 BIG2 domain-containing protein
ATGACAGACACGACAATTCCTAATCCACTTGCGCCGGTAAAAGGCGCAAATACCACATTCTGGATGTACAACAGCGAGGGTGATCCGTTCGCCAGCCCGCTAAGTGATAATAACTGGCTGAG
GCTGGCAAACGTAAAAGACCTGCAGCCGGGAGAACTGACGGCAGATGCAGAAGATAATAACTATCTTGATGACGAGAACGCTGACTGGAAAAGTACCACACAGGGACAGAAGTCTGCCG
GGGATACTTCTGTGACGCTGGCGTGGAAGCCAGGTGAGGACGTACAGAAAAAGCTTATTCAACTGTTCACGACCGGGCAGAAGCGCGGATTCCGGATCAAGTATCCGAACGGCACTGTTG
ATGTGTTCAGAGGCTGGGTGAGCTCGCTGGGGAAAACGGTGCAGAGTAAGGATGAGATCGCCCGTACAGTAAAAATTACCAGTGTGGGCCGTCCGTACATTGCTGAAGAGGATGCACCG
GAAGTGGTTAGTGTTACCGGACTGACAGTGGAACCGACAAATGCCACCGTGAAGGTCGGCGCCACAACAGCGGTTACGTTCACGGTGAAACCGGATAACGTGACAGATAAATCACTGCGT
ATCGCAACGTCAGATCCGACTACAGCCACCGTCACGCAGGCGGAAAATATTGCCACTGTAAAAGGTGTTAAGGCAGGTACAGTGAAAATTATTGGCATGACAACAGACGGTAATTTTACCG
CTATTGCGGATATTACTGTTCAGGCATAA
>HMDLIN_01775 Phage tail protein
ATGACCCGACACAGTGCAGTACGGCAGGCCATTATTGCCGCATTAAAAAAGACCGATGATGGCTCCACCACGTTTTTTGACGGTCGTCCGGTCGTGGTGGAGGAGGATGAGTTACCTGCGG
TGGCGGTGTACCTGAGTGATGCTCAGTATACCGGTACTGAGGTGGACGGTGATATCTGGAGCGCGGTGCTACATGTGGAAGTATTTCTGAAAGCCACTGCACCGGACAGCGCCCTGGATGA
ACAGATGGAGAACAGGGTGTATCCGGCACTGGGGAGCGTGGCGGGACTGGGTGACATCATCCGGACAATGTCGGCGCAGGGGTATAACTATCAGCGAGACGATGAAATGGCGATGTGGG
GGTCAGCCGATCTGAGTTACGACATCACCTATTCCATGTAG
>HMDLIN_01780 Phage tail protein
ATGGAGGTAAAGGGGCTTAAAGAGGCCATTTCAGTACTTAAAGAAATTGATCGTGGATATGTGACCCGGGCAAAAATTCGGCCATTAACCGGGTGGCAAAACGGGTGGTCAGCGTGTCA
GTTCGCAGTGCTGCTGCTTTGGTGGTGGCCGGAGACAACCGACGGCAGGGTATTCCCGTCAGAACGGTAAGACGTCGCGCCAGAGTCAGGCTGGCCAGAGCGGACAAGCCTTTTGCCAA
CATTTATGTGAACTGTGATCCGCTGACTGCCATCAGGTTACTGAGTTCGCCACCATCAACCCCGATGAGGGGGAGAAAAGGTAAGCCGCTACGTATAGGAAAATACCGTTTTGACCGGGGG
TTTATTGCGCAGGCTCCGAACGGATGGTGGCAGGTATTTGAGCGTTCCGGTGCCGGAAGATACCCACTTAATGTGTGGTGAAAATCCCGGTTGCTGATGCATTGCGCCATGCGTTCAACACGCA
GGTTGTTTTACAGATGAAAACGGAGATGCCCAAAGAGCTGAAGCATGAAATCAGTTATGAACTGAGGAGATTCACTAAAAAATGA

>HMDLIN_01785 ATP-binding protein
ATGGATGCACTTATCGCAGAACGCTTCGGCATGAAGGTCAACATCAACGGGACTGACTGCATAGTGGTGGAGTCTGATTTTCTGGCTGAACTGGGGCCTGTTGAAGGAAACGGAAAAAAC
GTAGTGGTGTTTTCAGGCAACGTTATCCCGCGCCGGGGAGACAGGGTGGTACTGCGGGGCAGTGAGTTTACCGTGACCCGTATCCGGCGTTTTAACGGTAAGCCACAACTGACTCTGGAG
GAGAACAATGGAGGTAAAGGGGCTTAA
>HMDLIN_01790 DUF2190 domain-containing protein
GTGGCTAAGAATTACGTGGAAGACGGCAAAACGATTGAAATTGTGGCGACCACGTCACTAAAGAGCGGAGATCTGGTACAGGTCGGCGATATGTTCGCTGTGGCTGTTACCGATATTGCCG
CCGGGAGCGCCGGAACCGGCATCGCAGAAGGGGTATTCAGCATACCAAAACTGACAACAGAGGATATTGCCGTCGGGAAAAAAGTGTATCTGAAGGATAACGTGGTTCAGACGGATGCA
ACCGGCAGCCTGCCGTATGTCGGGGTGGTATGGGCGCCAGCGGCAAACGGTGATGAAACCGTTCCGGTAAAAATTAATGGCTGA
>HMDLIN_01795 ATP-dependent Clp protease proteolytic subunit
ATGCCGCCGGTGATGACCACAGGGAACGAACGCGGGGAGGGAAGCAGTAATAGCTGGTATTCCATCAGGGCTGCAGTCAATAACACGGCGGAGGTCCGCATTTATGACGAGATTGGTGG
ATGGGGAATTTCGGCTCGCTGGTTTGCAGAAGAACTGGCTGCACTGGGACAGATTAACCGGATAAATCTGCATATTCATTCACCTGGCGGAGCAGTACTGGACGGAATAGCCATTTATAACC
TGCTGAAAAATCATCCGGCGCAAAAAACGGTGTATATCGATGGAATGGCCTGCTCAATGGCATCCGCTATTGCGATGGTAGGTAATCCCATCATTATGCCGGAAAACGCCATGATGATGATTC
ATAAGCCGCGCGGAGTGGCGGGGGGGGAGGCGGAGGATATCCGGGAATATGCTGACCTGCTTGACAAGATCGAAAGCGTCATTATCCCCATCTATACCGAAAAAACTGGAAAAACGCCTG
AAGATATTGCTGCCATGCTGGCAAAGGAGACCTGGATGAGCGGCGCGGAGTGCGTCAGTGAAGGGGTTTGCCGACAAACTGATACAGCCTGTAAAAGCAATGGCCTGTATTCATTCAAAAC
GTGTTGAGGAGTTTGAGCATATGCCACAGAGCATTAAAGGTATGATTATCGCCCCGCAGGGCAATGCAGGCGCGCAACCGCAGCCACAGGCAAAAGCCCCTGAGTCACAGATTCAGTCGC
AGGCTCAGTCGCCGGCTGTAGATGAAAATGCCATTCGCGCACGCCTGCAGGAAGAACAGCGTAACCGTATCACCGGCATTCAGAATGTGTTTTCACTTTCCGGCGATCGTTATGCCTCGCTG
ATGGCAGAGTGCATTGCTGATGTGGATTGCTCTCTGGAAATGGCGAAGGACAGACTGTTGGCCGAGATGGCGAAAGGTATTACGCCGACTAACCAGCTGAATGGTCCGCAGAATCGCGCA
GAGTTTCATGCCGGGATGTATACCGGAAACGGTAATATTACCGGCGACGCCGTTCGCGCTGCCGTGATGGCCCGCGCAGGCTATGAAGAAGCGCAGAAGGATAACCCGTACAACTGTATGA
CCCTGCGTGAACTGGCGCGAATTTCGCTGGTGGCGAGGGGTACAGGCGTGTCCAGCATGAATCCCATGCAGATGATCGGCATGGCATTTACGCACAGCACCTCCGATTTCGGTAACATTCT
GCTGGATGTTGCAAACAAGTCCATTCTGCAGGGCTGGCAGGAAGCGCCGGAAACCTTCGATGCCTGGACCAAAAAAGGACAGTTGTCTGATTTCAGGATTGCGCACCGTGTGGGTATGGG
GGGATTCAGTTCACTGCGTCAGGTTCGTGAAGGAGCGGAATACAAATACGTCACCACAGGGGATAAACAGGCGACCATTGCGCTCGCCACCTATGGGGAGCTGTTCAGCATTACCCGCCA
GGCCATCATCAATGATGATATGAATATGCTGACGGATGTCCCGATGAAGCTGGGACGTGCGGCAAAAGCCACCATTGCCGATCTGGTTTATGACGTTCTCATCAGTAACCAGAAACTGTCCAG
TGATAATGTGGCGCTGTTTGACAAGACGAAACACGCAAACGTCCTTGAAAAAGCCGTTATGGATGTGGCGTCCCTGGATAAAGCGCGCCAGCTAATGCGGATGCAGAAAGAGGGCGATCG
CCACCTCAATATCCGTCCGGCATTTGTACTGGTGCCGACGGCGCTGGAGTCTGTGTCAAATCAGGTGATTAAATCTGTCAGTGTCAAGGGGGCGGATATTAACGCCGGGATCATTAACCCGG
TGAAAGATTTCGCCACGGTGATCGCGGAGCCTCGTCTTGATGACGCCAGTCAGTCCACTTTCTATCTTACTGCAGCAAAAGGCAGCGATACCGTTGAGGTGGCTTACCTTAACGGCGTGGAT
GAGCCGTATATCGACCAGCAGGAAGGATTCACGGTGGATGGCGTAACCACGAAAGTTCGTATTGATGCGGGCGTTGCGCCGGTGGATTATCGCGGTATGGTGAAATGCACCGTATAA
>HMDLIN_01800 Phage portal protein, lambda family
ATGAATTTTATCGATAAAGCTATCAGCATGATGTCGCCGGGATGGGCTGTATCAAGACTCCGATCGCGGGCAGTAATAAAAGCTTACGAGGCTGCAATACCCACCCGGACACATAAAATTAAG
CGTGAAAACCGCAACGCCAATCAACTAAACCAGATTGCCGGAAAGTCCCTGCGGGAGCAGGCCCGCTGGTTCGATAACAATCACGATCTGGTGGTGGGGGCGCTCGATAAGATGGAAGA
GCGCGTTATCGGGGCTAAGGGGATCATCGTTGAGCCACAACCACTGACGGTGGCCGGAACGCTGAATAACGCGCTGGCGGAACAAATCCACGCCAGATGGGCAGAGTGGTCCGTGTCGC
CGGACGTGACCGGACAATATACGCGCCCGGTACTGGAGCGCCTTTTGTTACGCACATGGTTGCGGGACGGGGAGGTGTTCTCCCAGATGGTGGCGGGAAAAATGCCGGGGCTTGAGCCG
GTGGCGGGCGTACCGTTCTGGCTTGAGGCGATGGAACCGGATTATGTCCCGATGGAACAGACGGACAGTACAAATAATCTGGTTCAGGGGATCTATTTTAACGACTGGCAAAGGCCAAAA
AGTTACATCGTCTGTAAATCCTGGCCGGGATTTGCCACCGCTATGGTGGCCACCAAGCTTATCGATGCAGAAAATATGCTGCATCTTAAATTCACCCGTCGCCTGAATCAGGCGCGGGGCGTC
ACATTGCTGGCGCCGGTTATTATCCGGCTGCTGGATCTGAAGGAGTATGAGGACAGTGAAAGGCTGGCCGCGCGTATTTCGGCGGCTTTTGCCATGTTTATCCGGCGCAGTGACGCAATGG
TACAGGATGGTGACGCGCCAGATTATGCGGATAAAGACCGCGATCTGGATATTGAACCCGGCACCATTCTGAAAGACCTGTTGCCGGGAGAGGATATCGGCACCATCAAATCTGACAGGCC
AAATGCCAACCTGGAGTCTTTCCGTATGGGGCAACTGCGAGCGGTTGCCGCAGGTGTACGCGGAAGTTTTTCTTCTATTGCCAGAAACTACGACGGCACCTACAGCGCCCAGCGTCAGGA
GCTGGTGGAGGCTCAGGAGGGATACACCATTCTACAGGATAATTTTATTGCTGCCGTCAGTCGCCCGGTTTACCGGCGATGGCTGGCGACGGCGATTACGGCGGGTGTGATAGATGTGCCG
ACGGACACGGACATGGGGACCCTGTTTAATGCCGTGTATTCCGGTCCGGTTATGCCGTGGATAGATCCGCTGAAAGAGGCGAATGCCTGGCGGGTGCTGATACGCGGCGGGGCGGCAACA
GAGAGTGACTGGGTGCGTGCCCGTGGCGGCGCACCTGCTGAAGTGAAACGCCGCCGTAAGGCGGAAATTGACGAAAACCGTAAGCTGGGACTGGTATTTGATACTGACCCGGCACATGA
CCCGGGGGAACAGGATAATGCCGGAAGTGAAGACAACAGTGGCGGCGATAAAAATGCCGCCGGTGATGACCACAGGGAACGAACGCGGGGAGGGAAGCAGTAA
>HMDLIN_01805 Primosomal replication protein PriB/PriC domain protein
ATGACCAGGGCAGGGCTACAGGAGTTGTACAACGCCTATCTGGAGGCCGAGCTTGCTGTACTGAAAGGCAAGTCCATCATGCTTAACGGTCAGTCAATGACAATGGAAAGCCTGGAGGAA
ATCAGGAAGGGGCGCCGCGAGATTGAAGACAGGTTACAGCGGTTAAATAATCCGCGACGGCTTTTCACCCGGGCGAGGTTGTCATGA
>HMDLIN_01810 Terminase, large subunit
ATGACAACGGTGACGAAGGGCAAGAGGAAGAGCAGGAATAGTTTAGCCTCTCTGCTGTTGTTGCCGGATGTGATATCTCATCGATGTTCAGGCCGCCGCGCAGGATGAAGATATCGGATGCG
GTCAGAAAATATATGCGCGTACCACGCGATGCGGGAAATTCTGTGGCATGGGAATCCACCCTGACCCCCTATGTGGTGGAAGCCATGAACTGCCTGTCATCACGCAGCTACGATGCCGTGGT
ATTTGTCGCCCCGGCAAGAACCGGTAAGACGCTGGGGCTGATTGACGGCTGGATTTCCTACAATATTGTCTGCGATCCGTCTGACATGCTGGTGGTTCAGATGACTCAGGACAAGGCGCAG
GAGCACTCAAAGCGGCGTCTGGCAAAGATGTTTCGCCACAGTCCGGCGATAGCAAGAAGACTCAGCCCTCACCGTAACGATAATAATGTGCATGATAAAACGTTTCGTGATGGCTCGTTTCT
GAAAATTGGCTGGCCATCAATCAATGTTTTTTCGTCTTCGGATTTTAAATGTGTGGCGCTGACTGACTACGATCGGTTCCCCGAAGATGTTGATGGTGAGGGGGATGCATTCTCGCTGGCGTC
GAAGCGTACCACAACGTTTATGTCGCTGGGTATGACACTGGTGGAGAGCTCGCCGGGGCGGGAAATTACCGATACCAGATGGAAACCGTCATTACCGCACGAAGCACCGCCGACCACAGG
AATACTTTCCCTGTACAACCGTGGCGATCGTCGTCGCTGGTACTGGCCGTGTCCGCATTGTGGCGAATATTTTCAGCCATCAATGGCGAATATGACCGGCTATCGCCATATTGCCGACCCTATG
GAGGCCAGTGAAGCAGCGCGGATTCAGTGCCCACACTGCCATAAGTTGACTGAACCACAGCAGAAGCGTGAGCTGAATAATCGTGGCGTCTGGTTGCGGGAAGGTCAGCACATTGACCG
CGACGGCAATATTACCGGCGAAGCCCGCCGCTCACGTATCGCCAGTTTCTGGATGGAGGGACCGGCGGCGGCTTATCAGACCTGGGCGCAACTGGTTTACAAGCTGCTGACCGCCGAAGA
GGAATACGAACGAACCGGCAGCGAGGAAACCCTGAAAGCCGTCATCAATACGGACTGGGGATTACCCTATCAGTCCCGTCGCTCGCTGGAGGCACGCAGCGGTGACGCGCTGATGGCAC
GCGCTGAGGACGTATCAAAACGTACCGTTCCTGACGGGGTGCGTTTTATTGTGGCAACCGTTGACGTACAGGGCGGTAAAAAGCGGCGCTTTGTGGTCCAGGTAGTGGGCTATGGCGCAT
ATGGTGAACGCTGGATCATTGACCGCTACAACATCCGTTATTCCCTGAGAGTGAATGAGGATGGCGAAAGCCAGCCTGTCAATCCGGCTGCAAGACCGGAAGACTGGGATTTGCTGAGAA
CGGATGTGCTGGAAAAGACGTATCCGCTGGCGGCAGATCCTGAACAGTTTATGCCGGTACTGGCAATGGCGGTGGACTCAGGTGGTGAAGACGGTGTTACCGATAATGCCTACGCGTTCTG
GCGAAGGTGTAAGCGAAAAGGCGTTGCCGGGCGCGTTTATCTGTTCAAGGGAGACAGTACCCGACGCGAGAAGCTGATCACCAAAACCTATCCCGATAACACCGAACGCTCAGAACGTC
GGGCAAAAGCCAGGGGGGATGTGCCGTTGTATCTGTTACAGACCAATGCCCTGAAAGACAGGGTAGCTGCCGCACTGGAGCGTGAGGAGCCGGGGGCGAATTACATTCATTTTCCTGACT
GGCTGGGGCCGTGGTTCTACGAGGAACTGACCTATGAAGAGCGCAGCGCAGACGGAAAGTGGAAAAAACCGGGACGCGGTAATAACGAGGCGCTCGATCTGATGTGTTACGCCCACGC
GCTGGCTATTCTCCGTGGCTATGAGCGAATCAACTGGGAAAAACCACCAGGATGGGCTCGCTTACCGGAAAAAGACGCCGCAAAAACCACGCAGCCTGTTGAGGTTCGCCAGCAGGGGC
AACACGAAGGAGGAGAGAAAGCCGTGAAAGCCAGGAAGAAAAAAATTTACCCGCCTGGGGCGGTGGTTCCGGAGGAGGGTGGTTATGA
>HMDLIN_01815 DUF1441 domain-containing protein
GTGGATCAGGAAATTAAAAGCCTCGAATTAAACATCACACAGCTTTCGGCCATCACTGGTGCACACCGACAGACCATCGCCAGCAGGCTGAAGGGCGTAAAAACCTCAGGTGGGAACGGT
AGTAACCTGAAAATCTACCGGCTGGTGGATATTCTGACCGCCATGATGACGATGCCGGCTGTTACCGGGGAGAATGACCCCAATAAGATGAAACCCTCAGATCGACGGGCATGGTTTCAGTC
GGAAATGACGCGTATTGAGCTGGAAAAGGAGATGAGAACTCTGATCCCGGCCAGCGAGGTGCTGAGCGTTTATGCTGTCATGGCAAAAACGGTCGTCAAGACACTGGAGATACTGCCGG
ACTTACTGGAAAGGGATGCGGCATTGCCGCCTGATGCGCTGGAAATGACGCAAAAAATTATTGATCAACTCAGGGAAGATCTGGCCAGCATGACATACCAGGCCTGCGCGGATGCTATAAA
CGGAGATGATGATGACAACGGTGACGAAGGGCAAGAGGAAGAGCAGGAATAG
>HMDLIN_01820 Spanin, inner membrane subunit

ATGAATCGTATAACCACCGGCGTAATAGTCTCGTTGCTGATAGTGATCGCAGCGCTGGCATGGGCCACTGACCACTACCACGGTAACGCGGTGAAGTACAAAGAGCAGCGCGACACCGTTA
CTCATAAGCTGGCGCTGGCGAACGCGACAATTACTGACATGACGAAGCGCCAGCGTGACGTCGCCGCCCTCGATGCAAAATACACAAAGGAATTAGCTGATGCGAAATCTGAGAATGATGC
TTTGCGCGATGATGTTGCCGCTGGCCGTCGTCGCCTGCTCGTCAACGCAACATGCCCCACAGTGCCGACAGGTAAATCCACCTCCACCGCCCGCGTGGATAATGCAGCCAGCCCCAGACTG
GCAGACTCCGCTCAACGGGATTATTTCACCCTCAGAGAGCGAGTAAAGACGATGCAAAAGCAACTCGAAGGGGCGCAGGCGTACATTCGTACCCAATGCCACAGTAATGCAGGAAAAAAT
AATAATAAATAG
>HMDLIN_01825 hypothetical protein
ATGATTGAGCAATTACCAAACTGGAAATTTGTCCTGATCTGGCTGGTACTGTTTGTCGCCGCTATTGGCTATCTGATAGGACAAATCCGTTGGTGGTGA
>HMDLIN_01830 Endolysin
ATGGCGCTCAGGACAAAAGTTAAATACGGTCTTTCCGCCGCCATGCTGGCGCTGATTGCCGCCGGTGCCAGCGCACCGCAGCTACTCGACCAGTTTTTACAGGAGCGGGAAGGAAATACG
CTGGTGGCCGTTCGTGATAACGGCGGCGTCTGGTCAGTATGCCGTGGCGTGACCCGTATCGATGGTAAACCCGTCGTGAAAGGCCAGCGACTGACACAAAGCCAGTGCGACCATTACAAC
GCCATCGAGCGGGATAAAGCGCTGTCATGGGTAAATAAACATGTTCATATACCGCTGACTGAACCGCAGAAAACCGGTATTGCGTCGTTCTGTCCGTATAACATCGGTCCCGGTAAATGTTTT
CCGTCCACGTTTTACCGGAAGCTCAACGCAGGAGATCGTAAGGGAGCGTGTGCAGAAATCCGCCGTTGGATATATGACGGCGGCAAAGACTGCCACAACAGGGAAAATCAGTGTTACGGC
CAGGTGATACGACGCGACCAGGAATCGGCGCTGGCGTGCTGGGGTATTGAACAATAA
>HMDLIN_01835 Alpha/beta hydrolase
ATGACAATGGATAAACATACAACATGGCTGGCCTACATCTGGGCATTAATCAGCGGCATATGCGCCCAGTGGACGTTAAACGACTATGGCGCATTGATAGGTATTGTTCTGGGTATTGGTACG
TTTCTGGTTAATAAGCATTACAAAAAGAAATCAGAGCAGGCTCAGGCAAGGCAGGCTGCCGCGATGGAGGAGCGTAACAGGCTAATCGCCCGGATTCTGGAAAAAAACGACCATGACAG
CACGTTAAAAATGCTGGCGGTATCTGAAATGCCGGAGGGGAATAATGGCGCTCAGGACAAAAGTTAA
>HMDLIN_01840 DUF2441 domain-containing protein
ATGAACGGGAAAACGTTGTACACCCTGGACAGGCTCGGAACATTATCAGCAGGAGCTCGCATTGAACATCAAACAGCTTGTTGTTCCATCGAACTTCAGGAGCACGTCGCAAATCGTTTTT
GGTCACAAGTATCAAGGCACGGCAATAATTATTTCTTCAACCACAATATCAACCTTCTTAAATCCAAAGAAAATATGAGTATTTTCATGGAAATGCTTCTTGAAGAAAGAAGAAGAGCAAACT
TTCCAGATAAACCATCCCGGTTTCGTTCTCTCTTTGCCTGTGAAGCTATCCATGATGCAGCCAGGTTTAGATTGTTAAGCCATGTGCCTTCAAATACAGCTATCTATGAAGTCCATCAGACCGCA
GGTTGCCACAGAGCTGATATGAATTTACTCAATGTAAACTGTACCCCTCCAGAAATGTCACATCGACTGGATCTTTACTGGCAAGGTAAAACAAAAGAATTATATCCCGGCTATGAACCATTTT
GGGAAGTACTTGTACCGTTGCCTGCAATCATTGGTGGGAGGATTCAGGAATAA
>HMDLIN_01845 OKR-DC-1 domain-containing protein
ATGTATCAATCGGGTCAACACAGTCACTCATTGTTTCCGGGAGTGAACAGCGACAACCTCAAAACGTTTGTGCTTGAAGTTGACTGTTATCATTTAGAGGATGCGCTGAAAGCAATGGAAAA
AGTTATCCAGGAGTTAAAGCTGAGTCAGAGCTATTTTGGCTACGGAAGTAAAACCGGAGTCAGGACAGCAATCCGTTATTCCTGA
>HMDLIN_01850 isrI Hfq binding RNA
ATTGTCGGTGTCTGGCATCTGACAAGGGGCGATGATATCTGCCATAACGGTGAAGCAGAAGTACTGTCGGGGCCGTTACAACCACCAGCTTAGATAT
>HMDLIN_01855 Hydrolase
ATGAAAGAAGGGTATTACTGGATTCAGCATAACGGTGTTGTTCAGGTGGCATACTATACGAATGACACAGTTGATGATCTGGAATCAGGACGGCTTATTGTCGGTGTCTGGCATCTGACAAG
GGGCGATGATATCTGCCATAACGGTGAAGCAGAAGTACTGTCGGGGCCGTTACAACCACCAGCTTAG
>HMDLIN_01860 Antitermination protein Q
ATGAATCTTGAGTCCTTACCGAAATATTTTTCACCTAAATCCATGATGCCCGGCGCAGTACCATGCGGAATAACGTCTGATACGCTGACTATTACTGACGTAATGGCATCACTCGGGCTACTTAC
TGCAAAAGCCGCAGTGGGTATTGAATTGTATCTTGCAAAAGCTGGGGTTTTATCTTCTGAAAATATCATCGCCTATATCAGGCTATTAGCAGAGCAGCGTGCAGAACGGCATGGGGCATTACG
GAAAATGGAAGAGGGTAAGCGTTCAAAATTTCTCGACACTATGGCGCGTTATGTATTTCGCGATTATTCCCTCAGCGCGGCCAGCCTGGTGACGTGCAGTAACTGTCATGGTGCTAAATTAAT
TGATGCTGAGGTTTTCACGAACAAGGTTACCTACCCAGATGGTAAGCCACCAAAATGGGTAAAAGATACGAAAGGTATTTCTCCGTCCGACTGGGAGGTGTGGAAATCAGTTCGTGAGCAG
GTACGCGTAGTGTGTAAGGTGTGTGATGGCAAAGGCCATGTGAAAAATGAATGTCGTTGCCGGGGACGCGGAGAAATTCTCGATAAGAAAAAATCTGAGTTGCAGGGCGTGCCGGTTTAT
AAAAAATGCCCAAGATGCAAGGGAAGAGGCTACCCACGTCTCAAAGATACCGAGATTTTTAAAGCACTAGGAGTAACGGAAATGGTATGGCGGTACAACTATAAACTGTTTTTCGATCGGC
TGGTGGAGCATTGCCATATTGAGGAATCGTATGCAGAAAAGGTTCTGGGAAACGTGACTCGATGA
>HMDLIN_01865 Protein ninG
ATGGCTAAATTACCGCGCCGTAAGTGCAAAGTTTGCCGGGAATGGTTTCATCCTGCTTACAGCAACGTTGTCTGGTGCTGTCCTGAACATGGCGCTATCTATGCCCTGGAACTGCGTGCCAAA
GAAAAGATTAAAGCCGCAGCCAGGCGTATCAGGGAGAAACACCAGGCGGATAAAGCCGAACGCCAGCGCCGCCAGGCTAAGCGTGAGTCGTTCAAAACTAAAGCTCAGTGGGATAAAG
AGGCGCAGGCCGCCTTTAACCGTTACATCAGGATACGGGATGAAGGTAAACCCTGCATTAGCTGCGATGCGCCGCTGGTTGGTAAAAGCAATTTCCTGACCGGAAGCGCCATCGATGCAAG
CCATTACCGCTCGCGCGGTGCCGCCTCACATCTCAAATTCAACGTATTCAACGTTCATTCGGCCTGCACGCGCTGCAACCGGCAGTTAAGCGGTAATGCGGTCGAATACCGAATACGCCTCAT
CAGGCGTATAGGCTTGGAAAGGGTGGAGCGTCTTGAATCTGACAATGCGCCACGTCGTTTCGATATCCCGTACCTGAAACGCATCAAATCCATATTCACCCGCAAAGCCCGGGCGCTGGAG
AAGCGCCGCGCACGTCGGCAGGAACATGCAGCATGA
>HMDLIN_01870 Phage protein
ATGGCGATGAAATACTCCTGGTTCCATCATCATGACTGCACAACCGAGCAGGCCGACACGCTGATATCGGATTATCAGAAGCGGGGCGTAAGGACAGAAAAGAGCCTGAACCCTGACTTCA
TTACCTGGACTGTCAGCGCGAAATTACCTGAATATGCACACCGGGTGCGGACGCCAAAATCCTTACGCCAAAAGGTCTGGGGGTGA
>HMDLIN_01875 DUF1367 domain-containing protein
ATGGCGCACGAATTACAACTCATCAAGCAGTCATCTGGAATTCTGATCCCCGCAACGCCGGAGACCAGTGATATTCTGCAATCAAAAATCAAACTCGGCGCCGTGCTGGTGGCTGAGTTCCG
TCAGGTGAGGAATCCTGCATTCCATCGCCGCTTTTTCGCGTTGCTTAATCTTGGGTTTGAATACTGGGAACCCACCGGCGGCGCCATTTCTGCCAATGAGCGCAAACTGGTAAACGGTTATG
CAAAGTTTCTCGCTGCATATGGCGGGAATGAAAGCGCATTACTGGATGCGGCTGAACAGTATCTGGAACAGATTGCAAACCGCCGGGTAACAAACGGGATTAGCCTGTGTAAATCATTCGAT
GCCTACCGCGCATGGGTGACGGTTGAGGCTGGTCACTATGACGCCATCCAGCTACCGGACGGCACCCTTCGCAAACATCCCCGCAGCATCGCTTTTTCCAGTATGGATGAGGTCGAATTTCA
GCAGTTGTATAAATCTGCGCTTGATGTTCTCTGGCGCTGGATTTTATCACGTACATTCCGTACTCAGCGCGAGGCCGAGAACGCCGCCGCCCAGCTCATGAGCTTTGCGGGGTGA
>HMDLIN_01880 DNA damage-inducible protein I
ATGAGAATTGAACTTGTTATCAGCCGGACAAAACAGCTTCCGGAAGGTGCCGTTCCTGCACTTGAAAAAGAATTAATTACCCGTCTCCAGAATCAGTATGAAAACTGCAACTTAACCATCCG
TCGAGGCAGTCAGGATGGTCTGAGTATCGTCGGTGCTGCTGATGGCGATAAAAAACGTATACAGAGCATTCTGCAGGAAACGTGGGAAAGCGCTGACGACTGGTTTTATTAA
>HMDLIN_01885 hypothetical protein
ATGATAACTGCATTAGATATTGAAAAAGTAATCACCGATAAAGGGCCAATGAGCAACATCAAAGGTCCGCTTATCAGCAGTCAGCGCTACCTCGACAAGGCAAAGGTAAACGACAGAGCGG
CAAGATTTAAGCGTTTTATCGTATCTGTTTACCCGATAGTTCTGCGTGGGCAGCAATACACCATCCTGATGGATGGCCACCACAACTACGCGGCGGCATCCGGGAATGGTCCGGCTTAA
>HMDLIN_01890 Replication protein P of bacteriophage
ATGAAAAACATTGCTGCGCAGATGGTTAATTTTGACCGTGAGCAGATGCGCCGTATTGCCAACAACATGCCGGAACAGCATGACGATAAGCCGCAAGTTGAGCAGGTTGCTAAGGTCATCA
ACAACGTGTTTAGTCAGCTTATGGCAGCGTTCCCTGCTACCACAGCTAATCGCAGCCAGGCTGAGATGAACGAAATCCGGCGTCAGTGGGTTCTGGCTTTCCGTGAGAACGACATCACCAC
CATGGAGCAAGTTGCGGCCGGAATGCGTGTCGCCCGCCGTCAGGAACGTCCGTTTCTTCCGTCTCCAGGCCAGTTCGTCGCATGGTGTAAGGCTGAATTGGCAACTGCTGCCGGGCTTCCT
GATGCCAATGAACTGGTCGATATGGTTTACCAGTATTGCCGCACGCGTGGTTTATACCCGGATGCAGAATCATATCCATGGGAATCCAAAGCCCATTACTGGCTTGTCACCACACTGTACTCAA
ACATGCGAGCTAACGCTCTTAGTGATACCGAGTTGCGCCGCAAGGCAGTCGAAGAACTCAACCACATGGTTACCAGGATAAACCGTGGAGAGGAGATCCCGGAGCCAGTTAAGCAGCTTC
CTGTTCTTGGTGGCAGACCACTAAACCGCGCGCAAAACCTCGCGAAGATTGCAGAAATTCGGGCCAAATTCGGACTAAAGGGAGTCAGATCATGA
>HMDLIN_01895 Phage replication protein O

ATGGCAAACACTGCCGAAGTAATCAATTTTCCTGTGCCTGACGTGGCACCTAAGGAGCCGCGCGTGGCAGATCTCGATGATGGCTATACGCGCCTGGCAAATGAACTTCTGGATGCCGTGAT
GTGTTCTGGTTTGCCGGAGACTGAGCTGTGCATCCTGATGGCCGTATGGCGCAAAACGTATGGATACAACAAGAAAATGGACTGGATCAGCAACGAGCAGTTAGAGGAGATGATTCAGAA
GCATCATACCCATTGCTCGACAGCAAAAAACAGTCTGATCAGGAAGAAGGTACTGATTCAGGAAGGCCGCAGGGTTGGTATGAATATCCATATTTCCGAGTGGCAAACTAAAAATAACGGAT
TCTGCAAAACATTAGCTAAACCTGCTAAGAAAACCTTAGCGGAAGTTGCTAACGCACCTAAGCAGAAGTTGCTAACCACAAAAGACAAACTAACAAAAGACAATATTAAAAGATCTACGTCA
GAGAATTCTGACGAATCCTCTGACAAGCCAAGAAAGAAACCTCATGTTCTAAAACCCGAAGCAGCGATTCAGAGAGGCAACAAGTGGGGAACTGCTGAAGACCTAACTGCTGCCGAGTG
GATGTTTGACCTGATAAAAACCATTTCTCCATCAGCCAGAAAACCTAACTTGGCAGGATGGGCTAACGATATACGCCTGATGCGTGAATGTGACGGACGAACACATCGCGACATGTGCGTGC
TGTTTCGCTGGGCGTGCCATGACAGCTTCTGGGCTGGCAACGTCATTAGCCCGGCAAAGCTCCGCGAAAAGTGGACTCAACTCGATATCAACCGCAACAAGCAACAGACTGGCACAACTG
CCTCTAAGCCAAAACTTGACCTGAATAACACTGACTGGATATACGGAGTGGAGCTATGA
>HMDLIN_01900 Regulatory protein CII
ATGGAGAACGCAATTGCACGAAAGTTAGACCCACCAGAAATCAACCCGGTTGAGATAGAGAGTGTCCTGCTCAACCGGCTTGCATCAGTAGGGCAGAAATCATACGCCGAGCATATGGGC
ATCAGCGAGTCGACAGTCAGCAGGCGTAAAGCTGAGGGATATTTCTGCAACATGGCGAAAGAGCTGGCTTTTCTTGGGATTCAGGCCGCGCCACCGGAGGCGGTACTGGTATCCAGAAAC
TATCTCACAGCTGTAGAGATTCTCGCTGATGCAGGGCTAAAGGCTGAACGAGCCAGGCCGGATGCGCTGGGGTGGGACTGA
>HMDLIN_01905 Helix-turn-helix domain-containing protein
ATGGACAACCAATTAAAAATCAAAATCAGTAACCACATGACTCAAATGAGCATCGGCGAACATTTTGGTATCTCGTCTCAAGCTGTAGGCAAGTGGCTGAGAAAAGGAGTAATTCCTCCCCG
TCGCATCCTGCCGTTATGTGAAATTCTTGAATGGAAAGTCACTCCTCACGAGATTGACCCAGCAGCATACCCAAACCCAACCGATGGCTTACCAAGCCAAGAGGCATCAGCCAAATAA
>HMDLIN_01910 Helix-turn-helix domain-containing protein
ATGAACAAAAATCTTCATCCCATTTTCGCCAAGCGTATCCAGCAAGTTCTGGATGAGAACGGCTGGTCTATGGCTGACCTCTCACGGCGCGTAATGCTTTCTCACACATCTGTGAGAAAGTGG
GCCTCTGGCACATCTGTAGCCAGCGGAGAGCGCTTGAAAAGGTTATCGGCGGTGACCGGAAGGCCTGAATATTGGTTCTTCATGGAGCCAGGGGATGAAAGTGAAGGCGAAAGGGATGA
ACCTAAACCCAGAGTCCTTGATGAAAAAGAAGAAACATTGCTTTCTCTTTTCAATCAACTCCCGGAAGCAGAGAAACTGCGTGTTATCCTCCATACAAAAGCAGTCCTCCAAGAGATGGATC
TGCTGAAGAACAACGTTTTTGATCTAATTAACGACCTCAAAAAATAG
>HMDLIN_01915 Prophage protein
ATGCCTCAAAATAGAACTTTTAGTTGCGATTTTTACTTTACAAATCGAACCTTTGGTTTTATTGTTGATTTCATCGACAACAAGCGCATCGTTGTCAGGTGTAAAACGTTCCGCTGGCCGGCGA
TAAGGCAAACGAGGGTGAGAATGATTGATTTCGCACGTAAACCAGCTCGACAGCAGGCCGTCCCGCTCAACCGGATTGAGGTTTTAATCCGCCGCCTCTGCTACCTGCTGGCGCAGAAAG
GAGATCCGGATGCATAA
>HMDLIN_01920 hypothetical protein
ATGCATAACCAAAAGACATGCGCTTACCACCTGTGTGGAAAGACGATTGAGCAAGGCAAAGAAGTAAAAAACGAGCTGACGCTGATTCGCGGCGCGCAGCTGACACATGAAGAGCGCGA
TTACTGCTCTGTACGTTGTGCCTCATACGACCAGATGGCGCACGAAAGTTAA
>HMDLIN_01925 DUF3799 domain-containing protein
ATGGAATTTTTCTATGTAGTAAAAGCTACGCAGAAATCCGGAAAGCAAGATGCGACGGTCTGGTTCACTGCAAAATCAGAAGCGCGCGCCAACCTTATGCTGGATGTCGTTCTGGAAGATG
CTGAAATTGAAACCGGCCGCGGTAAGGATTATGCAAGGCCGATCCGCACCAATTTTCCGGTAGTCAACGAGCTGCCGCCGGAAGGTGAAATAAGTTTTACCTTCACTAATTATTATCGCCTC
GGTGAAGATGGCATGACTTGGGAACAAATCCCCGGCGTCACCCTGCCATCATCTGAAGCCGCCGCCGCGGCGCGCCAGCATATCGTCGACGGTGTTGATACCGAAACAGGCGAAGTGCTG
GAAGACCACACCGAAAATTTTGGTAACGAAAGCAACAGCCCTGCCCAGGCAACAGCCCCAGCCCCCGAGCTGACTGTTGTCGCAACTATGCCTCTCCGTCACCGCGTTCTTGCTCAGTACA
TAGGTGAAGGTGAGTATCTTTATCACGTCGACGCCTCCCAGAAAAAAGAAATTCTGCGTCTCGAAATGGACACCGATAATTCATATGTCCAGAACCTGCTGCTTGCCGCCGAGAATGTTGAA
GCGTTCAAGAAAGCCATTGAACATGACATTCACAAAATAGTGAATGCCGTTAAAAAAGTATTCCCTGTCGATGGAAAAACTCCTGAACTGGCGACTGTTATCCAGTTCCTTAAAACATGGTTC
GAGACGGAGCATATCGATCGCGGTTTGCTCGTTAAGGAGTGGGCGAAAGGCAACCGTGTATCGGCTATTCAACGCACTGAAAGCGGCGCCAACGCTGGCGGTGGCAATAAGACTGACCG
TAACCCTGATTACGAACACACTCTCGATACTCTGGACGTAGAGATTGCAATGGCCACTTTGCCTATGGACTTTAATATCTATGAGCTACCTGGCAGCGTTTACCGTCGCGCAAAAGAAATCGTA
AAGAAAAAGGAAAGTCCGTTCAAAGAATGGTCGCGCAGCACTTCGCGCAAACGCCCGGTATCCTGGATTATTCCCGCGCCGCTATTTTCGCGCTGATCCGAAGCGCACACCCTGAGTTTTATCA
CTACCCCGGACGCCTTCAGGGGTATATCAACGCCAACTTAACGGAGACTGATCACGAGACCCCCACCGAGGAAGCTCTCACGGCTGCCCGACACACTCCGGAAAAAGACGCGGTAGAAGA
AGCCAACCGACAGCTTGCCGCCGCGCGCGGTGAATATGTGGAAGGCATCAGCGACCCGAACGACCCAAAATGGGTGAAGACCGGGACAAGCCAGCCGACCACCGAACCTGAACTGGTT
AAAAATGTTGGCAACGGTATTTTCGACGTGTCCGCTTTAATGCAGAACTCATCAACTCATGGCACAGAAACGAATCCGGAGACCACCAGCAATGTGCAGGTTCAAAAAGCTGACAGTGATG
AAAAACAGGCTGGTGATGCGGTGCAGGCAGGCGAAGGCGATCTGGGTACTGGTAAAGAAGCAGTTACCGTAGAGAACCAGAATCAGGCTGAGACGCACCAGAACAACGATTCTGTGAG
CCAATCTGAACCTGAGGCGCAACAAAACGTACCGGAATCGCAACAAGAAGAGCCAGAAGCAGCCTGGCCGGAATACTTCGAGCCGGGCCGCTATGAAGGTGTACCAAACGAGGTTTACC
ACGCCGCCAACGGGATCAGCTCAACTCAGGTGAAAGATGCTCGCGTGTCGCTGATGTACTTTAACGCGCGTCACGTAGAGAAGACTATCGTCAAAGAGCGCTCTCCAGTGCTTGATATGGG
CAACCTGGTACATGTTCTGGCTCTACAGCCGGAAAACCTCGAAGCGGAGTTCAGCGTAGAGCCGGAGATCCCTGAGGGTGCTTTCACCACCACCGCCACCCTGCGCGAGTTCATCGACGC
GCACAACGCCAGCCTGCCAGCGCTGCTGAGTGCTGACGATATCAAAGCGCTGCTGGAAGAGTACAACGCCACCCTGCCGTCGCAGATGCCGCTTGGAGCTTCGGTAGATGAAATCTATGCA
TCGTATGAGCAGCTTCCCGAAGAATTCCAGCGCATTGAAAACGGCACCAAACATACAGCCACGGCGATGAAAGCCTGCATCAAAGAGTACAACGCCACCCTGCCCGCGCCGGTTAAAACC
AGCGGCAGCCGTGACGCGCTGCTGGAGCAACTGGCAATAATCAACCCTGACCTGGTCGCTCAGGAAGCGCAAAAATCGTCGCCGTTGAAAGTCTCTGGCACGAAGGCCGATCTGATTCAG
GCCGTGAAATCAGTCAACCCGGCAGCGGTATTCGCCGACGAATTGCTGGATGCGTGGCGGGAGAACACCGAAGGGAAAGTGCTGGTCACCCGCCAACAGCTCAGCACCGCGCTGAACAT
TCAGAAAGCCCTGCTGGAGCACCCGACCGCCGGCAAATTGCTGACTCACCCAAGCCGCGCTGTCGAGGTTAGCTATTTGGGATTGATGAGGAAACCGGGTTGGAAGTTCGGGTACGCCC
TGACCTTGAGCTCGATATGGGCGGCCTGCGCATTGGCGCCGACCTGAAAACTATCAGCATGTGGAACATCAAGCAGGAAGGCCTGCGTGCGAAGTTGCACCGGGAAATCATCGATCGGGA
CTATCACCTGAGCGCGGCCATGTACTGCGAAACTGCGGCGCTGGACCAGTTTTTCTGGATTTTCGTCAACAAAGACGAGAACTACCACTGGGTCGCCATCATTGAGGCGTCTACCGAGTTG
CTGGAACTTGGCATGCTGGAATACCGCAAAACAATGCGAGAGATAGCAAACGGCTTCGACACTGGTGAATGGTCAGCGCCTATCACAGAAGACTACACCGACGAACTGAACGATTTTGATG
TGCGCCGCCTTGAAGCGTTGCGCGTACAGGCATAA

Region of unknown function 1

>BDIOMP_08460 Polysaccharide biosynthesis protein
ATGAATCGTATTATTAGAATGTTAGGTGTAGATAAAGCAATTCGTTATGTTATTTTTGGTAAGATAATATCTGTATTAACGGGTTTACTGTTAATAATGTTAATATCACACCATTTATCTAAAGACGC
ACAGGGCTATTATTATACATTTAATTCAGTAGTGGCACTACAGATAATATTTGAATTGGGGCTATCAACGGTAATCATTCAATTCGCTAGCCATGAAATGTCAGCGTTAAAATATGATTATTCTGA
ACGAGATATTATAGGTGAAAGTAAAAATAAGCAACGTTACCTATCGTTATTTCGGTTGGCAATAAAATGGTATGCAGTAATAGCTTTGCTAATAATATTAATAGTCGGTCCCATCGGGTATGTTTT
TTTTACGCAAAAAGAAGGCTTAGGTGTACCTTGGCAAGGGGCATGGTTATTATTAACAATAGTTACAGCTTTTAATATTTTTCTTGTTTCTGTACTTTCTGTCGCTGAAGGGAGTGGGTTAATT
ACTGATGTGAATAAAATGAGAATGTATCAGTCGCTGTTAGCTGGTATATTGGCAGTAAGCTTACTTATTAGTGGCTTTGGACTATATGCTACGTCTGCAATAGCTATTTCAGGGACTATCATATTC
TCCATATTTTCATATAAGTATTTTAAAAAAAATTTTCCTGCAATCTTTAAAGCATAAAAATAAATATACTGAAGGTGGTATTTCATGGGTTAATGAAATATTTCCTATGCAATGGCGAATTGCTCTAA
GTTGGATGTCAGGGTATTTTATTTATTTTGTTATGACCCCCATTGCATTCAAATATTTCGGGGCTATATATGCAGGGCAGTTAGGGATGTCTTTAACATTATGCAATATGGTAATGGCTACGGGCC
TGGCTTGGATATCCACTAAATATCCAAAATGGGGAGTAATGGTTTCCAACAAACAGCTTGCGGAACTGAGTAAATCGTTCAAAAGTGCAGTAATGCAATCATCCTTTTTTGTCTTGACAGGAT
TAACTGGTGTATACATTTCATTATGGTTATTGAAATTATCTGGTTCAAACATTGGCGAGCGGTTTTTGGGATTGCAGGATTTTTTCTTTTTATCTTTAGCAATTATTGGTAATCACATTGTAGCTT
GCTTTGCAACCTATATAAGAGCGCATAAAACTGAAAAAATGACATTGGCATCATGTATAATGGCTCTCTTGACTATAACTACAATGTTGTTTGTTGCATATTTAGAGTACTCGAGGTTCTACATGT
TAATGTATGCAGCACTAACGTGGTTATATTTTGTTCCTCAAACTTATATAATCTTTAAAAGATTCAAGAGTTCTTATGAGTAA
>BDIOMP_08465 Glycosyl transferase

ATGAGTAAAAAACCTCTTCTTACTATTGCTATTCCGACATATAACCGCTCTTCATGTTTGGCTCGTTTACTTGATAGTATAATTCAACAGGAGAACTATTGTCATGATGAACTCGAGGTTATTGTT
TGTGATAATGCTTCAACAGATGAAACAGCAAGAATAGCCAAGAGTGGCTTAGATAAAATAAGAAATAGTACTTATCATCTAAATGAAGAAAACTTAGGAATGGATGGTAACTTCCAGAAATGT
TTTGAGTTATCAAATGGAAAATATCTTTGGATGATTGGCGATGATGATCTAATAGTCAAAAATGGTATTTCGAAGGTTTTTTCGATATTAAAGTCCCGGCCTGCATTAGATATGGTGTATGTAAAT
TCAGCAGCAAAGACTGAGTTAAACTATAATGCTGATGTGAGGACGTCATTCTACACAAATGATGTAGATTTTATTTCAGACGTGAAAGTTATGTTCACGTTTATTTCTGGAATGATATGTAAGA
AAACTGATGCAATTGTCAAAGCCGTTGGTATTTTCAGTCCGCAAACTACTGGAAAATATCTTATGCATTTAACATGGCAATTGCCATTACTTAAACAGGGTGGAGAGTTCGCAGTTATCCATAA
TAATATAATTGAGGCTGAGCCAGATAATTCAGGTGGATATCATTTATATAAGGTTTTTTCTAATAATCTTGCGACAATCTTTGATGTTTTTTATCCCAGAGAGCACCGTGTAAGTAAAAGAGTTC
GCGCATCAGCATGTTTATTCTTACTTAACTTCATAGGCGATGAAGATAAAACCAAAAATTTTGCTACAAATAATTATTTAAGAGATTGCGATAGTGCATTTATAGATTTAATTATATATAAATATGG
GCTTAGGTTTTTCTATCTATACCCTAAAACTGTGCCTTTATTTAGAAAAATAAAATATATTATAAAGACGGTTTTAATGCGTAAATAA
>BDIOMP_08470 Transferase hexapeptide (Six repeat-containing protein)
ATGTTTGTCTATAGTTTAAGATTAAAATTAAATCTTATCATATCATTATTGAGTAAAGTTAGGCGGAAATCAAAAGCAAAGTTTCTTGTTCTGCTTAGCGGATATGATTTTAAAATGGTTGGGAA
GAATTTTAAATTGAATGTCAAACCTTACTCTGCAAAAAATAACACCTCTTCCAAATGGGGTAGTATGCGGGTTGGTGATAACTGCTGGATTGAAGCTGTATATAATTATGGTGATGAAAAATTT
GAACCTTATTTGTACATAGGTGATCGTATATGTTTAAGTGATAATGTTCATATTTCTTGCGTATCATGTTTAATTTTAGAAAACGATATATTAATTGGTAGCAAAGTTTATATAGGCGATCATAGCCA
TGGCAGTTATAAAGTATGCAGTCCGAAAATAGAACCGCCAGCAAATAAGCCATTAGGTGATATTGCTCCTATTAAAATAGGTAATTGCTGCTGGATTGGAGATAATGCAGTAATTCTGGCTGGT
AGTGAAATTTGTGATGGCTGTGTAATCGCAGCTAATTCAGTCGTCAAGGATTTAAAAGTCGATAAGCCATGTTTAATTGGTGGGGTTCCTGCTAAAGTAATAAAGGTATTTTAA
>BDIOMP_08475 Abequosyltransferase RfbV
ATGAATGTTTTTATCAGTATTTGTATACCGTCTTATAATAGAGCTGAGTTTTTAGAGCCACTACTGGATAGCATATATAATCAAGATTATTGTTTAAAGAATAATGATTTTGAGATCATTGTTTGTG
AAGATAAATCTCCACAGAGAGATGAGATAAACTCTATTATCGAAAACTATAAAGCAAAAAATAATAAACAAAATCTTTATGTTAATTTCAATGAAGATAATTTAGGCTATGATAAGAATTTAAAA
AAATGCATTAGTTTGACGACAGGTAAATATTGCATGATCATGGGCAACGATGATCTATTAGCAGATGGAGCGTTATCAAAAATAGTGAAAGTTTTGAAGGCTAATCCTGAAATTGTATTGGCTA
CGCGAGCGTATGGTTGGTTTAAGGAAAATCCGAATGAGTTATGTGATACTGTTCGTCATTTAACAGACGATACTTTATTTCAGCCGGGGGCTGATGCCATTAAATTTTTCTTCCGTAGAGTTGG
AGTTATTTCAGGCTTTATTGTCAATGCTGAAAAAGCAAAAAAACTATCGAGTGATTTATTTGATGGGCGTTTATATTATCAAATGTACCTTGCTGGTATGCTAATGGCTGAAGGTCAGGGATACT
ATTTTAGCGACGTGATGACATTGTCGAGGGATACAGAGGCTCCTGACTTTGGTAACGCTGGAACTGAAAAAGGAGTTTTCACCCCGGGGGGGTATAAACCAGAGGGCCGTATACATATGGT
TGAAGGCTTGTTGCTAATTGCAAAATATATAGAAGATACAACAAAAATTGATGGCGTTTATGCTGGAATTAGAAAAGACTTAGCGAACTATTTTTATCCTTATATTCGAGATCAACTCGACTTGC
CTCTTTATACTTATATTAAAATGATAAATAAATTTCGGAAAATGGGATTTTCAAATGAAAAGCTTTTCTATGTGCATGCCTTTTTAGGGTATGTACTAAAACGGAGGGGCTATGATGCTTTAATTA
AATACATTCGTAGCAAAAAAGGCGGTACTCCGCGTCTTGGTATTTAA
>BDIOMP_08480 O-antigen polymerase
ATGCTGCCATTTCCACCAGGAGCAATCCTAAGGGATGTACTCAATGTATTTTTTGTGGCGTTAGTGCTAGTTCGATTTGTCATTGATAGGAAAAAAACTTATTTCCCGTTGGTTTTTACTATTTT
TTCATGGTCGGCGGTAATACTATGGGTAATAGCGTTAACTATATTCTCACCGGATAAAATTCAAGCAATTATGGGGGGGCGGAGTTATATTTTATTCCCGGCAGTTTTCATAGCATTAGTGATTT
TAAAAGTATCATACCCGCAATCCTTAAATATTGAAAAAATAGTTTGCTACATAATTTTTCTAATGTTTATGGTTGCGACAATATCTATTATTGATGTACTAATGAATGGAGAGTTCATTAAATTGCTC
GGATATGATGAGCATTATGCAGGAGAACAATTAAACTTAATTAATAGCTATGATGGGATGGTCCGGGCTACAGGCGGTTTTAGTGATGCTCTCAATTTTGGATATATGCTCACATTAGGTGTTT
TGTTATGTATGGAGTGTTTTTCCCAAGGATATAAAAGATTATTGATGCTCATTATTAGTTTTGTGCTATTTATAGCGATCTGCATGAGTCTTACTAGAGGAGCAATACTTGTTGCTGCGCTTATTTA
CGCACTTTATATAATTTCAAATCGGAAGATGCTTTTTTGTGGAATAACTTTATTTGTAATAATTATACCCGTTTTAGCAATTTCTACTAATATTTTTGACAACTATACAGAAATTTTGATCGGCAGG
TTTACAGATTCGTCTCAGGCATCGCGTGGATCTACACAGGGGCGGATAGATATGGCAATTAATTCATTAAACTTCCTGTCAGAACATCCATCAGGTATAGGTCTGGGTACTCAAGGTTCAGGA
AACATGCTTTCGGTAAAAGATAATAGGTTAAATACGGATAATTATTTTTTCTGGATCGCCCTTGAGACTGGTATTATTGGCTTAATCATAAATATTATTTATCTGGCAAGTCAATTTTATTCTTCAA
CTTTACTAAATAGAATATATGGCAGTCATTGTAGCAATATGCACTATAGATTATATTTTCTCTTTGGAAGTATATATTTTATAAGTGCAGCGTTAAGTTCAGCACCTTCGTCATCAACTTTTTCTATA
TATTATTGGACAGTTTTAGCTTTGATTCCATTTTTAAAATTAACAAATAGACGGTGCACGCGATAA

Region of unknown function 2

>BDIOMP_17535 SymE-toxin domain-containing protein
ATGAATGCCTCCGGCGTCAGAGTCCGCCACATCAACAGCGAAACCTGCATGACCACCAGTTACTCACATATCCCCAGCCAGCACCTTAAGGGTGACTGGCAGGAAGAAGCGGGATTTGAG
ACCGGGCGCAGCGTGACCGTGAAGATCTCCGAGGGATGTTTGATCCTGATGGCTGAGACGGACGAAGTGAGGGAGCTACGGAAGGAGCTTTATCAGGTGAAAAAGTCGATGAAGCATAT
TAAGGCGGGGGTAAATGATGTGGTGAACGGGGATTAA
>BDIOMP_17540 isrA Hfq binding RNA
ATCTTCACGGTCACGCTGCGCCCGGTCTCAAATCCCGCTTCTTCCTGCCAGTCACCCTTAAGGTGCTGGCTGGGGATATGTGAGTAACTGGTGGTCATGCAGGTTTCGCTGTTGATGTGGCG
GACTCTG
>BDIOMP_17545 Phosphotriesterase
ATGAGTGATTACAAATTTTGGGGCTGGGATAAGAAGCCGAGAACAATGCTGCGTTTTGTGAAGCCCGGAGACATATTTTGTTTTAAATTAGATGAAGATAGATATTGTTTTGGGCGAATTATA
ACACTAATGACTGTCGGACATCTTTCTGAATTATTTGATATAATTAAAAAATCCCCTGGAATAACAGAGTTAGAAATTAGTAATGCAAGGCGAATTATTGAACCAATTATAGTGGATACATATTCT
TTATTTGATAAGAAATTAGAAAATGGAAGTGACTGGAGAATTATTGGTCATCAGGATAATTACAATCCAAAAAAATTTAGATGGTATCTATTTTGCACTTGGAATAGGTGATTCCTGTAAAAAGA
AAGACTGTTACGGAAATGATTTTCTCATTTCAGAAAGTGAGTGGAAAAACACTTCCTAAATTATCTCCTAAAGGGGATTTTGATATCAAAAAACGGCTTGAAATTGCCTGA
>BDIOMP_17550 Cytoplasmic protein
ATGTTAAATTCAAATATGTCTGAACTTAGAATCGAACTGGAGAATGCGATTAAAAATCTCGGTATTCATGATTATCGTGTCGATAAACCCGAACAAATCGTTTCTGAGATAAAAGAGATATATGT
TAATGGTAATCCTAGAACCTGGTGGTTATCATTAAAACATAGACAATATGTCTTTTCTTATACCGATAATTCTGGATATAAAAACATATCACAAATAGTAAGTAAACAACTCAATGAAAGCAATGT
AATCAACAAACATATATTTTTGATTGCTGATGAAGATAATGAGCAAATATATGTATATAACGTTCCTCTTAACTCCCTGCCTGAAATTATAGAAAATTGCAGATATTTTGAATATTATGTTGCAGAT
CATGAACTATCTTGGCTTATATGTGAAAATGATCATGGTGATTTGATTGTATGCTCAACCATTAAGTAA
>BDIOMP_17555 hypothetical protein
ATGCGGATGTTGTGGGCCATCATAAGTAATATTACCTCTATTAGCTCCTCCGGAACCTCTTTTTCCTGCGGATTGCCAACCAACATGTGGTTTATCATGCCCTGCTGTCACATCCATATCTGCATG
AGGTGCATCATAAGCAACTTTAGCCCCATTTGGACCTTTAAACTCAACAACAGTACCAGTTTTGGGATCGGCTTTCGAGAAAGTGACATCCTCAGGGTTTGTCATACCCGCATTTTCAAATCC
GGTTCTCCTTGCCGCATCAAAATTAGATTGATGCTCCAGGATAGCAAGTCCTAA
>BDIOMP_17560 SHQ1 domain-containing protein
ATGTTCAAAATAGAAGTTGATGATACTTACGAAAAAGGACTTATGGATGAAGAGGATACTAGTTTGTCGGAAGCTATATTTTCCACATATCCCATATCATCCGGATATTTTTCTATAAACTGGAAT
GGCATTTATATCCCGCTAACACTTAATAGCCTGAGTGATATCATTGATGATATTATTAAAATGCTTGATAGTATCATCTCCGGGTTTGACTTTGAATGCAGTTTTTTATGTGAGTCTTTTTCGGTGA
TATTGAATTTTAAAATATATAAAAAGAATGTTATAATTACTTCAAAATGGATTTCCATAAACACAGATGAAATTTTTAATTTAAATTCAAGCAAGAGTGTATTGCTTATAAATAAGGATAAATATAT
CTGTGAATGGGTGAGATTATTATCTGTCATTAGTAATGATCTAAAATTAGTTGGGTATAGCTTTTTATTTTTAGATAAAAAAATTATCTTTTTATAATGAATTTTTGAAACAGAAAGACAATATGTGA
>BDIOMP_17565 hypothetical protein
GTGACGGATTATTTTTACGACACGACGGGGCGCATCACGGCGTGCCGGAATGAGGCATACCTGGACAGCTGGCAGTACGACGCGGCGGCGAACCTGCTGGACAGGCGGCAGGGAGAGA
CCGCGCAGGCGGGTGCAGGCAGCGTGGTGCCGTTCAACCGGATAACGTCATACCGTGGGCTGCATTACCGTTACGATGAATATGGCCGGGTGGTGGAAAAGCGGGGCCGCAACGGTACG
CAGCACTATCGCTGGGATGCGGAGCACCGGCTGACGGAAGTGGCGGTCATCCGGGGGAGCACCGTACGGCGTTACGGGTACGTGTACGACGCGCCGGGCAGGCGGGTGGAGAAGCAC
GAACTGGACGCGGAAGGAAAGCCGTATAACCGGACGACGTTTTTATGGGACGGAATGCGGCTGGCGCAGGAGTGCAGGCTGGGAAGAAGCAGCAGCCTGTATATCTACAGCGACCAGG

GGAGCCACGAGCCGCTGGCGCGGGTGGACAGGGCGGCGCCGGGCGAAGCGGATGAGGTGCTGTATTACCATACGGACGTAAACGGCGCGCCGGAGGAGATGACGGACGGCGGGGGC
AATATTGTCTGGGAAGCGGGCTATCAGGTATGGGGGAACCTGACGCATGAAAAAGAAACCCGGCCCGTACAGCAGAACCTGCGTTTCCAGGGGCAATATCTGGACAGGGAAACGGGGCT
GCATTACAATTTATACAGATTTTATGATCCGGATATCGGGAAGTTTATATCGGGCGACCCTATTTCGATCCGGGGCGGGATAAATCTCTATCAGTATGCGCCTAATCCTCTCTCTTGGATCGATCC
TCTTGGATTAAGTACCACTTGTTTAAAAGCCGAGAATGGGTACTCAAGAGGGAAAAGACATGGGATGAAGTTACATCCTAATGCAGCTCAAGCAATTGCGGATAAAGAAAACAAACCTCAT
GGAGTTTGGAGAAGTAAGGATGATCTGAAGTATGCTGGAGAACAAGCTGCCACACTAAAACCGGGGGAGATGAAAGATTTTCCTATAAATCCAAATAGTAAGAGTGAAGTTTATATTCCCG
GAGGCAATGGAGTCCCTATTAAACCGGATAAAATTAGAGTCAGAAATAATGGCGATGGGACTTTTCACGGTTTTCCAATTGACTCAACGACGGCAGGACCCATTTTTGAAAAGGGCAAATG
A
>BDIOMP_17570 isrA Hfq binding RNA
ATCTTCACCGTCACGCCGCGCCCGGTCTTAAATCCCGCTTCTTCCAGCCAGTCGCCCTTAAGATGCAGCTCGGGAATATGCGTACAGCGGCCAGGATGAGCTGCTGAAAAAGCGGCACAG
>BDIOMP_17575 hypothetical protein
ATGAATACTAAAGAAATAAATACTAGAGAAGATTTTATTAAATTTCTTGAAATTTTATCTTCAAATGCCAGAAATAATCTAAATGAGTGGGAGAATAAAGACTTGCCCTCATATTTTGAATCGAT
GGCATCTTGGGTTGAAGATATGGATGGATATTATTTAAATCAAAAATTACCTGCTCCAGAAAATGTTAACTGGACATTTATTGCGGATATTTTAATGGCTGCAAGAGTTTATGAATAA
>BDIOMP_17580 DUF6531 domain-containing protein
ATGGGAGAAGCATTCTGGGCGGCAAGGGAAGGCGACGCGCTGCTGCATACGTCCTTTCTGGCAGATCTGGTGGGCAGTGCGCTGGAATTCGCCATCAATGCGGTTATTGATTTTGCGGCA
CTGGCTGTTGTGGCGCTGGCGACGGGGGCTACGGTGGCAACGCTGGGATGCAGCGCCGTATTACTTGTGGGGACCGTGGTCGGCGCGACGATGTTGCTGAGTGGCGCCGGGGAGAAAA
TCAGTAAAGCCTGTGAAGACATAGCCAACAGCCTTTTTCCGCCGAAAATTGAGGGATATATCCTGACCGGCTCAGGCGACACCCGGATTAACAGCAAACGGGCGGCGCGGGCGGCGGCA
ACGGCATTATCACGCCATGATGTGGAAACCCTGGATGCGCAGGCGCAGGCAGAGGCAGAAGAAGAAAAACGCAGGCAGGACGCCAAATCAATGTGGGATGTGGCCGGGGAATATCTGG
ATGCAGTAAAAGGCCGGGCAGAGAATATGGCTAAGGGCGTCGTTTCCGTGGTGAGCCGGATGGGATCAGCGGAAGGATGGGCCTCGCTGGGAAATGATGCTCTGGCAGAGGCGGGCGA
TATGCTTTCCGACACGGGCCACTTTATTTCAGAGATGTGGCAGCCCACGGTAGCAACGGCATATCCGGGGTCTGACCCGAAACAGGATGATAAAATCGACTGCCACAAACATCCCTCTTCAT
TCACCCAGTTTATGGCGCAGAAACTGCAGGCGCTGACGGATGACCCGGTGGGGACGGTACTGGGGGCGATGAACACTTTCGATGTGCTGGAAACCGGTTTTCAGGCGGCAAGCGCGCTT
ATCGGCAGCGTGTCGAACCTGTTCAAAGGCGACGATGAGCCGCCCGCAGCAGAATATATCGCCGAAGGGACGCGGGATGTGCGTATCAACAGCCAGCCTGCGGCGCGCAGCGGGGTACG
CTGTACCTGCGAGGCAAAAGTGGTGGATGAGCCTGAGAACGGCGTACATGTATCCGGTGATGTACGTATCGGCGGGCCGCTTCTGGTGGTACGGGATATCAAAAGCGGTAAGTCACAAATA
ACCCTGGTGACCACCATTGCGCTGACGTTCATGCAGCCCGGACGGGCGTTAGCTAAAAGTGCCTGCTTTATGATGGGATTGGGCATTAACATGATGGTTCAGAAGGCGGGGAGTGCGCTG
AACCGCCCGGTAAACGCCGCCACCGGAGCCAAGTACCTGGCAGGCGATGATGACGTTGATTTCAGTCTGCCCGGCCACTTCCCGCTGGAGTGGCAGCGGACCTACAGCAGCCGTGATGAA
CGGACGGAGGGGATGTTCGGGCGGGGCTGGAGCGTGCTGTATGAAGTGTGCCTGGAGCGTACGCCGGACAACCCGGATGAAAACTGCATGACGTATGTATCCCCAATGGGACGGCGAAT
TGACCTGCAGGCGGTGGAGCTGGGGAGCGGTTTTTACAGCCCCGGCGAGGGGCTGGCGGTACGGCGCAGCGAACAGGGCCACTGGCTTATCAGCAGTGATGACGGCGTGTACAGGCTG
TTTGAAGCGGACCCGTCCAGCCCACAGCGCCGGCGGCTGAAAATGCTGGGCGACCGCAACAGTAACTGCCAGCACCTGACCTACGACAACCACGGGCGTCTGGTGGAAATCAGCGGCGA
CCGGCAGCGCCCCTGCATCCGGCTGCACTATGAGCTGGCAGCGCACCCGCAGCGCGTGACGCGCATCTTCCGCCATTACCCGGAAGGGGAGCCGGAGCTGCTGCGGTGTTACCGTTACGA
TGAGGCGGGGCGGCTGAACGGGGTGGTGGACAACGCAGGTCAGTATCAGCGTGAGTTTGCGTATGACGACAACGACTGCATGACGATGCACCGGGAGCCGGGCGGCGAACGGTATTAC
TACACCTGGGCGTGGTTTGAAGGCCCGGACGATGCGGCGTGGAGGGTGACGGGCCATCATACGGACAGCGGCGAGCAGTACCGTCTGGACTGGAATCTGGCAGAACGTTCGCTGTGCGT
GACGGATAGTCTGGGGCGTACGCGCTGCCACTGGTGGAATGCGCAGGGCCTGGTGACGGCGTACCGGGACGAGGCCGGGCAGATGACCACTTTCCGCTGGAGCGATGAAGAGCGGTTA
CTGCTGGGGATGATGGACGCGCAGGGCGGCAAATGGCGTTATGTCTATGACCGTCTCGGCCACCTGACGGAGACGCATGACCCGCTGGGCCGGGTTGAGCAGACGCAGTGGCACCCGGT
GTGGCACCAGCCGGAAACGGAGGTGGATGCCGCGGGGGCGGCGTGGCGTTATGAGTATGATGAGCGGGGCAACCTGCAGGCGGTCAGCGACCCGCTGCACCAGCGCACGGTATACGG
GTACGACCGACACGGTCAGGTGGTGCGGATAACCGACGCGCGGGGCGGAGATAAATACCTGCAGTGGAACGAAGACGGGCAGCTTATGCGCCACACGGACTGTTCTGGCTCGCAGACG
GCATGGTTTTATGATGAACGCACGCGGCTGGAAAGGGTGACGGACGCGGAGAGTAACAGTACGCGTTACAGCTATGACGGCAACGGACATCTGACGGAGGTCATGTTCGCGGACGGGCG
TACGGAGCGTTACCAGCCGGATGCGGCGGGACGGCTGGTGAAATACACCAGCCCGGCGGGGCAGATAACACGCTGGCAGCGGGACGGCCAGGGGCGGGTGCGCAGGCAGACGGATGC
GACGGGTCGCAGGACGGCGTATGAGTACGACGCTTACGGGCGGCTGACCACGCTCACGAACGAGAACGGGGAAAGCTACCGGTTCCGGTACGATGTTCTGGACCGGGTGACGGAACAG
ACGGACCCCGGCGGCAGCCGCCGGGCATACGGGTATAACGCGCTGAATGCGGTGACGGCGGTGATATACGGCGGGGAGCGCGGGGGAGAAATCCGTCACGGTCTGGAGCGTGATGCGG
CGGGGAGGCTGACGGCGAAAATCACGCCGGAGACGTGCACGGAATACCGGTACGACGCGGCGGACCGTCTGCTGGAAATCCGCCGCAGGCGGCATGATGCGGCGGAAGGCGGAGAGC
CGGAAGTTATCCGGTTCAGCTACGACAGTGCGGGTAACCTGCTGAGCGAGGAGACGGCGCAGGGCGTGCTGCAGAACCGGTACGATGTTCAGGGCAACCGCACAGAAACGCAGATGCC
GGATGGGCGGACGCTGCGGTACCTGTACTACGGGAGCGGCCATCTCCAGCAAATCAACCTGGGGCGTGATGTCATCAGCGAGTTCACGCGCGACCACCTGCACCGTGAGGTGCAGCGGA
GCCAGGGGCGGCTGGACACGCGGCGGATGTACGACCGGACGGGCCGGTTAACGCGGAAACTGACCTGTAAAGGAATGCGCGGTGTGGTGCCGGAGACGTTTATCGACCGGGAATATGC
GTACAGCGGCCAGGATGAGCTGCTGAAAAAGCGGCACAGCCGGCAGGGGGTGACGGATTATTTTTACGACACGACGGGGCGTATCACGGCGTGCCGGAATGAGGCATACCTGGACAGCT
GGCAGTACGACGCGGCGGCGAACCTGCTGGACAGGCGGCAGGGAGAGACCGCGCAGGCGGGTGCAGGCAGCGTGGTGCCGTTCAACCGGATAACGTCATACCGTGGGCTGCATTACCG
TTACGATGAATATGGCCGGGTGGTTGAAAAGCGGGGCCGCAACGGTACGCAGCACTATCGCTGGGACGCGGAGCACCGGCTGACGGAAGTGGCGGTCATCCGGGGGAGCACCGTACGG
CGTTACGGGTACGTGTACGACGCGCCGGGCAGGCGGGTGGAGAAGCACGAACTGGACGCGGAAGGAAAGCCGTATAACCGGACGACGTTTTTATGGGACGGAATGCGGCTGGCGCAG
GAGTGCAGGCTGGGAAGAAGCAGCAGCCTGTATATCTACAGCGACCAGGGGAGCCACGAGCCGCTGGCGCGGGTGGACAGGGCGGCGCCGGGCGAAGCGGATGAGGTGCTGTATTAC
CACACGGACGTAAACGGCGCGCCGGAGGAGATGACGGACGGCAGGGGCAATATTGTCTGGGAAGCGGGCTATCAGGTATGGGGGAACCTGACGCATGAAAAAGAAACCCGGCCCGTAC
AGCAGAACCTGCGTTTCCAGGGGCAATATCTGGACAGGGAAACGGGGCTGCATTACAATTTGTACAGATTTTATGATCCGGATATCGGGAAGTTTATATCGGGCGACCCTATTTCGATCCGG
GGCGGGATAAATCTTTATCAGTATGCGCCTAATCCGATAAGCTGGATCGATCCACTTGGGCTTACACCTTGTGGGAAACCTGACGGGAATATTAATCCAAAAGATGTTCGATATATGCAAAGTT
CTATAAAAAACCAAACGGGGGAATATACTGTGTTAGGTAATGCAGAGGCTTTGCTTAATGGTACTTTGAAACCATCTGCTCTACCGCCAATTAAAATATGGAAGGATAATCTTGGTAAAATATG
GACGCTGGATCATCGTAGATTAGCTGCATTCAAATTAGCAGGGCTTGATAGTGTTCCATTTAAATGGGCCTCTCCTAATGAAGTTGCTAATCAGATGTGGAAAATGACAACAAAAACAGATGG
TAAGTCGATTATTCTCAAATTGGGTGATGGAATAAAAAAAGTACTGGAGTAA

Region of unknown function 3

>CFICBL_23240 Integrase
ATGGCTCTAACCGATATACAAATCAAACGCGCAAAACCACAAGACAAACCATATACAATGAATGATGGGCACGGTTTGTCACTACTCATCAAGCCAGACGGTTCAAAGGGCTGGCGCTTCCG
CTTTCGTTTCGCCGGTAAAGCACGGCTAATGTCATTTGGTAGTTACGAACTTGTTAGCCTCGCAGAAGCACGAGAGAAACGTGATACTGCACGTAAGCAGGTAGCAAACGGCATAGACCCA
GTAGAAGAACGGAAGGCTCAGAAACTTGCTCAAAAATTATCAACTGAAAATTCTTTTGAGTCAGTTAGCAGAGAATGGCATGCAGCAAAAGCAGATCGCTGGACTATCGCTTATCGGGAGG
AAATTATTAAAACATTCGAACAGGATGTTTTTCCTTTCATTGGTAAACGCCCAATATCTGAAATTAAGCCATTAGAACTGCTTGAAGTACTTAAGCGCATCGAAAAACGTGGAGCGTTGGAGA
AAACCAGAAAAGTTCGCCAACGATGTGGAGAAGTGTTTCGCTACGCGATCATCACTGGCCGTGCTGAATACAATCCGGCGCCTGATTTAGCAATCGCTTTGGCTATTCCCAAGCAAAAGCAT
CATCCGTTTTTATCCGCGGAAGAGCTACCTCATTTTGTTAGAGATCTAGAGTCATATACTGGAAGTATTATTACCAAAAACGCAACCAAGATCCTAATGATGACAGGAGTCAGGACGCAGGAA
ATGCGTTTTGCAACTTGGAGTGAAATTGATTTGATAAAAGGTATCTGGGAAATACCTGCTGAACGGATGAAAATGCGTCGGCCACATATAGTACCTTTATCATCTCAGGTAATTGAACTGTTTA
AACAACTACAACCGATAACATCGGACTATCCCTATGTTTTTATTGGCAGAAATGATCGTAGAAAACCGATAAGCAAAGAAAGTGTAAACCAAGTTATTGAATCATTAGGTTATAAGGGAAGAA
CTACTGGTCATGGTTTTAGGCATACACTATCGACAATTCTTCATGAAATGGGATTTGAGAGCGCATGGATTGAGATTCAGCTCGCACATGTTGATAAGAATACTATCCGAGGCACATATAATCAT
GCTTTGTATCTTGATAAAAGAAAATATATGATGCAAAAATACTCGGATTTTTTGATGAATTTATCTACCGGAGTTCAAAATGATTGA
>CFICBL_23245 UvrD-C-2 domain-containing protein

ATGATTGAAATATTGGGAATGAAACCTCAGAATCATACTGTTTCTGATATTCTAGAAAATTCAATAAGAAAGATAAATGATATAACTGGATATTATTATCGCGGTTTTCCTGTTATTTTTCTGAGC
GAAAACAATGTTACTCTTGATGGTCTAATTTGCTCATCAAAGCATGGTGTTGTCATTTTCCATTTCAATGAGGAAAGAGAGTTAACCCAAGATGATATTGATATCATTGACGAAATGCACATGA
AATTCGTCGCAAAACTTTCTGAAGTAAAAGCACTGACTAAAAACAGAATATTAAACGTACCTGTGAACAGTATTGTTTTCGCACCAAACTGTATAAATATTGATGATTTTGATTGTGACAGTGG
TTTAAGCATTAGTTCCAATGTTGATGAAATATTATCTATTCTCAATGACATTACCTGGGATGATGGTTCCCTTCTTGAAACTTTACTATCCCATGTTCAATCACTTTCAAAATTGAAGCCCAAGAG
AAAAAGAAGCTATGTCAGACAGATTGACTCAAAGGGTAGCGTACTAAAAAAATTGGAGAATTCACTTGCCACTCTTGATTCTAGTCAAACGACTGCCGTCTTGGTCAATATTGATAATGTCCA
AAGGATTCGTGGGCTTGCTGGCTCAGGTAAGACTATTGTTCTCGCTCGAAAAATTGCACATATACACTCACAAAATCCTCACTGGAAAATAGCAGTTACTTTCAATAGTCGCTCTCTCAAAGA
GCAACTAAAACATTTAGTCTCAGCATTCGCAGATGATAATGGAATTGATTGGGATAACGTAGACATTATTCATGCCTGGGGCGGCTCTAAAACAGAAGGTTTATATTACAACGCATGTGTGGC
ACATAATATACCTTATTACGATTATAACGGCGCACGTCGTCTTGCAGAATTTAATGAAACACCTTTTCAGGCTGCCTGTAAGAACTTCCTCGATAATAAAAAAGAAGATAAGAAATTATATGATT
TCATCCTTATTGATGAGGCTCAAGATTTTTCAATTGAGTTTCTCCGCATATGTTATTCTTTGCTAGGGGAAAAGAAACGATTAGTTTACGCTTATGATGAATTACAAAATCTTGGGGATTCATCC
ATGCCTTCTCCTGAACAAATTTGGGGAGTAGACGAGCATGGAAAACCAATTGTTAGTTTCACCAGTGAAGAACAAGATATAACTTTAGACATTTGTTATCGAAATCCAGGCCCAATCTTAACC
ACGGCTCATGCTTTAGGGTTTGGTATTTATCATAAACCAATAATTCAGATGTTTGATTTTGAAGGATTATGGACAGAGATTGGTTATGAAAAAATAGATGGTGAATTAGTTGAGGGAAAGGAG
GTTATATTATCAAGGACAACGAAATCTAGCCCATCCCTATTGAGTGCACATAACTCAATTGATGAGATGATCCAGTTTGTTAAATTTGAAGACAAGCATTCTCAATCTAATTGGATTGCGCAGG
AAATATTTAAAAATATTCATGAGGAAGAGATATTACCTAGCGACATTGTTGTAATTCACCCAGATACAATACGTATGAGAAATGAAGTAGGTTATCTGCGTGACCTTTTATTCCAAAACGGAATT
AATACATCTATAGCTGGAATTACAAGCTCTCCTGATGAGTTTTTCTCAGACAATAGTGTAACATTTACATCCATATTCAGAGCAAAGGGAAACGAAGCAGCTATGGTATACATCATGGATGCTCA
TTATTGCAACGTGGATTATGAACTGGCAAAGCGTAGAAATATCCTTTTTACTGCAATGACAAGAACTAAAGCATGGCTTCGTGTATGTGGTGTGGGTTCTTCCTTTGATGGCCTGATTAACGA
ATACAATGAGGTGAAACAAAGAGGATTTAAACTGGAATTTACATACCCAACAGAAGCAGAGCGTAAAAAAATGCGACTGGTGAACAGGGATATGACTTCTCAAGAGAGAAAAAGAGTACA
CCAAGCTAAATTGAACGCCCATAATCTCATGGTTCTATTGGATGGACAAGTTAGAGTTGAAGATATACCAGAAGAATTACGCCTTGCTTTAATTAAGCAACTGAAGGGTGAATGA
>CFICBL_23250 DUF2290 domain-containing protein
ATGAACTCAAGTAGCATTTGCGCAGCAATTAGAAAAATCACATCTCATCTGATATCAGTGGGTATCAGTGACGCACAAAACTATCCTGTAATAACACGGGATAAAGAATATTATTATGTCAGCTA
TAGCGGATTCGCTGATACTTCAATTGCATTAAGAAATATTGAGTATGTTGATATCTATAACTTCTTGGACGAAAAAAGGCAATATAATATCAAATTATTAGATGGTGGGTTACTCCATCTTTTATTT
CAGTTTGATCGTCGAATGAAACTTGTAAAAGAAAGACTATGTTATTTCCCCGCGCCTAATTACGAATCATTCCAGAATGACCCTGAGTTATATTTAGACGAAAGCAACTTTTATGCTGACATTG
TCCAAAAATCAATTTTACCCGTTCCGATACGAGTGGATTATGCCCCTGATGACGCTAATGATGTTATTCATCCAGCAGCACACCTAACCCTAGGTCAGTTTAAAAATTGCCGGATACCATTGTCG
TCACCATTATGTCCGGTAACGTTTGTGAAATTTATTTTAAGCTCTTTTTATAATACTGCTTATCATGATTTTGGTTTTGAAATGGAACGGTTAGTTCATCCCAACACCATTACACCGAGAGAGAG
AGGAATGTTACATTTTTCAATTGAGTAA
>CFICBL_23255 Polarity suppression protein
ATGGAAAGCACAGCCTTACAGCAGGCCTTTGACACCTGTCAGAATAACAAAGCAGCATGGCTGCAACGCAAAAATGAGCTGGCAGCGGCCGAACAGGAATATCTGCGGCTTCTGTCAGGA
GAAGGCAGAAACGTCAGTCGCCTGGACGAATTACGCAATATTATCGAAGTCAGAAAATGGCAGGTGAATCAGGCCGCTGGTCGTTATATTCGTTCGCATGAAGCCGTTCAGCACATCAGCAT
CCGCGACCGGCTGAATGATTTTATGCAGCAGCACGGCACAGCACTGGCGGCGGCACTGGCACCGGAGCTGATGGGCTACAGTGAGCTGACGGCCATTGCCCGAAACTGTGCCATACAGC
GTGCCACAGATGCCCTGCGTGAAGCCCTCCTGTCCTGGCTTGCGAAGGGTGAAAAAATTAATTATTCCGCACAGGATAGTGACATTTTAACGACCATCGGATTCAGGCCTGACGCGGCTTCG
GTGGATGACAGCCGTGAAAAATTCACCCCTGCGCAGAACATGATTTTTTCGCGTAAAAGTGCGCAACTGGCATCACGTCAGTCTGTGTAA
>CFICBL_23260 Transactivation protein
ATGATTTACTGCCCGTCGTGTGGACATGTTGCTCACACCCGTCGCGCACATTTCATGGACGATGGCACCAAGATAATGATTGCACAGTGCCGGAATATTTATTGCTCTGCGACATTTGAAGCG
AGTGAAAGCTTTTTCTCTGACTGTAAAGATTCAGGAATGGAATACATTTCAGGCAAACAGAGATACCGCGATTCACTGACGTCAGCCTCCGGCAGTATGAAACGCCCGAAAAGAATGCTTG
TTACCGGATATTGTTGTCGGAGATGTAAAGGCCTTGCACTGTCAAGAACATCGCGGCGTCTGTCTCAGGAAGTCACCGAGCGTTTTTATGTGTGCACGGATCCGGGCTGTGGTCTGGTGTTT
AAAACGCTTCAGACCATCAACCGTTTCATTGTCCGCCCGGTCACGCCGGACGAACTGGCAGAAAGCCTGCATGAAAAACAGGAACTGCCGCCAGTACGGTTAAAAACACAATCATATTCGC
TGCGTCTGGAATGA
>CFICBL_23265 Glycoprotein 3
ATGTCTGACAACACCATCCCTGAATATCTGCAACCCGCGCTGGCACAACTGGAAAAGGCCAGAGCCGCCCATCTTGAGAACGCCCGCCTGATGGATGAGACCGTCACGGCCATTGAACGG
GCAGAGCAGGAAAAAAATGCGCTGGCGCAGGCCGACGGAAACGACGCTGACGACTGGCGCACGGCCTTTCGTGCAGCCGGTGGTGTCCTTAGCGACGAGCTGAAACAGCGCCACATTG
AGCGCGTGGCACGCCGGGAGCTGGTACAGGAATATGACAATCTGGCCGTGGTGCTGAATTTCGAACGTGAACGCCTGAAAGGGGCGTGTGACAGCACGGCCACCGCCTACCGGAAGGC
ACATCATCACCTTCTGAGTCTGTATGCAGAGCATGAGCTGGAACACGCCCTGAATGAAACCTGTGAGGCGCTTGTCCGGGCAATGCATCTGAGTATCCTGGTACAGGAAAATCCGCTCGCCA
ACACCACCGGCCATCAGGGCTACGTCGCACCCGATAAGGCTGTCATGCAGCAGGTGAAATCATCGCTGGAACAGAAAATTAAACAGATGCAAATCAGCCTCACCGGCGAGCCGGTTCTCCG
GCTGGCCGGACTGTCAGCGGCAACACTCCCGCACATGGATTATGAGGTGGCAGGCACACCGGCACAGCGCAAGGTGTGGCAGGACAAAATAGACCAGCAGGGAGCAGAGCTTAAGGCC
AGAGGACTGCTGTCATGA
>CFICBL_23270 putative transcriptional regulator
ATGCAAGCTGTTTTTTCTTCCCCGTCTCCCGCCCCAGTGACGCCACTGATGCCGCTGCCGGACATCACGCAGGAGCGTTTTTTACGTCTGCCGGAAGTGATGCACCTGTGCGGCCTGTCACG
CTCGACCATCTATGAACTCATCCGTAAGGGGGAATTTCCGCCGCAGGTGAGTCTTGGCGGTAAAAATGTGGCCTGGCTGCACTCTGAAGTCACCGCATGGATGGCCGGGCGCATTGCCGGA
CGCAAACGGGGGTACGACGCATGA
>CFICBL_23275 isrK Hfq binding RNA
GCTGTCGCAAAATCGGCAGCCGGGCGTAGGAACCCGTGTAACTCGAAGGCGACATATGACGCGCCATGCGTCTTTTTT
>CFICBL_23280 Protein ash
ATGGTGTGGCGTGTGGTTTGCCGTGCAGGTATGATCCTGTTCGCAATCGCATGTTATGCCACTGAGTCAATGGTAGCTCAGGCGGGGCAGCCTCCGGGCTGGCCGGTATTCTTCGAGGCCG
GTATTCCTACCCCCGTCTGGGCTATCGCCATCGAGCGTAGGAACTCCGGCGATAGCAGTTATTTGCTACTCGAAGGAGACGGCCTTATGGCTACAACCCTTACCCCCTCACACCCTGAATTTGT
CTTTGTGTTTGCGGCTGTCCGTCGCGCAGACCGTCATCCCCGTATCTGTATGCTTCGCACCGTCGCCGGTGATGAACGCAGTGCCCGCCGTTCCCTTGTCCGTGACTATGTGCTCTCCCTTGCT
GCCCGTCTGCCGGTGGTGGAGGTGTCCCGTGCGTAA
>CFICBL_23285 C4 antisense RNA
GTCAATGGTAGCTCAGGCGGGGCAGCCTCCGGGCTGGCCGGTATTCTTCGAGGCCGGTATTCCTACCCCCGTCTGGGCTATCGCCATC
>CFICBL_23290 Derepression protein
GTGCGTAATAAAAAAGCCCCTCAGACCGTCTCAGCGCGTCATGACGCCCGTGAACACCTCAGCATTGAGGCTTACCATAAGCTCAACCGCGCCAGCGCCGTATCCCAGTTTGTTGGGGGTG
ATTTGATTCACCGTGAACTCTCCGGCCTGCATCAGCTCTACATTCCGCATATTTTCAGCTACCTGAATGAAGATATTGATTTTGTGCTGAATGAGCTGAAAGCCAAAGGCCTGTGCCGCGATTT
TCTCGCCCAGCAGAAAGACCGGGGAGACAGGACGCATGTTTGA
>CFICBL_23295 Uncharacterized protein ORF151
ATGTTTGATTTTCCCCAGCCCGGTGAGATTTACCGTTCTGCCGGTTTTCCCGATGTGGCCGTGGTCGGCATTCTGGAAGACGGTATTCCGTGGGAAATGCCGTACCGCTGCCCGGACATTGT
CTGGAACCCGTACCGCCGTAAATTCAGTATCCTTGTGCGTATCCTCGCTGACGGGCGCACCACAGACATCCCGCTGGGGCGTTTTCTGCGGGAATTTACCTGTGACCGTCCTGACCTGTTTAA
ACGCAGCCCCGTAAACCGGCATGCGGTACTGAAAGAAATGGCCGGAGACCCGGAATTACAGAAATGGCGGGAGAAATATCTGGATATTTACCCGCAGGACACTGTTCCGGCCAGCCGGGC
GGCACCGGTGGCGCGGGAATGGCGGGAAATTCCCCGCACGGAGCCTGACCCGGAAATCACCCCGGATAACAGTTACCGCAATTATCTGTAA
>CFICBL_23300 Uncharacterized protein ORF106
ATGAAAACACCCTTACCGCCCGTCTTACGCGCAGCCCTTTACCGTCGCGCTGTCGCCTGTGCCTGGCTGACCGTGTGCGAACGTCAGCACCGCTACCCGCATCTCACCCTTGAATCACTGGA
GGCGGCCATCGCCGCTGAGCTGGAAGGCTTTTATCTGCGCCAGCACGGTGAGGAAAAAGGGCGCCAGATAGCCTGTGCCCTGCTGGAAGATTTAATGGAATCCGGCCCCCTGAAGGCCG
CGCCGTCGCTGTCCTTTCTCGGGCTGGTTGTGATGGATGAACTCTGTGCCCGTCACATAAAAGCGCCGGTACTGCACTGA

>CFICBL_23305 DNA primase
ATGAAAATGAACGTAACCGCCACCGTCAGCCATGCGCTCGGCCACTGGCCGCGTATTCTCCCGGCGCTGGGGATTCAGGTGCTGAAAAACCGTCATCAGCCCTGTCCGGTCTGTGGCGGGA
GTGACCGCTTCCGTTTTGATGACAGGGAGGGGCGCGGCACCTGGTACTGCAATCAGTGTGGTGCCGGTGACGGCCTGAAACTGGTTGAAAAGGTGTTTGGTGTTTCCCCGTCCGACGCG
GCCGCAAAGGTGGCTGCCGTGACCGGCAGTCTGCCCCCGGCTGACCCGGCAGTGACGGCCGCCGCCGTTGCTGAAACAGACGCTGCCCGGAAGAACGCCGCCGCACTGGCACAAACCC
TGATGGCAAAAACCCGTCCCGGAACCGGTAACGCCTACCTGACCCGCAAGGGCTTTCCCGGCCGGGAATGCCGGATGCTGACCGGCACACACAGAGCCGGTGGCGTGAGCTGGCGCGCC
GGTGACCTTGTGGTGCCACTGTATGACGACAGCGGCGAACTGGTTAACCTTCAGTTAATCAGTGCTGACGGCCGTAAGCGCACCCTGAAAGGCGGACAGGTCAGGGGCACCTGTCACATC
CTTGAAGGACAGAATCAGGCCGGAAAACGTCTGTGGATAGCGGAGGGATACGCGACCGCACTTACCGTACATCACCTGACCGGTGAAACGGTGATGGTGGCGCTTTCTTCCGTGAACCTC
CTTTCTCTGGCCAGCCTTGCCCGGCAGAAGCATCCGGCCTGTCAGATTGTCCTTGCCGCAGACCGTGACCTCAGCGGTGACGGCCAGACAAAAGCCGCCGCAGCCGCAGATGCGTGTGAA
GGTGTTGTTGCCCTGCCGCCGGTCTTCGGTGACTGGAATGATGCCTTCACGCAGTGCGGCGGGGAAGCCACCCGTAAGGCCATTTATGATGCCATCCGGCCACCGGCTGAAAGCCCGTTCG
ACACCATGAGCGAAGCGGAGTTTTCCGCCATGAGTACCAGCGAAAAGGCCATGCGTATCTATGAGCATTACGGCGAGGCGCTCGCGGTCGATGCCAACGGCCAGCTTCTGTCCCGTTATGA
AAATGGTGTCTGGAAGGTGCTGCCGCCACAGGACTTTTCCCGGGATGTGGCCGGGCTGTTTCAGCGTCTGCGCGCGCCGTTCTCCTCCGGGAAGGTGGCCTCCGTGGTGGACACCCTGAA
GCTGATTATTCCGCAGCAGGAAGCCCCCTCCCGCCGCCTGATTGGCTTTCGTAACGGCGTGCTCGACACGCAGAACGGCACGTTCCACCCGCACAGTCCGTCACACTGGATGCGTACCCTG
TGTGATGTGGATTTCACCCCGCCGGTGGAAGGGGAAACGCTGGAAACCCACGCCCCCGCGTTCTGGCGCTGGCTTGACCGTGCCGCCGGTGGCCGTGCGGAAAAACGCGACGTGATTCT
GGCCGCACTGTTTATGGTGCTGGCAAACCGCTACGACTGGCAGCTCTTTCTGGAGGTGACCGGTCCCGGCGGCAGCGGCAAAAGTATCATGGCCGAAATCGCCACCCTGCTGGCCGGGGA
GGATAACGCCACGTCGGCCACCATTGAGACGCTGGAATCCCCGCGTGAACGTGCCGCGTTAACTGGCTTCTCACTGATACGCCTGCCGGACCAGGAAAAATGGAGCGGCGACGGAGCCG
GACTCAAGGCCATCACCGGCGGCGATGCGGTGTCCGTTGACCCGAAATACCGGGATGCGTACTCCACGCATATCCCGGCGGTGATTCTGGCCGTGAACAATAACCCGATGCGCTTCACCGA
CCGCAGCGGCGGCGTGTCACGCCGGCGGGTGATTATTCACTTCCCGGAACAGATAGCCCCGCAGGAGCGCGACCCGCAGCTTAAGGACAAAATCACCCGCGAGCTGGCGGTCATCGTGC
GTCACCTGATGCAGAAGTTCAGCGACCCGATGCTCGCCCGGTCACTGCTTCAGTCCCAGCAGAACTCAGACGAGGCGCTGAACATCAAACGGGATGCCGACCCGACGTTTGATTTTATCGG
CTATCTGGAAACCCTGCCGCAGACCAGCGGCATGTATATGGGGAACGCCAGTATCATCCCGCGTAATTACCGTAAATACCTCTATCACGCCTATCTGGCCTACATGGAGGCAAACGGCTACCG
GAATGTACTCAGTCTGAAAATGTTCGGGCTGGGGCTGCCGGTGATGCTGAAGGAATACGGACTGAATTACGAGAAGCGCCATACCAAACAGGGGATACAGACCAACCTGACGCTGAAAGA
GGAAAGCTACGGCGACTGGCTGCCAAAATGTGACGACCCTGCAACAATCTGA

Region of unknown function 4

>HMDLIN_05230 Lipoprotein
ATGAAAAGCATAAAAAAATTGATTATCGCAAGTGCGTTGAGCATGATGGCTGCTAGTTGTTATGCTTCAGTTTTGCCGAACACAGAGCAACAGAAATCAGTGGATATTAGCTTTGCCGCTCCC
GAACATTTAACCATATCGTTTGATCAGATGCCAGGCTTAATGGCTGGGAAGAATGCTAAGGATATGGATATTGCGAAGCTTACAGTTGATTCTGCTTCTATTAAAGAGTTCGGGGCCAGGGG
GGTTTCTGGCACGCTAATGGACGATGCTGGCAGTGAATGGAAAATAACTGGTAAAAATGGTGGCAACCCAATCATTGTGCGCTTTTCAAATTATGCTCTAAACAAAACTCATGTCCCGCTAAT
GTGGAATGGGAGAAAATGGCTCACCTTTGATACCAATGTCCCAATTGATATTATTGCAGTAGCGGGTCAGGATATCTCACCTGATACGTACCCACTGACTGTAGACGTAGTGGGATATCAGCC
TTAA
>HMDLIN_05235 hypothetical protein
ATGTGTAAAAGCCGGAGGGGTTATCTTTTCCCGGCTTTTTATTATCAATTACTCATTAACTCCTGTTCCGTTCTTTTGCGTTTAATCACCGGAATATCTCCGGTATTGTTCAGCGCCCCGGAAAT
GTTTTTAACCACTGTTCTGCACTCCGTTTATTAA
>HMDLIN_05240 Cytoplasmic protein
ATGGTTAACGATAAGGATACAGCTATATTAATTAGTGATTTAATGTTGAGGTTCGGTAAGGAGCTTGATGAATCTGTTGCTGTTGTTCAGTCCCGTTGTGATGAGGATGAATTTAATGTATATCG
AGAAACGGTTGGTTTTATCATGGGTGAAATGCTTATTAAAATAATGAATCCATTATATGAAAAACATCCAGAAATAAAACCAAAAGGATTGAAATAA
>HMDLIN_05245 hypothetical protein
ATGGTGTTGATTGTGTTGTTCGTTATTCTATTTTTTACATGGTTAGGTAGAGAGAATATTAAAAATGATAGCGCTATTCGAGAAGTTGCGAAAGAAGAAGTTGATAAGCTTTTTTCTCTTTATAA
TGAAGGGGAGTATGCTGAGATATATGATTTGTCATGTGACTCTTTCAAAAATGCCACTGCGAGAAAAGATTTTTTAACAGTTATGGGAACTAAAATGAAAATACTCGGTGAATTTAAAGGGC
GAAAACTTCAATATAGCAATGTCATTAATTCAAAATCCGTGGAGTTATATTATAGAGTGGACTATATTAACTATTCTTTAATTGAAGAATTTAATTATATTAAAAATGATGGACAAAAAATATGCTT
GCAAGCAATGTATACGGATGATGCGGGGAAACATGGGGAGGTAATTAAATTACATTAA
>HMDLIN_05250 hypothetical protein
ATGGCGGGGCAATTGGAGCCGGAGTACACACCCCAAAGGAAAGAGAGGTTTGTGCGTATTATTCATTGTGTGATCATAGCGGTACAGGTACTTCTGCTGGTTTGTTATTCACAATATCAGCC
CCTGATGCTGATTTATTACCAGGAATATCAGAGTCTGGAGGGGATTCTTATGGAGGCACTTCTTGCGGGAATGGCTTATACAGATGCTTGTGATCAAACTGATAGTTATAAATATACAAAAGGT
GTGAGTATGGGACCCGGATTTGGAGACTAG
>HMDLIN_05255 isrA Hfq binding RNA
ATCTTCACCGTCACGCCGCTCCCCGGTCTCAAATCCCGCTTCTTCCTGGCAGTCGCCGCTGGGGATTTGTGAGTAACAGGTGGTCATGCAGGTTTCGCTGTTGATGTGGCGGACGCTG
>HMDLIN_05260 SMI1-KNR4 domain-containing protein
GTGATCAATATCATTTATTCTAAAAAACCGGCAACATTAGAGGATATCTCTGATTTTGAGAGAGAGATAAAGGGGGGTTTACCAATTGATTATAAAAATTTTTTGATGAAACATAATGGAGGGC
AACCTCAGCCTGACTCGTTCAAATTTTTTTCTGATAGAAATGATGCATCTTCAGTTGATCGGTTTCTTAGCTTAGGTAAAGAGAAAAATAGTAACCTTCTTAAATATTACAATAATTATAAAGATA
GAATCCCATCAGGCTTTATCCCTATTGCTCATGATGCGGGAGGTAACCTTATTATTATGGAGCTGAAAGATAACGAAAATAAAATTTATTTTTGGGATCATGAGGTTGAAGTTGATGAAGGAG
AGACTCCTGATATGAGTAATGTTTATTTAATCAATGAATCTTTTTCTGATTTTATAAATAATCTTTATGAAGAAGTTATCTAG
>HMDLIN_05265 hypothetical protein
ATGGTGTACCATTTAATCATGCAGGATTCCCTGATTTTTCACGTTATTCAATTAAAAATACTCGCATTGAATTGGGTGCGTCAAGGAATGTGGATTTTGGCAGGGCTGATCGTGCTGTGGGATA
TGGTCCCGGAAATCCTCGTCCTGAAGGATATACTTGGCATCACCATCAGGATTCTGGATATATGCAATTAG
>HMDLIN_05270 CdiI-2 domain-containing protein
ATGAGAATAATTAAAGGGTTTGATAGTTTATTATCTGAAGTTAAGACATTGCCGGATGTTGGCTGGCTATATGTGGATAAGGAATTTAATTTAAAATCTAAAATGGATATTTTAAATAAAGATTAT
TATCTCGCTGAAAATCGTGATGAGTCTTTTGATATGGCAGAAAATGATAAGATTAGAACATTTTTAGAATCTCCAACATTTTGTGACATTATTGATAATAGACTTAATCATCATCCCAATTCCAATA
GAGATGAACTTTTAGAAGCAGTGATTTATTATCTGGAAGAAGATGACTTTATGGATTAA
>HMDLIN_05275 DUF6531 domain-containing protein
ATGGGAGAAGCATTCTGGGCGGCAAGGGAAGGCGACGCGCTGCTGCATACGTCCTTTCTGGCAGATCTGGTGGGCAGTGCGCTGGAATTCGCCATCAATGCGGTTATTGATTTTGCGGCA
CTGGCTGTTGTGGCGCTGGCGACGGGGGCTACGGTGGCAACGCTGGGATGCAGCGCTGTATTACTTGTGGGGACCGTGGTCGGCGCGACGATGTTGCTGAGTGGCGCCGGGGAGAAAA
TCAGTAAAGCCTGTGAAGACATAGCCAACAGCCTTTTTCCGCCGAAAATTGAGGGATATATCCTGACCGGCTCAGGCGATACCCGGATTAACAGCAAACGGGCGGCGCGGGCGGCGGCAA
CGGCATTATCACGCCATGATGTAGAAACCCTGGATGCGCAGGCGCAGGCAGAGGCAGAAGAAGAAAAACGCAGGCAGGACGCCAAATCAATGTGGGATGTGGCCGGGGAATATCTGGAT
GCAGTAAAAGGCCGGGCAGAGAATATGGCTAAGGGCGTCGTTTCCGTGGTGAGCCGGATGGGATCAGCGGAAGGATGGGCCTCGCTGGGAAATGATGCTCTGGCGGAGGCGGGCGATA
TGCTTTCCGACACGGGCCACTTTATTTCAGAGATGTGGCAGCCCACGGTGGCAACGGCATATCCGGGGTCTGACCCGAAACAGGATGATAAAATCGACTGCCACAAACATCCCTCTTCATTC
ACCCAGTTTATGGCACAGAAACTGCAGGCGCTGACGGATGACCCGGTGGGGACGGTACTGGGGGCGATGAACACTTTCGATGTGCTGGAAGCCGGTTTTCAGGCGGCAAGCGCGCTTAT
CGGCAGCGTGTCGAACCTGTTCAAAGGCGACGATGAGCCGCCCGCAGCAGAATATATCGCCGAAGGGACGCGGGATGTGCGTATCAACAGCCAGCCTGCGGCGCGCAGCGGGGTACGCT
GTACCTGCGAGGCAAAAGTGGTGGATGAGCCTGAGAACGGCGTACATGTATCCGGTGATGTACGTATCGGCGGGCCGCTTCTGGTGGTACGGGATATCAAAAGCGGTAAGTCACAAATAAC

CCTGGTGACCACCATTGCGCTGACGTTCATGCAGCCCGGACGGGCGTCAGCTAAAAGTGCCTGCTTTATGATGGGATTGGGCATTAACATGATGGTTCAGAAGGCGGGGAGTGCGCTGAA
CCGCCCGGTAAACGCCGCCACCGGAGCCAAGTACCTGGCAGGCGATGATGACGTTGATTTCAGTCTGCCCGGCCACTTCCCGCTGGAGTGGCAGCGGACCTACAGCAGCCGTGATGAACG
GACGGAGGGGATGTTCGGGCGGGGCTGGAGCGTGCTGTATGAAGTGTGCCTGGAGCGTACGCCGGACAACCCGGATGAAAACTGCATGACGTATGTGGCCCCGATGGGACGGCGAATT
GACCTGCAGGCGGTGGAGCCGGGAAGCGGTTTTTACAGCCCCGGCGAGGGGCTGGCAGTGCGGCGCAGCGAACAGGGCCACTGGCTTATCAGCAGTGATGACGGCGTGTACAGGCTG
TTTGAAGCGGACCCGTTCAGCCCACAGCGTCGGCGGCTGAAAATGCTGGGCGACCGCAACAGTAACTGCCAGCACCTGACCTACGACAACCACGGGCGTCTGGTGGAAATCAGCGGCGA
TCGGCAGCGCCCCTGCATCCGGCTGCACTATGAGCTGGCAGCGCACCCGCAGCGCGTGACGCGCATCTTCCGCCATCACCCGGAAGGGGAGCCGGAGCTGCTGCGGCGTTACCGCTACGA
TGAGGCGGGGCGGCTGAACGGGGTGGTGGACAACGCAGGTCAGTATCAGCGCGAGTTTGCGTACGACGACAACGACTGCATGACGATGCACCGGGAGCCGGGCGGCGAACGGTATTAC
TACTCCTGGGCGTGGTTTGAAGGCCCGGACGATGCGGCGTGGAGGGTGACGGGCCATCATACGGACAGCGGCGAGCAGTACCGTCTGGACTGGAATCTGGCAGAACGTTCGCTGTGCGT
GACGGATAGTCTGGGGCGTACGCGCTGCCACTGGTGGGATGCGCAGGGCCTGGTGACGGCGTACCGGGACGAGGCCGGGCAGATGACCACTTTCCGCTGGAGCGATGAAGAGCGGTTA
CTGCTGGGGATGACGGACGCGCAGGGCGGCAAATGGCGTTATGTCTATGACCGTCTCGGCCACCTGACGGAGACGCATGACCCGCTGGGCCGGGTTGAGCAGACGCAGTGGCATCCGGT
GTGGCACCAGCCGGAAACGGAGGTGGATGCCGCGGGGGCGGCGTGGCGTTATGAGTATGATGAGCGGGGCAACCTGCAGGCGGTCAGCGACCCGCTGCACCAGCGCACGGTATACGG
GTACGACCGGCACGGTCAGGTGGTGCGGATAACCGACGCGCGGGGCGGAGATAAATACCTGCAGTGGAACGAAGACGGGCAGCTTATGCGCCACACGGACTGTTCTGGCTCGCAGACG
GCATGGTTTTATGATGAACGCACGCGGCTGGAAAGGGTGACGGACGCGGAGAGTAACAGTACGCGTTACAGCTATGACGGCAACGGACATCTGACGGAGGTCATGTTCGCGGACGGGCG
TACGGAGCGTTACCAGCCGGATGCGGCGGGACGGCTGGTGAAATACACCAGCCCGGCGGGGCAGATAACACGCTGGCAGCGGGACGGCCAGGGGCGGGTGCGCAGGCAGACGGATGC
GACGGGGCGCAGGACGGCGTATGAGTACGACGCTTACGGGCGGCTGACCACGCTCACGAACGAGAACGGGGAAAGCTACCGGTTCCGGTACGATGTTCTGGACCGGGTGACGGAACAG
ACGGACCCCGGCGGCAGCCGCCGGGCATACGGGTATAACGCGCTGAATGCGGTGACGGCGGTGATATACGGCGGGGAGCGCGGGGGAGAAATCCGCCACGGTCTGGAGCGTGATGCGG
CGGGGAGGCTGACGGTGAAAATCACGCCGGAGACGCGCACGGAATACCGGTACGACGCGGCGGACCGTCTGCTGGAAATCCGCCGCAGGCAGCATGATGCGGCGGAAGGCGGAGAGC
CGGAAGTTATCCGGTTCAGCTACGACAGTGCGGGTAACCTGCTGAGCGAGGAGACGGCGCAGGGCGTGCTGCAGCACCGGTACGATGTTCAGGGCAACCGCACAGAAACGCAGATGCC
GGACGGGCGGACGCTGCGGTACCTGTACTACGGGAGCGGCCATCTCCAGCAAATCAACCTGGGGCGTGATGTCATCAGCGAGTTCACGCGTGACCACCTGCACCGTGAGGTGCAGCGGA
GCCAGGGGCGGCTGGACACGCGGCGGATGTACGACCGGACGGGCCGGTTAACGCGGAAACTGACCTGTAAAGGAATGCGCGGTGTGGTGCCGGAGACGTTTATTGACCGGGAATATGC
GTACAGCGGCCAGGATGAGCTGCTGAAAAAGCGGCACAGCCGGCAGGGGGTGACGGATTATTTTTACGACACGACGGGGCGCATCACGGCGTGCCGGAATGAGGCATACCTGGACAGC
TGGCAGTACGACGCGGCGGCGAACCTGCTGGACAGGCGGCAGGGAGAGACCGCGCAGGCGGGTGCAGGCAGCGTGGTGCCGTTCAACCGGATAACGTCATACCGTGGGCTGCATTACC
GTTACGATGAATATGGCCGGGTGGTGGAAAAGCGGGGCCGCAACGGCACGCAGCACTACCGCTGGGACGCGGAGCACCGGCTGACGGAAGTGGCGGTCATCCGGGGGAGCACCGTAC
GGCGTTACGGGTACGTGTACGACGCGCCGGGCAGGCGGGTGGAAAAGCACGAACTGGACGCGGAAGGAAAGCCGTATAACCGGACGACGTTTTTATGGGACGGAATGCGGCTGGCGC
AGGAGTGCAGGCTGGGAAGAAGCAGCAGCCTGTATATCTACAGCGACCAGGGGAGCCACGAACCGCTGGCGCGGGTGGACAGGGCGGCGCCGGGCGAAGCGGATGAGGTGCTGTATT
ACCATACGGACGTAAACGGCGCGCCGGAGGAGATGACGGACGGCGGGGGCAATATTGTCTGGGAAGCGGGCTATCAGGTATGGGGGAACCTGACGCATGAAAAAGAAACCCGGCCCGT
ACAGCAGAACCTGCGTTTCCAGGGGCAATATCTGGACAGGGAAACGGGGCTGCATTACAATTTGTACAGATTTTATGATCCGGATATCGGGAAGTTTATATCGGGTGACCCTATTTCGATCCG
GGGCGGGATAAATCTTTATCAGTATGCGCCTAATCCGATTAAATGGATAGATCCACTTGGCTTATATAACGGTGAAGGTCAACGTGAACTTGGCAAATATCATGTATTTCATGAACACAATCTAG
ATATTACTGAATATGGATTATCAGATGCGGAACATTTTAGCCGTGGAAATCAGGCTATTTATGAACGGATGAAAAATGATCCTGCGTTCAGACGAGAAATGCAAACTAAGTATCCGGGTGTTG
TTGAGCATGTACAACCAAGTTCAGGCGGAAAATTCAGTACGGAATCACCTCCAGGTTTGACGTGGCATCACGAAAATAAACCTGGGGTATTAAGTTTAGTTGATCGCTTGGATCATAAAACT
TACCACAAGATTTATCACCCTGATGGTTCAGGTGGGCGTAAGAAATGGGGGGGAGGTACTGGATGTCGTTAA

PTU-I1

>JIALED_00005 IS110 family IS5075 transposase
ATGGAAAACATTGCGCTTATTGGTATCGATCTGGGTAAGAACTCTTTCCATATTCATTGTCAGGATCATCGTGGGAAGGCCGTTTACCGTAAAAAATTCACCCGACCAAAGCTAATCGAATTTC
TGGCGACATGCCCGGCAACAACCATCGCGATGGAAGCCTGTGGCGGTTCTCACTTTATGGCACGCAAGCTGGCAGAGTTAGGGCATTTTCCAAAGCTGATATCACCGCAATTTGTCCGCCC
ATTCGTTAAAAGCAACAAAAATGACTTCGTTGATGCTGAAGCTATCTGTGAAGCAGCATCACGTCCATCTATGCGTTTCGTGCAGCCCAGAACCGAATCTCAGCAGGCAATGCGAGCTCTGC
ATCGTGTCCGTGAATCCCTGGTTCAGGATAAGGTGAAAACAACTAATCAGATGCATGCTTTTCTGCTGGAATTTGGTATCAGCGTTCCGCGAGGTGCTGCCGTTATTAGTCGACTGAGTACCC
TTCTTGAGGACAGTAGTTTGCCTCTTTATCTCAGCCAGTTACTGCTGAAATTACAACAGCATTATCACTATCTTGTTGAGCAGATTAAAGATCTGGAATCTCAGTTGAAACGAAAGTTGGACG
AAGATGAGGTTGGACAGCGCTTGCTGAGTATTCCCTGCGTTGGAACGCTGACTGCCAGTACTATTTCAACTGAGATTGGCGACGGGAAGCAGTACGCCAGCAGCCGTGACTTTGCGGCGG
CAACAGGGCTGGTACCCCGACAGTACAGCACGGGAGGTCGGACGACATTGTTAGGGATTAGCAAGCGGGGCAACAAAAAGATCCGAACTTTGTTGGTTCAGTGTGCCAGGGTATTCATAC
AAAAACTGGAACACCAGTCTGGCAAGTTGGCCGACTGGGTCAGGGAGTTGTTGTGTCGGAAAAGCAACTTTGTCGTCACCTGTGCTCTGGCAAACAAGCTGGCCAGAATAGCCTGGGCA
CTGACGGCGCGACAGCAAACTTACGAAGCATAA
>JIALED_00010 YadA
GTGGCTAATTACAGTAACGACCGCTGGGAAGCACCGCAACGTGCCTCACGCCTGGCTGCCAGCGTAAAGCGTTACAAGACATCAGAAATGCTGCGCTTTATTTTTGCTACCATTGCCTACGA
TCCCGATCCAGATTTAACCCCGCTGACTGTGAGACGTTTGTGCAAGGCATTGTTCGGCAGAACCGGAAGCCAGTGGCTGGTTGTTGAAGTTTTTGGGGAGAAGGGGCGTCAGCACCGCA
GCGCGGACAGCAATCCGGAGATGGTGGAAAAAAATGGCTGCCAGATATCGTCATGCCGCCGAACTACACTGGTCCGCAACGCTGGCAGAAATTGAACGGGTAAAGCGGCTTTATCAGACGA
AAATTAAGAAATCCAAAAAATAA
>JIALED_00015 DUF3951 domain-containing protein
ATGTCGTCACGCAACTCTGTACATGATTTTATAATGCTGCTCCCGGCGCTGCTGTTGGTTGGGTGGTTAGTTTTGGTGATAATTCTCATGTTTAGGGATGATAAATGTGACAAAACTTACCGTG
AAGGTTCGTTTCCCGAATTTATTTGTCTCCATTCTGATCGCTCGTCACTTCGAGAAACCGATTCTGAGAGACTCATTGTCAAATATACAGAAATAAAATCACAAAAATAG
>JIALED_00020 YaeB protein
ATGATCACAGGAAATGGAATAAACACTGTAACTGTCAATGGAAAAGTGAAACACATCACAGAACTGGATGATATAACACTTTGTTTAGAGTGGACAAAACTACGAGAGGAAAACAATCGTC
TTTATGAAATTAATAACCAGGCAAACAGAGGATGGCGTGGTTTCATCCTAAGATTGATAGGGGTTAACCTGCCAGATAAAAGGACTGAGTTTACACAACGTCTCTTATTGACGCGCAAAATTT
CGGGTTCGGTCATGAAAAAGTAA
>JIALED_00025 Protein YafA
ATGTGTGATTCAGAAGCGGAGAAGATTTTATTCATCCTGAATAAACATGAACGGGCATACAGACGCCTGATCCCCGTATATATTTTTTTAGCTATAGTATTATGGATTTTACCTATTCTCCTGATT
GTATACCTGTATTTCTGTATAGTTGACAATGAGTTTCTCAGAATCGGTTTCTGTATTGCATGGACAGCATACTACTTTCTTTCGCTTTTCAAGTATTTTGTAAAATACTTCTTTCCTCTCGATCGTG
AAGATTTGCTTAAACTGATGGAGCTCGCCAAAGATTCTCCTGATGCGAAACAGGAGCTTCTTGAGCGTCTTTTGTCCGGAAAAAGAATGACCGGGCGGGATGAAATGGATATCAAGCGCAT
TCTAAAAACAAAGGAAGCACTTGATAACATCAGGAATTTTATTGATAAACAGCAGTTAACGAATAGTGATACTCCGAAGAAAAGCACGGTCCCAAATGAAGATAAAAAAGGCATAAGATGA
>JIALED_00030 Fertility inhibition FinO-like protein
ATGGGAAAAAATCAGGAAGAAAGGAAAACTCCCGTCATTGTGGTGAAAAAACGTCGTACTTTTTCGCTACCGTCCTTATCCGAAAAAACAGATATTATCGCACCTGTATTTACTGAACAGAC
GGCAGAATCAGCCCCCGCCGGAATAAATTCATCTGCCGTCGAAACACATATCCCCGAAGCGCCAGCCAGGAAGAAGAAAAAGAAAAGACACCGTTTTCCACGTCCCTCCCACTGGACCCG
TGAGTATACTCACGAGTGCGTGGAAAAAATAAAGGCCCTGTTTCCTCATCTTCGTGCTGAAGGTGGTGGATTTATTCCCCTGAAAATTGGCATAAACAATGATATTTCGGCATTTCTGGCAGA
ACACCCGGAGACAGAACTGACCATGGACGAATGGCTCTGTGCAGTGTCCTGCATCACATCCCGCCGCGTATATCTGCAGCGGACAGCTGTCGCCGGCGTCCCACGTTATGGCCTGGATGGT
CACCCCAAAGGGCAGGTCTCAGACTCTGAAGCACAGAGTGCCGGTCGCAGACTGGCGACATTAGAACAAAAGTGGCTCAGGATGCAGGCACAGCAGGAGAATATTTCTGGGCAATAA
>JIALED_00035 SWIM-type domain-containing protein

ATGCCAATGATACTTGTAAAGAAGAATCCCCGGGGCAAAGTGATTCGGGAGCTTAGCAGTGAGGAAGAGACTGCAGTAAAGACGGTCTGTGGACTGAAAAAGCCAGCAACCATGGCACT
ACATAACCTGGCCAATGATATTCTCCGGGAAATGCGTGAGTATGATGCATGGTTACAGTGTGACTGTATTCCGGGAGATTCGCCGGCCATGAATTTTGCTGCTCTTAAAAACAATACGGGTAC
TCTTTATCTGTCCAGTTTTAATCATGAGCATGCACCTGAATGCCCCATGTATCGTCAGTTATCCGGAAATGAAGAGGAAACATCTTCCGGAGCAAGCCGGCATCCCGTTAGCACCCGTATAAAT
TACAGAAACTTTTTACCACCTGATGACAGTAATTCTGTTATCAGACTGCAGGCCCGCTCTGCTTACCATAATGGTGAGAGCTCCTCCGTTCGCAAGAAACGTCTGCGCCTTGCCCGTTTACTCC
TGTCTCTTATTGACGATGCTGGGCTGAATAAACTGGATTCCCTGGCTAATCCCCGTATCCGGACTAATTATCGTGAGTGTCTTGATGCCATACGGCAGGTTACACTGCAGCAGGAGTATATTCG
GGGAAGATCCTTATCTGAAATTATCCATTTCCGGCCTGGAATGTCTGAAAGGTCTCAGGAGCGGTTAATGGAAACGCTGGAGAATTCAGAACGACACTGGCCTGCCAGAAGAAAGCATATG
TTCTTTCAGATTTTTATGGCGCAGCACATCTGTCGTGATGCTGTGGAAATTCACTGGGCGAACGGTAATATTCAGGTCATCAGACCTGTGCGGGGGATCAGTATTAATGGTGAAGCGCAGGG
CGGAATACGTCCCCCTTACTGGGTTATTCTTGCTTTTTGCCGGAGTGCCGATGGCAGAATCATCTGCAGTGAAGGGTATGCTCATGCTCTTTATCAACTGACATGTCCGGTGCCTGTGGACAG
CAAACTGGAACGAAACACGCTCACTGCTCTTCTGAATGTGGCCAGCTGGCTTAAAAGAAAGCCAGGTACGCCGGAATTAAGTCTGGAAAGGCCCCTGTTTGATACAGAAGTTTATGTTAAT
GGTGAAAAGAAATATGTACTGCCGGATTTCATTGTTACAGCAAGGGCTCCTGACGGAAAGACGGCCAGAGTGGTCATCGAAACGATGGGATATGAAGACAGTGATTACTGCGCGAGAAAA
TCCAGGCAGCATACCGGCATGAAGCAGATTGGTGTTCTGCATACCGATCCACCGAAATGGCTGGATAACGATCATCCCCCTTTTGAGAAACATATGTACGGTGTTTTTATGCATCTCAGGTACT
GA
>JIALED_00040 Colicin-Ia
ATGTCTGACCCTGTACGTATTACAAATCCCGGTGCAGAATCGCTGGGGTATGATTCAGATGGCCATGAAATTATGGCCGTTGATATTTATGTAAACCCTCCACGTGTCGATGTTTTTCATGGTAC
CCCGCCTGCATGGAGTTCCTTCGGGAACAAAACCATCTGGGGCGGAAACGAGTGGGTCGATGATTCCCCAACCCGAAGTGATATCGAAAAAAGGGACAAGGAAATCACAGCGTACAAAA
ACACGCTCAGCGTGCAGCAGAAAGAGAATGAGAATAAGCGTACTGAAGCCGGAAAACGCCTTTCTGCGGCAATTGCTGCAAGGGAAAAAGATGAAAACACACTGAAAACACTCCGTGCC
GGAAACGCAGATGTCGCTGATATTACACGACAGGAGTTCAGACTCCTGCAGGCAGAGCTGAGAGAATACGGATTCCGTACTGAAATCGCCGGATATGATGCCCTCCGGCTGCATACAGAGA
GCCGGATGCTGTTTGCTGATGCTGATTCTCTTCGTATATCTCCCCGCGAGGCCAGGTCGTTAATCGAACAGGCTGAAAAACGGCAGAAGGATGCGCAGAACGCAGACAAGAAGGCCGCTG
ATATGCTTGCTGAATACGAGCGCAGAAAAGGTATTCTGGACACGCGGTTGTCAGAGCTGGAAAAAAATGGCGGGGCAGCCCTTGCCGTTCTTGATGCACAACAGGCCCGTCTGCTCGGGC
AGCAGACACGGAATGACAGGGCCATTTCAGAGGCCCGGAATAAACTCAGTTCGGTGACGGAATCGCTTAAGACGGCCCGTAATGCATTAACCAGAGCTGAACAACAGCTGACGCAACAG
AAAAACACGCCTGACGGCAAAACGATAGTTTCCCCTGAAAAATTCCCGGGGCGTTCATCAACAAATCATTCTATTGTTGTGAGTGGTGATCCGAGATTTGCCGGTACGATAAAAATCACAAC
CAGCGCGGTCATCGATAACCGTGCAAACCTGAATTATCTTCTGACCCATTCCGGTCTGGACTATAAACGCAATATTCTGAATGACCGGAATCCGGTGGTGACAGAGGATGTGGAAGGTGACA
AGAAAATTTATAATGCTGAAGTTGCTGAATGGGATAAGTTACGGCAACGATTGCTTGATGCCAGAAATAAAATCACCTCTGCTGAATCTGCGGTAAATTCGGCGAGAAATAACGTCAGTGCC
AGAACAAATGAACAAAAGCATGCAAATGATGCTCTTAACGCCCTGTTGAAGGAAAAAGAGAATATCCGTAGCCAGCTTGCTGACATAAATCAGAAAATAGCTGAAGAGAAAAGAAAAAGG
GATGAAATAAATATGATAAAGGATGCCATAAAACTCACCTCTGATTTCTACAGAACGATATATGATGAGTTCGGTAAACAAGCATCCGAACTTGCTAAGGAGCTGGCTTCTGTATCTCAAGGG
AAACAGATTAAGAGTGTGGATGATGCACTGAACGCTTTTGATAAATTCCGTAATAATCTGAACAAGAAATATAGCATACAAGATCGCATGGCCATTTCTAAAGCCCTGGAAGCTATTAATCAG
GTCCATATGGCGGAGAATTTTAAGCTGTTCAGTAAGGCATTTGGTTTTACCGGAAAAGTTATTGATCGTTATGATGTTGCTGTGGAGTTACAAAAGGCTGTAAAAACGGACAACTGGCGTCC
ATTTTTTGTAAAACTTGAATCACTGGCAGCAGGAAGAGCTGCTTCAGCAGTTACAGCATGGACGTTTTCCGTCATGCTGGGAACCCCTGTAGGTATTCTGGGTTTTGCAATTATTATGGCGGC
TGTGAGTGCGTTTGTTAATGATAAGTTTATTGAGCAGGTCAATAAACTTATTGGTATCTGA
>JIALED_00045 Colicin-Ib immunity protein
ATGAAACTGGATATATCTGTAAAGTATTTACTGAAGAGCCTGATACCAAGCCTCATTATTCTTACAGTTTTTTATCTGGGATGGAAAGATAGCCAGGAAAATGCAAGAATGTTTTATGCGTTCAT
CGGATGCATTATCAGTGCCATTACTTTTCCTTTTTCAATGAGGATAATACAGAAAATGGTAATAAGGTTTACAGGGAAAGAATTCTGGCAAAAAGACTTCTTTACAAATCCAGTTGGCGGAAG
CTTAACTGCAATATTTGAATTATTCTGTTTCGTTATATCAGTTCCTGTGGTTGCCATTTACTTAATTTTTATATTCTGCAAAGCCCTTTCAGGAAAATGA
>JIALED_00050 Transmembrane protein
GTGACCTTTGAGCAGAAAAAAGCCCGGGCCATTGCCCTGATGGACAGCAAAAAGATGTGGCGGAGCAATTATGCCCCTCCTCTTCTGTGTATTCTCTGGCGGCTGGGTATCCGGCTTCCGC
CATTACCCTTTATGCCGTTCTGGCAGGTCACGCTGTTGATGGGGAGCTTATGGGGCATTTCCTGGGGATGTGCAATGTGGTTTATCTACCGGGGGCCGTCAGGAATGGTGGCAGGTGAGGC
CATAATTATAAGTATCACGGGTGGTTTTTTGTTCGGACTGCTCACGGCCTCTTTCCACTGGTGGCGCAGGAAAGTTAACCGGCTGCCACCATGGGGTGATGTGTAA
>JIALED_00055 YdeA protein
ATGACTCAGAGCCGCCGACCGTCGCCACTGCAGCGGAGAGTGCTGATTGTGCTGGCTGCCCTTGATGAAAAACGGCCAGGACCCGTGCTGACGCGGGACATAGAACGGGTGCTGGAGCG
AAGCGGGGAAGCCCCGGTCTACGGGCCAAACCTGCGCGCCTCCTGCCGTCGTCTGGAGGATGCCGGCTGGCTGCGTACCCTGCGCGCTCCGAACCTGCAGCTGGCAGTGGAGCTGACGG
ACGCCGGCCGGGCCGTGGCACAGCCTCTGCTGCTGGCAGAACAGGACCGCCTCCGGGCAGAACAGCGTGCGGCAGAAGTCGTCGTACTGCCGCTGGTCCCGGCAGCAGGTCTGCCCGC
AGATGGCACTTCTGCCACTGATCTGGAGGTGGAGCTGAACGGCATAACGTATCAGGCCTGCCGTGGGGATTTTGTGGTGCGTCTCGACGGCAGCACCTGTCTGCAGTTATGGAATAAAGAA
GGCAGAGTGGTACGCCGGGAGGGCGATCCGCTGGAAGTGGCGCAGTGGCTGCAGGCCTGTCATGATGCCGGAATAGAAGTTCGTGTACAGGTTAATGAAAGCGTCACTCCGTAA
>JIALED_00060 YdfA
ATGGATGAAGAAAGGGTGTTTTCACTCAGCTATGAACAACTAACCCGGTTTACGGAACGACGCATCCGGGAATGCAATCTGGACAGCCAGGGCGCGATTTATCTCTGCGAGTCAGCCAAAG
CCGGAGCCGTGCTGATCTTCTGGCATGAACTGGCCATAAATGGCTATGCCTCAATGAATGCCATAAAGAGACAGGAGCTCATTGACGCCGACTTTCAGCGCCTCAGAAAGCTCATCTGGCCG
GAGGATGACTGGAAATGA
>JIALED_00065 DUF3293 domain-containing protein
ATGTTTTTTATTGAGAATGAAGGTCAGGCTGTCGCCGGAACGGATTACTGGCAGTCTGTACAGGCGCAGGCCGGATATGTCTACCTCAGCTGGAATGCCGGCGCAGCCAGGCTGCTTGTCC
CGGATGCGGCAAAACATTTACTCAGGGAGATGCGGGGGGCTGAGTACGTCATCATCAGTAAGGGAGCACTGCATGGCCGCGATGCGCTGGAACTGGTATTTGAAGACGGCAGCGATGCG
CCGTTTGTGATCCACATGCTGAGTGAGCAGTGCGATCGCCTGCTCCCCGAAAACAACCAGGGAGGGGGTTTTGTTGTCACCGTCTGGACGCGTGGAGGTAACCAGCTCCGTTATCCGGGA
AAGTACCGTGTTGTGGAAAACCTGCCCGACGTTTCCCCGTGGAGTGAACACTGA
>JIALED_00070 Resolvase
ATGCAGCACCTGCCGGCACCGATCCACCATGCCCGGGATGCTGCTCAGCTTCCTGTTGCCATCGATTATCCGGCAGCGCTGGCACTCCGCCAGATGTCGATGGTTCATGATGAACTGCCAAA
ATACCTGCTGGCCCCTGAAGTGAGCGCCCTGCTCCATTACGTGCCGGATCTGCGCCGCAAAATGCTGCTGGCCACACTGTGGAACACCGGTGCGCGCATTAATGAAGCACTGGCGCTGACG
CGGGGGGATTTTTCGCTTGCGCCTCCGTATCCGTTTGTGCAGTTGGCCACTCTGAAGCAGCGGACCGAAAAAGCCGCCAGGACGGCAGGAAGAATGCCCGCCGGTCAGCAGACTCACCG
GCTGGTTCCGCTCTCTGACGCCTGGTACGTCAGCCAGCTGCAGACGATGGTGGCCACACTGAAAATCCCCATGGAACGGCGTAATAAACGAACAGGCAGGACAGAGAAAGCGCGGATCT
GGGAAGTGACGGACAGAACGGTCAGGACCTGGATTGGGGAGGCGGTTGCCGCCGCTGCCGCTGATGGAGTGACGTTCTCTGTCCCGGTCACGCCACATACGTTCCGCCATTCCTATGCGA
TGCACATGCTGTATGCCGGTATACCGCTGAAGGTCCTGCAGAGTCTGATGGGGCATAAGTCCATCAGCTCAACGGAGGTCTACACGAAGGTCTTTGCGCTGGATGTCGCGGCGCGTCATCGT
GTGCAGTTCGCCATGCCGGAAGCCGATGCGGTAGCGCTGATAAAACAACTTGAGAGGCGTTAA
>JIALED_00075 hypothetical protein
ATGGCCTGGATAGTTGGTTTTATTTCTCAGAAAGGTGGTGTGGGCAAAAGCACTAAAGCGAGAGCGTTAGCGCGTGAAGCATCCGCCTGTGGTATCAAAACAAAATTAGCTGATCTCGACT
TGGAACAGGCAACGAGTGCTGAGTGGCATCGTAGACGTCTGTCAGCTGGTTTGTCACCGGTTGCTTCCGTTGAAGTGTTTTCAACAGCAAAGCAGGCGATAGAATCTGCCGGCGATTTTG
ACCTTCTGATTCTTGATGGCCCTGCTCGTGCAAGTAAAGGTACTTCAGAAATTGCTAAGGTCGCCGACCTTATCGTCCAGCCAACGGGAGCCTCCCTTGACGATCTTGTCCCGGCAATTAAAG
TTTTCAACGCGTTGGTAAAAGAAGGTATACCACGTTCAAAACTGGTCTTCGCTTTAAGCCGGGTAGGTACCGAAGCGGAAGAGCTGGATGCTCGTGCATATATCAGTGAAGCAGGTTATGA
AACTTTATCTGGATGCCTTTTTGAGAAGCCAGCATACAGAAAAGCGATGAATTCAGGACTCGCTGTGACAGAAACACGTTATAAAGGTTTAAACGAGAGAGCTGATGAACTTATCCAGGCA
CTCATCGATAAGATTGGAGAAGAGTAA
>JIALED_00080 Stability/partitioning determinant
ATGGCTGATGCGTCAAAACTTAGAACTAAAAGAACCCGTAGTGGGTTGGGGGTACCCCCGGGCCCCGACGAAGTGGCTACCAGCTTGAATGCGCCTGAGATAGCTCCGGCTGTCGCTGCC
ACTGATGTTCAGAGACAACAGGCAGACTCATCGCCAGTGCACCATGAGCCTGTGGTTACGCAAACAGTGACCGAGGTATATACCCGCCGCGATGGCCGTTCTGCCAGAAAAACAAACAGG

ACGCTGGCATTTGCAACACGTGTCACACCTGCTTTCGACCAAGAGATCCGGGATATCGCAGAGCGTGAGGGGTTGAAACTGGTGGAAGTTCTGGAAAAAGCAGTTGAGGCATATAAGGAA
AAACAGGGCTATTAA
>JIALED_00085 StbA domain-containing protein
ATGAGAATTTTTTGCGATGATGGTTCTACAAATGTGAAACTTGCCTGGTTTGAAGGCAAGACGCTGAAAAGTGCGGTATCAGTGAACTCTTTTCGCCATAACTGGAAAGTCGAGGGCTTAG
GCAGCAGTCGTACTTATAACTATCTGCTTGATGGCCGTAAATATACCTACGACCCGGTCAGCGAAGATGCGATCAGCACGACTCATATTGAGTATCAGTACTCTGATACAAATGTGTTGGCAGT
TCACCACGCATTACTGAACAGTGGCATTGAACCCCAGGAGATCGATCTCACCGTTACGTTACCTATCAGCGAGTTTTACACGGCCGACTGCCAGAAAAATACGCTTAACATTGAGAGAAAAA
TTCGCAACCTGATGCGAGAAGTCACCCTGAACAAAGGGGGAACTTTTACCATTAAAAGCGTTGAAGTGATGCCTGAATCACTCCCGGCTGTATTTACTCGCCTAGTTGCTGACAACGTTGGT
CAGTACGAAAAATCATTGGTTATCGATCTCGGTGGTACAACTCTGGATGTCGGGGTTATTGTTGGCCAGTTTGAGGATGTTTCGGCTGTACATGGTAATCCTGATATTGGTGTATCAATGGTCA
CGAAAGCCACGCTCACTGCACTGAAAATGGCCAGCAGTGATACCAGTCCAATGATTGCTGACGAGTTGATCAAAAACAGGAATAACCTGGATTTTGTGGGGCAGGTTGTAAATGAGGTCTC
TAAACTAAATCTGGTGCTGGATACCATTGATACAGCGATAAATAAACTGGGCGAGCTAGTTGTTGATGATTTGCTGCAGTATCGAAACGTAAACCGCGTATATATTGTCGGTGGCGGGGCTGC
ACTCATTGCTGATGCTGTTCGTAAGGCATGGAACCACCTTGGCGAAAAGGTGGTTCTTATGGATGAACCTCAGACAGCGCTGGTCCAGGCTATTGCACGCTTCAAAGCGGAGGAGTAA
>JIALED_00090 Plasmid partitioning/stability family protein
ATGTACGAAGGCGAAGAAAGGAAAAAACTTTCACTCTACCTTCACCCGGAGGACTCTGCCGATTGTCTTGCACTGGCTGAAATCGAAACTGTTCCCCGGAAAAAACGGGGGGAGTTATATC
GGCAGGCTTTGATAACCGGCCTGATAATGCATCAGCTTGATGAGCGAATTCCCGCCGTGTTAACCGCGCTATTCACCAGAGAATTGAATGCGGATGAGGTCATTAGTCAGATTGCCAGGATA
ACAGGCTGGAAACCCTCCTCCGGTGATTTAAAAGAGGTACTGAAGGCGTTGGGAGGGCTTCAGAGTACAGTTTCACCAGAACACTCTCAGGATGACGGTGAACAGGCCCGTCTGAAAGC
GGCTAGGGTGAAGATGCAAAACCTGATCTAA
>JIALED_00095 DinI family protein
ATGATCCGCATTGAAATTCTTTTCGACCGCCAGAGCACAAAAAAACTCAAATCAGGCACACTTCAGGCGCTACAGAATGAAATCGAACAACGTCTGAAACCACACTACCCGGAAATCTGGC
TGCATATGTGGGAATCCCCGTCCTTCAGAGTGAGGAGCTGTCAACCAGCACTCCATTGA
>JIALED_00100 DUF1281 domain-containing protein
ATGCCAAACTGGTGCAGTAACCGTATGCATTTTTCTGGTGAACCGGCACAGATTGCTGAGATTAAACGACTGGCCAGTGGTGCCGTCACACCGTTTTATCGCCGCGCCACAAATGAAGGTAT
TCAGCTGTTTCTGGCCGGAAGTGCCGGACTTCTGCAGACCACTGAAGATGTGTGGTTTGAACCGTGCCCCGGACTGACGGCTGCCGGACGTGGCGTTGTATCGCCGGAGAATATCGCGTT
CACCCGCTGGCTGACACACCTGCAGAACGGTGTACTGCTGGATGAGCAAAACTGCCTGATGCTGCATGAACTCTGGCTGCAGAGCGGTACTGGCCAGCGTCGCTGGGAAGGATTACCGGA
CGATGTCAGGGATACCATCACCGCGCTTTTCACCGCAAAAAGAGGTGACTGGTGTGGCTTCTGGAGTAACGAGGATGTATCGGTGTGGTGGAACCGTCTGTGTGACAACGTACTGCCGGA
AAAAACCATGCCGTTTGACCTGCTGACGGTTCTGCCCACCCGCCTTGATGTTGAAGTGAATGGCTTTAACGGTGGTGTTCTGAACGGTGTTCCTTCTGCATATCACTGGTATACCGAACAGTA
TGGCGTGAAGTGGCCTTGTGGGTATGACCTGAATATCAGCAGTCAGGGAGACAACTTCATTCAGGTTGATTTCGACACGCCGTGGTGTCAGCCGGAAAGCGACGTTATTGCAGAATTAAGC
CGCCGTTTCAGCTGCACGCTGGAGCACTGGTATGCCGAACAGGGCTGTGATTTCTGTGGCTGGCAGTTGTATGAGCGCGGAGAGCTCGTTGATGTGCTGTGGGGGGAGCTGGAATGGTCT
TCCCCGACAGATGACGATGAGCTGCCGGAAGTCACCGGACCTGCGTGGATAGTCGACAAGGTGGCGCATTATGGCGGATGA
>JIALED_00105 hypothetical protein
ATGAAGTATGACGGAGACGGGCGGGCTACCGCCCGTTTCTTTTCCGGTAAAGGATGTCGCCGTGCTCCTCTTTTTACTGCGCCGGCTGACGCGGCGCGGCATAAACGCTGCCTGTGGTCAG
TGTCCCGCGTCCGGCCGGGCACGGGACGGGCGCTTTTACCGCTCCCGGCTGGCGTCGGTGACAGTGTACGCCAGCCTGTCGCCCTTTTCTGA
>JIALED_00110 Methyltransferase
ATGTCCCGTTTTGTCCTCGGTAACTGCATCGATGTTATGGCCCGTATCCCTGATAACGCCATTGATTTCATCCTCACCGACCCGCCATATCTCGTCGGTTTCCGTGACCGTTCCGGGCGCACCAT
CGCCGGCGATAAAACCGATGAGTGGCTGCAACCGGCCTGTAATGAAATGTTCCGCGTACTGAAAAAAGACGCGTTAATGGTGAGCTTCTACGGCTGGAACCGCGTCGATCGCTTTATGGCC
GCCTGGAAAAAATGCGGGATTCAGCGTTGTTGGTCACCTGGTCTTCACCAAAAACTACACATCGAAGGCCGCATATGTGGGCTATCGCCACGAATGCGCCTACATCCTGGCAAAAGGCCGTC
CACGTCTGCCACAAAACCCGCTGCCGGACGTGCTGGGCTGGAAATATTCGGGCAATCGCCATCACCCGACGGAAAAGCCCGTTACCAGCCTGCAACCGCTGATTGAGAACTTCACACACCC
GAACGCAATTGTGCTGGACCCGTTTGCAGGCAGCGGCTCAACCTGCGTCGCCGCCCTCCAGTCCGGACGCCGGTATATCGGTATCGAGCTGCTTGAGCAGTATCACCGTGCCGGGCAGCAA
CGCCTTGCCGCCGTGCAACGGGCCATGCAGCAGGGGGCCGCGAATGATGACTGGTTTATGCCGGAGGCTGCGTAA
>JIALED_00115 Cytoplasmic protein
ATGAACTATGCAGGACACGAAAAACTGCGCGCCGAAGTGGCGGAGGTGGCCAATGCCATGTGCGACCTGCGTACAACCATGAATGAGATGGAGCAGCGGTACAGCTTTAATGCCGACACC
CTGCCGGAACGTCTGGTGCGTCAGACGCTGTTTCGCGCAAACCGCCTCCTGATGGAGGCATATACCGAAATTCTGGAACTGGAAGCGTGCTTTAAAGATTGA
>JIALED_00120 DUF1380 domain-containing protein
ATGTACGGAACATGCGAAACGCTATGCCGGGAGCTGGCAGTAAAGTATCCGGGAGACATGCCGCTGATGCTGGTTATCTGGTCCCCGGAAGAGATTCAGGCCCTCGCTGACGGAATGGATA
TTTCCCTGTCCGATCATGAAATCAGAACCGTCCTGGCGCGCCTGGAGGACATCCCGGAAGACCAGCGGACTGAATCCGGTATTTCTTCCGGCGTGGCGATGGAGATCATCAATAACGTGAG
CGAAAACCGCCAGGTGACCGTCCCTGCTGAACTGCTGGCGTCCCTGATTCAGACCGCTGAACAGGCATTGTGGAAACGTGAATGGGCCGCCCGGGATCATGGCCTCGCCGTCCCGGAATG
CGTCACCCGCCGTCAGGCGGTGATTAATCAGGCCCGCACCCTGCTGAAAAACAACAGACACGAAAACGACTGA
>JIALED_00125 YchA
ATGAACGAAAACACAACACTGAACGCACTGATTTGTCGTCACGCCCGCAACCTGCTGCTGGCGCAGGGCTGGCCGGAAGAGACGGATGTTGACCAGCGGAACCCGAACCATCCGGGCTG
GATCAGCATTTATGTTCTGCTGGATGCGCCCCGGCTGGCGACGTTACTTATCAACCGTCACGGCGGCGCACTGCCGCCGCTCCTGGCCTCCGCCATTCACAAACTGACCGGAACCGGGGCG
GAACTTGTACTGTCCGGCAGTCAGTGGCAGTCGCTGCCGGTACTTCCGGCAGACGGAACGCAGGTGTCTTTCCCGTATGCCGGTGAGTGGCTGGCAGAGGACGAAATCAGGGCAGTTCTT
GATGCGGTACGCGATGCAGTACGCTGTGTCAGTTACCAGGTGGCAGAAGATACGCGGCGTATCCGGGCGGCGCTGACCACCACCGGTCAGACGTTACTGACCCGCCAGACGCGCCGCTTT
CGCCTGGTCGTGAAGGAAAGCGATCACCCCTGCTGGCTCGATGAGGACGACGAAAACCTGCCCGTGGTGCTCGACGCCATCCTGAACCGGGGCGCACGTTTTTCGGCGGTGGAAATGTAT
CTGGTCAGCGATTGTATTGAACATATCCTGTCCAGTTGGCTGGCCTGGGATGTGCTGCGTATACCGGATGAACCGCCCCGCCGCTGGTTTGACCGTGGTGTTCTGCGGGAGGTGGTCCGGG
AAGCCCGGAACGAAATCCGCAGCATGGCGGATGCCCTGGCAAAAATCCGGAAATGA
>JIALED_00130 Antirestriction protein
ATGCAATATGCGAAACCTGTCACTCTGAACGTTGAAGAGTGCGACCGTCTGTCTTTTCTGCCTTACCTGTTTGGCCAGGATTTTCTGTATGCCGAAGCGTCTGTATACGCGCTGGCGAAACAA
ATGATGCCGGAATATGAAGGCGGATTCTGGCACTTCATCCGACTGCCGGACGGTGGCGGTTACATGATGCCGGATGGCGACCGTTTTCACATGGTGAACGGTGCAAACTGGTTTGACCGTA
CCGTGAGTGCTGATGCCGCAGGCATCATCCTTACCTCCCTTGTGATTAACCGCCAGTTGTGGCTGTACCACGACAGCGGGGATGCAGGACTGACCCAGCTTTACCGGATGCGCGATGCGCA
GTTGTGGCGTCACATCGAATTTCACCCTGAATGCAACGCGATTTACGCAGCACTGGACTGA
>JIALED_00135 DUF1380 domain-containing protein
ATGTACTGTACTGTTAAAGAAATTATCCGCGATGTACTGGATACAGACGTGCCGGACAGTGAATGCGTTTTTGCCGTGGTGCTGACCCGTGGGGATGTGCGCCACATAGCCCAGGACTGGA
GTCTGACAGACGATGAGCTGGAAACCGTCATGCAGCGGCTGGACGATGCCTTTGAATATGGTGCGGATGTCAGCGTTGTTCACGGCGTTGTTCGTGAACTGATGGAAGAAAAGCGCGCCA
GCCGTCAGGTGACAGTCCCGGCGGTGATGCTGGAAAAAGTGTTGGCGCTGGCAGGCAGTGAAATGAAGCGCCTGTATGCCGTCGGGAGTGAGAACGGGGGCGACGGTGATGCGTTCGT
CAGGGAAGAACGCGAAGCAATGGACGTTGTGTTACAGGCGCTGGACGGGGAGCACATGTCATGA
>JIALED_00140 hypothetical protein
ATGAATATCAGCACAGAAACCCGCGAAATTCTGCGCAATTACAAAGCCGTGATTAATGCGCGGCGTCGTGAAATGGGGCAGAAACCGCTCACCACTGCGCAGATTGTTGATGAAATCTGCG
ATTTTGTGGCGAATCAGCAGGCGGTTTTCCTCGGTGGTCACTATATCCTTCAGGGCAGCAGAAACAGGTGA
>JIALED_00145 hypothetical protein

ATGCAGGGAACAGGTGTGATATTCCGTGACACTGTCACCGTTTCTGGCTGTCTTTTATTGCGCCCACTTCGGCTCCCGGCTGCCTGGCGGCATCCGGTCGTCAGGCCCGACTCCGACAGGCA
GCTGCTGGCGCACCCGCCAGTCTCCGCCGAACCCGCTGGCGCGGTTTCGGGCTGCCGCTACGGTCCGATGCGTGAAAACCCGGCTTGCAGCGACCGTTCCGGTCGCGCCGCCCCTGTCGC
GCCGTGA
>JIALED_00150 Single-stranded DNA-binding protein
ATGAGTGCACGTGGTATCAACAAGGTCATCCTCGTCGGGCGTCTGGGCAATGATCCGGAAGTCCGTTACATCCCCAACGGGGGCGCAGTGGCAAACCTGCAGGTGGCCACATCAGAAAGC
TGGCGTGACAAACAGACGGGGGAGATGCGGGAGCAGACGGAATGGCACCGCGTGGTGCTGTTCGGCAAGCTCGCGGAAGTGGCAGGTGAATATCTGCGCAAGGGTGCGCAGGTCTAC
ATCGAAGGTCAGCTTCGCACCCGTAGCTGGGAAGATAACGGTATCACCCGTTACGTCACTGAAATTCTTGTTAAGACCACGGGCACCGTGCAGATGCTGGGACGTGCACCACAGCAGAACG
CTCAGGCGCAACCGAAGCCTCAGCAGAATGGGCAGCCACAGAGTGCTGACGCGACGAAAAAAGGTGGCGCGAAAACGAAAGGCCGTGGACGTAAGGCCGCGCAGCCAGAGCCTCAGC
CGCAACCGCCGGAGGGGGAGGATTACGGGTTTTCAGACGATATCCCGTTCTGA
>JIALED_00155 UPF0401 protein YubL
ATGAGCGAATATTTCAGAATACTTCAGGGACTGCCGGACGGCTCCTTTACCCGCGAACAGGCGGAAGCCGTTGCCGCACAGTACCGGAACGTCTTTATCGAGGATGATCAGGGAACGCATT
TTCGCCTGGTTGTCCGTCAGGATGGCACGTTGATCTGGCGCTCCTGGAATTTTGAGGACTGTGCCGGGTACTGGATGAACCAGTACATCAGGGATTTCGGGATTCTTAAGTAA
>JIALED_00160 ParB domain-containing protein
ATGCCTGTAACGAAGTGTGAACCAGAAACCACCCGCAAAGCAAGCCGTAAATCTGTAAAAACGCAGGAAACTGTCCTGTCTGCCCTGCTGGCGCAGACGGAGGAAGTGAGCGTGCCGCT
GGCCTCGCTGATTAAATCACCGCTGAATGTGCGCACGGTGCCGTATTCTGCGGAGTCCGTCAGCGAACTGGCGGAGTCCATTAAGGGCGTCGGACTGCTGCAGAATCTGGTCGTGCATACC
CTGCCTGGTGACCGTTACGGTGTCGCCGCAGGTGGTCGCCGACTGGCTGCACTCAACATGCTGGCAGAGCGTGGCATCATTCCGGCTGACTGGCCTGTACGCGTGAAGGTTATTCCGCAG
GAGCTGGCGACGGCTGCATCAATGACTGAAAACGGTCATCGTCGGGATATGCACCCTGCCGAACAGATTGCCGGATTCCGTGCGATGGCGCAGGAAGGCAAAACGCCTGCACAAATCGGT
GATTTGCTGGGCTATTCGCCCCGCCACGTTCAGCGAATGCTGAAACTGGCAGACCTTGCGCCTGTCATCCTCGATGCGCTGGCAGAAGACCGCATCACCACAGAGCACTGTCAGGCGCTGG
CGCTGGAGAACGACACCGCGCGTCAGGTGCAGGTGTTTGAAGCCGCCTGCCAGTCGGGATGGGGCGGTAAACCGGAAGTACAGACCATTCGTCGTCTGGTGACCGAAAGTGAAGTGGC
GGTGGCAGGTAACAGTAAATTCCGCTTCGTGGGGGCTGATGCTTTCTCACCTGACGAACTGCGTACTGATTTGTTCAGCGATGACGGGGACGGTTATGTCGACCGCGTGGCGCTCGATGCT
GCCCTGCTGGAAAAACTCCAGGCTGTCGCTGAACACCTTCGGGAAGCCGAAGGCTGGGAATGGTGCGCCGGACGCATGGAGCCTGTCGGTGAGTGCCGTGAGGATGCCGGAACATACC
GCTGTCTGCCGGAGCCGGAAGCGGTGCTGACGGAGGCAGAAGAAGAACGCCTGAACGAACTGATGACGCGTTACGACGCGCTGGAAAACCAGTGTGAGGAATCCGACCTGCTGGAAG
CAGAAATGAAGCTGATGCGCTGCATGGCGAAGGTCAGGGCGTGGACGCCGGAGATGCGTGCCGGAAGTGGCGTGGTGGTGTCCTGGCGTTATGGAAACGTATGTGTCCAGCGTGGTGTG
CAGTTGCGCAGTGAAGATGACGCGACTGATGACGCTGACCGCACGGAACAGGTGCAGGAGAAAGCGTCAGTGGAGGAAATCAGTCTGCCGTTGCTGACGAAAATGTCTTCCGAACGCAC
GCTGGCAGTCCAGGCAGCACTGATGCAGCAACCGGACAAATCTCTGGCACTGCTGGCATGGACGCTCTGCCTGAATGTGTTTGACAGCGGAGCGTACAGTAAACCAGCACAAATCAGCCT
GGAATGTAAACATTATTCGCTGACCAGCGATGCGCCGTCAGGGAAGGAAGGTGCCGCATTCCTGGCGCTGATGGCAGAAAAAGCCCGTCTTGCGGCCCTGTTACCGGAGGGATGGTCACG
GGACATGACGACGTTCCTGTCACTCAGTCAGGAGGTGCTGTTATCCCTGCTCAGTTTCTGCACCGCATGCAGCCTTAACGGTGTCCAGACCCGCGAGTGTGGTCGCACGTCACGCAGTCCG
CTTGACTCACTGGAGAGCGCCATCGGTTTTCACATGCGCGACTGGTGGCAGCCGACGAAAGCAAACTTCTTCGGACACCTGAAAAAGCCGCAGATTATCGCAGCCCTGAATGATGCCGGA
CTGTCCGGTGCCGCACGGGACGCAGAGAAGATGAAGAAAGGCGATGCCGCTGAACATGCAGAGTTCCATATGAAAGACAACCGCTGGGTGCCTGGCTGGATGTGTTCACCACGTCCACA
GACGGATGCCACTGAACGCGCCGATAACCTGGCTGATGCCGCCTGA
>JIALED_00165 Protein PsiB
ATGAATACCGAACTGACACTGAATGCCCTGCAGTCCATGAATGCCCAGGAATATGAAGAAATCCGTGCTGCGGGAAGCGATATGCGCCGTAATCTCACTCACGAGGTGATGCGTAAAGTGG
ACGCACCGGCTAACTGGATGATGAACGGAGAGTATGGCAGCGAGTTCGGGGGCTTTTTCCCCGTCCAGGTTCGTTTCACGCCAGCCCACGAACGTTTCCACCTGGCATTATGTTCGCCGGG
AGACGTCTCTCAGCTCTGGATGCTGGTTCTGGTGAATGGTGGTGGACAGCCTTTCGCCGTCGTTCAGGTGCAACATATCTTCACGCCTGCCGCGATCAGTCACACGCTGGCGCTTGCCGCG
ACACTGGATGCGCAGGGATACAGTGTTAACGACATCATCCATATCCTGATGGCAGAAGGAGGTCAGGCATGA
>JIALED_00170 Plasmid SOS inhibition protein A
ATGAGCGCACGTTCACGGGCACTGATCCCCCTCAGCGCAGAACAACAGGCCGCCATGCAGGCGGTGGCTGTCACAGAACAACGTCGTCGTCAGGGACGCACACTTTCAGCATGGCCTTAT
GCCACCGCTTTCTTTCGCTGCCTGAATGGCAGTCGCCGGATTTCGCTGACCGATCTCCGCTTTTTTGCCCCTGCGCTGACGAAGGAGGAATTTCATGGCAACCGTCTCCTGTGGCTGGCTGC
CGTGGATAAACTGATTGAAAGTTTTGGGGAAGTCTGTGTTCTTCCCCTGCCATCCGATGCGGGGCACCGTCTGTTCCCGTCCGTGCCTTTTCGTGAAGGTGAGCGTCGTCGTCAGAAAACC
ACGCTGACAGAGCAGAAATACAGCCGCCAGCGGGAACGTGAGGCAGAACGACGGGAACTGGAATACCAGACATGTTTTGCTCAGGCGCAGATTGACCTTGCGTTTCATACTCCCGCCACG
GTCGGAAGCTGGTTGTCCCGCTGGTCTGGTGTTGTTGAGGAGCATGATCTGGAAACGATTTTCTGGGGGTGGTGCGGGCGTTTTCCATCACTGTCATCATTTGACCGGTTTTTCTGGCAGG
AGGAACCACTCTGGCGGCTGATTTTTGAAGCCGGTGAGGCCGGTCGTGGTGCACCGGTACAGGTACGTGCACTTGAGCAGTGGATGATCCCGAACAAGCTGGAGAACGTAATATGA
>JIALED_00175 hypothetical protein
ATGATGAAATCAGACGAAAAATACCAGGTTCCCGCGTGGATGCGACCTCTGTTGCCGTTGCTCTGCAACACCGGGGGGAACGATCCGGAAGAACTGCTGAATGATACAGAAACCACTGCC
AGTGCGAATGTTGTCCGTTATGTACTGATAGTTGCTGTGCGGTCGCAGGTTGATCTGCTGCAGCTTCTGTACAGGAAAGGGCTGTTGCGCACAGAGATACCAGGTGGCTTTTCACCGGAAG
AAGCGCAGGCACTCCTGGATAATCTGGTGCGCAGCCATATCAGCAAGGCGCTGTCGGGCGAGCGAATGGCAGCCCGTGACAGAAATGCCGATCTGGCCTGGATTGGACAGCAACTGGTC
GATGACGCCTGGTTTGTCCGTGCCACACTGGAAGCGCATGGGATGAGCGTCGGAAATGAGAGTCCCCCTGCTCCGCCGGAGACAATGCCGGACATACAGACACGGGAACTGGTGATGTTG
ATCAAGCGACTGGCATCATCGCTGAAAGCGGTGAAACCGGACAGTTGTGTGGTGCGTGAAGCGCAGGACTGGCTGCGCGACAGAAAACTTGTGGATATCACAGATATTCTCCGGTGA
>JIALED_00180 Post-segregation killing protein PndC
ATGACAGTCAGCAGCACGATTTCCGTTTTTTGCCGCGACGGGGTGTTCCGTACCGTTTACTGCCACCTGCACGGGGAGCCGACCTGGAACGGTCGCATCCTGCATACCCACTATGCCACCG
GTCAGCAGGCCGAAGCCCTGGTTGAACACGGTGATATCCGTTGCCTCGGTCCCCGTTGCGACAAACCCGCCGGGCATACGCTTCAGAACCCGGTGGACGGTGTGACGGCTTATTACGGAC
GTGACAGTGGTTTCCGGATGGACAGTGAGGCGCGTGAGTACCGTTCTTTCAGGGAGGCTATTGCCACTGAAAGCACTGAAGAGGTGCGCTTCCATTATGTGTTCATCGACGGCTACTGGAA
GGTGATGTACCGCACGCCGGAAGGCTGGAAGATGAAAGCGCTCGCGCTGGCACTGCGTCGCTGTCCGAAATGA
>JIALED_00185 hypothetical protein
ATGAGACCATCAATTATCTTCGCAACCGCCGAGTATGTAAAGCGTCTGCGTGAAGAGTGCCTGCGGGAGAATAAACCCCTGCACCGCCATACCCGCTTCAGACGTCAGGAGCTGGCACAGG
ATGAGATTAACCCGGACGTCCTGGCGATGAGCGGCCATATCGCCAGACGCTGCAGTGAGCAGAAGCGGGTGCGTATCCCCGCTATGAAAGTCAGCGAATGGGGCCACCTGCTCCGCGCGC
TTGAAATTGAGCGGGTCTGCCACTGA
>JIALED_00190 Transposase-31 domain-containing protein
GTGAGTCAGAAGAAAACCACCACGACGCCCACGCCGCATGATGCCGCGTTCCGGTCGTTCCTGGCGAATCCCGACGTCGCCAGAGATTTTCTGGAACTGCATCTTCCGGCGGAGTACCGG
CAGTTGTGCGACCTGTCCACGCTGAAGCTGGAACCCGCCACCTTTGTTGAGCCGGACCTGCATCAGTACGCCAGCGATATCCTCTGGAGTGTGAAAACCACCGGGGGTGAAGATGGCTATG
TTTATACGCTCATCGAGCACCAGAGCACCGAAAATCTGTACATGCCTTTCCGCATGTTACGTTACAGTGTGGCGGCGATGCAGAGACATCTGGAGCAGCACAAAACGTTGCCACTGGTGATT
CCTGTACTGTTCTATCATGGTGAGCGCAGCCCGTACCCGTACAGCATGAACTGGCTGGACTGTTTTGAGAATCCGGCACTTGCGGCTAAAATATACACAAAGCCGTTTCCGCTGGTTGATATC
ACTGTCGTTGATGACAATGAAATCATGAACCATCGCCGGATGGCCGCACTGACGCTGCTGATGAAGCATATCCGCCATCGTGACATGATGGAGCTGCTGGACAAACTCCCGCAGGTCATGGT
GGAAATTTCAGACGAGCAGGTGCGTGTTCTGATTCATTACATCGTTAACGCAGGGGACTCTGTATCACCGGAATTTATGCGGGCGCTGGCTGAGCGTCTGCCGCAGCATGAGGATAAACTG
ATGACTATCGCAGAACGTCTTGAGCAAAAAGGTCGCCAGGAGGGCAGGATGGAAGGACGCATGGAAGGACGCATGGAAGGACGCATGGAAGGAGCGCTTGAAAAAGCCCTGGCTATTG
CGTGCCAGCTTCAGAAAATGGGGATGACGCCGGAGCAGATTAAGCAGGCTACCGGACTTTCCGATGACGAACTGAAGAAAATCACTCACTGA
>JIALED_00195 Transposase
ATGACGATTGCAGAACGTCTTATTCAAAAAGGCGCACTTGAAGTGGCGCGGGAAATAGCCTGCCGGCTGCGGGATATGGGCTGGACGCCGGAACGGATTCAGGAGGCAACCGGACTTTC
CGGTGAAGAACTGAAAAAGCTGTTTCCTGATGAGCAGTAG
>JIALED_00200 XRE family transcriptional regulator

ATGACCGGCTGGGAACTGCGAATCTGGCGAAAAAGCATGCTCTGGTCACGCGAAAAAGCGGCCAGAGAATTTGGCGTGACGCAGAGAACATGGCACGCCTGGGAAAATGCGGAACAGG
TGGACGTCACCGTCTGGAGGACCACCCAGGCACTTTCAGTCCGTGACCTGCTGCCTCACATGCAGGGGATGCGTAAGGCAGATATCATCAGGCGACTGGAAAATGAACTGGGGGAAACG
GCAGAGGATGTGTGA
>JIALED_00205 DUF4942 domain-containing protein
ATGAATCAGACTTTACCCACTGCTGACCTGAATACTGCCGGCACGACAGATGTTATTCCGTCTGTGGCCATCGACCGCATCATCGCGCAGCGTAACGAAGGTGTTGCACTGTTCATGCAGGC
GATGGAATGCCTGGCGACAGCGCGCAGGATTCTGCTTGATGCGTCAGGTGATATTTTTCTTTACGGGTTTGAAGACTGCGTGACTGATTCCGTTCGTCGCATAGATAAACCGGAAGAAGCG
AAAAGGAATATCACCCGTCTTGCCGACCGGAAAATCTGGGACCGCCTGATGACAGATACGGGCATGTACACCTTCATGAGTTCATGCCAGCGTGATGAGTGGAACAGCCAGCTGATGAGCG
ACACCTGTCCTGAAATCACCCTGGACAATGTACTGGCAACATTCCGCCATCTGAATGCCTGCAAGATGCAGACATTTGAACAGGGACTGATTGATGTCTACCGGAAATTGTCATGGGATTACA
GAACCAATAATCCCTGCCGTCTGGGTAAGAAAATCATTATTGAAAACCTGCTGTACCGCTGGAGTAACGGGCGTGTGACGCTGGACTGCAGCGGACGGGAGGCACTGGATGACCTGGTAC
GTCCGTTTTATCTGCTGGAGGGGCGCAACGTTCCTGACTTCAGGAACAGTATCGGGGCGCAGTATGGTGAATTTCTCGGGAACGGCGACAATGTCGGTAAGCTGTTAGAAGGGGAATATTT
TACGGTGCGTGGCTACCAGAAAGGGACCGTACACATTGTCTTTAAGCGTTCTGACCTTGTTGAAAAACTGAATGATATTATTGCACGGCATTATCCAGGTGCATTGCCGCCACGAGTCTGA
>JIALED_00210 Molybdopterin-guanine dinucleotide biosynthesis protein MobC
GTGCCAATGGCTTCTAAAAAATTTTACTCAGATGATGACATCCAGCTGGCAAAAGCTGCTTTATCGGAATTGCCAGATCTCACCGCTCAACGGAAAACATTGCGTGATTTTCTTGATGCCATTC
GTGATGACATTATTGTCCTGGTAAGGACAAAGGGTTATACACTGGCAGATGTTCGTGACACGCTCAAAAATGCAGGGTATGAAGTAGGAGAGAAGGCACTTCGTGACATTATTCGGGAAGC
AGAATCGAAAAAACCATCCCGCCGTTCTTCCTCTAAAACAGCACCTAAAAAGACAGATTCAGGCAGGAAAGATGGCATCGATATGAACAACAATTGA
>JIALED_00215 Conjugal transfer relaxosome component TraJ
GTGAGTGATAAACGTCAAAGAAGTGGTAGTGAAAGACGGCAAAAGACGATCCTGAGAGCGGTCCGGTTCTCACCGGATGAGGATGAAACTATCTGTAAGAATGCTGAAGATTCCGGACTT
ACTGTATCTGCTTACATCCGAAATGCAGCACTGAATAAGCGAATTAACTCCCGTACAGATGATGCTTTTCTGAAGGAGTTAATGAGACTGGGAAGGATGCAGAAGCATCTGTTTGTTCAGGG
AAAAAGAACCGGTGACAAGGAATACGCAGAAGTGCTTGTTGCCATAACCGAACTTACAAATACATTGCGTAAACAGTTAATGGAAGGTTGA
>JIALED_00220 IncI1-type relaxase NikB
GTGAATGCAGTCATTCCGAAAAAGAGAAGGGACGGCAAGTCTTCGTTCGAAGACCTGGTATCCTACGTCTCTGTCCGGGATGACATGACCGATGAGGAACTGGATTTATCTTCTTCTTCGCA
GGCTGAACAACCGCACCGAAGCCGTTTCAGTCGCCTTGTTGATTATGCGACACGTCTTCGAAATGAGTCATTCGTGGCGCTGGTTGATGTCATGAAGGACGGCTGCGAATGGGTCAACTTT
TATGGTGTCACCTGCTTTCATAACTGTACTTCTCTTGAAACTGCCGCTGCAGATATGGAGTACATTGCCCGGCAGGCACACTATGCAAAAGATGACACTGACCCTGTTTTTCACTACATCCTTT
CCTGGCAGTCACATGAAAGCCCGCGTCCGGAACAGATTTATGACAGTGTACGTCATACGCTGAAATCACTCGGCCTTGCCGACCATCAGTATGTCTCTGCCGTGCATACTGATACAGATAATCT
GCACGTCCATGTGGCTGTTAACCGGGTACACCCTGAAACGGGTTATCTGAACCGGTTATCATGGAGTCAGGAAAAACTCAGTCGTGCCTGCCGTGAGCTTGAACTGAAGCATGGTTTTGCT
CCGGATAACGGTTGCTGGGTCCATGCGCCGGGAAATCGTATCGTTCGCAAAACTGCCGTTGAACGTGATCGCCAGAACGCCTGGACACGCGGAAAAAAACAAACTTTTCGTGAGTATGTT
GCGCAGACAGCGGTCGCTGGTTTACGCAGTGAACCTGTACATGACTGGTTATCCCTGCATCGTCGTCTTGCGGAAGATGGTCTGTACCTGTCTCAGATGGACGGAAAATTTCTGGTGATGGA
TGGCTGGGATCGCAACAGGGAAGGTGTACAGCTTGACTCGTTTGGTCCCTCCTGGTGTGCAGAAAAACTCATGAAAAAAATGGGTGACTACACGCCAGTGCCAAAAGACATTTTCAGCCA
GGTGGAAGCACCAGGACGCTATAATCCGGACTTCATTGCAGCTGATGTTCGCCCGGAAAAAATCGCTGAAACAGAAAGTCTGCAGCAGTATGCCTGTCGTCATCTTGGAGAACGTCTGCCG
GAAATGGCACGGGAAGGACGGCTGGAAAACTGCCTGGCTATTCACCGCACACTGGCTGAAGCGGGATTATGGATGCGTGTTCAGCATGGTCACCTGGTTATCTGTGACGGTTATGATCATA
ACCAGACTCCCGTTCGTGCTGACAGCGTATGGTCACTGCTGACGCTCGACAATGTGAATCAACTCGATGGTGGCTGGCAGCCTGTACCAACAGATATTTTCCGCCAGGTTACGCCAACAGAA
CGCTTCCGTGGTCGTCGTATGGAGAGCTGTCCTGCGACCGATAAAGAATGGCACCGTATGCGTACAGGTACGGGGCCGCAGGGGGCTATCAAACGCGAACTGTTTTCTGACAAAGAAAGT
CTGTGGGGATACAGCATCAGCCATTGTAGCCCTCAGATTGAAGAAATGATCACACAGGGTGAGTTTACCTGGCAGCGTTGTCATGAGTTGTTTGCACAACAGGGACTGATGCTACAGAAAC
AACATCACGGGCTTGTTGTCGTTGATGCTTTTAATCATGAGCAAACGCCGGTGAAAGCCAGCAGCATTCATCCTGATCTGACACTGGGGCGTGCGGAGCCACAGGCCGGACCTTTTGTGAG
TGCTCCAGCAGATTTGTTTGACAGGGTGCAACCTGAAAATCGCTATAACCCGGAGCTGGCTGTCAGTGACAGGTACGGGGTCAGCAGTAAACGTGACCCAATGCTGCGCCGGCAGCGACG
TGAAGCCAGGGCTGAGGCCCGTGCTGACCTGCGTGCCCGCTATCTGGCATGGCGTGAACAATGGCGTAAACCGGATCTGCGTTATGGGGAACGTTGTCGGGAAATTCATCAGGCATGCCG
TCTGCGGAAGTCACACATTCGTGCGCAGTACGATGATCCGGCATTACGTAAGCTGCATTATCACATTGCAGAAGTTCAGCGAATGCAGGCATTGATCAGGCTGAAAGAAGACATCAGGGAT
GAGCGACAGAAACTTATTGCTGACGGGAAATGGTATCCACCCTCTTACCGTCAGTGGGTCGAAATTCAGGCTGCTCAGGGAGACAGGGCTGCTGTATCGCAGTTGAGAGGCTGGGATTATC
GCGATCGCCGTAAAGACAAGTCACGCACAACGACGACAGACCGCTGTGTCGTGCTTTGTGAACCGGGCGGAACGCCGGTATACGGTAATACCGGTGATCTCGAGGCCCGTCTGCAGAAGA
ACGGGAGTGTTCGTTTCCGTGACCGTCGGACGGGAGAGTTTGTCTGTACGGATTATGGTGACAGAGTTGTTTTCCGTAATCACCATGACCGCAATGCGCTGGCAGATAAACTGGATTTGATT
GCACCGGTATTATTCGGGCGTGATCCACGCATGGGGTTTGAACCAGAAGGAAACGACAAACAATTTAACCAGGTCTTTGCTGAAATGGTTGCCTGGCATAATGTGACAGGCCGTACCGGTC
ACGAAGACTACAGGATTACGCGACCGGACGTGGATCATCACCGTGAAGGAAGTGAACGCTACTACCGCGATTATATCGCGGCGAACAGCAACGATGACGCATCACTGCCTCCGCCGGAGC
AGGATAAACGCTGGGAACCGCCTTCTCCAGGTTAA
>JIALED_00225 TraG-D-C domain-containing protein
ATGAGTGAACATCGCGTTAACCCCGAGCTTTTACATCGTACAGCCTGGGGAAATCCTGTCTGGAATGCACTCCAGAGTCTCAATATCTATGGCTTTTGCCTGGTGGCAAGTCTTGTGGCCAGT
TTTATCTGGCCACTTGCATTACCGGCATGTCTGTTGTTCACACTGATAACAATGCTGGTATTTAGTCTGCAGCGATGGCGCTGCCCTCTTCGTATGCCCATGACGCTGGAATGTGCTGACCCGT
CACAGGACAGAATGATTAAAAGAAGTCTTTTCAGCTTCTGGCCAACCCTGTTCCAGTATGAGGTCATTCTGGAATCACCGGCCAGCGGCATATTCTATGTTGGTTATCAACGCGTCAGGGATA
TTGGCAGGGAGTTATGGCTGAGTATGGACGATCTGACCCGCCATATAATGTTTTTCGCCACCACCGGTGGCGGTAAAACGGAAACCATCTTTGCCTGGGCGATTAACCCACTTTGCTGGGCA
AGAGGATTCACCCTTGTCGATGGCAAGGCACAGAATGATACGGCAAGAACGATCTGGTATCTGGCCAGACGCTTTGGACGTGAAGACGATGTGGAAGTCATTAACTTCATGAACGGAGGA
AAATCCCGCAGTGAAATCATTCTGAGTGGTGAAAAAACGCGTCCTCAGTCAAATACCTGGAATCCCTTCTGTTACAGCACCGAGGCATTCACAGCGGAGACAATGCAGTCAATGCTGCCCCA
GAACGTCCAGGGGGGCGAGTGGCAGTCCCGGGCCATTGCCATGAATAAGGCACTGGTATTTGGTACTAAATTCTGGTGTGTGCGCGAAGGCAAAACAATGTCGCTGCAGATGCTCCGTGA
ACATATGACTCTCGAAGGGGATGGCCAAACTTTACTGTCGTGGCCTCGATGATCAGTGGCCGGAAGAAGCTATCGCACCGTTACGTAACTACCTTCAGGACGTTCCCGGTTTCGACCTGTCGC
TGGTCAGGACACCATCAGCCTGGACGGAAGAACCCAGAAAACAACATGCTTATCTCTCCGGGCAGTTTTCTGAAACTTTCAGCACATTCACGGAAGCATTCGGGGATATTTTTGCTGAAGA
TTCCGGTGATATCGATATCCGTGACAGCATTCACAGCGACAGGATTCTGATGGTAATGATCCCCGCTCTGGATACATCAGCGCATACAACGTCTGCTCTTGGACGAATGTTTATCACCCAGAAA
AGTATGATTCTCGCCCGTGATTTGGGCTACCGCCTCGAAGGAACTGATTCCGATGCACTGGAAGTCAAAAAATATAAGGGGCGTTTCCCTTATCTGTGTTTCCTTGATGAAGTAGGGGCATAC
TATACGGACCGTATTGCTGTGGAAGCCACTCAGGTGCGTTCACTGGATTTTGCTCTGATTTTGATGGCACAGGACCAGGAACGTATAGAAGGACAGACAACAGCCACGAATACTGCAACAC
TGATGCAGAACACGGGGACAAAATTTGCCGGCAGGATTGTCAGCGAGGGTAGTACGGCCAGAACGCTGAAAAGCGCCGCCGGTGAAGAAGCCAGGGCGAGAATGAACAACCTGCAGC
GTCAGGACGGCATATTTGGTGAATCATGGATTGACAGCCCACAAATCAGCATCCTGATGGAAAGCAAGATAAATGTTCAGGAGTTGATTGAACTTCATCCTGGCGAGTTCTTTTCTATTTTCC
GGGGAGAAACCGTACCATCAGCATCATTCTTCATCCCTGACGATGAGAAAAGCTGCAGCAGCGATCCCGTTGTTATAAACAGGTATATCAGCGTGGATGCGCCACGCCTGGACCGTCTCCGC
CGTCTTGTTCCCCGTACTACTCAACGTCGAATACCGTCACCGGAAAATGTCAGTGCCATCATTGGCGTCCTGACTGCAAAACCCTCCAGAAAAAGACGAAAAATTCGCACAGAGCCGCACAC
GATCGTGGATACATTTCAGCAACGCATTGCAGGACGTCAGGCGGCTATGGCAATGCTGGAAGAGTACGACACAGATATCAACGCACGTGAAAGTGCCTTGTGGGAAACAGCGGTGAATAC
GCTTAAAACGACCACACGGGAAGAGCGACGAATTCGCTATATCACGCTGAACAGACCAGAACTACCTGAAACAAAAGAGGAAAATCAGATATCAGTGAGAGCAGAAAGAGCAGGTATAA
ACCTGTTAACGCTTCCACAGGACAATAACCACCCGACAGGGCGTCCTGTAAACGGGTTTCATCATAAAAAAACCAACCGTCCGGACTGGGACGGAATGTACTGA
>JIALED_00230 IncI1-type conjugal transfer protein TrbB
ATGACAATCGAGTACTTTGCAACACGTATTGCAAAACATATTTCCGTGCAGGCAATCTGGCCGGATGGCAGGACAGAAATTATTGCCGTCCTTCCACGGGATGCCCTGTCAGGGCTGGTAAC
ACGAAATAATCAACTGATTCATATAGCCTTTGACGCCATGGGCACCCGTAACAGAGAAACCGTCCTGATTGATAAACAGGAGCATAACGCTGACCAGATACTTACAGCTATTGATTCCTGCCT
GCGCCTTGAATATCTGATGGAACGCCGCTTCAGCAGAACACCTTTGTTCCGTGGCATAATCGCCAGCGTTGTGCTGTTCGTAATGGCAACGATTGGCTTCTCTCTGTTCCGCTATGTTGATCGT
GTATTCTGGGATGATACAACGCCTGAAGCAGTACAGACAGCAGGTGAGCCACGACTGCTTCCCCCTCACCTGAATCACACTGTGCCATTAAATGAAGGCATTCAACTCCCGGTTCCCCCGAA
AAAAGACGTCCAGGTACCGGAGAAAACAATCACAAGCAAAAATCCGGAAGCCGCAGCTGCGCGACATAATCTGGCTACTGTTCTGAAGCGGAACGCCGATCGGGGCATGTTTACCATCAA
CCTGTCATCCGGTCATGAACGAACCCTGTTCGCATTTCTCGATCCTGCCTGTCCTAACTGCAGACTTCTGGAGCCGGCCTTAAAACGCCTGGCCAGCGACTTCAATGTCGTTATCTACCCGGTT

TCAGTTATTGGTGGAGAGGAATCCACCGACCGTGTGGCACCACTGCTGTGTGAAAAAGATGCTCAGAAACGTGCTGCCGGCTGGCACCGACTGTATTCCGCAGATAACGGAATGATGACGC
CATCAGAAGAAACAACGCCTGCAGATGAAACATGCCTGAAAGCAGCCAGAGCTGCGATTGATGTTAACAATGTCGCCTTCCGTAAGTTCGGATTTGCCGGTACCCCATGGGTGCTGTCCGA
CACCGGCTGGCATCTGCCTACAGGAATTCTGCAGGAAACTGGCACGCTTAATTTATTCCTGAAAACCACCGACAGCGAGTCTGGCCATGAGTGA
>JIALED_00235 IncI1-type conjugal transfer protein TrbA
ATGTCTTACAATCGACAGCCTGTCGCAGAAGATCCTATGCAGATATGGGGCGCTGTTGGTGTCCTTCTTATCCTGCTGCTATTCGTTATATGGTTATTCTTGCCGGAAGTGGTTTACGCCAGCTG
CCTGATTCTTCATACCTTATGGGGACTGGTTGACTGGGGACCATTTCATAACTATGCAGCACCACGATACAACCTGCTTGCCATGACCGGTAACAACGCCGCCAACATAAGCTATAGCCAGTG
GGTTAACGTCATGGAGCAGACCATAGGTATTCTGTGGATGTACCTCTTACCTGTGACACTCTGGTGTTTGTGGGAGTGGTATCAACATCCGGGACAAAGTCGTTTCACCCGCAGACCAGTAG
ATATTACCCGCCTGCCATACATCTTTGCTTCGTTGTCCCCTGCTATCGCCCCTGTACTGGCAGATGGTGATCCGGAAAAACTCTTTCATGGTGGCAAACGACCTGAACGGCGGGTTGCTCTCAC
CCCGGAAGCATTTGTCGAGCAGCATACTCTCATCACCAATATGCAGCTCGATGTTGCAGCTGCAAGACGATGTTTCATGGCCCAGTTGGGTAAACCTTTAACGTCCTGGAAAGACATGGCAC
CACATGAAAAAGCGCTCTTTGCCATATTTGGTCTTCAGTATTTCCTGGATGATCGTAAGGCAGCATTAAAGCTCATGGATACACTGAACCTCTCATGCAGAATTAAAAGCAAACGGGACAGTG
GGAAATTCTGTACCCCTGTTTATTCACTGGCCAAATCAGCTTTCCAGAGAGTGATTAAAAGCAACGGAGCACAGCAATGGCTGAAACAACATCGCTACGTCCGCAGTGGGCTGGTCTGGCT
GTATGCACATGACCTGCGGCTTACGCCACCCAACTGGATTTGGCTGAAAGGCGTGGACAGGACGCTGTTTTATGCATTACACCGCGCCAATACCACGAAAGGATTTATTGAAGGCGCTGGC
GTGGTGGCCGTTGCACGTGCAGAGGCCGAAGCCATGCGTTTTGGCCTTCCCTGCCCTGAACCCTGTGTTGATGAAGCGGTGGAAGGCTTACGGCGGGATATGCTCAGTCTCGGTCTTATCT
GGGATGAACCACAACCAGATCGCGACCGGAAACGCCGCATCCTGACCAACTGGTCCCTCACCGACGACATCCTGCCCCGCACACCTGCTACAGACAACGAATTCTGA
>JIALED_00240 sok antitoxin (CssrC)
GGGCGCTTGAGGCTGTATGCCGAAAGCGTTTTGTGGACGGTATACAGCAGAAAGCCCCTGGAGATTTTTTTATCAATCAACCAAGGGCTCTACTGTAATGCCTAGACAACATTATAGTAGCC
CGATAACCGCCGTAAGGCAATGGAGGGGCTATGATG
>JIALED_00245 Membrane permeability altering protein
ATGCCACAGCGAACGTTTTTAATGATGTTAATCGTCATCTGTGTGACGATTCTGTGTTTTGTCTGGATGGTGAGGGATTCGCTTTGCGGACTCCGGCTCCAGCAGGGAAACACAGTGCTTGT
GGCAACGTTAGCCTACGAAGTTAAACGTTAA
>JIALED_00250 ArsR-gov region gene B
TCAGTTATAGACAGTTCTTTTCCGACCAGAGCCACCCACTCTGTAACGGAACAGTAAAAAAACCGACCTCACCAGTCGGTTTTTTTAC
>JIALED_00255 CNNM transmembrane domain-containing protein
ATGTCAGCTCTGAGGCATGTTCTGTCCGGCATATTGTTCAGCGGGAAGAACAGGTTACAAGTCCTGGCCAAAACATCAGGTGGTGCTGGTTTTGGTGCTCTGGTCGGAGAAGAACTGTCTA
TAACTGACACTATAGAAACCGTTGTGACAGGCAGTGTAAGAGCGAACAACGGGGTTCTTTCTCCAGTGATGGAGAAGCGGATGATTAATCTGTTAATCATCGTTCTCAGGGCAGTAGTTGCC
GTTGCAAACGCGCTGATTGCTGTTCTGGAACTGATCCGGGAACTTATCGACTGA
>JIALED_00260 hypothetical protein
ATGAATCTGGTAGATGCCTTTGTCAAAAAAGTAATATCCGGGCCATATAAAGAATATGGAAAATGGTGGATAGATGTTGAATACATTTCATGGGGCGTTCCCGGGAAAACAAGACTGATGTTT
GAGTCAAAGGAGCAGGCACTGGAAGTTAAAGAAGGGTACAAATTTTTAACGTGA
>JIALED_00265 HEAT repeat domain-containing protein
ATGAGCATGCCTCCTGCGATAGCTAATACTTTCTTGTTTGAAATGATGAAGAGTAAATCAAAAGACGTTACACTTGCTGCGATCTATGCATTAGGTGAAGGCCGGTGTCAGGCGGAGAACATT
ACTCGTGAATTACATAGATTGAGTCAAAGTGATGATATGGAAATAAAAATTGCTGCGATTAAAGCACTTGGTCGAATTTATCGGTAA
>JIALED_00270 Exclusion-determining protein
ATGAAAGATAAAAAAGAGACACCTGACAGATTTCCAACTTGGTGGTTGTTATATTATGTATTAAGGAAGGCTTACTTCTTTTTAGGAATACCTTTCTTTTTATTTTGCGCACTGACATCTACACT
GATGTTATTTAGTTCTCGTTATTATGGTGACAACATTGAAGATTATGTTGTCACTTTTGGTAGTTGGTTCTTATTACTTGCGCCAGGTATATGGATGTATTCACGTGCAAAAACAAGGAGAGAAA
AGATAAGAAAAGTTGTACAAACAATAAAAGAGTCAGGCTTCTACTCTCCAGAGAAGGGGTATGAAGGATTATCTTTAACTCAGGGAGCTTATTTCGGTATTGACCTTAAAAATGGCACAATG
TTGTATGTCCGTATCTATCCAGGCAACATTATGGATGTTATCGGTTTTGACATTCATAATTTCACTCGTACTGTAACAGACGACAAAACACTGGAAATTCATACGAAATACATCAACCTGCCAATG
GTTCCTATTCCATCATGGTGTACTCATCCAGAAACCGCATCAAACACCATGCACGCTATGGCAAGTCGAGGATATGATTACCCTGTCGACTTTCCTCGCCTGATTCAGGAGAAGCGTAAGGAG
TGGGAGCAGATTGCAGGCATGCCTGTAGCCGAAGTATTTTAA
>JIALED_00275 DotA/TraY family protein
GTGAAAATTTTACTTCGCGCACTCTGTGCAGGCCTTGCTATATCCTCACTGCCGGCAATGGCTTCAGTGACCTATCAGGATATTGTGTCTGCCGCAACCAATCCTGACGATCTGTCTCGCCAGG
CGCTGGTCACGATATTCGGTGATGTAGTCACCAATCCACTGTCAACCAGCGCCCCCACTCTGATCGGAAGCATGTTCGGGGCGTTCAACAGTATCATCGCCGTACTTGCCGTTGTCTGGTTTA
TGTTCATTGGTATCCGTCATGTTGTGCGTTCGGGGCATCAGGGACAGGTATTCAGCACCGGACGCGATATCGTCGGCACACTGAGTGTCGTTGCAGGTTTTCTGATGATCGTTCCAACCGGA
AATGGCTGGAGTATCGCTCAGCTCATTATGTTATGGGGCGCATCGATAATGGGGGTTGGGTCCGCCAATGTGATGGTGCAACTTGCCGCTGATAATATCGCAAATGGTTATTCAATGACTGTAC
AGCCAGTTCAGGCATCCACACAAACAGCAGCGCGTGGCATTTTTGAAATGGAACTGTGTAAGTATGCGGTTAACGCAGGACTAAATGATTTTAATCAGACGGCCAAATCCTCGACTTCACTG
ATGACAGAATCAGCTAAAACCGCCAGTGGTAATTACACTGTCACGGTATCCAATGGCAGCGGTATCTGTGGTTCAGCCAGCCTGTCAGTAGAAGGAAACGGAACAACAGATCAATCGACGA
TTGGAAAATTCTTTAATCCCTTCAGTAAAAACGAATACAGCGGTGTTATCTCAGCACAACGTGCAGCCATGGATAATATGATTAGCGACATGGACAACGCTGCCAGTGAGTTTGTGACAACAT
TTCTGGAAAAAAGAAATTCTGGCAACGGAACATTGCCGGATATCGAGACACGGATTCAGAGAGCAGCCGACGAGTATGAACGTGCTGTACAAAAATCGCTACCCACTGATAACGGAGAGC
AATCCAGAAAAGAGGCTCTTAAATCTTATCTCACAACCTACGGGTGGGTAACGCTTGGCGCATGGTATCAGACCTTTGCCACTGCTAACCAGCGTCTGGCCGAGCTGGCTGACAGAGCTCCA
GCTGTAACTTCAATGTCATCACTGGGAGAAGTTGGTGATACTGACTTATTCAGTGCAGTCATGAGTGCCTACAAGACTCAATTACAGAACACCACCTATACACCACCTCTGGGCACTGTTACAT
CAGCCAATGAGCAACGAATGGCCAATGCGCAAGATCCTAAGTCTGCCTTATCTGAAATGATGAGCTTCTTTGTCTATCGAACCAACTGGATAGCAACAGAGATAAGTGGCACAGGAACAACA
AGCGATCAGGTCAATCCATTGATAAAGATGAAAAATATTGGTGATTATACATTAGGAACAACAGAGATTCTCTGGAGTGGATATACAACAGTTAGAGTTCTCGCGACAGTAGGTGATAACTCT
ATTTTCGGAAAGGTTGCCAATTTATTCTCTGGATTACCTAAAGGATTAGTTGCTCTGCTGGATGCTGCAGCTCCCCCAATATATTTCTTGTTGTTCCTGTTGTTTTGTGCGGGTTTTTCCTTATCA
ATTTACCTACCATTTATACCATTCATTTTCTGGATGACAGGCATTGGGAACTGGATAGTCAGTGTATTAATAGGATGTACCGCCGGACCTCTGTGGGCTGCAACACATCTCGGAACATCAGAAG
ATAGAGGAAGTCGGGCAGCCTACGGTTATATATACTTAATTGATAGTATGATAAGGCCGCCCATCATGGTCTTTGGTTTTTTCTTTGCTTCTGTTGCCATCATCGCCGTAGGCACAATACTGAAT
GCATTATTTGGCGCTGCCCTGGTTAACGTACAGTTCAATTCGCTGACCGGTATTTTCAGTCTTGCTGGTTTCTTGCTGATATACGCCAGAATTTGTACAACCTCTGTAGCTGCCATATTTGCCCT
TCAGGCCTACCTACCTGATCATGTTATTAATTTCCTTGGTGGGCGTGAGGGAGCCAACACTCTTGGCAGCATGGCAAATTCTGTTAAAGAAATATTTATTGGAGGAAGTCGCAATATCCGACA
TACTCCTGGAATGAAAACAGATCGTCTTAAAGATAAAGGAAGTGACGACAAGGACGGAATAAAAGGATAA
>JIALED_00280 IncI1-type conjugal transfer protein TraX
ATGAATAAGTTACCGGAAAATTCAGACCAGACGAAAAACGTCAAAAAATCACCATCCATAACCCGCATAGCCACCCGGGGGATTATTAACATTTTTGTTCCTCTGATGGAGACTCGCCTGATA
ACGAATTCTGTACAGCATATGCTGGAACAACAAAAACGCAGACTGGACAGATTTCGTCAGGTAAATAATAAAAAGGCGCAACTCCACCTCACGTGGGAAGAAGCTCTGCAGGCCAGCCATA
TGTCAGTAGACGATCTGGATCGTCGTTTCCGCCGGCGCAGAACAGTATGGCGATTCTGTTGCTGGAGTCTGCTGGCCATCGCTTTGTTTCTCAGTGGTATGCTCTTTGCCGCCTCTTCATTACC
TCTTACGACTCTGGTACGCGCCATCAGCACTCTTGTGCTGATACTGTCAGGCGTCGCTCTTTGTGCTTCCAGAGCACTGATTGTTACATACCGCCTCTGGCAGTTGCATGAACGAAAAGTCAG
CGAACCCGAGCAGGGGACTTTCCGTGATTTTCTGAACGACCGCAATGGCTGGCGCAATGCCACGTTAATTGCCGTAACCAGTAAACAATACTGA
>JIALED_00285 IncI1-type conjugal transfer protein TraW
ATGCAAAGAAAAACATTACTCGCAGCCCTTATAGCCACCCTGAGTGGAACGGCATGCCAGGCTCATGCTTATTCAGTCACCGTCGTCGCCAGCCGGCCTGTTGAAGAGCAGGTCATCCCCA
GAATGGAAGCCATCAAAGATGTCCTTGGCAACATACTGAGTACCCAGACAGCCACGGGTACCGCTATCAATCAGAACTCTGAAAAGCTGGCAAGTGTCATTGCCCAGAACGGACAGGCGA
CACGTCAGCAGATGATTTTCAGTAATGAAACTCAGCGCCTCGAGGAGGCACGGAAAAGCTTCACCGTGCCAGACTCCATCTGCAGTGAATCCGCATCAGGGATTGCAACAGAAAGCAAAA
GTGCCTCAGCATCCGCAGCCTCAAAACTCAGTAAAGGTGGCGGTGTGAGCAACCGTAGCATCCGCGACCGTCTGGCCAGCGCAGCAAACTCTCCCGTGCGGGAAGCTTATGATGGCGCGG
CCATACACGCCAGTTACTGTACAGAAGCAGAATATGCACGTTTTGGAGGAACCGCCGTTTGTCCTTCTGTGGGCGAAATACCAGGTGGAGACAGTCAGGTACGCTCGATTTACCACGGCGC

AGGTACAGCGGACACACCTGCAGCACTGACATGGGATCAGAAGCAAATAGATGCAGCGACGGCTTACATGAAAAACACTTCCCGCCCCTCTGCCGGTCGTGCTCTGGGTAAAGGAGAAGT
GAATACCCAGAGTGGGCGAACCTACGTCGGACTCCAGAACGAATACAACGGCATCATTGATTCTGCGTCGAATCCCCAACTGACGCTGATTGCTGACAGCACGCCTAATGAAAGCACACGT
AAAGCACTGGCTGAAACACTGCAGTCAGACTCAGCGGCCGCTTACTTCGACCAGGTGGCTTCACCAGAAGCAAAAGCACGGGGATATATGAGTACCCGTGAGTTTGAAGCTTTTGAGGCT
GGCCGGCGCTACGCCAACACAGCATACCTGGTGGATTTACAGGAAATGCAGGGAGATAACCTGCTCCGTGAACTGGTAAGAATCACTGCGCAAATGAACTGGCAACTGAACGACCTGAAA
GAACAGATCCGCCAGGGTAATGTCATCTCTGGACAGCAACTGGCTCTGACCGCCCGCCAGTATTATGAAAAACAACTCGGTAGCCTTGAGAAAACAATCAATCAGGCAAACGCACGTTGA
>JIALED_00290 IncI1-type conjugal transfer protein TraV
ATGTGTACAATTCATATCACCCCACGCTGCCGGAGACCTGCCAGAGGGCAGGTATTTCTTAAAAAACTTGAGGTAAAAGGTGACAGTTTCTCATTCACTCTGCCGTCACGTGTCTTCAGGTTA
CATCCGGCCCCCAATCCCAGAGTGATATACGCCTCGGATGCCAGAACACAGGGAAAGGTTACAGGCATGCGGTTGTTGTTGCCGTCCAGCACAGATGAGCTGATAACAACGGCAATATGGC
AGGTTTCTGTCCCGGATGAAGCACCATTCGACGTTCAGCATGTCATCCAGTGGATACTCCCTTCACCGCTGGCTTCGCTGATGTCCGATGATACCTGGTTATGGCCATCCGTTCCCGGAACGC
CGGCACTGAGTGCCGGTGACTGGCCTGTTATGGATACTGACCCTTCCACCTTACGGCGCGTCAGACGTCGTTCTTTCAGCATCATCGATAACCGGAACAGGAAAGGTTATATCACCAGGCGA
TTTCAGTGTTTCCCACTGGCCCCACTACCTGAAAACAGAAAATACGACAGCCTTCCTGACCTTGTGCTCCATCCCGAGCAGGAGACAATAAAATGCAAAGAAAAACATTACTCGCAGCCCTT
ATAG
>JIALED_00295 IncI1-type conjugal transfer protein TraU
ATGAACATAAACCGTTTAATCTTCACTATCGAAGACTGTCTGAACACGCTATCCCGTTTCTCTGTCGCAAGCAGTTTTGTTGAGTATTGCGATTTACGTACTGTAATAGGTCTGGACAGACAGG
ATCGTGAACGTCGCCCATGGCTGAATTCGCCCTATATTGCAGCCACAAAGCGTGGAGAATATCTCTCTGTCTTTGAAGTCAGCGGTGCTTTCAGGGAAATGGATGAAGCATCCGACCAGACT
GGCCCAGGTTCACTGGAATCGCTGATCACCAGCATGAGTGACAGCCTGAATACGGCATATAAAAACAGCGGTCATAAAATCAGCTTCGTTTTTGAGCGTGATCCTGACATGGGGAAAGAAG
AAATCGAAGACATGGTCGCCCCACAAAAGCGCTCACTGGCTAATACAGGTATCCAGTTACAGGATGTGGTCGATGAGAAAGTCACAACACTGTCCCCATGGCTGGTCCGGGAGCGTTGCTG
GCTGGCGATCTGGAGTGGACCCGACCTTATCAGCAACAGCGATCGTACCGCCCATGATGAGCTTGTCAGAAGACTGGCTGAACGGGTACCGAAAGCACGCTTTGCCCAGTCCCCCTGGCA
GTGGACCTTATCTGCACTGAAAATCCGCCACGAAGCTTTTCTTGATAATGTGGAACAGGCACTGAGACATTCATCTGATGGCCTGATTCTCCGGCTACTGGATATACACGAGGTCGGGCGTG
AAATACGTCGTCAGACAGAACGGCACAGCACACCGCGTAACTGGCAACCCCACCTGCCTGAAGATGCCCAGCCAGCGGGATATCGCTGGACTGACGATGAATCCGTACTACATGCTCCTTC
ATTGCATTTGCAGTTATTCAACACTCAGGTGACCACACAGGGCAACCTGGTTCAGGCGGGCGGACTCTGGCACGGAATGGTATCGATTACTTTACCTCCGCAAAATCTGCAGACGTTTAACG
AACTGGTCCGTGCCGTCCCACGCGCAGTCCCCTGGCGCATCAGAATGGATCTGATGCCCGGCGGGATGAAGGCTCTTAATCTGAAGAAAACGCTTCTGACTTACAGCAGTTTCATTTCTGCC
GTTCGTCCAATGTATGAATCCGTTATGGCCCTGGCGGCAACGGATGAAAAAGAACCTGTCTGCATAATGACCATCATGGCCTCCACGTGGGGAAAAACACGTGAAATATGCACACGAAACCA
GGCCATTCTGAAATCAGCCATTGAAGGCTGGGGCGTATGTGGCACCACGACAACATTTGGCGATCCCCGACGCGCCTGGGTGAATACCATTCTCGCGGCATCCGGAGGCTCCGGCCCGGTT
CCCCTGTATCCCCCGTTGTCACATGCCATCTCGCTTTTCCCGCTTAACAGGGCAGGCTCTGTCTGGCGAGGCAAAGGGAATCTGATGTTACATACAGAAGACGGTTCCGCAATTGAGGTTGG
TCTGGCGTCATCCCAACAAAATAAACATACAGAACTCGCACCAGGCGATCCGGGACTGGGTAAATCAGTTCTGATTAATACATTGTCGGAAATACAGATATCATCTGCTCAGAAAAACCTGCC
CTTCATTGCCTATATCGATAAGGGATACAGTGCTCAGGGACTGGTTCAGCTTATTCGCGACAGTCTTCCTCCGGAAAGAAAAGACGAAGCTGTCGGTATTATTCTGAGCAATGATCCGGAATA
CACCCGTAACCTTTTTGACGTGATGTACGGCGCTAAAAAGCCTATTACGCCGGAAAAGAATTTCATGTCATCGGTGCTGTGTGCTCTCTGTGTCGATACCGGTACCGGTCAACCCTGTAACCC
CGGAGACACCCGACAAATTATCAACCAGCTTATCGAGCTGGCTTTTAAAGAGTACGGTGAAAATAATCCGCGACTTTATCGCGCCTCAACGGAAGAACTGGTGGATTCAGCACTGCAGGAC
TCGGGACTGTATGAAAAACATGATGCAACCTGGTGGGCCAGATCAACCTGGTTCGAAGTCCGGGATATGCTCCACAATGCCGGGTATATCATGGCGGCTCAGCGGGCACATTATCAGGCAAT
GCCCCAGCTTCCCGAAGTGTCCTCGATGCTGGGCCATACCAGTCTCAGGGATGTTTTCGGTACTGTTCAGCGTGATGGTTCAAACGAGCTTCTGCTGGACTACATCCGCCGCGCCCTGGAGC
AGGGACATAACGACTACCCCATGATATCCGGGTATACCCGGTTTATGATCAACCCGGAAACCCGGGTCATCGCTGTCGATCTGAATAATGTTGCCGGTGACAAAACGCCTGCAGGCAGGCTT
AAAACCGGCATCATGTACCTGCTTGCAGGTCAGATCGCCGGTGGTGACTTCACCCTGCCACAATACAGGGATGAAGTGTTAAAGCAGCTTCCACGGGAATACCATGAAATCGCTCTTAAACG
TATTAACCAACTTGATCAGGAGGTTAAGACAAAAGTTTATGATGAACTGCATAACGCCCGGGGCATTGACTTCATCTGGGAAAATCTGGATACCCAGGAGCGTGAGCAACGCAAGTTTGCTA
TCCGCACGGTTCTGAGTACGCAGTATCTGAGGGACTACCCGGAAAGCGTTCTTAAGTCAGCAAATACTCTCTGGTTACTGCGCTACAAACCCGAAGATATCCCCGTACTGAGGGATAACTTC
AACGTTCCTGAATTTATGCTGAAACGCTTCCTGAAAATGCCGGAAGGCCCGGCACCAGATGGTAGTGGTGTTCCTGTTCTGGGAGTATTCAGGGTAAAAAGTGGCACGCTTGCGAGAATAC
TCAAATTTACCGTCGGTCCACTGGAGCTATGGGCGCTGAACTCATCGCCAAAAGACAGTGCCCTGCGTAAAACACTGACAAATAAGTTAGGTTCAGTTCGGGCACGTAAGATTCTGGCCGA
AAACTTCCCCAGAGGTTCTGCAACGTCATTAATTGAGCACCGGGCGGGCCAACATAACAGTGACAATGTTATTGAAGAACTGGCCTCGGAGCTCATCCGAAAACAGGGATACAATCTCTGA
>JIALED_00300 IncI1-type conjugal transfer protein TraT
ATGCAACACAGCATTAAAGACTTGTGGTTGTATCCCTTTCCAGAAATTGATGTGGTTCATACACAGGAGCCACTGTTACCGGAACCGGAATTAACAACGCCCGGTCGATGTATCTGCTGCCGG
CAAAACGTCAGGCACCGTTTCAGACTGGATGACTCATGGCCACTCAGACAGCTTACCGACACAATCAGTGACACACGAGTTCGTCTTAATAAGGCCACAGAACATCTGGATAAGTTAAAAA
AGCGTGGTGAACCAGTCGCAACCGGCGAAAAGGAAAAGTACAACACGGCAGTAAAAGCCGCTGAACGGGCGCTTGAGCAGGCCCGTCTGTCTGCCAGACGTCTTTCACTCCGCCATGTA
CAGAAAGCTGAAATCACCAGTACAGAGTCGTTGTCAGAGAAAGAGCAGGAACTGTTTCATGAAGACGGCCCGCCTTACAGCTTATGTGCTTTCTGCCACGCCTGGCATTCACTGAATGGTT
ATGCCGCAGCACAGGGCGTTATGGTGTGGCTACCCGACCTTCATCCATCCACGGTCGTGGCCCTTAACCGTCGTTCATTACAGGAAGTTTTCAGTAATGATAAGTTCCGGGTCCGGCGGGGA
CGGGAAGCGCTCTCTGCACTGATGCAAAACAGACTGGCGGTTGAGGATAAATTCCGCAGTTTCCGCCCGGCCGATTTCGCTGACGTTTTTCGTCGCTATCCACCTTCCGGTCGTTCGCCCCT
GCGTGAAAAAATGAACGGGATTGCCCTGATTCTGACACCAGATTCATTCATCAAAAAAGAATACGTTGATTGA
>JIALED_00305 putative conjugal transfer protein TraS
ATGTCTGCCTGGAGCCCGGAGCACATAACGTTACGACGAACAGAAAACGGCAATGTGATTGCCCACGACAGTCGGGACGATCAGGAATTCATTTTTGCAGAATGCTGTTCCGATGAAATCG
CAAACATCATTCTGGAAGCCGTGAAACGTTACACCAGTACGGAGCCTGCTCATGCAACACAGCATTAA
>JIALED_00310 IncI1-type conjugal transfer protein TraR
ATGTCACGTATTCGTGTCGTAGCGGCCCGTCTGTACGCCGCATTTATCCACGCACAACTGTTTGTAATGTCATGCCGGAGCAAACTGACTAAGTTCCTGCTGCTCATTCCCATGATGTTGCTGC
CCAAAGCCGTTCTTGCCGATGGAGATCTTGCCGATATGGTTCGCAATGTCGAACAAGGTGCCAAAACAGCACAGTCATCATCACTGACTATTGCGCAGTTCATCGGCGTAATTGCGTTTCTCG
GGGGCCTGATTGGGCTGAAAAAAGTTGGGAAACAAGGGGGGATGGGACTGGCTGGTTGCATTCTATCTATCGTCATTGGGGCGCTACTTGCCGCCGGACCTGAAATGATGAGTCGCAGCC
AGAAACAGTTGGGGATTTCCTCAATTTCCATTGGCTAA
>JIALED_00315 Conjugal transfer protein TraQ
ATGAACATGGATGCCCTGACCGCCATTGAGAACTTTGCCAGCAGTATTTTCAGTGCCGGAATGGATTTCCTGTTTACCTGGGGAGAATTCATCGGGGTGATCAGCATGATTACCCTGTTTGCC
CGCGCCCGTTCAGCCGGTCCGGTAAAAATGTCTCCCGGAAAATTTATTGCGGGGATGTTAACCAGCTGCATGCTCGTATCACTCCCGGCAATGATTAACGCCGGAGGCGTACAAATGGGATT
CAGGGCTGACTCATTTGGCCCGATTGCATATGTACAACCTCAGACGTTTGGCGCTGCTGCTGGCGCAGCAAATGCAGTTCTGTCGCTGGCCAAGCTGGCCGGTGTTGGATTTGTAATGAAC
GGGATATCCATCTGGCGCAAGGCCGGACTCGATGGACATACCGCACTGAGTGCCAGTGAATCCGTCAGTAAGGGAAACGTCAAATTTATTGCCGGCGTCCTGCTGGTCTTTATCGATCGTGT
CCTCAATGCCCTGCTGGCAAGCATTGGAATCGTCTTCTGA
>JIALED_00320 IncI1-type conjugal transfer protein TraP
ATGAAACCTGAAACAGAAATAGATGAAACCGGCAGCTTCGGTGAACCCGAAGAGAAACCCGCCCCCTTCTGGAAACGTTCCGTCTGGGGGATATCTGTAGCAACATGGGGGCTATGTGCA
GTCGTGTTGCTCGCAGCCATATGGTATCTGTTCCTGAGAGCGCCATCGGAAACAGGCATGCCTCCGTTTAATGATGCGGATGCTGGCGTACAGACCTGGCAAACCACAAAAGAATCATCACC
TTCCGTTCAGTCCACTGAGACTATGACTGTCCGGACAGGAGAAGATATGAGCCAGCTTGCCCGGGATGTAAAAACAGAGCTGGATAACCGCGATGAAAAAATTCAGGCAACACTGAATATG
CTTCACGACAGCATTAACAAGCTGGGCGAAGCTATAAAAAAGGATGAAGAATACGCACAGGAAACACGCCGTCAGCTGGATGATATCCGGAGCCGCCTGAACGGAATAATGACACAGAAA
AGCGTCACTGAATCCTCTTCCACACCGCATCCAGCAAAGAAAAAAACGTCGTCTGTACTTAACGGAATGAAAATCATGTCGATGGAAACTGGTATGGCCTGGATTCGCTGGCAGGGTAGTAC
CTGGGCTGTCAGAGAAGGTCAGACGCTGGGCAATGTGGTGATCCAGCGGATTGATCCGACTACCCGGACCATCATCACCAGTGCAGGGACGTTACGCTGA
>JIALED_00325 Conjugal transfer protein TraO
ATGCAGTCAGCCGTGAACATAAACAGTGCTGCAAGTGGCGGTACGCGCAGCGTTACCGAAACGCCTCACTATCGTGAACTGTTAAGAGCGGATAATGAACGAGGGGCTGCGGCGGCAGCC
CGCAATAATCAGACGTTCATTGCCAGTCTTCCGCAGGGGCTTGATATTCCGGACACTCCGGAAAAGCAACAACAGCCTGCAGCAAAACCGGAAAACTATGCTTATCGCCAGGCTTCCGGCA

CACCACAGGAGGATCGAGCAGCCAGTGAAAAGCGTATGGAACGGCTGCAAAAGCTGATTGTAAGAATAAAAGATCAGCATCCTGCCGGTAGTACACCAACCATAGCAACGACTATGTGGA
ATAAATCTCCGGCAGAAACAACAGGGCAGAATGGTACGCAGCAATTCGCATTACAGAATGCCAGCCTGTCCACGCCTGTAGCAGAAAAAGGGATACAACTGATACCTGCCCTGACCCGTATA
CCGGCATATATTGATACTGCAGTGGATTCTGATAACCCGTCATCCAAGGTTATCGCAACCATTCCCGCTGGCCCCTGGGCAGGGGCAACGCTGTTCAGTCCGGGAGTCAAACTGGTGGGTAA
CGGCGTGGAGATCCATTTCGACCGGATGAGCTGGAACGGCATGGACCTTAAGGTCAATGCATATGCTCAACGGGAAGACAACCTGATGTCATCCGTGGCCAGCAATGTGAATACACGCTGG
TTTAAACATATCATTCTGCCGTCAGTGTTAGGAGGTGTGGGCAGTATTGGCACACTTTATAAAGATGCCAACACGCAGGTTATTCAGGGTAACTATGGCACGGTTACCGGACGCGTGGGAAT
GCCCAGTGGTGAAGCTGTTGCGGGCGTTATTGCCGGTGGTATGGCGGAAAGAGGAAGTCAGATATTAACCCGCCAGGCAGAAAGTGAGCCCTATAAACAGGTAGAAGTGTATCAGCATGA
AGTCGTATCAATTCTGTTTGTTGACCCGGTAATGACAAACGACGCCAGGTCATCTTCCCTTTCCAGTGGCATTTCCCCTTCAGTTAATCGTACATCACAGGCGGAACAACGCTCGCAGGCACG
AATGCAGACAGCCATGGAGCAAAGAAAAGCTGTGATGCAACGTCGGTATGATGAACAACCGGAGACACCATAA
>JIALED_00330 hypothetical protein
ATGAGTGCTGAACAGGATGCCGGTAAATCCGGAAAGAAACTGGCTGCCCTGCTTGGGCTGAAAGCAGCATCATCTTATTTGGCGGCAGCTATATCGCGTTTTCCAAAGTATCAGGGAGTAA
CAGCGATATGCAGTCAGCCGTGA
>JIALED_00335 IncI1-type conjugal transfer protein TraN
ATGCAATCACGTTATTTACTGAGCACACTGCTCCTGGTGTGCAGCGCCGCCACGTCTGCCGATAACGCAGGATGGCAGAACGCACGCACACCACAAACGACAAATTCTGCCAGCGATCACG
TTCAGAACGCCTCCCAAAACACCGGAGGTGTTCCGGCAACACTGGTAAAAGGTGAGCTGCCTGCCCCCGGGCAGGCCAGCCCGCTGGTACAGGATGCGGCAAGACTTGATTCTGAGCTC
TCCGCCGATGAGATTCGCTCACTACGCAGTCTCATGGCGGATAACGAGCGGGCCATCAATGCGCCCATCACCAGTGTTGTACCCCGTATCAGCTCTCTGACAGTTAATCTGTCCCCCGGTGCC
AGCCTTCCTCTTGTTCGCACAGCCATGAACAACCTGAGCGTAGTGACATTTACCGATATCAACGGTTCACCATGGCCACAATCCGATCCTCCTTATAACGCGGCACCAAAACTCTTTGATGTGC
AGTACAACGAGAACATGGTGACAATCACACCATTGCGGCCATGGGCATCCGGAAACATTTCTGTCTACCTGAAAGGTCTGTCGGTACCGGTCATCCTGAACGTAACCAGTGGCGAAACAGA
TACGCCGTCATCCAGTCAGGAAATGGACAGTCGTCTGGATCTGCGAATACCACGGCAGGGACCAACCAGCCCGGTTGTCTCCATCCCGACGGATAAAATCGCCCTGCATGATGCAACATTAC
AGGCATTTCTGGATGGTATTCCTCCCCGGGACCCTTCAGTTAAGAGGCTGAAATTCACAGGTAATGTCCCGGATACAACGATCTGGCAACACGGCGATGACCTTCTGGTTCGTTCCCGGGCG
ATATTGCGCGATGAATTTGAACAGACCTTGTCCTCTGCCGACGGAACTCATCTCTGGAAGCTTCCCGTCACCCCACTACTGACTTTCTCTGTGAATGGCCAGTCCGTTCACGTGACACCAGAA
CTGGAGTAA
>JIALED_00340 Conjugal transfer protein TraM
ATGACCGAAAATACCGAGCTATCCTCGCCATCACATCCGGCACCTGACCCGGAAATACTGAAGAGCACAATCAGGGCTATGAAGCAGTCAGAACAGCGGGCAAATAATGTCCCTGCGCTGA
TAAAAGCGCTTTTATGTACGGGCACATGTCTTCTTATCAGTATTACTGGCAACGCCATTCAGTACTGGCACTCAACGAATGTGGAGCGCGAATATTTCGCTACAGACAATGGTCGTCTGGTCA
GGCTGGCACCAACAAATCAGCCAGCATGGAGTCAGAATGACGCAATGGCCTTTGGCAGCCAGGCACTGGCGACCGCATTCAACCTGGATTTTGTGCACTATCGTTCACAGATTTCCTCCCT
GTCTCCGCGTTTTTCTGACGAAGGTTTTGCCGGATATGTGGTTCGTGAATGCATTACAGGCATCTAACATTCTGGAGACGATCAAGAAAGAGAAGATGAATCTGACCGCCACGACAGGCGCTGGCGTT
CTGGTACGACAGGGACAAATGTCCGACGGCGTATGGTTCTGGACATTTCAGTATCCTGTCCGTATGCGTCTGGTCGGCCAGACAACCAGCAAGCCAGAACAGTCTTTTGTTTTCGAAATCAC
CATCCAGCGTGTTGACCCCAGACTGAAACCATCCGGTATGGAAATCCGGCAGATGATCTCCCGTAACGCCGGACCGAACTCCTGA
>JIALED_00345 Conjugal transfer protein
ATGAACATGCATAAGTCTCTGGTTGCCGTGCTCGGCCTTATCCTGATTTCATCCACGGCAAGTGCTGCAACCTGTCGTGCGGGTAGCGCGGCACAGGCCGGTAGTAATACAGGATATGAGCG
AGCACGTGCAGCTGCGGATGCATGGTCACAACGTGAAAACGATGTTTCCTCATCCCTGCAGTCCTGTCTGTCACGAATCAAAAAAATCAGCATAAACCTCCCCCAGTTCCCCAGCCTTGACG
ACATTCTGTCGCAGCTGGAAAACCAGGTCTGCGACGCGGTCGTGGACAAGGTGAATGAAAAGCTGCCGGGGAATATTGATCCCTGGAAGGACTACAACCTATGA
>JIALED_00350 SogL DNA primase
ATGCCGTCTGTATCCCGCTACCGCACCTGGCTTGCCGTGCCTGCAGATGAAATAGAAGATCTCAAAAAAGCACATCCCCCGATGAACGGGCACACGCCCGTCATCTGGGACAAGGAGCACA
AACTCTGGTTCGCCCGCCCGGGGGCGGACCTGTCCAGGCTGGACCGCTGGCTGCCCCGGCCACAGGACGTCTCCATGAACGGCAGTGATCCGGTCACCGAGTTTGCCCAGGTTCTGGAG
AATGCCGGACTGGTTCTGAAAGAACTGCCGGTCATGGACGGGAAAATCCACCGTGTCCCGACTGCCGATGACAAAAAAGGACAAAAAAGTGGCGCATATCGCGGATTCCTGGACGGCAG
ACCTGCCGGCTGGTACAGGGATTATCGCAGCGCGGATATTCGCCGATCACCTGGACCTTCTCCGGCGGAGAACAGACAGATCCCCGCGCAAGACTCCACCTGAAAGCGCATTCAATGCAA
CGCAGGGAGGACGCGGAGCGGGAACTGAAAGCACAGTACAACCGACAGGCCGCGTATGCCCGCCGCTATGTCAACAAATGGCCGCAGGCCACAGCACATGAATACCTGACCCGGAAAG
GCATTCAGGCTGCACCGGGGGTGCGGGTAAACAACAAAAATGAACTGGTTATTCCCTTCAGTAACCGTAACGGCGCGATACGCTCTTACCAGCGTATTCCGGTCACCGGGGGGAAGGATG
CCCGCATCCTGAAAGATTCAGAGAAAACCGGTAACTGGTTTGCGCTGGGCACACCGCGCAACGGTCAGCCGGTACTGTTTGCCGAGGGCTATGCCACTGCGGCCTCCCTGCACGAAGCCA
CGGGCCTTCCGGTACTGATGACGGTGGATGGAGGCAACATGATTGCCGTTGCCGAAAATGCCCGGCAAAAGTGGACGCAAAGCCCCTTTATCTTCTGTGCCGACAACGATCATGCAATCCG
GGTTAACAAAGGTATCGTCAGTGCAACAAAAGCCGCAGAACTGACAGGAGGAACGGTCATCTTTCCTGCGTTCACCGATGCCGAAAAAGCGCAGGGGCTGACCGATTTCAATGACCTGGA
CGCAAGCCGGGGACGGGCCGCTTTTCAGCATGTCATCAACGCCCAGTTAGAACACATCGGGGTTTCCACCCCCAACAGTAACACCCCGGAAATCCGCGAGGCACTGGTGATCGGCAACCT
CGTTTTTACACCGGTCCACACTGAGGAAAAAACCATGACTCCGACAGAATATCCTGAAACATCACCCGATACAGGGCACAGCCATGACCAGGGACCTTCTCTGCCGTCCGCCACGCAGCAG
GAACAACCAGCTGCTTCCAGCAGTAACATTGCTGACGAAACACAGTCATTTACCAGTCACGCCACTGAAAATAACGGCAAAGATGAGCGGCACGCTGATAACGTTCAGGCGCAAACGGAA
AGCTCTGTAGTTCAGGACTCCCCTGCAGAATCACCGGCGTCCCCGGAAAGTACAACGGCATCTGCCCCTGATGAACCCGCTCATCCGGCGGAACCTCCGGAAAAAGTTGTCTCGGTGGCCA
CAGATAAAGACTGGCGTGAATTTGAAGCCGAACTGTCACAACCGGAAAAAAACGAGTCACAACAGGAATCCGGCACTTCACCTGAAATACCGGCCCCTGCTGCCGCCCCCGCATCCCCTG
AAGACAGTCCCTCATCTGAAACCATGAATGAATCAGCCGAGCCATCACCTTCCCCTGATGCAGTCAAAGAGGAAATGACCGTGATGACGGAGCCTGATGACAAACAAAGCACCGGAATATC
CCCGGATACTCCCCGGGAAGAGGCGGTCAGCCATGTAACTGAAGACATTCCCCTTCAGCCAGAGCCGTTCCTTCCTGAGTCAGATGGCCCCGAAGAATACGAAAACTACAGCGCATATCAG
GAGCTGATGAACAATGAAAATCCTGAGCATCTGCAGGAAAACAGTGATATGCCGTCGCAACCGGACACAGACCACGTTCAGGCAGATGAGTCAGAACTTCAGGCCACAACAGATACTGCA
GAACCGGCTGTCCGGGATGAACCCAATCCACAGCAACCTGTTCAGAGCACATCACCTTCTGATAATACTTCATCGTTTCTCGACAAAGCGCGTGGCTTTTTTACGCGTAAAAAAACGGATTC
ACAGGCACATGAGAACAGTGATCCCACGCCCTCACCGGAAACCACGACGACCACACCGACACCTGACAGTATTGTCTACGCCCCGGAACGTCCTGATGCACCGATATCGCTTAATCTGGACG
AAATCATAAAATCACTGAAGGGTGAAAACGAGCAGACAGAACCGTCCTGTACAAGCTGGATGGCAAACCAGCCTTCATTGACCGGGTAAACCGGCTGGAAATGGTCGACGGTGCCAGC
AATGACGATCGCAGTGTTCTGGCCGCACTTGCTGTGGCCACTAATTTCTATGGTGGCGTCATTGAGCTGACAGGCAGTGATGCCTTTAAGCAAAAAGCGATGCGGTTGATTATCGAGCACAA
CATCAATGTGCGCATGAAACACGCTGATCAGCGTCTGGAGCTCGATACATTACGTAAGGAAATGGCTGTCGGTAAGGATGCTGTTGTCACCCATCAGCCAACGCCGGAACTTAACCGGAATA
CCCCTGAACAACCGGTTGTCCCTGACCCCGTTCAGGAAAAAGAAGCCACACAATCCCCGGCGTCCCCAGTTGCCCCTGAAGCATCAACCGTCAGCACCGTCCCTGCATCATCACCGGCTGA
ACCTGGCAAAACGGCAGATGCGGCACAAGGTGAAGAACCACCAGGAAAACTTCGCCCGGGAGAGAGCGTGACTGCCGTACTGCACAATTTTGGTCGTCGTGCTGAATATGCACCCGGCAAAG
GAGAGAGTTTCTTTGTTGAGCTTAAAAACCGCAGTGGCAGCAAACTCTACTGGGGGGAACAACTGGAAAGTCTGGTCAAAAACCATCAGAAAGGTGACGTGGTGACACTGACACTTCAG
AACCGCGAACAATTCATTCTCCCTGGTGAGCAAAAAGCACGTTTCCGGAATAAGTGGTCCATGGAGTCAGTTACCAACGGGATCTCGGTATCACATGATAATCCGGATAAAGGTCAGCGTAT
TCAGGCGATTCCGGTCGAAACCTTTATGAAAGTGGCTGCGCAAATCAGCCAGGGGTGGCCCGAAGAGATGAAAGCGTTACGTATGCCAGAAAACGTCGGCTCACATCTGTTTATCGGTGA
AGACCGACATCCGGTCTCCGCGCCACAGAACGCAAATCAGGTAACAGAAATAACCAGCGCAGCCCCTGACAAGCTGACACCTGTTCTGGGATCAGTGGATAAGGATACGCGTGAACTCAA
CCTGCTACTTGTCCAGAGTGCCGATGAACATCTGCAGGGTGTGGTCCGGCTGAACGGCACATTGTATCCGGCACTGGCACCCCATCGGCCGACAACAGTCAGTTGGTTATCAATGCGCTGA
CCGACAAAGGTTTACGCTTTGCAGGTTATGGTGAAGCGGTAAACCACGATGCTGACAGCACGAATCGCCCCGCACCGGAGCTTATGCAGTTTCATCTGAAAACCCGGGAAGAACCGCTTTT
TGCTGCTGTCTACACGCCGGAAAAACAACCTGATGCGTTGTACCGGAACCTGGGCTTCGAGCAGTCATGGCAGCAGTGGTCCAACAGCCAAAAGCCGGAGGACAGGCAGGAAAAAACTC
TGCATCAGGATCTCTCCCACAGCCCCGGCCGATGA
>JIALED_00355 Endonuclease
ATGTCAGGTAAAAAAACTATCGTTACTCTGCTCCGGGTATCGCTACTGGCATGCCCGTTACTGTTCACGACACCATCGTTTGCAATGATCGACACGCCATCCGTGAAGGTCGGCTTTTCTCCTG
AAGGAAGCGCATCCGCTCTGGTGCTTGACACAATCAACAGTGCGGAAAGCTCCATCAGGATGATGGCATATTCCTTCACCGACCCGGATGTAATGCATGCGCTGGCAAAAGCGAAAAAACG
CGGAGTGGACGTCCGTATTGTTGTTGATGACAAGGGGAATACGAACCGGGCAAGTCAGGAAGCGATGAAATATATCAACCTGCTGGACATTCCTCTCCGGACTGTAGATGCCTTCCCCATCC

ATCACGACAAAGTCATCATTGTTGACGGAAACACGGTTGAAACTGGCTCCTATAACTTCTCTCGTGCTGCTGCACGCAAGAATTCAGAGAATGTCGTTGTGCTCAAGAATATGCCGGATGTA
GCCGCACAATATCTTGAACACTGGCAGGATCGCTGGAATAAGGGTACAGACTGGAAACCCTGA
>JIALED_00360 Conjugal transfer protein
ATGAAAAGAGAAGGTTTCTGGCGATATGCAACACCGCCAGTCAACATCCAGGGCGTACCTCTGCCGTTCACCATGATTTATCTGTTTATATGCCCCTTTCCCTCCAAAACAACCTTCTGGATTT
GCACCGGCATCATTTTATTTTTCGTTATTCTCGACCGTTCAGGCTGGACTGTCAGGACTATCTGTACACGCATTTTCAGCATTCTGAGAGGGGCCATCGCCTCCGGTCGTCCATGGTGGTATCG
CCACCATACGGAATCACCACGAGACTGGACTGGCCTCGATTAA
>JIALED_00365 Conjugal transfer protein
ATGAATATTCCTGATGAGTTTGGTCTTTTTCCGTTTCAGCGTTTCACTGCAGATGAACTACGGCATTTCTTTGTCTGGTGTGCCGCACATAAAGTCAGTGATGTGGATCTGACCGGCGGGTCA
CCTGTATCTGTCAGTCGCTTTGGCCGACGGGTGCGTTGTTCCAGTGCAACGTTACCAACCACACTCATGTCCTCCCTGATTGATGAACTGTTCGGTCGGGAAGTCATTCCCCGCGTTCTGGCA
GGCAATCCGGTTGACAGGACGATACAGATTAACGGGGATGCATCCGGGCGCTATGGTCTTAAACGCGGAGAACGCATCCGGTTACGCTCTCACCTCATTCAGGGCACATCTGGTGCGGAGG
AGAAAGCAATCTCAATAACCATGCGTGTCATCCCGACAGAAATACCGGACATTCTCTCCATGAACATTGAACCAGACCTGCTTGAAGCAATGGTCTGCAAAAGCGGTCTGGGATTTGTATGT
GGTGAAACCGGGTCAGGAAAATCAACGCTGTGTTCAGCACTGTACCGTTACATTATGGACAATTTCCCCGACGCCAAAATCGTGACATATGAAGACCCGGTGGAATATATCCTGGGAAATGA
AAACGATCTGCTCCCACCGCATCAGGCAGAAATCGGGCGTGATGTGGTCAGTTTTGCTGCCGGACTGCGTTCTGCTGTACGACGTAATCCCGAAATTATCGGTGTTGGTGAGATCAGGGATA
ACGAAACCGCCGACGCAGCGGTACAGGCAGGCAACACCGGGCATTACTGCCTGAGCACCATGCATACGAAATCTCCTGGTGAAACGCTGGCACGTCTTCTTGGGCTGTTTCCCCCTGTCAT
CAGGGATTCGATGGCCTGGGCTGTCCTGTCACTGTTGCAGTTCATTCTGGTTCAGGTTCTGGTCAGAACAAATGACGGTGGTCGTAAAGCCGTCCGGGAATATATCGTCATTAACGATGAGC
TTCGCGACAATCTTTCCGGAATGCCGCATGCAGAGTGGGGGCATCACATTGATGCCATAATCCGGCAGGAGAAGCGCCGTATCCGGGACCAGATTCTGGAAATGTATATCCGGAATGAAGTT
GACCGAAGAGAAGCGATTCTTTTTATCCCGCCAGGGGAGTTACGGTCATGA
>JIALED_00370 IncI1-type conjugal transfer lipoprotein TraI
ATGAAAGCACTCACCTCTCTCCTTTTGCCAGTAATACTGCTTACTGGTTGTTCTTCTTCCCCTACCATCTCGCCAGGTGGTGTCAATACGCCACCACCTGACATTAATGCATGGCTTAAACCACG
AAGTGAAAAGCCGGATAGTCTGTCAGAAACACGCTGGAAAATGCTGACAGACGCAGGTAAAACGCTTGGATTTCGTGGTGGCAAGGCTCAGCGTTCATGGGAGCTGATTCAGGCGCTCA
ACGCCCGGGAAAGCACACTTAATGCCTTATACGATTTCCGGCCACTGATTAGCCCGGAAGGCTGGCTACCACCGGTTATTGATGAAGCACAGGACGTAGCTCATATCACGCCAGATCAAATC
CGGACCTCTTCCCGGGTCTGGACCATCATTCGCCCTGAACGCTTTGTCAGCAATCCACCGGGCTGGCGTGACTGGCTTCTCCGGGGGCTTTCAACAACAGCCACTCCGGGTACAGAAGGCA
GCGTCGTTCCAGAAGACAGCGTGCAACGAAAAGTGTGGGAAACCGCATTACGGCAGGGATGGCAGGAAGGAAGACAAAACGCCGACCTGACGCTTGAAGCCAACCAGAAAACCCTCAC
CCGTGATTACCGGGGCATGATGCTGTACTCACTTCTCTGGCGTCAGGGCATGATCACCCGTCCGGATGTTTCTGATCAAATGCAAACCGTTACAGGTGACGGGAAAAAACTGGTCACTGGA
GATCGTGTTCGTCGCCTTAAAAATCATGCGGAATTCAATCTTCAGAAATCTCACTGGCGTCCGCTTATAGGCACTGAAGGAGGCTCCCGATGA
>JIALED_00375 IncI1-type conjugal transfer lipoprotein TraH
ATGAACCGTATTATACCTGCGGGGTGTCTTGCCGCCCTTTTTCTGGCTGGCTGCAACTCCCCTTCTCAGCATACTCCTGTTCCGCGTAACTACGGTGCTGCCTCTGATGTGGCGGTTACCCGGC
ACACTCAGTACACATTATGGGACAAAGGTGTATTTTCCGGCCAACTTCCGGTTTCAACGGGGTCTCATCTTCTGACAGCTAACAGCGATCGCCTTTCCTTTGACTGGGAAGGTGATGCAATT
GAATTACTGAACGAACTGGCCAGAGTCAGGGGGATGCAGTTTAACTACAATGGTGTCCGCCTGCCGTTACCAGTCAACCTTCACGTCCGGGATATGACGTTTTCCAATACGTTGAGACTTAT
TGAAGCCCAGACAGCCTGGAGAGCAACAATCCATCAATATCCGGGCTTGTTACAGGTCAGCTTCATGCAGCCGGAGAACCGGAAAAAATGA
>JIALED_00380 Histidine phosphatase family protein
ATGAAACCACGTTCTTCTTATTCAAAAACAGCATTCATTCTTTTATTTTCTGTTTTCCTGGTAGCGGCAGTGACAAAAGCAAAAAGCTCGCTTCCCGATATCACCCTGGAACAGGCAAAAGAA
ATTAATGCTGATAACACCGTTATTTTCCTTTTCCGCCATGGAGAACGTTGCGACCGTTCAGATATGCCATGCTATTCGGATAAAAGCGGAATCACTATTACCGGTACAGAGAAAGCACAGCAG
GAAGGTATTAAGTTCGCAACAATCTTCAGTGAATATGATATTTACTCCAGCAATGCGGTAAGAACCATACAAACTGCAAAATTCTTCTCCGGTAAAGAACCCGTCGTTATGGACAGTTTATCAG
ACTGCAATAATGATCTTTATAAGACGCTTGAATCAATCGCAAGAGAATCTCATAAAAGGAACATAGTAATAATGACACATAATCATTGTTTATCATTTCTGGCCAGAGATAGGCTCGGCAAGAA
ATTCAAACCGGCGTACCTTGATGCCCTGATCATGCATTACGATGGTACCCGGCTCATACTTGACGGAAAATACAACAAAGAGGCATAG
>JIALED_00385 Conjugal transfer protein TraF
ATGATGAAGAAAAATCATATTACCCGCACCATCATTGCATCTGCTGTATTATTTTCCTTTAACGCTGCAGCAGCGACGTCATATTTTGAAGCGCGTAACGATGCAATGGGTGGTACCGGGGTAG
CATCTTCCCATTATGGTGTGGCACCTCTGGCAAACCCGGCTTTACTGACAAAGCACAACAGCAATGATGATTTCAGCCTTCTTCTGCCGTCTGTTGGGGCACAAGTTGCTGATCCTGATGATG
TATCAAATAAAGCTGATGATGTTAAAGATGACTGGGATTTGTTTGACAGTGCTGTAGATAACCAGCATGGCGTACAACAGGCAGCGGCAAATCTTAAACATCGTCTGCAGGAATTCCGCAATA
TCAATGCAGATGCTCAGGTAGGTGTATCAGCTGTAGCAGCCATGGCAAATGACACTCTGCCGTTTGCCCTTATGGTCAAGTCTTACGGAACTGTCAGCGTAAACGGAAAAGTAAACGATGCA
GACCTGGATTATCTGGACAAAGTTGCCAATGGAACAATTACCGACGTCGATAAGAATGCCCTGACCTCACGCGCATTCGGGCGTGCAGCTGTAATTACCGACGTCGGAATTTCATTTGCCAA
AGAACTGGAAACGGCCGGACAAAAATGGTCTCTTGGCGTCACACCAAAATATCAGCGTGTGGACCTGTTTAACTACAACGTAACGGTTCGTGATTACGACAAAGACGATTTTGACGGTGAC
AAGTACCACAACACGAAAAACGGATTCAACGCCGATATTGGTGCTTATACAGACCTGAATGACAACTGGACTGTCGGTCTTGTTGCCCAGAACATCATTCCACGCAGTATTGATACTAAAGTC
GTCAATGGCTTTAAAGAAACCTTCAAAGTTCGTCCGCAGGCAACCGCCGGTGTATCCTGGCATAATGACCTGTTTACCACTGCTCTTGACATTGATCTGACTCCTGCCAGTGGCTTCACTTCT
GACAGCAAACGTCAGTTTGCAAGTGTTGGTGCTGAATTTAACGCATGGAAATGGGCGCAATTGCGTGCCGGTTATCGTCAGAACATGGCATCAAACTCCGGAAGCGCCTTCACCGCTGGCG
TTGGTATCTCTCCATTTGACGTCGTACATATCGATGTTTCCGGCCTGGTAGGAACTGACCACGATTACGGTGCAATGGCACAACTTCAGTTCACTTTCTGA
>JIALED_00390 Conjugal transfer protein TraE
ATGAGACTGAATACGACCGGTATTGCGGCCCGAATGATGCTCTCGCTGGACAAAAAAGCAATCGGCGAAAAACTCATCAACGGTCGTCAGATAAACCCGACACCTCTGCAGGTCCGTTACC
CCCAAGGGGTAAGAGAAGTTCTGGGGATAATGAGCGAACAATTAGCCATATCCACCGCTGATTTAACCCGAATCCTGCTTGAGGATGCTCTTCATAATATGTTTATGCCTGCGGATAACACCA
CAGGAAACATAATAAGCAGAATTGAATACATCATGCTGTCGCATGATATAAATGCGCCATTACTGGCAACATTGCTGTCGCCATGGAATATCCGCTCCACGGTTATACAGGACCCGGCCAGACT
TGCAGACTATCTTTCAGCTGACGCTCTCGAACATCTTGCTGAATGTTTTAATCTCAATCCGGACTGGCTGAATGGCCATGAAAACTATCCGATAGCGTTGTCCGGTGAATGGCCAGATACTGCT
GATAATTTCAGAATGCTTATTAGTGATAGCAGCAACACTGAAGTTATTTTCTGGCATAGTTTCCCCTTCGCAGGAAATACGAAAAGAGAATATTGCGGGGTAATATTGAGACAGAAAAAAGAA
ATAAATGGCTCTGTAATATATCCGGCACTATCATTATCACCAACACTTCTGAACGATGAAAAAAGAAAATGGTTAACTGAATATACAACCAGGCAGAATACAACAATGTCTTTGCGACGCGTCA
CGTTGAGACCTGGCCTTGCCGGGAACCTTATTACAGGGCAAATACTACCTGTTAGCTTATTTAATACGTCTTTATTACCCTGGTAA
>JIALED_00395 Tyrosine recombinase XerC
ATGCCGTCTCCACGCATCCGTAAAATGTCCCTGTCACGCGCACTGGATAAGTACCTGAAAACAGTTTCTGTTCACAAGAAAGGGCATCAACAGGAGTTTTACCGGAGCAATGTTATCAAGCG
ATATCCCATTGCCCTTCGGAATATGGACGAAATAACAACCGTTGATATTGCTACATACAGAGACGTTCGTTTAGCAGAAATCAACCCCCGAACGGGTAAACCCATTACAGGTAATACTGTACGT
CTTGAACTCGCCCTTCTGTCATCTCTGTTCAATATTGCTCGTGTTGAATGGGGAACCTGTCGTACTAACCCGGTTGAACTGGTTCGCAAGCCGAAAGTATCCTCGGGACGAGATCGCCGGCTA
ACGTCTTCAGAAGAACGTCGCCTTTCTCGCTATTTCCGCGAAAAAAATCTGATGTTGTATGTCATTTTCCATCTTGCCCTTGAGACAGCCATGCGGCAGGGCGAAATACTGGCCTTACGTTGG
GAGCACATTGATTTGCGCCACGGTGTGGCTCATTTACCTGAAACCAAAAACGGTCACTCACGGGATGTTCCTCTGTCCAGACGTGCCCGTAACTTTCTTCAGATGATGCCCGTTAATCTCCAC
GGCAATGTTTTTGATTACACCGCATCCGGCTTTAAAAATGCCTGGAGAATAGCCACACAACGACTTCGCATCGAGGACCTGCATTTTCACGATCTACGGCATGAAGCAATAAGCCGCTTCTTC
GAACTGGGTAGCCTGAATGTAATGGAGATTGCTGCAATATCAGGACATCGTTCCATGAATATGCTGAAACGGTATACTCATCTTCGTGCATGGCAACTGGTCAGTAAGCTTGATGCCCGCCGG
CGGCAGACACAAAAAGTAGCAGCATGGTTTGTGCCGTATCCTGCCCATATCACGACCATCGATGAAGAAAATGGGCAGAAAGCGCATCGTATTGAGATCGGTGATTTTGATAACCTTCACGT
CACTGCCACAACGAAAGAGGAAGCAGTTCACCGCGCCAGTGAGGTTTTGTTGCGTACACTGGCCATTGCAGCACAGAAAGGCGAACGTGTCCCATCTCCCGGAGCGTTACCTGTTAACGA
CCCTGACTACATTATGATTTGCCCTCTGAACCCGGGCAGCACCCCGCTGTAA
>JIALED_00400 hypothetical protein
ATGCCAACAATCATTCTCTGCAAGCGTGCCAATCCGGTACCTGGGGGCGCTCCTAAAATTCAATTCACAACGCAGACCTACAACATAGCTAAAAACACTCGTAATTTAAGGCTCGGGGTCCAT
GCATATTGCTCATGGACTTACCTGAATGGCTCACCCTTTGGTGGTTTTCAACAGGTATATTCCGACCAAAACAACGTTTGGTATGTGAGTAATTATGCTTGGGGAAATTATGAGTCTGGTGGG
ACCATATCAGTCACATGCCTCAATCTTCCTGGTGCTGGAGCTTAA

>JIALED_00405 Shufflon protein B
ATGAAAAAATATGACCGAGGCTGGGCATCGCTGGAAACTGGCGCGGCTTTACTGATTGTTATGCTCCTGATTGCCTGGGGAGCGGGCATATGGCAGGACTATATTCAAACAAAAGGATGGC
AAACTGAAGCACGTCTTGTCAGCAACTGGACCAGTGCGGCCCGCTCTTATATAGGGAAAAACTACACGACTCTCCAGGGCAGTAGTACCACAACAACTCCTGCCGTTATCACGACAACCATG
CTGAAAAATACCGGCTTTTTGTCCAGCGGGTTTACTGAGACAAACAGCGAGGGGCAGCGGTTACAGGCATATGTGGTTCGAAACGCCCAAAACCCGGAATTACTACAGGCAATGGTTGTAT
CCAGTGGTGGCACACCTTATCCAGTGAAAGCACTTATCCAGATGGCTAAGGATATTACCACTGGTCTTGGTGGATATATCCAGGACGGCAAAACAGCCACAGGTGCATTACGTTCCTGGTCA
GTAGCTTTAAGTAATTATGGTGCCAAAAGCGGTAACGGGCATATTGCCGTATTGTTATCGACAGATGAACTTAGTGGTGCAGCTGAGGACACTGATCGTCTTTACAGATTCCAGGTCAATGGT
CGCCCTGACTTAAACAAAATGCACACGGCCATTGATATGGGATCAAATAACCTGAATAACGTTGGGGCAGTAAATGCCCAGACAGGTAATTTCAGCGGCAATGTGAATGGTGTAAATGGCAC
TTTCAGCGGTCAGGTAAAAGGCAATAGCGGAAACTTTGACGTAAATGTCACCGCTGGCGGTGATATCAGAAGTAATAATGGTTGGTTAATTACTCGTAACAGTAAAGGCTGGCTCAATGAAA
CTCACGGTGGCGGATTTTATATGTCCGATGGATCATGGGTTCGAAGTGTAAACAACAAGGGCATCTATACCGGCGGTCAGGTGAAAGGCGGTACTGTTCGGGCTGATGGTCGCCTTTATACT
GGTGAATACTTACAACTGGAAAGAACTGCCGTTGCTGGCGCATCATGTTCGCCTAACGGCCTTGTAGGCCGCGATAATACAGGGGCAATACTTTCGTGCCAATCCGGTACGTGGGGGACAA
TACTGGTGGAAAACTCAAAGTTACTCAGCTTTCCACCACAGGTTATCTGGGGCAATTCGACTTCTGTGCCATTGCCAGAATGGGCAACGCAGAGGATGCCCACTACTGCCAGGTAG
>JIALED_00410 IncI1 conjugal transfer prepilin peptidase PilU
ATGCAGACAATCGTAGGCATAAGTCTATCCCCTGTTTATATACTGCCATTGATGTTCACATTTTCCATTATTGGACGAACTCTGCTATTCAGAGTTCGTTATTTTTTATCTGATACGGGACATCTCT
GGTATAAGACACACCCTGCTGTTCTGTCAGGAATATGGCTATACAGTATAGCTGTGGCGTTAATTATTTTATCATCATCACCACTGCTCTACCGTATTCATGCTGTCCTTATTCTTTCGTTCATACT
GCAAATGGCGGTAACGGATGCACTGACAGGTTTATTACCAGGAACATTCACCCGACGTTTTTTAATTGCAGGAATGCTATCACAAATCACGACTGATATATGGTGGTTCCGTACAACAGAATT
TGCCACTGCTGCAATTGTCTTATTCTGCCTTCATAAATTAGTTAACCGCCATCGTCTTAATATTGGCACTGGAGACCTGTGGCTTATTGCAGGTATTACCGCATGGTCAGGGTTATATAACGCCAT
ATGGTGTGTGTTGCTGGGCACAGGAGGATTTGTCTTATGGCATTCAACATGGTGCATAAAAGGGCATAAAGAAGGCCCTCTGGGTCCATGGCTATGTTTTGGCCATGTCCTTTTGCTGCTGG
ATAATCTTTATCAACCACTATGGGTAATCTGA
>JIALED_00415 Lytic transglycosidase pilt
ATGTCAGGGGGTACAGTACCCCTCAGACTTATTTCATTAATCCTGCTTTTTTCTCTTATTCCACCCGCCCGAGCTTCATACGTAGCTCCCAAACAGATCTGGACCAACAAGTGGGACAATTGCT
TTGCCGCTGCCGGCGCGCGCTATCAAATTGAACCATTATTATTGAAGGCTATTTCTGCCGGGGAATCTTCGTTGAGGCCGGGCGCAATTAATATAAATAAGGACAAAAAAACAGGTAAAGCC
AGTAGCACGGATTATGGGTTGATGCAAATAAACTCAACCCATATTCCAAAGCTGATAAATATGGGCGTAATAAAAAAGTCTGAGGATTTAATTACCAAACCATGCCTTAATATCCATATAGGAA
GCTGGATACTGGCAAGACATTTTCAGATATGTGGCGTCAGCTGGAATTGCCTTGGGTCATACAATGCCGGATTCCGTAAGGACAGACATGAAACAAGAGAGCAATATGCCAATAAAATATGG
AGAATTTATCGTGACATGAAAGGGATATGTCTTCCCGGACAGGGAGGCAGGCAATGCAGACAATCGTAG
>JIALED_00420 Pilus assembly protein PilX
ATGTTAGTAGAAAATATTAATACCACTTTAACTGGAAATAATAAGAAGAATGAGCCTCATGATAAAGGATGGGCAATTTTGGAGCAGGGAACAATAGCGCTCGTTGTTCTTTTTGTTATAGTCG
TTGTTCTGGGTAGTTTGTATGCGTTGCGTACAAGAGCCAATGTGGCAACTGAAACCGCAAATATACAAACAATTATCACAAGTGCCCAGAGCCTGCTAAAAGGTAGCGATGGATATACATTTA
CCAGCAGTGCCAAAATGACAGGTGCCCTCATTCAGATGGGTGTCATTCCCTCTGGGATGACCGTACAGGGCGACAAAACATCTGGTACAGCAACGCTCTATAACGCATGGGGCGGTGCTGT
AACTGTTGCCCCGGCTTCAACATCAGGGTTCAACAACGGTTTTACCGTTACCTATGATAAAGTCCCACAGGATGCCTGTATCCAGATTGCCACACGAATCAGTAAAACTGGTTTAACTAACGG
CATTACCCTGAACAGTACTGCTCACAGTGATGGAAAAGTCACTACTGAAGAAGCCAGTACGCAATGCAAGGCAGATAATGGCAGCACTGGTACAAACAAACTTATCTTCACCATCAATGGTT
AA
>JIALED_00425 Conjugative transfer inner membrane protein PilR
ATGCGTGAAATGAATTTCAGTCAACGGCTGAGGCGTTTCATTGTCAGAAAAACGTTCAGCGCCCCCTACAGGGTGCAGTTTTACGAAGCCCTGCGTTTTCTGCTGGAAAATAAACAGCCATT
AAAGACCGCACTGGAACAGATGCGGGATGCCTGGACAGACTTTGGACGAAAATGGCATCCCTTTGCTGAACTGGCCACAGACTGCATTGAGTCTCTTCGTGAAAACAGTGGCGAAAACTC
TCTGGAATATACTCTTAGTTTATGGGTTCCTCAGGAAGAGGCCGCAGTAATCAGCGCCGGGATAAGAAGTGGTTCAATTGTGGATGCACTGCAATTCGCCACCACTCTTACAGATGCAAAAG
AACAAATCCATCAGGCTATATGGCAAATGGCTATTTATCCGGTGGGATTGTTAATCATGATGACGGGGACATTATATGTTCTGAATACAGAGCTAATACCTGAACTGAGCAAAATAAGCTCCCC
CGATTCATGGAGTGGTGCTCTTGGTTTTCTTTATGGATTATCTGTTTTTGTCGATAATTACGGCGCTATATGTGCCGTTCTTTTTGCAGTGATCACAGGTTTAATATCATGGTCTCTGCCAAACTG
GAAAAGCCCTGACAGTGTTCGTACTTTTGCCGACAAGATAATGCCATGGAGTATTTATCAGGATATACAGGGAGCAACATTTCTCCTGAATATGGCTGCACTTCTGAAAGCCAAAATGACGAC
GCTCAACTCGCTAAACATTCTTCAGGAGTTTGCATCACCATGGTTAAGCACACGTCTTGACAGCATTATTTACCGCGTCCGCCAGGGGGATCATCTTGGACTGGCTTTGCGTCAGTGCGGTTA
TCAGTTCCCATCCAGGGAAGCAGCCAACTTCCTGTCTTTATTACAGGGAGATGGCGCAACAGAGCTAATCAGCAATTATGGTCAACGATGGCTTAGTCAAACACTTCAACGTGTAAAGAAAC
GGGCAAACGTGATTCGTTTAATTATGCTGATATTTCTTGTCATGTCTCTCATGTTACTGGTTTTTGCCATTATGGATATTCAGTCTATTAGTGACAACAGCATGGGTAACTTTTAA
>JIALED_00430 ATP-binding protein
ATGGATAAATATAATCTGAAGGATGCTCTCCTTTTTATATCCAGGGGAGACACCCATGAAATTCTTATAGAAACAAACCAGCGGACAAGGCCTGACGTACAAAGTAATCTCCAGGAGCTGCTA
AAATTATATCCGGATATCAACCCAAAAGTAGTCTCGTTGTCGGAGTTACAGGAGGCAGGTCAGGATGAGAAAAACAGAGGCCCGAAAGAACGAATTATTCATCTGAAAGACCTTGCTGATG
TCAGTGAAAGTGAGAAGAAAGTTCTATCTTATTTTGAAACTGCCAGAAAACTTGGTGCTTCAGATATTCATTTCTTAATTTCAGAGTCTATTTTCAAAGTTCGCATGAGAATATTCGGTGAACT
GCAGACTGTAGATGAAGATCAACCAGCACTGGGCTATTCACTTTGCGCAACAGCCATTCTGTCAATGGCTGATGTCACTGAAACCAGTTTCTTCCCTCAAAGGGAGCAGGATGCGCGCCTGT
CCCCACAATTAATGCGAAAAATAGGAATATTTGGTGCACGCTACAGTCACAGACCTACGGGAGACGGTCTGATTGCAGTTATGCGCCTGATACCCGATGACGGAGATAAAGTTCCCACGTTT
AAACAACTGGGCTTTATCCCGGAACAAATCCGGTTGCTGAACATAATGCTGCGCCGCCCGGAAGGAAAAATAGTCCTGTCAGGTCCAACCGGTTCCGGTAAATCGACAACGTTACGCAGCG
CCTGCCGTGTATATCTTGACGATAATCAGGGGCGACACCTGCTAACAATCGAAGATCCGCTGGAAGGACAGATACTGGGAGCAACACAGACTCCGATTATCTGCGACAAATCCGACGAAGAT
GCCGTCAAACTGGCATGGAGTCGGGCTATTTCATCGGCAATGCGACTTGACCCTGATGCCATCATGGAAGGGGAAATGCGCGATCTTATCTCGATGATGTCAACCACCTACGCGGCACAGAC
CGGGCACATAGTTCTGACGACATTACATACAAACTCAGCTCTGGGTATACCGGAGCGAATGATTACGATGGGTATGAATGCCGATCTTATTTGCGATGCGCAATTACTGATTGGGATGATCAG
CCAGCGCCTTGTACCAACTCTGTGTCCCTCCTGCCGTATTCCATGGGAAAAGCGAGCACCTGAACTTAGCGACGACGAGCGTGATTACCTTGAGCGGCACTGCAATAAAGATTCACTCTGCA
GTACAGATAATATCTGGTTCCGAAATCATCACGGATGCTCTGAATGTAATCACGACGTAATCATTAACGGCAGAAAGCGCGGTGAAATAGGTAAAGGCCTCACCGGCAGGACTGTAATTGCT
GAGGTGATAGAACCTGATAACCGCTTATTTCAGATTCTGAAAACACGCGGCAAAGTTGCAGCACGTAAATACTGGCTGGAAAACATGAAAGGCATCAGTCGTGTAGAGCATCTTCTTCGCC
GTATCAATGAAGGTCTGGTAGACCCTCTTGAAGCCGACAGAATCATCCCTCTGGACGAAGACGAGCGCCTCAGCATCGATGATGTCTAA
>JIALED_00435 Type IV pilus biogenesis protein PilP
ATGCGTCCAGGTAAATTACTCATTATTCCATCCATATTTTTCTTCTCCGGTTTTTCATTTGCAACAACACAACCTCTTGTCACAATAGGAGAACTTGAAGCACAACAAAACCGGAATATTCTTTT
ACAGGCAAAAGTTCAGGGCGCACAGCTACAGAAACAACTCGAAGAATCAGATGTTACTTCATCATCTGAAACTGTTTCAGGTTTCTCTGGTGTGACTGCCTCTTTGCCATCAGTTTCAGAGC
AGCCGACATCTAAAAACAGAAAGGAATTGCCCGTTATTATGGAAATAAACGGCAAAGATAAACGTCTTAATGCCGTTCTTCGCATGGCTGATGGACGTCAGACAAGCGTCACCACTGGCAG
CCAGCTTCCAGGCACATCCGTCACAGTGAAGTCGATCTCATTGTCTGGCGTCACGTTAAGTGATGGCACCACCCTTACTTTCTGA
>JIALED_00440 PilO
ATGGCTGATGAAGATATCAACCCGGTTGTACTTCTCGCAGATCCTAAAGTAAATCATCGCGTGTGGGCAGCCTGTCTTAAATGGAGTCCTGTCGTAAAAAAACAGCGAGTCCCTTCTCATCAG
AAACACAAGCCCCATGTAAAATCCAGACGGCTTACCTCTCTGAAAGTGACTGTAGGCAGCAGAAGTTCCCGTGGGAAGATCAGCCGCATTACCGGCACAGGAATACTGGCCAGGCCGGAG
CGCAATCATTATTTTTCCCTTGCTCTGGCTTTCTGCTCATGGGTGAGAAATGGATACGGCGTCTTCCGGTACAGCGATAAGGAGCTTTTATTCCTTGCCTCCATTAATGGCCAGCCTGCCGTTAT
GGCTGATCTGTCAGGAAATGATGCTGATGTTGCTCAAAAGGTCAGTCTTTTTCTGACGATGAACGAAGAGCCCCCCGAAAAATGGCAGGTCGTATCTCCTCTGGAACATCCTGATAACTGGG
AAAGTATTATTACCCGGTTATCATCAGCAGATCTCCGCCGTTGCAAACTTACTGTCGGCAATCGCAGCAAATTCACACTGCCTGCCGTACTTTTTTTAGTAGCAGCCAGCGCAGGCACTGTATT
CTGGATGACTCAGCCAGAGCCGGATGTCGTACCGACGCCCGAGGAGATAGCTGCACGGGCAAGATTGCAGTTTAAAAAGCCCGATCCACCGCCAGAACTCCCGCATCCATGGGCATCACA
ACCTGTAATCAGTGATTTTCTGAAAGCCTGCGCGGACCTTCGAAAACCATCACCGGTCGCGCTAGAAGGCTGGAAACTTACCGGTGGAACATGTACTCCCGAGACATTCACACTGATTTACG
AACGACAGCCAGGAGGAACTATTGAAGGCTTTCTGGCAAGAAGCAAAGAAATCTTTAATGTTATTCCTGACTTTAACCTTAAAGATGGTGCACGCCTGGCTTCTGTAACCCGGCCACTGCCT
TCCCTGCCCCGCAGGGATGAGGCAGTCCCTACCCCCTCAGAACAACTGATGCGGGTGTTCACGTGGTTTCAGAAAAAACAGTTAACGCCGGCCATTAATGAAATAGCCATACCGGAACCAT

TACCAGGTAACGATGGTGAACCAGCCCCTGTTCAGAAATGGAAAGAATATCAGTTCAGTTTATCAACGCCTGTAAATCCTGATGAGTTGTTCCCTTTATTTCAGGATACCGGCGTTCGTCTGA
GCAATATCCATTTTGAATTAAACGGCGGTACTTTCAGTTATTCCTCAGAAGGTCATATTTATGCGTCCAGGTAA
>JIALED_00445 Secretin-N-2 domain-containing protein
ATGAAAAAATCACACCAGCGTTCAATGAAGCTGGCGGTGCTCCCCTGCATGATCGCCGTCGCTCTTTCCATCTCCGGATGTACTTTCAGCGAAATCAACAAAATGCAGAAAAAAGCACAGGA
AGACTCAGCACATGCACGGGAAAAGGTATCAGCCCTTTCGGCCCGTAAATCGCAGGCTCTTACCTGGCTCGATAATCAATGGATAAACCCTGTTCCGGTCGCTCAGGTATCAAGAGAGAAAA
AACAAACAGCTCCGGCCTGCTACATCACGCAGGCAAGAAAAGGAGAGATCACTCTGCAGGAACTGGGGCAACGTATTACTGCCGTATGCGGCATCCCTGTGATCATCACGCCTGACGCAGC
CAATTCAACTCTTGAAGGAGGCGCTACCCGCCAGATGACAGGAACACTACCAGCACCAGATGAAAATGGGCGTCTACCGTTAAGCAGCCTGGGCAGCACAACAATGACTACCTCCACTCAG
CCATTAACGCTGAATAACCTCATGTGGCAGGGAGATATCAATGGTCTTCTGGATCTGATGGCCAGCCGGAGTGGTCTGTACTGGCGCATGGATAATGGTCGGATTGTATTCTATCTGACTGAA
ACCAGAACGTATCCACTTCATATGCTGAACACCAAAACCAGCAGCAGTTCCAGTGTCAGCTCTGGCTCAACAAGCACAATGGGGGCAACAGGAGGCCAGGATAACTCAGCATCCGGTGAT
GCAACGTCCTCTCAGAGCACAACCGTTGGTCAGGAATACGATCTGTATGAAGACATCCGGAAAACTATTGAAGCAATGCTGACACCAGAAAAAGGCCGTTACTGGTTATCTGCATCGAGCTC
AACGCTGACTGTCACTGATACTCCAGCTGTCCAGGAAGCCGTCGCACGATATGTGGACGAACAAAACAGTATTATGAACCGCCAGGTAGCCCTGAACGTACAGGTTCTGAGCGTCAGCAAT
ACCAGAAACGAACAGTTCGGTCTGGACTGGAACCTTGTTTATAAATCGCTACATTCCGCCGGAGCAACGTTGAACAATGCAAGCGGAGATTTTACAGGCGCTACATCTGCAGGCGTATCAAT
TCTGGATACGGCAACAGGGAATGCCGCCAAATTCAGCGGTTCCAGTCTTCTGATTAAAGCGCTGAGTGAACAGGGCGATGTCAGTGTTGTGACTTCACAAGAAAGCACTGTCACAAACCTG
ACGCCGGTACCTATCCAGATGGCAGATCAGACGGTTTACGTCGCCCAGTCAGCAACAACAACGACTACGGATGTAGGAGCAACAACAACATTAACGCCGGGCATGATCACCACCGGATTCA
ATATGACCCTGCTGCCTTTAATTCAGAAAACGGGCAATCTCCAGTTGCAGATGAATTTTAATCTGTCAGATCCCCCAACAATCCGTAGCTTTACGTCAAAAGACGGAAACAGTTACATCGAAA
TGCCGTATACCAAACTGCGTTCACTGAGCCAGAAGGTCAATCTGAAAGAAGGGCAATCACTTGTCGTTACTGGTTTCGATCAGAACAATACGACGACAAGTAAAGCCGGTACGTTTACGCC
AGCAAATCCATTATTTGGTGGTTCACAAACCGGGAAAAATGAACGCAGCACGCTTGTAATCATCATTACCCCGACTTTCCCGTCAGGAGGCAACAATGGCTGA
>JIALED_00450 PilM
ATGGGATGGCTGGTCATGGCCGTCGGCCTGACCATTCTGATTATCACTGGTAGCTACCAGAATCAGAAGATGTCTGAAACCACAAACGCCCAGCAATATGCAAGTGCGTCTGTATGGGCAAG
TCAGATACTGATGATTGCTAACAGAATCAATGATATCAGATACGTATCAGGCCAGCAGGACGGAGTAATTTCCTCAGATAAACTGGCTCTCCCTGTCACTCCGGACTCCCGAATTAAACATCAG
TTACAACAGGGGCGTCTGTGGGTATGGATGCCTGAACAACCAGGTCTTGTTGAAACTTTGCGTTCAAAAAGTCGTGGTTCTGCACTAATAGGGATCTTCCAAAACGGGCAACTCACCTGGC
TATCAGGAACTGCAACCGGTCTGACACCCCCGGCAGGCATTACTGCTGGTTCTGTCGTCTACGTTAACTAA
>JIALED_00455 hypothetical protein
GTGCCAGAGACAGTAAAAACAGCACATCCAGTCAGCGAATCCCGACCGTTACCTTCACCAACAATAAGTCAGAATTTCTCCCGGTTAATCATTACAGGCAAGCCTGGTAGCGCCCCTGTAAC
ACTATCGCCGACGGCCCGTAACCGGACCGTCAGTCAGTGGATAAAAACATTACTACCGGAAGGATGGAAATTCAGACAAGAGAATGCAGCCACGCCCAAGCTGAATACCAAACTGGTTTCA
TGGTCTGCCAATGATCAGTGGACCCGTTCTCTGAATCGGTTGCTGGAAGAACAACACCTATGGGGCCATCTGGACTGGGATAATAAAACGTTGACCGTCACAACATCAGCTGCTGCACCGG
CACAGGACACGGCAACCACTATCTCGCCAGCAGCCATGGTAACCACATCCACTTCCCCTGAATCTCCAACAACAGCAAATAGCCAGAACAAACCACGAAATCCATTCAGAGGAAACAGCGT
ATCTTCGTCAACCCCCGCCGCCACCCCGACTCCAACCGGCTCCACAGTCAAGTCTATCCCGCTCATGACCGGAACGCCTGTTAAACCGGTATCTCAGGGTAAAGAGTGGCGTGCGCCGGCA
GGCACCACATTAAGAGAGAACATTATCAAGTGGGCAGAAGAAACAAAATGCGAAAGCATGGCATCAACGCACTGGATGGTCATATGGCCACTTTCTGTAACCGATTACCGTCTGGATGCGC
CTTTAATGTTCCGGGGATCATTTGAATCAGCCATGGAGCAGGTATTTGAGTTATATCGGACTGCGCAAAAACCTTTATACGCACAGGCTTCTCGCATGCAGTGCATCATTACAGTTTCTGATAC
AAGGGATGGTCGCTAA
>JIALED_00460 hypothetical protein
ATGAAAAAAAACTATCTGACACTTATACTGCCGTCATTATTGTCCGGCTGTGCCGGAATTTCCGGCAGCAATAACGTGGCACCATCAGATACGCCTGCTCTGGTTTTTGTTGACGGACAAATC
AGTGAGAGTGCTGGCGTTCTGGCACTTACGCAACAACGTCTTTCTCCACCTAAAATAACTCCCCCCCGAGAGTCAGCGTGCCAGAGACAGTAA
>JIALED_00465 Pili assembly chaperone
ATGAAAAATAATAACGTACCATTATATCTATGGTTACTTTGGTGTACGTTTTGCCTGTACATCACTACCCCGTGGCAATACTTCCGTTATCTATACACTGACCACATATCACTATCTTATATATTATAT
TTCTGTATAGTGGTTATTATCTGCTTTTCCCCACTACTTTTCAGATGCGTGCTTCTTATCGTAAGGTCTGTATCTAAATACAACTTCACATTCTTTGCGAAAAAAAATGGCAGTAAAGAATCAAGC
ATACATCTTAGCCCCTATGCCGATACACGTTGCCCGGAGTCAATGAGGAAACACTGGGATGTGTTACTTTCTCTGATATCAAAAGCAATTAATAATGATGAAACCATTGTTATGCATTCACATCT
CTTTACTCCTGCAAGAATAAATAAACTTATAAAGAGACTGGAATCTTATGGTTTGAATTTCTCTGTTATTACATGCCCACGGAAAACAAGTCTGTATGAAAGATTATCAATTCCTTTTGTATATCT
CTTATTTCAGTGGTCTCTCCCGCATTTGCATTCGGACGGAATGACAGTCGTCATTCGTCCTCTCCAGCCGCCTCACACCAATTAA
>JIALED_00470 Pili assembly chaperone
ATGTCGTTTTTATTTAAATTAACCATCGCGATTTTGATTGTCTTTGCAATCCTTTTTCCGATTTTAATTTCATCAGATAACAAACTGTCCGCACTTTTTTGGAAGATACCAAAAGATTTCTGGGTT
TTCCTGATTATCATGTACGGGGCAATCATCACGGTGGTGTTCATAGAAAGCAGTGAACAGGCACGCATCCGCCGCCAACAATATTTGCAACAGTACAAAATGCCAAATGAGATACATGAAAAT
GTGGTGATAAAAAACAGTATTATTTCAAGAGAAACAACAGATGACGAATCTGACATTAACACCCTGTACCTTGATTCATTTAAAAGTAAAAACAACTGCAAATCTTTTGAAACCAATTCAAATT
TACTGGATAAAAATGGTAAGCCTGTGGAAATAATGGTGTGTGATGTTCAGGAACTGCATTTTTTCGTTCCAAAGAATTAA
>JIALED_00475 Type IVB thin prepilin protein
ATGCCACAGCAACATCCCGGACGACTACAGGTACTGGTCGTCGACACGCACTGCAAACGCAAGCTGTTTTCAACGAAAACACAGACAGATCCCGATGAGCTTGCCCGACGCTTCTGCACGC
CGGATAACTGCCTTGTGGTGGTACTATGCAACAACCGATTTCTGTTCCGCCTGGAACGTGCACCCGGTAGCCACTGCCGCTGGCGCAAGGGTAGCCGCTCCCGCCATCAGCACCTGCAGGA
CTGGCTGTCGTAA
>JIALED_00480 Conjugal transfer protein TraD
ATGAAACTCCAATATCGTATTCCATTAGCTATATATAGCGTCTGGTGCATTATAGCGATTTTGCTCTGCAAGGATGGCTTGTACCAGATGAACATATGGACGCTAGTAGAGATATTGGGGATGAT
AGCTCTCCCGCTGTTAGTCCGTCCGTTTTTCATTCTGCTTAGAATAATATTTAGGAAAAATAGTTTTAAAACCAAAGACATGTCTGACAATCACACAGTATGTGTTTTCCTGAGCGCACTCGCA
GATACTTCCTCAAAGAAGGCGATAAAAAAACACTTCAAGCAATTACTCGAAAAGCTGCCCCCCTTATTACGCCAGAAGAAACGTATCTACATGAAATCCCATCTTCTGACTGAAGCCAGGAC
ACAAAAGCTGATTTGCTCTCTGCGCCGAAAAGGGCTGGATGTAACAGCGGAACGAAGGGAATCCGCCATGAATAGCGTCATGTTCCGCATCCTCATCGTCAGCAGAATCTTATCTCAGTGGA
AAGTGCCTCACATAAATCCCCGCTGTGGTATTGTCATTCTGACCCTGAAAAACGATTCTGGAAACAGGGAATAA
>JIALED_00485 DUF2913 domain-containing protein
ATGACCACGACAGAACATCCCGTGCGCCAGTTGCTCTGGTGTGCACTGAATGCCCTGAAAACGGCACAGGAAAACCACAATATAACCAGTGAAACCGGATTACGGCATTACCTGCTTGAAT
GGCTTTCTGGTGCCAGCAGACATCCTGAGTTCCGTAGTCTCCCTGGCGAAATCATGGCGTTAAAAGCATTGACCGAAAAAGATCGCAATATTCCCATCATCGGAACGCTGAATACGCTCTTTC
TCAGCTCTGCCACTGTCGGGGAATGTCCGTTGTTTCGTTTTCGGGCTGCACTGGGTAAATTACAGAAAGCAGGCTGGCGCACATATGTCTGCCCATGGCCTGAGAGGGTGTTCAATGAATCC
ATTGAACGGGCCTGTTCCGGGAAACGTCATCTGCTACAACTAAGTCGCACCGAAGAGTGTTTTCTACCGACAGGCGATATGAGGGCACCAGTTACGTTGCAGCTGATCACTCCACTACACCG
CAAAACTATGCAGCTTCTGGAAGCGGCAGAGTTAATATTCTCCGACGAAGGATTTCAGGTGGTGAGAGGATACGAACATCAATTCGGGATCGAACGCAGAGAAACCCTCTTTCACACCTTA
TTTATCGGGCTGCCCAGCCTGCCAGAAGATGTCTGGGGTGCAACACAGGAGCAGACGACTATCACATTACACTGA
>JIALED_00490 Conjugal transfer protein TraB
GTGAATATAGAACACCTGAATAACCGGAACTGGTATCTTGCGCAATATAATACAGCCGGCAAAAACAGAGAATCTTTATTCTCATGGCTGAATGAGCAAAACGTCGTACCATGGACTCCATTA
ATAACAAGAAAGATAAGACGGGCAGACTCCAGGTGCTGTTACCGTGAAAGAATATTCGCTATTTTTCCAGGGTACTTTTTTATTCTGGCTAATTTTGACATTCACCCAGTATCAGCATTACGTC
GACATAGCGCCTTCATTGATTTCGTGAAGTTTGGCGGTGAAATAAAACCCGTCAATAAAGATATTGTTGATGGATTAATGAAGATTTACCCGGACCCTGTGCTAAATCCGGGAGCCAGGGAA
GAGCTGAATGCGGCTTCCAGCATATGGCTGACAAAAGCACAATATCAGTATCTCCTCAGAATGGAAAATACTCTCCAGCCTGAGTCGAGGATATCCCTGCTCCTGGAACTGGTATCAAATGCC
GAACATCACGGCTTTATGGAGCGCCTTGTAAACATCCCATAG
>JIALED_00495 Conjugal transfer protein TraA

ATGTCACCTCAACTGATACAGAAACTCCCGGCCATCACTCTGTTGGAAGGCATGTTTCCCGAGCTATCAACAAACCAGCTTAAAGTCTGCGTTTTTTACGCAATGGGTGTTCCTTACGACGCC
ATTGCTCAGAACTGTCGGCTTTCTCCCGAAACGGTACGAACCTACCTGAAGCGAAGCCTGAAGAATTTAAATCTGGAAGGATATGATGCACTTCGCTCTGCTGTGCTGATGCGAACTTTCGT
TTTCATGATTAGCAATACAGCTAAAGAAAACGAAAAAATGTGA
>JIALED_00500 C4 antisense RNA
ATCTATGGTAGCTCAGGCGGGGCAGGCATTAGCCTGGCCGGTTTCCGATGACGCCGGTATTCCTACCCCCGTCTGGGCTATCACCAAT
>JIALED_00505 Replication initiation protein
GTGTATAAATACACGGCACAATCGCTCCGCCATAAGCGACAGCTTGTGGCAGGTCTGAAGAATACTCCATATAACGCAGTACACTGGAGTCAGTTAGCACCCGAAGAGCAGATCCGTTTCTG
GGAAGACTATGAAGCGGGAAGGGCGACCACTTTCCTGGTTGAACCGGAAAGGAAGCGCACGAAGCGTCGTCGTGGTGAGCACTCCACCAAACCCAAATGCGAAAATCCGTCCTGGTATC
GTCCTGAGCGCTATAAGGCGCTGAAAGCCGGGCAGCTCGGGCACGCCTACAACCGTCTGGTGAAAAAGGACCCGGTGACCGGCGAGCAGAGCCTGCGCATGCACATGTCTCTGCATCCTT
TTTACGTGCAGAAACGAACGTATGTCGGTCGCAAATATGCTTTCCGTCCGGAAAAACAACGCCTCCTCGATGCCGTCTGGCCGGTTCTGGTCAGCTTCAGTGATGCGGGCACACATACCGTA
GGCATGAGTGTTTCCCGTCTGGCCAGAGAAATCAGCCCGAAAGACAGCAAGGGGAAGGTTATTCCGGAACTGGAAGTGACGGTCTCCCGCCTTTCCCGTTTGCTGGCCGAACAGGTACGT
TTTGGTGTGCTGGGTGTTTCAGAGGAAACCCTGTGGGACCGTGAAACCCGCCAGCGTCTGCCACGTTACGTCTGGATAACACCGGCAGGCTGGCAGATGCTGGGCGTCGACATGGTAAAA
CTTCACGAACAGCAGCAGAAACGACTGCGTGAAAGTGAAATCCGCCAGCAGCTCATTCGGGAAGGTGTTCTGCGTGAGGATGAAGATATCTCCGTACATGCGGCCAGAAAACGCTGGTAT
CTGCAGCGCAGTCAGGATGCGCTGAAACACCGTCGTGCGAAAGCAGCAGCCAGTAAGCGCGCCAGACGCCTGAAGAAACTGCCTGCCGACCAGCAGATTCATGAGATGGCAGAGTATCT
CAGGAAGCGTCTGCCTCCGGATGAAGCCTATTTTTGTTCCGATGACCATCTGAAGCGAATGGCCATCAGGGAGTTGCGTCAGCTTGAACTGACGCTGGCTGCCCCGCCACCGCACTAG
>JIALED_00510 YacA
ATGGCACAGGTTAATATGAGTTTAAGAATCGACGCTGAACTGAAGGATGCTTTTATGGCCGCTGCAAAAAGCATGGACCGTAATGGCTCTCAGTTAATCCGGGATTTTATGCGCCAGACCGT
GGAACGGCAGCATAATACCTGGTTCCGTGACCAGGTTGAGGCAGGACGTCAGCAACTCGAGCGCGGCGATGTGCTTCCCCATGACATGGTCGAATCTTCCGCTGCTGCATGGCGGGATGA
AATGAGCAGGAAAGTCGCCGGTAAATGA
>JIALED_00515 Type II toxin-antitoxin system RelE/ParE family toxin
ATGCTGGCCAGCCAGGACAGGAAGCGCATTCGTGAGTACATTGCGGAGCAGAACCTGATGGCCGCCATTGAACTGGACGAACGGATTGGTTATTCAGCCAGCAGCCTGGCCGGGCAGCC
GTATAAAGGCCGCAACGGTCGTGTGGAAGGTACCCGGGAACTGGTTATTCACCCGCATTTTGTTCTGGTTTATGAGGTCGACAGCCAGTGGGGAAAAGTGTATATCCTGCGCGTGTTGCATA
CTGCACAGAAGTGGCCATAG
>JIALED_00520 hypothetical protein
ATGAATAATGATGAGCTGGCCACCAGACGTGCGCAGGCGATTGCCGAAGACAGGTGTTTCTCGAAAGGACGTTTGCGCGATGAATTCAGAATGAAACCAGCCCCCGGTGCTTGA
>JIALED_00525 3'-5' exonuclease DinG
GTGCTTGAGCCGGTTAAGTGGTATAAAAACACTTATGGTGGCAGGTTCGCTGTATACCGAATTGCAGACTGTGTTCCGATGCGTGAGAAACGCCCTCTGACGAGTAAACAACAGCTGGCTG
GCCAGCGACTTTCCGTTTTATCCAGACTTAACAGCACCAGTGGCCGGATGGCACGGCAGGCATATGACTGGCTGAGTCTGGCACCGCTCTTTCTGGATACTGAAACGACAGGACTGGATAA
TACCGCGGAAGCCCTGGAAATCGGCCTGACCGATGCGGCCGGTCAGGTGGTATTTGAAACCAGGCTGAAGCCCACGGTCGCCATCGGGGCGCAGGCCGCCGCTGTGCATGGTATCAGCG
AGCAGGCTCTCTGCGGCGCACCATCATGGACAGATGTGGCGCGGCAACTGCGTCATGCAATCGGGGACCGACCAGTAATTATCTTTAATGCCCGGTTCGACATCCGCATTCTGAAACAGACT
GCTGCCGCACATAGCGATCCGGCTGACTGGCTGGAAGAACTGACGGTATATTGTGCGATGGAGCTGGCTGCAGGATATTATGGGGCCACCAACCGCTATGGCACGATTTCACTGGCCTGTG
CTGCCAGCCAGGCCGGACTGACCTGGGAAGGGCAGGCACACTCAGCGATCGCCGACGCACGGATGACGGCAGGGGTGGTAAACGCTATTGCTGCATATCATCTGGAACTGCTGCAGGAA
CAGGCACGGCTGAAAATCTGA
>JIALED_00530 Integrase catalytic region
ATGGCAGGCGCACACGAGTTCAGACAGCACGGTTTCCATGCGCGCCGTCGTGCGGAGATCATCAGTCCGTTGGCGCAGTCGGAGACGGTCGGGCACGAAGCGGCCGATATGGCGGCTCA
GGCGCTGGGCTTGTCTCGGCGCCAGGTATACGTTCTGATCCGGCGTGCCCGGCAAGGCAGCGGCCTCGTGACGGATCTGGTGCCCGGCCAGTCCGGTGGAGGTAAAGGTAAGGGGCGCT
TGCCGGAACCGGTCGAGCGCGTCATCCACGAGCTACTGCAAAAGCGGTTCCTGACCAAGCAGAAGCGCAGCCTAGCGGCCTTTCACCGCGAAGTCACTCAGGTGTGCAAGGCTCAAAAA
CTGCGAGTGCCGGCGCGCAATACCGTGGCCTTACGGATCGCTAGCCTTGACCCGCGCAAGGTCATCCGCCGGCGGGAAGGCCAGGATGCCGCTCGTGACCTACAAGGTGTGGGCGGCGA
GCCTCCTGCCGTGACCGCGCCGCTGGAGCAGGTGCAGATAGACCATACGGTCATCGACCTGATCGTGGTCGATGACCGCGACCGGCAACCTATTGGCCGCCCGTACCTGACCCTCGCCATC
GACGTGTTCACCCGCTGCGTGCTCGGCATGGTCGTCACGCTGGAAGCGCCGTCTGCCGTTTCGGTTGGCCTGTGCCTCGTGCATGTCGCCTGCGACAAGCGCCCTTGGCTGGAAGGACTG
AACGTGGAAATGGATTGGCAGATGAGCGGCAAGCCCTTGCTGCTCTACCTAGACAACGCGGCCGAGTTCAAGAGCGAGGCCCTGCGCCGGGGTTGCGAGCAGCATGGCATCCGGCTGGA
CTATCGCCCGCTGGGACAGCCGCACTATGGCGGCATCGTGGAACGGATCATCGGCACGGCGATGCAGATGATTCACGACGAACTGCCGGGAACGACCTTCTCCAACCCTGACCAGCGCGG
CGACTACGATTCCGAAAACAAGGCCGCCCTGACGCTGCGCGAGCTAGAGCGCTGGCTCACATTGGCGGTCGGCACCTACCACGGTTCGGTGCACAACGGCCTGCTCCAACCGCCGGCCGC
GCGCTGGGCCGAGGCCGTGGCGCGTGTCGGCGTACCGGCCGTCGTCACACGCGCTACTTCGTTCCTGGTCGATTTTCTGCCGATCCTCCGGCGCACGCTGACCCGCACCGGCTTTGTCATC
GACCACATCCACTACTACGCCGATGCGCTCAAGCCGTGGATTGCGCGGCGTGAACGCTGGCCGTCCTTTCTGATCCGGCGCGATCCGCGCGACATCAGCCGTATCTGGGTCCTGGAACCGG
AGGGACAGCATTACCTGGAAATTCCCTACCGTACCTTGTCGCATCCGGCTGTCACCCTCTGGGAACAACGGCAGGCGCTGGCGAAACTGCGGCAGCAAGGGCGCGAACAGGTGGATGAG
TCGGCGCTGTTCCGCATGATCGGCCAGATGCGTGAGATTGTGACCAGCGCGCAGAAGGCCACACGCAAGGCGCGGCGTGACGCGGATCGCCGCCAGCACCTCAAGACATCAGCTCGGCC
GGACAAGCCCGTTCCGCCGGATACGGATATTGCCGACCCGCAGGCAGACAACTTGCCACCCGCCAAACCGTTCGACCAGATTGAGGAGTGGTAG
>JIALED_00535 Transposase
GTGGACGAATATCCCATCATCGACCTGTCCCACCTGCTGCCGGCGGCCCAGGGCTTGGCCCGTCTTCCGGCGGACGAGCGCATCCAGCGCCTTCGCGCCGACCGCTGGATCGGCTATCCGC
GCGCAGTCGAGGCGCTGAACCGGCTGGAAGCCCTTTATGCGTGGCCAAACAAGCAACGCATGCCCAACCTGCTGCTGGTTGGCCCGACCAACAATGGCAAGTCGATGATCGTCGAGAAG
TTCCGCCGCACCCACCCGGCCAGCTCCGACGCCGACCAGGAGCACATCCCGGTGTTGGTCGTGCAGATGCCGTCCGAGCCGTCCGTGATCCGCTTCTACGTCGCGCTGCTCGCCGCGATGG
GCGCGCCGCTGCGCCCACGCCCACGGTTGCCGGAAATGGAGCAACTGGCTCTGGCACTGCTGCGCAAGGTCGGCGTGCGCATGCTGGTGATCGACGAGCTGCACAACGTGCTGGCCGGC
AACAGCGTCAACCGCCGGGAATTCCTCAACCTGCTGCGCTTCCTCGGCAACGAACTGCGCATCCCGTTGGTTGGGGTAGGCACGCGCGACGCCTACCTAGCCATCCGCTCCGATGACCAGT
TGGAAAATCGCTTCGAGCCGATGATGCTGCCGGTATGGGAGGCCAACGACGATTGCTGCTCACTGCTGGCCAGCTTCGCCGCTTCGCTCCCGCTGCGCCGGCCTTCCCCAATTGCCACGCT
GGACATGGCTCGCTACCTGCTCACACGCAGCGAGGGCACCATAGGGGAACTGGCGCACTTGCTGATGGCGGCGGCCATCGTCGCCGTGGAGAGCGGCGAGGAAGCGATCAACCATCGCA
CACTCAGCATGGCCTGTTGA
>JIALED_00540 IS21 family IS1326 transposase
GTGGCGATATTGAGCGCAATTCGACGCTGGCATTTTCGCGATGGTGCGTCGATTCGGGAAATAGCCCGACGAAGCGGCCTGTCCAGGAACACCGTTCGCAAGTATTTGCAAAGCAAGGTG
GTTGAACCGCAGTACCCAGCGCGAGACAGCGTTGGCAAGTTAAGTCCTTTTGAGCCCAAGTTAAGGCAGTGGCTCTCCACCGAGCACAAAAAGACAAAGAAGCTGCGCAGAAACCTGCG
CAGCATGTACCGGGATTTGGTCGCTTTGGGCTTTACCGGGTCTTATGACCGAGTGTGTGCCTTTGCCCGACAGTGGAAAGATTCCGAACAGTTCAAGGCGCAAACCTCGGGCAAGGGTTG
TTTCATCCCCTTGCGCTTTGCTTGTGGCGAAGCCTTCCAATTCGATTGGAGTGAGGACTTTGCCCGCATAGCGGGCAAACAGGTCAAACTTCAGATTGCCCAGTTTAAGTTGGCCCACAGCC
GGGCCTTTGTGCTTCGGGCTTACTACCAGCAAAAACATGAAATGCTGTTTGATGCCCACTGGCATGCCTTTCAAATCTTCGGTGGCATTCCCAAGCGCGGCATCTACGACAACATGAAGACC
GCTGTGGATTCGGTGGGGCGTGGCAAAGAGCGCAGGGTCAATCAGCGGTTCACTGCCATGGTCAGCCACTACCTGTTTGATGCGCAGTTCTGTAATCCAGCATCGGGTTGGGAGAAAGGC
CAGATTGAGAAGAACGTGCAGGATTCCCGCCAACGCCTGTGGCAAGGGGCACCAGACTTTCAAAGCCTTGCTGATTTGAATGTGTGGCTTGAGCATCGCTGCAAAGCGCTGTGGTCTGAG
CTGCGCCACCCCGAATTGGACCAAACCGTGCAAGAGGCCTTTGCCGATGAACAAGGCGAGTTGATGGCGCTACCCAATGCCTTTGATGCATTCGTGGAGCAAACCAAGCGAGTCACTTCA
ACCTGCCTTGTTCACCACGAGGGCAATCGCTACAGCGTTCCTGCCAGTTACGCCAACAGGGCCATCAGCCTTCGGATTTATGCAGACAAGCTGGTGATGGCTGCCGAAGGCCAACACATTG
CCGAGCATCCAAGATTGTTTGGCAGTGGCCACGCTCGGCGTGGCCACACACAATACGACTGGCACCATTACTTGTCTGTGCTTCAGAAGAAACCTGGGGCGTTGCGCAATGGTGCGCCATT
TGCTGAATTGCCACCCGCGTTCAAGAAGCTTCAATCCATCTTGCTGCAACGCCCCGGCGGTGACCGTGACATGGTGGAAATTCTGGCCCTTGTATTGCACCACGATGAAGGTGCGGTACTCA

GTGCTGTGGAATTGGCATTGGAGTGTGGCAAGCCATCGAAGGAGCATGTGCTTAATCTGTTGGGACGTTTGACCGAAGAACCTCCACCCAAACCGATTCCAATTCCCAAGGGGTTAAGGCT
GACATTGGAACCACAGGCCAACGTGAACCGCTATGACAGTTTAAGGAGAGCCCATGATGCAGCATGA
>JIALED_00545 IS21-like element IS1326 family helper ATPase IstB
ATGATGCAGCATGAAGGCCATGTGAGAATCCTCAAATCCTTGAAACTCTTTGGCATGGCACACGCCATTGAGGAGTTGGGCAATCAGAATTCACCAGCATTTAATCAAGCCTTGCCCATGCT
GGACAGCTTGATTAAAGCTGAAGTGGCAGAGCGTGAAGTACGTTCGGTGAACTATCAATTGCGGGTGGCCAAGTTCCCCGTGTATCGGGACTTGGTGGGCTTTGACTTCAGTCAAAGCCT
GGTTAATGAGGCCACGGTCAAACAATTGCACCGGTGCGACTTCATGGAACAAGCCCAGAACGTGGTGCTGATTGGTGGGCCAGGCACAGGCAAGACTCACCTGGCCACAGCCATTGGTA
CACAAGCAGTGATGCACTTGAACCGACGGGTGCGTTTCTTCTCCACCGTGGATTTGGTCAATGCACTGGAGCAAGAGAAATCATCTGGGCGTCAGGGACAAATCGCAAACCGTCTGTTGTA
TGCCGATTTGGTGATTCTGGATGAGCTGGGATATTTGCCTTTTAGCCAAACCGGTGGGGCACTGCTGTTTCACCTGCTCTCAAAGCTGTACGAAAAAACCAGCGTGATACTGACCACCAACT
TGAGCTTCTCGGAATGGAGCCGAGTGTTTGGCGATGAAAAGATGACAACAGCGTTGTTGGACCGACTAACCCACCACTGCCACATTCTGGAAACCGGCAATGAAAGTTACCGCTTCAAAC
ACAGTTCAACTCAGAATAAGCAGGAGGAAAAACAGACCCGCAAACTGAAAATCGAGACATAA
>JIALED_00550 Integron gene cassette protein
ATGAAGGTTGGATCCCAGCCGGGATTGAAAGTGTCGACGTGGGTGAATCCGAGCCGCTCGTATAGGCCACGCAGGTTCGGGTGGCAGTCGAGCCGCAGCTTGGCGCACCCCTGCGTTCG
CGCGGCATGGCGGCAAGCCTCGATCAGCGCGGAGCTGACACCCCGGCCCGCATGTGTCCGTCGCACCGCGAGCTTGTGCAGATATGCGGCCTCCCCCTTGAGGGCGTCGGGCCAGAACT
CGGGATCCTCGGCCGACAAGGTGCAACAGCCGACGATGCCGTCGCTGCAACTCGCGACTAGGAGCTCGGATCTCAGGACGAAGGTCTCCGCGAATGTCCGGTCGATCCGCGCGACGTCCC
AGGCGGGCGTTCCCTTGGCGGACATCCACGCCGCAGCGTCGTGCATCAGCCGCACAACCTCGTCGATATCACCCGAGCAGGCGACCCGAACGTTCGGAGGCTCCTCGCTGTCCATTCGCTC
CCCTGGCGCGGTATGA
>JIALED_00555 hypothetical protein
ATGAACCGCCGCCTCATAGTGCAGTTTGATCCTGACGAGCCCAGCATGTCTGCGCCCACCTTCGCGGAACCTGACCAGGGTCCGCTAGCGGGCGGCCGGAAGGTGAATGCTAGGCATGAT
CTAACCCTCGGTCTCTGGCGTCGCGACTGCGAAATTTCGCGAGGGTTTCCGAGAAGGTGA
>JIALED_00560 sulfonamide-resistant dihydropteroate synthase Sul1
ATGGTGACGGTGTTCGGCATTCTGAATCTCACCGAGGACTCCTTCTTCGATGAGAGCCGGCGGCTAGACCCCGCCGGCGCTGTCACCGCGGCGATCGAAATGCTGCGAGTCGGATCAGAC
GTCGTGGATGTCGGACCGGCCGCCAGCCATCCGGACGCGAGGCCTGTATCGCCGGCCGATGAGATCAGACGTATTGCGCCGCTCTTAGACGCCCTGTCCGATCAGATGCACCGTGTTTCAA
TCGACAGCTTCCAACCGGAAACCCAGCGCTATGCGCTCAAGCGCGGCGTGGGCTACCTGAACGATATCCAAGGATTTCCTGACCCTGCGCTCTATCCCGATATTGCTGAGGCGGACTGCAG
GCTGGTGGTTATGCACTCAGCGCAGCGGGATGGCATCGCCACCCGCACCGGTCACCTTCGACCCGAAGACGCGCTCGACGAGATTGTGCGGTTCTTCGAGGCGCGGGTTTCCGCCTTGCG
ACGGAGCGGGGTCGCTGCCGACCGGCTCATCCTCGATCCGGGGATGGGATTTTTCTTGAGCCCCGCACCGGAAACATCGCTGCACGTGCTGTCGAACCTTCAAAAGCTGAAGTCGGCGTT
GGGGCTTCCGCTATTGGTCTCGGTGTCGCGGAAATCCTTCTTGGGCGCCACCGTTGGCCTTCCTGTAAAGGATCTGGGTCCAGCGAGCCTTGCGGCGGAACTTCACGCGATCGGCAATGGC
GCTGACTACGTCCGCACCCACGCGCCTGGAGATCTGCGAAGCGCAATCACCTTCTCGGAAACCCTCGCGAAATTTCGCAGTCGCGACGCCAGAGACCGAGGGTTAGATCATGCCTAG
>JIALED_00565 quaternary ammonium compound efflux SMR transporter QacE delta 1
ATGAAAGGCTGGCTTTTTCTTGTTATCGCAATAGTTGGCGAAGTAATCGCAACATCCGCATTAAAATCTAGCGAGGGCTTTACTAAGCTTGCCCCTTCCGCCGTTGTCATAATCGGTTATGGCA
TCGCATTTTATTTTCTTTCTCTGGTTCTGAAATCCATCCCTGTCGGTGTTGCTTATGCAGTCTGGTCGGGACTCGGCGTCGTCATAATTACAGCCATTGCCTGGTTGCTTCATGGGCAAAAGCTT
GATGCGTGGGGCTTTGTAGGTATGGGGCTCATAATTGCTGCCTTTTTGCTCGCCCGATCCCCATCGTGGAAGTCGCTGCGGAGGCCGACGCCATGGTGA
>JIALED_00570 hypothetical protein
GTGCTGAGACGCGCGGCTCCACTCGGCGGTGATGTTCCGCAGCGCCAGCCAGATCAGCTTGGTGGCGGCCTCGTCGCTGGGGAAGTGGCCGCGGGTCTTGATGATCTTGCGCAGGCGCG
CATGCACGCTCTCGATTGCGTTGGTCGTGTAGATCACCCGGCGCACGTCCGGCGGGAACGCGAAGAACGGCACCACGCGCGTCCAGGCACGGCGCCAGGCGGCGACCACCGTCGGGAA
GCGCTCGCCCCAGTCGCCGGCCTCGAACGCATCCAGCTCGGCCTCTGCGGCCTCGGCGGTCGGGGCCGTGTAGATCGGCTTGATCGCCGCCGCCAGCGCCTTGCGGTCCTTCCAGCCCGC
ATAGTCCAGGCTGTTGCGGATCAGGTGCACGATGCAGGTCTGCAGCGTGGTCGCCGGGAACACCGCTTCCAGCGCCTCCGGGAAGCCCTTCAGGCCGTCAGCCACGCCGATCAGGATGTC
GTTGACGCCGCGCGTCTTGAGGTCGTTGAACACCTTCATCCAGAACTTCGCGCCCTCGGTGTTCTCGATCCACAGCCCGAGGATGTCGCGGGTGCCGTCGGCCAGCACGCCCAGCGCCAG
ATACACCGCCTTGTTGCGCACCACGCCGTCCTCGCGGATCTTCACCCGCAGCGCGTCGAAGAACACCACCGGATACATCGCTTCCAGCGGCCGCGCCTGCCATGCGGTGACCTCGGCCATC
ACCTCGTCGGTGACCTTGCTGATGAACTCCGGCGACACCTCGACGCTGTACATCTCGGCCAGGTGCCCCTGGATCTCACGCACCGTCATGCCGCGGGCGTACATCGAGACGATCCGATCGTC
GAACCCGGCGAAGCGACGCTCGTGCTTGGGGATCAGCTGCGGCTCGAACGTACCCGTCCGGTCGCGCGGCACGTCGATGCGCAGCGGACCCTCGTCGGTCAGCACCGTCTTGCCGCTGC
GGCCGTTGCGGTGGTTGCCGCTCCCGCCCTCGGCGTCGGCCAGGTGCAGGCCGAGCTCCGCGCCCAGCGCCCGCTCGATCACGGCCTTCTTCAGCTGCTGGAACACGGCTTCGACCCCG
GCCGCGTCCATCGGACCCGGGATCAGCTTCTCGAGAAATTCCTTCGGCAGATCAATGGCCGGCGTGACCACCGGCGTCTTCTTCTTGCGTGGCATACATGCTCCTTGGCAGCATCATGGTAT
GCCTCTCACACAAAATTTCTGACACCCTCGGAAGCGGCCGCGCAAGCGCCGCTTTGGGCACAGATCGGGCACAGATCGGGGGTTCATGCGTGGGTTTTCGACATCGCGGGGCCACGCAG
CGGCTCAGTGCGTAGCCATTTCTCACCGAAACCCGGCACAGGGCCGAGCGCAGCCCTGCGGGGTCGTGGCCGCATCGGATCGGACTCTGGCTTGGCTGCCGGCTGTGTGGCCGGCACCT
CGTCACGCGGCCGGCGGCGGCGCGCGCGCTGCGGGCCGGTAGTGCGCTTGCTCCAGCGCGCCGTGTTTCTCGAAGTGGGCGAGGATGGCGCGAATGGCCTTGGGGTCTTCGATGCTGG
CGACGATCCGCACCGCGCCGCCGCAGTGGGCACAGGTGGTGACGTCGATGGAAAAGACCCGCTTGAGCCGTTGCGCCCAGCTCATCGCACGGCGCTTCTGCTCGGGGCTGCGCGGCTCG
TCGTGGGCGCTTGCGTCAACCGGCACCGCATCGCCCGCAGGCCGCCTGCCGCGCCCCGAGGGCGTCAGCTGCGCACGCAGGTTTGCATTCGGGGCGAATACGCCGTGGAAGCGGGTGA
GATGCGCGCGAGGTGGCGGGACCAGTGCCGCCAGCTTGGCGATGAAGTCCACCGCATCCCATTCGACATGCGTGGTCCCATTTCGCCACGGCGTCTTGAGCTGATACCGCACCCTACCCTG
CGGTGA
>JIALED_00575 hypothetical protein
ATGCAGCGAGAAGCCGCCGACCTTGCCGGCGTCGCCCTCCAGCGAGCCTGCGTCACCGGGCAATGTTTGCAGCGTGACGACCTTGCGGCCAGCGTCGCGACCGGTGGCGATGCGGTAGG
TCATCGAACTCATCCGCAGCCCATCCATGCCGTCGTCGCTACCCGCGCTGTCGGACAGGAACACGGATTCGTCTTCGCCTTCGAGCCAGCCGCGGCGCGACAGGTGCCGGCACACGCGATG
CGCGATGGTGCCGGCCAGTTCCGTCAGTTGCGCCGATGTGGGCGCACGGGTGCGGTGCAGGCGCGGCTTGCGCTGCGGACGCTCGGTGGTGTCCTCGTACACGCCGTCGAGCCACAGCA
TGTGGAAGTGAACATTCAGGTTCAGCGCGCTGCCGAAGCGCTGGATCAGGGTCACCGCGCCGCATTGCGCGCTGGCGTGGTCGACGCCGGCTTGATCGGCCAACCAGCCGGCGATCACA
CGATGCACGATGCCCAGCACCGGGCCGATGGCCTCCGGCTTGCTGGCGAACAGGAAGCGCAGCGGGTACGGAAAACTCAGCACCCATTGCCGCACCGGCCGCGGGCCAAACACCTCCTC
CACCAGGTGCCGCGCGCTCTCGGCCATGCGCCGCGCCCCGCAGCTGGGGCAGAAGCCGCGCTTCTTGCAGGAGAAGGCCACCAGCCTCTCGGCCCGGCACTGCTCGCACACCACGCGCA
GGAAACCGTGCTCGAGCACGCCGCAACGCAGGTAGGTCTCGAACTCCTCGCGCACATACTCGGGCAGCGGGCGGTCTTCGGCCTCAAGACGCGCGATGAAGTCCGGGTAGTGCGCCTGC
ACTAG
>JIALED_00580 hypothetical protein
TTGGCAGCCATTGCGGAATTACCTCAGTTCTGTGATTGCGGGTTGGGATGCGGCGAAGCGGCGGAACCAGCCCAGCACGGCGAAGATGTCGTCTTCCTTCACCACCAGGTACTCGGTGCC
GTCGAGCTTGACCTCGGTGCCGGCGTACTTGCCGAACAGCACCTGGTCGCCGACCTTGACCTTCGGCGCGCGCACGTTGCCGTTGTCGAACACCTTGCCCTCGCCCACGGCGACGACCTG
GCCCTTGATCGGCTTCTCGGTGGCCGAATCCGGGATCACGATACCACCGGCGGAGACCTTCTCTTCCTCGGTGCGCTTGATGACGACGCGGTCGTACAGCGGCTTGATGTTAATCGCCGCG
CATATCTTCCTCGTGGGGCTACCCATCGCCTTTATCGCGCGCCGCCGCGACCGTGCGCGCGCGCAAAAATTCGCCGCCTGATCGCCTCGGGCAATCATCCTTGTCGAGGGTGCCAAGCTGTT
CTTTACTGA
>JIALED_00585 hypothetical protein
ATGTCCGGAATAATCGACGACTCAGTCGCGGCGTTGTTTCGGCTTCAGCGCTGCCGCCGCTGGCGCGGCGTGGCGCGCCTCGAGCCATTCGATGCCGAAGGAAACGATATCGGCCGCGTC
GATCAGCCGGGATTGCGCGCCGCCGACCGGGCCTTGCGCAACCCTGGTGCGGGCGAGATAGTCGCGGGCGATCCGTTCGACCGCGTCGGGGCCGTCGGGCGCGGCATATTCGTCGAGG
ATGCCGTTCGAATCCCAGTCGGACGTGGTGACCCAGACGCGCTTGCCTTCGCGCAGTAA
>JIALED_00590 hypothetical protein

ATGGAGCGCGGTGACTGCATCGGGCCCGGCGCCGATCGACAGGATTTTTCCTGCGTGGGCGAGAAAACGCGCGATCGGCGAGCGGGGGCCATAAGCGGCGCCCATCTCGTGCGGCGCC
ACCAGCCACGCCGCATCGGGCCCGATCGCCGCCATCGATGCGTCGGGATGGGCGCTGCGCCGACACCCCGGATAGGTTCGGATAAATTCGTTGATCGCGCCAAAGCCGGGGTAGGTGGGC
GCATGGTCGGGGTCGAACGCAGGCCAGCTTTGGGCGATGGCGGCGGGCGGCGCATCATGACCCAGCGTCTGTTCATAGGGCGAATCGCGCCACGAGACGAAGGCGAGAATATTCCCGGT
GGGGCCGACGGCCTCGATCAGCGCATCGACAAGTGTCTGCGGTCCGTCCGCCAGCGGCCCGACGGCGCGCAACGACACATGCGGCATCACCATATCGCCTGA
>JIALED_00595 ANT(3'')-Ia family aminoglycoside nucleotidyltransferase AadA1
GTGATCGCCGAAGTATCGACTCAACTATCAGAGGTAGTTGGCGTCATCGAGCGCCATCTCGAACCGACGTTGCTGGCCGTACATTTGTACGGCTCCGCAGTGGATGGCGGCCTGAAGCCAC
ACAGTGATATTGATTTGCTGGTTACGGTGACCGTAAGGCTTGATGAAACAACGCGGCGAGCTTTGATCAACGACCTTTTGGAAACTTCGGCTTCCCCTGGAGAGAGCGAGATTCTCCGCGC
TGTAGAAGTCACCATTGTTGTGCACGACGACATCATTCCGTGGCGTTATCCAGCTAAGCGCGAACTGCAATTTGGAGAATGGCAGCGCAATGACATTCTTGCAGGTATCTTCGAGCCAGCCA
CGATCGACATTGATCTGGCTATCTTGCTGACAAAAGCAAGAGAACATAGCGTTGCCTTGGTAGGTCCAGCGGCGGAGGAACTCTTTGATCCGGTTCCTGAACAGGATCTATTTGAGGCGCT
AAATGAAACCTTAACGCTATGGAACTCGCCGCCCGACTGGGCTGGCGATGAGCGAAATGTAGTGCTTACGTTGTCCCGCATTTGGTACAGCGCAGTAACCGGCAAAATCGCGCCGAAGGAT
GTCGCTGCCGACTGGGCAATGGAGCGCCTGCCGGCCCAGTATCAGCCCGTCATACTTGAAGCTAGACAGGCTTATCTTGGACAAGAAGAAGATCGCTTGGCCTCGCGCGCAGATCAGTTG
GAAGAATTTGTCCACTACGTGAAAGGCGAGATCACCAAGGTAGTCGGCAAATAA
>JIALED_00600 Uncharacterized protein
GTGATCGAAATCCAGATCCTTGACCCGCAGTTGCAAACCCTCACTGATCCGCATGCCCGTTCCATACAGAAGCTGGGCGAACAAACGATGCTCGCCTTCCAGAAAACCGAGGATGCGAACC
ACTTCATCCGGGGTCAGCACCACCGGCAAGCGCCGCGACGGCCGAGGTCTTCCGATCTCCTGAAGCCAGGGCAGATCCGTGCACAGCACCTTGCCGTAGAAGAACAGCAAGGCCGCCAA
TGCCTGACGATGCGTGGAGACCGAAACCTTGCGCTCGTTCGCCAGCCAGGACAGAAATGCCTCGACTTCGCTGCTGCCCAAGGTTGCCGGGTGACGCACACCGTGGAAACGGATGAAGG
CACGAACCCAGTGGACATAAGCCTGTTCGGTTCGTAA
>JIALED_00605 hypothetical protein
ATGCGTGTAAATCATCGTCGTAGAGACGTCGGAATGGCCGAGCAGATCCTGCACGGTTCGAATGTCGTAACCGCTGCGGAGCAAGGCCGTCGCGAACGAGTGGCGGAGGGTGTGCGGTG
TGGCGGGCTTCGTGATGCCTGCTTGTTCTACGGCACGTTTGAAGGCGCGCTGAAAGGTCTGGTCATACATGTGATGGCGACGCACGACACCGCTCCGTGGATCGGTCGAATGCGTGTGCTG
CGCAAAAACCCAGAACCACGGCCAGGAATGCCCGGCGCGCGGATACTTCCGCTCAAGGGCGTCGGGAAGCGCAACGCCGCTGCGGCCCTCGGCCTGGTCCTTCAGCCACCATGCCCGTG
CACGCGACAGCTGCTCGCGCAGGCTGGGTGCCAAGCTCTCGGGTAA
>JIALED_00610 DUF3330 domain-containing protein
ATGAACGCCAATGAACCGAGCACCAGTTGCTGCGTGTGCTGCAAGGAAATCCCGCTCGATGCCGCCTTCACGCCGGAAGGGGCCGAGTACGTGGAGCATTTCTGCGGGCTGGAGTGCTAT
CAGCGCTTCCAGGCGCGGGCCAGCACTGCGACCGAAACCAGCGTCAAACCGGACGCTTGTGATTCGCCGCCGTCAGGTTGA
>JIALED_00615 hypothetical protein
GTGGCATATTTACTGATCCGTTCTCAAGTATTGATACAGGGTTTCGCGACTGATTCCGAATTCACGAGCAAGCTTGGTCTTTGGCTCGCCAGCCTCGACACGTTGGCGCAGTTCGGCAATACG
CTCAGACGACAGGGATTTCTTCCTGCCACGGTAAGCCCCGCGTTGCTTGGCGAGCGCAATACCCTCGCGCTGACGCTCGCGGATCAGGGCGCGCTCGAACTCGGCGAACGCGCCCATCAC
CGAGAGCATCAGGTTCGCCATCGGAGAGTCTTCGCCAGTAAAACTGAGGTGTTCCTTGACGAATTCGATATGCACGCCGCGTTGTGTCAGCGTTTGCACGATCCGGCGCAAATCATCGAGA
TTGCGCGCCAGGCGATCCATGCTATGCACCACCACGGTGTCGCCGGTGCGGGCGAAGCTTATCAGCGCTTCCAGTTGCGGACGCTTGACATCCTTGCCGGATGCCTTGTCGCTAAAAGCGC
GATCAACCTTGACGCCTTCCAGTTGCCGTTCCGGGTTCTGGTCGAAGGTGCTGACCCTGATATACCCAATGCGCTGTCCAGTCATGGAATTCCCTGCAAAATGTCAGGGAAGACTCTATGA
>JIALED_00620 hypothetical protein
ATGCTTATTCTTGGCAGCGTTAAACAGCTTACCCAGGATGCGGTCGTGCAGGTCGATGATTTCGTCGGTGACGGTGGCCATGCCCTCGGTGGCCAGCGCCACGAGAGTGGCGTAGCGCCGT
TGCGGCTCGAATTTGGCCAGGTCGGCGGGTGTCATCTGGCCGCCCTCGCGGGCAATCTTGAGCAGGCGGTTCTGGTGAACCAGCCGCTCGATGCCGGTAGGCAGATCGAGTGCCTGCCAT
GCCTTGAGGCGTTCGATGTGTTCCAGCATATGCCGCGAATTTGGCTTGGCCGGAGACTGGCGCAACCAAGCCAACCAGGTCGTCTTGCCGTTGTCCCGGCGCTTGAGCAGATCGTCGAGG
CGGCGGCGATGCGCGTCCGCCAGTGGTTCGGCCAAGGCGTCGTAGATGCGCCGGTTAGCACGGGTGATCGCCTCGGCACTCGCCCGCTCGACGGCGTTGAGGGCGGGCAGAATGACCG
ACTGCCGCCGCAGGTGCCCGATCAAGGCGCTGGCCAGCACGATGCCTTTGTCGGTTTGCATCGCCAGCTCGGTCAGCATCTGGACGGCCTGCCGGTAATGGCTCATGGTGAAGGGCCGGA
AACCGAACACGGTTTGCAGCTCGCTCAGGTGCTCGCGCCGGGTCTGCTCCCGCTGGCCGTACTCGTTCCAGCTTTCGACGCCGACCTTGAGCTGGTCGGCGACCAGCTTCAACAAGGGCG
GGAACGGTAG
>JIALED_00625 hypothetical protein
ATGCCGAAACTGAGCGTTGACCAGTTCAGCCAACGCTGTCGAGTACGTTTCGTCGCGGGTATGCCAGGCTTGCAGCCAAGCGAGCTTCGCGTAGGTCGTGCCGGGGCAGGACTCGGCCAT
CTTGGTCAGGCCCAGGTTGATCGCGTCGGCCAGGATCGTGGTCAACAACAGGTTCTTGTCCTTGGCCAGATCGCCCGATTTCAAGTGCGTGAAGTGCCGGGTGAAGCCCGTCCACTCATCG
ACTTCGAGCAGCAGTTCGGTGATCTTGACGTGCGGCAGGACCATGGCTGTCTGGTCTATCAGCGCCTGCGCGGTGTCGGGCACCGCCGCATCCAGCGGCGTGATCTTCAAGCCCGACTCG
GTGATGATGGCATCCGGCAGGTCGTTGGCTGCCGCCATGCGGTTGACGGTGGCAAGTTGTGCTTCCAGCAGCGTCAGCCGCTCATGCAGATATTGTTCGCAGTCGGTGGCCACGGCCAGC
GGCAATTCGCTGGACTGCTTGAGGCTGGTGAACTTCTCGGGCGGTACCAGGTAGTCCTCGAAGTCCTTGAACTGGCGTGA
>JIALED_00630 hypothetical protein
TTGCAGCCAGTCGAGGATGAACAGCGTGCGCTCGATGCGGCCCAACTCGCGCAGCGCGACGGCCAAGCCGTTCTGGCGCGGGTAGCTGCCGAGTTTCCTGAGCATCAGCGAGGCCGTCA
CCGTGCCCTGCTTGATCGAGGTGGCCAGCCGCAGGATTTCGTCCCAATGGGCGCGGACGTGCTTGATGTTGAGCGTGCCGCCGATCATCGGCTTGAGCGCGTCATAGGCGGCATCGCCCTT
CGGGATGTAGAGCTTGGTGTCGCCCAGGTCGCGGATGCGCGGCGCGAAGCGGAAGCCCAAGAGGTGCATCAGGGCGAAGACGTGATCGGTGAAGCCCGCCGTGTCGGTGTAGTGCTCC
TCGATCCGCAGGTCGGATTCGTGGTACAGCAGGCCGTCGAGCACGTAGGTTGA
>JIALED_00635 hypothetical protein
ATGACCATTGCCGCGCAACGCATGCGCCGCACGCTCCAGGTAGACCGTGTTCCACAGCACGATGGCCGCCGTCACCAGGTTGAGGCCGCTGGCCCGGTAGCGCTGCTGCTCGAAACTGCG
GTCACGGATTTCACCAAGGCGGTTGAAGAACACGGCACGGGCCAGCGCATTGCGCGCCTCGCCCTTGTTCAGCCCGGCATGCACGCGGCGGCGTAG

PTU-X1

>MOLJOM_00005 hypothetical protein
GGCAGAAATGCCGGTATTGGTGCATGGAAAGGTCTGCGTCGTCAGGAAGACGGGTTTGGTCAGTCTCCGGGAATAACGGGTAAGACCGCTAACCTGGCCGGACAGGGCGTTAATATTGGT
GCCAAAAAGCTTCGTCAGGCAGCTATTGAGAGAGCAAAGAAAATGTATGGTGGGTAA
>MOLJOM_00010 PilX7
ATGAAACTACTTATTGTGGCTTTCGTGACGCTTTGTCTCGCTGGTTGTCAGGCGTCACATAAACTACCGCCCGTTTCCGGGAAAAGCGAACCTGTTAATTCTGCTGAGGTAATGCAAAATGGA
ATTTAA
>MOLJOM_00015 VirB8 domain-containing protein
ATGGAATTTAAACTTCCCGGATTTAAAAATAAAAAAGACGTTACTGACTCATCAGTTTCATTTGAAGAAAAAAACATTGCACTACAGGAGAGAATGAATCGTATTTATAAATTCGGTGGTATCG
GAGGCATGTTAATTGGTGGGCTGTCTTTACTTGCATTAAATGCAGCATTACCACTGAAAACAACAGTTGTTGATGCCTACCTTATCGATAAGGTTACAGGTGTGGCTGAACGTCTGACTTCTG
TTAAAAAAGAAAATCTTTCTGAAAACGAAGCCATTGCCCGATATTTTATCACCCAGTATATAAAACATCGTGAAGGTTATAATTTTTTCAGTCTCCAGCATGATTATGATTATGTAATGGCTTACA
GCGCGGAGAATGTCGCGGCAGATTATAACGCATTATTTAACAGTGAACAGGCACCAAAACTTGTTTATAACAAAGCAGAAAAAACGGCAATGGTTCAGGATAATCCATCTGTCATAATTTCA
CCTTCGTCACGGGCAGATGATAAAGATATCGGTGCGTATATTCGTTTTCGTCTGACCATCAGGGATGTTGCTACCGGACAAACCCGCCAGGAGTTCTGGAATGTTCGCCTGACTTATCGTATC
GAACCGCAGGTTGAAATGGTGTCAGGGGAACGTAATAACAATCCTCTTAAATTCGTTGTAACAAGCTACGTTCGCGATAAAGAAGCCAGAGGTTAA

>MOLJOM_00020 Conjugal transfer protein
ATGAAAATGAATAAAGGAGCGTTAATTATGGCGCTTCTGATGGCGGCGCACGTCTGTCATGCAGCTGTTCTTCCTTCAGGCAGTCGCTTTGACCCACGCAATCAGATAGTCAGTTATAACCCC
AATAATACCACCATAATTAACAGTGCCGTTGGATACACCACCACACTGGTATTTGATGAAGATGAAACAGTTATCAGTGCCAGAACTGGTTTTCCGCAGGGATGGGCGGTTAATAAAGAAGAT
AACCTGGTATACCTGGAAGTTCGTCCTGTTAAACAGACTGTTCAGAAAAATAATATGGATGAAAACGGTAATACCTCTTCTGAATCCGTCAGTGTTGCTCTTGACCCGGAAAATGAGCTTGAA
CGCTGGCGAACGAATTTGTTTGTTCGCACCACGAAGCGTAATTACAGCATGGAGCTGAACGCCCGGACGTTCCGGCAGCCGGAGAAAATTGCGTTTGTGGTGAATTACCAGTATCCGCAG
GAACGCCGGAAGGAACAGGCCGAAATTGAGAAGAAACGCACAGAGGCTCTTGCCAGACGCCAGGAGGAGCAGGCAATCAACCGTTCCCTGGAAAATGCGAAATCGCCCCGTAACTGGC
AGTACTGGAAGCGGGTTGCTGAAGGCAGCCAGGATATCAGCCCTGATTATGCATATGACGATGGCCGTTATACCTGGTTCGGCTTCAGTCCGTTAAAGAAAATTCCCAGCGTCTTTGTGATG
AACGGTATGCAGGAGACTCTTACCAATCCTGTGATTAAACAGAGCGGGAGTTTTACGGCTGTTGGCGTACCGGTTGATAAGCGTTTTGTTTTACGTCTTGGTGAGCAGGTGGTGGGGATTG
AGAACCAGGGCTTCGGAAAAGTACGTTTACCAGCCGGAGATACGGTATCCCCGGATGTTAAGAAAGAGGTGATCCAGTGA
>MOLJOM_00025 TrbI/VirB10 family protein
GTGACTGAACAGGAAAATAAAATCCCGACTGCAACCGAAATTGAACAGCAGCTACGGGAACGCAGACAGAAAGAACTGGAACAGGCCGGGAAGACTCCGGAAGAAGAGCCTGGCAAG
CCAGCATTGCAGCTTGGTATTGAAAAACTTAAAAAGTCACGTAAAGGGATGATTATCCTCGTCGTGGGTTTTCTTCTGCTTGCTGCCGGTGTTTCTGTTTATTATATCCCGTCCATTATCCGTTC
TGTGTCGTCAGGGGATGAGAAACCCGCAAGTCAGCCGCTTGCAACCGGAACGGCTAAACGTCAGACCGGACTGAGCGAAGATATCGATCCTTTTAATACCGCACAGAAAAAAACAGAGAA
ACCAGAGGAAGAAAAAGTCATTTCTTCTGAAAAGACTGAACCGCCGGAAAATAAACAGCAGAGCTTCAGCCGTGCACTTGACGTTTCTCTTGATGGCAGCCAGACAGGAAACAGCAGCA
GTTCAGCGGGAACGTCAGTTTCACATACTGCGGCCAGTGAGCCAGAAAGCGATAAAAAGGATGAAGCAAAAGCAACCGCACAGACTACAGAATCTGCGCCACTGGCGAAAATAACGAAA
CTTCCATATGACCCAAATTTGTTTATCCCGGAAGGGACATCAATTCCCTGTTCACTGGACAGGCGTTTTGTTTCTGACCTGGCGGGGAAACTGGAATGTACGGTCAACAGCGATATATACAGC
GCCAGCGGTAATGTAAAACTTATCGAAAGAGGAACCGCCGCAAAACTGATGTATAAGGCCGGGTCTTTAAATCATGGACAAGGGCGTGTGTTTGTTATGGCTTACAAGCTACGTACCCGCA
GTAAGCCTTTTATTGATATTCCCCTGGTTGACTCACAGGCGGCTGGCGCGTTAGGCGAAGCCGGTGCTTCTGGGTGGATTGACACTCATTTCAGTGAACGTTTTCTTGGTGCAATGATGGTC
GGGATGATACCGGATTTAAGTCAGGCCGCCAGTGGTATTGCACAGAACAACAGGGACAGCCAGACCGACTATACGGCAAACAGTCGCCAGGCTTTTGCTGATATAGCACGCGAAGCATTTT
CTAATAGTGTGAATATTCCGCCAACGCTTTATAAAAATCAGGGCGAAATTATTACTCTGATTGTCGGTCAGGATCTGGATTTTTCAGGCATTTATAAACTGAAAATGAAAGGGGGTTAA
>MOLJOM_00030 Type IV secretion system protein
GTGAATAACGAAAACAGACATCTGATTTATGATGTGGTCAACGATTATTTTTATCACTGGCTGAATGAGATTGAGGGTGTCACGGAAATTGCTGTTAACCGACCAGGAGAAATATTTATAAAG
GTCAGGGGAAAGTGGCAATGGTATGAACAAAAGATGAGTTACAGTGATTGTCTTTCTTTTGCATCCACACTGGCCGATTTTCATGACGGCGGTTCTGTGACTCCTGAATATCCCCTGCGCTCT
GCCACGCTTCCGGGTGGAGAACGTGTTCAGGTTGTGATCCCACCGGCAACTGAAAAAGACACTGTTTCTATAACAATCCGTAAGCCGTCAGGTATTTTTATCAGTCATGACAAATTTATAAAA
CAGGGATTTTATTCACGCGTCAGTGGTTTAAGTGGTGACTCGGTTATGGAAGATAATATTTCTGCTTTAATCACTTCCGGATATTTTGATCGGGTTATACCTGAATCACTGCGTCAGGGGAAAA
CGATAGTTTTCTGTGGAGGGACGGGTTCAGGTAAAACTACCTTTGCAAATGCCTGTCTGGAATATATACCGCATCATCTGCGGTGTATTTCTATTGAAGATACTGATGAGGCAAAATTCAGATT
CCATAAAAACCATGTAAAACTTTACTATCCGGCAGAGGGTGAGAGTAAGGTTATTACCTCAGCGAGTCTTCTGCGTTCCTGTTTTCGTATGAATCCGGACAGGATTCTGATGACAGAAATCAG
GGGGGCTGAGGCATGGGATTTTCTGAAAGCATCGAGTTCAGGCCATGCAGGAAACATTACCACCGTTCACGAAAGTAGTCCTGAATATGCTGTGCTTGGGATTGTTCAGCGATGTTATATGA
ATCCTGAATGTCAGAATCTACCATTCAATGTCATTTTAAGACGTGTACTGAGTAATATTGATATTATCATGAGTATTAAATACCTTGATGATGAAGATTTTCGTTTCGCTTCCGGTATTTATTACAA
ACAACTTCATTTTGATGACTATTTCAGAAAACTGAAGGAGTGA
>MOLJOM_00035 Conjugal transfer protein
ATGTCTTTAAAACTCCCCGATAAAGGCCAGTGGGTTTTTATCGGTCTGGTTATGTGTCTCGTGACATATTATACTGGTTCTGTTGCTGTTTACTTCCTGAACGGAAAAACGCCGCTTTATATATG
GAAAAATTTTGATTCCATGCTCCTGTGGCGAATAATAACAGAGAGTAATATACGGACAGATATCAGGTTAACCGCTATCCCCTCTCTTTTATCAGGTATGGTTTCGTCTCTCATCGTGCCTGTTTT
TATTATCTGGCAACTGAATAAAACGGCTGTTGCTCTTTATGGTGACGCGAAGTTTGCCAGTGATAATGATTTAAGGAAATCGAAACTTCTGAAATGGGAGAAAGAAAACGATACTGATATTCT
CGTCGGAGCATATAAAGGTAAATACCTGTGGTATACCGCACCAGATTTTGTATCACTTGGCGCAGGAACCCGCGCAGGTAAAGGTGCCGCCATTGGTATCCCTAATCTTCTGGTCAGAAAACA
CTCTCTGATTGCGTTAGATCCAAAACAGGAATTGTGGAAAATCACCAGTAAGGTGCGTGAAATACTGCTGGGTAATAAAGTTTATCTGCTCGACCCTTTCAACAGTAAAACACACCAGTTTAA
TCCCCTTTTCTATATTGATTTAAAAGCGGAGAGTGGGGCTAAGGATCTGCTTAAACTGATTGAAATTCTGTTTCCGTCTTATGGCATGACAGGGGCAGAAGCGCACTTTAATAATCTTGCGGG
TCAATACTGGACAGGACTGGCTAAGTTGCTTCATTTCTTTATTAACTATGAGCCGTCCTGGCTTAATGAGTTCGGGCTTAAACCCGTTTTCTCAATCGGTTCTGTCGTCGACTTGTACAGCAAT
ATTGACCGGGAACTGATACTCAGTAAGCGGGAAGAACTGGAGGGAACAAACGGGCTTGATGAAAACGCGTTGTATCATTTGCGCGATGCCCTGACCAAAATCAGGGAATATCACGAAACG
GAAGATGAACAGCGTTCAAGCATTGATGGTTCTTTCCGTAAGAAAATGAGCCTGTTTTATCTCCCAACCGTTCGTAAATGTACTGATGGTAATGATTTCGATCTCCGTCAGTTGCGACGGGAA
GATATCACTGTTTATGTCGGTGTTAATGCGGAAGATATATCACTGGCTTACGATTTTCTGAACCTGTTTTTCAACTTCGTTGTTGAAGTGACATTGCGTGAAAATCCTGATTTTGATCCCACCCT
GAAACATGACTGCCTGATGTTTCTTGATGAGTTCCCTTCGATTGGTTATATGCCAATTATTAAAAAGGGATCAGGGTATATTGCAGGTTTCAAACTTAAACTGCTGACAATTTATCAGAATATCA
GTCAGCTAAATGAAATCTATGGTATTGAGGGAGCCAAAACGCTGATGAGTGCTCATCCCTGCCGTATTATCTATGCTGTCAGCGAAGAGGATGATGCCGCGAAGATATCAGAAAAACTTGGG
TATATTACCACTACATCAAAGAGCACAAGCAAGAACCGGGGACGATCAACTTCACAGGGCGAATCAGAAAGTGAAGCCCGAAGAGCACTGGTGCTTCCACAGGAACTGGGAACGCTGGA
CTTTAAAGAAGAGTTTATCATCCTGAAGGGGGAGAACCCTGTTAAAGCAGAAAAGGCACTTTATTACCTTGATCCGTATTTTATGGACAGGTTAATGAAGGTCAGTCCTAAACTGGCATCATT
GACGATGAAACTAAATAAGACGAAAAAAATATTTGGTGTGAAAGGGCTTAAATATCCGTCAAAAGAAAAAATGCTCTCCGTAGGAGAGCTGGAGTCTGAGGTTTTGCTATGA
>MOLJOM_00040 Conjugal transfer protein
ATGAAAAAAATACTTATGATCAGTATTCTTGTCCTCACAGCCTGTTCCTCTCCACCTGAACCGCCACAGGTTGAATGGGAAAAAAGGCCTGAAGTTATGAATACACAAATAATGAACTGGAAA
CCAACATCCGGTGTTATTAAATCAAATAATATAAATTCCTCATGGAGTAAGGTGTTGCCTGATTTTAAACCAGAAAACCATCTTTACGATGATTCTGTTTTTTATGCCGTTGCCCATTCTGAAAAA
ATAGTTGTAAGGACATCTTCTTTTGATAGTTACTGGTCAGCGAAAGACTGGCTAAGAAAAAATGGTGCAACAGGTGTTATTGAATATCAGCCACTAAAAAGATGGTTGAATAATGACTATGTT
GAAATTTATCTGTCAAGAATAAATATTCAGAGGTTACTATGA
>MOLJOM_00045 DNA topoisomerase III
ATGCAATATGCATTATTTGATGGGATGGAACGAAAGTTTTTGCTGGATGCTCTTGAATTTGGTGTTCTGAAGGACTGGAAAGAAAATCCGGTAAAAGAACTTCCTGATATTGATGAATCTGTT
CACCCCTTCCATGTCTGTTATGGTGGATATTTATTAAACCCTGATGTTTCAGATTTAGATATTAGCAGAAAAATAAAAGACCAGACAGGATTCTGGCTGGCAGCTATTGATGATACCCGTATGGA
TTGTCATTCAATAGCTTATTATGATATTCACACCCTCCCTTTAATTTCGTGTGGTCATCAGAAGATAGTTCCTTTTGCAGCGTTAATAAAAGCTGATGAATGCATCATTTCAAAAATTGCTTCGTAT
TCTGGTTTTGCCGTAACAGCCTTTTTGAGAATTAAAGACCAGAATATCGCAACGAATATACTTAACCGTGAGGGAATTTTTGCCTTTAATGGCTGTGAGCACAGATTCAGACAACCTGTAAGT
GAAGATAACTGGCAACAGGCAGTGTCAGAAGAACGCGCTATCCGTTGTGCCAAAAGATTAATTCAATGTAAAGGATAA
>MOLJOM_00050 DNA topoisomerase
ATGAGACTTTTTATCGCTGAAAAACCCGCAGTAGCAAATGATATTGTTAAGGCACTTGGTGGCAATTTTACCCGCCATGATGGCTGGTTTGAAAGTGATAACGCCATTGTGACTAACTGTTTT
GGTCATATTATCGAATCACAACCGCCGGAAAACTATAATCCTGAATACAAAGCCTGGAAGGTTGAAACGCTTCCTTTACGTCTTTATCCCGTGAAGTATCAGCCTGTTGAAAGTGCAGCAAAA
CAGGTTAAAACGATTCTCGAACTTATCAGACGTGGAGACGTGACTGAAATTGTTCACGCTGGCGATCCTGATGATGAGGGACAGCTACTTGTTGATGAAGTCCTGGAATATGCAGGAAACA
CAAAACCCGTAAAGCGCGTTCTGATAAACGACAACACGCTTCCGGCAGTGAAAAAGGCACTGGCAAATCTTAAAGATAATCGTGATTTCAAAGGGCTTTACCTTAAGGCGCTGGCGCGTTC
AGTTGCCGATGCCGTCTATGGATTCTCCATGACGCGTGCTTACACCATTCCTGCAAAAGCCAGAGGATATCAGGGCGTTCTGTCTGTCGGGCGCGTCCAGACACCCGTTCTTGGCCTGATTGT
GAATCGTACCCGTGCTAACCAGAACCATAAATCCAGTTTTTACTACACCATGACCGGAGTTTTTCAGCGTGGTGCTGATGTTCTCAGGGCGAACTGGAAACCAGGTGAATTTGCTCCGCTGA
CCGACCGTAAATTACTTGATAAGGCGTGGGCAGACGGAACGGCAGCATCCCTTGCAGGAAAACCGGCTACAGTTGAAGCAGCAGCAACTGATGATAAAAAAACGGCTGCGCCGTTGCCGT
TTAACCTGGTCAGACTCCAGCAATACATGAACAAGAAGTTTAAAATGACGGCACAAAAAACGCTGGATATTACGCAACAACTACGTGAAAAATATAAAGCAATTACTTATAACCGCTCAGATT
GCTCATATCTTTCTGATGAACAATTCAGTGAAGCGCCGCAGGTTATCGATGCCCTGAAATCAGTCTTTCCTCAGTCGCTGGATATTGATTCTTCACGTAAAAGCAAAGCGTTTAACAGTGCAA
AGGTGACTGCGCATACTGCGATAATCCCGACCTCCAGTGTGCCTGATGTTAACGCACTCAGCACCGACGAGCGCAATGTTTACCTGGCGATCGCACAACACTATCTTGTTCAGTTCATGCCTG
AAAAAGCATACCAGGAAGTATCGGTTGCCATTCAGTGTGGTGATGAGTCGTTCTATGCCCGTGCCAGAAAAACAACTGACAGCGGATTTGAGGCGTTTCTTGGCGCGGAAATCACAGACG
AAGGTGAATCAGAAGATAATGATGATTCCGCTTTTGAACTGCTCTGTAAAATTCGCACAGGAGAAACACTGACGACAAAAGAAGTTGTTGTTAATGAGAAGAAAACAACACCGCTGCCGTT

ATTCACCGAAGCCTCCTTGCTTGCTGCGCTTGTTCGTGTCGCGGATTTTGTCACTGATCCAACGATTAAAAAATTACTGAAAGATAAGGATAAAGACAAAAAAGATGAACATGGCGGTATTG
GTACGCCAGCTACCCGTGCAGCCATTCTGGAAACGCTGAAGAAGAGAAACTATATCACGCTGGAAAAAGGGAAACTTATTCCGACTGATACCGGATATGCGCTTATTGATGCCCTGCCAGGT
ATAGCGGTTAATCCTGATATGACAGCATTATGGTCTGAAAAGCAGACTGCCATTGAAAATGGCGACCTGACGGTTGAACAGTTTATTAATGAGCTGTACGGTGAATTGACAGGCATGATTTCT
GATGTTGACCTGGGCAAGATGAAGATTGAACCCGCTGCGCCAGCAGGGCAGTTTCAACGCCTGGACTCTCCCTGCCCTTCCTGTGGTAAACATATTGTTATCAGGCCGAAAGGTTATTTCTG
TACCGGATGTGAATTTAAAATCTGGAGTGAGTTTTCTGGTAAGAAAATCACCCAGGCACAGGCCGAAAAACTGGTTAAATCAGGGAAAACCGATTTGATTAAGGGATTTAAAAAGAAAAGT
GGTGGAACGTATGATACAGTTCTTGTCCTTGAGGATAAGAAAACAGGGAAGCTGGGTTTTCCGGCAAGGGCTAAGAAGTGA
>MOLJOM_00055 Gene expression modulator
GTGAAAACAAAGCAGGAATGGCTTTTTCAGTTAAGAAAATGTACATCAAGAGATACTCTTGAAAAAGTTATTGAGATTAACCGTTACAAGCTGCCTTTATCAGAATCAGAGGCATTTTATTCT
GCCGCAGATCACCGCCGTGCAGAACTGGTGATGAATAAACTTTATGATAAGGTTCCTTCCGGCGTATGGAAGTACGTCCATTAA
>MOLJOM_00060 DNA-binding protein
ATGAGCGAACTGACTAAAGAAGATGAATACGGCATTATCAGCCGGACTATGATGAATATTCGTTCATTGCGTGTGTTTGCCCGTGAGATTGATTTTGAGCAGTTGCTCGAAATGCAGGAAAA
GCTCAACGTTGTTATTGAAGAACGTCGTGAAGATGCTGAACGTGAAGCGGCTGAACGAGCAGAGCGTGAACGGAAACGTCAGGAACTGCTTCAGTTAATCGCCGGAGAGGGTTTCTCAC
CGGAAGAACTGCTTGGTCTGTCCGAAGAAGCACCAAAATCACGTAAAAAAACGTTACCAAAAGCGCCACCCAAGTATCAATTTGAAGAAAATGGTGAAACGAAATACTGGTCTGGTCGTG
GACGTGCGCCAAAACCGATTGATGAAGCGTTGAAAGCCGGGCGTTCTCTGGAAGATTTTCGTATCAATAAGAGTTTGAACGGAGTAACAGATGAGCAGTAA
>MOLJOM_00065 Methionyl-tRNA formyltransferase
ATGAGTAATACATCCTACAAACAAATTATCCCTGCGACAGACTGGTATTTCCGTCACGATAATGTCTCCGGTGTGGCAGGAAAGTCAACAGTATACCAACTGGCTGCATGGGCGCTTAAAGAA
AATGGTGAGGTAGTTGGTCTGGTGACGGTTCGTGATGATAATGGGCGTCCTAAACTGGTTACTCCTCCCCCTGTCCCTGGTGATTATTTGCATAAAGAACAACTCACCGATGATGAAAAAGA
GTGGGCGAAGAGACGCTAA
>MOLJOM_00070 hypothetical protein
ATGCTATTCAATGATTTTTATGCCCCCGACTCAAAGATAGAATATTGTGATTTATCCTTGCACGGATTGCAAGAATTTATCCACCCTCACGGTTTTTTCACTCAATTGAAACATTTAGAACATCCA
ATATTTATCGCAAAAGGAAACCATTGGACTCTTCAACTTGTCAACCATGTATCTTTTAGCGTAATTGGTGATGATCTATTAAACATAATAAATTGCCAAAACAAAGCCGCACTGGAGAACATAAT
TCATCAACTAAAAAAGACAAAAGAGTTATACCCTGATGCTTTTTTCTCCATTAGAAAAGAACTTGTTTTCTACTTTAGGATAAAATCATCTAATGATTTAGGAATAAAAGACCATATATCAAAGT
GCTGGGATATTTCCGGGTTATTTTCAATTCTTCTGAACAAACCGACTCTTCCTGAAGAAATAAACATAAAGTTCAAGGGAAATGATTCGAAGACACCATGTCTCTTAACAACGGGGTTTGAAC
AAAGAACTATTGACCTAGCGCTGAGGGAAATAAAACATCAACTCCTTCCAATAAACAGGAAGCATATTAATCTTGGAAAGATATTCTGCAAATGGTTCAAAATAGCAGAAAGATATATGCCAT
TGACAATAACATATCAGTATGAAACCGGTTTTCGAACACTGCATCAAGCTCATACTGATATTATATTGTTTGCGACTCAATTAGAAGCAATAAACAAAACAATAGGTGGCTCAAAAAATGAAAA
ATACATGAAACCCATTAATGAATATGCAAGTTTGTTTCTTATTCAAGAGATAGAGATGTTTTTTAAGAAATTCAACAATAAATCTATTGGTGAAAATATTGCCACACTCAGAAATGAATTAGCAC
ATGTTGACCGCAAAAAGGAGTTAATGAACATATTGACGATTGGTGATTATGTTAAAATTGGAAACTACCTAAAAACAATTGTAACATCTTATCTGCTATCAGATTTAGGAATCAATAACATTATT
ATTGAAAAAATATCAGGCTCAGACAATTCAAGAGTAA
>MOLJOM_00075 HEPN-RiboL-PSP domain-containing protein
GTGGGAGCTACAAAAGATTGGGTTATGCAAGTCGAGGAAAGTCGCCGTGAGGAATGGATACGAGAACGGCTTTCCAGCCCAGATCTTGAGGAGGATTCTGAAGAATGGCAGTTACTTGA
AAAGGACTATGACGAGTATCAGGACTTTCTTTCTGATATGGCTATGGAAGAATATGAAAACGAAAAATGGTTAAAACAGCATCCACATACAGAAATTTATAAAATAGCAATAAACCTTTTAGA
ACAAATAAAAGAAGAGGGAAAACAATCTACAAGTGAAGTTTTCATAAAAATGAAAATCGCATACATTGTGACAATAATGGAAAACTGTCTTAGCGAAATGATTAAAAGCGTTGTATTGTCAC
ATAACAGATATGTAGAAAATGCCATAAGAAACATAAATGAACTGAAAGCAAAGAATATCTCTCTGTCTGAGTTGATAAATAAAGAGTCTAATGCAAACAAATATGTACAGGAGTACCTTTCAGA
TATTCTTTATCATAGAATCCAACTGGTAGTTGAAATATATAAAGCAGTCCTGCAACCTAAGCAATATCCACGTTTTCCACTCAAAAATATCAATGAATTGATGAAACTGAGGCATGACATAGTTC
ATCGTAACGGGAAAACGAAAACAACGGATGAAAAGATACATACATTCAATACAGCTACGCTCAATGATGCGTTTAAAGTTGTAGAGGAGTTTCTTAATAACATGATGAATTTGATCAGTGATG
CTGTTGAGCATCATGAAAATGAGCAGATAGCCAGAGATCTTGAAGACGAGTTTTGA
>MOLJOM_00080 ArsR-gov region gene B
TTGGATGTAGAGTATCTTCCTCCAGTCAGAGCCACCAACTCAGGACTGGAAAACACGAAAAAGCCCGATTACCAGTCGGGTTTTTTTG
>MOLJOM_00085 hypothetical protein
ATGGCCATTTCTTTAAATGAATTTTATGCGTGCATCTGTATAATCTTTGTGTTGGCGCTGTACAGTGTGCCTCCCTTGCTTCTAACCCCTGCGCTATCTGGGGATGAACGAATTAAAGTCCTGGC
TGAACAAACAGGTGGTGCTGTGTTCAGTGCTCTGGCAGGGGGAAGGTACTCTACATCCAACAATGTGAGAGAGCCGTTGTGA
>MOLJOM_00090 ArsR-gov region gene B
TCAGTTGTAGAGAGTTTCTCTTCCGGGCGAGTTACCAGCTCGTACCGGAACTGTAAAAAAGCCGACCTCACCAGTCGGTTTTTTTAC
>MOLJOM_00095 Lipoprotein
ATGGATTTTATGAGCATTTTGGGGCGCTGTTTACTTCTTCCTTTCCCTGTTGCGATGTTTGTCGGTTGCCTGTTTCCAGGCATCGATGATCGTTTTACTGGAATGTTCATGAGTTTTATTGTCGG
TTGTCTATCGTGGTATATCACCAAGCCAAAACCGCAAAAGGCTGAGGTCGCGTAA
>MOLJOM_00100 Helix-turn-helix domain-containing protein
ATGACTTCAAATGCACCAACAAACTTTGTCCGCGCTTATCTGAGAGCTTCAACAGCAGAACAGGATGCTTCGCGTGCCCTGGAAACTATCGAGGCTTTTGCCAGGGAAAGAGGCCTGATCA
TTTGCAGCTATTATATTGAGAATGAATCCGGCTCCCGTCTTGACCGCCCGGAACTCTTCCGTCTTCTGAGAGATTGCCAGCAAAATGACATTTTGCTTATCGAGGATGTGGACAGACTTTCCC
GACTGTCAGGGGAAGACTGGAATACGCTGAAGAAAATGATCCGTCAGAAAGATATCCGGGTTATGGCCGTGAATGTGCCGACGACCTGGATTAATTCTGGTATGAGTGAGTTTGACAGCCG
GTTGTTTGCAGCCATTAACGATATGCTGCTGGATATGCTGGCCGCTGTTGCTAGAAGGGACTATGAACAACGGCGGGAACGCCAGAAACAGGGGATAGAGAAAGCAAGGAAAGACGGCA
AATATAAAGGGCGTAAACCTAATCAAGCCAGACATGATGCAATTATCAGGTTGATAGAAAGTGGCAGCTCCTGGACACAGGTACAGAAGGTACTAGGGTGCAGCCGCGGCACAATCAGTAG
CGCAATAAAACGTAAATCCCTGCAATCCTCCGGTGAGTAG
>MOLJOM_00105 Sporulation initiation inhibitor protein Soj
ATGAAAGTAATCTCATTTCTGAATCCGAAAGGGGGTTCAGGTAAAACAACTGCCGTAATCAACATAGCCACTGCGTTGAGCAGAAGCGGATACAACATTGCTGTGGTAGATACAGATCCACA
AATGAGCCTGACGAACTGGAGCAAAGCGGGCAAGGCAGCATTTGACGTATTTACAGCTGCATCTGAAAAAGACGTCTATGGAATCCGAAAAGATCTGGCGGACTATGACTTTGCTATTGTG
GACGGGGCAGGTTCGCTCTCAGTAATCACCTCCGCAGCCGTCATGGTAAGCGATCTGGTAATTATCCCTGTTACACCCAGCCCCCTGGATTTCTCCCGCAGCAGGAAGCGTCGTTACTGTTCTG
GAAGCACAGGCTTACAGCCGCAAAGTTGAAGCCCGCTTTCTGATCACCCGTAAGATAGAAATGGCAACCATGCTCAATGTGCTGAAAGAAAGTATCAAAGACACTGGTGTTAAATCTTTCC
GTACGGCCATTACACAACGTCAGGTTTACGTGAAATCAATTCTGGATGGTGACAGCGTGTTTGAATCCAGTGATGGCGCAGCAAAAGGTGAAATAGAAATCCTTACAAAAGAGATAGTTAG
CACATTTGAGTAA
>MOLJOM_00110 ParG
ATGTCACTTGAAAAAGCGCATACGGCAGTAAAAAAAATGACCTTTGGTGAAAACAGAGATCTGGAACGAGTAGTAACAGCACCAGTATCATCTGGAAAAATCAAACGTGTTAACGTCAATT
TTGACGAAGAAAAACACACCCGGTTTAAGGCTGCATGTGCCAAAAAAGGTACATCGATCACAGATGTGGTGAACCAGCTTGTAGATAAATGGCTCAAAGAGAACGAATAA
>MOLJOM_00115 Relaxosome protein TraY
ATGGATAAAAACGTCGTTGATGCGCTGAAAACGCTATTAGAAGCGTTACCGGAAGAGGTGGTAACAGAAGTCACATCAAAACTAAATCCTTCGGCAAGCCATATTCCTGAAGAAAACAGTA
AGCAATTGACAGCAAAAGCAAGACTACTGAATTTCCGGCTAACCGAAGCCTATGAAGAAATCCTGGAAGTCGAAGCTATCAGAACAGGCCAGAGCAAAACTACCGTTCTAAAGGCAGCAC
TGGCGATGTACAACAGCCAGGATGAAAACATAAAGAATCACTGGCTACTTGAGTCTGCAAAAATTAACTAA
>MOLJOM_00120 Lipocalin-like domain-containing protein
TTGAGTCTGCAAAAATTAACTAATTCAGGATATAGATTATTAGTAGGAGTTCGGGCAATAATGGATGATAACTCTACGTCAGTAACCAGCAAAGAAGATGTGCAGGATATTTCTTTAAAAAACT
GGCTAAAGAATAAAAAAACCATTGTATTACTTATATTTACTGCATTAACAAATGTTGGCAATGTCCTTTCGACGATTGATATTATCACAGATAAAACCAGTTCTTTTTACACATGGCTTGGGGAA

TCGAAAAAATTCGAAGGCCATTGGACCAACAATACAGAAGGATTCATAGACGGCACTCCTGATTTTTTACTCAAGAATGCAGGCGATGTCCTCATAAAATTTGACCTGAACATCAAAGGTGG
CGAAGTGAGGGGAGAGCTTCACACCGACGCACGACTAAAAATGTGTGAAACTATAGAAGAAAAGTTAAAAACTTTGTGTACATTAATAGCCTCACATCCACTAATGATTGAGGGGGAAAAA
TCACCATTTTCCAATGAATTCGATGCCTATATTACTGAGTATAGAAATGGAGATAAAAAAATAGTCGCAATATTAAATATGAAAATTACAGACGATGACAAAATGACAATAACAAATACAATGAG
AACACCAGAATCAGCATTATTTTCAACTAAAATATATGCTATAAAAATAATAACCGAAGAATAA
>MOLJOM_00125 HEPN-AbiU2 domain-containing protein
GTGAATACTGGAATAACAATTGATTTGACCAACCTTTCAGAAGATGAATTGCTTGATTTATATTCAATGTATAAAAGTGCAAACATAGCACATCAACTATGGTGCAGACGCCATGAAAATATACC
AGAGCATTTTTCGATAATATTCGTGACGCTTTTAGAACGAATAAAAAGGGTTACAGAAAAGAACTCAGAAGGGGTAAAAACACCAGATGTAGACCTGGATGCATTAATTGACACCATTTATAT
TGGTTGTCGTTCAATGTTCTGTGAAAACCCTGGCTTAAAAAATAACTACACTCTGCAAAACTGCCTGAGGAAAGCCAATTATCACAACGAAGCTAGAGTGATAGATAATATTTTACAGGAAAA
AAAATTCACAGATTCCATCATGAAAGATGAGTCATTTTTTAGTCTGGTGAAATTAGTTTCCAATAAGTCCATTGCACACCAGGAGAGCCTTTCAGGAAAAAAACGGGAAAAGATAGACTATC
GATATAAATTCTTAAATGACAATTCAAATATCTGTGAGTTTCAGTATTACATTTTTAGATGTCACCGTATTTATGAGAATATTGTGAAAGAATATGGGGATACATTACTGAATGATCTTAAAATAAA
AAACAATGATATATAA
>MOLJOM_00130 hypothetical protein
ATGAAATATTTGGTCAAAGAGTTTATTAACGAAAAATATACTAAGGCTGTTAATATTTTAAAGGATAACCTTAAAGAACACTATCATGTTTTTTATTGTGTGAGATTAAGTGAGATTCTTTTTCCT
GCCAGTGAATATGGAACTGAACAGTTTTTTAGTGATTTTGAAAAGATAAACTCCATTTCATTACCTGTCTTAGTGTTTGATCTCAAAGAGGGTGTTCCTGTGATTGTCATTAGTCTTGATGATGT
CAGCTACTGTGATGTTCTGGACGAAATGGATATTGATTTTATGAAATGTGATTCGCTGGCAGAATTGCTTACCAGCGATAAGCTGGAAAATTTATTAGTATGA
>MOLJOM_00135 hypothetical protein
ATGATGCCACTGCGATGCAGGATAGGGCAAACGCCGCAAAATGACGTCTCTGACGCCATTCCGCAACGTTTGCCCGATGACCTACAGTCGGTTCTTGTTGGAGAGTTTTATGAAATATTTGG
TCAAAGAGTTTATTAA
>MOLJOM_00140 Bis protein
ATGGCTAAAATCTATCAATTCCCTCAGGGGGAAGAACGTGCTAAATTCAGGAAAGAAATCGCCAGGGAGCGTAAGAAACGTTTTGCAGTGAAAACAGGGAGCACTTTTGTTAAGTGGCTT
GACTGGGGATGGTTTTATCTTCGTCTTCTTGCCGCGAGTGTTTTACATTTTATCTTTGTTTTTGTGTTAGCCATTCTTGGTGCGTTTAAATGGGCTGTGTTCTGGCTGGGTGGTATTTTGTGTGT
CATCACCTGGTATCATCTCGATCACCAGTTATGGACGCCACAAAATCTCACTATACCGGTGATTACGGCAACCTGGATTTTGGGACTGCAGGCTGAGCCTCTGATGGAGCTGCTCAACAAAAC
TATGCCCTGGCACAAGTTACTTGTTCCCGAACCTGCGGACGAAAGCACCGAGAACAGTAGCGAGATAAGCCAGAAATGA
>MOLJOM_00145 PI protein
ATGAACTTACTGAGCGTTACAAAGAAAACAAAAATTCGCCACAGAAATGAAATTAACAGCACATTCTCATCGTTGCCTTTGTCTGCCAGAAGAATTTTGTTTATGGCTATGGCGCAGATTGAT
TCACGTGAAGAGTTATCTGAGGGGGCAACCTTTCGTATTACGGCTCGGGAATATGCATTTATCGCTGATATTGATGTAACAGGTGCTTACCGGCAGTTGAAAGAGGGGGCAGAAGAACTTC
AGGCCAGTGTTATTCGCATTCCTCGAAATCAACTTCTCACAGCTCAGGGCAATCAAATCTCTGGACATACGAAGAAGAGAGGTAAATTACCTTCTGATGCAGTCAGGCTTATGAACCTGACTG
CGTTTTGTGATTATGTTGAAAGTGAAGGATATATTGATATTGCTTTTTCACATGTCATTGAGCCATATATCACGATGTTAAAGGATTCTTACACTACTCAGGTTTTGATATCGTCTGTAAGACTTG
CTGATCAAAATTCAAATATGCTTTATCAGTTTATCAGGAAACAATACAGCAGTGGTAAAAAGACATTTTTTGACATTGAAGTAGATGTATTAAAGGATGAACTTGAATTATTCAGAATAGATAAT
GGCGAAAAAGTTTACCTTTATCAGAATTTTAAGGACTTTAATAAAGTTTTTCTGCAAAAAAACATTGAAAAAATAAATCAATATACAGAGATTAATCATCTCGAAGTTAAAATCGTTGAGAGA
GTCGCAAGAAGAGCATCAAAGCTAAGGTTTTCTTATAAAATAGATAAAGAAAGTGAAGGCCTAGATATCAGAATACCATACGGTTTCAGAGGATAA
>MOLJOM_00150 Antitoxin
ATGAGCTATCAAATTCTGACAACCACAGCGGCCAGTATTACTGACCTGAAAAAAAATCCTATGGGAACCGTAGCTGAAGGTGAAGGGGACGCTGTTGCGATCCTGAACCGAAACGAACCG
GCGTTCTATTGCGTTCCACCAAAACTTTACGCCTACTATCGGGAACTCGCTGAAGATGCTGAGTTAAACGCTGTTGCTGATGAGCGCATGAAAAACCCGGAAATTGTGAAGGTTAACCTGGA
TGACCTATGA
>MOLJOM_00155 mRNA interferase RelE
ATGACCTATGAACTGGCTTTTGACCGTAGAGCACTGAAGGAATGGCAGAAACTCGGCCACACCATCCGTGAACAATTCAAAAAGAAACTGGCAGAACGGCTGGAAAATCCACGCGTACCC
GCAGCCCGGTTACATGGTCATGCTGATCGCTATAAAATCAAACTTCGTGCATCTGGCTACAGACTTGTATATCAAGTCATTGATGAGAAAGTCGTTTTACTTGTTATTTCCGTTGGAAGAAGGG
AAAGCAGCGAAGTCTATCAGATCGCAGATTTGCGCTAA
>MOLJOM_00160 hypothetical protein
ATGCAAAACTACGCTAAGTCTGTAGCCACAGAGATTTTACGTCAACTTGGTGGTAATCGTTTTATTGTTATGACTGGTGCTAAAAGTTTTTCTTACTTTGATGAAAACGGTGAGTGCGGGTTA
ACTTTCCGTTTGCCGTCCAATTTTGCAATGAAAGGCATCAACTTAGTAAAAATTAAACTGGATTTTACTGATACGTACCAGGTGAAATTTTCTCGTGTACGGGGTGATGAAGTTAAAGATATTT
CAAGATTCGATAATATCTATTGTGATCAGTTAGCGTGTTTATTTACACAAGAAACAGGGTTACATACCGTGTTATAG
>MOLJOM_00165 Transposase
ATGGATAAAGATAAGATAATTAAGAAAAACAGAGGTAATTACTCATATGTAATCAGAACGATGGATGAAGATGGGGATGCGGTTTTTCACGTCTTAAAATATGTTAAGACGATTGATAAAACT
AAAAGCAGGAAAACAGTAAGAAAATTGATAATGGACGAAAAACTCAACCTGGCATCATTGATGCTTCTGGATAATGGGATTTTGTGTGATTGTCTGACAAAAGGGGATGAAAATGCAGAAT
AA
>MOLJOM_00170 hypothetical protein
ATGCAGAATAAACCTACACCTGAAGAAGTAAAGAATGCACGGGTTGCGGCAGGTCTTACTCTTAAAGAAGCTGCTGATATTTTTGGTTATCAACTGAATTCCTGGCAGATGAAAGAAAGTGC
AGGTAAGGCCAGTCGTTCTTTATCTGTTGGTGAATATCAGTATTTATTGCTATTAGCAAATATGCATCCGTCTTACAGGCTGGTAAAAAAAATAA
>MOLJOM_00175 Transposase
ATGAAAGTATTAACTTTTAAAAATGATACTGTCTCTGTTGGTGATGTCTTTGTATCATCCTGGGGCTATGAGCAAACCAATGTAACCTTCTACCAGGTTCTTTCTGTTCACGGTAAAAAAAACCGT
CACCGTTCGCGAGATTCGCGCTAATTCAGAATATACCGATTCAATGGTCGGCTTTAAAACTCCCGTTTTAAATGATTTTACTGGTGAATGTTTTAAGCGCCAGATAAAAGATTTTGGTGATGAG
CTGGCAATCAAAATCGAAGATTTTGAAACTGCGTATAAAACTCTACCGGAAGAAAAACATCGATTTTCTTCTTACTACTGA
>MOLJOM_00180 hypothetical protein
ATGGAAAGAGAGTTTAGCGCAAAAGCATCATTAAACCGAAATATAAAATTTTGGTTTGAGCAATGTGGGTTATCTAAAGAAAGAGTTATTCGTTGTATTGATAACTGGTATGACCTTGCATACC
CACCATCAGAACAGGAGAAAGCAAAAAAAGAAGCAATTGAGAAGTTAATAAAGTAA
>MOLJOM_00185 hypothetical protein
GTGGGGCAACCTGATACCGTGACACCTGATTCTATTGCGAGGAATAAAATGTCGGAACGCATATTATCAGCAATAAATGACGTTGAAAAGGGTGGGCGTCCGGTTTTCCCTTTGATGCCATTC
CATGTCTTTCCTGAGTATATGGCATTACTCAGAAAAGCACTGGAAAAAAAGACACAAAAGAGAACAGATAAATAA
>MOLJOM_00190 hypothetical protein
GTGCTTGAGCATCAACAAAAATGCAAAAAGGCTGATGTTGTCATGGCTGAACATACGTACGTCTGCGACCGGAAACCCGATCAATGCGGTAAGGACAGTGCCGTCAGGTTCGCTCCGCGAA
CGGTCTGGCAGGAAGCGCAAGCGCAGTCCCGCGCAATATATCAGAAAGCACCTCCCGAAATACGGGAGGGCTTCGGCTATTCAGCTAAGGTTGCTGAGTGTAGCCTTCCATGCCCTGACG
GGCATCAAAAAGCCAGCGCCGGAAGTTACTCATTCTTTCAAGGTTAG
>MOLJOM_00195 DNA distortion polypeptide 1
ATGAAAAAAGTTCAATTCAGAATTGATGAAAATCAGCATAATGATTTGCTGGATTGTCTTAAAACTCTTTATCCAGATGAACCAGCTTTAACAGTAGCTAAAGGCATGAAACTTTTAGCAAATG
CTTTATTAAAAAGTAAAGCTGGCAGTAAGGACATAAATACGTTTTTTGATAATAATGATTTTATCAAAACAACGATGTACTTAACAGGTAAACAAAGGGCTGATATTGAAAGAGCTGCTAATCG
TCACGGATGGACGTTATCACGAGAATGTCGTTACCGCATACAGACGACACTTGAAAATGAACTGGATTTCTTTGACCAGGAACTGCTGATGATGAATCGTTGCCGTAATTCAATTGATAAGAT
CGGTCGTAATTTCCATTATATCATTGTTAATGATCAGACCAGGGTTCTTGATAAAGATGGTTTCTATCAGGATGCGGAGCGTCTTACAACAGAAATTTTTAATCTTAAGAATCAGTTTGAGAATT
ACATTATGTTATGTAAAGGGAGAACTGTTTCAAATAAAGTGGAGATGTAA

>MOLJOM_00200 Relaxase/mobilization nuclease domain-containing protein
ATGGGCGTTTACGTTGATAAAGAATATCGTGTTAAACGAAAGTCATCAGAAAATGGTCGTAAGTCAGCTTTCGCTCACAAAGTCAAAAATGGTGGAAAGAACTATAGCCGCAATGTTCAGGA
ACGTATCAACCGCAAGGGTGCCAGTAAGGAGGTTGTTGTCAAAATATCTGGAGGTGCTGTTACTCGTCAGGGGATTCGGAACAGTATTGATTATATGAGCCGTGAGTCAGAGCTACCAGTG
ATGAGTGAAAGCGGTCGGGTATGGATGGGTGCCGAAATTCTGGAGGCTAAAGAGCACATGATAGATCGTGCTAATGATCCTCAGCATGTGATGAATGATAAAGGTAAAGAAAATAAAAAAA
TCACACAGAATATTGTCTTCTCGCCTCCAGTTTTAGCGAAAGTAAAGCCTGAAGATTTGTTGGAGTCTGTCAGGAAAACGATGCAGAAAAAAATATCCTAATCACCGTTTTGTTCTTGGATACC
ACTGTGACAAGAAAGAACATCCTCACGTTCATGTTGTTTTTCGTATCCGAGATAATGACGGTAAACGCGCTGATATCAGGAAAAAAGATTTACGGGAAATTCGTACAGGTTTTTGTGAAGAG
TTGAAGTTAAAAGGTTATGACGTTAAAGCGACCCATAAGCAACAGCATGGACTTAATCAGTCTGTTAAAGATGCACATAATACAGCACCAAAAAGACAGAAAGGTGTTTATGAGGTTGTTGA
TATTGGCTATGACCATTATCAGAACGATAAAACAAAGTCTAAGCAACATTTTATAAAGCTAAAGACTCTTAACAAGGGGGTTGAGAAAACATACTGGGGGGCTGATTTTGGGGACTTATGTTC
GCGGGAAAGTGTTAAAGCAGGTGATCTTGTCAGGCTGAAGAAACTTGGTCAGAAAGAAGTAAAAATCCCGGCGCTCGATAAAAACGGTGTTCAGCATGGCTGGAAAACGGTTCACAGAA
ATGAGTGGCAGTTAGAAAATCTGGGGGTTAAGGGCGTAGACAGAACACCTTCAGCCAGCAAAGAGCTGGTACTTAACAGCCCTGATATGCTGCTGAAGCAACAACAGCGAATGGCGCAGT
TTACGCAGCAGAAAGCATCCACGTTACAGTCAGAACAGAAGCTGAAAACAGGGATTAAGTTTTTGGGCTTATAA
>MOLJOM_00205 RHH-3 domain-containing protein
ATGACAGCTATAAAAGGAAAGCGGAAACCGCAACGCAATGTACTCTACCTTCCTACAGAAGTTCGTGTTGAAGTTGAAAAAATTGCAATTGAAATAAGCTTCAAGAGGGGGCGACGTATCT
CTGATTCTGGTTTTGTTCAGTATCTTATTAAAAAATACAAATCTCAGGCGATGAAAGAACTTATTCATGGGGCTGATATTCCTGACGAGTAA
>MOLJOM_00210 IncX type IV secretion system transglycosylase VirB1
ATGTTATCTACTTCTACTTTTCTTGCGCTTGCCATGCAATGCGCTGCCAGCGTTCATCCCGACACAACGCACGAAGTCGCCAGGGTTGAATCAGGTTTTAACCCATATGCGATTGCGGAAATAA
TACCAAAGGTTAAACGTAAAACTGGTGATAAAGGCGTAGTATCTTACTTTCCTGAATCAAAGGAGGCAGCACTTAAGATCGTTAAAAACATTGAATTACGGAATCATCGTTACTCTGTAGGAC
TTATGCAAATAACGAGTACCAATTTTGCAAAGTTCGGTACAACAGCAGAGAAAATGTTTGATCCATGCGAAAATCTTAAGGTATCAGAAAAAATACTGGTTGACTGTTATAAACGAGGTGGC
GACTTAGTGCGTGGGCTGAGTTGCTATTATTCTGGCAATCAAGAAACAGGAGTAAAGCCAGAACCTGAATTTAATAATACAAGTTATGTACAACGTATAGGATTTAGCCCTCCTGATAATAAAA
AAAGTTTTATTGTTCCCTCTGTAAAGGAAATGATTAAAAAGGAGAATAAGACGACTATCACACCTGAAGAAATCATTATATATCCTCAATACGCCATGCGTGGCACTGTATCAAATGAAAAGGA
AACAAAAGATGTTGAAATTAAATCTGAATAA
>MOLJOM_00215 PilX2
ATGTTGAAATTAAATCTGAATAAACGTTATTTAACGCTTTCTCTATTTATGGCTGCGTTGATGCTTTGTGTTGCAGAACCTGCTTTTGCGGATGATGTGTCTACGAAGACAACTGGTTTTTTACA
GAAAATAATTGATTTTTTAACGGATATTCGTAAACCTGCAATTACAATTATTGCTCTTGTTATTGGGTATATTGCGATATTTTCTCGTCAACATACCTCATGGATAGTCCCACTTGTTATCGGAATT
ATCATCTTTATTGTTGCACCATATATTCCTGACTGGCTTGCGTAA
>MOLJOM_00220 Conjugal transfer ATPase protein Pilx3-4
ATGAGTACTCTTTTTAAAGGTCTTACGCGCCCTGCTTTAATAAGGGGGCTGGGCGTTCCGCTCTACCCCTTTCTTGGAATGTGCATTATTTGTGTTCTGCTTGGTGTCTGGATTCATGAGGCTA
TGTATGCCCTTATTCTTCCTGGCTGGTATGCCATCAGGCGTGTAACACAGTTTGATGAACGCTTTTTTGACCTTCTGTATCTGAGAACTCTTGTCAAAGGGCATCCTTTATCAAACAAGCGATTC
AGCGCAGTCCATTATGCGGGGAGCCAGTACAATGAAGTTGATATTTCAAAAGTGGATAACTTTATGAAGCTGAAAGACCAGTCTTCTGTTGAAGAGTTAATTCCGTACTCTTCACATATCACT
GATAATATTATCGTTACAAAAAACCGGGATTTGCTGGCAACCTGGCAGATAGACGGTGCTTACTTTGAGTGTGTTGATTCTGAAGATTTGTCAATTCTGACAGATCAGCTTAATACGCTTATAC
GTAGCTTTGAAGGGAAATTTGTTACGCTTTATCCTCATCGTATCAGGTGTAAAAAGGGCGTCAGACCAGTATTTAACAGTAAAATTCCTTTTGTGAACAGAGTAATGAATGATTATTACGAGTC
ATTCCCTCAGTCTGAATTTTTCGAGAATAAATTATTTCTGACGATTTGTTTTAAACCTTTTACTACGGAAGATAAAGTAACACATTTCTTTTCACGCAGTAAAAAACAAAAAGATATCTTTAAAG
AGCCTGTTAATGAAATGAATGAAATTTGCGACAGGTTGAATACCTATCTGTCCCGTTTTCATTCCCGACGTCTTGGGCTTTATGAAGATCATGGGGTTGTTTATTCAGATCAGTTATCTCTGTTC
CAGTATCTGCTTTCTGGTCGATGGCAAAAGGTCAGGGTTAGCAGTAGTCCGTTTTATACATATCTGGGAGGAAAAGACCTGTTCTTTGGTAATGATGCCGGACAAATTACCGCGTCAGACCAT
GCCCGGTATTTTCGTTGCATAGAGATTAAGGATTATTTTCAGGAGACGGATGCCGGTATTCTGGATGCTCTGATGTATCTCCCCGTTGAGTATGTCGTGACATCGTCCTTTACTGCGATGGATAA
GCAGTCAGCGATTAAGGCGCTGGATGATCAGATCGATAAGCTGGAAATGACAGATGATGCTGCCAAATCTTTGCTGGCAGATCTGAAAGTCGGACTGGATATGGTTTCCAGTGGATATATTT
CTTTCGGAAAATCGCATCAGACCCTGGTTGTCTTTGCGGATTCACCGGAGCGGCTGGTGAAAGACACCAATATCGTGACTTCCACTCTGGAAGATTTGGGGCTGATTGTCACTTATTCAACA
CTGAGTCTTGGCGCAGCTTATTTTGCTCAGCTACCAGGAAATTATACGCTTCGCCCTCGTCTGAGTACCCTCAGTAGTCTTAATTTTGCCGAAATGGAAAGTTTTCATAATTTCTTTTCAGGAA
AAGAAAAAGGAAATACCTGGGGGGAAAAACTGATTACTCTTCGGGGGTCAGGTAATGATATCTACCATCTGAATTACCATATGACGACTGAACATCAGAATTTCTTCGGTAAGAACCCGACG
CTGGGGCATACCGAAATTCTCGGTACGTCTAACGTGGGTAAAACCGTATTACTGATGACAAAAGCATTTGCCGCCCAGCAGTTCGGTACGCCGGAATCATTCCCTGCAAACAGAAAACTGAA
AAAACTGACCACGGTTTTTTTTGATAAAGACCGGGCAGGTGAAGTCGGTATACGGGCAATGGGGGGATCTTATTACCGGGTGAAGGAGGGAGAGCCGACAGGCTGGAATCCCGCCGCAC
TGCCGCCAACAAAGCGTAATATCGCTTTTATGAAGGACCTGGTGAGGCTGCTTTGTACTCTCAACAGTGAGCCGCTCGATGATTACCAGAACAGCCTGATTTCAGATGCGGTTGAACGTCTT
ATGCAACGGTCAGATCGCTCTTATCCTGTCAGTAAACTACGGCCTCTTATCCAGGAGCCGGATGATACTGAAACCAAACGTCATGGACTTAAAGCCCGTCTTAAGCCGTGGACGCAGGGGGA
AGAGTTTGGCTGGGTGTTCGACAATCGGGAAGACACGTTTGATGTCGATAACCTGGATGTTTTCGGTATTGATGGAACGGAGTTCCTGGATAATAAGGTGCTGGCCAGTGCTGCTTCATTCT
ATCTCATCTATCGGGTCACCATGCTGGCCGATGGTCGCAGGCTTCTTATCTACATGGATGAGTTCTGGCAATGGATCAATAACGAAGCGTTCAGGGACTTTGTTTACAACAAGCTGAAAACCG
GACGTAAACTCGATATGGTGCTTGTCGTAGCCACACAGTCACCGGATGAACTGATTAAATCACCCATTGCGGCAGCGGTTCGTGAGCAATGCGCCACTCATATCTATCTGGCAAACCCGAAA
GCCAAACGTAGTGAATATGTTGATGGTTTGCAGGTCAGGGAGCTTTATTTTGACAAAATTAAAGCTATCGATCCGCTGTCCCGCCAGTTCCTTGTTGTTAAGAACCCACAGAGGAAAGGTGA
AAGTGATGATTTTGCTGCTTTTGCCAGACTGGAGCTGGGAAAAGCAGCGTATTACTTACCGGTTCTCAGTGCATCAAAACCCCAGTTAGAACTGTTCGATGAAATCTGGAAAGAAGGAATG
AAGCCGGAAGAGTGGCTTGATACCTATCTGGAACAGGCGAACCTGATTTGA
>MOLJOM_00225 Pilus assembly protein
ATGAAAAAGCAAATTATGGCGGCATTCGTCGCTTCACTGATTGTTATTTCCGGCGCTCAGGCAGGGATACCTGTTGCCATCGACGCCAACCCTGAATGGGCGATTGAAGCCGGACGATGGA
CAGAACGCCTTAAGCAATGGGCGGAAACGGTAAAACATTACGAAAATCAGATAAATGCGTACAAACAGGAGCTGCTGTCAAAAACGGGTATCCGTGATGTGCAGGGACTGGTGCAGTCCG
CACAGTCAGTGAGTCAGGAACTGATGCAGATTTATGATCAGGGGAATGCTTTTATTGACGATTACATTAAAAATCCTGAAGGGGCGTTATCGGAACAGGCCAAATCGTTATTGTCAGATTACA
AAGTAACGGATACCTGCCAGAACCTGGGATATTCCGGCGACCTGGTACGGGGATGTGAAGCGACGTTCCTTTCTCAACTGGCAAGCGTGGAATACGGTAACAAGCTGGAGAGCAAGCTTC
GTCAGGACAACCAGACGATGAAAGACCTTATTGATCAAGTCAAAAATGCGAAGGATACGAAGGCCACGCAGGATGCAACAAACGCTGTTGCACTTGAACAACTGAAGTTCGAGAAGCTCA
AATTTCAGTATCAAATGTATCGCGATAAGCAGCGAGATCTTGCAGAATATAAAGAGAAGATGGCTCAGGCAGCTTTCAGAAAACAGCAACGTGAAGCCGTGCCACCTTCTTACAGAAAAGC
TTATATGGCAATGAAATCATATGAGGATGATTAA
>MOLJOM_00230 EexN family lipoprotein
ATGAAAAGTATCGCTACTGCTGGCCTTGTCATTATATCTGTTTTTAGTCTTTCTGGATGTTTTGAAGAAACAAAATCGGTTGATTGGTGGCTTGCACATCCGAAGGAGACATACAAAAAATTTG
AAGAGTGTCAGAAGTCTGGTAGCGATTCTGATAATTGTAAGAACGTTAAGCGAGCACATTTGTCTTTTGAACGGAGAAAGGCGGTTGGTTTACCAATAAATTGA
>MOLJOM_00235 TrbL/VirB6 plasmid conjugal transfer protein
ATGGGGATTGTCACTGGTATACCGGATGGTACTGATTTATCAGGTAATGTAACCTCTCCCGCAAATGCGGGAGGTGTTTCTGGATTTGATTCTGATTTTTTTCAGACAACTCATGAAGTTATATT
TAATATTCTCAACAAGAGTATATCTGGAAAATTAAGTGAATATTCAGATGTGGCTTATACTCTTGGTAAATATGGAGTTTCTCTGTATGTTTTATGGTATGCTTTTACTGTATTAGCAAGGAAACA
ACAGACACCTGTACCTGATTTTATCTGGAATATCTGTAGGTTTTACATAATATTGCTTTTTGTTAAGAATACAGGGGGAATACTTACATCAGCAACAGATGCTATTGATGGATTGAAAAATACAT
TGGCAGGGGGAGATCCGTGGGTATGGATGGATCAGTTATGGGTGAAGGTTATACAAGTTGCAACTCTTATTTTTGATAAAGATACATCTACTGTGCCTGTTGCCGGTGGGATTGGTGCTTTAT
TAACTTATGTCGGTGGTGTTTTGGCATTATTGCTTTGTTCTATAGTATTTGCATCTGCTGAATTAACATTACTATTACTTTCTGTCACTGCGCCAATATTTATCATGTGTCTGATGTTTGGTTTACTT
CGGCAAATGTTTAATAGCTGGCTACAGCTTAATTTTAGCTCGTTACTGGTTTTTCTATTTGCAGCATTAGCACTAAGAGCTGGGACATGGCAATTAAACATGGCATTAAGTACGTCTATTGCTAC
AGCATCAGAAAACAATCTTCTTCAAACGGGAGTAACTTCATTAGCTGCTGGCATTTTCATGGCCTGGATTATCTGGCAGGCGAAAAGTTATGCTTCACAGATTGCAGGTGTGGGTGTTGAAG
GTGCCATGCAGGGCGCAGCCGCTATGGGGATTGGTGCTGGCGTTTTCGGTGCATCCCGTATGGCGCGTGGCGCACTTGGT

PTU-NA 1 (IncI1, MOBP)

>PHINNC_00005 Replication initiation protein
GTGGCAGGTCTGAAGAATACTCCATATAACGCAGTACACTGGAGTCAGTTAGCACCCGAAGAGCAGATCCGTTTCTGGGAAGACTATGAAGCGGGAAGGGCGACCACTTTCCTGGTTGAA
CCGGAAAGGAAGCTCACAAATCGCCGTCGCGGTGAACACTCCACTAAACCCAAATGCGAAAATCCGTCCTGGTATCGTCCTGAGCGCTATAAAGCGCTGAAAGGGCAGCTCGGGCACGCC
TATAACCGTCTGGTGAAAAAGGACCCGGTGACCGGCGAGCAGAGCCTGCGCATGCGCATGTCGCGGCATCCTTTTTATGTACAGAAACGGACGTTCGTCGGTCGCAAATACGCTTTCCGGC
CGGAAAAACAACGTCTCCTCGATGCCATCTGGCCGGTTCTGGTCAGCTTCAGTGATGCCGGCACACATACCGTGGGGATGAGCGTCACTCGGCTGGCTGAAGAAATCAGCCCGAAAGACA
GCGAGGGACATGTTATTCCGGAACTGGAAGTGACGGTCTCCCGTCTTTCCCGTCTGCTGGCCGAACAGGTGCGCTTTGGTGTGCTGGGTGTGTCAGAGGAAACAATGTGGGACCGTGAAC
ATCGTCAGCGTCTGCCACGTTACGTCTGGATAACACCGGCAGGCTGGCAGATGCTGGGCGTCGACATGGTAAAACTTCACGAACAGCAGCAGAAACGACTGCGTGAAAGTGAAATCCGCC
AGCAGCTCATTCGGGAAGGTGTTCTGCGTGAGGATGAAGATATCTCCGTTCATGCGGCCAGAAAACGCTGGTATCTGCAGCGCAGCCAGGATGCCCTGAAGAAGCGCCGTGAAAAAGCCG
CAGCCAGCAAGCGTGCCAACCGCCTGAAGAAATTACCTGTTGACCAGCAGATTTATGAGATGGCTGAGTATCTCCGGAAGCGTCTGCCGCCGGATGAAGCCTATTTCTGTTCCGATGACCAT
CTGAAGCGACTGGCCATCAGGGAGTTGCGTCAGCTTGAACTGACGCTGGCTGCCCCGCCACCGCACTAG
>PHINNC_00010 Antitoxin YafN
ATGCCCAGTATTATTTTGTCTGACACCAGTGCAAGCGTCAGTGAACTGAAAAAAAATCCAATGGCGACAGTCAGTGCCGGAGAGGGTTATCCTGTTGCCATTCTTAACCGGAATCAGCCTGC
ATTCTACTGTGTTCCAGCTGAGCTCTATGAAAGGATGCTTGATGCCCTGGATGACCAGGAGCTGGTGAAACTGGTTACTGAGCGCAGCAATCAGCCACTACATGATGTGGACCTGGACAGTT
ATTTATGA
>PHINNC_00015 Toxin of the RelE-RelB toxin-antitoxin system Qin prophage
ATGACTTATACGGTTAAATTCAGGGATGATGCCCTCAGGGAATGGCTCAGACTGGATAAGGCCATTCAGCAGCAGTTCGCAAAAAAACTAAAAAAAATGCAGTGAAAATCCTCATATAGCCTC
TGCAAAACTACGTGGATTAAAAGATTGCTACAAAATAAAACTTCGCGCATCAGGTTTTCGTCTGGTCTATCAGGTGATTGATGACATGTTAATTATTGCGGTTGTCGCAGTAGGTAAACGTGA
ACGTAGTCATGTTTACAATCTCGCCAGCGAGAGAATGAGATAG
>PHINNC_00020 ABC transmembrane type-1 domain-containing protein
ATGCTTTTAAGTTTTAGTGAGTTAGATGTTCTTATTTATCAGGGGAAGGTTGGGGGGTTATTTCGTGAAAGAAAAAATGAAAGGGTTGCTACAGACATTGATTCCAGAAACAAGTCATCATC
AAGCAGTCATGAATATGATTCAGTTTTATCATTTCAATTTATTTGTGATAATGGAAAAGAAATAAATGTGAATTTAACAAACTGGAACATTAACATATTTCCAGGTCAAGAGCTTATTATTTATTT
TGTGGAAAACTCAAAGAAAAACAAAAAAAATACCATACATGATAAAAAACAAAAGCACATCGGAAGTGACTAATAATGTTCAAGAAATGTATAAAGAGTTTTTTTATACATCATACTTCAAACT
GATAATACCTTTCCTGTTAATCATTTCTTACTTTATGTCATCCATATCTGTGGCTATCATGTCATTCGTTTTATATGTCATAATCAGAAGAAGAATAGGAAAAGATTTCTTTTCAGAAATAAAAAGG
AAAAGGTTAAAAAAAGAAGATATCATGGGGTTAGAGCACGTAATATAG
>PHINNC_00025 Serine acetyltransferase
ATGCAGAGGCAGAGAGGTTTTCTTCTGTGGTGGCGCCTGGCTAATGAAATGTATATATCGGGGAATAAAAAACAACGAAAATGTGCGATCAAGATAAAAAATGCGCTTATGGCAAAGTATG
GATGTGATATTGGCCTGGGGGCGCAGATTGGTAAAGGCCTCGTGCTGCCCCACCATTCAGGTGTCGTCATTCACGGTAATGTAAAGATCGGTGAGAATGTGATTATACGGCAGAACACGACC
ATTGGCGAAAAGGAATCTGACAGCAGAGAGAATTATATCGTTATTGGTGATAATGTCGATGTAGGTGCCCATACGTGTATTATCGGGCTTAACGTAAAAATAGGCAGTAATGTAAAAATTGGA
GCCATGTCTTTTATTATGGAGGAGGTTCCGGATAACTGTACCTACGTTACCAGGAAAGAATCACGGGTAATTATGCATTAA
>PHINNC_00030 YafB
ATGGAAAAACATCAGGAAGAAAGGAAAACCCCCGTCATTGTGGTGAAAAAACGTCGAACTTTTTCGCCACCATCCTTATCCGAACAAACAGATATTATCGTGCCTGTATCTACTGAACAGGC
GACAGAATCAACACCCGCCGGAATAATTTCATCTGCCGTCGAAACACATATCCCCGAAGCGCCAGCCCGGAAGAAGAAAAAGAAAAGACACCGTTTCCCACGTCCCGCCCACTGGACCCG
AGAGTATACTCACGAGTGCGTGGAAAAAATAAAAACCCTGTTTCCTCATCTTCGTGCTGAAGGCGGTGGATTTATCCCCCTGAAAATTGGCATCAGCAATGATATTTCGGCATTTCTGGCAGA
AAACCCGGATACAGAACTGTCCATGGACGAATGGTTCTGTGCAGTGTCCTGCATCACCTCCCGCCGCGTATATCTGCAGCGGACCTCTGTCGCCGGCGTCCCTCGTTATGGGCTGGATGGTC
ATCCCGATGGGCAGGTCTCAGAAACTGAAGCACAGAGTGCTGGTCAAAGACTGGCGATAATAGAACAACGGTGGCTCAGGATGAAGGAACGGCAGACTGAGCAATAA
>PHINNC_00035 Small membrane protein
ATGATACCGGTACTGGAACCGTGTTTTCAGGGAGTACCGGATATGTGGTTTTTTTCGACAGTTCTGTTTCTGCTCATTGTTATCTGCAGTGTGGCTGGCGTTGTGAATGCACTGGCTGTGCGG
CGTGAATTACATGCACGCAGGGAACAGGAATATCATGCCCGGGGGCGGACTGCGACAGGCGAGCGTATCCTGCGGGAACGGTACGAAAAGTTCCGGCACCGCAGACAGCACTAA
>PHINNC_00040 HTH-type transcriptional regulator AppY
ATGGATGCTGTATGTTCCGTTGTTCTGGTTTGCCAGCCTTTCGACTTAATTATCTGTAAGAAACCTGTATCATTCAGGAAAAAATACGGTTTTACTTCTGGAGCCAGCAGCCAGAAGTAAATTAT
CTGACTGCCCCTCTCTCGTCAGAACGGTCGAACTTGACCATAAAACAGTATTGTCCTTTCTGAATGATGTGAATAACAGGCTGCCCGAAATGTTCTGTATTGACAGACAGGGATATGTCGTTG
AAGAAGATATTCCGCTGTCGCTGGTTTATTCTCTTTTTGAAGGGATAAGAATTGCAGATGCTTACACTACGTCACTAAGAGAAAAACTCTGTTTGTCCCTGCTTTCTGTATTTCAGGAGCGTAC
AAAAGTGATTTCTTTCTTACTGACGTATATGAACATATTCTCGTATAAGATCATGGGGATTATCGGGGGAGATTTAGAACGTGCCTGGCATCTTAAAGATATTGCTGAACGCCTCTATGCCAGTG
AAAGCCTGATCAAGAAAAGACTGAAGGAGGAGGGAACCTCATTTTCTGAAATATTAAGGGATATGCGTATGGAGTCAGCACGAAAGATGATACTTGAAAATACTTACTCTGTCTCCATGGTT
GCACAAAAGTGTGGCTACAACAGTACGTCATATTTCATCTCAGCGTTTAAGGATTATTATGGGATGACACCATTACACTATTATGATAATGCCGTAAGTGAAATGGCTGAAAATAAACAGCAGG
ACCCCTTACGGGGAACATTCTGA
>PHINNC_00045 Outer membrane porin C
ATGAAAAAGCTAACGGTGACAATTTCTGCAGTAGCAACAGCAGTCCTGATGGCTGTATCCGCACAGGCAGCTGAAATATACAATAAAGATGGTAATAAACTTGATCTGTACGGAAAAGTGGA
CGGGCTCCATTATTTTTCAAGCAATAAGGATGAAAATGGTGACAAATCCTACATGCGTATGGGGATCAAGGGGGAAACCCAGATCAACGATGATTTGACGGGATATGGTCACTGGGAGTACC
AGATCCAGGCAAACACGCCGGAAAATGAAAACCATGCCTGGACCCGTCTGGCTTTTGCCGGCCTGAAGCTCTCCGATTATGGGTCTCTGGACTACGGGCGTAATTATGGTGTTGTCTATGAC
GTCACCTCCTGGACGGATGTGCTGCCGGAGTTTGGTGGTGATACTTACAGCGCGGATAACTTCATGCAGGGACGCGGTAACGGTTTTGCCACCTACCGTAACACCGACTTTTTTGGCCTTGT
TGATGGTCTTAATTTTGCCGTGCAGTATCAGGGGAGAAAACGGCGGCGTGAGCGGTGAAAATACGACAAATAACGGGCGTGGTGCACTACGGCAGAACGGTAATGGTGTTGGTGGCTCAAT
TACCTACGAATATGACGGTTTCAGTATTGGTGGTGCGGTGTCCAGTTCCCAGCGTACATGGGATCAGAACCATACGGGACTGATGGGAACCGGCAACCGTGCGGAAACCTACACCGGTGGG
CTGAAGTATGATGCCAATAACGTCTATCTGGCAGCACAGTATACCCAGACATATAATGCCACACTTGCCGGCAACCTGGGCTGGGCAAACAAAGCCCAGAACCTTGAACTGGTTGCCCAGTA
TCAGTTTGATTTTGGTCTGCGTCCGTCCCTTGCCTTTCTGCAGTCCAAAGGGAAAAAACTGGGAAGCATCAATGGCCGCAATTATGACAACCAGGATATCCTGAAGTATGTTGATGTGGGCG
CGACCTACTACTTCAACAAAAATATGTCCACCTATGTGGATTATAAAATCAACCTGCTGAAAGACAACCAGTTTACACGTGATGCCGGCATCAATACCGATGATATTGTTGCGGTAGGTCTGGT
CTATCAGTTCTGA
>PHINNC_00050 hypothetical protein
ATGAATAGTCCGGACAAGAAATCACTCGTGACGATCAAAAATTCTGACGTTGACATCATTCACTCCCGCAGGACAAAAAGAAGCCAGCCATCCTGGCTGGCGAACATGTCATGTACCTCTGG
CAAATAA
>PHINNC_00055 YdeA protein
ATGACCCGGAGCCGCCGACCGTCGCCACTGCAGCGGAGAGTGCTGATTGTGCTGGCTGCGCTTGATGAAAAACGGCCGGGCCCCGTGCTGACGCGGGACCTGGAACGGGTGCTGGAGC
GAAGCGGGGAAGCCCCGGTCTACGGGCCAAACCTGCGCGCGTCCTGCCGCCGCCTGGAGGATGCCGGCTGGCTGCGCACCCTGCGCGCCACGAATCTGCAGCTGGCTGTCGAGCTGAC
GGACGCCGGCCGGGCCGTGGCACAGCCTCTGCTGCTGGCAGAGCAGGACCGCCTCCGGGCAGAACAGCGCGCGGCGGAAGTTGTCGTACTGCCGCTGGTCCCGGCATCAGCTCTTCCC
GCAGACGGCACTTCTTCCACTGATCTGCCGGTGGATCTGAACGGCATGACGTATCAGGCCTGCCGTGGGGATTTTGTGGTGCGTCTCGACGGCAGCACCTGTCTGCAGTTATGGAATAAAG
AAGGCAGAGTGGTACGCCTGGAGGGCGATCCGCTGGAAGTGGCGCAGTGGCTGCAGGCCTGTCACGATGCAGGAATAGAAGTTCGTGTACAGGTAAATGAAAGCGCCACCCCGTAA
>PHINNC_00060 YdfA

ATGGATGAAGAACGGGTGTTTTCACTCAGCTATGAACAACTGACCCGGTTTGCGGAACGACGCATCCGGGAATGCAATCTGGACAGCCAGGGCGCGATTTATCTCTGCGAGTCAGCCAAA
GCCGGAGCCGTGCTGATCTTCTGGCATGAACTGGCCATAAATGGCTATGCCTCAATGAATGCCATAAAGAGACAGGAGCTCATTGACGCCGACTTTCAGCGCCTCAGAAAGCTCATCTGGCC
GGAGGATGACAGGTAA
>PHINNC_00065 DUF3293 domain-containing protein
ATGTTTTTTATTGAGAATGAAGGTCAGGCTGTCGCCAGAACGGATTACTGGCAGTCTGTACAGGCGCAGGCCGGATATGTCTACCTCAGCTGGAATGCCGGCGCAGCCAGGCTGCTTGTCC
CGGATGCGGCAAAACATTTACTCAGGGAGATGCGGGGAGCTGAGTACGTCATCATCAGTAAGGGAACGCTGCATAGCCGCGATGCGCTGGAACTGGTATTTGAAGACGGCAGCGATGCGC
CGTTCGTGATCCACATGCTGAGTGAGCAGTGCGATCGCCTGCTCCCCGAAAACAACCAGGGAGGGGGTTTTGTTGTCACCGTCTGGACGCGTGGCGGTAACCAGCTCCGTTATCCGGGAA
AATACCGGGTTGTGGAAAACCTGCCCGACGTTTCCCCGTGGAGTGAACACTGA
>PHINNC_00070 Resolvase
ATGCAGCACCTGCCGGCACCGATCCACCATGCCCGGGATGCCGCTCAGCTTCCTGTTGCCATCGATTATCCGGCAGCGCTGGCACTCCGCCAGATGTCGATGGTTCATGATGAACTGCCCAA
ATACCTGCTGGCCCCTGAAGTGAGCGCCCTGCTCCATTACGTCCCGGATCTGCGCCGCAAGATGCTGCTGGCCACACTGTGGAACACCGGTGCGCGCATTAATGAAGCACTGGCGCTGACG
CGGGGGGATTTTTCGCTTGCGCCTCCGTATCCGTTTGTGCAGCTTGCGACCCTGAAACAACGGACCGAAAAAGCCGCCAGGACGGCAGGAAGAACGCCCGCCGGGCAGCAGACTCACCG
GCTGGTTCCGCTCTCCGACTCCTGGTACGTCAACCAGCTGCAGACGATGGTGGCCACACTGAAAATCCCCGTGGAACGGCGTAATAAACGAACAGGCAGGACAGAGAAAGCGCGGATCT
GGGAAGTGACGGACAGAACGGTCAGGACCTGGATTGGGGAGGCGGTTGCCGCCGCTGCCGCTGATGGTGTGACGTTCTCTGTCCCGGTCACGCCACATACGTTCCGCCATTCCTATGCGA
TGCATATGCTGTATGCCGGTATACCGCTGAAGGTCCTGCAGAGTCTGATGGGGCATAAGTCCATCAGCTCAACGGAGGTCTACACGAAGGTGTTTGCGCTGGATGTGGCTGCCCGGCACCG
GGTGCAGTTTTCGATGCCTGAGTCCGATGCGGTGTCTATGCTCAAAAGAATTCCTTAA
>PHINNC_00075 ParA family protein
ATGCCTGTTATTGCTAATACGCATCCCAAAGGTGGTGTGGGTAAGACAACTTCATCCGTAAACATTGTTGGTGAAATGAAGTCTGATACAGTTGATCTGGATACTCATACCGGGCTTTCTATAA
TTCTGGGGCTGAGACCTGAAGGAAAAGAAATTTCCGTGAAAGTACCCAAAACAGTGGATGAGTTAATCGAAATTATGACTCCCTACAAGAACAGCGATAAGACGTTACTTATTGACTGTGG
GGGATTTGACTCAGATCTTACCCGTACTGCGATTGCCTTTGCAGACTGCGTTATTGTCCCTTCAAAAGACTCCCTGACTGAGCGTATTGGTTTGATGCATTTTGATGGAGTACTGGACGAAAT
CAGCTCTATCATGGGGACCGATATAACTGCTCACCTGTATCTCTGTAAAGTTAATCCTAACAAGAAGAAATTCCCTAAGCTTGATGCGATCCTGCCATCGTTCAAACATCTGAAGCTGATGAAG
AGTCGCATTTCTGCCCGTGCTGAGTTTGAGGATGTCATTGAGACTGGACTGGGAATCACAGAGTCTGTTCATGGGCGTTATTCGGCCGGAGGTAAGGAAGTTATTGCCCTGATCGAAGAAA
TCAATCAGCTGATTGACAGTAATAAAAAGTAA
>PHINNC_00080 Partition protein ParA
ATGAAAATTTTTATTGATGATGGATCAACCAACATTAAACTTGCCTGGTTGGAGGATGGTGACGTGAAAACGTTAATCAGCCCTAACAGCTTTAAACCTGAATGGTCTTTTAGTTTACTGGAT
GACGCTGCTCCGGCTAATTACGAGATCGACGGCGAGAAGTTTTCATTTGATCCATTAAGTGCTGATGCTGTCGTTACGACTGAAACACGCTATCAGTACAGTGATGTGAATGTTGTGGCCATT
CAGCATGCGCTACAGCAGACCGGCCTGAAAGCGCAGCCTGTTGATGTCATCGTCACATTGCCGATCTCTGAATACCTTGACGCCAACAATCAGAAAAATAAGCAAAACATTGAAAGAAAGA
AAAAAAATGTGATGCGTGAAGTACGGGTGCAGGGGGGGGATGCATTTGTTATTCGCAGTGTTTCCGTTCTTCCTGAAAGTATTCCCGCTGGTTTCAGTGTTCTTGCGGGGCTTGAGGATGA
TGAATCCCTTTTGATCGTGGATCTCGGTGGTACAACGCTTGATGTGTCGCATGTTCGTAGCAAAATGACGGGGATTACAAAAACCTGGTGTGATCCGAATATCGGCGTGTCCCTGATTACATC
CGGGGTTAAGGAACAGATGGCTGTACATGCTAATACAAGAGTGAGCTCATTCCAGGCTGACAATATCATTGTGCACCGTAATGAACCTGACTACCTTTCCCGTCGCATTTATAATGCTGAACA
GCGTGAGTCTATCATTAATGTTATCAATGAACGGCAGAAGTTGCTTATTAAGCGAGTTAATGATGTCATCAGCCGTTTCACCGACTATACACATGTAATGTGTGTTGGTGGTGGGGCTGAAATC
GTGGCCGAGGCTGTGAAGAACCTGACTAAGGTGCCTGATGAACGCTTTTATCTGAGCTCATCCCCGCAGTTTGATTTAGTCATGGGAATGATAAAAATGAAAGGTGGTGTGACTAATGAGT
GA
>PHINNC_00085 hypothetical protein
ATGAGTGATGAAAATAAATCCAGACGCTGCTCATTTGAACTTTTCCCGGATGAACGGACAGGAGATAAGATAGCAGATGAGCTCATTGCAAATGAAAAACTGAAGGAGCGGGGACGTTTC
ATGCGCGCGATGTTAGTTACTGGCGCGGCGTTTGCAGCTATAGATAAACGTCTTCCTTTGCTGATATCGGAGTTACTGACAGAAAATACGACTCTGGATGATATTAATAAGGTGATTTCCAGCG
TAATACCAGGTGCTTTTTCAGTTGAAAAGAAATTGCTTGAATTACTGGAAAAACAATCTGGTCTCCATACGTCTGTGGATTGCAGTGCTCCTTTTACAGAGCAGAATCTGGCGAGAAATGAC
GGTGAAGACCAGACCAGAAGAAACGCTGAAAATATGTTCGGAGATGACTGA
>PHINNC_00090 Protein UmuC
ATGTTTGCACTGGCTGATATCAACAGTTTCTACGCCTCATGTGAAAAAGTTTTCCGCCCGGACCTTCGCAACGAACCGGTCATCGTACTCAGCAATAACGATGGCTGTGTGATCGCGCGCAGC
CCGGAGGCAAAAGCCCTTGGCATCAGAATGGGGCAGCCCTGGTTTCAGGTGAGACAGATGCGCCTGGAGAAGAAAATACATGTATTTTCCAGCAATTATGCGCTGTACCACAGCATGAGC
CAACGGGTTATGGCTGTTCTGGAGTCGCTTTCTCCCGCAGTTGAGCCCTACTCAATTGATGAAATGTTCATTGATTTGCGGGGGATAAATCATTGCATCTCTCCGGAGTTTTTTGGTCATCAGC
TCAGGGAACAGGTAAAAAGCTGGACAGGACTCACCATGGGGGTGGGCATTGCGCCTACAAAAACGCTGGCTAAAAGTGCACAGTGGGCAACAAAGCAATGGCCACAGTTTTCCGGTGTG
GTCGCGCTGACGGCAGAAAACCGTAATCGGATCTTGAAGCTACTGGGGCTGCAGCCAGTTGGTGAGGTCTGGGGAGTAGGACGCAGACTGACGGAAAAGCTGAATGCGCTGGGTATTAA
CACAGCACTGCAGCTGGCGCAGGCTAACACGGCATTCATCCGGAAAAACTTCAGCGTCATTCTTGAGCGTACGGTACGCGAACTCAACGGCGAGTCCTGCATATCCCTGGAAGAAGCACCA
CCGGCAAAACAGCAGATTGTCTGTAGCCGCAGTTTTGGTGAACGAATCACAGACAAAGATGCCATGCACCAGGCTGTTGTTCAGTATGCAGAGCGGGCCGCAGAGAAACTACGTGGGGA
GCGTCAGTATTGCCGGCAGGTGACAACATTTGTACGGACATCCCCCTTTGCAGTAAAAGAACCCTGTTACAGCAATGCCGCTGTGGAAAAGCTTCCATTGCCCACACAGGACAGCCGGGAC
ATTATTGCCGCCGCATGCAGAGCCTTGAACCATGTCTGGCGTGAAGGGTACCGCTATATGAAGGCAGGAGTCATGCTGGCTGATTTCACACCATCGGGTATAGCGCAGCCAGGATTATTTGA
TGAAATCCAGCCCCATAAAAACAGCGAGAAGTTAATGAAAACACTCGATGAACTGAACCAGTCGGGAAAAGGGAAAGTGTGGTTTGCGGGGCGAGGAACCGCCCCTGAATGGCAAATGA
AACGGGAAATGTTGAGTCAGTGTTATACAACTAAATGGCGAGATATTCCCCTGGCCAGGCTGGGTTAG
>PHINNC_00095 Translesion error-prone DNA polymerase V autoproteolytic subunit
ATGAGTACCGTTTATCACCGTCCGGCTGACCCTTCAGGCGATGACAGTTACGTGCGACCGTTGTTTGCCGATCGTTGCCAGGCCGGTTTTCCTTCGCCTGCCACTGATTATGCTGAGCAGGA
ACTGGATCTGAACAGCTATTGCATCAGCAGACCTGCAGCCACCTTCTTTCTGCGCGCCAGCGGTGAATCGATGAACCAGGCTGGCGTGCAGAATGGTGATCTGCTGGTAGTGGACAGGGCC
GAAAAACCACAACACGGGGACATCGTTATCGCTGAGATCGACGGTGAGTTCACCGTCAAACGACTGCTGTTGCGCCCACGCCCGGCACTGGAGCCGGTTTCAGACAGCCCGGAGTTCCGC
ACACTGTATCCGGAAAACATCTGTATTTTTGGTGTTGTCACTCACGTGATACACAGGACGCGGGAGTTACGCTGA
>PHINNC_00100 hypothetical protein
ATGATCCGCATTGAAATTCTTTTCGACCGCCAGAGCACAAAAAACCTCAAATCAGGCACACTTCAGGCGCTACAGAATGAAATCGAACAACGTCTGAAACCACACTACCCGGAAATCTGGCT
GCGTATAGACCAGGGCAGCGCCCCGTCTGTATCTGTCACAGGAGCCCGCAACGACAAGGATAAAGAACGTATCCTGTCTCTGCTGGAAGAAATCTGGCAGGACGACAGCTGGCTGCCTGC
AGCATGA
>PHINNC_00105 DUF1281 domain-containing protein
ATGCCAAACTGGTGCTCGAATCGTATGTATTTTTCTGGTGAACCGGCACAGATCGCTGAGATTAAACGACTGGCCAGCGGTGCAGTCACACCGCTTTATCGCCGCGCCACAGATGAAGGTAT
TCAGCTGTTTCTGGCCGGTAGTGCCGGACTTCTGCAGACCACTGAAGATGTGCGGTTTGAACCGTGCCCCGGACTGACGGCTGCCGGACGTGGTGTTGTATCGCCGGAGAATATCGCGTTC
ACCCGCTGGCTGACACACCTGCAGAACGGTGTGCTACTGGATGAACAAAACTGCCTGATGCTGCATGAACTCTGGCTGCAGAGTGGTACTGGCCAGCGTCGCTGGGAAGGATTACCGGAT
GAGGTCAGGGAAACCATCACCGTGCATTTCACCGCCAAAAGAGGTGACTGGTGTGGCTTCTGGAGTAACGAGGATGTATCGGTGTGGTGGAACCGTCTGTGTGACAATGTACTGCCGGAA
AAAACCATGCCGTTTGACCTGCTGACGGTTCTGCCCACCCGCCTGGATGTTGAAGTGAATGGCTTTAACGGTGGTGTTCTGAACGGTGTTCCTTCTGCGTATCACTGGTATACGGAACAGTAT
GGCGTGAAGTGGCCTGTGGGGTATGAGGTGAATATCAGCAGTCAGGGAGACAACTTCATTCAGGTTGATTTCGACACGCCGTGGTGTCAGCCGGAAAGCGACGTTATTGCAGAATTAAGC
CGCCGTTTCAGTTGCACGCTGGAGCACTGGTATGCCGAACAGGGCTGTGATTTCTGTGGCTGGCAGTTGTATGAGCGCGGAGAGCTCGTTGATGTGCTGTGGGGGGAACTGGAATGGTCT
TCCCCGACAGATGACGATGAGCTGCCGGAAGTCACCGGACCTGCGTGGATAGTCGACAATGTGGCGCATTATGGCGGATGA
>PHINNC_00110 Uncharacterized protein YubC

ATGAAGTATGACGGAGACGGGCGGGAAACCGCCCGTTTCTTTTCCGACAAAGGATGTCGCCGTGCTCCTCTTTTTACTGCGCCGGCTGACGCGGCGCGGCATAAACGCTGCCTGTGGTCA
GTGTCCCGCGTCCGGCGGGCACGGGACGGGCGCTTTTACCGCTCCCGGCTGGCACCGGTGACAGTGTACGCCAGCCCGTCGCCCTTTTCTGATGAACGCCCTTCAAGCCGCTTTCGCGGC
ATAACCTTGCCGTCAGAAAGACGACGGCTGCGGTATTCCACGGTCGGCCTGACCCGTTACCGGACGCGGTGA
>PHINNC_00115 Putative methylase YubD
ATGTCCCGTTTTATCCTGGGTGACTGCGTGCGAGTTATGGCCACGTTTCCGGACAATGCTGTCGATTTCATCCTCACCGACCCGCCGTATCTCGTCGGTTTCCGTGACCGTTCCGGGCGCACC
ATTGCTGGCGATAAAACCGACGAATGGCTGCAACCGGCCTGTAATGAGATGTACCGCGTGCTGAAAAAAGACGCGCTGATGGTGAGCTTCTATGGCTGGAACCGCGTCGATCGCTTTATGT
CTGCCTGGAAAAATGCGGGATTCAGCGTTGTTGGTCACCTGGTCTTCACCAAAAACTACACATCGAAGGCCGCATATGTGGGCTATCGCCACGAATGCGCCTACATCCTGGCAAAAGGCCGT
CCACGTCTGCCACAAAACCCGCTGCCGGACGTGCTGGGCTGGAAATATTCAGGCAATCGTCATCACCCGACGGAAAAGCCTGTTACCAGCCTGCAACCGCTGATTGAGAGCTTCACACACC
CGAACGCAATTGTGCTGGACCCGTTTGCAGGCAGCGGCTCAACCTGCGTTGCCGCCCTCCAGTCCGGACGCCGGTATATCGGTATCGAGCTGCTTGAGCAGTATCACCGTGCCGGACAGCA
ACGACTGGCTGCCGTACAACGGGCCATGCAGCAGGGGGCCGCGAATGATGACTGGTTTATGCCGGAGGCTGCGTAA
>PHINNC_00120 Cytoplasmic protein
ATGAACTATGCCGGACACGAAAAACTTCGCGCCGAAGTGGCGGAGGTGGCCAATGCCATGTGCGACCTGCGTACAACCATGAATGAGATGGAGCGGCGGTACAGCTTTAATGCTGACACC
CTGCCGGAACGTCTGGTGCGTCAGACGTTGTTTCGCGCAAACCGCCTCCTGATGGAGGCATATACCGAAATTCTTGAACTGGATGCGTGCTTCAAAGATTAA
>PHINNC_00125 DUF1380 domain-containing protein
ATGTACGGAACATGCGAAACGCTCTGCCGTGAGCTGGCAGCAAAGTATCCGGGAAACACGCCGCTGATGCTGCTTATCTGGTCCCCGGAAGAGATTCAGGCACTGGCCGACGGTATGGAA
ATTTCTCTGACCGATCATGAAATCAGGACTGTCCTGGCGCACCTGGAGGACATCCCGGAAGACCAGCGGATTGAGTCCGGTATTTCTTCCGCCGCTGCGATGGAGATTATCAGCAACGTGA
GCGAAAACCGCCTGGTGACCGTCCCTGCTGAACTGCTGGCGTCCCTGATTCAGACCGCTGAACAGGCACTGTGGAAACGTGAATGGGCCGCCCGTGATAACGGCCTTGCCGTCCCGGAAT
GTGTTACCCGCCGTCAGGCAGTGATTAATCAGGCCCGCACCCTGCTGAAAAACAACACACACGAAAACAACTGA
>PHINNC_00130 YchA
ATGTTATCGCCGCCTGCCCTGCGGGCGGCGATACAGGGAGAACGCTTAATCATGAACAAAACGCTGAATGCACTGGTTTGTCGTCACGCCCGTAACCTGCTGCTGGCGCAGGGCTGGCCG
GAAGAGACGGATGTTGACCAGCGAAATCCGAACTATCCGGGCTGGATCAGCATTTACGTGCGGCTGGATGCGCCCCGGCTGGCGACGTTACTTATCAACCGTCACGGCGGCGTACTGCCG
CCGCTCCTGGCCTCCGCCATTCAGAGACTGACCGGAACCGGGGCGGAACTGGTACTGTCCGGCAGTCAGTGGCAATCGCTGCCGGTACTTCCGGCAGACGGAACGCAGGTGTCTTTCCCG
TATGCCGGAGAATGGCTGACCGAAGACGAAATCAGGGCTGTTCTTGATGCGGTGCACGATGCGGTACGAAGCATCTGTTACCAGGTGGCAGACGATGCGCGGCGTATCCGTGCGGCGCTG
ACCACCACCGGTCAGACGTTGCTGACCCGCCAGACGCGCCGCTTTCGCCTGGTCGTGAAGGAAAGCGATCACCCCTGCTGGCTCGATGAAGATGACGAAAACCTGCCCGTGGTGCTCGAT
GCCATCGTGAACCGGGGAGCACGTTTTTCGTCGGTGGAAATGTACCTGGTCAGCGATTGTATTGAGCATATCCTGTCCAGTGGGCTGGCCTGCGATGTGCTGCGTATACCTGATGAATCGCC
CCGCCGCTGGTTTGACCGTGGTGTTCTGCGGGAGGTGGTCCGGGAAGCCCGGACCGAAATCCGCAGCATGGCGGATGCCCTGGCAAAAATCCGGAAATGA
>PHINNC_00135 Antirestriction protein
ATGCAATATGCGAAACCTGTCACTCTGAACGTTGAAGAGTGCGACCGTTTGTCTTTTCTGCCTTACCTGTTTGGCCAGGATTTTCTGTATGCCGAAGCGTCTGTATACGCGCTGGCGAAACAA
ATGATGCCGGAATATGAAGGCGGATTCTGGCACTTCATCCGCCTGCCGGACGGTGGCGGTTACATGATGCCGGACGGTGACCGTTTCCACATGGTGAACGGTGCAAACTGGTTTGACCGTA
CCGTGAGTGCTGATGCCGCAGGCATCATCCTTACCTCCCTTGTGATTAACCGCCAGTTGTGGCTGTACCACGACAGCGGGGATGCAGGACTGACCCAGCTTTACCGGATGCGCGATGCGCA
GTTGTGGCGTCACATCGAATTTCACCCTGAATGCAACGCGATTTACGCAGCACTGGACTGA
>PHINNC_00140 DUF1380 domain-containing protein
ATGTACTGTACTGTTAAAGAAATTATCCGTGATGTACTGGATACAGACGTGCCGGACAGTGAATGCGTTTTTGCCGTGGTGCTGACCCGTGGGGATGTGCGCCACATAGCCCAGGACTGGAG
TCTGACAGACGATGAGCTGGAAACCGTCATGCAGCGACTGGACGATGCCTTTGAGCATGGTGCGGATGTCAGCATTGTTCACGACGTTGTTCGTGAGCTGATGGAAGAAAAGCGCGCCAG
CCGTCAGGTGACAGTTCCGGCGGTGATGCTGGAAAAAGTGATGGCGCTGGCAGGCAGTGAAATGAAGCGCCTGTATGCCGTCGGGAGCGAGAACGGGGGCGACGGTGATGCGTTCGTC
AGGGAAGAACGCGAAGCAATGGACGTTGTGTTACAGGCGCTGGACGGGGAGCACATGTCATGA
>PHINNC_00145 hypothetical protein
ATGAATATCAGCACAGAAACCCGCGAAATTCTGCGCAATTACAAAGCCGTGATTAATGCGCGGCGTCGTGAAATGGGGCAGAAACCGCTCACCACTGCGCAGATTGTTGATGAAATCTGCG
ATTTTGTGGCGAATCAGCAGGCGGTTTTCCTCGGTGGTCACTATATCCTTCAGGGCAGCAGAAACAGGTGA
>PHINNC_00150 Single-stranded DNA-binding protein
ATGAGTGCACGTGGTATCAACAAGGTCATCCTCGTCGGGCGTCTGGGCAATGATCCGGAAGTCCGTTACATCCCCAACGGGGGCGCAGTGGCAAACCTGCAGGTGGCCACATCAGAAAGC
TGGCGTGACAAACAGACGGGGGAGATGCGGGAGCAGACGGAATGGCACCGCGTGGTGCTGTTCGGCAAGCTCGCGGAAGTGGCAGGTGAATATCTGCGCAAGGGTGCGCAGGTCTAC
ATCGAAGGTCAGCTTCGCACCCGTAGCTGGGAAGATAACGGTATCACCCGTTACGTCACTGAAATTCTTGTTAAGACCACGGGCACCGTGCAGATGCTGGGACGTGCACCACAGCAGAACG
CTCAGGCGCAACCGAAGCCTCAGCAGAATGGGCAGCCACAGAGTGCTGACGCGACGAAAAAAGGTGGCGCGAAAACGAAAGGCCGTGGACGTAAGGCCGCGCAGCCAGAGCCTCAGC
CTCAACAACAGCCAGAACCTCCTTATGGCTTCGACGATGATGCGCCATTCTGA
>PHINNC_00155 UPF0401 protein YubL
ATGAACAAATCCGATGAAATGATTCAGGAACTACCCGATGGGGCATTCACCCGCGAGTGTGCTGAAACCGTTGTTACACAGTACCGGAATGTCTTTATCGAGGATGATCAGGGAACGCATTT
TCGCCTGGTTGTCCGTCAGGATGGCACGTTGATCTGGCGCTCCTGGAATTTTGAGGACTGTGCCGGGTACTGGATGAACCGGTACATCAGGGATTTCGGCATTCGTAAGTAA
>PHINNC_00160 ParB domain-containing protein
ATGTCAGTAGTAAAGTCTGAACCAGACACCACCCGCAAAGCAAGCCGTAAATCTGCAAAAACGCAGGAAACCGTCCTGTCTGCCCTGCTGGCGCAGACGGAGGAAGTGAGCGTGCCGCT
GGCCTCGCTGATTAAGTCACCACTGAATGTGCGCACGGTGCCGTATTCTGCGGAGTCCGTCAGCGAACTGGCTGAGTCCATTAAGGGCGTTGGCCTGCTGCAGAATCTGGTCGTGCATGCC
CTGCCTGGTGACCGCCACGGTGTCGCCGCAGGTGGTCGCAGACTGGCAGCACTCAATATGCTGGCAGAGCGTGGCATCATTCCGGCTGACTGGCCTGTCCGCGTGAAAATTATTCCGCAG
GAACTGGCGACGGCTGCATCGATGACCGAGAACGGTCATCGTCGGGATATGCACCCAGCCGAACAGATTGCCGGATTCCGCGCAATGGCGCAGGAAGGCAAAACACCTGCGCAGACTGG
CGACCTGCTGGGCTATTCACCCCGCCACGTCCAGCGAATGCTGAAACTGGCAGACCTTGCACCCGTCATCCTTGATGCGCTGGCAGAAGACCGCATCACCACCGAACACTGTCAGGCGCTG
GCGCTGGAGAACGACACCGCGCGTCAGGTGCAGGTGTTTGAAGCCGCCTGCCAGTCAGGATGGGGCGGTAAACCGGATGTGCGGGTTATCCGCAACCTGATTACCGAAAGTGAAGTGGC
GGTGGCGGGGAACAGTAAATTCCGCTTCGTGGGGGCTGATGCCTTCTCGCCAGACGAACTGCGCACCGATTTGTTCAGCGATGACGGGGACGGTTATGTCGACCGCGTGGCGCTCGATGC
CGCCCTGCTGGAAAAACTCCAGGCTGTCGCTGAACACCTTCGGGAAGCCGAAGGCTGGGAATGGTGCGCCGGGCGCATGGAGCCTGTCGGTGAGTGCCGTGAGGATGCCGGAACATAC
CGCTGTCTGCCGGAGCCGGAAGCGGTGCTGACGGAGGCGGAAGACGAACGCCTGAACGAACTGATGACGCGTTACGACGCGCTGGAAAACCAGTGTGAGGAATCCGACCTGCTGGAAG
CAGAAATGAAGCTGATGCGCTGCATGGCGAAGGTCAGAGCGTGGACGCCGGAGATACGTGCCGGAAGCGGTGTGGTGGTGTCCTGGCGTTATGGCAACGTATGTGTCCAGCGTGGTGTG
CAGTTGCGCAGTGAAGATGACGCGACTGACGACGCTGACCGCACGGAACAGGTGCAGGAGAAAGCGTCAGTGGAGGAAATCAGTCTGCCGTTGCTGACGAAAATGTCCTCAGAGCGCA
CGCTGGCAGTCCAGGCTGCACTGATGCAGCAGCCGGACAAATCCCTGACACTGCTGGCATGGACGCTCTGCCTGAATGTGTTTGGCAGCGGAGCGTACAGTAAACCAGCACAAATCAGCC
TGGAATGTAAACATTATTCGCTGACCAGCGATGCGCCATCGGGGAAGGAAGGTGCCGCATTCATGGCGATGATGGCAGAAAAATCCCGTCTTGCAGCCCTGCTGCCGGAGGGATGGTCAC
GGGACATGACGACGTTCCTGTCACTCAGCCAGGAGGTGCTGTTGTCCCTGCTCAGTTTCTGCACTGCATGCAGCCTTAACGGTGTCCAGACCCGTGAGTGTGGTCACACGTCACGCAGTAC
GCTTGACTCGCTGGAGAGCGCCATCGGCTTTCACATGCGCGACTGGTGGCAGCCGACAAAAGCAAACTTCTTCGGACACCTGAAAAAGCCGCAGATTATCGCAGCCCTGAATGAGGCAGG
ACTGTCCGGTGCCGCACGGGACGCGGAGAAGATGAAGAAAGGCGATGCGGCTGAACATGCAGAGTTCCATATGAAAGACAACCGCTGGGTGCCTGGCTGGATGTGTGCACCACGTCCAC
AGACGGATACCACTGAACGCACTGATAACCTGGCTGATGCCGCCTGA
>PHINNC_00165 Protein PsiB
ATGAAAACTGAACTGACCCTGAATGCCCTGCAGTCCATGAACGCACAGGAATATGAAGATATCCGTGCTGCGGGAAGCGATATGCGCCGTAATCTCACTCACGAGGTGATGCGTGAAGTGG
ACGCACCGGCTAACTGGATGATGAATGGCGAGTATGGCAGTGAGTTCGGGGGCTTTTTCCCCGTCCAGGTTCGTTTCACGCCAGCCCACGAACGTTTCCACCTGGCATTATGTTCGCCGGG

AGACGTCTCTCAGCTCTGGATGCTGGTTCTGGTGAATTGTGGTGGACAGCCTTTCGCCGTCGTTCAGGTGCAACATATCTTCACGCCTGTCGCTATCAGTCACACGCTGGCGCTTGCCGCGA
CACTGGATGCGCAGGGATACAGTGTTAACGACATCATTCATATCCTGATGGCAGAAGGAGGTCAGGCATGA
>PHINNC_00170 Plasmid SOS inhibition protein A
ATGAGCGCACGTTCACGGGCACTGATCCCCCTCAGCGCAGAACAACAGGCCGCCATGCAGGCGGTGGCTGTCACAGAACAACGTCGTCGTCAGGGACGCACACTTTCAGCATGGCCTTAT
GCCACCGCTTTCTTTCGCTGCCTGAATGGCAGTCGCCGGATTTCGCTGACCGATCTCCGCTTTTTTGCCCCTGCGCTGACGAAGGAGGAATTTCATGGCAACCGCCTCCTGTGGCTGGCTGC
CGTGGATAAACTGATTGAAAGTTTTGGGGAAGTCTGTGTTCTTCCCCTGCCATCCGATGCGGGGCACCGTCTGTTCCCGTCCGTGCCTTTTCGTGAAGGTGAGCGTCGTCGTCAGAAAACC
ACGCTGACAGAGCAGAAATACAGCCGCCAGCGGGAACGTGAGGCAGAACGACGGGAACTGGAATACCAGACATGTTTTGCTCAGGCGCAGATTGACCTTGCGTTTCATACTCCCGCCACG
GTCGGAAGCTGGTTGTCCCGCTGGTCTGGTGTTGTTGAGGAGCATGATCTGGAAACGATTTTCTGGGGGTGGTGCGGGCGTTTTCCATCACTGTCATCATTTGACCGGTTTTTCTGGCAGG
AGGAACCACTCTGGCGGCTGATTTTTGAAGCCGGTGAGGCCGGTCGTGGTGCACCGGTACAGGTACGTGCACTTGAGCAGTGGATGATCCCGAACAAGCTGGAGAACGTAATATGA
>PHINNC_00175 hypothetical protein
ATGATGAAATCAGACGAAAAATACCAGGTTCCCGCGTGGATGCGACCTCTGTTGCCGTTGCTCTGCAACACCGGGGGGAACGATCCGGAAGAACTGCTGAATGATACAGAAACCACTGCC
AGTGCGAATGTTGTCCGTTATGTACTGATAGTTGCTGTGCGGTCGCAGGTTGATCTGCTGCAGCTTCTGTACAGGAAAGGGCTGTTGCGCACAGAGATACCAGGTGGCTTTTCACCGGAAG
AAGCGCAGGCACTCCTGGATAATCTGGTGCGCAGCCATATCAGCAAGGCGCTGTCGGGCGAGCGAATGGCAGCCCGTGACAGAAATGCCGATCTGGCCTGGATTCGACAGCAACTGGTCG
ATGCCGCCTGGTTTGTCCGTGCCACACTGGAAGCGCATGGGATGAGCGTCGGAAATGAGAGTCCCCCTGCTCCGCCGGAGACAATGCCGGACATACAGACACGGGAACTGGTGATGTTGA
TCAAGCGACTGGCATCATCGCTGAAAGCGGTGAAACCGGACAGTTGTGTGGTGCGTGAAGCGCAGGACTGGCTGCGCGACAGAAAACTTGTGGATATCACAGATATTCTCCGGTGA
>PHINNC_00180 DUF1472 domain-containing protein
GTGTCCTCTGCACTCGCCGGAATAAGGAAGTCGCCGGCGGCTCCGCTTTTACCCGGCCATGCGGGGCATGGCCTTGTGGGTTTTCAGCTCTGTGGCCTCAGCGTCGTGTGCGGGCTGTGCC
GTGCCTCCATCTTAGCCGGGCTGGCAGGGATGCAAGGGTACGCTTCGCCGCTGCGGTCACCCGGTCCCTCCTTCCCGTCTGCCGTGATTTTCCGGTCGTTCACCCGCTCAGATTTCGCGGTC
TCACCCTCCAACTCCCGCCAGCCGTGTGCGTCAGGCTGCGGCTTCCCTTGCGCCCTGTGCATCCCCGCCTTATCGCCCGGCTTTTATGGAGGCACGGCACCGCCCGGTGCCGCAGAACATGT
GACTATGGAGGATTCGGGAATGTCTGTTGTTGCACCTGCTGTATACGTTGGAACCTGGCACAAATACAACTGTGGAAGCATCGCCGGACGCTGGTTTGACCTGACCACGTTTGATGATGAGC
GCGACTTTTTCGCCGCCTGCCGTGCTCTTCACCAGGATGAAGCCGATCCTGAACTGATGTTTCAGGATTATGAGGGATTCCCGGGGAATATGGCCTCTGAATGCCATATCAACTGGGCCTGG
GTTGAAGGCTTCCGCCGGGCACGGGATGAAGGCTGCGAAGAGGCTTATCGTCTCTGGGTGGATGATACCGGTGAGACGGATTTTGACACCTTCCGCGATGCCTGGTGGGGCGAGGCTGA
CAGTGAGGAGGCTTTTGCGGTTGAGTTCGTCAGTGATACCGGCCTGCTGGCTGACGTGCCGGAGACGGTGGCGCTCTATTTTGACTATGAGGCGTATGCGCGGGATTTATTCCTGGACTCC
TTCACCTTTATTGACGGTCATGTGTTCCGTCGGTGA
>PHINNC_00185 Post-segregation killing protein PndC
ATGACAGTCAGCAGCACGATTTCCGTTTTTTGCCGCGACGGGGTGTTCCGTACCGTTTACTGCCACCTGCACGGGGAGCCGACCTGGAACGGTCGCATCCTGCATACCCACTATGCCACCG
GTCAGCAGGCCGAAGCCCTGGTTGAACACGGTGATATCCGTTGCCTCGGTCCCCGTTGCGACAAACCCGCCGGGCATACGCTTCAGAACCCGGTGGACGGTGTGACGGCTTATTACGGAC
GTGACAGTGGTTTCCGGATGGACAGTGAGGCGCGTGAGTACCGTTCTTTCAGGGAGGCTATTGCCACTGAAAGCACTGAAGAGGTGCGCTTCCATTATGTGTTCATCGACGGCTACTGGAA
GGTGATGTACCGCACGCCGGAAGGCTGGAAGATGAAAGCGCTCGCGCTGGCACTGCGTCGCTGTCCGAAATGA
>PHINNC_00190 YDFB protein
ATGAAAAAAAGGCAGGGCGATAAACCCTGCCTCTCTCCGCCGGCGCTTCCCCGCCAGGAAGTCCGGCATCTCATCACTATCTATGGAGATTATGCCATGAGACCATCAATTATCTTCGCAACC
GCCGAGTATGTAAAGCGTCTGCGTGAAGAGTGCCTGCGGGAGAATAAACCCCTGCACCGCCATACCCGCTTCAGACGTCAGGAGCTGGCACAGGATGAGATTAACCCGGACGTCCTGGCG
ATGAGCGGCCATATCGCCAGACGCTGCAGTGAGCAGAAGCGGGTGCGTATCCCCGCTATGAAAGTCAGCGAATGGGGCCACCTGCTCCGCGCGCTTGAAATTGAGCGGGTCTGCCACTGA
>PHINNC_00195 Transposase-31 domain-containing protein
GTGAGTAAGAAGAAAACCACCACGACGCCCACGCCGCATGATGCCGCGTTCCGGTCGTTCCTGGCGAATCCCGACGTCGCCAGAGATTTTCTGGAACTGCATCTTCCGGCGGAGTACCGG
CAGTTGTGCGACCTGTCCACGCTGAAGCTGGAACCCGCCACCTTTGTTGAGCCGGACCTGCATCAGTACGCCAGCGATATCCTCTGGAGTGTGAAAACCACCGGGGGTGAAGATGGTTATG
TTTATACGCTCATCGAGCACCAGAGCACCGAAAATCTGTACATGCCTTTTCGCATGTTACGCTACAGTGTGGCGGCGATGCAGAGACATCTGGAGCAGCACAAAACGTTGCCACTGGTGATT
CCGGTGCTGTTCTATCATGGTGAGCGCAGCCCGTATCCGTACAGCATGAACTGGCTGGACTGTTTTGAGAATCCGGCGCTTGCGGCTAAAATATACACAAAGCCGTTTCCGCTGGTTGATATC
ACTGTCGTTGATGACAATGAAATCATGAATCATCGCCGGATGGCCGCACTGACGCTGCTGATGAAGCATATCCGCCATCGTGACATGATGGAGCTGCTGGACAAACTCCCGCAGGTCATGGT
GGAAATTTCAGATGAGCAGGTGCGTGTTCTGATTCATTACATCGTTAACGCAGGGGACTCTGTATCACCGGAATTTATGCGGGCGCTGGCTGAGCGTCTGCCGCAGCATGAGGATAAACTG
ATGACTATCGCTGAACGTCTTGAGCAAAAAGGTCGCCAGGAGGGCAGGATGGAAGGTGCACTTGAAAAAGCCCTGGCTATTGCGTGCCAGCTTCAGAAAATGGGGATGACGCCGGAGCA
GATTAGGCAGGCTACCGGGCTTTCCGATGACGAACTGAAGAAAATCACTCACTGA
>PHINNC_00200 Transposase
ATGAGAGTATCACAGCCCGGCAAAATGCCGGGCTTTTTATCCAGCAGGAGCAGAGCAAACAGCATGACGCTTGCAGAACGTCTTATACAGAAAGGTTTCGACGAGGGTTTTAAAGAAGGT
TTTAAAGAAGGTTTTAAACAAGGGGCTCTTGAAGTGGCGCGGGAAATAGCCTGCCGGCTTCGGGATATAGGCTGGCCGCCAGAACGGATTCAGGAGGTCACGGGACTTTCCGGTGAAGA
ACTGAAAAAACTGTTTCCTGATGAGCAGTAG
>PHINNC_00205 XRE family transcriptional regulator
ATGACCGGCTGGGAACTGCGAATCTGGCGAAAAAGCATGCTCTGGTCACGCGAAAAAGCGGCCAGAGAATTTGGCGTGACACAGAGAACATGGCACGCCTGGGAAAATGCGGAACAGG
TGGACGTCACCGTCTGGAGGACCACCCAGGCACTTTCAGTCCGTGACCTGCTGCCTCACATGCAGGGGATGCGCAAGGCAGATATCATCAGGCGTCTGGAAAATGAACTGGGGGAAACG
GCAGAGGATGTGTGA
>PHINNC_00210 DUF4942 domain-containing protein
ATGAATCAGACTTTACCCACTGCTGACCTGAATACTGCCGGCACGACAGATGTTATTCCGTCTGTGGCCATCGACCGAATCATCGCGCAGCGTAACGAAGGTATTGCACTGTTCATGCAGGCG
ATGGAATGCCTGGCGACAGCGCGCAGGATTCTGCTTGATGCGTCAGGTGATATTTTTCTTTACGGTTTTGAAGACTGCGTGACCGACTCCGTTCGGTGCATGGATAAACCGGAAGAAGCGA
AAAGGAATATCACCCGTCTTGCCGACCGTAAAATCTGGGATCGCCTGATGACGGATACGGGCATGTACACCTTCATGAGTTCATGCCAGCGTGATGAGTGGAACAGCCAGCTGATGAGCGA
CACCTGTCCTGAAATCACCCTGGACAATGTGCTGGCAACTTTTCGCCATCTGAATGCCAGCAAGATGCAGACATTTGAACAGGGACTGATTGATGTCTACCGGAAATTGTCATGGGATTACA
GAACCAACAATCCCTGCCGTCTGGGCAAGAAAATCATTATTGAAAACCTGCTGTACCGCTGGAGTAACGGGCGTGTGACGCTGGACTGCAGCGGACGGGAGGCACTGGATGACCTGGTAC
GTCCGTTTTATCTGCTGGAGGGGCGCAACGTTCCTGACTTCCGGAGCAGCATCGGGGTGCAGTATGGTGAATTTCTCGGGAACGGCGACAATATCGGTGAGTTGTTCGAAGGGGAATATTT
TACGGTGAGGGGATACCGGAAAGGTACGGTGCATATCACCTTCCGTCGCCCTGACCTTATTGAGCAACTGAATAACATCATTGCCCGCCATTATCCCGGCGCACTGCCGCCACGTGTCTGA
>PHINNC_00215 Lipoprotein
ATGAAAATGCCATCAGGATATACCCGTATTCTCATTGCGGCGGCCATTATGAGCTGCTCAGGAATGGTATTTGCTGCACCACAACCTGCACCATCGGCTGAACAGCTGCCGCCTCCATGCCAG
GAACAACTGGTACCACCACCGGCACAGCCACCGTTCTATGCGTTATCACAGCAGACCACCACACCGGCAGAAACACTGAGCACGGTGGTGAAAAATATTCCTGCCGTGGAAAAAGGGAAG
AAGTATGAAATCCGCGTGGAAGTTCGTGAGGTGACACCACCACCGGCACCGGATATGGATGCACCAAATCCTTAA
>PHINNC_00220 Molybdopterin-guanine dinucleotide biosynthesis protein MobC
ATGGCAGGAAAGAAATTTTTTTCCGATGACGATATTGAAGCGGCACGTCTTGCACTCTCTGAACTCCCTGACCTGACCGCAAAACGTAAAACACTGCATGATTTTCTGTCTGCAATTCGGGAT
GACATCATCGTCCTTGTCAGGACAAAAGGTTATACCCTTGCCGACATCAGAAGCACACTGAAGGAAGCCGGTTATGAGGTGGGGGAAAAGACACTGAGGGATATCATCCGTGAAGCTGAA
TCGAAGAAAACCCGACGCCGTTCTGGCAGGTCTTCAGCAACAGAAAAAGCAACATCTTCTCATATTGAATCACAGGATAACGTAAAAGTACCGTCAGCACAGTGA
>PHINNC_00225 Mobilization protein
ATGAGTAAAAGCGAAAAACGGATTAAGGATGCAGTGATTCCCCGGATTCGTTGCACTCAGGCAGAGAAGGAAACCATCACCGAAAAGGCGAACTTCTTTGGCGTCAGTGTTCCCGAATAC
CTGCGCCGGCTGGCTCTGGGAAAGCCGCTGATCCCGGCCATCGACCAGGACATGCTTTTCGAACTTCGCCGTCTCGGGGCACTGCAGAAACACCTCTTTCTTGAGGGAGGACGTGTCGGC
GACAAAGAGTATTCAGAAGTGATAGTGGCCCTGCGTGAGTGCGCGGATGCGCTGAAAAAACGCATCAGAAACTGA

>PHINNC_00230 Relaxase/mobilization nuclease domain-containing protein
ATGGTTCCGGTAATACCACAAAAACGACGCGACGGCGGGTCATCCTTTGATGACCTGGTGTCGTATATTGTGGTCCGTGACCATGAAGAGATGGATAAAAAATACCAGAAGGTTGAGGCCT
GCGAACTGCGCGGTTCGGAGACGAAGGGTAATCGTTTTTCGCGTATTGTCGACTATCTCTGCAAGGAGAGGCAGTCTGGCGTTTCATCGCTTGAGAAGGTCTACAAGGATGGCGTTTTTCA
GGAGAACTTCCACGGAGTGACCTGCTTTCATAACTGCAGTTCAGTGGAAACGGCAGTGGATGAAATGCAGTCTGTCGTTGATCGTAAACGCAGTACGCGTGGCGATACTGATCCTGTTTTTC
ATTACATTCTGAGCTGGCCGGAGCATGAATGTCCCCGTCCGGAGCAGATTCAGGACTGCATTAGTCATACGCTGAATCGTCTGGGGCTGACCGGGCATCAGTTTATGGCTGCAGTACATACG
GATACGGATAACCTTCATGTGCATGTTGCCGTCAACCGGATACACCCGGAAACGTTTCGAACCCGTGCTGTCGGAATGGCAATAGATCGTCTGCATAAAGCCTGTCGCGAGCTGGAAATAAA
ACATGGCTTTGCTCTGACAAATGGCAATTTTATTGTTGATGAAAACCAGCAGATTGTTCGTCGTTCCGCGCATGAACGGAAACAACTGAGTGCATGGAACTGTGATGCGCAGGAGAATTTCA
GAAACTATATTCGTGTAAAAACGTTACCTTTTGCGGGTAAATTCCAGACTCAGGGATGGCGTGCTTTTTATCTGTTTATGGGTAGTAAGGGTCTCACGCTGGACAGAATGAGCAATGGTGAAT
TTGCAGTTCGCGATATTCGCAATCCTGAACGTTTTGTTGTGCCGTTATCTGATTTTGGTCATGAGTGGAAGATGCAGTCGCTCGTAGCCGTCATGGGAGAATGGCAGGTGCCTTCCGAAAATA
TTCTGCAGCAGCTGGAAAAGCTACGGCACAGACCGGTATTCTTCGAACCCAAAACAGATTATGTATCCCGTCCGCTTTACCGGATGCAGGAGAAATACAGTCTGGCTGATTATGCGCGGGA
GCATATTCATGATGCATTTGAAAAAATGACATGGCGTGGCGCTCATCATACAGTTGATGAGCTTCATGCCCTGTTTGCCCGTAACGGTCTGTATCTGCAGGTCATGAATAACGAGCTTGTGGTG
TGTGACGCTTACGACAGAAAACGAACTCCCGTGCGTGCTGAATCTGTTCATCCTAAATTGCAGGTGCCTCTGTTAGCGGATCTTGATGGCGGCTGGAAAGCCGTGCCACGGGACATTTTTAC
GCATGTCCCGCCGGAGCGTGCATATAAATCTGAAGGGCTTGAGCGTGTGGTGCTCAGTGACAGTGAACGGCAGAAAACTTTCTTCGGTGCAGGTCCACAGGGAGCACTGAAACGCGAAA
TGTTCTCTGACAGGGAGTCCCTTTATGGCTATGCGGTTGATCACTGTCGGCAGAAAATTGATGACATGATCCGGGAAGGTCGTTTCACGTGGCAGGCGTGTCATGAGATGTTTGCGAAGGA
AGGACTGCTGCTGGTTCCGCAGTACAAAGGGCTGGTAGTGATGGATGCGTGGCGGAGTATTCCAACACCGGTGCGTGCAACGGCCATTCATCCTGACCTGTCACTGCAGATTGCGGAGCA
GCATGCTGGTCCGTTTGAGCCGGTGCCGATGGATATCTTTGAACGAGTGCCTCCGCAGTCTCAGTACCGGCAGTCGTTAAGTGCACGTGAGAAAGCGGTGCGTATGGAGCGTCGTATAGAG
CGTGCCACTGAACGTGCGAATCTGCGCGAACGCTATAACACATTCCGTGCAACCTGGCAGAAACCGGATCTGCAGGCAAAGGAACGTTATGCGCGTATCAATGAGGCGACCCGTCGTGAG
AAAGCGATCATCCGGCAGCGTTTTCGTGACCCGCGTATCCGGAGAATACATTACAACGCAGCAGAGCTGCGGCGTTATCAGGCCAGAATGAACCTGAAGGACATGCTGAAAGAAGAACGC
GCACGTCTTGCTGAAGCCGGGAAGCTGCATCCACCTTCATGGCGTCAGTGGGTGGAGCAGGAGGCGCTGAAGGGTGATAAAGCAGCGATTTCCGCGCTCAGGGGGATGGCATATCGTGA
AAAACGGGGTAAGAAAGAAACCGTCACTACGCCGGGATTTGGTGTCATTAAATTTGATGCCGGAATTGATCCACAGATGATGAAACTGGAGGGGGCTACCGGTGAGTTACGTCGTGACGG
CAGCATTGTATACAGGCAGGATGATAACGGACGGACAATCTGTCGGGATAACGGGGACAACATTGTTCTTAACCGCGACCCACAGAGTGGTGTGCTGGGACGGTCTGCACTTAAAACAGTA
CCGTTGATATTTGGGCGGGAGAGTGAGCGTTTTGAACCGGATGGTAATGATCCTGCTCTACTCCGCTCGTTTGGTGAGATTGTTGCCTGGCACAACCGGAAGGACCCGCAACATATCCGTAT
TATCAGCAGAAAAGATGCCGATGATTATCGTCAGGCCGCTGTGAGCAGACACCAGAAGCGGGTTGAAAAATCCGAACGGGATGCGCGCAACTGGCAGTATGTTGCAGAGTACCTGGCGGA
ACAAATGGATCGGGAACAGCAACAGCAGGACGGTAATGCTCATGGCCCGACTCCGGTTGGTCATAAGGGGCCGGGGATGGGACGTTGA
>PHINNC_00235 Conjugal transfer protein TrbC
ATGCAACACAAAGTCAACGAGCAGTTTATACGCCGCAGCGCGTGGCACTCCCCCGTCACCTCGCTGCTGCAGAATCCCGACTTCTTTGGCCCTGCCATCATGGCAATGATGATACTCCCGCTC
TTTCTGCCGTTACTGACCCCGCTCAGTATCCTGGGCATGCTCTTTACAGGCGTCATCTTCAACAACCACCGCTGGCGCTGCCCCATGAGGATGCCCGCCACGCTGAAGTGTGACGACCCCAG
CGAAGATCGAGAGGAGACAGCCCGCTTTCTTGGTGCATGGAATTACACACGCATCAGGCCGGGTAAAGCTCGGGGGGTATTCTTCCTCGGCACACAACGAGGCAGTGATGTCGGTCGCGA
GCTCTGGTTATCCCAGGCGGACCTGGTCCGACACATCATGTTCTTCTCCACGACAGGGGGCGGTAAGACAGAAACACTGTTCTCCCTGATGCTGAACTCACTCTGCCATGCCCGGGGCTTCA
CCATGATGGACGGCAAGGCACAGAACGACACGGCGAGGACCATCTGGTATCTGGCCCTGCGCTTCGGTCGTGAAGATGATGTGGAGTTCATCAACTATATGACCGGCGGACGCTCCCGCA
GTGAACTGCTCCATGCCGGCGACAAATCCCTGCCGGAATCCAACTCCTTCAATCCCTTTGTCTACGCCACGGAGACACTGGTCACCGAAACGCTTCAGTCGATGCTGCCCCAGAACGTCCAG
GGGGGGGAATGGCAGTCCCGCGCCATCGCGATGAACAAGGCTCTGGTGTCGGGTGTTAAATACCTGTGCGTCACAGAAAAAAGGTGCATGTCGATGCAGCTGCTGCGCGAATACATGCCT
CTGGAAAAGATGGCCGCGCTTTACGTGACCGGCGTCGATGCCCGCTGGCCGGAAGAGGCGCTTTCTCCCCTGAAAAACTACCTGCAGGAACTGCCCGGCTTCGATATGGCGCATGTCAGG
ACGCCGGCCATGTGGACCGAAGAACCACGCAAACAGCACGGCTACCTGACCGGCCAGTTCTCAGAGACATACTCCACTTTCACCGAAACTTACGGTCACATTTTTGCCCAGGATGCGGGTG
ATATTGACCTGCGGGACAGTATTCACAGTGACCGTATCCTCATCACGATGATCCCGGCCCTTGAGCTGTCCTCGCATACCGCATCCGCGCTGGGGAAAATAATGGTGACGCAGCAGAGCATG
ATCCTCGCCCGTGACCTCGGTCACCGGGTTAACGGGACGGACGAGGAAGCACTGGAAGTCAAAAAGTACGCAACGACGCTTCCTTATATTAATTATCTTGATGAGATTGGTGCCTACTACAC
AGAGCGAATCACCGCAATGGCAACGCAGGTACGCTCCCTCAACTTTGCCCTGCTGATGGCCGCACAGGATGCGGAGCGTCTTGAAGGCACTGCCGGAGGAAACAGCGTTTCCACCCTGAT
GCAGAACGCCGGCGTCAAGATCAGCGGGAAAATCGTCAGTGACGAGAAAACCATGAACATCCTGCGTCGTGCAGCCGGCCAGGAAGCTCACGCCCGGATGGATACGCTGAAGAGGAAG
GATGGCCTGCTGGGAACCGACTGGCAGGACGGTGATCAGACGCGGATTGTCGATGAAGACAAGCTGAAACTCAAAGACCTGATCAATCTTCAGGCAGGGGAAAACGTCACCATTTTCAA
CGGACATCCCGTGGCCGGCGCATCAATGTATATTCCGGATTCAGAAAAAACGTCTGGCACTCCGGTGCAAATCAACCGGCTGATACAGGTCGACCCGATTCCGCTGGCAGAACTGCGGCAT
CTGGTGCCCCGGGAAAGTGAACGCCGCATTCCGCGACCGGAGAAAGTCTCCCGCCTGATCGGGATACTGAAGGGAGAAGTGGAAAGTCCCGTCGACAGCTTCACACTCATCAGTCCGATG
ATGCGCAGGCTGCACGATGAATGCGCCAGCCAGGTGCTCGACATGCCGGATATTTTCGACATCAGCGAAGCGGTTTACAACCTTTACAGCCTGCTGTATACGGCAGATATCCGTACCAACCG
GATATCACTTATCAGCAATGCGCCTCCTCAGCTGTCGTCCGGGGATGGGGAGTGCGATATTATACCTTTCAGTCTGTCCGGCACCCGGCCTTCCCCTGAGCAGCTTTTCTCTGCGATGATGAA
GGCAGCTACACACTCAGGTGAAGATACTGCGGATGCTGACATGCAGAATGCCGGTACTGACGCTGGCGTGGCTCCCTCAGCAAACGAGGCCTCATCCGGTGAGGACCACGACGAACTCTT
CGACAAAGTGTTGTCAGGGTGCTGA
>PHINNC_00240 Single-stranded DNA-binding protein
ATGGCTTCGAAAGGTATTAATAAAGTAATTCTGATAGGTAATCTGGGGCAGGACCCGGATATTCGATATATGCCAAACGGCGGCGCTGTGGCCAGCATTGCGCTGGCCACCTCCGAAACCTG
GCGGGACAAACAGACAGGGGAGATGCGGGAACAAACCGAATGGCACCGCGTGGTGCTGTTCGGCAAACTGGCGGAAGTGGCCAGTGAATACCTGCGCAAAGGAGCTCAGGTGTACATC
GAGGGTCAGCTGCGTACCCGAAAATGGACCGACCAGACCGGCCTGGAACGATACACGACTGAAGTGGTGGTGCATACCGGCGGCACCATGCAGATGCTGGGCTCCCGGCAGAATAGTGC
ACAGGGTGGCCGGGTACAGCCTCAACAACCACAACAGGGCAGACAGTTTTCAGGTAATTCGCCATCTCCGGGCAATACCACACCGTCCGGCAGTTTCAGCGACGATGACGACAGTATCCC
GTTCTGA
>PHINNC_00245 DUF4912 domain-containing protein
ATGTACCTGCATCTTGTTCCAAAACTTTTTCATCGCATGGCTAATAAATGTAGCCTGAAATCCATTTCCATACCTGAACTTGATTTTATAATTGACAGCGATTCGTTATCTGTAGGCAGGCCATGG
CCTAATAAATGTGTATGGGTTGGAATGAGGAAAGGAAGGAAAGCCGTTAATGGTCTGATTTTGCAAACAAATAAAAAATTAAGACGGTTTACCACCATATACACCTGGGATATTGAAAACAT
GGGGTTAATTCATCATCAGGTCAATGCTTATATAGAAGATGATGAATTTGATATGGTGTCACAGGAATTTCTTTTAAACGGTGCTTTTGATAAGTGGAGCGACAGAGTACATACCGCTTACGAG
AATAAGCCGCCAGCAAGAATCCAGCCCAAAATGGAGTCCTTGTTAAATAGTCCCGGTAAAGACAGTCATGATGTATGGGAAGAGTTTGAATGGGGAAACTTCCTTATATCCAGAGAAGAAA
GTATTTTACTTCACACAATTCAGTCTGAACGATTAAACACCGACTTCTCTTTATTCAGGAGACAGCCACCAGTTAATCAACCATTACTTGTTTAA
>PHINNC_00250 Thiol:disulfide interchange protein
ATGTTTCAGAACGATATTTTTGACGAAGACCAGAACAGCACCTGGCATGCAGAATCCACTGACAACTATTTCATCGTCACGGAACGCTACAGTGACAACACAGAAAGGGTGGCACTGGTAC
TGAAAAAGGACATAACACACCTGTACTCCCCGACGGACGCGAGCGGCATCAGACTCTCTTACAGTAAATGGAGCGGTGATGAGCTGACTTTAATGCCCGACCTGTCGCCGGAAAAAGCAC
GCAGAGTGGTTGATGCCATTGCGGATGAAATCCGCCGCAGGGAGAGTACCCCGGTCATACTTCACCCGCTAAACAACATAAAGGCTGAACGCAGTATCACCCGGTTCGCCCGGCGAATTGC
TCCCGTGGTCGGCGGCATACTCATCGCAGCCGGACTTATGTGGATGATATCGGGCTGGAGCGGCCCATTCACCGCCGCTGAGCATATCTACCGTACCGGGATGACGGAACAGTATGTCGGG
GATCTGAACAGGCCGGAGCCGGCGCACCGCGTTAATGAACACTCCCTTCGCCAGGTGGTCAGTTCAGCGGTGGCAACCCGTGACGCCGCCACACAGCATATCCGTGACCGTTTGCCGGCG
ACACCTGATGCCTTGACTCAGCCACAGCCGTCTGCAGAAAGGGCGGCCCCTCCGGACACGTCGACACAACCGACGGCAGGTGAACAGCGTCTGGCGGAAAACCTGAAACGCGCAGATG
AACGCAACATTTTCACGGTTCCGCTCTCTGAAGGACATGAGCGGACGCTTTATGTTTTCTCTGACCCGTCATGTCCGCACTGCCGAGATATCGAACCTGTGCTGGAGCGTCTTTCCGGCAGTT
TCAACATTCGCATATTCCCGGTGTCCGTGATTGGCGGAGAAAAATCGGCCAGCATGGCACAGAAAGTGCTGTGCGAATCCGATGCCACTGCCCGCCGCAACGCCTGGAAGGCACTGTTCAG
CCCGGGCGAGGAGATGCTGTTTCCGGTTAAAAAGGAGAACGGGGATACGAAAGCAGCATCAGACTGTGAGCTGGCTTCCCGCGCCGTCAGTGTGAATGACAAGGCATTCCGGGCTTATC
AGTTTGCCGGAACGCCGTGGGTGCTCAGTGACCGTGGTCAGGCCGTCCCGCAGGAGCTACTGAACAACGCAACAGAAGTCGCTCTCTGGCTGAAACTGAACGACAACACCACGAAATCC
GAAAAATAA
>PHINNC_00255 Conjugal transfer protein TrbA

ATGAGTACGTCCACAAACAGTGTGCAACAGGGTGAGCAGGGGCTTGCCCTGGTTGCCATGATCATCATTTCGATCATTCTCTTTCTGGTTGTCTGGCTGAAAGGCACGGTATATACCACCTGT
GCGTTTCTGCACAGCTGCTGGGGCCTTCTGCTGGCTCTTCCCCGGGATTTCGCCCTGCACAGGCTGGCAGCAGAGAAGTACAACCTGCTCGCAGAAACGGCCGCACGAGCTGACAGTATC
ACCGCCTCCGAATGGCTGGACGTCATGGCAGACACCGTCACGATACTTTATGCCGTGCTGTTCCCGCTGGGGCTGCTGCTGTTATATCTCTGGTGGGGACATCCGTCCCAGAACCGGTTCAG
CCGCAGACGCGTCAGCATCACCACACTGCCACACATGCTCGCCCCCCTGTCACCGGCCTTGCAGTACATCCTCGCCCGCAGTAACGGTCGGAAACGGCTTCTGCTGGACCTGCCGTCCCCG
ACAAACCGCCTGGCACAGACACCCCAGGCCTTTGTTAAAAAGTACAGACTGGTTTCCGGCAGAATTCTGGATGAAAGGAAGACGGCAGCCTGTTTCATGAAACAGGCCGGTCCGGCGCT
GGCGGGGTGGAAAAGCCTGAGACCACATCACCGGGCTTTATTTACCGTCTTCGGACTACAGTTTTTTCTGCAGGACCGCCCCGCCGCAGAAGCCCTGCTGGACAAACTGAACATCTCCGCC
GCCACGGCCCGGAAAACGGGGGCTTACCGGAAATGCCGGCATCCGTTGCCGGACTGGTCACTGGTGACACAGGATTTCATGCGCGTGGCACGCCATCCTGCGGCACGCCGGTGGCTGAA
AGAGCACCGTTTTGTACGCAGTGGTCTGGTCTGGCTGTATGCCCATGACCTTCGCCTGAATTCGCCCCGCTGGTACTGGCTGAAAGAACTCGAGCGGCCACTGTGGTATGCCCTGCACAGG
GCAAACAGCAGCAAGGGATTCATTGAGGGGGCGGGTATTGTGGCCATAGCCAGAAACGAAGGGCTGGCGAAAAGTCTGCACCTTCCGGTTCCGGAGCCTGACGTCAGTATGGCGGTCCG
GGGACTCCGGGCAGACCTTATCGCCTGTGGCCTGTTGTGGGAGGAAGAATCCGCCCCGCCCGTACAACAGGAAACACACAACGCCGGCTGGGAATGCCCGGAGCTGTGA
>PHINNC_00260 DNA topoisomerase
ATGAGACTTTTCATCGCCGAAAAGCCTCAGGTGGCCATGGCCATCGCTGAGGCACTCTCCGATATCAACAACACGCCTGTTTCCAGAAAAGACGGCTACATCCTCTGCGGGAAGGACGCCA
TCACCTGGTGTCTCGGACATCTCCTGCAGCTTCAGGAGCCGGAGACCATTAATCCTGACTGGGCAATCTGGAAAGCAGAAGACCTGCCCATCTGGCTGTGGCCTCCCCGTTACTGCCCGAG
AACGCGCCGGGAGCCTGGCAAGCCTCCTGCCGAAGACCCCGCTGTACTCAGGCAACTGAACGTGATCTCTTCCCTTGCCCGACAGGCCTCACGTATCGTGCATGCCGGAGACCCGGATGA
CGAGGGGCAGTTGCTTGTCGAGGAGGTTCTGGTCTGGATCGGGAATCAGTTGCCGGTGGACCGTATCCTGATTAACGACATCACGCTGGCCACCGTCAAAAAAGCCCTGGCGGACATCCG
CGACAACCGCCAGTTCTGGCCGCTCTACCAGCGTGCACTGGCCCGCAGTGTGGGTGACCAGATGTTTGGCTACAACATGACCCGGGCCTGTACCGTGGCGGCCCGCGCACGTTACCCGGA
AATGCGCGGCGTGCTTCCGGTGGGCCGGGTTCAGACCCCCACGCTCGGACTGGTCGTTAACCGCTGGCTGGCCGTCAAAAACCATCAGGAGGCCTGGTATTTCACCGTGGATGCCGCATT
CTCTGGCGCGGAACACGCTATCGAAGCCCGGATGCAGGTACCGGAAGATGCCCCCTGCGATGAGAAAAAACGTATCAGTGACGAGGGCTGGGCGACCCGTTGCGCGCAGTCACTGACAA
AACAGCCCGCCGTGGTGGAGAACGTCAGGACAGGCACCGAGGTAAGTAATCCGCCGCTGCCACTGACGCTGCTGGATCTGCAGCGCCTCATGGCCCGTCAGCACGGCATCGATGGCCAG
ACCACGCTGGCCATCACACAGTCCCTGCGCGACAACTGGAAAGCCATCACCTACAACCGCAGCGACTGCCCGTACCTTTCTGATGATCAGTATGCGGAATCCCCCGCACTGCTTGACGCACT
GCAGCGCACGGGCTGCTGGCATAGCCTGCCGCTGGACCCGTCACGCAAATCTGCCGCTTTCAACACTGCAAAGGTGGGGGCGCATACCGCTATCATACCCACGACGTCGCTCCCCGATCTG
AGCAGACTGAATGACCAGGAGCGCCTCGTCTGGCAGACCATTGCGGAATACTTCCTGGTGCAGTTCATGCAGCCCCGCCAGCGGGAAGTGGTGACCGTAACGCTGACCTGCCGGGGACA
ACGTTTCATGGTTCGTGCACGACGCCTGCTGGAGCCGGGATACACCTCGCTGCTGAAAGCCTCCCGACAGGAAGACACGGAGCAGGATGAACAGGGCACACTGGCCGGGGATAACACGG
CCTGGCTGAAACGTCTGGTACCGGGACAACAGCTGCAGTGTGTCTCCGCCACGGCAGTCCGCCACAAAACCACACCGCCGCCCCTGTTTACCGAAGACACGCTGCTGGCAGCGATGGCAC
GCATTGCTGACTTCGTCACCGATGAACGGGTCCGGGCGCTGTTGCGCCAGAAGGATGAAGACAAGGAAAAAGAGAACGGCGGTATTGGAACAGCCGCCACGCGGGCCGGGCATATCCA
GCTTCTCAGGCGTCGCGGCCTCATCACGGAGACGAAAGGCAAACTCATTCCCACCGAAAACGGCATCGCCCTTATCCGCAGCCTGCCGCCCACCATCACCCGCCCGGACCTCACGGCGCTC
TGGGTCGAACGCCAGAATGCCATCATCGATGGCAGGATGACCGTGGAGCAGTTTGCTGAAAAGCTGCACGAGGAACTGAAAGTGCTGGTTGTCCGGGCTGCGGATATCAACGGAATAAA
ACCAGGCGGTGACCAGGCAGTCAGCCTGCCCTGCCCCGGTTGTCGTAAACCGCTCCGCTTCTGGAAGGACAACGTCAGCTGTTCAGCCCGGTGCGGGTTCCGCCTGTCAAGGACGCTGCT
CGGCAAGGAACTGACCATGCATCAGCTGACCACGCTTCTGACCACAGGACGCTCCCCGTTAATCAGGGGTTTTAAAGGGAAAAATGACAAAACTTTTGATGCGTATCTGGTGTTCCGGGAT
ATCAGGAAAGGCGATATCGGCTTTGTGTTCCCGGAGAGAAAGAAAAAACGTTAA
>PHINNC_00265 sok antitoxin (CssrC)
GGGCGCTTGAGGCTGTATGCCGAAAGCGTTTTGTGGACGGTATACAGCAGAAAGCCCCTGGAGATTTTTTATCAATCAACCAAGGGCTCTACTGTAATGCCTAGACAACATTATAGTAGCC
CGATAACCGCCGTAAGGCAATGGAGGGGCTATGATG
>PHINNC_00270 Membrane permeability altering protein
ATGCCACAGCGAACGTTTTTAATGATGTTAATCGTCATCTGTGTGACGATTCTGTGTTTTGTCTGGATGGTGAGGGATTCGCTTTGCGGATTCCGGCTCCAGCAGGGAAACACAGTGCTTGT
GGCAACGTTAGCCTACGAAGTTAAACGTTAA
>PHINNC_00275 DUF3150 domain-containing protein
ATGTCAGAAAAGTGGAGTAAAGAATTCGACTCTTGCTTTCAGGTGGGGAGCATAGAAGAAATTGCCGATGCTCTCGAGAAACGCAGCGGAAGCGAGGAAAATGCGTTAGCCATGCTGGC
AATGACAGCCTTCACACTCATGACCCGACGTGGTATCTGCGAAATGCAGAACGTGGCCCCCGATGGCAAAGGCATTCGGCTGGAGCTAATCGGCTTCACAGGAAATGAAACAATACCGGAT
ACGCTTCATTGA
>PHINNC_00280 ArsR-gov region gene B
TCAGTTATAGACAGTTCTTCTCCGACCAGAGCCACCAACTCTGTAGCGGAACAGTAAAAAAGCCGACCTAACCAGTCGGTTTTTTTAC
>PHINNC_00285 PH domain-containing protein
ATGTATAGCGAAGTAAAGAGATATAAATGGTATCAGGAAATCGCTCAGGCATTATGGTATCCGTACTTTTTTGCTGCATTCCCTTTAATTTTATTTTGCTGTTGGTTTACCATATCAAATAATACCG
GTTTTTATTATACCAGAACGCATGACAGATTTTACTACTGGGCATGGTTTTGGTTATTTATATCTTTCATTCCAGTCATCATCCACTTCTGGACTATGCACAAACGAACAGCAGTCCTGAAAAGA
GTTGTTGAATATGTGAAGGAAGAAACAGGATTCACTCTTGCACGGGGATGGAGAGACGAGGTATTTCTCCGCTACCATTCCACCTGGGTGGGTATAGATAACATTAACGGTAATCTGTTATAT
GTGAAAGTATTCCCCAACGGTGTACTGGATGTTATAGGCCTCAGCCCGTCTAATATCAGTGCTTACGGAATTGAAAGTAATAAGTTAATCACATTACATACGAATCTGCCGCGATATCCATTCAT
CAAATGGAGTGATTTCGGTAAAAACGAAAGATTTTATGAGCTTATCACTCAATTCATGAATAATCACAGCGGTGACAACAGATTCCAGTCACGCATATTCTCGTTGCGCGAGAAACTGGAGG
ATATATCAGGCCTCATCGTTCCGGATATTTCTATCACAACCGGCAGTAATATGACTCATGCCGAAAAAGTCGCACAGGCCCATACAAACGCGCAAAAACTTAAAGCCTGGCAGGCGGAAATGC
AGCGCAAGGAAGACGAGGAGCGCGCGCGTGAACGCCAGCAGCAGAATGCAAACCATTCAGCACCGGCAATGGATGAACCGGAATATGTTGAACTGCCACGAATCCCTGAATTGTCACCG
GAAGAGCAGGCAGCACTGGACAAATCGACGGAAAAAGAGCGCGCGGAAATGGAGAAAATTCTGGGACCAATCCCCACGTCATTCAATTAA
>PHINNC_00290 DotA/TraY family protein
ATGAATAAAAAAATCAGCCTGCTGGTCACAGCGGGCCTGCTGCCATTCTGTCTGCCGGCACAGGCAGACGACGTCACATACGAAACCATTGCTTCCGCAGCGGAGAAAAGCACTGACCTGT
CCCGTCAGGCGCTGGTCACCATATTCGGAGATGTGGTCGTCAACCCGTTCAGTTTCACGAACGCCAGTGTCATCGGCAATACATTTGCCATATTCAACGGGATTCTGTGCGGGCTGGCTCTGT
TCTGGTTCGGCTTCATTGGTATCCGCAAGGTTATCAGCGCGGCTCAGGCCGGACGTTTTCAGGGCGCATCCGCAATTATCAGCACCCTGCTGGCGTTTCTGGGCATTGTGCCCACGGCCAGC
GGCTGGTCACTCTCGCAGCTCATTTTCCTCTGGGGCGTCTCAGTGATGGGCGTGGGGGGAGCAAACCTGATAGTCACGACTGCCGGTAACGATATCGCCGGCGGGTACAGTCTGACGACC
CAGCCAACCAGTGCCAGCACAAGGACGGCAGCGCGAGGCATCTTTGAGATGGAGTTGTGCAAATATGCCATCAACCAGTCGCTGTCAGATCTGAACAGCCTGATGCAGTCGGAGACGGCC
TACATGACCACCACAACCAGCAGTACCGACAAAAGCTACACGCTCACGGTCAGTAACGGCAGTGGCACATGTGGCTCTGTTGAACTGCCTTATTCATCTGCCAGAACAGCTGTCTCAGACAG
CTTCTTCAATGGTCTGAGCGACCAGGATGTCAGCGGTATTTATACTGCCCAGAAAAACGCGCTCAGCACGATGATTAACACGATGGACTCGGCAGCAAAAACTTTCCTGACGACCTTCATTG
AACGCCGGGATAACAACACCGGCACCTTCGAAGACGTGGAAAGTATCATTCAGTCGGCTGCATCCAGCTATGAAAGCACGGTGCAGCAGGCCATCAACAACGTGAACGGTGAAAACACGA
TACAGGAAGCACTGACAGAGTACCTGGACACCCAGGGTTGGATCACACTGGGCGCGTGGTATCAAACATTTGCGACCGCCAACCAGAGACTGGCAAGCATTGCTAATCAGGCACCTTCCG
TGACATCGCTCTCATCAATCGGAGAGTCCGGTCCAACCGACCTGTACCAAGGGGTAATGGCGGCCTACAAAACCAACCTGCAAAACACAACTTATACACCGACATCAAGCACATCGGGAGT
TTCATTATCCAAATATGAAATGGATAGTCAAAATGCTGAGGACCCCAACACACTAATATCCAAGTTATTAGAACAGTCATTTGGAATTAGCATGATCCAAGGTTTCATAAATACCTTAAATGAGG
ATACTTCCAGCCCATTAATAACGATGAAAAAGATTGGTGACTATACACTAAGCGCAGTTGAAACAACTTATGGCACATATATTGCTGCACAAGCCATAACTAAAGGAGCAGGAAGTTCTGCCA
TTGGCTGGTTCTTTGATAAATTCACTGGAGCAGCTACATTTATTAATAAAATATTCTCCGCCACCGCACCGGCTATTAACTTTTTCCTTTTATGGGTCTTTGTTATTGGCTTTCAGCTTTCTATATT
TTTACCTGCAATACCATTCATCTTCTGGATGATTGCTGTCGGCAATTGGATAGTGAGTGTATTAATAGGTTGTGCAGCAGGTCCTCTCTGGGCTGCAACACATCTCGGCGTAGAACATGACAG
GGGAAGTCGTGCTGCCTATGGATATATTTATCTGATCGATGGGATGATCCGTCCTTCATTGATGGTGTTGGGTTTTATATTCGCATCTGTCGCCGTTGTTGCCGTAGGAACAATACTAAACAAGA
TTTTCGGTATTGCCCTAACGAATATCCAAGCAATTTCTCTGACGGGCATTATTAGCGTCATAGGATTCCTGATGATTTATGCAAGGATATGTACCTCGATGGTAACCAATATATTTGCACTTCAGG
CTTACATGCCAGATTATATTATAGCTTTCCTCGGAGGCAGAGAGGCAGCAAATACATTCTCCGGCATGGTTGAGTCTGTAAAAGGTATATTTGTTTCTGGCGGCAGTAACTTAAGAAGATCTCC
TGCCACAAACATTATTCAGAAGAAGGATACAAATAAAAATGATGATGGTATAAAAAGTTAA

>PHINNC_00295 hypothetical protein
ATGCCACTCATCATTCAAATCACCATCAGTGAAGACACTGAACGTTCACGCCGTAAAGGACAGGTTCCGGGCTGTCATATCCATGTGGATCAGATATCTCCCGTCATGGCTGCCACAGAGGCC
GAACAAATGACGGCAGAAATGCTGCTCAGTCACGGCTTATCGCTCATGACCCCGTCAGCATCTTTTCCCACCACGCCAGACGCCACCACCAAAAAACACTGA
>PHINNC_00300 Conjugal transfer protein TraX
ATGACACACTCCCCTGAAAACAAAGAGAGCAACATTCTCACACGCATGGGCCGGACCGTGCTCTGGGTCCTTCTTCCGGTCGACGCCCTGCGTGTTATCGGGAAAACCGCCCGCAACCAG
GCTAACGCCCGAATGAAGGATGCCCGGAAACACCTTCCCGGCCTGTCCGGCACAGCCGGCGATGAAACAACAGCACCGGACAATCAGACCACTGAGGGCTGGGCACAGGCCGTGGCAG
CGTCAGGCAAAACACCGGCACAGCTGGAGCAGGGATACCGCCGCCAGCAACTGGTATTCCGCTTTATTTTCTGGATATCTGCGCTCTCAACCGGTTACTGGACTACCCGGACTCTTGACACC
CCGTCCGGGCTGTTTTTCTCCAACATATTTCTGATGCTGGGCTGCGCCATGTGCGGATTATCGCAGTCACTCATTGCAGGATTCCGGCTGTGGCAGATACGCAACCGCCGTGTCAGCCCGGC
AGAAAAAGGCACATTTCGTCATTTCCTCGCTGAGTCAAACTGGTTCAGCGAGGCCACAGGTATCGGCAAAGGGAAACACTGA
>PHINNC_00305 Conjugal transfer protein
ATGCGCCTTACTTTTTGTCGGCTCCCTGCTGCCGTCTTCGGCCTGGGATGCTACTGCATACCACTGGCGGTCTCCGCAGTGACCGTGAATGTGAATTCAAGCGTTCCCATATCAACCGAGGTT
GTTCCCCAGCTCAGTACCGCAAACACGGCACTGGCCAGCATCCTGACCACTGAGCAGGAAATCGCAACGGCGATTGCGGAAAACGCGTCGAAAACCAATGCCATCATCACGCAGACAGCG
CAGGCAAACCGCGAACAGCAGATTTTCAGTAATCAGGTCCAGCGGCTTGAGGAAGCCCGTAGGAGCTTCACTCTGCCGGAAAGTATCTGCAGTGAATCCGCCTCCGGCGTGGCCGTTCAG
GCCCGCAACGAGGCGCGGGCCACCACATCCAGCCTGTCTTCCGGTTCAGGGATTTCAAAAGCCGCCGTGAAGGAGCGTTTCACCACGCCGCCGGCCTCCGAAGCCACGGATGCCTATACA
GGTGCAGCTGTTCATGCGTCGTACTGTACGGAGGTCGATTACAGCGCTTTCGGCGGAACCACTCTCTGCCCTTCCGTTGGCAGTTATCCGGGCGGCGACAGCCAGGTCCGGTCACTGTATG
ACGGAGCGGGGAAAACAGGAAAGACTCCCGACCTGACGTTCTCTCAGGACCAGATAGATGCCGCACAGGCCTACCTGAAAAACACGGCCAGACCTTCTGCCGGGCGAACGCTGAAAAA
AGGGGATGCCGTCACGACTACTGGCATGGCCTACACCGGCCTGCAGAATGAATTCAATGCCATTCTGGATGCAGCTGCAGAACCCCAGCAGTCCCTGATTGCTGACAGTACCCCCAATTCCG
CCACCACCGCTGCCGTGAACGAGGCCATTAACGGCTCTGATTCGGCAAAAAGCTATTACGAAGCCACGGTGTCAGATACGGCCAGAACGTCCGGAATGAGCAACCGGGAGTTTGAAGCCT
TTGAAGTGGGACGCCGCTATGCCAACACGGATTATCAGACGGACCTGCAGCAGATGGACGGGGACAACCTGACACGAGAACAAATCCGCATCAGTTCGCTGATTGCCTGGGAGCTGCACG
ACATTAAAGAGCAGATCAGGAAAAACAACATTCTCACAGGACAACTGCTGGCCGTCACCGCACGCGATTCCTACGCGGCACGACTGGGCGCGCTTGAAGACAAAATCAATAACGGAGTCA
GCCGATGA
>PHINNC_00310 ATP-binding protein
ATGTCAATCATCACCCGCATTGAAGACAGCCTCATCTGGCTGTCCCGCTACGGACTTGCCAGAGAATTTCCGGAATACTGCGATCTGGAAACAGTCATTGCACTGACCGACGATGACCGTATC
CGACACCCGGAACTGACCTCCCCTTACATTGCCGTCACTGCCAGAGGAGAATACATGTCGACACTGGAAATCGCCGGCGCACTGAAAGAGATGAGGGAGGATGTTCCACCGGATCAGATT
CCACCAGGAAGCCCGTGCACCTCTCTGGCGCATTTTGCACAGCACCTGACACCTGCGCTGGCGACGTTTTTCCGGACCCTCGGACACAAAATCAGTATGGTTCTCGAGCGGGACCCCGAA
GGCGGAGAAGCGGAAATCCGGAACATGCTGGCCCCCCAGTATGCCTCCCTGCGCCGGACCGGCATGCAGATGGAGGATATTCTTGACGAGAAAATCGCCACGCTGTCTCCCTGGCTGATC
CGCGAACGCTGCTGGCTGTCCGTCTGGAGCTCACATGAGGCAACCAGCCGTACGGAGTACACGGATTACCTGAAAAAGAAAACGCAGACAGAAACAGCAATGCCTCATGCCAGGTACGG
ACAGCCGCTCTGGGAACCGATGTTCTCCGGGCTGAAAATCCGGCATGACGCACTTATCAACACACTGATTCATGCCCTGTCTGCCGACAGGGAAGGTCAGCTTGTCAGACTGATGGAAACG
GCAGAAGTCTGCAGGGAACTGCGCAGCCAGATACTGCGCGGACAGGTTCCCCGGGACTGGCGACCGGTCCTTGCCGGTGAAAACAGACCCGCAGGCTGGCCACAGCAGGATGATATATC
CGCACTGCTCGCCCCACCACTGAACTTTCAGCTGTTCAGCGATGATGTGGAGACATCGGGCAGTCTGGTCCGGGCTGGCGGACTCTGGCATGGCACGGCGAGTATCGTGCTGCCACCGCA
GCAACTGCAGAGCATGAACGACCTGATTGTCCGTATTCCCCGGAGCGTCCCCTGGCGGATACGCATGGATATTGCGCCCGGCGGCATGAAAAAGCTCCAGGGAAAACGTTTTCTGGCCAGT
ATGGGACGCTTCATTAATTCCATCCGCCCCCTGAGCGATGCCATTGAGACGCTTGCTGAAACTGACCGCACCACGCCGGTCTGCGTCATGACCATCGTGGCATCCACCTGGGCGGCCACAAA
GGAGGACTGCCGACGTAACCTGATGCTGCTGCGTTCAGCACTGGAAGGCTGGGGCATCTGCGAGGTCACCACTACGTTTGGTGACCCACGCCGTGGCTGGGTGAACAGCCTGCTGGCTG
CCTCCACCTCATCCGGCCCGAACCTGCTCTACCCGCCGCTGAACGAGGCACTCTGGCTGGCGCCCTTCAACCGTCCGGCATCGGCCTGGTCAGATGACGCGCAGGTGATGGGGCATGTAGA
GGACGGGAAACCCGTTCCGCTGGGGCTGGCCAGCTCCCGACAGACAAAGTTCACGGAGATTGTCGGTGGTGAACCGGGCACCGGGAAATCGGTACTGGTGAACGCCCTCAGTAACGCA
ATGGTGACCTCTGCCATGCAGAAACTGCCGTTTCTTGCTGTCATTGACAAAGGGTACTCCGCGCTGGGTCAGATCCAGCTGGTTCGCGATAACCTGCCGGAAGACAGGAAAGATGAGGCG
GTGGGCATCGTACTGAAGAACAGCGACGAATACTGCCGCAACCCCTTTGACATTCTGTTCGGTGCCCGTTATCCCATCCCGCCGGAGCGGGAGTTCATGCTCAACCTGCTGTATGCCCTCTG
CATTGACGCCTCGAAAGCCGATGCCCCCAACCCGGCAGACACCCGACAGATTTTGTCCCGGATCATCGATGCCGCCTTCACGCAGACCGCAGAGACCGCTCCCCGCCTCTATGAATACGGC
ACCCTCCCGGCGGTGGACCGGGCGCTGGCCGAGAGCGGCCTGCGGGAAATGCATGACGCAGAGTGGTGGAGTCTCTGCACCTGGTACGACGTGCGGGATATGCTGCACGACAGAGGGT
ATCAGACAGAGGCGCAGCAGGCGCACTACCAGGCCGTTCCGGAGCTGACAGACATGATCGGTCTGCTCTCCACTGAAGCCATCCGGACCACCTTCGGCACCGTGCAGCGTGACGGCTCAC
AGGAGCTGCTGCTGAGTTATATCCAGCGCTCGCTGACCAGCGCCCGCAGCGAGTACAGAATGCTGGCAGGCCGGACACGTTTCGCCCTGAACCCGGACACGCGCCTGATTGCCGTTGACC
TGAACCTGGTGGCCGGAGATGCCACGCCGGCAGGAAGGCTGAAAACCGGGATTATGTATCTGTTCGCCGGGCAAATCACCAGCGGGGACTTCATCCTGCCGCAGTACCAGGAAGAGGTG
AAAAAAACGCTGGCACCGGCATACTGGCCGGCCATGCAGGAACGTATTCGCCAGCTGGACCAGGAAATTAAAACAAAGCTGTACGATGAGCTGCACAATGTGCGGGGTATTGATTTCATTC
TGCAGGCGCTGGAAACCAACGACAGGGAAATGCGTAAGTTCGGTATCCGGACCGTGCTGTGCTCCCAGTATCTGGGGGACTTCCCGGAGGCAATATTAAAATCTGCCAACTCACTGTGGCT
GATGAAAATCCGGGAAGGCGACAAGGAGCTTCTCCGGAAGCACTTTGGCGTACCGGACGTGACGCTGACACGCTTCTCACGACATACCGGAGGTGCCGCACCGGACGGCAGCGGAACAT
CGTTCCTCGGCGTGTTCAGAACCCGCCTGGGAACAATGGCCCGGATACTGAAAAACACACTGGGGCCCAGAGAACTCTGGGCACTGAACTCATCACCGGAAGACAGCGCCCTGCGCCGT
CTGCTGAATGAAGAGGTGGGTACGAAAACCGCACGCCGTATTCTGGCAGAGAACTTTCCGCACGGCTCGGCAGCCCGGGTGATTGAGTACCGCCGGAAACAGGCCGGTGACAGGGAAG
CAACGTCAGTGACGGCAGGACTGGCGACAGAACTGCTCGGAAAGCAGGGTTATCGTCTTTAA
>PHINNC_00315 Conjugal transfer protein TraT
ATGAACCTGCCTTATCCGGACGTTGTACCCGACACAACGTACGACCAGCGGCCACAGCCGCATCACGGCGGCATTTGTCCGGCCTGCCGGATGAATGCCAGTACACTCTGGCAACTACCGG
ACGCCAGCGTTCTCCGCCACCATGAACAGCATATCGTCACGCTGCAACATATGCTCGAGCCCGGCAGGAATACAGCGGGGGCAACAGAAGCCTGCCGACACTACCTGGAGCAGCACCTGC
CGTTCGGCACAGATGAAGATCTGACAGACACGGTGGATATCAGGCCCCTGACATCAACTGAAGCCACACAACTGGATTCACGGCGTCACGCATTCAGGAAAAACGCCGTCTGTGATATCTG
CCATGCCTGGCTGTGGCTGAACGGACCAGCGGCCACGCAGGGTGAAATGGTCTGGATGCCCGACCTTGCCGCCCCCTGGATAGTTGGCCTGAACCGCCTGGCACTCCGCGACATGATGAG
CACCAGTGATACGCGCCAGCAGGCCGGACGCCGCGCACTGGATCACCTGTTGCTGTTCAGTCGCCCCGTGCGCCGTATGTTCGGCTCCTCCCGCCCTGCGGATTTTGCTGCAACCCTGCTCA
GCGCACCGCCCGGTGAACGCAGCAACATGCGCAAACGCCTGCGGGGACTGGCGCTCATCCTGCGCCCGGCATCCCTTCATATCTACCGTACAGAGAAACCGTAA
>PHINNC_00320 Putative phospholipase D
ATGCTGATTAACAGAAAAAAACTGCCGGCCCTTGCCGTTCTCTTCAGTCTTGTCATTCTGCCATCCGTCTGTCCGGCTGCAGAGCAGGTAACCGTGGGATTCTCCCCCGGCGGCAGTGCTCA
GGCGGCAGTGCTCGATGTCATCGGTCAGGCCCGGGAAAGCATTGACCTTGCTGCCTATTACCTCACCTCAAAGAGCACGGCACAGGCACTGGCTGAGGCTGCTGAACGGGGCGTCAGTGT
ACGCGTGGTGGCAGACAAAAAAGCCAATGGCTCACGCTATACCGCCGCCACCTGGCTGGCCCGTCACGGTATTCCCGTTCGTGTGAACGGACATTACGACATCATGCACAACAAGTTCATG
GTTATCGACGGGCGGACCGTGGAAACCGGCAGCTTCAACTACACCGCCTCTGCCGACAAACGAAACGCAGAAAACGTCATTGTCCTTCGCGATATGCCGGATGCAGCACAACCGTATGCCC
GGGAATTCGAACGCCTGTGGCATGAATCCGAACCGCTGAACAGCGGGCTGTAA
>PHINNC_00325 Conjugal transfer protein TrbC
ATGCGTAACTATCTCCGCACGCTGTCCCTGTGCCACCCGGCTGAACGCTGGCTGATGATCTGTGCCCGTATCAGCTGGCACCTGACCCGCCAGCAGCATCGTCTGGTGAAAGTACTGACCGC
GCTGCTGGTTCCCCTGCTGCCCATTCAGGCTCATGCCTCCTCAGACTGGGCCGCAATTCTGAACAGTTGGGCAAACGGTGCCGACTCCGGCCTCACCTCCCTGCTGACCTACGCAAAATTCG
GGGGGGCTGCGCTGTTCATCGGGGGCCTGGTGGGTTTCTCCCGTGTGGGTAAAACCCCCAACGTCACGGCGAAAGGCTGCTTTATTGCCCTGCTCGTCGGGGCCGGGCTGTTCTCCGTGG
GGGCGCTAATCAACAGTACCCAGAGCCAGTCCGGTCTGGAAACCACCACCGTCGGTTAA
>PHINNC_00330 Conjugal transfer protein TraQ
ATGTCCACAGATGCACTGTCAGCCATCATCAGTTTTGGCAACAACATCTACAGCTCCGGTCTCTCCTTTCTGGTTTCCTGGAGCACATTCGTTGGTGTGGCAGGCTTTGCGCTGGTACTGGCC
AGCGCCCACAGCAGGGGAAACCGGGGCGGTCAGGTGTATACAGGGAAGACACTGCTCAGCCTCTTCTTCTGTGCCGGTCTGACATGTCTGAAGCCGCTCGTGAATGCTTCCGGTGCGCAG
TTTGGCTTTGAAATGACCTCCTTTGACAGCATTTCCTGGGCCCCCACATCCACGTTTGGTCAGGGGGCTGAAGCCGTCAACGCCCTGCTGACAGTGGCACGCCTGTTCGGTATTGGCTGTTT

CATGGGCGGCCTCAACACCCTGAGACGTTCCGGACTGGAAGGTCATACGGCACTGAGTGCCAGTGAAAACACCAGTCTCGGCTTCGGAAAAATCATTTTCGGCACGCTGCTCGCGTTCATC
GACAACGTGCTGGATGCCACGCTCACTTCGCTGAACATCCATTTTTAA
>PHINNC_00335 Conjugal transfer protein TraP
ATGAGTAAGCAGCACAACAACACCGGTGACTTTGACATCCCGGACATACTCCCTGATCCCGCCACAGACACAGACCTGCAGGTGCTCACGGCACCGCCACCGGACACAGACCCCGACCTTC
CTGAAGCGGACGAGGAAATCACGGATACCGATGACGATCCGGAAAGTGCCCCGGAAGATGAGGAAGATACCCCACTCCCCTTCTGGCACTGGCAGCGCCCGCTCTTTCTCGGCCTCAGTC
TTCCCATTATCGGCGGTATTCTGCTGGCCATTGTCGCCCTTGTCGTCACGTCACTGCCCTCCTCACCGCCGGCAGGCAACGACCTGACACTCACCCCGGAGGAAACGACGGCGGCCGACAC
AGCTCTCCGGACGGCACACACGCAGCCCCGGGCGATGGCGATGGACAACAACCAGCCCGGAGAAGCCGTCCCCCCGACCGGAGGCACTGACCTTCAGGCACTGACCCGCGCTCTGAAG
GAGGAAATGGATCAGCGAACAGACGCCCTGCAGTCGCAGATAACAAAACTGTCCGGGGGCGTCGCCGGCCTGTCAGATGCCCTGCGCAAGGATGAAGACGCCCTGCGTAACCTGATGAC
CCGGGTACAGGCGCTGGAGGCGGCAGTCCGGCAGACAACCACGGCCCCGCGAACTGCGGCGCAGCCTGCACAGGCGACAGACACCGCACCGGCTTCCCGGCGCACCACCGTCCACTCC
GCCATCAGTGGCATGCGTATTGTGTCGATGGACAGCGGCATGGCCTGGATACGCTGGAAAGACAGTACCTGGTCTGTCCGTCAGGGAGACCGACTAGGCAGTGTGACCATCACCGGTATTG
ACCCTGCTGAACGCACTGTGACCACCACCGGCGGAGTGATCCACTAA
>PHINNC_00340 Conjugal transfer protein TraO
ATGTCCACAGAAAAGGATGCCGGACAGTCCGGCAAAAAAGGCGTTGTACTGGTCGTCGCTGCGCTGGTACTCATCGCCGGCATAAGCTACGTCGGGATGGGCTGGTTCCAGCCACAGGAA
AGAACACAGAGCAGACTAGCCCTGAGTGCCGCCAGCGTCCGGACAGGCACCACCACCGATGAAACGGCGCATTACCGGGAGCTCCTGCGTCAGGATAACGATCAGGGCGCAGCAGAGG
CAGAAAAAAACAATACATCCTTCCTGGCCAGTATTCCCCGTGGCGTGGATATCCCCGTCGAAAACACAGACACGGGAAAAACCGGCCAATCCGGTAACGACGAAAAACGCACAACACGGG
AAAATAACCGGACGCCGCCGTCACAGCAACGCGTGAACGACAAGGACAGGCAGGAGCGCCTGAAGCAGGTGCTGGCACGCCTGCGAAATACGACACCGGCCACACTGACGGTGGCTAC
CGTACCCGGCGGGGAGAGCGGCGGGAGCGGGCGCAGCAACGGGGGACAGAGTCAGGGCATCTGGCACAACTGGACCTCATCGCTGTCCAGTGGGAACAGCATCATTCCTGCCACCCTC
CGCAGCACGGATGTGACCACACCGCCCGTCACGATAATTCCGGCGCTGACCCGTGTGCCGGCTTATATCGAGACGGCAGTGGATTCGGACAACACCACGTCTCAGGTGGTGGCGAATATCC
CTGCAGGTCCCTGGGCCGGGGCCAGACTGCATGCGGCCCGTATCACCCTTGTCGGCGATGGCGTTGAAATCAACTTCACCCGGATGTTCTGGCAGGGTACGGAATACAAGGTTAATGCCTG
GGCACAGGATCAGAAGACGCTGCAGTCTTCCATCGCCAGCGACGTTAACCACCGCTACGTGTCCCGCATTTTCCTTCCCGCCTTTCTGGGAGGCTTTGGCGAGGCCGGCTCGCTGTTCAAA
TCCGCCAACACACAAATCCTCACCAACCAGTACAGCACCCTCGCCAGTACGGCAATGCCGTCCGGCAAGGTGCTGGCAGGCGTGATTGGCGGAGGAATGGCAGAAAAAACTGGCAGTGT
CCTGCAGCAGGATGCGGCAAAACTGCCTGCCACCCAGGTCACCGTAAACCGCAATGAAACCATCGCCATCCAGTTTGTGGACGGCGTCTACAGCACGGATGCCATCAAACCCGGAGCCAG
CCGGACAACACAAAACGCACAGGTCACGTCGGAGAGCCGACAGCCAACACTGGCGCAACTTCGCGCCCGACAGAAAACAGAGGACGAATCCGATGAGTAA
>PHINNC_00345 Conjugal transfer protein
ATGCCAAAATTACTCCCTTTGACGGGGCTGCTTCTTGCCGTCCTGTCAGGCCAGGTACTGGCCGAATCCACAGGAACAGGAACGCCGGGGACATCCGCGCAGGGTACGCGGGGAAGTGT
GCCGGCCTCCCCCATCAGCGGCACCGCCCCCCTGCCGGGACTGTCTCCGGCAGCTCAGGAGGCGGCTGCCCTTGACTCACCGCTGACCCCGGACGAAATCCGGGAACTGGCAAAACGGC
TGCAGGCCACGGAGCAGGCGCTGGCCGCCCCGGTGACCAGCGTCGTTCCCCGCATCAGCTCACTGACGGTCAGTCTTTCTCCGGGGGCCAGCCTGCCGGTTCTGCGCCTTGCCCTCAATA
ACGCCAGCACGGTGACGTTCCAGGACAGCACGGGGGCCCCCTGGCCTCTGGCGGCACCGCCGATGAATCCGAATGAGCATCTCTTTCAGATCAACTACATCCCGGATTCCCCAGTATTTTCC
GTCCTGCCGCGTACCGCCTGGGCATCAGGCAACGTGACTGTACTTCTGAAAGGGCTGGAAGTGCCGGTCATCATCAGTCTGACCAGCGGCGATCCCGACAGCAATATATCGTCACGTGAAA
TCGATGCCCGTCTTGATTTGCGCATCCCCCGGCAGGGACCGCAGGCCGCTGCCGGCCCCACACCACCGGCACGGGTGGCGCTTCATGATGAAACGCTGCAGGCCTTTCTGGACGGGACAC
CGCCCGATGGTGCCCGTCGCCTGAAGGTCAGGGGTGGGCCGGATAATCTGCGGGTGTGGCGTATCGGCGATGACCTGTATGTCCGGTGCCGTGCAATGCTGCGCGATGAGTTCGAACAGA
CGCTGTCATCTGCCGACGGAACCCGCCTGTGGAAGCTGCCCGTCACCCCGTTGTTAACGTTTTCCGTTGACGGTCGCACCGTCACAGCCTCACCGGAACTGGAGTAA
>PHINNC_00350 Conjugal transfer protein
ATGCAGAAAACAAAAAAACCGGCCTCGGCCGGAAAAACAAAACAATCGTCACGGGTGAAAACTCCTTACGCCGATGCCGTGGCCCTGATGAAAAAAGCAGCCGACGTGCTGACCATCGG
ACGCGGCATGATCCACATCACGCTTGTGCTGGCGTATGCCCTTGCCGTGGTCGTCACGCTGGCAGGCTGGCTGGCCTGGAAGGTCGCCCACCCCCCGGTGCACTACTTCTCAACAGAGAAC
GGCGTTATCACCCGTATTGTGCCGACAGATGAACCCGGATGGTCCCAGAATGACGCCATTGATTTCGGTGCCCGCGCCCTGCGCGAATCCTTCTCTCTGGATTTCGTGCACTTCCGGGAGCA
GACGGCGGCGGTCCGTCCGTACTACTCCGCCGAAGGCTATACCGGCTACGTCAGGGGACTGGAAAGTTCCAACATCCTCTCCTCCATACGCGATGACAGGATGAACCTGACCAACAGTATC
GGTGCCGGCGTGGTGTCAAAAACCGGCCGCCTGAGCGACGGCACCTGGGTCTGGGTGATCCAGTACCGGGTCAGCCTGCAGCTTGTTGGTCAGAAAACCAAACGTCCCGAACAGAAGC
AACTATTCGAAGTCACCATCCAGCGCACGGACCCCCGTGTGAAATACGCCGGGATGGAAATCCGCCAGATCATCTCCCGCGACGCACCGCGCGATTAA
>PHINNC_00355 TraL protein
ATGACACAGAGCTCCGTCAGACGGGTGGCTGCAGCCACCTGCCTGCTTCTTTTGCCTTTATTCGCCACCCCGGCCTCCGCCGCTTCCACATGTCGGGCATACACAGCAGCACAGGAAGGGG
CCTCCCGGGGCTATTCGCTGGCCAAATCAGCGGCGCAGTCCTGGTCTGACTATGAGTCGGATTATTCCGGTGCCCTGGAAGACTGCCTGTCTCAAATCCGGGATTTGTCCCTGACGGTCAAT
CTCAGTGCCAACATCGATGCCCTGCTGCAGTCACTGGAGAAGAAAGTCTGCAGTGTCGTGGTCTCCTCAGCCAACAGTTACATCCCCCCCAGCGTGACGCTGGACCCCTGGAGCGAGGTTA
CCAGTTCTGTTAATTCACTGTTCTGA
>PHINNC_00360 DNA primase TraC
ATGCCGTCTGTATCCCGATACCGCACCTGGCTTGCCGTGCCTGCAGATGAAATAGAAGATCTCAAACAAGCGCATCCCCCGATGAACGGGCACACACCCGTCCTCTGGGATAAAGAGCACAA
ACTCTGGTTTGCCCGCCCGGGGGCGGACCTGTCCATGCTGGACCGCTGGCTGCCCCGACCGCAGGAAGTCTCCATGAACGGCAGTGATCCGGTCACCGAGTTTGCCCAGGTACTGGAAAA
TGCCGGACTGGTTCTGAAAGAACTGCCGGCCATGGACGGTAAAATCCACCGGGTTCCGACCACCGGTGACAAAAACGGGCAAAAAAGTGGCGCATATCGCGGCTTCCTGGACGGCAGAC
CTGCCGGCTGGTACAGGGATTATCGCAGCGCGGATGATTCGCCGGTCAACTGGACCTTCTCCGGCGGAGAGCAGACAGATCCCCGCGCAAGGCTTCACCTGAAAGCGCATTCGCTGCAAC
GCAGGGAGGACGCAGAGCGGGAACTGAAAGCACAGTACAACAGACAGGCCGCGTATGCCCGCCGCTATGTCAACAAATGGCCGCAGGCCACGGCACATGAATACCTGACCCGGAAAGG
CATTCAGGCTGCGCCGGGGGTGCGAATAAACGACAAAAATGAGCTCGTTATTCCTTTCCGCAACCGTAACGGCGCAATACGCTCCTACCAGCGCATTCCGGTCACCGGCGGGAAGGATGCC
CGCATCCTGAAAGATTCCGAGAAAACCGGTAACTGGTTTGCGCTGGGTACACCGCGCAACGGCCAGCCGGTGCTGTTTGCCGAGGGCTATGCCACTGCCGCCTCCCTGCACGAAGCCACC
GGCCTTCCGGTGCTGATGACAGTGGATGCGGGCAACATGATTGCCGTGGCCGAAGAGGCCCGGCAAAAATGGAAGCAAAGCCCCTTTATCTTCTGTGCCGACAACGATCATGCACGCCAG
GTTAACAAAGGTATCGTCAGTGCAACAAAAGCCGCAGAACTGACAGGAGGATCGGTCATCTTTCCTGCGTTCACCGATGCCGAAAAAGCGCAGGGACTGACCGATTTCAATGACCTGGAC
GCCAGCCGGGGACGGGCCGCTTTTCAGCATGTCATCAACGCCCAGTTAGAACATATCGGGGTTTCCACCCCCAACAGTAACACTCCGGAAATCCGGGAGGCACTGGTGATCGGCAATCTCG
TTTTTACACCGGTCCACACTGAGGAAAAAAACATGACTCCGACAGAACATCCTGAAACATCACCCGATACAGCACACAGCAGTGAACATGAACCGGCACCTTCTCTGCCTCCTGCACCGCCT
GAAGTGCAGCCGTCACTCTCCGGTAACGTTCATCATGAGCCGGTGACGACCGGGGAGGAGATGAAGCAACCGGAAAGCATCGCTGACCAGGACTCTCCGGAGGCCCCCACGGACAACAC
ACAGACGTCTGACGTTGTACCTGACACCTCCGGTACAACCGCTGAGCTCCGGGAAGGACATGAGGCTGTCACGATGGCGACAGCGCAGGACTGGCAACACTTTGAGGAGGAGATGAAAC
AGCCCGAAAATATCGCTGGGCACGACGCGGATGAGGCATCCGCAGAGCACACTGCGTTCGTATCGTCGGCAGATGCCCCGGAACCTGAGCCCTCTGCCACAGAGACAGCATCCACTGAAG
CGGCTGTCAGCAATGACCAGGCAGAGACAACGCTCACACAACAGGAAAAACCTGACACGGCAGAAACCTCCGCAGAACATACGGATGCCACTGCAGCAGCGCCGGCAGAACAGACACC
GTCTCCGTCAGCGAGTGAGCCAGCCCTCCCTGAAGATACTGCCGGCGATGAGCAGCTGGCAGCAGAGACATCCCTGCAGCCGGATGTACCGGGTGCAGGTGATGACGGATACCCTGGCTA
TGACGTCGAATACCAGGACTACAGCGCGGAATATGAGGACTATGATGCCAGTGCCCTGCAGGAGTACGCAGCCATGCAGCAGGCAGCCACGATGCAGCATGCAGAGGCTGAAGCAGGAA
CGGAGACAGAACAGACAACGGACGTGCCCCTTCAGCAGCCCCAACAACCCCGTACAGAACCGGCACAACCGCAGGCCACCTCAGCCGCCCCCGGAGGCACTGCATCCCCGGCATCAGAA
GCGTCAGACAACAGCGAGCCCGACGCCATCACCTATGGCCCTGAACGAAAAAATCATACCCGCGTCCGCACGGATATCGACAAACTGGTCCGCAGTCTCCAGACCAGAGAGCAGTCTGAC
CATACACTGCAGTATCACCTTCCGGATGGCACGCACGCCTTCACAGACGTCGGAGGGCATCTGCTCATGGCTAAAGGGCAGAGCACGCAGGAGCAGTCCATCCTGGCCGCTCTCGGGGTG
GCCATGCAGTATTACGGCGGGGTGATTGAGCTCACCGGCAGCGATGAGTTCAAAAAACGGACAATGGAAATCATCGTCGACTATGACCTGAAGGTCACGATGAAAGATCCCGTGCAGCGA
AATGCGCTGGAGCAGATGCGGGCAGAGCGGGAGAATCCTGACACCATCATGACGCACATGCCCACACCGGAACTGAACAGAACAACCGGACAGGCCATGACGCAGCCGGAAGCACCGG
TACCGTCACAACCCGCTGCTGTACCGCCCCAACAGCCCGCTGAAGCTGCGGCATCGCCGACACAGGATGAAACGCCACAGCCGCCGGCAGAGGCATCCGCACCACCGGTCACACCCGTAC
CGCCGGCAACAGAAACGCAGAACGCTCCGGCACCCACAACCAGCGATAATGCGCGTATGGTTCCGGGCAAACCTGTCACTGGCCGTCTGGAGGCATCCGGACGGGGCACCGATCCGGAA
AGCAACCAGATGACTGACTGGATACAGCTGAAGACACGCTCACAGACCGTCACTTTCTGGGGGGATGCCTTCGGCAAACTGACCCGGGATTACAAAGCCGGGCAGATGGTCACTGTGACA

ATGGCCTCACGCAACGAAGCACAGTGCGAATGGACTATGCAGTCTGCAGGAAAAGCAACAGCAAGGATCTCTGAAGAGCCGCAACCCGGCGAACAGATGAAACTCTTCGATGCCGGGAC
ATTCCGGGCAGCAATGGAGCAGATTTGCCGGATACTGCCACAGTGGCAGGATGAGCTGAAACAACTTCCTCTGCCGTCACAGGATATCGCCCTGCTGGACAACGGGCGTCCGGGCACCCC
GACTGCCTCATCCCGTTGCCTGCCCGCACCGCTGCCGCGCAACAGTCTCACAATGATCGCCGGTGGCAGTAATGACAACGGCCTGCAGGCAGCCCTGTTCCGGACAGCCGGGAATTACTAT
CAGGGCGTGATGCGGCTGGGCGGGCACGGGCTTTTCCCCGTTCTTGTCACACCGGTCAACCAGAAACTGGTCGTTACGGCCATCACGAAAGACGGCCCACGCTATGCCGGATACGGTAAT
GCCCGGACGTCCTCTCAGGACGGCACCCCCGTACCTCCGGACACGCTCAGCTTCACACTGGCCGGTAATAAAACCACACTGCATGTGCCGCTGACGGCCCCCCGTAAGCTGTCTGCCGACA
ACTTCCGGCTGCTCGGTTTCCCGCAAACCATGCAGCAGTGGGACCAGCAGCAACAGGCAAAAAAAACACAGCAGGCGGAGATGGCAGCACAGCACAACCCCGCGCCGGGACGGTGA
>PHINNC_00365 hypothetical protein
ATGTATTACGCATGCCGCTGGCGGGATGCATCGCCGCCCGTCGCTGTTTTTGGCATTCCGTTGCCGTTCACCCTGATTTACCTGTTCTGGTGCCCGTTTCCGTCCGGCAACACGATTTTCATCT
GCACGCTTGTCCTGGGCGCATTCGTTCTGCTGGGCCATTTTGGCTGGCGACTTCAGGTGCTGCTCCAGCGACTGGTTTATCTCTGGCACGGAAGCCGGGCTGCCGGTCGCCCGTGGTGGTA
TCGCCACTTCACGGAAGCGCCCCGTCGCTGGTCCGGTCTGTGA
>PHINNC_00370 Plasmid transfer ATPase TraJ
ATGTCCCATGAATTCACACCGTATGACTTTCACGGCACACCGTCTGCGGAAGACTTCAAAGCCTTCATGGCATGGTGTGCCACACAACAGGTATCCGATATCAACATGCAGGGCGGCAGTCC
GCTGTCCGTGGCCCGCTTCGGTCGCCGTTGCCGGGCAAGTCACATGGTGCTGGCCTGCGATACGGTCAGCCGGCTCACGGATGAAGTCTTTGGGCTGGATATCCGCCCGGTCATTACCAGC
GGGCAGCCGGTGGACCGGGCCTTCCAGCTTGACGGCGACATGAACGGCCGTTACGGCCTGCAGCGCGGGGAGCGGGTCCGCTTCCGCTGCAATTTTATCCTCGGCACTGCCGGACGCTT
TGAAGAGTCCATCTCCCTGACCATGCGGGTCATCCCCTCGGCTATTCCGTCGCTGGATTCGCTGAATATTGAACCGGAACTGAAAGCAGCGCTGCTGCCACAACAGGGGCTGGGACTGGTC
TGCGGGGCCACCGGCTCCGGCAAGTCCACGCTGCTGGCAGCAATATACCGTTACTGTCAGGACAATTTTCCTGACCGCGTCATCGTGACTTACGAAGATCCGGTTGAATATATTCTCGGGCG
GGACACTGACCTGCTCAAACCTCACCAGTCACAGATTGGCCGGCACGTGCCCGGCTTTGCTGAAGGTATTCGTGCTGGCATGCGACGAAACCCGGACCTGATTGGGGTGGGTGAAATCCG
CGATCAGGAGACGGCAGAGGCGGCCATTGCCGCCGGAGAATCCGGTCACGTCTGCCTTGCCACCTGCCACACCCACTCACCGGGTGAAACCATTTCAAGGATGCTGGGTCTGCTGCCGGT
GCAGTCCCGTGATGCCATGGCGCATGCCCTGCTGGGCACCCTGTCATTCATTGTTGTGCAAGTGCTGGTCAGAACCCGGGATGGACGCCGACGTGCTGTCCGTGAGTTCATCACTTTTGACA
CGGCACTGCGAAGTGCACTGGGTCAGCTTCCTCATGAAAAGTGGGGAGAACATATCGACAACATCATCCTGCTCGAAAAACGCAGAATTGCCGATCAGGTTGCCACCCTGTACCGCCGTCA
TGACATCACCCGTGAGGAGGCCTGTCTGTTCATCCCCCATCACGAGTGGTCACGACTGCCCGACGCGTGA
>PHINNC_00375 Conjugal transfer protein
ATGTTTCGTCATCTCATTCTGGCCACCACCATTGCCGTCGCACTGTCCGGCTGTACGCAGATGGCAGCCGGCAGCCGCCCGCACACAGACACCGGCACGCCGGATATCAGTACCTTCCTGCA
TCCGGAAAATGCCCGCCCCGACGGCATGTCGGATATCCGCTATCAGTTGCTGAACGAGACAGGAAAAACACTGGGATTCCGGGGAGGACGTGCAGAGCGCGCCGCCGAGCTGCGCAGGA
CACTTGAACACCGCGCCACAACAACTTGATGCCCTGTACGACTTCCGGCCGCTGCTCAGCCCGGAAGGCTGGCTGCCACCGGTGATTGACGAAGCCCGGGACGTGGCACATGTCACGGGCG
AACAGTTCAGAACGGCAGACACCGTGTGGACCATCGTCTCTCCTGAACGCTTCGTCAGCAATCCACCCGGCTGGCGAACCTGGCTGTTCACCGGTCTGTCGGACAAAGTCACGCCGCCTG
ACGGCAGCGTTATCCCGGAGGACCGCAGTCAGCGAAAAGTCTGGGAGCAGGCGGTCAGGACAGGCTGGCAGGAAGGACGTGATGCAGCAGACCAGATACTTGAAGCCAACTTTAACCG
TCTTAACCGTGACTATCGCGGCATGATGCTGTACGCCATGCTGCGCTCACGCGGAATGATTTCAGCACCTGCCGTCACCGACACCGTTCAGTCCGTCACCGGCTCCGGCACAAAACTGGTCA
CCGGCGACCGCGTCCGCAGGCTGAAACAGCACGCCGGCTTCGAACTGAACAAAACCCACTGGCGTCCCGTCATCAGCACGGAGAAACCGTAA
>PHINNC_00380 Cellulose synthase regulatory subunit
ATGAACCGTATGATACCTGCGGGGTGTCTTGCCGTACTCCTGCTGGGCGGCTGCCACGCAACACCACACAAACCCGATTACGGCGCTGCATCCGATGTGGCCAGAACCCGCCACGCGCAGT
ACCAGCTGTGGGAGCACGGTGCACTGTCAGGACAGACGCACGCTGTAACCTCCCAGCCCGCGCTGACCGCAAACAGCGGACGACTTTCCTTTGACTGGGACGGCGATGCCATTGAACTG
CTGGACAGTCTGGCCCGTGCCCGGGGTCTGCAGTTTGGTTACAACGGCGTCCGCCTCCCCCTGCCACTGACGCTGCATGTCCGGGATATGACCTTTGAAAATACGTTAAGGCTGATACAGG
CACAGACCGCCTGGCGCGCCACACTCACTCAGTATCCCGGCGTACTCTGGCTGGACTTCATGCCGGTCCGGAAAAACCACAAGGAGTGA
>PHINNC_00385 Urease accessory protein UreE
ATGTTTGACAAAGAAACGTACATCGAAAACAAAGCCTTTACGGTGCAACCGTTTGATCCCGCAAACCTGCGATTGATCACCGACAGGAAAAATGAATCTTTCATTTTCCAGACAGAATGCAT
AGTCGCTGTGGAACCGCGCCAGTCGTTCCGGGACCCGATGGTTACCGAATCTGACGTCATCCTGGCGAACGACGTTCGTATCCGGGTATCGCTATCACCTTCTGACGTGTTCCAGCAGCTCA
GCAAAAACACAGGGCTGTAA
>PHINNC_00390 Conjugal transfer protein TraF
ATGATGAAGAAAAATCATATTACCCGCACCATCATTGCATCTGCTGTATTATTTTCATTTAACGCTGCAGCAGCGACGTCATATTTTGAAGCGCGTAACGATGCAATGGGTGGCACCGGGGTG
GCATCCTCCCATTATGGTGTGGCTCCTCTGGCAAACCCGGCTTTACTGACAAAGCACAACAGCAATGATGATTTCAGCCTGTTACTGCCGTCCGTGGGCGCACAATTGTCAGACCCGAACGA
CATCGCTAACAAAGCCGATGACATTCAGGATGACTGGGATGCCTTCGACCGCGCGGTCGACAGCAATTACGGTGTTGACCAGGCAGCGGCAAACCTGAAACAGCGCCTGCAGGAGTTCCG
CAATATCCACGCTGATGCGCAGGTGGGTGTTTCTGCCGTTGCGGCACTGCCGAATGACACGCTGCCGGTTGCCCTGATGGTGAAATCCTACGGTACGGTCAGCGTTGACGGCAAAGTCAGC
GACGCCGACCTGGAATATCTGGAGAAAGTCGCCAACGGCACAATCACGGATGTGGACAAAAACGCGCTCACCTCCCGCGCGTATGGTCGTGCCGCAGTGGTCACTGATGTGGGTATTTCCT
TTGCAAAAGAACTGGAAACCGCCGGTCAGAAATGGTCACTGGGCTTCACACCCAAATACCAGCGCGTGGATCTGTTCAACTACAACGTCTCCGTGCGTGACTACGACAACAGCGATTTCAG
CGACGACAAATACCACAACACGAAAAACGGCGTGAACGCCGACATCGGGGCATATACCGACCTCGATGACAACTGGACGCTGGGTCTGGTAGCGCAGAACCTCATTCCACGCAGCATCGA
TACTAAAGTGGTCGACGGTGTTAAGGAAACCTACAAAATCCGTCCGCAGGCCACTGCCGGTGTGTCCTGGCACAACGACCTGTTCACCACCGCACTTGATGTTGACCTGACACCGGCCAGC
GGATTCACCTCCGACAGCAAACGTCAGTTCGCCAGCATCGGCGCAGAATTCAACGCATGGAAATGGGCACAACTGCGTGCCGGTTATCGCCAGAACATGGCATCAAACTCCGGAAGTGCC
TTCACCGCAGGCTTTGGCATCTCTCCATTCGACGTCGTACATATCGATGTATCCGGCCTGGTCGGGACCGACCATGATTACGGTGCAATAGCACAACTCCAGTTCACTTTCTGA
>PHINNC_00395 Conjugal transfer protein TraE
ATGAGACTGAATACGACCGGTATTGCGGCCAGAATGATGCTCTCGCTGGACAAAAAAGCAATCGGCGAAAAACTCATCAACGGTCGTCAGATAAACCCGACACCGCTGCAGGTCCGTTACC
CACAAGGGGTGAGAGACGTGCTGGGGATAATGAGCGAGCAATTAGCCATATCCACCGCTGATTTGACCCGAATCCTGCTTGAGGATGCTCTGCATAATATGTTCATGCCTGCGGATAACACC
ACGGGAAACATTATAAGCAGAATTGAATGCATCATGCTGTCGCATGATATAAATGCACCATTGCTGGCAACATTGTTGTCGCCATGGAATATCCGTTCGACAGTTATACAGGACCCGGCAAGA
CTTGCTGACTATCTCTCAACTGACGCGCTCGAACATTTGGCTGAATATTTTAATCTAAATCCAGACTGGGTGAATGGCCGCGAAAATTATCCGATAGCATTGTCCTGTGAATGGCCAGATACTG
CTGAAAACTTCAAAATGCTTGTTAATGATGACGGCAACACTGACATTATTTTCTGGCATAGCTTCCCCTACGCAGGAAACGCTAAAAGGGAATATTGCGGAGTAATATTGCGACAGAAAAAA
CTTATAAACGGTTCCGTAATATATCCCGCTCTGTCATTAGTACCCACACTAATGAACGATGAGAAAAGAAAATGGTTAACTGAATATACAACCAGGCAGAATGCAACAATGTCTTTGCGCCGCG
TCACATTGCGACCTGGCCTTGCCGATAACCTTATTACAGGACAAATACTACCTGTTAGTTTATTTAATACGTCTTTATTACCCTGGTAA
>PHINNC_00400 Tyrosine recombinase XerC
ATGTATAAGAAGTCTCTACCTTCGTCGCTTTTTGAGGTTCTTATGCCGTCGCCACGATTCCGCATCCGCAAAATGACACTCGCCCGCGCCCTCGACAAATACCTGAAAACCGTCTCCGTACACA
AAAAAGGACACCTTCAGGAGTTTTATCGCGCGAATGTCATCCGACGCTACCCCATCGCACAGCGGTTCATGGACGAAATCACCACCGTGGACATCGCCGCATATCGCGATATGCGACTCGCT
GAAATCAACCCGCGGACAGGAAAAACCATCACAGGAAATACCGTCCGTCTCGAGCTTGCCCTGCTCTCGTCGATGTACAACATCGCCCGGGTCGAATGGGGAACATGCCGCGATAACCCCG
TTGAACTGGTGCGTAAGCCCCGTGTCTCTCCGGGGCGCGAACGACGTCTGACGTCCTCGGAAGAACGCAGACTTTCCCGCTATTTCAGGGAACGGAATATCTCACTTTATGTTGCTTTCCAC
CTGGCGCTGGAAACAGCCATGCGACAAGGGGAGATTCTGTCACTACGTTGGGAACACATTGACCTGCGCCACGGCGTGGCTCACCTGCCTGAAACAAAAAACGGTCATTCCCGTGACGTG
CCATTATCTCGCCGGGCACGCAATTTCCTCCAGATGCTGCCCGTAGCGCTGCACGGAGAGGTCTTTCCCTATACTTCTTCCGGCTTCAAAAGCGCCTGGCGCATCGCCACACAAGCTCTGCAC
ATTGAAGACCTGCATTTTCACGATCTACGGCATGAAGCAATCAGCCGCTTCTTCGAACTGGGTAGTCTGAATGTAATGGAGATTGCTGCCATATCAGGACACCGTTCCATGAACATGCTGAAA
CGGTATACTCATCTTCGGGCATGGCAACTGGTCGGTAAACTTGATGCCCGCCGACGGCAGACACAAAAAGTGGCAGCATGGTTTGTGCCATATCCTGCACATATCACAACTATTGATGAAGA
AAATGGGCAGAAAATACATCGTATTGAAATCGGTGATTTTGATAACCTTCATGTCACAGCAACAACGAAAGAGGAAGCCGTCCACCGCGCCAGTGAGGTTTTATTACGTACACTGGCTATTG
CGGCACAGAAAGGCGAACGCGTCCCATCTCCCGGAGCTTTGCCTGTAAACGACCCTGACTACATAATGATTTGCCCACTGAACCCGGGCAACACTCCGCTGTAA
>PHINNC_00405 Shufflon protein D

GTGCTGACCGGCAGAATTGCTAATGGACAACAAATACCCCTACCGTCAGGATTTTCTGCCAACCAGTGTACCTGGAGTGTCAGCAATGCTGAAAACCCGCATGGCTGGAAGCCAAATTACTT
CGCTGGTTCGGTTGCGACGTATGATTCGAACAGGATTGTGAAGTGTGGTTACTACGATGAGTACAATTTCCACGGCGGCACGAACAGGACCGATCTGACGGGAAAATGCAGTTATGTGGTG
TCATGCCAGAGTTGA
>PHINNC_00410 Shufflon protein C
GTGAACTACAATGCTTGCAAATGGTACGAGTCCTCAGTGGCATTTAATCATTTCATCGGCGGTGATAGCGGTGGGTCGATCTACTACAAACCGGTTTCCTGTCCTACAGGATATGTGATGACG
GGCACCCGGATGTATGGCATAGGTGATGGAGTCGATGAGGAGCATGTTGATGCTTACTGTTGCCCGTTTAATTGA
>PHINNC_00415 Shufflon protein C
GTGGTTGGGTACAAGGTGAAGGATGTTACTGATGTCCACGGCGCAGCAGAACCGATTGATTTCCACGAACCGGATTGGCACGGAGGCCAGTGCCAATCCGGTGCCTGGAAATATACAACG
CTGAACTTCACCACAAGCACGTACAACATCGGCAAAAACACATACAATAAGAGTATTGGCGTGCACGCTTATTGCGCGTGGACGTACCTGAATGGTTCCCCCTTTGGCGGTTTTCAACAAGT
GTATTCCGACAGCAACAATAACTGGTATGTGAACAATTACGCTTGGGATAACTACGCCTCCGGTGGCACGATCTCTGTGACGTGCCTTAATCTCCCCAATGCCGGCGTCTGA
>PHINNC_00420 hypothetical protein
ATGACAAGAGTCTGGCCGACAGATAATCCAGATGTAAACGGCGAGATGAATTGGAGTGCGTACAATGGCACATTGTATAGCAGCTATTACATCACTGTTCACTGTTTCCGTTGA
>PHINNC_00425 hypothetical protein
ATGGCACATTGTATAGCAGCTATTACATCACTGTTCACTGTTTCCGTTGAAAGCTCTGTCGCACATTATCTGGCACATACAGCTACTGAGCGGAAACAGCTCGTGATAGTTCCGGTACCTCCGA
CAACCCAACGTTTATTATTCCAGTCTGGGTAA
>PHINNC_00430 Shufflon protein C
GTGAACTACAATGCTTGCAAATGGTACGAGTCCTCAGTGGCATTTAATCATTTCATCGGCGGTGATAGCGGTGGGTCGATCTACTACAAACCGGTTTCCTGTCCTACAGGATATGTGATGACG
GGCACCCGGATGTATGGCATAGGTGATGGAGTCGATGAGGAGCATGTTGATGCTTACTGTTGCCCGTTTAATTGA
>PHINNC_00435 hypothetical protein
ATGCACCGCACTCTTCCTAGAAAACGCCCCGCCCGGGGGATTGATCTTATCGGCACCTTTGCATCCGTGGCTGTTGGTTTTTTGATTGTCGGTCTGGCACTGACCATGCTCTACCGGAGCAGC
GAGGACAGCAATAACAACGTTGTGGCCCGTCAGGCAACCAGCTGGGCCACAGCGGCCAAACTGTATATTGCCGCGAACTACGACACCCTGCTGGCCTCAGCCCCCACCACAATCAGCCGC
AGCACGCTGGTCAACAGCGGATACCTGAACAGTGGTGAACCGGAGACCAATCTGTTCCAGCAGACCTGGAAAGTCGCTGTCAGGAAAAACACCAAAAACAGCGATCAACTGGATGCCGT
TCTGCTGTCCAGTGGCGGGCAGGCAATGGATTACCCGACGCTGCGCTACATTGCGGCGGCCATTGACGGTTACGGCGGTTTTGTCTACAGCGGCAGCATGCCGTCAGGCTGTACCAGCAG
CACCACCACGGTGAACGGTGCGGCCTGTGGCTGGACGCTGGATCTGTCCGGATTCGGTATCAGCACAACCAGCGGTCACATTGCCGTCAATCTGGGGGCAGACATTGCCGGCAGCGCCAG
CAGCGGCGGCGACTTTCTGCACCGCTATTCCGTGCCGGGTCATGCCGAATACAACCAGATGAACACGGCGCTGGACATGAACGGCAAAAACGTAAACCGTGCTGGCAACCTCCTGGTGAC
ATCTGACGGAAAATCATCAGATGATCATGGCAGCAGCGGCTCAACCGGTTTTGTGGGATGGGCATCAGGCAGTGCAAGCGGAACCGCCAGTGATGCAACATCACTGAGAGCAGGCATGTA
CTTTTCGACCTCTGCGCCATCCAGCAGTGACAAAGAGAGCGTGTGGATCAAAACGACCAGCAATACCAGTCTGGATGTGACAGGCAAGGTTCGCGCAGCGACGGTGAAAGGAACCCGGC
TCATCACATCCGAGGGCTATCTGATGCCAGGAAAAACGGTGACGGCAAACACATCCTGTACGCCAGCCGCACTGGGTATTTCGGACCTTCTGGCAGACACAACAGGCCTTGTAGCCCATGA
CAGCACTGGTGCCACACTTTCGTGCCAATCCGGTGTGTGGAAAAAAAAATAGCTCCGGGAGCACTGTGCTGACCGGCAGAATTGCTAATGGACAACAAATACCCCTACCGTCAGGATTTTCT
GCCAACCAGTGTACCTGGAGTGTCAGCAATGCTGAAAACCCGCATGGCTGGAAGCCAAATTACTTCGCTGGTTCGGTTGCGACGTATGATTCGAACAGGATTGTGAAGTGTGGTTACTACG
ATGAGTACAATTTCCACGGCGGCACGAACAGGACCGATCTGACGGGAAAATGCAGTTATGTGGTGTCATGCCAGAGTTGA
>PHINNC_00440 Peptidase
ATGGCTCTGACATTCTTCTTTGTACCGGCTGGTACGCTTGCTGCCTTCGCCCTGATCCGCTACTACGACGATCATTCCGGACAGCTGAACCGTCGGGATATCTGCACCGGCCTGCTCTGGTGT
GCTCTGTGGCTGCTACTGAGTGTTTATTCACGCAAGGAGCTGTCCCTGCCACTGTCGATAAGCATGGGATTGCTGTGCGCATGCACCGTAACTGACGCCCTGCGCACCTGGCTGCCGGCCGA
ACTGACGCTGCCTCTTGCCGTGACGATGGTCTGGCATGCCAGTCTCTCCCCGTGGGGGTTTCAGAGTGCCCTCATCTGGGGGGCGGTCTGGACTGCATTTTATGGTGGTCGCTGGCTGTTTT
ACCGGCTGCAACATCGGGAGGACTCTCCAGGCAGCGGCGACATTATTCTTGCTGGCATCACCGGCATCGCCGGCGGCCCGTCATCCGCGTTTCTGATTGCATCGGCCATCACAGGGCATCT
GGCCTGGGGAACCGTGCGCCATCTGCACGAAGCCCCGTTTGGCCCCTGGCTCTGTCTGGCTATCACTGTTCAACTGCTCTTCTTCTGA
>PHINNC_00445 Pilus assembly protein
ATGACGACACGCATCCTGTCGTGCCTGCTCCTTTTTATCTGTTTTCTTTTTCTTCCGGCACACACGCAGGCTGCCGTGCAGCCATCAGTGAGAATGTGTTTTTCCACTGCCGGGAAAATGTAC
CAGATAGACCCACTGTTACTGGAAGCCATCGCCCGGCATGAAAGCGGTCTGAATCCCCGGGCAATGAATCTCCGCAACCGTAACGGTACGGTGGACTATGGACTGATGCAGGTGAACTCAT
CGCATATCCCGCTCCTGAAACAGAAAGGGATTATCCGAAACCCGCAGGACCTGCTCGATCCCTGTCTGAATATTCAGATAGGTGCCTGGGTACTGGCACGCCACTTTCAGAGCTGTGGTGTG
AACTGGAACTGTCTTGGCAGCTACAACGCTGGATTCCGCAGCACAATGAACCAGCGACGGGAGGACTACGCAGACAATATTTATGCCATCTACCGGAAACTGAACCGGGAGCGCCGCAAC
ATGGCATTACGTTAA
>PHINNC_00450 Pilus assembly protein
ATGAAAACGAAACCGAATGTATTACCAGCGCATAATGACTGCCCGCAGACCACGACAACTGGCAACTCACACCGTCGCAAGGCACATCGTGGGATCGAGATGCTGTCCGCAATGGGGTGGT
GGAGTTATGCGATCATCATCGTCGTGTGCGTACTCGGAATGCTGTTTGGTCTGTACATCTTCGGCCGCTCCGGCAGTGAAGCATCCAACCTGACCCAGCTGGCCACTGCCACGCAGCGTGCC
GCCCGGACACAGGCGGGGTTCGGCACAGCCAACCTGAACACCGTGCTCTACAACGCCGGACAAATCCCGAATGGCTGGACCTACAACAGCGGAACCATCTACAACCAGTGGAACGGCAC
CGTCACCGTGACCGGGAAAACCTCCTATTTCACCATCACGGATACAGGTATTCCGAGCTCTGAGTGTATTTCTATCGCTCAGACACTGGCGGCCGGTGGTGTGGCGAACTACCTGACAGTCG
GTGGTACGAAAATCACCCCGGCCAGCGATATTGCCACCATCAACACCGCCTGCGGTAACGCCAACAGCAGCGTCAACAGCACGACAAAAACCATCGTCATGTATGTTGGCGCTCTCCAGTAA
>PHINNC_00455 T2SSF domain-containing protein
ATGGCCAGTGAAATGCAACGCAGAATGGCCCGTCTGGCACTGTCCGGCAGCAATCGTATCCGCTTCTATGAAGTGCTGAAAATGCAAATCATGAGCGGACGGACACTTGAGCAGGCCATGC
TCAACATCCGCGAGGTGTACACACGGGGAGGGCAACGCTTTCACCCCTTTGGCGTATTGGCACAGGACTGTCTGGACTCCCTGTCGGTGAACCGGGAAGGAAACCTGCTCCCGGACGTTT
TCCGGCGCTGGTTTTCCGGTGAGGAGGCTGCATCGCTTGCCGTGGGCATGCGTGCCGGCTCCCTGCTGACGGCGCTGGACAGTCTCATCCGGCTGATCAAGTCCCTGCAGTCCATCCGTTC
AATCATTACCGGGGCTGCCCTTGCCCCGTTCGGTGCCATTCTTGCCCTGAGCGGGATACTGACCCTGCTGTCCGTTCAGCTGGTTCCGCAGTTCGTCAAAATCATCCCGCCGGAGAAAGCCC
GTGGTGCCCTTGCCATCACTTACGGTCTGGCGGACTTCGTCACCACAGCAGGTCCCTGGGTGCTGGGTATGATCATTGTGTTTTTTTTGCTGGCGCTCTGGTCCCTGCCCCGCTGGACCGGC
CGGATACGGCAGTTTTTCGATCATATCCTTCCCCCCTGGACTATGTACAAGCTGATGCAGAGCAGTGTTTTCCTGCTCAACCTCTCCACGCTGATTGGTGCCCGCATGACCTTTCAGGATGCCC
TGAAAACGCTCAGCGACGGCGGTGTGCCGTGGCTGCAGGAGCGTCTGATACCGGCACAGCATCATTTCAGCAACGGTGCTGATCTGGGGACTGCCCTTGAACGCGCCGGGCACAACTTCC
CCTCACAGGAAATGATCCTTCACCTGAAGGCACTTTCTGCCGGGGAAGGCGCGGAGCTGGCCATCGCCAACCTGGCCGACTGGTGGCTAGGAAGGACCCGCGCAATTGCCAAAGGCATG
CTCTACCTGAGCCTGCTGATTTCGGTCGTCATGGTCTTCGGCTGTCTGGGCATTATGGCCAGCAGCATGATGGCCCTGCGCGACCTTGTGAACATGTAA
>PHINNC_00460 Type II secretion system protein E
ATGGTCAGTAACACGGATATACCGGACGGACTCCGGCATCTGGTACAGGTGCTGCCGGCCGCAGCGGGGAAGCCCGGCCACCGAGTCTGTTTTCCTGTCACGGAAACCAGCAATCCGGAG
ATACAGACATTCATCACCGGCCTGTTGCGCACCACCGGTCAGCCCGTCATCCCTGAGCCGGTGTCACCGGAGGAGTTCCGCAACCTGCAGAATGATGCGGGGGATGCCTTCCGCCACGACG
GTAGCGAGCACAGTCGTTCAGAGGTGCAAACCCGTGTGCTGAACATGTTCCGTGAAGCCCGCCGGGTGGGGGCATCGGATATTCACATCACCGTTTTCGTCGCACAGACACGCATTGAGAT
GCGTATCAACGGTCTCCTCTACCCGGTTGCCACGCTCTCCAGAGACGAAGGCATCAGCTGGCTGATGACCATCTATCAGTCCATGTCTGACCTGTCCGACAACATGTTCAAGGCCTACAAACC
ACAGGATGCGCGTCTGAAACAAGAGTTCCTGCAGGATGTGGAGCTGTTCGGTGCCCGTTATGCGCACATCAACACGCCGAAGGGCGTCTTTGCCGTAATGCGTCTTATCCCCGACGACGGC
AGTGCCCCACCCTCGCTGGAGACGCTCGGCTTTCTGCCGGCACAGATTGCGGTCATCCACGCCATGCTCGCCAGTCCGGATGCCATCATCATCAACTCCGGCCCCACCGGTTCCGGTAAATC
CACGACCGGGCGCAGCTTCATGGCGCTCTGGCTACAGCACGGCAACGGACAGCGACGCATCATCACCCAGGAGGACCCGGTGGAGGGGGGAAGTCGAAGGGGCCATCCACACTTCGGTG
ACCGCCGACCGCTCCAGCGAGGAGGACATCAACCTTGCCTGGCTGCGTGGTCGTCGTGCCATGCTCCGTCTGGACCCGGATGCGGTCTATATCGGTGAAATCCGCGACCATGACTCCGCCA
TGGCCGCCATCAACTTTGCCGAATCCGGCCACATCGTGCTGACCACACAGCACGCCAACAGTCCGCTGGGAAATCTCAACCGCCTGCAAATCATCGGCGTGCCACAGGGCTTCCTGCGCAG
CAGTCGTCTGTTTCAGGGACTGATTGCCCAGCGTCTGCTGCCGACACTCTGTCCGCACTGCGCACGGACACGGGAAGAGGCCGTGCTGACACCGGAGCAACAGCAGTTCACGGACCGCT

GGTTCACGCCACAGCAGCAGGAAGCCCTGCGTTTCCGCAATCCTGACGGGTGCAGTGAATGCATCCGTCCGGTGATGGGCACCGCAACCAGTCACGGTCTGAAAGGCAGGACGGTGGCA
GCGGAAGTGATGAAAACCAGTCCGGAAATCCTCCGCACCTACCACGATGAGGGCGAAGATGCGGCACGCACGCTGTGGCTGCGTCAGGGTGGAATCAGCCTGAGTCAGCATGCCCGGCA
TCATGTGCTGGCCGGACGGGTGGACCCGTTTATGGCCAATGACATCGTGCCGTTTGAGGATGCAAATGCACAGGAGGCAGAGCATGGCCAGTGA
>PHINNC_00465 hypothetical protein
ATGAATAACATCCCTCCGGCAGCACGACGGCTGCTCATCAGCACCCTGCTGCTGATGTCCGCCGGCAGCAGCCACGCGGCCCTGACCGTCGGTGACGTCGATGACCTGCAGCAGCAGAAC
GTCATTCTGTCCCAGCAGGCCCGTGCCGCTGAACTGCGCCAGCGCATTGCCCAGGCACAGAGTCAGGGGGCCGCCACCGTCCCCGCGCCGGTTATCAGCAGCGACGGTGCACCCGCCGC
GCCACAGGAAAATGCCCGTCCGCAACTGACAGGCATTCAGGGAGCACGCGGACACCTTGAGGCCCGTGTGCAGCATGGACGCCAGGTCGCGAGACTCACCACCGGCCAGAACTGGCCC
GGTACCACCATCACCCTGCGGGAAATCCGTCCTGACAGCGTGACGCTGAGCGACGGAACCCGCCTCACTCCGGGGGACAGCTATGGTCAGTAA
>PHINNC_00470 Type 4b pilus protein PilO2
ATGAGCACATCAGAGATTATTCCTGTCACTCTGCCCGGAAGACGTCCGGGACGCCCCGGACGTCGCATCTCCGTCATTGCCGGGCTTGAGTGGAAAGATATTCCCGCCGGACAGTCCACAG
GCAGACACATCCCGCCAGGCCGGGGGCAGTATGTAAAACTGCGCGCCGGCAGCGGTGGCACACACGTCCGCGTGGGATTCACCCGGTCACCGGGTCGCCTGTATTCACTGGCCGTGCTG
GCCATCCCGGCCCTGAAGGGGAACGGGTATGCCTTTGTCCGCCACGGAGAACGAATTGCTTTCCTGGCCTGTCTGCATGATATGCCCGCCCTGACCGGTGACGTGACCGGCAGCACGGAG
CTGGTCCGTAACGCCTGTACGGTGTTCAGGACAATGCACGATCGGGATGAACCGACCGACGGCTGGATGATCCTCGGCTCGCCGGAGGCACCGGTGGATATACAGGACGTCCTGCCGTCC
ACCCTGCCGGGCCGGGCACGTCTGCATCCGGTCAACCAGGCAATACGGATTGTCACTCTGACCGTGCTTGTCGCCGCCCTTGCCGGCAGTGCCCTGTGGGGCTACAGCCGCTGGCAGGAA
GAGCAGCGGCTGCGTCAGCTGCAGGCCATGAATCTCCGGCAAATGCAGGATGCAAAGCAGAACGGTCCGGAGCGGCAGGCCGAACCCCATCCGTGGCCGCAACTGCCGCTGGTGCAGA
CCTTCCTGGATACCTGTATTCCGGCACTTCGTTCGCTGCATGTCTCAGAGGGCGGCTGGCAGCTGACGGCCGCAGAATGTACGCCGGACACGCTGCGGGCTGACTATGAACGTCCGAAAGG
CTCCACGGTCACCTATGAAGACTTTCGCAGGGCTATCACACAGCACAACCGTTTTTCCTTTGTCACCCTGCAGGACGGCGGAGAAAAAGCCCAGGCAGGTTACAGCCTGAGCATGACGAC
CGGAACGGATGAATCCCTGCAACCCGTTGAGCAGCATCTTGCCCGCTTCCTGGCCCCCTTCCAGCGCGGGGACCGCCCGCTGCAGATGGAAACGGAGAAAATGCCGGATATCGCCGGGCA
CACGGTCACTATCGGAGACCGGGAGGTTCTGCTTTTCCGCGACTGGCAGCAGTGGCAGTTCTCACTGGAAGAACGTCTGCAGCCGGATGCAGCCCTGTTCACGCAGCCGGGCACGCGGC
TGACCGCCGTCACCCTCCTGCTCAGCGAAGACGGCGAACTCACCTGGAAAACCCGTGGCCAGACCTGGGCCACGCAGGCACAACAACCCACGGAGAACACTCATGAATAA
>PHINNC_00475 Outer membrane lipoprotein BfpB
ATGCGAATAAAACCCATCGCGGCGGCCCTTGTGGCGCTGCAGTTGTCCGGCTGCGCCACGCACCGGGAGTTCAGGGAGATCGATGCACAGACATCGGCCACCCGTGATCACGTTCAGTCT
GTACTGGACGAAACAAACAGACAGATGTCCGGTAGCGCACTGACCGTATCAGACAGCACCTGGATCAACCCGGAGCCGGTCCGCGCCACTAATGAAAAGAAGCAGGTCCCTGTTTGCTGG
CTGCAGGTCAACCGGACTGACAGCCTGTCCGTGGATGATGTGGCGGCCCTGATTGCCGACAGCTGCCACGTCAATGTCCGTGTCCTGCCGGATGCGCGCGTCAGTCACGTGGCCACAGCC
GGTCTCACCCGTGCCGTCCAGGGTGAACTCCCGCTGCCCGGCAGCACCTCCGCCGCCGACAGCAGGGGAAGAACGCCGCTCGGAAACACAGGCAGCAGTGGTCCAACCACGTCGGTATC
CACCGGAGGAACCCTGTCCGGCCTGCGCTGGGACGGCAGTCTCAGCGGCCTGCTGGATGACGTGACGGCGCGGATGGGCATTTCCTGGCGCTGGCGCAATAATCAGATACAACTTTTCCG
TCTCGACACCCGCACATTCCAGCTGGCAATCCTGAATGCCGACACATCAATGAATGCGAAGGTTGTGGGTGGAACCACCTCGTCCTCCGGGTCTTCCGGAACCTCCGGCAGCAGTTCACTG
AGCGGCGAACAGAGCACCTCCCAGCAGACCAACGTGGCGGTATCTTCAAAAGTGTACGACGACATCAAAGCCACCGTGTCTGCCATGCTTTCCTCAGAAGGCTCCTCCTGGCTGGCACCG
GGCAGCGCCACGCTGACCGTGACGGACACACCGGAAGTGCTTAACCGAATTGGGGAATACATCGACCGCCAGAACGCCATTCTTGACCGACAGGTGAAACTGAAGGTTGATGTCTACCGC
ATCACCTTCCGTGACACGAAGCAGATTGGTATCGACTGGACGGCGGTGTACAGCGCAGCAAAGGGCATGGGCTACACGCTGACCAGCGCCTTCGGGGATGCTGCATCTGATGTGGCCACC
ATCGGTGTCACCTCCTCTTCCGGTCGTCTGTCCGGTTCAGAGGTACTGATTAAGGCACTGAACCAGCAGGCTAATATCAGTTCGGCCACCACCACCAATACGGTGACCACCAACATGGTGCC
GGCCCCACTGCAGATAGCGAAAGAAACCACCTATCTGGCCAAAACGTCCACCACCACGTCGGACAGCTACAGCTCCACAGAGCTGGAACCCGGCACCATCACCACCGGTCTGTTCATGACA
GTGCTGCCGTATATCCGCGACAACGGCACCATACAGTTGCAGATGAGTTTCAGCCTGAGTGATGACCCGACCATCACCACAGTCAGCGCGAAAGACGGCAGCACCTCCATCCAGACGCCGA
CCACAGATGTTCGTTCACTGACCCAGCGTGCCAACCTGAAGCCCGGACAGACACTGGTGCTGAACGGTTTTCAGAGTCTGTATAACGAAGCAAACCGACAGGGCACAACGTCAAAAAGCT
GGTTCGGCTTCGGAGGAGGGGAAAGTGCTGAGCGTGACCGTTCCATGCTGCTCGTACTCGTGACCCCGGTACTCTCATCATGA
>PHINNC_00480 Type IV pilus biogenesis protein PilM
ATGCCTTTTGCCGTACTCGCACTGGCCATCGTGATGACATTCTTCACGATCCTTTCATACGAAACCGGGAAACAGGCCACCCGCACGGAAGAGCGCGTGCTGAAACGGGAGGCAGATGAC
GTCGCCTCACAGATGATGTCGCTGAGTCAGGCCCTGACGCACTGGCGGTGGAAGAATCCGTCCGCCACCGCCTTACCGGCTGTCAGCACACTCGGACTGCCGTTCTCCACACCGGACTCGC
GTATAGGGTATGCACTCAGTGGCGGACGCCTGTGGGTCTGGTCTGCAGAAGACAGCACCCCCGGTCTGGCGGCCCGTCTCACGACACTCACACTCGGCTCCGGCCTCCTTTTCCGGTTCAG
CAACGGCACCCTGAAGGACATGCAGGGCAACACCGTCAGCACCTCCGGTCTGACCCTACCCTCTGCCCTGCAGTCCACTTCAGGCACCCGGCTGGTTCACCTCAATTAA
>PHINNC_00485 hypothetical protein
ATGCAAAAAAAACACCTGACGCTCCTTCTGCCGTCACTCATTACCGGCTGCGCCGGTTTCACCTCTCCGCCGGAGACTGAAAACGCAGCCACTGCACTGGTCTTTGTCGACGGGCAAATCA
GCGAAAGCAGCTCGCTCCTGGCACAGACACAGCGGCGGCTGAGTCCACCGCCTGTCAGCCACCCTGCTGCCGTGACGAACACGTCCGCTCAGGCCGTGACGGCGCAGTCCCTGCCCCTG
CCGGCACCGGTACTCACGGCAGATGCCTCCCTGTCAAATATCATCATGACCGGCACTCCGGGAAGCGCTCCGGTCACGGCGCAGCCCCTGACACGCAACCGCACGGTCAGCGACTGGGTG
AAACAGCTTCTTCCCGCCGGCTGGCAGTTCCGCCAGGAGAATGCCGCCACACCGGCACTCAGGACGCGTATCGCATCCTGGTCTGCCAATGACCAGTGGACGCGTTCACTGAACAGATTGC
TGGTTGAACAACATCTGTGGGGGCATCTGGACTGGAAAAACCGCACCCTGACCGTCACCACTTCACCGGCAGCGCCAGCCACAGACAGCAGCCCGCAGGCCACCGCGCCGGCATCAACC
ACGTCTTCGGCAGCAGCATCGCCGGTGAAAACGAGGCCCTCTCCCGCACCGGTCAGCCCTTTCCGTAACAGCACGGCCACCGGTAAATCATCCCCCTCACACCTTAAGGTGTCAGGACAGT
CCGGTGACATCCCGGTCGTTTTCCTGTCGGAAGCCCGTAATCTCACCACGGAACAGTGGGTGAGCCGGTTACTGCCTGACGGCTGGAAAACCGACTGGCGTACCGACAGACAGGCACGTC
AGCACCGCACTGACCGGCTTGCTGTGAACGACCAGTGGACACGGGCGCTCGATGCCCTGCTGACACGCCAGAACCTCTGGGGACACCTGGACTGGAACAGCCGCACGCTGGTCATCACC
CCGACCGCTGCCGCACCACTCAGTACCAGCGGGACAGGCACGGCGAAGGCGACAGGGGCCACCGTTCAGGCACCGGCCCCCCTTCCGGCACCGGGCTGGACAGCAGAAAAAGGTGCC
ACACTGCGACAGACACTCACCCGCTGGGCAGCACAGGTGCCCTGCACCGTTACCGGTCATCAGCCAGGCCAGACCTGGTCTGTGGTCTGGTCCGCGTCTGTGGACTACCCCATCGACGCG
GCATTCACCCTGCGCGGGGGCTGGAAAGATGTTCTGACGCAGACCTTCGACCTCTACAGTCGCGCCAATACGCCGCTGCTGGGAACGCTGTTTGAGAAACAGTGCATCCTCAGCGTCCGC
GACCGGAAAGACTGA
>PHINNC_00490 Transmembrane protein
ATGAATATAAATTCCTTACCATCCCCCGAAATAAAAGACACCGATGTTTTCATTACCGTCATAACCGTTCTGGTGATATTTACTGCAGCATTAATCCCGTCGACTCCCCCGCCCCCGGAAGTGCA
GGAAACATTTGATGCCGTTTATTATGCATCCCTGCCTGCACTTACGCCCGTCATCCTGCAGGGATGGCAGTTCTGCTGCTCCGTGTTTGACAAGACGCCGGGGAGACAGGAGTTATCACCAG
TAGTGAGAACAGTGAATCTGCATCTTGCACTGGCCCCATTTCTGATAAACGTGATATCTGCGCTCGTTTTTTTTCGGCTGCATCGCAGATTGCGTCGTCATTTTTATTCACGGTCATGAACCTTCA
GCCCGGGGAATTATTCTTCCCGGCGCTGTTGCCCTTTGCCTGGGTTATATATTTTCTGTGTACAGATTGTACGCCTCGTCCCATGTACAGAACGGCAGACACATGACACTGCTCATCATGCTTT
GCTGTCTGACAGCCTGTTTATCGCTGGCAATTATTGCAGGCCGACAGGACATTTTTTCCGGAGCAGAAAGAGAAAGCCTGCTGCGTATCATAGTCTCGGGATTATGCTTCACGTTTTGCCTGC
TCATCGGATACACCCATCTCTGGCTTAATTCATCAGTCCCCCATCTGGAACAGGAGGATCTGAAAAACCGCCGATAA
>PHINNC_00495 Type IVB thin prepilin protein
ATGCCACAGCAACATCCCGGACGACTGCAGGTGCTGGTCGTCGACACGCGCTGCGAACGCAGACTGTTTTCAACGACAACACCGACAGACCCCGAGACGCTTGCCCGCCGTTTCTGCACG
CCGGACAACTGTCTCGTGGTGGTACTACGAGACAACAGGTTTCTTTTCCGTCTGGAGCGAGCCCCGGGCAGCCACAACCGCTGGCACAGGGGACGTCGTTCCCGCCATCAGCATCTGCAG
GACTGGCTATCATGA
>PHINNC_00500 hypothetical protein
ATGAAAACACGTATTTTATCGATAATCACCGCCGCCACAGTACTGTCAGGGTGCGCAGGCAGCAGCACGCAACCGGCGTCAGGGATGCAGACGTTACTCCCGGAACATCACCCGCAGGTGA
CATTCACGGTCGATACTGTGCGAACGGACACAACGGCCAGCAATCTTGTATGCCTGCCGGAAAAATACGGAAGCCTTCTTAATGAGCGCCATCCGGACCTGTTTGCCGGTGCTCAGGGGAT
TATTTCACGCAGTGCGTTTTTTAATGACAGCCTGACGCTGACACCTGAAACGCTCCACGGGATGATGCAGATTACGCCCTGTGTTGAATCACTGGTAACGTCCGCAGGTCCGGATACTTTCAC
TGCAGATGTCTCCCCCGGTGTCAGAATGCAGGTATCACCGGATATTCACGGCACGCCCGGAAATATCACTCTGCGACTCTCGACCACAGACCAGAAACGAAAACAGACTGTTGAACAAAGC
ATTTCTCTCAGACCGGAACAGTCCCTTGTCGTGAATGGATTCACCGGAGACGGAGCTGGCGAAAAGCGCGTCGTTCTCATCACACCTCACGTGCGTTAA

>PHINNC_00505 ClpB-D2-small domain-containing protein
ATGAACAACGAAGACAGCACCAGGAAAACATCCACAGGTATGCATCTGTGCTTTTCTCCCCTTTCAAAAAAAATTGTACTGGCCAGAATGCGGGAATCGAAAGGTCGCCTGCGCCGCGTCG
GCAATGACCGGGGACGTGATGTCACGAATGAAGCTGCCCGCCTGGTATGGGAGGTCGTCCGGGCTGAAGGCGGAAAAATCTGGTGGGAAGAGGAAGATGGCACCTGCCTGGTTTTAAA
AGCCGAAACCATAAAAACAGATGAGAAAGAAAACGCATGA
>PHINNC_00510 Inner membrane protein
TTGAAACGTGAACATCTTATTCCACTGGCCATGCTGGGCGGCTGGTGTGTATTCGTACTGTTCATATCCCTGCCGGGGTTGCGTCAGATGAGCACATGGCCTGCACATGACCGGATCGTCATG
TTGCTCATGATGTTCACCACCATGTGCCTTCCCCTGCTGTTACGCCCGCTGTCCGTGTTCTTTCGCAAAATATGCAGGCAGAACAGCTTTTATGACCGGGAGCTGCCGGACAATCACACGGTC
CATATTTTCCTGAGTGCACACGCCGACACGTCCTCCCCGGTGGCCATGCGTCGCCACTGGAAAGTGCTGAACGAACTGCTGACCACCGCATTACGCCAGGGCAAACGAGTCTCGATGACAT
CACATCTCCTGACGCAACCCCGGACGGACAAACTGGTTCGTGCCCTGCAGAAGCAGGGGCTGGAGGTTTCTGTGAAACGGGATGAGTGCCCCACACCGGCTTTCGAACGCTGGACCATC
ACCGCCAGCTGGACCATCAGCCAGTGGAAGATTCCCCACGTAAACAGACGCAGCGGCATTGTCATCCTGACGCCGGAGAGCTGGCGTCAGCCATAA
>PHINNC_00515 hypothetical protein
GTGAACACGACAGAACATCCCGTGCGCCAGTTACTCTGGTGCGCCCTTAATGCCCTGCATGCCGCTCAGGAAAACCACGGCATCAACAGTGAATCCGGACTCCGCCGCTACCTGCTTGAGT
GGCTCTCCGGTGCCGGCAGACACCCTGAATTCCGGAACATCCCGGAAGAAATCATGGCGCTGAAGGCACTGGCGGAACAGGACCGCGATATCCCCATTACCGGCACGCTGAACACGCTCT
TTCTCAGCTCGGCCACAGTCGGGGAGTGCCCGCTGTTCCGCTTCCGGGCCGCAATGGGCAGGTTACAGAAGGCCGGATGGCGCACAACCGTCTGTCCCTGGACGGAGCGGGTCTTCAGC
GAGTCCATTGAACTGGCCTGTACCGGCAGACGTCATCTGCTGCAGCTGAGTCGCACGGAGGAATGCTTCATGCCGTCCGGACAGATGTGCGCCCCCGTATACCTGCAGCTCATCACCCCGCA
GCATCGCAAAACAGAGCAACTTCTGGAAACGGCAGAAAACACCCTGGCTGATGAAGGTTTTCAGGTGGTGAGGGGATTTGAGCATCAGTTCGGCATCGAACGACGTGAAATCCTCTTTCA
CACCCTGTTTGTCGGCCTTCCCGGCCTGCCTGAAGAGGCATGGGGCGTCAGGCCGGAACAGACCATCACCGTGCATTAA
>PHINNC_00520 Conjugal transfer protein TraB
GTGAATATAGAACACCTGAATAACCGGAACTGGTATCTCGCACAATATAATACAGCCGGTAAAAACAGAGAATCTTTATTCTCATGGCTGAATGAGCAAAACGTCATACCATGGACTCCATTAA
TAACAAGAAAGATAAGACGGGCAGACGCAGGATGCTGTTTCCGTAAACGGGTATACGCTATTTTCCCTGGGTATTTTTTTGTTCTGGTTAATTTTGATATTCAGCCGGTGTCGGCATTACGTC
GACATAGCGCCTTTATTGATTTCGTGAAGTTTGGCGGTGAAATCAAACCCGTCAATAAAGATATTGTTGATGGGTTAATGAAGATTTACCCGGACCCTGTGCTGAATCCGGGAGCCAGGGAA
GAGCTGAATGCGGCTTCCGATATATGGCTGACAAAAGCACAATATCAGTATCTCCTCAGAATGGAAAATACTCTCCAGCCTGAGTCGAGGATATCCCTGCTCCTGGAACTGGTATCAAATGCC
GAACATCACGGCTTTATGGAGCACCTTGTAAACATCCCATAG
>PHINNC_00525 Conjugal transfer protein TraA
ATGTCACCTCAAATGATACAGAAACTCCCGGCCATCCATCTGTTGGAAGGAATGTTTCCCGAGCTAACAACAAATCAGCTTAAAGTCTGCGTTTTTTATGCGATGGGTGTTCCTTATGATGCCA
TTGCACAGAACTGCCGCCTTTCTCCCGAAACGGTACGAACCTACCTGAAGCGAAGCCTGAAAAATTTAAATCTGGATGGATATGATGCACTGCGCTCTGCCGTGCTGATGCGAACGTTCGTT
TTCATGATTGGCAATACTGCCAAAGAAAACGAAAAAATGTGA
>PHINNC_00530 C4 antisense RNA
TTCTATGGTAGCTCAGGCGGGGCAGGCATTAGCCTGGCCGGTTTCCGATGACGCCGGTATTCCTACCCCCGTCTGGGCTATCACCAAT
>PHINNC_00535 hypothetical protein
GCTGTCGCTTATGGCGGAGCGATTGTGCCGTGTATTTATACACTGCACAGGGTTGAATCTCTGGTACGGTTTCATTATACTTTACCCGTATTCTGTGAGGCCCCCGTAATCTTCTAG

PTU-NA 2 (IncI1, MOBP)

>LOLKGF_00005 Protein ImpA
ATGCTTCGTCTTTATTCGGCGGCATCAACCGCTGAGCCTTTTTCCGCCCCACTGTTTCTGGAGCGGTGCCAGGCAGGTTTTCCTTCGCCGGCAGCGGACTACACAGAATCTGAACTGGATCT
GAACGAGTATTGTATTCAGCGTCGCAGTTCAACTTACTTTGTCAGAGCCATCGGCAACTCTATGCGGGATATGGGTCTGCACAGCGGCGATCTGCTGATTGTGGACAAGGCAGAACGTCCTC
AGCACGGCGACATCGTGATCGCGGAAATTGACGGAGAGTTCACGGTCAAAAAGCTGCTGTTGACGCCACGACCGGGGTTACAGGCGATGAACCCGGACTACCCTACCCTTTGGCCAGATC
CCGATAACCTGCAGATATTTGGGGTCGTTACGGCATTCGTACATAAGACACGGGGACGGGACTGA
>LOLKGF_00010 Protein UmuC
ATGTACGCCCTGGCTGACGTCAACAGCTTCTACGCGAGCTGCGAGAAAGTATTTCGTCCCGATTTGCGAAACAAACCGGTTGTCGTTCTGTCCAATAATGATGGTTGTGTAATCGCCCGCAG
CCCCGAAGCAAAACGGCTGGGCATAAAAATGGGGGTACCGTGGTTCCAGCTGAAGATGACACAATTCCCGGAGCCGGTCATCGCGTTCTCAAGCAATTACGAGCTCTATGCGTCTATGTCG
AACAGGGTAATGTCTCATCTCGAGGAACTGGCGCCGCGCGTGGAGCAGTATTCTATTGATGAAATGTTTCTTGACGTGAGTGGTATTGACAGTTGCATTGACTTTGAAGACTTTGGCCGACA
GTTGCGTGAACACGTCCGGAACGGGACCGGCCTGACTATCGGCGTGGGCATGGGGCCAACCAAAACGCTGGCAAAAAGCGCGCAGTGGGCATCGAAGGAATGGCCACAATTTGGTGGC
GTACTGGCATTAACAACGGGTAACCCCCGACGCACGACAAAACTGCTCTCGCTCCAGCCGGTCGAGGAAATCTGGGGAGTTGGTCGCAGGATATCCAGGAAACTGAGCACAATGGGTATA
ACCACTGCACTGCAGCTGGCCCGCGCGAATCCGGCCTTCATCCGGAAAAATTTTAATGTTGTGCTGGAGAGAACGGTCCGCGAACTCAATGGTGTAAGCTGTATATCACTGGAAGAAGCCC
CCTCACCAAAACAGCAGATAATCTGCAGCCGGAGTTTTGGTGAACGTGTCACAACTTACGAAGCGCTACGTCAGGCCATCTGCCAGCATGCAGAGCGAGCGGCCGAGAAACTACGTGGTG
AACGCCAGTTCTGCCGGCATATTGCTGTCTTCGTTAAAACATCACCTTTTGCCGTCAACGAGGCGTATTACGCCAATGTGGCCAGCGAAAAACTACTCCTGCCTACCCGAGACACCCGGGAC
ATTATCAGCGCCGCAGTAAAGGCCCTGGACAGTATCTGGCTCGATGGTCATCGATATGCAAAGGCTGGCGTAATGTTGAATGATTTCACGCCCAGCGGCGTGTCTCAGTTGAATCTGTTTGAT
GAAGAACAGCCCCGTGCCCAAAGCGACGAACTGATGAAGGTTCTGGATAGGATCAATCATTCCGGGAAGGGAAAAATCTGGTTTGCAGGGCGGGGAATAGCCCCTGAATGGCAGATGAA
GCGAGATATGCTCTCGCCTGCGTACACTACAAGATGGAGTGATATCCCTGTTGCACTATTGTGA
>LOLKGF_00015 Molecular chaperone GroEL
ATGAGCAAAAATAAGATTAACGAACAGACTGAGAAACTCAAAAATTTAGTGGGCTCAGCTCGGCTGCAAACGTCCACACAAATGGCTGCGAAAAATGATGCAGCTCACGGTGGTGACGAA
ATTAGCAACGAAGCTAAGGGTTCTGAAAAGCCGATCCAGGTTACAGAACGTCCTGAACCGGTGAGAACTAAGGCTAAAAATCTCAAGGCCATTCCTGTTACATATTTTGAGGCTCATGACG
AGCTAAAAGCCACCTGTAAGACGTCTCTCGATTTTAGCTCATATATTATTGAAGCTATCCGAGAAAAGCTTGAGAGAGATGGTGCTATTGGGAGTTGA
>LOLKGF_00020 ParA family protein
ATGGGTATAATTGCTAATGCTCACCCGAAAGGCGGTGTAGGTAAAACTACATCTTCAGTTAACCTGGTTGGTGAAATGAAAACAGATGCTGTGGATCTTGACACACACACTGGGCTTTCAATC
ATCCTGGGGTTACGTCCAGATAGTAAACAGGTTTCTGTAAGGGTACCGAATACTGTCGAAGAGCTGATTGAAATAATGACGCCTTATAAAAATAGCGATAAAGACCTGTTTATAGATTGTGGG
GGGTTCGACTCTGATCTAACTCGTACCGCAGTAGCGTTCGCTGATTGTGTCATCGTTCCATCTAAGGACTCTCTAACAGAACGGATTGGGCTTATGCACTTTGATGCTGTTCTCGCTGAAATCA
GTTCAATCATGGAAACAAATATCACAGCACATCTTTACCTGTGCAAAGTTAACCCAAACAAGAAAAACTTCCCGAAACTTGAGGCATTACTCCCTACCTTCAAGCACCTGAAATTAATGGACA
GCCGGATATCTGCTCGTGCTGAGTTTGAAGACGTTATTGAAAGTGGAATGGGGATAACTGAAACCTTGCATGGACGCACCACACAGGGTGGCAAAGAAGTCATAGCTTTAGTTAATGAGAT
TAAGCAATTGATGGCGAATAAACGTTAA
>LOLKGF_00025 Protein RepA
TTGAATAAAATCAATGAGGCTATGAGTCCGTTTGCCATTATCAAAAAGGACTCAGGCACAGCTTACGAACTGGTTCCCAACAGTAGTAAAACGGTACAACCAGTAGCTCTACTCAGGCTGAG
CGTCTTTACGCCAGTATCACCGCGTGAGAAAGGTAAACGCGATTTTCAGATAGATGCGTCAGAAGAGCTTTCAAGCCTGGAAGTAGCTCGTCAGGAAGGTTATACAAACATCAAAATTCAA
GGTGCTAAGCTGGGGATGTCCACTGATTTCAAAACGTGGATCGGCATAATATCTGCTTTCTCAAAATACGGGTATGAGAGCGAGAAAATCACGCTTCCTTTTTCAGAGTTCGCGAGAATGTG
TGGATTAAAGCCTACTGATATCAACGGGCGGGCCCGCACACGTCTGAGCGATTCTCTGTTTAATCTTTCAAGCGTTACTCTTTCTTTTCGGAGCAAGGACGGCAAGCGAAGCCTCATTACCC

ACTTGGTCCAGCGTGCGGTTCTGGATATGGAAGCGGACGTTGTAGAGATTGTTGGTGATAAGAGCCTATGGGAATTGTATCGATATGACCATAAGGTTCTGCTCGGGTTGAAGGCATTATCT
GAACTCTCACGAAAAGAAGCTGCACAATCGCTCTATGTTTACTTTGAAAGCATGCCAGCTGGAACTCTTTACGTTTCAATGAAGCGTCTGCGAGAGCGTCTGGCTATGGAGTCACAAATCAA
GGATCAGAACGCGATAATAAGACGTGCTATGGGTGATCTGCGTAGAATTGGGTATCTCGATTACAATGAAACAAAAAAAGGTAGGGAAATCATGTTCATCATTCATAACCGCTCGCCTAAATT
AGGGCTTGCAGCCCCACGGAACCCAGATTAG
>LOLKGF_00030 Tyr recombinase domain-containing protein
ATGGTGGCCACGCTGAAAATCCCGCTGGAGCGTCGTAACAGACGTACCGGCAGAACGGAAAAGGCACGCTTCTGGGAGATAACCGACCGGACGGTCCGTACCTGGATTGGGGAGGCGG
TTGAGGCCGCAGCGGCTGACGGCGTGACGTTCTCAGTGCCGGTGACACCCCATACGTTCCGCCACAGTTACGCGATGCACATGCTGTACGCCGGCATCCCGCTGAAGGTGCTGCAGGCGC
TGATGGGGCACAAGTCTGTCAGCTCGACAGAAGGTCTATACGAAGGTGTTTGCGCTCTATGTCCCGCACGGCACCGGGTGCTGTTTCAGATGCCGGGTACTGATGCTGTGCCCATGCTCAA
AGGAATTTGTTAG
>LOLKGF_00035 MchC protein
ATGAACCCAATATGTTCGCTTGCTGAACTCAATGAAAACCTGGTGCCCTTTACTGCCCGACAGGTAACCTCAAAGTTGATCTGGCGTGCTGAGGATAGCCTGAATATCGAAGTATTACAAAAA
GCCTGCAGCTATATAATAGATTCGGCCAGTTCATCTTCACACAAAATATTCCATGCAGAGCGTTATGGTGGAAGTGGTATTCAGCGTAACGGCGGGGGCGCTCGTTGTGGTTTTGATGGTAGT
TATCAGATTAAAGGTATGGGAACCAATCCCTTAGTCGGTAAAGGGACTGATGGGCGTCACTCTAATGGTGCCCTCGGTGCCATCCATGCAATCTATGAGGCTTTATGGGGAGAGGTGCTGGC
ACAAATATTACCCTATGGTGCTGTGCGTGCCAGGGCAGTTTTACTTACGGATATATATACCGACAAGGCATTTGACCGCCCTCACGGGAAATCCCGACGAGCCCTGTTGGTACGCGAACCAGT
GATTCGCCCGGCGCATTTTGAACGAGCCCCCTATTTCAGACCTCAGCCGGAATATGTCACTCAGTTAGTACATGATGCCCGGCGGGTTCGGTCTGTGATCCACATGTTGCCCGGAAATTTACC
TGTACCGCCAGAGGGAGTCAGCGAAGAGGCTCAACGTGATCACCGGGTTTATTGTATTGAGGGGTTATGTGAACTGGCCCGCCGTGAAGCATGGCAGATGGCGTTTTGTCGTACACGCTTC
CTGAGGTTGACAACTTCTCCTTCCAATATTGCGATAGATGGCAGGTTAATGGATTTTAACGGACTCAGTTGTCTGTTCCCTGGAGATTATCCCGATGATTTTGGATATCGGCTCAGGCTTGCCG
AACTGCAGAAAGAACCGGTGGTACTGATTCAGGGACTGTCAGATCTCTGTCTTTATCTCGGGAAATACCTGTTTGATCCCGATTTCACAATGGTGGCACGCCAGAAGGTTGAGGAGACCTTT
CAGAAAACTTTCCATGAAGCATGTTATTACTGTTATCTGGAACAACTGGGTATCCCCACAGAGTTTATGCCAAAGGAGGGTATCCCGGATACGTTGAAAAAACAGGTCAACAGTTTCGTTGTA
TTAGTCAATAAACGCAGCGATCGGTTGTACTGTCCAGATGTCGGATGCAAAGAGGATTCACCATTGCAAAGACTCGTGGTGGAACTGATTCGGCAGAGCCATGGCCCAATCAGACCTGTGG
ATAATGACGCTCAGCATGATGTTCATTTCACAGAAGCGCAACAGTGTTTTACCTGCGCGATACAATGGCTGATACAGGTCGGTATCAGATACCCGACTAATGTTTCATCTCTTCTTAAGGAAAT
GGAAAACCATGCCAGGAAGCGACTGCAACCCAGAAAAGACCTGGGGAAAGTCACTATGTCCGAGAAAATTGCTTCCCTTCTGGATAAACACGGTGATGATCACCATTTTTTGCAGGAGGC
ATTTTCTGATATGGGTGTCCAAATGCTGGAGTTTTGCAGAGAAGCAATCGGCCATTTTAGTCCTGTCAGAATTGCGGTGTAA
>LOLKGF_00040 RTX toxin-activating lysine-acyltransferase
ATGATGTCGTATATACGAGAAACCATCAGTGGAAGAGCGGAATGGACCGTCTATGAACAGATTGGTTTTGCTGTCAGCTGTATGCTCCATAACCGTAATTACAGCCTGTATCCTGTGTTGACCA
TTCAATACTGGACTGAATACGCTATACAACACCATCAGATTAAATTCCTGTTTGATTCGCGTGGTTTTCCACTGGCATATATCACTTGGGCATATCTCGAGACAGATACAGAAGCGCGTCTGCTA
AACGATCCTGAGTTCATACTACATCCTTCTGAATGGAATGAAGATGGAAGGATATGGATACTGGATTTTTGTTGTAAGCCTGGTTTTGGGCGTAAGGCTATTGAATGTTTTACAGATCTTCGCC
CATGGGGAGAAGGAGAAGTGCGATGGCTGAACAGAAGAAAGAAAATCATGACAGTACGGCAGAATAAATTACGACATATCCCAGAGCTACAGAAAAAAATATAA
>LOLKGF_00045 Colicin V secretion protein CvaA
TTGTTTCGTCAGGAAGCGTTAGATAACAGGAAAATGAAATGGCGTGGACGAGCTATTTTACTGCCAGGAATGCCGTTATGGTTAATCATGCTGGGAAGCATTGCGTTTATCACGGCATTTCT
GACGTTCGTTATCGCTGGTACCTATAGCCGTCGTGTCAATGTCAGTGGTGAGGTCACGACCTGGCCGAGAGCCGTCAATATATACTCCGGTGTGCAGGGGTTTATAGTCAGGCAGTTTGTGC
ATGAAGGTCAGTCGATAAAAAAAGGTGATCCGATTTACCAGATTGACGTCAGTAAAAGCACACGCAGTGGCGTTGTTACCGATAATCAGCGAAAGGATATTGAAAATCAGCTGGTTCGCGT
GGATAACATCATTTCCCGTCTGGCGGAAAGTAAAAAAATAACGCTGAATACGCTGGAAAAGCAACGTCTGCAATACAGCGATGCGTTCCGTCTCTCATCAGATATTATACGGCAGGCAGCGG
AAGGGATAAAAATAATGAAGAACAACATGGAGAATTACAGAAACTATCAGTCAAAAGGGCTGATTACTCAAGATCAGTTAACTAACCAGGTGGCATTATATTATCAACAACAAAATAACCTTC
TCAGCCTGAGCGGACAAAATGAACAGAATGCCCTGCAGATAACCAGTCTGGAGAGCCAGATCCAGACTCAGGCTGCGGATTTTGATAACCGTATCTATCAGATGGAACTGCAACGGTACGA
GTTGCAGAAAGAGCTGGTTGATACTGATGTGGGGGGAGAAATCATTATTCGTGCGTTGTCTGACGGGAAAGTTGACTCCCTGAGTGTCACTGTCGGGCAAATGGTTAATGCCGGAGACAG
TCTCCTGCAGGTTATCCCTGAAAATATCGAAAACTACTACCTTATTATCTGGGTCCCTAATGATGCTGTTCCTTATATTTCGGCAGGAGATAAAGTGAACATTCGTTATGAAGCCTTTCCTGCAG
AAAAATTTGGTCAGTTCTCTGCTACGGTGAAAACCATATCCAGAACGCCAGCCTCAACGCAGGAAATGCTGACCTACAAGGGGGCGCCTCAAAATACGCTGGGCATCTCTGTTCCCTGGTAT
AAAGTCATCGTTAAACCTGAAAAACAGATTATTTCTTATGACGGGAAATCTCTCCCTCTTGAGAATGGAATGAAAGCTGAAAGCACATTATTCCTGGAAAAAAGGCGTATTTACCAGTGGATG
CTTTCTCCTTTCTATGACATGAAACACAGTGCAACAGGGCCGATCGATGAGTAA
>LOLKGF_00050 microcin H47 export transporter peptidase/ATP-binding subunit MchF
ATGAGTAACGGGAATGTCAGACGAATGATAAATCAGCTTGATATGCGCTGGCGACGTCGGGTTCCGGTTATTCACCAGACGGAAACCTCGGAGTGTGGACTGGCCTGTCTGGCAATGATAT
GCGGACATTTTGGCAAGAATATTGACCTGATATCCCTTCGACGGAAGTTTAATCTTTCTTCCCGTGGGGCAAACCTTGCGGGAATAAACAGTATAGCGGAACAGTTGGGGATGGTAACCCG
GGCACTTTCGCTGGAGCTGGATGAACTTGGTGCGCTCAAAACGCCATGTATTCTCCACTGGGATTTCAGCCATTTTGTTGTACTGGTCAGCGTAAAGCGTAACCGTTATGTACTGCATGATCC
GGCCAGAGGCAGAAGAAATGTCGGACTGGAGGAAATGACCCGGTATTTTACCGGAGTTGCGCTTGAGGTCTGGCCAGGCAGTGAATTTTCTGCGGAGACCACGCAGAATCGAATCCATCT
ACGCTCACTTATTAACAGTATTTATGGTATTAAAAGTACACTGGCGAAAATCTTCTGTCTGTCAGTTGTTATTGAAGCTATCAATCTCATAATGCCTGTGGGTACTCAACTGGTTATGGATCATGC
GATTCCGGCGGGGGACAGAGGGTTACTGACGCTCATTTCTGCCGGCCTTATGTTCTTTATACTGCTCAGGGCAGCCACGGGTATGCTCCGTGCATGGTCCTCGCTGGTTATGGGCACACTCA
TCAATGTTCAGTGGCAGTCGGGTCTGTTTAACCATCTGCTCAGGCTACCGCTGGCATATTTTGAACGTCGCAAATTAGGTGATATCCAGTCGCGTTTTGGTTCCCTTGATACTCTGAGAGCCAC
CTTCACCACCAGTGTGGTGGGGGCCATTATGGACAGTATTATGGTTGTGGGTGTTTTCGTGATGATGCTGTTATACGGTGGGTATCTTACATGGATAGTTCTCGGGTTTACCACGGTTTATGTT
CTGGTTCGCCTGGTGACATACAGCTATTACCGGCAGATATCGGAGGAAACGCTGGTCAGGGGGGCCAGAGCCAGTTCCTATTTTATGGAAACCCTGTATGGCATTGCCACTGTAAAAATTCA
GGGCATGGCTGAACGGCGGGGAACGCACTGGCTTAACCTTAAAATTGATGCCATCAATTCAGGCATTAAGTTAACCAAAATGGATTTATTCTTTGGCGGGATAAATACTTTTGTTGCAGCCT
GTGACCAGGTGGCGATTTTATGGCTGGGTACCAGCCTTGTGATCGATAATCAGATGACAATAGGGATGTTTGTGGCGTTTGGTTCTTTCCGTGGGCAGTTTTCGGATCGGGTTGCCTCTCTG
ACCAACTTTCTTCTGCAACTGAGAATGGTGAGTCTGCATAACGAGCGTATTGCTGATATCGCACTGCATGAAAAGGAAGAGAAGAAACCTGAGCTTGATATTGTTGCTGATATGAGCCCGGT
CTCTCTGGAAACCACTGATTTAAGTTACCGGTATGACAGCCAGTCAGCGCCGGTGTTCAGTGGTCTGTATCTGTCAGTGACTCCCGGAGAAAGTGTGGCCATAACCGGTACATCCGGTGCAG
GAAAAACCACGTTAATGAAAGTATTATGTGGGTTGTTTGAGCCAGATACGGGAAAAGTACTGGTGAATGGTACGGATATACGGCAACTGGGGATAAACAATTATCACCGGATGATTGCCTGC
GTGATGCAGGATGACCGATTATTTTCAGGATCTATCAGAGAGAATATTTGTGGGTTCTCTGAAGAGATGGACGAAACCTGGATGATCGAGTGTGCCAGGGCCAGTTATATTCATGATGTGATA
ATTAAAATGCCTATGGGATATGAAACCCTCATTGGTGAACTGGGGGAAGGGCTTTCCGGAGGGCAAAAACAACGTATATTTATAGCCAGGGCTCTGTACCGTAGACCATGCATATTATTTATG
GATGAGGCCACCAGTTCACTTGATACTGACAGTGAGCGTTTCGTCAATGTGGCTATAAAGAATATGAATATCACAAGGATTATTATTGCGCACAGAGAAACAACGTTAAGAACAGTTGACAG
AGTAATTCTAATTTAA
>LOLKGF_00055 hypothetical protein
ATGCGTAATCTTAAAGAGAATGAGTATTCTTTTATCTACGGCGGGGGTCAAGACGGAGGAAATTCCGGAGTATCCAAGGAAACGATTGGCGGAGCTATAGGTGGGGCCATTGGTGGGACAT
ACGGAGGTATCCCTGGAGCTGCCATAGGTACAATCATGGGGGCATCTATTGCTAATGGCTCCCGAGGTACAGGTAGTGTTGGCCCTTCAGGTATTCCATGGGGTGGCTCCAATGGTGGTGGG
GTAATGTGGGGCAGTAGCAGCAGCTCCTCTGCAAATGACGGAAGTTGA
>LOLKGF_00060 CAAX protease family protein
ATGGAAGACAAGAAATACAGTTGTGAATATATTGAAAGAAATAAAGCTGTGTCCGGATGTTTTGCTATGTTCATGCTGTTTGTCGGAATAACCTTTATCCCATTTTTTTAAAAGATGGTGCGG
AACTTATGGCTAAGGGGCTACTATTCCCAATAATCGTTACTCTTGAATTTATATTTATTGTTCCTCTCTATTATATCTTTTTTCGTAAAAGAGAGGGGGTTGGGCCTTGGCAGCTTCCGGTTTAAAA
CGTTTTTTATACTGTTTCTGCTCATTCTTCTTATTCAGTATCTGCTCCCCTATATTTCCGGAGTAAATGAAACTGAAAGTTGGTCCGAGTCCCAGATGGCTCTCGGAGGCTATATTTTCTGGATAA
ATGCCATTTTGCTAATCTTCATTGCTCCTGTCTATGAAGAAATAATTTTCCGTGGATGCCTGTTTAATATAACTTATTTTTGGTTTGGGAATAATGTTTTTGGTACCGCTGTTATTGTTTCTATGATG
TTTTCAGTTGTCCATTTGCAGTATACTGAAATAAGAACATTTATCGTTTTATTTCTGGTGTCTCTAACATTATCGGCGGCACGAGTTAAAAGCCGTGGGCTTCTGATGCCAGTGCTTCTTCATATA
TTGATGAATGCGGTGGTTACCGGTATACAGTATGCGGCTTACATGCTTCCTGCCTCATCCGGTTAA

>LOLKGF_00065 Resolvase ResD
ATGAGTCAGCCACCGTTACCTGCTGTCTTTACGCAGACCCCGTCCGCCCTGCTGCCGGTGGCCATTGATTACCCGGCTGCCCTCGCACTGCGCCAGATGGCAATGCAGCATGACGACTACCC
GAAATATCTGCTGGCACCGGAGGTGAGTGCCCTGCTCCACTACGTCCCGGATCTGCACCGCAGGATGCTGCTGGCCACTCTCTGGAATACCGGCGCACGGATTAACGAAGCGCTGGCCCTG
ACACGGGGAGACTTTTCGCTGGCGCCACCGTATCCGTTTGTGCAGCTGGCCACCCTGAAACAGCGGGCGGAAAAGGCGGCCAGAACGGCAGGACGAATGCCGTCCGGCAGCCAGCCCC
ACCGTCTGGTGCCGCTGTCAGATAACCAGTATGTCAGTGAGCTGCAGATGATGGTGGCCACGCTGAAAATCCCGCTGGAGCGTCGTAACCGACGTACCGGCAGAACGGAAAAGGCACGCC
TCTGGGAGATCACCGACCGGACGGTCCGTACCTGGATTGGGGAGGCGGTTGAGGCCGCAGCAGCTGACGGCGTGACGTTCTCAGTGCCGGTGACACCCCATACGTTCCGCCACAGTTAC
GCGATGCACATGCTGTACGCCGGCATCCCGCTGAAGGTGCTGCAGGCGCTGATGGGGCACAAGTCTGTCAGCTCGACGAAGGTCTATACGAAGGTGTTTGCGCTTGATGTCGCCGCACGG
CACCGGGTGCAGTTTCAGATGCCGGGGGCTGATGCTGTGGCCATGCTGAAAGGCGAAATGTGA
>LOLKGF_00070 hypothetical protein
ATGACACCACCGGTGGCCGGACGGCAGAACCGTCACAGCGGCGACTGGGATCCTTTATACAGGGTTCAGACTGAAACCAGAGATGACTCCGGGAAAAAACATGATGTCCCGGAAGATTTT
ATTTAA
>LOLKGF_00075 Cytoplasmic protein
ATGACACGGTCACGCGTCTGTCCGGATACTGAATCCACCGGTCTCAGTCCGGCATCGGATGCCCTGCCGGTCAGGGAGACGGCAGAACAGTTTGCACAGGTCTTTTTCGGAAAATCCGTGT
CCCTGCTGACGTCGGAAGAGCGGTTCACGACGGTGTACACCGGCTCCCGGGACAGACCTGCGGATCTGCATCCGGCGAGCTGGTTTCCGGCCGGCACCTGGTTTTGTCACGAACTCCACG
CCTGTGGTGACCGGTGCGGACGTCGTCAGGGCTGGCCACTGTATCACGCTTCAGAAGCGGAACGGCTGCGCGCACTCTGTCCGCCACGGCTCGCCACGCCCGCGACCGGTCCGGGAGAA
CAGTTGCTGACCCGTACGGTGCTGCTGAAAGCGGGTTACTCACGTCGGCCATGA
>LOLKGF_00080 Cytoplasmic protein
ATGCGTGTCAGCGAACAGGTGCTGCTCAGTAGTCTACGTCAGGGCGGCTGTGTGCGGAGTTTCTGGCGCCGCTCCGCCAGACTGACGGGTACGCCCTCTCCGATCGTTCCGGACGGGCTT
GTGCTGGAAACCCCCGGTGAACGCGGGGATACCCCACTCTGTCATGTGGATTTTGCGGTGGTACAGAAGTGGCTGGTCTGTGATGAAACCTGGACACAGACCGTCGGGGGAACAGAGTT
CGGCGGGGCGGTCTGGCGACTGCGTACTGACAGGGAAAACACCACCTCATGA
>LOLKGF_00085 Toxin CcdB
ATGCAGTTTAAGGTTTACACCTATAAAAGGGAGAGTCGCTACACCCTGTTCGTGGATGTCCAGAGTGACATTATCGACACCCCCGGACGGCGGATGGTGATCCCGCTGGCCAGCGCCCGTCT
GCTGTCGGATAAGGTCTCCCGCGAACTTTATCCTGTGGTACATATCGGGGATGAGAGCTGGCGTCTGATGACCACAGATATGGCCAGTGTGCCGGCCTCAGTCATCGGGGAAGAGGTAGCC
GATCTCAGTCACCGGGAGAACGATATCAAAAACGCCATTAACCTGATGTTCTGGGGGATTTGA
>LOLKGF_00090 hypothetical protein
ATGAAGCAGCGAATTACAGTGACAGTGGACAGCGACAGCTATCAGCTGCTCAAGGCATACGACGTGAATATCTCCGGTCTGGTCAGTACGACCATGCAGAACGAAGCCCGCCGTCTGCGA
GCCGAACGCTGGCAGGAAGAAAACCGGGAAGGTATGGCTGAGGTGGCCAGCTTTATAGAGGCTAACGGATCGTTTGCTGACGAAAACAGGAACTGGTGA
>LOLKGF_00095 AbrB/MazE/SpoVT family DNA-binding domain-containing protein
ATGAGAACCGTATCCATTTTTAAAAACGGCAACAACCGCGCCATCCGCCTGCCCCGTGACATGGATTTTGAGGGGGTGAGCGAGCTGGAGATCGTCCGGGAAGGGGACAGCATTATTCTG
CGCCCCGTCCGGCCGACCTGGGGCTCATTCCTGGAGCTCGAAAAGGCAGATCCGGACTTTATGGCGGAGCGCGAAGACGTTGTCACCGATGAAGGACGATTTAACCTGTGA
>LOLKGF_00100 PilT domain-containing protein
GTGAAGAAAATGTATATGCTCGACACGAACATCTGCTCGTTCATCATGCGCGAGCAGCCCGAAGCGGTGCTGAAGCGCCTGGAGCAGGCGGTGCTGCGCGGCCACCGTATCGTGGTCTCG
GCCATCACCTACTCCGAGATGCGCTTCGGTGCCACCGGCCCGAAGGCCTCGCCGCGCCACGTGCAGCTGGTTGACGCGTTCTGCGCCCGCCTCGATGCCGTCCTGCCCTGGGACCGTGCCG
CGGTGGATGCGACCACGGAGATTAAGGTGGCGCTTCGCATGCCGGCACGCCGATCGGCCCGAACGACACGGCGATTGCCGGGCATGCCATCGCCGCCGGTGCCGTGCTGGTGA
>LOLKGF_00105 KAP NTPase domain-containing protein
ATGAGAAGACCATCCCGAACGGAAGCACTGATGCCAGTACTGAGTGCTAACAATGCTCAGGAAGAACAGATTAACACCACCTATGAACCTCTGACTCCCTCAGTCATTGAAGATGAAAAAG
CACAAAGCTATATCGAAGCTCTGAATTTTGCCTGTTCTCGCCGGGATATCAGAAATATTGCTGTAACAGGCCCGTACGGGGCCGGTAAAAGTTCGGTATTACTGACATGGGAAAGGGCAGAG
GATAATGACTTTCGGGTGATGACCGTATCACTGGCCGACTTTGAAATGCAGCGGGCATACTCCGGAGATTCCCAGGCAGTTGAGGGTAAACCCGGCTACGATGATAACGATAAAAAAGCGG
GAAAAGCCGAAGAGAAAACAATCGAGTACAGCATCCTGCAACAGCTGCTCTACAAAGAAAAAAAAAGTGTGTTGCCCTATTCCCGTCTGGAGCGCATTTCTGACGTAAGTGCGTGTCAGAT
AGCCATGATGACCGCCAGTCTGTTATTCATACTGGCTTCGACGGCAACTGGCCTGCTTTTTCTTTTCCCTGATTACATCTGTGCTAAGCTCTCCCTGCCGCCAGAACTAAGTCAGTTTCTGCTG
GGGTGGCCTGTACTCGCCAGGTTTGGTAGCGCCGGGATATTTCTTTTTACTGCCTTATTCTTTGCACTGAAGAAACTGCACCGTACAGGGATGTTTGATCGGCGTGTAAGTATTGATAAAATT
GACATACTGAAAGGGGCCATTTCAACACGCCCTGCGGCCCCTTCTCTTCTCAATGTGTACATTGATGAAATCGTCTATTTTTTCGAACAGACACAATACAATGTGGTCATATTTGAGGATCTTG
ACCGTCATAATGACGGAGCCATTTTTATCAAACTCCGTGAAATAAATCAGCTTATCAATAACTGCCTCCCCACAGACAATCCTGTCAGGTTTATCTACGCTGTCAGGGATAATCTTTTCATCACA
CCAGAATCACGGACAAAATTTTTCGATTTCGTCATACCGGTGATCCCGGTGATGGACAGTGAGAATGCTTCTGAACATTTTTTGAGTAAATTTACACCTGACGAACTCAAGCAGGAAGGATT
CAAAGACTGCCTTGCCAGACTGGCTCTTTTTATTCCTGATATGCGGGTTATGCATAACATTGCGAACGAATTTCGTCTTTACCGGAATATTGTGAATAATGGAGAGGATTTAAAGCGACTTATT
TCACTGATCACCTATAAAAATCTTTGTGCTGAGGACTATCACAGGATAGATCAGAAAAAAGGGATGCTTTATAGCATTGTGTCCGAATATATATCAGGTAAATTAAGAGAAGAGTTTTGCAAAA
ATCTAAAAAACAAAATTGAAACGTCCCTAACTGAACTATCTCAACTACAAAATGAAAAAGTGGCCACTGAACATGGGTTGAGGAGTGAAATTCTGCGCTCATATATCAGTGAGATAACATCAT
CAATGTTGCATTTTAAAACTCACACGGGCACTCATTATGATTTGAAAGATGTTATAGAAAATGAAACCTCATTTCTTTCTTTGCTGAATAACCATCCGATTAGGATTCAGGTAAAGGGATATAGC
ACAACCATTGCTACAATAAATAGAAGTACAGTTGAGGTTATGTCGGAACAATATCAGGAAAGAAAAGTTATTATTCAAAAAAAGTCAGATGGTGACATTTCCCGCTTAGAGGAAATTATTAAA
AGAAGCAGATCTGAAATTCAGAATGCATCCTCGTATGATCTTGCTTTTTTCATCAATAAGATGGGCAGATCCGGTTTCGAAAGATGTATTGCAGGCTATTCAACGCCTGAACAACACGACGAA
GAAACCTTTACTGATAATGCCGGAAATATTGATTTTATTTATTTCCTTTTATCTCATGGTTATCTTTCCACTGACTATATGGCCTACCGCTCAGTATTTATGCCAGGTAGTCTGAGCACGGAAGAC
AATAACTTTATCAGGGCTGTGACGTCAGGAGTAATACCTGATGAAACGGCAAAAATGCCCCTCAGCAATATTGCTAACACCGTGGAAAAATTGCATGGACTGGGCATGCTGATGCATGACAA
TGCATGGCATCCTCAGATTCTCTGGTACCTCATGCACAATGATACGAATTCCCTGAAGACTATCATGAGGATGCAGTCAGAAGCCGGAGCCGAACAGCGGATGGTAAGACTGGCCAATGAGA
TCTTCCCCTGCTGGGAATCCACTGCGCAGCGGGACTATATCAGGTTAATGGTTGACGGGGATGGTCATCTGTCCACTATGATCCACCAGATTGGGAGCCTGAATGATACCGTAGCTGAACAA
AATCTGCTCCCGTTATTACTGTCCTTACCGGTGCTGTCCTGGGGAGCCGTCTCTCAGATAACTCGGGATGAACTACAGAGACTCATCGATTCGCAATTTAATCTCGTAACGTCCTTACCAGATA
ATTGTGCGCAGTTTTTCTGTGAAAACCTGCGGAATTCAGGTTGTCGCCTGACTAACATTCCTTTGGCCCGGAGTGACTCCGGGCAGGAAACACTGCATTCGGTGGTACAGGAAAAATTGTG
GACCTATTCCACATTGAACCTGCAAAACATTTGTTTCTCACTTTCTCATGTGTCAGAGAATAACAGTGATACCTTCCGTAAAAAGCCTGTTGCATTAATAAAATCGCTTCGCATTCCGAACCTTG
AAACATATGTTTATGAAAACATATCATCATTCATTCGTGATGTTTTTATACACTCTGAAGAAAATGAACTCATTCCTGATCTTCTTAATTCGACATTCGTTGACTGGGATGATGCAAGGTACCTGA
CAGAAAGTATGATCTTCTTACTGGAGGACATATCAGTTATCCTGAACAAAGAGAATACCGAAACCACAGGAATTTCTTATGATCAAAACCTGTATTCACTTCTGGCTCATCATAATCATATTACC
ACTTGCTGGGATAATGTTATTTCTCTACTGAGTGAAGATGCGTCACTTGCTGATTATACTTTTTGTGATTGGCTTAATTTTAATTATTGTTTATTGCCAAATGACACGTTGCCTTTGACGGATGAT
CAGTTCTCGCAATTACTTATTAAGGCTGTTACATCACCGTATATCAATAGAGAAGCGCTTGTCGTGTTAACCAGGACATTCAGGATAACGCTTATTTATGTGCCAGATAACTTACCACTGAATAA
TGCGGCGGTACTGATCGAGGAAAAATGGCTGGCCCCAACCATCAGTGTCTTTGAACAACTTTATCAGGTATTGAACGAAATGGGCGAACGGTTGACACCACTTCTTTATGATCTTATTTGTAT
CCGCCCCGCGTTGCTGAATAGGAATTACGAGCTTGTTTTGTTTGCTGATGACCAGTTTAACCGGGGGATTACCCGTTTAATTCTGAATGGCGATAAGATTTCAGACGAAGTCTGTATCAGTAT
CCTGAACTGGCTCTGGGAAAAAGATGAAGCCCTGCTCTCTGAGGCACCACTTCTCAGCCAGCAAGCCCTGATCAGGTTCTCCACCAAAATAACCGATGACCGGCAGAAGCAGGCACTGCT
GATGCAATGTCTTAAAAATGACGGTTGGCCACACAAGTTCATCAGACAGGTGCTCATGACGTTCGGGCATCAGGATTATGCGGCTTTCCTCACAGAGAGGAATTATCGTAGCATTCCCCGAA
GTGATGCCATGTGGCAACTGGCTGTCCGGCTGGGAAAAAGTGGATTTATCAGGCCGCCGAAGCTCACTCACGGTGATACACGTATCCGCATAGAGCCCTTTTTTAACGCTGAAAATGAATAC
GACTGA
>LOLKGF_00110 EF-hand domain-containing protein

ATGGATAACGAAGTGACGTTTTCACTTAGCTATGAGTTGTTGACGCAAAAAGCTGAAGAAGAAATCAGGAATATCAATCTTGACCAGGACGGAATAGGTTACATCGAGGCGCTGGCGAAAG
CCTCCGCCATTCTGAATTTCTGGTATGGTCTGGCTTTGCAGGGTTATCCGGGCTCAACAATGGATGAACATGTTGATGCCGATCGGCTGCGCCTGCACGCCCTCGTCTTTAAAAGAGAGGAC
TGA
>LOLKGF_00115 DUF3000 domain-containing protein
ATGTTGATGCCGATCGGCTGCGCCTGCACGCCCTCGTCTTTAAAAGAGAGGACTGACATCGTGACAAAGGTGATCGTCCGCCGTCCGTCGCCGCTGCAGCGACGGGTGCTGATTGTGCTGG
CAGCTCTCGATGCGAAACGTCCGGGACCGGTGGCGACGCGGGATATTGAGCGGGTACTGGAGCGGGGCGGGGATGTCCCGGTGTACGGCCCCAACCTGCGTGCCTCCTGCCGGCGTATG
GAAGCGGCAGGCTGGCTGCATACCCTCCGGGCACCTAACCTGCAACTGGCTGTCGAACTGACAGACGCCGGCCGTGAGCTGGCCGCTCCGCTCCTGGCTGCCGAGCAGGAGCGCGAGCT
GGCGGAACGGCGCGCGACGGAGATCAGGGTTCTGCCCCTGGTCCCGATCAGGCCGGTGGATACCGGTGACGCCCGGGCAGGCGATCGGCCGGTCCGGCTGGACAACATCTGGTACATG
GCATGCCGGGGCGATTATGTCATCCGCGCCGACGGAACCACCTGCCTGCAGCTGTGGAATACGGCCGGACAGGTGACGCGCCCCGAAGGCGATGCCGTGCAGGTGGCGGTGTGGCTGCA
GGCCTGCCATGATGCAGGTATTGAGGTCCGCCTGCAGATAAACGAAAGCCACGCGCCGGAAGAGGGCTGCATCTCCGGCACGGCACCGGTCGATCAGACGGAGGCCTGGTTCCGGCAGC
TGGACGCTGAACTTCAGATACTGGGGATCACCGGCCTGACAGAAACAGATCGGCAGGCTGTTGTGGTTCCCGGGGAGACGCTCCGGTCACTGCCGGCACCGGCACGGTTGCTCCATATAC
TGCGGGAAAGTGCGGAGGCGTTTCCGCTCACGGCCTCCCGGCATGAGACAGACGCAGGCGACGCGCTGGACGCCCTGCTGGCACACGCCGGATTCAGCGCCGCCCAGGCACAGGAGCT
ACGGTGGCACCGCATACGCTGGCCGCTGATGGGAGATGAAGAATTTGAGCAGCGCTACGGGAAATTTTGA
>LOLKGF_00120 Upf93-6
ATGGCCAGAATTCAGTACAACCGTGACTGGGGCAATTATCTGGAAGTGTACGACTGCGATTCGCTGACTGATATGAACGAAAAGCTGTACCAGCACTGCGCTGATACGATGGGGGCTAATTC
TCCATATATCGTCGTGGAGTATCCTGTATATCTCCGTGATATTTATTCCGTACAGGAACCAGGTAAATTAACACGTATCGGCTACGTGAGCCTGGTTTATGAAAACGACGCCGATGGTTATGTAG
CTCACCATTACACGCTCGACAAGAAGGAGCTCTCTGACGAATGGAGCGCCGTAAGATTTTATGGCGGGGAGTACGGTTTTAAAACTGAGGAAAAATGA
>LOLKGF_00125 hypothetical protein
ATGAAAATTACCGCAGATCAATTCGTAACACGTAGTGGCCGTAGAGTTCTGACCGACGATGGCCAGCAAGGTATGGGTGGGAAGCCTGGAACAGGGTCCACCACTGAGAGAAAACAGGG
TCAGGTAGCAGCGGTGATTTATGCAAACTGCGCCGAATTAGACAACAATCAGCTCGATGAGATAATAGAGTGGGTTCGCCTGTTCAAGTGCTGA
>LOLKGF_00130 SWIM-type domain-containing protein
ATGCCGATGAAACTGGTCCGCAAAAAAAATCCCAGGGGAGGCCTGTTCCGCTGGCTTACGCGTGATGAAGAATTAGCAGTAAAAAATATTTCCGGACTCAGGGACTTTACCGATCGGACAC
AATATACGCTGAACAATAAAGTCCTCCGGGAAATGCGTCAGCTTGGTGCCTGGTTACAGTGCGACTGTATTCCGGGGGATTCACCGGCAATGAACAGTGCCAATCTGTACAGGGATACCGG
AACCCTTTTTCTGTCCGGATTTAACCATGAGCATGCTGCTGACTGTCCTATGTACCGCCCGTTCAGTGGTGATGCCGGAGCCACCTCTTCCGGAACACGAAAACATGCCAGTAGCCGCCGGA
TTAACTACAGAAATTTTCTGCCCCCGGATGATACTGAAGCAACAACCAGGAAACCTGGACATCCGGAACCCCGGGGAGACGATCGAACCCGACGAACACGCCGATCGAGGATTGCCCGTC
TTCTGCTCTCTCTTATTGAAGATGCCGGCCTTAACCAGCTGGACACACTCTCTCCTATTCCTTCCAGGAGTGTCAGGGACTCCCTGAATGCCATGCAACTTGTAACACAAAATCAGGAGTTTAT
TCGCGGACGTCAGTTGTCAGAAATTGTTCGTTTTCAGCCCGGCATGAGTGCCCGGGGGCGCGAAAAACTGATGAAAGAACTGGAGCACCCGGACATGCACTGGCCCGCCGGACGAACCC
GGATATTCTTCCAGATTTTTATGTCCGCACATGTCAGCCGTGATGAAGCTGAGTTTCGCTGGCCGGGCGGGGCTGTGGTGTTCCGGCCAGAACGCGGAATCAGCATCAATGGAGAGTCGCT
GGAGGGAAGGCGTCCACCCTACTGGGTTATTCTGTCGTTTCGCCGGGGAACTGACGGAGATATTATCTGTAGCGAAGGATATGCTCATGCACTCTTCCGTATGGGGTGTCCGGTACCAGTGG
ACAGCGAACTTGAGCGTGGTACGCTGGCTGGTCTGTCTGTTGTTTCAGAATGGCTTAAAAATAAAACGGAAGCACCGGCACTGAGTCTGGAAAAACCGTTATTTGATATTGAGGTCTCTACC
GAGGGTGAAAAAGGGTATGTACTGCCTGATTTTATCATCACGGCCAGAATGAATGACGGCAATGAATATAAAGTGGTTATTGAAACGATGGGATACGCTGACGATGACTATTGCGAGAGAAA
AGCCGAACAGCATAAGGGAATGAGACAGATTGGTATTCTCCAGACCGATCCCCCCAGGTGGCCACAGGAAATTGAGACTCCTTTCGAAAAGCATATCTTTGGCGTTTTTCTTCACCTCAATA
AGTTTTTTAAAAATAATAGCAACACATAA
>LOLKGF_00135 ProQ domain-containing protein
ATGACGGCAGGTAAACAAAAAAATACACCGGTCATTGTGATGAAGAAAAAACGCCACATTATTTCTCCTCCACCAACGGAGGAACATGTAATCACTCCTCCGCCTCCGGAGCTTCCTTCCGG
GACTGTGGAAAACGAAATGCCGGCACATCAGGCGAAAAAAAATAAAAAATGGACGCCGGAATTCATCAACACCAGTCTCGAAAAGGTTAAAGCACTCTTTCCCCTGTTGCGGGCTGAAGA
AGGCGGTTTCCGTCCGCTGAAAATTGGCATCACCAGGGATGTTACCGACTTTATTGCACAGAACCCCGACGCCGGTTTAACCCTCCCGGAATGGCAGTGTGCTGCCCGGATTATCACCCGCA
GGTGGAAATACCTTGAGCGAATTTCTGTTCCCGGTGCTTTACGGTATGGCATCGATGGATTGCCTGCCGGTGTCGTTTCTGAGCATGAAGCTCAGCATGCCCGCGCCTTTCTGGCATCAAAG
CTGGCCAGCAAAAACAAAAATAATGGGTCGAACGACAAATCAGCAACATAA
>LOLKGF_00140 hypothetical protein
ATGATGGATGCTGTAACTAAACTACTGAGTGGCGTTATGTCCCGTCTGGCTTTGCAGGGCGATGGCCCACTGCGTGAACCATCACCCCGCCAGCAGGGTATTTTATGTGCAGTTGTCAACCAC
TGA
>LOLKGF_00145 Replication initiation protein
TTGTCTCCCGAAGACCAGATGAAATTCTGGGCTGAATATGAAGCGGGGAGGACGACGTCTTTCCTGGTCGAGCCGGAAGGAAAGCGTACCCGGCGTTGCCGGGGGGAGCACTCCACCAA
ACCTAAATGCGAAAATCCATCCTGGTTCCGCCCTGCGCACTATAAAAAGCTCGGGGGCCAGCTGGGCTATGCCTACAACCGTCTGGTGAAAAAAGACCCGGTCACGGGAGAATGCAGCCTG
CGCATGCATATGTCCCTGCACCCCCTGTACGTGAAGGAGCGTAAACGGGCGGGACGGCAGTATGCTTTCCGGCCGGAGAAGCGTCAGTTGCTCGATGCCATCTGGCCGGTGCTGGTCTCCT
TCTGTGATGCCGGCATGCACACGGTCGGCATGTCCGTGTCGCGCCTGGCAAAAGAAATCAGCCCCAAAGACAGTAAAGGTAACGTCATTCCGGAACTGGCAGTCACGGTCTCCCGTCTGTC
GCGCCTGCTGGCCGAACAGGTGCGTTTCGGTACGCTGGGCCTGTCAGAAGAAACCCTGTGGGACCGGGAAAACCGCCAGCGCCTGCCGCGCTACGTCTGGATAACCCCGGCCGGCTGGC
AGATGCTGGGCGTCGACATGATGAAACTGAAGGCGCAGCAGGAAGAACGGCTGCGTGAAAGTGAGATTCGTCGGCAACTCATCAGCGAGGGCGTTCTGGGCGAGGACGAAGATATTTC
AGTGCATTCTGCGAGAAAACGCTGGTACCTGCAGCGCAGCCAGGAGGCACTGAAATATCGTCGTAAGAAAGCGGCCGCCCGTAAACGGGCCAACCGGCTGGCAGAACTGCCGCGCGACA
GACAGATACATGAAATGTCACAGCATATCCTGAAGTGTATGCCCCCGGATGAGGCGTACTGGTGCACGCCTGATCGTCTCGAGCAGCTGGCTATCCGCGAACTGTACCAGATGGAACTGGTG
CTGGCCACGCCGCCTCCGCATTAG
>LOLKGF_00150 C4 antisense RNA
TTCAATGGTAGCTCAGGCGGGGCAGGCTTTTCGCCTGGCCGGTTTCCGATGACGCCGGTATTCCTACCCCCGTCTGGGCTATCGCCACG
>LOLKGF_00155 Conjugal transfer protein TraA
ATGTCGCCTCAGTTATTTCAAAAAATGGCATCAATCAGCCTGCTCGAAGATATGTTCCCCGAACTCTCTGGCAATCAGCTGAAGGTATGTATTCTTTACGCAATGGGCGCTTCTTACGATGCGA
TTGCGCAAAACTGTCATATTTCTACTGAAACGGCACGCACCTACTTAAAACGAAGCATCAGGAAATTAAACCTGGAAGGTTATGATGCTTTACGATCCGCTGTTCTGATGCGGGCTTTCTTTC
TCATAATTAGTAAACGATAA
>LOLKGF_00160 NGN domain-containing protein
ATGCCTGTCAGAATTGCGGTATGGTCAGACGAGGGCTGGCCGCCGCTTCAGACATATGATTTAGCTTGTTTGCCAGATAAACGCAGAGTCATCCCGGATGGATCGATTCTGCGCGGAGCGTT
GAAATTCATGAATATTGAAAGCCTGATAAACCGGCACTGGTATCTTGCACAATATATTACTACCGGTAAAAACAGGGAGTCCTTATTTTCATGGCTAAGCAGCCAACATGTTGTGCCATGGACT
CCATTAATTAAAAGAAAAGTCAGACGGGCTGACTCACCCTCATGTTACCGCCAACGTGTACATGCCATATTCCCTGGATATTTCTTTATGCGTGCTGATTTCAATATACAGTCCGCATCCAGATT
ACGGCAACACAGTGCATTTATAGATTTCGTAAAATTTGACGGGACAATTAAGCCTGTCAACAATGATATTGTTGATGGGTTAATGAAGATATATCCGGACCCTGTTTTAAATCCAGGAGCCAG
GGAAGAGCTGGATGCTGCGTCCAGTATCTGGCTGACAAAAGCACAGTACAAGTATCTCCTCAGAATGGAAAATAATCCCCTGCCTGAGTCGAGGATATCCCTGCTTCTTGAACTTGTATCAAA
TGCGGCAAATCACGGTTTTATGGAGCGCCTCCCATAG
>LOLKGF_00165 DUF2913 domain-containing protein
ATGAGTACTCATGAACACCCGGTGCGCCAGTTACTCTGGTGTGCACTGACAGCCCTGCGCACGGCACAGGAAGAACATCACATTCATTCTGAAGCCCGACTGCGTCGATATCTCCGGGAATG
GTTACAGGGGGCCGCGAAACTCCATCAGTTCCGGAGTATCCCGGAAGAAATCATGACACTAAAAAGGCTCCTTGATGAAGACAGACACGTTTCCATAATCAGCACGCTCAATGCCCTGTTTA
TGAGTTCGGTTTCTGCTGATAACTGTGATCTGTTTCGATTCCGTGCGGCCCTGAGCGCAATTTCCCGCAAAGGCTGGCGCTGTGGCGTTTGTCCATGGCCAGAACAGGTTTTTAGCGAGAC
GATTACCCAGGCCTGTACCGGACGGGAAAGCCTGCTGCAGTTGACACGTACCGAAGAGTGCTTCTCATCCACCGGTAAAATGACCGCGCCTGTTACTCTGCAACTTATCTTCCCACTGGATA

ACGATAATACGACTGCTGAAGATCTATTTTCTAACGAGAGGTTTCAGGTAGTACATGGACGCGAAAACCAGTATGGTATTGCCGGAACAGAAATATTAGTCAGAACATTGTTTCTTGGACTAC
CGTCCCTGCCTGAAGAATTATGGGCTCTCCACCAAAGCCCATTGATGAGTGATTTCAGGATGTCAGAAGAAAAGGCTTCTGTTATTCACTGA
>LOLKGF_00170 Pili assembly chaperone
ATGCAAAAAAAAATACATTCCCTTTTGCATTTGGATTATCTGGAGCCTTATTTGGCTATGCTTTTACTATCCTGTGCTACGTTACAGCTACTTTTATAGCGAGTACACAGGATCTGTTTATCTTTCTT
CAGTAATATTTATCGTTGCAGGAACCTGTCTGGCCCCTCTTACATTTAGAGCTGGTCTGAAGATTATGCGTCGAATATCTGATAATCCTTTCAGTTTCTTTATAAAAAAAGCAGGTAAAAAAGG
CCAGTATATTATCCATCTCAGTCCTTATGCTGACACACGTACACATGAGGAGATAAGCAAGCACTGGAGTATATTGATCGAGTTAATTAGCAATGCCATGACTCATCATGAAACCGTCATAATGC
AGTCACATTTATTGACTCCAGCCAGAATTAAAAAGTTAAAGAACAAACTGGAATCGCACGGGGTTTTTACATCTTTTAAAGTTTATCGGAGAAAAACCACTATAAATGAAAAATTGTCAGTTC
CGTTAATATATTTATTGCTTCACTGGTCATTACCTTTACTTCCTTCAGAAAGTTCTACAGTAATTATATCAGCAAAGTGA
>LOLKGF_00175 PilI type IV pilus biogenesis protein
ATGATGAGTATTTCTGCTCCATCTTATTCAGCATTAAGAATTATTGTCATCACAAATAATTGTGAACAACGAATTCATAAATACAAATCAGATGAGTATTTAATGGATTATCTGCAGAGTTTTTGC
ATGCCAGAAAACTGCATGGTGTGTGTATTTGAACGACAACGACCATTATTCAAACTTGAGCGCGTGCCGGGCAGCACAAATCAGTGGAGCCAGGTAGAAATTCATAAACCTCGTCGGCTGC
GTTCATATCGTCTTCATCAGCATTAA
>LOLKGF_00180 Type IVB thin prepilin lipoprotein
ATGAAAAAAAGCTATCTTTCATTCATTCTGCCGTCGTTATTATCCGGCTGCACAGGTATTTCAGGCAGCAATAATATAACTGATGCAGACACACCGGCGCTGGTTTTTGTCGATGGACAAATAA
GTGACAGTGCAGCCGTACTGGCTCACACCCAGCAAAGACTGTCAACGCCAGCAGTGGCTACGACACCACAAACGGTGTCTTCACCGGGCACAATTAAAACAATGGAATCATTCAGTGAATC
ACGTCCGCTACCCTCTCCATCGTTCAGCCAGAACTTCTCCAGACTTATTATGACAGGGACGGCAGGTGCTGTACCGGTCACTTTTTTGCCGACAGTTCGTAATCGTACAGTCAGTCAGTGGAT
CAAAACGTTATTGCCTTCTGACTGGCAGTTCAAACAGGAAAATGCCGCAACACCAAAACTTAATACCCGGTTGGCCTCATGGTCCGCCAATGACCAGTGGACACGTTCTCTGAATCGCTTAC
TGGAGGAACAACATCTGTGGGGACATCTGAGCTGGGCAGACAAAACACTGATTGTAACCACATCATCTTCTGTACCTGCGGTCGACAGTTCTGGCGTGCAAATATCGCCTCCATCCGTAACT
GTGATAACACCTGAGATTAACCAGTCCTCCCGGATTCACTCTGACAACCAGATAAAACCTCGGAATCCTTTCCACGGCAGTACTAAAACAAATACAGACGAAACCGCAGTTCTCCCCGGAAA
TAAGCCTGCTCAGTTAATTACGGGTACACCGGTAACCCCTGTCGCTGAGGGCAAGGAATGGCGTGCAGCAGCAGGGACCACTCTGAAAGAAAACATTACCAGATGGGCCGAGGAGACAA
AATGTGAAAGCATTCCATCTTCCCACTGGATGGTGTTATGGCCATCATCCGTTAAAGATTATCGCCTGGATGCCCCTCTTTTTTTCAGAGGCTCTTTTGAATCTGTCATTGGAGAGGTGTTTGA
ACTCTATCGCACGGCTCAGACCCCCCTGTATGCTCAGGGCTCCCGCATACAATGTATCGTGACCGTTTCGGACACCAGGGACGGGCAATAA
>LOLKGF_00185 PilM
ATGGGCTGGCTGGTTATGGTCGCCGGCCTGACCATTCTGATTATCATCGGGGACTTTCAGAATCAACAACTGGCGGCAACAACAAATGCCCAACAACAAGCAAGCGGTTCTGTATGGGCAA
GCCAGATGTTGATGATTGCCAACAGAATTAATGATATCAGATATGTATCAGGCCAGCAGAACGGCACAATTTCCCCGGATCAACTGGCACTCCCTTTCACACCTGATTCCCGTATCAGGCATCA
GTTACATCAGGGTCGATTATGGATCTGGATGCCGGAACTGCCCGGACTTATTGATGCACTTCGTTCCAGAAGCCGGGGATCTGCACTTATCGGTATTTTTCAACACGGACAACTCACCTGGTT
ATCAGGAACTGCTCCAGGCCTGACACCTCCGGCAGGCATTACAGAAGACTCCGTCGTCTACGTTAACTAA
>LOLKGF_00190 Secretin-N-2 domain-containing protein
ATGAAAAAAATTGCACCAGCGCTCCATGAAACTGGCGGCGTTACCCTGCATGATCGCCCTCGTACTGACAATGTCTGGATGTACCTTCAGCGAAATAAACAAAATGCAGAAAAAGGCCCAGA
CAGACTCAGCCAGCGCCAGAGAAAAGATGGCTGCGCTTTCAGAACACAAATCACAGGCAATGACCTGGCTTGATCGCCAGTGGATTAATCCGGTTCCACTCTCCCGTATAACCCGTGAGAA
AAAAGCGATCGCACCTGACTGTTACATTACCCAGGCCAGAAAAGGTGAAATCACACTTCAGGAACTGGGACAGCGTATTACTGCCGTCTGCGGGCTACCTGTCATCATTACTCCTGATGCCA
CATCTGCAACCCTGGAAGGTGGGGTAACCCGTCAGATGACAGGGACTCTTCCGACACCGGACGAAAATGGACGTCTCCCGCTGAACAGCCCTGGCTATACTTCCCTGTCAGCTTCCAGCCA
GCCACTTACGCTAAACAATCTGATGTGGCAGGGGGATATCAACGGACTTCTGGATCTGATGGCCAGTCGGAGCGGTCTGTACTGGCGTATGGATAATGGCCGTATTGTCTTCTACCTGACCG
AAACCAGAACATACCCGCTTAATATGCTGAATACCAAAACCAGTAGCAGCTCCAGTGTCAGCTCTGGTTCAACCAGCACAATGGGTGCTACCGGCGGCCAGGATAACTCGGCATCCGGCGAT
GCGACTTCATCCCAGAGTACCACCGTTGGACAGGAATATGACCTGTACGAGGACATCAGGAAGACAATAGAAGCGATGCTAACGCCTGAAAAGGGACGTTACTGGTTATCAGCATCCAGCT
CAACATTGACCGTAACAGATACTCCGGCTGTTCAGGAAGCCGTGGCCAGATATGTTGATGAACAAAACAGCATTATGAACCGGCAGGTTTCGCTGAATGTACAGGTTCTGAGTGTGAGCCAT
ACCAGAAACGAGCAGTTCGGTCTTGACTGGAATCTCGTTTATAAATCACTGCACTCTGCAGGCGCAACACTCAACAATGCCAGTGGTGATATTACGGGTGCCACTTCTGCAGGCGTATCAAT
TCTCGATACCGCAACAGGGAATGCAGCCAGGTTTAGTGGTTCCAGCCTCCTGATCAAGGCATTAAGTGAACAGGGGGATGTTAGTGTCGTTACGTCTCAGGAAAGTACCGTCACCAATCTG
ACACCGGTTCCCATCCAGATGGCAGATCAGACTGTCTACGTTGCACAATCGGCCACAACAACGACAACAGATGTCGGCGCCACGACGACATTAACACCCGGGATGATCACCACTGGGTTCA
ATATGACCCTTCTGCCGTTAATTCAGAAAACAGGCAATCTCCAGCTGCAGATGAATTTTAACCTGTCAGATCCTCCGACAATCAGAAGTTTTACATCAAAAGACGGCAACAGCTATATCGAAAT
GCCCTATACCAAGCTGCGATCGTTAAGCCAGAAGGTCAATCTGAAAGAAGGGCAGTCTCTCGTTGTTACGGGCTTTGACCAGAACAATACGACCACACGAAAAGCCGGTACATTCACCCCG
TCTAATCCACTGTTTGGTGGTTCGCAGAACGGGAAAAATGAACGCAGCACTCTGGTCATTATTATCACCCCAGCATTCCCGTCAGGAGGCCGTAATGGCTGA
>LOLKGF_00195 hypothetical protein
ATGCCGGAACGTCACCGCTATTTTTCTCTTGCACTGGGTTTTTGTTCGTTGGTCCGTAATGGTTACGGTATTTTCAGATATGACAATGAAAAACTTTTATTTCTGGCCTCAGTAGATGGACAGC
CTGCAGTTATGGCTGACCAGTCAGGAAGTCCGGAGGACATCTCTAAGAAGATAAACCTTTTCCTTGTGATGAACGAGGAGCCCACTGAAAAATGGCAGGTTATTTCTTCTTTGGAAAATCCA
CATAACTGGCAGACCATTATCGAGAAATTGTCACCTGCGGACATACGTCACTGTAAATTAACAGTTGATACCCGTCAGCGATTTATACTACCCGTTGCTCTGTTGTCTGCTGTGGCTTGTGCCG
GTGTGATTTTCTGGCTGACACAGCCAGAACCTGATACTGGCCCCACCGCAGAAGAGATTGCAGCACGGGCCAGACTGCAGTTCAGTAAACCGACCATACCACCTGAGCTACCACACCCATG
GGCCACACAACCCCTAATTAGCGATTTACTAAAGGCCTGTGCAGACCTGAGAAAACCGTCGCCGGTCGCACTGGGCGGATGGAAGCTGACAGAAGGCTTCTGCACGCCGGAGTCATTCAC
TCTGGTTTACGAACGGCAGCCCGGAGGAACAGTCCAGGGCTTTCTGACCAGAAGCAAAGAGGTTTTTGGCATTATTCCTGAATTTAACCTCAAAGATGGTGCCCGGCTGGCTTCTGTCCCC
CGTCCACTACCTGCTTTACTCCACAGAGATGAAGCTGTACCGGCACCTTCTGAACAGCTGATGCGTGTGCTCACATGGTTTCAGGAAAGGCAGATAACGCCGTCTGTTAACGAAACAGCAAT
ACCAGAACCGCTACCAGGTAATGACGGCGAACCCGCACCTGTTCAGAATTGGAGAGAATATCAGTTCAGCGTATCGACACCGTTAAATCCTGATGAACTATTCCGTGTTTTCCAGGATACAG
GGGTTCGGATCAGCAGCATTCATTTTAAATTAAATGGCGGTACTTTCAGCTATACCACAGAAGGTCACGTTTATGCATCTAAGTAA
>LOLKGF_00200 Type IV pilus biogenesis protein PilP
ATGCATCTAAGTAAATTACTTATAATTACCTCACTTCTTCTGCTTCCTGAATTATCCTCCGCAACCACCCAACCACTGGTTACCATTGGAGAGCTGGAAGCCCAGCAAAACCAGAATATATTGTT
ACAGGCAAAAGTACAGGGTGCACAATTACAGAAACAACTGGATGGATCTGATGTTGATTCGTCCATGACAGTACCTCCGGATGCTGGTCAGCTCACATCCTCCCTGCCGTCTGTATCCGGCC
CGGCTCAGCGTAGACCCCCAACAGAATTACCTGTTATCCTGGAAATTAATGGCAAAGACAAGCGACTTCATGCCGTTCTCCGACTGGCCGACGGACGACAGACCCGTGTTACAACCGGAAG
CTCGATCCCCGGTACAAAAGTAACGGTTAAATCAATCTCTCTGTCGGGCGTGACGCTGAGCGACGGTACAACGCTGACCTTCTGA
>LOLKGF_00205 ATP-binding protein
ATGCATGAATATAATATAAAGGACGCACTCCTTTTTATAACTAAAGAGGATGCTTATGAAATTCTTATTGAAAACAATCAGCGAACACGGCCAGATATACAAAGTAATGTACAGGCATTAATGCA
GAAATATCCGGGAGCTACAACCAGACTGGTCAGCTTATCGGAAATGCAGGAAGCAGTTCAGAATGAAAAAAACAGTATGCCCAGAGAACGAGTTATTCATTTGCGTGATCTGGGAGACGTC
AGCGAAAGTGAAAAAAAAGATTCTCAATTACTTTGAAGTGGCCAGAGAACTCGGTGCATCAGATATTCACTTTTTAATCTCCGAATCACTTTTCAAGGTAAGAATGCGTATATTCGGTGAACTC
CAGACAGTTGATGAGGATCAGCCAACCCTGGGGTATTCACTTTGTGCGACAGCCATTCTTTCAATAGCAGATGTCACCGAGACCAGCTTCTTCCCACAGCGAGAGCAGGACGCACGACTGT
CACCTCAACTGATGCGAAAAATTGGAATATTTGGCGCGCGATACAGCCACAGGCCAACAGGGGACGGGCTGATTGCTGTTATGCGCCTGATACCTGATGACGGCGACAAGGTTCCCACTTT
CACTCAACTGGGTTTTCTCCCTGAACAAATCAGGTTGCTGAACATCATGCTGTGCCGTCCGGAGGGAAAAATTATCCTGTCAGGGCCGACTGGTTCTGGTAAATCAACAACATTACGCAGTG
CCTGCCGCGTGTATCTGGATGAGAACCCGGGGCGGCATCTGCTGACTATCGAAGACCCCCTGGAAGGGCAAATTTTGGGCGCGACACAAACCCCTATTATCTGTGACAAATCCGACGAGGA
TGCCGTTAAGCTGGCATGGAGCCGGGCAATCTCATCTGCAATGCGACTTGACCCGGATGCCATCATGGAAGGGGAAATGCGCGATCTCGTCTCAATGATGTCAACAACCTATGCCGCACAAA
CCGGCCATCTGGTTCTGAGCACGTTGCATACAAACTCTGCGCTGGGTATTCCCGAACGCATGATTACGATGGGTATGAATGCTGACCTGATTTGTGATGCACAACTACTGATTGGAATGATAA
GCCAACGCCTTGTGCCAACCCTTTGCCCTTCCTGCCGTATTCCATGGGCAAAGCGTATTCCTGAACTTAGCGATAATACCCGAACTTACCTTGAGCGCTATTGCAACAAAGAATCGATCTGCA
GTACAGAAAATATCTGGTTTCGTAATCACGATGGCTGTCCTGATTGTAATCGCGACGTCATTATTAACGGAAGAAAACGCGGTGTAGTTGGTAAAGGTCTGACTGGCAGAACGGTAATCGCT

GAGGTGATAGAACCTGATAACCGTTTGTTTCATATTCTCAAAACGCACGGCAAGGTTGCTGCCCGTAAATACTGGCTGGAACGTATGAACGGGATCAGTCGTGTTGATCACCTGCTGCGTAG
TATTAATGCAGGTCTTGTGGACCCCCTGGAAGCCAACAGGATTATTCCTCTCGATGAGGATGAACGTCTCAATATTGACGATGTTTAA
>LOLKGF_00210 Conjugative transfer inner membrane protein PilR
ATGCGTGAAATGAATTTCGGTCAACGGCTGAGACGTTTCATCGTCAGAAAAACATTCACTGCGCCCTACCGGGTACAATTCTATGAAGCCCTGCGCTTTCTGCTTGAAAATAAACAGCCATTA
AAATCAGCACTGGAGCAGATGCGGGACGCCTGGACCGATTTCGGTCGTCACTGGCATCCTTTTGCTGAACTCGCCACGGACTGTATTGAATCCCTGCGGGAGAATACAGAACAGAATTCAC
TGGAATATACTCTCAGTTTGTGGGTACCACAGGAAGAAGCCGCTGTAATAAGTGCGGGGATCAGGAGTGGCTCCATTGTTGATGCGCTCAAATTTGCCACTACACTGACAGATGCAAGGAA
ACAAATCCACAAGGCTATATGGCAAATGGCCATTTATCCGGCAGCACTGTTAATTATGATGAGTGGCACATTATATGTTCTGAATACAGAATTAATTCCCGTGCTCAGCAAAATAAGCCCTCCG
GATTCATGGAGTGGAGCACTTGGATTCCTTTATGGTTTATCTGGTTTTATCGATAACTATGGCGTGATCTGTGCAATTCTTTTTATAGTGTTCATCGTTCTTATATCATGGTCACTGGCTAACTGG
AGTCGACCGGATAGTATTCGTTCTTTTGCAGATAACCTGATGCCATGGAGTATTTATCAGGATATTCAGGGCGCAACGTTCCTGCTTAATATGGCAGCACTCCTGCAAGCCAAAATGACCACTC
TCAATTCATTGACCACCCTGCAGGAATTTGCTTCACCTTGGTTAAGTGTTCGTCTTGACAGTATCATTTACCGTGTACGCTTGGGTGAACACCTGGGACTGGCTTTGCGTCAATGCGGTTACC
AGTTCCCGTCAAGGGAAGCGGCCAACTTCCTGTCGTTGCTACAGGGTGACGGCGCAACAGACCTTATCAGCAATTATGGGCAACGCTGGCTTGTACAAACACTGGAGCGTGTAAACAAAA
GAGCAGTTATTGTCCGTTTAATTATGCTGGTATTTCTTGTCATGTCTCTCCTGTTACTGGTTCTGGCCATAATGGATATTCAGTCCATTGGTGACAGTAGCATGGGCAATATCTGA
>LOLKGF_00215 Pilus assembly protein PilX
ATGTTTATTCAAAATTTGAATGGTAATTCAATGAAAAAAAACGGAAACAACAACGTACCTCATGCAAAAGGATGGGGGATTATGGAACAGGGCGCAATTGCACTTATAGTCATTGTTGTAATT
GCGATTGTTCTTGGTGGTTTATATATGCTTCGTTCAAGAACCAGCGTTGCAAATGAAAGCGCAAATATACAAACCATCATTACAAGTACCCAGGGACTGTTAAAAGGCAGCGATGGATATACC
TTTACCAGCGCTGCAAAAATGACAGGAGCTCTTATTCAAATGGGAGGGGTTCCCAAAAGTATGACCGTTCGAGGGACGCCATCATCCGGAACAGCAACTCTTTATAATTCCTGGGGTGGTG
ATGTGACTGTCGCTCCGGCGTCAACTTCCGGATTTAATAATGGTTTTTCAGTTACGTATGAAAAAGTACCGCAGGATGCCTGCATCCAGATAGCCACTCAGATAAGTCGTTCCGGGCTGGCTA
ACGGCATCACGCTGAACAGTACCGCCCATAATGACGGTAAAGTCACGACTGAACAGGCCAGTGCGCAGTGTACGGCTGACAACGGCAGCACCGGTACCAACAAACTGATTTTCACTATTAA
TGGTTAA
>LOLKGF_00220 Lytic transglycosidase pilt
ATGTCCGGGGGTAAAAAGCCCCTCAGACTTATTTCATCCATCCTGTTTATTACTCTTCTGTCCCCCGCGCGGGCTTCATACGTTGCTCCTTCACAAATCTGGACTAACAAGTGGGACAATTGTT
TTGCAGCTGCTGGCGCACGCTATCAGATTGAACCGTTATTGTTAAAAGCTATTTCTGCCGGAGAGTCATCACTGCAGCCAGGCGCAATTAACATAAACAAAGACAGAAAAACAGGAAAAGC
AAGAAGTACGGATTATGGGTTGATGCAGATAAATTCAACCCATATTCCAAAACTTGTTAAAATGGGTGTAATTAAAAAGTCTGAGGAATTAATTACCAGACCATGCCTGAATATTCAGATAGGT
AGCTGGATATTAGCAAGGCATTTCCAGATATGCGGTATCAGCTGGAATTGCCTGGGTTCATATAATGCCGGATTCCGCAAGGACAGGCACGAAACGAGGGAGCAATATGCGAATAAAATATG
GCGCATCTATCGTGATATGAAGGGGATATGTCTGCCCGGACAGGGAGGCAGACAATGCACACAATCATAA
>LOLKGF_00225 IncI1 conjugal transfer prepilin peptidase PilU
ATGCACACAATCATAAGCATTAGTTTTTCCCCGTTTTACCTGTTACCGTTAATACTCATATTTTCAATAGTTGGGCGAACCCTGTTATTCAGGGTCCGTCATTTTTTTTCATACACAAACTATGTGT
GGCAGGAAACTCACCCTGCCGTTCTTTCAGGTATCTGGCTGTATGGTATCACTGGAGGGGTAATTGTTTTATCGTCATCGACACCGATCTGCCGTATTCATGCCATTATTATTCTGTCATTTATGC
TGCAAATGGCAGTCACCGATGCTGTAAGTGGCTTATTACCAGCTACATTCACCCGAAGGTTTTTGATTGCCGGCGTTCTTATTAATATTACAACCGACACATGGTGGACGCGTACAACCGAAA
CGCTTATCGCTGCAATTATACTGTTTTGTTTTCACATAATTCTCAATCGTCGGCGTCCTTATATTGGCACAGGCGATTTGTGGCTTATAGCAGGTATTACGGCATGGTCAGGGCTGTATAACGCT
ATCTGGAGCGTTTTGCTAGGCGTTGGGGGATTCGTGTTATGGCATTCAACCTGGCGCATTAAAAGACACATAGAAGGCCCTCTTGGACCCTGGATCTGTATCGGCCATGTTCTCCTGCTACTG
GATAATCTCTACCAACCACTATGGGTAATCTGA
>LOLKGF_00230 Shufflon protein B
ATGAAAAAACACGATCGGGGCTGGGCCTCCCTGGAAACCGGCGCAGCTTTACTAATTGTCATGTCAATAATCGCCTGGGGCGCAGGCATGTGGAAAGATTACATTGAAATGAAGAGCTGGC
AAACGGAGGCTCGTCTGGCCAGCAACTGGGCCAGTGCAGCCCGTTCCTACATTGGAAAAAACTATGCCAGCCTCCAGGCCACCAGTTCCACGACCACACCTGCTGTCATTACAACAACCAT
GCTGAAAAACACAGGTTTTCTGGCCAGCGGTTTTACCGAAACCAACAGTGAAGGGCAGCGGCTACAGGCCTTCGTCGTTCGAAACGCCCAAAATCCTGAGCTACTGCAGGCGCTGGTTGT
GTCCGCCGGTGGAAATGCTCTGCCAACAAAAGCCATTATTCAGATAGCAAAAGATATTACAACTGGTCTTGGTGGATATATTGAGGACGGGAATACAGCGACTGGCGCATTGCGCTCCTGGA
AAATTGCTTTAAGCAATTATGGAGCAAAAAGCAGTAACGGTCATATTGCAATTTTGTTGACAACAGATGAACTTAGCGGCGCTGCAGAAGACACAGATCGCCTATATCGATTTCAGGTAAATG
GTCACCCTGACTTAAATAAAATGCACACCTCAATTGATATGGGTTCAAATAATCTGAATAATGCTGGCTCATTAAATGCCCAGACAGGTAATTTCAGCGGCACCGTTAATGGTGCAGATGGTAA
TTTTAGTGGTGTTGTTAAAGGGAGCAGCGGAAACTTTGATGTCAATGTAACAGCCGGTGGCGATATAAGGAGTAATAGTGGATGGTTGATAACACGCAGTGGTAAAGGCTGGTTAAATGAA
AGCCACGGAGGGGGTTTTTATATGTCAGACGACTCATGGGTACGCAGTATTAATAACAAAGGTATTTACACCGGCGGCCAGTTAAAGGGCGGTTCAGTCCGGTCTGACAGCGACCTTTCTG
CTGGGGGGATATTAAAGCTCGACCAAGTGAACGTTGTAGGCGCATGGTGTCCACAGAACGGGGCGATAAGTCACGACAGCACTGGTGGAATACTTTCGTGCCAATCCGGTGCGTGGCAAC
TTTCAGGTAAAACATATTGGCGTGTCGGTGGAACATTTCAGGTATGGCCCGGGCAAACAAGAGATTTGGGGCGTTTTAAGTTGTGCATCAACACTTACCGGATTGATGGACGTGAAATGGC
ATTAACACAACTGATTCCTACTGACAATCCGGATGCCAATGGCAACATGAACTGGCGTGCATATAACGGTACGCAATACGCATCTTATTATATGGGTATCCATTGTTTTATCTAA
>LOLKGF_00235 Conjugal transfer protein TraF
ATGATGAAGAAAAATCAAATTACCCGCACACTGATTGCATCAGCTGTATTATTTGCCTTTAATGCATCTGCTGCTACTTCATATTTTGAAGCTCGAAACGATGCAATGGGAGGAACGGGTGTTG
CATCATCCCATTATGGTGTGGCTCCTTTGGCAAACCCGGCACTTCTGACAAAGCATGACAGCAGCGATGATTTCAGCCTGCTTTTGCCATCCGTCGGAGCACAACTTTCCGATCCAGATAATAT
TTCAAACAAAGCGGACGATATTAAAGCTGACTGGGACGCTTTTGATCGTGCTGTAGACAGCAATTACGGCGTGAAGCAGGCTGCCGGGAATCTTAAGCGTCGCCTGCAGGAGTTTCGGAA
TATCCACGCTGATGCCCAGGCAGGTGTTTCAGCTGTAGCCGCCATGCCTAATGACACGTTGCCGTTCGCTCTCATGATCAAATCATATGGCTCTGCAAATGTGGATGGAAAGGTCAGCGACGC
CGATCTGGACTATCTGGAAAAAGTCGCCAACGGCACAATCACGAATGTTGATAAAAACGCCCTGACTTCCCGCGCATATGGTCGTGCTGCCGTCATCTCTGATATCGGAATCTCCTTCGCTAA
GGAGCTGGAAACCGCAGGACAGAAATGGTCCCTTGGCCTCACACCCAAATACCAGCGCGTGGATCTATTTAACTACAATGTCACGGTTCAGGACTACGATAAAAAATGACTTTGACAGTGAC
CAGTACCACAGTACCAAAAACGGATTTAACGCGGATATCGGCGCATATACCGACCTTAATTCAAACTGGACAGTTGGTCTGGTTGTCCAGAACATCGTGCCACGCAGCATTGATTCCAAAGA
GGTGAATGGTGTGACTGAAACCTTTAAAATCCGTCCGCAGGCCACTACTGGTGTGTCCTGGCACAACAGTCTTTTCACTACCGCACTGGACGTGGATCTGACTCCCGCCAGTGGATTTACCT
CCGACAATAAACGCCAGTTTGCCAGCGTAGGCGCCGAGTTTAATGCCTGGAAATGGGCGCAACTACGTGCCGGTTATCGTCAGAATATGGCATCAGACAGTGGCAGCGCATTTACTGCAGG
TGTGGGTATTTCTCCGTTTGATGTTGTTCATTTAGATGTCTCTGGACTGATTGGTACCGATCATGATTACGGGGCCATCGCTCAGCTACAGTTTACATTCTGA
>LOLKGF_00240 hypothetical protein
ATGAACCGTATTATACCTGCGGTGTGTCTTGTCGCCCTTCTGCTGGCTGGCTGCAATACCTTCCCCGAAGCCACATCCCATAGCCGTAATTATGGCGCTGCCTCTGAAGTTGCTGTTACTCGCC
ATAAACAATACAGTCTACAGGATCAGGGAGTATTCTCCGGTCAGTTAACTTCAGCACCGACAACACTACTCACGGCCAACAGTGACCGTTTATCATTCGACTGGGAGGGCGATGCTATTGAG
CTGCTGAATGAGCTGGCCAGAATTCGTGGCATGCAGTTTAACTATAGCGGTGTTCGCCTGCCGTTGCCGGTAAACCTTCATGTCAGGGATATGACATTCTCAAATGCCCTGCGGCTGATAAAA
GCCCAGACAGCCTGGAGAGCAAACATTCATCAGTATCCGGGGCTTTTGCAGGTCAGTTTTATGCCGCCGGAGACACGAAAAAAATGA
>LOLKGF_00245 Conjugal transfer protein
ATGAAAGTGCTCATTCCTCTCATGCTGTCGGCAATTTTACTGACCGGATGCGTCGCAAACACTGTGGTATCCCCGCAGAACGTCAATGCTCCACCGCCAGATATAAATGCCTGGCTGAATCCG
ATGAATGAAAAACCCGAAGGTATATCGGAAACCCGCTGGAAAATGTTGACTGATGCAGGAAGAACACTGGGATTCAGGGGAGGTAAAGCCCGACGCTCATGGGAATTGATTCAGGCTCTG
AACTCCCGTGAAAGCACTCTCAATGCTTTATACGATTTTCGTCCCCTTATAAGCCCTGAAGGATGGCTTCCTCCCGTCATCGACGAAGCACAGGATGTCGCACATATCACACCAGATCAAATTC
GAACATCGTCTCATGTCTGGACAATTATTAGACCTGAACGCTTTGTCAGTAACCCACCGGGATGGCGTGACTGGCTACTTAAAGGGTTGTCCACAACAGCCACCCCCGGAACAGAAGGCAG
CGTTGTCCCGGAAGACAGAACCCAACGTAAAGTCTGGGAAATGGCATTGCGGCAGGGCTGGCAGGAAGGACGACAGAATGCTGACCTGACACTGGAGGCGAACCAGAAACGCCTTACA
CGTGACTTCAGGGGAATGATGCTGTACTCACTGCTCTGGAGACAGGGCATGATTACCCGACCAGATGTGACCGATCAACTGCAGACAATTACCGGTAACGGACAAAAACTGGTGACGGGT
GATCGCGTCCGCCGTCTGAAAAATCACGCCGAATTTAACCTGCAGAAATCGCACTGGCGTCCCCTCATCACAACCGAAAGGACAAATAAAAATGCGCAGACACCATAG
>LOLKGF_00250 Plasmid transfer ATPase TraJ

ATGATGCCACTCATTGATGAGCTGTTTGGTCGTGAAGTCCTCCCCCGGGTATTATCAGGTAATCCTGCCGACCGAACTATCCAGATAAACGGAGATGCTTCCGGTCGCTATGGCCTTAAACGT
GGTGAGCGCGTCCGGTTACGCTCCCATCTCATCCAGGGAACATCCGGGGCGGAAGAAAAGGCCATATCGATAACAATGCGGGTAATACCGACAGCAATACCGGATATCCGGACCATGAATAT
TGAACCGGATCTCCTTGCAGCGATGGTCAGTAAGAACGGTCTGGGGTTTGTCTGTGGCGAAACCGGCTCCGGGAAATCCACGCTGTGCGCAGCGCTGTACCGTTATATCATGGATAACTTCC
CCGACCGTAAAATCGTGACATACGAAGATCCAGTTGAATACATCCTTGGCCGCGAAGGCGATCTGCTTCCACCACACCAGGCTGAAATAGGTCGTGACGTTGTCAGCTTTGCTGCAGGACTT
CGTTCTGCCGTGAGACGTAATCCTGAAATTATCGGTGTTGGTGAGATCAGGGACAACGAAACCGCAGATGCTGCGGTACAGGCCGGAAATACAGGACATTTATGCCTCAGTACCATGCATAC
GAAATCGCCGGGGGAAACTTTATCCCGCCTCCTCGGTCTGTTTCCTTCCATCATTCGTGACGCAATGGCCTGGTCTGTTCTGTCGCTGCTGCAATTCATTCTGGTTCAGGTACTGGTCAGAAC
AAATGACGGTGGTCGTCGCGCTGTCCGGGAATACATTGTCATTGACGATGAGCTACGCAATAAGCTCACCAGAATGCCGCATTCTGAGTGGGGTTATCACATAGATGAGATCATCCGGACCG
AGAAACGACGTATCCGGGATTATGTACTGGAAATGTATATCAAAGGGGAAATTGACCGCAGCGAGGCAGTTATTTTCATTCCTCCAGGAGAAATGCGCCAGTGA
>LOLKGF_00255 Conjugal transfer protein
GTGAAAAGAGAAGGCTTCTGGCGGTATGCCACGCCGCCCATAAACATACAGGGGGTTCCCCTGCCGTTTACCATGATTTATCTGTTGATCTTCCCATTCCCAGCCAAAACAACCTTCTGGATT
TGCACCGGCATTATTTTATTTTTCATCATTCTTGATCGTTCTGGCTGGTCCGTTCGTACTCTCTGCACCCGTCTGTTCTGCATCCTGAGAGGTGCTGTAGCTTCCGGTCGTCCATGGTGGTACCG
GCATTTTACAGAATCACCGAGAGACTGGACCGGGTTTAAATAA
>LOLKGF_00260 Endonuclease
ATGTCAGGAAAAAAAAAGCTTATAACTCTGCTTCAGGTATCGCTGCTGGCGTGCCCACTATTTTTCACGTCTCCTTTGTTCGCAATGACCGACACACCATCTGTACAGGTCGGTTTCTCCCCCG
AAGGAAGTGCTTCCGCACTGGTTCTGGACACCATTAACAGTGCTGAAAATTCCATCAGAATGATGGCTTATTCGTTCACTGATCCGGATGTAATGTATGCACTTGCCCGGGCAAAAAAGCGC
GGAGTGGATGTCAGAATTGTTGTTGATTACAGGGGGAATACCAACCGGGCAAGTCGGGAAGCTATGAAATATATCAACCTGCTTAAGATCCCCCTCCGGACAGTGGATGTTTTCCCCATACA
CCATGACAAAGTCATTATCGTCGACGGCAGTACGGTTGAAACCGGTTCATATAATTTCAGTCGGGCAGCGGCCCGCAAAAATTCTGAAAACGTCGTTGTTATTAAAAACATGCCTGACGTTG
CCGCACAGTATCTTGAGCACTGGCAGGATCGCTGGGACAGGGGAACTAACTGGCAGCCAGTGAACTGA
>LOLKGF_00265 hypothetical protein
ATGTTTCTGACAGATACAGCGCCGAATATAGACGCTCACTGTTTCAGCGGACGTGATTTCCTGATTCTTGACCGTATTTCAGAAGTAAAACGCCATCTCTTTTTAGCGGATGGAATCACACTGT
AA
>LOLKGF_00270 DNA primase
ATGAAAGCCCGTTACAGAACCTGGCTTGCCGTGCCTCCTGATGAGCGCGAGCTCGCCCGCAATGCCCACCCGCCGGTCAATGGACAGAAAGCGGTTGCCTGGAACAACGAACATAAGCTG
TGGTATGCCCAGCCCGGCGTGGAACTCTCCCTGCTGGATAAATGGCTCCCTCGTCCTCAGAATTTTTCCACGAATGACAGTGATCCTGTCATCGAGTTTGCCCAGGAACTCGAGAAGGCCGG
TCTGATTATCAAAGGACTTCCCGTCATGGACGGTGCCCTGCACCGGGTACCGACAAAAGCCGATAAAAAGGGTGTGAAAAGCGGCGTTTATAAAGCATTTCTTGATGGCCAGCCCGCCGGC
TGGTACCGGGATTACCGCAGCGGCGATACGGAGGTGACCCGCTGGGTGTTTTCTGGCGGTGACAAGATAGATCCGCTGGCCCGCCTCCATCTGAAGGCGCAGGCGCAACAGAACCGGGA
GGACAGCGCGAGAGCACAGGCACAGCAGTATAACCGCCAGGCCGGATATGCTTCCCGCTACGTCTCACGCCTGCCGCAGGCCACCACCAGTCCCTATCTGACACGCAAGGGCGTCACAGC
AGCGCCGGGCGTACGTATCAATCCCGGGGGTGAACTGGTGATCCCGTTCAGTAATGCACAGGGGAAAATCCGCACCTACCAGCGCATTCCGGAAACGGGCGGGAAAGATGCCCGGCTGC
TGAAAGACGCAGAAAAAACCGGTAACTGGTTCGCCCTGGGTTCTCCCGTAAACGGTCAGCCACTGCTGTTTGCCGAAGGCTATGCCACCGCGGCGTCACTGCATGAATCCACAGGCCTGC
CGGTACTGATGTGTATCGATGCCGGCAACCTGATTGCTGTTGGCCAGAATGCCCGTACCGTCTGGCCTGACAGTCCGTTTATTTTCTGCGCCGATAACGACCATCACCTGCAAAACCCGCAGA
CCGGAGAGCCGGAGAACAAAGGCATTCTCAGCGCCATAAAGGCTGCTGAACTGAGTGGAGGAAAGGTCATCATCCCGGCATTTACTGAGGATGAGAAGGCACAGAAACTCACTGATTTC
AACGATCTCGACACAGCCCGGGGGCGTGACACATTCCGCCGGGTAATCAACGTGCAGTTGCGCGAACTGGGCGTCAGAACAGATTTCCAGGACACGTACGACGTACGTGAGGCATTCACT
GTCGGCCCACTGACGTTCACCCCCGTCCAGTCTGAGGAGCAAATCATGGATAACCCGACGATAACAGAGCCTGAAACACCGGCACCGGCATTAACGGAGCCGACGGCAGTACCACAGTCA
CCAGAACTGCAGCCGGAAATACAGCCCACTCCCGGGTCTGACAACACAAAACCGGACAATGTAGCCGGAGAAGCACCATCTGACCCCGCCGTGACTACCGCAACCGCCGAAAACAACCA
GACGTTTGAACATACCCAGGAACTACCGGAATCGGGCATTACTGAACCAGTCCGCTCATCAGGAACCTCCCCACTCCCTGACACTGGAACTTCACCTGCAGGGCAGAATGAGAAAGAGGAT
TCGGTATCAACGACGGACAAAGCCTCACAGGACACTTCAGTGCCAACTGAACCAGATAAAAAAACAGGGGCTGAACCTGTGTCAATCTTCCCACAGGAAGAGATTGTGGCACATTCTCCTG
AAGACGTCCTGTTTCAGCCTGAATCGTACCCTCCTGAGTCTGAAGCACCCGATAACTATGAAGATTACCGCGCATATCAGGAAATGATGATGACCAGCGAATCTTCAGAGCAACAGCAGGAA
GCCGGAGATTTCGCCACTCAACCTGATCTTCCTGATACAGCTGGAGTACAGACAGAAAAACCTTCAGACCGAAATGGCCCAGGCCACGAACCAACCAGCACTCCAACGAATCTAACGGAA
ACACAGGCCTCCGGAATTGCTCCTTCATTCATGGGAAAAGCACGCGGTTTTTTCTCACGCAGGACGGTGAAAGCTAAGGCGAATGAAAACAACACGTCAGACATATCATCTGAATCTGATG
TCGATTCATCCACACAAGAAGCGGACACTATCGTCTGTGGTCCCCGCCGTCCTGATGCCCCTGTATCGCTCGACATGGACGAAATCCTTAAAGAACTGAAGTGGGAAACGCAACCGGACAA
AACAGTTCTGTACCGTCTGGATGATACCCCTGCATTTATTGACCGGGGCAACAGGCTGGAAATGGTCAATGGTGCCAGCGAGAACAACCGATGTGTACTGGCCGCACTCGCAGTGGCCACA
AAATTTTATGGTGGTGTCATTGAACTGACCGGCAGTGACGCCTTTAAGAAAAAAGCGATGCAGTTGATCATCGATAACAATATCGATGTACGGATGAAATTACCGGATCAGCGCGCTGAACT
GGAAAAATTACGTCAGGAGGTGGGCACCAGTCATAATACGATCGTCACTCACCAACCGACTCCTGATCTTAACCAGAACACCCCCGCACAGCCGGCCATGCCAGAACCGACTCAGGAGAAT
ATACAGAAAACTGCGGCAGAGCCACAACCGACTACTTCTGCAGTACAACCAGCTCCTGCATCCGGATCTCTGACGCCCCCTCCTGCAGAACCGGACCAGCCGGATACTCAGGCGCCAGCCG
AAGAACCGCCGGGGAAACTTAATCCTGGCGAAAGTGTCACCGCCGTTCTGAATAATTTTGGTCGTGCCGAATACGCCCCGGGCAAAGGTGAAAGTTTCTTTGTTGAACTGAAAAATCGCAG
TGGTAGCAAAATTTACTGGGGACAGCAACTGGAAGACCTGGTTAAACAACATCAGAAAGGTTCTGTGGTCACCCTGACGCTTCAGAACCGTGAACAGTTCACGCTCCCTGGCGAAACCAG
AGTACGCCTTCGTAATAACTGGCGCATGGAGTCAGTCAATAATGGTATTTCAGTCTCCCATGATAATCCTGAGAGCGGTCAGCAAATCCAGGCGTTTCCGGTAGAAACCTTCAGGAGTGTTAT
TGAGCAGATGCGCCAGGGATGGCCAGAAGAAATGAAAGAGCTGCGGGTGCCTGAAAATACAGAATCACATTTATTCCTTGGTCCAGACAGACATCCTGTATCTGCCTCACTCGATTCAAAC
AGGGTAACAGTGACAGATGGTGATGCGCCTGAAAAACTGATCCCGGTACTGGGATCTGTAGATAAGGAAACCCGTCAGCTCAATCTGCTACTGGTACAGAGCGCAGAAGATAACCTGCAG
GGAGTGGTCCGCCTTAACGGTACGCTGTATCCCGCATTGGCCACGCCTTCTGCTGATAACAATCAGTTGGTTATCAACGCACTGACAGACAAAGGCCTGCGGTTTGCCGGCTATGGTGAAGC
AGTAAACCATGATGCCGACAGCACATCCAGGCCGGCACCAGAACTCATGCAGTTTCATCTGAAAACCCGGGAAGAACCACTTTTTGCGGCTGTATATAACCCGGATCAGCAACCCAACGAA
CTGTACCGTAACCTGGGTTTTGAGCAGTCATGGCAACAGTGGTCAGACAGCCAAAAGCCGGAGGGCAGACAGGAAAAAACTCTGCATCAGGATCTCTCCCACAGCCCCGGTCGATGA
>LOLKGF_00275 Conjugal transfer protein
ATGAACATGCATAAATCTCTGGTTGCCGTGGTATCCGGCCTTATTCTGATTTCCTCCTCAGCCGGTGCTGCTACCTGTCGTGCGGGAAACGCGGCACAAGCCGGAAGTGATACAGGATATGA
GCGAGCACGCGCAGCTGCGGATGCATGGTCACAACGTGAAAACGATGTTTCCTCATCCCTGCAGTCCTGTCTGTCCAGAATCAAAAAAATCAGTGTGAATATTCCTCAGTTCCCCAGCCTGG
ACGACATCCTTTCTCAACTGGAAAACCAGGTCTGTGATGCTGTCGTGGACAAAGTTAACGAGAAACTACCAGGAAATATTGATCCCTGGAAAGATCACAACATTTAA
>LOLKGF_00280 Conjugal transfer protein TraM
ATGAATACCATCAGAATCATGAAGCAGGCAGAGCAACGTGCCAACCTGGTACCCGGGCTGCTGAAAGCGCTGTTATGTACAGGTACAGCGCTGCTTATCAGCCTTGCCGGCAATGGTGTGC
AGTACTTGCGTTCCACGAACGTGGAACGTGAATACTTTGCAACAGATAACGGACGTCTGGTGCGTCTGGCACCGACAAGCCAGCCAGGATGGAGCCAGAATGACGTAATGTCTTTTGGCA
GCGAAACCCTGAGCATGGCTTTTAATCTGGACTTTGTTCATTACCGCTCACAGATATCTGCGTTGGCCCCTCGTTTTTCAGATGGAGGATTTGCCGGATACGTGAATGCGTTACAGGCATCTA
ATATTCTTGACACGATCAAAAAAGAGAAAATGAACCTGACAGCCACTATCGGCGTCGGTGTACTGATCCGCCAGGGACAACTGAACAACGGCGTCTGGTTCTGGACTTTTCAGTATCCTGTA
AAAATGCGCCTGGTTGGCCAGACTGTCAGTAAACCGGAACAGTCCTTCATTTTTGAAATGACTATCCAGCGAGTTGACCCCCGCCTGAAACCTTCCGGTATGGAAATCAGACAGATGATCTC
CCGTAACGCCGGTTCGAACTGA
>LOLKGF_00285 Conjugal transfer protein
ATGCGATCACGCCATATTCTTAGCGCACTGTTCCTTGTGTGCGGCACTGCCGCATCTGCAGATAATGCCGGGTGGCAGAACGCCCGTACTCCCCCCCCGGATAAGACCACCGCCAGCGAAAA
TGCGCCACCGGTGGCGAATACCGGCGGTGTTCCTGCCACACTGGTCAGGGGAGAGCTTCCTGCTCCCGGACAAACCAGCTCACTGGTTCAGGAAGCAGCCAGGCTGGACTCTGAACTGT
CAGCGGATGAGATCCGGGAGTTACGCAGCCTGATGGCTGACAATGAAAGAGCCATCAGCGCCCCAGTGACAAGTATCGTCCCCCGCATCAGTTCGCTGACGGTTAACCTGTCGCCGGGAG
CCAGTCTTCCGTTGGTTCGTACAGCCATGAATAACCTGAGTGTGGTGACATTTACCGACATTAACGGTTCTCCATGGCCGCAATCAGACCCACCCTATAATGCGGCGCCAAAACTCTTTGATGT
GCAGTACAACGGGAATATGGTCACAGTCACGCCACTGCGCCCGTGGGCATCAGGAAACATATCAATCTATCTGAAAGGCCTGTCGGTCCCGGTCATTCTGAACGTAACCAGTGGCGAGACA
GATACACAGGTCGCCAGCCAGGAAACGGACAGCCGTCTTGATCTGCGTATACCACGACAGGGGCCGACCAGCCCGGTAGTAAACGTTCCGGCAGATAAAATAGCTCTGCATGATGCCACAC

TACAGGCATTTCTGGATGGTGTACCTCCCCGAGATCCGTCAGTAAGAAGGCTTAAATTCACCGGCAACGTCCCGGATACGACAATCTGGCAACATGGTGATGATCTTCTGGTTCGCTCCCGTG
CGATGTTACGGGATGAATTTGAGCAGACACTATCCTCTGCGGACGGTACACATCTCTGGAAGCTTCCCGTCACCCCACTACTGACTTTCTCTGTGAATGGCCAGTCTGTTCACGTGACACCAG
ATCTGGAGTAA
>LOLKGF_00290 Conjugal transfer protein TraO
ATGAGTGCTGAACAGGATGCCGGTAAATCCGGCAAGAAACTCTTTGCCCTGTCGGGACTGGGTGCCATCGTTTTGTTTGGCGGTGGCTATTTTGCCCTGTCCTGGTTAACGCAGAGTGACA
GCGACGTGCAGTCTGCCGTGAACATTAACAGCACAATGAACGGCGGTGGACGTCATGTGACAGAAACACCACATTATCGTGAATTATTACAGGCTGATAATGAACGTGGCGCTGCAGCTGC
AATCCGTAATAATCAGACTTTCATTGCCAGCCTTCCGCAGGGGCTTGATGTACCGGACACATCAGAAAAACATCAGCCACCTGCGCACGAACCTGAAAAAAACAGACAACAACCGCCAGAC
AGACCTGCACCGGAAGATCGCGCTGCCGGAGAAAAACGTGTTGAGCGACTACAGAAACTGATTGCCAGAATAAATGCGCAGCAATCCGCCGGTAATGCCCCTACCACAGCAGCGGCAATG
TGGAACAGACCACCTGCAGAAACGGCGGAACAAAGTGGCAGACAACGATTCGACTTACAGAATGCCAGCCGATCAAGGTCTGTTGCTGACCAGGGCATACAGCTGATACCGGCGTTAACC
CGCGTACCTGCCTATATCATCACAGCCGTGGATTCTGATAATCCGTCATCCAAGGTTATTGCCTCCATTCCGTCCGGCCAGTGGGCTGGCGCCGTACTGTACAGTCCGGGCGTAAAACTGGCT
GGAGATGGTGTTGAGATCCATTTCAACCGAATGAGCTGGAATGGCATGAATCTGAAGATCAATGCGTATGCACAGCGGGAGGATAACCTGATGTCATCTGTTGCCACCAGTGTAAACAATCG
CTGGTTTAAACATATTGTTTTGCCGTCAATTCTGGGGGGACTGGGGGGAGTAGGTACACTTTACAAGGATGCCAACACACAGGTTATTCAGGGAAATTATGGCAGTGTCACTGGCCGTGTT
GGGATGCCGGACGGTAAAGCGGTTACCGGCGTCATCGCCGGTGGTATGGCCGAGAAAGGAAGTCAAATCCTGACCCGTCAGGCAGAAAGTGAACCTTATAAACAGGTGACAGTGTCACA
ACACGAAGTGGTTTCAGTTTTGTTTGTTGATCCGGTCATGAGTAACAGCATCCAGGCTGATGCAACGGGTACTCCTTCCCTTCAGCCAGACACACGTCCTGCCACACTGGCGGAAGCAGAG
CAACAAACCCGGCACAGGCTGGAAGCAGCAATAGAACGCAGAAGAGCAGAAATGCAGCGCCGTTATGATGAACCACTGGAGATTCCACAATGA
>LOLKGF_00295 Conjugal transfer protein TraP
ATGAGATCTGAAACAGAGCATGATGAAACAGGCAGCTTCGGCGAAACAGATGAGGTACGGCTTCCCGTCTGGAAACGTCCGATATGGGGAATAAGCCCGACAATGTGGGGGTTCTGTGCA
ATTCTTCTTCTGGCTGCTATCTGGTATTTGTTTCTGAATACGCCGACAACCGGTCAGCAGTCAGACCCGCCGTTTAATGATATGAATAACAACATGCAGACGTGGCAGTCCCCTGCTTCTTCCA
CTTCCCCTGTCATGGAAACAGACCTCCCGCAACCATCGGCTTCTGCCAGTGATATGAATCAGCTTGCCCGGGATCTGAAAACGGAACTGGATGATCGTGACGAAAAAATCCAGGGAACGCT
GAATATGCTCCGGGACAGTATAAACAAACTCAGTGAGGCTATAAAAAAAGACGAAGATTATGCGCAGGAAACGCGGCATCAGCTGGATGAGATCCGTACCCGGCTGAACGGCGTGATAAA
CCAGAAAACCACGGCAGAAACGACCAGCGTTTCACATTCCCCTGTAAAAAAAGCACCTTCTCCGGTTGCGGGAATGAAAATTATGTCCATGGAAAACGGCATGGCATGGATTCGCTGGCAG
GGAAGTACATGGGCTGTACGCGAAGGTGACAAACTTGGGAAAATCATCATCACACGAATTGACCCCGACTCCCGAACCATTATCACCTCCGGCGGGACATTACGCTGA
>LOLKGF_00300 Conjugal transfer protein TraQ
ATGAATACAGATGCCCTGACCGCGATAGAACACTTTGCCAATGGCATCTTCAGTGCCGGAATGGATTTTTTGTTCACATGGGGTGAGTTTCTGGGGGTTATTGGTTTAATTGCACATCTGGCC
AGATCCCGGGTTGACGGTCCTACCCGGATGACTCCTGGCAAATTTGTTGGAGGGCTTTTTACCTGCGCCATGCTGGTCTCACTCCCGTCCATTATTAATGCTGGCGGGATTCAGATGGGGTTT
CATGCCGATTCTTTCGGCCCTATCGCTTACGTTCAACCTCAGTCATTTGGTGCCGCTGCCGGTGCGGCTAACGCTGTATTGTCACTGGCAAAGCTGGCTGGCGTTGGATTCGTAATGAGTGGC
ATTTCAATCTGGCGTAAAGCCGGACTTGACGGACATACATCACTGAGCGCCAGCGAAACCATCAGCAAAGGAAACGTTAAGTTTATCGCCGGTGTTCTGTTGGTTTTTATTGATCGTGTTCTT
GATGCCCTCCTGGCCAGTATTGGCCTTGCCTTCTGA
>LOLKGF_00305 Conjugal transfer protein TraR
ATGTCACGTCTTCGTGTCATGATGGCGCGTGTCTGCACCGCTTTTATTTACGTACAAATCTTTCTGATGAACTGCCGCGACAAAATTATCAGTCTGTTACTGCTTCTTCCTGCAATGCTGTTACC
CCGTACTGTTATGGCTGATGGTGATCTTGTTGACATGATCAGAAATGTGGGGGATGGAGCAAAAGGCGCCACAGGTTCCACCCTGAACATTGCTCAGCTTATCGGTGTTGTGATGTTCATTG
GTGGCCTGCTTGGATTCAAAAAAGTGGGTAAGCAGAATGGACTTGGTCTCGCTGGCTGCATAGTGTCCGTCATCATTGGAGCGATACTTGCTGTCGTCCCTGAAGTGATGAACCGTTCACAG
CGCCAGTTGGGTATATCTCCGACCACGATAAATTAA
>LOLKGF_00310 hypothetical protein
ATGCATACCAAGAATCTGGATAATTTCACATTATTGCGAACTGAAAACGGTAACGTTATTGCCCGAGACAATCGGGACAATCAGGAGTTTGTCTTTGCAGAGTGTTACTCCCGTGAAATAGCT
TCAGTCATTATGGAGGCTGTTAAGCGCTACTGCAGCGCGGAGATACCCCATGCCACAAAACATTAA
>LOLKGF_00315 Conjugal transfer protein TraT
ATGCCACAAAACATTAAGGACTTATGGTTATTCCCGTTTCCGGTCCCCGACGTGGTACATACCCAGGAACCACTGTTACAGGCCCCGGAGTTAACAACACCAGGACGCTGTACCTGCTGCCG
TCAGACCGTCAAACACCGCTTCCGGCTGGATGAATCCTGGCCTCTAAGACAGCTTTCCGACAACATCCTCGATGTGACTGCCCGGTTGCACAAAACAACAGAAAATCTGGAGCGTTTACTG
AAACGCGGAGAACCCGTCACAAAAGATGAAAAAGAGAAATACCTGACTGCACTGATAGCAGCGGAACGGTCGCTGGAGCAGGCACAACTCTCTGCCAGAAGGCTTGCTCTTCGTCACGT
ACCCAAAGCTGAAATCACCCATACCGATAAACTGTCGGAAGATGAACTGAACCTGTTGCATGAAACCAGCGCCCCCTATGAACTGTGTGCATTCTGTCATGCCTGGCATGCTCTGAATGGTTA
TGCCGCTGCACAGGGAGTAATAGTCTGGTTACCAGATCTCCATCCTTCAACTGTCGTTGCCCTGAACCGCCGGGCATTACAGGAAATTTTCAGTGGTGATAAAACTCGTGTGCGGCAGGGCC
GTGAAATACTGACCGCACTTATGCAAAACAGGCTGGCTGTTGAAGATAAATTTCGTAGCTTCCGGCCAGCAGACTTTGCAGATGCTCTTCGCCGTTATCCCCCTTCCCGGCGTTCCCCACTCA
GGGAAAAAAATGAACGGAATTGCGCTGATTCTGACACCGGATTCATTTCCGGAAAAAGCCTTTGTTGATTGA
>LOLKGF_00320 ATP-binding protein
ATGAACATTAACCGACTCATCTTCACGATAGAAGACTGCATAAACACGTTATCCCGTTTTTCCGTCGCGACCAGTTTTGTGGACTATTGTGATTTACGCACCGTAATCGGTCTGGATAAACAGG
ATCATGAACGACGCCCATGGCTTGAGGCTCCCTATATTGCTGTTACAAAGCGTGGTGAATATCTTTCCGTCTTTGAAGTCAGCGGCGCCTTCAGGGAAATGGATGAAGATCCTGGCCAGACA
GAAAGTGGCACACTGGAATGGCTGATTGCCAACCTGAGTGACAGCCTCAATACGGCATATAAAAACAGTGGTCATAAAATCAGTTTTGTTTTTGAGCGTGATCCTGAACGCGGAAAGGACG
AGATTGTTGATATGATCGCCCCCCAGAAGCGTTCACTTGCCAACACCGGTATTCAGTTACAGGATGTACTGGATGAGAAAATAGCAACGCTTTCCCCCTGGCTTGTCAGGGAACGTTGTTGG
CTGGCTATCTGGAGCGCACCAGACCTTGTCAGTAACGGCGATCGCTCCGCCCATGATGAACATATCAGGAAGTTGGCAGAACGTGTTCCGAAATCCAGATTTGCCCAGTCACCATGGCAATG
GACATTATCGGCCCTGAAAATCCGCCATGATGCCTTTCTGGATAACGTGGAGCAGGCTCTGAGACATTCATCTGATGGCCTGCTTCTCAGGCTGATGGATATACACGAAGTCGGACAGGAAA
TACGTAGGGAAACAGAGCGGGACAGCACACCACGTAACTGGCAACCACATCTGCCCGAAGACGCACAACCAGCAGGATATCGCTGGGGAGATGATGACTCCATACTCCACGCCCCTTCATT
ACATCTGCAGTTATTCAGTACCCAGCCTGAAACAATGGGCAACCTGGTACGCGCAGGCGGACTCTGGCACGGAATGGTCGCTGTCACGCTTGCACCGCAAAATCTGCAAACTTTTAACAAA
CTGGTCCATGCTGTCCCCCGCGCCGTCCCCTGGCGTATCCGGATGGATCTGATGCCCGGAGGGATGAAAGCCCTTGGACTGAAGAAAACTATTCTGACTTACAGTAGTTTTATCAGTGCCGT
TCGCCCGATGTACGACTCCGTGATGGCCCTGTCCGCAACAGACGAAAAAGACCCCGTCTGTATTATGACCATCGTTGCTTCCACATGGGGCAAAACACGTGAACTCTGCGCACGAAACCAG
GCTATCCTCAAATCAGCCATAGAGGGCTGGGGCGTATGCGGTACCACGACTACGTTTGGCGATCCCCGGCGTGCCTGGGTAAATACCATTCTTGGTGCATCAGCAGGTTCTGGTTCCGTTCC
ACTGTATCCTCCTTTATCACATGCGCTCTCACTTTTCCCCCTGAACCGGGCAGGTACGGTCTGGCGTGGAAAAGGAAACCTGATGCTGCATACAGAAGATGGTGCCGCGTTTGAAGTGGGTC
TGGCATCATCCCAACAAAACAAACATACAGAACTGGCGCCGGGTGATCCCGGTCTGGGTAAATCAGTCCTCATCAATACCTTGTCAGAAATACAGGTATCGTCCGCGCAGAAAAACCTGCCC
TTCATTGCGTATATTGATAAGGGATACAGCGCTCAGGGGCTCGTTCAGCTTATTCGCGACAGCCTGCCCGAGGAAAGAAAGGACGAAGCCGTTGGTATCATCCTGAGTAACGATCCGGAATA
CACCAGAAACCTGTTTGATGTAATGTACGGGGCTAAAAAGCCCATTACACCAGAAAAGAACTTCATGTCTTCTGTTCTGTGCGCCCTCTGCATCGATACCAGCACAGGACAACCCTGTAACCC
CGGAGATACCCGACAGATCATCAGCCAGTTAATTGATCTGGCCTTTAAGGAATATGGTGAAAATAATCCCAGATTATATCGCGCCGCAACCGAGGAACTGGTGGACACAGCACTTCAGGACT
CTGGTCTGTCTGAAAAATATGATACTGAATGGTGGGCAACTGCCACCTGGTTTGAAGTCCGCGATATGCTGCACAATGCCGGTTTTATTGCAGCAGCCCAGAGGGCACATTATCAGGCTATGC
CCCAGTTGTCAGAAATGTCCGCCTTGCTGGGCAATCCAGGTATAAGAGATATTTTTGCGGCTGTTCAGCGTAATGGCTCCTCTGAACCTCTTCTGGATTATATCCGACGTTCTCTTGATCAAGG
GCATAACGACTACCCCATGATGTCCGGGCATACCCGATTTATGCTGAATCCGGAAACAAGGATTGTCGCTGTCGATCTGAACAATGTTGCAGGTGACAAGACACCGGCAGGCAGACTGAAA
ACCGGCATTATGTATCTGCTTGCAGGACAGATTGCCGGCGGTGATTTTACCCTACCGCAATATAAAGATGAGGTGCTGAAAAATCTCCCACGAACATACCATGACATCGCGCTTAAACGTATTA
ACCAGCTTGATCAGGAAGTTAAAACTAAAGTTTATGATGAACTGCATAATGCGAGAGGTATTGACTTCATCTGGGATGGTCTGGATACACAGGAACGTGAACAACGTAAATTTGGTATCCGTA
CTGTATTAAGTACGCAATATCTTAAAGATTACCCGGAAAGTATTCTCAAATCAGCGAACACACTCTGGCTATTGCGTTACCGGCCGGAGGACATTCCGTTTCTGAAGGATAACTTCAACGTGCC
TGAATTCATGCTCAGGCGCTTCCTGAAAATGCCGGAGGGGCCTGCGCCAGATGGCAGCGGTGTTCCTGTACTGGGAGTATTCAGGGTTAAAGGCGGTACGCTCGCGCGCATACTGAAATTT
ACCGTCGGTCCTCATGAGTTATGGGCGCTGAATTCCTCACTGAAAGACAGTGCATTACGCAAAATACTGACCAAAACGTTAGGCTCTGTTCGCGCACGCAAAATACTGGCTGAGAACTTCCG
ACAAGGCTCGGCAACATCGTACATTGAGCACAGGGCAGAACAACATAACAGTGACAACGTCATTGAAGAACTGGCCACTGAGCTCATCAGACAACAGGGATACAACCTCTGA

>LOLKGF_00325 Conjugal transfer protein TraV
ATGTGTACAATCCATATCACGCCCCGCTGCCGTCGACCAGCAAGAGGACAGATATTCCTGAAAAAGCTCAAAGTGGATGGTGACAGCTTTTCATTCTCCCTGCCATCCCGGCACTTCAGGTTA
AATCCAGCCCCCAGTCCCAGGGTGATGTATGCCACTGATGCAAGAGCGCAGGCGCAGGAAAAGGCATCCGGAATCCGCCTTGTTCTGCCCGGGAAAACAGAACGTCTGATCACAACGGCC
ATCTGGCAGGTCACTATCCCGGATATTCCCGAGTTTGACGCTCTGCATGTCATTCAGTGGATACTACCGTCCCCACTGGCCCCACTGATGTCTGATAACACCTGGCTATGGCCAGCCATACCTG
GTGCCCCGGCTCTGAGCGCCGGAGACTGGCCCGTAATGGATACCGATCCGTCAACCCTGAGACGGGTCAGACGTCGCTCATTCAGCGTTATCGATACCAGAAACAGGAGGGGCTACATCAC
CCGACGGTTCCAGTGTTTCCCTCTCGCCCCCCTTCCTCCGGAAGGAAACTACGACATCATTCCTGATTTTGTTATCGATCCAGGACAGGAGAAAACAAGATGCACAGAAAACATTTACTTGCC
GCGCTTATCGCAGCCATAA
>LOLKGF_00330 Conjugal transfer protein
ATGCACAGAAAACATTTACTTGCCGCGCTTATCGCAGCCATAAGCGGCGCCACAATTCAGGCACAGGCCTACACGGTCACAGTAGTGGAAAGTCGCCCGGTAACCACACAAATTTTGCCAG
AAATGGCTGCTATTCAGGGAGTTCTGGGGGAAATACTGGCCATGCAGACAGCCACAGGAACCGCCATAAATCAGAACTCTGAAAAGCTCGCAACGGTTATAGCGCAGGATGGACAGGCAA
CCCGCCAACAGATGATATTTGGTAATGAAACACAGCGTCTTGAGGACGCCAGACAAAGCTATTCCGTACCGGACTCTATCTGCAGTGAATCTGCATCCGGGATAGCAACAGAGAGTAAAAAC
GCCTCCATGTCCACGGCGTCAAAGCTAAGTAAGGGCGGCGGTATCAGTAATCGTAACATTCGAGAGCGCCTGGCTGTTGCTGCTGCCTCCCCTGCGCGCGAAGCTTATGACGGTGCATCCAT
ACACGCCGGTTACTGCACTGAAGCAGAATATGCCCGTTTTGGTGGTACTGCGGTTTGTCCTTCTGTTGGCGAACTCCCCGGTGGCGACAGCCAGGTTCGCTCGGTCTATTACGGGGCAGGA
ACGGCAGACACGCCGGCAGCCCTTACATGGGATCAGAAGCAGATCGACGCTGCCACTGCTTACATGAAAAATACTGCCCGACCGTCAGCTGGCCGGGCGTTGGGGAAAGGAGAAGTAAA
TACCCAAAGCGGCCGCACTTACGTTGGTCTCCAGAATGAATATAACGGCATCATAGATGCGGCATCGAACCCCCAACTGTCGCTGATTGCTGACAGCGCACCGAATGAGGCAACCCGGAAA
GCGCTGGCAGAAACCCTTCAGTCAGATTCTGCGGCAGCTTATTTCGACCAGATAGCATCAAAGGAAGCAAAATCGCGGGGATATATGAGCACCAGGGAGTTTGAATCCTTTGAAGCTGGTC
GCCGCTACGCAAATACCGCGTACCTTACTGACCTGCAGGAAATGCAGGGGGATAACCTGTTACGCGAACTGGTACGAACCACATCTCAAATCAACTGGCAGCTGAACGATTTGAAAGAGCA
GATTCGCCAGGGGAATGTGATCGCCGGTCAGCAACTGGCACTTACGGCGCGCCAGTATTATGAACAGCGGCTGAGTAGTCTGGAAATGTCCGTTAACCAGGGAAATGCCAGATGA
>LOLKGF_00335 Inner membrane protein
ATGACAGCAAAAATTAAAAACCACACAACGTCCCTGCAGAACGCAAAACACATCATGCATGAGTTATGGATCATCGGTATCAGGATGCCGGCTACAATCGCCGGCCTCTTCCTGTTTGTATTG
ATACTCACCCTACACGATATGGCAGGATTACGAAACTGGATGGTGCTTTCCCTGCTTTGCCTGATTCTGCGATTCGGACAGTTTATTTTCTTTCACTTCAAAACCCGGAAAGGCATCAGAAATG
AGTAA
>LOLKGF_00340 IncI1-type conjugal transfer protein TraX
ATGAGTAAATTACCCGGCGATACAGAACAAGAAGCAACTGTAAAAAAATCACCGTCTATAGCACGCCTGGCTACGCGTGGCGTGGTTAATATTTTCGTTCCGGTAATTGAAACCCGCTGGATT
GCTCGCTCATGTCGCCACATGTTGAGTCAGCAAATACGGCGGATTAAAGAATTAATCCGGAGAACACACGAGGAGACCTTCAGTAACCTGAGCTGGGAAGAAGCTGTACAGACAAGCGGT
CTGCCAGTTACTGAACTGAATCGCTGTTTCCGTCGGCGCAGATTATTCTGGCGGGCGCTTTTCTGGTTATTACTGATTCCTGCCGTTTTCTTGTGCGGTATGGCGTTTGCAGCTTCAACGCTCC
CCTTAATTACACTACTACGGTTAATCACAACGATCGTACTGATAATTACAGGCGCAGCGCTTTGCGCTTCAAAAGCACTCATCACCTCATACCGTCTGTGGCAGTTGCATGAGCGTAAAGTCAG
TGAGGCAGAAAAAGGGACTTTCCTGGACTTTCTGAATGATCGAAATGGATGGTGCAACGCTTCATGGCTGGCAATTACCAGTAAAAAATACTGA
>LOLKGF_00345 DotA/TraY family protein
GTGAAAATTATACTTCGCGCACTCAGTGCAGGCCTCGCCGTATCTGCAGTCCCGGCAATGGCTTCAGTGACCTATCAGGATATTGTGTCCGCAGCCACTAACTCTGACGATCTGTCGCGTCAG
GCGCTGGTCACAATATTTGGTGATGTGGTCACGAACCCCCTATCGACCAGCAGTCCTACCCTGATCGGCAATATGTTCGGTGTATTTAACGGCATTATTTCCGTACTTGCCGTAGTCTGGTTTG
CTTTCATCGGCATACGGCATGTTGTCAGATCCGGACATCAGGGCCAGGTCTTTGATACAGGACGTGACATCGTTGGCACTCTCAGTGTCGTCAGTGGATTTCTGATGATCGTACCTACAGGAA
ATGGCTGGAGTCTCGCCCAGCTTATCATGCTGTGGGGTGCATCAATTATGGGTGTTGGTTCCGCCAATATCATGGTGCAGCTGGCCGCAGATAATATTGCAAACGGTTACTCTATGACCGTGC
AACCAGTCCAGGTCTCAACACGCACCGCCGCACGCGGCATCTTTGAAATGGAATTGTGTAAGTATGCCATTAATTCCGGGCTGAATGACTTTAACCAAACGGCCAGGTCCTCCACTTCGCTT
ATGACCGAATCAAGTAAAACGGTCGGTGGAAATTACACCGTTACGGTATCCAATGGCAGTGGCATTTGTGGTACTGCCAGCCTGTCTGTTGAAGGCAACGGTACAACTAATCAGTCATCCAT
CAGTAAGTTTTTCAACCCCTTCAGCAGTACCGAATATAACAGCGTTATCGCTGCTCAACGTACAGCCATGGATAACATGATTCGTGACATGGACAGTACCGCTAACGAGTTTGTGACGACTTT
CCTGGAAAAACGCAACACCGGAAACGGTACGTTGCCCGATATCGAAACCCGCATACAGCGGGCAGCTGACGATTACGAGCGGGCAGTACAAAAAGCCCTGCCTGCAGATAACAACGAAC
AATCCAGGAAGGAGGCTCTGAAATCATATCTGACCACTTACGGGTGGGTGGCTTTAGGTGCCTGGTACCAGACGTTTGCGACAGCCAATCAGCGCCTTGCCGAACTGGCCGACAGGGCGC
CAGCAGTAACCTCAATGTCATCGATTGGTGAAGTTGGTGATACTGATTTTTTTGCTGCAGTGATGGGAGGGTACCGGGCACAGTTACAAAACTCCACATTTACTCCAACAATAGGAACTATAA
CCACTCAAAATGAAAGTAATGCTGCCAATAGCATTGATCCTCAGAGCGTAATAATGAAAAGTATTGCGCCTACTATATTAAAATGGACGAACCAAGTTGCTACTGAATGGTCAGGAACAGGAA
CAACCAGCGACCAGGTTAATCCATTAATTAAGATGAAGAATATCGGTGATTATACCCTAGGAACAACAGAGGTTCTCTGGGCCGGATATACAACAGTCAGAGTTCTCGCAACAATGGGGGAT
AACTCAGTTTTCGGTAAAGCCGCTAATTTTTTCACTGGGTTACCAAAAGGGTTAGTGGCCCTACTGGATGCTTCAGCACCTCCAGTATATTTCCTGTTGTTCCTGTTGTTTTGTGCGGGTTTTT
CGTTATCAATCTATCTGCCCTTTATACCTTTTATTTTCTGGATGACCGGAATAGGTAACTGGATTATCAGCGTATTAATCGGATGTACCGCAGGACCTCTTTGGGGGGCTACTCACCTCGGCACA
TCTCAAGACAGAGGAAGTCGTGCTGCCTATGGTTATATTTACCTGATTGATAGCATGATAAGGCCACCATTAATGGTGTTTGGGTTTTTCTTTGCATCAGTAGCAGTTGTTGCGTCAGGGACTA
TATTAAATGCCTTATTTGGCGCAGCCCTAGTTAATGTTCAAGTAAACTCCTTTACGGGCGTATTTAGTCTTGCTGGTTTTTTACTAATTTACGCACGGATTTGCACAACAATTGTTGCAGCTATAT
TTGCGTTACAGGCATACCTTCCTGACCATGTAATCAATTTTCTGGGGGGGCGGGATGGTGCAAATACTCTTGGAAACCTTACATCATCCGTGAAAGATATTTTTGCAGGTTCCAACAGGAATA
TTAGGCATACCCCTGGAGTTAAAGAGGACAGGCTAAAAAATGTCACTCAGGGAGATAATAGCAAAGATGGAATAAAAGGATAA
>LOLKGF_00350 Plasmid IncI1-type surface exclusion protein ExcA
ATGCAAATGGTAAAAACAAAAGACAGATTTCCGGGATGGTGGCCATTATATTACCTACTCAGGATTGCATATTTTTGTTTAGGCATACCATTTTTGTTACTTTTTATTATCTTTGGTATGTTATCA
ATCACTTCATCTAAATATGTCACGCAAGCAGATTACATTTACACTTACGTTTGTCTATTTCTGTTAATTGCTCCTTGTCTATGGCTATACACTAAAGCCAAAAGGAAAAAAAACACTATCCATTAT
GTTGTACAAAAAATAAAAGATACTGGTTATTTTTCCCCGGAAAAAGGCTTTGAGGGGCTTTCATTAATCAATAGCACATATTTCGGCATTGATATCAGAAAGGGCACTATATTATATATCAGGAT
TTATCCAAACAATATCATGGATGTCATTGGCCTTGATATACATAACTTCACCCGAACAGTCACTGAAGACAAGGAACTGAAGATATACACAAAGTATGTGAACATGCCTATGATACCTGTAACGT
CATGGTGTACTTCACCCAGCAGCGCTGCGAACACTATGCATGCTATGGCCGAAAGAAGTTATGATTACCCGGTAGACTTCCCCCGGATGATTCAGGAAAAGCGTAAAGAGTGGGAGAAGGT
GGCTGGCATACCCGTTGCAGAAGTATTTTGA
>LOLKGF_00355 Lipoprotein
ATGAAAAAGACCGCTCCGGGTAAGATCATTTTTATCGTCGGCACCGCTTTGCTGCTTGGTGGCTGTAGTGGCATGGCTATGCCAACTTATGCCACCTACGGTACGCCGGTTGACATCATCGCG
CCGGCGGGAAGCTATAGCGAGTGGCACACGGACAGCAGCAACCGCACCACAGGAGAAAGTCACAGCCTGTCACAGGGAAACTGTACCCAAAGTGAAGATAGCCAGCTCAGCAAAAATG
GTCTCACACGGACACATCAAAGTAACTGTAACACCCGTAGTCAAACCCACAGCCGCAGTACCAGCAAAATCCGCTCCAGCAGCGTTGGTTTCAGCGTAGGGGGACCTGTTGGCGCCAGCCT
GGGGTTGATCAAGCAGATGGAGTCGATGAACCGTGCGCCTGCCAACGATATGAATAGCAATGAGATGTTCAAAAAGTTCGGTTTCTAA
>LOLKGF_00360 IncI1-type conjugal transfer protein TrbA
GTGTCTTACGTTCGTCAGCCCATAGGGGAAGACCCCTTGCAAGTCTGGGGGGCTGTTGGAGCCCTGTTTATCCTTTTCCTTGTTGTTGTATGGTTGTTTTTACCCGAGGTGGTATACGCCACC
TGCCTGATACTCCACACCTTATGGGGATTTATTGACTGGGGACCATTTCACGAATACGCAGCACCCCGATACAACCTTTTGGCCATAACAGGTAACAACGCCACCAACATCAGTTTCAGTCAG
TGGCTTAATGTTATGGATCAGACCATCAGTATTCTGTGGATCTTTCTGCTTCCGTTAACAGCATGGTCACTGTGGGAATGGTATCAGCATCCGGGACAAAGCCGTTTCACCCGCAGGCCTGTC
GATATCACCAGATTACCGCATATCTTTGCCCCCCTGTCCCCGGCGATTACTCCGGTTATGACTGATGGTGATCCCCGAAAGCTCCTCCACGGTGGAAAGCGACCTGAACGGAGAGTCGCGCTT
ACACCAGAAGCTTTTGTCGAACAACATAGCTTAATCACCAGTATGCAGCTCAATGTTGCGGCGACAAGACAATGTTTCATGGCACAACTGGGTCACCCTCTGACGTCATGGAGGGACATGG
CACCACACGAAAAAGCCCTCTTTGCCATATTTGGACTTCAGTATTTTCTGGATGACCGCAAAGCGGCATTAAGTCTCATGGATGCCCTCAACCGCTCCTGCCGTCTCAAAAGCAAACGGGATA
ACGGAAGATTCAGTACTCCCGTTTATTCGCTGGCCAGAACAGCATTCAGCAAAGTGATCAAAAGCGAGGGCGCACAAAAGTGGCTGAAACAACATCGCTACGTCCGGAGCGGTCTGGTCT
GGCTTTACGCACATGATCTGCGGCTTACGCCGCCCAACTGGCTGTGGCTGAAAGGTGTGGATCGGACGTTGTTTTACGCATTACACCGCGCCAATACCACGAAAGGTTTTATTGAGGGTGC
CGGTGTGGTGGCGGTTGCCCGTTCAGAAAATGAAGCCATGCGTTTTGGTCTTCCCTGCCCTGAGCCCTGCGTTGATGAGGCAGTGGAAGGTCTGCGACGGGACATGCTCGGTCTCGGCCT
TATCTGGGATGAGCCACAGCCCGACCGCGACAGAAGACGCCGTATCCTGACCAACTGGTCCCTCACCGACGACATACTGCCCCGTACACCTGCAACAGATAACGAATTTTAA

>LOLKGF_00365 hypothetical protein
ATGACACCCCGTTACAGTGCAACCCGTACAGCTAAAAATATCATCGTATATGCGCATCATGAAGATGGACGTCAGGAAGTTCTGGCCATCCTGCAAATTAAGGATGGCGTCAGTATGGTTTCT
GACAAGGAAAAACTTTTGCTGGTATCTCCGGAATATTCGTATCGGGATACAGAGACATCCCTGTTCGCGTGTTCAGGCCACAATGTCAGTGATATTCTCGGCGCTGTCGCGGACTGCCTTGTA
AACGACACCAGACGTCGTAAGAAAAAACTACGCGGTATTGCTTACTGTGCTGTCAGCCTGGCCCTTGGAGCCCTCATCGCGTCCTGTGTTTTCCTGACAAGAGTCCCTGTTCCGTTTAATCAA
CCGCCGGCTTCTGCAACCAGCCAGGTGCTACCGGCACAGAATCCCGTGGATGATGATATTCAGGCGTCTGTTTCTGCGTCAAGCAGGCCGCTGCCGGTTACTCTGGAGAGCATTCCACGAG
TAAATAACAAGTCCGGAAAAACGGTTTTACAGCCAGAGGCAACAAAAAAAGAGACGCCGGAGGCCACGAAAGCCCGACAAAATCTGGCTACGGTGCTGAAATGTAATGCCGACCGGGG
AATGTTCACTGTCAACTTATCGTCAGGTCATGAAAGGACACTGTATGCCTTTCTCGATCCCCGCTGTCCGAACTGCCGGGCACTGGAGCCGGCACTGAAACGACTCTCTGCCGACTTTAATGT
GGTGGTCTATCCGGTATCTGTCATAGGAGGCGATAAATCAGCCTCCCACATGGCACCGTTGCTGTGTGAAAAGGACCCGCAGAAGCGCGCAGAAGGATGGCACCATCTGTATTCGGCGGAT
GCCGGCATGATGTTACCGGGAAGTACCGAAGCAATCCCTGATGAAACATGCCTGAAAGCCGCCAGGGCAGCCATCGACGTCAACAATCTGGCTTTCCGCCAGTTTGGCTTTGCCGGCACAC
CATGGGTGCTGTCCGATACAGGCTGGCACCTGCCGACAGGACTTCTTCAGGAGTCCGGCACTGTAAAGTTATTCCTGAAAACCACTGACAGCGAGTCTGGCCATGAGTGA
>LOLKGF_00370 TraG-D-C domain-containing protein
ATGAGTGAACATCGCGTTAATCCTGACCTCATACATCGCACAGCATGGGGAAACCCGCTCTGGAACGCGCTTCAGAACCTCAACATATATGGAATGTGTCTTGCAGGAAGCCTGGTAGTCAG
CTTTATCTGGCCGCTTGCATTACCGGCCTGCCTGCTGTTCACGCTGATAACTGTTCTGATATTCAGCCTGCAACGGTGGCGCTGTCCCCTTCGTATGCCCATGACGCTCAGGTGCGCAGATCCC
TCTCAGGACAGGATGATCAAGCGAAGTCTATTTGAGTTCTGGCCAACCCTGTTCCAGTACGAAACCATCAAAGAGTTTCCGGCCAACGGGATATTCTATGTGGGCTACCAGCGAATCCGGGA
TATCGGCAGAGAATTATGGCTGAACATTGACGATCTTACCCGCCACGTCATGTTTTTTGCCGCTACAGGCGGCGGTAAAACAGAAACTATCTTTTCATGGATCATCAACCCTCTTTGTTGGGG
AAGAGGCTTTACGCTCGTTGACGGTAAGGCTCAAAATGATACAGCAAGAACAATCTGGTATCTGTCCAGACGTTTTGGCCGTGAAGATGATGTTGAGGTCATCAACTTCATGAACGGGGGT
AAATCACGCAGCGAAATCATTCTGAGCGGCGATAAATCCCGCCCCCAGTCCAATACCTGGAACCCCTTCTGCTACAGCACCGAAGCATTCACTGCTGAAACAATGCAGTCCATGCTGCCGCA
GAACGTACAGGGCGGAGAATGGCAGTCCAGGGCAATCGCCATGAATAAGGCACTTGTGTTCGGAACCAAGTTCTGGTGTGTACGTGAAGGCAGAACGATGTCCCTGCAGATGCTGCGGG
AACATATGTCTCTTGAAGGGATGGCAAAACTCTATTGCCGGGGTATCGACGACCAGTGGCCGGAAGAGGCAATCGCGCCGTTACGTAACTACCTGCAGGACGTCCCCGGCTTTGATATGTCA
CTGGTCCGGACACCATCTGCCTGGACGGAAGAACCCAGGAAGCAGCATGCTTATCTTTCTGGCCAGTTTTCTGAAACTTTCAGCACATTCACCGAGGCTTTTGGCGATATCTTTGCAGAGGA
CTCCGGTGATATTGACATTCGTGACAGCATACACAGTGACCGGATTCTGATGGTGATGATCCCTGCCCTGGATACGTCTGCACATACCACATCAGCCCTTGGACGCATGTTCGTTACCCAGCAA
AGTATGATCCTGGCCCGTGACCTGGGATACCGTCTCGAAGGTTCTGACTCTCAGGCACTGGAGGTGAAAAAATACAAAGGACGTTTCCCGTACCTGTGCTTCCTGGACGAAGTTGGCGCTT
ATTACACTGACCGTATTGCAGTTGAAGCTACCCAAGTCCGCTCCCTGGACTTCGCACTCATTCTGATGGCACAGGATCAGGAGCGTATCGAGGGACAAACATCAGCCACCAGTACGGCAACA
CTGATGCAGAACACAGGGACGAAATTTGCCGGCAGGGTCGTCAGCGACGACAAAACGGCAAGGACGCTGAAAAATGCGGCAGGAGAAGAAGCGCGGGCCAGAATGAACAGCCTGCAA
CGTCAGGACGGCGTGTTTGGAGAGGCCTGGATTGACAGCTCACAGATAAGTATCCTGATGGAAAGTAAGGTAAACGTCCAGGAACTGATAAAGCTGCATCCCGGCGAGTTCTTTTCCATTT
TTCAGGGAGAATCGGTCCCCTCTGCATCATTCTATATTCCTGATGAGGAGAAAAGCTGTAGCAGTGATCCTGTCGTTATTAACCGCTATATCAGCGTGGATGCGCCACGTCTCGAGCAGCTTC
GCCGTCTGGTTCCCCGCACGGTACAACGCCGTATTCCTTCACCGGAAAATGTCAGCGCTATCATTGGCGTACTGACAGCAAAACCTTCCCGTAAAAGGAGAAAAAGCCGCACGGAACCACA
TATTATCGTGGATACTTTTCAGCAACGGATCGCAGGCCGGCAGGCAGCCATGGCACTACTGGACGAGTACGATACGGATATCAACGCCCGGGAAATGGCACTGTGGGAAACTGCACTGAAT
ACCCTTAAAACGACCACACGGGAACAACGGCGAATCCGCTTCATCACTCTGAATCGTCCGGAGACGTCGGCAGCAGATGAGGGGCATCAGGTATCAGTCAGGGCAGAGAGAGCAGAAAT
TAACCTGTTATCTATGCCAAAACAAAATTACCGTCCTGCAGAACCTTCTGCGAACGGCTCCCGCCATAAAAATAACAGTCGTCACGAATGGAAAGGACTGTACTGA
>LOLKGF_00375 CaiF/GrlA family transcriptional regulator
ATGCCTGCGTTACGGAGAGCACGGAAAACGGTCCTGGAGCGTAACACTGCGGTATGGGTATGGCAGCAGGAAAGACCGGTGAGTGTCGCTGAAATTGCCGGGCAGTTTTTTATTCCTCTT
TATGTGGCAAGGAAGGTGGTACATAACCTGATGCGGAGTGCAGATGGTCTGCGCTGCAGGCTGGAAACAGTCCGGGGAATTAACAGTGCCGGGCACCGGGGACTGGTAAAATATTTTTCG
GTGCAGCACGTTCCGGAAGAGTACCTGCCGGGTGGAAGGATGAAAACCGCAGAAAGGGGATTACAGAATGATGCAGGATTTTTTCCTCCGGAACAGCAGATGCCGGAGGAGTGA
>LOLKGF_00380 hypothetical protein
ATGAGTGACATTCTCAGCGCCTTTGAGCCGGCATCGTTGTTTATCCTGAAAGTGGATATTGAAGGCGGAGAGAAAGATTTATTTTCGGGAGATGTGTGCTGGTTTGATGATTTTTATCTGTGC
ATTATTGAGTTACATGACTGGCTGTATCCGGGAGAAGGAACATCAGGACCGTTTCTGAGGTTGTGCGGGCAGAGAGACCGGGATTTCATTTACCGGGGCGAGAACATTTTTTCGGTAAGTA
ACCGTCGGGAATGGTGA
>LOLKGF_00385 Methyltransf-21 domain-containing protein
ATGACAGAGTTGATGAAGGGTGATATTACCGTGCAGGGCAGACAGCGGGTATTTTACTGGCGGGATATCCGGTCAGACCGTGCTGTTATCCGGCAGAACTTTATTGACCGGGAGTATGCGA
CAGGAATGTTTATACGGGATGCGGATATCACGGCACGGTACCGTTCGATTCTGGAGGCCGGACTGACGCCGGTGATAGTGGACTGCGGGGCGAATATCGGAACGTCAGTGCTGCATTTTCT
GGAGGCATATCCCCGGGCGCATGTGGTGGCGATTGAGCCGGCACCGGATAACTTTGCCCTGCTGTCCCGCAATACAGAAGGTCTGAATGTAACCCTGATAAATAAGGGGGTCTCCTCCTGC
GCAGGAGAAATGACACTGGTTGATACGGGAGAGCCTTTTGCATACCGTCTGACAGACAGACAGACAGACAGACAGACAGACAGACAGACAGACAGACAGACAGACAGACAGACAGACAGGAT
ACCGGTTATTGCAATGA
>LOLKGF_00390 HTH luxR-type domain-containing protein
ATGAGCCGGGAGACAGTGCCGTCGGTGGTGATTCTGGGGGAGGACTGGACGCTGACGGCGGCGGTCCGGGGACTGATATCAGAGCGGTGGCCCGGGGTGGCCACGGCGGAGGCCGG
TTCGCCGGCCGGGCTGTCAGCCTGGCTGGAGCGGTATCCGGCGGCGGGAGTGCTGCTGTGCCTGCGTCCGCATGAGAGTCTGCCGTGGCTGGACAGCCTGAGCCCGCTGCTGTGGGACC
GGGCTGTCAGGGTTGTCTCGCCGACGGTCTGGTACAGCGACAGGATGGTACTGGGCTATCTGGGATACCGTCGCGCAGTGAGTACGGATACGCTGACCGGGTGGCTGCCGGGAAGGCAG
GGCGCCGGGAGGTGCAAAGGGGGGCATCCGCTGTCGGGTTTTATGGATGAGGTGGTGCGTGGCGCGGAGAAGCGCCGGGGGGGAAAGGAGCTGCCGGTGCCAGGGCGGATGAGCCG
GACACGGACGGCGTGTCTGGTCAGGGAGGTACAGAAGGAGTCGCTGAGGATGCTGCCGGCGCAGGTGACGGCACGGCAGTGGTTTATTTTATGCCGTCTGACAGACGGGATGAAGGGG
GGGCAGGTGGCGGCGTTGACGGGGCTGCAGGAAAAGACGGTAAGCCTGTACAGGCGGCATGTGTTGTGTGCGCTGGGTATGGAGGAGGTGAAAAGCGGGATGCCGCTCTACCGTGGC
GTGATGGTACGGGAGTCTCTGCAGCGTTACCCGTCAGGAGCCGGATTAACGGAAAACAGATACGGTCAGCCGGGGAGAGAAGAGCGAATGGCAGATAAAGAGGGAGAGGTATGA
>LOLKGF_00395 hypothetical protein
ATGACGGGCAGGGAGAACAGAAGGGGGCGGAGCCGGCGCCGCGAACCGCAGCAGAGCAATCACGACGTGTGCGTGGTGCCGGAGGAAGTGTCGGCGCATGAGGGGAAGCCGCTGTA
TGTTCTGGTGGCACTGTGGTGCCTGAAGCGGGGGGGATGGGTGAACCGGGGGCAGATAGCAGATGCCTTCGGTATCAGTGAGCGGAGTGCGACGTTCCAGCTGACCTACCTGTCGAGAA
AAAAGGAGCAGATATGCTGCGAGCTGAGAAAAGTGAAGCGCGCGGGGGTACCGGTGGAGAGTTACGAGGTCAGGGTGACGGAGGTGAGTCCGGAAGCGGGCGTACGGAAAGTGTCG
GAGAAGCAGCGGGAGGCGGTGAAAACCATTCAGCGCGGTCGGGTGGGGAATGCTGACGGGGACGTCAGGGAGCTGACGAGGAATATCTGGAACAGTCTGCAACGGGGGAGAAAGGC
ATGA
>LOLKGF_00400 Transcriptional regulator
ATGGAGAAGTCGGTGTTTTACCGGGAAGTGGCGCACCGTACAGAGTGTCTGCAGATGAGTGTGTCCCGGATGGCCGTGGCGCGGTGGTGTGACAGCTCGGAGCACCGGGAGGCGCTGT
GGCAGATATGCCGTGACACGGCGGCGTTTATGGTACCGCCGGCAGAAGACGGGGAACCGGCCTGGAGAAAGGCGCTTTGGGCGCGGTTGCAGGAGACGTCTCCGGATGCACTCCGTCA
GTTGCTGGCGCTTTCAGGTGGGGCGGTGCTGCGTAACCAGCTGGCCCGGGGAGAGGTGTATGCCGGCGCCGTGCTGCACAGCCTGCTGAAATCGTGGCTGTCGCAGTACGGCCGGGGA
AAGGAGAGGATGCGCCAGGCAGCTCAGGGCGTGACGTCAGTACGGGGATACGGGGGAGGGACGGGATGA
>LOLKGF_00405 DUF2919 domain-containing protein
ATGAAGGAGCAGAATGTGATGGCGGGGACACGTTCACCGGGGCGGCGTTACCGGGCGGAGGACTATGACGACGAGGGGAACCTGAAGGCCTCATTATGGATATGGCCCGGGGTGGCAT
GGCTGCTGCTGCCGTGGTGGCTGACGGCGGCCGGAGTGATGTCAGGCAGCACGCCGCTGATGGCGGAGGTGCTGTACCCGACGACGGCGGATGCGGTGTTAAGCCTGGTCACGGCGCT
GCCGGTGGTGGCGCTGTGTGCGGTGTACCCGCTTCGGGGGAGGATACCCCGGGCGTCGCTGCTGACATACGGGGCGGTGTTTACGGGGCAGGTGGTTGAGCTGTTGCGGACCGGGAGC
GTCCTGCTGCGTGAGACGGGATGGGGGCATGACGGGACTGACTTGCTGCTGAGCTGCCTGTGCGTGGATTTTGCGGTCCTGCTGGGCATGTTGCTGTCGCCGCGGTTATGGACAGTGTTT
GGCTGGCGGCGGTGA
>LOLKGF_00410 EtpC

ATGAATATGGATACCGAACTGCCTGCCACTCCGGCCCCGTCCGGAGAGGCGGCTCCGCTGCCGGCGGTAACCCTGTTTAACCTTGAGCATTTTGAGTGGCGGGTCCACACGGCGCAGACG
GAGCTGGCCTGTCGTGAACTGATGTTTCTGCTGGAAGAGCTGGACCGAAACTATGGCCAGTGGGGAGAGCGGTTCACGGCTTTTGCGCCCGGCCAGACGCCGTACGGGCTGAACCGTCA
TCTGTGCACCCGGCTTGCAGGGGCGATAACGACCCTGTTTTCCCGTCCGGGGTTCAGCATCAGCGACACGGGCTATGTGCGGCTGATGAACCTGCACCGCTGGCTGGCGCTGATTTTTGCG
GTGAGCAGCTACCGCCATGCCGACCACATCATCCGTAACCTGAATGCAGCCGGCGGCGGAGTGGTTGACCCGCTGACGCTGAACGCCGGGAACCTGCGGTTGTTCTGCCTGTGTTATTTCC
CGGACTCGGAAATCGCCCTGCAGCCGGACGTGCTGTGGCAGTACGACCGGGAGACGGTGGTCCGGCTGTTCTGTGCGCTGATTAGCGGGCGCGCCCTGCCGACGCCGGCGGCCCATGGA
AAGCGCGAGCAGTTACTTGCCTGGCTGCCGGACAGGCTGACGGAGCTGGATTCGCTGTCCTTTCTGCCGGTGGCTGTGCTCCATGATATGTACATGCACTGCTCGTATGCGGATTTACCGGA
AAAGCACGCCATCAAACGCAGCATCAATGCGCTGGTCAGACGGACGCTGGAGCAGGAAGGGCTGACGGACCGGAGCGCGGAGACGGCGGAGGAAAACGGGAAGCCGGTGATGATGG
TGGTGCTGGAGCATTTCACACGCCAGCACAGCGTGTACCGGACGCATTCTGTGGCGTTCAGGGCGCTGCGGTCCCGGTTTACGGTACATGGCGTGGCACTGGAAGGCTCAGTGGATGAG
GAGACGGCACAGGTGTTTGATGTGTGCCACCGCGTCCCGGCCGGTGATGCGGTCCCGGCAACCCGCAGACTGGCAGAAACACTGAAGCCGGCGGTGGTTTTTTATGCCGGGGTGGGGAT
GTTCCCGTACACCATCTTCCTGAGCAATTTGAGGCTGGCGCCGCTTCAGCTGGTCGGCCTGGGGCACGGAGCCTCAACGTTCTGCCCGGCCCTGGACGGTTTTGTGGTGGAGGAGGATTT
TGTTGGCGCGGAGGCGTGCTTCAGCGAACGGGTGTATGCGGTACCGGTGGACAGTATGCCGTTTGTTCCGCCGTCACAGACGCAGCGGGTGTTGTCGGTGCGCCGGTCATTTGAAGAAA
GACGCCTGCTGGCAGAGCCGTTTGCGCTGCCGGTTCGTATAGCGGTCTGCGCGTCCATCATGAAAATCAACCCGGGTTTTCTGCAGACGCTGGCGGAGATACAGCGCCGCAGCCGGGTCC
CTGTACAGTTCTGCTTTTACATGGGGTTTGCACAGGGGCTGACGCTGGATCACCTGCGTGAGGTCATTCTGTCCGTGTTGCCTGATGCGGAAGTGAATGCCCATATGGCCGTTCAGGATTAC
CAGCAGGCACTGAACAGCTGCGAGCTGTTTGCGAATCCGTTCCCGTACGGAAACATGAACGGGGTGGTGGATGTGGTACGGCAGGGGCTGCCGGGTGTTTGTCTGTCAGGCCCGGAGGT
GCACACGCATATTGATGAAGGGCTGTTCCGCCGCCTGGGGCTGCCGGATGAACTGATTGCTGACGACCGGGAGGCGTATATCCGGGCGGTGCTGAAGCTGGCGGAAAATACCGGCTGGC
GGGAGTCACTGCAGGTACAACTGCGGGACAACGACCCGGAGCAGGTGCTGTTCCGCGGGCATCCTGAAAAGTTTGCTGAGGTGGTGTGGCAGGCGTGGATGGCGCGACAGGTGCCGCC
GGAAAAACCGGCAGGCCGGGGTAAAGCCGGTAAGGGGGGGACCCGTAAGGAAAAGGAGACACCATGA
>LOLKGF_00415 Haemagg-act domain-containing protein
ATGAACAAGATATACAAACTGAAATATGACCGCCGCCGCCAGCAGCTTGTGGCGGTAAGCGAGCTGACCACGGGGGCCGGTAAGGAAGCCACGGGTCAGGTGTGTGGTCTGTCAGACAT
CAGCAGCTTCCGTAAACTGCTGGGAACCCTGACGCCGCTGGCGTTCCTGACGGGGCTGATAGTGAGCCTGCTGCCGGGGATGGCGCTGGCGAACCCGTCACTGCCGACCGGCGGACAGA
TAGTGGCGGGCCAGGGGAGTATCAGCACCAGTGGCAACCAGATGACCGTGAACCAGAACACGCAGGGGATGGTGACCAACTGGAACAGCTTTGATATCGGGAAGAACCACACGGTACA
GTTCGTGCAGCCGGGCAGCAGCGCGGTGGCCCTGAACCGTGTGACGGGCGGCCATGAGTCGCAGATCCTGGGGACGCTGAAGGCGAACGGCCAGGTGATGCTGATTAACCCGGCCGGG
GTGATGTTCGGTAAGGGCGCGAAGGTGAACACGGCGGGCATGGTGGCCTCGACGAAGAACATCAGTAACGAAGACTTTATGGCGGGCCGCTACACGTTCAGCGGAGGGAGTCAGCCGG
GCGCGGAAATCGTGAACCAGGGGAGTCTGACCACGACGAAAGGGGGGTATATCGTTCTGGCGGCGGACCGGGTGAGAAACCAGGGGGAGATACGCACGCCGGGTGGCCGGGTGGTGC
TGGCGGCGGCGGACCGGGTGACGCTGCAGCTGGACAACACGGGGCTGACTGCTGTGTCGGTGAACGGGAGCGTGGTGAACGCCCTTGCGGAGAACCGGGGGTTAATCTCGGCGACGA
ACGGTCAGGTGTACCTGACGGCGCGCGGGAAGGACATGCTGCTGAACACGGTGGTGAATAACAGCGGGACGGTGGAAGCGAAGGGGCTGAGCGAGCGCGGGGGAGAAATCGTACTG
GACGGCGGTGACAGCGGTGTGGTGAGCCAGTCGGGGATGCTGCTAGCAGACAGCGACAGCGGCCGCGGGGGTAAAATCACGCTGGAGGGACAGAATATCCACCTGGCGGGCGGGAGC
CTGATATCGGCGACGGGTGAGACCGGCGGCGGTGAAGTGTATGTGGGTGGCGGCTGGCAGGGTAAGGACAGCAGCATCCGGCATGCGTCGAAGGTGGTGATGGACAAAACTGCGGTGA
TTGACGTGTCGGCGAAGGCGCGGGGCCAGGGCGGCACGGCGGTACTGTGGTCGGATGATTACACGAACTTCCGCGGCACCATTCTGGCGCGGGGCGGTCTGCAGGGGGGAGACGGTG
GCCGGGTGGAGACGTCGTCGCACCATAACCTGCAGGCGTTCGGGGATGTGGATGCATCGGCGGTGAAGGGGAATGCCGGTGAATGGCTGCTGGACCCGTTTGATATCACGGTTGTATCCG
GCTCCACTGATACTGATGTGAATGAAGGCAGCGGTAATGACGGTATCTTCACGCCGGACACCGGCACCTCGCAGGTGAGTAACGGCACCATTAATAATCGCCTTAACAGTGGCACAAACGT
GACCATTAAAACGGCCAAAGAGAACAGTGGCTCAAATCAGTGGGGCAATATCACCGTTAATGCGGATATCAGTCATACAGCCACTAATAACGTCAGCCTGACGCTGGAAGCGGACGGTAAC
ATCAATATCACGAACCACAGCATCACGTCGACGACAGGTAAGCTGGATGTGAACCTGCTGGGCGCGGGGAGCCATGACGGCACCATCACGCTGAACAATGCGACGGTGAGCAGCAACGG
CGGGAACATCACGCTGGGGCAGCTGAATGCCGGGAGTGATGGCACCACTTCAGGTCTGGCGGTCAGCATCACAAACAGCACCCTGAATGCGACGTCGGCCGGGAATATCAGTATCACTGC
GAACAATGGCACGACGCTGGGTAATGGCACCCTGAGCGGGAATGAGGTCAGCGTTAGCGCCAGCAGTGGTACCGGCGATGCGCTGGTAATTAACCTCAGCAGTAACCTGACTGCGGCCG
GTAACCTGACGGTGAGCGGAGACACTTCGGCAACGGGCAATAAATACGGTATTCACACCAGTGATAGTCAGTTCACGGCCGGTGACACGCTGAATATGACGGCAGAGGCTGCCGGCGGTA
CGGACGGAGCCTTTAAGGCATCGGATATCAATGTCAGTGCACAGAATGCCGTGATGAAGGGGACCTCGGGCGGAGCCGGTGGTACGGGTGTCTGGCTGACCGGCAATGTCACCAACACC
CGGGACGGCGGTAACCTGACCATCACCGGGACGACGACCGGTTCAGGAAACAGTATCGGAGTGAATGTGCTGGCGAACCTGACGGCAAACGGCACGGGTAATCTGAACATCACAGGGA
AAGGCGCGCAGGGTGTCGGGGTTAAGGTGGACGGAAAACCCTGACCGGGGGTAATGTGACGGTGACCGGTACGTCCGGCAATAGTAACGGTAAAGGTCTGGAGGTGAAAGGTGGCA
CCCTCAATGCCACAGGCGGGAATATCGCACTGACCGGGTGTATGACCGGTCGCAATGGCGGGTTTGGGGCGCATATCTATAGCGGCAGCAGCTTTACGGCCAGGGATAATATCACGATAACC
GGGTATGCCATGGATGGAACGAATGGTGGCCTGAACCTGAACGGGGGGACATTTAACGCGACCAGTACGGTGCTGAACGGGACCTCACAGAATAATAACCTCGGTGCCAAAGTGGGCGG
GGTGATTACGGTCAGCCAGGGTAACCTGAGCCTCAGCGGTACGGCGAACCGCGTAAACTCAGCCCCTAACGTGACCGGCGTGGTGTCAGACGGCACGCTGAGTATCACAGTCAGCAGTG
GCACCCTGAACGTTACCGGTAAGGTGAATGACACGGCTAATAATCCGACTAGTGCCAGCACCACCAGGGGCCTGAGTCTGGTTAACACCACCCTGAATGCGACGGAGGTCAGCCTGTCAG
GGGAAGTGGCGGGGGGGCGTGATGGCACCGGTGCATCCCTGACGAACACGACGATAAACGCCACCACAGGCAATGCCACCCTGAATGCGACTGTGGCGAACGGTAATGCGCTGGTTGTG
TCGGGCGGCAATATTACCGCCGGGAAGGACATCAGCCTGACCGGTACGGCGAAGGCCGGCTCGGGGTATGGCGTCTCCCTGACCAACGGAAACATGACAGCCTCGTCGGGCAATATCAGC
GTGAACGGCACGGGCTTTGATTCAAACCGTGGAGCCCTGAACGTGAACGGCGGCAACTTCTCCGCACAGAATACGGTGCTGGAAGGTACCGCAGGCCGTAATAATAAAGGCACGGTGCT
GAATGGTAACATCAACGTCACACAGGGTAACCTGGCGGTGACGGGGACGGTCACTCAGACTGCCAACAGCCTTGATAATAATCAGTTTACTGGTTTGCTTGCGTCAGGGAATATCACAGTC
AGCCAGGGGAGCCTGAATCTTACGGGACATATTACCGGGAAAAGTGGCTTGAATGCCAAGGCAGTTAACCTGACGAACCTCAATATCAGCGCCAGTAATGCCAGCATTACGGGGGTTAACC
AACGCAACGGAACAGGTTTTATTCTGAGCAATGTAAATCTGACCGGTGACATTGAAAAGGGTGCGAACACCACCTTTAGCTCTGCGGGAAGCGACTCGTCAGTGACCAACGTGATTGGTA
GCGGGGTACTGAACGCGACCACTACAGATGTACTGATGCGGGCTGGTATTGAGAACGACACGCAGATTAGCGCGTCGGGTATGGCACTGGGAGGAAGCGGTGATAACTGGAATCAGAAC
TATACCAGCAGCAAGGGAGGGGGCTGGATATTCGATGGTGCCACAGTCAGCAAGACGGGGAATATCAGTCTGCAGGGGGTGGGTTTTGTCAACAGCAGCGTCACGGCGGGTAAGGACCT
GACCATTAACAACGGCGATGACTCGCTGACAGTACAGAACACGACCCTGAATGCGACGACCATCAGTCTGTCTGGGGGAAGTATCAGCCTGACAGGCAACAGCAGTGTGAACGCTGTAAA
TGATATCACGCTGAACGCCCCAAAAGGGGGAGTAAATATAAACGGGCAGAGTAATGTCAACACGGTGAATATCAGTTCAGAGAGTGGCAATATCAGTATTGAGGGGAACGGGGCAGGATT
TAACCGTGATGGCGTTTTAATCAGCAACGCTTTATTAAATGCCAGTAAGGGGGGAATTACTGTCACAGGTGTGGCTGATGGTGCTGATTATCGCTTAAGAGGGGGAGTGAAGTTTACTGATA
GTGTGAATCTGACTTCACAATCAAACACGATTAATGGCACGCATCAGAGAGGCAACGCAGGCAGAGTGAATTTTGCAGGTGTGGTAATAAACCCAGGAAATTATCACTTTAAGGGTAATAC
GACTATTAATGCAGAGTCTGACGATTATGCAGGGCTGGCCTTTAGTTCATCTGTTCCCAGAACTTACATAACGTTCTCTGATGGTAACTTTGTTATTGATGCTAAAAATAACAGTACTGCGTTCA
ATAGTATAGGAGGGATTTCTATTGATAACTGGGAGGGGACTCAGGCAAAAATTATCATAACAACGCTGAACGGGACGCTGAATATTTCCGGACAAGCCCGAAACAGAGAGGGTATTAACAG
TGTTTCCTCGAGTTTTTCTATCAACCCCAATGATGGTAGCGGATACCACGGATATGTATTTAAGGGCGATGGTGATGTCAATGTAAAAGGGGTATCAGATTCGTCAAACGGTGTGGATATGCGT
TTTTTTGATAATACCGGTGTAACCGGTAACTTCACCATTACCGGGGAAAGTAATACGGGTAACGGGGTGGCTGTTCCTAAGTTTGGTGAGATCAGTCTTGTAAATACCACAATTACCGGCAGC
AGTAAGAGTGGAGCCGGGATACTGATGAATGCCAGTGACAAACAGATTAAGAAAATTGACCTCAACGGCAATACGCTGACCGGGACATCTGTGAGCGGTGCTGGTATTAAAATTAACGGTA
ATAATGTTTCCATCGCCAACGGTTCCCTGAACGGAACCTCCCAGGGTAACGGGGCCGGCGTTGAACTGACCGGGGGAAATAACTACACCGTCAGTCATGTCACCGTCACCGGTCAGTCTGC
CGGCGGAAATGCTGTCGGGGTTAACGGTAACCTGACGCTGAATGACAGCACTCTGAGCGGTAATACCGCGAATGGCAGTGGTGTCAGCGTTTCCGGCAACGTGAACTCCACCAACACCAC
CATCAGCGGAGGGGCAACCGGCAGTGGTAATGGTGTGTCGCTGGGCGGTAATGCCAGCATCACTGACGGGACGGTGACCGGGAATGCTGTGGACGGTAACGGTGTCAGCGTTTCCGGCA
ACGTGAACACCACCAACACCACCATCAGCGGAGGGGCATCCGGCAGTGGCAATGGTGTGTCGCTGGACGGTAATGCCAGCATCACTGACGGGACGGTGACCGGGAATGCTGTGGACGGT
AACGGTGTCAGCGTTTCCGGCAACGTGAATACCACCAACACCACAGTGACAGGTAATGCCACAGGAACGGGTTCCGGGGTTGACCTGGCGGGCAACGTGACCGGGGGCACCGTGAACG
GTAATGCGACAGACGGCACCGGGGTGAACGTGAGCGGGGACAGCACGCTGACGGACGTCACGGTGAACGGTAACACCACCAGTGGTACCGGCGTGGATGTTAACGCGAACCTCACTAA
CCAGGGCAGCACCACCGTGAACGGTAATGCCACAGGAACGGGTTCCGGGGTTGACCTGGCGGGCAACGTGACCGGGGGTACCGTGAACGGTAATGCGACAGACGGCACCGGGGTGAA
CGTGAGTGGGGACAGCACGCTGACGGACGTCACGGTGAACGGTAACACCACCAGTGGTACCGGCGTGGATGTTAACGCGAATCTCACTAACCAGGGCAGCACCACCGTGAACGGTAATG
CCACAGGAACGGGTTCCGGGGTTGACCTGGTGGGCAACGTGGCCGGGGGCACCGTGAACGGTAATGCGACAGACGGCACCGGGGTGAACGTGAGCGGGAACAGCACGCTGACGGAC
GTCACGGTGAACGGCAACACCACCAGTGGCACCGGCGTGGATATCAGTGGCAACCTTACCAACAAGGACAACACCACCATCACCGGTAACTCCGGCAGCGGAGCCGGCGTGGGGCTGAA

CGGCACCGTGACGGGCGGCAGTCTGGCCGGTAACTCGGTCAGCGGTCCGGGGCTTCACGTCACGGGTAACAGCACCCTGAACGGCGTGGACGTCACGGCCTCTTCGCAGAGCGGCCCG
GGCACGCAGATGGACGGCATGCTGTCGGTGTCGGGTGGTACGACACTGAACGGCGAGGAGCAGAAGGACTCGGCAGAGTTACGCCGTCAGGTGTATGAGCGTCAGCAACAGCTGTCAC
GGTCTGATACCGTCCGGGATGCCTATCGCACCTCCGGTTACCGCGTGGAGGAAAAACCGGTAAGTGTGGAAATCTGCACGGACGGTGAATGCCGTGCGCTGGAAACGGGATACGCGGAC
GCGCCGAAAGCGCGGTAG
>LOLKGF_00420 ShlB/FhaC/HecB family hemolysin secretion/activation protein
ATGGTGTTCTCCATTACCCACAGGAGAGGGATCTGCTCGTCCCGCATTCCTGCCCGCTGTCGTCTGTCCCCGCTGGCCGTCATGGTCCTGCTCGGGGCTGGCATGTGCAGCTCCGGTGCCAA
GGCAGCCTTCCTCACTCCGCCCACCGGTGCCGGTCAGCTCGGTAACCAGCTGCGTCAGCAGGCAGAGTCCGCTCCGGCCGCGCCCGTCGCGCCGTCACTCTCCCTGCCGTCCGGTGACCG
CGGGCAGGCCCGCGCTCCGGCCGGGAACGCCCGTGTGACGGTGAAGCAGGTCAGCTTTACCGGGGAGGTGACCGTCCCGGGCGCCCGCAGCGTCACGGAACCCGCCCTGCAGAAAGT
GGTTTCCCCCTGGCTGAACCGGCCGCTCTCCTTTGCCGACCTGCAGACCATGACGGAGGCTGTGACGCAGTACTACCGTGACCGCGGTGTCCTGCTGGCCCGCGCGGTTCTGCCTCCCCAG
ACCATTAAGGACGGCCTGCTGACCGTCAGGGTCATTCCGGGTAAGTACGACCGTGGCGTCCTGCACAACAGCAGCCGGCTGCGCGACAGCCAGGCTGAGCGCATGGTGAACGCCACGGC
CCCGGCAGGGAAGGTGGTGAAAAAAGCCGGTCTGGAGCGTCTGGCGCTGCTGATGAGTGAAGTGCCGGGCGTGGAGGCGCAGGTGTCCCTGCAGTCCGGCAGCGTGCCGGGGACCTC
GGCGCCGGACATCACCCTGAAACCCGGAAAACGGTTCGGGGGGTACGTGGGCCTGGACAACCAGGGGGACCCGACCACCGGTCGCAGCCGCGTGATGGCCGGGGTGTATGCGAACGG
ACTGCTGGGCTACGGCGACCAGCTGCGGGTGGATCTGCTGGACGCGTATGAGAAGAGCGATCTGTTCAACGGCAGCCTGGACTACAGCCTGCTGGCCGGCGGGTACGGTACCCGGGTCG
GGGTGAACTACAGCCATCTGAACTACCACTACACCTTCCTGGGAGAAGGCTATAACGGCTACTCGGACAACTGGGGACTGTATGTGACGCATCCGTGGATACGGACGGCGCGCGCGCGGG
TGGACGTCCGTGCGGACTTCGGCCAGCAGTTCCTGACGGACAAATACCCGGCCTCGGTCTTTATGGGCGTGGGTGAAGAGGGGCGCAAGCGGGCGACGCTGGGCTCGCTGACGCTGTCA
GGGAGCGTGGCTGGCGTGCCGGGCGGCATAACCGGGTTCCGCGTACAGGGAACGGCAGGCAGCCTGGACTACCGTAACGAGCTGTCACGCCTGATTAACTTCAGTGACGAGCTGGGGT
CAGCCGGGCAGTTCGCGCGGCTGAACTGGGGACTGAGCCACGACCAGCAGGTGTGGGGGCCGTTCTCGGTGTACGCGGGGGTGAACGGCCAGCTGGCAAACCATAACCTGGACTCCTC
GCAGAAGTTCCTGCTGGGCGGGCCGGGGGCGGTGCGGGCGTACGACATCGGGACAGGGTCAGTGGACAACGGCACGGTGGCGACGGGTGAGGTGCGCTGGCAGCACGCCATTCCGC
AGACACGCTGGACGGCCTGGGCGGGCACGGCCCCGTCGGTGACGGTGGCGGCGTTTTATGACCAGGGATGGGGAGAGCAGTATGCGGAGAACCGTAACCAGAACGGTGGCGGGAAGA
TAACACCGGATAATCATGTGAACCTGGCCGGGGCGGGGCTGTACACGACAGTGGCGGACGCCGGGAATTACGCCCTGACTTTGACATGGGCGCACCGTACGGGGGATGCAGACCCGGTC
TCGGGGCTGGCAGACCGTGACCGCTTCTGGGTATCGGCGGTGAAATCATTCTGA
>LOLKGF_00425 Hypothetical DNA-binding protein
ATGGAATCAATTATTAATCATAAACTTACTGATGATGACAGGCAGTTGCTGGCGTCCTGTATTCCATTTATTGACTGCCTTGGTCAGTTTCTGGGTGAGTACTGCGAAATTGTCCTGCACAGCTT
TGAGGATTTGCATCATTCCGTCATCCATATAGTGAATGGCCATGTGACAGGCCGGGAAAAGGGCGCTCCTGTTACGAATATCGCCCTGGAGAAACTTGCAGAATTCAGTTCAACAAATGATAT
GTGGGACGTCTATTTCAGCCATAAAGGGGTACGGCCTTTTAAATCATCATCAACACTCATTGTGAATAAGGACAAAGAGCCTGTAGGCATGATATGTATGAATTTTGCTCTCGATATGCCCCTG
AACGCCTTTATTGAAAACTTTACAAAATCCAGTAACTGTAAGAATGAGAATTTCACACAGGATATTAATAACCTCGTTCTCAGTCATCTTGAGCCTGTAAAAAACATGGTTTATGCTAATAAAAA
TATCTCCAGCAAGAACAGAAATTTCGAAATTATCAAAGAGCTGAACTCAATTGGCTTGTTTAAATTTCCGGTTACAACAAAAATTGTGTCATCGGGTCTCGGGATCAGTTCTAATACTATTTATA
AACACCTTCGAACGTTGAATGGCAAGATGACTGAAAAATGA
>LOLKGF_00430 AP-endonuc-2 domain-containing protein
ATGAAACTTGGTCTTGAAACTGAGTCATATCATCTTTTCTTTCAGCAGGGTGTGATGGATATTTTCAGCTTCATTTCAAAAACAAGTGAACTGGGGCTGGATGGTGTCGAAATAAATATTATTC
CGGATGTTGGGTTGCACCCTGTGTTTGGGGTACTAAGCAGTGATTCACCTGAATATCTGGCAAAAGTCCGGAAAACTATCGAGGCATACGGACTTTACTGTGAGATAGACACCCGTTTTACT
TCTCACGAATCTGTTACCAAAGCAGTGGAAATTGCATCTGCTCTGGGAGCTGATGTTATCAGAACCTATATGTTCCGAAGGGGAGAATATAATCCACAGAGATACCCTGAAATCATTGCACAG
CTTAAAGCGCAGGTTCCGTTGCTCAGTAAAAAGCGAATCAGGCTGGCAATAGAAAATCATGAAGACGAAACTGCCGATGAGATTATTAACATAGTCAAATCGGTGGATTCAGAGTGGGTTG
GCGCACACTGTGATATTGGTAATGGCATGATGGCATGGGAAGAACCCGTGGATACAGTACGTAAGCTGGCCCCTTATGCTTACAGTACACATTTCAAAGATCATATTGTCACCCGCAACGGAG
AGGAACTGGTTGTCTGTGGCGTTCCTGTCGGGGAAGGCAGTATTGATATTGATACCTGTTTCAAAACGCTGGTTGATGAATCCTCTTTAACCCGTATCAACATTGAAACATGTTTTCCATATGC
TTCCCGTTTTTCCCGCCCGAAAGGCACGACGAATACACTGAAAGGAACGTTTACCGTAAAACCTGCACCATTTGATGAGTTAAAAATAAAACCTCTGGAGTATTACTATCCCGCAAAAATATC
TGAAGCAGTATTGCAGGAGTTAATGGAGGCTCAGGAACGCTGTGTTCAGGTTTCCGTTCAGGCTCTTAAAAATTTACGAAAAAAATATTGTTAA
>LOLKGF_00435 YjgF-family lipoprotein
GTGATCATTAAAACTAAAAATGCACCGGAAGCCATTGGCCCATACTCCCAGGCTGTTTCAACTGGTAATTTATTGTTTATATCAGGATGCTGCCCTTTCAGCCCGGAAGATGGTTCAGTTAATG
GGGGTGACATCGAAGCTCAGACGAAACAGGCCATGAATAATTTAAGGTCTATTGTTGAAGCTGCCGGTCTGGAAATGGATGACATCGTCAAAACAACTTGCTTCATCAGCGATATGAATAAC
TTCTCATTGTTTAACCAGGTATACAGTGGGTATTTCGCTTCCGGAACTTATCCTGCCCGTTCCTGTGTTGAAGTCGCCCGATTACCGAAGGATGTACTGATAGAAGTTGAAGCTGTTGCTGTTG
TCAAATAA
>LOLKGF_00440 MFS domain-containing protein
ATGAATAAATGGATAAAGCTATGCTTCCTCATACTTGGAGGGGGTACTATTTTTAAACTGTCCAGTATGAAGGACGTATTTTACGTTCCCATGCAGGCTGACTGGGGACTGACTAATACTCAGA
TTGGGTTTGGCTTTACGGTTTATGCCATTGTCCAGACTGTCGGGTTATTCAGTTCATTATATATTGCCGATCGTTTTTCCAAGAAAATACTTCTCCCGGTCGGTTTGATAGGTGTGGGGCTATGT
GGTGCTTATCTGACCACACTTCCTTCCTTTTCCGGTTATCTGATAGCCTTTGGGGCTATGGCGTTTTTTGGTGAAGTTGTCTACTGGCCCGTTCTGCTTAAAGCTGTCCGTTTACTGGGTACCC
GTGATGAACAGGGGAGGATGTTTGGTTTTCTGGAAGCCGGGCGGGGAGTTGTTGACGTACTGATAGCGTCCGGAGCTTTATTTGTGTTTGTTCATTTCGGTGAGGGAAAAACAGGCATGC
AGGCCGGCCTTGTCTATTACACGCTGGTCACTATTCTTGTCGGGATCATTACCTACTTTATTGTGGATTCAGATGACAGATCCAGTTCCCGTGATGTTGAAGATGTCAATAAACAGGTGTTTAC
GGGGATTAAGAATGTCATAAAATCACCCAATCTGTGGCTGGCCTCTTTCTGTATATTCTTTGTATATTCTGCATATTGTGGGCTGACATACTTCATTCCTTTCCTGAAAGATATCTACGCGCTGCC
TGTGGCTCTTGTTGGAGCCTATGGAATCATTAATCAGTACGGGCTGAAAATGGTGGGGGGGCCGGTTGGGGGCTTTCTTGCAGATAAAATTGCACACTCGCCGGTTATTTATCTGAAATGGA
CCTTCCTTATTTCGGCAATCGCTATGCTGCTTTTCATTCAGCTTCCCCATGACTCAATGAATGTTTATCTTGGCATGGCAGCGACACTGGGATTTGGCGCAATCATTTTTTCACAACGGGCCATC
TTCTTTGCTCCAATGGATGAAATCGGCACCCCCCGTGAATTTGCCGGATCAGCTATGGCCTTCGGCTGTATCATTGGTTATATGCCTTCCATGTTTGCCTACACACTTTACGGCTCACTTCTGGA
TAATTTCTCCGGTATACAGGGCTATAACTATGTATTTTCTGTCATGGTGGCATTCAGTCTGTTGGGCTTCGTCTGTGCAACACTGCTGACCAGAAGAATGCGGATCAAAAAATAG
>LOLKGF_00445 hypothetical protein
ATGTTCGCAAGCACCGTGGGTGATGGTTGGCCAGCCAGATACTCACCGGGAAGCTCCCGGACCACAACAACTGAAATTAGGCTCATACCCAAAGGCTCACTTCCTGTAGTGGGCCTTTTTT
TTGCTGCAGGAAAGGCGGGAACCACCCGACAGCATGATGCTGCCGGGATCGGGTAA
>LOLKGF_00450 Gammaproteobacterial sRNA STnc100
GCAGACTATACATTCAATAATGTTGTGGTGGATCTCTCAGTTGCAGGGATTGAAAATGGCCGGAAAATAGTCATGTTCGCAAGCACCGTGGGTGATGGTTGGCCAGCCAGATACTCACCGGG
AAGCTCCCGGACCACAACAACTGAAATTAGGCTCATACCCAAAGGCTCACTTCCTGTAGTGGGCCTTTTTTTTGC
>LOLKGF_00455 Nucleoside transporter
ATGAAAAATGGCATTTATCATGTGAGTTTTGAAAGCAATCTCTCCTCCTTTGGTGAAGGGATTGTTGTTGTCAGTGATGGAAAAGTTCATGGCGGAGATATCGGATTCACGTGCCGGGGTTAT
CTGCACCGGCATGTTATGGAGGTGAGTGTTTGTCAGTATAACCGTGAGATCCCTTCTGCCCTTGGAGCGGAAGGTGATTACCAGTTGAGTCTGCAATATGAAAAAGACAGTGAAGGAAAAT
ATTGCTTTACCGGACATGTTAAAGGAGACAAGAAAAAACAAATTACAGCATACGCCGTCTTTCTTGATGAGCTGCTAAAATAA
>LOLKGF_00460 non-specific acid phosphatase
ATGAAAAGTCGTTATTTACTATTTTTTCTACCCCTGATCGTAGCTAAATATACATCAGCAGCAACAGTGCAACCCTTTCATTCTCCTGAAGAATCAGTGAACAGTCAGTTCTACTTACCACCACC
GCCAGGTAATGATGATCCGGCTTTCCGCTATGATAAGGAGGCTTATTTCAAGGGCTATGCGATAAAGGGTTCCCCACGATGGAAACAAGCTGCTGAGGATGCAGATGTAAGCGTGGAAAAT
ATAGCCAGAATATTCTCGCCAGTAGTGGGTGCTAAAATTAACCCCAAAGATACGCCAGAAACCTGGAATATGTTACAGAATCTTCTGACAATGGGCGGCTACTACGCTACTGCTTCGGCGAAA
AAATATTATATGCGTACTCGTCCCTTTGTCTTATTTAATCATTCCACCTGCCGTCCTGAAGATGAGAATACTTTGAGAAAAGATGGCTCTTACCCTTCCGGGCATACTGCTTATGGTACACTTCTG
GCATTAGTATTATCCCAGGCCAGACCGGAACGCGCGCAGGAGCTCGCCAGACGTGGATGGGAGTTCGGGCAAAGCAGAGTGATATGTGGTGCTCACTGGCAAAGCGATGTTGATGCTGGC

CGTTATGTGGGAGCAGTAGAGTTTGCAAGGCTGCAAACAATCCCGGCTTTTCAGAAGTCACTGGCAAAAGTTCGTCAGGAGCTGAACGACAAAAATAATTTATTGAGTAAAGAAGAACAC
CCCGAACTTAATTACTCAAGGTGA
>LOLKGF_00465 IncI1-type relaxase NikB
GTGAACGCCATCATTCCGAAAAAGAGAAGGGACGGCAAGTCTTCATTTGAAGACCTGGTATCCTATGTCTCCGTCCGGGATGATTTGGAGGATGAGGAGCTGCAGGCGCTTTCGTCAGTGC
AGTCTGAACTGTCCCACAGAAGCCGCTTCAGTCGTCTTGTTGACTACGCAACCCGTCTTCGTGATGAGTCATTTGTTTCACTGGTTGATGTTATGAAGGATGGCTGTGAATGGGTCAATTTTT
ACGGCGTAACCTGTTTCCATAACTGTACTTCACTGGAAACGGCAGCTGAAGAAATGGAATACACAGCACGTCAGGCCCGTTATGCCAAAGATGATACAGATCCGGTATTCCACTACATTCTTT
CCTGGCAGGCGCATGAAAGCCCACGTCCTGAACAGATTTATGACAGTGTTCGTCATACGCTGAAAGCGCTTGGTCTTTCGGAACATCAGTATGTTTCTGCCGTTCATACCGATACGGATAACC
TGCATGTTCATGTGGCCGTAAATCGGGTCCATCCGGATACCGGCTATCTCAACTGCCTTTCCTGGAGTCAGGAAAAACTCAGCCGTGCGTGTCGTGAGCTTGAGCTTAAACATGGTTTTGCG
CCGGATAATGGTTGCTGGATACACGCACCGGGAAGCCGTATCGTTCGTAAAACGGCAGCTGAACGTGATCGCCAGAATGCCTGGACGCGTGGGAAAAAACAGACTTTTCGTGAGTACGTT
TCGCAGACGGCTGTTGCCGGTTTGCGCAGTGAACCCGTGCATGACTGGTTATCCCTGCATCGTCGTCTGGCTGAAGACGGACTGTATCTGACACTGCAGAATGGCAGTTTTATGGTGATGGA
TGGCTGGGATCGTAACCGTGAAGGTGTGCAGCTTGATTCCTTCGGCCCTTCCTGGAATGGCGATAAGCTCAGGAAAAAGATGGGGGAATATACGCCAGTTCCGAAAGATATCTTCAGTCAG
GTGGGGGTACCCGGACGCTACAAACCGGAGGTCATTGCCGCTGATACTCGTCCGGAAAAAATGGCTGAAACCGTGAATCTGATGCAGTATGCCTGCCGTCATCTTGGGGATCTTTTACCTGA
AATGGCACGGGAAGGGAAGCTGGAAAGTTGTCTGGATATTCACCGGACACTGGCTGAAGCCGGATTATGGATGCGTATTCAACACGGGCACCTGGTGATTTGTGACGGGTATGATCATAAC
CAGACGCCGGTCCGGGCTGATAGTGTGTGGCCGTTGTTGACACCCGAAAATGTTAATCAACTTGACGGTGGCTGGCAGCCTGTGCCGGACGATATTTTCCGTCAGGTCACGCCTGCAGAAC
GTTTCAGTGGACGTCGTCTTGAAAGTTGCCCTGCAAGTGACAGAGAGTGGCACCGTATGCGTACAGGCCCGGGACCACAGGGTGCCATCAGGCGGGAGCTGTTTTCTGATAAAGAAAGT
CTGTGGGGGTACAGCATCAGTCATTGCAGTCCGCAGATTGAAGAGATGATCACTCGAGGGGAATTTACCTGGCAACATTGCCATGAATTATTTGCACGACAGGGGCTGATGCTCCAGAGAC
AACATCACGGACTTATTGTCGTTGACGCTTTTAACCATGAGCAAACGCCGGTTAAAGCCAGCAGCATTCATCCGGATTTGACACTGGATCGTGCCGAACCGCAGGCAGGCCCGTTTGTGAAT
GCACCGGCTGATTTGTTCGACAGGGTAAAACCCGAAAGCCGCTATAACCCGGAACTGGCTGTCAGCGATCAGTCACGGGAGGACTGCAGGCGTGATCCTGTTCTGCGTCGTCAGCGTCGC
GAGGCGAGAGCAGAAGCACGGGCAGACCTTCGTGCCCGCTATCTTGCCTGGCGGGCACAGTGGCGTAAACCTGAGCTGCGTTATGGTGAACGCAGCCGGGAAATTCACCAGGTGTGTCG
TCTGCGTAAGTCTCATATTCGTGTTCAGTATGACGATCCTGTTTTACGTAAGCTGCATTATCACATTGCCGAGGTACAGCGTATGCAGGCCCTGATCAGGCTTAAAGAAGAGGTCAGGGATGA
GCGACAGAAACTTATTGCTGAAGGGAAATGGTATCCTCCGTCATATCGCCAGTGGGTGGAAGCTCAGGCTGTACAGGGAGACAGGGCTGCTGTTTCGCAACTCCGGGGATGGGATTATCG
CGATCGTCGTAAGGACAAGTCACGAACCACGACAGCCGATCGCTGCGTGATACTTTGCGAACCTGGCGGAACGCCGGTATATGAAAACAGGGGGGAGCTGGAGGCGCGCCTGCAGAAAA
ACGGCAGTGTCCGGTTCCGCGATCGCCGGACTGATCAATTTGTCTGCACGGATTATGGTGACAGGGTGGTATTCCATAATCATCATGACCGTAACGAACTGGCAGATAAACTGGATCTGATTG
CTCCGGTATTGTTTGAGCGGGACCCCAGAATGGGCTTTGAACCCGAAGGTAACGACCGTCAGTTTAATCAGGTTTTTGCTGAAATGGTGGCCTGGCATAACGTGACAGAACGTACCGGTCA
CGGTGATTACACTATTTCCCGGCCGGATGTGGATCATCATCGAGAAAGTAGTGAGATGTATTATCGCGACTATGTCAATGTGCATGAATGTAGCGATAGATCTCAACGGTCTCATGAGGATGAA
AAAGGCTGGGAGCCTCCGACCCCTGTGTAG
>LOLKGF_00470 Conjugal transfer relaxosome component TraJ
GTGAGTGATAAGCGAGAAAGAAGTGGTAGTGAAAAACGGCAGAAGACCCTCCTAAGAGCGGTCCGGTTTTCTCCCGATGAAGATGAGGTCATAAGAAAAAAAGCTGAAGATGCCGGGCT
TACTGTTTCTGCCTACATACGAAGTGCGGCCTTGAACAAACGCGTAAATTCCCGAATTGATGATAAGTTCCTGAAGGAACTCATGAGGCTGGGAAGGATGCAAAAGCATCTGTTTGTTGAAG
GGAAAAGAACGGGTGACAAAGAGTATGCCAGTGTGCTTGTTGCCATCACTGAACTGTCTAATACAGTGCGTAAACAGTTAATGGAAAACTAA
>LOLKGF_00475 Molybdopterin-guanine dinucleotide biosynthesis protein MobC
ATGGCAGCAAAAAAGTTTTATTCAGATGATGACATTCAGTTGGCGCGAACAGCTTTATCAGAACTTCCCGATCTGACAGCACAGCGTAAAACACTTCGTGATTTTCTTGATGCTATCCGTGAC
GATATTATCGCCCTGATAAAAACAAAGGGCTATACGCTGGCTGACATTCGTAACACACTGAAAGAAGCAGGTTATGAGGTAGGTGAAAAAGCACTGCGTGACATTATTCGGGAAGCCGAAG
CGAAGAAATCAGTCCGCCGCTCCTCCGGTAAGTCAGATAAGAAAAAATCATCACAGGACAGAAAAGATGGCACTGATACGACAGGACTTTAA
>LOLKGF_00480 DUF4942 domain-containing protein
ATGAAACAGACTATGCCAGCTACAGTGCTGAATACTGCCAGTACAACAGAAGTTATCCCGTCCGTGGCGATTGACCGTATCATCGCGCAACGTAACGAAGGTATAGCGCTTTTTATGCAGGCA
ATAGAGTGCCTGGAGTCTTCGCGTAAAATTCTGCGGGAGGCATCCGGCCATGACTTTCTTTACGGATTTGAAGACGCCGTAACTGACGCTGTTCGTCGTGCTGACAAGCCGGAGGAGAACA
GAAAGAATATTTCTCGTTTTGCCGACCGTAAAATCTGGCACCGCCTGATGACGGACACTGGTATGTATACGTTCATGAGCTCATGCCAGTGTGATGAGTGGAACAAACAGCTGAAGAGTGAA
ACCTGTCCTGAAATCACTCTGGACAATGTGCTGGCAACATTTCGCCACATGAATGCCAGAAAAATGCAGACATTCGAGCAGGGGCTGATTGATGTATACCGTAATCTCTCCTGGGATTATAAA
ACGAATAATCCCTGTCGTCTTGGCAAGAGAATCATTGTCAGTAATCTGCTGTATCGCTGGAGTGACGGTCATGTATCGCTTGACCACAGTGGTCGTGAAAAAATTGATGACCTTGCACGGCCG
TTTTATCTTCTGGCAGGGCGAAACATACCTGACTTTCGCCACTCCACAGGTAAGCTATATAGTGATTTTTTAGGCGCCGGTATTAATGTCGGTGAACTGTTCGACGGAGAATATTTCACGGTAC
GGGGCTTCCTCAAAGGAACGGTACATATCACCTTTAAGTACCCTGACCTTGTCGAAAAACTGAATGACATCATTGCCAGACATTATCCAGGTGCATTGCCGCCACGTGTCTGA
>LOLKGF_00485 XRE family transcriptional regulator
ATGACAGGTTGGGAGCTGCGCCTCTGGAGAAAGGGGATGTGCTGGTCACGCGAAAAAGCCGCGCGGGAGTTCGGCGTAACTTTGCGGACATGGCACGCATGGGAAAATGCGGAACAG
GTGGATGTCACAGTCTGGCGAACCACCCAGGCACTTTCTGTCCTCGACCTGTTACCGCTCATGCATCGGATGCGTAAAACTGACATCATCACTCGTCTGGAAAATGAACTGGGGAAAACAGC
AGTGGGAGTATGA
>LOLKGF_00490 hypothetical protein
ATGACTATTGCTGAACGTCTTGAGCAGAAAGGTCGCGAGGAAGGACGCAAGGAAGGTCTCCAGGAAGGTCTCCAGGAAGGGCGTCAGGAGGGCGCTGCTGAAAAAGCACAGACTATTG
CACGACAGCTCCGGAATATGGGAATGACACCAGAGCAGATTGAACAGGCAACAGGGCTTTCCGGTGCTGAACTGAAAAAATTATTTGCTGACTGA
>LOLKGF_00495 Transposase-31 domain-containing protein
GTGAGTAAAAAGAAAAACACCACAACGCCCACGCCGCATGATGCTGCGTTCCGGTCGTTCCTGGCGAATCCCGATGTCGCCAGAGATTTTCTGGAACTGCACCTTCCGGCTGAACTACGGG
CTGTCTGTAACCTGGACACGCTGAAGCTGGAATCCGGTACGTTCGTTGAGGAAGATCTGCGCCAGTATGCCAGCGATATCCTGTGGAGCATGAAAACCACTGACGGTGATGACGGATACAT
CCATGTTTTAATCGAACATCAGTCCAGCGATGACAAAATGATGGCGTTTCGCCAGATGCGTTATGCCATTGCAGCGATGCAACGGCACCTGGAAGCCGGGCATGGCAGGCTGCCGCTGGTC
ATACCGCTGCAGTTCTATCACGGAGAGCGCAGCCCCTACCCATATTCAACAAACTGGCTGGATTGCTTCAGTAATCCGGAAGTTGCGGGTAAAATATATACAAACCCGTTTCCGCTGGTTGAT
GTCACCGTCATTGATGACGATGACATCATGTGCCATCGCCGCATGGCCGCGCTGACACTGCTGATGAAGCATATCCGCCAGCGTGACCTGATGGAGCTACTGGATAAGCTCCCTCTGTTAATG
GTGGAGATGGTATCTGATGAGCAGGTACGTGTACTGATTCATTACATGGTGAATGCGGGAGATTCACCTTCCCCGGAATTTATGCGGGCGCTGGCTGCGCGTCTGCCGCAACATGAGGATAA
ACTTATGACTATTGCTGAACGTCTTGAGCAGAAAGGTCGCGAGGAAGGACGCAAGGAAGGTCTCCAGGAAGGTCTCCAGGAAGGGCGTCAGGAGGGGCGTCAGGAGGGCGCTGCTGA
AAAAGCACAGGCTATTGCACGACAGCTCCGGAATATGGGAATGACGCCGGAGCAGATTAAACAGGCGACCGGGCTTTCCGACGCTGAACTGAAAAAGGTTATTCACTAA
>LOLKGF_00500 Transposase
ATGAGGCCTTCAATTATCATGGCAATGGCTGATTATGTAAAGCAGCAGAGCGAAGAGTGTCTGCAGAAGGATAAAAAAGTACGTCGTCAGACCCGCGTCACACGGCGGCAGATGACACCG
GATGAAATTGACCCCAAAATCCTGGCGCTGGGTTGTCATATTGTCAGACGCTGCAGCAAGCAGCAGCGGGTACATGTTCCCGCCATGCGTTCAGGCGAGTGGGGGCATTTACTCCGCGCAC
TTGAAATGAAACGTGCCTGGAACTGA
>LOLKGF_00505 Post-segregation killing protein PndC
ATGACAGTCAGCAGCACGATTTCCGTTTATTGTCGCGATGGTATGTTCCGTTCCGTTTACTGCCATCTGCACGGTGAGCCGTCCTGGAACGGCAGAATCCTGCATACCCACTATGCCACCGGT
CAGCAGGCGGAAGCCCTGATTACACATGGTGATATCCGTTGTCTCGGTCCCCGCTGTGACAAGCCTGCCGGGCATACTCTACAGCATCCGGCGAAAGGGGTGACGTCATATTACGGACGTG
ACAGCAATCTGCTGATGGACAGTGAGGCACGTGAATACCGTTCCCTGACGGAGGCTATAGCCACAGAAAGTACGCCAGAGGTGCGTTTTCATTATTTGTTCATCGATGGCTACTGGAAGGT
GATGTACCGCTCGCCGGAAGGCTGGAAGATGAAACCGCTCGCGCTTGCACTGCGTCGCTGTCAGGAATGA
>LOLKGF_00510 Antirestriction protein
ATGTCTGTTATTACACCTGCGGTATACGTTGGCACCTGGCACAAATACAACACTGGCAGCATCGCCGGTCGCTGGTTTGATCTCGTCACCTTTGATGGCGAAGGTGAATTTGTCGCGGCCTGT
CGCACTCTCCATCAGGATGAGACCGATCCGGAACTGATGTTTCAGGATTTTGAAGGTTTCCCCGGGAATATGGCGTCGGAGTGCCATATTAACTGGGACTATGTTGAAGGCTTTCGCCAGGC

GCAGGGAGAAGGCTGTGAGGACGCGTATAACATCTGGGTCGATGATACCGGCGAGACGGATTTTGACACCTTCCGCGATGCCTGGTGGGGGGCTGCTGACAGTGAAGAGGCGTTTACCA
TTGAATTTGTGAATGATACCGGGTTACTGACTGGCATTTCTGAAGAAATAGCGCGTTATTTTGATTATGAGGCGTATGCACGGGATTTATTCCTGGACAGCTTTACCTTTATCGATGGCCATGT
GTTCCGTCGGTGA
>LOLKGF_00515 hypothetical protein
ATGAAGTGGCGTTATTCGCTTCGCTGGAAACTGCCCTCTCCTTGTCCCGGCGAACAGGAACTTGTGTCTGAAGTGGTGGAAGCAGGGCAACAGGCTCCCGAATCCGTTATGTCACGCTGGG
TGGCGGGGGCCGGGTATGCCGTGTGTGTCGATTTTCTGGGGGAGAGGCAGGTTCGTCGCTGGAGTAATGAGCGTAAAGCCGCCACCCGTCGCCGGAATCTCGAACGGCGTATTAACCGG
ATTGCTCCACTGTTTGCGGATGAACTGATCACAAGGGAGCTTGAGGCACGGCCTGAATATTTCCGTGGAAAATCATAG
>LOLKGF_00520 Plasmid SOS inhibition protein A
ATGATCCCCCGTTCGCACGCCCTGGTACCACAACTGCCGGAACAGGAAGCTGCTGCTAAGGCGATTATTTATGTCGAGGAGAAAAGGGCGAAAGATCCGACATGGAAATGTTATTCCAGTC
CTTATGCGCAGGCGTTTCTCCGGTTTTTGTGCGGGAAAGGAAAAATTTCCGGCAAATCACTTAACCAGATAAGAGGCATTATCTGGGATAAGGAGGATAAAATACCGCTGTCGTCTTATGAG
CGGGCGCTTGATGATTTCATATCAAGCCGGGGACGGTATTGTCCGACCCCGCTGCCGTCAGATCTTGCCCGGTATGTGTTCCCTGAAAACTTGTTCCGACGAAGTGACCGTCAGGAAAAAC
GCCGTACAAGAGAGTTCCATCAGTATTCCCGCCGTGAACAGCGCAAAAGACAGGAGAGGGAGAACAAATATGCCTGTCTGGTCGGGCAGGCTGAAATTGACCTCGCTTTTCAGACACCGG
AGTCGTTGCGGGCCTGGTATTTAAGATGGTCGCAGTCTGACATTAAGCAATATGATCTGGAGCGGATGCTCTGGATATGGCTGGAGCGATGCCCCTCGTTGTCTCATCTGGAACGATGGCAG
TATTCAGATTGTCCTGTCTGGGTTCTGGAAGCTGACATCCGCGATGCAGCCGCATCACTGACGACGGAGCAGAAAGCCCTTGAACGCTGGCTTGTGCCTGACAAACTGACAGTGTCAGTAA
GGAGCCAGATATGA
>LOLKGF_00525 Protein PsiB
ATGCGTAACATTATTACCCCCGCTGTACTGAATACCATGATCCCCCAGGAGTTTGAAGACTGGCGCGACGGCGGCGAAGACCTGCGCCGCGAACTGACCCACGCCGTGATGCGTGACCTGA
CATGTCCCGCAGGCTGGGACATGAACGGCGAGTACCGCAGCGAGTTTGGTGGTTTCTTCCCGGTGCAGATCCGTTTCACGCCAGCCCACGGCAATTTCAGCCTGGCGGTATGCAGCCCCG
GCGATATCAGCCCGTCCTGGATGGTGGTCTTTATTCCGGTCAGCGGTCGCCCGTTCTCTGTTATCCGCACGCTGCCAGCCTGGTCGCCGGAGGTTATCACGCATACACTCAGCCTGGTGGCTC
ACCTCGATGCCGATGGTTACAGTCAGGCAAGCATCATCAGCGTTCTGGCGATGGAGGGCGCAGTATGA
>LOLKGF_00530 ParB domain-containing protein
ATGTCAGCCACCGAGTCTAAAGCAAAAAAGGCAAAAAAAGCCAGCCCCAAAGCGGCAGCGCATAAAGCGAATACCCAGTCAGACGTTCTGGTTACCGCACTGGACAGCGCCCCCATCCGT
TACACCGCCTGGTCCCGCCTGTTTATCGGGGAACTGAACTGCCGCATTGTGCCGCACACCCCGGAAGAAATCAGGGAATATGCCGACAGTATCCACGCAGTCGGGCAGATCCATAACATCGT
GGTGGTGGAATGTGACGATGGTCGGCTGGAAGTGGTTTGCGGCGGGGGACGGACAAAAGCGATCGGTCTGCTGGTTGAAGAAGGGAAGGTAAACCCGGATCACGAATGGATTTCTTATA
AAGCGGTACCGCGTGAACTGGCACGGGCTGTCTCCCTTACCGAAAACGGCAGACACCGCGCCATGCACCCTGCCGAGCAGATTGCCGGATTCCGCGCCCTGGCTGAAGAAGGGAAAACG
CCCGCGCAAATCGGGGACCTGCTGGGCTACGGCGTGAAACACGTCCAGCGCATGCTCAGACTCGCAGGACTGGCTCCGGTTATTCTTCAGGCACTGGCAAAGGACAGGATCACCACCGA
GCACTGTCAGGCGCTGGCGCTGGAAGATAATCCTGACCGTCAGGTGCAGGTGTACGAGGCGGCGTGTCGGGAGGGCTGGAACAATAAACCGGAAGTGCGCGTGATCCGTAACCTGATCA
CCGAAAGTCAGGTGCCCACCCTCAACAACAGCAAATATGCGTTTGTCGGTGAAAAAGCCTTCTCCGGCGATGAAATCCGTGCAGACCTGTTCAGCGACGAGCAGGGCGGTTTTGTGGACA
AACTGGCGCTGGATACGGCCCTGCTGGAAAAACTTCAGTGGGTGTCGGAGTGCCTGAAAGAGGCCGAGGGCTGGGCATGGGCCACCGGACGTCTGGACGCGATCAGTTCTCACGGCGA
GGACAGCGAGGTGTACCGTATTCAGGATGAGCCTGACGCGGTTTACACAGAGCAGGAGCAGCAGCGCATGGATGACTTGCAGGAGCAGTATGACGAGAACCAGACCGCCAGCGATGAA
ACCGACGCGATGGAGTCAGAGATGGAGGCCATCGAATGCGCGGCACAACTCCGGGCATGGACGCCGGAAATGCGCGCACAGTCCGGCGTTGTGGTGTCATGGCGTCAGGGTGACGTTTA
TGTCCAGCGCGGAGTGATCCTGCGCGAACCGTCTGAAACGGAAGACGAACCTGCCCAGGTTAAAACATATGAGCGCCAGCCAGAGCCTGTGGACGATATCAGTGTTCCCCTGCTGACCCG
AATGTGTGCGGAGCGGACGCTGGCAGTCCAGGCGGCACTGATGCAGCAGCCGGAAAAAATCCGTCGCCCTGCTGGCATGGACGCTGTGCCTGAATGTGTTCGGCAGCGGCGCGTATAACC
GACCCGCGCAAATCTCCCTGGACTGTAAGCATTACTCCCTGACGAATACCGCCCCGTCCGGTAAAGAGGGCATGGCATTTCTGACCCTGATGCAGGAGGGGAAACGCCTTGAAACGCTGCT
GCCGGAAGGGTGGAAACAGGATTTCACCACGTTCTTTACCTTCAGCACTGCCGATCTTCTGGCCCTGCTCAGTTTCTGCACGGCATGCAGTCTCGATGGCATGCAGACGCGTGGCACCGGA
GGCACCACCCGCAGCCCGCTCGATAAGCTGGAAACCGCTCTCGCTTTCCATCTGCGCGACTGGTGGCAACCGACGAAGGCCGATTTCTTCACTGGTCTCAGAAAGCCACAAATCATCGCCG
CACTGAACGAGGCCGGACTGACAGGCGCGGCGCGGGATGCGGAGAAGATGAAGAAAGGCGATGCGGCTGAACTGGCAGAGGACAAAATGCGGGACAACCGCTGGGTGCCTGTCTGG
ATGCGCGCCCCTGACGCAGAAAAATCCCCGTCTGATGCAGAAAACGACGTATCTGATACCGAAAACGGCAGCGCTGATACACCCGATGCGGCTTCTGATGCAGACAGCCACCATACCCTGC
CTGATGCAGCCTGA
>LOLKGF_00535 DUF905 domain-containing protein
ATGACAGACAACACCATGCCCCCACTGAAATCCCTGCCTGACGGGACTTTCACCCGCGAACAGGCCGAAGCGGTGGCGGCTCAGTATCAGAATGTGGCCATCGAGGACGATCAGGGGAC
GCACTTTCGCCTGGTCATTCGCAAGGATGGCGAAATGGTCTGGCGGGTCTGGAATTTTGAACCGGGCGGCGAGTACTGGATGAACCGAAATATCCAGAGCTACGGCATCCGTAAAACGCA
GTAA
>LOLKGF_00540 Single-stranded DNA-binding protein
ATGTCAGCACGTGGTGTTAACAAGGTCATTCTGGTCGGGAATCTGGGGCAGGCCCCGGAAGTTCGTTACATGCCTAACGGTGGCGCAGTGGCAAATCTGCAACTCGCAACGTCAGATAACT
GGCGTGATAAACAGACGGGCGAGATGAAAGAGCAGACGGAATGGCATCGGGTGGTGCTGTACGGAAAACTCGCGGAAGTGGCTGGCGAGTACCTGCGCAAGGGGTCTCAGGTGTATAT
CGAGGGACAACTGCGAACCCGTAGCTGGGACGATAACAACGGCGTCACCCGCTATATCACAGAAGTTATTGTTAACACCACGGGCACCATGCAGATGCTGGGTGCCCGCCGTGAAAGTTAT
CCTCAGCAGGGCAGTCAGCAGTTCAGCGGTCAGCCGCAACCTCAGACCCCACCTCCGGCGGCAAAGGGAGAGGGCAAAGCGGCAAAAGGGGCAGGGAAAGCAGCCAAAGGAAAAAA
CGCCGCAGCACCGCAGCAGCCGCCAGCGCAGCCCGAACCGGCGTATGACTTCGACGATGACATTCCCTTTTGA
>LOLKGF_00545 hypothetical protein
ATGTACCGGCCACAAAACTTTTTGCCGCAGGCGTCAAAAACTTTTGCGTCCGCCCCCGGACAGCCGCCCCGTGACGTGGTGACTGGCGCCCCACCGGAAAGGTCCACTTCGGGGAGTAAC
TCCTGA
>LOLKGF_00550 Secreted protein
ATGTCCTGGCCGGATTATGCAGTGATACTGTCATCACGTCTCTTGTTTCTTTCTTTGGTCGCCCCTTCGGCTCCCGGCTGCCTGACGGCATCCGGTCGTCAAGCCCGACTCCGCCGGGCAGCT
GCTGGCGCACCTGCCAGGCTCCGCCGAACCCGCTGGCGCGGTTTCGGGCTTCGCTACGGTCTGATGCGTGAACACCCGGCTTGCAGCGACCGTTCCGGTCGCGCGCCCCTGTCGCGCCGG
AAACGGCGCTCCCCGCACCCCTTACGGGGTGTAAGGTCACAGCGCCGGATGGCGCTGCTGTTTATCCTCCCGCTGCCACTTCTGCGGCCTGTTCCCCGCAGCCGGACTTTCCGGGGGCTGA
AATAA
>LOLKGF_00555 hypothetical protein
ATGATGGCAGGCGGGGAAGCCGCCTGTCAGCCCGCGCGAAAAAAACTGGCAGGCTGTCGCCTGCCGGAAAAGCGGGAAGCAGCGCCTGCGGCGCTGCAACCCCGCCTTTCCGGTTACG
GGATGATGAGAGGTTGTATCATATGTCCTGGCCGGATTATGCAGTGA
>LOLKGF_00560 Plasmid protein
GTGAACATTAAACCAGAAACCCGTGAAATTCTTCGCCAGTACAAAGAGCTGATTAATGCCCGCCGCCGTGATGCCGACCAGAGTGAACTGACGATCGCGCAGGTCATGGATGAAATCTGCG
AGTACATGGCCTGTCAGTGTGCCGTTTACATCGGCGGTCACTTCATCTTACAGGGAGGAAAAAGCAGGTAA
>LOLKGF_00565 DUF1380 domain-containing protein
ATGTACGCACCCATTAAAGAAATCATGACCGATCTCCTGAAATATGACATGGCGCCTGAAGAGGCTTTGCTGGTTCTGTGTATTACGCGTGAGGATATCCGTATGCTGACCACTGAATGGAATA
TGTCCGATGAGGATATTACATGTGTCATGCAGCGACTGAATGCAATGTATGAGCAGGGGGCTGATGTGAGCATGATCCGCTGTATCGCTGAAGAACTGATGGATGAGCGCCGCGCAATAGGT
GATGTCAGCGTTCCGGCCAGTGCGCTGCAAAAAATAATGTTACTGGCAGGTTCTGAAATGCAGCGACTGTATGCCGTGGCAGAGGAAGGGGGCGGGGATGCAGACGCCTTCCTGGCGGA
AGAAATAGACGCCATGCGCCAGTTACAGACTGCGATCGACGGATAA
>LOLKGF_00570 Antirestriction protein

ATGCAATACGCAGCAACTGCCCCCCTGAACGTTGAAGAATCTGATCGTCTGTCATTTCTGCCGTATCTGTTCGGCGATGATTTCATGATTGCAGAAATGCAGGTGTACGCGCTGGCCAGAAAA
ATGATTACCGGATACGAAGGCGGCTTCTGGCACTTCATCCGTCTGCCGGACGGTGGCGGCTATATGATGCCGGACTGTGGGCCAGTGCATCTGACTAACAGTGAAAACTGGTTTGACTCTAC
TGTCAGCGCGGATGCAGCAGGGATTATCCTCACATCCCTCGCTATTAACCGCCGTTTATGGGCGCATCACGCGTGTGGTCATGCTGCGCTGACGCACCTTTTCCGGACAAGGGATGCGCAGT
TATGGAGCCATATTGAGTTCCATCCTGAATGTAACGCCATTTACGCCGCACTTGACTGA
>LOLKGF_00575 Inner membrane protein
ATGAGAAATAACACGCTCAGTGCACTGATAGTGCACCACGGAGACAGTCTGTTACACCGGTCCGGCTGGCCCGAAACCGTCGGAGTGACACAGGTAGCCCCCGGTGTGGTTCCCGGCTGG
CTGGCCGTCTGCGGTGTGCTCAGTGCAGATGAAATCCTGACGCTGGTGACGCATCTTTGCCGGTCCCTTAATTATGGACGGGCGCAACTACTGACGGCCAGTGCGCAACGGCTGGCGGGG
ACGCCAGTCCGGTTACATCTTTACCCGGTACAGGTATATCCGCACCCTGCCACGGTACGTGAATGTACCGAAATCCGCCTTCCGTATGCTCAGGAGTGGCTGACTATGGACGAATGTGCAGAC
CTGCTGGCGCTCCTGAAAGATTCCACTGACCGGGTATGCGAAATCGTCCGCCAGGATGCGCGACGTATTGCGGCGGCGCTGGCGCCGTCTGGCGAGCCCCGTTTGATGGAAAAACAAATC
GGGAGCTGGCGGCTTCTGGCTGATGAATACGACCATGAGAACTGGCTTGATGCAGACGACGGGGACGAACTGGATAAGGTGCTGGATGCGGTACTGGTCCGTAACGCACGTTTCTGCCCG
GTTCTGCTGACGCTGGTTAATGAGCAGGAGGAAGGTATTGAAAGTTGCGGCGTGATAACGGACTGTCTGCGGTTTCCCGGCGCCCCCTCCCGACGCTGGCTTGACCGCCGTGTCCTCCGG
GAAGTGGTGAACGAGGCCAGACTTATCAGCCATTCTGACAGTGAATGTTGA
>LOLKGF_00580 DUF1380 domain-containing protein
ATGATGTATGGCACTCGTAAGGAACTTAACAAAAAACTGAAACGCGTTTTTGGCAACGACGAGCGTTTTGCCCTGCTGGTCTGGACGAAGCAGGACGTTATGTCACTGGCTCAGGGGATG
ACCGAAGTGGAGGCCGATGCGATTTTACGTGAGATTGGCAAAACCGGTTTCGGCGACCATGCCGAAGCGGGCATTTCATACCGTACAGTACAGGAGCTGTACGCCGGATTACGGGAAATG
CCGAGCGTCAGTGTTCCCGCCGACCTGCTGGCAAGGATAACGGACATCGCAGGACGGGCGCTGGATACGGAGGATGCTCAGGCCTGGCCTCTTGTATGCCGACAGTACCCCAGCGTTGCC
GATGCTCAGGCCGATATTGCCCGGCTCAGGCAACAGGCGCTGGCAGCATAA
>LOLKGF_00585 Cytoplasmic protein
ATGAATTACGTCGGAAACGAGAAGTTGCGGGCGGAGGTGGCGTTGCTGACCAACAGCATGTGCGACCTGCGCACCACCCTGAAAGTACTGGAAGATCGTTATCACTGGCAGCGTCACGG
GCTTACTGAACGACTGGCCGGGCAGAGTCTGCGGCGCATCAACATCCTGCTGGATGAAGCGTTCAACGAAAGCCTGATGCTCGATGAGTGTTTTAAAGACTGA
>LOLKGF_00590 Putative methylase YubD
ATGTCCCGTTTAATCCAGGGGAACTGCGTCGATATTATGTCCACCTTTCCGGACCACGCCGTCGATTTCATCCTTACTGATCCACCCTACCTTGTCGGATTCCGTGACCGTTCCGGCCGCAGTAT
TGCCGGTGACAAAACCGACGAATGGCTGCAACCCGCCTGTAACCAGATGTACCGCGTACTCAAAAAAGACTCGCTGCTTATCAGTTTCTATGGCTGGAACCGCGTTGACCGTTTCATGGCG
GCCTGGAAAAGTGCCGGTTTCAGCGTGGTCGGACACCTGGTATTCACCAAAAACTACTCGTCAAAATCCACGTATGTCGGCTACCGCCACGAATGTGCCTATATCCTGGCGAAAGGCCGTCC
GGATCTGCCGCAGAATCCGCTGCCGGACGTGCTCGGCTGGAAATATTCCGGCAACCGCCATCATCCGACTGAAAAGCCTGTAACCAGCCTGCAACCGCTGATTGAGTCTTTCACACATCCCG
GCGCGATTGTGCTGGACCCGTTTGCCGGCTCCGCCTCCACCTGCGTTGCCGCGTTACAGGCGGGGCGCCGATATATCGGTATCGAGCTGATGGAGCAGTACCACAGGGCAGGTCAGCAAC
GCCTGGCCGCCGTTCAGCAGGCCATGCAGCGCCCGGTAGCCAACGATGACTGGTTTATCCCGGAGGCTGCCTGA
>LOLKGF_00595 DUF4253 domain-containing protein
ATGAAACGCATGACTGTTAAGGCTTTTCAGGAACGTCTTTCCCGGTATCCTGATTATGCGCTTTGTTGTGGCACGTTCTGGCTGTCCAGTGACTTTCTGGCACTGGACAGTTCGCTCACGGA
GGACGATATCGATGCGGCGATTGAGCTGGCGCAGTACAGCCATGATGCGGATGAGGAGTTCCTCGATGCGTGGACAGTGACCGGCTCAACGGATGCGCCGGTACTGCGCGACGAGTGGG
GGAATGACTGGCGGGATATCACCCGCTGA
>LOLKGF_00600 DUF1281 domain-containing protein
ATGCCAAACTGGTGTTGTAACCGGATGCGTTTTTCTGCTGTACCTGAGCAAACAGCAGCGATTAAGGCACTGGCTGAGGGTGCTGTGACACCGTTTTATCGTCGGGCGACTGAAGAGGGG
ATTCAGTTATTCGTGGCCGGATGTGTCGGGCTTCTGCAAGTGACAGAGGAAATTCAGTTCATACCTTATCCTGCACTGACTGCAGCAGGAATTGGTGTGTTATCGCCGGAAAACCTGGCGTT
TACTCGCTGGCTGACCCAGTTGCAGGATGGGGTGCTTCTGGATGAGAAAAACAGTCAGGTACTTCATGAAATCTGGCTGCAAAGCGGTATCGGCGGTCGTCGGTGGGAAACGTTGTCCGA
CAGTACCAGAAATGAGATTTCGCGTCTTTATGCATACAAATGCCATGACTGGTGCGGAATATGGGACAGGAAAGACGTGGCTGTATGGTGGACGCAACTGTGTGATAACCCGTTACCCGCGC
GAACGAATCCGTTTGACCTGCTTCTGGTTCTGCCGTCCCGACTTGATGTGGAAATTAACGGCTTCAACGGAAAACTGCTGGATGGCATTCCTTCAGCGTATAACTGGTACCTGTCGCAATACG
GAACAAAGTGGCCGGTGGGGTATGAGCTGAATATTTGTTCTCAGGGCAGTGACTTTATCGTGATTGATTTTGATACCCCCTGGAGCCCGGTCAGCGAAGAAGTTGTGGCTGAACTCAGCAA
GCGTTATGGCTGTGAGGTAGAGCACTGGTTTGTGGAGCAGGGATGTAATTACTGCGGTTATGCCAGGTACGTCAAGGGCGAAACGGAGGTGTACATCACGGACGAGCTGGAGTGGGGTA
ATGCTGACCCGGATGATGAGGACAGCTTCCAGGATATAACCGGCCCTGAGTGGATTATCAACAATGTGGCGCACTTTGGCGGCTGA
>LOLKGF_00605 DNA-binding protein
ATGACCGATCTCAGGGAGTATGGTAAGCAAATCCGACAATTCCTCAAGCTGGCTCGGGAGCTGCAGACACTGAATATTGTTGAGGATTTCGAAAATAAGACGCTGACTGAAATCCGGGAAG
TGTTAACCCGGCGTTCTTCTCCGGGGACAGGCTATAAAGATGCGTATCCACGCCACGGAGCCCGGTGGGAGGAAGAGGAAAAGCAGCATCTCATCGCGCTGGCAGAGGCTGGCATGCTC
GACGTGGATCAGTTTGCTGAGGATTACCAGCGCAGGCCTGCGTCAGTATTTAAATACATGAAAAAAATTGGGTTACTGAATAAAAACTTTAACGATTTCTGA
>LOLKGF_00610 ABC transporter
GTGTCTGCCGGAAATTTCTTTCTCTACCTGAATCATGATGATCATACGGATATGCGTGCCCTGGCCACTATTGAGTCAGTGTCCCGCCCATTACGCGGAGAGTTTATTCGCGTCGCGTCCGTGG
CTGGATCGGTTCTTTACCGGATTGATGTCCGTCTGCCGCCAATGTTGGGCGCTATGTTTGCAGGCACAATTACGCCATCGCAGTTTGCCGGTATACTGGCATTACTGCGGGGAACCGGAGAG
ACTGATGTTTCAGGAGAGGGAAAGGTGTATCCATGGATTCCGGGCAGATATACCGGCACGTCTCAGCGCCGTCGGTTGATACTGCAACTGGAGGATGATGTGTGCGCCAGCCTGGTACTCC
CTCTGCTGGATGATGCTTCTGACCGGCAACGTATGACACTTCTGCGCAGTCTGATGATCGCAGGTTGTGTACTTCATGAGCTCGATCAACGATTTCCCCGGTTGCTCGCCAGCATGCCGGAGC
CTCCTGCCACCCTGGATGCCCTGCAGCACCTGGCTACGGGATTATTTAAGGAGAGGGGGCTGAGTATGCCCCTGCCCGGAGAGGTAAAAAGTACCGGGCCGGAGTCCTCTCCTGAAATCA
GCGTCGCGCGGGCGAATATGAAAAAATTATTTTAA
>LOLKGF_00615 StbA domain-containing protein
ATGATTAACATTTTCTGTGACGATGGTTCCACTAACGTTAAGCTTGCCTGGTTTGAAAACGATATTCTGAAGACGTCCGTTTCACCTAACTCGTTTCGCCACGGCTGGAAAGTGGATTTCGGC
GGTCCGGTATTCAATTACATGGTCTCCGGCCTGAAATACACCTGGGATGCGGTCAGCCGGGAAGCGGTATCAACCACAAACGTGGAATACCAGTACGGCGATCTCAATTTACTGGCGGTACA
TCACGCGTTACTGAACTGTGGCCTGAGCCCGCAGGATGTATCGCTGACAGTAACGCTGCCACTGAGTGAGTATTACGACCAGGACTGCCAGAAAAATGAGGAAAATATCAGCCGCAAAAAA
GCTAACCTGATGCGCGATATTTCTCTCAACAAGGGAGTAACGTTTACAATCAAAGACGTCAGTGTTATGCCTGAGTCATTGCCGGCGGCGTTCTCCCGCCTGGCTGAAATTAAGCCCGGCCC
AGGAGAGTCAACGTTGATCATTGACCTCGGAGGAACAACGCTGGATGCGGGGGTGATCGTGGGACAATTTGAAGATATCAGCGCCATCCATGGGAACCCGACCATTGGGGTATCTGTTGT
CACTCAGGCTGCGCAGGGAGCCCTGCGTGCTGCGGAAAGTGAAACCAGTCCGCTGATTGCTGACAGGGTCATTCGGGAACGAAAAGATCGTGCATTTCTTCACAGCATCATTAATGATTCG
ACCAGAATTGGATATGTCACTGACAAAATAGAAGAGGCTATCGGAGCGCTGGGAGCCAGAGTGGTGAGTGAACTTACACGCTTCCGTAACGTGAACCGTGTTCTGCTTACAGGTGGTGGT
GCTCCGCTCATCGAAGAAAGCGTTCGTCGCGCCTGGCCGCTTGCCGCCGACCGGATCGAGGTTATCAAAGATCCTCAGCTGGCACTGGCGCGGGAGATAGCGCTCTTCAAACACGAGGAC
TGA

PTU-E78

>DLDPCG_00005 DUF3560 domain-containing protein
ATGACACAGAATACCGCACAGACACTGACTGCCAGCATCACCCCGCCGCCCGCGTATGAAGACAGCCCCGCGCAGTCGTACCGGGCGACATACTCCCCGGATGACAACAAAATGCGCCTGT
ATGCCGCGCTGCGTCTGGATGATGAAACGTATCAGAAGATCCACGCCGTGGGTTTTCGCTGGGCGCCGAAACAGGAACTGTTTGTCGCGCCAGCCTGGACGCCAGCCCGTGAGGACGTGC
TTCTGTCTCTTGCTGGAGATATTGAAGACGATGACAACACCCTGTTTGACCGTCAGGAGCAACGCGCCGACCGTTTCAGCGGCTACAGCGAAAAACGCGCCGGGGAGTCAGAGCAGACG
CTCGCGCAGGTGGACGCGCTGGCGTCGGTTATCCCGTTCGGACAGCCGATCCTGGTTGGTCATCACAGCGAACGCCGCGCGCGACGGGATGCGCAGCGTATTGAAAACGGCATGAAACG

CGCCGTGATGCTCTTTGAACGTGCGGAATACTGGGAAGAACGGGCGCGGTCGGCACTGCTTCACGCGAAGTATAAAGAACGTCCGGACGTTCGCTGGCGTCGTATCAAAAAAATCGAAGC
TGATTTGCGCAAGGCTGAAAAGACCATTGCGCAGTCGCAGAAATATCTGACGATGTGGCGGGCTGAATCGCTGGATCTGAATATGGCAAAACTCATCAGCAGTCATGACCATATCAGCGCCT
GTTTCCCGCTGGATACGTATCCGCGCCCGGCAGAAAAAAGCCAGTATGAAGGGAGTCGATCGTTATGGTCGGCCCTGGATGATGACATCATCACCACGGAGCAGGCCCGCGAAATTGCGAT
CCGCTGTCATGAACGGCAGATTCAGCATCAGCAACGCTGGGTTAACCACTATCAGAACCGCCTGATCTATGAGCGTGCCATGCTGGACGAAAGCGGCAGCGTGGTTACCCGGACGCAGGAT
TTTGAGCCGGGCGGGCAGATTTGCAGCCGAGGCGAGTGGCTGACCATCATTCGCGTGAACAAAAGCAACGGGGCGGTGAGTTCTGTCACAACGCCAAATTACAGTTTCCTCGGGTACAG
CGGCACGATGAAAGTGACGCCCGATCGCATTACGGACTACAAAGCGCCATCGACAGAAGAGATCGTTGTCGCCAGACAGGCCGCGAAGCGCCCGCCTGTTGTCAACTACCGGGAGAAGG
TTTCCGGGAAATGA
>DLDPCG_00010 hypothetical protein
ATGACAAAGGCACAGTGGGCCACCCTGCCCCGGGACTGTAAGGCCGTGCGCAGTGTGGCAGAAGCAGAAGACCACGGGGCATACCGCTACCGCCGCACGATGGACAATAATTTCCGCCT
GGTGAATGTGTATATCACCGACATGAAAATTACGGAAATCCCACAGAAATAA
>DLDPCG_00015 MTS domain-containing protein
ATGCACAGTCAGTTAAAAGAACGTATCCGGCTGATGCGCGCAAGGCTGGATAACGCCGCGCCGGTTGCTGAAATCCGGGCTGAATCTCAGCTTTTTGTGACTCCTGCCCCGGTCTGTGATC
GCCTGGTGACGCTGGCGGAGATCAGCAACCGTGATCACATTCTGGAACCCTCTGCCGGCACCGGAGCCATACTGCGGGCGATTCGGGATACCGCGCCGGGGGCCATGTGTGATGCGGTGG
AAATCAACAGCGGGCTGGTCCGGTATCTGCGGGAAAATTTTAACGGTGTCAGGGTTCAGTGTGGCGACTTCATGGAATGGCAGCCGGTGCAGTATTACAGCCGGGTTATCATGAATCCGCC
GTTCAGCCACGGGCAGGATATCCGGCATATCCTGCGGGCCTTTTCCCTGTTGCGTCCGGGGGGTGTGCTGGTTGCCGTCTGTCTGAACGGGCCGCGCCAGCAGGAGAAGCTGTTACCGTT
TTCTGACGTCCGCGAGGAGCTGCCACGCGGTACGTTTGCTTATACCAGTGTTCCGACGATGATTATTCGTCTGCGGGCCTGA
>DLDPCG_00020 Plasmid-derived single-stranded DNA-binding protein
ATGGCAGTTCGTGGCATTAACAAGGTCATTCTTGTCGGTCGTCTGGGGAAAGACCCGGAAGTCCGTTACATCCCCAACGGGGGTGCGGTGGCAAACCTGCAGGTGGCCACGTCTGAAAGC
TGGCGTGACAAACAGACGGGGGAGATGCGGGAGCAGACGGAATGGCATCGTGTGGTGCTGTTCGGCAAGCTCGCGGAAGTGGCAGGTGAATATCTGCGCAAGGGCGCGCAGGTCTACA
TCGAAGGTCAGCTTCGCACCCGTAGCTGGGAGGATAACGGTATCACCCGTTACGTCACCGAAATTCTTGTTAAGACCACGGGCACCATGCAGATGCTGGGACGTGCCGCAGGTGCTCAGAC
TCAGCCGGAAGAGGCGCAACAGTTCAGCGGTCAGCCTCAGCCGGAACCACAGGCGGAGGCCGGTACGAAAAAAGGAGGCGCAAAAACGAAAGGCCGTGGACGTAAGGCCACGCAGC
CGGAGTCGCAGCCGCAACCGCCGGAGAGTGACGATTACGGGTTTTCAGATGATATCCCGTTCTGA
>DLDPCG_00025 UPF0401 protein YubL
ATGAGCGAATATTTCAGAATACTTCAGGGACTGCCTGACGGCTCCTTTACCCGCGAACAGGCCGAAGCCGTTGCCGCACAGTACCGGAACGTCTTTATCGGGGATGACCACGGCGAACAGT
TTCGCCTGGTTGTCCGTAATAACGGTGCAATGGTCTGGCGCACCTGGAATTTTGAGGACGGTGCCGGGTACTGGATGAATCATGTCATCCGTGATTTCGGGATTCTTGAGTAA
>DLDPCG_00030 ParB domain-containing protein
ATGTCAGTTACAGAGTCTAAGGCAAAAACGGAGCGTAAATCCAGCCGTAAACCTGCAAAAACGCAGGAAACAGTCCTGTCGGCCCTGCTGGCGCAGACGGAGGAAGTGAGCGTGCCGCT
GGTCTCGCTGATTAAGTCACCGCTGAATGTGCGCACGGTGCCGTATTCTGCGGAGTCCGTCAGCGAACTGGCAGAGTCCATTAAAGGTGTCGGCCTGCTGCAGAATCTGGTTGTTCATGCCC
AGCCAGGTGACCGTTACGGTGTCGCCGCAGGTGGGCGCAGGCTGGCGGCACTCAATATGCTGGCAGAGCGTGGAATCATTCCGGCGGACTGGCCTGTCCGTGTGAAGGTCATCCCGCAG
GAACTGGCGACGGCTGCATCGATGACCGAGAACGGTCATCGTCGGGATATGCACCCTGCCGAACAGATTGCCGGATTTCGCGCAATGGCGCAGGAAGGCAAAACACCTGCACAAATCGGT
GATTTGCTGGGCTATTCACCCCGCCACGTCCAGCGAATGCTGAAACTGGCAGACCTTGCGCCTGTCATCCTCGATGCGCTGGCAGAAGACCGCATCACCACAGAACACTGTCAGGCGCTGG
CGCTGGAGAACGACACCGAGCGTCAGGTGCAGGTGTTTGAAGCTGCCTGCCAGTCGGGATGGGGAGGTAAACCGGAAGTTCAGACCATTCGTCGTCTGGTAACCGAAAGTGAAGTGGC
AGTGGCGGGGAACAGTAAATTTCGCTTCGTGGGGGCTGATGCCTTCTCGCCAGACGAACTGCGTACTGACTTGTTCAGCGACGACGAGGGTGGATATGTGGACTGCGTGGCACTCGATGC
TGCCTTGCTGGAAAAACTCCAGGCCGTTGCTGAATACCTTCGGGAAGCCGAAGGCTGGGAATGGTGTGCCGGACGCATGGAGCCTGTCGGTTTCTGCCGTGAGGATGCCGGAACATACCG
CACTCTGCCGGAGCCGGAAGCGGTGCTGACGGAGGTGGAAGAAGAACGCATGAACGAACTGATGACGCGTTACGACGCGCTGGAAACCGCTATCGGATTCCACATGCGCGACTGGTGGC
AGCCGACAAAAGCAAACTTCTTCGGACACCTGAAAAAGCCGCAGATTATTGCAGCCCTGAATGATGCCGGACTGTCCGGTGCCGCACGGGACGCAGAGAAGATGAAGAAAGGCGATGCG
GCTGAACATGCAGAGCACCATATGAAAGACAACCGCTGGGTGCCTGGCTGGATGTGTGCACCACGCCCACAGACGGATGCCACTGAACGCACCGATAACCTGGCTGATGCCGCCTGA
>DLDPCG_00035 Protein PsiB
ATGAAAACTGAACTGACCCTGAATGTATTACAGACCATGAACGCACAGGAATATGAAGATATCCGGGCTGCCGGAAGTGATGAACGTCGTGAGCTGACACACGCCGTGATGCGGGAGCTG
GATGCGCCGGATAACTGGACGATGAACGGCGAGTACGGCAGCGAGTTTGGCGGATTTTTCCCCGTCCAGGTCCGTTTCACGCCTGCCCACGAACGTTTTCACCTGGCGTTATGTTCACCGG
GCGATGTCTCTCAGGTCTGGGTGCTGGTTCTGGTGAATGCCGGTGGTGAGCCGTTTGCGGTGGTTCAGGTACAGCGGCGGTTTGTACCGGAAGCCGTCAGCCATTCACTGGCACTGGCGG
CGTCACTGGATGAACAGGGATACAGCGTCAGTGACATCATCCATATCCTGATGGCGGAAGGTGGTCAGGTATGA
>DLDPCG_00040 Plasmid SOS inhibition protein A
ATGAGCGCACGTTCACGGGCACTGGTTCCCCTCAGCACAGAGCAACAGGCCGCATGGCGGGCGGTGGCGGAGACGGAAAAGCGCCGCCATCAGGGGAACACGCTGGCGGAATATCCGT
ATGCGGGGACGTTTTTCCGCTGTCTGAACGGAAGTCGCAGGATATCGCTGTCTGACCTGCGATTTTTCATGCCGTCACTGACCGCAGAAGAACTGCGCGGCAACCGTCTTCAGTGGCTGTAT
GCCGTCGATGTGCTGATTGAAACACAGGGCGAGGTCTGCCTCCTTCCCCTTCCCGGTGATGCAGCAGAGCGGTTGTTCCCGTCGGTGCGGTTCCGTGTCAGGGAACGCAGTCGCCATAAAT
CCGCGCTGGTCATGCAGAAATACAGCCGCCAGCAGGCACGGGAAGCAGAGCAGAAAGCCCGTGCGTACCAGGCGTTGGTTGCTCAGGCTGAAATCGAACTGGCGTTCCATTCACCGGAA
ACAGTCGGAAGCTGGCATGCCCGCTGGAGTGACCGGGTGGCAGAGCATGACCTGGAAACCCTGTTCTGGCAGTGGGGTGAGCGTTTTCCGTCACTGGCCGGGATGGAACGCTGGCAGT
GGCAGGATATGCCATTCTGGCAGGTTATCGCGGAAGCCAGTCTGGCAGCAAAGGAGGCCGGTCATGCTGTCCGTGAGATGGAGCGGTGGATGGTGCCGAACAAACTCCGGGAGGCGGC
GTGA
>DLDPCG_00045 Theronine dehydrogenase
GTGAAGTGGCGTTACTCACTGCGCTGGAAACTGCCTTATCCCTGTCCTGGCGAGCATGAACTGGTTTCAGAAGTGGTGGGGGCGGGGCAGCCTGCACCGGCATCCGTTATGTCTCGCTGG
GTTGCCGGTGCAGGGTATGCGGTCTGTCTGGATTTTATTTCTGACCGCCAGGTGCGCCGCTGGAGCGAAGAACGTAAGGCTACGGTACGGCGGCGGAATCTGGAGAAACGTATCAACAGG
CATGCACCACTGTTTGCGGATGAACTGATTGCCCGTGAACTGGCTGAACGACCGGATTATTTTCAGGGAAAGTGA
>DLDPCG_00050 sok antitoxin (CssrC)
CGGCGCTTGAGGCTTTCTGCCTCATGACGTGAAGGTGGTTTGTTACCGTGTTGTGTGGCAGAAGGACAAAAGCCCCGTAGTTAATTTTTCATTAACCCACGAGGCCCCTGCATGCTTAGACA
ACATCAGGATAGCCTCTTACCGCGCTTTGCGCAAGGAGAAGAAGGCCATG
>DLDPCG_00055 hypothetical protein
ATGCTTAGACAACATCAGGATAGCCTCTTACCGCGCTTTGCGCAAGGAGAAGAAGGCCATGAAACTACCACGAAGTTCCCTTATCTGGTGCGTATTGATCGTGTGTATCACACTCTTGATATTC
ACAGGTCTGACCCGGAATCGCTTATGTGA
>DLDPCG_00060 hypothetical protein
GTGCGCCAGCAGCTGCCTGTCGGAGTCGGGCCTGACGACCGGATGCCGTCAGGCAGCCGGGAGCCGAAGGAGCGACCAGCAGATGAAGCAACGCAGCAGGAGCCGTCACCAGAATCA
GGGAAGGAGGAAAAAGAGGAACATCATTATCAGGAACCGGGAAATGCCAGGAATGAGCAGACAGAAGCACTAGACAGAGCAGCCGCGCAATGA
>DLDPCG_00065 SAM-dependent methyltransferase
ATGAGTACCCGAAATATCCACGTTAACACTGTGTCGTACACGATTCTTGTTGCCGAGAGGGAAAAAAACACCGGGGAAGAGTGGGACGTCCTGGAATTCAGCAGCCTGACTGAGCTGAAA
AAATATCGCAAAAGCCACCCGGAAAAGATGGCCTTCAGCTATAGCTACGCGCTCAGCCGGGGTGTGGATACGCAGTTCCGCCATATCAACATTACGGAAGCCGATCACTTCAAACAGTTCCT
GCGTCAGATTAAGCGTGCCGGCCTCGATATCCGGGCGATCTGCTGA
>DLDPCG_00070 DUF945 domain-containing protein
ATGCGATTAGCTTCCCGTTTTGGTCGGTATAATTCCATCCGCCGTGAACGTCCTTTAACGGATAATGAATTAATGCAGTTCGTGCCTTCGGTATTTTCCGGTGATAAACATGAGTCCCGGAGTG
AACGTTATACGTATATTCCAACAATCAATATCATCAATAAGTTACGTGATGAAGGTTTTCAGCCGTTCTTTGCCTGCCAGAGTCGTGTGCGGGATTTGGGACGCAGGGAGTACAGTAAACATAT

GTTACGTCTTCGCCGTGAAGGAAGTATTAACGGGCAGGAAGTTCCGGAAATTATTTTGCTTAACTCTCATGATGGTTCGTCCAGCTATCAGATGATCCCCGGTATTTTTCGTTTTGTCTGTACC
AATGGCCTGGTATGTGGAAATAATTTTGGCGAAATCCGTGTTCCCCATAAAGGAGATATTGTCGGGCAGGTCATTGAGGGGGCGTATGAGGTCCTGGGTGTGTTTGATAAAGTCACAGAAA
ATATGGAGGCAATGAAGTCCATCTCTCTTAACAGGGAAGAGCAGTACTTATTTGGGAAGGCAGCATTAACGGTACGGTACAACGATGACAATAAAATCCCTGTGACACCGGATCAAATAATT
ACCCCGCGTCGCTATGAAGATAAAAAGGATGATCTGTGGACCACCTGGCAGCGCGTCCAGGAGAATATGATAAAGGGGGGATTAACAGGACGAAGTGCTTCCGGTAAGAATACCAGAACA
AGAGCCATCACCGGTATCGATGGTGATATCAGACTCAATAAAGCGCTATGGATGATTGCCGAACAGTTCAGGGAGTGGAAGTCATGA
>DLDPCG_00075 SLT domain-containing protein
ATGAAAAAATGGCTTCTGACGATCTGTCTTTTATTGATAAACAGCGACTGTTTTGCAACGGACTGCTTTGAACTGGCTGGTCGGGATTACAAAATAGACCCGGATTTACTGAGAGCAATATCA
TGGAAAGAATCCCGTTACCGGGTTAATGCCATCGGTATTAATCCGGTAACGGGATATGGCTCCGGACTGATGCAGGTTGACTCCCAGCATTTCAACGAACTGGCCCGCTACGGAATAAAACC
GGAACATCTGACAACAGATCCGTGCATGAATATCTACACTGGTGCTTATTATCTGGCAATAGCGTTTAAAAAATGGGGCGTCTCCTGGGAGGCTGTTGGTGCGTACAATGCAGGATTCAGGA
AGAGCGAACGACAGAACCAGCGACGTCTCGCCTATGCATCAGATGTTTACCGGATTTATTCAGGGATAAAAAGCAGTAAAGGTATCCGGCTTCCGGCCACGAAGAAATCATTTTCGGAAAT
TAACGGTGTGCAGAACAATTAA
>DLDPCG_00080 Relaxosome protein TraM
ATGGCCAGAGTAAATCTGTATATCAGTAATGAGGTTCATGAAAAAATTAACATGATTGTTGAAAAGCGTCGTCAGGAGGGAGCAAGAGATAAAGATATAAGCCTTTCAGGAACTGCTTCAAT
GCTTCTTGAATTGGGGCTTCGCGTATATGATGCACAGATGGAGCGTAAAGAGTCTGCGTTTAACCAGACAGAGTTTAATAAACTACTTCTTGAATGTGTTGTAAAAACACAGTCAACGGTGG
CAAAGATTTTAGGTATTGAGTCTCTCAGTCCTCATGTCTCCGGAAACCCGAAGTTTGAATATGCCAGTATGGTTGACGATATCAGAGAGAAAGTGTCTGTTGAGATGGACCGGTTTTTTCCAA
AAAATGATGATGAATAA
>DLDPCG_00085 FinP
CACACATAGGAACCTCCCTTAAGGATTCTATGGACAGTCGATGCAGGGAGGTCTGAACTCCCTGCATCGGCGATTTT
>DLDPCG_00090 Protein TraJ
ATGTGTGCGCTGGATCGTAGAGAAAGGCCACTTAACAGTCAATCTGTAAATAAATACATCCTTAACGTTCAGAATATCTACAGAAATTCTCCCGTTCCGGTTTGTGTCCGTAACAAAAACCGG
AAAATCCTTTATGCCAATGGGGCTTTTATTGAACTCTTTTCCAGAGAAGATAAACCCTTATCCGGAGAGAGTTATATACGTCTGCAGGTTGAAATTTTTCTTTCATCACTTGAACTGGAATGCC
AGGCTCTTGGACATGGCTCTGCATTTTGTCGTCGTTTTAATTTTCATGGCGAAATCTATCAGATAAGGATGGAGAATGTTTCTTTTTATAATGACGAATCTGTTGTTTTATGGCAAATTAATCCG
TTTCCTGATTATCCATTTTTTGCGTTAAATCAGAGTGGAAGTAATACAAATACTTCTGATAAATTAACGATATGGAATGATCTTTCCCCCGGAACATTGCTTGTTTTCTCTTTTTATATGCTGGGTG
TTGGTCATGCAACAATTGCCAGAGAATTGGGTATTACAGACAGAGCATCTGAGGATCGAATTAAACCAGTTAAACGGAAAATAAAAGAATTTTTTGAACACTTTGATTTATTCAGAGTGTCAT
GTATCTATAAAGGGGAAATAGATTCGCTATTAAGTATAATTCGTGAATTTTATAGCGTTAAGTAA
>DLDPCG_00095 Pilin
ATGGACGCCGTTTTAAGTGTTCAGGGTGCTTCTGCGCCCGTCAAAAAGAAGTCGTTTTTTTCTAAATTCACTCGTCTGAATATGCTTCGCCTGGCTCGCGCTGTGATCCCGGCTGCTGTTCTG
ATGATGTTCTTCCCGCAACTGGCGATGGCCGCTGGCAGCAGTGGGCAGGACCTGATGGCAAGCGGTAACACCACGGTTAAGGCGACCTTCGGTAAAGATTCCAGTATCGTTAAATGGGTTA
TTCTGGCTGAAGTCCTGGTCGGTGCGGTCATGTACATGATGACCAAAAACGTCAAGTTCCTGGCCGGTTTTGCCATTATCTCTGTATTTATTGCTGTGGGTATGGCCGTCGTTGGTCTCTGA
>DLDPCG_00100 Protein TraL
ATGTCGGGAGACGAGAATAAACTTAAGAAATATCGTTTTCCGGAAACACTGACCAACCAGAGCCGCTGGTTTGGCCTGCCACTTGACGAACTCATTCCTGCAGTAATCTGTATCGGCTGGG
GAATGATGACATCAAAATATCTGTTTGGCATCGGTGCAGCGGTTCTGGTTTATTTCGGGATTAAAAAACTGAAAAAAGGGCGGGGCAGTTCCTGGTTACGTGACCTGATTTACTGGTATATG
CCAACAGCCCTGCTGCGCGGTATTTTTCATAATGTTCCCGATTCGTGTTTCCGGCAGTGGATTAAATAG
>DLDPCG_00105 Type IV conjugative transfer system protein TraE
ATGGAACACGGTGCCCGTTTAAGTACCAGTCGTGTAATGGCCATCGCCTTTATATTTATGTCAGTGCTTATTGTTCTCAGCCTCTCTGTTAACGTCATTCAGGGGGTGAATAACTACCGTCTTCA
GAATGAGCAACGCACTGCCGTGACGCCAATGGCATTTAATGCCCCCTTTGCCGTGTCACAGAACAGTGCCGACGCCTCTTATTTACAGCAGATGGCGCTGTCATTTATTGCCCTCCGTCTGAA
TGTTTCATCAGAAACCGTCGATGCCTCACATCAGGCGCTTCTGCAATATATCCGCCCGGGCGCACAGAACCAGATGAAAGTTATTCTGGCTGAAGAAGCGAAGCGTATTAAAAACGATAACG
TGAACTCAGCCTTTTTCCAGACCAGTGTTCGTGTCTGGCCTCAGTATGGCCGTGTGGAAATTCGTGGTGTGCTTAAAACCTGGATTGGTGATTCAAAACCTTTCACTGATATCAAACATTACAT
TCTTATTCTGAAGCGGGAAAACGGGGTGACCTGGCTGGATAATTTCGGGGAAACAGACGATGAGAAAAAAATAA
>DLDPCG_00110 TraK protein
ATGAGAAAAAAATAATACGGCAATAATATTCGGCAGCCTGTTTTTTTCCTGCAGCGTGATGGCCGCAAACGGTACGCTGGCCCCCACCGTGGTGCCAATGGTGAACGGTGGTCAGGCCAGCA
TTGCCATCAGCAATACCAGCCCGAATCTGTTTACTGTTCCCGGTGACCGGATTATCGCCGTGAACAGTCTGGATGGTGCCCTGACTAATAATGAGCAGACTGCCTCCGGCGGTGTGGTGGTG
GCCACCGTCAACAAAAAGCCCTTTACGTTCATTCTGGAAACAGAACGTGGTCTTAACCTTTCCATTCAGGCCGTTCCCCGTGAAGGCGCGGGGCGTACCATTCAGCTGGTCAGTGACCTGC
GCGGAACCGGAGAAGAAGCCGGAGCGTGGGAAACGTCCACGCCTTACGAATCCCTGCTTGTGACCATCAGCCAGGCCGTCCGTGGCGGAAAATTACCCGCCGGCTGGTATCAGGTTCCT
GTGACAAAAGAAACCCTGCAGGCCCCGGCGGGGCTGTCTTCAGTGGCAGATGCCGTATGGACGGGGAATCACCTGAAGATGGTCCGCTTTTCCGTGGAAAATAAAACGCTGTCTGCCCTG
AATATCCGGGAAAGTGATTTCTGGCAGCCGGGAACCCGTGCCGTGATGTTCAGCCAGCCTGCCAGCCAGTTACTGGCAGGTGCGCGCATGGATGTGTATGTCATCCGTGACGGGGAGGGC
AACTGA
>DLDPCG_00115 Protein TraB
ATGGCCAGTATCAATACCATTGTGAAACGCAAGCAGTACCTGTGGCTGGGGATTGTGGTTGTCGGTGCAGCCTCCGCGATTGGTGGTGCACTGTATCTGTCTGATGTGGACATGTCCGGTAA
CGGTGAGGCCGTGGCTGAACAGGAACCCGTGCCGGATATGACCGGTGTGGTGGATACGACCTTTGATGACAAGGTGCGTCAGCATGCCACCACAGAGATGCAGGTGACGGCAGCGCAGA
TGCAGAAGCAGTATGAGGAAATCCGTCGCGAGCTGGATGTCCTGAACAAACAGCGAGGTGATGACCAGCGTCGTATTGAAAAGCTGGGACAGGATAATGCCGCCCTGGCAGAGCAGGTG
AAAGCCCTGGGTGCTAATCCTGTCACGGCGACCGGTGAGCCAGTACCGCAGATGCCAGCCTCACCGCCCGGCCCGGAAGGCGAACCACAGCCAGGAAACACACCCGTATCCTTCCCGCCG
CAGAGCAGCGTTGCTGTTCCACCGCCGACGGCGTTTTATCCCGGGAATGGTGTCACGCCACCACCGCAGGTGACGTACCAGTCTGTGCCGGTGCCTAACCGGATACAGCGTAAGGTGTTTA
CCCGTAATGAGGGAAAACAGGGACCATCGCTGCCGTACATTCCGTCAGGAAGTTTTGCGAAAGCCATGCTGATTGAAGGGGCGGATGCCAATGCCTCAGTCACCGGTAATGAATCCACGG
TGCCGATGCAGTTGCGTATCACCGGTCTGGTGGAAATGCCGAACAGCAAAACGTATGACGCAACGGGATGTTTTGTGGGGCTGGAAGCCTGGGGGGATGTGTCCAGTGAACGTGCCATTG
TACGCACCCGCAATATCAGCTGCCTGAAGGACGGCAAAACTATTGATATGCCGATTAAGGGGCATGTCAGCTTCCGGGGTAAAAACGGTATCAAGGGCGAAGTGGTGATGCGTAACGGCAA
AATCCTCGGCTGGGCATGGGGCGCGGGATTTGTTGACGGCATCGGTCAGGGAATGGAGCGCGCCTCCCAGCCGGCTGTCGGGCTGGGGGCCACTGCCGCTTACGGGGCCGGTGACGTC
CTGAAAATGGGTATCGGTGGCGGCGCATCGAAAGCCGCACAGACGCTCAGTGACTACTACATCAAACGTGCCGAGCAGTATCACCCGGTGATACCGATTGGTGCGGGCAACGAAGTGACC
GTGGTGTTCCAGGACGGCTTCCAGCTGAAAACCGTGGAAGAGATGGCGCTGGAACGCACGCAGAGCAGAGCGGAAGAAGACAATCCGGAAAGTCCGGTTCCTGTTCCGCCATCAGCTG
AAAGTCATCTTAACGGCTTTAATACTGACCAGATGCTGAAGCAGCTGGGCAACCTGAATCCGCAGCAGTTTATGTCCGGCAGTCAGGGAGGGGGCAATGATGGCAAATAA
>DLDPCG_00120 Protein TraP
ATGATGGCAAATAATATGTCGCCCCGTCAGGCGGGCCATGCCGCCCGGTATGTTGTCGCCCGGGTTCTGCGAGGACTGTTCTGGTGTCTGAAATACGCCGTCATTCTGCCACTGGCCACAAT
GGCCCTGATGGCTCTGTTTGTTTTATGGAAGGACAACACCACGCCGGGGAAATTACTGGTTAAGGAGATTAACTTCGTAAGACAAACAGCCCCTGCCGGGCAGTTTCCTGTAAGAGAATGT
TTGCCTTCTCTGCATGATCTTCCAGGAGCAGAAGAGAAAATCTGCCGTTACCGTGCTGCTGATGCTGCAGATTATGTCAGGGAGACTGACCGGTCACTGATGCAACTGGTCACCGCATTCTG
GGCAACGCTGGCACTTATGTATATGTCGTTTGCGGCAGGCACCGGGAAATATCCGGTGCGACCCGGAAAAATGAAATGTGTCCGTGTGGCAACAGCAGATGAGCGTCTGAAGGAAGTTCT
TGCAGAAGACACTTCCCTGCCGGGAAAAATCAGGGAATGCCATGTTTATTTCCCTGATGACAGAACAATAAGAAACAACGGAGATAAAAATGAACATGCGTAA
>DLDPCG_00125 Conjugal transfer protein TrbD
ATGAACATGCGTAATATTAATGTTATTACAGCCCTTTCTGTGCCGGGTAAAACCGTGTCAGATGATTTTATGCATGCTGTTCTCAGTAACTGCACCACAAGAATCGTCCTTCCTGCACCGAAGG
AATTCAGTTCTGAATCATTGCCGCACAATTTTAATATGGCTGCTGTCGGTGTGATGAAGGAGGGAATGTCGATGCGTTTATTTTCTTCTCCGGGGCATATTGTGGTTGCCGGACAATCCGGTG
GCGGAAAATCTGTTCCGGTGAAAGAACTAATCGACAGAATTATTCAGAAATATAAGGAACCCTTCGATGAATAA

>DLDPCG_00130 Conjugal transfer protein TrbG
ATGAATAAGCTGGTTTTTGATGGCAGCGTCAAAAAAATAAATTATCCGGTGCTGTATGAGTCCGGAATAACACCTCCGTTATGTGAAGTCAGTGCACCAGAACCTGATGCGGGTGGGAAACG
CATTGTGGCATATGTTTATAAATCATCACGGAATACGGTTTTTGAAAATCCTGACATTGTAAAAAACTGTACGGTCAGGGATTTGAAGAATGATTTTGTGAACTGCGATGAGAAAGGTGAGG
GACAATGA
>DLDPCG_00135 TraV protein
ATGAAACAGATTTCTTTATTTATTCCTCTGCTGGGGACCTTATTACTTTCCGGCTGTGCCGGAACCAGTACGGAATTTGAGTGTAACGCCACCACATCCGATACCTGTATGACGATGGAGCAG
GCCAACGAGAAGGCCAAAAAGCTGGAACAGCCTGCAGATGCAAAGCCGGCTGCGGCATCACTGCCGCGCCTGGCTGAAGGGAACTTCCGGACAACCACGGTGCGGGCCGCCAGCACG
GCCAGTCCATCTGGCAGCGGGCCTGTAGTGACTGCACTTCCGGAGCAGAAACTGCTGGCACCACGTCCGTTGTTTACGGCTGCCCGGGAAGTGAAAACGATTGTTCCGGTCAGTTCAGTT
GCGCCGGTAACACCTCCCCGTCCGCTAAGAACGGGTGAGCAGACGGCTGCATTATGGATAGCGCCTTATATTGACAGCCAGGATATTTACCATCAGCCGTACGGTGTATTTTTTGTCATTAAA
CCGTCCGTGTGGGGAAAGCCACGGATTAATTAA
>DLDPCG_00140 Protein TraR
ATGAGCGATGAAGCCGATGAGGCATATTCAGTGACAGAACAACTGACCGTGACGGGAATTAACCGGATACGGCAGAAAGTAAACCGTAAAGGTATTCCGGTTTACCGGTGTGAAGCATGC
GGTAATCCCATTCCGGAAGCGCGGCGAAAAATATTTCCTGGCGTGACGTTGTGTGTCGAATGCCAGTCTTTTCTGGAAAAACAGGGCAGGCATTATGCATAA
>DLDPCG_00145 Conjugative transfer protein
ATGCATAAATCAGACGCTGAAAAAATGGAATCCATTATTGATAAACATGACCGGATATACCGGCGACAGGTTCGGGTATTTAAAAAACTGGGCTTCCTCTCAATAATTACCGCCGTACTGAGT
GTGATTACTGCGTTATTCAGTACCTCCCTGCTCCTGAAATACACGCTGTGCGTCGTGATATGCGTTTGTCTGATTTATCTGGTTTTTATGTTCTTCTGCCTGGACAGGTATATCGCGCTCAGTCGT
CAGACACTGCTGTGGCTGATGCGCCGGGTGGAAGATACCGCAGACGTTCGCCGGGAGCTGTTCCGCCGCCTGCTGTCCGGGAAAACGCTGACCGGACGGGATGAAGACGACATCCTGCG
TCTCTGGTGGGAGAGCAGTAATGAAGTGGAGGAGCTTGCAACCCGTCAGCGTGAACAGGATGCCATCCGGAAGTTTGTCGGCGGGGATAAATCAGAATGA
>DLDPCG_00150 Inner membrane protein
ATGAAGGTTTATCTGCAGCGTTACGGCGTGGCCGGTTTTCTCCTGGTGTACTGGAGCCTTTCCTTTGTGAATGAATATTTCCTGCATGAGCATCCGCTTTCTGCTGAATCCTGGAAGTGGATG
AGATTACAGGCGCTGCTGGTAACAGGCTCTGTTGTGGTGACCGTGCTGCGGTTTTTTCTCCTGAACTCCGCGGTGCGCCGGGAGGCAACGGCCATCATGACTGGAGTCGCAGCAGGTCTT
CATGTTTTTGCTCTGCTGGCTCCCTCCGTCTGGTTGTTTTATCAGTGGCAGTGGAACGGACTGTTTCTTCTGCAGTGGCAGGGAGCAGTCTTCCTGTTCTGTCTGTTCGCGCTTCCCGTTTCA
ACATTCTGTTTTTCTTAA
>DLDPCG_00155 Protein TraC
GTGAAGAACCTGCTTGATACTGTCACTCAGGCGGCCAACTCGCTGCTGACCGCGCTGAAGCTGCCGGATGAATCGGCACAGGCCAATCAGATCCTCGGCGAGATGAGCTTCCCGCAGTTC
AGCCGTCTGCTGCCGTACCGTGATTACAACCAGGAATCCGGTCTGTTCATGAATGACACCACGATGGGCTTTATGCTGGAAGCCATTCCCATCAATGGTGCGAATGAGTCCATTGTGGAGGC
GCTCGACCACATGCTGCGCACCAAACTGCCGCGCGGAGTACCGTTCTGCATCCATCTGATGTCCAGCCAGCTTGTTGGTGACAGAATTGAATACGGGCTGCGTGAGTTCTCCTGGTCAGGT
GAACAGGCTGAACGGTTTAACGCCATTACCCGTGCCTATTATATGAACGCGGCAGCGACACAGTTTCCGCTGCCGGAGGGGATGAATCTGCCCCTGACCCTGCGGCATTACCGGGTATTCTT
CTCGTACTGTTCTCCCTCGAAGAAAAAAAGCCGGGCCGACATTCTGGAAATGGAAAACCTGGTGAAAATCATCCGGGCGTCGTTACAGGGGGCCAGTATCACCACACAGGCGGTGGATGC
ACAGGCCTTTATCGATATTGTCGGGGAGATGATTAACCATAACCCGGACTCCCTGTACCCGAAAAGACGTCAGCTGGACCCGTATTCTGATCTGAATTATCAGTGTGTGGAGGACAGTTTTGA
CCTGAAGGTCCGGGCAGATTACCTGACGCTGGGCCTGCGTGAGAATGGCAGGAACAGCACGGCCCGCATCCTGAATTTCCATCTGGCCCGTAACCCGGAAATCGCCTTCCTGTGGAACATG
GCCGACAACTACAGCAACCTGCTGAACCCGGAACTGTCCATCTCCTGTCCGTTCATCCTGACGCTGACTCTGGTGGTGGAAGACCAGGTGAAAACCCACAGCGAAGCCAACCTGAAGTAC
ATGGACTTGGAGAAAAAGTCGAAGACCTCCTATGCCAAATGGTTTCCGTCCGTGGAGAAAGAGGCGAAGGAGTGGGGGGAACTGCGTCAGCGGCTGGGCTCCGGTCAGTCTTCTGTCGT
GTCCTACTTCCTCAACATCACCGCATTCTGCAGGGACAATAATGAAACGGCGCTGGAAGTGGAGCAGGACATCCTGAACAGCTTCCGTAAAAACGGTTTTGAGCTGATTTCACCGCGCTTTA
ACCACATGCGCAATTTCCTGACCTGCCTGCCCTTTATGGCCGGGAAAGGGCTGTTTAAACAGCTGAAAGAGGCCGGTGTGGTTCAGCGCGCAGAGAGCTTTAATGTGGCCAACCTGATGC
CCCTGGTGGCGGATAATCCCCTGACACCGGCAGGTCTGCTGGCACCCACCTACCGTAACCAGCTGGCGTTTATCGATATTTTCTTCCGGGGGATGAATAACACCAACTACAACATGGCGGTC
TGTGGCACCTCCGGGGCCGGTAAAACCGGGCTGATACAGCCACTTATCCGTAGCGTGCTGGACTCCGGAGGCTTTGCCGTGGTGTTCGACATGGGGGATGGCTACAAGTCCCTGTGTGAG
AACATGGGCGGGGTGTATCTGGACGGTGAAACCCTGCGTTTTAACCCGTTTGCGAACATCACCGATATTGACCAGTCAGCGGAGCGTGTCCGTGACCAGCTGTCGGTGATGGCCAGCCCCA
ACGGTAACCTGGATGAAGTGCATGAAGGTCTGCTGCTGCAGGCGGTCAGGGCCTCCTGGCTGGCCAAAGAGAACAGGGCACGTATTGATGATGTGGTGGATTTCCTGAAAAACGCCAGT
GACAGCGAGCAGTATGCTGAGTCACCGACCATCCGCAGCCGTCTGGACGAAATGATTGTGCTGCTTGACCAGTACACTGCCAACGGCACTTACGGCCAGTATTTTAACTCTGATGAGCCATC
CCTGCGGGATGATGCGAAAATGGTGGTGCTGGAGCTGGGCGGACTGGAAGACCGTCCGTCACTGTTGGTTGCGGTGATGTTCAGCCTCATTATTTACATCGAGAACAGGATGTACCGCACG
CCGCGTAACCTCAAAAAACTGAACGTTATTGATGAGGGGTGGCGTCTGCTGGACTTCAAAAATCACAAGGTCGGTGAGTTTATTGAGAAAGGTTACCGTACCGCCCGTCGTCATACCGGTG
CCTATATCACCATCACACAGAACATCGTCGACTTTGACTCTGACAAGGCGTCCAGTGCGGCCCGTGCGGCATGGGGTAACTCCTCCTACAAAATTATCCTCAAACAGAGTGCGAAGGAGTTC
GCGAAATACAACCAGCTGTATCCGGACCAGTTCCAGCCACTACAGCGCGACATGATTGGTAAGTTTGGTGCGGCCAGAGATCAGTGGTTCAGTTCCTTCCTGCTGCAGGTGGAAAACCATT
CCTCCTGGCACCGCCTGTTTGTGGACCCGTTAAGCCGCGCCATGTACAGCTCTGACGGCCCGGATTTTGAGTTTGTGCAGCAGAAACGTAAAGAGGGGCTGAGTATTCATGAGGCGGTGT
GGCAGCTGGCGTGGAAGAAGTCAGGGCCGGAGATGGCTTCGCTGGAAGCCTGGCTGGAAGAACATGAGAAATACAGGAGGAGTGTTGCATGA
>DLDPCG_00160 Protein TrbI
ATGGTGTTACTGAACGCAGCCATCAGTTACGGCATTGTCAGACTGAACGCACCGGTGACTGCTGCCTTCAACATGAAACAGACCGTGGATGCGTTTTTTGACAGCGCCAGTCAGAAACAAC
TGTCAGAAGCACAGTCAAAAGCCCTTTCAGCGCGTTTTAACACGGCACTGGAAGCCAGCCTGCAGGCGTGGCAGCAGAAACACCACGCGGTCATTCTGGTGTCGCCTGCCGTGGTACAG
GGGGCTCCGGATATCACCCGGGAAATCCAGCAGGATATTGCCCGGCGAATGAGGGCAGAACCATGA
>DLDPCG_00165 Protein TraW
ATGAAATACCGGGGGCTGATTGCCCTGCTGGTATGGGGGCAGAGTGTGGCCGCAGCCGATCTTGGCACCTGGGGCGATCTGTGGCCGGTACAGGAGCCGGACATGCTGACGGTGATCAT
GCAGCGTCTGACGGCACTGGAGCAGTCCGGTGAGATGGGCCGGAAAATGGATGCGTTTAAGGAGCGGGTGATCCGCAACAGCCTGCGGCCCCCTGCCGTGCCCGGCATCGGACGCACG
GAGAAATACAGCAGCCGGTTGTTTGACCCGTCCGTCAGACTGGCCGCGGATATCCGGGATAACGAAGGGCGGGTGTTTGCCCGCCAGGGTGAGGTGATGAACCCTCTGCAGTATGTGCCT
TTCAACCAGACGCTGTATTTCATCAACGGCGATGATCCGGCGCAGGTGGCATGGATGAAACGCCAGACACCTCCCACACTGGAAAGCAAAATTATCCTCGTGCAGGGCAGTATTCCGGAGA
TGCAGAAGACCCTGGACAGCCGTATTTACTTTGACCAGAACGGTGCGCTCTGCCAGCGTCTCGGCATTGATCAGGTTCCGGCACGGGTGACAGCCGCCTCCGGCGAACGTTTTCTGAAGG
TGGAGTTTATACCGGCAGAGGAGGACAGAAAATGA
>DLDPCG_00170 Protein TraU
ATGAAACGGTGCCTGTGGCTGTGTGTGGTGTTTCTGCTGGCCGGCCATATCCCTGTGGTGTCAGCGGATGCGGCCTGTGAGGGGCGTTTCGTCAACCCGATCACCGATATCTGCTGGAGCT
GTATTTTCCCGCTGTCGCTGGGCAGCACAAAAGTGAGTCAGGGAAAGGTACCGGATACGGCAAACCCGTCAATGCCGATCCAGTTTTGTCCGGCCCCGCCCCCCATTTTTAAACGTATCGG
ACTGGCTATCGGATACTGGGAGCCGATGGCACTGACGGACGTCACCCGGTCACCGGGATGCATGGTGAACCTGGGCTTCAGCCTGCCGGCTTTTGGTAAAACGGCACAGGGAACGGCGA
AAAAGGATGAGAAGCAGGTGAACGGGGCGTTCTATCACGTTCACTGGTACAAATACCCGCTGACGTACTGGCTGAACATCATCACATCGCTGGGCTGTCTGGAAGGTGGTGACCTGGATAT
CGCTTATCTTTCTGAAATTGACCCCACCTGGACGGACAGCAGCCTGACCACCATTCTCAATCCGGAAGCTGTCATCTTTGCCAATCCGATAGCACAGGGATCCTGTGCCGCAGATGCGATTGC
CAGCGCCTTTAATATGCCTCTCGATGTTCTGTTCTGGTGTGCCGGTTCGCAGGGAAGTATGTACCCGTTCAATGGCTGGGTGAGTAATGAGTCCAGTCCGTTGCAGTCCTCCCTGCTGGTCAG
TGAACGCATGGCGTTCAAGCTGCACCGTCAGGGCATGATTATGGAAACCATCGGGAAAAATAACGCCGTCTGTAATGAATATCCGTCTCCAATCCTGCCCAAAGAACGCTGGCGTTACCAGA
TGGTGAATATGTATCCGGACAGCGGACAGTGCCACCCGTTCGGGCGCAGCGTGACCCGCTGGGAAACCGGGAAAAATCCGCCCAACACAAAGAAAAACTTCGGCTACCTGATGTGGCGTA
AACGTAACTGTGTATTCCTGTAA
>DLDPCG_00175 ATP-binding protein
ATGTATAAACAGGCAACAGAATTAATGCTGAACTTTAAAGACCGCATTCTGATTAAGGGGGAAGAAGATACCGGCAAGTCAACTCTCCTGACTGAAATAAGAATCAGTGATAGTGATTCCCG
GTATTACAACTTTAAAACTCTTAACTCTGCGGGGTATAACCAGCTTTGTGATGAAAATATTGATAATTTTGATTTTCTGAATACACCTGAAAAGACGTTAATCCTTGATGGAGTCAGGCTGTGC

GAAAAGAAAATGACATCAAAAGTTATCAGGTTAATTAAACAGGCCAGAAAATATCATAAACGCCTGGTTGTGGTTGCAGACTCATGTGAGTCTGAGTTTATCGAACTGTTGTTTGATGGTGTT
ATTGCATTGTCCTTCAATTCTGACAGAGAAAGGAGCTGCAATGTTTATACGCCATCAAGGTGTCGTAATACAGATAATATCTCGCCATATAAATGA
>DLDPCG_00180 hypothetical protein
ATGTTTATACGCCATCAAGGTGTCGTAATACAGATAATATCTCGCCATATAAATGAGAAGGCGTTTATGAAAGCTGTATTTGTGAGTGTTTTTGTCGCTGTGTTTTTAAAATATTTGTACTCTGAT
GTATTTATGCCAGGTAAATGTGTAAACAAAAACAGAGGAGGGTGTAATGAGTAA
>DLDPCG_00185 DUF4942 domain-containing protein
ATGAGTAATAATATAAAATATGGCTCCAGAATACTTGATATGGGTGTTCTGCTGGAGATGAAAGCGCTTGTGAATGCAAATGGTGGTGTGTCACGGTGCATTATGGAGTGTCGGCAGCTTGCC
AGTGAGGTCCGTCATTCGCAACAATTACTGGATAAGCTGTCATGTATTGACGACTGGCTGTGCCGGTTTTCCTCAATAATGTATCACCGTTTTGATAAAGAACAGATTCAGTGGCGGGAGGAT
GTGGACAGGAATTATCCCAGAGAAAAGGTTTATATTTTCAGTCCCGATAATGTCTCCGTCCATAATAATGATATTAAAGACTCTGCCAATAACACATCAGGAATAACCGTGTCGGTTACAGCCA
GTAAAATATGCGCAGGTCAGAAAGAGTTTGATGCTTTTTTAAAACTGGCAGAAAAACTGAGAGAAAACCCTACGGGTAGTTAA
>DLDPCG_00190 Periplasmic protein TrbC
ATGAACTGGAAAATATTTGCGCTTTTTGCCCTGACTGCAGGAATGCATATCACTGTTCATGCCTCAGACAATGTGAACACTCCTGAAAACCGCCAGTTCCTGAAGCAGCAGGAAAATTTAAG
CAGACAACTGCGTGAAAAAACTGACCATCAGCTGAAAGCCTGGGCGGAGAAACAGGTGCTGGAAAACCCCCTTCAGCGTTCAGATAACCATTTCCTGGATGAGCTGGTTCGTAAACAGCA
GGCTTCGCAGGACGGGAAATCCCGGCAGGGGGCCCTGTATTTTGTGTCGTTTTCCATTCCCGAAGAGGGGCTGAAACGAATGCTGGGCGAAACCCGGCACTACGGTATACCCGCCACACT
GCGGGGCATGGTGAACAATGACCTGAAGGCCACCGCTGAAGCCGTGTTGTCCCTGGTGAAAGACGGCGCGACCGATGGTGTTCAGATCGACCCGACGCTGTTTTCACAGTACGGCATTCG
CAGCGTACCGGCACTGGTGGTGTTCTGCAGTCAGGGATACGACATCATCCGGGGAAACCTGCGGGTCGGCCAGGCGCTGGAGAAAGTGGCCGCCACGGGTGACTGCCGGCAGGTGGCT
CATGATTTACTGGCAGGGAAAGGAGATTCCGGGAAATGA
>DLDPCG_00195 Mating pair stabilization protein TraN
ATGAAACGTATTTTACCTCTGATACTGGCTCTGGTTACCGGCATGGCACAGGCTGACAGCAACAGTGATTACCGGGCCGGCTCTGATTTTGCTCATCAGATCAAAGGACAGGGAAGCAGCA
GTATTCAGGGCTTTCAGCCACAGGAGAATATCCCCGGCTATAACGCGAATCCGGACGAGACAAAATATTATGGTGGCGTGACTGCCGGCGGGGATGGTGGCCTGAAAAATGACGGCACCA
CGGAATGGGCGACCGGTGAAACCGGTAAAACCATCACAGAGTCCTTCATGAACAAGCCGAAAGACATTCTGTCACCGGATGCACCGTTCATTCAGACCGGCAGGGATGTGGTGAACCGG
GCTGACAGCATTGTGGGAAATACCGGACAGCAGTGTAGTGCACAGCAGATAAACCGCAGTGAATTCACAAACTATACCTGTGAACGGGATACGATGGTGGAGGAATTCTGCACACGGACT
GCCAGGATCACCGGAGACTGGAAATACACGGATGAATATCGTGAGGTGACGATACCGCACTCACAGTTCCGGTTCAGTATGAACGGGCTGAAACTGGTGTTCAGTGTCACGGCTCCGGTAA
CCGGTACAGTGGAAAGTGCATCATTGTCTGTATACGCCGCTTTTTTCTTCCTGAATTCTCGTTATACCTTCATGAATTCAACGTTCAATGTTGGCCTGGTAAACGGGCAGTCGGGTACTTTCCC
CTTGTCCGGGGCGACAGGTTTGCAGGTGACACAGGGGCAGGTACTGACCGGCAGTGGCTGTACTGCTAACGGTAACTGTCTTTCTGGCGGGAACGGTGACCGACAGGTTTATGAGAAAC
TGGCCAGCGGCGCGTCCACCTTTACGCTGAAACTCAGGATGAAGGTCCGTGATAAAGAATGGGTTCCACGGGTGGAGTGGGTGGAGAGTTGTCCGTTTAATAAAGCGGATGGCGTGCTG
AAAGGCACTGAGTGTTCAGAGCCGGGTGGTACGAAAACAGGTGTGATGGAAGGTAAACCCTGGAGCATCACACAGGCGTGCTGGGCATACCGGGATAAATATGTCACACAGTCCGCAGAT
AATGGTACCTGTCAGAAATATGTCGATAATCCGGCCTGTACGCTGGCCTCACGTCAGTGTGCGTTTTATTCCGATGAGGGAACCTGTCTGCATGAGTATGCCACGTACTCCTGCGAGTCAAAA
ACATCAGGAAAAGTGATGGTCTGTGGTGGTGATGTCTTCTGCCTTGACGGTGAATGCGATAAGGCACAGAGCGGAAAAAGCAGTGATTTTGGTGCAGCCGTATCACAGCTGGCTGCGCTT
GCCGCTGCTGGTAAGGATGTTGCCGCCCTGAACGGGGTGGATGTCAGGGCTTTCACCGGCGAGGCGAAATTCTGCCGTAAGGCCGCAGCCGGCTTCAGTAACTGCTGTAAGGATGGTGG
CTGGGGTCAGGATGTGGGTCTGGCGAAGTGTAACAGTGAAGAAAAGGCGCTGGGAAAGGCAAAGGACAACAAACTGACGGTGAGTGTTGGCGAGTTCTGTTCGAAAAAGGTACTCGG
TGTCTGCCTGCAGAAAAAGCGCAGCTACTGCCAGTTTGATTCGAAACTGGCGCAGATAGTCCAGCAGCAGGGGCGTAACGGGCAGCTGCGTATCGGGTTTGGCAGCGCCAAAAGTCCTG
ACTGTCGTGGAATAACCGTGGATGAACTGCAGCGTATTAAGTTCGACAGACTGGATTTCACTAACTTCTACGAAGACCTGATGAATAACCAGAAAATCCCGGACAGTGGCGTTCTGACGCAG
AAAGTGAAAGAGCAGATTGCTGACCAGCTGAAACAGGCAGGACAGTAA
>DLDPCG_00200 Protein TrbE
ATGAAGGTCATATTCACTTCTAATCGTATTGTTGATTTTCTTATCCGGCTTTTGATTACAGCGATTGTCATTTCCCCGGTAATTATCTGGTCGTGGGATACGGTAAAAGAAACCACGGTGGACGG
GATGCTTGCTGCTGCTTTCGTTATCCTTTACTCCGGTGTTCTCCTGTTTATTCTGTATTTTTGTTTTTCTGCCCTGACTGACCTGCTGAAACCTGATGAAAGAAAAGATGATGAAAGGAATGAA
GATGAATAA
>DLDPCG_00205 Type-F conjugative transfer system pilin assembly protein TrbC
ATGAATAAAGCATTACTGCCACTGCTACTCTGCTGCTTTATTTTTCCGGCGTCAGGGAAAGATGCAGGCTGGCAGTGGTATAACGAGAAAATAAATCCGAAGGAAGAAGAAAATCAACCTGT
ACCTGCAGCCCCCCGTCAGGAACCGGATATTATGCAGAAACTGGCCGTGCTGCAGACGGCAACAAAGCGGGCGCTGTACGAAGCCATTCTGTATCCCGGCGTGGATAATTTTGTGAAATAT
TTCCGGCTGCAGAATTACTGGACTCAGCAGGCCGGGCTTTTCACCATGAGCGCCAAAAAGGCCATGCTGGCACATCCTGAACTGGACTATAACCTGCAGTACAGCCATTACAACGGCACGG
TCCGGAACCAGCTGGCTGCAGACCAGGCGCAGCAGCGACAGGCCATTGCGAAACTGGCTGAACACTACGGCATCATGTTCTTTTACCGGGGGCAGGACCCTATCGACGGGCAACTGGCG
CAGGTCATTAATGGCTTCAGGGAGACGTATGGTCTGAGTGTTATTCCCGTTTCCGTGGATGGCGTGATTAATCCACTGTTGCCGGATTCCCGGACTGACCAGGGGCAGGCGCAGCGCCTCG
GCGTGAAATATTTCCCGGCCATGATGCTGGTTGACCCGAAACAGGGCAGTGTTCGTCCGTTATCATACGGTTTTATTTCGCAGGACGACCTGGCAAAACAGTTCCTGAACGTTTCTGAAGAC
TTTAAACCGAATTTTTAA
>DLDPCG_00210 GGDEF domain-containing protein
ATGAATTTTATTTCTTTATGGCCTTTATTGCTGGCCCAGGTGACAGTGATGTCACTGACAGTGATTTTCCTTGCTGCTGACAGGCGTTTTTTTTCATGGGACTGTTTTGTTGTTTGCACCCTGTC
CATGATGATGGCAGAAGGTATTGTTATTATGAAATATGAACTGTGGCATATCCCTCCTGCCCTGCTGGGTCTGGCAGGAGTGTTGTCATGGTTGCTGCTGCTACTCCATACAGGATATTTTTCA
GGCATATCAGAAGGAGTGCATAAGCGTTTGTGGCTCGCTGTTGCCGCAGTGTTTTCTGGTGCGGGAATGATTATATTCTACTGGTTGCTCCATGCATCAGAAAACATAATAAAATAA
>DLDPCG_00215 hypothetical protein
ATGAGTTTATTTTTTGACAGAAAAACACTGGAAGCTATCAGAGACATTATCCGGGAAACAATGTTAGGCAATGTTGCCATTGTCTTACTGATAAACATTTCACCGGACTATATGGTACAAAAG
AACAGTCCATCAATTATCATGCTCTCTGTTTTTATATTAATAATAATATGCATTGAACTCTGGATGCTCCTCTCTGTAATAAATTACTTATCCGCGTTACGAATAAGAAGATCAAAACTCGCACTTAT
AATATTTATTCTCTGGATGTGTCTTATTCTCGCATTAACTATTCGTTATTTTATTATGTTTTCTGATGCATGGAGCAACCAGTAG
>DLDPCG_00220 Protein TraQ
ATGAGAAAATATAAATTCTCTTTACCCCGGCTTGATATTACAGGAATGTGGGTATTTTCCCTGGGTGTCTGGTTTCATATCGTCGCGCGTCTTGTTTACAGTAAACCCTGGATGGCTTTTTTTCT
GGCGGAGTTGATTGCGGCCATCCTTGTGCTGTTCGGCGCATACCAGGTGCTGGATGCATGGATTGCCCGTGTCAGCCGGGAAGAGCGTGAGGCACTGGAAGCCAGGCAACAGGCCATGA
TGGAAGGGCAGCAGGAGGGCGGACATGTCTCTCACTAA
>DLDPCG_00225 Type-F conjugative transfer system pilin assembly thiol-disulfide isomerase TrbB
ATGTCTCTCACTAAATTACTGCCGGTCATTCTGTTGATGATGGCCACAGGTGTGCAGGCCTCCACCCGGGATGAAATAGAGCGACTCTGGAATCCACAGGGTATGGCCGCGAAGCCTGCACA
ACCGGCAGCAGGCGTGCCAGCCAGAACAGAAAAGCCGGCTCCCCGCTGGTTCCGCCTCAGTAATGGCAGGCAGGTTAACCTGGCCGACTGGAAGGTGGTGCTGTTTATGCAGGGGCATT
GCCCTTACTGTCACCAGTTTGACCCGGTACTGAAACAGCTGGCGCAACAGTACGGCTTTTCCGTTTTTCCCTACACCCTGGATGGTCAGGGTGATACGGCCTTTCCGGAGGCATTGCCGGTG
CCACCGGACGTGATGCAGACCTTCTTCCCGAATATCCCGGTGGCCACACCGACCACCTTCCTGGTCAACGTCAACACGCTTGAGGCATTACCGCTTTTACAGGGGGCAACGGATGCCGCCG
GTTTTATGGCGCGGATGGATACCGTTCTGCAGATGTACGGAGGAAAAAAAGGTGCGAAATAA
>DLDPCG_00230 hypothetical protein
GTGCGAAATAAGCAGGTGGTGTTACTCATTGCCGGAATATCCGGCATCGCGACGGGAATAATCGTCAGCCTGAATATCCCCTTTATCCGTCAGGGGCTTTTTTATCCCGCCAGCCCTGTAGAA
ATTGTCGTTTCGCTGTGTCTTACCTTTTCTGTTTCGGTTGTGTTTTTTGTGGGGGCAATTGTGGGATGGATTTTGGTGTCTGAAATATATTACAGCAGCATGACCGGCCTGAATAAATTATCAG
AGGAAACTTATAATGAAAGAAAATAA
>DLDPCG_00235 Protein TrbF

ATGAAAGAAAATAAAAATAATCAGGAATTCAAAATCAGAAAAATTAAACGGGATATTGAGCGCAGTTGTGATAATTCCGAAAAATACTTCTGGCTTTTTGTGGTTTTCTTTGTTGCCGGATTG
CTTTTATGGCGCGGTATGCATGCTATTTTTAGCTCCGGTATTGATTCGTGGAAAGCTGACCCGGAATTAAATAATTTTCGTTATATGTGGAACATCCTGATGTATGCCATTCCGTATACGTTATATG
CGCTTGCAGCCGGGTTTCTTGTTACATTTTTTCTGTCCCCAATGTACGAATTAATATTCGGGAATATCATGATATTCCTGCTGAAACGCCGTATGCGCCGGGAGAACAAATTAAGGGAGGGCA
GCAATAATGCTTCGCATTAA
>DLDPCG_00240 Conjugal transfer protein TraH
ATGCTTCGCATTAAACCTCTTTTTATTCTTTGTGCCGCCTTACTGACGGTGACGCCTGCAGCGTCAGCGGATGTGAACAGCGATATGAATCAGTTCTTTAACAAGCTGGGCTTCGCTTCCAATA
CCACCCAGCCGGGCGTCTGGCAGGGGCAGGCTGCCGGCTATGCTTACGGTGGCTCCCTGTATGCCCGTACTCAGGTCAAAAATGTTCAGCTGATTTCCATGACGCTGCCGGATATCAACGCC
GGATGTGGCGGCATCGACGCCTATCTTGGTTCGTTCAGTTTTATTAATGGCGAGCAGCTGCAGCGTTTTGTTAAGCAGATTATGAGCAATGCTGCCGGTTACTTTTTCGATCTTGCCCTGCAG
ACAACGGTGCCGGAAATCAAAACCGCAAAAGACTTCCTGCAGAAAATGGCAAGCGACATTAACAGTATGAACCTCAGTTCCTGCCAGGCGGCACAGGGGATTATCGGCGGGCTTTTCCCC
CGGACGCAGGTGTCCCAGCAGAAAGTCTGTCAGGACATTGCCGGTGAGAGCAATATTTTTGCTGACTGGGCGGCTTCCCGGCAGGGATGTACCGTTGGCGGGAAATCTGACAGCGTCAG
GGATAAAGCCGGCGACAAGGATAAGGAGCGGGTGACCAAAAACATCAACATCATGTGGAATGCGCTTTCCAAAAACAGAATGTTTGACGGCAACAAAGAGCTGAAAGAGTTTGTGATGA
CGCTGACCGGCTCACTGGTGTTTGGTCCCAACGGCGAAATCACACCGCTGTCAGCCAGAACCACTGACCGCTCAATTATCCGGGCCATGATGGAAGGCGGCACAGCAAAAATTTATCACTG
CAACGATTCTGATAAATGCCTGAAAGTGGTGGCAGACACACCGGTGACCATCAGCCGGGATAATGCACTGAAGTCTCAGATTACTAAACTTCTGGCCAGCATTCAGAACAAGGCGGTCAGT
GACACGCCTCTGGATGACAAGGAAAAAGGCTTTATTTCCAGTACCACCATCCCCGTCTTCAAATACCTGGTTGACCCGCAGATGCTCGGTGTTTCCAACAGCATGATTTACCAGCTGACGGA
CTATATCGGCTACGACATCCTGCTGCAGTACATTCAGGAGCTGATACAGCAGGCCCGGGCGATGGTGGCCACGGGAAATTATGACGAAGCGGTTATCGGGCATATTAACGACAACATGAATG
ATGCCACCCGGCAGATTGCGGCGTTTCAGTCACAGGTGCAGGTACAGCAGGATGCGCTGCTGGTTGTCGATCGTCAGATGAGCTACATGCGTCAGCAGCTTTCCGCCCGCATGCTCAGTCG
TTACCAGAACAACTATCACTTCGGAGGGAGCACGCTGTGA
>DLDPCG_00245 Protein TraG
GTGAATGAAGTTTATGTGATTGCCGGTGGTGAGTGGTTGCGGAATAACCTGAATGCCATTGCCGCCTTTATGGGAACCCGGACGTGGGATTCCATTGAGAAAATTGCTCTCACCCTGTCTGT
TGTTGCGGTGGCCTTTATGTGGGTACAGCGCCATAACGTGATGGATTTGCTGGGCTGGGCGGCGGTGTTTGTACTTATCAGCCTGCTGGTTAATATCCGTACGTCGGTGCAGATTATTGATAA
CAGTGACCTGGTCAAAGTCCATCGGGTGGATAATGTGCCGGTCGGACTGGCGATGCCACTTTCACTGACGACCCGTATCGGGCATGCAATGGTGGCCAGTTACGAGATGATCTTCACGCAA
CCGGACAGTGTCACCTACAGCAAAACGGGGATGCTGTTTGGCGCAGAACTGGTATCAAAAAGCACCGACTTTTTGTCCCGCAATCCGGAAATCGCCAATCTTTTCCAGGATTATGTGCAGA
ACTGCGTGATGGGGGATATTTACCTGAACCACAAATACACGCTGGAGGAGCTGATGGCCTCCGCTGATCCCTACACGCTGATTTTTTCCCGCCCCAGCCCGCTGCGGGGCGTTTATGACAGC
AATAATAATTTCGTGACCTGTAAGGACGCGTCGGTTTCGCTGAAAGACAAGCTGAATCTGGACACACAGAGTGGCGGAAAAACCTGGCATTATTATGCCCAGCAGCTATTTGGTGGACGCC
CTGATCCTAACCTGCTGTTCAGTACGCTGATTGGCGACAGTTACAGTTACTTCTATGGCTCAAGTAAATCAGCCAGCCAGATCATCCGCCAGAATGTCACCATTAATGCCCTGAAGGAGGGGA
TCACCAGTTATGCAGCACGTAATGGTGATTCAGCCAGCCTGGTGAATCTGGCCACCACGTCATCGATGGAGAAGCAACGTCTGGCACATGTCTCCATCGGCCATGTGGCGATGCGGACACTG
CCCATGACGCAGACCATCCTGACGGGGATCGCCATCGGTATTTTCCCGCTTCTGGTACTGGCGGCTGTCTTCAACAAACTGACGCTGTCCGTCCTGAAAGGCTATGTGTTTGCCCTGATGTGG
TTGCAGTCATGGCCAATGTTGTACGCCATTCTGAACAGCGCCATGACATTTTATGCGAAGCAGAATGGTGCACCGGTGGTGCTCTCGGAAATTTCACAGATACAGCTGAAATACTCCGATCTG
GCTTCCACTGCCGGGTATCTTTCCATGATGATCCCGCCATTATCCTGGATGATGGTCAGGGGGCTCGGGGCCGGATTTTCCAGTGTGTACAGCCATTTCGCTTCCTCTGCTATCAGCCCGACTG
CCAGTGCGGCAGGCAGTGTGGTTGACGGTAATTACTCCTATGGCAACATGCAGACGGAGAACGTGAACGGCTTCAGCTGGAGCACCAACAGCACCACGTCGTTTGGTCAGATGATGTACC
AGACCGGAAGTGGCGCAACTGCCACACAGACCCGTGACGGTAATATGGTGATGGATGCAAGCGGAGCGATGTCCCGGTTACCGGTTGGTATCAATGCAACGCGTCAGATTGCAGCGGCAC
AACAGGAAATGGCCCGGGAGGCGTCGAACAGAGCAGAAAGTGCCCTGCATGGGTTCAGCAGCAGTATTGCCAGTGCCTGGAACTCGCTCAGTCAGTTTGGTTCTAACCGGGGGAGCAGT
GACTCTGTCACCGGTGGTGCTGACAGCACCATGAGTGCACAGGATTCCATGATGGCCAGCCGTATGCGCAGTGCAGTGGAAAGCTATGCGAAAGCGCATAATATCAGTAATGAGCAGGCGA
CGAGAGAACTGGCCTCGAGAAGTACAAATGCGTCGCTTGGCCTTTACGGCGATGCTTATGCTAAAGGGCATTTAGGCATAAGTGTAATGGGTAACGGTGGAGGGGTTGGTTTCCAGGCTG
GAGCTAAAGCAAGCATTGATGGCAGTGATCTTGACTCACATGAAGCCAGTAGTGGTTCACGGGCCAGCCATGATGCTCGTCATGATATTGACGCTAAAGCCAGCAAGGACTTTAAAGAGGC
CAGCGATTACTTTACCAGTCGCAAGGTCAGTGAATCCGGCAGCCATACTGACAATAATGCCGATTCCCGTGTGGATCAGCTGTCTGCAGCCCTGAACTCAGCGAAACAGAGTTACGACCAGT
ACACGACGAATATGACCCGCAGCCATGAATATGCTGAAATGGCATCCCGTACTGAAAGCATGAGTGGGCAGATGAGTGAAGACTTGTCGCAACAGTTTGCACAGTATGTGATGAAACACGC
GCCGCAGGATGCAGAAGCTATTCTGACAAACACCAGTTCACCGGAGATTGCAGAACGCCGTCGGGCTATGGCGTGGTCTTTTGTGCAGGAACAGGTACAGCCTGGTGTTGATAATGCCTG
GCGTGAATCCCGTGGCGATATTGGTAAAGGAATGGAGAGCGTACCTTCGGGCGGTGGCAGCCTGGATATTATTGCTGACCATCAGGGACATCAGGCCATTATTGAACAGAGAACGCAGAAC
AGTAATATCCGTAATGATGTAAAACATCAGGTTGATAATATGGTCACAGAATATCGAGGCAATATCGGAGATACTCAGAACAGCATCCGCGGCGAGGAAAATATTGTAAAAGGACAGTATTCT
GAACTACAAAATCATCATAAAACAGAGGCATTATCTCAGAATAATAAATATAATGAAGAAAAGTTGGCTCAGGAAAGAATACCTGGCGCTGATAGTCCGAAAGAACTTTTAGAGAAAGCAAA
GAGTTACCGGCATAAAGAATAA
>DLDPCG_00250 Antisense to traG
TGGCTGATTTACTTGAGCCATAGAAGTAACTGTAACTGTCGCCAATCAGCGTACTGAACAGCAGGTTAGGATCAGGGCGTCCACCA
>DLDPCG_00255 Protein TraS
ATGATTACACAACAAATCATCAGTAGTGAGTTGGAAGTTTTAAAGAAACATATTGACTCAGGAGATATTAGAATCCCGTCTCTATGGCAGGGATTAAAACCTGGACTTATCATCATGGGATGG
ATGATTTTTTGTCCCTTGCTAATGTCGTTTCTAATAACACAAAAGACAAGTGAAACACTCACTGCTGTGTTAGCCGGAGGATGGCTTGGGCTTATCATTCTGTTTATTGTCGCAAGGATTAGAA
TGTTATATTTCTCCCTGCCGGAAGAATTCCTAAAGACATCATTGGTAATGAGGATTATATCATCAAAACTTAAAGTGTATTTCATTGTGTATATGGGAATTATATTTTTATGGTCTTTTTTAGGTGG
AGGGATTATATACGGTTTTGGCGCAATTTTAGTGACGGTTATTATGGCGTTCTTGATTCAATTAGATATCGGTAGATATCAATTTGTTGGTGTTATTGATGCAATCAATAGTTACGTCAAAAACAA
GAAGTTATCAAGAGTAAAATAA
>DLDPCG_00260 TraT complement resistance protein
ATGATGAAAACAAAAAAACTAATGATGGTTGCACTGGTCAGTTCCACTCTGGCCCTTTCAGGGTGTGGTGCGATGAGCACAGCAATCAAGAAGCGTAACCTTGAGGTGAAGACTCAGATG
AGTGAGACCATCTGGCTTGAACCCGCCAGCGAACGCACGGTATTTCTGCAGATCAAAAACACGTCTGATAAAGACATGAGTGGGCTGCAGGGCAAAATTGCTGATGCTGTCAAAGCAAAA
GGGTATCAGGTGGTGACTTCTCCGGATAAAGCCTACTACTGGATTCAGGCGAATGTGCTGAAGGCCGATAAGATGGATCTGCGGGAGTCTCAGGGATGGCTGAACCGTGGTTATGAAGGC
GCAGCAGTTGGTGCAGCGTTAGGTGCCGGTATCACCGGATATAATTCCAGTTCTGCCGGTGCCACACTCGGTGTGGGCCTTGCTGCCGGTCTGGTGGGTATGGCTGCAGATGCGATGGTGG
AAGATGTGAACTATACCATGATCACGGATGTGCAGATTGCAGAGCGTACTAAGGCAACGGTGACAACGGATAATGTTGCCGCCCTGCGTCAGGGCACATCAGGTGCGAAAATTCAGACCAG
TACTGAAACAGGTAACCAGCATAAATACCAGACCCGTGTGGTCTCAAATGCGAACAAGGTTAACCTGAAATTTGAAGAGGCGAAGCCTGTTCTCGAAGACCAGCTGGCCAAATCAATCGCA
AATATTCTCTGA
>DLDPCG_00265 Coupling protein TraD
ATGAGTTTTAACGCAAAGGATATGACCCAGGGCGGTCAGATTGCGTCCATGCGTATCCGCATGTTCAGCCAGATCGCCAATATCATGCTTTACTGCCTGTTTATTTTTTTCTGGATACTCGTTG
GTCTGGTTTTATGGGTAAAAATAAGCTGGCAGACGTTTGTGAACGGCTGTATTTACTGGTGGTGTACCACGCTGGAAGGCATGCGGGATTTAATCAAGTCCCAGCCGGTATATGAGATCCAG
TATTACGGCAAAACCTTCCGGATGAACGCTGCTCAGGTACTGCATGATAAATATATGATCTGGTGCGGAGAGCAGCTATGGTCCGCATTCGTTCTGGCCACAGTTGTGGCACTGGTTATTTGC
CTGATTACCTTCTTTGTTGTCTCCTGGATTCTGGGGCGTCAGGGTAAACAACAGAGCGAAAATGAAGTCACAGGTGGTCGTCAGCTGACAGACAATCCGAAAGACGTTGCCCGGATGCTGA
AAAAAGACGGCAAGGACTCCGATATCCGGATTGGCGACCTGCCGATTATCCGGGATTCTGAAATCCAGAACTTCTGTCTGCACGGCACGGTCGGGGCCGGTAAGTCGGAAGTTATCCGTCG
TCTGGCCAACTACGCCCGTCAGCGTGGAGATATGGTGGTGATTTATGACCGTTCAGGGGAATTTGTTAAAAGTTACTATGATCCCTCCATCGATAAGATCCTGAATCCGCTGGATGCACGCTG
TGCCGCCTGGGATTTATGGAAGGAGTGTCTGACACAGCCGGATTTTGATAATACGGCAAATACCCTGATCCCGATGGGCACAAAAGAGGACCCGTTCTGGCAGGGTTCAGGACGTACCATT
TTTGCGGAAGCGGCGTACCTGATGCGTAATGACCCCAACCGCAGCTACAGCAAACTGGTGGACACACTGCTTTCCATCAAAATCGAAAAACTGCGTACCTACCTGCGTAATTCACCGGCGG
CCAACCTGGTGGAAGAGAAAATTGAGAAAACGGCGATTTCCATCCGTGCTGTGCTGACCAACTACGTGAAAGCCATCCGTTACCTGCAGGGGATTGAGCATAACGGTGAACCCTTCACCAT
CCGTGACTGGATGCGGGGTGTCCGGGAAGATCAGAAAAACGGCTGGCTGTTTATTTCGTCGAACGCCGACACCCATGCCTCCCTGAAGCCGGTGATCTCCATGTGGCTGTCCATTGCCATT
CGTGGTCTGCTGGCAATGGGAGAAAACCGTAACCGTCGTGTGTGGTTTTTCTGTGACGAGTTACCCACGTTACACAAACTGCCGGACCTGGTGGAGATCCTGCCGGAAGCCCGTAAGTTCG

GTGGCTGTTATGTGTTTGGTATCCAGTCCTATGCCCAGCTGGAAGATATCTACGGTGAGAAAGCGGCTGCCACGCTGTTTGACGTCATGAACACCCGTGCCTTTTTCCGTTCTCCCAGCCATA
AGATTGCAGAGTTCGCTGCCGGTGAGATTGGTGAGAAAGAGCACCTGAAAGCCAGCGAGCAGTATTCCTACGGTGCTGATCCGGTACGTGACGGGGTATCGACCGGTAAGGATATGGAGC
GCCAGACGCTGGTCAGTTATTCCGACATTCAGTCTCTGCCGGATCTGACCTGTTATGTCACCCTGCCCGGACCGTATCCGGCAGTAAAACTCTCTCTGAAATATCAGACACGACCGAAGGTCG
CTCCGGAGTTTATTCCGCGTGACATCAATCCGGAAATGGAGAACCGTCTGAGTGCCGTACTTGCTGCAAGGGAAGCAGAAGGTCGCCAGATTGCCAGCCTCTTCGAACCGGAGGTTGCCT
CCGGAGAAGACGTGACTCAGGCTGGACAGCCACAACAGCCACAACAGCCACAACAGCCACAACAGCCACAACAGCCACAACAGCCACAACAGCCACAACAGCCACAACAGCCACAACA
GCCACAACAGCCACAACAGCCACAACAGCCAGTGTCTTCTGTCATCAATGATAAGAAGTCAGATGCAGGTGTGAATGTTCCGGCAGGGGGGATCGAGCAGGAGCTGAAAATGAAACCGG
AAGAAGAGATGGAACAGCAACTGCCACCCGGGCTCAGTGAATCCGGTGAAGTGGTGGATATGGCCGCTTATGAGGCATGGCAACAGGAAAATCATCCGGACATTCAGCAGCAGATGCAG
CGTCGTGAAGAGGTGAACATTAATGTGCACCGGGAGCGCGGGGAGGATGTTGAGCCGGGAGATGATTTCTGA
>DLDPCG_00270 Ribonuclease VapC
ATGCTGAAGTTTATGCTCGATACCAACATCTGCATTTTTACGATAAAGAACAAACCCGCCAGCGTCAGGGAGCGTTTAACCTGAACCAGGGGAGAATGTGTATCAGTTCGGTCACCCTGAT
GGAGCTGATATATGGTGCAGAAAAAAGCCAGATGCCTGAACGTAATCTCGCTGTGATCGAGGGATTTGTTTCCCGCATTGATGTTCTGGATTACGACACTGCTGCAGCCACACACACCGGCC
AGATAAGAGCAGAACTTGCCCGTCAGGGACGCCCTGTCGGGCCATTTGATCAAATGATCGCAGGTCATGCCCGCAGTCGGGGGCTGATTATTGTGACTAATAACACCCGGGAATTTGAACG
TGTGTGCGGCCTGAGAATTGAAGACTGGAGCTGA
>DLDPCG_00275 toxin-antitoxin system antitoxin VapB
ATGGAAACCACCGTATTTCTCAGCAACCGCAGCCAGGCGGTCAGACTGCCAAAAGCGGTTGCATTGCCGGAAAACGTAAAACGCGTTGAAGTGATTGCTGTCGGACGAACCAGAATCATT
ACGCCAGCCGGAGAGACATGGGACGAATGGTTCGACGGAAACAGCGTCAGCGCCGATTTTATGGATAACAGGGAACAGCCCGGTATGCAGGAAAGGGAGTCATTCTGA
>DLDPCG_00280 Conjugative transfer relaxase protein TraI
ATGATGAGTATCGCGCAGGTCAGATCGGCCGGAAGTGCCGCTAACTATTATACCGACAAGGATAATTACTATGTGCTGGGCAGCATGGGAGAACGCTGGGCCGGCAGGGGGGCTGAACAG
CTGGGGCTGCAGGGCCGCGTCGATAAGGATGTCTTTACCCGTCTTCTGGAGGGCAGGCTGCCGGACGGAGCGGATCTAAGCCGCATGCAGGATGGCAGTAACAGGCATCGTCCCGGCTAC
GATCTGACCTTCTCCGCCCCCAAAAGTGTCTCCATGATGGCCATGTTAGGTGGCGATAAGCGCCTGATTGATGCACATAATCAGGCCGTGGATTTTGCTGTCCGTCAGGTGGAGGCGCTGGC
CTCCACACGGGTGATGACGGACGGACAGTCAGAAACGGTACTGACCGGTAATCTGGTGATGGCACTGTTTAACCACGACACCAGTCGCGATCAGGAACCACAGTTACACACGCATGCGGT
GGTGGCTAATGTCACGCAGCATAACGGTGAGTGGAAGACACTGAGCAGTGACAAAGTGGGGAAAACGGGGTTCATTGAGAATGTGTACGCTAATCAGATTGCCTTTGGCAGGCTCTACCG
GGAAAAACTGAAAGAGCAGGTTGAGGCGCTGGGCTATGAAACCGAAGTGGTGGGTAAGCACGGTATGTGGGAAATGCCGGGCGTACCGGTGGAGGCCTTTTCCGGACGCTCACAGGCT
ATCCGGGAGGCCGTCGGGGAAGATGCCTCGCTGAAATCCCGGGATGTGGCGGCACTGGATACGCGAAAATCCAAACAGCACGTCGATCCTGAAGTCAGAATGGCTGAATGGATGCAGAC
GCTGAAGGAAACCGGGTTCGACATCCGGGCATATCGTGACGCGGCGGATCAGCGTGCGGAGACCCGCACGCAGGCGCCCGGGGCTGTTTCACAGGAGGGGCCGGATGTGCAGCAGGC
GGTGACACAGGCGATTGCCGGATTAAGTGAACGAAAAGTGCAGTTTACGTACACGGACGTGCTGGCCAGAACGGTCGGTATACTGCCGCCGGAAAATGGTGTGATTGAACGGGCACGCG
CCGGTATCGATGAGGCCATCAGCCGTGAGCAACTCATCCCCCTTGACCGTGAGAAGGGGCTGTTCACGTCCGGGATTCATGTGCTCGATGAACTGTCAGTCCGGGCACTCAGTCGTGACAT
CATGAAACAGAACCGGGTGACCGTACATCCGGAGAAAAGTGTCCCCCGGACGGCCGGTTACAGCGATGCCGTCAGTGTTCTGGCACAGGATCGCCCGTCGTTGGCCATTGTGTCCGGGCA
GGGCGGTGCAGCCGGGCAGCGTGAGCGGGTGGCTGAACTGGTCATGATGGCCCGGGAGCAGGGGCGGGAGGTGCAGATTATCGCTGCTGACCGTCGTTCGCAGATGAACCTGAAACA
GGATGAACGGCTGTCCGGTGAGCTGATAACCGGACGTCGTCAGCTGCAGGAGGGCATGACCTTCACGCCGGGCAGTACTGTTATCGTTGACCAGGGCGAAAAACTCTCCCTGAAAGAGA
CGTTAACCCTGCTGGACGGTGCCGCACGTCATAACGTACAGGTCCTGATAACCGACAGCGGGCAGCGAACCGGTACAGGCAGTGCGCTGATGGCCATGAAGGATGCCGGGGTGAACACAT
ATCGCTGGCAGGGGGGAGAACAGCGACCGACCACCATCATCAGTGAACCGGACCGTAATGTCCGCTATGCCCGGCTGGCAGGAGATTTTGCGGCCAGCGTGAAAGCCGGAGAAGAGAG
TGTGGCACAGGTCAGCGGGGTACGGGAACAGGCCATTCTGACACAGGCCATTCGCAGTGAGCTGAAAACGCAGGGCGTGCTCGGACACCCGGAGGTAACCATGACCGCCCTGTCACCG
GTCTGGCTGGACAGCCGGAGCCGTTATCTGCGGGATATGTACCGTCCGGGGATGGTGATGGAGCAGTGGAACCCGGAGACACGCAGTCATGACCGCTATGTTATCGACCGGGTGACGGCG
CAGAGTAACAGCCTGACCCTGCGGGATGCGCAGGGTGAAACGCAGGTGGTGCGTATTTCCTCCCTGGACAGCAGCTGGTCGCTGTTCCGGCCGGAAAAAATGCCGGTGGCAGACGGCGA
GCGACTGAGGGTGACAGGGAAAATTCCCGGACTCCGCGTCTCCGGCGGTGACCGCCTGCAGGTGGCATCCGTCAGTGAAGATGCGATGACGGTTGTTGTGCCGGGGCGGGCTGAACCG
GCCTCCCTGCCTGTGAGCGATTCACCATTCACGGCACTGAAGCTGGAGAACGGCTGGGTGGAAACGCCCGGGCATTCCGTCAGCGACAGTGCGAAGGTTTTTGCCTCCGTCACACAGATG
GCGATGGACAACGCGACCCTGAACGGTCTGGCCCGCAGTGGTCGTGATGTCCGGTTGTATTCCTCACTGGATGAAACCCGTACTACGGAAAAACTTGCCCGCCATCCCTCCTTTACGGTGGT
TTCTGAGCAGATAAAGGCGCGTGCCGGTGAGACATTGCTGGAAACCGCTATCAGTCTGCAGAAAGCCGGGCTTCACACGCCGGCACAGCAGGCCATTCATCTGGCCCTTCCTGTGCTGGA
AAGTAAAAACCTGGCCTTCAGCATGGTGGACCTGCTGACAGAGGCGAGGTCGTTTGCTGCAGAAGGAACCAGTTTTACTGAACTGGGAGGGGAAATCAATGCGCAGATAAAACGCGGTG
ACTTACTGTATGTGGATGTGGCAAAAGGCTATGGCACAGGCCTGCTGGTTTCCCGTGCGTCGTATGAGGCAGAAAAGAGCATTCTTCGCCATATTCTCGAAGGTAAGGAGGCGGTCACGCC
GCTGATGGAGAGAGTACCTGGCGAACTCATGGAGAAACTGACATCAGGACAGCGCGCCGCCACCCGCATGATACTGGAAACGTCCGACCGTTTCACGGTGGTACAGGGCTATGCCGGTGT
GGGTAAGACCACACAGTTCCGGGCGGTGATGTCAGCCGTGAACATGCTGCCGGAGAGTGAACGTCCCCGTGTCGTGGGGCTGGGGCCCACACACCGTGCGGTCGGGGAGATGCGCAGC
GCCGGCGTGGATGCACAGACACTGGCGTCCTTTCTGCATGACACGCAGCTGCAGCAGCGCAGCGGAGAAACGCCGGATTTCAGCAACACGCTGTTCCTGCTCGATGAGAGCTCTATGGAG
GGCAATACCGACATGGCACGGGCATACGCCCTGATTGCGGCCGGTGGTGGTCGTGCCGTGGCCAGCGGTGACACGGACCAGCTGCAGTCCATCGCGCCCGGTCAGCCTTTCCGTCTCCAG
CAGACGCGCAGTGCTGCCGATGTGGCCATCATGAAGGAGATTGTGCGTCAGACGCCGGAACTGCGGGAGGCGGTATACAGCCTGATTAACCGGGATGTGGAAAGGGCACTGTCCGGGCT
TGAGAGTGTGAAACCGTCTCAGGTGCCACGTCAGGAGGGCGCATGGGCACCGGAGCACTCCGTGACGGAGTTCAGTCACAGCCAGGAAGCGAAACTGGCAGAAGCGCAGCAGAAGGC
GATGCTGAAAGGCGAGGCTTTCCCGGATGTCCCCATGACACTGTATGAAGCCATTGTCCGCGACTATACCGGCAGAACACCGGAAGCACGGGAGCAGACGCTGATTGTCACGCACCTGAA
TGAGGACCGGCGCGTACTGAACAGCATGATTCATGATGCACGGGAAAAGGCCGGTGAGCTGGGGAAAGAGCAGGTCATGGTGCCTGTCCTGAACACAGCGAATATACGCGACGGAGAG
CTGCGTCGTCTCTCCACTTGGGAGACACATCGGGACGCACTTGTCCTGGCGGATAATGTGTATCACCGGATTGCCGGTATCAGTAAGGATGACGGGCTGATAACCCTGCAGGATGCGGAAG
GTAACACGCGGCTGATTTCGCCCCGGGAGGCGGTGGCTGAAGGCGTCACACTGTACACCCCGGACACCATCCGGGTGGGAACCGGTGACCGGATGCGCTTCACGAAGAGTGACCGGGA
GCGCGGTTATGTGGCCAACAGCGTCTGGACGGTGACAGCAGTTTCCTGTGACAGTGTCACACTGTCGGACGGACAGCAGACCCGGGTGATTCGCCCCGGCCAGGAGCGGGCAGAGCAA
CATATTGACCTGGCCTATGCCATCACCGCCCACGGTGCGCAGGGGGCAAGTGAAACCTTTGCCATCGCGCTTGAAGGCACGGAAGGTAACCGGAAACAGATGGCCGGCTTTGAGTCAGCC
TACGTGGCCCTGTCGCGTATGAAGCAGCATGTGCAAGTGTACACCGATGACCGTCAGGGCTGGACGGATGCCATCAACAATGCCGTACAGAAAGGAACTGCCCACGATGTACTGGAGCCG
AAAGCTGACCGGGAGGTCATGAATGCAGAGCGGCTGTTCAGTACGGCACGGGAGCTGCGGGACGTGGCGGCAGGCCGAGCCGTTCTTCGTCAGGCGGGGCTGGCCGGGGGAGACAGT
CCTGCACGGTTTATTGCACCGGGACGTAAATATCCGCAACCGTATGTGGCACTGCCGGCGTTTGACCGTAACGGCAGGTCCGCCGGTATCTGGCTGAACCCACTGACCACGGATGACGGAA
ACGGGCTGCGGGGATTCAGCGGTGAAGGCCGTGTGAAGGGCTGCGGGGATGCGCAGTTCGTGGCCCTGCAGGGCAGCCGTAACGGAGAGAGCCTGCTGGCTGATAACATGCAGGATG
GTGTCCGGATTGCCCGTGATAATCCTGACAGCGGTGTGGTGGTGAGAATCGCCGGTGAAGGTCGTCCGTGGAATCCCGGTGCCATCACCGGTGGCCGCGTGTGGGGGGACATCCCGGACA
ACAGTGTCCAGCCGGGTGCCGGAAACGGTGAGCCGGTCACGGCAGAGGTACTGGCACAGCGGCAGGCTGAAGAGGCCATCCGCCGTGAAACGGAACGCCGTGCGGATGAAATTGTCCG
TAAAATGGCAGAAGAACAAACCTGA
>DLDPCG_00285 Antisense to traI
CGGCGCGCTGTCCTGATGTCAGTTTCTCCATGAGTTCGCCAGGTACTCTCTCCATCAGCGGCGTGACCGCCTCCTTA
>DLDPCG_00290 hypothetical protein
GTGAAACGGAACGCCGTGCGGATGAAATTGTCCGTAAAATGGCAGAAGAACAAACCTGACCTGCCGGACGGCAAAACAGAGCAGGCTGTCAGGGATATTGCCGGGCTGGAGCGTGACC
GGTCTGCCATATCTGAACGGGAAGCCGCGCTGCCGGAGAGTGTACTGCGTGAACCACAACGGGTGCGGGAAGCGGTCCGGGAGGTTGCCCGGGAAAATCTGCTGCAGGAGCGACTGCA
GCAGATGGAGCGGGATATGGTTCGTGACCTGCAGAAAGAGAAAACCCTGGGCGGAGACTGA
>DLDPCG_00295 Conjugal transfer pilus acetylation protein TraX
ATGACAACGGATAACACGAACACAACACGTAATGATTCACTGGCTGCCCGGACCGATACCTGGTTGCAGTCGTTGCTGGTCTGGTCACCCGGACAGCGGGATATCATCAAAACGGTGGCAC
TGGTGCTGATGGTTCTGGACCATGCAAACCGCATTCTGCATCTGGACCAGTCGTGGATGTTTCTGGTCGGGCGTGGGGCCTTTCCGCTGTTTGCCCTGGTGTGGGGGCTGAATCTGTCCCG
TCATACGCATATCCGGCAGGAGGCCATTAACCGGCTGTGGGGATGGGCGGTGATTGCCCAGTTCGCATATTATCTGGCCGGCTTTCCCTGGTATGAGGGGAATATTCTGTTTGCCTTTGCAGT

TGCGGCTCAGGTGCTGACATGGTGTGAAACGCGCAGTGGGTGGCGTACTGCAGCGGCCATACTCCTGATGGCACTGTGGGGGCCTTTGTCCGGCACCAGTTACGGTATTGCCGGGCTGCT
GATGCTGGCAGTCAGCCACAGGCTGTACCGGGCGGAAGACAGAGCGGAACGTCTGGCGCTGGTCGCCTGCCTGCTGGCCGTTATTCCGGCGCTTAATCTTGCCACCAGTGATGCCGCGGC
GGTGGCCGGCCTTGTGATGACGGTGCTGACCGTTGGTCTGGTGTCCTGTGCAGGAAAATCATTACCCCGTTTCTGGCCCGGGGATTTTTTCCCGACGTTCTATGCCTGTCATCTGGCTGTGC
TGGGCGTTCTGGCACTGTGA
>DLDPCG_00300 Fertility inhibition protein
ATGACAGAGCAGAAGCGACCGGTACTGACACTGAAGCGGAAGACAGAGGGAACAGCGCCTGTCCGCAGCCGGAAAACCATCATCAATGTCACCACGCCACCAAAATGGAAGGTGAAAA
AGCAGAAACTGGCCGAGAAGGCGGCCCGGGAAGCAGAGCTGGCGGCAAAAAAAGCGCAGGCCAGACAGGCGCTGTCCATTTATCTGAACCTGCCCACGCTGGATGAGGCCGTGAACA
CCCTGAAGCCCTGGTGGCCGGGATTATTTGACGGTGACACGCCCCGGCTTCTGGCCTGCGGTATCCGGGACGTGTTACTGGAAGACGTGGCGCAGCGGAATATCCCGCTTTCGCATAAAAA
ACTGCGCAGGGCGCTGAAGGCCATCACCCGTTCAGAAAGCTATCTGTGTGCCATGAAAGCCGGTGCCTGCCGGTATGACACGGAAGGGTATGTGACGGAGCATATTTCTCAGGAGGAGGA
AGCGTATGCGGCAGCGCGTCTGGATAAAATCCGTCGCCAGAACCGGATAAAGGCAGAACTTCAGGCCGTGCTTGATGAGAAATAA
>DLDPCG_00305 Antisense to pHK01_099
CCGACCGGATATTACAAAAGCAAATCTCATGCATTATTCAGCCTTCTGAATCTCTGGATTAATGAAAGCTGCTTTTTTCGTTACACCCTTCCCCGGCAGCGCCGGTGGCAGTGCGCAGCCCTTT
TTCGGGCAAACTGCTGCCAGTCTGTCCGGGGCGCAACACCGGCAGCCCGTTGCCATGCTT
>DLDPCG_00310 Uncharacterized endonuclease
ATGAGGAAATATATTCCACTGGTATTATTTATCTTTTCATGGCCGGTATTGTCTGCAGATATTCATGGACGGGTTGTTCGTGTTCTGGACGGTGATACCATCGAGGTCATGGACTCACGGAAGG
CCGTGCGGATCAGGCTGGTAAATATTGATGCACCGGAAAAGAAACAGGATTATGGTCGGTGGTCGACAGACATGATGAAATCACTGGTTGCAGGAAAAACGGTTACCGTCACATATTTTCA
GAGAGATCGTTATGGTCGTATTCTCGGGCAGGTTTATGCACCAGACGGAATGAATATTAATCAGTTTATGGTCCGGGCCGGGGCGGCCTGGGTATATGAGCAGTACAACACAGATCCCGTGC
TGCCTGTCCTTCAGAACGAAGCCAGACAACAAAAACGTGGGCTCTGGTCAGATGCTGATCCGGTTCCCCCCTGGATATGGAGACACCGTAAATAA
>DLDPCG_00315 Gene expression modulator
ATGGCGAAAACAAAGCAGGAATGGCTGTATCAGTTACGGCGATGTTCATCAGTCAATACGCTGGAAAAAATTATTCATAAAAACAGAGACAGTTTATCGAACAGTGAACGTGAATCATTTAA
TTCAGCAGCAGACCACCGGTTAGCAGAATTAATCACCGGAAAGTTATATGACAGAATACCAAAAGAGATATGGAAATATGTAAGGTAA
>DLDPCG_00320 DUF2726 domain-containing protein
ATGAAGTTGATTATTTTTATTCTCATTGTTCTGATTATTGCAGCGTTACTTATCCGGATTATCCTCAGAAGTGTAAATCAGTATTCTCCTTTGCTGATGCAATTGCATGCGGCAGGTATACGGACC
GGTGATGCAGAGCGAATACTGTCCAGCGGTGAATACTGGCAACGTCAGAAGACGCTGCTGACAGAAAGGGAAGTCAGTTTTTTGACTGCTCCCCGGCCTTCAGGCCGGGGTTTCCCGGA
GGCATTCTGA
>DLDPCG_00325 hypothetical protein
ATGAAAGGACTGTTCAGAATTGTGGATATGAAGCGGTGGTATCTGTGTCCGCAGGTACGGGGCGCGGATATCGTCCAGCTGAACGGGAATATCCGGCCACGATCACGCCAGTGGTGGCAG
TTATTCAGGATGGTGTCTCAGTGGCATGTTGATGTGGTTATCGTTGAGCGGTGTTCGTTCAGTATTGTTGCCGCAGTAGAGCTGGATGATGCCAGCCATTTACGACCGGAACGCAGACGCCG
GGATATTCTTCTGGAAGAGGTTCTGAGGCAGGCTGGTATTCCGTTGCTCAGAAGCCACGATGCCAGAAAACTGCTGCAGATGACCGGAGAATGGCTGAATACAACAGGGGCTGATCAGCA
GTCACCGGAACATCGTAGCTGA
>DLDPCG_00330 hypothetical protein
ATGTCGCAGACAGAAAATGCAGTGACTTCCTCATTGAGTCAAAAGCGGTTTGTGCGCAGAGGTAAGCCTATGACTGACTCTGAGAAACAAATGGCCGCTGTTGCAAGAAAACGTCTTACAC
ACAAAGAGATAAAAGTTTTTGTCAAAAATCCTCTGAAAGATCTCATGGTTGAGTACTGCGAGAGAGAGGGGATAACACAGGCCCAGTTCGTTGAGAAAATCATCAAAGATGAACTGCAGA
GACTGGATATACTAAAGTAA
>DLDPCG_00335 CopA-like RNA
ATAGCTGAATTGTTGGCTATACGGTTTAAGTGGGCCCCGGTAATCTTTCTGACTCGCCAAAGTTAAAGAAGATTATCGGGGTTTTTGCTT
>DLDPCG_00340 Positive regulator of RepFIC repA1 expression
ATGCCCGGAAAAGTTCAAGACTTCTTTCTGTGCTCACTCCTTCTGCGCATTGTAAGTGCAGGATGGTGTGACTGA
>DLDPCG_00345 Replication initiation protein
GTGACTGATCTTCAACAGACGTATTACCGCCAGGTAAAGAACCCGAATCCGGTGTTTACACCCCGTGAAGGTGCCGGAACGCTGAAGTTCTGCGAAAAACTGATGGAAAAGGCGGTGGGC
TTCACCTCCCGTTTTGATTTCGCCATTCATGTGGCGCATGCCCGTTCGCGTGGTCTGCGCCGGCGCATGCCACCGGTGCTGCGTCGACGGGCTATTGATGCGCTCCTGCAGGGGCTGTGTTT
CCACTATGACCCGCTGGCCAACCGCGTCCAGTGCTCCATCACCACACTGGCCATTGAGTGCGGACTGGCGACGGAGTCTGCTGCCGGAAAACTCTCCATCACCCGTGCCACCCGGGCCCTG
ACGTTCCTGTCAGAGCTGGGACTGATTACCTACCAGACGGAATATGACCCGCTTATTGGCTGCAACATTCCGACCGATATCACGTTCACACTGGCGCTGTTTGCCGCTCTCGATGTGTCTGAG
GTGGCGGTGGCTTCCGCACGCCGCAGCCGTGTGGAATGGGAAAACAGACAGCGCAAAAAGCAGGGACTGGATGCACTGGGTATGGATGAACTGATAGCAAAAGCCTGGCGTTTTGTGC
GTGAGCGTTTCCGCAGTTACCAGACAGAGCTTAAGTCCCGTGGAATAAAACGTGCCCGTGCGCGTCGTGATGCGAACAGGGAACGCCAGGATATCGTCACCCTGGTGAAACGGCAGCTGA
CGCGTGAAATCTCGGAAGGGCGCTTCACTGCTAATCGTGAGGCGGTAAAACGCGAAGTGGAGCGTCGTGTGAAGGAGCGCATGATTCTGTCACGTAACCGTAATTACAGCCGGCTGGCCA
CAGCTTCCCCCTGA
>DLDPCG_00350 YacA
ATGTCACAGGTTAACATGAGTTTAAGAATTGATGCCGAACTGAAAGACGCCTTTATGGCTGCAGCAAAAAGTATGGACCGTAACGGGTCCCAGTTAATCCGGGATTTCATGCGACAGACTGT
TGAAGGCCAGCATAATGCCTGGTTCCGTGAACAGGTTAATGCCGGGCGCCAGCAACTGGAACGCGGTGAAGTGCTTCCACACGACATGGTGGAAGCCTCCGCTGCCGCATGGCGGAATG
AGATGATGAAAAAGGCTGCCGGTAAATGA
>DLDPCG_00355 hypothetical protein
ATGATGGAAATTTTCTGGACCATGCTGGCCAGTCAGGACAGAAAGCGTATTCGCGAGTACATTGCAAAACAAAATCTGATGGCTGCTGTTGCACTGAACGAACGGATTAGTTAA
>DLDPCG_00360 UPF0216 protein
ATGGAAAATATGCTGAAAACTGTGCGTGAGCATTTCCCCACGCGAACAGTGGTGATCGAACAGTCTCTGGATATGTTTGGCATCAAACGAAATGAGGACGGGACGTTACTCTTTCCCCGCCA
CTCCGATAAACGTTTTCCCATTGCACGTATCCGTAAGCGACTGGCGGCTTATGGCTGGCGAGGGGAACGCCTGAATAAGGAGGTGGAGCGAATCGTCGCGGGCCCTGATATCCACCCTGAG
AGGAGGGACTACAGCATTATTTTCCCTTACGGATGGGAGAAGAAGCAGCTGACGCGTGAGATGCGGCGAATGTTTCCATGTAAGACTGGCCGACGTGTGCTGATTATGCTCGATGAAATAC
ATCAGTTTATTGACTCACGGGAGAAGCAGTGA
>DLDPCG_00365 DUF2913 domain-containing protein
GTGACGCTGACAGAAAAATCAGGCCATCTGGCATGGTGCGCCCTGGTGGCGCTTGCACTGGCCAGGCAGAACGGCGATGTGCTGTCACCGGCACAGGAAAATCTCTTTCTTACCCGATGG
CTGGCGACCGCGCTGAAGCAACGGCGTTTTTCGCGTGATGTTGCCTCCGATATCGAGTGGCTTCTGAAACAGGGACGCCAGCTGGGCGTCAGCGCGAAGCTGGCCAGCAAGCTGAATTAC
CTCTGGCGTTCCTGCACAGGTGAGTTGTCCGAGCAGAACGACCTGTTCCGACTGACGTATGCTCTGGAAACAGCGAAGGATATGCACTGGAACTACCGTCTGCTGAGCGCTCGGGAATGG
TCCGGCCGGAATGCTGTGGCGCTCAATGCGGGCGTGAACGGCATTTATCTCTCACGGGCCAACCTCGATGTCGCTTTTGACGACAGCGGCCGACAGATAAATCCGCTTACAGCACGTCTGA
CGGGTAACGTAGCAGGGGTGATGAAGGTGTTTAATCGCTGTGGCTGGCAGGCAGAGCCTGACTCTGGTATCTCCCTGCCCCACCAGTATTCGCTGATGGCCGGACAGGGAGTGCCAGGAA
AGGGGGATTAG
>DLDPCG_00370 ISNCY family ISSen7 transposase
ATGAAGAAGAAAAACAGCACACCTACTCCTCATGATGCCACGTTCAGGCAGTTCCTGACGCAACCGGATATTGCCCGGGATTTCATGGAAATCCACCTGCCGGCAGAGCTGCGCGCAGTCT
GCGATCTCAGCACGCTGAAACTGGAATCAGGCTCGTTTGTTGAGGATGATCTCCGCCAGTATTTCAGCGACGTCCTCTACAGCCTGAAAACTACAGCCGGCGACGGATATATTCATGTTCTG
GTCGAACACCAGTCAACGCCGGACAAACATATGGCTTTCCGCCTCATACGCTATGCGGTAGCCGCCATGCAGCGCCATCTGGAGGCAGGGCATAAAAAACTGCCACTGGTGATACCGGTGC
TGTTCTATACGGGTAAGCGCAGCCCGTATCCGTACTCTACCCGTTGGCTGGATGAATTTGACGATCCCGAGCTGGCTGGCAAACTCTACAGCGGCGCTTTCCCGCTGGTAGACGTTACGGTT

ATTCCGGATGATGAAATCGCTGGCCATCGCAGCATGGCCGCCCTGACTTTACTGCAAAAGCATATTCACCAGCGGGACCTGGCAGAACTGGTTGACCGGCTGGCGCCCATCTTGCTGGCGG
GATACTTGTCTTCATCGCAGGTGATATCGCTGGTACACTATATAGTACAGGCAGGCGAAACATCCGACGCCGAAGCCTTTGTACGCGAACTGGCACAGCGTGTGCCGCAACACGGAGACGC
ACTTATGACCATCGCACAGCAGCTTGAACAAAAGGGCATCGAGAAGGGGATTCAGCTCGGTGAACAACGTGGTATTGAAAAAGGCCGTTCAGAAGGGGAGCGCGAAGCGACTCTGAAA
ATAGCCCGTACCATGCTCCAGAACGGCATTGACCGCAATACCGTCATGAAAATGACCGGTCTGACTGAAGACGACCTGGCGCAGATCCGCCACTAA
>DLDPCG_00375 Magnesium transporter
ATGATGACAAATAAATCAGGGACCAACGGGCATTACGGTTATCTTTTCGAAGAGCCAGTAGATGAAATATCTGGTGATATCCTTATCGCTTTAGAAAATCTACCGCCAGAGATAAAGCAGACT
ATTCTCTTGCTCGTTAAAAATGCAGCTGACCGTGATGCGGCGCAGAAGGCGCTGTCCGACATAATGAAGCAAATTGAGTTTGATATAACTGCAGCTGAACTGGCAGCATATCTTTTTAAACCA
GGTCTCGCTTAA
>DLDPCG_00380 Resolvase/invertase-type recombinase catalytic domain-containing protein
ATGTACATTTATGCATATCTTCGAGCTTCAACAATGGAACAGGACGCGCTTCGTTCAAAAAACAGGCTAAAAGAATTTGCAGCACTTCATGGCCATCGAATTGCTGGCTGGTATGTGGAAAAT
GCTTCCGGTGCATCTCTGAACCGTCCTGAGTTAACCCGCATGCTTTCAGACATGGAGCCAGGGGATGTCATTTTGCTCGAACAGGTGGACCGGCTTTCCCGCCTGAATGATGCTGGCTGGC
AGATTCTCCGGAAAGAAATAACAGATAAACGTTTGTCTGTTGTCAGTCTTGATCTCCCTACCAGCCATATGGCTCTGACCAACGCTGTTAATGATGACTTCACCAGAAGTATGTTGAAGGCCG
TTAATGGCATGATGCTGGATATGCTCGCCGCGATTGCCCGAAAAGATTACGAAGACCGTCGACGTCGTCAGTCAGAGGGGATCATTAAGGCGAAAGCTGAAGGAAAATACCGGGGCAGGG
TAGCCGATGCGCAGAAACATGAACTGATTCGCACTCTGCGCCTGGTCAATGGAAAATCGCTGCGAGAAACAGCTCGCCTGGCTGGCGTTTCTAAGATGACAGTCATCAGGGTCTGCAATGT
TCAGAAGGTGAATCAGGATAACTATAGAGGACATAATGATGACAAATAA
>DLDPCG_00385 Partitioning protein
ATGAAACGAGATTACGGTAGTGTCGGTACCATAGCGCTCAGAGCAAGTGCTCTACTTCAGGCCATGAGTCGGGATATTGAGGAGCAAAGAAAAGAATTCAATCTGACAGAGTATCATCAAA
CATATACTCGTAATGCGGTCGCAAAATTGCCGAAGCTGAGCCGACGCATCGTAGAACTGGCCGTTAAAGAAATGGAAGAAAGCGGCTATGAATTTAATAAAAAGCAGGTAGGCAACGTCGA
GCAATATGCATTAACAATTCAGAACGTTATTGATATTTACGCTCACCGACAGATACCAAAATATCGCGATGTTCATAAAGAACCTTACGTAATTTTTGTCGTCAACTTGAAAGGTGGGGTTTCA
AAAACCGTCAGCACAGTAACGCTGGCGCATGCACTACGAGTACATCAGGATTTGCTGCGTCATGACCTTCGTATCCTTGTGATCGATCTTGACCCGCAGGCGTCCAGCACTATGTTTTTGGAC
CATACCCATAGTATTGGTACTGTCCTTGAAACTGCCGCCCAGGCAATGCTAAATAATCTCGATACTGAAACACTGCGAGAAGCGGTTATCCGACCCACTATTATTCCTGGCGTTGATGTCATCCC
TGCTTCTATTGATGACGGCTTCGTAGCAAGCCAATGGGAGTCTTTGGTTGCTGAACACCTTCCTGGTTTTAAGCCTTCTGAAGTACTCAGGAAAACCATCATTGAGCGTATCGCTGATGATTAT
GATTTTGTCTTTATTGATACTGGCCCTCATCTTGATCCATTTCTTCTCAATGGCCTGGCTGCGAGTGATTTACTACTCACCCCTACTCCCCCGGCGCAAGTTGATTTCCATTCAACTCTCAAATAT
CTGACTCGTTTACCTGAAATGCTAGAACGTTTGGAAGAAGAAGGCGTTGAGCCTCGCCTGAGTGCGAGCATAGGGTTTATGTCCAAAATGACCAGTAAGCGCGATCACGAAACTTCCCATA
GTCTGGCTCGTGAAGTGTATGCAAGTAATATTCTTGACTCATCGCTTCCGAGATTGGACGGGTTTGAACGATGTGGTGAATCTTTTGACACTATCATCAGTGCTAACCCTGTTTCGTATCCAGG
AAGTGCAGAGGCATTGAAAAAAGCTCGTACCGAAGCGGAACGGTTTACTAAAGCCGTTTTTGACCGCATTGAATACATTCGGGGAGCATCCAAATGA
>DLDPCG_00390 Partitioning protein B
ATGAAAAAGGTTTTAGCTCGAGGGCGAGTTCTCGGCAATAGCAATTCAGAGTTCGCTCGCATGCTTGAAGGTGACGGAAAGATCAAAACGTTTACTCTTAAGTCTGGGAAACAAGCAAAA
TTCATTAAAACGGTTGTATTAAGCGGAGATATAGAATCTAAAACGTTTGTTGATGCATCTGTTAATGGACGCGATCAGACTATGCTTTCGCGTGAGTCAGTCAGTGACATAACTCGTACGATTA
AACTTCAGCAATTTTTTCCTGCTATTGGTCGGGAAGTAGATGGACGAATTGAAGTTTTAGACGGTACGCGTCGTCGCGCTGCCTGTATCTATAACAACGTAAAATTCGAAATACTGGTGACAA
AAGACAGTATTGATATTGCCGACGCGAGACAACTTGCAAAAGATATACAGACAGCCAGAGAACATAGCCTTCGAGAACTAGGAAAACGCCTTGAAGTTACTTATGGGACAAGTATGACAAA
AGAAGAGATCGCTCAGGCTGAAAGCATCTCTCCGGCAAAAGTAACACGCGCATTTCAAGCGGCATCAGTACCGGATGAAATGATCTCTGTATTTCCGGTAATAAATGATATTTCGCTGTCGGA
CTATCAATTTTTGTTGAAGCTGTCTGAAGAGGCAACCAACAAAAAAATGTCAGTGGAAGAGTTTATGGATAGAGTTCGCCAAAAACTGATGGCTTTACCTGACTATCCAACAATTGAGAAAA
ACAAAATCTTGGCGGTCTTCAGGTCTGAGGGAAAATTGCTTACAGACCGGCCTGTAAAAACAGTTGAAACTGAGAAGTTGAAAGAGTTTTCTGATCGTAACCAATTTGCCAGAAAGAAGA
CTGACCCCAGAAAGCGGCTTGTTGTTTATGAGTTCTCCCGTATATCTAATGAGGTGCAGTTAGAAATCGATGAGGCTATAAAAAACATTCTAAGAAAACTGCCTGAATAG
>DLDPCG_00395 hypothetical protein
ATGCAAGTACTGGATGGTATTAACCATTCCGGTCTGGGGAAAGTGTGGTTTGCAGGCCGAGGCATTGCGCCAGAATGGCAGATGAAGCGTGAGATGTTGTCACCGGCATACACTACTCGCT
GGAACGAACTACCGGTCGCCAGACTTTGA
>DLDPCG_00400 DNA polymerase V subunit UmuC
ATGTACGCGCTTGCTGACGTGAATTCGTTCTATGCCAGCTGCGAAAAGGTTTTTCGCCCTGACCTGCGAAACAAACCTGTCGTCGTTCTCTCAAATAACGATGGCTGCGTCATTGCGAGAAG
TCCTGAAGCGAAGCGGCTGGGCATCAAAATGGGGGTACCGTGGTTTCAGCTGAGGTCGGTGAAATTTCCAGAGCCGGTTATCACGTTCTCCAGTAATTACGCCCTCTACGCATCAATGTCGA
ACAGGGTCATGGTTCACCTGGAAGAGCTGGCGCCGCGCGTGGAGCAGTACAGCATCGACGAGATGTTTCTGGATATTCTCGGTATAGACAGCTGCATCGATTTTGAGGATTTCGGCCGGCA
GCTGCGTGAGCACGTTCGTTCCGGCACAGGGCTGACCATCGGCGTCGGAATGGGGCCGACCAAAACGCTGGCTAAAAGCGCACAATGGGCGTCGAAAGAGTGGCCACAGTTTGGTGGC
GTGCTGGCGTTAACGCCAGGCAATATCCGCCGTACGGAAAAACTGCTGTCACTGCAGCCGGTGGAGGAAATCTGGGGGGTTGGCCGCAGGATCTCAAAGAAGCTGAACACAATGGGGAT
AACAACTGCATTGCAGCTGGCGCGCGCGAACCCTGTATTTATCAGGAAAAATTTCAATGTAGTTCTGGAGAGAACGGTCCGGGAACTCAATGGAGAAAGCTGTATTTCACTGGAGGAAGC
ACCGCCCCCCAAACAGCAGATAGTCTGCAGCAGGAGCTTTGGGGAGCGTGTCACGACGTATGAAGCCATGCGACAGGCAGTCTGTCAGTACGCTGAGCGTGCAGCTGAAAAGCTGCGCG
GCGAGCGTCAGTTCTGCAGGCATATTGCCGTGTTTGTGAAAACATCGCCGTTCGCCGTAAATGAACCCTACTACGGTAATCTGGCCAGTGAAAAACTACTCATCCCCACGCAGGACACACGG
GACATCATTGCCGCTGCCGTCAGAAGCTCTGGACAGGGTCTGGGTGGATGGTCACCGTTACGCAAAGGCAGGCTGTATGCTGAACGATTTCACCCCAACCGGAGTTTCTCAATTGAACCAG
TTTGA
>DLDPCG_00405 Protein ImpA
ATGTTACTTTTTGTCGCCCCGGAACAGGAGCCGGTGCAGTCCACTGCCCCGCTGTTCACCGAACGTTGCCCGGCGGGTTTTCCGTCGCCGGCCGCCGATTACACGGAGGAGGAACTGGAC
CTGAATGCCTGGTGCATCCGGCGTCCGGCTGCAACGTTCTTCGTCAGGGCTATCGGGGATTCGATGAAAGAGATGGGCCTGCATTCCGGCGACCTGATGGTGGTCGACAAAGCAGAAAAG
CCGCTGCAGGGTGACATTGTCATCGCGGAGACTGATGGCGAATTCACGGTTAAACGCCTGCAGCTGACACCCCGCATCGCCCTGCTGCCGATGAACCCGGCCTATCCCACTCTCTATCCGGA
AGACCTGCAGATTTTTGGGGTGGTGACAGCCTTCATACACAAAACACGGGGCATGGACTGA
>DLDPCG_00410 IS110 family ISShdy1 transposase
ATGACTGTTGTTACTGTCGGTATTGATTTAGCTAAAAATGTGTTCGCTGTGCATGGCGTTAATGCTGCAGGAAAAGCTGTTTTAGTTAAGCCTCTTGTGCGTCGGGCTAAATTGCTTGAATTGA
TTGCAACACTTCCAGCATGCCTGATTGGAATTGAGGCATGTACAGGTGCACATTACTGGGCAAGAGAGTTCCAGAAAATGGGGCATACAGTTCGAATTATGGCACCGCAACTGGTTTCCCC
ATACCGTATGAGTGGCCGTCATGGGAAAAATGATGCAGCAGATGCCGCTGCCATTTGTGAAGCTGTTGCCCGTCCTAATATGCGGTTTGTTCCCATAAAATCAGTTGAGCAGCAAGGGCAAT
TATTTGTACATAGAGCTCATCAGGGATATGTCAGCGAGAGAACGGCATTGATTAATCGCATCCGGGGATTACTCAGTGAGTTAGGTATTGTTCTCCCACAGGCGTTTAAAGCTTTTTTATCCGG
AGTGGCTCAGGTTCTTGAGGATTTGCCCGGTTACTGTAATCAGGTTATTGGTGATCTTCTTAATGAGCTTTATCACATAGAAGAAAAGATTAAAAACAGTGAAAAACTCATTCAGCAACTTGC
CAGAGATAACTCAGCAGCACAACTACTAATGCAATTTAGGGGGATAGGCCCGATGACTGCTACAGCAATCGTTGCCAGCATAGGTAATGGTCATGATTTCAAGAACGGGCGTCAGTTTAGTG
CTTGGCTGGGGTTAACACCAAGACAACAAAGCTCCGGGGGTAAAGCACGGTTGGGGAGAATAACGAAAGCCGGCGACAGCTATCTCAGAACACTTCTTGTTTTGGGGGCGAGATCTGTA
CTGTTGGGAGCAGCTAAAAATGATGATCCGGTTAGTCGATGGGCCCTGCAATTAAAAGAAAGGCGAGGATATGGACGAGCGCTTGTTGCGATAGCAGCAAAAAATGCACGTATGTGCTGG
GCAATGTTGCACCTGAGAGATGAATTCAGATTTCCTGCCTAG
>DLDPCG_00415 hypothetical protein
ATGCCGAACTGGTGTGCCAACCGACTGATGTTCAGTGGTATTCAAAACAGTGATGCCCTCAAAACGTGGATTGCCGGAGGCGGGCTGTCCCTGCATCGCCGCGCCAGAAAAGAAGGTATC
CAGCTTTTCCTGGCGGGCTGCGCGGGGATACTGCGCCCCCCGACAGAGCAGCATTACTCACCATACCCGCAGCTGGTTTCTTACGGAGCGGCCGCAGACAACCGGCCTTCAGCGCAGGCA
TACAGCGACTGGCTGGCGATGTTTATGGGCTGA
>DLDPCG_00420 DUF1281 domain-containing protein

GTGGTCATCCGGCAGCTGTACCAGCAAAAATCCTGTGACTGGGGGGACAGTTTCCGGCCTGCGCCGGTGGATGAATGGTGGGACAGCCTGTGCGACGCTGGTTCTGCCGTACCTGCTGCT
GATCCAATGGATTTCCGCGACGTGCTGCCGACGCGTCTGGATATTGAGGTGAACGGCTTCAACGGTGGCCTGCTGACGGGTATCCCGTCGTCATATGACCACTATCTGACCCGGTACGGGTG
TAAGTGGCCGGGAGGGTATGAGGCAAATATCTGCTTTGCCGGAGAGAACACGCTGACAGTGGATTTTGACACGCCCTGGAGTCCGGTCGGCGAAGACGTTGTGGCTGAACTCAGTCAGC
GGTATGGCGGGGAGGTGGAGCACTGGTTTGCGGAGCAGGGCTGCAATTACTGCGGCTATGCCCGGTACGTCAACGGCGAAACGGACGTGTACATCACGGACGAGCTGGAGTGGGGCGA
GGCTGACCCGGACGATGAGGACAGTTTCCCGGACGTAACCGACCCGGAGTGGATTATCAACAACGTGACGCATTTTGGCGGCTGA
>DLDPCG_00425 Cytoplasmic protein
ATGGAATTAAATCTGAATACCTGGCTTGTTGGCCTGATTGTCGATGTCGGTGCCACTGAAATGATGGTGTATTACCTGATTAGCGCGGCTGACCTGGAGCATGCCGAAGCGGGCGTCATGGA
AATGGGGCGCACCTGGTGGCCAACGCTGCAGCGCGAGGATGACCGGCATCGGTGGGAATATGCCGCAGGCGTGGTCTGGTTCAACAGCATCATTTTGCTCGATGACGTGGAAAACAGCAT
CCTGCGCGGGCTGAAGTTCCTTGATGCGTGGACCGTGACCGGCTCAACGGATACGCCGGTACTGCGAGACGAGTGGGACAATGACTGGCGGGATATCACCCGCTGA
>DLDPCG_00430 hypothetical protein
ATGACGCATGAGCATGAGCATGAGTTGAGAGTTCAACAACTTCTGGACGCTGCAAACGGGAAAAAGCTGCAGCGCCACAGGGCTGTGCCCCTCCTCACCACAGCCCAAAAATTAAAGAAT
AAGAAACGTGAAGAAAAGACAAAACAGTTCATCCTGATAATGGTGTTACTGGTTCTTTTATTTGCCACAGCTTATCATGAAGGCGTGATTGACAGCTTTACAGCGTTATTTTCTGCGGGTTGA
>DLDPCG_00435 Putative methylase YubD
ATGTCCCGTTTTATCCTGGGTAACTGCGTCGATATTATGTCCACCTTTCCGGATCGCGCTGTTGACTTCATCCTGACTGATCCCCCGTATCTGGTCGGGTTTCGTGACCGTTCCGGCCGCAGTAT
TGCCGGTGACAAAACCGACGAATGGCTGCAACCCGCCTGCAATGAAATGTACCGCGTCCTCAAAAAAGACGCACTGATGGTGAGCTTCTACGGCTGGAACCGTGTCGATCGTTTCATGGCC
GCGTGGAAATGTGCCGGGTTCAGCGTGGTCGGGCATCTGGTTTTCACCAAAAACTACTCGTCGAAATCGGCCTATGTCGGCTACCGCCATGAATGCGCCTATATCCTGGCGAAAGGCCGTCC
GGCCCTGCCGCTGGACCCGTTACCGGACGTGCTGGGCTGGAAATACAGCGGCAACCGCCACCACCCGACCGAAAAACCTGTTACCAGCCTGCAACCGTTGATCGAGAGCTTCACACACCC
GAACGCCATTGTTCTCGATCCGTTTGCCGGTTCCGGCTCCACCTGTGTCGCCGCCGTCCAGGCAGGGCGCCGTTATATCGGCATTGAACTGATGGAGCAGTACCACAAAGCCGGGATTACCC
GCCTGTCAGCCGTGCAGCGCGCCATGCAGCGCCCGGCCGCTAACGACGTCTTTTATCCGGAGGCCGCATGA
>DLDPCG_00440 DUF1380 domain-containing protein
ATGTACGGAACCTGTGAAACCCTCTGCCGGTTGTTGAGTGAGCAGTATCCCGCAGAAACCCCGCTGAACCTGATTGTCTGGTCCCCGGCGGATATCGAAGCGCTGGCCGACGGAATGGAAT
ATGCCGTTTCTGAACAGGACATAAAGGACGTTCTGGCGCGTCTGGACGCCATACCGGAAGAAGAACGGCTTGAGTCGGGCGTGTCTGCCAGTGCAGTCATGGATCTGATTGATCAGGTGA
AACAGGCCGTTCCGGCGGTAAGTGTACCGGCTGACCTGCTTGAAACCCTGCTGATGACCGCCGAACAGGCGTTATGGCACAGGGAATGGACCGCCCGCGACGGCAATTATCCGGTGCCGG
AAAGCGTTACCCGCCGCCTGGCTGATACGGCGAAAGTCCGCGCATTACTGAAAAACTGA
>DLDPCG_00445 DUF1380 domain-containing protein
ATGATGTATGGAACCCGTAAAGAACTGAATAAAAAGCTGAAACGTATGTTTGATAACGATGAACACTTTGCCCTGCTGGTCTGGACGAAGCAGGACGTGATGGCCCAGGTGGAAAACATGA
CGGAAAGCGAGGCCAGTGCCATTTTGCAGGAAATCGGCAGCGTAACCGGGCATACCGAAGAGGGTATTTCATTTCGCACGGTCCGGGAGATGTATGCCGGATTACGGGCCGAAATCCCGA
CTGTCATCGTTCCCGCCGATCTGCTGGCCCGTCTGACTGACGTAGCGGGGCTTGCGCTGGACACAGAAGACGCCCGGGCCTGGCCGCTTGTTTGCCAGCACTACCCGTCCGTGGCTGATGC
CCAGGCCGATATCACGTGGCTCAGGCAGCTCCTGGCCGCCTGA
>DLDPCG_00450 Inner membrane protein
ATGAATAACGATACCCTGAACGCCCTCATTCTGCGCCACGGTGACAATCTGTTGCGCCGGTCCGGCTGGCCCGACTCAGTTGGTATGACGCCCGTCGCGCCCGAAACCGTACCCGGCTGGCT
GTTCGCCTGTGGCGTGCTCGATGCAGACGAGATCCTGGCGCTGACAACCCGGCTGTGCCAGTCCCTGAATGACGGACGGGCGAAACTGCTGACCGCCAGCGCGCAACGGCTGGCAGGG
ACACCCGCCCGGCTGCATCTGTACCCGGCGCAGTCTTATCCCCGGCCTGAAGCCCTGCCTGACTGTACGTGTATCAGTCTGCCCTATGCCCGGGAATGGCTGACAAAAGACGAATGCGACGA
CCTGCTGGCCTTCCTGAAAGATTTCATTGACCGGGTGTGTGAAATCGTTCGCCAGGACGCGCAGCGCATCGCTGCGGCTCTGGTGCCGTCTGCCACGCCGCGGCTGATGGAAAAACGTTTC
GGAGACTGGCGACTGGTTGCGGACGAATACGGGCATGACAACTGGCTGGACAGTGAGGACGGGGAACGGCTGGATCAGGTGCTGGACGGCATACTGGTCCGGGATGCGCGGTTTTGTC
CGGTCCTGCTCACCCTGGTGAATGAGTCCCGGGAAGAAATCGAAGCGGCCGGGGTGATGACCGATCTCCTGCGTTTCCCGGGGGAACCGGTCCGCCGCTGGTTTGACCGCCGCGTGTTGC
GGGATGTCATGAATGAGGTGCGCAACACTGGCTCCACAGGGGATTAA
>DLDPCG_00455 Antirestriction protein
ATGCAGTTAGCTACATGTCAGCACACCCTTAACGTCGAAGAATCCGACCGCCTGTCTTTCCTGCCGTACATGTTCGGCAACGATTTTATGCTGGCTGAATTACAGGTATATACCCTGGCGAAAA
AATGGATGCCTGAATACAACGGTGGTTTCTGGCATTTCATCCGTCTGCCGGAAGGCGGCGGATACATGATGCCCGACTGCGATCGGGTACACATGATCAACGGTGAAAACTGGTTTGATCGT
ACCGTCAGCGCCGACGCTGCCGGAATTATTCTGACTTCGCTCGCCATTAACCGCCGTTGCTGGACGCATCACGACTGCGGCAACGCTGCGCTCACTCGCCTGTATATGCTGCGTGATACCCA
GCTCTGGAATCACATTACGTTTCACCCTGAATGCAACGCCATTTACGCCGCGCTCGACTGA
>DLDPCG_00460 DUF1380 domain-containing protein
ATGTACGGAACCGTCAGCACCATCTGCTGCGACTTACTGAAAGCGTATGAGAATCACGAAAAAATCGCTGTCATCATCTGGTGTGAGGGCGATGTGCGGGAAATGGGGGCGGAGTTTAATC
CGACCGCTGATGACATACAGGCCGTACTGCGCGCCATTGGCGAGTCGGACAGTGATGCGCTCTGGCGTGATGGTATTGGCCAGAATTTTGTTGAGGGCGTATTACGCGATTTAGTCGCACT
GCGCCCACCGCGCCAGATTGCTGTACCTGAAGATGTGCTGCACACGCTTTTACCCCTGATGGAAGCCGGAAAGAACCTTCCGCGTTACCAGACGCAGGACAGGGCTGCCGAAGAAGAAA
CCGCGCTGGATACGTTGCGATACCTGCTTTCCACAAATATTCCTCCCCATAACCAGACCGAGGGGAATTACTGA
>DLDPCG_00465 hypothetical protein
ATGGCAGGCGGGGGAGGCCGCCTGTCAGCCCGCGCGAAAAACCCTGCAGGCTGGCGCCTGCGGGGGAAGGACACGCGCCGCGCTTCGCGCCGCTCACTGTCCTTTTCCCCATCGGTCTC
TGATGGCGTACACTTACAGCCGGTTTCCATTTCTGTCGGAGGATGTCATGTCCGGACTGAATCTCGCGGTTACAACTAA
>DLDPCG_00470 hypothetical protein
ATGTCCGGACTGAATCTCGCGGTTACAACTAAAGAAGAGCAGGACAGGGTGCCATTGATCTGGTCGCTTCAGGTGTGGCATATAAAGAAAGGCTGGCCATGCCGGTGGTCGCTGAACTGG
TGGCCAGGGAGCAGCCTGAACATTTGA
>DLDPCG_00475 hypothetical protein
ATGCCGGTGGTCGCTGAACTGGTGGCCAGGGAGCAGCCTGAACATTTGAGAGAGTATTTTTGGGTGCGGCTGGAGCATTATCGCCTGGTCAGTGCGCAGTTCCCTGACAAAAATGCCCCG
GAATATGCCAGAAAAGAGGATGAGGTTAAAAAATAG
>DLDPCG_00480 hypothetical protein
GTGAAAAGGCACCAGAAGTGGATGAGAGCAAGGGCCCGCCGCAACGTCGGGACGCGGCACGCGAAACGGCGTTGCGCCTTACAGCCCGGCAGCCTTACCGCCGGGGTGCGAAAAGCG
CAGGAAACGGCGCGCCAGGGGCGGCGTGACCGTAACGGTCGCAGCAAGCCTGCCGTTGACGTTGCGACAGAGGGATGGAAACCCGCTGCCCCGAAGGGGCCGGGCAGGGACAGCGC
AGCTGGCCCGGTGCGCAGCATGACAGCCCCGGTTCCCGCCAGGGAAGTCGCCCGGCGTCAGCGAAACCCGCCAGGGAATACCAGCAACTGA
>DLDPCG_00485 VWA domain-containing protein
ATGCAGGCTCAGGCGATGCGTGTTTATCAGATTGCGTTCAGTGGACGCGATGCAAAGGGCGTTTTACCGATGTTTACCCGCGTAAAAGCTATGACCGGTAAAAAGGCGGTGAGAGCGTTTG
TTGAACGATATAAACCCGTCAGCGGCTGGTTTTTAGGCGACCCCGAAGACATTACGGACAAAGTACAGAAGGAGGCAGAAGACACGGGCAGCAACCCACAGACATGA
