# Supplementary material for: Pangenome dynamics and population structure of the zoonotic pathogen Salmonella enterica serotype Hadar
Source: Nat Commun. 2026 Jan 24;17:1270. doi: 10.1038/s41467-025-68026-3 (PMC12868874; doi:10.1038/s41467-025-68026-3)
Supplement: Supplementary file 9 — Reporting Summary [file 41467_2025_68026_MOESM9_ESM.pdf]

nature portfolio

Corresponding author(s): Hattie Webb

Last updated by author(s): Nov 14, 2025

Reporting Summary

Nature Portfolio wishes to improve the reproducibility of the work that we publish. This form provides structure for consistency and transparency in reporting. For further information on Nature Portfolio policies, see our Editorial Policies and the Editorial Policy Checklist.

Statistics

For all statistical analyses, confirm that the following items are present in the figure legend, table legend, main text, or Methods section.

n/a	Confirmed	
☐	☒	The exact sample size (*n*) for each experimental group/condition, given as a discrete number and unit of measurement
☒	☐	A statement on whether measurements were taken from distinct samples or whether the same sample was measured repeatedly
☐	☒	The statistical test(s) used AND whether they are one- or two-sided<br>*Only common tests should be described solely by name; describe more complex techniques in the Methods section.*
☒	☐	A description of all covariates tested
☐	☒	A description of any assumptions or corrections, such as tests of normality and adjustment for multiple comparisons
☒	☐	A full description of the statistical parameters including central tendency (e.g. means) or other basic estimates (e.g. regression coefficient) AND variation (e.g. standard deviation) or associated estimates of uncertainty (e.g. confidence intervals)
☐	☒	For null hypothesis testing, the test statistic (e.g. *F*, *t*, *r*) with confidence intervals, effect sizes, degrees of freedom and *P* value noted<br>*Give P values as exact values whenever suitable.*
☒	☐	For Bayesian analysis, information on the choice of priors and Markov chain Monte Carlo settings
☒	☐	For hierarchical and complex designs, identification of the appropriate level for tests and full reporting of outcomes
☒	☐	Estimates of effect sizes (e.g. Cohen's *d*, Pearson's *r*), indicating how they were calculated

Our web collection on statistics for biologists contains articles on many of the points above.

Software and code

Policy information about availability of computer code

Data collection	All sequencing reads were downloaded from the National Center for Biotechnology Information (NCBI) and EnteroBase public repositories. BioProjects and queries have been explicitly stated in the methods section where relevant. Accession numbers for all genomes are included in Supplemetary Data.
Data analysis	All commercial software, open-source tools, and custom code were previously described in published literature and are cited in the manuscript along with version numbers and parameters where relevant.<br>Assembly: shovill v.1.0.9<br>Serotype: SeqSero 2.0 v1.2.1<br>Allele code: BioNumerics v7.6.3<br>Detection of accessory elements: PanGraph v0.7.3<br>Plasmids: PlasmidFinder (updated 17JUL2019), MOBscan, CONJscan, COPLA, MOB suite v3.1.9<br>Annotation: Bakta v1.9.1<br>Antimicrobial resistance: staramr v0.4.0<br>Jaccard Index calculation: https://github.com/PenilCelis/Salmonella_Typhi_JINA<br>Genome kmerization and network construction: BinDash v1.0<br>Network visualization: Gephi v10 (employing ForceAtlas2 algorithm and Louvain method)<br>Network figures: igraph package in R<br>Contig mapping: BLASTn using BLAST+ v2.15.0<br>Phage analysis: PhageScope 1.2.1, PHASTEST v3.0<br>Pangeome comparison: v3.13

Gene prediction: Prokka v1.14.6
Core gene alignment: snippy v4.6
Maximum likelihood tree generation: IQ-TREE v2.3.3
Statistical analysis: SciPy v1.14.1, Python v3.11.7, R v4.4.0
Geographic map generation: R v4.4.0, packages: ggplot2 (3.5.2), dplyr (1.1.4), tidyr (1.3.1), gridExtra (2.3), scatterpie (0.2.5), RColorBrewer (1.1-3), usmap (0.8.0), sf (1.0-21).

For manuscripts utilizing custom algorithms or software that are central to the research but not yet described in published literature, software must be made available to editors and reviewers. We strongly encourage code deposition in a community repository (e.g. GitHub). See the Nature Portfolio guidelines for submitting code & software for further information.

Data

Policy information about availability of data

All manuscripts must include a data availability statement. This statement should provide the following information, where applicable:

- Accession codes, unique identifiers, or web links for publicly available datasets
- A description of any restrictions on data availability
- For clinical datasets or third party data, please ensure that the statement adheres to our policy

The authors declare that all genomic data supporting the findings of this study are publicly available through NCBI and Enterobase using accession numbers listed within the paper and its supplementary information files available through Figshare. Ssource data are provided with this paper. Epidemiological data is available within supplementary information files. Some human patient information collected as part of routine public health surveillance or through supplementary standardized questionnaires are not publicly available due to data privacy laws; deidentified data are available on request by contacting pulsenet@cdc.gov, per data sharing policies.

Research involving human participants, their data, or biological material

Policy information about studies with human participants or human data. See also policy information about sex, gender (identity/presentation), and sexual orientation and race, ethnicity and racism.

Reporting on sex and gender	Sex was not identified a priori as a risk factor associated with Salmonella Hadar infections and was therefore not included in this study.
Reporting on race, ethnicity, or other socially relevant groupings	These data are not consistently available through routine surveillance programs and therefore was not analyzed as part of this study.
Population characteristics	See above.
Recruitment	Data analyzed in this study were collected as part of routine national surveillance and supplementary interviews for outbreak investigations. Participants were not recruited for the purposes of this study. Patient recall at the time of interview may have been limited or patients may not have been queried about relevant exposures. These limitations are described in depth in a companion publication cited throughout this manuscript.
Ethics oversight	This activity was reviewed by CDC and was conducted consistent with applicable federal law and policy (see e.g., 45 C.F.R. part 46, 21 C.F.R. part 56; 42 U.S.C. §241(d); 5 U.S.C. §552a; 44 U.S.C. §3501 et seq.).

Note that full information on the approval of the study protocol must also be provided in the manuscript.

Field-specific reporting

Please select the one below that is the best fit for your research. If you are not sure, read the appropriate sections before making your selection.

☒ Life sciences ☐ Behavioural & social sciences ☐ Ecological, evolutionary & environmental sciences

For a reference copy of the document with all sections, see nature.com/documents/nr-reporting-summary-flat.pdf

Life sciences study design

All studies must disclose on these points even when the disclosure is negative.

Sample size	No statistical analyses or sub sampling was used in this study. All sequenced Hadar isolates collected between January 1st, 2016, and August 30th, 2023, were included in this analysis. This start date aligns with the introduction of routine sequencing for national Salmonella surveillance. For years prior (2005–2015), all Hadar isolates in PulseNet USA's national database with WGS data available were included (n=55); these represent a small proportion of total isolates collected from this time period that were sequenced for various special interest projects. For non-human sourced samples, we included all sequenced Hadar available with linked metadata from other national surveillance systems.
Data exclusions	Data points were only excluded if they represent duplicated entries or did not meet quality metrics described.
Replication	Genomic analyses were repeated with a global Salmonella Hadar collection, using the same JI threshold and Louvain clustering algorithm. Genomes that fell within named clusters reproducibly fell within the same clusters. Additionally, genomes that were represented by both short and long-read assemblies fell within the same algorithmically-defined clusters. Thus, genetic clusters were found to be robust. However,

network topography, which is for visualization only, will change based on input genomes.

Randomization	Randomization was not relevant to this study.
Blinding	Blinding was not relevant to this study.

Reporting for specific materials, systems and methods

We require information from authors about some types of materials, experimental systems and methods used in many studies. Here, indicate whether each material, system or method listed is relevant to your study. If you are not sure if a list item applies to your research, read the appropriate section before selecting a response.

Materials & experimental systems

n/a	Involved in the study	
☒	☐	Antibodies
☒	☐	Eukaryotic cell lines
☒	☐	Palaeontology and archaeology
☐	☒	Animals and other organisms
☒	☐	Clinical data
☒	☐	Dual use research of concern
☒	☐	Plants

Methods

n/a	Involved in the study	
☒	☐	ChIP-seq
☒	☐	Flow cytometry
☒	☐	MRI-based neuroimaging

Animals and other research organisms

Policy information about studies involving animals; ARRIVE guidelines recommended for reporting animal research, and Sex and Gender in Research

Laboratory animals	Study did not involve laboratory animals.
Wild animals	Sequencing data and metadata for Salmonella from wild animals were previously published. Samples were not collected for the purposes of this study.
Reporting on sex	Sex information was not collected as part of routine surveillance programs.
Field-collected samples	Sequencing data and metadata for Salmonella from non-human sources were collected as routine surveillance or diagnostic activities. Samples were not collected for the purposes of this study.
Ethics oversight	See above.

Note that full information on the approval of the study protocol must also be provided in the manuscript.

Plants

Seed stocks	*Report on the source of all seed stocks or other plant material used. If applicable, state the seed stock centre and catalogue number. If plant specimens were collected from the field, describe the collection location, date and sampling procedures.*
Novel plant genotypes	*Describe the methods by which all novel plant genotypes were produced. This includes those generated by transgenic approaches, gene editing, chemical/radiation-based mutagenesis and hybridization. For transgenic lines, describe the transformation method, the number of independent lines analyzed and the generation upon which experiments were performed. For gene-edited lines, describe the editor used, the endogenous sequence targeted for editing, the targeting guide RNA sequence (if applicable) and how the editor was applied.*
Authentication	*Describe any authentication procedures for each seed stock used or novel genotype generated. Describe any experiments used to assess the effect of a mutation and, where applicable, how potential secondary effects (e.g. second site T-DNA insertions, mosiacism, off-target gene editing) were examined.*
